# Temporal stability of the rumen microbiome and its longitudinal associations with performance traits in beef cattle

**DOI:** 10.1038/s41598-024-70770-3

**Published:** 2024-09-05

**Authors:** Joana Lima, Marina Martínez-Álvaro, Jennifer Mattock, Marc D. Auffret, Carol-Anne Duthie, Matthew A. Cleveland, Richard J. Dewhurst, Mick Watson, Rainer Roehe

**Affiliations:** 1 Scotland’s Rural College, Edinburgh, UK; 2 Universitat Politècnica de València, Valencia, Spain; 3 The Roslin Institute and the Royal (Dick) School of Veterinary Studies, University of Edinburgh, Edinburgh, UK; 4 Agrifirm, Drongen, Belgium; 5 Genus Plc, DeForest, WI USA

**Keywords:** Microbial communities, Environmental impact

## Abstract

The rumen microbiome is the focus of a growing body of research, mostly based on investigation of rumen fluid samples collected once from each animal. Exploring the temporal stability of rumen microbiome profiles is imperative, as it enables evaluating the reliability of findings obtained through single-timepoint sampling. We explored the temporal stability of rumen microbiomes considering taxonomic and functional aspects across the 7-month growing-finishing phase spanning 6 timepoints. We identified a temporally stable core microbiome, encompassing 515 microbial genera (e.g., *Methanobacterium*) and 417 microbial KEGG genes (e.g., K00856—adenosine kinase). The temporally stable core microbiome profiles collected from all timepoints were strongly associated with production traits with substantial economic and environmental impact (e.g., average daily gain, daily feed intake, and methane emissions); 515 microbial genera explained 45–83%, and 417 microbial genes explained 44–83% of their phenotypic variation. Microbiome profiles influenced by the bovine genome explained 54–87% of the genetic variation of bovine traits. Overall, our results provide evidence that the temporally stable core microbiome identified can accurately predict host performance traits at phenotypic and genetic level based on a single timepoint sample taken as early as 7 months prior to slaughter.

## Introduction

The rumen microbiome, a fundamental component of the digestive processes of ruminants, is the focus of extensive investigation regarding its association with host productivity traits and methane (CH_4_) emissions^1–3^. Short-term fluctuations of the rumen microbiome after feed enters the rumen have been described to be associated with different microbial colonization events. Following the displacement of the epiphytic microbiome, two colonization events align with dry matter disappearance rates, and increases in microbial richness and alpha diversity^4–10^: a primary colonization occurs usually within the first hour (potentially amylolytic microbes and methanogenic archaea), followed by a stabilisation period (of variable length, depending on substrate composition), and a secondary colonization (potentially microbes specialized in cellulose and hemicellulose degradation). The community structure at each colonization event differs depending on dietary composition^11,12^. However, the long-term temporal stability of both its taxonomic and functional dimensions remains largely unknown. Snelling et al. studied the long-term stability of the rumen microbiota in growing-finishing cattle (at start of test more than 1 year of age) and revealed that after adapting to dietary interventions, a single sample could be considered reasonably representative of rumen microbial communities^13^. However, their research focused only on the rumen microbiota profiles (i.e., microbial communities characterized based on 16S rRNA amplicon sequencing), and no investigation was performed regarding the functional aspects of the rumen microbiome, nor into their longitudinal associations with host traits on phenotypic or animal genetic levels.

In studies based on a single sample per animal, the rumen microbiome has been found to be closely linked to host performance traits, such as feed conversion ratio (FCR) and methane production^14–17^ at the phenotypic level, and is strongly influenced by the diet composition^18–20^. In addition, after adjustment for diet effects, the host animal genetics substantially influence rumen microbiome profiles, with many microbial taxa and genes showing moderate to high heritabilities^21–23^. Further supporting evidence for bovine genetic effect on the composition of the rumen microbiome has been reported; when a near-total exchange of ruminal contents was conducted between cows differing in pre-feed ruminal pH and total VFA concentrations, their pH, followed by VFA total concentrations and microbiota in the rumen, recovered and became similar to their original state, likely due to host genetic control over salivation, rumination and extent and rate of VFA absorption^24,25^.

Associations between rumen microbiome and host traits in beef cattle have been extensive explored, most often based on single-timepoint sampling, usually immediately after slaughter. Therefore, investigating the temporal dynamics of rumen microbiome profiles and the longitudinal stability of the association of microbiome profiles with host traits is crucial. If rumen microbiome profiles are indeed temporally stable at taxonomic and functional levels, a single sample could be representative of the growing-finishing period, reinforcing results from previous investigations. Additionally, the possibility of using earlier samples to characterize the rumen microbiome would be advantageous, for example, to improve the annual gain of microbiome-driven breeding as developed by Martínez-Álvaro et al.^26^, as a result of faster genetic turnover.

Our study aimed at closing knowledge gaps around long-term stability of rumen microbiome profiles during the growing-finishing period of beef cattle over six timepoints (approximately one month apart), using microbiome data obtained from high-depth whole metagenome shotgun sequencing, by addressing microbiota profiles (microbial taxa) and by investigating rumen microbial gene profiles (functional attributes). We further investigated the longitudinal stability of associations between rumen microbiome profiles and key bovine productivity traits including FCR, average daily gain (ADG), daily feed intake (DFI), residual feed intake (RFI), as well as highly potent greenhouse gas traits, daily methane production (CH4P, g CH_4_/day), and methane yield (CH4Y, g CH_4_/kg dry matter intake, DMI). In a previous study, we revealed that part of the microbiome is controlled by the host genome and identified microbiome features with significant heritability^22^. Building up on this finding, we investigated the temporal stability of microbial genes under the influence of the animal’s genetics and of their longitudinal associations with genomic breeding values of animal performance traits, further contributing to the understanding of the cattle genetic effects on the longitudinal composition of the microbiome.

Therefore, our main aims included the investigation of (1) the temporal stability of microbiome features (microbial taxa and microbial KEGG genes), (2) the temporal stability of the associations between microbiome profiles at the taxa and gene levels and animals’ performance traits, (3) the temporal stability of previously identified microbial biomarkers (microbial genera and/or KEGG genes) linked to animals’ production and methane emissions traits, and (4) temporal stability of the associations between animal-genomically influenced microbial KEGG gene profiles and bovine genomically affected performance traits.

## Results

**Fig. 1:**
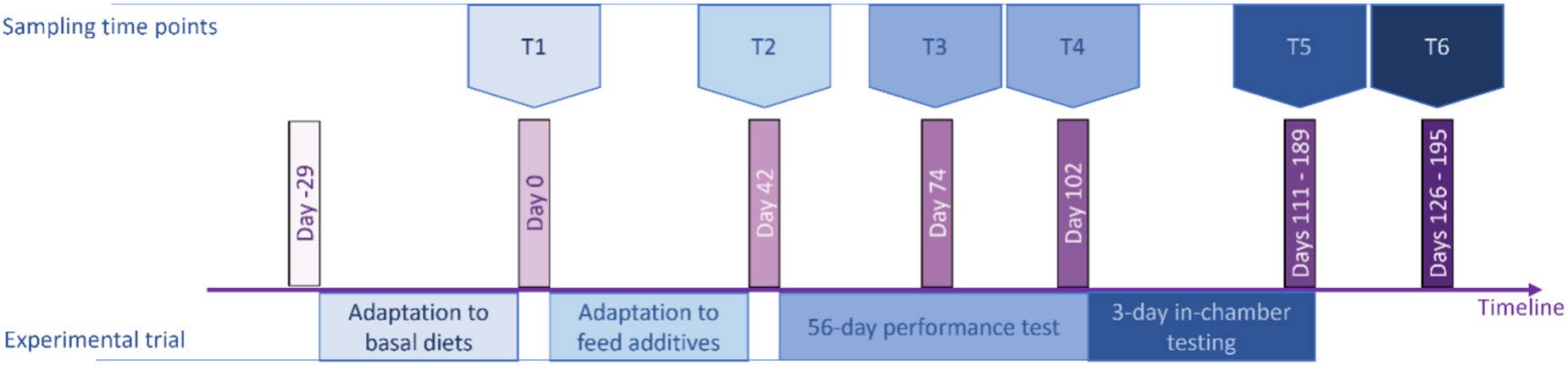
Schematic representation of the experimental timeline. Data was recorded during the 56-day performance testing period and methane emissions during the 3-day-long respiration chamber period. T1 to T6 represent each of the timepoints in which the rumen digesta was sampled from live animals. T6 corresponds to shortly after slaughter.

The unique longitudinal data comprised microbiome abundance profiles of microbial taxa at the genus level (MT) and at the microbial KEGG genes (MG) level. These were generated based on deep whole metagenomic sequencing of microbial DNA from rumen samples taken at six timepoints (T1 to T6) approximately one month apart, throughout the growing-finishing phase of beef cattle (Fig. 1). A total of 1178 MT, classified based on genetic and evolutionary characteristics, were identified by aligning sequenced reads against the Kraken database^27^ including both genomes of organisms from the Hungate 1000 collection^28^ and the NCBI RefSeq database^29^. Additionally, 6,916 MG, categorised by their biological functions, were identified by aligning sequenced reads against the KEGG database. Economically important performance traits FCR, ADG, DFI and RFI were obtained based on measurements taken during a 56-day-long performance test period (T2–T4). The emissions of the highly potent greenhouse gas methane were individually measured in respiration chambers, 3 days prior to timepoint T5. The traits FCR, ADG, DFI, RFI, CH4P and CH4Y were on average ± standard deviation 8.29 ± 1.69, 1.42 ± 0.32, 11.30 ± 1.27, -0.08 ± 0.65, 183.12 ± 53.12 and 19.53 ± 5.32, respectively.

**Fig. 2:**
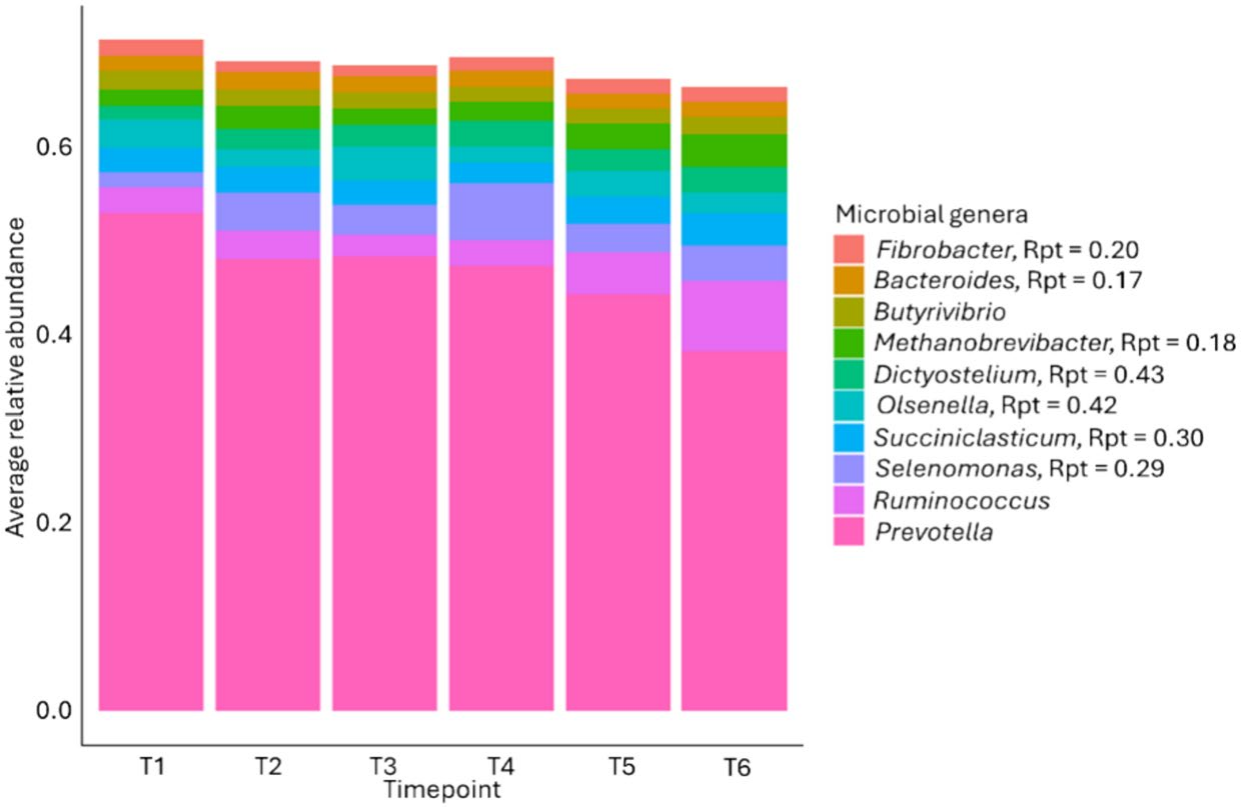
Rumen microbial genera. Stacked bar chart of the ten microbial genera with the highest average relative abundances over all timepoints and animals. Rpt refers to repeatability, analysed across both diets. Microbial genera without an Rpt value were not significantly repeatable in this analysis.

The rumen microbiota was dominated by *Prevotella*, *Ruminococcus*, and *Selenomonas* (Fig. 2), with average relative abundances ± standard deviations across all animals and timepoints of 0.46 ± 0.14, 0.04 ± 0.06 and 0.04 ± 0.05, respectively.

### Temporal stability of microbial genera and their genes

Our first aim in this study was to investigate the temporal stability of the microbiome profiles over the experimental period of 7 months. The temporal stability was evaluated by alpha and beta diversity criteria, as well as using linear mixed models, repeatability and correlation analyses.

#### Diversity indices

Diversity was assessed through Shannon index (alpha diversity) and Bray–Curtis dissimilarity matrices (beta diversity) to evaluate stability patterns of the rumen microbial community and functional profiles over time. The diversity indices were remarkably stable throughout the growing-finishing period. The adjusted Shannon index (measure of evenness) was used to evaluate and compare alpha diversity in samples from each timepoint. At the MT level, no significant differences were observed (*p* value = 0.09). At the MG level, T6 samples (shortly after slaughter) had significantly higher adjusted Shannon diversity than T3 (i.e., mid-test), with averages of 0.891 ± 0.007 and 0.880 ± 0.013, respectively (*p* value = 0.02), whereas no significant differences were observed for any other pairwise comparison between timepoints (*p* value > 0.05).

**Fig. 3:**
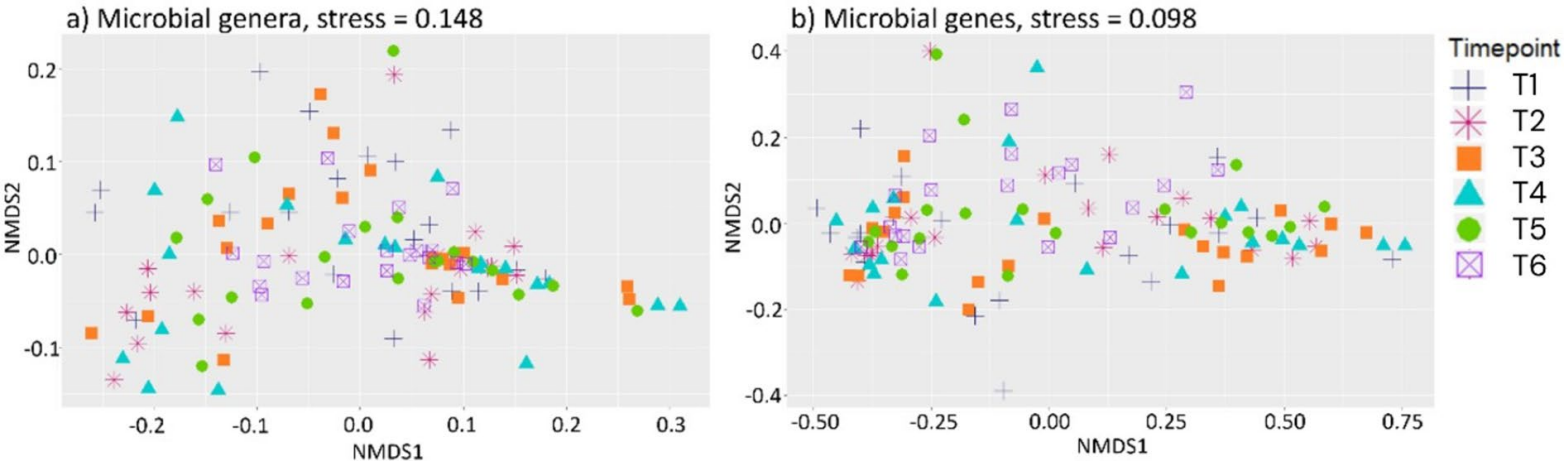
Clustering of samples based on beta diversity. Non-metric multi-dimensional scaling (NMDS) plot of Bray–Curtis distances between samples at a microbial genera and b microbial genes level. The stress values of < 0.2 above the panels indicate that the two-dimensional plot is a fair representation of the original high-dimensional distances with some acceptable distortion.

Bray–Curtis dissimilarity indices (BC) were estimated for all samples and the effect of timepoint was evaluated in PERMANOVA. A significant effect of timepoint was detected when exploring MT profiles. The post hoc tests revealed that these were only between T6 (at slaughter) and previous sampling timepoints (*p* value = 0.001). However, these differences were small, i.e., variances explained by the factor timepoint were 0.143, 0.101, 0.114, 0.106 and 0.097 when comparing T6 to T1, T2, T3, T4, and T5, respectively. Regarding MG, timepoint had an insignificant effect (*p* value = 0.419). We plotted the BC matrices using the non-metric multi-dimensional scaling (NMDS), and these revealed no distinctive clustering of samples that could be explained by timepoint (Fig. 3).

#### Linear mixed model analysis

To further evaluate temporal stability, we fitted two linear mixed models for each microbiome feature (MT or MG), one model with and one model without timepoint as fixed effect, and then compared these two models in an ANOVA (*p* values were corrected using the Bonferroni method). The results revealed that only 11 out of 1050 MT and only 14 out of 1901 MG were better described by models including timepoint (i.e., showed Bonferroni adjusted *p* values of less than 0.05, as well as lower  Bayesian Information Criterion (BIC) value), indicating that only a few microbiome features were affected by temporal variation. The effect of feed additives on the microbiome (at both MT or MG levels) was also evaluated using linear mixed models, and it was found to be negligible. Only 10 MT and 16 MG were better described by fitting the factor feed additive in the model, i.e., showed Bonferroni adjusted *p* values of less than 0.05.

#### Repeatability analysis

To evaluate the repeatability of each microbiome feature over time, we fitted repeatability (Rpt) models for each MT and MG. The results showed that 515 MT and 417 MG (49% and 22% of the total, respectively) had significant Rpt (*p* value < 0.05) of up to 0.79 (MT) and 0.43 (MG).

Rpt models were also fitted for each MT and MG within diet group (CON and FOR). Within CON diet, 412 MT and 376 MG were significantly (*p* < 0.05) repeatable over time (Rpt up to 0.73 and 0.49, respectively). Within FOR diet, 223 MT and 165 MG (Rpt up to 0.84 and 0.52, respectively) had significant Rpt (*p* value < 0.05, Supplementary Table 1). A total of 144 MT and 50 MG had significant (*p* value < 0.05) Rpt within both diets. Comparison of the magnitude of the Rpt of these 144 MT and 50 MG revealed no significant differences (*p* value ≥ 0.05) between diets i.e., feeding different basal diets to the animals did not lead to their rumen MT/MG showing higher or lower Rpt through time. Within the 144 MT, *Negativicoccus*, *Cryptobacterium*, *Methanothermobacter*, *Methanobacterium and Anaerovibrio* were among the most repeatable (average Rpt across two diets > 0.40). Of the 50 MG, 22 had an average Rpt > 0.30 across two diets, and most of these were involved in ribosomal pathways, widespread in all organisms, including small and large ribosomal units e.g., L10e and S4e.

Regarding features significantly repeatable exclusively within each diet, *Cryptobacterium*, *Isaria*, and *Cutaneotrichosporon* had the highest Rpt within CON, whereas within the FOR group, the most repeatable were *Selenomonas*, *Thermovirga*, and *Lysobacter*. Additionally, a total of 47 MG had Rpt > 0.30 within CON, several associated with biosynthesis of cofactors, including e.g., *bioF*: 8-amino-7-oxononanoate synthase (K00652), *ndk*: nucleoside-diphosphate kinase (K00940) and *ribF*: riboflavin kinase / FMN adenylyltransferase (K11753), whereas within FOR, 43 MG had Rpt > 0.30, which were involved in biosynthesis of amino acids (e.g., *leuB*: 3-isopropylmalate dehydrogenase (K00052), *metX*: homoserine O-acetyltransferase/O-succinyltransferase (K00641), and *gltB*: glutamate synthase (NADPH) large chain (K00265)).

#### Correlations between microbiome profiles collected at different timepoints

**Fig. 4:**
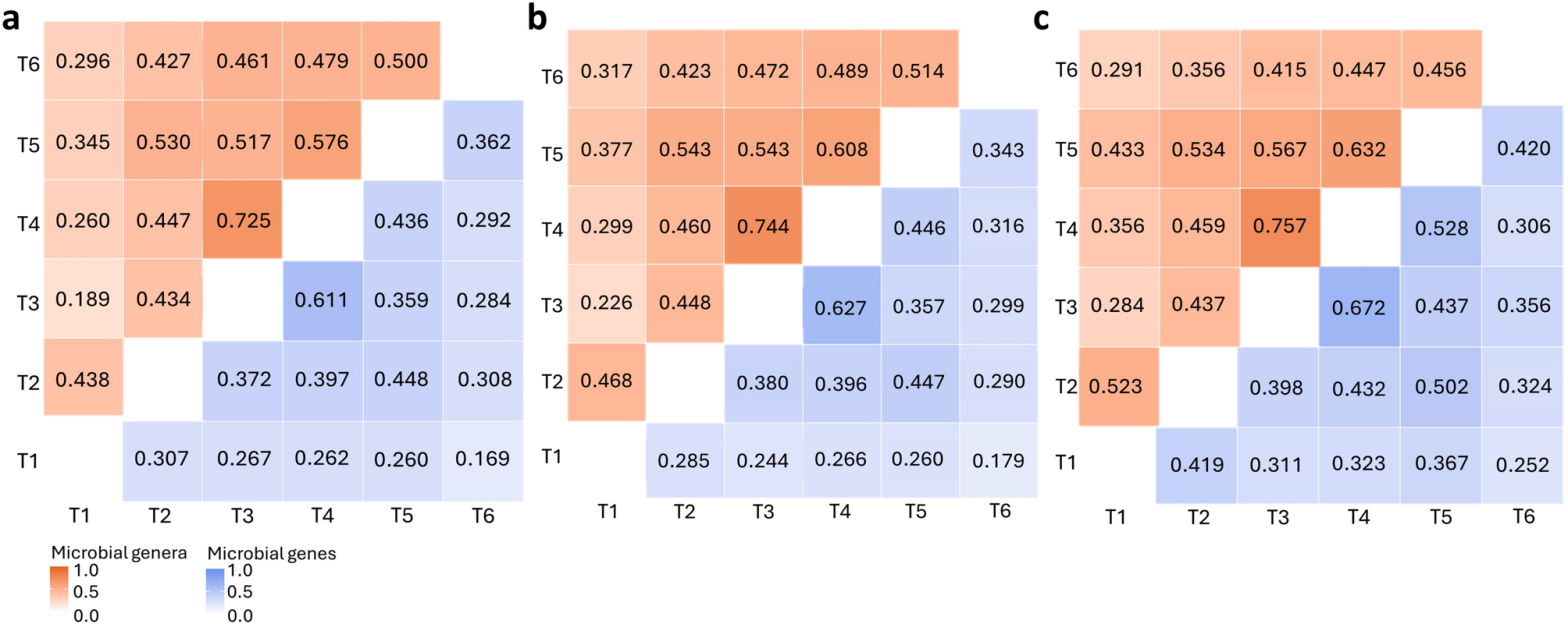
Correlations between microbiome profiles of different timepoints. Pearson correlations between vectorized matrices of (**a)** 515 microbial genera (MT) and 417 microbial genes (MG), (**b)** 412 MT and 376 MG from animals fed concentrate (CON), and (**c)** 223 MT and 165 MG from animals fed forage (FOR), respectively. MT and MG abundances were centred and additive log-ratio transformed and then centred based on their mean abundance throughout all timepoints. Correlations of MT profiles from different timepoints are represented above the diagonal and MG are represented below the diagonal.

To determine the degree of associations between microbiome profiles of MT or MG over time, we estimated the Pearson correlation between profiles from each timepoint. Prior to this analysis, microbiome profiles from each timepoint were condensed into 6 vectors, resulting in one vector per timepoint for MT and one vector per timepoint for MG. When considering the entire microbiome profiles (i.e., 1050 MT and 1901 MG), correlations between timepoints were low to moderate, varying from 0.117 (T1–T3) to 0.593 (T3–T4) for MT and from 0.089 (T1–T6) to 0.472 (T3–T4) for MG (Supplementary Table 2). When analysing only the significantly repeatable 515 MT and 417 MG, the correlations were higher, ranging from 0.189 (T1–T3) to 0.725 (T3–T4) for MT and from 0.169 (T1–T6) to 0.611 (T3–T4) for MG (Fig. 4a). These correlations were also estimated for MT and MG that had significantly repeatability within each diet group. Within CON diet, correlations fluctuated from 0.226 (T1–T3) to 0.744 (T3–T4) for MT and from 0.179 (T1–T6) to 0.627 (T3–T4) for MG, whereas within FOR diet, correlations ranged from 0.284 (T1–T3) to 0.757 (T3–T4) for MT and from 0.252 (T1–T6) to 0.672 (T3–T4) for MG (Fig. 4b,c).

### Temporal stability of the associations between microbial genera or genes and performance traits throughout the growing-finishing phase

**Fig. 5:**
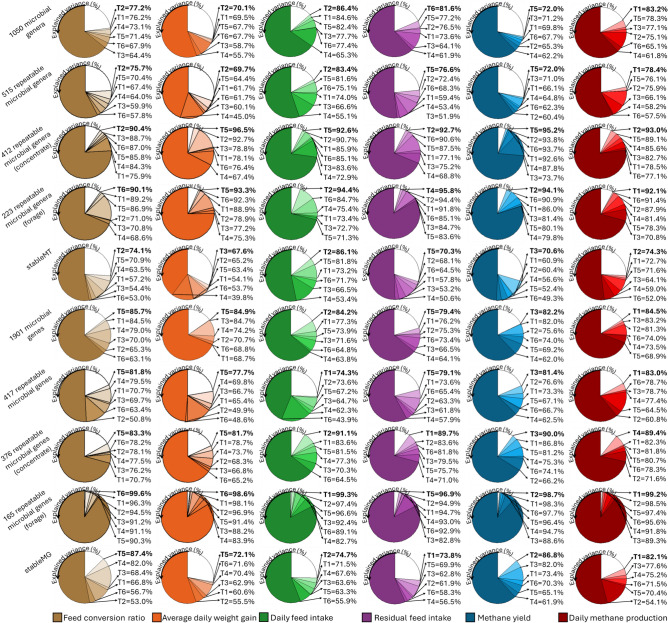
Proportion of variance of each trait explained by repeatable microbiome features in six timepoints. Phenotypes (feed conversion ratio, average daily gain, daily feed intake, residual feed intake, methane yield and daily methane production) were predicted using partial least squares models including as explanatory variables different sets of microbial genera (all (i.e., 1050), 515 repeatable across both diets, 412 repeatable within concentrate-fed animals, 223 repeatable within forage-fed animals, and stableMT) or microbial genes (all (i.e., 1901), 417 repeatable across both diets, 376 repeatable within concentrate-fed animals, 165 repeatable within forage-fed animals, and stableMG). Each pie chart is read counterclockwise, in a cumulative manner; for example, using 1050 microbial genera from timepoints T3, T6, T5, T4, T1, and T2 explains 64.4%, 67.9%, 71.4%, 73.1%, 76.2% and 77.2%, respectively, of the variance in feed conversion ratio.

After evaluating the temporal stability of the rumen microbiome, we aimed at exploring the temporal stability of the associations between the microbiome profiles and the animal host performance traits (FCR, ADG, DFI and RFI). In this analysis, we used partial least squares models (PLS) because the methodology accounts for the multicollinearity of microbiome datasets and, by providing a measurement of Variable Importance in Projection (VIP) for each predictor variable, facilitate the identification of important variables (i.e., microbiome features) associated with the predicted variables (i.e., the host traits). In these PLS models, the significantly repeatable 515 MT and 417 MG were included as explanatory variables and the host traits as dependent variables (one model per timepoint and per host trait). On average (over 6 timepoints), 515 MT and 417 MG explained up to 72.63 ± 10.45% and up to 73.86 ± 8.95% of the variance in host traits, respectively. The amount of variance explained was similar over time, showing low standard deviations of up to 11.47% (Fig. 5, second and seventh rows).

The same analysis (based on PLS models) was performed within diet. The results showed that fewer MT and MG (in comparison to those selected based on both diets) explained higher proportions of host traits’ variance. The repeatable 412 MT and 376 MG obtained within the CON diet group, and the repeatable 223 MT and 165 MG within the FOR diet group explained a higher proportion of the variance than the analyses including both diets (84.6 ± 7.9% and 77.9 ± 7.2%, respectively for CON and 83.4 ± 8.2% and 93.8 ± 4.7% for FOR) (Fig. 5, CON: third and eighth rows, FOR: fourth and nineth rows).

Since the performance traits were based on measurements taken during a 56-day performance testing period, the microbiome datasets generated from sampling during this period (i.e., T2, T3, and T4) were expected to explain the highest trait variance. However, only some of our results agreed with these expectations (Fig. 5). For example, FCR, ADG, and DFI had their highest variances explained when MT profiles were generated from T2 (FCR_MT_ = 77.2%, ADG_MT_ = 70.1%, DFI_MT_ = 86.4%). However, microbiomes collected at T1 explained, on average, higher amounts of variances across all traits (MT = 76.2 ± 6.5% and MG = 78.9 ± 6.1%). Considering all timepoints simultaneously, the trait best predicted by MT profiles was DFI (79.0 ± 7.6%) and by MG profiles was CH4P (77.6 ± 6.3%).

After exploring the explained variances of performance traits using repeatable microbiome features, we used VIPs (obtained from PLS) to identify specific MT and MG strongly associated with each animal performance trait over time, and we used regression coefficients (obtained from PLS) to evaluate the stability of the direction of these associations. Based on these results, we identified 273, 359, 365, 312, 169, and 321 MT important for the prediction of FCR, ADG, DFI, RFI, CH4Y ﻿and CH4P, respectively (VIP > 0.8 in at least 4 timepoints). Of these, 224, 264, 270, 252, 162, and 288, respectively, had the same direction of association with the traits (at least 4 timepoints with all positive or all negative regression coefficients). Based on VIP > 0.8, 177, 177, 267, 268, 267, and 232 MG were identified as important, and of these, 144, 133, 194, 214, 223, and 204 expressed the same direction of association with the traits FCR, ADG, DFI, RFI, CH4Y and CH4P, respectively. From this point on, we will refer to these sets of MT and MG as stableMT and stableMG, respectively.

StableMT included e.g., *Methyloversatilis* (positively associated with FCR and negatively associated with ADG and CH4P), *Paucibacter* (positive association with FCR, negative association with ADG), *Shinella* (positive association with RFI, DFI, and ADG, negative association with FCR), *Negativicoccus* (positive association with DFI, CH4Y, CH4P), *Methanobacterium* (positive association with DFI, RFI, CH4Y, CH4P), *Methanothermobacter* (positive association with DFI, CH4Y, CH4P), *Methanobrevibacter* (positive association with DFI and CH4P), *Legionella* and *Candidatus Protochlamydia* (both positively associated with RFI).

StableMG positively associated with FCR were mostly involved in biosynthesis of amino acids (e.g., *hom*: homoserine dehydrogenase), carbon metabolism (e.g., *por*: pyruvate-ferredoxin/flavodoxin oxidoreductase) and ABC transporters (e.g., *ecfT*: energy-coupling factor transport system permease protein) and those negatively associated with FCR were involved in purine (e.g., *ndk*: nucleoside-diphosphate kinase) and sulphur metabolism (e.g., *cysH*: phosphoadenosine phosphosulfate reductase) pathways. In the case of ADG, both positively and negatively associated stableMG were involved in biosynthesis of amino acids (e.g., *leuC*: 3-isopropylmalate/(R)-2-methylmalate dehydratase large subunit and *hom*: homoserine dehydrogenase), however, negatively associated stableMG were more scattered through other pathways, including e.g., *pncB*: nicotinate phosphoribosyltransferase (biosynthesis of cofactors and nicotinate and nicotinamide metabolism). Most stableMG positively associated with DFI were involved in biosynthesis of amino acids (e.g., *leuB*; 3-isopropylmalate dehydrogenase), and biosynthesis of cofactors (e.g., *thiE*; thiamine-phosphate pyrophosphorylase), but also included in pathways related to carbon and methane metabolism (e.g., *hxlB*; 6-phospho-3-hexuloisomerase), whereas those negatively associated with DFI were mostly ABC transporters (e.g., *ecfA2*: energy-coupling factor transport system ATP-binding protein). Several stableMG positively associated with RFI were small and large subunit ribosomal proteins (e.g., L10e, L14e, S6e), and others were involved in carbon metabolism (e.g., *hxlB*: 6-phospho-3-hexuloisomerase). While most stableMG positively associated with CH4Y were involved in biosynthesis of amino acids (e.g., *metX*: homoserine O-acetyltransferase/O-succinyltransferase), most of those with negative association were ribosomal protein subunits (e.g., L19, S9). Regarding CH4P, most positively associated stableMG were involved in biosynthesis of cofactors (e.g., E2.7.8.26: adenosylcobinamide-GDP ribazoletransferase) and amino acids (e.g., *hisC*: histidinol-phosphate aminotransferase) and some were also involved in methane and carbon metabolism (e.g., *frhB*: coenzyme F420 hydrogenase subunit beta). StableMG negatively associated with CH4P included e.g., *psuG*: pseudouridylate synthase (pyrimidine metabolism) and *hemL*: glutamate-1-semialdehyde 2,1-aminomutase (biosynthesis of cofactors).

**Fig. 6:**
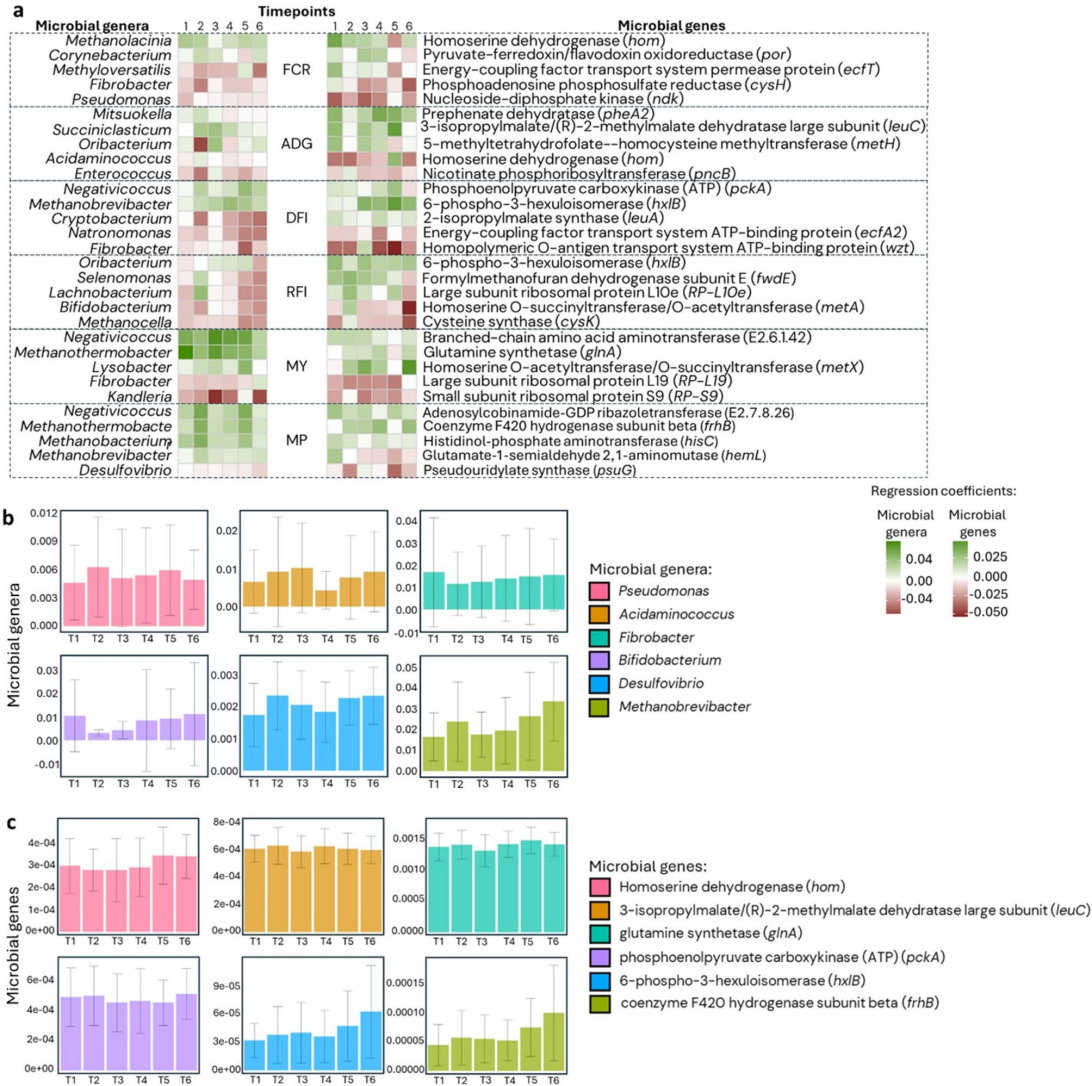
Microbial genera and genes with temporally stable and strong relationship with host traits. Heatmaps show the direction of longitudinal associations (positive or negative) between selected **a** microbial genera and microbial genes and host performance traits. Bar charts present the average relative abundance and standard deviation of each **b** microbial genera and **c** microbial genes over six sampling timepoints.

To validate the stableMT and stableMG as reliable biomarkers for predicting host traits, we did an in-depth analysis using their abundances across the 6 timepoints. The results showed that the stableMT and stableMG were strongly associated with the host traits over the growing-finishing phase (Supplementary tables 3 A-D). For example, the microbiome profiles based on 270 MT explained on average over all timepoints 72.1 ± 11.6% of the variation in DFI, whereas 223 MG explained on average 73.3 ± 9.6% of the variation in CH_4_ yield (Fig. 5, fifth and tenth rows). The relative abundance and regression coefficients of some of these MT and MG are represented in Fig. 6.

### Temporal stability of microbial biomarkers identified in previous studies

Our previous research has identified microbial biomarkers of host performance traits, based on microbiome profiles generated from slaughter samples. Out of a total of 25 previously identified microbial genera, 8 were removed from the present study because they did not fulfil the criterion of being identified in all timepoints and animals. A further 2 genera were removed because they had relative abundance lower than the threshold chosen in the current study. Out of 149 microbial genes previously identified as biomarkers, 51 were not considered in the present study because they did not meet our inclusion criteria of being identified at sufficient abundance in all animals and all timepoints. The remaining microbial genera and genes were analysed for their ability to predict host traits based on their abundances in each of our timepoints. A total of eight sets of biomarkers were used in this analysis, including 7 MG sets (with 4 to 40 genes), and one MT set (with 15 genera). On average over the 6 timepoints, MG sets explained from 26 to 56% of the variance of traits, and the MT set explained 42% (Supplementary table 4A and 4B).

### Temporal stability of the associations between animal-genomically influenced microbial gene profiles and the animal genomic effects on host performance traits

In a previous study, Martínez-Álvaro et al.^26^ identified 1002 significantly heritable MG (h^2^ from 0.20 to 0.58). Of these, 673 were present in our longitudinal microbiome database. In addition, the animal genomic effects in form of estimated breeding values (EBVs) of FCR, ADG, DFI and CH4Y for the animals included in the present study were available^22^. Therefore, to evaluate the stability of associations between heritable MG and EBVs of traits, we used the heritable MG as predictors of EBVs of host performance traits in PLS models.

These analyses revealed that a high proportion of the variability of the EBVs of each trait was explained by the host-genomically influenced MG throughout all timepoints, with averages of 74.0 ± 6.5%, 67.8 ± 5.0%, 70.3 ± 6.0%, 67.6 ± 8.6%, and 68.4 ± 9.2%, for FCR, ADG, DFI, RFI, and CH4Y, respectively. In addition, 304, 395, 377, 380, and 415 MG were important for the prediction of FCR, ADG, DFI, RFI, and CH4Y, respectively, in 4 or more timepoints, and, of these, 226, 282, 271, 268, and 312 were found to maintain the direction of their relationship with the host performance traits in at least 4 timepoints (Supplementary tables 5A and 5B).

## Discussion

In this research, we have explored the temporal stability of the rumen microbiome and investigated the temporal stability of associations between rumen microbiome profiles during the growing-finishing phase and important animal performance phenotypes including FCR, DFI and methane emissions. Although previous works on the longitudinal stability of the rumen microbiome have been conducted, these were overall focused on the rumen microbiota^13^, whereas the rumen microbial functional potential and the temporal stability of its associations with host traits remained unclear. Here we used whole metagenome sequencing to characterize rumen microbiome profiles and to explore their temporal stability, filling important knowledge gaps. Our results, based on analyses of microbial diversity, timepoint effects, repeatability estimates, and correlation analyses between microbiome profiles from different timepoints, identified a core rumen microbiome which is temporally stable over the growing-finishing phase of beef cattle.

The rumen microbiome has been shown to significantly shift over the early life of calves as they mature, e.g., progressive depletion of aerobic and facultative aerobic groups, establishment of cellulolytic microbes, and increased taxonomic diversity^30^, stabilizing around 21 days of age^31^, whereas other studies indicate a longer period of change of 6 months to even 2 years^13,30^. In older animals, there is growing evidence that, in the absence of major perturbations (i.e., diet changes), the microbiome remains relatively stable^13^. For many research questions in animal nutrition and animal breeding, to understand whether an established rumen microbiome of growing-finishing animals (in our dataset analysed from 1 to 1.5 years of age) is temporally stable and consistently associated with animal performance traits is crucial. In particular, understanding the stability of diversity measures over time is critical for the reliability of using samples from different timepoints for microbiome analysis. Our results, based on diversity analyses of MT and MG in absolute counts without any restriction regarding prevalence or relative abundance, showed that alpha diversity remained stable over time, with only MG profiles of T6 samples (slaughter) showing a significant increase in evenness compared to T3, in agreement with previous works^32^. Minor differences observed in beta-diversity of MT profiles were likely due to lowly abundant microbial genera. The rumen liquid digesta comprises distinct communities from those in the rumen epithelium^33,34^ and solid fraction^35^. Differences between preceding timepoints and T6 might be associated with sampling methods; samples from live animals were obtained through a nasogastric tube (i.e., liquid digesta samples), whereas post-slaughter samples were directly collected from the open rumen, which are, as a result of the slaughter process, well mixed. In a study based on 16S rRNA gene amplicon sequencing, Snelling et al. reported Bray–Curtis dissimilarity indices to significantly differ between microbiota datasets from samples taken before the introduction of a feed additive and from after 25 days in concentrate-fed animals, but no difference was reported for forage-fed animals^13^. Considering that the adaptation period from an original forage-based to a high concentrate-based diet lasted 4 weeks, the reported variations could be due to the adaptation of the microbial communities to the new diet, rather than to the passing of time. In fact, in another study based on 16S rRNA gene amplicon, Clemmons et al. (2019) showed that the rumen microbiome started to become stable at 4 weeks after a dietary change, but continued to stabilize over the next 5 weeks, and recommended an adaptation period of at least 8 weeks following the transition from a forage-based to a concentrate-based diet^20^.

The ten most abundant microbial genera in the rumen of all animals at all timepoints (Fig. 2) included microbes typically found in the rumen, in accordance with previous authors^36^. Of these, *Prevotella*, which is central to carbohydrate and hydrogen metabolism and associated with a healthy rumen microbiome^37^, *Ruminococcus*, a cellulolytic and amylolytic microbe^38^, and *Butyrivibrio*, an important genus involved in the degradation of xylan, pectins and hemicellulose, protein breakdown, and biohydrogenation of fatty acids^39^, were not significantly repeatable when Rpt was analysed across both diets nor within diet.

Our results suggested that the vast majority of microbiome features was not significantly influenced by temporal effects. In addition, the analysis based on repeatability models enabled the identification of 515 significantly repeatable MT including 144 repeatable within both diets, such as *Negativicoccus*, previously identified as enriched in the rumen of healthy goat kids in comparison to counterparts infected with *Cryptosporidium parvum*^40^. Another example is *Cryptobacterium*, which we found to be negatively associated with DFI. This result agrees with a previous report of negative association between *Cryptobacterium* and RFI in beef cattle^15^, because RFI is positively correlated with DFI. It suggests that an increase in abundance of *Cryptobacterium* would improve feed conversion efficiency since increase in negative values of RFI are indicative of less feed needed to achieve the same performances as the population. Methanogens *Methanothermobacter* and *Methanobacterium* were also temporally stable in both diets, and this is expected, because methanogens remove hydrogen from the rumen which would hinder fermentation processes^41–43^. However, the dominant ruminal methanogen *Methanobrevibacter*^44^ was temporally instable over time. This was also the case with highly dominant genera such as *Prevotella*, which showed nonsignificant repeatability. These findings suggest the need for more in-depth studies into temporal stability of the rumen microbiome at species and even strain levels using metagenome-assembled genomes^45^. Most of the 417 significantly repeatable MG were associated with biosynthesis of amino acids (e.g., *hom*: homoserine dehydrogenase, K0003), biosynthesis of cofactors (e.g., *thiE*: thiamine-phosphate pyrophosphorylase, K00788), whereas the 50 MG repeatable in both diets had a majority of genes associated with ribosome, which may reflect different functions found in the microbial genetic repertoire of animals fed different diets.

Correlations between microbiome profiles from different timepoints were strong (between 0.43 and 0.73 for MT and between 0.31 and 0.61 for MG), further highlighting the temporal stability of microbiome profiles at taxonomic and genetic levels. While no comparable analyses have been reported in the literature in the context of temporal stability of microbiome profiles including both microbial taxa and microbial KEGG genes, our findings support the hypothesis that there is a core taxonomic and functional microbiome that remains stable over time.

Overall, the rumen microbiome as generated from samples throughout the growing-finishing phase explained a high amount of variance of each trait, particularly when using MG as predictor variables. Microbial taxa and genes identified as temporally stable and strongly associated with host performance traits (stableMT and stableMG) explained more than 39.8% and more than 53.0%, respectively, of the variation of host traits. In addition, the amount of explained variance of each trait was similar over time, suggesting that core microbiome profiles obtained from samples collected at any timepoint during the growing-finishing phase are highly associated with and are therefore suitable for prediction of host performance traits.

Most of the MT identified as temporally stable and strongly associated with host performance traits (i.e., stableMT) included microbial genera belonging to phyla Proteobacteria and Ascomycota. Actinobacteria were also highly prevalent, maintaining stable associations with FCR, ADG and CH4P, whereas Firmicutes were associated with DFI and RFI, and Euryarchaeota with CH4Y (g/kg DMI) (Supplementary tables 3A and 3B). The betaproteobacterium *Methyloversatilis* had a stable negative association with FCR, in agreement with Auffret et al.^15^, and a positive association with ADG and CH4P. *Paucibacter* showed a stable positive association with FCR and negative with ADG, in agreement with Freetly et al.^46^ (in caecum microbiome) and Huws et al.^6^ (short-term temporal stability). *Shinella* (phylum Cyanobacteria) has previously shown short-term temporal stability^6^ and in the present work it showed long-term temporal stability with strong positive associations with various host traits, including FCR, ADG, DFI, and RFI. *Negativicoccus* showed positive associations with DFI, CH4P and CH4Y. This genus belongs to the family Veillonellaceae, which, together with Cyanobacteria, has previously been identified as a core component of the active rumen microbiome^3^. Three Methanobacteriales (hydrogenotrophic methanogens) were also identified, including *Methanobacterium* (positive stable associations with DFI, RFI, CH4P and CH4Y), which can also use formate as a substrate^47^, *Methanothermobacter* (positive stable associations with DFI, CH4P and CH4Y) and *Methanobrevibacter* (positive stable associations with DFI, and CH4P).

The identification of opportunistic pathogens *Legionella* and *Candidatus protochlamydia* as temporally stable and positively correlated with RFI, indicating unfavourable associations with feed conversion efficiency, could be due to their association with the protozoa *Acanthamoeba*, which is usually found in soil and water^48,49^. Both genera were identified at very low relative abundances (0.006 ± 0.04% and 0.002 ± 0.001%, respectively).

Exploring the temporal stability of previously identified biomarkers is essential to ensure research continuity and enhancing the reliability of these biomarkers in predicting host performance traits over different timepoints. Typically, associations of MT or MG with host performance and CH_4_ emissions traits are investigated based on microbiome profiles generated from slaughter samples^1,2, 21, 25, 34, 46^. However, the suitability of slaughter samples as representatives of the microbiome composition in earlier stages of the hosts’ life were unclear. Our findings reveal a remarkable temporal stability in the rumen microbiome, affirming that previously identified biomarkers maintain significance even when analyses are conducted using data from timepoints other than slaughter. Importantly, the suitability of these biomarkers is further supported when considering that previous studies applied different statistical procedures (e.g., correlation networks), data transformation methods (e.g., relative abundances), and reference databases for MT and MG identification (e.g., through update of KEGG databases). Although a biomarker should be consistently detected in different experimental trials when factors such as breed and diet are similar, some previously proposed biomarkers were not included in our study. The studies that identified these biomarkers were based solely on slaughter samples, whereas our inclusion criteria were more stringent, requiring (i) prevalence in all animals at all timepoints, and (ii) average relative abundance across all samples of at least 0.001%.

The presented study is to the best of our knowledge the first to analyse the longitudinal associations of the animal genomically affected functional microbial genes and the animal genomically controlled part of performance phenotypes. The results indicate that there are strong associations explaining 54.0–86.9% of the variance of the animal genomic effects of economically and environmentally important animal performance traits. This suggests a temporal stability of the associations between host genomically affected functional microbiome and host genomically effects on performance traits. These results open the opportunity that the recently developed microbiome-driven breeding strategy^22,26^ could be based on a single sample taken as early as one year of age, the first sampling in our population, to save cost of sampling without loss of accuracy of genetic evaluation and to reduce the generation interval and thus increasing annual genetic improvement.

## Conclusions

In conclusion, our study unveils the remarkable stability of the rumen microbiome throughout the 7-month-long growing-finishing phase of beef cattle. Our results underscore that microbiome profiles derived from samples collected at any timepoint during this phase effectively represent the dynamic microbial composition characterising this critical production phase.

Moreover, we identified a core set of microbial genera and microbial genes that exhibit temporal stability, maintaining robust associations with economically significant host performance traits across the growing-finishing phase not only on phenotypic but also at animal genetic level. These microbial components emerge as pivotal candidates for future investigations seeking biomarkers to predict host performance traits.

Significantly, our findings highlight strong and stable associations between host-genomically influenced microbial gene profiles and the host genomically controlled part of animal performance traits. This implies that microbiome profiles obtained as early as T1 (corresponding to about one year of age) can reliably inform the prediction of host genomic effects of the analysed performance traits.

In summary, our study contributes valuable insights into the temporal dynamics of the rumen microbiome, offering a foundation for future research aimed at leveraging microbial signatures for improved prediction of economically and environmentally important performance traits in bovines.

## Methods

This study was based on data collected from a previous animal trial investigating the effect of different diets and feed additives on animal performance and CH_4_ emissions in different breeds of beef cattle during the growing-finishing phase^50^.

### Experimental design, animals, and diets

The animal trial followed a 2 × 2 × 3 factorial design and included 84 steers from crossbred Charolais (n = 42) and purebred Luing (n = 42) breeds. Before adaptation to the basal diet, all animals were fed the forage diet (FOR, forage:concentrate ratio of 520:480 g/kg dry matter basis). These animals were allocated to one of two basal diets (FOR, or a concentrate diet, CON, with a forage:concentrate ratio of 84:916 g/kg dry matter basis). On allocation day, these animals weighed on average 414.1 ± 35.7 kg. Steers allocated to the concentrate diet were adapted to it in a stepwise manner over a period of 4 weeks. Animals fed each basal diet were then allocated to one of three treatments; the control (CTL), the supplementation of 21.5 g nitrate/kg dry matter (NIT), and the use of high oil content rapeseed cake (RSC). Animals were adapted to the NIT and RSC treatments in a stepwise manner over a period of four weeks. During the trial, the steers were offered diets ad libitum, at approximately 1.05 times their average DFI. Diet composition and more details on the feeding trial can be found in Duthie et al.^50^.

The experiment was conducted at Scotland’s Rural College (SRUC) Beef and Sheep Research Centre in Edinburgh in 2013. The experimental protocol was approved by SRUC’s Animal Welfare and Ethical Review Body, the Animal Experiments Committee, and was conducted in accordance with the requirements of the United Kingdom Animals (Scientific Procedures) Act, 1986.

### Animals selected for rumen digesta sampling

A total of 20 animals with an average age of 418 ± 32 days at start of test were selected for rumen digesta longitudinal sampling (10 from each breed). Twelve animals were randomly selected (6 from each breed) from the group fed concentrate and considering a balanced design of the three additive groups (CTL, NIT, or RSC), whereas 8 animals (4 of each breed) were chosen from the group fed forage, considering a balanced design of 2 feed additive groups (CTL or NIT). The feed additives were found to have neglectable influence on the microbiome (at both MT or MG levels), and therefore were not included in further analyses.

### Bovine host performance traits

Performance traits were measured during a 56-day testing period. DFI was assessed by measuring dry matter intake (DMI, kg/day), which was recorded using electronic feeding equipment (Insentec, Marknesse, The Netherlands). Body weight (BW) was measured weekly using a calibrated weight scale (in the morning before fresh feed was offered). ADG was modelled by linear regression of BW against test date. FCR was calculated as average DMI divided by ADG. RFI was estimated as deviation of actual DMI (kg/day) from DMI predicted based on linear regression of actual DMI on ADG, mid-MBW, and fat depth at 12th/13th rib at the end of the 56-day test^50–52^, whereby negative RFI indicates increased feed efficiency. CH_4_ emissions were individually measured for 3 days in respiration chambers, from which the final 48h were used to obtain the measurements of CH4P. CH4P were then divided by the average DMI measured within the respiration chambers, resulting in CH4Y. A more detailed description of the traits can be found in Duthie et al.^50^.

### Rumen digesta sampling timepoints

All animals had their rumen digesta sampled after adaptation to the basal diets but before introduction of feed additives (T1) at an average age of 418 ± 32 days, at the start (average of 460 ± 32 days, T2), mid-point (average of 492 ± 32 days, T3), and end (average 520 ± 32 days, T4) of the 56-day performance test period, on the day the animal left the respiration chamber (T5), and at the abattoir within 2h of slaughter (T6).

At each sampling, approximately 50 mL of rumen liquid were taken by inserting a stomach tube (16 × 2700 mm Equivet Stomach Tube, Jørgen Kruuse A/S, Langeskov, Denmark) nasally and aspirating manually. This liquid was filtered through four layers of muslin and 5 mL strained rumen fluid were mixed with 10 ml phosphate buffered saline containing glycerol (30% v/v). These samples were stored at − 20 °C and their DNA extracted as soon as possible, which was stored at -80°C until metagenomic sequencing. DNA quality was checked prior to sequency. Animals were slaughtered in a commercial abattoir where two samples of rumen digesta (~ 50 ml) were collected immediately after the rumen was opened to be drained. Due to the slaughter process, in which the carcass is hung upside down and continuously moved along the chain and the rumen is removed, the rumen contents collected post-mortem are expected to be very well mixed, as opposed to being organized in layers (gas cap, fibrous mat and liquid) like in live animals. Filtering the rumen digesta through muslin allows to separate large feed particles out and to keep small particles, with associated microbes, in the filtrate^53,54^. The post-mortem samples were stored and processed in the same manner as described for rumen samples taken from live animals.

### Whole metagenomic sequencing

DNA was extracted from the samples of rumen digesta (0.25g) obtained from 20 steers following the methodology described in Yu and Morrison^55^. Illumina TruSeq libraries were prepared from genomic DNA and sequenced on Illumina HiSeq systems 4000 by Edinburgh Genomics (Edinburgh, UK). Paired-end reads (2 × 150 bp) were generated, resulting in between 16 and 42 GB per sample (between 55 and 140 million paired reads). The KOunt pipeline (https://github.com/WatsonLab/KOunt) was used to measure the abundance of known functional MG. Whole metagenome sequencing reads were quality trimmed using Fastp^56^ and assembled using MEGAHIT^57^. Proteins were predicted using Prodigal^58^ and searched against the Kyoto Encyclopedia of Genes and Genomes (KEGG) database (https://www.genome.jp/kegg/ko.html) (version 2020-10-04)^59^ using KofamScan database^60^. Hits that passed KofamScan’s default thresholds were assigned to KEGG orthologs (KOs). Proteins that passed the threshold for multiple KOs were grouped separately, as were those that did not have a hit. The resulting KO grouping corresponded to a highly similar group of sequences. For phylogenetic annotation of rumen samples, the same pipeline as described in Martínez-Álvaro et al.^22^ was applied. Briefly, the sequence reads of the samples were aligned to the Kraken database^27^ including both genomes of organisms from the Hungate 1000 collection^28^ (which only includes cultured rumen microbiota) and those of the NCBI Refseq database^29^.

### Statistical analyses

#### Data cleaning and transformation

The microbiome data included a total of 1178 MT and 6916 MG, which were used, without any restriction regarding prevalence or abundance, in the diversity analyses. For all other analyses, MT and MG were removed from the dataset if they were absent from at least one animal and/or if they had average relative abundance lower than 0.001% because analyses including low abundant microbiota are associated with increased noise making it difficult to identify relevant differences and relationships, show higher risk to be influenced by random fluctuations rather than meaningful biological variation, are more susceptible to taxonomic misclassification, as a consequence leading to less reliable and robust results.

To deal with the compositional nature of microbiome data, the MG abundances were transformed using the additive logratio (ALR^61^) with denominator *K01783*. This MG was identified as an appropriate denominator for our dataset based on the high Procrustes correlation between the ALR-transformed abundances (ALR-As) and the centred logratio-transformed matrix (CLR, ensuring isometry between samples), and the lowest variance in the denominator (simplifying the interpretation of results), according to the criteria proposed in Greenacre et al.^62^, and previously applied in Martínez-Álvaro et al.^22,26^. The MT abundances were CLR-transformed because we did not find a suitable microbial genus fulfilling the above criteria to act as denominator for the ALR transformation.

#### Diversity analyses

To investigate the temporal stability of the samples in terms of diversity within sample, and dissimilarity from other samples, we estimated alpha diversity (Shannon index) and beta diversity (Bray–Curtis dissimilarity) using the vegan package^63^ in R. In these analyses, we used the original datasets (in absolute counts), and analysed separately datasets of 1178 MT and 6916 MG.

The original Shannon index evaluates evenness in each sample (i.e., each animal in each timepoint) and it is influenced by the richness of the sample (the number of features extant in the sample). To account for different sample richness, we adjusted the Shannon index of each sample by dividing it by the maximum Shannon index possible for that sample (i.e., Shannon index of maximum evenness for the sample’s richness)^64^. Then, to evaluate the impact of timepoint on the adjusted Shannon indices, we fitted a linear mixed model, including as dependent variable the adjusted Shannon indices and as explanatory variables the fixed effect timepoint and the random effect animal (with *N*(0, Iσ^2^_s_), where σ^2^_s_ is the variance associated with repeated estimates of Shannon indices within animal and I the identity matrix of order 20). The *p* value of the fixed effect was calculated by Wald chisquare test. Significance was assessed at *p* value < 0.05. When significant effect of timepoint was observed, post hoc tests (least squares means) were used to identify which samples differed. These analyses were performed using the lme4^65^, car^66^ and emmeans^67^ packages in R.

Bray–Curtis dissimilarities were estimated between each sample in each timepoint. Then, the effect of timepoint was evaluated in a PERMANOVA with timepoint included as fixed effect. Significance was assessed at *p* value < 0.05. When significant effects were observed, a post hoc pairwise comparison was performed. Heteroscedasticity was also tested, however, no significant differences in dispersion of samples was observed. These analyses were performed using the vegan^63^ and the pairwiseAdonis^68^ packages in R. Additionally, to allow for the visualization of the similarity between samples, Bray–Curtis dissimilarities were projected using non-metric multi-dimensional scaling (NMDS), with random seeds set at 10403 and 432 (first and second elements of the .Random.seed vector in R) for MT and MG level analyses, respectively.

#### Temporal stability of microbial genera and microbial genes throughout the growing-finishing period

To evaluate the influence of timepoint on MT or MG abundances, we fitted, for each MT and MG, two different mixed models. The first model included diet, breed and timepoint as fixed effects and animal as random effect, assumed to be normally distributed as *N*(0, Iσ^2^_w_) where σ^2^_w_ is the variance associated with repeated observations of MT or MG within animal and I the identity matrix of order 20. The second model included the same effects except for timepoint. We compared these models using ANOVA and extracted the *p* value and the BIC of each model. The inclusion of feed additives in the diets was also tested following the same method; the first of these models included the fixed effects diet, feed additives, breed and timepoints and the random effect animal ID, whereas the second model included the same effects except for the fixed effect feed additives. We compared these models in an ANOVA and extracted the *p* value and BIC of each model. In both cases (testing for (i) timepoint and (ii) feed additives) we adjusted the *p* values based on the Bonferroni correction. Significance was assessed at *p* value < 0.05. The feed additives were found to have only an effect on a few centred log-ratio transformed MT abundances (MT-CLR) and additive log-ratio transformed abundances (MG-ALR), as the comparison of models including and excluding feed additives (fixed effect) revealed that only 10 out of 1050 MT and only 16 out of 1902 MG were better described by the model including feed additives.

To estimate the repeatability of each MT or MG we fitted the animal as random effect with *N*(0, Iσ^2^_w_) and diet and timepoint were modelled as fixed effects. Although timepoint was not significant, we kept this effect in the model to avoid any even small temporary impacts on the repeatability. Significance was assessed at *p* value < 0.05. Further analyses were performed within diet, including animal as random effect and timepoint as fixed effect. We then compared the magnitude of the repeatability of MT and MG found to be significantly repeatable in both diets. The repeatability was calculated as σ^2^w / (σ^2^e + σ^2^w), applying the rptR^69^ package in R. Additionally, we compared the abundances generated from sampling MT and MG throughout the growing-finishing phase across both diets, and within diet. To achieve this, we centred the MT CLR-As and MG ALR-As prior to calculating the Pearson correlations between the vectorized matrices obtained from each timepoint in a pairwise manner.

#### Prediction ability of performance and methane emissions traits based on data generated from different timepoints

Partial least squares (PLS) models were used to understand which of the 1050 MT and 1901 MG were most associated with each trait in each timepoint. Traits considered in the PLS analyses were the residuals of FCR, ADG, DFI, RFI, daily CH_4_ emissions, and CH_4_ yield after adjustment for the animals’ weights as measured on the day they were allocated to the basal diet and the basal diet. The PLS analyses were performed using the mixOmics^70^ package in R. Variable importance in projection (VIP) scores were obtained for each explanatory variable in each predictive model using 2 components. Regression coefficients were used to assess the direction of the relationship between each predictor variable (MT or MG) and the trait.

#### Stability of the association of microbial genes with moderate to high heritability with the estimated breeding values of host performance traits

With the goal of evaluating the temporal stability of the microbiome features influenced by the host genetics throughout the growing-finishing phase, we aimed to predict breeding values of performance traits using PLS models based on the MG ALR-As available in the present dataset generated from sampling at each timepoint. In the analyses, 673 MG were used which were identified by Martínez-Álvaro et al.^22^ to be influenced by host genomics. These MG presented significant host-genomic effects after stringent multiple testing, estimated using a larger data set (n = 359 animals) with metagenomics and host genomic information (described in Martínez-Álvaro et al.^26^) which included the animals of this study. The EBVs of the performance traits FCR, ADG, DFI, RFI and CH4Y were estimated with 359 (FCR, ADG, DFI and RFI) and 285 (CH4Y) records. A univariate genomic BLUP model was fitted, including diet, breed and trial as fixed effects and the genetics of the animals as a random effect. The random effect was assumed to be normally distributed with mean 0 and variance equal to the genomic relationship (estimated by Van Raden method 2 based on 38,807 SNPs after standard filtering) multiplied by the genomic variance. Bayesian inference was used^71^, with bounded flat priors for all effects and variances. The models were solved using BGLR software^72^. A more detailed description of the statistical analysis (applied in different traits) can be found in Martínez-Álvaro et al.^26^.

All statistical analyses were performed in R v. 4.1.1 (August 2021).

## Supplementary Information

Supplementary Tables.

## Data Availability

The raw data can be downloaded from the European Nucleotide Archive under accession PRJEB21624. Host phenotypes, diets and breeds can be found in the Supplementary Table 6.
