# Supplementary material for: Temporal stability of the rumen microbiome and its longitudinal associations with performance traits in beef cattle
Source: Sci Rep. 2024 Sep 5;14:20772. doi: 10.1038/s41598-024-70770-3 (PMC11377694; doi:10.1038/s41598-024-70770-3)
Supplement: Supplementary file 1 — Supplementary Tables. [file 41598_2024_70770_MOESM1_ESM.pdf]

**Supplementary Table 1. Repeatability of abundances of 1050 microbial genera or 1901 microbial genes abundances over all animals or within animals fed concentrate and forage separately.**

| | Overall repeatability (Rpt) | | | Rpt within concentrate-fed animals | | | Rpt within forage fed-animals | | |
|---|---|---|---|---|---|---|---|---|---|
| Microbial genus/ microbial gene | Rpt | SE | p-value | Rpt | SE | p-value | Rpt | SE | p-value |
| *Acanthamoeba* BOTH | 0.281 | 0.102 | 0.01 | 0.270 | 0.122 | 0.010 | 0.292 | 0.181 | 0.010 |
| *Acidihalobacter* BOTH | 0.265 | 0.097 | 0.01 | 0.240 | 0.133 | 0.020 | 0.317 | 0.167 | 0.020 |
| *Actinomyces* BOTH | 0.222 | 0.106 | 0.01 | 0.231 | 0.133 | 0.010 | 0.199 | 0.146 | 0.040 |
| *Acytostelium* BOTH | 0.271 | 0.107 | 0.01 | 0.284 | 0.124 | 0.010 | 0.269 | 0.140 | 0.020 |
| *Aeromicrobium* BOTH | 0.347 | 0.112 | 0.01 | 0.332 | 0.145 | 0.010 | 0.354 | 0.178 | 0.010 |
| *Agrobacterium* BOTH | 0.304 | 0.107 | 0.01 | 0.300 | 0.109 | 0.010 | 0.274 | 0.156 | 0.020 |
| *Alcanivorax* BOTH | 0.200 | 0.096 | 0.02 | 0.170 | 0.102 | 0.040 | 0.278 | 0.165 | 0.020 |
| *Alkalilimnicola* BOTH | 0.211 | 0.092 | 0.01 | 0.200 | 0.119 | 0.020 | 0.252 | 0.158 | 0.020 |
| *Allisonella* BOTH | 0.210 | 0.095 | 0.01 | 0.189 | 0.127 | 0.020 | 0.266 | 0.154 | 0.010 |
| *Aminomonas* BOTH | 0.331 | 0.096 | 0.01 | 0.342 | 0.136 | 0.010 | 0.244 | 0.159 | 0.040 |
| *Anaerovibrio* BOTH | 0.429 | 0.121 | 0.01 | 0.429 | 0.133 | 0.010 | 0.459 | 0.170 | 0.010 |
| *Arthrobotrys* BOTH | 0.450 | 0.106 | 0.01 | 0.498 | 0.133 | 0.010 | 0.332 | 0.168 | 0.030 |
| *Aspergillus* BOTH | 0.428 | 0.091 | 0.01 | 0.512 | 0.142 | 0.010 | 0.303 | 0.166 | 0.020 |
| *Atopobium* BOTH | 0.226 | 0.106 | 0.02 | 0.253 | 0.131 | 0.010 | 0.236 | 0.157 | 0.020 |
| *Beauveria* BOTH | 0.354 | 0.117 | 0.01 | 0.394 | 0.155 | 0.010 | 0.245 | 0.150 | 0.030 |
| *Beijerinckia* BOTH | 0.280 | 0.094 | 0.01 | 0.256 | 0.130 | 0.010 | 0.343 | 0.168 | 0.010 |
| *Bifidobacterium* BOTH | 0.226 | 0.100 | 0.01 | 0.159 | 0.113 | 0.040 | 0.338 | 0.169 | 0.010 |
| *Blastocystis* BOTH | 0.428 | 0.122 | 0.01 | 0.471 | 0.149 | 0.010 | 0.334 | 0.178 | 0.010 |
| *Blastomyces* BOTH | 0.447 | 0.115 | 0.01 | 0.502 | 0.122 | 0.010 | 0.253 | 0.157 | 0.040 |
| *Bradyrhizobium* BOTH | 0.263 | 0.108 | 0.01 | 0.230 | 0.119 | 0.020 | 0.440 | 0.157 | 0.010 |
| *Burkholderia* BOTH | 0.243 | 0.113 | 0.01 | 0.252 | 0.120 | 0.010 | 0.220 | 0.166 | 0.020 |
| *Candida* BOTH | 0.482 | 0.117 | 0.01 | 0.531 | 0.145 | 0.010 | 0.339 | 0.177 | 0.020 |
| *Candidatus Methylopumilus* BOTH | 0.247 | 0.103 | 0.01 | 0.232 | 0.127 | 0.010 | 0.256 | 0.146 | 0.010 |
| *Candidatus Phytoplasma* BOTH | 0.300 | 0.110 | 0.01 | 0.297 | 0.127 | 0.010 | 0.296 | 0.148 | 0.040 |
| *Candidatus Protochlamydia* BOTH | 0.370 | 0.086 | 0.01 | 0.374 | 0.144 | 0.010 | 0.346 | 0.169 | 0.020 |
| *Candidatus Sulcia* BOTH | 0.177 | 0.106 | 0.01 | 0.153 | 0.107 | 0.040 | 0.193 | 0.129 | 0.040 |
| *Chaetomium* BOTH | 0.367 | 0.108 | 0.01 | 0.418 | 0.123 | 0.010 | 0.300 | 0.174 | 0.020 |
| *Clavispora* BOTH | 0.433 | 0.099 | 0.01 | 0.453 | 0.132 | 0.010 | 0.388 | 0.174 | 0.010 |
| *Coccidioides* BOTH | 0.441 | 0.114 | 0.01 | 0.511 | 0.137 | 0.010 | 0.270 | 0.163 | 0.020 |
| *Cordyceps* BOTH | 0.308 | 0.105 | 0.01 | 0.325 | 0.124 | 0.010 | 0.253 | 0.157 | 0.040 |
| *Cryptosporidium* BOTH | 0.447 | 0.110 | 0.01 | 0.472 | 0.148 | 0.010 | 0.372 | 0.188 | 0.020 |
| *Curtobacterium* BOTH | 0.298 | 0.099 | 0.01 | 0.317 | 0.133 | 0.010 | 0.288 | 0.148 | 0.010 |
| *Dactylellina* BOTH | 0.427 | 0.103 | 0.01 | 0.472 | 0.126 | 0.010 | 0.316 | 0.171 | 0.030 |
| *Dehalogenimonas* BOTH | 0.211 | 0.101 | 0.01 | 0.181 | 0.112 | 0.020 | 0.320 | 0.153 | 0.010 |
| *Dermacoccus* BOTH | 0.244 | 0.104 | 0.01 | 0.263 | 0.120 | 0.020 | 0.234 | 0.173 | 0.040 |
| *Desulfitobacterium* BOTH | 0.276 | 0.100 | 0.01 | 0.219 | 0.128 | 0.020 | 0.385 | 0.159 | 0.020 |

| | | | | | | | | | |
|---|---|---|---|---|---|---|---|---|---|
| *Desulfobacterium* BOTH | 0.210 | 0.086 | 0.02 | 0.178 | 0.110 | 0.030 | 0.306 | 0.167 | 0.020 |
| *Dictyoglomus* BOTH | 0.226 | 0.096 | 0.01 | 0.202 | 0.127 | 0.010 | 0.250 | 0.142 | 0.020 |
| *Dictyostelium* BOTH | 0.426 | 0.108 | 0.01 | 0.482 | 0.135 | 0.010 | 0.309 | 0.150 | 0.020 |
| *Entamoeba* BOTH | 0.455 | 0.098 | 0.01 | 0.531 | 0.139 | 0.010 | 0.321 | 0.163 | 0.010 |
| *Enterocytozoon* BOTH | 0.291 | 0.113 | 0.01 | 0.345 | 0.142 | 0.010 | 0.223 | 0.130 | 0.030 |
| *Eremothecium* BOTH | 0.278 | 0.097 | 0.01 | 0.257 | 0.143 | 0.020 | 0.330 | 0.165 | 0.010 |
| *Eutypa* BOTH | 0.430 | 0.122 | 0.01 | 0.519 | 0.129 | 0.010 | 0.270 | 0.161 | 0.040 |
| *Exiguobacterium* BOTH | 0.282 | 0.097 | 0.01 | 0.273 | 0.125 | 0.010 | 0.261 | 0.153 | 0.020 |
| *Faecalibaculum* BOTH | 0.223 | 0.106 | 0.01 | 0.203 | 0.120 | 0.010 | 0.281 | 0.161 | 0.010 |
| *Fibrobacter* BOTH | 0.203 | 0.091 | 0.02 | 0.197 | 0.113 | 0.040 | 0.200 | 0.146 | 0.040 |
| *Fomitiporia* BOTH | 0.205 | 0.095 | 0.01 | 0.172 | 0.117 | 0.030 | 0.291 | 0.158 | 0.030 |
| *Fonsecaea* BOTH | 0.555 | 0.111 | 0.01 | 0.462 | 0.119 | 0.010 | 0.716 | 0.145 | 0.010 |
| *Geoalkalibacter* BOTH | 0.205 | 0.100 | 0.02 | 0.188 | 0.122 | 0.020 | 0.315 | 0.171 | 0.010 |
| *Gloeobacter* BOTH | 0.276 | 0.101 | 0.01 | 0.255 | 0.121 | 0.010 | 0.338 | 0.181 | 0.010 |
| *Guillardia* BOTH | 0.249 | 0.100 | 0.01 | 0.286 | 0.135 | 0.010 | 0.250 | 0.173 | 0.040 |
| *Halapricum* BOTH | 0.746 | 0.082 | 0.01 | 0.733 | 0.112 | 0.010 | 0.797 | 0.111 | 0.010 |
| *Halococcus* BOTH | 0.706 | 0.098 | 0.01 | 0.612 | 0.132 | 0.010 | 0.806 | 0.112 | 0.010 |
| *Halocynthiibacter* BOTH | 0.392 | 0.103 | 0.01 | 0.403 | 0.143 | 0.010 | 0.389 | 0.177 | 0.010 |
| *Halorhabdus* BOTH | 0.329 | 0.103 | 0.01 | 0.347 | 0.131 | 0.010 | 0.231 | 0.158 | 0.010 |
| *Halothiobacillus* BOTH | 0.286 | 0.105 | 0.01 | 0.256 | 0.131 | 0.020 | 0.443 | 0.161 | 0.010 |
| *Helicobacter* BOTH | 0.205 | 0.091 | 0.01 | 0.185 | 0.119 | 0.020 | 0.220 | 0.148 | 0.040 |
| *Heliobacterium* BOTH | 0.247 | 0.091 | 0.01 | 0.193 | 0.115 | 0.010 | 0.370 | 0.165 | 0.010 |
| *Histoplasma* BOTH | 0.444 | 0.106 | 0.01 | 0.499 | 0.120 | 0.010 | 0.308 | 0.156 | 0.010 |
| *Ichthyophthirius* BOTH | 0.442 | 0.115 | 0.01 | 0.496 | 0.130 | 0.010 | 0.306 | 0.178 | 0.030 |
| *Kandleria* BOTH | 0.306 | 0.103 | 0.01 | 0.373 | 0.135 | 0.010 | 0.246 | 0.151 | 0.040 |
| *Komagataella* BOTH | 0.218 | 0.094 | 0.01 | 0.236 | 0.142 | 0.030 | 0.204 | 0.142 | 0.020 |
| *Kwoniella* BOTH | 0.374 | 0.110 | 0.01 | 0.368 | 0.137 | 0.010 | 0.374 | 0.171 | 0.010 |
| *Lachnoclostridium* BOTH | 0.263 | 0.102 | 0.01 | 0.230 | 0.122 | 0.040 | 0.314 | 0.170 | 0.010 |
| *Mageeibacillus* BOTH | 0.214 | 0.095 | 0.01 | 0.219 | 0.119 | 0.030 | 0.213 | 0.142 | 0.030 |
| *Melampsora* BOTH | 0.375 | 0.110 | 0.01 | 0.409 | 0.127 | 0.010 | 0.234 | 0.134 | 0.030 |
| *Methanobacterium* BOTH | 0.612 | 0.100 | 0.01 | 0.596 | 0.124 | 0.010 | 0.636 | 0.140 | 0.010 |
| *Methanocaldococcus* BOTH | 0.258 | 0.107 | 0.01 | 0.243 | 0.120 | 0.010 | 0.296 | 0.187 | 0.030 |
| *Methanocella* BOTH | 0.344 | 0.103 | 0.01 | 0.340 | 0.121 | 0.010 | 0.365 | 0.162 | 0.030 |
| *Methanofollis* BOTH | 0.198 | 0.087 | 0.01 | 0.190 | 0.128 | 0.030 | 0.282 | 0.162 | 0.020 |
| *Methanoregula* BOTH | 0.256 | 0.116 | 0.01 | 0.277 | 0.121 | 0.010 | 0.269 | 0.159 | 0.010 |
| *Methanothermobacter* BOTH | 0.625 | 0.093 | 0.01 | 0.489 | 0.142 | 0.010 | 0.808 | 0.143 | 0.010 |
| *Methanotorris* BOTH | 0.222 | 0.085 | 0.01 | 0.203 | 0.142 | 0.020 | 0.232 | 0.156 | 0.030 |
| *Millerozyma* BOTH | 0.231 | 0.096 | 0.01 | 0.235 | 0.124 | 0.010 | 0.266 | 0.159 | 0.040 |
| *Murdochiella* BOTH | 0.423 | 0.112 | 0.01 | 0.433 | 0.145 | 0.010 | 0.324 | 0.170 | 0.010 |
| *Naegleria* BOTH | 0.496 | 0.102 | 0.01 | 0.526 | 0.140 | 0.010 | 0.372 | 0.157 | 0.020 |
| *Negativicoccus* BOTH | 0.792 | 0.064 | 0.01 | 0.704 | 0.100 | 0.010 | 0.844 | 0.102 | 0.010 |
| *Neofusicoccum* BOTH | 0.223 | 0.095 | 0.01 | 0.202 | 0.119 | 0.040 | 0.325 | 0.155 | 0.010 |
| *Neospora* BOTH | 0.223 | 0.101 | 0.01 | 0.195 | 0.112 | 0.020 | 0.303 | 0.180 | 0.020 |
| *Neurospora* BOTH | 0.352 | 0.092 | 0.01 | 0.406 | 0.146 | 0.010 | 0.256 | 0.155 | 0.040 |
| *Nitrosopumilus* BOTH | 0.208 | 0.097 | 0.02 | 0.210 | 0.115 | 0.020 | 0.217 | 0.157 | 0.020 |

| | | | | | | | | | |
|---|---|---|---|---|---|---|---|---|---|
| *Nosema* BOTH | 0.450 | 0.115 | 0.01 | 0.470 | 0.148 | 0.010 | 0.348 | 0.171 | 0.010 |
| *Olsenella* BOTH | 0.415 | 0.112 | 0.01 | 0.407 | 0.124 | 0.010 | 0.427 | 0.166 | 0.010 |
| *Paenibacillus* BOTH | 0.320 | 0.121 | 0.01 | 0.324 | 0.139 | 0.010 | 0.314 | 0.165 | 0.020 |
| *Pantoea* BOTH | 0.220 | 0.102 | 0.01 | 0.215 | 0.113 | 0.020 | 0.208 | 0.141 | 0.040 |
| *Paracoccidioides* BOTH | 0.413 | 0.099 | 0.01 | 0.461 | 0.123 | 0.010 | 0.332 | 0.152 | 0.020 |
| *Paramecium* BOTH | 0.423 | 0.114 | 0.01 | 0.444 | 0.137 | 0.010 | 0.348 | 0.169 | 0.010 |
| *Paucibacter* BOTH | 0.275 | 0.105 | 0.01 | 0.283 | 0.130 | 0.010 | 0.249 | 0.153 | 0.020 |
| *Pectobacterium* BOTH | 0.429 | 0.106 | 0.01 | 0.296 | 0.147 | 0.010 | 0.529 | 0.177 | 0.010 |
| *Perkinsus* BOTH | 0.422 | 0.111 | 0.01 | 0.468 | 0.132 | 0.010 | 0.333 | 0.142 | 0.010 |
| *Phaeoacremonium* BOTH | 0.279 | 0.113 | 0.01 | 0.246 | 0.136 | 0.010 | 0.398 | 0.183 | 0.010 |
| *Phialocephala* BOTH | 0.378 | 0.099 | 0.01 | 0.430 | 0.138 | 0.010 | 0.321 | 0.160 | 0.020 |
| *Phycomyces* BOTH | 0.414 | 0.104 | 0.01 | 0.487 | 0.132 | 0.010 | 0.281 | 0.171 | 0.040 |
| *Pichia* BOTH | 0.431 | 0.123 | 0.01 | 0.435 | 0.145 | 0.010 | 0.410 | 0.161 | 0.010 |
| *Planktothrix* BOTH | 0.389 | 0.097 | 0.01 | 0.348 | 0.136 | 0.010 | 0.512 | 0.165 | 0.010 |
| *Plasmodium* BOTH | 0.454 | 0.123 | 0.01 | 0.528 | 0.121 | 0.010 | 0.269 | 0.173 | 0.020 |
| *Pluralibacter* BOTH | 0.277 | 0.104 | 0.01 | 0.274 | 0.140 | 0.010 | 0.270 | 0.170 | 0.010 |
| *Pneumocystis* BOTH | 0.407 | 0.116 | 0.01 | 0.437 | 0.134 | 0.010 | 0.316 | 0.144 | 0.010 |
| *Proteiniclasticum* BOTH | 0.305 | 0.109 | 0.01 | 0.299 | 0.142 | 0.010 | 0.284 | 0.159 | 0.010 |
| *Pseudogymnoascus* BOTH | 0.252 | 0.098 | 0.01 | 0.258 | 0.128 | 0.010 | 0.256 | 0.142 | 0.030 |
| *Puccinia* BOTH | 0.357 | 0.103 | 0.01 | 0.438 | 0.130 | 0.010 | 0.241 | 0.161 | 0.040 |
| *Rathayibacter* BOTH | 0.387 | 0.113 | 0.01 | 0.410 | 0.133 | 0.010 | 0.284 | 0.160 | 0.020 |
| *Rhinocladiella* BOTH | 0.307 | 0.103 | 0.01 | 0.342 | 0.131 | 0.010 | 0.258 | 0.156 | 0.030 |
| *Rhizobium* BOTH | 0.223 | 0.093 | 0.01 | 0.190 | 0.128 | 0.010 | 0.349 | 0.183 | 0.010 |
| *Roseburia* BOTH | 0.302 | 0.098 | 0.01 | 0.313 | 0.130 | 0.010 | 0.257 | 0.152 | 0.020 |
| *Saccharomyces* BOTH | 0.349 | 0.093 | 0.01 | 0.391 | 0.136 | 0.010 | 0.241 | 0.156 | 0.010 |
| *Saccharophagus* BOTH | 0.243 | 0.103 | 0.01 | 0.232 | 0.119 | 0.030 | 0.220 | 0.157 | 0.040 |
| *Salinispira* BOTH | 0.255 | 0.099 | 0.01 | 0.246 | 0.132 | 0.010 | 0.261 | 0.167 | 0.040 |
| *Sanguibacter* BOTH | 0.222 | 0.094 | 0.01 | 0.213 | 0.122 | 0.030 | 0.246 | 0.142 | 0.040 |
| *Scedosporium* BOTH | 0.321 | 0.098 | 0.01 | 0.333 | 0.127 | 0.010 | 0.313 | 0.158 | 0.020 |
| *Schizosaccharomyces* BOTH | 0.386 | 0.107 | 0.01 | 0.420 | 0.139 | 0.010 | 0.362 | 0.152 | 0.020 |
| *Setosphaeria* BOTH | 0.336 | 0.114 | 0.01 | 0.346 | 0.131 | 0.010 | 0.338 | 0.163 | 0.010 |
| *Spathaspora* BOTH | 0.403 | 0.105 | 0.01 | 0.435 | 0.136 | 0.010 | 0.337 | 0.178 | 0.030 |
| *Sphaerulina* BOTH | 0.326 | 0.107 | 0.01 | 0.351 | 0.140 | 0.010 | 0.297 | 0.150 | 0.020 |
| *Sphingobium* BOTH | 0.213 | 0.100 | 0.01 | 0.224 | 0.124 | 0.020 | 0.173 | 0.155 | 0.040 |
| *Sphingopyxis* BOTH | 0.281 | 0.109 | 0.01 | 0.286 | 0.140 | 0.020 | 0.250 | 0.148 | 0.020 |
| *Spizellomyces* BOTH | 0.366 | 0.108 | 0.01 | 0.376 | 0.137 | 0.010 | 0.324 | 0.148 | 0.020 |
| *Sporothrix* BOTH | 0.302 | 0.110 | 0.01 | 0.268 | 0.135 | 0.020 | 0.366 | 0.166 | 0.020 |
| *Stereum* BOTH | 0.209 | 0.094 | 0.01 | 0.207 | 0.113 | 0.030 | 0.305 | 0.154 | 0.010 |
| *Sugiyamaella* BOTH | 0.373 | 0.113 | 0.01 | 0.388 | 0.125 | 0.010 | 0.351 | 0.188 | 0.010 |
| *Sulfurovum* BOTH | 0.258 | 0.105 | 0.01 | 0.245 | 0.126 | 0.010 | 0.325 | 0.189 | 0.020 |
| *Synechococcus* BOTH | 0.349 | 0.109 | 0.01 | 0.362 | 0.131 | 0.010 | 0.295 | 0.154 | 0.010 |
| *Synechocystis* BOTH | 0.212 | 0.093 | 0.01 | 0.193 | 0.120 | 0.040 | 0.356 | 0.170 | 0.020 |
| *Syntrophomonas* BOTH | 0.194 | 0.098 | 0.01 | 0.192 | 0.133 | 0.040 | 0.233 | 0.156 | 0.040 |
| *Tetrahymena* BOTH | 0.451 | 0.116 | 0.01 | 0.497 | 0.148 | 0.010 | 0.334 | 0.172 | 0.010 |
| *Tetrapisispora* BOTH | 0.367 | 0.098 | 0.01 | 0.407 | 0.127 | 0.010 | 0.253 | 0.155 | 0.030 |

| | | | | | | | | | |
|---|---|---|---|---|---|---|---|---|---|
| *Theileria* BOTH | 0.406 | 0.112 | 0.01 | 0.443 | 0.142 | 0.010 | 0.336 | 0.171 | 0.030 |
| *Thielavia* BOTH | 0.201 | 0.104 | 0.01 | 0.227 | 0.123 | 0.030 | 0.240 | 0.159 | 0.020 |
| *Toxoplasma* BOTH | 0.320 | 0.092 | 0.01 | 0.325 | 0.132 | 0.010 | 0.344 | 0.164 | 0.010 |
| *Trichoderma* BOTH | 0.321 | 0.103 | 0.01 | 0.366 | 0.139 | 0.010 | 0.270 | 0.160 | 0.030 |
| *Trichodesmium* BOTH | 0.407 | 0.115 | 0.01 | 0.442 | 0.144 | 0.010 | 0.271 | 0.157 | 0.020 |
| *Trichophyton* BOTH | 0.416 | 0.106 | 0.01 | 0.492 | 0.135 | 0.010 | 0.270 | 0.140 | 0.020 |
| *Trueperella* BOTH | 0.223 | 0.095 | 0.01 | 0.179 | 0.117 | 0.030 | 0.376 | 0.161 | 0.010 |
| *Trypanosoma* BOTH | 0.440 | 0.113 | 0.01 | 0.516 | 0.118 | 0.010 | 0.292 | 0.144 | 0.030 |
| *Tuber* BOTH | 0.410 | 0.110 | 0.01 | 0.478 | 0.131 | 0.010 | 0.303 | 0.156 | 0.030 |
| *Vanderwaltozyma* BOTH | 0.408 | 0.118 | 0.01 | 0.415 | 0.119 | 0.010 | 0.376 | 0.180 | 0.010 |
| *Verruconis* BOTH | 0.268 | 0.107 | 0.01 | 0.306 | 0.130 | 0.020 | 0.243 | 0.172 | 0.040 |
| *Wallemia* BOTH | 0.463 | 0.118 | 0.01 | 0.495 | 0.121 | 0.010 | 0.386 | 0.180 | 0.010 |
| *Wickerhamomyces* BOTH | 0.464 | 0.113 | 0.01 | 0.500 | 0.149 | 0.010 | 0.373 | 0.159 | 0.020 |
| *Xanthomonas* BOTH | 0.362 | 0.106 | 0.01 | 0.346 | 0.125 | 0.010 | 0.364 | 0.172 | 0.020 |
| *Xylona* BOTH | 0.372 | 0.113 | 0.01 | 0.391 | 0.138 | 0.010 | 0.286 | 0.179 | 0.030 |
| *Yamadazyma* BOTH | 0.313 | 0.092 | 0.01 | 0.327 | 0.130 | 0.010 | 0.233 | 0.142 | 0.030 |
| *Yangia* BOTH | 0.229 | 0.085 | 0.01 | 0.187 | 0.116 | 0.030 | 0.291 | 0.165 | 0.010 |
| *Zygosaccharomyces* BOTH | 0.439 | 0.108 | 0.01 | 0.485 | 0.138 | 0.010 | 0.313 | 0.167 | 0.010 |
| *Acaryochloris* CON | 0.271 | 0.107 | 0.01 | 0.306 | 0.128 | 0.010 | 0.028 | 0.090 | 0.340 |
| *Acetohalobium* CON | 0.150 | 0.102 | 0.02 | 0.246 | 0.127 | 0.010 | 0.000 | 0.000 | 0.310 |
| *Acetomicrobium* CON | 0.244 | 0.116 | 0.01 | 0.269 | 0.130 | 0.010 | 0.024 | 0.089 | 0.440 |
| *Achromobacter* CON | 0.141 | 0.084 | 0.01 | 0.232 | 0.127 | 0.020 | 0.000 | 0.071 | 1.000 |
| *Acidaminococcus* CON | 0.314 | 0.118 | 0.01 | 0.318 | 0.139 | 0.010 | 0.289 | 0.163 | 0.050 |
| *Acidothermus* CON | 0.335 | 0.109 | 0.01 | 0.381 | 0.145 | 0.010 | 0.102 | 0.121 | 0.190 |
| *Acidovorax* CON | 0.266 | 0.100 | 0.01 | 0.360 | 0.136 | 0.010 | 0.028 | 0.081 | 0.460 |
| *Acinetobacter* CON | 0.041 | 0.058 | 0.27 | 0.173 | 0.115 | 0.040 | 0.000 | 0.072 | 1.000 |
| *Adlercreutzia* CON | 0.253 | 0.106 | 0.01 | 0.327 | 0.138 | 0.010 | 0.016 | 0.086 | 0.490 |
| *Aerococcus* CON | 0.235 | 0.096 | 0.01 | 0.285 | 0.126 | 0.010 | 0.074 | 0.111 | 0.280 |
| *Agaricus* CON | 0.254 | 0.116 | 0.01 | 0.297 | 0.141 | 0.010 | 0.181 | 0.147 | 0.090 |
| *Agarivorans* CON | 0.232 | 0.099 | 0.01 | 0.294 | 0.129 | 0.010 | 0.000 | 0.086 | 1.000 |
| *Agromyces* CON | 0.293 | 0.103 | 0.01 | 0.320 | 0.142 | 0.020 | 0.194 | 0.140 | 0.070 |
| *Alicycliphilus* CON | 0.179 | 0.088 | 0.01 | 0.229 | 0.119 | 0.020 | 0.000 | 0.000 | 0.990 |
| *Alicyclobacillus* CON | 0.237 | 0.105 | 0.02 | 0.257 | 0.145 | 0.010 | 0.095 | 0.093 | 0.290 |
| *Allochromatium* CON | 0.118 | 0.077 | 0.07 | 0.168 | 0.117 | 0.030 | 0.000 | 0.059 | 1.000 |
| *Altererythrobacter* CON | 0.183 | 0.097 | 0.04 | 0.246 | 0.138 | 0.010 | 0.001 | 0.068 | 0.560 |
| *Anabaena* CON | 0.286 | 0.106 | 0.01 | 0.310 | 0.130 | 0.010 | 0.180 | 0.154 | 0.050 |
| *Anaeromyxobacter* CON | 0.214 | 0.090 | 0.02 | 0.292 | 0.123 | 0.010 | 0.000 | 0.069 | 1.000 |
| *Aphanomyces* CON | 0.125 | 0.084 | 0.07 | 0.166 | 0.108 | 0.020 | 0.000 | 0.061 | 1.000 |
| *Archangium* CON | 0.214 | 0.092 | 0.01 | 0.246 | 0.138 | 0.010 | 0.134 | 0.125 | 0.190 |
| *Aromatoleum* CON | 0.185 | 0.078 | 0.02 | 0.210 | 0.113 | 0.020 | 0.000 | 0.084 | 1.000 |
| *Arsenicicoccus* CON | 0.148 | 0.084 | 0.02 | 0.185 | 0.126 | 0.020 | 0.059 | 0.111 | 0.330 |
| *Arsenophonus* CON | 0.257 | 0.101 | 0.01 | 0.303 | 0.127 | 0.010 | 0.114 | 0.127 | 0.180 |
| *Arthrobacter* CON | 0.239 | 0.095 | 0.01 | 0.252 | 0.123 | 0.020 | 0.184 | 0.152 | 0.090 |
| *Asticcacaulis* CON | 0.154 | 0.076 | 0.04 | 0.180 | 0.121 | 0.010 | 0.000 | 0.065 | 1.000 |
| *Aureimonas* CON | 0.172 | 0.091 | 0.01 | 0.208 | 0.127 | 0.010 | 0.017 | 0.069 | 0.490 |
| *Azoarcus* CON | 0.185 | 0.096 | 0.03 | 0.197 | 0.126 | 0.010 | 0.065 | 0.110 | 0.230 |

| | | | | | | | | | |
|---|---|---|---|---|---|---|---|---|---|
| *Azorhizobium* CON | 0.266 | 0.109 | 0.01 | 0.283 | 0.130 | 0.010 | 0.004 | 0.058 | 0.580 |
| *Azospira* CON | 0.211 | 0.093 | 0.01 | 0.244 | 0.108 | 0.040 | 0.000 | 0.062 | 1.000 |
| *Azospirillum* CON | 0.243 | 0.096 | 0.01 | 0.275 | 0.128 | 0.010 | 0.113 | 0.128 | 0.150 |
| *Babjeviella* CON | 0.170 | 0.096 | 0.02 | 0.188 | 0.125 | 0.040 | 0.163 | 0.127 | 0.080 |
| *Basfia* CON | 0.195 | 0.084 | 0.01 | 0.281 | 0.141 | 0.010 | 0.045 | 0.093 | 0.370 |
| *Bernardetia* CON | 0.140 | 0.084 | 0.03 | 0.171 | 0.106 | 0.030 | 0.000 | 0.063 | 1.000 |
| *Bibersteinia* CON | 0.162 | 0.097 | 0.04 | 0.168 | 0.129 | 0.030 | 0.087 | 0.111 | 0.290 |
| *Bipolaris* CON | 0.197 | 0.099 | 0.01 | 0.202 | 0.118 | 0.030 | 0.121 | 0.110 | 0.160 |
| *Blattabacterium* CON | 0.282 | 0.103 | 0.01 | 0.315 | 0.128 | 0.010 | 0.133 | 0.126 | 0.230 |
| *Blautia* CON | 0.232 | 0.094 | 0.01 | 0.246 | 0.137 | 0.010 | 0.203 | 0.149 | 0.060 |
| *Bordetella* CON | 0.284 | 0.125 | 0.01 | 0.313 | 0.124 | 0.010 | 0.197 | 0.148 | 0.070 |
| *Borrelia* CON | 0.153 | 0.085 | 0.02 | 0.223 | 0.124 | 0.010 | 0.055 | 0.116 | 0.300 |
| *Brachyspira* CON | 0.234 | 0.095 | 0.01 | 0.314 | 0.137 | 0.010 | 0.152 | 0.138 | 0.140 |
| *Brevundimonas* CON | 0.249 | 0.105 | 0.01 | 0.291 | 0.135 | 0.020 | 0.077 | 0.114 | 0.260 |
| *Caldilinea* CON | 0.155 | 0.086 | 0.03 | 0.154 | 0.124 | 0.030 | 0.105 | 0.121 | 0.160 |
| *Caldisericum* CON | 0.196 | 0.087 | 0.01 | 0.226 | 0.118 | 0.030 | 0.036 | 0.096 | 0.410 |
| *Calditerrivibrio* CON | 0.177 | 0.091 | 0.02 | 0.232 | 0.121 | 0.020 | 0.019 | 0.089 | 0.540 |
| *Calothrix* CON | 0.409 | 0.109 | 0.01 | 0.461 | 0.123 | 0.010 | 0.172 | 0.136 | 0.070 |
| *Campylobacter* CON | 0.250 | 0.115 | 0.01 | 0.326 | 0.140 | 0.010 | 0.090 | 0.106 | 0.190 |
| *Candidatus Methanomethylophilus* CON | 0.289 | 0.102 | 0.01 | 0.347 | 0.114 | 0.010 | 0.000 | 0.074 | 1.000 |
| *Candidatus Methanoperedens* CON | 0.310 | 0.104 | 0.01 | 0.340 | 0.142 | 0.010 | 0.198 | 0.146 | 0.070 |
| *Candidatus Phaeomarinobacter* CON | 0.137 | 0.089 | 0.06 | 0.176 | 0.115 | 0.030 | 0.000 | 0.068 | 1.000 |
| *Candidatus Solibacter* CON | 0.173 | 0.094 | 0.02 | 0.231 | 0.124 | 0.020 | 0.000 | 0.085 | 1.000 |
| *Capronia* CON | 0.162 | 0.081 | 0.01 | 0.160 | 0.109 | 0.040 | 0.108 | 0.122 | 0.180 |
| *Carboxydothermus* CON | 0.137 | 0.081 | 0.04 | 0.226 | 0.134 | 0.020 | 0.000 | 0.069 | 1.000 |
| *Castellaniella* CON | 0.179 | 0.092 | 0.02 | 0.212 | 0.131 | 0.020 | 0.000 | 0.086 | 1.000 |
| *Caulobacter* CON | 0.287 | 0.093 | 0.01 | 0.328 | 0.109 | 0.010 | 0.159 | 0.120 | 0.100 |
| *Cellulomonas* CON | 0.262 | 0.100 | 0.01 | 0.352 | 0.135 | 0.010 | 0.000 | 0.083 | 1.000 |
| *Chamaesiphon* CON | 0.145 | 0.082 | 0.02 | 0.145 | 0.108 | 0.040 | 0.071 | 0.100 | 0.390 |
| *Chelativorans* CON | 0.177 | 0.090 | 0.02 | 0.184 | 0.119 | 0.030 | 0.044 | 0.096 | 0.390 |
| *Chelatococcus* CON | 0.217 | 0.100 | 0.01 | 0.264 | 0.121 | 0.010 | 0.000 | 0.078 | 1.000 |
| *Chlorobaculum* CON | 0.205 | 0.101 | 0.01 | 0.284 | 0.141 | 0.010 | 0.000 | 0.067 | 1.000 |
| *Chloroflexus* CON | 0.229 | 0.108 | 0.01 | 0.236 | 0.128 | 0.020 | 0.155 | 0.143 | 0.120 |
| *Chromobacterium* CON | 0.207 | 0.096 | 0.01 | 0.205 | 0.109 | 0.020 | 0.203 | 0.135 | 0.070 |
| *Chthonomonas* CON | 0.174 | 0.096 | 0.02 | 0.205 | 0.122 | 0.010 | 0.000 | 0.074 | 1.000 |
| *Cladophialophora* CON | 0.197 | 0.094 | 0.02 | 0.228 | 0.123 | 0.030 | 0.216 | 0.136 | 0.080 |
| *Clavibacter* CON | 0.301 | 0.112 | 0.01 | 0.373 | 0.119 | 0.010 | 0.154 | 0.131 | 0.110 |
| *Cloacibacillus* CON | 0.271 | 0.104 | 0.01 | 0.349 | 0.138 | 0.010 | 0.105 | 0.130 | 0.260 |
| *Clostridioides* CON | 0.199 | 0.090 | 0.01 | 0.258 | 0.140 | 0.020 | 0.021 | 0.082 | 0.470 |
| *Comamonas* CON | 0.117 | 0.079 | 0.06 | 0.316 | 0.147 | 0.010 | 0.000 | 0.075 | 1.000 |
| *Conexibacter* CON | 0.151 | 0.090 | 0.05 | 0.165 | 0.109 | 0.030 | 0.064 | 0.110 | 0.420 |
| *Coraliomargarita* CON | 0.187 | 0.105 | 0.04 | 0.213 | 0.108 | 0.020 | 0.000 | 0.065 | 1.000 |
| *Coriobacterium* CON | 0.257 | 0.109 | 0.01 | 0.280 | 0.142 | 0.010 | 0.198 | 0.150 | 0.080 |

| | | | | | | | | | |
|---|---|---|---|---|---|---|---|---|---|
| *Corynebacterium* CON | 0.311 | 0.093 | 0.01 | 0.383 | 0.136 | 0.010 | 0.164 | 0.138 | 0.090 |
| *Croceibacter* CON | 0.292 | 0.110 | 0.01 | 0.301 | 0.145 | 0.020 | 0.186 | 0.140 | 0.090 |
| *Croceicoccus* CON | 0.173 | 0.089 | 0.03 | 0.222 | 0.112 | 0.020 | 0.002 | 0.067 | 0.460 |
| *Cryptobacterium* CON | 0.492 | 0.105 | 0.01 | 0.537 | 0.129 | 0.010 | 0.144 | 0.141 | 0.110 |
| *Cupriavidus* CON | 0.337 | 0.097 | 0.01 | 0.369 | 0.121 | 0.010 | 0.120 | 0.152 | 0.180 |
| *Cutaneotrichosporon* CON | 0.437 | 0.107 | 0.01 | 0.494 | 0.117 | 0.010 | 0.196 | 0.144 | 0.060 |
| *Cyanobium* CON | 0.282 | 0.107 | 0.01 | 0.331 | 0.129 | 0.010 | 0.000 | 0.071 | 1.000 |
| *Debaryomyces* CON | 0.305 | 0.103 | 0.01 | 0.327 | 0.148 | 0.010 | 0.198 | 0.152 | 0.090 |
| *Dechloromonas* CON | 0.154 | 0.095 | 0.03 | 0.179 | 0.122 | 0.030 | 0.074 | 0.109 | 0.300 |
| *Deferribacter* CON | 0.141 | 0.086 | 0.03 | 0.176 | 0.118 | 0.020 | 0.049 | 0.099 | 0.340 |
| *Defluviimonas* CON | 0.202 | 0.107 | 0.01 | 0.214 | 0.131 | 0.010 | 0.067 | 0.098 | 0.300 |
| *Dehalococcoides* CON | 0.268 | 0.104 | 0.01 | 0.275 | 0.132 | 0.010 | 0.200 | 0.151 | 0.050 |
| *Deinococcus* CON | 0.228 | 0.102 | 0.01 | 0.266 | 0.132 | 0.020 | 0.134 | 0.139 | 0.160 |
| *Desulfobacter* CON | 0.175 | 0.092 | 0.02 | 0.186 | 0.121 | 0.040 | 0.118 | 0.119 | 0.110 |
| *Desulfocapsa* CON | 0.293 | 0.099 | 0.01 | 0.323 | 0.142 | 0.010 | 0.065 | 0.107 | 0.310 |
| *Desulfococcus* CON | 0.291 | 0.093 | 0.01 | 0.305 | 0.139 | 0.010 | 0.166 | 0.136 | 0.120 |
| *Desulfosporosinus* CON | 0.209 | 0.104 | 0.01 | 0.243 | 0.128 | 0.010 | 0.025 | 0.085 | 0.430 |
| *Desulfovibrio* CON | 0.220 | 0.095 | 0.02 | 0.311 | 0.143 | 0.010 | 0.053 | 0.094 | 0.320 |
| *Dorea* CON | 0.259 | 0.093 | 0.01 | 0.265 | 0.135 | 0.010 | 0.203 | 0.146 | 0.050 |
| *Dyella* CON | 0.317 | 0.098 | 0.01 | 0.342 | 0.134 | 0.010 | 0.098 | 0.109 | 0.250 |
| *Ectothiorhodospira* CON | 0.153 | 0.094 | 0.01 | 0.275 | 0.131 | 0.010 | 0.000 | 0.079 | 1.000 |
| *Eggerthella* CON | 0.198 | 0.097 | 0.02 | 0.275 | 0.136 | 0.020 | 0.015 | 0.097 | 0.440 |
| *Ehrlichia* CON | 0.111 | 0.075 | 0.08 | 0.200 | 0.134 | 0.020 | 0.000 | 0.066 | 1.000 |
| *Eimeria* CON | 0.338 | 0.112 | 0.01 | 0.418 | 0.137 | 0.010 | 0.234 | 0.141 | 0.060 |
| *Emiliania* CON | 0.219 | 0.095 | 0.02 | 0.263 | 0.138 | 0.020 | 0.115 | 0.122 | 0.190 |
| *Endocarpon* CON | 0.160 | 0.088 | 0.01 | 0.252 | 0.121 | 0.020 | 0.000 | 0.000 | 0.530 |
| *Ensifer* CON | 0.146 | 0.084 | 0.07 | 0.185 | 0.108 | 0.040 | 0.057 | 0.116 | 0.300 |
| *Enterobacter* CON | 0.186 | 0.079 | 0.02 | 0.202 | 0.113 | 0.020 | 0.216 | 0.156 | 0.090 |
| *Enterococcus* CON | 0.162 | 0.095 | 0.04 | 0.274 | 0.118 | 0.020 | 0.000 | 0.069 | 1.000 |
| *Erysipelatoclostridium* CON | 0.298 | 0.096 | 0.01 | 0.372 | 0.133 | 0.010 | 0.147 | 0.131 | 0.120 |
| *Eubacterium* CON | 0.109 | 0.082 | 0.04 | 0.200 | 0.123 | 0.040 | 0.000 | 0.087 | 1.000 |
| *Faecalitalea* CON | 0.332 | 0.104 | 0.01 | 0.384 | 0.151 | 0.010 | 0.121 | 0.127 | 0.200 |
| *Frankia* CON | 0.174 | 0.094 | 0.01 | 0.235 | 0.123 | 0.020 | 0.000 | 0.072 | 1.000 |
| *Frateuria* CON | 0.146 | 0.086 | 0.05 | 0.193 | 0.119 | 0.020 | 0.000 | 0.059 | 1.000 |
| *Gemmatimonas* CON | 0.202 | 0.093 | 0.01 | 0.195 | 0.129 | 0.030 | 0.232 | 0.143 | 0.070 |
| *Geobacillus* CON | 0.241 | 0.102 | 0.01 | 0.332 | 0.143 | 0.010 | 0.122 | 0.131 | 0.140 |
| *Geobacter* CON | 0.257 | 0.105 | 0.01 | 0.291 | 0.145 | 0.020 | 0.076 | 0.112 | 0.210 |
| *Geosporobacter* CON | 0.194 | 0.093 | 0.02 | 0.251 | 0.125 | 0.030 | 0.003 | 0.067 | 0.560 |
| *Gilliamella* CON | 0.121 | 0.078 | 0.04 | 0.228 | 0.142 | 0.010 | 0.000 | 0.076 | 1.000 |
| *Glarea* CON | 0.153 | 0.086 | 0.03 | 0.174 | 0.115 | 0.040 | 0.144 | 0.128 | 0.150 |
| *Gordonia* CON | 0.187 | 0.093 | 0.02 | 0.182 | 0.123 | 0.040 | 0.200 | 0.152 | 0.090 |
| *Gordonibacter* CON | 0.189 | 0.104 | 0.02 | 0.314 | 0.146 | 0.010 | 0.000 | 0.072 | 1.000 |
| *Gregarina* CON | 0.196 | 0.099 | 0.01 | 0.180 | 0.120 | 0.020 | 0.212 | 0.142 | 0.050 |
| *Haemophilus* CON | 0.172 | 0.093 | 0.03 | 0.257 | 0.122 | 0.030 | 0.000 | 0.066 | 1.000 |
| *Halanaerobium* CON | 0.140 | 0.087 | 0.04 | 0.185 | 0.116 | 0.030 | 0.054 | 0.112 | 0.300 |
| *Halobiforma* CON | 0.128 | 0.083 | 0.09 | 0.148 | 0.107 | 0.040 | 0.071 | 0.113 | 0.340 |

| | | | | | | | | | |
|---|---|---|---|---|---|---|---|---|---|
| *Halopiger* CON | 0.235 | 0.091 | 0.01 | 0.295 | 0.127 | 0.010 | 0.025 | 0.092 | 0.390 |
| *Halorhodospira* CON | 0.181 | 0.094 | 0.01 | 0.184 | 0.108 | 0.040 | 0.078 | 0.100 | 0.270 |
| *Halorubrum* CON | 0.092 | 0.074 | 0.13 | 0.102 | 0.102 | 0.040 | 0.051 | 0.108 | 0.290 |
| *Hammondia* CON | 0.173 | 0.099 | 0.01 | 0.239 | 0.132 | 0.010 | 0.153 | 0.150 | 0.120 |
| *Herbaspirillum* CON | 0.212 | 0.101 | 0.01 | 0.229 | 0.125 | 0.030 | 0.093 | 0.118 | 0.210 |
| *Hyphomonas* CON | 0.258 | 0.097 | 0.01 | 0.269 | 0.118 | 0.010 | 0.083 | 0.116 | 0.240 |
| *Immundisolibacter* CON | 0.187 | 0.092 | 0.02 | 0.199 | 0.123 | 0.020 | 0.054 | 0.102 | 0.260 |
| *Isaria* CON | 0.387 | 0.120 | 0.01 | 0.530 | 0.134 | 0.010 | 0.160 | 0.140 | 0.120 |
| *Jeotgalibacillus* CON | 0.198 | 0.097 | 0.01 | 0.219 | 0.119 | 0.020 | 0.000 | 0.083 | 1.000 |
| *Jeotgalicoccus* CON | 0.147 | 0.090 | 0.04 | 0.159 | 0.120 | 0.040 | 0.102 | 0.120 | 0.250 |
| *Jonquetella* CON | 0.326 | 0.103 | 0.01 | 0.350 | 0.123 | 0.010 | 0.221 | 0.144 | 0.070 |
| *Kalmanozyma* CON | 0.194 | 0.093 | 0.01 | 0.219 | 0.138 | 0.030 | 0.007 | 0.069 | 0.510 |
| *Ketogulonicigenium* CON | 0.227 | 0.108 | 0.01 | 0.266 | 0.128 | 0.010 | 0.113 | 0.129 | 0.190 |
| *Kingella* CON | 0.233 | 0.113 | 0.01 | 0.271 | 0.119 | 0.020 | 0.049 | 0.092 | 0.490 |
| *Kluyvera* CON | 0.231 | 0.094 | 0.01 | 0.259 | 0.132 | 0.010 | 0.193 | 0.148 | 0.080 |
| *Kosakonia* CON | 0.130 | 0.088 | 0.04 | 0.179 | 0.114 | 0.040 | 0.105 | 0.125 | 0.190 |
| *Kosmotoga* CON | 0.230 | 0.095 | 0.01 | 0.302 | 0.132 | 0.010 | 0.000 | 0.066 | 1.000 |
| *Kytococcus* CON | 0.246 | 0.101 | 0.01 | 0.256 | 0.120 | 0.010 | 0.175 | 0.143 | 0.110 |
| *Lachnospira* CON | 0.151 | 0.096 | 0.04 | 0.188 | 0.111 | 0.030 | 0.016 | 0.084 | 0.520 |
| *Lactobacillus* CON | 0.136 | 0.090 | 0.03 | 0.231 | 0.142 | 0.030 | 0.000 | 0.000 | 0.840 |
| *Laribacter* CON | 0.252 | 0.106 | 0.01 | 0.253 | 0.136 | 0.020 | 0.218 | 0.139 | 0.060 |
| *Legionella* CON | 0.138 | 0.083 | 0.04 | 0.193 | 0.105 | 0.010 | 0.000 | 0.072 | 1.000 |
| *Lentibacillus* CON | 0.144 | 0.087 | 0.05 | 0.164 | 0.117 | 0.020 | 0.000 | 0.059 | 1.000 |
| *Leptomonas* CON | 0.144 | 0.095 | 0.03 | 0.181 | 0.118 | 0.040 | 0.126 | 0.119 | 0.190 |
| *Leptosphaeria* CON | 0.267 | 0.105 | 0.01 | 0.314 | 0.133 | 0.010 | 0.140 | 0.133 | 0.140 |
| *Leuconostoc* CON | 0.052 | 0.065 | 0.34 | 0.222 | 0.123 | 0.020 | 0.000 | 0.070 | 1.000 |
| *Libanicoccus* CON | 0.268 | 0.109 | 0.01 | 0.274 | 0.121 | 0.010 | 0.262 | 0.171 | 0.050 |
| *Limnohabitans* CON | 0.147 | 0.086 | 0.05 | 0.185 | 0.124 | 0.030 | 0.000 | 0.067 | 1.000 |
| *Lodderomyces* CON | 0.385 | 0.110 | 0.01 | 0.452 | 0.150 | 0.010 | 0.240 | 0.157 | 0.060 |
| *Luteipulveratus* CON | 0.208 | 0.101 | 0.02 | 0.254 | 0.134 | 0.030 | 0.000 | 0.000 | 0.700 |
| *Magnaporthe* CON | 0.303 | 0.112 | 0.01 | 0.391 | 0.142 | 0.010 | 0.132 | 0.140 | 0.150 |
| *Magnetococcus* CON | 0.152 | 0.089 | 0.02 | 0.158 | 0.113 | 0.040 | 0.025 | 0.086 | 0.460 |
| *Magnetospira* CON | 0.188 | 0.103 | 0.01 | 0.248 | 0.112 | 0.020 | 0.000 | 0.084 | 1.000 |
| *Mannheimia* CON | 0.171 | 0.093 | 0.01 | 0.287 | 0.144 | 0.010 | 0.000 | 0.090 | 1.000 |
| *Marinithermus* CON | 0.286 | 0.099 | 0.01 | 0.323 | 0.143 | 0.010 | 0.123 | 0.121 | 0.130 |
| *Marssonina* CON | 0.317 | 0.102 | 0.01 | 0.378 | 0.144 | 0.010 | 0.130 | 0.125 | 0.190 |
| *Massilia* CON | 0.160 | 0.088 | 0.03 | 0.197 | 0.104 | 0.020 | 0.055 | 0.110 | 0.350 |
| *Melioribacter* CON | 0.272 | 0.080 | 0.01 | 0.299 | 0.134 | 0.010 | 0.216 | 0.155 | 0.070 |
| *Methanobrevibacter* CON | 0.185 | 0.086 | 0.04 | 0.224 | 0.132 | 0.030 | 0.093 | 0.140 | 0.210 |
| *Methanococcoides* CON | 0.289 | 0.115 | 0.01 | 0.289 | 0.131 | 0.020 | 0.172 | 0.136 | 0.080 |
| *Methanococcus* CON | 0.216 | 0.097 | 0.01 | 0.246 | 0.137 | 0.010 | 0.150 | 0.136 | 0.130 |
| *Methanoculleus* CON | 0.339 | 0.103 | 0.01 | 0.423 | 0.116 | 0.010 | 0.147 | 0.136 | 0.100 |
| *Methanohalophilus* CON | 0.188 | 0.096 | 0.01 | 0.208 | 0.128 | 0.010 | 0.037 | 0.085 | 0.390 |
| *Methanolacinia* CON | 0.189 | 0.097 | 0.01 | 0.208 | 0.127 | 0.030 | 0.056 | 0.099 | 0.380 |
| *Methanolobus* CON | 0.175 | 0.098 | 0.01 | 0.209 | 0.119 | 0.020 | 0.107 | 0.112 | 0.270 |

| | | | | | | | | | |
|---|---|---|---|---|---|---|---|---|---|
| *Methanomassiliicoccus* CON | 0.246 | 0.097 | 0.01 | 0.251 | 0.123 | 0.010 | 0.204 | 0.148 | 0.070 |
| *Methanosarcina* CON | 0.226 | 0.097 | 0.01 | 0.222 | 0.130 | 0.010 | 0.205 | 0.140 | 0.050 |
| *Methanothermococcus* CON | 0.307 | 0.102 | 0.01 | 0.379 | 0.146 | 0.010 | 0.180 | 0.148 | 0.100 |
| *Methylacidiphilum* CON | 0.258 | 0.086 | 0.01 | 0.284 | 0.137 | 0.010 | 0.196 | 0.135 | 0.080 |
| *Methylibium* CON | 0.230 | 0.103 | 0.01 | 0.241 | 0.130 | 0.020 | 0.021 | 0.077 | 0.440 |
| *Methylobacterium* CON | 0.280 | 0.096 | 0.01 | 0.326 | 0.144 | 0.010 | 0.190 | 0.133 | 0.080 |
| *Methylomonas* CON | 0.163 | 0.089 | 0.04 | 0.150 | 0.107 | 0.030 | 0.216 | 0.130 | 0.050 |
| *Methyloversatilis* CON | 0.269 | 0.095 | 0.01 | 0.333 | 0.136 | 0.010 | 0.000 | 0.071 | 1.000 |
| *Microbacterium* CON | 0.181 | 0.087 | 0.02 | 0.244 | 0.131 | 0.020 | 0.067 | 0.097 | 0.290 |
| *Microsporum* CON | 0.399 | 0.118 | 0.01 | 0.460 | 0.161 | 0.010 | 0.177 | 0.125 | 0.090 |
| *Microterricola* CON | 0.181 | 0.090 | 0.01 | 0.193 | 0.120 | 0.020 | 0.160 | 0.131 | 0.100 |
| *Mitsuokella* CON | 0.327 | 0.113 | 0.01 | 0.382 | 0.130 | 0.010 | 0.212 | 0.145 | 0.060 |
| *Moesziomyces* CON | 0.309 | 0.106 | 0.01 | 0.329 | 0.130 | 0.010 | 0.195 | 0.145 | 0.110 |
| *Moorella* CON | 0.282 | 0.103 | 0.01 | 0.347 | 0.112 | 0.010 | 0.095 | 0.123 | 0.200 |
| *Moritella* CON | 0.215 | 0.104 | 0.01 | 0.241 | 0.130 | 0.010 | 0.013 | 0.081 | 0.560 |
| *Nakaseomyces* CON | 0.323 | 0.104 | 0.01 | 0.343 | 0.127 | 0.010 | 0.206 | 0.142 | 0.070 |
| *Natranaerobius* CON | 0.255 | 0.117 | 0.01 | 0.293 | 0.125 | 0.010 | 0.047 | 0.097 | 0.330 |
| *Natronomonas* CON | 0.182 | 0.084 | 0.01 | 0.207 | 0.125 | 0.020 | 0.090 | 0.121 | 0.230 |
| *Naumovozyma* CON | 0.390 | 0.112 | 0.01 | 0.452 | 0.130 | 0.010 | 0.227 | 0.165 | 0.070 |
| *Neisseria* CON | 0.232 | 0.089 | 0.01 | 0.263 | 0.127 | 0.010 | 0.131 | 0.128 | 0.170 |
| *Neorhizobium* CON | 0.116 | 0.080 | 0.07 | 0.154 | 0.109 | 0.030 | 0.020 | 0.086 | 0.500 |
| *Neorickettsia* CON | 0.112 | 0.087 | 0.05 | 0.145 | 0.113 | 0.030 | 0.000 | 0.071 | 1.000 |
| *Nitratireductor* CON | 0.146 | 0.090 | 0.02 | 0.181 | 0.118 | 0.020 | 0.100 | 0.116 | 0.190 |
| *Nitrobacter* CON | 0.257 | 0.113 | 0.01 | 0.279 | 0.135 | 0.010 | 0.000 | 0.078 | 1.000 |
| *Nodularia* CON | 0.389 | 0.120 | 0.01 | 0.439 | 0.152 | 0.010 | 0.163 | 0.139 | 0.060 |
| *Nostoc* CON | 0.410 | 0.095 | 0.01 | 0.461 | 0.143 | 0.010 | 0.166 | 0.125 | 0.080 |
| *Novosphingobium* CON | 0.210 | 0.090 | 0.01 | 0.235 | 0.126 | 0.020 | 0.180 | 0.135 | 0.080 |
| *Octadecabacter* CON | 0.154 | 0.087 | 0.04 | 0.160 | 0.123 | 0.030 | 0.041 | 0.095 | 0.400 |
| *Oenococcus* CON | 0.209 | 0.087 | 0.01 | 0.311 | 0.130 | 0.010 | 0.000 | 0.063 | 1.000 |
| *Ottowia* CON | 0.173 | 0.101 | 0.01 | 0.179 | 0.116 | 0.030 | 0.129 | 0.132 | 0.150 |
| *Pandoraea* CON | 0.186 | 0.094 | 0.01 | 0.191 | 0.107 | 0.020 | 0.138 | 0.121 | 0.140 |
| *Pannonibacter* CON | 0.274 | 0.112 | 0.01 | 0.280 | 0.137 | 0.010 | 0.182 | 0.141 | 0.070 |
| *Paracoccus* CON | 0.154 | 0.094 | 0.02 | 0.201 | 0.114 | 0.030 | 0.000 | 0.090 | 1.000 |
| *Parascardovia* CON | 0.240 | 0.102 | 0.01 | 0.273 | 0.115 | 0.010 | 0.142 | 0.151 | 0.220 |
| *Pelagibaca* CON | 0.169 | 0.093 | 0.01 | 0.176 | 0.122 | 0.020 | 0.103 | 0.131 | 0.240 |
| *Pelobacter* CON | 0.156 | 0.088 | 0.03 | 0.214 | 0.123 | 0.010 | 0.073 | 0.119 | 0.330 |
| *Penicillium* CON | 0.136 | 0.086 | 0.02 | 0.183 | 0.117 | 0.020 | 0.068 | 0.095 | 0.300 |
| *Phaeodactylum* CON | 0.295 | 0.110 | 0.01 | 0.321 | 0.145 | 0.010 | 0.181 | 0.134 | 0.090 |
| *Planococcus* CON | 0.174 | 0.092 | 0.02 | 0.186 | 0.109 | 0.020 | 0.137 | 0.130 | 0.120 |
| *Pochonia* CON | 0.350 | 0.114 | 0.01 | 0.377 | 0.125 | 0.010 | 0.231 | 0.147 | 0.050 |
| *Podospora* CON | 0.227 | 0.095 | 0.01 | 0.257 | 0.136 | 0.010 | 0.182 | 0.142 | 0.090 |
| *Porphyrobacter* CON | 0.268 | 0.102 | 0.01 | 0.273 | 0.122 | 0.010 | 0.172 | 0.138 | 0.080 |
| *Propionibacterium* CON | 0.313 | 0.102 | 0.01 | 0.358 | 0.137 | 0.010 | 0.166 | 0.131 | 0.130 |
| *Pseudogulbenkiania* CON | 0.194 | 0.106 | 0.01 | 0.260 | 0.110 | 0.010 | 0.017 | 0.080 | 0.520 |

| | | | | | | | | | |
|---|---|---|---|---|---|---|---|---|---|
| *Pseudomonas* $_{CON}$ | 0.189 | 0.098 | 0.01 | 0.219 | 0.118 | 0.020 | 0.099 | 0.121 | 0.230 |
| *Pseudothermotoga* $_{CON}$ | 0.178 | 0.093 | 0.01 | 0.193 | 0.123 | 0.030 | 0.244 | 0.149 | 0.050 |
| *Pseudoxanthomonas* $_{CON}$ | 0.174 | 0.098 | 0.02 | 0.229 | 0.138 | 0.010 | 0.000 | 0.062 | 1.000 |
| *Psychromonas* $_{CON}$ | 0.133 | 0.074 | 0.05 | 0.167 | 0.110 | 0.040 | 0.000 | 0.060 | 1.000 |
| *Ralstonia* $_{CON}$ | 0.193 | 0.089 | 0.01 | 0.204 | 0.129 | 0.020 | 0.071 | 0.104 | 0.310 |
| *Rasamsonia* $_{CON}$ | 0.212 | 0.100 | 0.01 | 0.237 | 0.127 | 0.020 | 0.178 | 0.141 | 0.080 |
| *Rhodanobacter* $_{CON}$ | 0.181 | 0.099 | 0.01 | 0.195 | 0.122 | 0.030 | 0.058 | 0.104 | 0.370 |
| *Rhodoferax* $_{CON}$ | 0.202 | 0.092 | 0.01 | 0.226 | 0.129 | 0.020 | 0.171 | 0.157 | 0.090 |
| *Rhodopseudomonas* $_{CON}$ | 0.243 | 0.106 | 0.01 | 0.284 | 0.144 | 0.020 | 0.093 | 0.112 | 0.240 |
| *Rhodovulum* $_{CON}$ | 0.233 | 0.092 | 0.01 | 0.266 | 0.129 | 0.010 | 0.076 | 0.107 | 0.280 |
| *Rivularia* $_{CON}$ | 0.233 | 0.107 | 0.01 | 0.234 | 0.124 | 0.010 | 0.217 | 0.153 | 0.070 |
| *Rothia* $_{CON}$ | 0.222 | 0.103 | 0.01 | 0.283 | 0.132 | 0.010 | 0.065 | 0.120 | 0.260 |
| *Rubrobacter* $_{CON}$ | 0.209 | 0.094 | 0.01 | 0.245 | 0.131 | 0.010 | 0.003 | 0.087 | 0.590 |
| *Ruegeria* $_{CON}$ | 0.276 | 0.101 | 0.01 | 0.296 | 0.133 | 0.010 | 0.165 | 0.138 | 0.110 |
| *Ruminiclostridium* $_{CON}$ | 0.273 | 0.115 | 0.01 | 0.333 | 0.120 | 0.010 | 0.112 | 0.129 | 0.120 |
| *Salegentibacter* $_{CON}$ | 0.235 | 0.100 | 0.01 | 0.246 | 0.113 | 0.010 | 0.165 | 0.151 | 0.080 |
| *Salimicrobium* $_{CON}$ | 0.311 | 0.108 | 0.01 | 0.371 | 0.135 | 0.010 | 0.069 | 0.115 | 0.260 |
| *Salinicoccus* $_{CON}$ | 0.313 | 0.102 | 0.01 | 0.318 | 0.130 | 0.010 | 0.192 | 0.148 | 0.090 |
| *Sandaracinus* $_{CON}$ | 0.209 | 0.097 | 0.02 | 0.263 | 0.130 | 0.020 | 0.000 | 0.072 | 1.000 |
| *Sarcina* $_{CON}$ | 0.121 | 0.086 | 0.06 | 0.185 | 0.112 | 0.010 | 0.026 | 0.089 | 0.470 |
| *Scheffersomyces* $_{CON}$ | 0.404 | 0.121 | 0.01 | 0.450 | 0.128 | 0.010 | 0.080 | 0.113 | 0.310 |
| *Sclerotinia* $_{CON}$ | 0.337 | 0.112 | 0.01 | 0.396 | 0.136 | 0.010 | 0.118 | 0.123 | 0.160 |
| *Sedimenticola* $_{CON}$ | 0.191 | 0.092 | 0.01 | 0.234 | 0.126 | 0.010 | 0.030 | 0.102 | 0.420 |
| *Sharpea* $_{CON}$ | 0.257 | 0.093 | 0.01 | 0.429 | 0.143 | 0.010 | 0.103 | 0.102 | 0.210 |
| *Shinella* $_{CON}$ | 0.351 | 0.109 | 0.01 | 0.378 | 0.137 | 0.010 | 0.167 | 0.130 | 0.100 |
| *Sideroxydans* $_{CON}$ | 0.200 | 0.094 | 0.01 | 0.227 | 0.116 | 0.020 | 0.000 | 0.065 | 1.000 |
| *Sinorhizobium* $_{CON}$ | 0.311 | 0.114 | 0.01 | 0.333 | 0.119 | 0.010 | 0.150 | 0.133 | 0.110 |
| *Sorangium* $_{CON}$ | 0.176 | 0.085 | 0.03 | 0.194 | 0.118 | 0.010 | 0.009 | 0.066 | 0.520 |
| *Sphaerochaeta* $_{CON}$ | 0.399 | 0.107 | 0.01 | 0.429 | 0.138 | 0.010 | 0.182 | 0.149 | 0.060 |
| *Sphingomonas* $_{CON}$ | 0.298 | 0.113 | 0.01 | 0.338 | 0.139 | 0.010 | 0.178 | 0.160 | 0.060 |
| *Spirochaeta* $_{CON}$ | 0.178 | 0.083 | 0.04 | 0.209 | 0.116 | 0.040 | 0.032 | 0.092 | 0.450 |
| *Starkeya* $_{CON}$ | 0.194 | 0.099 | 0.02 | 0.267 | 0.137 | 0.020 | 0.000 | 0.064 | 1.000 |
| *Stenotrophomonas* $_{CON}$ | 0.118 | 0.080 | 0.05 | 0.187 | 0.126 | 0.030 | 0.006 | 0.077 | 0.570 |
| *Stigmatella* $_{CON}$ | 0.206 | 0.103 | 0.01 | 0.235 | 0.140 | 0.030 | 0.061 | 0.108 | 0.350 |
| *Succiniclasticum* $_{CON}$ | 0.302 | 0.100 | 0.01 | 0.331 | 0.138 | 0.010 | 0.103 | 0.127 | 0.190 |
| *Succinivibrio* $_{CON}$ | 0.104 | 0.074 | 0.05 | 0.176 | 0.116 | 0.040 | 0.000 | 0.068 | 1.000 |
| *Sulfitobacter* $_{CON}$ | 0.202 | 0.099 | 0.03 | 0.244 | 0.119 | 0.020 | 0.009 | 0.071 | 0.560 |
| *Sulfurihydrogenibium* $_{CON}$ | 0.296 | 0.111 | 0.01 | 0.414 | 0.138 | 0.010 | 0.000 | 0.073 | 1.000 |
| *Syntrophobotulus* $_{CON}$ | 0.364 | 0.100 | 0.01 | 0.428 | 0.137 | 0.010 | 0.030 | 0.088 | 0.460 |
| *Syntrophus* $_{CON}$ | 0.160 | 0.085 | 0.02 | 0.170 | 0.107 | 0.030 | 0.109 | 0.101 | 0.270 |
| *Talaromyces* $_{CON}$ | 0.254 | 0.097 | 0.01 | 0.283 | 0.136 | 0.010 | 0.151 | 0.141 | 0.140 |
| *Taylorella* $_{CON}$ | 0.152 | 0.083 | 0.02 | 0.192 | 0.123 | 0.020 | 0.000 | 0.072 | 1.000 |
| *Thalassiosira* $_{CON}$ | 0.166 | 0.094 | 0.02 | 0.215 | 0.119 | 0.020 | 0.000 | 0.088 | 1.000 |
| *Thalassospira* $_{CON}$ | 0.197 | 0.096 | 0.02 | 0.182 | 0.126 | 0.040 | 0.192 | 0.146 | 0.080 |
| *Thermaerobacter* $_{CON}$ | 0.271 | 0.107 | 0.01 | 0.309 | 0.138 | 0.010 | 0.078 | 0.107 | 0.340 |
| *Thermoanaerobacter* $_{CON}$ | 0.184 | 0.087 | 0.02 | 0.222 | 0.123 | 0.030 | 0.000 | 0.071 | 1.000 |

| | | | | | | | | | |
|---|---|---|---|---|---|---|---|---|---|
| *Thermoanaerobacterium* CON | 0.153 | 0.084 | 0.03 | 0.227 | 0.107 | 0.010 | 0.000 | 0.073 | 1.000 |
| *Thermodesulfobium* CON | 0.102 | 0.080 | 0.1 | 0.188 | 0.111 | 0.020 | 0.004 | 0.075 | 0.570 |
| *Thermus* CON | 0.191 | 0.109 | 0.01 | 0.208 | 0.123 | 0.020 | 0.123 | 0.118 | 0.130 |
| *Thioalkalivibrio* CON | 0.229 | 0.103 | 0.01 | 0.233 | 0.102 | 0.020 | 0.181 | 0.144 | 0.080 |
| *Thioflavicoccus* CON | 0.200 | 0.091 | 0.01 | 0.216 | 0.105 | 0.020 | 0.066 | 0.098 | 0.360 |
| *Thiomonas* CON | 0.193 | 0.091 | 0.01 | 0.225 | 0.115 | 0.040 | 0.046 | 0.098 | 0.360 |
| *Thioploca* CON | 0.210 | 0.093 | 0.01 | 0.251 | 0.133 | 0.010 | 0.000 | 0.057 | 1.000 |
| *Trichomonas* CON | 0.183 | 0.089 | 0.03 | 0.189 | 0.116 | 0.040 | 0.220 | 0.151 | 0.070 |
| *Trichormus* CON | 0.250 | 0.097 | 0.01 | 0.274 | 0.121 | 0.010 | 0.116 | 0.125 | 0.160 |
| *Tsukamurella* CON | 0.175 | 0.092 | 0.02 | 0.192 | 0.123 | 0.040 | 0.000 | 0.071 | 1.000 |
| *Tyzzerella* CON | 0.218 | 0.108 | 0.01 | 0.260 | 0.133 | 0.010 | 0.132 | 0.123 | 0.170 |
| *Variovorax* CON | 0.268 | 0.102 | 0.01 | 0.280 | 0.132 | 0.010 | 0.199 | 0.144 | 0.080 |
| *Veillonella* CON | 0.353 | 0.113 | 0.01 | 0.396 | 0.127 | 0.010 | 0.145 | 0.128 | 0.140 |
| *Wenzhouxiangella* CON | 0.221 | 0.099 | 0.01 | 0.226 | 0.126 | 0.020 | 0.195 | 0.151 | 0.070 |
| *Yarrowia* CON | 0.206 | 0.100 | 0.01 | 0.189 | 0.123 | 0.040 | 0.267 | 0.152 | 0.050 |
| *Zhongshania* CON | 0.218 | 0.098 | 0.01 | 0.218 | 0.132 | 0.020 | 0.198 | 0.152 | 0.100 |
| *Acetitomaculum* FOR | 0.152 | 0.087 | 0.08 | 0.122 | 0.101 | 0.100 | 0.210 | 0.150 | 0.030 |
| *Aequorivita* FOR | 0.000 | 0.042 | 1 | 0.000 | 0.056 | 1.000 | 0.371 | 0.179 | 0.010 |
| *Alteromonas* FOR | 0.158 | 0.098 | 0.01 | 0.000 | 0.061 | 1.000 | 0.524 | 0.178 | 0.010 |
| *Ammonifex* FOR | 0.175 | 0.085 | 0.01 | 0.095 | 0.096 | 0.110 | 0.368 | 0.151 | 0.020 |
| *Babesia* FOR | 0.129 | 0.090 | 0.09 | 0.084 | 0.086 | 0.190 | 0.258 | 0.138 | 0.020 |
| *Candidatus Amoebophilus* FOR | 0.223 | 0.101 | 0.02 | 0.154 | 0.121 | 0.060 | 0.481 | 0.166 | 0.010 |
| *Candidatus Carsonella* FOR | 0.192 | 0.102 | 0.01 | 0.132 | 0.115 | 0.060 | 0.246 | 0.169 | 0.040 |
| *Candidatus Profftella* FOR | 0.172 | 0.100 | 0.01 | 0.095 | 0.098 | 0.110 | 0.250 | 0.154 | 0.040 |
| *Cedecea* FOR | 0.141 | 0.080 | 0.03 | 0.122 | 0.110 | 0.090 | 0.199 | 0.152 | 0.040 |
| *Chondromyces* FOR | 0.123 | 0.082 | 0.05 | 0.105 | 0.101 | 0.160 | 0.388 | 0.156 | 0.010 |
| *Chryseobacterium* FOR | 0.181 | 0.103 | 0.01 | 0.081 | 0.090 | 0.150 | 0.245 | 0.155 | 0.040 |
| *Colletotrichum* FOR | 0.154 | 0.095 | 0.04 | 0.077 | 0.092 | 0.280 | 0.278 | 0.147 | 0.030 |
| *Collimonas* FOR | 0.092 | 0.069 | 0.12 | 0.049 | 0.074 | 0.360 | 0.221 | 0.157 | 0.040 |
| *Cronobacter* FOR | 0.184 | 0.094 | 0.01 | 0.159 | 0.111 | 0.070 | 0.290 | 0.158 | 0.030 |
| *Cyanothece* FOR | 0.109 | 0.079 | 0.04 | 0.038 | 0.075 | 0.430 | 0.232 | 0.164 | 0.040 |
| *Desulfurispirillum* FOR | 0.144 | 0.085 | 0.03 | 0.121 | 0.098 | 0.120 | 0.266 | 0.167 | 0.040 |
| *Devosia* FOR | 0.145 | 0.082 | 0.02 | 0.090 | 0.096 | 0.150 | 0.324 | 0.160 | 0.030 |
| *Edwardsiella* FOR | 0.232 | 0.095 | 0.01 | 0.203 | 0.114 | 0.050 | 0.336 | 0.166 | 0.020 |
| *Elizabethkingia* FOR | 0.150 | 0.082 | 0.03 | 0.140 | 0.105 | 0.090 | 0.224 | 0.150 | 0.020 |
| *Escherichia* FOR | 0.082 | 0.075 | 0.11 | 0.000 | 0.049 | 1.000 | 0.238 | 0.158 | 0.040 |
| *Ethanoligenens* FOR | 0.186 | 0.087 | 0.02 | 0.132 | 0.107 | 0.090 | 0.290 | 0.157 | 0.030 |
| *Flavonifractor* FOR | 0.047 | 0.064 | 0.32 | 0.000 | 0.049 | 1.000 | 0.271 | 0.153 | 0.040 |
| *Fluviicola* FOR | 0.078 | 0.069 | 0.14 | 0.034 | 0.083 | 0.390 | 0.301 | 0.158 | 0.010 |
| *Fusarium* FOR | 0.146 | 0.084 | 0.04 | 0.040 | 0.084 | 0.300 | 0.371 | 0.167 | 0.010 |
| *Gaeumannomyces* FOR | 0.175 | 0.091 | 0.05 | 0.093 | 0.097 | 0.160 | 0.360 | 0.175 | 0.020 |
| *Glaciecola* FOR | 0.027 | 0.050 | 0.32 | 0.000 | 0.057 | 1.000 | 0.176 | 0.138 | 0.030 |
| *Grosmannia* FOR | 0.087 | 0.070 | 0.13 | 0.067 | 0.098 | 0.290 | 0.306 | 0.161 | 0.010 |

| | | | | | | | | |
|---|---|---|---|---|---|---|---|---|
| *Haematospirillum* FOR | 0.080 | 0.075 | 0.11 | 0.033 | 0.076 | 0.420 | 0.187 | 0.155 | 0.040 |
| *Halobacillus* FOR | 0.147 | 0.089 | 0.02 | 0.129 | 0.109 | 0.090 | 0.231 | 0.147 | 0.030 |
| *Halogranum* FOR | 0.139 | 0.069 | 0.06 | 0.113 | 0.090 | 0.090 | 0.254 | 0.153 | 0.040 |
| *Halomonas* FOR | 0.168 | 0.097 | 0.02 | 0.139 | 0.101 | 0.090 | 0.301 | 0.163 | 0.020 |
| *Herminiimonas* FOR | 0.023 | 0.059 | 0.36 | 0.000 | 0.066 | 1.000 | 0.313 | 0.146 | 0.010 |
| *Intestinimonas* FOR | 0.117 | 0.084 | 0.05 | 0.050 | 0.077 | 0.310 | 0.302 | 0.177 | 0.010 |
| *Lachnobacterium* FOR | 0.177 | 0.090 | 0.02 | 0.136 | 0.101 | 0.080 | 0.329 | 0.171 | 0.010 |
| *Lacinutrix* FOR | 0.162 | 0.083 | 0.02 | 0.119 | 0.106 | 0.110 | 0.240 | 0.154 | 0.010 |
| *Leishmania* FOR | 0.045 | 0.061 | 0.21 | 0.000 | 0.045 | 1.000 | 0.224 | 0.137 | 0.030 |
| *Lentzea* FOR | 0.044 | 0.067 | 0.25 | 0.000 | 0.056 | 1.000 | 0.248 | 0.158 | 0.040 |
| *Leptolyngbya* FOR | 0.111 | 0.077 | 0.04 | 0.000 | 0.054 | 1.000 | 0.360 | 0.175 | 0.020 |
| *Leptothrix* FOR | 0.136 | 0.086 | 0.03 | 0.102 | 0.094 | 0.140 | 0.354 | 0.164 | 0.010 |
| *Lysobacter* FOR | 0.194 | 0.096 | 0.02 | 0.095 | 0.095 | 0.140 | 0.495 | 0.176 | 0.010 |
| *Marinobacter* FOR | 0.130 | 0.088 | 0.09 | 0.077 | 0.080 | 0.240 | 0.254 | 0.134 | 0.010 |
| *Methanothermus* FOR | 0.174 | 0.092 | 0.03 | 0.148 | 0.113 | 0.090 | 0.264 | 0.140 | 0.010 |
| *Myxococcus* FOR | 0.070 | 0.076 | 0.19 | 0.000 | 0.050 | 1.000 | 0.212 | 0.158 | 0.040 |
| *Nannochloropsis* FOR | 0.290 | 0.092 | 0.01 | 0.127 | 0.102 | 0.100 | 0.362 | 0.161 | 0.010 |
| *Natrialba* FOR | 0.185 | 0.096 | 0.01 | 0.159 | 0.105 | 0.060 | 0.247 | 0.146 | 0.030 |
| *Ndongobacter* FOR | 0.192 | 0.089 | 0.01 | 0.160 | 0.125 | 0.060 | 0.276 | 0.157 | 0.020 |
| *Niastella* FOR | 0.115 | 0.076 | 0.04 | 0.096 | 0.107 | 0.220 | 0.276 | 0.170 | 0.020 |
| *Nitrosomonas* FOR | 0.118 | 0.076 | 0.06 | 0.111 | 0.094 | 0.170 | 0.219 | 0.151 | 0.030 |
| *Oceanimonas* FOR | 0.176 | 0.093 | 0.02 | 0.173 | 0.112 | 0.060 | 0.182 | 0.139 | 0.040 |
| *Ogataea* FOR | 0.050 | 0.062 | 0.32 | 0.000 | 0.057 | 1.000 | 0.299 | 0.159 | 0.010 |
| *Oribacterium* FOR | 0.189 | 0.105 | 0.04 | 0.147 | 0.113 | 0.070 | 0.232 | 0.156 | 0.010 |
| *Oscillibacter* FOR | 0.123 | 0.093 | 0.05 | 0.046 | 0.073 | 0.350 | 0.353 | 0.173 | 0.020 |
| *Owenweeksia* FOR | 0.100 | 0.073 | 0.08 | 0.079 | 0.096 | 0.260 | 0.230 | 0.155 | 0.040 |
| *Paraburkholderia* FOR | 0.124 | 0.079 | 0.03 | 0.070 | 0.086 | 0.220 | 0.289 | 0.156 | 0.040 |
| *Pasteurella* FOR | 0.147 | 0.087 | 0.02 | 0.113 | 0.098 | 0.120 | 0.238 | 0.153 | 0.040 |
| *Pelodictyon* FOR | 0.108 | 0.081 | 0.04 | 0.065 | 0.081 | 0.280 | 0.234 | 0.163 | 0.040 |
| *Phanerochaete* FOR | 0.066 | 0.067 | 0.16 | 0.026 | 0.077 | 0.500 | 0.333 | 0.187 | 0.020 |
| *Phytophthora* FOR | 0.181 | 0.092 | 0.01 | 0.076 | 0.099 | 0.230 | 0.338 | 0.160 | 0.030 |
| *Polymorphum* FOR | 0.178 | 0.093 | 0.03 | 0.149 | 0.110 | 0.080 | 0.267 | 0.147 | 0.030 |
| *Pusillimonas* FOR | 0.104 | 0.083 | 0.11 | 0.073 | 0.083 | 0.270 | 0.251 | 0.172 | 0.020 |
| *Rahnella* FOR | 0.174 | 0.084 | 0.01 | 0.123 | 0.097 | 0.110 | 0.337 | 0.156 | 0.010 |
| *Rhodoplanes* FOR | 0.074 | 0.074 | 0.22 | 0.049 | 0.071 | 0.260 | 0.244 | 0.159 | 0.040 |
| *Salinispora* FOR | 0.167 | 0.091 | 0.02 | 0.129 | 0.092 | 0.090 | 0.245 | 0.148 | 0.030 |
| *Selenomonas* FOR | 0.239 | 0.097 | 0.01 | 0.058 | 0.085 | 0.330 | 0.594 | 0.156 | 0.010 |
| *Serpula* FOR | 0.171 | 0.070 | 0.01 | 0.149 | 0.111 | 0.090 | 0.256 | 0.167 | 0.020 |
| *Stackebrandtia* FOR | 0.060 | 0.064 | 0.12 | 0.000 | 0.000 | 0.580 | 0.220 | 0.159 | 0.040 |
| *Stanieria* FOR | 0.000 | 0.042 | 1 | 0.000 | 0.062 | 1.000 | 0.332 | 0.154 | 0.020 |
| *Sulfuricella* FOR | 0.074 | 0.076 | 0.22 | 0.000 | 0.058 | 1.000 | 0.288 | 0.168 | 0.040 |
| *Teredinibacter* FOR | 0.151 | 0.094 | 0.02 | 0.096 | 0.113 | 0.190 | 0.340 | 0.164 | 0.020 |
| *Thalassolituus* FOR | 0.112 | 0.076 | 0.09 | 0.065 | 0.084 | 0.210 | 0.225 | 0.159 | 0.020 |
| *Thermodesulfobacterium* FOR | 0.108 | 0.087 | 0.08 | 0.103 | 0.091 | 0.180 | 0.223 | 0.158 | 0.040 |
| *Thermosipho* FOR | 0.105 | 0.083 | 0.03 | 0.000 | 0.053 | 1.000 | 0.326 | 0.169 | 0.010 |

| | | | | | | | | | |
|---|---|---|---|---|---|---|---|---|---|
| *Thermothelomyces* FOR | 0.142 | 0.096 | 0.02 | 0.147 | 0.122 | 0.110 | 0.212 | 0.170 | 0.040 |
| *Thermotoga* FOR | 0.157 | 0.100 | 0.01 | 0.107 | 0.098 | 0.170 | 0.286 | 0.148 | 0.020 |
| *Thermovirga* FOR | 0.268 | 0.105 | 0.01 | 0.185 | 0.123 | 0.050 | 0.581 | 0.169 | 0.010 |
| *Thiolapillus* FOR | 0.018 | 0.043 | 0.43 | 0.000 | 0.056 | 1.000 | 0.197 | 0.140 | 0.040 |
| *Vibrio* FOR | 0.136 | 0.074 | 0.02 | 0.056 | 0.093 | 0.360 | 0.311 | 0.169 | 0.040 |
| *Weeksella* FOR | 0.047 | 0.067 | 0.3 | 0.000 | 0.063 | 1.000 | 0.263 | 0.154 | 0.010 |
| *Wenyingzhuangia* FOR | 0.155 | 0.087 | 0.01 | 0.106 | 0.097 | 0.140 | 0.283 | 0.150 | 0.020 |
| *Acetoanaerobium* | 0.076 | 0.066 | 0.18 | 0.150 | 0.114 | 0.070 | 0.000 | 0.071 | 1.000 |
| *Acetobacter* | 0.000 | 0.043 | 1 | 0.000 | 0.049 | 1.000 | 0.000 | 0.088 | 1.000 |
| *Acetobacterium* | 0.000 | 0.044 | 1 | 0.000 | 0.064 | 1.000 | 0.001 | 0.075 | 0.510 |
| *Acholeplasma* | 0.002 | 0.051 | 0.59 | 0.027 | 0.076 | 0.480 | 0.000 | 0.070 | 1.000 |
| *Acidimicrobium* | 0.115 | 0.082 | 0.05 | 0.111 | 0.103 | 0.120 | 0.105 | 0.119 | 0.150 |
| *Acidiphilium* | 0.110 | 0.074 | 0.04 | 0.118 | 0.095 | 0.090 | 0.000 | 0.077 | 1.000 |
| *Acidipropionibacterium* | 0.035 | 0.068 | 0.28 | 0.057 | 0.068 | 0.260 | 0.000 | 0.000 | 0.740 |
| *Acidithiobacillus* | 0.060 | 0.071 | 0.2 | 0.046 | 0.080 | 0.390 | 0.102 | 0.139 | 0.180 |
| *Acidobacterium* | 0.050 | 0.060 | 0.25 | 0.039 | 0.086 | 0.340 | 0.079 | 0.107 | 0.320 |
| *Actinoalloteichus* | 0.019 | 0.057 | 0.35 | 0.000 | 0.048 | 1.000 | 0.087 | 0.111 | 0.200 |
| *Actinobacillus* | 0.065 | 0.070 | 0.2 | 0.084 | 0.086 | 0.210 | 0.000 | 0.079 | 1.000 |
| *Actinoplanes* | 0.184 | 0.096 | 0.01 | 0.174 | 0.135 | 0.050 | 0.233 | 0.151 | 0.060 |
| *Actinosynnema* | 0.056 | 0.054 | 0.18 | 0.084 | 0.084 | 0.210 | 0.000 | 0.073 | 1.000 |
| *Actinotignum* | 0.101 | 0.086 | 0.08 | 0.095 | 0.092 | 0.140 | 0.101 | 0.134 | 0.220 |
| *Advenella* | 0.097 | 0.077 | 0.08 | 0.153 | 0.110 | 0.070 | 0.000 | 0.077 | 1.000 |
| *Aeromonas* | 0.150 | 0.079 | 0.02 | 0.173 | 0.115 | 0.060 | 0.030 | 0.089 | 0.460 |
| *Aggregatibacter* | 0.025 | 0.052 | 0.35 | 0.018 | 0.066 | 0.540 | 0.061 | 0.084 | 0.360 |
| *Akkermansia* | 0.000 | 0.043 | 1 | 0.000 | 0.055 | 1.000 | 0.000 | 0.072 | 1.000 |
| *Alcaligenes* | 0.000 | 0.040 | 1 | 0.000 | 0.063 | 1.000 | 0.000 | 0.076 | 1.000 |
| *Algibacter* | 0.079 | 0.085 | 0.15 | 0.095 | 0.092 | 0.120 | 0.057 | 0.100 | 0.350 |
| *Algoriphagus* | 0.000 | 0.045 | 1 | 0.000 | 0.053 | 1.000 | 0.117 | 0.123 | 0.170 |
| *Aliivibrio* | 0.018 | 0.048 | 0.47 | 0.030 | 0.065 | 0.450 | 0.000 | 0.083 | 1.000 |
| *Alistipes* | 0.000 | 0.042 | 1 | 0.000 | 0.052 | 1.000 | 0.048 | 0.110 | 0.400 |
| *Alkaliphilus* | 0.148 | 0.093 | 0.03 | 0.157 | 0.115 | 0.060 | 0.125 | 0.122 | 0.140 |
| *Alloactinosynnema* | 0.061 | 0.081 | 0.19 | 0.042 | 0.083 | 0.320 | 0.118 | 0.133 | 0.190 |
| *Aminobacter* | 0.000 | 0.040 | 1 | 0.000 | 0.052 | 1.000 | 0.038 | 0.097 | 0.420 |
| *Amphibacillus* | 0.000 | 0.045 | 1 | 0.000 | 0.062 | 1.000 | 0.000 | 0.067 | 1.000 |
| *Amycolatopsis* | 0.048 | 0.066 | 0.19 | 0.099 | 0.107 | 0.160 | 0.000 | 0.091 | 1.000 |
| *Anaerococcus* | 0.030 | 0.056 | 0.29 | 0.088 | 0.089 | 0.230 | 0.000 | 0.079 | 1.000 |
| *Anaerolinea* | 0.097 | 0.082 | 0.1 | 0.090 | 0.098 | 0.180 | 0.099 | 0.130 | 0.240 |
| *Aneurinibacillus* | 0.016 | 0.055 | 0.45 | 0.068 | 0.082 | 0.320 | 0.000 | 0.075 | 1.000 |
| *Anoxybacillus* | 0.080 | 0.067 | 0.12 | 0.154 | 0.106 | 0.100 | 0.000 | 0.069 | 1.000 |
| *Anthracocystis* | 0.000 | 0.040 | 1 | 0.000 | 0.050 | 1.000 | 0.067 | 0.107 | 0.350 |
| *Arachidicoccus* | 0.035 | 0.053 | 0.2 | 0.023 | 0.067 | 0.470 | 0.053 | 0.102 | 0.320 |
| *Arcanobacterium* | 0.091 | 0.076 | 0.12 | 0.071 | 0.085 | 0.240 | 0.126 | 0.129 | 0.170 |
| *Arcobacter* | 0.111 | 0.075 | 0.04 | 0.118 | 0.110 | 0.090 | 0.101 | 0.126 | 0.170 |
| *Arthrospira* | 0.000 | 0.045 | 1 | 0.000 | 0.058 | 1.000 | 0.039 | 0.096 | 0.390 |
| *Asaia* | 0.000 | 0.048 | 1 | 0.000 | 0.000 | 0.810 | 0.000 | 0.084 | 1.000 |
| *Aureobasidium* | 0.084 | 0.075 | 0.14 | 0.065 | 0.080 | 0.210 | 0.157 | 0.144 | 0.100 |
| *Aureococcus* | 0.044 | 0.062 | 0.24 | 0.055 | 0.080 | 0.350 | 0.018 | 0.082 | 0.430 |
| *Auricularia* | 0.073 | 0.072 | 0.17 | 0.071 | 0.084 | 0.240 | 0.153 | 0.141 | 0.130 |
| *Azotobacter* | 0.084 | 0.085 | 0.13 | 0.072 | 0.092 | 0.210 | 0.179 | 0.148 | 0.080 |
| *Bacillus* | 0.053 | 0.066 | 0.24 | 0.003 | 0.059 | 0.550 | 0.146 | 0.129 | 0.160 |
| *Bacteroides* | 0.166 | 0.087 | 0.01 | 0.166 | 0.116 | 0.070 | 0.150 | 0.137 | 0.160 |
| *Barnesiella* | 0.065 | 0.069 | 0.2 | 0.126 | 0.103 | 0.130 | 0.000 | 0.081 | 1.000 |

| | | | | | | | | | |
|---|---|---|---|---|---|---|---|---|---|
| *Bartonella* | 0.007 | 0.032 | 0.47 | 0.038 | 0.088 | 0.300 | 0.000 | 0.075 | 1.000 |
| *Basilea* | 0.000 | 0.045 | 1 | 0.000 | 0.066 | 1.000 | 0.045 | 0.098 | 0.320 |
| *Baudoinia* | 0.027 | 0.058 | 0.37 | 0.041 | 0.076 | 0.340 | 0.000 | 0.086 | 1.000 |
| *Bdellovibrio* | 0.101 | 0.075 | 0.08 | 0.141 | 0.101 | 0.110 | 0.000 | 0.082 | 1.000 |
| *Belliella* | 0.000 | 0.041 | 1 | 0.000 | 0.057 | 1.000 | 0.023 | 0.076 | 0.480 |
| *Beutenbergia* | 0.110 | 0.080 | 0.09 | 0.164 | 0.120 | 0.060 | 0.000 | 0.075 | 1.000 |
| *Blastochloris* | 0.181 | 0.096 | 0.03 | 0.183 | 0.123 | 0.050 | 0.131 | 0.125 | 0.190 |
| *Blastococcus* | 0.040 | 0.067 | 0.38 | 0.018 | 0.069 | 0.500 | 0.157 | 0.140 | 0.100 |
| *Blastomonas* | 0.127 | 0.085 | 0.01 | 0.151 | 0.099 | 0.100 | 0.000 | 0.074 | 1.000 |
| *Borreliella* | 0.088 | 0.075 | 0.14 | 0.098 | 0.110 | 0.150 | 0.082 | 0.105 | 0.310 |
| *Bosea* | 0.000 | 0.038 | 1 | 0.000 | 0.063 | 1.000 | 0.088 | 0.111 | 0.250 |
| *Botrytis* | 0.205 | 0.098 | 0.01 | 0.195 | 0.116 | 0.050 | 0.216 | 0.148 | 0.060 |
| *Brachybacterium* | 0.000 | 0.048 | 1 | 0.000 | 0.047 | 1.000 | 0.075 | 0.106 | 0.240 |
| *Brenneria* | 0.000 | 0.041 | 1 | 0.000 | 0.000 | 0.150 | 0.000 | 0.066 | 1.000 |
| *Brevibacillus* | 0.052 | 0.068 | 0.31 | 0.101 | 0.108 | 0.160 | 0.000 | 0.076 | 1.000 |
| *Brevibacterium* | 0.032 | 0.056 | 0.36 | 0.097 | 0.099 | 0.150 | 0.000 | 0.081 | 1.000 |
| *Brucella* | 0.130 | 0.087 | 0.05 | 0.135 | 0.110 | 0.090 | 0.168 | 0.146 | 0.110 |
| *Buchnera* | 0.166 | 0.089 | 0.07 | 0.137 | 0.102 | 0.070 | 0.215 | 0.158 | 0.070 |
| *Butyrivibrio* | 0.028 | 0.052 | 0.29 | 0.006 | 0.070 | 0.620 | 0.081 | 0.112 | 0.290 |
| *Caldanaerobacter* | 0.025 | 0.059 | 0.29 | 0.000 | 0.063 | 1.000 | 0.124 | 0.117 | 0.110 |
| *Caldicellulosiruptor* | 0.016 | 0.048 | 0.41 | 0.045 | 0.077 | 0.370 | 0.000 | 0.070 | 1.000 |
| *Caldithrix* | 0.127 | 0.078 | 0.06 | 0.093 | 0.101 | 0.200 | 0.225 | 0.155 | 0.050 |
| *Candidatus Accumulibacter* | 0.052 | 0.068 | 0.23 | 0.037 | 0.067 | 0.410 | 0.099 | 0.123 | 0.190 |
| *Candidatus Arthromitus* | 0.000 | 0.040 | 1 | 0.012 | 0.055 | 0.600 | 0.000 | 0.073 | 1.000 |
| *Candidatus Azobacteroides* | 0.047 | 0.064 | 0.25 | 0.088 | 0.087 | 0.170 | 0.000 | 0.073 | 1.000 |
| *Candidatus Babela* | 0.050 | 0.053 | 0.37 | 0.000 | 0.051 | 1.000 | 0.191 | 0.153 | 0.050 |
| *Candidatus Blochmannia* | 0.059 | 0.073 | 0.26 | 0.130 | 0.101 | 0.070 | 0.000 | 0.083 | 1.000 |
| *Candidatus Cardinium* | 0.018 | 0.060 | 0.42 | 0.026 | 0.074 | 0.440 | 0.000 | 0.078 | 1.000 |
| *Candidatus Cloacimonas* | 0.137 | 0.083 | 0.03 | 0.171 | 0.119 | 0.070 | 0.054 | 0.103 | 0.330 |
| *Candidatus Desulforudis* | 0.000 | 0.040 | 1 | 0.000 | 0.050 | 1.000 | 0.000 | 0.067 | 1.000 |
| *Candidatus Hamiltonella* | 0.104 | 0.069 | 0.06 | 0.100 | 0.101 | 0.150 | 0.104 | 0.113 | 0.250 |
| *Candidatus Ishikawaella* | 0.127 | 0.085 | 0.02 | 0.142 | 0.117 | 0.060 | 0.075 | 0.110 | 0.280 |
| *Candidatus Izimaplasma* | 0.000 | 0.046 | 1 | 0.000 | 0.060 | 1.000 | 0.000 | 0.076 | 1.000 |
| *Candidatus Koribacter* | 0.161 | 0.089 | 0.02 | 0.187 | 0.114 | 0.070 | 0.000 | 0.079 | 1.000 |
| *Candidatus Liberibacter* | 0.102 | 0.080 | 0.07 | 0.123 | 0.097 | 0.070 | 0.016 | 0.079 | 0.370 |
| *Candidatus Methanoplasma* | 0.122 | 0.083 | 0.09 | 0.110 | 0.094 | 0.100 | 0.143 | 0.128 | 0.120 |
| *Candidatus Mikella* | 0.044 | 0.064 | 0.29 | 0.087 | 0.093 | 0.130 | 0.000 | 0.085 | 1.000 |
| *Candidatus Paracaedibacter* | 0.120 | 0.085 | 0.03 | 0.123 | 0.106 | 0.110 | 0.107 | 0.123 | 0.180 |
| *Candidatus Pelagibacter* | 0.034 | 0.060 | 0.37 | 0.012 | 0.064 | 0.440 | 0.055 | 0.094 | 0.330 |
| *Candidatus Portiera* | 0.097 | 0.072 | 0.06 | 0.170 | 0.099 | 0.060 | 0.000 | 0.070 | 1.000 |
| *Candidatus Puniceispirillum* | 0.095 | 0.074 | 0.1 | 0.133 | 0.094 | 0.110 | 0.000 | 0.076 | 1.000 |
| *Candidatus Symbiobacter* | 0.026 | 0.058 | 0.37 | 0.000 | 0.052 | 1.000 | 0.159 | 0.142 | 0.120 |
| *Candidatus Tachikawaea* | 0.113 | 0.080 | 0.07 | 0.116 | 0.084 | 0.110 | 0.119 | 0.113 | 0.230 |
| *Capnocytophaga* | 0.000 | 0.045 | 1 | 0.000 | 0.051 | 1.000 | 0.000 | 0.072 | 1.000 |
| *Carnobacterium* | 0.000 | 0.040 | 1 | 0.015 | 0.064 | 0.440 | 0.000 | 0.086 | 1.000 |
| *Catenulispora* | 0.071 | 0.069 | 0.13 | 0.082 | 0.095 | 0.200 | 0.022 | 0.082 | 0.430 |
| *Celeribacter* | 0.018 | 0.060 | 0.42 | 0.030 | 0.078 | 0.420 | 0.000 | 0.079 | 1.000 |

| | | | | | | | | | |
|---|---|---|---|---|---|---|---|---|---|
| *Cellulophaga* | 0.110 | 0.076 | 0.11 | 0.173 | 0.116 | 0.070 | 0.000 | 0.094 | 1.000 |
| *Cellulosilyticum* | 0.048 | 0.053 | 0.28 | 0.083 | 0.096 | 0.220 | 0.000 | 0.070 | 1.000 |
| *Cellvibrio* | 0.148 | 0.083 | 0.02 | 0.151 | 0.111 | 0.080 | 0.116 | 0.129 | 0.170 |
| *Chania* | 0.107 | 0.081 | 0.07 | 0.117 | 0.102 | 0.100 | 0.125 | 0.139 | 0.160 |
| *Chitinophaga* | 0.051 | 0.065 | 0.33 | 0.070 | 0.104 | 0.170 | 0.006 | 0.075 | 0.490 |
| *Chlamydia* | 0.038 | 0.068 | 0.38 | 0.000 | 0.051 | 1.000 | 0.207 | 0.146 | 0.080 |
| *Chloracidobacterium* | 0.036 | 0.050 | 0.4 | 0.032 | 0.070 | 0.450 | 0.115 | 0.122 | 0.170 |
| *Chlorobium* | 0.160 | 0.080 | 0.02 | 0.186 | 0.127 | 0.050 | 0.003 | 0.066 | 0.650 |
| *Chloroherpeton* | 0.051 | 0.056 | 0.19 | 0.070 | 0.077 | 0.230 | 0.000 | 0.100 | 1.000 |
| *Chromohalobacter* | 0.125 | 0.078 | 0.03 | 0.139 | 0.109 | 0.090 | 0.048 | 0.102 | 0.360 |
| *Chroococcidiopsis* | 0.000 | 0.041 | 1 | 0.000 | 0.053 | 1.000 | 0.000 | 0.074 | 1.000 |
| *Citrobacter* | 0.033 | 0.054 | 0.42 | 0.000 | 0.053 | 1.000 | 0.111 | 0.129 | 0.190 |
| *Citromicrobium* | 0.000 | 0.043 | 1 | 0.000 | 0.054 | 1.000 | 0.000 | 0.000 | 0.930 |
| *Clostridium* | 0.033 | 0.055 | 0.32 | 0.051 | 0.066 | 0.320 | 0.000 | 0.071 | 1.000 |
| *Colwellia* | 0.076 | 0.067 | 0.17 | 0.095 | 0.096 | 0.210 | 0.000 | 0.065 | 1.000 |
| *Confluentimicrobium* | 0.094 | 0.079 | 0.11 | 0.115 | 0.094 | 0.130 | 0.043 | 0.104 | 0.420 |
| *Congregibacter* | 0.000 | 0.043 | 1 | 0.000 | 0.044 | 1.000 | 0.061 | 0.109 | 0.290 |
| *Coniosporium* | 0.000 | 0.045 | 1 | 0.000 | 0.053 | 1.000 | 0.000 | 0.071 | 1.000 |
| *Coprinopsis* | 0.076 | 0.067 | 0.16 | 0.099 | 0.090 | 0.190 | 0.009 | 0.079 | 0.540 |
| *Corallococcus* | 0.000 | 0.043 | 1 | 0.000 | 0.052 | 1.000 | 0.000 | 0.087 | 1.000 |
| *Coxiella* | 0.031 | 0.052 | 0.34 | 0.007 | 0.053 | 0.480 | 0.102 | 0.120 | 0.260 |
| *Crinalium* | 0.103 | 0.078 | 0.12 | 0.104 | 0.091 | 0.200 | 0.000 | 0.000 | 0.550 |
| *Cryobacterium* | 0.008 | 0.044 | 0.55 | 0.021 | 0.065 | 0.440 | 0.002 | 0.073 | 0.510 |
| *Cryptococcus* | 0.000 | 0.042 | 1 | 0.000 | 0.056 | 1.000 | 0.000 | 0.000 | 0.280 |
| *Cutibacterium* | 0.078 | 0.077 | 0.16 | 0.091 | 0.090 | 0.170 | 0.018 | 0.086 | 0.450 |
| *Cyanobacterium* | 0.002 | 0.047 | 0.57 | 0.000 | 0.051 | 1.000 | 0.046 | 0.104 | 0.360 |
| *Cyclobacterium* | 0.000 | 0.041 | 1 | 0.000 | 0.062 | 1.000 | 0.000 | 0.066 | 1.000 |
| *Cylindrospermum* | 0.116 | 0.070 | 0.03 | 0.109 | 0.092 | 0.110 | 0.147 | 0.148 | 0.150 |
| *Cyphellophora* | 0.053 | 0.064 | 0.24 | 0.054 | 0.084 | 0.290 | 0.081 | 0.097 | 0.270 |
| *Cytophaga* | 0.181 | 0.082 | 0.02 | 0.171 | 0.101 | 0.060 | 0.190 | 0.150 | 0.100 |
| *Defluviitoga* | 0.039 | 0.056 | 0.27 | 0.038 | 0.083 | 0.260 | 0.062 | 0.097 | 0.280 |
| *Dehalobacter* | 0.039 | 0.056 | 0.35 | 0.025 | 0.069 | 0.460 | 0.083 | 0.120 | 0.270 |
| *Delftia* | 0.102 | 0.081 | 0.04 | 0.132 | 0.113 | 0.140 | 0.071 | 0.112 | 0.230 |
| *Denitrobacterium* | 0.061 | 0.067 | 0.18 | 0.085 | 0.092 | 0.130 | 0.014 | 0.057 | 0.470 |
| *Denitrovibrio* | 0.000 | 0.049 | 1 | 0.000 | 0.062 | 1.000 | 0.106 | 0.125 | 0.280 |
| *Dermabacter* | 0.150 | 0.090 | 0.04 | 0.173 | 0.119 | 0.060 | 0.000 | 0.073 | 1.000 |
| *Desulfarculus* | 0.000 | 0.042 | 1 | 0.000 | 0.063 | 1.000 | 0.069 | 0.115 | 0.270 |
| *Desulfatibacillum* | 0.066 | 0.075 | 0.16 | 0.149 | 0.107 | 0.050 | 0.000 | 0.091 | 1.000 |
| *Desulfobacca* | 0.000 | 0.048 | 1 | 0.000 | 0.061 | 1.000 | 0.157 | 0.143 | 0.130 |
| *Desulfobacula* | 0.000 | 0.046 | 1 | 0.000 | 0.051 | 1.000 | 0.000 | 0.079 | 1.000 |
| *Desulfobulbus* | 0.126 | 0.088 | 0.08 | 0.141 | 0.117 | 0.060 | 0.074 | 0.119 | 0.260 |
| *Desulfohalobium* | 0.000 | 0.043 | 1 | 0.000 | 0.053 | 1.000 | 0.000 | 0.071 | 1.000 |
| *Desulfomicrobium* | 0.104 | 0.081 | 0.1 | 0.078 | 0.082 | 0.210 | 0.141 | 0.116 | 0.180 |
| *Desulfomonile* | 0.000 | 0.043 | 1 | 0.000 | 0.058 | 1.000 | 0.045 | 0.112 | 0.410 |
| *Desulfotalea* | 0.000 | 0.034 | 1 | 0.000 | 0.056 | 1.000 | 0.000 | 0.070 | 1.000 |
| *Desulfotomaculum* | 0.089 | 0.085 | 0.17 | 0.114 | 0.109 | 0.110 | 0.052 | 0.111 | 0.400 |
| *Desulfurella* | 0.077 | 0.069 | 0.14 | 0.120 | 0.108 | 0.120 | 0.000 | 0.085 | 1.000 |
| *Desulfurivibrio* | 0.032 | 0.068 | 0.3 | 0.022 | 0.065 | 0.470 | 0.056 | 0.098 | 0.370 |
| *Desulfurobacterium* | 0.000 | 0.051 | 1 | 0.007 | 0.052 | 0.480 | 0.000 | 0.067 | 1.000 |
| *Desulfuromonas* | 0.110 | 0.077 | 0.07 | 0.142 | 0.102 | 0.060 | 0.053 | 0.101 | 0.350 |
| *Devriesea* | 0.104 | 0.081 | 0.12 | 0.102 | 0.087 | 0.170 | 0.047 | 0.105 | 0.410 |
| *Dialister* | 0.167 | 0.096 | 0.04 | 0.174 | 0.120 | 0.070 | 0.028 | 0.103 | 0.530 |
| *Dichelobacter* | 0.058 | 0.066 | 0.17 | 0.062 | 0.080 | 0.220 | 0.046 | 0.098 | 0.350 |

| | | | | | | | | | |
|---|---|---|---|---|---|---|---|---|---|
| *Dichomitus* | 0.095 | 0.079 | 0.17 | 0.087 | 0.099 | 0.150 | 0.142 | 0.134 | 0.120 |
| *Dickeya* | 0.173 | 0.095 | 0.01 | 0.160 | 0.115 | 0.060 | 0.175 | 0.132 | 0.070 |
| *Dietzia* | 0.000 | 0.034 | 1 | 0.000 | 0.051 | 1.000 | 0.207 | 0.159 | 0.060 |
| *Dinoroseobacter* | 0.024 | 0.056 | 0.35 | 0.024 | 0.063 | 0.420 | 0.107 | 0.112 | 0.210 |
| *Dokdonella* | 0.170 | 0.081 | 0.04 | 0.192 | 0.114 | 0.050 | 0.000 | 0.073 | 1.000 |
| *Dokdonia* | 0.068 | 0.073 | 0.15 | 0.099 | 0.105 | 0.110 | 0.000 | 0.070 | 1.000 |
| *Donghicola* | 0.033 | 0.056 | 0.28 | 0.000 | 0.063 | 1.000 | 0.213 | 0.146 | 0.050 |
| *Draconibacterium* | 0.064 | 0.063 | 0.23 | 0.007 | 0.061 | 0.530 | 0.149 | 0.146 | 0.070 |
| *Dyadobacter* | 0.133 | 0.081 | 0.04 | 0.144 | 0.105 | 0.070 | 0.069 | 0.104 | 0.330 |
| *Echinicola* | 0.043 | 0.058 | 0.42 | 0.000 | 0.050 | 1.000 | 0.141 | 0.130 | 0.210 |
| *Elusimicrobium* | 0.000 | 0.039 | 1 | 0.000 | 0.051 | 1.000 | 0.000 | 0.067 | 1.000 |
| *Emticicia* | 0.038 | 0.066 | 0.28 | 0.032 | 0.079 | 0.350 | 0.010 | 0.087 | 0.540 |
| *Endomicrobium* | 0.093 | 0.079 | 0.09 | 0.104 | 0.085 | 0.150 | 0.043 | 0.099 | 0.300 |
| *Endozoicomonas* | 0.096 | 0.081 | 0.08 | 0.081 | 0.087 | 0.180 | 0.198 | 0.157 | 0.080 |
| *Erwinia* | 0.108 | 0.087 | 0.06 | 0.090 | 0.092 | 0.220 | 0.158 | 0.114 | 0.120 |
| *Erysipelothrix* | 0.103 | 0.076 | 0.07 | 0.147 | 0.111 | 0.070 | 0.000 | 0.083 | 1.000 |
| *Erythrobacter* | 0.039 | 0.051 | 0.26 | 0.000 | 0.057 | 1.000 | 0.225 | 0.164 | 0.070 |
| *Exophiala* | 0.102 | 0.079 | 0.13 | 0.167 | 0.122 | 0.060 | 0.000 | 0.079 | 1.000 |
| *Ezakiella* | 0.019 | 0.050 | 0.34 | 0.009 | 0.065 | 0.500 | 0.074 | 0.115 | 0.260 |
| *Fermentimonas* | 0.000 | 0.052 | 0.54 | 0.000 | 0.062 | 1.000 | 0.105 | 0.111 | 0.220 |
| *Ferrimonas* | 0.091 | 0.082 | 0.12 | 0.080 | 0.105 | 0.250 | 0.168 | 0.129 | 0.090 |
| *Fervidobacterium* | 0.036 | 0.057 | 0.35 | 0.064 | 0.091 | 0.270 | 0.000 | 0.064 | 1.000 |
| *Fibrella* | 0.044 | 0.062 | 0.23 | 0.076 | 0.084 | 0.220 | 0.000 | 0.071 | 1.000 |
| *Fictibacillus* | 0.085 | 0.066 | 0.11 | 0.134 | 0.106 | 0.130 | 0.000 | 0.067 | 1.000 |
| *Filifactor* | 0.031 | 0.070 | 0.38 | 0.032 | 0.069 | 0.400 | 0.022 | 0.086 | 0.490 |
| *Filomicrobium* | 0.017 | 0.051 | 0.54 | 0.010 | 0.052 | 0.520 | 0.046 | 0.094 | 0.390 |
| *Fimbriimonas* | 0.080 | 0.074 | 0.09 | 0.088 | 0.090 | 0.190 | 0.113 | 0.125 | 0.110 |
| *Finegoldia* | 0.053 | 0.076 | 0.21 | 0.119 | 0.106 | 0.120 | 0.000 | 0.091 | 1.000 |
| *Fischerella* | 0.000 | 0.046 | 1 | 0.000 | 0.065 | 1.000 | 0.000 | 0.078 | 1.000 |
| *Flammeovirga* | 0.000 | 0.039 | 1 | 0.000 | 0.058 | 1.000 | 0.000 | 0.071 | 1.000 |
| *Flavisolibacter* | 0.116 | 0.077 | 0.06 | 0.145 | 0.114 | 0.130 | 0.009 | 0.074 | 0.550 |
| *Flavobacterium* | 0.059 | 0.076 | 0.16 | 0.083 | 0.082 | 0.220 | 0.000 | 0.073 | 1.000 |
| *Flexistipes* | 0.102 | 0.084 | 0.13 | 0.126 | 0.111 | 0.130 | 0.000 | 0.062 | 1.000 |
| *Formosa* | 0.000 | 0.037 | 1 | 0.000 | 0.062 | 1.000 | 0.000 | 0.087 | 1.000 |
| *Francisella* | 0.027 | 0.056 | 0.41 | 0.038 | 0.067 | 0.440 | 0.000 | 0.092 | 1.000 |
| *Frischella* | 0.138 | 0.088 | 0.03 | 0.146 | 0.107 | 0.070 | 0.059 | 0.102 | 0.320 |
| *Frondihabitans* | 0.104 | 0.070 | 0.1 | 0.136 | 0.105 | 0.050 | 0.086 | 0.127 | 0.340 |
| *Fuerstia* | 0.000 | 0.040 | 1 | 0.000 | 0.056 | 1.000 | 0.000 | 0.076 | 1.000 |
| *Fusobacterium* | 0.066 | 0.073 | 0.21 | 0.077 | 0.098 | 0.080 | 0.054 | 0.109 | 0.440 |
| *Gallibacterium* | 0.077 | 0.069 | 0.1 | 0.146 | 0.110 | 0.100 | 0.000 | 0.080 | 1.000 |
| *Gallionella* | 0.065 | 0.062 | 0.19 | 0.050 | 0.075 | 0.360 | 0.127 | 0.115 | 0.130 |
| *Gardnerella* | 0.103 | 0.080 | 0.09 | 0.094 | 0.099 | 0.190 | 0.100 | 0.119 | 0.230 |
| *Geitlerinema* | 0.029 | 0.053 | 0.4 | 0.045 | 0.070 | 0.390 | 0.000 | 0.074 | 1.000 |
| *Gemella* | 0.026 | 0.046 | 0.36 | 0.009 | 0.075 | 0.450 | 0.044 | 0.090 | 0.370 |
| *Geminocystis* | 0.000 | 0.044 | 1 | 0.000 | 0.063 | 1.000 | 0.000 | 0.085 | 1.000 |
| *Geodermatophilus* | 0.101 | 0.072 | 0.06 | 0.078 | 0.092 | 0.250 | 0.187 | 0.129 | 0.060 |
| *Gloeocapsa* | 0.000 | 0.044 | 1 | 0.000 | 0.046 | 1.000 | 0.105 | 0.122 | 0.220 |
| *Gloeomargarita* | 0.068 | 0.078 | 0.22 | 0.097 | 0.096 | 0.140 | 0.003 | 0.075 | 0.490 |
| *Gluconacetobacter* | 0.048 | 0.056 | 0.24 | 0.072 | 0.091 | 0.230 | 0.000 | 0.085 | 1.000 |
| *Gluconobacter* | 0.019 | 0.050 | 0.46 | 0.048 | 0.073 | 0.340 | 0.000 | 0.069 | 1.000 |
| *Glutamicibacter* | 0.000 | 0.043 | 1 | 0.000 | 0.068 | 1.000 | 0.000 | 0.075 | 1.000 |
| *Gottschalkia* | 0.000 | 0.046 | 1 | 0.000 | 0.062 | 1.000 | 0.000 | 0.084 | 1.000 |
| *Gramella* | 0.000 | 0.043 | 1 | 0.000 | 0.055 | 1.000 | 0.000 | 0.068 | 1.000 |

| | | | | | | | | | |
|---|---|---|---|---|---|---|---|---|---|
| *Granulibacter* | 0.026 | 0.050 | 0.34 | 0.018 | 0.066 | 0.490 | 0.029 | 0.080 | 0.370 |
| *Granulicella* | 0.105 | 0.074 | 0.09 | 0.083 | 0.088 | 0.200 | 0.126 | 0.139 | 0.100 |
| *Grimontia* | 0.132 | 0.086 | 0.05 | 0.129 | 0.109 | 0.090 | 0.105 | 0.121 | 0.210 |
| *Gynuella* | 0.080 | 0.069 | 0.17 | 0.077 | 0.084 | 0.220 | 0.024 | 0.078 | 0.480 |
| *Hafnia* | 0.000 | 0.041 | 1 | 0.000 | 0.046 | 1.000 | 0.008 | 0.072 | 0.560 |
| *Hahella* | 0.002 | 0.045 | 0.51 | 0.000 | 0.057 | 1.000 | 0.156 | 0.132 | 0.150 |
| *Haladaptatus* | 0.005 | 0.045 | 0.54 | 0.000 | 0.064 | 1.000 | 0.199 | 0.154 | 0.080 |
| *Haliangium* | 0.089 | 0.070 | 0.08 | 0.155 | 0.111 | 0.110 | 0.000 | 0.064 | 1.000 |
| *Halioglobus* | 0.135 | 0.081 | 0.05 | 0.133 | 0.113 | 0.090 | 0.114 | 0.115 | 0.160 |
| *Haliscomenobacter* | 0.000 | 0.045 | 1 | 0.000 | 0.062 | 1.000 | 0.000 | 0.060 | 1.000 |
| *Haloarcula* | 0.000 | 0.037 | 1 | 0.000 | 0.044 | 1.000 | 0.025 | 0.079 | 0.380 |
| *Halobacteriovorax* | 0.000 | 0.028 | 1 | 0.000 | 0.057 | 1.000 | 0.098 | 0.113 | 0.220 |
| *Halobacterium* | 0.078 | 0.065 | 0.1 | 0.111 | 0.101 | 0.110 | 0.000 | 0.070 | 1.000 |
| *Halobacteroides* | 0.055 | 0.064 | 0.28 | 0.096 | 0.098 | 0.130 | 0.000 | 0.061 | 1.000 |
| *Haloferax* | 0.099 | 0.071 | 0.1 | 0.063 | 0.078 | 0.210 | 0.199 | 0.140 | 0.080 |
| *Halogeometricum* | 0.000 | 0.042 | 1 | 0.000 | 0.058 | 1.000 | 0.000 | 0.082 | 1.000 |
| *Halolamina* | 0.181 | 0.099 | 0.03 | 0.207 | 0.117 | 0.050 | 0.000 | 0.088 | 1.000 |
| *Halomicrobium* | 0.102 | 0.078 | 0.08 | 0.109 | 0.104 | 0.200 | 0.000 | 0.084 | 1.000 |
| *Haloquadratum* | 0.160 | 0.089 | 0.02 | 0.172 | 0.103 | 0.060 | 0.023 | 0.084 | 0.510 |
| *Halosimplex* | 0.190 | 0.099 | 0.02 | 0.200 | 0.126 | 0.050 | 0.025 | 0.091 | 0.430 |
| *Halotalea* | 0.153 | 0.085 | 0.02 | 0.168 | 0.115 | 0.050 | 0.056 | 0.093 | 0.310 |
| *Haloterrigena* | 0.107 | 0.078 | 0.06 | 0.040 | 0.078 | 0.290 | 0.227 | 0.157 | 0.050 |
| *Halothermothrix* | 0.056 | 0.063 | 0.23 | 0.078 | 0.093 | 0.240 | 0.000 | 0.077 | 1.000 |
| *Heterobasidion* | 0.000 | 0.053 | 1 | 0.000 | 0.053 | 1.000 | 0.000 | 0.063 | 1.000 |
| *Histophilus* | 0.000 | 0.033 | 1 | 0.000 | 0.051 | 1.000 | 0.000 | 0.072 | 1.000 |
| *Hoeflea* | 0.037 | 0.066 | 0.3 | 0.024 | 0.059 | 0.470 | 0.069 | 0.100 | 0.360 |
| *Hoyosella* | 0.146 | 0.080 | 0.04 | 0.173 | 0.121 | 0.070 | 0.000 | 0.064 | 1.000 |
| *Hydrogenobaculum* | 0.003 | 0.050 | 0.54 | 0.004 | 0.057 | 0.550 | 0.000 | 0.078 | 1.000 |
| *Hydrogenophaga* | 0.162 | 0.091 | 0.01 | 0.189 | 0.113 | 0.050 | 0.000 | 0.084 | 0.570 |
| *Hymenobacter* | 0.028 | 0.057 | 0.33 | 0.032 | 0.070 | 0.420 | 0.051 | 0.103 | 0.470 |
| *Hyphomicrobium* | 0.123 | 0.075 | 0.04 | 0.117 | 0.097 | 0.130 | 0.133 | 0.134 | 0.190 |
| *Idiomarina* | 0.053 | 0.069 | 0.19 | 0.066 | 0.081 | 0.200 | 0.114 | 0.114 | 0.220 |
| *Ignavibacterium* | 0.030 | 0.060 | 0.3 | 0.068 | 0.084 | 0.210 | 0.000 | 0.061 | 1.000 |
| *Ilumatobacter* | 0.000 | 0.043 | 1 | 0.000 | 0.051 | 1.000 | 0.000 | 0.058 | 1.000 |
| *Ilyobacter* | 0.000 | 0.044 | 1 | 0.000 | 0.058 | 1.000 | 0.000 | 0.000 | 0.560 |
| *Intrasporangium* | 0.099 | 0.076 | 0.11 | 0.136 | 0.101 | 0.140 | 0.005 | 0.073 | 0.490 |
| *Isoptericola* | 0.127 | 0.086 | 0.1 | 0.171 | 0.116 | 0.050 | 0.119 | 0.120 | 0.140 |
| *Isosphaera* | 0.000 | 0.042 | 1 | 0.000 | 0.067 | 1.000 | 0.066 | 0.107 | 0.310 |
| *Janibacter* | 0.133 | 0.081 | 0.03 | 0.180 | 0.113 | 0.050 | 0.018 | 0.075 | 0.480 |
| *Jannaschia* | 0.075 | 0.063 | 0.15 | 0.078 | 0.089 | 0.150 | 0.053 | 0.098 | 0.340 |
| *Janthinobacterium* | 0.045 | 0.061 | 0.3 | 0.077 | 0.085 | 0.220 | 0.019 | 0.088 | 0.430 |
| *Jeongeupia* | 0.179 | 0.105 | 0.03 | 0.194 | 0.134 | 0.050 | 0.163 | 0.160 | 0.070 |
| *Jeotgalibaca* | 0.088 | 0.070 | 0.14 | 0.089 | 0.093 | 0.160 | 0.077 | 0.110 | 0.240 |
| *Kangiella* | 0.108 | 0.086 | 0.06 | 0.130 | 0.106 | 0.170 | 0.020 | 0.082 | 0.520 |
| *Kibdelosporangium* | 0.054 | 0.061 | 0.19 | 0.101 | 0.099 | 0.210 | 0.000 | 0.057 | 1.000 |
| *Kineococcus* | 0.050 | 0.061 | 0.26 | 0.043 | 0.067 | 0.300 | 0.058 | 0.110 | 0.310 |
| *Kinetoplastibacterium* | 0.000 | 0.042 | 1 | 0.000 | 0.062 | 1.000 | 0.000 | 0.069 | 1.000 |
| *Kiritimatiella* | 0.000 | 0.038 | 1 | 0.039 | 0.070 | 0.270 | 0.000 | 0.085 | 1.000 |
| *Kitasatospora* | 0.092 | 0.070 | 0.12 | 0.096 | 0.101 | 0.160 | 0.094 | 0.118 | 0.220 |
| *Klebsiella* | 0.190 | 0.092 | 0.01 | 0.187 | 0.119 | 0.050 | 0.163 | 0.131 | 0.120 |
| *Kluyveromyces* | 0.101 | 0.085 | 0.04 | 0.064 | 0.082 | 0.330 | 0.225 | 0.155 | 0.050 |
| *Kocuria* | 0.056 | 0.064 | 0.19 | 0.068 | 0.096 | 0.260 | 0.000 | 0.072 | 1.000 |
| *Komagataeibacter* | 0.105 | 0.076 | 0.05 | 0.089 | 0.093 | 0.170 | 0.170 | 0.147 | 0.080 |

| | | | | | | | | | |
|---|---|---|---|---|---|---|---|---|---|
| *Kozakia* | 0.045 | 0.062 | 0.26 | 0.043 | 0.077 | 0.460 | 0.046 | 0.108 | 0.410 |
| *Kribbella* | 0.100 | 0.084 | 0.13 | 0.138 | 0.110 | 0.080 | 0.000 | 0.058 | 1.000 |
| *Kurthia* | 0.034 | 0.064 | 0.34 | 0.064 | 0.092 | 0.280 | 0.000 | 0.085 | 1.000 |
| *Kutzneria* | 0.076 | 0.072 | 0.15 | 0.095 | 0.088 | 0.110 | 0.031 | 0.099 | 0.570 |
| *Kyrpidia* | 0.057 | 0.067 | 0.24 | 0.099 | 0.089 | 0.220 | 0.000 | 0.061 | 1.000 |
| *Labrenzia* | 0.094 | 0.078 | 0.12 | 0.108 | 0.108 | 0.110 | 0.020 | 0.088 | 0.490 |
| *Laccaria* | 0.168 | 0.092 | 0.02 | 0.158 | 0.116 | 0.080 | 0.182 | 0.136 | 0.090 |
| *Lacimicrobium* | 0.078 | 0.069 | 0.19 | 0.062 | 0.090 | 0.150 | 0.165 | 0.124 | 0.120 |
| *Lactococcus* | 0.000 | 0.062 | 1 | 0.042 | 0.090 | 0.350 | 0.000 | 0.068 | 1.000 |
| *Lawsonella* | 0.000 | 0.000 | 0.7 | 0.000 | 0.063 | 1.000 | 0.068 | 0.099 | 0.250 |
| *Lawsonia* | 0.068 | 0.072 | 0.2 | 0.064 | 0.069 | 0.240 | 0.088 | 0.103 | 0.250 |
| *Leadbetterella* | 0.118 | 0.082 | 0.06 | 0.123 | 0.095 | 0.080 | 0.029 | 0.078 | 0.490 |
| *Leclercia* | 0.148 | 0.091 | 0.03 | 0.176 | 0.125 | 0.050 | 0.009 | 0.073 | 0.560 |
| *Leifsonia* | 0.100 | 0.064 | 0.07 | 0.134 | 0.119 | 0.070 | 0.000 | 0.000 | 0.320 |
| *Leisingera* | 0.132 | 0.096 | 0.05 | 0.136 | 0.112 | 0.100 | 0.127 | 0.135 | 0.140 |
| *Lelliottia* | 0.062 | 0.074 | 0.25 | 0.022 | 0.080 | 0.440 | 0.162 | 0.149 | 0.100 |
| *Leptospira* | 0.036 | 0.068 | 0.36 | 0.055 | 0.072 | 0.290 | 0.000 | 0.073 | 1.000 |
| *Leptospirillum* | 0.000 | 0.046 | 1 | 0.000 | 0.059 | 1.000 | 0.030 | 0.090 | 0.490 |
| *Leptotrichia* | 0.022 | 0.056 | 0.36 | 0.050 | 0.082 | 0.300 | 0.000 | 0.075 | 1.000 |
| *Limnochorda* | 0.014 | 0.048 | 0.53 | 0.011 | 0.069 | 0.600 | 0.006 | 0.079 | 0.480 |
| *Listeria* | 0.000 | 0.039 | 1 | 0.000 | 0.056 | 1.000 | 0.000 | 0.071 | 1.000 |
| *Luteibacter* | 0.019 | 0.054 | 0.44 | 0.040 | 0.082 | 0.420 | 0.001 | 0.074 | 0.500 |
| *Lutibacter* | 0.090 | 0.084 | 0.07 | 0.092 | 0.089 | 0.210 | 0.056 | 0.101 | 0.350 |
| *Lysinibacillus* | 0.000 | 0.047 | 1 | 0.000 | 0.058 | 1.000 | 0.000 | 0.080 | 1.000 |
| *Macrococcus* | 0.096 | 0.074 | 0.12 | 0.153 | 0.114 | 0.080 | 0.000 | 0.073 | 1.000 |
| *Magnetospirillum* | 0.132 | 0.091 | 0.01 | 0.175 | 0.120 | 0.050 | 0.000 | 0.000 | 0.870 |
| *Mahella* | 0.072 | 0.073 | 0.15 | 0.023 | 0.059 | 0.490 | 0.218 | 0.156 | 0.060 |
| *Malassezia* | 0.125 | 0.084 | 0.03 | 0.107 | 0.092 | 0.140 | 0.219 | 0.159 | 0.060 |
| *Maribacter* | 0.000 | 0.039 | 1 | 0.000 | 0.069 | 1.000 | 0.000 | 0.063 | 1.000 |
| *Maricaulis* | 0.127 | 0.080 | 0.03 | 0.127 | 0.101 | 0.070 | 0.095 | 0.140 | 0.230 |
| *Marichromatium* | 0.000 | 0.047 | 1 | 0.013 | 0.056 | 0.590 | 0.000 | 0.072 | 1.000 |
| *Marinilactibacillus* | 0.011 | 0.047 | 0.47 | 0.088 | 0.079 | 0.150 | 0.000 | 0.070 | 1.000 |
| *Marinitoga* | 0.026 | 0.042 | 0.39 | 0.000 | 0.054 | 1.000 | 0.124 | 0.123 | 0.120 |
| *Marinobacterium* | 0.090 | 0.073 | 0.13 | 0.082 | 0.092 | 0.170 | 0.092 | 0.124 | 0.290 |
| *Marinomonas* | 0.021 | 0.057 | 0.4 | 0.015 | 0.055 | 0.470 | 0.025 | 0.089 | 0.400 |
| *Marinovum* | 0.000 | 0.044 | 1 | 0.000 | 0.045 | 1.000 | 0.152 | 0.130 | 0.140 |
| *Marivirga* | 0.150 | 0.102 | 0.03 | 0.151 | 0.115 | 0.080 | 0.061 | 0.110 | 0.280 |
| *Martelella* | 0.086 | 0.070 | 0.2 | 0.083 | 0.091 | 0.210 | 0.125 | 0.117 | 0.160 |
| *Megamonas* | 0.000 | 0.044 | 1 | 0.000 | 0.056 | 1.000 | 0.000 | 0.000 | 0.990 |
| *Megasphaera* | 0.111 | 0.082 | 0.07 | 0.123 | 0.107 | 0.150 | 0.092 | 0.130 | 0.280 |
| *Meiothermus* | 0.000 | 0.042 | 1 | 0.000 | 0.060 | 1.000 | 0.000 | 0.078 | 1.000 |
| *Melissococcus* | 0.000 | 0.047 | 1 | 0.000 | 0.062 | 1.000 | 0.000 | 0.056 | 1.000 |
| *Mesoplasma* | 0.102 | 0.083 | 0.04 | 0.058 | 0.077 | 0.260 | 0.171 | 0.145 | 0.050 |
| *Mesorhizobium* | 0.206 | 0.085 | 0.01 | 0.212 | 0.132 | 0.050 | 0.104 | 0.133 | 0.210 |
| *Metarhizium* | 0.097 | 0.074 | 0.07 | 0.201 | 0.115 | 0.050 | 0.000 | 0.073 | 1.000 |
| *Methanocorpusculum* | 0.064 | 0.070 | 0.18 | 0.086 | 0.088 | 0.150 | 0.009 | 0.087 | 0.370 |
| *Methanogenium* | 0.025 | 0.057 | 0.53 | 0.040 | 0.066 | 0.330 | 0.000 | 0.067 | 1.000 |
| *Methanomicrobium* | 0.024 | 0.048 | 0.46 | 0.000 | 0.066 | 1.000 | 0.114 | 0.118 | 0.140 |
| *Methanosaeta* | 0.048 | 0.063 | 0.21 | 0.079 | 0.094 | 0.230 | 0.000 | 0.078 | 1.000 |
| *Methanosphaera* | 0.167 | 0.084 | 0.01 | 0.147 | 0.099 | 0.080 | 0.184 | 0.134 | 0.070 |
| *Methylobacillus* | 0.023 | 0.050 | 0.4 | 0.034 | 0.087 | 0.430 | 0.010 | 0.078 | 0.490 |
| *Methyloceanibacter* | 0.125 | 0.084 | 0.03 | 0.125 | 0.107 | 0.090 | 0.099 | 0.134 | 0.180 |
| *Methylocella* | 0.161 | 0.092 | 0.01 | 0.173 | 0.109 | 0.060 | 0.132 | 0.147 | 0.140 |

| | | | | | | | | | |
|---|---|---|---|---|---|---|---|---|---|
| *Methylococcus* | 0.104 | 0.087 | 0.04 | 0.135 | 0.109 | 0.110 | 0.005 | 0.089 | 0.550 |
| *Methylocystis* | 0.011 | 0.045 | 0.38 | 0.014 | 0.069 | 0.350 | 0.000 | 0.093 | 1.000 |
| *Methylomicrobium* | 0.037 | 0.057 | 0.35 | 0.000 | 0.000 | 0.100 | 0.113 | 0.115 | 0.180 |
| *Methylophaga* | 0.108 | 0.075 | 0.06 | 0.173 | 0.112 | 0.050 | 0.013 | 0.079 | 0.540 |
| *Methylophilus* | 0.034 | 0.059 | 0.31 | 0.004 | 0.054 | 0.560 | 0.069 | 0.111 | 0.280 |
| *Methylotenera* | 0.110 | 0.085 | 0.08 | 0.093 | 0.110 | 0.140 | 0.151 | 0.135 | 0.130 |
| *Methylovorus* | 0.000 | 0.053 | 1 | 0.000 | 0.056 | 1.000 | 0.046 | 0.097 | 0.360 |
| *Metschnikowia* | 0.064 | 0.074 | 0.21 | 0.031 | 0.071 | 0.440 | 0.209 | 0.159 | 0.080 |
| *Meyerozyma* | 0.085 | 0.067 | 0.21 | 0.095 | 0.091 | 0.270 | 0.014 | 0.077 | 0.540 |
| *Micavibrio* | 0.033 | 0.052 | 0.3 | 0.023 | 0.081 | 0.390 | 0.106 | 0.123 | 0.170 |
| *Microbulbifer* | 0.140 | 0.081 | 0.02 | 0.141 | 0.114 | 0.140 | 0.116 | 0.120 | 0.140 |
| *Micrococcus* | 0.050 | 0.060 | 0.23 | 0.055 | 0.086 | 0.260 | 0.063 | 0.092 | 0.230 |
| *Microcoleus* | 0.022 | 0.054 | 0.36 | 0.000 | 0.059 | 1.000 | 0.181 | 0.130 | 0.100 |
| *Microcystis* | 0.022 | 0.053 | 0.3 | 0.000 | 0.056 | 1.000 | 0.085 | 0.113 | 0.210 |
| *Microlunatus* | 0.000 | 0.038 | 1 | 0.004 | 0.056 | 0.520 | 0.000 | 0.070 | 1.000 |
| *Micromonospora* | 0.063 | 0.070 | 0.28 | 0.083 | 0.095 | 0.170 | 0.082 | 0.134 | 0.220 |
| *Mitsuaria* | 0.130 | 0.085 | 0.04 | 0.193 | 0.123 | 0.050 | 0.020 | 0.078 | 0.420 |
| *Mobiluncus* | 0.000 | 0.047 | 1 | 0.000 | 0.066 | 1.000 | 0.223 | 0.169 | 0.050 |
| *Modestobacter* | 0.163 | 0.103 | 0.03 | 0.188 | 0.121 | 0.050 | 0.000 | 0.067 | 1.000 |
| *Moniliophthora* | 0.130 | 0.088 | 0.06 | 0.127 | 0.098 | 0.130 | 0.150 | 0.137 | 0.170 |
| *Moorea* | 0.000 | 0.042 | 0.47 | 0.009 | 0.064 | 0.470 | 0.000 | 0.077 | 1.000 |
| *Moraxella* | 0.123 | 0.090 | 0.04 | 0.118 | 0.102 | 0.100 | 0.088 | 0.116 | 0.220 |
| *Morganella* | 0.046 | 0.058 | 0.29 | 0.039 | 0.069 | 0.440 | 0.033 | 0.099 | 0.440 |
| *Mucilaginibacter* | 0.050 | 0.066 | 0.29 | 0.012 | 0.063 | 0.500 | 0.116 | 0.117 | 0.150 |
| *Mucinivorans* | 0.000 | 0.046 | 1 | 0.000 | 0.060 | 1.000 | 0.000 | 0.079 | 1.000 |
| *Muricauda* | 0.075 | 0.081 | 0.12 | 0.070 | 0.093 | 0.260 | 0.139 | 0.151 | 0.120 |
| *Mycobacterium* | 0.124 | 0.080 | 0.05 | 0.130 | 0.101 | 0.100 | 0.121 | 0.140 | 0.160 |
| *Mycoplasma* | 0.124 | 0.090 | 0.03 | 0.181 | 0.113 | 0.070 | 0.069 | 0.109 | 0.240 |
| *Myroides* | 0.029 | 0.047 | 0.3 | 0.048 | 0.080 | 0.350 | 0.000 | 0.066 | 1.000 |
| *Nakamurella* | 0.000 | 0.000 | 0.56 | 0.000 | 0.054 | 1.000 | 0.000 | 0.070 | 1.000 |
| *Nannizzia* | 0.152 | 0.072 | 0.02 | 0.138 | 0.097 | 0.080 | 0.188 | 0.134 | 0.070 |
| *Natrinema* | 0.082 | 0.079 | 0.13 | 0.126 | 0.100 | 0.120 | 0.000 | 0.064 | 1.000 |
| *Natronococcus* | 0.041 | 0.061 | 0.31 | 0.020 | 0.075 | 0.490 | 0.168 | 0.145 | 0.100 |
| *Natronorubrum* | 0.098 | 0.074 | 0.15 | 0.115 | 0.104 | 0.090 | 0.000 | 0.072 | 1.000 |
| *Nautilia* | 0.110 | 0.070 | 0.1 | 0.169 | 0.109 | 0.060 | 0.000 | 0.000 | 0.560 |
| *Neomicrococcus* | 0.041 | 0.052 | 0.33 | 0.000 | 0.000 | 0.840 | 0.193 | 0.148 | 0.080 |
| *Niabella* | 0.028 | 0.048 | 0.36 | 0.000 | 0.057 | 1.000 | 0.106 | 0.108 | 0.240 |
| *Nitratifractor* | 0.002 | 0.046 | 0.52 | 0.000 | 0.059 | 1.000 | 0.085 | 0.123 | 0.210 |
| *Nitratiruptor* | 0.116 | 0.086 | 0.09 | 0.085 | 0.112 | 0.190 | 0.208 | 0.159 | 0.060 |
| *Nitrosococcus* | 0.000 | 0.040 | 1 | 0.000 | 0.055 | 1.000 | 0.000 | 0.098 | 1.000 |
| *Nitrosospira* | 0.130 | 0.085 | 0.03 | 0.150 | 0.123 | 0.070 | 0.000 | 0.000 | 0.620 |
| *Nitrospira* | 0.000 | 0.041 | 1 | 0.000 | 0.064 | 1.000 | 0.000 | 0.074 | 1.000 |
| *Nocardia* | 0.039 | 0.055 | 0.25 | 0.026 | 0.064 | 0.380 | 0.069 | 0.120 | 0.310 |
| *Nocardioides* | 0.038 | 0.059 | 0.33 | 0.055 | 0.098 | 0.360 | 0.040 | 0.095 | 0.370 |
| *Nocardiopsis* | 0.000 | 0.049 | 1 | 0.094 | 0.091 | 0.100 | 0.000 | 0.061 | 1.000 |
| *Nonlabens* | 0.111 | 0.081 | 0.13 | 0.107 | 0.099 | 0.090 | 0.135 | 0.129 | 0.140 |
| *Oblitimonas* | 0.047 | 0.060 | 0.25 | 0.064 | 0.086 | 0.220 | 0.009 | 0.084 | 0.570 |
| *Oceanithermus* | 0.017 | 0.053 | 0.47 | 0.023 | 0.056 | 0.480 | 0.000 | 0.075 | 1.000 |
| *Oceanobacillus* | 0.054 | 0.061 | 0.18 | 0.123 | 0.109 | 0.080 | 0.000 | 0.083 | 1.000 |
| *Ochrobactrum* | 0.002 | 0.041 | 0.5 | 0.024 | 0.075 | 0.420 | 0.000 | 0.080 | 1.000 |
| *Odoribacter* | 0.133 | 0.074 | 0.07 | 0.084 | 0.099 | 0.210 | 0.215 | 0.149 | 0.060 |
| *Oligotropha* | 0.000 | 0.042 | 1 | 0.000 | 0.055 | 1.000 | 0.000 | 0.072 | 1.000 |
| *Opitutus* | 0.088 | 0.080 | 0.11 | 0.142 | 0.104 | 0.050 | 0.000 | 0.079 | 1.000 |

| | | | | | | | | | |
|---|---|---|---|---|---|---|---|---|---|
| *Orientia* | 0.051 | 0.068 | 0.27 | 0.080 | 0.089 | 0.180 | 0.000 | 0.070 | 1.000 |
| *Ornithobacterium* | 0.000 | 0.044 | 1 | 0.000 | 0.056 | 1.000 | 0.000 | 0.079 | 1.000 |
| *Oscillatoria* | 0.000 | 0.043 | 1 | 0.000 | 0.044 | 1.000 | 0.000 | 0.093 | 1.000 |
| *Paludibacter* | 0.006 | 0.054 | 0.44 | 0.000 | 0.067 | 1.000 | 0.035 | 0.099 | 0.440 |
| *Paludisphaera* | 0.000 | 0.048 | 1 | 0.000 | 0.051 | 1.000 | 0.000 | 0.076 | 1.000 |
| *Parabacteroides* | 0.003 | 0.050 | 0.47 | 0.048 | 0.078 | 0.240 | 0.000 | 0.075 | 1.000 |
| *Parachlamydia* | 0.066 | 0.070 | 0.17 | 0.062 | 0.082 | 0.320 | 0.076 | 0.105 | 0.310 |
| *Parageobacillus* | 0.007 | 0.058 | 0.54 | 0.044 | 0.079 | 0.240 | 0.000 | 0.072 | 1.000 |
| *Paraglaciecola* | 0.043 | 0.060 | 0.28 | 0.021 | 0.066 | 0.450 | 0.100 | 0.117 | 0.200 |
| *Paraphaeosphaeria* | 0.070 | 0.071 | 0.19 | 0.109 | 0.096 | 0.080 | 0.019 | 0.083 | 0.360 |
| *Pararhodospirillum* | 0.023 | 0.059 | 0.38 | 0.015 | 0.068 | 0.380 | 0.031 | 0.089 | 0.410 |
| *Parastagonospora* | 0.022 | 0.052 | 0.35 | 0.058 | 0.098 | 0.310 | 0.000 | 0.074 | 1.000 |
| *Parvibaculum* | 0.136 | 0.090 | 0.05 | 0.148 | 0.107 | 0.080 | 0.062 | 0.099 | 0.260 |
| *Parvimonas* | 0.041 | 0.053 | 0.31 | 0.061 | 0.094 | 0.360 | 0.017 | 0.076 | 0.400 |
| *Parvularcula* | 0.000 | 0.051 | 1 | 0.000 | 0.057 | 1.000 | 0.114 | 0.129 | 0.140 |
| *Pediococcus* | 0.000 | 0.042 | 1 | 0.000 | 0.046 | 1.000 | 0.000 | 0.072 | 1.000 |
| *Pedobacter* | 0.000 | 0.049 | 1 | 0.000 | 0.054 | 1.000 | 0.052 | 0.103 | 0.350 |
| *Pelagibacterium* | 0.133 | 0.081 | 0.07 | 0.160 | 0.109 | 0.070 | 0.089 | 0.116 | 0.270 |
| *Pelosinus* | 0.134 | 0.079 | 0.04 | 0.164 | 0.111 | 0.050 | 0.047 | 0.096 | 0.380 |
| *Peptoclostridium* | 0.022 | 0.053 | 0.41 | 0.011 | 0.061 | 0.510 | 0.091 | 0.103 | 0.250 |
| *Peptoniphilus* | 0.017 | 0.055 | 0.4 | 0.040 | 0.077 | 0.320 | 0.000 | 0.066 | 1.000 |
| *Peptostreptococcus* | 0.096 | 0.076 | 0.1 | 0.143 | 0.113 | 0.080 | 0.000 | 0.070 | 1.000 |
| *Persicobacter* | 0.015 | 0.052 | 0.45 | 0.000 | 0.064 | 1.000 | 0.150 | 0.125 | 0.110 |
| *Pestalotiopsis* | 0.149 | 0.085 | 0.05 | 0.172 | 0.109 | 0.070 | 0.105 | 0.111 | 0.250 |
| *Petrimonas* | 0.174 | 0.084 | 0.01 | 0.185 | 0.129 | 0.080 | 0.136 | 0.149 | 0.120 |
| *Phaeobacter* | 0.146 | 0.088 | 0.02 | 0.139 | 0.109 | 0.060 | 0.132 | 0.107 | 0.200 |
| *Phenylobacterium* | 0.001 | 0.042 | 0.55 | 0.000 | 0.050 | 1.000 | 0.008 | 0.090 | 0.650 |
| *Photobacterium* | 0.011 | 0.051 | 0.4 | 0.000 | 0.051 | 1.000 | 0.106 | 0.126 | 0.150 |
| *Photorhabdus* | 0.024 | 0.054 | 0.4 | 0.031 | 0.057 | 0.370 | 0.000 | 0.074 | 1.000 |
| *Phycisphaera* | 0.140 | 0.086 | 0.04 | 0.150 | 0.110 | 0.070 | 0.086 | 0.105 | 0.220 |
| *Pimelobacter* | 0.042 | 0.063 | 0.34 | 0.029 | 0.059 | 0.390 | 0.161 | 0.155 | 0.120 |
| *Pirellula* | 0.008 | 0.047 | 0.45 | 0.000 | 0.055 | 1.000 | 0.052 | 0.090 | 0.290 |
| *Piscirickettsia* | 0.109 | 0.073 | 0.11 | 0.057 | 0.085 | 0.220 | 0.186 | 0.151 | 0.060 |
| *Planctomyces* | 0.071 | 0.065 | 0.12 | 0.121 | 0.105 | 0.080 | 0.000 | 0.075 | 1.000 |
| *Planctopirus* | 0.007 | 0.049 | 0.46 | 0.000 | 0.065 | 1.000 | 0.054 | 0.106 | 0.320 |
| *Plesiomonas* | 0.000 | 0.040 | 1 | 0.000 | 0.058 | 1.000 | 0.017 | 0.102 | 0.450 |
| *Polaribacter* | 0.000 | 0.041 | 1 | 0.000 | 0.052 | 1.000 | 0.027 | 0.099 | 0.400 |
| *Polaromonas* | 0.051 | 0.061 | 0.27 | 0.031 | 0.074 | 0.330 | 0.148 | 0.135 | 0.090 |
| *Polynucleobacter* | 0.062 | 0.073 | 0.19 | 0.104 | 0.106 | 0.100 | 0.000 | 0.089 | 1.000 |
| *Pontibacter* | 0.000 | 0.042 | 1 | 0.000 | 0.060 | 1.000 | 0.000 | 0.069 | 1.000 |
| *Porphyromonas* | 0.115 | 0.084 | 0.13 | 0.126 | 0.101 | 0.080 | 0.108 | 0.125 | 0.180 |
| *Pragia* | 0.132 | 0.093 | 0.08 | 0.124 | 0.099 | 0.140 | 0.140 | 0.143 | 0.100 |
| *Prevotella* | 0.065 | 0.061 | 0.22 | 0.000 | 0.000 | 0.960 | 0.168 | 0.128 | 0.130 |
| *Prochlorococcus* | 0.093 | 0.070 | 0.11 | 0.126 | 0.110 | 0.110 | 0.050 | 0.100 | 0.420 |
| *Propionimicrobium* | 0.045 | 0.065 | 0.18 | 0.043 | 0.078 | 0.420 | 0.028 | 0.089 | 0.410 |
| *Prosthecochloris* | 0.044 | 0.055 | 0.23 | 0.042 | 0.077 | 0.410 | 0.056 | 0.118 | 0.300 |
| *Proteiniphilum* | 0.000 | 0.046 | 1 | 0.000 | 0.056 | 1.000 | 0.000 | 0.065 | 1.000 |
| *Proteus* | 0.006 | 0.045 | 0.56 | 0.116 | 0.091 | 0.090 | 0.000 | 0.088 | 1.000 |
| *Providencia* | 0.000 | 0.000 | 0.28 | 0.111 | 0.107 | 0.100 | 0.000 | 0.078 | 1.000 |
| *Pseudarthrobacter* | 0.012 | 0.052 | 0.42 | 0.021 | 0.076 | 0.460 | 0.002 | 0.088 | 0.600 |
| *Pseudoalteromonas* | 0.068 | 0.068 | 0.21 | 0.060 | 0.082 | 0.350 | 0.066 | 0.101 | 0.380 |
| *Pseudobutyrivibrio* | 0.047 | 0.062 | 0.3 | 0.032 | 0.072 | 0.450 | 0.070 | 0.124 | 0.200 |
| *Pseudocercospora* | 0.038 | 0.058 | 0.35 | 0.039 | 0.083 | 0.400 | 0.040 | 0.104 | 0.460 |

| | | | | | | | | | |
|---|---|---|---|---|---|---|---|---|---|
| *Pseudohongiella* | 0.020 | 0.054 | 0.36 | 0.027 | 0.068 | 0.420 | 0.026 | 0.086 | 0.380 |
| *Pseudonocardia* | 0.117 | 0.086 | 0.06 | 0.163 | 0.110 | 0.060 | 0.030 | 0.111 | 0.500 |
| *Pseudopedobacter* | 0.065 | 0.068 | 0.17 | 0.063 | 0.082 | 0.300 | 0.020 | 0.099 | 0.550 |
| *Pseudopropionibacterium* | 0.155 | 0.096 | 0.01 | 0.171 | 0.118 | 0.070 | 0.000 | 0.061 | 1.000 |
| *Pseudovibrio* | 0.025 | 0.047 | 0.37 | 0.042 | 0.082 | 0.340 | 0.000 | 0.075 | 1.000 |
| *Psychrobacter* | 0.000 | 0.036 | 1 | 0.016 | 0.063 | 0.470 | 0.000 | 0.086 | 1.000 |
| *Psychroflexus* | 0.172 | 0.096 | 0.01 | 0.184 | 0.107 | 0.060 | 0.000 | 0.073 | 1.000 |
| *Punctularia* | 0.024 | 0.059 | 0.41 | 0.055 | 0.088 | 0.300 | 0.000 | 0.075 | 1.000 |
| *Purpureocillium* | 0.000 | 0.050 | 1 | 0.000 | 0.064 | 1.000 | 0.000 | 0.000 | 0.680 |
| *Pyrenophora* | 0.054 | 0.064 | 0.22 | 0.103 | 0.101 | 0.100 | 0.000 | 0.058 | 1.000 |
| *Pyrococcus* | 0.020 | 0.050 | 0.5 | 0.045 | 0.072 | 0.300 | 0.000 | 0.092 | 1.000 |
| *Ramlibacter* | 0.089 | 0.068 | 0.11 | 0.110 | 0.102 | 0.130 | 0.000 | 0.085 | 1.000 |
| *Raoultella* | 0.163 | 0.101 | 0.04 | 0.189 | 0.124 | 0.060 | 0.008 | 0.081 | 0.420 |
| *Renibacterium* | 0.078 | 0.072 | 0.12 | 0.120 | 0.091 | 0.120 | 0.000 | 0.062 | 1.000 |
| *Rhodobacter* | 0.171 | 0.081 | 0.03 | 0.193 | 0.118 | 0.080 | 0.012 | 0.081 | 0.490 |
| *Rhodococcus* | 0.129 | 0.087 | 0.05 | 0.118 | 0.095 | 0.130 | 0.190 | 0.152 | 0.070 |
| *Rhodoluna* | 0.042 | 0.060 | 0.31 | 0.000 | 0.061 | 1.000 | 0.195 | 0.140 | 0.070 |
| *Rhodomicrobium* | 0.146 | 0.092 | 0.02 | 0.173 | 0.117 | 0.060 | 0.000 | 0.075 | 1.000 |
| *Rhodopirellula* | 0.000 | 0.039 | 1 | 0.000 | 0.053 | 1.000 | 0.000 | 0.000 | 1.000 |
| *Rhodospirillum* | 0.125 | 0.079 | 0.08 | 0.127 | 0.107 | 0.160 | 0.105 | 0.122 | 0.160 |
| *Rhodothermus* | 0.106 | 0.081 | 0.08 | 0.125 | 0.101 | 0.060 | 0.059 | 0.108 | 0.390 |
| *Rhodotorula* | 0.114 | 0.073 | 0.06 | 0.134 | 0.116 | 0.100 | 0.002 | 0.089 | 0.540 |
| *Rickettsia* | 0.000 | 0.048 | 1 | 0.000 | 0.047 | 1.000 | 0.000 | 0.078 | 1.000 |
| *Riemerella* | 0.000 | 0.036 | 1 | 0.000 | 0.051 | 1.000 | 0.000 | 0.085 | 1.000 |
| *Robiginitalea* | 0.015 | 0.054 | 0.51 | 0.000 | 0.062 | 1.000 | 0.105 | 0.136 | 0.240 |
| *Roseateles* | 0.149 | 0.087 | 0.03 | 0.133 | 0.109 | 0.080 | 0.204 | 0.134 | 0.060 |
| *Roseibacterium* | 0.000 | 0.041 | 1 | 0.008 | 0.064 | 0.440 | 0.000 | 0.078 | 1.000 |
| *Roseiflexus* | 0.075 | 0.068 | 0.12 | 0.144 | 0.111 | 0.090 | 0.000 | 0.076 | 1.000 |
| *Roseobacter* | 0.000 | 0.042 | 1 | 0.000 | 0.055 | 1.000 | 0.014 | 0.086 | 0.370 |
| *Roseomonas* | 0.052 | 0.067 | 0.18 | 0.050 | 0.071 | 0.310 | 0.044 | 0.107 | 0.310 |
| *Rubinisphaera* | 0.000 | 0.042 | 1 | 0.000 | 0.052 | 1.000 | 0.000 | 0.076 | 1.000 |
| *Rubrivivax* | 0.082 | 0.079 | 0.11 | 0.111 | 0.095 | 0.160 | 0.000 | 0.074 | 1.000 |
| *Rufibacter* | 0.000 | 0.040 | 1 | 0.000 | 0.065 | 1.000 | 0.000 | 0.069 | 1.000 |
| *Ruminobacter* | 0.014 | 0.044 | 0.57 | 0.000 | 0.059 | 1.000 | 0.151 | 0.132 | 0.140 |
| *Ruminococcus* | 0.000 | 0.039 | 1 | 0.000 | 0.052 | 1.000 | 0.000 | 0.087 | 1.000 |
| *Rummeliibacillus* | 0.102 | 0.079 | 0.13 | 0.164 | 0.109 | 0.090 | 0.000 | 0.075 | 1.000 |
| *Runella* | 0.000 | 0.040 | 1 | 0.000 | 0.061 | 1.000 | 0.000 | 0.069 | 1.000 |
| *Saccharomonospora* | 0.000 | 0.046 | 1 | 0.000 | 0.051 | 1.000 | 0.000 | 0.061 | 1.000 |
| *Saccharopolyspora* | 0.041 | 0.061 | 0.28 | 0.011 | 0.073 | 0.420 | 0.087 | 0.113 | 0.210 |
| *Saccharothrix* | 0.000 | 0.045 | 1 | 0.017 | 0.061 | 0.460 | 0.000 | 0.073 | 1.000 |
| *Salinibacter* | 0.000 | 0.047 | 1 | 0.000 | 0.049 | 1.000 | 0.032 | 0.094 | 0.370 |
| *Salmonella* | 0.062 | 0.067 | 0.21 | 0.030 | 0.082 | 0.400 | 0.192 | 0.160 | 0.080 |
| *Saprolegnia* | 0.050 | 0.075 | 0.22 | 0.038 | 0.077 | 0.390 | 0.057 | 0.096 | 0.270 |
| *Saprospira* | 0.000 | 0.036 | 1 | 0.000 | 0.000 | 1.000 | 0.000 | 0.069 | 1.000 |
| *Scardovia* | 0.045 | 0.055 | 0.27 | 0.000 | 0.057 | 1.000 | 0.124 | 0.128 | 0.200 |
| *Schizophyllum* | 0.060 | 0.069 | 0.2 | 0.026 | 0.061 | 0.420 | 0.208 | 0.156 | 0.060 |
| *Sebaldella* | 0.128 | 0.096 | 0.07 | 0.162 | 0.105 | 0.100 | 0.077 | 0.100 | 0.250 |
| *Sediminispirochaeta* | 0.000 | 0.044 | 1 | 0.000 | 0.052 | 1.000 | 0.069 | 0.105 | 0.370 |
| *Segniliparus* | 0.000 | 0.042 | 1 | 0.000 | 0.054 | 1.000 | 0.000 | 0.075 | 1.000 |
| *Seonamhaeicola* | 0.096 | 0.072 | 0.09 | 0.134 | 0.107 | 0.110 | 0.000 | 0.073 | 1.000 |
| *Serinicoccus* | 0.071 | 0.062 | 0.12 | 0.091 | 0.092 | 0.130 | 0.015 | 0.082 | 0.490 |
| *Serratia* | 0.096 | 0.082 | 0.06 | 0.164 | 0.103 | 0.070 | 0.000 | 0.064 | 1.000 |
| *Shewanella* | 0.096 | 0.079 | 0.06 | 0.123 | 0.097 | 0.110 | 0.050 | 0.104 | 0.320 |

| | | | | | | | | | |
|---|---|---|---|---|---|---|---|---|---|
| *Shimwellia* | 0.167 | 0.089 | 0.02 | 0.183 | 0.122 | 0.050 | 0.095 | 0.132 | 0.200 |
| *Siansivirga* | 0.000 | 0.050 | 1 | 0.013 | 0.066 | 0.410 | 0.000 | 0.084 | 1.000 |
| *Simiduia* | 0.000 | 0.045 | 1 | 0.000 | 0.047 | 1.000 | 0.069 | 0.099 | 0.290 |
| *Singulisphaera* | 0.028 | 0.056 | 0.4 | 0.053 | 0.070 | 0.250 | 0.000 | 0.072 | 1.000 |
| *Sinomonas* | 0.132 | 0.081 | 0.04 | 0.136 | 0.101 | 0.100 | 0.076 | 0.108 | 0.260 |
| *Slackia* | 0.056 | 0.072 | 0.2 | 0.061 | 0.083 | 0.260 | 0.055 | 0.099 | 0.300 |
| *Sneathia* | 0.008 | 0.047 | 0.52 | 0.000 | 0.056 | 1.000 | 0.008 | 0.081 | 0.550 |
| *Sodalis* | 0.135 | 0.084 | 0.08 | 0.138 | 0.096 | 0.070 | 0.041 | 0.077 | 0.430 |
| *Solibacillus* | 0.000 | 0.042 | 1 | 0.000 | 0.051 | 1.000 | 0.000 | 0.078 | 1.000 |
| *Solitalea* | 0.000 | 0.036 | 1 | 0.000 | 0.047 | 1.000 | 0.000 | 0.071 | 1.000 |
| *Sordaria* | 0.066 | 0.077 | 0.18 | 0.093 | 0.101 | 0.150 | 0.037 | 0.093 | 0.490 |
| *Sphaerobacter* | 0.014 | 0.057 | 0.42 | 0.042 | 0.075 | 0.260 | 0.000 | 0.000 | 0.870 |
| *Sphingobacterium* | 0.000 | 0.035 | 1 | 0.000 | 0.060 | 1.000 | 0.030 | 0.080 | 0.400 |
| *Sphingorhabdus* | 0.089 | 0.078 | 0.11 | 0.171 | 0.123 | 0.060 | 0.000 | 0.056 | 1.000 |
| *Spiribacter* | 0.168 | 0.094 | 0.02 | 0.152 | 0.108 | 0.050 | 0.180 | 0.137 | 0.050 |
| *Spiroplasma* | 0.075 | 0.088 | 0.14 | 0.020 | 0.071 | 0.400 | 0.104 | 0.114 | 0.150 |
| *Spirosoma* | 0.000 | 0.039 | 1 | 0.000 | 0.062 | 1.000 | 0.000 | 0.076 | 1.000 |
| *Spongiibacter* | 0.062 | 0.067 | 0.23 | 0.094 | 0.098 | 0.160 | 0.000 | 0.073 | 1.000 |
| *Sporosarcina* | 0.016 | 0.055 | 0.44 | 0.056 | 0.080 | 0.230 | 0.000 | 0.076 | 1.000 |
| *Staphylococcus* | 0.013 | 0.045 | 0.47 | 0.096 | 0.087 | 0.140 | 0.000 | 0.067 | 1.000 |
| *Steroidobacter* | 0.000 | 0.037 | 1 | 0.000 | 0.054 | 1.000 | 0.052 | 0.105 | 0.310 |
| *Streptobacillus* | 0.059 | 0.067 | 0.18 | 0.045 | 0.087 | 0.350 | 0.085 | 0.116 | 0.300 |
| *Streptococcus* | 0.089 | 0.078 | 0.08 | 0.039 | 0.067 | 0.370 | 0.145 | 0.127 | 0.110 |
| *Streptomyces* | 0.000 | 0.034 | 1 | 0.030 | 0.063 | 0.320 | 0.000 | 0.081 | 1.000 |
| *Streptosporangium* | 0.080 | 0.084 | 0.15 | 0.077 | 0.101 | 0.190 | 0.088 | 0.124 | 0.300 |
| *Sulfolobus* | 0.040 | 0.060 | 0.36 | 0.000 | 0.057 | 1.000 | 0.090 | 0.104 | 0.220 |
| *Sulfuricurvum* | 0.096 | 0.074 | 0.1 | 0.096 | 0.109 | 0.220 | 0.009 | 0.095 | 0.550 |
| *Sulfurimonas* | 0.006 | 0.043 | 0.51 | 0.033 | 0.065 | 0.430 | 0.000 | 0.066 | 1.000 |
| *Sulfuritalea* | 0.042 | 0.058 | 0.31 | 0.029 | 0.063 | 0.410 | 0.101 | 0.110 | 0.130 |
| *Sulfurospirillum* | 0.024 | 0.050 | 0.4 | 0.071 | 0.084 | 0.220 | 0.000 | 0.072 | 1.000 |
| *Symbiobacterium* | 0.045 | 0.064 | 0.29 | 0.051 | 0.079 | 0.280 | 0.030 | 0.097 | 0.340 |
| *Syntrophobacter* | 0.138 | 0.091 | 0.06 | 0.175 | 0.125 | 0.090 | 0.027 | 0.095 | 0.350 |
| *Syntrophothermus* | 0.000 | 0.047 | 1 | 0.000 | 0.049 | 1.000 | 0.017 | 0.091 | 0.460 |
| *Tannerella* | 0.000 | 0.057 | 1 | 0.000 | 0.057 | 1.000 | 0.025 | 0.074 | 0.470 |
| *Tateyamaria* | 0.000 | 0.043 | 1 | 0.000 | 0.050 | 1.000 | 0.039 | 0.090 | 0.440 |
| *Tenacibaculum* | 0.002 | 0.046 | 0.53 | 0.001 | 0.071 | 0.560 | 0.000 | 0.070 | 1.000 |
| *Tepidanaerobacter* | 0.000 | 0.045 | 1 | 0.018 | 0.067 | 0.420 | 0.000 | 0.072 | 1.000 |
| *Terribacillus* | 0.134 | 0.084 | 0.03 | 0.152 | 0.108 | 0.060 | 0.078 | 0.094 | 0.280 |
| *Terriglobus* | 0.085 | 0.082 | 0.08 | 0.072 | 0.085 | 0.180 | 0.144 | 0.148 | 0.150 |
| *Terrisporobacter* | 0.021 | 0.057 | 0.41 | 0.000 | 0.058 | 1.000 | 0.075 | 0.106 | 0.270 |
| *Tetragenococcus* | 0.085 | 0.077 | 0.1 | 0.121 | 0.108 | 0.160 | 0.000 | 0.083 | 1.000 |
| *Thauera* | 0.160 | 0.088 | 0.03 | 0.179 | 0.103 | 0.060 | 0.000 | 0.078 | 1.000 |
| *Thecamonas* | 0.063 | 0.077 | 0.21 | 0.058 | 0.094 | 0.280 | 0.095 | 0.110 | 0.220 |
| *Thermacetogenium* | 0.005 | 0.046 | 0.5 | 0.055 | 0.088 | 0.290 | 0.000 | 0.086 | 1.000 |
| *Thermanaerovibrio* | 0.000 | 0.050 | 1 | 0.000 | 0.058 | 1.000 | 0.000 | 0.078 | 1.000 |
| *Thermincola* | 0.075 | 0.083 | 0.13 | 0.100 | 0.101 | 0.130 | 0.000 | 0.068 | 1.000 |
| *Thermobacillus* | 0.008 | 0.050 | 0.45 | 0.120 | 0.102 | 0.130 | 0.000 | 0.101 | 1.000 |
| *Thermobaculum* | 0.104 | 0.076 | 0.09 | 0.156 | 0.120 | 0.070 | 0.000 | 0.084 | 1.000 |
| *Thermobifida* | 0.000 | 0.042 | 1 | 0.043 | 0.088 | 0.290 | 0.000 | 0.057 | 1.000 |
| *Thermobispora* | 0.000 | 0.043 | 1 | 0.000 | 0.055 | 1.000 | 0.000 | 0.066 | 1.000 |
| *Thermococcus* | 0.145 | 0.090 | 0.02 | 0.161 | 0.114 | 0.060 | 0.009 | 0.070 | 0.530 |
| *Thermodesulfatator* | 0.074 | 0.069 | 0.13 | 0.078 | 0.082 | 0.220 | 0.026 | 0.072 | 0.430 |
| *Thermodesulfovibrio* | 0.049 | 0.063 | 0.31 | 0.068 | 0.074 | 0.270 | 0.040 | 0.094 | 0.470 |

| | | | | | | | | | |
|---|---|---|---|---|---|---|---|---|---|
| *Thermomicrobium* | 0.000 | 0.039 | 1 | 0.000 | 0.061 | 1.000 | 0.000 | 0.088 | 1.000 |
| *Thermomonospora* | 0.006 | 0.042 | 0.46 | 0.000 | 0.059 | 1.000 | 0.059 | 0.104 | 0.310 |
| *Thermosediminibacter* | 0.042 | 0.048 | 0.28 | 0.056 | 0.072 | 0.330 | 0.018 | 0.074 | 0.460 |
| *Thermosulfidibacter* | 0.070 | 0.066 | 0.2 | 0.055 | 0.083 | 0.280 | 0.171 | 0.132 | 0.110 |
| *Thermosynechococcus* | 0.092 | 0.073 | 0.11 | 0.087 | 0.090 | 0.150 | 0.072 | 0.104 | 0.330 |
| *Thermovibrio* | 0.093 | 0.068 | 0.11 | 0.088 | 0.097 | 0.200 | 0.149 | 0.128 | 0.160 |
| *Thioalkalimicrobium* | 0.000 | 0.041 | 1 | 0.000 | 0.058 | 1.000 | 0.068 | 0.121 | 0.290 |
| *Thiobacillus* | 0.160 | 0.089 | 0.01 | 0.179 | 0.120 | 0.060 | 0.000 | 0.086 | 1.000 |
| *Thiobacimonas* | 0.185 | 0.092 | 0.02 | 0.194 | 0.107 | 0.050 | 0.123 | 0.119 | 0.220 |
| *Thiocystis* | 0.112 | 0.094 | 0.07 | 0.131 | 0.100 | 0.060 | 0.000 | 0.071 | 1.000 |
| *Tilletiaria* | 0.047 | 0.063 | 0.31 | 0.079 | 0.090 | 0.210 | 0.000 | 0.067 | 1.000 |
| *Tistrella* | 0.064 | 0.069 | 0.14 | 0.069 | 0.083 | 0.290 | 0.046 | 0.113 | 0.280 |
| *Tolumonas* | 0.111 | 0.074 | 0.04 | 0.095 | 0.100 | 0.190 | 0.131 | 0.122 | 0.220 |
| *Tremella* | 0.103 | 0.093 | 0.07 | 0.132 | 0.106 | 0.110 | 0.000 | 0.073 | 1.000 |
| *Treponema* | 0.015 | 0.042 | 0.5 | 0.005 | 0.058 | 0.430 | 0.067 | 0.121 | 0.350 |
| *Trichosporon* | 0.161 | 0.089 | 0.06 | 0.114 | 0.102 | 0.090 | 0.210 | 0.137 | 0.060 |
| *Truepera* | 0.153 | 0.087 | 0.05 | 0.174 | 0.112 | 0.060 | 0.000 | 0.067 | 1.000 |
| *Turicibacter* | 0.108 | 0.082 | 0.07 | 0.125 | 0.104 | 0.120 | 0.096 | 0.103 | 0.210 |
| *Turneriella* | 0.000 | 0.050 | 1 | 0.010 | 0.060 | 0.440 | 0.000 | 0.062 | 1.000 |
| *Uncinocarpus* | 0.126 | 0.081 | 0.05 | 0.122 | 0.108 | 0.070 | 0.140 | 0.142 | 0.150 |
| *Ureaplasma* | 0.052 | 0.071 | 0.25 | 0.053 | 0.079 | 0.330 | 0.059 | 0.083 | 0.320 |
| *Ustilago* | 0.174 | 0.085 | 0.02 | 0.187 | 0.122 | 0.050 | 0.142 | 0.125 | 0.090 |
| *Vagococcus* | 0.002 | 0.044 | 0.41 | 0.000 | 0.061 | 1.000 | 0.045 | 0.088 | 0.380 |
| *Verminephrobacter* | 0.001 | 0.048 | 0.58 | 0.042 | 0.079 | 0.310 | 0.000 | 0.091 | 1.000 |
| *Verrucomicrobium* | 0.000 | 0.045 | 1 | 0.000 | 0.055 | 1.000 | 0.000 | 0.086 | 1.000 |
| *Verrucosispora* | 0.015 | 0.053 | 0.38 | 0.050 | 0.075 | 0.300 | 0.000 | 0.075 | 1.000 |
| *Verticillium* | 0.000 | 0.043 | 1 | 0.000 | 0.059 | 1.000 | 0.100 | 0.118 | 0.230 |
| *Virgibacillus* | 0.000 | 0.039 | 1 | 0.059 | 0.088 | 0.320 | 0.000 | 0.062 | 1.000 |
| *Vulgatibacter* | 0.058 | 0.070 | 0.2 | 0.059 | 0.078 | 0.280 | 0.050 | 0.097 | 0.460 |
| *Weissella* | 0.000 | 0.047 | 1 | 0.043 | 0.079 | 0.320 | 0.000 | 0.077 | 1.000 |
| *Wigglesworthia* | 0.106 | 0.088 | 0.06 | 0.112 | 0.094 | 0.120 | 0.088 | 0.103 | 0.270 |
| *Winogradskyella* | 0.122 | 0.079 | 0.09 | 0.131 | 0.109 | 0.060 | 0.075 | 0.102 | 0.300 |
| *Woeseia* | 0.068 | 0.075 | 0.16 | 0.035 | 0.073 | 0.370 | 0.178 | 0.144 | 0.090 |
| *Wolbachia* | 0.097 | 0.088 | 0.12 | 0.146 | 0.119 | 0.080 | 0.000 | 0.085 | 0.560 |
| *Xanthobacter* | 0.124 | 0.084 | 0.11 | 0.160 | 0.118 | 0.070 | 0.000 | 0.090 | 1.000 |
| *Xenorhabdus* | 0.113 | 0.080 | 0.04 | 0.140 | 0.113 | 0.080 | 0.042 | 0.102 | 0.380 |
| *Xylanimonas* | 0.000 | 0.041 | 1 | 0.000 | 0.051 | 1.000 | 0.000 | 0.091 | 1.000 |
| *Xylella* | 0.078 | 0.077 | 0.08 | 0.066 | 0.093 | 0.240 | 0.120 | 0.136 | 0.110 |
| *Yersinia* | 0.168 | 0.091 | 0.03 | 0.174 | 0.104 | 0.070 | 0.117 | 0.133 | 0.100 |
| *Zobellia* | 0.166 | 0.107 | 0.02 | 0.156 | 0.125 | 0.060 | 0.190 | 0.154 | 0.110 |
| *Zunongwangia* | 0.000 | 0.045 | 1 | 0.000 | 0.051 | 1.000 | 0.000 | 0.092 | 1.000 |
| *Zymomonas* | 0.000 | 0.048 | 1 | 0.000 | 0.000 | 0.590 | 0.000 | 0.060 | 1.000 |
| *Zymoseptoria* | 0.021 | 0.052 | 0.37 | 0.013 | 0.059 | 0.470 | 0.124 | 0.117 | 0.200 |
| $K00057_{BOTH}$ | 0.173 | 0.091 | 0.03 | 0.180 | 0.124 | 0.040 | 0.196 | 0.143 | 0.030 |
| $K00441_{BOTH}$ | 0.283 | 0.103 | 0.01 | 0.276 | 0.136 | 0.010 | 0.297 | 0.163 | 0.030 |
| $K00820_{BOTH}$ | 0.199 | 0.098 | 0.02 | 0.208 | 0.112 | 0.020 | 0.248 | 0.148 | 0.040 |
| $K00826_{BOTH}$ | 0.199 | 0.093 | 0.01 | 0.169 | 0.108 | 0.020 | 0.263 | 0.152 | 0.040 |
| $K00856_{BOTH}$ | 0.426 | 0.106 | 0.01 | 0.421 | 0.143 | 0.010 | 0.425 | 0.175 | 0.010 |
| $K01156_{BOTH}$ | 0.317 | 0.100 | 0.01 | 0.272 | 0.129 | 0.010 | 0.412 | 0.192 | 0.010 |
| $K01183_{BOTH}$ | 0.260 | 0.113 | 0.01 | 0.236 | 0.130 | 0.030 | 0.281 | 0.150 | 0.020 |
| $K01465_{BOTH}$ | 0.261 | 0.104 | 0.01 | 0.236 | 0.121 | 0.010 | 0.287 | 0.157 | 0.020 |
| $K01657_{BOTH}$ | 0.242 | 0.098 | 0.01 | 0.178 | 0.114 | 0.040 | 0.279 | 0.156 | 0.010 |

| | | | | | | | | | |
|---|---|---|---|---|---|---|---|---|---|
| $K01703_{BOTH}$ | 0.339 | 0.103 | 0.01 | 0.258 | 0.144 | 0.010 | 0.462 | 0.188 | 0.010 |
| $K01999_{BOTH}$ | 0.227 | 0.106 | 0.01 | 0.255 | 0.121 | 0.010 | 0.209 | 0.159 | 0.040 |
| $K02025_{BOTH}$ | 0.260 | 0.110 | 0.01 | 0.308 | 0.130 | 0.010 | 0.177 | 0.138 | 0.030 |
| $K02355_{BOTH}$ | 0.187 | 0.096 | 0.01 | 0.212 | 0.122 | 0.010 | 0.223 | 0.144 | 0.030 |
| $K02837_{BOTH}$ | 0.274 | 0.109 | 0.01 | 0.245 | 0.132 | 0.020 | 0.333 | 0.177 | 0.030 |
| $K02866_{BOTH}$ | 0.313 | 0.091 | 0.01 | 0.357 | 0.136 | 0.010 | 0.249 | 0.156 | 0.030 |
| $K02875_{BOTH}$ | 0.385 | 0.097 | 0.01 | 0.401 | 0.132 | 0.010 | 0.352 | 0.166 | 0.010 |
| $K02889_{BOTH}$ | 0.368 | 0.101 | 0.01 | 0.406 | 0.129 | 0.010 | 0.305 | 0.159 | 0.010 |
| $K02908_{BOTH}$ | 0.391 | 0.110 | 0.01 | 0.415 | 0.141 | 0.010 | 0.352 | 0.177 | 0.010 |
| $K02912_{BOTH}$ | 0.388 | 0.102 | 0.01 | 0.404 | 0.137 | 0.010 | 0.361 | 0.173 | 0.010 |
| $K02921_{BOTH}$ | 0.388 | 0.114 | 0.01 | 0.399 | 0.145 | 0.010 | 0.351 | 0.185 | 0.020 |
| $K02936_{BOTH}$ | 0.365 | 0.103 | 0.01 | 0.382 | 0.135 | 0.010 | 0.328 | 0.159 | 0.010 |
| $K02966_{BOTH}$ | 0.354 | 0.099 | 0.01 | 0.380 | 0.130 | 0.010 | 0.312 | 0.160 | 0.010 |
| $K02979_{BOTH}$ | 0.315 | 0.108 | 0.01 | 0.323 | 0.132 | 0.010 | 0.292 | 0.149 | 0.010 |
| $K02987_{BOTH}$ | 0.407 | 0.105 | 0.01 | 0.434 | 0.134 | 0.010 | 0.356 | 0.174 | 0.010 |
| $K02991_{BOTH}$ | 0.349 | 0.103 | 0.01 | 0.379 | 0.127 | 0.010 | 0.302 | 0.161 | 0.030 |
| $K02995_{BOTH}$ | 0.395 | 0.108 | 0.01 | 0.422 | 0.158 | 0.010 | 0.320 | 0.176 | 0.030 |
| $K03105_{BOTH}$ | 0.248 | 0.105 | 0.01 | 0.242 | 0.130 | 0.030 | 0.283 | 0.163 | 0.020 |
| $K03120_{BOTH}$ | 0.351 | 0.101 | 0.01 | 0.395 | 0.134 | 0.010 | 0.286 | 0.161 | 0.020 |
| $K03215_{BOTH}$ | 0.159 | 0.097 | 0.02 | 0.175 | 0.117 | 0.040 | 0.181 | 0.147 | 0.030 |
| $K03236_{BOTH}$ | 0.329 | 0.104 | 0.01 | 0.351 | 0.130 | 0.010 | 0.282 | 0.153 | 0.010 |
| $K03263_{BOTH}$ | 0.362 | 0.098 | 0.01 | 0.403 | 0.147 | 0.010 | 0.286 | 0.166 | 0.030 |
| $K03555_{BOTH}$ | 0.197 | 0.091 | 0.01 | 0.256 | 0.135 | 0.010 | 0.231 | 0.162 | 0.040 |
| $K03626_{BOTH}$ | 0.347 | 0.104 | 0.01 | 0.379 | 0.141 | 0.010 | 0.281 | 0.151 | 0.030 |
| $K03737_{BOTH}$ | 0.332 | 0.106 | 0.01 | 0.362 | 0.145 | 0.010 | 0.308 | 0.168 | 0.020 |
| $K03778_{BOTH}$ | 0.251 | 0.090 | 0.01 | 0.284 | 0.132 | 0.010 | 0.243 | 0.165 | 0.020 |
| $K04043_{BOTH}$ | 0.258 | 0.103 | 0.01 | 0.205 | 0.120 | 0.040 | 0.350 | 0.167 | 0.010 |
| $K04564_{BOTH}$ | 0.288 | 0.100 | 0.01 | 0.277 | 0.135 | 0.010 | 0.270 | 0.149 | 0.030 |
| $K04798_{BOTH}$ | 0.285 | 0.097 | 0.01 | 0.296 | 0.124 | 0.020 | 0.243 | 0.155 | 0.040 |
| $K06204_{BOTH}$ | 0.235 | 0.103 | 0.01 | 0.245 | 0.118 | 0.010 | 0.255 | 0.153 | 0.040 |
| $K06867_{BOTH}$ | 0.243 | 0.097 | 0.01 | 0.217 | 0.122 | 0.030 | 0.277 | 0.134 | 0.020 |
| $K06874_{BOTH}$ | 0.217 | 0.090 | 0.01 | 0.207 | 0.136 | 0.040 | 0.212 | 0.158 | 0.010 |
| $K07316_{BOTH}$ | 0.292 | 0.111 | 0.01 | 0.213 | 0.129 | 0.030 | 0.403 | 0.175 | 0.010 |
| $K07464_{BOTH}$ | 0.256 | 0.090 | 0.01 | 0.236 | 0.123 | 0.020 | 0.292 | 0.173 | 0.030 |
| $K07720_{BOTH}$ | 0.390 | 0.106 | 0.01 | 0.422 | 0.137 | 0.010 | 0.314 | 0.163 | 0.030 |
| $K08094_{BOTH}$ | 0.228 | 0.107 | 0.01 | 0.254 | 0.128 | 0.010 | 0.211 | 0.143 | 0.040 |
| $K10117_{BOTH}$ | 0.232 | 0.105 | 0.01 | 0.216 | 0.125 | 0.010 | 0.239 | 0.171 | 0.020 |
| $K10218_{BOTH}$ | 0.283 | 0.115 | 0.01 | 0.190 | 0.112 | 0.030 | 0.371 | 0.158 | 0.010 |
| $K14475_{BOTH}$ | 0.252 | 0.092 | 0.01 | 0.206 | 0.137 | 0.030 | 0.292 | 0.170 | 0.020 |
| $K19755_{BOTH}$ | 0.294 | 0.107 | 0.01 | 0.276 | 0.123 | 0.020 | 0.323 | 0.169 | 0.020 |
| $K20742_{BOTH}$ | 0.252 | 0.107 | 0.01 | 0.150 | 0.113 | 0.030 | 0.430 | 0.179 | 0.010 |
| $K00020_{CON}$ | 0.199 | 0.090 | 0.02 | 0.306 | 0.151 | 0.020 | 0.084 | 0.115 | 0.330 |
| $K00048_{CON}$ | 0.134 | 0.091 | 0.08 | 0.244 | 0.137 | 0.030 | 0.000 | 0.071 | 1.000 |
| $K00134_{CON}$ | 0.137 | 0.087 | 0.06 | 0.207 | 0.114 | 0.020 | 0.094 | 0.121 | 0.220 |
| $K00266_{CON}$ | 0.218 | 0.084 | 0.01 | 0.289 | 0.141 | 0.020 | 0.117 | 0.132 | 0.170 |
| $K00286_{CON}$ | 0.208 | 0.108 | 0.01 | 0.264 | 0.128 | 0.020 | 0.103 | 0.125 | 0.170 |

| | | | | | | | | | |
|---|---|---|---|---|---|---|---|---|---|
| $K00297_{CON}$ | 0.311 | 0.101 | 0.01 | 0.359 | 0.139 | 0.010 | 0.257 | 0.168 | 0.050 |
| $K00348_{CON}$ | 0.083 | 0.081 | 0.17 | 0.169 | 0.119 | 0.020 | 0.000 | 0.085 | 1.000 |
| $K00390_{CON}$ | 0.219 | 0.109 | 0.02 | 0.230 | 0.132 | 0.010 | 0.189 | 0.143 | 0.090 |
| $K00567_{CON}$ | 0.107 | 0.077 | 0.06 | 0.203 | 0.121 | 0.040 | 0.000 | 0.084 | 1.000 |
| $K00573_{CON}$ | 0.189 | 0.096 | 0.02 | 0.187 | 0.111 | 0.020 | 0.195 | 0.149 | 0.080 |
| $K00582_{CON}$ | 0.132 | 0.086 | 0.06 | 0.193 | 0.123 | 0.030 | 0.007 | 0.072 | 0.490 |
| $K00583_{CON}$ | 0.133 | 0.093 | 0.07 | 0.186 | 0.116 | 0.040 | 0.004 | 0.087 | 0.520 |
| $K00604_{CON}$ | 0.165 | 0.092 | 0.01 | 0.297 | 0.143 | 0.010 | 0.000 | 0.086 | 1.000 |
| $K00626_{CON}$ | 0.178 | 0.092 | 0.04 | 0.269 | 0.129 | 0.010 | 0.000 | 0.068 | 1.000 |
| $K00652_{CON}$ | 0.170 | 0.094 | 0.02 | 0.306 | 0.112 | 0.020 | 0.005 | 0.063 | 0.440 |
| $K00684_{CON}$ | 0.148 | 0.090 | 0.01 | 0.191 | 0.122 | 0.040 | 0.044 | 0.082 | 0.440 |
| $K00688_{CON}$ | 0.139 | 0.080 | 0.06 | 0.281 | 0.119 | 0.010 | 0.000 | 0.065 | 1.000 |
| $K00703_{CON}$ | 0.090 | 0.078 | 0.13 | 0.171 | 0.125 | 0.030 | 0.014 | 0.083 | 0.460 |
| $K00763_{CON}$ | 0.133 | 0.079 | 0.02 | 0.199 | 0.125 | 0.040 | 0.034 | 0.077 | 0.400 |
| $K00784_{CON}$ | 0.130 | 0.088 | 0.03 | 0.257 | 0.135 | 0.010 | 0.000 | 0.070 | 1.000 |
| $K00788_{CON}$ | 0.152 | 0.095 | 0.02 | 0.307 | 0.126 | 0.020 | 0.000 | 0.086 | 1.000 |
| $K00836_{CON}$ | 0.120 | 0.088 | 0.06 | 0.172 | 0.110 | 0.010 | 0.000 | 0.060 | 1.000 |
| $K00847_{CON}$ | 0.136 | 0.085 | 0.08 | 0.189 | 0.123 | 0.040 | 0.044 | 0.085 | 0.310 |
| $K00872_{CON}$ | 0.078 | 0.067 | 0.13 | 0.141 | 0.111 | 0.040 | 0.000 | 0.086 | 1.000 |
| $K00878_{CON}$ | 0.164 | 0.085 | 0.01 | 0.308 | 0.146 | 0.010 | 0.009 | 0.075 | 0.510 |
| $K00882_{CON}$ | 0.186 | 0.093 | 0.02 | 0.238 | 0.129 | 0.030 | 0.020 | 0.089 | 0.490 |
| $K00927_{CON}$ | 0.149 | 0.088 | 0.02 | 0.283 | 0.126 | 0.020 | 0.099 | 0.130 | 0.230 |
| $K00940_{CON}$ | 0.252 | 0.084 | 0.01 | 0.309 | 0.135 | 0.010 | 0.051 | 0.104 | 0.420 |
| $K00941_{CON}$ | 0.171 | 0.088 | 0.02 | 0.256 | 0.123 | 0.020 | 0.000 | 0.058 | 1.000 |
| $K00950_{CON}$ | 0.248 | 0.112 | 0.01 | 0.275 | 0.125 | 0.010 | 0.191 | 0.142 | 0.110 |
| $K00962_{CON}$ | 0.084 | 0.064 | 0.12 | 0.175 | 0.119 | 0.040 | 0.051 | 0.104 | 0.350 |
| $K00975_{CON}$ | 0.134 | 0.081 | 0.05 | 0.197 | 0.116 | 0.020 | 0.062 | 0.099 | 0.340 |
| $K00978_{CON}$ | 0.212 | 0.104 | 0.02 | 0.249 | 0.129 | 0.030 | 0.165 | 0.133 | 0.090 |
| $K00991_{CON}$ | 0.182 | 0.080 | 0.01 | 0.243 | 0.120 | 0.020 | 0.064 | 0.104 | 0.280 |
| $K01005_{CON}$ | 0.151 | 0.097 | 0.04 | 0.235 | 0.132 | 0.010 | 0.055 | 0.103 | 0.310 |
| $K01008_{CON}$ | 0.120 | 0.081 | 0.05 | 0.269 | 0.144 | 0.010 | 0.000 | 0.000 | 0.900 |
| $K01012_{CON}$ | 0.032 | 0.059 | 0.38 | 0.205 | 0.115 | 0.020 | 0.000 | 0.071 | 1.000 |
| $K01051_{CON}$ | 0.088 | 0.070 | 0.16 | 0.191 | 0.118 | 0.020 | 0.000 | 0.076 | 1.000 |
| $K01060_{CON}$ | 0.082 | 0.068 | 0.15 | 0.201 | 0.122 | 0.010 | 0.000 | 0.069 | 1.000 |
| $K01091_{CON}$ | 0.213 | 0.099 | 0.02 | 0.219 | 0.123 | 0.020 | 0.193 | 0.137 | 0.070 |
| $K01092_{CON}$ | 0.173 | 0.088 | 0.01 | 0.205 | 0.130 | 0.030 | 0.056 | 0.108 | 0.380 |
| $K01095_{CON}$ | 0.083 | 0.077 | 0.15 | 0.133 | 0.106 | 0.040 | 0.000 | 0.075 | 1.000 |
| $K01139_{CON}$ | 0.167 | 0.097 | 0.04 | 0.219 | 0.121 | 0.030 | 0.133 | 0.124 | 0.210 |
| $K01193_{CON}$ | 0.205 | 0.088 | 0.02 | 0.247 | 0.129 | 0.010 | 0.134 | 0.133 | 0.170 |
| $K01218_{CON}$ | 0.119 | 0.083 | 0.09 | 0.206 | 0.116 | 0.040 | 0.000 | 0.080 | 1.000 |
| $K01223_{CON}$ | 0.144 | 0.090 | 0.04 | 0.336 | 0.130 | 0.010 | 0.000 | 0.071 | 1.000 |
| $K01258_{CON}$ | 0.201 | 0.102 | 0.01 | 0.277 | 0.133 | 0.010 | 0.103 | 0.116 | 0.250 |
| $K01267_{CON}$ | 0.203 | 0.097 | 0.01 | 0.366 | 0.130 | 0.010 | 0.000 | 0.079 | 1.000 |
| $K01297_{CON}$ | 0.162 | 0.102 | 0.03 | 0.182 | 0.123 | 0.040 | 0.000 | 0.070 | 1.000 |
| $K01304_{CON}$ | 0.126 | 0.082 | 0.05 | 0.195 | 0.125 | 0.040 | 0.050 | 0.097 | 0.250 |
| $K01433_{CON}$ | 0.242 | 0.109 | 0.01 | 0.306 | 0.149 | 0.010 | 0.001 | 0.084 | 0.530 |

| | | | | | | | | | |
|---|---|---|---|---|---|---|---|---|---|
| $K01443_{CON}$ | 0.026 | 0.052 | 0.41 | 0.160 | 0.130 | 0.030 | 0.000 | 0.064 | 1.000 |
| $K01470_{CON}$ | 0.126 | 0.084 | 0.04 | 0.189 | 0.116 | 0.020 | 0.000 | 0.077 | 1.000 |
| $K01496_{CON}$ | 0.089 | 0.084 | 0.09 | 0.168 | 0.118 | 0.030 | 0.000 | 0.068 | 1.000 |
| $K01500_{CON}$ | 0.210 | 0.102 | 0.01 | 0.250 | 0.130 | 0.030 | 0.113 | 0.116 | 0.240 |
| $K01515_{CON}$ | 0.072 | 0.075 | 0.13 | 0.175 | 0.123 | 0.040 | 0.000 | 0.092 | 1.000 |
| $K01591_{CON}$ | 0.117 | 0.085 | 0.06 | 0.189 | 0.121 | 0.030 | 0.037 | 0.090 | 0.450 |
| $K01610_{CON}$ | 0.220 | 0.105 | 0.01 | 0.235 | 0.122 | 0.010 | 0.212 | 0.146 | 0.050 |
| $K01613_{CON}$ | 0.136 | 0.078 | 0.02 | 0.188 | 0.127 | 0.020 | 0.063 | 0.114 | 0.260 |
| $K01620_{CON}$ | 0.103 | 0.076 | 0.07 | 0.158 | 0.121 | 0.040 | 0.000 | 0.088 | 1.000 |
| $K01624_{CON}$ | 0.118 | 0.085 | 0.09 | 0.211 | 0.127 | 0.020 | 0.051 | 0.105 | 0.350 |
| $K01666_{CON}$ | 0.090 | 0.074 | 0.08 | 0.169 | 0.105 | 0.010 | 0.000 | 0.077 | 1.000 |
| $K01678_{CON}$ | 0.135 | 0.091 | 0.03 | 0.262 | 0.130 | 0.010 | 0.000 | 0.066 | 1.000 |
| $K01728_{CON}$ | 0.129 | 0.088 | 0.07 | 0.209 | 0.129 | 0.030 | 0.000 | 0.078 | 1.000 |
| $K01734_{CON}$ | 0.179 | 0.101 | 0.01 | 0.225 | 0.140 | 0.030 | 0.138 | 0.125 | 0.150 |
| $K01738_{CON}$ | 0.172 | 0.096 | 0.04 | 0.230 | 0.115 | 0.030 | 0.100 | 0.126 | 0.270 |
| $K01752_{CON}$ | 0.179 | 0.092 | 0.01 | 0.245 | 0.127 | 0.010 | 0.057 | 0.099 | 0.400 |
| $K01759_{CON}$ | 0.155 | 0.081 | 0.02 | 0.244 | 0.125 | 0.020 | 0.069 | 0.107 | 0.370 |
| $K01771_{CON}$ | 0.158 | 0.092 | 0.03 | 0.183 | 0.125 | 0.040 | 0.089 | 0.124 | 0.270 |
| $K01815_{CON}$ | 0.175 | 0.101 | 0.02 | 0.234 | 0.121 | 0.020 | 0.015 | 0.090 | 0.430 |
| $K01823_{CON}$ | 0.217 | 0.099 | 0.01 | 0.247 | 0.106 | 0.020 | 0.079 | 0.117 | 0.260 |
| $K01835_{CON}$ | 0.118 | 0.083 | 0.11 | 0.179 | 0.131 | 0.040 | 0.000 | 0.086 | 1.000 |
| $K01897_{CON}$ | 0.165 | 0.085 | 0.02 | 0.220 | 0.124 | 0.030 | 0.145 | 0.130 | 0.090 |
| $K01902_{CON}$ | 0.105 | 0.077 | 0.1 | 0.171 | 0.116 | 0.040 | 0.000 | 0.000 | 0.540 |
| $K01921_{CON}$ | 0.129 | 0.088 | 0.08 | 0.208 | 0.138 | 0.020 | 0.000 | 0.067 | 1.000 |
| $K01926_{CON}$ | 0.077 | 0.078 | 0.12 | 0.192 | 0.129 | 0.040 | 0.000 | 0.076 | 1.000 |
| $K01938_{CON}$ | 0.143 | 0.092 | 0.03 | 0.255 | 0.123 | 0.020 | 0.000 | 0.000 | 0.640 |
| $K01955_{CON}$ | 0.239 | 0.097 | 0.01 | 0.344 | 0.138 | 0.010 | 0.180 | 0.145 | 0.110 |
| $K01989_{CON}$ | 0.266 | 0.099 | 0.01 | 0.340 | 0.119 | 0.010 | 0.133 | 0.133 | 0.200 |
| $K01997_{CON}$ | 0.201 | 0.091 | 0.01 | 0.265 | 0.135 | 0.010 | 0.116 | 0.114 | 0.180 |
| $K02007_{CON}$ | 0.130 | 0.090 | 0.06 | 0.260 | 0.133 | 0.010 | 0.000 | 0.083 | 1.000 |
| $K02026_{CON}$ | 0.261 | 0.110 | 0.01 | 0.309 | 0.126 | 0.010 | 0.175 | 0.137 | 0.100 |
| $K02027_{CON}$ | 0.245 | 0.100 | 0.01 | 0.293 | 0.139 | 0.010 | 0.182 | 0.150 | 0.080 |
| $K02029_{CON}$ | 0.175 | 0.085 | 0.02 | 0.325 | 0.130 | 0.010 | 0.000 | 0.075 | 1.000 |
| $K02031_{CON}$ | 0.147 | 0.081 | 0.03 | 0.235 | 0.134 | 0.020 | 0.022 | 0.101 | 0.490 |
| $K02032_{CON}$ | 0.228 | 0.112 | 0.01 | 0.286 | 0.138 | 0.020 | 0.126 | 0.133 | 0.140 |
| $K02033_{CON}$ | 0.136 | 0.091 | 0.05 | 0.255 | 0.122 | 0.010 | 0.000 | 0.000 | 0.770 |
| $K02034_{CON}$ | 0.172 | 0.095 | 0.02 | 0.285 | 0.124 | 0.020 | 0.045 | 0.093 | 0.390 |
| $K02035_{CON}$ | 0.137 | 0.080 | 0.04 | 0.221 | 0.120 | 0.030 | 0.079 | 0.115 | 0.340 |
| $K02036_{CON}$ | 0.249 | 0.099 | 0.02 | 0.327 | 0.138 | 0.010 | 0.000 | 0.071 | 1.000 |
| $K02037_{CON}$ | 0.232 | 0.099 | 0.01 | 0.324 | 0.136 | 0.010 | 0.000 | 0.058 | 1.000 |
| $K02038_{CON}$ | 0.207 | 0.099 | 0.03 | 0.323 | 0.134 | 0.020 | 0.000 | 0.065 | 1.000 |
| $K02039_{CON}$ | 0.231 | 0.094 | 0.02 | 0.330 | 0.128 | 0.010 | 0.000 | 0.078 | 1.000 |
| $K02066_{CON}$ | 0.205 | 0.093 | 0.02 | 0.232 | 0.132 | 0.020 | 0.143 | 0.128 | 0.160 |
| $K02067_{CON}$ | 0.144 | 0.095 | 0.05 | 0.195 | 0.129 | 0.040 | 0.064 | 0.097 | 0.350 |
| $K02072_{CON}$ | 0.141 | 0.091 | 0.01 | 0.236 | 0.129 | 0.010 | 0.000 | 0.073 | 1.000 |
| $K02073_{CON}$ | 0.125 | 0.085 | 0.07 | 0.216 | 0.122 | 0.040 | 0.000 | 0.063 | 1.000 |

| | | | | | | | | | |
|---|---|---|---|---|---|---|---|---|---|
| $K02078_{CON}$ | 0.154 | 0.088 | 0.04 | 0.176 | 0.118 | 0.040 | 0.110 | 0.101 | 0.220 |
| $K02086_{CON}$ | 0.165 | 0.093 | 0.03 | 0.265 | 0.121 | 0.020 | 0.019 | 0.078 | 0.450 |
| $K02099_{CON}$ | 0.217 | 0.089 | 0.01 | 0.251 | 0.145 | 0.010 | 0.102 | 0.116 | 0.300 |
| $K02107_{CON}$ | 0.262 | 0.099 | 0.01 | 0.464 | 0.148 | 0.010 | 0.000 | 0.072 | 1.000 |
| $K02114_{CON}$ | 0.221 | 0.100 | 0.01 | 0.305 | 0.127 | 0.010 | 0.000 | 0.073 | 1.000 |
| $K02115_{CON}$ | 0.152 | 0.095 | 0.03 | 0.218 | 0.123 | 0.020 | 0.000 | 0.088 | 1.000 |
| $K02217_{CON}$ | 0.185 | 0.096 | 0.01 | 0.236 | 0.116 | 0.030 | 0.046 | 0.079 | 0.380 |
| $K02237_{CON}$ | 0.115 | 0.079 | 0.05 | 0.287 | 0.131 | 0.010 | 0.000 | 0.079 | 1.000 |
| $K02242_{CON}$ | 0.095 | 0.075 | 0.07 | 0.156 | 0.118 | 0.030 | 0.000 | 0.063 | 1.000 |
| $K02358_{CON}$ | 0.210 | 0.101 | 0.01 | 0.278 | 0.135 | 0.020 | 0.191 | 0.153 | 0.100 |
| $K02434_{CON}$ | 0.122 | 0.087 | 0.08 | 0.200 | 0.122 | 0.030 | 0.033 | 0.096 | 0.500 |
| $K02469_{CON}$ | 0.203 | 0.092 | 0.01 | 0.381 | 0.116 | 0.010 | 0.155 | 0.126 | 0.090 |
| $K02474_{CON}$ | 0.146 | 0.086 | 0.05 | 0.198 | 0.122 | 0.030 | 0.032 | 0.093 | 0.410 |
| $K02483_{CON}$ | 0.159 | 0.086 | 0.05 | 0.260 | 0.126 | 0.010 | 0.000 | 0.076 | 1.000 |
| $K02557_{CON}$ | 0.129 | 0.079 | 0.04 | 0.180 | 0.121 | 0.040 | 0.000 | 0.075 | 1.000 |
| $K02647_{CON}$ | 0.184 | 0.104 | 0.03 | 0.204 | 0.121 | 0.030 | 0.143 | 0.129 | 0.130 |
| $K02884_{CON}$ | 0.140 | 0.092 | 0.04 | 0.241 | 0.134 | 0.020 | 0.063 | 0.111 | 0.300 |
| $K02944_{CON}$ | 0.137 | 0.082 | 0.05 | 0.188 | 0.118 | 0.020 | 0.000 | 0.000 | 0.800 |
| $K02990_{CON}$ | 0.107 | 0.077 | 0.07 | 0.190 | 0.121 | 0.040 | 0.068 | 0.117 | 0.300 |
| $K02996_{CON}$ | 0.142 | 0.090 | 0.03 | 0.218 | 0.133 | 0.020 | 0.036 | 0.108 | 0.380 |
| $K03048_{CON}$ | 0.125 | 0.080 | 0.01 | 0.256 | 0.117 | 0.010 | 0.007 | 0.079 | 0.540 |
| $K03070_{CON}$ | 0.149 | 0.081 | 0.04 | 0.184 | 0.101 | 0.030 | 0.118 | 0.127 | 0.150 |
| $K03086_{CON}$ | 0.064 | 0.062 | 0.27 | 0.179 | 0.111 | 0.030 | 0.000 | 0.074 | 1.000 |
| $K03106_{CON}$ | 0.135 | 0.083 | 0.03 | 0.169 | 0.121 | 0.040 | 0.081 | 0.109 | 0.210 |
| $K03149_{CON}$ | 0.105 | 0.079 | 0.06 | 0.192 | 0.123 | 0.020 | 0.000 | 0.076 | 1.000 |
| $K03181_{CON}$ | 0.202 | 0.096 | 0.01 | 0.242 | 0.124 | 0.030 | 0.106 | 0.121 | 0.140 |
| $K03183_{CON}$ | 0.152 | 0.084 | 0.03 | 0.177 | 0.115 | 0.040 | 0.088 | 0.124 | 0.240 |
| $K03205_{CON}$ | 0.127 | 0.083 | 0.07 | 0.160 | 0.113 | 0.040 | 0.106 | 0.126 | 0.220 |
| $K03208_{CON}$ | 0.271 | 0.106 | 0.01 | 0.320 | 0.150 | 0.010 | 0.123 | 0.131 | 0.110 |
| $K03218_{CON}$ | 0.065 | 0.074 | 0.2 | 0.211 | 0.116 | 0.040 | 0.000 | 0.070 | 1.000 |
| $K03264_{CON}$ | 0.266 | 0.116 | 0.01 | 0.302 | 0.142 | 0.010 | 0.188 | 0.132 | 0.070 |
| $K03292_{CON}$ | 0.126 | 0.085 | 0.05 | 0.218 | 0.119 | 0.030 | 0.017 | 0.084 | 0.500 |
| $K03312_{CON}$ | 0.195 | 0.088 | 0.01 | 0.236 | 0.125 | 0.020 | 0.081 | 0.100 | 0.340 |
| $K03327_{CON}$ | 0.243 | 0.109 | 0.01 | 0.267 | 0.120 | 0.010 | 0.192 | 0.162 | 0.050 |
| $K03431_{CON}$ | 0.085 | 0.074 | 0.09 | 0.173 | 0.105 | 0.040 | 0.008 | 0.068 | 0.500 |
| $K03453_{CON}$ | 0.117 | 0.084 | 0.07 | 0.179 | 0.120 | 0.040 | 0.000 | 0.063 | 1.000 |
| $K03473_{CON}$ | 0.125 | 0.079 | 0.05 | 0.181 | 0.110 | 0.020 | 0.000 | 0.071 | 1.000 |
| $K03474_{CON}$ | 0.194 | 0.095 | 0.03 | 0.220 | 0.128 | 0.010 | 0.033 | 0.084 | 0.340 |
| $K03496_{CON}$ | 0.093 | 0.072 | 0.12 | 0.153 | 0.127 | 0.030 | 0.023 | 0.092 | 0.500 |
| $K03499_{CON}$ | 0.073 | 0.082 | 0.19 | 0.157 | 0.121 | 0.030 | 0.022 | 0.084 | 0.450 |
| $K03503_{CON}$ | 0.201 | 0.094 | 0.01 | 0.158 | 0.110 | 0.040 | 0.265 | 0.172 | 0.060 |
| $K03529_{CON}$ | 0.135 | 0.083 | 0.02 | 0.247 | 0.131 | 0.010 | 0.000 | 0.069 | 1.000 |
| $K03538_{CON}$ | 0.228 | 0.100 | 0.01 | 0.269 | 0.127 | 0.010 | 0.154 | 0.138 | 0.130 |
| $K03540_{CON}$ | 0.169 | 0.082 | 0.01 | 0.172 | 0.122 | 0.040 | 0.139 | 0.141 | 0.170 |
| $K03559_{CON}$ | 0.104 | 0.082 | 0.1 | 0.169 | 0.112 | 0.020 | 0.000 | 0.068 | 1.000 |
| $K03564_{CON}$ | 0.161 | 0.089 | 0.02 | 0.239 | 0.123 | 0.010 | 0.000 | 0.082 | 1.000 |

| | | | | | | | | | |
|---|---|---|---|---|---|---|---|---|---|
| $K03581_{CON}$ | 0.157 | 0.087 | 0.01 | 0.204 | 0.118 | 0.020 | 0.116 | 0.122 | 0.210 |
| $K03585_{CON}$ | 0.226 | 0.098 | 0.02 | 0.352 | 0.148 | 0.010 | 0.034 | 0.097 | 0.430 |
| $K03596_{CON}$ | 0.159 | 0.096 | 0.02 | 0.191 | 0.129 | 0.020 | 0.106 | 0.130 | 0.220 |
| $K03601_{CON}$ | 0.219 | 0.099 | 0.01 | 0.293 | 0.146 | 0.010 | 0.100 | 0.120 | 0.200 |
| $K03634_{CON}$ | 0.112 | 0.081 | 0.09 | 0.229 | 0.112 | 0.020 | 0.000 | 0.085 | 1.000 |
| $K03639_{CON}$ | 0.101 | 0.076 | 0.14 | 0.181 | 0.117 | 0.040 | 0.027 | 0.081 | 0.510 |
| $K03650_{CON}$ | 0.151 | 0.095 | 0.05 | 0.280 | 0.130 | 0.010 | 0.000 | 0.081 | 1.000 |
| $K03660_{CON}$ | 0.131 | 0.084 | 0.05 | 0.259 | 0.131 | 0.010 | 0.000 | 0.091 | 1.000 |
| $K03688_{CON}$ | 0.142 | 0.098 | 0.05 | 0.213 | 0.127 | 0.010 | 0.088 | 0.132 | 0.230 |
| $K03698_{CON}$ | 0.232 | 0.095 | 0.01 | 0.352 | 0.124 | 0.010 | 0.000 | 0.092 | 1.000 |
| $K03700_{CON}$ | 0.132 | 0.074 | 0.07 | 0.184 | 0.109 | 0.020 | 0.000 | 0.077 | 1.000 |
| $K03703_{CON}$ | 0.124 | 0.080 | 0.05 | 0.198 | 0.118 | 0.030 | 0.047 | 0.100 | 0.410 |
| $K03705_{CON}$ | 0.094 | 0.075 | 0.1 | 0.236 | 0.140 | 0.020 | 0.000 | 0.076 | 1.000 |
| $K03709_{CON}$ | 0.175 | 0.086 | 0.01 | 0.341 | 0.140 | 0.010 | 0.000 | 0.084 | 1.000 |
| $K03710_{CON}$ | 0.177 | 0.094 | 0.01 | 0.291 | 0.126 | 0.010 | 0.003 | 0.072 | 0.560 |
| $K03724_{CON}$ | 0.180 | 0.101 | 0.02 | 0.225 | 0.125 | 0.020 | 0.152 | 0.129 | 0.100 |
| $K03734_{CON}$ | 0.099 | 0.071 | 0.11 | 0.140 | 0.098 | 0.020 | 0.063 | 0.100 | 0.270 |
| $K03741_{CON}$ | 0.224 | 0.089 | 0.01 | 0.310 | 0.132 | 0.010 | 0.000 | 0.077 | 1.000 |
| $K03753_{CON}$ | 0.140 | 0.094 | 0.04 | 0.156 | 0.137 | 0.030 | 0.237 | 0.154 | 0.050 |
| $K03798_{CON}$ | 0.162 | 0.101 | 0.01 | 0.213 | 0.122 | 0.010 | 0.133 | 0.117 | 0.140 |
| $K03816_{CON}$ | 0.143 | 0.086 | 0.03 | 0.215 | 0.112 | 0.020 | 0.000 | 0.070 | 1.000 |
| $K03855_{CON}$ | 0.169 | 0.103 | 0.01 | 0.205 | 0.130 | 0.020 | 0.154 | 0.117 | 0.070 |
| $K03892_{CON}$ | 0.097 | 0.087 | 0.11 | 0.219 | 0.113 | 0.010 | 0.000 | 0.069 | 1.000 |
| $K03924_{CON}$ | 0.146 | 0.084 | 0.02 | 0.227 | 0.120 | 0.020 | 0.000 | 0.068 | 1.000 |
| $K04034_{CON}$ | 0.029 | 0.058 | 0.36 | 0.193 | 0.118 | 0.040 | 0.000 | 0.074 | 1.000 |
| $K04047_{CON}$ | 0.337 | 0.111 | 0.01 | 0.372 | 0.138 | 0.010 | 0.205 | 0.150 | 0.090 |
| $K04062_{CON}$ | 0.224 | 0.112 | 0.01 | 0.236 | 0.124 | 0.010 | 0.159 | 0.145 | 0.100 |
| $K04070_{CON}$ | 0.142 | 0.084 | 0.02 | 0.280 | 0.143 | 0.010 | 0.000 | 0.069 | 1.000 |
| $K04085_{CON}$ | 0.253 | 0.103 | 0.01 | 0.282 | 0.139 | 0.020 | 0.148 | 0.137 | 0.130 |
| $K04486_{CON}$ | 0.105 | 0.066 | 0.1 | 0.248 | 0.136 | 0.010 | 0.000 | 0.085 | 1.000 |
| $K04487_{CON}$ | 0.120 | 0.086 | 0.08 | 0.216 | 0.124 | 0.030 | 0.071 | 0.111 | 0.310 |
| $K04518_{CON}$ | 0.177 | 0.088 | 0.03 | 0.264 | 0.137 | 0.020 | 0.028 | 0.075 | 0.350 |
| $K04751_{CON}$ | 0.120 | 0.078 | 0.04 | 0.176 | 0.120 | 0.040 | 0.000 | 0.064 | 1.000 |
| $K04759_{CON}$ | 0.050 | 0.064 | 0.28 | 0.297 | 0.124 | 0.020 | 0.000 | 0.088 | 1.000 |
| $K04796_{CON}$ | 0.132 | 0.072 | 0.01 | 0.147 | 0.100 | 0.030 | 0.064 | 0.095 | 0.390 |
| $K05801_{CON}$ | 0.226 | 0.100 | 0.01 | 0.289 | 0.138 | 0.010 | 0.109 | 0.125 | 0.160 |
| $K05832_{CON}$ | 0.288 | 0.099 | 0.01 | 0.411 | 0.143 | 0.010 | 0.127 | 0.119 | 0.200 |
| $K05833_{CON}$ | 0.208 | 0.086 | 0.01 | 0.359 | 0.147 | 0.010 | 0.000 | 0.081 | 1.000 |
| $K05919_{CON}$ | 0.136 | 0.083 | 0.03 | 0.292 | 0.136 | 0.010 | 0.000 | 0.076 | 1.000 |
| $K06020_{CON}$ | 0.192 | 0.096 | 0.01 | 0.215 | 0.119 | 0.020 | 0.124 | 0.124 | 0.130 |
| $K06023_{CON}$ | 0.082 | 0.082 | 0.12 | 0.190 | 0.120 | 0.040 | 0.000 | 0.074 | 1.000 |
| $K06041_{CON}$ | 0.113 | 0.083 | 0.05 | 0.182 | 0.126 | 0.020 | 0.000 | 0.087 | 1.000 |
| $K06077_{CON}$ | 0.144 | 0.090 | 0.03 | 0.185 | 0.125 | 0.040 | 0.024 | 0.082 | 0.440 |
| $K06147_{CON}$ | 0.116 | 0.076 | 0.05 | 0.214 | 0.110 | 0.010 | 0.016 | 0.075 | 0.520 |
| $K06201_{CON}$ | 0.153 | 0.084 | 0.03 | 0.274 | 0.134 | 0.010 | 0.030 | 0.087 | 0.370 |
| $K06207_{CON}$ | 0.151 | 0.093 | 0.02 | 0.220 | 0.119 | 0.020 | 0.106 | 0.123 | 0.210 |

| | | | | | | | | | |
|---|---|---|---|---|---|---|---|---|---|
| $K06213_{CON}$ | 0.184 | 0.088 | 0.01 | 0.337 | 0.121 | 0.010 | 0.000 | 0.069 | 1.000 |
| $K06346_{CON}$ | 0.101 | 0.084 | 0.12 | 0.228 | 0.121 | 0.020 | 0.000 | 0.072 | 1.000 |
| $K06402_{CON}$ | 0.094 | 0.077 | 0.13 | 0.190 | 0.115 | 0.040 | 0.000 | 0.081 | 1.000 |
| $K06412_{CON}$ | 0.072 | 0.071 | 0.23 | 0.175 | 0.111 | 0.020 | 0.000 | 0.062 | 1.000 |
| $K06413_{CON}$ | 0.162 | 0.098 | 0.03 | 0.178 | 0.108 | 0.020 | 0.000 | 0.000 | 0.560 |
| $K06442_{CON}$ | 0.106 | 0.076 | 0.08 | 0.222 | 0.126 | 0.020 | 0.000 | 0.080 | 1.000 |
| $K06885_{CON}$ | 0.163 | 0.090 | 0.02 | 0.181 | 0.113 | 0.040 | 0.092 | 0.118 | 0.220 |
| $K06933_{CON}$ | 0.108 | 0.085 | 0.06 | 0.318 | 0.134 | 0.010 | 0.000 | 0.082 | 1.000 |
| $K06961_{CON}$ | 0.153 | 0.080 | 0.02 | 0.175 | 0.121 | 0.020 | 0.115 | 0.110 | 0.140 |
| $K06962_{CON}$ | 0.122 | 0.077 | 0.06 | 0.152 | 0.113 | 0.030 | 0.073 | 0.112 | 0.270 |
| $K06967_{CON}$ | 0.087 | 0.076 | 0.13 | 0.192 | 0.132 | 0.010 | 0.000 | 0.075 | 1.000 |
| $K06972_{CON}$ | 0.172 | 0.096 | 0.03 | 0.350 | 0.133 | 0.010 | 0.000 | 0.084 | 1.000 |
| $K06974_{CON}$ | 0.128 | 0.081 | 0.05 | 0.151 | 0.108 | 0.030 | 0.077 | 0.104 | 0.270 |
| $K06998_{CON}$ | 0.211 | 0.102 | 0.01 | 0.249 | 0.141 | 0.010 | 0.052 | 0.102 | 0.400 |
| $K07005_{CON}$ | 0.199 | 0.101 | 0.01 | 0.277 | 0.125 | 0.010 | 0.058 | 0.105 | 0.320 |
| $K07009_{CON}$ | 0.102 | 0.076 | 0.06 | 0.170 | 0.123 | 0.040 | 0.022 | 0.081 | 0.400 |
| $K07015_{CON}$ | 0.036 | 0.065 | 0.31 | 0.193 | 0.119 | 0.030 | 0.000 | 0.091 | 1.000 |
| $K07030_{CON}$ | 0.116 | 0.097 | 0.1 | 0.236 | 0.118 | 0.010 | 0.000 | 0.075 | 1.000 |
| $K07032_{CON}$ | 0.141 | 0.089 | 0.03 | 0.191 | 0.119 | 0.020 | 0.035 | 0.090 | 0.390 |
| $K07063_{CON}$ | 0.214 | 0.093 | 0.03 | 0.244 | 0.112 | 0.020 | 0.164 | 0.132 | 0.180 |
| $K07095_{CON}$ | 0.116 | 0.084 | 0.05 | 0.171 | 0.117 | 0.030 | 0.000 | 0.071 | 1.000 |
| $K07105_{CON}$ | 0.141 | 0.084 | 0.04 | 0.225 | 0.125 | 0.020 | 0.000 | 0.084 | 1.000 |
| $K07118_{CON}$ | 0.272 | 0.117 | 0.01 | 0.336 | 0.142 | 0.010 | 0.141 | 0.130 | 0.190 |
| $K07133_{CON}$ | 0.128 | 0.082 | 0.05 | 0.173 | 0.118 | 0.020 | 0.000 | 0.067 | 1.000 |
| $K07137_{CON}$ | 0.180 | 0.106 | 0.03 | 0.225 | 0.123 | 0.020 | 0.156 | 0.148 | 0.120 |
| $K07138_{CON}$ | 0.204 | 0.091 | 0.02 | 0.223 | 0.128 | 0.030 | 0.177 | 0.128 | 0.090 |
| $K07154_{CON}$ | 0.174 | 0.093 | 0.01 | 0.274 | 0.119 | 0.010 | 0.000 | 0.081 | 1.000 |
| $K07165_{CON}$ | 0.196 | 0.094 | 0.01 | 0.266 | 0.142 | 0.010 | 0.123 | 0.134 | 0.210 |
| $K07166_{CON}$ | 0.161 | 0.100 | 0.02 | 0.304 | 0.125 | 0.010 | 0.000 | 0.069 | 1.000 |
| $K07238_{CON}$ | 0.042 | 0.058 | 0.25 | 0.223 | 0.122 | 0.020 | 0.000 | 0.080 | 1.000 |
| $K07284_{CON}$ | 0.150 | 0.084 | 0.04 | 0.239 | 0.107 | 0.010 | 0.000 | 0.079 | 1.000 |
| $K07334_{CON}$ | 0.133 | 0.087 | 0.03 | 0.216 | 0.128 | 0.040 | 0.000 | 0.083 | 1.000 |
| $K07335_{CON}$ | 0.123 | 0.079 | 0.04 | 0.267 | 0.138 | 0.010 | 0.004 | 0.078 | 0.510 |
| $K07402_{CON}$ | 0.162 | 0.081 | 0.02 | 0.236 | 0.130 | 0.030 | 0.070 | 0.104 | 0.240 |
| $K07441_{CON}$ | 0.098 | 0.082 | 0.13 | 0.179 | 0.106 | 0.040 | 0.000 | 0.081 | 1.000 |
| $K07458_{CON}$ | 0.147 | 0.081 | 0.03 | 0.262 | 0.131 | 0.030 | 0.000 | 0.079 | 1.000 |
| $K07478_{CON}$ | 0.160 | 0.088 | 0.01 | 0.246 | 0.140 | 0.040 | 0.000 | 0.064 | 1.000 |
| $K07482_{CON}$ | 0.264 | 0.106 | 0.01 | 0.337 | 0.139 | 0.010 | 0.005 | 0.066 | 0.550 |
| $K07485_{CON}$ | 0.130 | 0.079 | 0.05 | 0.173 | 0.108 | 0.020 | 0.000 | 0.074 | 1.000 |
| $K07487_{CON}$ | 0.111 | 0.076 | 0.11 | 0.169 | 0.111 | 0.030 | 0.000 | 0.063 | 1.000 |
| $K07496_{CON}$ | 0.141 | 0.082 | 0.01 | 0.219 | 0.126 | 0.030 | 0.035 | 0.091 | 0.440 |
| $K07502_{CON}$ | 0.171 | 0.086 | 0.04 | 0.256 | 0.121 | 0.020 | 0.037 | 0.086 | 0.460 |
| $K07569_{CON}$ | 0.142 | 0.079 | 0.04 | 0.170 | 0.105 | 0.010 | 0.086 | 0.124 | 0.300 |
| $K07573_{CON}$ | 0.211 | 0.095 | 0.02 | 0.275 | 0.115 | 0.010 | 0.106 | 0.121 | 0.190 |
| $K07667_{CON}$ | 0.105 | 0.084 | 0.09 | 0.196 | 0.121 | 0.030 | 0.000 | 0.063 | 1.000 |
| $K07979_{CON}$ | 0.194 | 0.090 | 0.01 | 0.188 | 0.113 | 0.010 | 0.184 | 0.142 | 0.060 |

| | | | | | | | | | |
|---|---|---|---|---|---|---|---|---|---|
| $K08138_{CON}$ | 0.057 | 0.078 | 0.3 | 0.227 | 0.133 | 0.030 | 0.000 | 0.077 | 1.000 |
| $K08234_{CON}$ | 0.183 | 0.098 | 0.02 | 0.243 | 0.132 | 0.010 | 0.044 | 0.101 | 0.350 |
| $K08307_{CON}$ | 0.217 | 0.095 | 0.01 | 0.254 | 0.139 | 0.010 | 0.082 | 0.106 | 0.360 |
| $K08364_{CON}$ | 0.400 | 0.111 | 0.01 | 0.487 | 0.121 | 0.010 | 0.148 | 0.129 | 0.140 |
| $K08591_{CON}$ | 0.050 | 0.056 | 0.26 | 0.199 | 0.129 | 0.040 | 0.000 | 0.084 | 1.000 |
| $K08600_{CON}$ | 0.180 | 0.088 | 0.03 | 0.258 | 0.129 | 0.010 | 0.097 | 0.105 | 0.240 |
| $K08602_{CON}$ | 0.165 | 0.094 | 0.02 | 0.316 | 0.117 | 0.010 | 0.000 | 0.073 | 1.000 |
| $K08963_{CON}$ | 0.187 | 0.101 | 0.03 | 0.248 | 0.139 | 0.020 | 0.099 | 0.110 | 0.190 |
| $K08986_{CON}$ | 0.126 | 0.080 | 0.04 | 0.232 | 0.139 | 0.010 | 0.000 | 0.053 | 1.000 |
| $K08999_{CON}$ | 0.203 | 0.093 | 0.01 | 0.314 | 0.136 | 0.020 | 0.000 | 0.000 | 0.190 |
| $K09116_{CON}$ | 0.180 | 0.093 | 0.01 | 0.196 | 0.128 | 0.020 | 0.104 | 0.125 | 0.210 |
| $K09131_{CON}$ | 0.148 | 0.099 | 0.02 | 0.194 | 0.120 | 0.040 | 0.044 | 0.100 | 0.290 |
| $K09157_{CON}$ | 0.337 | 0.109 | 0.01 | 0.412 | 0.146 | 0.010 | 0.221 | 0.143 | 0.050 |
| $K09762_{CON}$ | 0.078 | 0.071 | 0.15 | 0.207 | 0.113 | 0.040 | 0.000 | 0.063 | 1.000 |
| $K09774_{CON}$ | 0.176 | 0.102 | 0.03 | 0.248 | 0.125 | 0.020 | 0.064 | 0.111 | 0.380 |
| $K09797_{CON}$ | 0.142 | 0.092 | 0.04 | 0.187 | 0.122 | 0.040 | 0.000 | 0.087 | 1.000 |
| $K09800_{CON}$ | 0.124 | 0.091 | 0.05 | 0.275 | 0.124 | 0.010 | 0.000 | 0.063 | 1.000 |
| $K09816_{CON}$ | 0.186 | 0.081 | 0.02 | 0.274 | 0.126 | 0.010 | 0.000 | 0.067 | 1.000 |
| $K09825_{CON}$ | 0.159 | 0.090 | 0.03 | 0.292 | 0.133 | 0.010 | 0.000 | 0.067 | 1.000 |
| $K09935_{CON}$ | 0.159 | 0.097 | 0.05 | 0.172 | 0.105 | 0.040 | 0.117 | 0.129 | 0.200 |
| $K09976_{CON}$ | 0.159 | 0.089 | 0.02 | 0.253 | 0.125 | 0.010 | 0.037 | 0.098 | 0.440 |
| $K10119_{CON}$ | 0.159 | 0.088 | 0.02 | 0.213 | 0.120 | 0.030 | 0.053 | 0.104 | 0.400 |
| $K10773_{CON}$ | 0.092 | 0.079 | 0.13 | 0.188 | 0.132 | 0.020 | 0.000 | 0.059 | 0.470 |
| $K10907_{CON}$ | 0.062 | 0.079 | 0.25 | 0.190 | 0.123 | 0.020 | 0.000 | 0.076 | 1.000 |
| $K11041_{CON}$ | 0.192 | 0.088 | 0.03 | 0.228 | 0.112 | 0.010 | 0.157 | 0.135 | 0.140 |
| $K11260_{CON}$ | 0.187 | 0.096 | 0.03 | 0.223 | 0.133 | 0.030 | 0.084 | 0.114 | 0.170 |
| $K11261_{CON}$ | 0.156 | 0.088 | 0.01 | 0.207 | 0.126 | 0.010 | 0.035 | 0.099 | 0.360 |
| $K11720_{CON}$ | 0.111 | 0.077 | 0.05 | 0.149 | 0.114 | 0.040 | 0.042 | 0.103 | 0.350 |
| $K11752_{CON}$ | 0.139 | 0.084 | 0.06 | 0.186 | 0.127 | 0.010 | 0.021 | 0.092 | 0.480 |
| $K11753_{CON}$ | 0.239 | 0.099 | 0.01 | 0.326 | 0.143 | 0.010 | 0.036 | 0.093 | 0.370 |
| $K12132_{CON}$ | 0.177 | 0.097 | 0.04 | 0.286 | 0.128 | 0.010 | 0.068 | 0.098 | 0.290 |
| $K12264_{CON}$ | 0.096 | 0.079 | 0.09 | 0.173 | 0.113 | 0.030 | 0.000 | 0.093 | 1.000 |
| $K12524_{CON}$ | 0.120 | 0.083 | 0.09 | 0.162 | 0.111 | 0.020 | 0.070 | 0.108 | 0.270 |
| $K12952_{CON}$ | 0.214 | 0.093 | 0.01 | 0.322 | 0.136 | 0.010 | 0.108 | 0.129 | 0.290 |
| $K12962_{CON}$ | 0.143 | 0.077 | 0.03 | 0.197 | 0.111 | 0.020 | 0.015 | 0.085 | 0.510 |
| $K12984_{CON}$ | 0.197 | 0.106 | 0.01 | 0.210 | 0.120 | 0.010 | 0.145 | 0.135 | 0.140 |
| $K12998_{CON}$ | 0.183 | 0.091 | 0.01 | 0.194 | 0.110 | 0.010 | 0.137 | 0.127 | 0.190 |
| $K13002_{CON}$ | 0.151 | 0.092 | 0.04 | 0.233 | 0.132 | 0.020 | 0.000 | 0.095 | 1.000 |
| $K13283_{CON}$ | 0.249 | 0.104 | 0.01 | 0.406 | 0.138 | 0.010 | 0.000 | 0.070 | 1.000 |
| $K13292_{CON}$ | 0.114 | 0.083 | 0.09 | 0.201 | 0.112 | 0.040 | 0.025 | 0.075 | 0.420 |
| $K13993_{CON}$ | 0.145 | 0.081 | 0.04 | 0.196 | 0.122 | 0.030 | 0.023 | 0.087 | 0.400 |
| $K14102_{CON}$ | 0.175 | 0.091 | 0.02 | 0.229 | 0.136 | 0.010 | 0.040 | 0.091 | 0.470 |
| $K14111_{CON}$ | 0.153 | 0.085 | 0.01 | 0.180 | 0.113 | 0.020 | 0.089 | 0.102 | 0.200 |
| $K14112_{CON}$ | 0.156 | 0.103 | 0.04 | 0.210 | 0.128 | 0.020 | 0.014 | 0.068 | 0.580 |
| $K14118_{CON}$ | 0.142 | 0.078 | 0.04 | 0.212 | 0.133 | 0.040 | 0.000 | 0.000 | 0.140 |
| $K14122_{CON}$ | 0.172 | 0.096 | 0.01 | 0.238 | 0.123 | 0.010 | 0.000 | 0.088 | 1.000 |

| | | | | | | | | | |
|---|---|---|---|---|---|---|---|---|---|
| $K14155_{CON}$ | 0.237 | 0.094 | 0.01 | 0.308 | 0.152 | 0.010 | 0.057 | 0.089 | 0.340 |
| $K14540_{CON}$ | 0.098 | 0.079 | 0.07 | 0.202 | 0.126 | 0.030 | 0.000 | 0.000 | 0.950 |
| $K14761_{CON}$ | 0.039 | 0.060 | 0.26 | 0.181 | 0.120 | 0.030 | 0.000 | 0.064 | 1.000 |
| $K15051_{CON}$ | 0.163 | 0.084 | 0.02 | 0.278 | 0.131 | 0.020 | 0.000 | 0.070 | 1.000 |
| $K15894_{CON}$ | 0.154 | 0.095 | 0.03 | 0.196 | 0.113 | 0.020 | 0.082 | 0.119 | 0.240 |
| $K16053_{CON}$ | 0.135 | 0.086 | 0.05 | 0.212 | 0.123 | 0.020 | 0.000 | 0.057 | 1.000 |
| $K16211_{CON}$ | 0.129 | 0.076 | 0.08 | 0.188 | 0.123 | 0.020 | 0.000 | 0.085 | 1.000 |
| $K16212_{CON}$ | 0.084 | 0.074 | 0.12 | 0.184 | 0.097 | 0.020 | 0.000 | 0.062 | 1.000 |
| $K16213_{CON}$ | 0.118 | 0.084 | 0.09 | 0.209 | 0.123 | 0.030 | 0.000 | 0.068 | 1.000 |
| $K16789_{CON}$ | 0.130 | 0.085 | 0.05 | 0.207 | 0.123 | 0.030 | 0.027 | 0.093 | 0.470 |
| $K16898_{CON}$ | 0.167 | 0.092 | 0.04 | 0.273 | 0.132 | 0.010 | 0.059 | 0.108 | 0.300 |
| $K16927_{CON}$ | 0.156 | 0.083 | 0.03 | 0.304 | 0.126 | 0.010 | 0.000 | 0.094 | 1.000 |
| $K17248_{CON}$ | 0.175 | 0.090 | 0.01 | 0.175 | 0.119 | 0.020 | 0.143 | 0.136 | 0.110 |
| $K17758_{CON}$ | 0.172 | 0.097 | 0.06 | 0.228 | 0.123 | 0.020 | 0.000 | 0.072 | 1.000 |
| $K17836_{CON}$ | 0.148 | 0.088 | 0.03 | 0.211 | 0.130 | 0.020 | 0.074 | 0.111 | 0.240 |
| $K18220_{CON}$ | 0.148 | 0.087 | 0.02 | 0.244 | 0.111 | 0.010 | 0.000 | 0.078 | 1.000 |
| $K18332_{CON}$ | 0.078 | 0.072 | 0.13 | 0.136 | 0.109 | 0.040 | 0.010 | 0.080 | 0.490 |
| $K18908_{CON}$ | 0.178 | 0.098 | 0.01 | 0.270 | 0.135 | 0.010 | 0.000 | 0.073 | 1.000 |
| $K18928_{CON}$ | 0.184 | 0.097 | 0.01 | 0.208 | 0.124 | 0.020 | 0.097 | 0.117 | 0.260 |
| $K19048_{CON}$ | 0.204 | 0.101 | 0.01 | 0.263 | 0.121 | 0.010 | 0.031 | 0.101 | 0.420 |
| $K19117_{CON}$ | 0.157 | 0.095 | 0.03 | 0.221 | 0.116 | 0.030 | 0.000 | 0.076 | 1.000 |
| $K19118_{CON}$ | 0.133 | 0.077 | 0.03 | 0.218 | 0.130 | 0.020 | 0.000 | 0.069 | 1.000 |
| $K19119_{CON}$ | 0.106 | 0.072 | 0.08 | 0.184 | 0.109 | 0.020 | 0.000 | 0.074 | 1.000 |
| $K19166_{CON}$ | 0.220 | 0.108 | 0.01 | 0.240 | 0.140 | 0.020 | 0.191 | 0.155 | 0.100 |
| $K19355_{CON}$ | 0.118 | 0.083 | 0.07 | 0.231 | 0.117 | 0.020 | 0.000 | 0.061 | 1.000 |
| $K19689_{CON}$ | 0.173 | 0.087 | 0.02 | 0.325 | 0.138 | 0.010 | 0.022 | 0.067 | 0.400 |
| $K20608_{CON}$ | 0.157 | 0.084 | 0.04 | 0.180 | 0.113 | 0.040 | 0.000 | 0.000 | 0.310 |
| $K20866_{CON}$ | 0.138 | 0.080 | 0.02 | 0.182 | 0.117 | 0.030 | 0.012 | 0.088 | 0.440 |
| $K21030_{CON}$ | 0.172 | 0.087 | 0.02 | 0.209 | 0.121 | 0.020 | 0.214 | 0.140 | 0.070 |
| $K21071_{CON}$ | 0.156 | 0.097 | 0.04 | 0.296 | 0.139 | 0.010 | 0.000 | 0.079 | 1.000 |
| $K21395_{CON}$ | 0.133 | 0.082 | 0.05 | 0.156 | 0.125 | 0.040 | 0.110 | 0.119 | 0.210 |
| $K21498_{CON}$ | 0.193 | 0.093 | 0.01 | 0.181 | 0.112 | 0.020 | 0.207 | 0.130 | 0.080 |
| $K21571_{CON}$ | 0.159 | 0.092 | 0.03 | 0.182 | 0.125 | 0.010 | 0.081 | 0.115 | 0.260 |
| $K21575_{CON}$ | 0.164 | 0.098 | 0.03 | 0.249 | 0.116 | 0.030 | 0.020 | 0.079 | 0.470 |
| $K21600_{CON}$ | 0.132 | 0.079 | 0.05 | 0.265 | 0.124 | 0.020 | 0.000 | 0.069 | 1.000 |
| $K21903_{CON}$ | 0.081 | 0.067 | 0.16 | 0.172 | 0.118 | 0.020 | 0.000 | 0.074 | 1.000 |
| $K21908_{CON}$ | 0.215 | 0.085 | 0.01 | 0.277 | 0.120 | 0.010 | 0.000 | 0.090 | 1.000 |
| $K22452_{CON}$ | 0.193 | 0.084 | 0.01 | 0.280 | 0.124 | 0.010 | 0.056 | 0.103 | 0.420 |
| $K23159_{CON}$ | 0.254 | 0.106 | 0.01 | 0.320 | 0.119 | 0.010 | 0.076 | 0.101 | 0.250 |
| $K23393_{CON}$ | 0.233 | 0.097 | 0.01 | 0.242 | 0.122 | 0.010 | 0.236 | 0.145 | 0.060 |
| $K23535_{CON}$ | 0.161 | 0.096 | 0.01 | 0.289 | 0.138 | 0.010 | 0.002 | 0.083 | 0.610 |
| $K23536_{CON}$ | 0.145 | 0.089 | 0.04 | 0.297 | 0.127 | 0.010 | 0.000 | 0.080 | 1.000 |
| $K23675_{CON}$ | 0.171 | 0.092 | 0.03 | 0.235 | 0.125 | 0.020 | 0.043 | 0.088 | 0.360 |
| $K23876_{CON}$ | 0.147 | 0.086 | 0.03 | 0.240 | 0.137 | 0.010 | 0.037 | 0.102 | 0.450 |
| $K23997_{CON}$ | 0.189 | 0.094 | 0.03 | 0.304 | 0.141 | 0.010 | 0.103 | 0.134 | 0.250 |
| $K00052_{FOR}$ | 0.162 | 0.092 | 0.02 | 0.078 | 0.088 | 0.190 | 0.301 | 0.162 | 0.040 |

| | | | | | | | | | |
|---|---|---|---|---|---|---|---|---|---|
| $K00077_{FOR}$ | 0.167 | 0.093 | 0.03 | 0.081 | 0.091 | 0.130 | 0.280 | 0.170 | 0.010 |
| $K00088_{FOR}$ | 0.149 | 0.085 | 0.04 | 0.000 | 0.059 | 1.000 | 0.318 | 0.167 | 0.010 |
| $K00125_{FOR}$ | 0.264 | 0.098 | 0.01 | 0.088 | 0.099 | 0.160 | 0.342 | 0.151 | 0.010 |
| $K00176_{FOR}$ | 0.050 | 0.063 | 0.24 | 0.000 | 0.050 | 1.000 | 0.266 | 0.152 | 0.020 |
| $K00243_{FOR}$ | 0.142 | 0.077 | 0.06 | 0.075 | 0.078 | 0.210 | 0.220 | 0.143 | 0.040 |
| $K00259_{FOR}$ | 0.152 | 0.098 | 0.02 | 0.131 | 0.110 | 0.150 | 0.200 | 0.155 | 0.040 |
| $K00265_{FOR}$ | 0.285 | 0.111 | 0.01 | 0.146 | 0.114 | 0.090 | 0.470 | 0.169 | 0.010 |
| $K00548_{FOR}$ | 0.246 | 0.104 | 0.01 | 0.174 | 0.109 | 0.050 | 0.304 | 0.162 | 0.020 |
| $K00558_{FOR}$ | 0.157 | 0.085 | 0.02 | 0.000 | 0.056 | 1.000 | 0.254 | 0.150 | 0.020 |
| $K00588_{FOR}$ | 0.084 | 0.076 | 0.17 | 0.029 | 0.062 | 0.420 | 0.209 | 0.159 | 0.030 |
| $K00615_{FOR}$ | 0.101 | 0.073 | 0.06 | 0.064 | 0.088 | 0.240 | 0.216 | 0.135 | 0.040 |
| $K00641_{FOR}$ | 0.198 | 0.096 | 0.02 | 0.113 | 0.114 | 0.120 | 0.343 | 0.161 | 0.010 |
| $K00766_{FOR}$ | 0.180 | 0.102 | 0.01 | 0.077 | 0.082 | 0.230 | 0.300 | 0.156 | 0.030 |
| $K00768_{FOR}$ | 0.093 | 0.071 | 0.09 | 0.000 | 0.058 | 1.000 | 0.266 | 0.146 | 0.030 |
| $K00794_{FOR}$ | 0.105 | 0.072 | 0.06 | 0.036 | 0.071 | 0.380 | 0.205 | 0.132 | 0.040 |
| $K00817_{FOR}$ | 0.170 | 0.089 | 0.01 | 0.108 | 0.095 | 0.130 | 0.254 | 0.159 | 0.010 |
| $K00831_{FOR}$ | 0.089 | 0.077 | 0.23 | 0.014 | 0.051 | 0.410 | 0.196 | 0.161 | 0.040 |
| $K00864_{FOR}$ | 0.201 | 0.102 | 0.01 | 0.135 | 0.095 | 0.060 | 0.276 | 0.162 | 0.030 |
| $K01179_{FOR}$ | 0.185 | 0.087 | 0.01 | 0.000 | 0.056 | 1.000 | 0.370 | 0.166 | 0.020 |
| $K01200_{FOR}$ | 0.081 | 0.081 | 0.23 | 0.009 | 0.061 | 0.530 | 0.248 | 0.152 | 0.040 |
| $K01463_{FOR}$ | 0.031 | 0.055 | 0.4 | 0.000 | 0.056 | 1.000 | 0.257 | 0.141 | 0.010 |
| $K01487_{FOR}$ | 0.059 | 0.062 | 0.23 | 0.000 | 0.063 | 1.000 | 0.391 | 0.171 | 0.010 |
| $K01488_{FOR}$ | 0.152 | 0.089 | 0.03 | 0.000 | 0.076 | 1.000 | 0.322 | 0.158 | 0.010 |
| $K01649_{FOR}$ | 0.260 | 0.105 | 0.01 | 0.135 | 0.110 | 0.060 | 0.397 | 0.164 | 0.010 |
| $K01652_{FOR}$ | 0.128 | 0.080 | 0.08 | 0.009 | 0.062 | 0.520 | 0.295 | 0.134 | 0.010 |
| $K01686_{FOR}$ | 0.195 | 0.091 | 0.01 | 0.138 | 0.112 | 0.100 | 0.260 | 0.161 | 0.030 |
| $K01687_{FOR}$ | 0.222 | 0.103 | 0.01 | 0.173 | 0.103 | 0.050 | 0.325 | 0.153 | 0.010 |
| $K01696_{FOR}$ | 0.125 | 0.091 | 0.04 | 0.004 | 0.061 | 0.560 | 0.427 | 0.160 | 0.010 |
| $K01710_{FOR}$ | 0.074 | 0.067 | 0.13 | 0.000 | 0.059 | 1.000 | 0.335 | 0.177 | 0.020 |
| $K01733_{FOR}$ | 0.211 | 0.090 | 0.01 | 0.106 | 0.093 | 0.170 | 0.351 | 0.156 | 0.020 |
| $K01740_{FOR}$ | 0.256 | 0.100 | 0.01 | 0.000 | 0.053 | 1.000 | 0.447 | 0.163 | 0.010 |
| $K01755_{FOR}$ | 0.079 | 0.075 | 0.19 | 0.000 | 0.062 | 1.000 | 0.194 | 0.135 | 0.040 |
| $K01756_{FOR}$ | 0.215 | 0.101 | 0.01 | 0.082 | 0.093 | 0.170 | 0.356 | 0.166 | 0.010 |
| $K01778_{FOR}$ | 0.065 | 0.066 | 0.17 | 0.000 | 0.061 | 1.000 | 0.203 | 0.142 | 0.020 |
| $K01874_{FOR}$ | 0.166 | 0.097 | 0.02 | 0.156 | 0.098 | 0.070 | 0.274 | 0.151 | 0.010 |
| $K01876_{FOR}$ | 0.146 | 0.088 | 0.02 | 0.073 | 0.099 | 0.190 | 0.262 | 0.149 | 0.030 |
| $K01915_{FOR}$ | 0.158 | 0.087 | 0.02 | 0.000 | 0.058 | 1.000 | 0.307 | 0.152 | 0.020 |
| $K01953_{FOR}$ | 0.210 | 0.101 | 0.02 | 0.170 | 0.118 | 0.070 | 0.274 | 0.167 | 0.030 |
| $K02231_{FOR}$ | 0.077 | 0.066 | 0.18 | 0.000 | 0.060 | 1.000 | 0.253 | 0.158 | 0.040 |
| $K02315_{FOR}$ | 0.162 | 0.096 | 0.03 | 0.086 | 0.097 | 0.120 | 0.289 | 0.151 | 0.020 |
| $K02379_{FOR}$ | 0.151 | 0.091 | 0.06 | 0.017 | 0.067 | 0.330 | 0.310 | 0.162 | 0.020 |
| $K02466_{FOR}$ | 0.099 | 0.077 | 0.07 | 0.000 | 0.058 | 1.000 | 0.431 | 0.174 | 0.010 |
| $K02472_{FOR}$ | 0.114 | 0.074 | 0.08 | 0.015 | 0.063 | 0.500 | 0.343 | 0.158 | 0.040 |
| $K02500_{FOR}$ | 0.192 | 0.098 | 0.02 | 0.074 | 0.094 | 0.220 | 0.333 | 0.173 | 0.010 |
| $K02529_{FOR}$ | 0.194 | 0.096 | 0.01 | 0.131 | 0.112 | 0.050 | 0.252 | 0.159 | 0.020 |
| $K02558_{FOR}$ | 0.073 | 0.080 | 0.14 | 0.000 | 0.048 | 1.000 | 0.280 | 0.154 | 0.030 |

| | | | | | | | | | |
|---|---|---|---|---|---|---|---|---|---|
| $K02759_{FOR}$ | 0.202 | 0.095 | 0.01 | 0.196 | 0.122 | 0.080 | 0.283 | 0.155 | 0.020 |
| $K02770_{FOR}$ | 0.111 | 0.078 | 0.07 | 0.082 | 0.095 | 0.220 | 0.179 | 0.151 | 0.030 |
| $K02781_{FOR}$ | 0.134 | 0.093 | 0.06 | 0.047 | 0.087 | 0.350 | 0.330 | 0.169 | 0.020 |
| $K02911_{FOR}$ | 0.099 | 0.076 | 0.08 | 0.005 | 0.054 | 0.510 | 0.218 | 0.147 | 0.030 |
| $K02913_{FOR}$ | 0.183 | 0.091 | 0.03 | 0.000 | 0.056 | 1.000 | 0.326 | 0.179 | 0.020 |
| $K02914_{FOR}$ | 0.114 | 0.086 | 0.05 | 0.009 | 0.065 | 0.580 | 0.251 | 0.156 | 0.040 |
| $K02916_{FOR}$ | 0.199 | 0.102 | 0.02 | 0.091 | 0.098 | 0.160 | 0.255 | 0.153 | 0.040 |
| $K02945_{FOR}$ | 0.147 | 0.086 | 0.07 | 0.076 | 0.093 | 0.210 | 0.222 | 0.146 | 0.040 |
| $K02954_{FOR}$ | 0.143 | 0.091 | 0.05 | 0.000 | 0.057 | 1.000 | 0.325 | 0.168 | 0.010 |
| $K02965_{FOR}$ | 0.132 | 0.075 | 0.05 | 0.017 | 0.067 | 0.430 | 0.259 | 0.162 | 0.040 |
| $K03046_{FOR}$ | 0.194 | 0.088 | 0.01 | 0.196 | 0.118 | 0.050 | 0.255 | 0.164 | 0.020 |
| $K03113_{FOR}$ | 0.112 | 0.081 | 0.05 | 0.005 | 0.062 | 0.500 | 0.445 | 0.195 | 0.010 |
| $K03217_{FOR}$ | 0.126 | 0.077 | 0.05 | 0.078 | 0.094 | 0.160 | 0.271 | 0.156 | 0.030 |
| $K03313_{FOR}$ | 0.088 | 0.086 | 0.09 | 0.000 | 0.076 | 1.000 | 0.200 | 0.146 | 0.040 |
| $K03394_{FOR}$ | 0.142 | 0.095 | 0.02 | 0.048 | 0.088 | 0.300 | 0.257 | 0.150 | 0.040 |
| $K03430_{FOR}$ | 0.096 | 0.073 | 0.12 | 0.033 | 0.071 | 0.420 | 0.235 | 0.164 | 0.020 |
| $K03534_{FOR}$ | 0.030 | 0.052 | 0.33 | 0.000 | 0.068 | 1.000 | 0.200 | 0.138 | 0.040 |
| $K03630_{FOR}$ | 0.134 | 0.087 | 0.06 | 0.034 | 0.072 | 0.380 | 0.258 | 0.157 | 0.020 |
| $K03702_{FOR}$ | 0.135 | 0.088 | 0.02 | 0.020 | 0.072 | 0.420 | 0.323 | 0.158 | 0.040 |
| $K03791_{FOR}$ | 0.128 | 0.091 | 0.05 | 0.129 | 0.110 | 0.060 | 0.293 | 0.165 | 0.010 |
| $K03929_{FOR}$ | 0.188 | 0.096 | 0.01 | 0.162 | 0.125 | 0.090 | 0.248 | 0.134 | 0.040 |
| $K03973_{FOR}$ | 0.097 | 0.080 | 0.12 | 0.057 | 0.087 | 0.340 | 0.253 | 0.155 | 0.040 |
| $K04078_{FOR}$ | 0.081 | 0.076 | 0.13 | 0.000 | 0.070 | 1.000 | 0.223 | 0.155 | 0.030 |
| $K04720_{FOR}$ | 0.119 | 0.078 | 0.05 | 0.030 | 0.076 | 0.420 | 0.222 | 0.160 | 0.030 |
| $K04761_{FOR}$ | 0.145 | 0.096 | 0.04 | 0.061 | 0.076 | 0.250 | 0.250 | 0.144 | 0.040 |
| $K05337_{FOR}$ | 0.161 | 0.091 | 0.01 | 0.094 | 0.091 | 0.160 | 0.224 | 0.161 | 0.030 |
| $K05595_{FOR}$ | 0.184 | 0.087 | 0.01 | 0.136 | 0.093 | 0.090 | 0.256 | 0.166 | 0.040 |
| $K05794_{FOR}$ | 0.046 | 0.063 | 0.26 | 0.008 | 0.069 | 0.480 | 0.312 | 0.165 | 0.020 |
| $K06177_{FOR}$ | 0.135 | 0.081 | 0.02 | 0.059 | 0.081 | 0.280 | 0.315 | 0.179 | 0.010 |
| $K06215_{FOR}$ | 0.117 | 0.084 | 0.08 | 0.043 | 0.086 | 0.360 | 0.239 | 0.148 | 0.040 |
| $K06394_{FOR}$ | 0.030 | 0.050 | 0.39 | 0.071 | 0.083 | 0.170 | 0.000 | 0.000 | 0.010 |
| $K06875_{FOR}$ | 0.172 | 0.094 | 0.02 | 0.146 | 0.100 | 0.090 | 0.219 | 0.162 | 0.030 |
| $K06919_{FOR}$ | 0.098 | 0.079 | 0.13 | 0.000 | 0.067 | 1.000 | 0.305 | 0.151 | 0.020 |
| $K06987_{FOR}$ | 0.300 | 0.115 | 0.01 | 0.056 | 0.075 | 0.240 | 0.524 | 0.176 | 0.010 |
| $K07058_{FOR}$ | 0.189 | 0.087 | 0.02 | 0.165 | 0.117 | 0.050 | 0.247 | 0.164 | 0.040 |
| $K07150_{FOR}$ | 0.122 | 0.086 | 0.05 | 0.015 | 0.056 | 0.450 | 0.234 | 0.130 | 0.010 |
| $K07343_{FOR}$ | 0.030 | 0.060 | 0.37 | 0.000 | 0.061 | 1.000 | 0.302 | 0.161 | 0.010 |
| $K07387_{FOR}$ | 0.183 | 0.090 | 0.01 | 0.129 | 0.114 | 0.120 | 0.262 | 0.151 | 0.020 |
| $K07454_{FOR}$ | 0.093 | 0.074 | 0.12 | 0.000 | 0.056 | 1.000 | 0.227 | 0.151 | 0.020 |
| $K07488_{FOR}$ | 0.073 | 0.072 | 0.16 | 0.000 | 0.067 | 1.000 | 0.196 | 0.133 | 0.040 |
| $K07492_{FOR}$ | 0.178 | 0.091 | 0.02 | 0.090 | 0.099 | 0.180 | 0.290 | 0.157 | 0.010 |
| $K08987_{FOR}$ | 0.140 | 0.075 | 0.06 | 0.080 | 0.073 | 0.280 | 0.243 | 0.160 | 0.040 |
| $K09690_{FOR}$ | 0.101 | 0.077 | 0.11 | 0.078 | 0.089 | 0.170 | 0.268 | 0.140 | 0.040 |
| $K09691_{FOR}$ | 0.144 | 0.085 | 0.03 | 0.092 | 0.093 | 0.190 | 0.316 | 0.181 | 0.030 |
| $K09705_{FOR}$ | 0.193 | 0.086 | 0.01 | 0.122 | 0.097 | 0.200 | 0.325 | 0.166 | 0.010 |
| $K09710_{FOR}$ | 0.073 | 0.070 | 0.19 | 0.000 | 0.054 | 1.000 | 0.341 | 0.186 | 0.030 |

| | | | | | | | | | |
|---|---|---|---|---|---|---|---|---|---|
| $K09793_{FOR}$ | 0.176 | 0.092 | 0.01 | 0.083 | 0.088 | 0.180 | 0.420 | 0.161 | 0.010 |
| $K10206_{FOR}$ | 0.084 | 0.081 | 0.16 | 0.000 | 0.050 | 1.000 | 0.344 | 0.166 | 0.010 |
| $K11751_{FOR}$ | 0.067 | 0.067 | 0.24 | 0.000 | 0.063 | 1.000 | 0.219 | 0.162 | 0.030 |
| $K12063_{FOR}$ | 0.131 | 0.084 | 0.06 | 0.092 | 0.092 | 0.200 | 0.184 | 0.145 | 0.030 |
| $K12257_{FOR}$ | 0.125 | 0.085 | 0.07 | 0.000 | 0.055 | 1.000 | 0.298 | 0.152 | 0.040 |
| $K12373_{FOR}$ | 0.111 | 0.083 | 0.03 | 0.055 | 0.078 | 0.290 | 0.188 | 0.146 | 0.030 |
| $K13018_{FOR}$ | 0.107 | 0.085 | 0.09 | 0.018 | 0.059 | 0.440 | 0.373 | 0.154 | 0.010 |
| $K13599_{FOR}$ | 0.103 | 0.075 | 0.12 | 0.000 | 0.049 | 1.000 | 0.314 | 0.183 | 0.010 |
| $K15876_{FOR}$ | 0.199 | 0.092 | 0.01 | 0.008 | 0.061 | 0.580 | 0.385 | 0.139 | 0.010 |
| $K15915_{FOR}$ | 0.053 | 0.074 | 0.28 | 0.000 | 0.055 | 1.000 | 0.360 | 0.174 | 0.010 |
| $K16329_{FOR}$ | 0.161 | 0.084 | 0.03 | 0.118 | 0.103 | 0.100 | 0.284 | 0.157 | 0.020 |
| $K16850_{FOR}$ | 0.140 | 0.086 | 0.02 | 0.070 | 0.089 | 0.280 | 0.199 | 0.148 | 0.030 |
| $K17290_{FOR}$ | 0.138 | 0.078 | 0.03 | 0.044 | 0.084 | 0.340 | 0.250 | 0.153 | 0.030 |
| $K17828_{FOR}$ | 0.075 | 0.063 | 0.2 | 0.000 | 0.052 | 1.000 | 0.253 | 0.162 | 0.040 |
| $K18346_{FOR}$ | 0.128 | 0.078 | 0.02 | 0.063 | 0.079 | 0.230 | 0.283 | 0.160 | 0.010 |
| $K18700_{FOR}$ | 0.081 | 0.071 | 0.11 | 0.024 | 0.060 | 0.470 | 0.350 | 0.164 | 0.020 |
| $K19158_{FOR}$ | 0.091 | 0.074 | 0.11 | 0.000 | 0.069 | 1.000 | 0.436 | 0.163 | 0.010 |
| $K19271_{FOR}$ | 0.207 | 0.088 | 0.02 | 0.205 | 0.125 | 0.050 | 0.213 | 0.151 | 0.030 |
| $K19304_{FOR}$ | 0.179 | 0.091 | 0.01 | 0.125 | 0.104 | 0.120 | 0.275 | 0.151 | 0.010 |
| $K22132_{FOR}$ | 0.059 | 0.074 | 0.2 | 0.000 | 0.053 | 1.000 | 0.252 | 0.161 | 0.020 |
| $K23351_{FOR}$ | 0.153 | 0.091 | 0.02 | 0.000 | 0.054 | 1.000 | 0.387 | 0.172 | 0.010 |
| $K24180_{FOR}$ | 0.077 | 0.081 | 0.12 | 0.000 | 0.056 | 1.000 | 0.217 | 0.144 | 0.030 |
| K00003 | 0.109 | 0.076 | 0.04 | 0.134 | 0.104 | 0.080 | 0.091 | 0.104 | 0.260 |
| K00012 | 0.097 | 0.078 | 0.04 | 0.111 | 0.102 | 0.170 | 0.055 | 0.115 | 0.320 |
| K00013 | 0.164 | 0.093 | 0.01 | 0.148 | 0.093 | 0.050 | 0.169 | 0.134 | 0.140 |
| K00014 | 0.065 | 0.070 | 0.26 | 0.065 | 0.089 | 0.240 | 0.041 | 0.093 | 0.370 |
| K00016 | 0.102 | 0.081 | 0.09 | 0.177 | 0.119 | 0.060 | 0.000 | 0.076 | 1.000 |
| K00018 | 0.000 | 0.000 | 0.99 | 0.000 | 0.050 | 1.000 | 0.000 | 0.068 | 1.000 |
| K00027 | 0.045 | 0.062 | 0.31 | 0.000 | 0.057 | 1.000 | 0.099 | 0.130 | 0.240 |
| K00029 | 0.111 | 0.081 | 0.11 | 0.101 | 0.094 | 0.160 | 0.100 | 0.118 | 0.300 |
| K00031 | 0.127 | 0.078 | 0.07 | 0.151 | 0.112 | 0.050 | 0.062 | 0.087 | 0.330 |
| K00041 | 0.000 | 0.035 | 1 | 0.000 | 0.056 | 1.000 | 0.000 | 0.076 | 1.000 |
| K00046 | 0.120 | 0.077 | 0.04 | 0.156 | 0.112 | 0.050 | 0.000 | 0.066 | 1.000 |
| K00053 | 0.101 | 0.079 | 0.08 | 0.064 | 0.085 | 0.250 | 0.137 | 0.119 | 0.140 |
| K00058 | 0.045 | 0.061 | 0.23 | 0.068 | 0.086 | 0.180 | 0.001 | 0.060 | 0.600 |
| K00059 | 0.000 | 0.044 | 1 | 0.000 | 0.056 | 1.000 | 0.000 | 0.077 | 1.000 |
| K00067 | 0.131 | 0.075 | 0.04 | 0.099 | 0.090 | 0.110 | 0.183 | 0.150 | 0.120 |
| K00074 | 0.000 | 0.043 | 1 | 0.000 | 0.065 | 1.000 | 0.000 | 0.077 | 1.000 |
| K00075 | 0.021 | 0.056 | 0.35 | 0.079 | 0.085 | 0.240 | 0.000 | 0.073 | 1.000 |
| K00078 | 0.000 | 0.052 | 1 | 0.000 | 0.062 | 1.000 | 0.000 | 0.071 | 1.000 |
| K00091 | 0.110 | 0.081 | 0.1 | 0.101 | 0.100 | 0.200 | 0.119 | 0.118 | 0.140 |
| K00096 | 0.003 | 0.043 | 0.46 | 0.000 | 0.062 | 1.000 | 0.163 | 0.135 | 0.070 |
| K00099 | 0.050 | 0.066 | 0.26 | 0.000 | 0.060 | 1.000 | 0.152 | 0.113 | 0.130 |
| K00112 | 0.007 | 0.045 | 0.54 | 0.053 | 0.069 | 0.320 | 0.000 | 0.068 | 1.000 |
| K00133 | 0.000 | 0.039 | 1 | 0.000 | 0.059 | 1.000 | 0.017 | 0.072 | 0.510 |
| K00145 | 0.184 | 0.089 | 0.02 | 0.175 | 0.108 | 0.070 | 0.186 | 0.166 | 0.090 |
| K00147 | 0.083 | 0.064 | 0.11 | 0.176 | 0.118 | 0.060 | 0.000 | 0.074 | 1.000 |
| K00169 | 0.077 | 0.073 | 0.13 | 0.048 | 0.081 | 0.340 | 0.147 | 0.140 | 0.130 |
| K00170 | 0.085 | 0.076 | 0.1 | 0.126 | 0.109 | 0.070 | 0.001 | 0.075 | 0.480 |
| K00171 | 0.099 | 0.088 | 0.11 | 0.102 | 0.103 | 0.140 | 0.075 | 0.099 | 0.260 |

| | | | | | | | | | |
|---|---|---|---|---|---|---|---|---|---|
| K00172 | 0.059 | 0.072 | 0.2 | 0.100 | 0.101 | 0.200 | 0.000 | 0.000 | 0.860 |
| K00174 | 0.007 | 0.048 | 0.54 | 0.000 | 0.062 | 1.000 | 0.050 | 0.111 | 0.340 |
| K00175 | 0.000 | 0.048 | 1 | 0.000 | 0.065 | 1.000 | 0.000 | 0.073 | 1.000 |
| K00177 | 0.000 | 0.041 | 1 | 0.000 | 0.061 | 1.000 | 0.000 | 0.075 | 1.000 |
| K00179 | 0.010 | 0.053 | 0.52 | 0.000 | 0.056 | 1.000 | 0.096 | 0.119 | 0.210 |
| K00180 | 0.028 | 0.063 | 0.34 | 0.008 | 0.071 | 0.470 | 0.098 | 0.117 | 0.150 |
| K00215 | 0.000 | 0.042 | 1 | 0.000 | 0.055 | 1.000 | 0.046 | 0.091 | 0.330 |
| K00239 | 0.047 | 0.061 | 0.29 | 0.079 | 0.098 | 0.210 | 0.000 | 0.073 | 1.000 |
| K00240 | 0.010 | 0.047 | 0.49 | 0.030 | 0.076 | 0.420 | 0.000 | 0.097 | 1.000 |
| K00241 | 0.000 | 0.033 | 1 | 0.000 | 0.000 | 0.590 | 0.000 | 0.070 | 1.000 |
| K00244 | 0.089 | 0.075 | 0.09 | 0.134 | 0.110 | 0.100 | 0.026 | 0.085 | 0.470 |
| K00262 | 0.000 | 0.043 | 1 | 0.054 | 0.081 | 0.340 | 0.000 | 0.074 | 1.000 |
| K00275 | 0.137 | 0.078 | 0.07 | 0.102 | 0.103 | 0.160 | 0.186 | 0.155 | 0.090 |
| K00278 | 0.054 | 0.068 | 0.17 | 0.118 | 0.105 | 0.150 | 0.000 | 0.065 | 1.000 |
| K00282 | 0.053 | 0.063 | 0.2 | 0.028 | 0.059 | 0.300 | 0.107 | 0.124 | 0.240 |
| K00283 | 0.000 | 0.046 | 1 | 0.001 | 0.048 | 0.460 | 0.000 | 0.072 | 1.000 |
| K00287 | 0.063 | 0.069 | 0.2 | 0.014 | 0.066 | 0.470 | 0.116 | 0.135 | 0.180 |
| K00290 | 0.084 | 0.081 | 0.14 | 0.075 | 0.096 | 0.240 | 0.108 | 0.132 | 0.250 |
| K00325 | 0.064 | 0.064 | 0.28 | 0.057 | 0.086 | 0.210 | 0.062 | 0.091 | 0.380 |
| K00330 | 0.121 | 0.069 | 0.06 | 0.112 | 0.116 | 0.120 | 0.129 | 0.125 | 0.240 |
| K00331 | 0.000 | 0.044 | 1 | 0.078 | 0.093 | 0.230 | 0.000 | 0.082 | 1.000 |
| K00334 | 0.000 | 0.047 | 1 | 0.000 | 0.068 | 1.000 | 0.000 | 0.082 | 1.000 |
| K00335 | 0.004 | 0.036 | 0.48 | 0.000 | 0.063 | 1.000 | 0.066 | 0.103 | 0.330 |
| K00338 | 0.038 | 0.057 | 0.27 | 0.056 | 0.076 | 0.240 | 0.000 | 0.084 | 1.000 |
| K00340 | 0.116 | 0.088 | 0.06 | 0.178 | 0.132 | 0.050 | 0.000 | 0.000 | 0.360 |
| K00343 | 0.108 | 0.073 | 0.03 | 0.104 | 0.102 | 0.140 | 0.097 | 0.133 | 0.210 |
| K00346 | 0.046 | 0.051 | 0.35 | 0.110 | 0.103 | 0.200 | 0.000 | 0.083 | 1.000 |
| K00347 | 0.051 | 0.064 | 0.29 | 0.125 | 0.112 | 0.080 | 0.000 | 0.078 | 1.000 |
| K00349 | 0.050 | 0.069 | 0.28 | 0.161 | 0.106 | 0.050 | 0.000 | 0.064 | 1.000 |
| K00350 | 0.044 | 0.055 | 0.22 | 0.119 | 0.103 | 0.130 | 0.000 | 0.071 | 1.000 |
| K00351 | 0.000 | 0.047 | 1 | 0.026 | 0.075 | 0.380 | 0.000 | 0.074 | 1.000 |
| K00382 | 0.000 | 0.045 | 1 | 0.000 | 0.055 | 1.000 | 0.000 | 0.073 | 1.000 |
| K00395 | 0.032 | 0.052 | 0.33 | 0.024 | 0.083 | 0.380 | 0.053 | 0.108 | 0.390 |
| K00425 | 0.000 | 0.044 | 1 | 0.048 | 0.075 | 0.360 | 0.000 | 0.068 | 1.000 |
| K00426 | 0.055 | 0.058 | 0.28 | 0.040 | 0.076 | 0.290 | 0.063 | 0.099 | 0.330 |
| K00432 | 0.060 | 0.060 | 0.2 | 0.082 | 0.090 | 0.240 | 0.000 | 0.083 | 1.000 |
| K00459 | 0.086 | 0.071 | 0.14 | 0.000 | 0.053 | 1.000 | 0.151 | 0.134 | 0.100 |
| K00525 | 0.115 | 0.095 | 0.1 | 0.129 | 0.116 | 0.090 | 0.135 | 0.139 | 0.130 |
| K00537 | 0.076 | 0.079 | 0.14 | 0.135 | 0.117 | 0.070 | 0.000 | 0.082 | 1.000 |
| K00549 | 0.095 | 0.075 | 0.08 | 0.139 | 0.098 | 0.060 | 0.071 | 0.113 | 0.370 |
| K00554 | 0.063 | 0.063 | 0.14 | 0.159 | 0.108 | 0.080 | 0.000 | 0.069 | 1.000 |
| K00560 | 0.083 | 0.077 | 0.14 | 0.065 | 0.084 | 0.280 | 0.135 | 0.118 | 0.120 |
| K00563 | 0.017 | 0.042 | 0.44 | 0.034 | 0.091 | 0.330 | 0.002 | 0.074 | 0.630 |
| K00564 | 0.013 | 0.044 | 0.51 | 0.049 | 0.077 | 0.360 | 0.000 | 0.055 | 1.000 |
| K00566 | 0.000 | 0.040 | 1 | 0.117 | 0.097 | 0.090 | 0.000 | 0.056 | 1.000 |
| K00571 | 0.000 | 0.035 | 1 | 0.000 | 0.049 | 1.000 | 0.000 | 0.080 | 1.000 |
| K00578 | 0.091 | 0.072 | 0.11 | 0.137 | 0.101 | 0.120 | 0.000 | 0.080 | 1.000 |
| K00595 | 0.102 | 0.079 | 0.1 | 0.090 | 0.102 | 0.140 | 0.107 | 0.118 | 0.240 |
| K00600 | 0.064 | 0.055 | 0.14 | 0.139 | 0.103 | 0.100 | 0.045 | 0.085 | 0.430 |
| K00602 | 0.096 | 0.078 | 0.15 | 0.076 | 0.089 | 0.180 | 0.104 | 0.108 | 0.190 |
| K00605 | 0.030 | 0.051 | 0.38 | 0.054 | 0.080 | 0.340 | 0.000 | 0.096 | 1.000 |
| K00606 | 0.049 | 0.061 | 0.28 | 0.115 | 0.106 | 0.130 | 0.000 | 0.090 | 1.000 |
| K00609 | 0.123 | 0.072 | 0.04 | 0.073 | 0.098 | 0.170 | 0.214 | 0.131 | 0.050 |

| | | | | | | | | | |
|---|---|---|---|---|---|---|---|---|---|
| K00610 | 0.144 | 0.082 | 0.06 | 0.149 | 0.106 | 0.090 | 0.122 | 0.106 | 0.170 |
| K00611 | 0.000 | 0.047 | 1 | 0.037 | 0.075 | 0.300 | 0.000 | 0.083 | 1.000 |
| K00616 | 0.001 | 0.039 | 0.59 | 0.075 | 0.091 | 0.270 | 0.000 | 0.064 | 1.000 |
| K00620 | 0.095 | 0.081 | 0.15 | 0.129 | 0.102 | 0.120 | 0.086 | 0.111 | 0.260 |
| K00625 | 0.093 | 0.076 | 0.12 | 0.124 | 0.111 | 0.080 | 0.075 | 0.105 | 0.250 |
| K00639 | 0.064 | 0.064 | 0.19 | 0.037 | 0.070 | 0.390 | 0.129 | 0.124 | 0.140 |
| K00640 | 0.152 | 0.092 | 0.05 | 0.145 | 0.104 | 0.070 | 0.156 | 0.145 | 0.140 |
| K00645 | 0.048 | 0.065 | 0.29 | 0.000 | 0.049 | 1.000 | 0.100 | 0.139 | 0.290 |
| K00647 | 0.056 | 0.068 | 0.19 | 0.038 | 0.071 | 0.320 | 0.086 | 0.108 | 0.280 |
| K00648 | 0.111 | 0.074 | 0.06 | 0.037 | 0.064 | 0.330 | 0.179 | 0.141 | 0.080 |
| K00651 | 0.191 | 0.093 | 0.01 | 0.183 | 0.118 | 0.060 | 0.185 | 0.144 | 0.110 |
| K00655 | 0.022 | 0.058 | 0.34 | 0.094 | 0.097 | 0.170 | 0.000 | 0.000 | 0.740 |
| K00656 | 0.145 | 0.088 | 0.03 | 0.198 | 0.112 | 0.050 | 0.064 | 0.108 | 0.210 |
| K00657 | 0.024 | 0.049 | 0.41 | 0.042 | 0.066 | 0.390 | 0.025 | 0.090 | 0.420 |
| K00661 | 0.000 | 0.039 | 1 | 0.008 | 0.055 | 0.500 | 0.000 | 0.090 | 1.000 |
| K00662 | 0.186 | 0.090 | 0.01 | 0.180 | 0.119 | 0.050 | 0.183 | 0.145 | 0.090 |
| K00666 | 0.000 | 0.035 | 1 | 0.000 | 0.061 | 1.000 | 0.000 | 0.072 | 1.000 |
| K00677 | 0.057 | 0.068 | 0.26 | 0.090 | 0.092 | 0.150 | 0.000 | 0.080 | 1.000 |
| K00682 | 0.000 | 0.040 | 1 | 0.023 | 0.063 | 0.520 | 0.000 | 0.071 | 1.000 |
| K00683 | 0.031 | 0.053 | 0.36 | 0.070 | 0.089 | 0.260 | 0.000 | 0.074 | 1.000 |
| K00700 | 0.085 | 0.075 | 0.11 | 0.037 | 0.081 | 0.380 | 0.155 | 0.162 | 0.100 |
| K00702 | 0.078 | 0.064 | 0.21 | 0.070 | 0.093 | 0.210 | 0.086 | 0.115 | 0.330 |
| K00705 | 0.149 | 0.087 | 0.05 | 0.155 | 0.116 | 0.060 | 0.163 | 0.128 | 0.100 |
| K00721 | 0.011 | 0.041 | 0.44 | 0.005 | 0.064 | 0.470 | 0.027 | 0.095 | 0.460 |
| K00748 | 0.107 | 0.082 | 0.07 | 0.113 | 0.097 | 0.110 | 0.083 | 0.104 | 0.350 |
| K00756 | 0.000 | 0.037 | 1 | 0.000 | 0.064 | 1.000 | 0.037 | 0.094 | 0.400 |
| K00757 | 0.115 | 0.093 | 0.04 | 0.107 | 0.106 | 0.110 | 0.134 | 0.125 | 0.130 |
| K00759 | 0.087 | 0.080 | 0.11 | 0.071 | 0.088 | 0.180 | 0.112 | 0.126 | 0.170 |
| K00760 | 0.000 | 0.047 | 1 | 0.000 | 0.057 | 1.000 | 0.000 | 0.069 | 1.000 |
| K00761 | 0.000 | 0.037 | 1 | 0.067 | 0.103 | 0.250 | 0.000 | 0.087 | 1.000 |
| K00762 | 0.040 | 0.058 | 0.29 | 0.117 | 0.091 | 0.120 | 0.000 | 0.092 | 1.000 |
| K00764 | 0.061 | 0.069 | 0.24 | 0.000 | 0.048 | 1.000 | 0.199 | 0.145 | 0.070 |
| K00765 | 0.016 | 0.055 | 0.4 | 0.000 | 0.061 | 1.000 | 0.025 | 0.096 | 0.430 |
| K00767 | 0.080 | 0.073 | 0.12 | 0.116 | 0.100 | 0.200 | 0.000 | 0.063 | 1.000 |
| K00773 | 0.000 | 0.052 | 1 | 0.000 | 0.053 | 1.000 | 0.000 | 0.087 | 1.000 |
| K00783 | 0.070 | 0.065 | 0.13 | 0.050 | 0.075 | 0.390 | 0.117 | 0.116 | 0.150 |
| K00789 | 0.026 | 0.050 | 0.44 | 0.023 | 0.067 | 0.430 | 0.032 | 0.094 | 0.370 |
| K00790 | 0.086 | 0.079 | 0.19 | 0.006 | 0.056 | 0.440 | 0.223 | 0.166 | 0.070 |
| K00791 | 0.000 | 0.043 | 1 | 0.000 | 0.052 | 1.000 | 0.031 | 0.092 | 0.490 |
| K00793 | 0.113 | 0.077 | 0.07 | 0.051 | 0.091 | 0.270 | 0.176 | 0.134 | 0.130 |
| K00796 | 0.104 | 0.088 | 0.08 | 0.151 | 0.116 | 0.090 | 0.048 | 0.085 | 0.360 |
| K00797 | 0.029 | 0.057 | 0.45 | 0.145 | 0.102 | 0.060 | 0.000 | 0.076 | 1.000 |
| K00798 | 0.082 | 0.079 | 0.14 | 0.092 | 0.096 | 0.180 | 0.044 | 0.099 | 0.380 |
| K00800 | 0.000 | 0.040 | 1 | 0.000 | 0.049 | 1.000 | 0.006 | 0.078 | 0.430 |
| K00806 | 0.000 | 0.038 | 1 | 0.000 | 0.061 | 1.000 | 0.000 | 0.071 | 1.000 |
| K00812 | 0.020 | 0.056 | 0.41 | 0.088 | 0.083 | 0.170 | 0.000 | 0.085 | 1.000 |
| K00821 | 0.000 | 0.041 | 0.56 | 0.024 | 0.056 | 0.350 | 0.000 | 0.059 | 1.000 |
| K00845 | 0.000 | 0.043 | 1 | 0.000 | 0.057 | 1.000 | 0.000 | 0.000 | 0.720 |
| K00848 | 0.000 | 0.057 | 1 | 0.000 | 0.052 | 1.000 | 0.000 | 0.074 | 1.000 |
| K00849 | 0.031 | 0.056 | 0.32 | 0.077 | 0.087 | 0.160 | 0.000 | 0.062 | 1.000 |
| K00850 | 0.000 | 0.046 | 1 | 0.000 | 0.056 | 1.000 | 0.036 | 0.088 | 0.430 |
| K00852 | 0.030 | 0.056 | 0.35 | 0.035 | 0.073 | 0.410 | 0.008 | 0.075 | 0.500 |
| K00854 | 0.144 | 0.087 | 0.02 | 0.136 | 0.112 | 0.110 | 0.174 | 0.157 | 0.090 |

| | | | | | | | | | |
|---|---|---|---|---|---|---|---|---|---|
| K00857 | 0.098 | 0.081 | 0.11 | 0.045 | 0.063 | 0.330 | 0.199 | 0.146 | 0.080 |
| K00858 | 0.000 | 0.050 | 1 | 0.072 | 0.085 | 0.250 | 0.000 | 0.083 | 1.000 |
| K00859 | 0.088 | 0.070 | 0.12 | 0.074 | 0.102 | 0.220 | 0.104 | 0.128 | 0.230 |
| K00865 | 0.087 | 0.072 | 0.09 | 0.053 | 0.069 | 0.200 | 0.149 | 0.140 | 0.120 |
| K00868 | 0.056 | 0.065 | 0.21 | 0.000 | 0.063 | 1.000 | 0.113 | 0.127 | 0.200 |
| K00873 | 0.064 | 0.068 | 0.27 | 0.026 | 0.065 | 0.420 | 0.124 | 0.142 | 0.130 |
| K00874 | 0.079 | 0.067 | 0.21 | 0.066 | 0.095 | 0.230 | 0.092 | 0.127 | 0.240 |
| K00876 | 0.090 | 0.086 | 0.1 | 0.109 | 0.088 | 0.090 | 0.087 | 0.119 | 0.290 |
| K00887 | 0.143 | 0.075 | 0.04 | 0.168 | 0.120 | 0.050 | 0.075 | 0.101 | 0.320 |
| K00891 | 0.018 | 0.051 | 0.45 | 0.000 | 0.063 | 1.000 | 0.040 | 0.098 | 0.340 |
| K00895 | 0.000 | 0.051 | 1 | 0.089 | 0.096 | 0.190 | 0.000 | 0.089 | 1.000 |
| K00912 | 0.087 | 0.071 | 0.17 | 0.107 | 0.086 | 0.150 | 0.031 | 0.093 | 0.410 |
| K00919 | 0.021 | 0.043 | 0.38 | 0.050 | 0.072 | 0.320 | 0.003 | 0.072 | 0.600 |
| K00925 | 0.020 | 0.045 | 0.44 | 0.030 | 0.069 | 0.310 | 0.022 | 0.083 | 0.490 |
| K00926 | 0.064 | 0.070 | 0.22 | 0.085 | 0.087 | 0.120 | 0.054 | 0.097 | 0.290 |
| K00928 | 0.000 | 0.041 | 1 | 0.000 | 0.047 | 1.000 | 0.000 | 0.075 | 1.000 |
| K00929 | 0.031 | 0.066 | 0.26 | 0.000 | 0.065 | 1.000 | 0.083 | 0.108 | 0.290 |
| K00930 | 0.071 | 0.071 | 0.14 | 0.044 | 0.081 | 0.320 | 0.093 | 0.122 | 0.210 |
| K00931 | 0.064 | 0.061 | 0.27 | 0.035 | 0.076 | 0.350 | 0.103 | 0.128 | 0.230 |
| K00937 | 0.163 | 0.085 | 0.04 | 0.162 | 0.110 | 0.050 | 0.154 | 0.147 | 0.160 |
| K00939 | 0.006 | 0.044 | 0.46 | 0.001 | 0.059 | 0.500 | 0.006 | 0.073 | 0.420 |
| K00942 | 0.007 | 0.042 | 0.46 | 0.009 | 0.063 | 0.500 | 0.000 | 0.000 | 0.560 |
| K00943 | 0.000 | 0.043 | 1 | 0.070 | 0.081 | 0.350 | 0.000 | 0.069 | 1.000 |
| K00945 | 0.000 | 0.041 | 1 | 0.000 | 0.049 | 1.000 | 0.000 | 0.065 | 1.000 |
| K00946 | 0.061 | 0.067 | 0.22 | 0.015 | 0.065 | 0.490 | 0.111 | 0.118 | 0.160 |
| K00948 | 0.101 | 0.086 | 0.11 | 0.180 | 0.109 | 0.070 | 0.000 | 0.075 | 1.000 |
| K00949 | 0.077 | 0.080 | 0.16 | 0.111 | 0.106 | 0.200 | 0.010 | 0.081 | 0.500 |
| K00954 | 0.017 | 0.044 | 0.45 | 0.002 | 0.051 | 0.560 | 0.034 | 0.092 | 0.390 |
| K00963 | 0.100 | 0.087 | 0.07 | 0.142 | 0.111 | 0.070 | 0.072 | 0.096 | 0.310 |
| K00965 | 0.082 | 0.077 | 0.14 | 0.086 | 0.087 | 0.210 | 0.099 | 0.127 | 0.200 |
| K00969 | 0.000 | 0.043 | 1 | 0.000 | 0.061 | 1.000 | 0.000 | 0.078 | 1.000 |
| K00971 | 0.032 | 0.058 | 0.34 | 0.058 | 0.082 | 0.270 | 0.000 | 0.082 | 1.000 |
| K00972 | 0.013 | 0.052 | 0.47 | 0.127 | 0.102 | 0.060 | 0.000 | 0.066 | 1.000 |
| K00973 | 0.008 | 0.047 | 0.48 | 0.000 | 0.061 | 1.000 | 0.062 | 0.099 | 0.270 |
| K00974 | 0.000 | 0.044 | 1 | 0.010 | 0.059 | 0.510 | 0.000 | 0.069 | 1.000 |
| K00979 | 0.114 | 0.078 | 0.1 | 0.105 | 0.101 | 0.130 | 0.136 | 0.119 | 0.120 |
| K00980 | 0.007 | 0.047 | 0.49 | 0.074 | 0.086 | 0.160 | 0.000 | 0.077 | 1.000 |
| K00981 | 0.000 | 0.049 | 1 | 0.000 | 0.049 | 1.000 | 0.000 | 0.085 | 1.000 |
| K00986 | 0.066 | 0.063 | 0.17 | 0.078 | 0.096 | 0.220 | 0.029 | 0.083 | 0.390 |
| K00992 | 0.058 | 0.066 | 0.21 | 0.049 | 0.073 | 0.430 | 0.069 | 0.105 | 0.290 |
| K00995 | 0.004 | 0.047 | 0.52 | 0.106 | 0.102 | 0.130 | 0.000 | 0.073 | 1.000 |
| K00997 | 0.061 | 0.068 | 0.22 | 0.141 | 0.111 | 0.050 | 0.000 | 0.101 | 1.000 |
| K01000 | 0.033 | 0.065 | 0.26 | 0.029 | 0.068 | 0.400 | 0.043 | 0.100 | 0.380 |
| K01006 | 0.079 | 0.075 | 0.14 | 0.081 | 0.096 | 0.190 | 0.106 | 0.116 | 0.200 |
| K01009 | 0.067 | 0.062 | 0.17 | 0.112 | 0.111 | 0.090 | 0.000 | 0.075 | 1.000 |
| K01046 | 0.005 | 0.037 | 0.52 | 0.028 | 0.074 | 0.400 | 0.000 | 0.065 | 1.000 |
| K01056 | 0.000 | 0.045 | 1 | 0.034 | 0.058 | 0.390 | 0.000 | 0.076 | 1.000 |
| K01069 | 0.000 | 0.047 | 1 | 0.000 | 0.050 | 1.000 | 0.048 | 0.104 | 0.410 |
| K01077 | 0.116 | 0.081 | 0.04 | 0.108 | 0.097 | 0.130 | 0.185 | 0.137 | 0.050 |
| K01079 | 0.144 | 0.090 | 0.01 | 0.092 | 0.091 | 0.160 | 0.218 | 0.164 | 0.070 |
| K01085 | 0.000 | 0.049 | 1 | 0.000 | 0.066 | 1.000 | 0.061 | 0.093 | 0.290 |
| K01089 | 0.068 | 0.073 | 0.12 | 0.121 | 0.112 | 0.190 | 0.000 | 0.068 | 1.000 |
| K01101 | 0.001 | 0.039 | 0.51 | 0.004 | 0.063 | 0.610 | 0.009 | 0.073 | 0.450 |

| | | | | | | | | | |
|---|---|---|---|---|---|---|---|---|---|
| K01104 | 0.049 | 0.072 | 0.27 | 0.067 | 0.088 | 0.170 | 0.000 | 0.074 | 1.000 |
| K01118 | 0.026 | 0.049 | 0.3 | 0.026 | 0.062 | 0.460 | 0.006 | 0.083 | 0.570 |
| K01119 | 0.079 | 0.072 | 0.14 | 0.000 | 0.071 | 1.000 | 0.162 | 0.136 | 0.100 |
| K01126 | 0.000 | 0.037 | 1 | 0.000 | 0.061 | 1.000 | 0.000 | 0.076 | 1.000 |
| K01129 | 0.119 | 0.092 | 0.09 | 0.138 | 0.102 | 0.050 | 0.087 | 0.107 | 0.270 |
| K01142 | 0.024 | 0.058 | 0.47 | 0.069 | 0.087 | 0.280 | 0.000 | 0.080 | 1.000 |
| K01144 | 0.019 | 0.051 | 0.34 | 0.084 | 0.093 | 0.220 | 0.000 | 0.063 | 1.000 |
| K01151 | 0.004 | 0.049 | 0.47 | 0.143 | 0.115 | 0.070 | 0.000 | 0.071 | 1.000 |
| K01153 | 0.009 | 0.044 | 0.48 | 0.000 | 0.063 | 1.000 | 0.065 | 0.094 | 0.320 |
| K01154 | 0.101 | 0.074 | 0.12 | 0.052 | 0.087 | 0.250 | 0.171 | 0.138 | 0.090 |
| K01155 | 0.021 | 0.047 | 0.52 | 0.037 | 0.069 | 0.330 | 0.004 | 0.065 | 0.510 |
| K01159 | 0.000 | 0.048 | 1 | 0.000 | 0.073 | 1.000 | 0.000 | 0.073 | 1.000 |
| K01160 | 0.000 | 0.039 | 1 | 0.000 | 0.055 | 1.000 | 0.000 | 0.077 | 1.000 |
| K01163 | 0.000 | 0.040 | 1 | 0.000 | 0.055 | 1.000 | 0.000 | 0.089 | 1.000 |
| K01167 | 0.000 | 0.048 | 1 | 0.000 | 0.062 | 1.000 | 0.000 | 0.088 | 1.000 |
| K01173 | 0.014 | 0.039 | 0.4 | 0.060 | 0.083 | 0.210 | 0.000 | 0.072 | 1.000 |
| K01174 | 0.005 | 0.044 | 0.53 | 0.000 | 0.061 | 1.000 | 0.170 | 0.136 | 0.060 |
| K01176 | 0.042 | 0.053 | 0.28 | 0.017 | 0.059 | 0.430 | 0.092 | 0.110 | 0.310 |
| K01181 | 0.000 | 0.039 | 1 | 0.061 | 0.078 | 0.280 | 0.000 | 0.068 | 1.000 |
| K01185 | 0.000 | 0.047 | 1 | 0.000 | 0.069 | 1.000 | 0.000 | 0.069 | 1.000 |
| K01187 | 0.010 | 0.049 | 0.39 | 0.006 | 0.053 | 0.490 | 0.018 | 0.077 | 0.440 |
| K01190 | 0.041 | 0.063 | 0.21 | 0.050 | 0.079 | 0.280 | 0.013 | 0.095 | 0.460 |
| K01192 | 0.092 | 0.073 | 0.09 | 0.174 | 0.116 | 0.060 | 0.000 | 0.068 | 1.000 |
| K01195 | 0.000 | 0.043 | 1 | 0.000 | 0.062 | 1.000 | 0.000 | 0.079 | 1.000 |
| K01198 | 0.027 | 0.059 | 0.37 | 0.067 | 0.082 | 0.220 | 0.000 | 0.070 | 1.000 |
| K01201 | 0.029 | 0.060 | 0.35 | 0.000 | 0.052 | 1.000 | 0.150 | 0.152 | 0.120 |
| K01205 | 0.034 | 0.059 | 0.34 | 0.042 | 0.082 | 0.320 | 0.052 | 0.101 | 0.360 |
| K01206 | 0.000 | 0.042 | 1 | 0.000 | 0.062 | 1.000 | 0.022 | 0.081 | 0.540 |
| K01207 | 0.026 | 0.055 | 0.42 | 0.041 | 0.080 | 0.340 | 0.000 | 0.070 | 1.000 |
| K01208 | 0.031 | 0.065 | 0.33 | 0.078 | 0.098 | 0.230 | 0.000 | 0.077 | 1.000 |
| K01209 | 0.097 | 0.071 | 0.07 | 0.075 | 0.085 | 0.230 | 0.120 | 0.126 | 0.190 |
| K01224 | 0.000 | 0.036 | 1 | 0.000 | 0.074 | 1.000 | 0.000 | 0.068 | 1.000 |
| K01235 | 0.056 | 0.075 | 0.21 | 0.096 | 0.098 | 0.150 | 0.006 | 0.085 | 0.560 |
| K01239 | 0.095 | 0.075 | 0.09 | 0.120 | 0.099 | 0.120 | 0.044 | 0.115 | 0.370 |
| K01243 | 0.060 | 0.071 | 0.18 | 0.021 | 0.067 | 0.360 | 0.099 | 0.118 | 0.250 |
| K01246 | 0.035 | 0.053 | 0.34 | 0.074 | 0.085 | 0.250 | 0.000 | 0.073 | 1.000 |
| K01251 | 0.000 | 0.042 | 1 | 0.000 | 0.059 | 1.000 | 0.000 | 0.000 | 0.620 |
| K01255 | 0.000 | 0.046 | 1 | 0.000 | 0.053 | 1.000 | 0.000 | 0.057 | 1.000 |
| K01259 | 0.000 | 0.043 | 1 | 0.020 | 0.060 | 0.420 | 0.000 | 0.063 | 1.000 |
| K01262 | 0.140 | 0.089 | 0.02 | 0.110 | 0.104 | 0.110 | 0.201 | 0.148 | 0.110 |
| K01265 | 0.000 | 0.039 | 1 | 0.000 | 0.051 | 1.000 | 0.000 | 0.091 | 1.000 |
| K01270 | 0.007 | 0.041 | 0.43 | 0.095 | 0.092 | 0.200 | 0.000 | 0.079 | 1.000 |
| K01273 | 0.014 | 0.049 | 0.39 | 0.073 | 0.090 | 0.230 | 0.000 | 0.077 | 1.000 |
| K01277 | 0.062 | 0.063 | 0.17 | 0.126 | 0.099 | 0.060 | 0.000 | 0.071 | 1.000 |
| K01278 | 0.024 | 0.056 | 0.36 | 0.085 | 0.107 | 0.210 | 0.000 | 0.082 | 1.000 |
| K01284 | 0.021 | 0.053 | 0.42 | 0.059 | 0.085 | 0.220 | 0.000 | 0.083 | 1.000 |
| K01303 | 0.072 | 0.073 | 0.17 | 0.075 | 0.090 | 0.210 | 0.093 | 0.119 | 0.270 |
| K01308 | 0.000 | 0.039 | 1 | 0.000 | 0.056 | 1.000 | 0.000 | 0.051 | 1.000 |
| K01338 | 0.000 | 0.049 | 1 | 0.000 | 0.064 | 1.000 | 0.000 | 0.074 | 1.000 |
| K01356 | 0.000 | 0.044 | 1 | 0.000 | 0.000 | 0.700 | 0.000 | 0.072 | 1.000 |
| K01358 | 0.000 | 0.046 | 1 | 0.000 | 0.068 | 1.000 | 0.000 | 0.096 | 1.000 |
| K01372 | 0.016 | 0.055 | 0.44 | 0.077 | 0.082 | 0.220 | 0.000 | 0.068 | 1.000 |
| K01409 | 0.049 | 0.066 | 0.27 | 0.117 | 0.104 | 0.090 | 0.000 | 0.085 | 1.000 |

| | | | | | | | | | |
|---|---|---|---|---|---|---|---|---|---|
| K01419 | 0.089 | 0.073 | 0.15 | 0.135 | 0.112 | 0.120 | 0.012 | 0.074 | 0.500 |
| K01421 | 0.063 | 0.074 | 0.25 | 0.017 | 0.068 | 0.540 | 0.217 | 0.162 | 0.050 |
| K01424 | 0.000 | 0.046 | 1 | 0.000 | 0.063 | 1.000 | 0.054 | 0.100 | 0.380 |
| K01425 | 0.046 | 0.068 | 0.32 | 0.053 | 0.095 | 0.340 | 0.000 | 0.000 | 0.580 |
| K01447 | 0.000 | 0.041 | 1 | 0.000 | 0.056 | 1.000 | 0.000 | 0.067 | 1.000 |
| K01448 | 0.000 | 0.041 | 1 | 0.078 | 0.085 | 0.240 | 0.000 | 0.081 | 1.000 |
| K01449 | 0.048 | 0.067 | 0.29 | 0.102 | 0.103 | 0.120 | 0.000 | 0.067 | 1.000 |
| K01462 | 0.000 | 0.038 | 1 | 0.000 | 0.048 | 1.000 | 0.000 | 0.083 | 1.000 |
| K01478 | 0.081 | 0.080 | 0.12 | 0.074 | 0.081 | 0.230 | 0.085 | 0.109 | 0.240 |
| K01480 | 0.000 | 0.045 | 1 | 0.046 | 0.080 | 0.280 | 0.000 | 0.071 | 1.000 |
| K01486 | 0.156 | 0.095 | 0.02 | 0.063 | 0.085 | 0.250 | 0.263 | 0.152 | 0.050 |
| K01489 | 0.000 | 0.039 | 1 | 0.000 | 0.050 | 1.000 | 0.105 | 0.130 | 0.210 |
| K01491 | 0.000 | 0.042 | 1 | 0.000 | 0.058 | 1.000 | 0.000 | 0.078 | 1.000 |
| K01493 | 0.089 | 0.066 | 0.07 | 0.054 | 0.082 | 0.310 | 0.143 | 0.125 | 0.100 |
| K01494 | 0.048 | 0.060 | 0.27 | 0.027 | 0.084 | 0.400 | 0.107 | 0.126 | 0.240 |
| K01495 | 0.084 | 0.074 | 0.12 | 0.044 | 0.074 | 0.330 | 0.138 | 0.132 | 0.140 |
| K01507 | 0.015 | 0.046 | 0.59 | 0.077 | 0.086 | 0.250 | 0.000 | 0.087 | 1.000 |
| K01512 | 0.112 | 0.082 | 0.1 | 0.150 | 0.110 | 0.050 | 0.010 | 0.068 | 0.570 |
| K01518 | 0.010 | 0.043 | 0.43 | 0.078 | 0.099 | 0.250 | 0.000 | 0.075 | 1.000 |
| K01520 | 0.000 | 0.034 | 1 | 0.000 | 0.049 | 1.000 | 0.000 | 0.080 | 1.000 |
| K01524 | 0.151 | 0.088 | 0.05 | 0.209 | 0.119 | 0.050 | 0.026 | 0.093 | 0.460 |
| K01534 | 0.112 | 0.079 | 0.05 | 0.173 | 0.126 | 0.050 | 0.072 | 0.105 | 0.370 |
| K01551 | 0.114 | 0.080 | 0.12 | 0.115 | 0.091 | 0.150 | 0.101 | 0.120 | 0.240 |
| K01573 | 0.016 | 0.048 | 0.43 | 0.033 | 0.073 | 0.400 | 0.000 | 0.079 | 1.000 |
| K01575 | 0.122 | 0.084 | 0.07 | 0.147 | 0.116 | 0.080 | 0.129 | 0.127 | 0.200 |
| K01579 | 0.035 | 0.065 | 0.36 | 0.000 | 0.057 | 1.000 | 0.114 | 0.126 | 0.230 |
| K01585 | 0.046 | 0.062 | 0.23 | 0.126 | 0.099 | 0.100 | 0.000 | 0.081 | 1.000 |
| K01586 | 0.000 | 0.047 | 1 | 0.000 | 0.067 | 1.000 | 0.000 | 0.075 | 1.000 |
| K01588 | 0.000 | 0.035 | 1 | 0.054 | 0.071 | 0.330 | 0.000 | 0.101 | 1.000 |
| K01607 | 0.043 | 0.055 | 0.32 | 0.130 | 0.111 | 0.100 | 0.000 | 0.071 | 1.000 |
| K01609 | 0.000 | 0.037 | 1 | 0.041 | 0.076 | 0.400 | 0.000 | 0.067 | 1.000 |
| K01611 | 0.000 | 0.038 | 1 | 0.000 | 0.000 | 0.800 | 0.000 | 0.068 | 1.000 |
| K01619 | 0.000 | 0.036 | 1 | 0.000 | 0.052 | 1.000 | 0.000 | 0.076 | 1.000 |
| K01625 | 0.092 | 0.077 | 0.07 | 0.151 | 0.115 | 0.060 | 0.017 | 0.075 | 0.570 |
| K01626 | 0.043 | 0.067 | 0.25 | 0.133 | 0.105 | 0.120 | 0.000 | 0.069 | 1.000 |
| K01627 | 0.143 | 0.092 | 0.03 | 0.170 | 0.104 | 0.050 | 0.089 | 0.112 | 0.310 |
| K01629 | 0.000 | 0.048 | 1 | 0.019 | 0.058 | 0.520 | 0.000 | 0.064 | 1.000 |
| K01633 | 0.042 | 0.065 | 0.33 | 0.072 | 0.098 | 0.150 | 0.000 | 0.079 | 1.000 |
| K01643 | 0.000 | 0.037 | 1 | 0.010 | 0.054 | 0.490 | 0.000 | 0.000 | 0.430 |
| K01646 | 0.019 | 0.049 | 0.4 | 0.010 | 0.060 | 0.490 | 0.039 | 0.085 | 0.290 |
| K01653 | 0.008 | 0.041 | 0.47 | 0.000 | 0.047 | 1.000 | 0.104 | 0.119 | 0.270 |
| K01658 | 0.072 | 0.072 | 0.19 | 0.141 | 0.096 | 0.100 | 0.000 | 0.076 | 1.000 |
| K01661 | 0.058 | 0.065 | 0.18 | 0.096 | 0.105 | 0.240 | 0.000 | 0.079 | 1.000 |
| K01662 | 0.036 | 0.059 | 0.45 | 0.000 | 0.049 | 1.000 | 0.086 | 0.111 | 0.250 |
| K01673 | 0.005 | 0.042 | 0.48 | 0.000 | 0.054 | 1.000 | 0.052 | 0.102 | 0.300 |
| K01676 | 0.000 | 0.050 | 1 | 0.034 | 0.090 | 0.370 | 0.000 | 0.070 | 1.000 |
| K01677 | 0.115 | 0.090 | 0.06 | 0.177 | 0.117 | 0.070 | 0.058 | 0.089 | 0.350 |
| K01679 | 0.011 | 0.050 | 0.52 | 0.000 | 0.063 | 1.000 | 0.034 | 0.099 | 0.390 |
| K01685 | 0.060 | 0.054 | 0.26 | 0.070 | 0.084 | 0.120 | 0.041 | 0.101 | 0.410 |
| K01689 | 0.099 | 0.074 | 0.09 | 0.186 | 0.123 | 0.050 | 0.000 | 0.000 | 1.000 |
| K01693 | 0.031 | 0.060 | 0.34 | 0.151 | 0.109 | 0.070 | 0.000 | 0.072 | 1.000 |
| K01695 | 0.024 | 0.052 | 0.41 | 0.042 | 0.072 | 0.320 | 0.000 | 0.056 | 1.000 |
| K01698 | 0.100 | 0.076 | 0.06 | 0.157 | 0.105 | 0.070 | 0.019 | 0.075 | 0.520 |

| | | | | | | | | | |
|---|---|---|---|---|---|---|---|---|---|
| K01704 | 0.060 | 0.075 | 0.16 | 0.051 | 0.082 | 0.320 | 0.052 | 0.102 | 0.410 |
| K01709 | 0.125 | 0.085 | 0.04 | 0.139 | 0.105 | 0.140 | 0.130 | 0.129 | 0.180 |
| K01711 | 0.159 | 0.089 | 0.05 | 0.147 | 0.101 | 0.090 | 0.150 | 0.135 | 0.110 |
| K01714 | 0.008 | 0.040 | 0.46 | 0.000 | 0.057 | 1.000 | 0.020 | 0.085 | 0.490 |
| K01719 | 0.106 | 0.088 | 0.05 | 0.134 | 0.102 | 0.080 | 0.022 | 0.079 | 0.500 |
| K01726 | 0.034 | 0.065 | 0.27 | 0.040 | 0.070 | 0.400 | 0.000 | 0.082 | 1.000 |
| K01735 | 0.093 | 0.069 | 0.12 | 0.000 | 0.000 | 0.360 | 0.201 | 0.141 | 0.050 |
| K01736 | 0.155 | 0.094 | 0.05 | 0.152 | 0.117 | 0.080 | 0.145 | 0.141 | 0.170 |
| K01737 | 0.066 | 0.073 | 0.11 | 0.000 | 0.057 | 1.000 | 0.142 | 0.144 | 0.160 |
| K01744 | 0.035 | 0.059 | 0.41 | 0.000 | 0.055 | 1.000 | 0.065 | 0.112 | 0.280 |
| K01745 | 0.039 | 0.064 | 0.39 | 0.000 | 0.056 | 1.000 | 0.163 | 0.154 | 0.050 |
| K01749 | 0.042 | 0.062 | 0.34 | 0.029 | 0.067 | 0.410 | 0.072 | 0.092 | 0.390 |
| K01754 | 0.000 | 0.047 | 1 | 0.046 | 0.085 | 0.380 | 0.000 | 0.070 | 1.000 |
| K01770 | 0.000 | 0.037 | 1 | 0.000 | 0.062 | 1.000 | 0.046 | 0.092 | 0.410 |
| K01775 | 0.000 | 0.032 | 1 | 0.095 | 0.090 | 0.150 | 0.000 | 0.065 | 1.000 |
| K01776 | 0.000 | 0.042 | 1 | 0.000 | 0.059 | 1.000 | 0.000 | 0.082 | 1.000 |
| K01779 | 0.010 | 0.046 | 0.59 | 0.009 | 0.059 | 0.470 | 0.044 | 0.097 | 0.320 |
| K01780 | 0.002 | 0.040 | 0.55 | 0.000 | 0.055 | 1.000 | 0.068 | 0.108 | 0.350 |
| K01783 | 0.000 | 0.033 | 1 | 0.000 | 0.056 | 1.000 | 0.024 | 0.088 | 0.490 |
| K01784 | 0.000 | 0.044 | 1 | 0.034 | 0.069 | 0.330 | 0.000 | 0.063 | 1.000 |
| K01785 | 0.050 | 0.062 | 0.23 | 0.099 | 0.096 | 0.150 | 0.000 | 0.070 | 1.000 |
| K01790 | 0.062 | 0.061 | 0.19 | 0.056 | 0.086 | 0.290 | 0.060 | 0.103 | 0.370 |
| K01791 | 0.000 | 0.047 | 1 | 0.000 | 0.059 | 1.000 | 0.038 | 0.091 | 0.380 |
| K01803 | 0.054 | 0.063 | 0.22 | 0.014 | 0.063 | 0.470 | 0.092 | 0.127 | 0.310 |
| K01804 | 0.000 | 0.040 | 1 | 0.000 | 0.059 | 1.000 | 0.000 | 0.062 | 1.000 |
| K01805 | 0.000 | 0.042 | 1 | 0.000 | 0.065 | 1.000 | 0.000 | 0.067 | 1.000 |
| K01807 | 0.019 | 0.050 | 0.43 | 0.048 | 0.080 | 0.360 | 0.000 | 0.063 | 1.000 |
| K01808 | 0.000 | 0.048 | 1 | 0.000 | 0.073 | 1.000 | 0.000 | 0.085 | 1.000 |
| K01809 | 0.000 | 0.048 | 1 | 0.000 | 0.061 | 1.000 | 0.008 | 0.085 | 0.440 |
| K01810 | 0.130 | 0.077 | 0.05 | 0.120 | 0.115 | 0.170 | 0.175 | 0.145 | 0.090 |
| K01811 | 0.018 | 0.056 | 0.43 | 0.038 | 0.077 | 0.490 | 0.000 | 0.064 | 1.000 |
| K01812 | 0.106 | 0.082 | 0.05 | 0.051 | 0.098 | 0.180 | 0.149 | 0.140 | 0.120 |
| K01813 | 0.000 | 0.054 | 1 | 0.003 | 0.056 | 0.510 | 0.000 | 0.084 | 1.000 |
| K01814 | 0.080 | 0.062 | 0.1 | 0.041 | 0.083 | 0.380 | 0.139 | 0.128 | 0.120 |
| K01817 | 0.032 | 0.052 | 0.37 | 0.045 | 0.090 | 0.390 | 0.000 | 0.080 | 1.000 |
| K01818 | 0.000 | 0.042 | 1 | 0.056 | 0.090 | 0.260 | 0.000 | 0.070 | 1.000 |
| K01821 | 0.061 | 0.058 | 0.11 | 0.030 | 0.066 | 0.400 | 0.103 | 0.116 | 0.250 |
| K01834 | 0.154 | 0.093 | 0.05 | 0.053 | 0.080 | 0.280 | 0.232 | 0.163 | 0.050 |
| K01838 | 0.045 | 0.055 | 0.25 | 0.076 | 0.090 | 0.220 | 0.000 | 0.060 | 1.000 |
| K01839 | 0.018 | 0.052 | 0.3 | 0.086 | 0.096 | 0.230 | 0.024 | 0.095 | 0.330 |
| K01845 | 0.150 | 0.086 | 0.04 | 0.166 | 0.109 | 0.050 | 0.122 | 0.132 | 0.170 |
| K01847 | 0.000 | 0.051 | 1 | 0.000 | 0.048 | 1.000 | 0.000 | 0.075 | 1.000 |
| K01854 | 0.068 | 0.074 | 0.16 | 0.123 | 0.117 | 0.160 | 0.020 | 0.079 | 0.500 |
| K01858 | 0.070 | 0.070 | 0.15 | 0.080 | 0.086 | 0.230 | 0.067 | 0.095 | 0.300 |
| K01866 | 0.000 | 0.052 | 1 | 0.000 | 0.063 | 1.000 | 0.063 | 0.100 | 0.350 |
| K01867 | 0.000 | 0.039 | 1 | 0.000 | 0.064 | 1.000 | 0.000 | 0.075 | 1.000 |
| K01868 | 0.036 | 0.058 | 0.35 | 0.060 | 0.079 | 0.280 | 0.068 | 0.109 | 0.330 |
| K01869 | 0.127 | 0.096 | 0.06 | 0.136 | 0.117 | 0.070 | 0.169 | 0.138 | 0.100 |
| K01870 | 0.040 | 0.057 | 0.29 | 0.070 | 0.098 | 0.320 | 0.082 | 0.091 | 0.190 |
| K01872 | 0.057 | 0.071 | 0.17 | 0.110 | 0.093 | 0.120 | 0.022 | 0.088 | 0.500 |
| K01873 | 0.115 | 0.085 | 0.12 | 0.121 | 0.102 | 0.110 | 0.128 | 0.134 | 0.210 |
| K01875 | 0.000 | 0.045 | 1 | 0.000 | 0.057 | 1.000 | 0.000 | 0.083 | 1.000 |
| K01880 | 0.083 | 0.064 | 0.08 | 0.121 | 0.110 | 0.080 | 0.032 | 0.080 | 0.500 |

| | | | | | | | | | |
|---|---|---|---|---|---|---|---|---|---|
| K01881 | 0.037 | 0.062 | 0.31 | 0.058 | 0.089 | 0.300 | 0.093 | 0.132 | 0.210 |
| K01883 | 0.009 | 0.040 | 0.47 | 0.061 | 0.090 | 0.280 | 0.000 | 0.069 | 1.000 |
| K01885 | 0.000 | 0.044 | 1 | 0.000 | 0.064 | 1.000 | 0.135 | 0.132 | 0.150 |
| K01886 | 0.042 | 0.064 | 0.31 | 0.061 | 0.079 | 0.280 | 0.000 | 0.081 | 0.490 |
| K01887 | 0.049 | 0.069 | 0.21 | 0.000 | 0.058 | 1.000 | 0.161 | 0.137 | 0.100 |
| K01889 | 0.000 | 0.036 | 1 | 0.000 | 0.048 | 1.000 | 0.000 | 0.092 | 1.000 |
| K01890 | 0.094 | 0.080 | 0.06 | 0.098 | 0.095 | 0.120 | 0.128 | 0.138 | 0.150 |
| K01892 | 0.000 | 0.041 | 1 | 0.000 | 0.056 | 1.000 | 0.000 | 0.075 | 1.000 |
| K01893 | 0.006 | 0.049 | 0.49 | 0.000 | 0.060 | 1.000 | 0.092 | 0.111 | 0.260 |
| K01910 | 0.000 | 0.053 | 1 | 0.000 | 0.056 | 1.000 | 0.000 | 0.057 | 1.000 |
| K01912 | 0.004 | 0.041 | 0.38 | 0.000 | 0.053 | 1.000 | 0.030 | 0.088 | 0.340 |
| K01914 | 0.108 | 0.088 | 0.05 | 0.150 | 0.114 | 0.090 | 0.000 | 0.000 | 0.550 |
| K01918 | 0.042 | 0.058 | 0.3 | 0.099 | 0.106 | 0.200 | 0.000 | 0.064 | 1.000 |
| K01919 | 0.000 | 0.044 | 1 | 0.000 | 0.064 | 1.000 | 0.000 | 0.000 | 0.350 |
| K01923 | 0.105 | 0.082 | 0.11 | 0.070 | 0.090 | 0.230 | 0.135 | 0.147 | 0.120 |
| K01924 | 0.000 | 0.042 | 1 | 0.000 | 0.066 | 1.000 | 0.000 | 0.081 | 1.000 |
| K01925 | 0.000 | 0.046 | 1 | 0.000 | 0.050 | 1.000 | 0.099 | 0.114 | 0.290 |
| K01928 | 0.084 | 0.074 | 0.09 | 0.006 | 0.061 | 0.500 | 0.183 | 0.149 | 0.070 |
| K01929 | 0.027 | 0.054 | 0.36 | 0.025 | 0.072 | 0.380 | 0.026 | 0.098 | 0.400 |
| K01933 | 0.068 | 0.075 | 0.21 | 0.049 | 0.071 | 0.360 | 0.100 | 0.129 | 0.280 |
| K01934 | 0.118 | 0.077 | 0.07 | 0.119 | 0.093 | 0.110 | 0.135 | 0.113 | 0.130 |
| K01935 | 0.000 | 0.046 | 1 | 0.035 | 0.070 | 0.380 | 0.000 | 0.071 | 1.000 |
| K01937 | 0.117 | 0.078 | 0.06 | 0.133 | 0.105 | 0.080 | 0.188 | 0.148 | 0.100 |
| K01939 | 0.044 | 0.062 | 0.27 | 0.013 | 0.061 | 0.450 | 0.098 | 0.115 | 0.170 |
| K01940 | 0.189 | 0.084 | 0.01 | 0.139 | 0.101 | 0.070 | 0.228 | 0.147 | 0.070 |
| K01945 | 0.112 | 0.085 | 0.08 | 0.035 | 0.081 | 0.440 | 0.238 | 0.153 | 0.070 |
| K01950 | 0.068 | 0.064 | 0.16 | 0.034 | 0.073 | 0.400 | 0.118 | 0.115 | 0.200 |
| K01951 | 0.016 | 0.049 | 0.46 | 0.000 | 0.050 | 1.000 | 0.056 | 0.093 | 0.300 |
| K01952 | 0.178 | 0.087 | 0.03 | 0.164 | 0.117 | 0.060 | 0.212 | 0.149 | 0.100 |
| K01956 | 0.102 | 0.091 | 0.08 | 0.021 | 0.077 | 0.440 | 0.191 | 0.135 | 0.090 |
| K01961 | 0.093 | 0.076 | 0.11 | 0.123 | 0.109 | 0.110 | 0.027 | 0.087 | 0.450 |
| K01962 | 0.000 | 0.034 | 1 | 0.000 | 0.058 | 1.000 | 0.000 | 0.080 | 1.000 |
| K01963 | 0.000 | 0.044 | 1 | 0.013 | 0.065 | 0.490 | 0.000 | 0.087 | 1.000 |
| K01966 | 0.028 | 0.048 | 0.36 | 0.082 | 0.088 | 0.260 | 0.000 | 0.071 | 1.000 |
| K01972 | 0.000 | 0.035 | 1 | 0.000 | 0.053 | 1.000 | 0.007 | 0.093 | 0.440 |
| K01975 | 0.040 | 0.061 | 0.26 | 0.000 | 0.060 | 1.000 | 0.185 | 0.149 | 0.080 |
| K01990 | 0.000 | 0.039 | 1 | 0.032 | 0.072 | 0.380 | 0.000 | 0.065 | 1.000 |
| K01991 | 0.052 | 0.064 | 0.29 | 0.066 | 0.087 | 0.220 | 0.000 | 0.083 | 1.000 |
| K01992 | 0.005 | 0.037 | 0.52 | 0.120 | 0.112 | 0.110 | 0.000 | 0.081 | 1.000 |
| K01993 | 0.063 | 0.070 | 0.25 | 0.089 | 0.098 | 0.200 | 0.023 | 0.088 | 0.320 |
| K01998 | 0.112 | 0.075 | 0.07 | 0.165 | 0.125 | 0.060 | 0.059 | 0.086 | 0.380 |
| K02003 | 0.021 | 0.051 | 0.47 | 0.000 | 0.063 | 1.000 | 0.070 | 0.093 | 0.340 |
| K02004 | 0.050 | 0.055 | 0.22 | 0.000 | 0.053 | 1.000 | 0.186 | 0.150 | 0.070 |
| K02005 | 0.075 | 0.072 | 0.15 | 0.071 | 0.092 | 0.210 | 0.074 | 0.113 | 0.330 |
| K02008 | 0.000 | 0.030 | 1 | 0.012 | 0.065 | 0.530 | 0.000 | 0.083 | 1.000 |
| K02009 | 0.119 | 0.086 | 0.06 | 0.165 | 0.106 | 0.060 | 0.019 | 0.077 | 0.480 |
| K02010 | 0.111 | 0.091 | 0.07 | 0.154 | 0.106 | 0.050 | 0.046 | 0.099 | 0.400 |
| K02013 | 0.029 | 0.055 | 0.34 | 0.085 | 0.100 | 0.200 | 0.000 | 0.077 | 1.000 |
| K02014 | 0.017 | 0.049 | 0.38 | 0.049 | 0.084 | 0.390 | 0.000 | 0.088 | 1.000 |
| K02015 | 0.065 | 0.059 | 0.13 | 0.100 | 0.107 | 0.200 | 0.019 | 0.091 | 0.390 |
| K02016 | 0.112 | 0.083 | 0.11 | 0.138 | 0.106 | 0.130 | 0.059 | 0.116 | 0.290 |
| K02018 | 0.009 | 0.052 | 0.53 | 0.047 | 0.089 | 0.330 | 0.000 | 0.000 | 0.140 |
| K02019 | 0.000 | 0.036 | 1 | 0.000 | 0.061 | 1.000 | 0.000 | 0.077 | 1.000 |

| | | | | | | | | | |
|---|---|---|---|---|---|---|---|---|---|
| K02030 | 0.089 | 0.086 | 0.14 | 0.175 | 0.112 | 0.050 | 0.046 | 0.107 | 0.380 |
| K02040 | 0.114 | 0.084 | 0.06 | 0.135 | 0.105 | 0.070 | 0.029 | 0.101 | 0.440 |
| K02049 | 0.000 | 0.043 | 1 | 0.000 | 0.054 | 1.000 | 0.080 | 0.102 | 0.250 |
| K02050 | 0.011 | 0.051 | 0.46 | 0.114 | 0.097 | 0.110 | 0.000 | 0.075 | 1.000 |
| K02051 | 0.042 | 0.062 | 0.21 | 0.000 | 0.064 | 1.000 | 0.135 | 0.142 | 0.120 |
| K02056 | 0.123 | 0.079 | 0.04 | 0.156 | 0.109 | 0.050 | 0.065 | 0.121 | 0.250 |
| K02065 | 0.079 | 0.072 | 0.07 | 0.086 | 0.083 | 0.180 | 0.017 | 0.092 | 0.470 |
| K02108 | 0.011 | 0.044 | 0.42 | 0.060 | 0.081 | 0.280 | 0.000 | 0.073 | 1.000 |
| K02109 | 0.085 | 0.076 | 0.11 | 0.118 | 0.107 | 0.110 | 0.000 | 0.000 | 0.480 |
| K02110 | 0.102 | 0.084 | 0.09 | 0.152 | 0.110 | 0.110 | 0.000 | 0.087 | 1.000 |
| K02111 | 0.128 | 0.091 | 0.05 | 0.100 | 0.089 | 0.150 | 0.168 | 0.150 | 0.090 |
| K02112 | 0.163 | 0.089 | 0.02 | 0.157 | 0.101 | 0.080 | 0.192 | 0.151 | 0.070 |
| K02113 | 0.065 | 0.070 | 0.22 | 0.109 | 0.098 | 0.120 | 0.000 | 0.079 | 1.000 |
| K02116 | 0.003 | 0.043 | 0.55 | 0.000 | 0.053 | 1.000 | 0.103 | 0.135 | 0.190 |
| K02117 | 0.055 | 0.063 | 0.28 | 0.081 | 0.091 | 0.290 | 0.036 | 0.096 | 0.350 |
| K02118 | 0.046 | 0.065 | 0.29 | 0.082 | 0.106 | 0.180 | 0.016 | 0.072 | 0.440 |
| K02119 | 0.012 | 0.059 | 0.53 | 0.082 | 0.094 | 0.230 | 0.000 | 0.059 | 1.000 |
| K02120 | 0.000 | 0.037 | 1 | 0.052 | 0.078 | 0.230 | 0.000 | 0.074 | 1.000 |
| K02121 | 0.000 | 0.033 | 1 | 0.008 | 0.047 | 0.490 | 0.000 | 0.083 | 1.000 |
| K02122 | 0.000 | 0.047 | 1 | 0.078 | 0.094 | 0.240 | 0.000 | 0.072 | 1.000 |
| K02123 | 0.000 | 0.036 | 1 | 0.052 | 0.082 | 0.300 | 0.000 | 0.075 | 1.000 |
| K02124 | 0.039 | 0.060 | 0.24 | 0.068 | 0.098 | 0.210 | 0.000 | 0.063 | 1.000 |
| K02160 | 0.000 | 0.034 | 1 | 0.048 | 0.074 | 0.380 | 0.000 | 0.069 | 1.000 |
| K02171 | 0.000 | 0.046 | 1 | 0.000 | 0.050 | 1.000 | 0.144 | 0.127 | 0.140 |
| K02172 | 0.000 | 0.042 | 1 | 0.000 | 0.051 | 1.000 | 0.000 | 0.087 | 1.000 |
| K02173 | 0.055 | 0.065 | 0.21 | 0.081 | 0.082 | 0.250 | 0.000 | 0.078 | 1.000 |
| K02188 | 0.024 | 0.053 | 0.29 | 0.041 | 0.077 | 0.250 | 0.000 | 0.074 | 1.000 |
| K02189 | 0.100 | 0.070 | 0.11 | 0.000 | 0.054 | 1.000 | 0.204 | 0.149 | 0.080 |
| K02190 | 0.024 | 0.055 | 0.34 | 0.074 | 0.093 | 0.260 | 0.000 | 0.071 | 1.000 |
| K02199 | 0.013 | 0.058 | 0.45 | 0.000 | 0.062 | 1.000 | 0.180 | 0.134 | 0.090 |
| K02203 | 0.000 | 0.039 | 1 | 0.080 | 0.079 | 0.220 | 0.000 | 0.076 | 1.000 |
| K02221 | 0.041 | 0.064 | 0.31 | 0.112 | 0.096 | 0.190 | 0.000 | 0.086 | 1.000 |
| K02224 | 0.019 | 0.054 | 0.48 | 0.001 | 0.060 | 0.570 | 0.036 | 0.100 | 0.440 |
| K02227 | 0.050 | 0.051 | 0.2 | 0.000 | 0.062 | 1.000 | 0.189 | 0.145 | 0.050 |
| K02232 | 0.090 | 0.078 | 0.12 | 0.024 | 0.065 | 0.430 | 0.177 | 0.129 | 0.050 |
| K02233 | 0.113 | 0.086 | 0.04 | 0.089 | 0.090 | 0.210 | 0.180 | 0.155 | 0.110 |
| K02238 | 0.000 | 0.037 | 1 | 0.000 | 0.059 | 1.000 | 0.000 | 0.078 | 1.000 |
| K02279 | 0.069 | 0.072 | 0.11 | 0.094 | 0.097 | 0.200 | 0.068 | 0.113 | 0.300 |
| K02283 | 0.030 | 0.055 | 0.32 | 0.084 | 0.095 | 0.180 | 0.000 | 0.075 | 1.000 |
| K02304 | 0.108 | 0.086 | 0.08 | 0.030 | 0.062 | 0.410 | 0.256 | 0.157 | 0.050 |
| K02313 | 0.160 | 0.077 | 0.02 | 0.114 | 0.095 | 0.100 | 0.235 | 0.144 | 0.070 |
| K02314 | 0.000 | 0.043 | 1 | 0.000 | 0.052 | 1.000 | 0.000 | 0.082 | 1.000 |
| K02316 | 0.000 | 0.042 | 1 | 0.000 | 0.046 | 1.000 | 0.111 | 0.137 | 0.240 |
| K02334 | 0.054 | 0.064 | 0.23 | 0.063 | 0.075 | 0.280 | 0.041 | 0.095 | 0.390 |
| K02335 | 0.104 | 0.080 | 0.12 | 0.147 | 0.107 | 0.080 | 0.109 | 0.123 | 0.140 |
| K02337 | 0.133 | 0.089 | 0.04 | 0.094 | 0.093 | 0.190 | 0.208 | 0.149 | 0.070 |
| K02338 | 0.000 | 0.045 | 1 | 0.019 | 0.070 | 0.530 | 0.000 | 0.077 | 1.000 |
| K02340 | 0.015 | 0.053 | 0.4 | 0.064 | 0.093 | 0.200 | 0.000 | 0.061 | 1.000 |
| K02341 | 0.073 | 0.067 | 0.18 | 0.151 | 0.109 | 0.100 | 0.000 | 0.000 | 0.400 |
| K02342 | 0.000 | 0.042 | 1 | 0.000 | 0.052 | 1.000 | 0.000 | 0.068 | 1.000 |
| K02343 | 0.044 | 0.059 | 0.23 | 0.000 | 0.061 | 1.000 | 0.136 | 0.147 | 0.110 |
| K02346 | 0.088 | 0.069 | 0.12 | 0.082 | 0.087 | 0.220 | 0.081 | 0.110 | 0.210 |
| K02348 | 0.067 | 0.068 | 0.12 | 0.066 | 0.086 | 0.250 | 0.057 | 0.093 | 0.280 |

| | | | | | | | | | |
|---|---|---|---|---|---|---|---|---|---|
| K02356 | 0.044 | 0.068 | 0.27 | 0.109 | 0.087 | 0.120 | 0.000 | 0.067 | 1.000 |
| K02357 | 0.010 | 0.050 | 0.36 | 0.000 | 0.052 | 1.000 | 0.073 | 0.115 | 0.310 |
| K02372 | 0.049 | 0.060 | 0.36 | 0.151 | 0.107 | 0.070 | 0.000 | 0.063 | 1.000 |
| K02377 | 0.155 | 0.087 | 0.03 | 0.166 | 0.106 | 0.050 | 0.080 | 0.111 | 0.160 |
| K02395 | 0.043 | 0.060 | 0.37 | 0.054 | 0.074 | 0.270 | 0.009 | 0.088 | 0.500 |
| K02398 | 0.063 | 0.065 | 0.21 | 0.043 | 0.085 | 0.390 | 0.111 | 0.108 | 0.220 |
| K02399 | 0.040 | 0.068 | 0.28 | 0.016 | 0.058 | 0.430 | 0.073 | 0.116 | 0.230 |
| K02408 | 0.019 | 0.050 | 0.4 | 0.014 | 0.062 | 0.520 | 0.045 | 0.084 | 0.350 |
| K02413 | 0.016 | 0.050 | 0.49 | 0.022 | 0.069 | 0.420 | 0.019 | 0.085 | 0.420 |
| K02420 | 0.054 | 0.063 | 0.25 | 0.062 | 0.087 | 0.260 | 0.038 | 0.091 | 0.390 |
| K02426 | 0.064 | 0.063 | 0.18 | 0.082 | 0.092 | 0.240 | 0.023 | 0.078 | 0.420 |
| K02427 | 0.000 | 0.044 | 1 | 0.000 | 0.060 | 1.000 | 0.005 | 0.095 | 0.570 |
| K02428 | 0.035 | 0.050 | 0.38 | 0.051 | 0.085 | 0.230 | 0.000 | 0.076 | 1.000 |
| K02429 | 0.017 | 0.052 | 0.39 | 0.054 | 0.072 | 0.370 | 0.000 | 0.067 | 1.000 |
| K02435 | 0.018 | 0.046 | 0.41 | 0.103 | 0.094 | 0.130 | 0.000 | 0.080 | 1.000 |
| K02437 | 0.110 | 0.076 | 0.1 | 0.093 | 0.087 | 0.150 | 0.145 | 0.137 | 0.120 |
| K02440 | 0.077 | 0.070 | 0.17 | 0.073 | 0.080 | 0.230 | 0.073 | 0.110 | 0.300 |
| K02456 | 0.003 | 0.045 | 0.56 | 0.066 | 0.074 | 0.200 | 0.000 | 0.070 | 1.000 |
| K02470 | 0.126 | 0.082 | 0.03 | 0.100 | 0.102 | 0.120 | 0.171 | 0.129 | 0.150 |
| K02477 | 0.019 | 0.057 | 0.49 | 0.011 | 0.055 | 0.400 | 0.068 | 0.097 | 0.300 |
| K02478 | 0.016 | 0.055 | 0.43 | 0.039 | 0.066 | 0.330 | 0.000 | 0.079 | 1.000 |
| K02492 | 0.052 | 0.060 | 0.18 | 0.084 | 0.097 | 0.210 | 0.009 | 0.083 | 0.520 |
| K02493 | 0.011 | 0.046 | 0.46 | 0.018 | 0.066 | 0.440 | 0.008 | 0.084 | 0.440 |
| K02495 | 0.042 | 0.064 | 0.25 | 0.081 | 0.096 | 0.210 | 0.000 | 0.076 | 1.000 |
| K02499 | 0.011 | 0.044 | 0.45 | 0.003 | 0.054 | 0.520 | 0.035 | 0.083 | 0.430 |
| K02501 | 0.091 | 0.070 | 0.13 | 0.000 | 0.057 | 1.000 | 0.210 | 0.134 | 0.090 |
| K02503 | 0.039 | 0.056 | 0.29 | 0.000 | 0.061 | 1.000 | 0.107 | 0.125 | 0.200 |
| K02517 | 0.084 | 0.078 | 0.15 | 0.171 | 0.114 | 0.060 | 0.000 | 0.083 | 1.000 |
| K02518 | 0.167 | 0.084 | 0.04 | 0.048 | 0.087 | 0.320 | 0.218 | 0.157 | 0.050 |
| K02519 | 0.154 | 0.079 | 0.04 | 0.145 | 0.117 | 0.070 | 0.191 | 0.148 | 0.060 |
| K02520 | 0.000 | 0.036 | 1 | 0.000 | 0.064 | 1.000 | 0.000 | 0.066 | 1.000 |
| K02527 | 0.115 | 0.078 | 0.09 | 0.157 | 0.119 | 0.070 | 0.000 | 0.000 | 0.710 |
| K02528 | 0.123 | 0.086 | 0.06 | 0.141 | 0.105 | 0.080 | 0.095 | 0.113 | 0.230 |
| K02536 | 0.135 | 0.092 | 0.04 | 0.116 | 0.095 | 0.160 | 0.156 | 0.136 | 0.160 |
| K02548 | 0.060 | 0.069 | 0.21 | 0.095 | 0.082 | 0.150 | 0.000 | 0.086 | 1.000 |
| K02549 | 0.113 | 0.090 | 0.07 | 0.138 | 0.110 | 0.130 | 0.045 | 0.087 | 0.340 |
| K02551 | 0.124 | 0.085 | 0.09 | 0.114 | 0.096 | 0.080 | 0.151 | 0.143 | 0.080 |
| K02563 | 0.026 | 0.051 | 0.42 | 0.000 | 0.054 | 1.000 | 0.114 | 0.111 | 0.280 |
| K02564 | 0.085 | 0.080 | 0.11 | 0.076 | 0.088 | 0.200 | 0.109 | 0.124 | 0.180 |
| K02574 | 0.058 | 0.063 | 0.29 | 0.092 | 0.103 | 0.180 | 0.000 | 0.078 | 1.000 |
| K02585 | 0.000 | 0.039 | 1 | 0.000 | 0.060 | 1.000 | 0.124 | 0.125 | 0.120 |
| K02600 | 0.048 | 0.064 | 0.3 | 0.053 | 0.077 | 0.360 | 0.059 | 0.112 | 0.350 |
| K02601 | 0.000 | 0.044 | 1 | 0.000 | 0.050 | 1.000 | 0.077 | 0.115 | 0.360 |
| K02614 | 0.016 | 0.043 | 0.42 | 0.008 | 0.065 | 0.510 | 0.015 | 0.085 | 0.490 |
| K02626 | 0.093 | 0.069 | 0.11 | 0.124 | 0.101 | 0.080 | 0.000 | 0.079 | 1.000 |
| K02651 | 0.064 | 0.065 | 0.22 | 0.044 | 0.084 | 0.350 | 0.144 | 0.144 | 0.130 |
| K02662 | 0.000 | 0.046 | 1 | 0.000 | 0.049 | 1.000 | 0.000 | 0.079 | 1.000 |
| K02687 | 0.049 | 0.069 | 0.27 | 0.000 | 0.055 | 1.000 | 0.244 | 0.157 | 0.050 |
| K02775 | 0.142 | 0.087 | 0.04 | 0.149 | 0.127 | 0.090 | 0.142 | 0.142 | 0.170 |
| K02784 | 0.072 | 0.064 | 0.18 | 0.170 | 0.117 | 0.060 | 0.000 | 0.063 | 1.000 |
| K02805 | 0.066 | 0.067 | 0.18 | 0.000 | 0.065 | 1.000 | 0.165 | 0.129 | 0.220 |
| K02822 | 0.186 | 0.096 | 0.02 | 0.183 | 0.121 | 0.050 | 0.199 | 0.139 | 0.050 |
| K02823 | 0.038 | 0.055 | 0.3 | 0.020 | 0.070 | 0.420 | 0.045 | 0.093 | 0.380 |

| | | | | | | | | | |
|---|---|---|---|---|---|---|---|---|---|
| K02824 | 0.086 | 0.082 | 0.11 | 0.047 | 0.074 | 0.310 | 0.167 | 0.143 | 0.090 |
| K02825 | 0.013 | 0.046 | 0.48 | 0.086 | 0.089 | 0.140 | 0.000 | 0.088 | 1.000 |
| K02834 | 0.085 | 0.069 | 0.14 | 0.000 | 0.055 | 1.000 | 0.159 | 0.144 | 0.060 |
| K02835 | 0.000 | 0.046 | 1 | 0.027 | 0.069 | 0.430 | 0.000 | 0.090 | 1.000 |
| K02836 | 0.155 | 0.084 | 0.03 | 0.184 | 0.123 | 0.050 | 0.125 | 0.135 | 0.180 |
| K02838 | 0.170 | 0.088 | 0.02 | 0.163 | 0.123 | 0.080 | 0.170 | 0.136 | 0.110 |
| K02847 | 0.000 | 0.000 | 0.53 | 0.000 | 0.049 | 1.000 | 0.041 | 0.084 | 0.360 |
| K02851 | 0.049 | 0.056 | 0.3 | 0.045 | 0.077 | 0.300 | 0.046 | 0.078 | 0.450 |
| K02856 | 0.019 | 0.053 | 0.48 | 0.077 | 0.093 | 0.270 | 0.000 | 0.075 | 1.000 |
| K02860 | 0.021 | 0.047 | 0.37 | 0.065 | 0.091 | 0.290 | 0.000 | 0.092 | 1.000 |
| K02863 | 0.042 | 0.059 | 0.27 | 0.086 | 0.095 | 0.110 | 0.012 | 0.080 | 0.530 |
| K02864 | 0.000 | 0.035 | 1 | 0.000 | 0.059 | 1.000 | 0.000 | 0.075 | 1.000 |
| K02867 | 0.067 | 0.068 | 0.19 | 0.110 | 0.095 | 0.100 | 0.010 | 0.077 | 0.560 |
| K02871 | 0.000 | 0.034 | 1 | 0.000 | 0.047 | 1.000 | 0.000 | 0.079 | 1.000 |
| K02874 | 0.109 | 0.087 | 0.06 | 0.000 | 0.064 | 1.000 | 0.247 | 0.163 | 0.060 |
| K02876 | 0.016 | 0.057 | 0.47 | 0.027 | 0.064 | 0.350 | 0.003 | 0.088 | 0.520 |
| K02878 | 0.031 | 0.055 | 0.42 | 0.066 | 0.085 | 0.270 | 0.000 | 0.091 | 1.000 |
| K02879 | 0.125 | 0.086 | 0.07 | 0.123 | 0.103 | 0.150 | 0.126 | 0.124 | 0.160 |
| K02881 | 0.023 | 0.049 | 0.46 | 0.000 | 0.057 | 1.000 | 0.112 | 0.110 | 0.220 |
| K02886 | 0.000 | 0.039 | 1 | 0.004 | 0.063 | 0.530 | 0.000 | 0.071 | 1.000 |
| K02887 | 0.000 | 0.041 | 1 | 0.000 | 0.054 | 1.000 | 0.056 | 0.095 | 0.360 |
| K02888 | 0.046 | 0.060 | 0.29 | 0.000 | 0.055 | 1.000 | 0.135 | 0.137 | 0.210 |
| K02890 | 0.000 | 0.043 | 1 | 0.000 | 0.052 | 1.000 | 0.065 | 0.095 | 0.270 |
| K02892 | 0.091 | 0.080 | 0.09 | 0.000 | 0.067 | 1.000 | 0.197 | 0.151 | 0.080 |
| K02895 | 0.068 | 0.057 | 0.09 | 0.000 | 0.049 | 1.000 | 0.133 | 0.139 | 0.180 |
| K02897 | 0.055 | 0.065 | 0.2 | 0.047 | 0.083 | 0.300 | 0.064 | 0.100 | 0.330 |
| K02899 | 0.136 | 0.086 | 0.07 | 0.036 | 0.061 | 0.380 | 0.222 | 0.147 | 0.060 |
| K02902 | 0.127 | 0.085 | 0.07 | 0.000 | 0.050 | 1.000 | 0.240 | 0.139 | 0.050 |
| K02904 | 0.093 | 0.071 | 0.1 | 0.136 | 0.118 | 0.060 | 0.052 | 0.086 | 0.370 |
| K02906 | 0.069 | 0.066 | 0.22 | 0.121 | 0.110 | 0.110 | 0.086 | 0.103 | 0.230 |
| K02907 | 0.123 | 0.080 | 0.05 | 0.001 | 0.059 | 0.530 | 0.196 | 0.147 | 0.070 |
| K02909 | 0.077 | 0.069 | 0.16 | 0.000 | 0.061 | 1.000 | 0.166 | 0.129 | 0.090 |
| K02919 | 0.115 | 0.089 | 0.03 | 0.084 | 0.092 | 0.250 | 0.144 | 0.132 | 0.120 |
| K02926 | 0.000 | 0.043 | 1 | 0.000 | 0.050 | 1.000 | 0.000 | 0.066 | 1.000 |
| K02931 | 0.000 | 0.045 | 1 | 0.000 | 0.048 | 1.000 | 0.000 | 0.000 | 0.950 |
| K02933 | 0.000 | 0.046 | 1 | 0.000 | 0.062 | 1.000 | 0.000 | 0.072 | 1.000 |
| K02935 | 0.038 | 0.051 | 0.35 | 0.096 | 0.098 | 0.220 | 0.000 | 0.079 | 1.000 |
| K02939 | 0.030 | 0.055 | 0.32 | 0.049 | 0.077 | 0.330 | 0.007 | 0.086 | 0.540 |
| K02946 | 0.038 | 0.045 | 0.3 | 0.000 | 0.062 | 1.000 | 0.074 | 0.097 | 0.310 |
| K02948 | 0.070 | 0.061 | 0.12 | 0.062 | 0.084 | 0.280 | 0.075 | 0.112 | 0.420 |
| K02950 | 0.000 | 0.037 | 1 | 0.008 | 0.067 | 0.520 | 0.000 | 0.064 | 1.000 |
| K02952 | 0.000 | 0.050 | 1 | 0.000 | 0.050 | 1.000 | 0.000 | 0.000 | 0.530 |
| K02956 | 0.115 | 0.085 | 0.09 | 0.009 | 0.063 | 0.510 | 0.184 | 0.151 | 0.060 |
| K02959 | 0.057 | 0.077 | 0.25 | 0.000 | 0.056 | 1.000 | 0.146 | 0.144 | 0.110 |
| K02961 | 0.078 | 0.075 | 0.16 | 0.000 | 0.062 | 1.000 | 0.145 | 0.142 | 0.100 |
| K02963 | 0.102 | 0.076 | 0.13 | 0.000 | 0.051 | 1.000 | 0.185 | 0.161 | 0.090 |
| K02967 | 0.007 | 0.061 | 0.56 | 0.000 | 0.062 | 1.000 | 0.033 | 0.103 | 0.410 |
| K02968 | 0.123 | 0.084 | 0.07 | 0.062 | 0.061 | 0.180 | 0.155 | 0.119 | 0.150 |
| K02970 | 0.091 | 0.081 | 0.11 | 0.062 | 0.073 | 0.250 | 0.102 | 0.100 | 0.140 |
| K02982 | 0.004 | 0.044 | 0.45 | 0.000 | 0.058 | 1.000 | 0.079 | 0.112 | 0.240 |
| K02986 | 0.000 | 0.042 | 1 | 0.000 | 0.057 | 1.000 | 0.000 | 0.084 | 1.000 |
| K02988 | 0.000 | 0.041 | 1 | 0.000 | 0.048 | 1.000 | 0.037 | 0.104 | 0.400 |
| K02992 | 0.006 | 0.057 | 0.46 | 0.062 | 0.075 | 0.270 | 0.000 | 0.069 | 1.000 |

| | | | | | | | | | |
|---|---|---|---|---|---|---|---|---|---|
| K02994 | 0.010 | 0.053 | 0.49 | 0.022 | 0.067 | 0.520 | 0.005 | 0.078 | 0.510 |
| K03040 | 0.000 | 0.042 | 1 | 0.054 | 0.094 | 0.250 | 0.000 | 0.088 | 1.000 |
| K03043 | 0.070 | 0.067 | 0.22 | 0.086 | 0.096 | 0.180 | 0.101 | 0.138 | 0.280 |
| K03049 | 0.065 | 0.068 | 0.17 | 0.057 | 0.096 | 0.250 | 0.055 | 0.089 | 0.310 |
| K03050 | 0.074 | 0.070 | 0.23 | 0.070 | 0.084 | 0.260 | 0.066 | 0.118 | 0.280 |
| K03051 | 0.094 | 0.080 | 0.11 | 0.131 | 0.102 | 0.130 | 0.033 | 0.087 | 0.450 |
| K03053 | 0.093 | 0.070 | 0.08 | 0.150 | 0.115 | 0.110 | 0.000 | 0.074 | 1.000 |
| K03057 | 0.121 | 0.079 | 0.04 | 0.177 | 0.134 | 0.050 | 0.012 | 0.077 | 0.490 |
| K03060 | 0.000 | 0.045 | 1 | 0.027 | 0.071 | 0.490 | 0.000 | 0.066 | 1.000 |
| K03071 | 0.016 | 0.049 | 0.49 | 0.023 | 0.080 | 0.440 | 0.000 | 0.084 | 1.000 |
| K03073 | 0.019 | 0.047 | 0.43 | 0.003 | 0.062 | 0.580 | 0.028 | 0.080 | 0.420 |
| K03075 | 0.102 | 0.080 | 0.05 | 0.032 | 0.070 | 0.370 | 0.170 | 0.133 | 0.090 |
| K03076 | 0.131 | 0.081 | 0.07 | 0.136 | 0.111 | 0.110 | 0.128 | 0.133 | 0.210 |
| K03077 | 0.067 | 0.069 | 0.21 | 0.040 | 0.069 | 0.360 | 0.104 | 0.122 | 0.200 |
| K03088 | 0.000 | 0.038 | 1 | 0.000 | 0.057 | 1.000 | 0.031 | 0.081 | 0.390 |
| K03091 | 0.000 | 0.042 | 1 | 0.000 | 0.053 | 1.000 | 0.000 | 0.077 | 1.000 |
| K03092 | 0.071 | 0.068 | 0.18 | 0.113 | 0.097 | 0.140 | 0.000 | 0.075 | 1.000 |
| K03100 | 0.096 | 0.079 | 0.06 | 0.127 | 0.101 | 0.090 | 0.048 | 0.089 | 0.410 |
| K03101 | 0.027 | 0.059 | 0.34 | 0.101 | 0.102 | 0.140 | 0.000 | 0.080 | 1.000 |
| K03110 | 0.000 | 0.042 | 1 | 0.000 | 0.051 | 1.000 | 0.159 | 0.124 | 0.060 |
| K03111 | 0.000 | 0.045 | 1 | 0.000 | 0.047 | 1.000 | 0.000 | 0.075 | 1.000 |
| K03116 | 0.085 | 0.073 | 0.15 | 0.068 | 0.078 | 0.230 | 0.147 | 0.136 | 0.150 |
| K03118 | 0.020 | 0.054 | 0.33 | 0.014 | 0.067 | 0.490 | 0.033 | 0.081 | 0.420 |
| K03147 | 0.134 | 0.094 | 0.04 | 0.157 | 0.111 | 0.050 | 0.182 | 0.134 | 0.070 |
| K03150 | 0.000 | 0.044 | 1 | 0.051 | 0.071 | 0.340 | 0.000 | 0.073 | 1.000 |
| K03151 | 0.060 | 0.066 | 0.26 | 0.034 | 0.069 | 0.400 | 0.122 | 0.123 | 0.140 |
| K03152 | 0.024 | 0.049 | 0.44 | 0.010 | 0.065 | 0.470 | 0.040 | 0.089 | 0.380 |
| K03154 | 0.141 | 0.086 | 0.04 | 0.107 | 0.104 | 0.100 | 0.202 | 0.150 | 0.070 |
| K03168 | 0.012 | 0.050 | 0.52 | 0.000 | 0.064 | 0.540 | 0.048 | 0.090 | 0.420 |
| K03169 | 0.079 | 0.074 | 0.12 | 0.009 | 0.060 | 0.500 | 0.235 | 0.157 | 0.050 |
| K03177 | 0.000 | 0.047 | 1 | 0.000 | 0.052 | 1.000 | 0.000 | 0.073 | 1.000 |
| K03186 | 0.039 | 0.058 | 0.29 | 0.056 | 0.073 | 0.300 | 0.000 | 0.070 | 1.000 |
| K03210 | 0.083 | 0.069 | 0.1 | 0.000 | 0.051 | 1.000 | 0.162 | 0.147 | 0.080 |
| K03216 | 0.000 | 0.047 | 1 | 0.036 | 0.076 | 0.430 | 0.000 | 0.067 | 1.000 |
| K03269 | 0.149 | 0.093 | 0.01 | 0.188 | 0.128 | 0.090 | 0.076 | 0.120 | 0.270 |
| K03270 | 0.151 | 0.089 | 0.05 | 0.145 | 0.113 | 0.060 | 0.168 | 0.120 | 0.080 |
| K03271 | 0.097 | 0.078 | 0.12 | 0.059 | 0.087 | 0.320 | 0.161 | 0.143 | 0.110 |
| K03273 | 0.065 | 0.069 | 0.22 | 0.058 | 0.086 | 0.230 | 0.071 | 0.103 | 0.260 |
| K03274 | 0.014 | 0.056 | 0.52 | 0.022 | 0.067 | 0.420 | 0.009 | 0.089 | 0.570 |
| K03281 | 0.000 | 0.038 | 1 | 0.050 | 0.090 | 0.310 | 0.000 | 0.074 | 1.000 |
| K03282 | 0.108 | 0.081 | 0.06 | 0.105 | 0.100 | 0.160 | 0.085 | 0.102 | 0.290 |
| K03284 | 0.093 | 0.068 | 0.08 | 0.124 | 0.113 | 0.120 | 0.059 | 0.107 | 0.350 |
| K03286 | 0.121 | 0.086 | 0.08 | 0.119 | 0.103 | 0.110 | 0.092 | 0.121 | 0.280 |
| K03297 | 0.000 | 0.043 | 1 | 0.000 | 0.053 | 1.000 | 0.015 | 0.081 | 0.440 |
| K03299 | 0.047 | 0.064 | 0.28 | 0.074 | 0.093 | 0.240 | 0.000 | 0.078 | 1.000 |
| K03303 | 0.052 | 0.064 | 0.34 | 0.000 | 0.062 | 1.000 | 0.222 | 0.159 | 0.050 |
| K03305 | 0.026 | 0.057 | 0.4 | 0.034 | 0.079 | 0.310 | 0.005 | 0.069 | 0.460 |
| K03307 | 0.034 | 0.056 | 0.36 | 0.087 | 0.099 | 0.170 | 0.000 | 0.066 | 1.000 |
| K03308 | 0.100 | 0.075 | 0.1 | 0.075 | 0.099 | 0.150 | 0.122 | 0.117 | 0.200 |
| K03310 | 0.128 | 0.082 | 0.06 | 0.101 | 0.091 | 0.210 | 0.180 | 0.132 | 0.080 |
| K03315 | 0.102 | 0.074 | 0.08 | 0.153 | 0.115 | 0.050 | 0.000 | 0.083 | 1.000 |
| K03320 | 0.152 | 0.081 | 0.02 | 0.116 | 0.092 | 0.130 | 0.190 | 0.150 | 0.060 |
| K03321 | 0.008 | 0.049 | 0.46 | 0.002 | 0.056 | 0.500 | 0.001 | 0.057 | 0.480 |

| | | | | | | | | | |
|---|---|---|---|---|---|---|---|---|---|
| K03324 | 0.039 | 0.057 | 0.37 | 0.020 | 0.067 | 0.440 | 0.067 | 0.102 | 0.330 |
| K03332 | 0.000 | 0.037 | 1 | 0.052 | 0.077 | 0.250 | 0.000 | 0.068 | 1.000 |
| K03340 | 0.031 | 0.055 | 0.33 | 0.109 | 0.108 | 0.090 | 0.000 | 0.071 | 1.000 |
| K03385 | 0.025 | 0.056 | 0.28 | 0.000 | 0.050 | 1.000 | 0.066 | 0.092 | 0.320 |
| K03402 | 0.025 | 0.053 | 0.39 | 0.024 | 0.065 | 0.420 | 0.019 | 0.072 | 0.420 |
| K03422 | 0.108 | 0.081 | 0.08 | 0.155 | 0.108 | 0.060 | 0.000 | 0.085 | 1.000 |
| K03424 | 0.000 | 0.042 | 1 | 0.000 | 0.060 | 1.000 | 0.000 | 0.067 | 1.000 |
| K03426 | 0.165 | 0.094 | 0.04 | 0.143 | 0.104 | 0.100 | 0.181 | 0.141 | 0.100 |
| K03427 | 0.032 | 0.063 | 0.3 | 0.024 | 0.074 | 0.350 | 0.039 | 0.099 | 0.440 |
| K03429 | 0.006 | 0.037 | 0.55 | 0.072 | 0.086 | 0.150 | 0.000 | 0.081 | 1.000 |
| K03437 | 0.000 | 0.045 | 1 | 0.000 | 0.049 | 1.000 | 0.000 | 0.073 | 1.000 |
| K03438 | 0.000 | 0.041 | 1 | 0.000 | 0.059 | 1.000 | 0.000 | 0.070 | 1.000 |
| K03439 | 0.000 | 0.037 | 1 | 0.000 | 0.054 | 1.000 | 0.000 | 0.071 | 1.000 |
| K03442 | 0.017 | 0.047 | 0.41 | 0.053 | 0.086 | 0.330 | 0.000 | 0.056 | 1.000 |
| K03455 | 0.012 | 0.046 | 0.45 | 0.029 | 0.064 | 0.370 | 0.000 | 0.070 | 1.000 |
| K03465 | 0.000 | 0.040 | 1 | 0.031 | 0.063 | 0.360 | 0.000 | 0.083 | 1.000 |
| K03466 | 0.025 | 0.051 | 0.35 | 0.000 | 0.057 | 1.000 | 0.082 | 0.126 | 0.310 |
| K03469 | 0.000 | 0.042 | 1 | 0.000 | 0.065 | 1.000 | 0.000 | 0.073 | 1.000 |
| K03470 | 0.031 | 0.057 | 0.3 | 0.074 | 0.093 | 0.180 | 0.000 | 0.068 | 1.000 |
| K03495 | 0.085 | 0.078 | 0.17 | 0.168 | 0.119 | 0.060 | 0.000 | 0.000 | 0.270 |
| K03497 | 0.100 | 0.073 | 0.09 | 0.125 | 0.109 | 0.100 | 0.074 | 0.105 | 0.330 |
| K03498 | 0.004 | 0.046 | 0.43 | 0.000 | 0.068 | 1.000 | 0.219 | 0.169 | 0.060 |
| K03500 | 0.064 | 0.071 | 0.29 | 0.112 | 0.094 | 0.130 | 0.000 | 0.062 | 1.000 |
| K03501 | 0.060 | 0.071 | 0.21 | 0.115 | 0.104 | 0.160 | 0.000 | 0.077 | 1.000 |
| K03502 | 0.052 | 0.064 | 0.21 | 0.087 | 0.102 | 0.300 | 0.055 | 0.091 | 0.310 |
| K03517 | 0.000 | 0.044 | 1 | 0.000 | 0.054 | 1.000 | 0.000 | 0.076 | 1.000 |
| K03521 | 0.123 | 0.087 | 0.07 | 0.125 | 0.107 | 0.070 | 0.176 | 0.140 | 0.100 |
| K03522 | 0.129 | 0.086 | 0.06 | 0.119 | 0.090 | 0.090 | 0.147 | 0.130 | 0.080 |
| K03523 | 0.056 | 0.066 | 0.16 | 0.126 | 0.105 | 0.060 | 0.000 | 0.000 | 0.980 |
| K03524 | 0.055 | 0.065 | 0.13 | 0.014 | 0.059 | 0.500 | 0.132 | 0.134 | 0.080 |
| K03525 | 0.000 | 0.045 | 1 | 0.000 | 0.067 | 1.000 | 0.030 | 0.095 | 0.540 |
| K03526 | 0.041 | 0.057 | 0.25 | 0.017 | 0.067 | 0.450 | 0.156 | 0.135 | 0.130 |
| K03527 | 0.104 | 0.073 | 0.1 | 0.157 | 0.100 | 0.080 | 0.000 | 0.067 | 1.000 |
| K03530 | 0.065 | 0.067 | 0.17 | 0.034 | 0.074 | 0.300 | 0.087 | 0.100 | 0.260 |
| K03531 | 0.000 | 0.041 | 1 | 0.000 | 0.057 | 1.000 | 0.056 | 0.107 | 0.400 |
| K03536 | 0.056 | 0.069 | 0.19 | 0.067 | 0.103 | 0.240 | 0.039 | 0.100 | 0.490 |
| K03544 | 0.000 | 0.034 | 1 | 0.000 | 0.037 | 1.000 | 0.000 | 0.073 | 1.000 |
| K03545 | 0.074 | 0.066 | 0.2 | 0.040 | 0.078 | 0.290 | 0.138 | 0.127 | 0.120 |
| K03546 | 0.048 | 0.063 | 0.25 | 0.123 | 0.115 | 0.100 | 0.000 | 0.070 | 1.000 |
| K03547 | 0.051 | 0.068 | 0.26 | 0.173 | 0.120 | 0.050 | 0.000 | 0.077 | 1.000 |
| K03550 | 0.000 | 0.046 | 1 | 0.000 | 0.044 | 1.000 | 0.000 | 0.082 | 1.000 |
| K03551 | 0.027 | 0.057 | 0.36 | 0.007 | 0.067 | 0.490 | 0.069 | 0.115 | 0.360 |
| K03552 | 0.084 | 0.077 | 0.16 | 0.118 | 0.102 | 0.120 | 0.036 | 0.091 | 0.400 |
| K03553 | 0.000 | 0.050 | 1 | 0.004 | 0.058 | 0.470 | 0.000 | 0.075 | 1.000 |
| K03558 | 0.008 | 0.045 | 0.56 | 0.042 | 0.073 | 0.380 | 0.000 | 0.058 | 1.000 |
| K03561 | 0.000 | 0.046 | 1 | 0.022 | 0.055 | 0.380 | 0.000 | 0.070 | 1.000 |
| K03565 | 0.073 | 0.060 | 0.2 | 0.055 | 0.085 | 0.240 | 0.101 | 0.107 | 0.190 |
| K03568 | 0.047 | 0.063 | 0.21 | 0.112 | 0.090 | 0.100 | 0.000 | 0.079 | 1.000 |
| K03569 | 0.067 | 0.074 | 0.18 | 0.114 | 0.107 | 0.180 | 0.000 | 0.000 | 0.930 |
| K03570 | 0.007 | 0.039 | 0.48 | 0.021 | 0.065 | 0.470 | 0.000 | 0.078 | 1.000 |
| K03571 | 0.000 | 0.046 | 1 | 0.044 | 0.074 | 0.360 | 0.000 | 0.075 | 1.000 |
| K03572 | 0.000 | 0.039 | 1 | 0.000 | 0.053 | 1.000 | 0.000 | 0.093 | 1.000 |
| K03573 | 0.047 | 0.056 | 0.27 | 0.071 | 0.081 | 0.240 | 0.006 | 0.074 | 0.520 |

| | | | | | | | | | |
|---|---|---|---|---|---|---|---|---|---|
| K03574 | 0.145 | 0.084 | 0.02 | 0.165 | 0.126 | 0.070 | 0.103 | 0.105 | 0.250 |
| K03575 | 0.023 | 0.053 | 0.45 | 0.000 | 0.052 | 1.000 | 0.154 | 0.129 | 0.110 |
| K03584 | 0.000 | 0.050 | 1 | 0.065 | 0.092 | 0.220 | 0.000 | 0.058 | 1.000 |
| K03587 | 0.000 | 0.045 | 1 | 0.000 | 0.054 | 1.000 | 0.000 | 0.081 | 1.000 |
| K03588 | 0.062 | 0.071 | 0.27 | 0.076 | 0.087 | 0.220 | 0.097 | 0.121 | 0.240 |
| K03589 | 0.000 | 0.043 | 1 | 0.000 | 0.055 | 1.000 | 0.008 | 0.069 | 0.480 |
| K03590 | 0.000 | 0.041 | 1 | 0.000 | 0.057 | 1.000 | 0.000 | 0.060 | 1.000 |
| K03593 | 0.000 | 0.045 | 1 | 0.000 | 0.055 | 1.000 | 0.000 | 0.066 | 1.000 |
| K03595 | 0.033 | 0.051 | 0.32 | 0.084 | 0.096 | 0.240 | 0.000 | 0.060 | 1.000 |
| K03602 | 0.025 | 0.055 | 0.38 | 0.000 | 0.061 | 1.000 | 0.125 | 0.132 | 0.090 |
| K03606 | 0.117 | 0.082 | 0.04 | 0.187 | 0.114 | 0.060 | 0.006 | 0.080 | 0.540 |
| K03608 | 0.022 | 0.055 | 0.41 | 0.000 | 0.062 | 1.000 | 0.077 | 0.095 | 0.180 |
| K03609 | 0.004 | 0.051 | 0.45 | 0.000 | 0.062 | 1.000 | 0.030 | 0.108 | 0.480 |
| K03610 | 0.003 | 0.044 | 0.51 | 0.015 | 0.070 | 0.420 | 0.000 | 0.086 | 1.000 |
| K03612 | 0.105 | 0.069 | 0.11 | 0.043 | 0.075 | 0.380 | 0.191 | 0.138 | 0.090 |
| K03613 | 0.027 | 0.052 | 0.28 | 0.018 | 0.064 | 0.450 | 0.037 | 0.090 | 0.390 |
| K03614 | 0.000 | 0.043 | 1 | 0.000 | 0.053 | 1.000 | 0.011 | 0.071 | 0.540 |
| K03615 | 0.000 | 0.037 | 1 | 0.000 | 0.057 | 1.000 | 0.070 | 0.113 | 0.290 |
| K03616 | 0.049 | 0.057 | 0.24 | 0.064 | 0.093 | 0.230 | 0.017 | 0.092 | 0.470 |
| K03617 | 0.000 | 0.032 | 1 | 0.000 | 0.057 | 1.000 | 0.047 | 0.093 | 0.340 |
| K03621 | 0.000 | 0.053 | 1 | 0.098 | 0.098 | 0.100 | 0.000 | 0.076 | 1.000 |
| K03623 | 0.095 | 0.073 | 0.11 | 0.090 | 0.090 | 0.190 | 0.089 | 0.127 | 0.200 |
| K03624 | 0.050 | 0.064 | 0.23 | 0.039 | 0.076 | 0.350 | 0.056 | 0.100 | 0.370 |
| K03625 | 0.005 | 0.058 | 0.51 | 0.000 | 0.075 | 1.000 | 0.069 | 0.101 | 0.350 |
| K03628 | 0.000 | 0.045 | 1 | 0.076 | 0.081 | 0.280 | 0.000 | 0.061 | 1.000 |
| K03629 | 0.113 | 0.078 | 0.05 | 0.107 | 0.103 | 0.100 | 0.183 | 0.155 | 0.110 |
| K03631 | 0.073 | 0.073 | 0.18 | 0.085 | 0.094 | 0.190 | 0.055 | 0.094 | 0.370 |
| K03637 | 0.000 | 0.038 | 1 | 0.055 | 0.077 | 0.280 | 0.000 | 0.073 | 1.000 |
| K03638 | 0.192 | 0.084 | 0.02 | 0.162 | 0.099 | 0.050 | 0.218 | 0.149 | 0.100 |
| K03640 | 0.070 | 0.072 | 0.15 | 0.085 | 0.093 | 0.200 | 0.000 | 0.000 | 0.890 |
| K03642 | 0.047 | 0.058 | 0.22 | 0.041 | 0.083 | 0.340 | 0.066 | 0.096 | 0.290 |
| K03643 | 0.091 | 0.075 | 0.07 | 0.110 | 0.103 | 0.210 | 0.021 | 0.097 | 0.430 |
| K03646 | 0.000 | 0.036 | 1 | 0.000 | 0.054 | 1.000 | 0.000 | 0.066 | 1.000 |
| K03648 | 0.000 | 0.051 | 1 | 0.000 | 0.062 | 1.000 | 0.000 | 0.082 | 1.000 |
| K03651 | 0.006 | 0.043 | 0.42 | 0.016 | 0.069 | 0.470 | 0.000 | 0.000 | 0.680 |
| K03654 | 0.000 | 0.047 | 1 | 0.000 | 0.046 | 1.000 | 0.000 | 0.073 | 1.000 |
| K03655 | 0.075 | 0.071 | 0.12 | 0.151 | 0.107 | 0.070 | 0.010 | 0.071 | 0.520 |
| K03657 | 0.113 | 0.074 | 0.08 | 0.171 | 0.120 | 0.110 | 0.121 | 0.128 | 0.220 |
| K03658 | 0.038 | 0.073 | 0.33 | 0.103 | 0.096 | 0.080 | 0.000 | 0.077 | 1.000 |
| K03664 | 0.000 | 0.044 | 1 | 0.000 | 0.056 | 1.000 | 0.050 | 0.096 | 0.300 |
| K03665 | 0.067 | 0.067 | 0.3 | 0.000 | 0.065 | 1.000 | 0.160 | 0.131 | 0.080 |
| K03666 | 0.054 | 0.068 | 0.24 | 0.040 | 0.071 | 0.370 | 0.059 | 0.108 | 0.320 |
| K03667 | 0.079 | 0.070 | 0.13 | 0.133 | 0.105 | 0.130 | 0.000 | 0.085 | 1.000 |
| K03671 | 0.065 | 0.068 | 0.18 | 0.000 | 0.066 | 1.000 | 0.150 | 0.146 | 0.110 |
| K03684 | 0.000 | 0.040 | 1 | 0.000 | 0.062 | 1.000 | 0.000 | 0.073 | 1.000 |
| K03685 | 0.150 | 0.090 | 0.02 | 0.080 | 0.089 | 0.270 | 0.232 | 0.152 | 0.060 |
| K03686 | 0.000 | 0.056 | 1 | 0.000 | 0.056 | 1.000 | 0.000 | 0.065 | 1.000 |
| K03687 | 0.058 | 0.061 | 0.19 | 0.137 | 0.106 | 0.090 | 0.000 | 0.077 | 1.000 |
| K03695 | 0.032 | 0.053 | 0.4 | 0.088 | 0.098 | 0.210 | 0.019 | 0.072 | 0.490 |
| K03696 | 0.070 | 0.063 | 0.2 | 0.135 | 0.104 | 0.090 | 0.008 | 0.069 | 0.430 |
| K03699 | 0.098 | 0.077 | 0.13 | 0.113 | 0.105 | 0.120 | 0.070 | 0.121 | 0.280 |
| K03701 | 0.087 | 0.074 | 0.12 | 0.104 | 0.098 | 0.110 | 0.073 | 0.112 | 0.290 |
| K03704 | 0.111 | 0.074 | 0.1 | 0.182 | 0.110 | 0.050 | 0.000 | 0.085 | 1.000 |

| | | | | | | | | | |
|---|---|---|---|---|---|---|---|---|---|
| K03706 | 0.048 | 0.061 | 0.33 | 0.016 | 0.056 | 0.450 | 0.098 | 0.110 | 0.280 |
| K03708 | 0.000 | 0.053 | 1 | 0.074 | 0.100 | 0.200 | 0.000 | 0.089 | 1.000 |
| K03718 | 0.056 | 0.063 | 0.16 | 0.053 | 0.068 | 0.360 | 0.087 | 0.111 | 0.230 |
| K03719 | 0.110 | 0.074 | 0.06 | 0.159 | 0.098 | 0.060 | 0.000 | 0.075 | 1.000 |
| K03722 | 0.042 | 0.058 | 0.34 | 0.075 | 0.086 | 0.250 | 0.000 | 0.080 | 1.000 |
| K03723 | 0.099 | 0.075 | 0.03 | 0.015 | 0.073 | 0.390 | 0.232 | 0.157 | 0.070 |
| K03742 | 0.000 | 0.035 | 1 | 0.020 | 0.062 | 0.480 | 0.000 | 0.089 | 1.000 |
| K03744 | 0.000 | 0.038 | 1 | 0.000 | 0.063 | 1.000 | 0.132 | 0.138 | 0.210 |
| K03750 | 0.133 | 0.088 | 0.04 | 0.119 | 0.099 | 0.140 | 0.255 | 0.163 | 0.050 |
| K03752 | 0.016 | 0.050 | 0.34 | 0.052 | 0.080 | 0.310 | 0.000 | 0.061 | 1.000 |
| K03768 | 0.000 | 0.051 | 1 | 0.000 | 0.061 | 1.000 | 0.000 | 0.074 | 1.000 |
| K03770 | 0.019 | 0.043 | 0.37 | 0.101 | 0.106 | 0.180 | 0.000 | 0.078 | 1.000 |
| K03771 | 0.115 | 0.084 | 0.07 | 0.101 | 0.100 | 0.150 | 0.150 | 0.124 | 0.110 |
| K03772 | 0.054 | 0.066 | 0.21 | 0.075 | 0.081 | 0.280 | 0.021 | 0.098 | 0.510 |
| K03775 | 0.003 | 0.042 | 0.44 | 0.000 | 0.065 | 1.000 | 0.151 | 0.156 | 0.130 |
| K03780 | 0.000 | 0.000 | 1 | 0.000 | 0.000 | 0.180 | 0.029 | 0.083 | 0.410 |
| K03783 | 0.014 | 0.045 | 0.45 | 0.042 | 0.076 | 0.360 | 0.000 | 0.066 | 1.000 |
| K03784 | 0.066 | 0.071 | 0.24 | 0.005 | 0.064 | 0.530 | 0.178 | 0.152 | 0.100 |
| K03785 | 0.000 | 0.039 | 1 | 0.040 | 0.067 | 0.300 | 0.000 | 0.077 | 1.000 |
| K03786 | 0.000 | 0.052 | 0.48 | 0.000 | 0.000 | 0.540 | 0.000 | 0.072 | 1.000 |
| K03787 | 0.012 | 0.040 | 0.47 | 0.000 | 0.062 | 1.000 | 0.071 | 0.110 | 0.360 |
| K03789 | 0.000 | 0.042 | 1 | 0.081 | 0.097 | 0.170 | 0.000 | 0.071 | 1.000 |
| K03790 | 0.006 | 0.050 | 0.5 | 0.000 | 0.063 | 1.000 | 0.040 | 0.104 | 0.400 |
| K03797 | 0.000 | 0.046 | 1 | 0.063 | 0.081 | 0.230 | 0.000 | 0.080 | 1.000 |
| K03799 | 0.000 | 0.044 | 1 | 0.000 | 0.053 | 1.000 | 0.000 | 0.066 | 1.000 |
| K03803 | 0.120 | 0.087 | 0.04 | 0.089 | 0.105 | 0.140 | 0.157 | 0.141 | 0.100 |
| K03811 | 0.146 | 0.099 | 0.08 | 0.159 | 0.104 | 0.080 | 0.076 | 0.099 | 0.280 |
| K03814 | 0.015 | 0.049 | 0.43 | 0.031 | 0.076 | 0.390 | 0.000 | 0.000 | 0.130 |
| K03820 | 0.121 | 0.086 | 0.04 | 0.165 | 0.115 | 0.050 | 0.061 | 0.119 | 0.340 |
| K03824 | 0.090 | 0.079 | 0.11 | 0.012 | 0.065 | 0.430 | 0.179 | 0.130 | 0.050 |
| K03828 | 0.152 | 0.083 | 0.05 | 0.193 | 0.123 | 0.050 | 0.094 | 0.120 | 0.230 |
| K03830 | 0.000 | 0.037 | 1 | 0.000 | 0.052 | 1.000 | 0.000 | 0.099 | 1.000 |
| K03832 | 0.086 | 0.069 | 0.09 | 0.068 | 0.073 | 0.230 | 0.128 | 0.133 | 0.080 |
| K03856 | 0.013 | 0.046 | 0.4 | 0.127 | 0.114 | 0.110 | 0.000 | 0.083 | 1.000 |
| K03925 | 0.049 | 0.059 | 0.28 | 0.016 | 0.067 | 0.520 | 0.092 | 0.117 | 0.210 |
| K03930 | 0.024 | 0.048 | 0.35 | 0.102 | 0.083 | 0.130 | 0.000 | 0.078 | 1.000 |
| K03932 | 0.032 | 0.056 | 0.42 | 0.005 | 0.053 | 0.560 | 0.105 | 0.118 | 0.270 |
| K03972 | 0.148 | 0.089 | 0.04 | 0.131 | 0.105 | 0.100 | 0.177 | 0.136 | 0.070 |
| K03975 | 0.102 | 0.068 | 0.11 | 0.113 | 0.108 | 0.120 | 0.109 | 0.134 | 0.200 |
| K03976 | 0.000 | 0.036 | 1 | 0.000 | 0.059 | 1.000 | 0.000 | 0.076 | 1.000 |
| K03977 | 0.114 | 0.073 | 0.06 | 0.171 | 0.104 | 0.050 | 0.075 | 0.110 | 0.240 |
| K03978 | 0.066 | 0.069 | 0.28 | 0.113 | 0.109 | 0.140 | 0.000 | 0.072 | 1.000 |
| K03979 | 0.000 | 0.045 | 1 | 0.000 | 0.000 | 0.790 | 0.000 | 0.000 | 0.110 |
| K04041 | 0.072 | 0.071 | 0.21 | 0.083 | 0.092 | 0.200 | 0.034 | 0.094 | 0.390 |
| K04042 | 0.063 | 0.070 | 0.16 | 0.144 | 0.121 | 0.100 | 0.000 | 0.080 | 1.000 |
| K04066 | 0.013 | 0.049 | 0.43 | 0.028 | 0.082 | 0.410 | 0.012 | 0.091 | 0.480 |
| K04068 | 0.000 | 0.037 | 1 | 0.000 | 0.065 | 1.000 | 0.000 | 0.078 | 1.000 |
| K04069 | 0.095 | 0.083 | 0.07 | 0.187 | 0.109 | 0.050 | 0.000 | 0.072 | 1.000 |
| K04072 | 0.132 | 0.092 | 0.06 | 0.158 | 0.110 | 0.050 | 0.112 | 0.118 | 0.180 |
| K04074 | 0.020 | 0.051 | 0.48 | 0.143 | 0.111 | 0.110 | 0.000 | 0.074 | 1.000 |
| K04075 | 0.029 | 0.057 | 0.35 | 0.038 | 0.079 | 0.200 | 0.028 | 0.101 | 0.440 |
| K04077 | 0.000 | 0.036 | 1 | 0.000 | 0.056 | 1.000 | 0.045 | 0.098 | 0.450 |
| K04079 | 0.000 | 0.040 | 1 | 0.068 | 0.078 | 0.300 | 0.000 | 0.067 | 1.000 |

| | | | | | | | | | |
|---|---|---|---|---|---|---|---|---|---|
| K04083 | 0.075 | 0.074 | 0.18 | 0.179 | 0.110 | 0.100 | 0.000 | 0.089 | 1.000 |
| K04087 | 0.047 | 0.065 | 0.32 | 0.058 | 0.084 | 0.290 | 0.000 | 0.069 | 1.000 |
| K04088 | 0.039 | 0.059 | 0.26 | 0.041 | 0.062 | 0.280 | 0.007 | 0.070 | 0.460 |
| K04094 | 0.040 | 0.066 | 0.36 | 0.131 | 0.120 | 0.070 | 0.002 | 0.077 | 0.650 |
| K04095 | 0.058 | 0.063 | 0.21 | 0.086 | 0.107 | 0.250 | 0.003 | 0.076 | 0.570 |
| K04096 | 0.027 | 0.056 | 0.3 | 0.088 | 0.093 | 0.190 | 0.000 | 0.000 | 0.800 |
| K04485 | 0.031 | 0.053 | 0.35 | 0.022 | 0.069 | 0.520 | 0.083 | 0.100 | 0.210 |
| K04488 | 0.036 | 0.056 | 0.35 | 0.129 | 0.107 | 0.080 | 0.000 | 0.065 | 1.000 |
| K04516 | 0.000 | 0.051 | 1 | 0.000 | 0.059 | 1.000 | 0.000 | 0.079 | 1.000 |
| K04517 | 0.000 | 0.041 | 1 | 0.045 | 0.074 | 0.310 | 0.000 | 0.076 | 1.000 |
| K04567 | 0.069 | 0.070 | 0.11 | 0.096 | 0.086 | 0.100 | 0.068 | 0.120 | 0.330 |
| K04651 | 0.000 | 0.035 | 1 | 0.000 | 0.058 | 1.000 | 0.000 | 0.071 | 1.000 |
| K04652 | 0.000 | 0.036 | 1 | 0.000 | 0.056 | 1.000 | 0.000 | 0.062 | 1.000 |
| K04653 | 0.000 | 0.040 | 1 | 0.010 | 0.060 | 0.500 | 0.000 | 0.066 | 1.000 |
| K04654 | 0.027 | 0.048 | 0.35 | 0.043 | 0.063 | 0.210 | 0.003 | 0.096 | 0.540 |
| K04655 | 0.031 | 0.067 | 0.32 | 0.068 | 0.085 | 0.260 | 0.000 | 0.076 | 1.000 |
| K04656 | 0.034 | 0.068 | 0.32 | 0.063 | 0.084 | 0.200 | 0.009 | 0.078 | 0.560 |
| K04744 | 0.000 | 0.042 | 1 | 0.000 | 0.055 | 1.000 | 0.000 | 0.073 | 1.000 |
| K04749 | 0.076 | 0.076 | 0.11 | 0.057 | 0.085 | 0.310 | 0.160 | 0.140 | 0.100 |
| K04757 | 0.063 | 0.069 | 0.19 | 0.056 | 0.084 | 0.280 | 0.060 | 0.123 | 0.340 |
| K04758 | 0.000 | 0.034 | 1 | 0.086 | 0.079 | 0.150 | 0.000 | 0.084 | 1.000 |
| K04762 | 0.108 | 0.072 | 0.04 | 0.064 | 0.083 | 0.300 | 0.125 | 0.121 | 0.110 |
| K04763 | 0.011 | 0.046 | 0.47 | 0.011 | 0.051 | 0.550 | 0.013 | 0.084 | 0.490 |
| K04769 | 0.000 | 0.047 | 1 | 0.000 | 0.000 | 0.630 | 0.000 | 0.056 | 1.000 |
| K04771 | 0.012 | 0.054 | 0.48 | 0.000 | 0.066 | 1.000 | 0.079 | 0.136 | 0.270 |
| K04773 | 0.084 | 0.067 | 0.09 | 0.128 | 0.110 | 0.090 | 0.000 | 0.064 | 1.000 |
| K04794 | 0.037 | 0.069 | 0.33 | 0.000 | 0.058 | 1.000 | 0.069 | 0.100 | 0.290 |
| K04940 | 0.000 | 0.039 | 1 | 0.000 | 0.045 | 1.000 | 0.000 | 0.092 | 1.000 |
| K05275 | 0.052 | 0.061 | 0.27 | 0.000 | 0.060 | 1.000 | 0.200 | 0.138 | 0.060 |
| K05303 | 0.000 | 0.040 | 1 | 0.000 | 0.056 | 1.000 | 0.000 | 0.075 | 1.000 |
| K05305 | 0.120 | 0.089 | 0.04 | 0.098 | 0.082 | 0.120 | 0.200 | 0.138 | 0.060 |
| K05341 | 0.074 | 0.068 | 0.24 | 0.150 | 0.106 | 0.050 | 0.009 | 0.092 | 0.450 |
| K05349 | 0.073 | 0.064 | 0.14 | 0.049 | 0.083 | 0.290 | 0.093 | 0.125 | 0.300 |
| K05352 | 0.076 | 0.079 | 0.17 | 0.047 | 0.071 | 0.340 | 0.105 | 0.120 | 0.230 |
| K05364 | 0.088 | 0.076 | 0.16 | 0.175 | 0.109 | 0.080 | 0.000 | 0.082 | 1.000 |
| K05366 | 0.009 | 0.045 | 0.51 | 0.026 | 0.061 | 0.440 | 0.000 | 0.072 | 1.000 |
| K05515 | 0.083 | 0.072 | 0.13 | 0.133 | 0.109 | 0.060 | 0.008 | 0.090 | 0.530 |
| K05521 | 0.015 | 0.053 | 0.46 | 0.000 | 0.047 | 1.000 | 0.052 | 0.091 | 0.410 |
| K05540 | 0.057 | 0.065 | 0.22 | 0.009 | 0.064 | 0.470 | 0.195 | 0.142 | 0.060 |
| K05566 | 0.018 | 0.056 | 0.44 | 0.000 | 0.058 | 1.000 | 0.174 | 0.140 | 0.090 |
| K05568 | 0.082 | 0.079 | 0.15 | 0.016 | 0.061 | 0.470 | 0.179 | 0.151 | 0.100 |
| K05569 | 0.002 | 0.046 | 0.51 | 0.004 | 0.052 | 0.520 | 0.005 | 0.071 | 0.520 |
| K05592 | 0.103 | 0.081 | 0.06 | 0.164 | 0.121 | 0.070 | 0.029 | 0.075 | 0.490 |
| K05601 | 0.020 | 0.051 | 0.45 | 0.174 | 0.117 | 0.070 | 0.000 | 0.085 | 1.000 |
| K05606 | 0.085 | 0.074 | 0.15 | 0.071 | 0.078 | 0.220 | 0.139 | 0.122 | 0.110 |
| K05770 | 0.000 | 0.050 | 1 | 0.053 | 0.078 | 0.310 | 0.000 | 0.063 | 1.000 |
| K05776 | 0.056 | 0.071 | 0.18 | 0.106 | 0.092 | 0.090 | 0.000 | 0.063 | 1.000 |
| K05795 | 0.109 | 0.073 | 0.1 | 0.185 | 0.105 | 0.050 | 0.000 | 0.073 | 1.000 |
| K05799 | 0.089 | 0.071 | 0.16 | 0.114 | 0.096 | 0.120 | 0.047 | 0.100 | 0.300 |
| K05807 | 0.098 | 0.095 | 0.12 | 0.097 | 0.099 | 0.150 | 0.108 | 0.134 | 0.190 |
| K05808 | 0.031 | 0.068 | 0.41 | 0.157 | 0.110 | 0.080 | 0.000 | 0.053 | 1.000 |
| K05810 | 0.123 | 0.087 | 0.06 | 0.020 | 0.072 | 0.410 | 0.218 | 0.148 | 0.050 |
| K05825 | 0.069 | 0.075 | 0.23 | 0.099 | 0.096 | 0.120 | 0.042 | 0.101 | 0.290 |

| | | | | | | | | | |
|---|---|---|---|---|---|---|---|---|---|
| K05837 | 0.017 | 0.051 | 0.43 | 0.007 | 0.062 | 0.520 | 0.027 | 0.097 | 0.400 |
| K05873 | 0.010 | 0.052 | 0.47 | 0.031 | 0.067 | 0.430 | 0.000 | 0.073 | 1.000 |
| K05879 | 0.016 | 0.051 | 0.43 | 0.000 | 0.000 | 0.700 | 0.161 | 0.133 | 0.080 |
| K05895 | 0.140 | 0.092 | 0.05 | 0.157 | 0.098 | 0.070 | 0.181 | 0.127 | 0.120 |
| K05896 | 0.007 | 0.046 | 0.56 | 0.162 | 0.111 | 0.070 | 0.000 | 0.083 | 1.000 |
| K05934 | 0.000 | 0.047 | 1 | 0.024 | 0.062 | 0.440 | 0.000 | 0.000 | 0.520 |
| K05936 | 0.002 | 0.048 | 0.42 | 0.000 | 0.055 | 1.000 | 0.071 | 0.111 | 0.280 |
| K05937 | 0.086 | 0.074 | 0.04 | 0.051 | 0.088 | 0.300 | 0.110 | 0.128 | 0.170 |
| K05946 | 0.000 | 0.050 | 1 | 0.022 | 0.065 | 0.410 | 0.000 | 0.089 | 1.000 |
| K05970 | 0.056 | 0.061 | 0.31 | 0.079 | 0.093 | 0.210 | 0.001 | 0.078 | 0.520 |
| K05979 | 0.016 | 0.052 | 0.47 | 0.000 | 0.058 | 1.000 | 0.070 | 0.104 | 0.300 |
| K05985 | 0.079 | 0.076 | 0.15 | 0.193 | 0.117 | 0.050 | 0.000 | 0.073 | 1.000 |
| K05989 | 0.077 | 0.070 | 0.16 | 0.126 | 0.101 | 0.060 | 0.002 | 0.073 | 0.590 |
| K06001 | 0.000 | 0.040 | 1 | 0.000 | 0.059 | 1.000 | 0.000 | 0.088 | 1.000 |
| K06006 | 0.035 | 0.063 | 0.27 | 0.059 | 0.084 | 0.230 | 0.000 | 0.081 | 1.000 |
| K06012 | 0.032 | 0.055 | 0.38 | 0.038 | 0.082 | 0.290 | 0.034 | 0.086 | 0.470 |
| K06024 | 0.020 | 0.049 | 0.53 | 0.151 | 0.118 | 0.070 | 0.000 | 0.063 | 1.000 |
| K06076 | 0.075 | 0.082 | 0.16 | 0.146 | 0.098 | 0.080 | 0.000 | 0.078 | 1.000 |
| K06113 | 0.064 | 0.072 | 0.22 | 0.060 | 0.090 | 0.270 | 0.063 | 0.105 | 0.290 |
| K06131 | 0.108 | 0.082 | 0.09 | 0.098 | 0.105 | 0.190 | 0.138 | 0.137 | 0.120 |
| K06133 | 0.002 | 0.049 | 0.57 | 0.000 | 0.064 | 1.000 | 0.054 | 0.101 | 0.340 |
| K06142 | 0.050 | 0.066 | 0.3 | 0.060 | 0.080 | 0.300 | 0.020 | 0.098 | 0.420 |
| K06153 | 0.051 | 0.062 | 0.26 | 0.074 | 0.094 | 0.240 | 0.018 | 0.089 | 0.440 |
| K06158 | 0.069 | 0.069 | 0.21 | 0.106 | 0.108 | 0.110 | 0.041 | 0.084 | 0.460 |
| K06167 | 0.036 | 0.062 | 0.41 | 0.085 | 0.098 | 0.160 | 0.000 | 0.062 | 1.000 |
| K06168 | 0.093 | 0.084 | 0.11 | 0.106 | 0.079 | 0.090 | 0.058 | 0.100 | 0.330 |
| K06173 | 0.033 | 0.059 | 0.35 | 0.039 | 0.061 | 0.410 | 0.046 | 0.104 | 0.400 |
| K06178 | 0.000 | 0.042 | 1 | 0.000 | 0.053 | 1.000 | 0.000 | 0.074 | 1.000 |
| K06180 | 0.037 | 0.060 | 0.22 | 0.006 | 0.073 | 0.480 | 0.078 | 0.112 | 0.350 |
| K06183 | 0.055 | 0.067 | 0.31 | 0.070 | 0.080 | 0.210 | 0.018 | 0.081 | 0.390 |
| K06187 | 0.000 | 0.042 | 1 | 0.000 | 0.061 | 1.000 | 0.013 | 0.079 | 0.540 |
| K06188 | 0.037 | 0.063 | 0.32 | 0.061 | 0.089 | 0.260 | 0.000 | 0.079 | 1.000 |
| K06198 | 0.000 | 0.045 | 1 | 0.000 | 0.053 | 1.000 | 0.000 | 0.091 | 1.000 |
| K06199 | 0.122 | 0.089 | 0.06 | 0.170 | 0.127 | 0.090 | 0.000 | 0.076 | 1.000 |
| K06200 | 0.018 | 0.054 | 0.54 | 0.000 | 0.062 | 1.000 | 0.145 | 0.135 | 0.150 |
| K06206 | 0.009 | 0.047 | 0.48 | 0.000 | 0.055 | 1.000 | 0.115 | 0.116 | 0.230 |
| K06209 | 0.071 | 0.069 | 0.12 | 0.129 | 0.107 | 0.090 | 0.000 | 0.059 | 1.000 |
| K06217 | 0.000 | 0.041 | 1 | 0.000 | 0.049 | 1.000 | 0.000 | 0.076 | 1.000 |
| K06218 | 0.097 | 0.077 | 0.13 | 0.067 | 0.079 | 0.220 | 0.135 | 0.131 | 0.150 |
| K06221 | 0.025 | 0.053 | 0.44 | 0.000 | 0.058 | 1.000 | 0.152 | 0.125 | 0.130 |
| K06223 | 0.000 | 0.033 | 1 | 0.000 | 0.056 | 1.000 | 0.000 | 0.075 | 1.000 |
| K06283 | 0.000 | 0.043 | 1 | 0.036 | 0.069 | 0.410 | 0.000 | 0.079 | 1.000 |
| K06284 | 0.000 | 0.000 | 0.87 | 0.000 | 0.066 | 1.000 | 0.000 | 0.078 | 1.000 |
| K06287 | 0.032 | 0.054 | 0.36 | 0.066 | 0.079 | 0.260 | 0.009 | 0.074 | 0.570 |
| K06295 | 0.066 | 0.071 | 0.16 | 0.057 | 0.077 | 0.280 | 0.093 | 0.118 | 0.190 |
| K06298 | 0.064 | 0.070 | 0.14 | 0.156 | 0.113 | 0.070 | 0.000 | 0.068 | 1.000 |
| K06317 | 0.000 | 0.046 | 1 | 0.021 | 0.071 | 0.500 | 0.000 | 0.072 | 1.000 |
| K06330 | 0.000 | 0.047 | 1 | 0.000 | 0.059 | 1.000 | 0.108 | 0.119 | 0.220 |
| K06333 | 0.000 | 0.042 | 1 | 0.054 | 0.080 | 0.300 | 0.000 | 0.066 | 1.000 |
| K06373 | 0.000 | 0.042 | 1 | 0.084 | 0.088 | 0.170 | 0.000 | 0.072 | 1.000 |
| K06374 | 0.000 | 0.056 | 1 | 0.085 | 0.100 | 0.160 | 0.000 | 0.074 | 1.000 |
| K06381 | 0.000 | 0.000 | 0.7 | 0.000 | 0.052 | 1.000 | 0.008 | 0.074 | 0.480 |
| K06382 | 0.036 | 0.044 | 0.36 | 0.051 | 0.085 | 0.300 | 0.009 | 0.074 | 0.620 |

| | | | | | | | | | |
|---|---|---|---|---|---|---|---|---|---|
| K06383 | 0.000 | 0.000 | 0.83 | 0.034 | 0.067 | 0.410 | 0.000 | 0.076 | 1.000 |
| K06384 | 0.137 | 0.080 | 0.05 | 0.112 | 0.109 | 0.140 | 0.170 | 0.164 | 0.110 |
| K06385 | 0.026 | 0.050 | 0.38 | 0.097 | 0.088 | 0.140 | 0.000 | 0.066 | 1.000 |
| K06387 | 0.008 | 0.041 | 0.49 | 0.067 | 0.081 | 0.210 | 0.000 | 0.078 | 1.000 |
| K06390 | 0.054 | 0.068 | 0.21 | 0.112 | 0.106 | 0.120 | 0.000 | 0.066 | 1.000 |
| K06391 | 0.009 | 0.043 | 0.48 | 0.033 | 0.069 | 0.400 | 0.000 | 0.078 | 1.000 |
| K06392 | 0.006 | 0.043 | 0.43 | 0.051 | 0.078 | 0.320 | 0.000 | 0.071 | 1.000 |
| K06393 | 0.023 | 0.049 | 0.47 | 0.097 | 0.085 | 0.150 | 0.000 | 0.068 | 1.000 |
| K06396 | 0.003 | 0.042 | 0.49 | 0.061 | 0.081 | 0.280 | 0.000 | 0.076 | 1.000 |
| K06397 | 0.015 | 0.051 | 0.39 | 0.064 | 0.090 | 0.280 | 0.000 | 0.085 | 1.000 |
| K06398 | 0.057 | 0.065 | 0.18 | 0.112 | 0.104 | 0.170 | 0.010 | 0.080 | 0.450 |
| K06399 | 0.063 | 0.073 | 0.14 | 0.130 | 0.103 | 0.120 | 0.000 | 0.078 | 1.000 |
| K06400 | 0.138 | 0.093 | 0.02 | 0.135 | 0.102 | 0.080 | 0.164 | 0.155 | 0.070 |
| K06405 | 0.013 | 0.049 | 0.55 | 0.046 | 0.073 | 0.270 | 0.000 | 0.068 | 1.000 |
| K06406 | 0.000 | 0.041 | 1 | 0.000 | 0.059 | 1.000 | 0.000 | 0.077 | 1.000 |
| K06407 | 0.000 | 0.043 | 1 | 0.054 | 0.079 | 0.270 | 0.000 | 0.087 | 1.000 |
| K06409 | 0.117 | 0.081 | 0.07 | 0.205 | 0.121 | 0.050 | 0.037 | 0.085 | 0.370 |
| K06410 | 0.020 | 0.051 | 0.38 | 0.070 | 0.087 | 0.240 | 0.000 | 0.078 | 1.000 |
| K06411 | 0.017 | 0.056 | 0.41 | 0.056 | 0.082 | 0.290 | 0.000 | 0.075 | 1.000 |
| K06416 | 0.116 | 0.082 | 0.04 | 0.040 | 0.089 | 0.290 | 0.199 | 0.138 | 0.090 |
| K06421 | 0.016 | 0.049 | 0.41 | 0.018 | 0.063 | 0.440 | 0.007 | 0.085 | 0.480 |
| K06438 | 0.043 | 0.056 | 0.32 | 0.111 | 0.096 | 0.110 | 0.000 | 0.077 | 1.000 |
| K06518 | 0.044 | 0.065 | 0.25 | 0.129 | 0.109 | 0.100 | 0.000 | 0.065 | 1.000 |
| K06610 | 0.043 | 0.056 | 0.31 | 0.066 | 0.092 | 0.230 | 0.000 | 0.075 | 1.000 |
| K06864 | 0.000 | 0.040 | 1 | 0.000 | 0.058 | 1.000 | 0.000 | 0.071 | 1.000 |
| K06871 | 0.109 | 0.087 | 0.1 | 0.126 | 0.097 | 0.160 | 0.040 | 0.091 | 0.350 |
| K06872 | 0.000 | 0.040 | 1 | 0.000 | 0.068 | 1.000 | 0.050 | 0.095 | 0.360 |
| K06877 | 0.017 | 0.052 | 0.36 | 0.004 | 0.056 | 0.450 | 0.033 | 0.083 | 0.450 |
| K06881 | 0.000 | 0.043 | 1 | 0.000 | 0.062 | 1.000 | 0.000 | 0.086 | 1.000 |
| K06889 | 0.000 | 0.037 | 1 | 0.000 | 0.061 | 1.000 | 0.000 | 0.080 | 1.000 |
| K06890 | 0.110 | 0.082 | 0.1 | 0.128 | 0.101 | 0.130 | 0.051 | 0.097 | 0.340 |
| K06891 | 0.068 | 0.074 | 0.2 | 0.057 | 0.081 | 0.250 | 0.120 | 0.129 | 0.180 |
| K06894 | 0.090 | 0.067 | 0.14 | 0.119 | 0.114 | 0.120 | 0.034 | 0.093 | 0.430 |
| K06897 | 0.052 | 0.068 | 0.24 | 0.024 | 0.072 | 0.390 | 0.177 | 0.140 | 0.110 |
| K06898 | 0.035 | 0.061 | 0.3 | 0.095 | 0.104 | 0.180 | 0.000 | 0.000 | 0.740 |
| K06901 | 0.026 | 0.049 | 0.4 | 0.000 | 0.058 | 1.000 | 0.149 | 0.133 | 0.100 |
| K06904 | 0.000 | 0.043 | 1 | 0.000 | 0.053 | 1.000 | 0.000 | 0.000 | 0.520 |
| K06909 | 0.000 | 0.038 | 1 | 0.000 | 0.061 | 1.000 | 0.000 | 0.083 | 1.000 |
| K06915 | 0.000 | 0.047 | 1 | 0.003 | 0.062 | 0.570 | 0.000 | 0.071 | 1.000 |
| K06920 | 0.091 | 0.086 | 0.1 | 0.099 | 0.097 | 0.150 | 0.041 | 0.096 | 0.380 |
| K06921 | 0.059 | 0.061 | 0.19 | 0.090 | 0.088 | 0.210 | 0.000 | 0.076 | 1.000 |
| K06923 | 0.045 | 0.062 | 0.27 | 0.138 | 0.104 | 0.070 | 0.000 | 0.091 | 1.000 |
| K06925 | 0.033 | 0.064 | 0.32 | 0.049 | 0.070 | 0.350 | 0.023 | 0.096 | 0.350 |
| K06926 | 0.000 | 0.051 | 1 | 0.000 | 0.057 | 1.000 | 0.000 | 0.081 | 1.000 |
| K06934 | 0.070 | 0.072 | 0.18 | 0.085 | 0.081 | 0.220 | 0.031 | 0.091 | 0.410 |
| K06940 | 0.068 | 0.071 | 0.19 | 0.146 | 0.105 | 0.130 | 0.000 | 0.055 | 1.000 |
| K06941 | 0.000 | 0.050 | 1 | 0.000 | 0.044 | 1.000 | 0.014 | 0.103 | 0.370 |
| K06942 | 0.000 | 0.044 | 1 | 0.000 | 0.054 | 1.000 | 0.000 | 0.065 | 1.000 |
| K06949 | 0.000 | 0.034 | 1 | 0.000 | 0.057 | 1.000 | 0.000 | 0.081 | 1.000 |
| K06950 | 0.000 | 0.042 | 1 | 0.000 | 0.043 | 1.000 | 0.068 | 0.116 | 0.300 |
| K06952 | 0.015 | 0.057 | 0.46 | 0.115 | 0.109 | 0.130 | 0.000 | 0.089 | 1.000 |
| K06958 | 0.015 | 0.054 | 0.52 | 0.099 | 0.082 | 0.130 | 0.000 | 0.069 | 1.000 |
| K06959 | 0.107 | 0.090 | 0.03 | 0.025 | 0.081 | 0.430 | 0.211 | 0.158 | 0.100 |

| | | | | | | | | | |
|---|---|---|---|---|---|---|---|---|---|
| K06960 | 0.045 | 0.058 | 0.27 | 0.071 | 0.091 | 0.210 | 0.024 | 0.079 | 0.440 |
| K06969 | 0.017 | 0.055 | 0.39 | 0.081 | 0.078 | 0.200 | 0.000 | 0.000 | 0.610 |
| K06973 | 0.130 | 0.073 | 0.04 | 0.104 | 0.095 | 0.210 | 0.183 | 0.130 | 0.100 |
| K06975 | 0.097 | 0.073 | 0.07 | 0.101 | 0.092 | 0.140 | 0.070 | 0.101 | 0.260 |
| K06976 | 0.000 | 0.039 | 1 | 0.000 | 0.053 | 1.000 | 0.000 | 0.080 | 1.000 |
| K06985 | 0.113 | 0.086 | 0.04 | 0.099 | 0.106 | 0.110 | 0.140 | 0.134 | 0.140 |
| K06990 | 0.061 | 0.070 | 0.21 | 0.136 | 0.119 | 0.150 | 0.000 | 0.070 | 1.000 |
| K06997 | 0.000 | 0.041 | 1 | 0.043 | 0.072 | 0.360 | 0.000 | 0.065 | 1.000 |
| K07000 | 0.123 | 0.071 | 0.04 | 0.158 | 0.108 | 0.050 | 0.055 | 0.110 | 0.280 |
| K07001 | 0.000 | 0.042 | 1 | 0.000 | 0.049 | 1.000 | 0.000 | 0.066 | 1.000 |
| K07003 | 0.049 | 0.065 | 0.28 | 0.005 | 0.056 | 0.480 | 0.095 | 0.126 | 0.250 |
| K07010 | 0.041 | 0.054 | 0.27 | 0.108 | 0.112 | 0.090 | 0.000 | 0.069 | 1.000 |
| K07012 | 0.109 | 0.077 | 0.06 | 0.174 | 0.121 | 0.070 | 0.000 | 0.053 | 1.000 |
| K07023 | 0.023 | 0.061 | 0.36 | 0.137 | 0.096 | 0.070 | 0.000 | 0.073 | 1.000 |
| K07025 | 0.141 | 0.089 | 0.02 | 0.097 | 0.095 | 0.150 | 0.225 | 0.155 | 0.070 |
| K07027 | 0.010 | 0.043 | 0.38 | 0.000 | 0.051 | 1.000 | 0.055 | 0.093 | 0.400 |
| K07029 | 0.000 | 0.045 | 1 | 0.000 | 0.050 | 1.000 | 0.005 | 0.082 | 0.540 |
| K07031 | 0.162 | 0.099 | 0.02 | 0.112 | 0.107 | 0.200 | 0.228 | 0.146 | 0.050 |
| K07035 | 0.000 | 0.038 | 1 | 0.097 | 0.100 | 0.160 | 0.000 | 0.074 | 1.000 |
| K07037 | 0.025 | 0.048 | 0.35 | 0.052 | 0.087 | 0.240 | 0.000 | 0.064 | 1.000 |
| K07038 | 0.146 | 0.089 | 0.04 | 0.174 | 0.101 | 0.050 | 0.134 | 0.142 | 0.150 |
| K07040 | 0.005 | 0.050 | 0.5 | 0.000 | 0.064 | 1.000 | 0.063 | 0.093 | 0.250 |
| K07042 | 0.035 | 0.060 | 0.33 | 0.000 | 0.049 | 1.000 | 0.143 | 0.132 | 0.120 |
| K07045 | 0.000 | 0.040 | 1 | 0.000 | 0.056 | 1.000 | 0.103 | 0.111 | 0.230 |
| K07052 | 0.000 | 0.044 | 1 | 0.000 | 0.060 | 1.000 | 0.000 | 0.083 | 1.000 |
| K07053 | 0.000 | 0.042 | 1 | 0.132 | 0.117 | 0.100 | 0.000 | 0.091 | 1.000 |
| K07054 | 0.074 | 0.066 | 0.1 | 0.094 | 0.088 | 0.250 | 0.000 | 0.075 | 1.000 |
| K07056 | 0.000 | 0.047 | 1 | 0.000 | 0.065 | 1.000 | 0.000 | 0.000 | 0.520 |
| K07068 | 0.000 | 0.037 | 1 | 0.000 | 0.059 | 1.000 | 0.071 | 0.106 | 0.240 |
| K07074 | 0.000 | 0.043 | 1 | 0.000 | 0.000 | 0.670 | 0.029 | 0.084 | 0.520 |
| K07075 | 0.159 | 0.094 | 0.01 | 0.172 | 0.110 | 0.070 | 0.075 | 0.113 | 0.370 |
| K07076 | 0.070 | 0.075 | 0.21 | 0.093 | 0.089 | 0.090 | 0.025 | 0.090 | 0.440 |
| K07079 | 0.000 | 0.042 | 1 | 0.000 | 0.055 | 1.000 | 0.000 | 0.073 | 1.000 |
| K07080 | 0.000 | 0.047 | 1 | 0.091 | 0.098 | 0.240 | 0.000 | 0.071 | 1.000 |
| K07082 | 0.028 | 0.049 | 0.3 | 0.053 | 0.081 | 0.360 | 0.000 | 0.085 | 1.000 |
| K07085 | 0.021 | 0.055 | 0.43 | 0.062 | 0.090 | 0.280 | 0.000 | 0.077 | 1.000 |
| K07089 | 0.059 | 0.062 | 0.27 | 0.056 | 0.095 | 0.350 | 0.034 | 0.101 | 0.360 |
| K07090 | 0.020 | 0.060 | 0.33 | 0.034 | 0.077 | 0.390 | 0.000 | 0.063 | 1.000 |
| K07091 | 0.007 | 0.042 | 0.53 | 0.054 | 0.076 | 0.320 | 0.000 | 0.066 | 1.000 |
| K07096 | 0.012 | 0.050 | 0.56 | 0.037 | 0.070 | 0.370 | 0.000 | 0.071 | 1.000 |
| K07098 | 0.029 | 0.049 | 0.36 | 0.042 | 0.078 | 0.380 | 0.000 | 0.000 | 0.570 |
| K07099 | 0.046 | 0.057 | 0.31 | 0.117 | 0.109 | 0.120 | 0.000 | 0.054 | 1.000 |
| K07102 | 0.088 | 0.075 | 0.13 | 0.138 | 0.108 | 0.090 | 0.000 | 0.074 | 1.000 |
| K07106 | 0.050 | 0.064 | 0.26 | 0.020 | 0.066 | 0.430 | 0.141 | 0.117 | 0.170 |
| K07107 | 0.021 | 0.048 | 0.32 | 0.022 | 0.078 | 0.500 | 0.020 | 0.075 | 0.430 |
| K07114 | 0.047 | 0.056 | 0.31 | 0.081 | 0.082 | 0.270 | 0.000 | 0.084 | 1.000 |
| K07124 | 0.042 | 0.068 | 0.3 | 0.082 | 0.088 | 0.170 | 0.000 | 0.065 | 1.000 |
| K07126 | 0.092 | 0.072 | 0.13 | 0.091 | 0.099 | 0.150 | 0.069 | 0.102 | 0.220 |
| K07130 | 0.088 | 0.078 | 0.13 | 0.177 | 0.114 | 0.080 | 0.000 | 0.081 | 1.000 |
| K07139 | 0.063 | 0.068 | 0.2 | 0.131 | 0.101 | 0.100 | 0.000 | 0.081 | 1.000 |
| K07148 | 0.126 | 0.082 | 0.04 | 0.166 | 0.109 | 0.080 | 0.000 | 0.086 | 1.000 |
| K07149 | 0.000 | 0.053 | 1 | 0.000 | 0.063 | 1.000 | 0.000 | 0.082 | 1.000 |
| K07158 | 0.147 | 0.084 | 0.02 | 0.163 | 0.113 | 0.050 | 0.102 | 0.107 | 0.270 |

| | | | | | | | | | |
|---|---|---|---|---|---|---|---|---|---|
| K07164 | 0.081 | 0.066 | 0.14 | 0.084 | 0.102 | 0.240 | 0.057 | 0.108 | 0.250 |
| K07171 | 0.018 | 0.047 | 0.47 | 0.123 | 0.109 | 0.120 | 0.000 | 0.072 | 1.000 |
| K07172 | 0.135 | 0.079 | 0.03 | 0.192 | 0.115 | 0.060 | 0.000 | 0.084 | 1.000 |
| K07173 | 0.044 | 0.070 | 0.25 | 0.060 | 0.080 | 0.350 | 0.018 | 0.087 | 0.370 |
| K07192 | 0.041 | 0.056 | 0.33 | 0.071 | 0.090 | 0.290 | 0.000 | 0.080 | 1.000 |
| K07220 | 0.063 | 0.064 | 0.21 | 0.102 | 0.101 | 0.140 | 0.058 | 0.099 | 0.380 |
| K07221 | 0.113 | 0.086 | 0.04 | 0.190 | 0.111 | 0.060 | 0.000 | 0.071 | 1.000 |
| K07240 | 0.021 | 0.047 | 0.31 | 0.055 | 0.081 | 0.350 | 0.000 | 0.072 | 1.000 |
| K07248 | 0.000 | 0.043 | 1 | 0.023 | 0.080 | 0.450 | 0.000 | 0.080 | 1.000 |
| K07258 | 0.000 | 0.043 | 1 | 0.073 | 0.083 | 0.290 | 0.000 | 0.060 | 1.000 |
| K07259 | 0.000 | 0.041 | 1 | 0.000 | 0.051 | 1.000 | 0.000 | 0.077 | 1.000 |
| K07260 | 0.000 | 0.042 | 1 | 0.000 | 0.062 | 1.000 | 0.000 | 0.071 | 1.000 |
| K07263 | 0.000 | 0.035 | 1 | 0.000 | 0.070 | 1.000 | 0.000 | 0.086 | 1.000 |
| K07270 | 0.082 | 0.079 | 0.17 | 0.057 | 0.086 | 0.290 | 0.195 | 0.133 | 0.090 |
| K07271 | 0.070 | 0.064 | 0.19 | 0.000 | 0.053 | 1.000 | 0.169 | 0.131 | 0.060 |
| K07273 | 0.068 | 0.064 | 0.15 | 0.076 | 0.081 | 0.200 | 0.036 | 0.106 | 0.490 |
| K07277 | 0.000 | 0.036 | 1 | 0.021 | 0.061 | 0.460 | 0.000 | 0.073 | 1.000 |
| K07301 | 0.056 | 0.052 | 0.2 | 0.074 | 0.104 | 0.270 | 0.000 | 0.076 | 1.000 |
| K07304 | 0.027 | 0.060 | 0.37 | 0.006 | 0.078 | 0.540 | 0.061 | 0.104 | 0.290 |
| K07313 | 0.000 | 0.042 | 1 | 0.000 | 0.067 | 1.000 | 0.000 | 0.056 | 1.000 |
| K07315 | 0.041 | 0.061 | 0.29 | 0.040 | 0.080 | 0.370 | 0.000 | 0.075 | 1.000 |
| K07317 | 0.043 | 0.051 | 0.23 | 0.062 | 0.094 | 0.210 | 0.026 | 0.077 | 0.460 |
| K07318 | 0.024 | 0.052 | 0.4 | 0.014 | 0.063 | 0.460 | 0.027 | 0.088 | 0.520 |
| K07321 | 0.000 | 0.046 | 1 | 0.000 | 0.000 | 0.140 | 0.000 | 0.071 | 1.000 |
| K07322 | 0.008 | 0.039 | 0.56 | 0.048 | 0.089 | 0.350 | 0.000 | 0.083 | 1.000 |
| K07339 | 0.050 | 0.064 | 0.25 | 0.070 | 0.078 | 0.210 | 0.015 | 0.074 | 0.490 |
| K07340 | 0.038 | 0.051 | 0.31 | 0.061 | 0.084 | 0.260 | 0.000 | 0.080 | 1.000 |
| K07341 | 0.001 | 0.040 | 0.54 | 0.068 | 0.090 | 0.260 | 0.000 | 0.078 | 1.000 |
| K07342 | 0.160 | 0.084 | 0.06 | 0.177 | 0.126 | 0.050 | 0.106 | 0.115 | 0.210 |
| K07386 | 0.043 | 0.059 | 0.3 | 0.046 | 0.084 | 0.240 | 0.024 | 0.086 | 0.450 |
| K07391 | 0.000 | 0.048 | 1 | 0.000 | 0.056 | 1.000 | 0.009 | 0.079 | 0.430 |
| K07404 | 0.000 | 0.041 | 1 | 0.048 | 0.078 | 0.370 | 0.000 | 0.076 | 1.000 |
| K07405 | 0.062 | 0.065 | 0.27 | 0.125 | 0.114 | 0.130 | 0.000 | 0.075 | 1.000 |
| K07407 | 0.056 | 0.059 | 0.2 | 0.053 | 0.080 | 0.280 | 0.055 | 0.079 | 0.500 |
| K07443 | 0.000 | 0.041 | 1 | 0.000 | 0.051 | 0.570 | 0.000 | 0.094 | 1.000 |
| K07444 | 0.000 | 0.038 | 1 | 0.000 | 0.058 | 1.000 | 0.000 | 0.079 | 1.000 |
| K07447 | 0.000 | 0.041 | 1 | 0.000 | 0.064 | 1.000 | 0.000 | 0.075 | 1.000 |
| K07448 | 0.029 | 0.056 | 0.35 | 0.000 | 0.065 | 1.000 | 0.081 | 0.112 | 0.260 |
| K07450 | 0.126 | 0.083 | 0.04 | 0.114 | 0.116 | 0.100 | 0.156 | 0.138 | 0.090 |
| K07451 | 0.000 | 0.040 | 1 | 0.000 | 0.070 | 1.000 | 0.000 | 0.075 | 1.000 |
| K07452 | 0.009 | 0.046 | 0.53 | 0.000 | 0.055 | 1.000 | 0.005 | 0.081 | 0.510 |
| K07455 | 0.000 | 0.045 | 1 | 0.000 | 0.050 | 1.000 | 0.000 | 0.082 | 1.000 |
| K07456 | 0.064 | 0.071 | 0.26 | 0.000 | 0.049 | 1.000 | 0.199 | 0.142 | 0.080 |
| K07459 | 0.022 | 0.051 | 0.42 | 0.022 | 0.074 | 0.460 | 0.011 | 0.074 | 0.470 |
| K07460 | 0.000 | 0.044 | 1 | 0.000 | 0.053 | 1.000 | 0.008 | 0.070 | 0.490 |
| K07461 | 0.000 | 0.043 | 1 | 0.000 | 0.052 | 1.000 | 0.000 | 0.075 | 1.000 |
| K07462 | 0.041 | 0.064 | 0.27 | 0.047 | 0.083 | 0.290 | 0.050 | 0.097 | 0.350 |
| K07473 | 0.066 | 0.073 | 0.25 | 0.108 | 0.090 | 0.140 | 0.000 | 0.077 | 1.000 |
| K07474 | 0.033 | 0.062 | 0.26 | 0.068 | 0.086 | 0.210 | 0.000 | 0.079 | 1.000 |
| K07481 | 0.000 | 0.038 | 1 | 0.000 | 0.000 | 0.630 | 0.000 | 0.072 | 1.000 |
| K07483 | 0.017 | 0.047 | 0.42 | 0.021 | 0.064 | 0.390 | 0.017 | 0.082 | 0.480 |
| K07484 | 0.183 | 0.097 | 0.02 | 0.161 | 0.113 | 0.060 | 0.212 | 0.153 | 0.050 |
| K07486 | 0.107 | 0.083 | 0.06 | 0.106 | 0.090 | 0.150 | 0.079 | 0.123 | 0.240 |

| | | | | | | | | | |
|---|---|---|---|---|---|---|---|---|---|
| K07491 | 0.055 | 0.064 | 0.27 | 0.079 | 0.096 | 0.280 | 0.000 | 0.079 | 1.000 |
| K07493 | 0.113 | 0.074 | 0.1 | 0.116 | 0.105 | 0.150 | 0.122 | 0.135 | 0.240 |
| K07494 | 0.105 | 0.074 | 0.09 | 0.073 | 0.096 | 0.280 | 0.133 | 0.129 | 0.150 |
| K07495 | 0.151 | 0.097 | 0.02 | 0.138 | 0.093 | 0.060 | 0.166 | 0.141 | 0.130 |
| K07497 | 0.077 | 0.068 | 0.14 | 0.060 | 0.085 | 0.280 | 0.158 | 0.147 | 0.100 |
| K07499 | 0.000 | 0.047 | 1 | 0.000 | 0.059 | 1.000 | 0.000 | 0.084 | 1.000 |
| K07503 | 0.168 | 0.098 | 0.02 | 0.149 | 0.112 | 0.050 | 0.179 | 0.140 | 0.090 |
| K07507 | 0.000 | 0.046 | 1 | 0.000 | 0.064 | 1.000 | 0.000 | 0.074 | 1.000 |
| K07559 | 0.000 | 0.044 | 1 | 0.057 | 0.091 | 0.340 | 0.000 | 0.076 | 1.000 |
| K07560 | 0.026 | 0.042 | 0.37 | 0.013 | 0.058 | 0.470 | 0.039 | 0.087 | 0.390 |
| K07566 | 0.037 | 0.062 | 0.43 | 0.072 | 0.081 | 0.190 | 0.000 | 0.083 | 1.000 |
| K07568 | 0.000 | 0.045 | 1 | 0.000 | 0.071 | 1.000 | 0.096 | 0.121 | 0.210 |
| K07571 | 0.000 | 0.044 | 1 | 0.000 | 0.052 | 1.000 | 0.000 | 0.079 | 1.000 |
| K07572 | 0.078 | 0.070 | 0.13 | 0.080 | 0.084 | 0.210 | 0.053 | 0.096 | 0.360 |
| K07574 | 0.000 | 0.046 | 1 | 0.089 | 0.102 | 0.240 | 0.000 | 0.075 | 1.000 |
| K07576 | 0.064 | 0.069 | 0.21 | 0.184 | 0.107 | 0.060 | 0.000 | 0.070 | 1.000 |
| K07584 | 0.000 | 0.038 | 1 | 0.000 | 0.064 | 1.000 | 0.000 | 0.076 | 1.000 |
| K07585 | 0.069 | 0.071 | 0.1 | 0.102 | 0.106 | 0.130 | 0.000 | 0.071 | 1.000 |
| K07588 | 0.041 | 0.057 | 0.3 | 0.075 | 0.083 | 0.230 | 0.000 | 0.081 | 1.000 |
| K07590 | 0.067 | 0.067 | 0.24 | 0.000 | 0.060 | 1.000 | 0.142 | 0.143 | 0.150 |
| K07699 | 0.000 | 0.045 | 1 | 0.050 | 0.081 | 0.330 | 0.000 | 0.066 | 1.000 |
| K07706 | 0.084 | 0.065 | 0.16 | 0.125 | 0.107 | 0.060 | 0.044 | 0.081 | 0.340 |
| K07726 | 0.004 | 0.054 | 0.47 | 0.059 | 0.085 | 0.340 | 0.000 | 0.071 | 1.000 |
| K07727 | 0.013 | 0.049 | 0.4 | 0.055 | 0.078 | 0.340 | 0.000 | 0.085 | 1.000 |
| K07729 | 0.000 | 0.049 | 1 | 0.015 | 0.060 | 0.500 | 0.000 | 0.062 | 1.000 |
| K07732 | 0.137 | 0.085 | 0.02 | 0.156 | 0.128 | 0.050 | 0.088 | 0.110 | 0.300 |
| K07733 | 0.014 | 0.051 | 0.33 | 0.042 | 0.073 | 0.390 | 0.000 | 0.073 | 1.000 |
| K07736 | 0.000 | 0.047 | 1 | 0.081 | 0.091 | 0.200 | 0.000 | 0.071 | 1.000 |
| K07738 | 0.000 | 0.039 | 1 | 0.104 | 0.108 | 0.180 | 0.000 | 0.078 | 1.000 |
| K07741 | 0.147 | 0.088 | 0.03 | 0.140 | 0.102 | 0.050 | 0.129 | 0.133 | 0.240 |
| K07742 | 0.017 | 0.048 | 0.47 | 0.117 | 0.106 | 0.100 | 0.000 | 0.079 | 1.000 |
| K07792 | 0.099 | 0.074 | 0.13 | 0.079 | 0.084 | 0.250 | 0.143 | 0.133 | 0.200 |
| K07794 | 0.107 | 0.076 | 0.09 | 0.054 | 0.077 | 0.290 | 0.157 | 0.143 | 0.120 |
| K07795 | 0.126 | 0.091 | 0.07 | 0.129 | 0.116 | 0.060 | 0.108 | 0.119 | 0.180 |
| K07813 | 0.052 | 0.068 | 0.22 | 0.101 | 0.103 | 0.150 | 0.007 | 0.062 | 0.500 |
| K07814 | 0.000 | 0.038 | 1 | 0.037 | 0.077 | 0.340 | 0.000 | 0.078 | 1.000 |
| K07816 | 0.103 | 0.083 | 0.11 | 0.097 | 0.089 | 0.170 | 0.111 | 0.134 | 0.210 |
| K07862 | 0.082 | 0.082 | 0.15 | 0.017 | 0.074 | 0.490 | 0.127 | 0.130 | 0.160 |
| K08177 | 0.051 | 0.062 | 0.31 | 0.006 | 0.051 | 0.470 | 0.117 | 0.136 | 0.220 |
| K08191 | 0.022 | 0.050 | 0.47 | 0.075 | 0.077 | 0.240 | 0.000 | 0.083 | 1.000 |
| K08217 | 0.000 | 0.041 | 1 | 0.000 | 0.058 | 1.000 | 0.000 | 0.000 | 0.710 |
| K08218 | 0.000 | 0.041 | 1 | 0.000 | 0.060 | 1.000 | 0.000 | 0.077 | 1.000 |
| K08223 | 0.121 | 0.085 | 0.06 | 0.172 | 0.114 | 0.060 | 0.023 | 0.087 | 0.490 |
| K08281 | 0.041 | 0.056 | 0.24 | 0.000 | 0.059 | 1.000 | 0.123 | 0.124 | 0.140 |
| K08289 | 0.136 | 0.091 | 0.05 | 0.199 | 0.128 | 0.060 | 0.032 | 0.097 | 0.340 |
| K08303 | 0.075 | 0.061 | 0.14 | 0.000 | 0.065 | 1.000 | 0.224 | 0.153 | 0.050 |
| K08309 | 0.000 | 0.050 | 1 | 0.003 | 0.054 | 0.570 | 0.000 | 0.069 | 1.000 |
| K08316 | 0.011 | 0.046 | 0.4 | 0.007 | 0.069 | 0.590 | 0.010 | 0.085 | 0.600 |
| K08640 | 0.058 | 0.067 | 0.17 | 0.050 | 0.081 | 0.340 | 0.083 | 0.119 | 0.260 |
| K08641 | 0.124 | 0.088 | 0.08 | 0.108 | 0.099 | 0.120 | 0.163 | 0.138 | 0.140 |
| K08659 | 0.015 | 0.046 | 0.45 | 0.000 | 0.059 | 1.000 | 0.092 | 0.100 | 0.250 |
| K08676 | 0.007 | 0.042 | 0.53 | 0.000 | 0.060 | 1.000 | 0.036 | 0.098 | 0.360 |
| K08678 | 0.097 | 0.086 | 0.07 | 0.111 | 0.111 | 0.110 | 0.000 | 0.066 | 1.000 |

| | | | | | | | | | |
|---|---|---|---|---|---|---|---|---|---|
| K08679 | 0.092 | 0.074 | 0.06 | 0.074 | 0.092 | 0.250 | 0.113 | 0.123 | 0.240 |
| K08681 | 0.081 | 0.074 | 0.07 | 0.064 | 0.087 | 0.340 | 0.099 | 0.115 | 0.230 |
| K08722 | 0.000 | 0.040 | 1 | 0.018 | 0.075 | 0.400 | 0.000 | 0.076 | 1.000 |
| K08968 | 0.000 | 0.043 | 1 | 0.000 | 0.064 | 1.000 | 0.070 | 0.127 | 0.360 |
| K08972 | 0.012 | 0.050 | 0.47 | 0.073 | 0.082 | 0.200 | 0.000 | 0.079 | 1.000 |
| K08974 | 0.018 | 0.044 | 0.44 | 0.043 | 0.076 | 0.420 | 0.000 | 0.079 | 1.000 |
| K08978 | 0.057 | 0.070 | 0.19 | 0.079 | 0.091 | 0.240 | 0.059 | 0.105 | 0.340 |
| K08992 | 0.009 | 0.046 | 0.49 | 0.039 | 0.073 | 0.340 | 0.000 | 0.065 | 1.000 |
| K08998 | 0.000 | 0.049 | 1 | 0.040 | 0.061 | 0.350 | 0.000 | 0.083 | 1.000 |
| K09001 | 0.000 | 0.041 | 1 | 0.011 | 0.060 | 0.490 | 0.000 | 0.067 | 1.000 |
| K09002 | 0.077 | 0.072 | 0.19 | 0.085 | 0.106 | 0.160 | 0.062 | 0.106 | 0.370 |
| K09005 | 0.090 | 0.074 | 0.13 | 0.090 | 0.088 | 0.230 | 0.131 | 0.153 | 0.160 |
| K09011 | 0.032 | 0.052 | 0.35 | 0.024 | 0.062 | 0.540 | 0.041 | 0.100 | 0.330 |
| K09013 | 0.000 | 0.047 | 1 | 0.042 | 0.078 | 0.270 | 0.000 | 0.076 | 1.000 |
| K09014 | 0.005 | 0.052 | 0.57 | 0.000 | 0.063 | 1.000 | 0.050 | 0.113 | 0.350 |
| K09015 | 0.039 | 0.065 | 0.3 | 0.108 | 0.094 | 0.120 | 0.000 | 0.071 | 1.000 |
| K09022 | 0.113 | 0.084 | 0.09 | 0.084 | 0.089 | 0.160 | 0.140 | 0.135 | 0.080 |
| K09117 | 0.000 | 0.051 | 1 | 0.048 | 0.071 | 0.340 | 0.000 | 0.072 | 1.000 |
| K09121 | 0.093 | 0.076 | 0.07 | 0.139 | 0.102 | 0.090 | 0.071 | 0.104 | 0.270 |
| K09124 | 0.006 | 0.046 | 0.43 | 0.000 | 0.060 | 1.000 | 0.086 | 0.096 | 0.190 |
| K09125 | 0.057 | 0.065 | 0.3 | 0.065 | 0.085 | 0.240 | 0.006 | 0.074 | 0.520 |
| K09128 | 0.068 | 0.065 | 0.14 | 0.026 | 0.067 | 0.390 | 0.129 | 0.122 | 0.160 |
| K09132 | 0.015 | 0.044 | 0.44 | 0.004 | 0.058 | 0.490 | 0.071 | 0.116 | 0.280 |
| K09144 | 0.029 | 0.049 | 0.45 | 0.142 | 0.109 | 0.140 | 0.000 | 0.088 | 1.000 |
| K09163 | 0.049 | 0.062 | 0.3 | 0.101 | 0.095 | 0.150 | 0.035 | 0.111 | 0.430 |
| K09384 | 0.007 | 0.047 | 0.47 | 0.000 | 0.058 | 1.000 | 0.034 | 0.094 | 0.480 |
| K09457 | 0.053 | 0.062 | 0.18 | 0.000 | 0.060 | 1.000 | 0.167 | 0.114 | 0.140 |
| K09458 | 0.120 | 0.088 | 0.05 | 0.057 | 0.077 | 0.200 | 0.196 | 0.153 | 0.080 |
| K09459 | 0.013 | 0.049 | 0.52 | 0.000 | 0.056 | 1.000 | 0.159 | 0.133 | 0.120 |
| K09474 | 0.108 | 0.092 | 0.07 | 0.096 | 0.092 | 0.180 | 0.164 | 0.144 | 0.150 |
| K09516 | 0.163 | 0.086 | 0.02 | 0.196 | 0.108 | 0.050 | 0.042 | 0.093 | 0.450 |
| K09607 | 0.000 | 0.038 | 1 | 0.034 | 0.077 | 0.340 | 0.000 | 0.070 | 1.000 |
| K09680 | 0.098 | 0.080 | 0.17 | 0.116 | 0.103 | 0.140 | 0.104 | 0.138 | 0.110 |
| K09692 | 0.000 | 0.038 | 1 | 0.000 | 0.053 | 1.000 | 0.000 | 0.079 | 1.000 |
| K09702 | 0.100 | 0.085 | 0.08 | 0.147 | 0.108 | 0.060 | 0.000 | 0.078 | 1.000 |
| K09704 | 0.090 | 0.074 | 0.08 | 0.057 | 0.091 | 0.380 | 0.144 | 0.142 | 0.140 |
| K09706 | 0.000 | 0.038 | 1 | 0.000 | 0.050 | 1.000 | 0.000 | 0.068 | 1.000 |
| K09707 | 0.018 | 0.045 | 0.45 | 0.001 | 0.056 | 0.500 | 0.042 | 0.100 | 0.360 |
| K09727 | 0.108 | 0.084 | 0.05 | 0.079 | 0.080 | 0.210 | 0.165 | 0.138 | 0.080 |
| K09728 | 0.105 | 0.087 | 0.07 | 0.128 | 0.098 | 0.120 | 0.041 | 0.092 | 0.430 |
| K09729 | 0.000 | 0.044 | 1 | 0.000 | 0.000 | 0.700 | 0.000 | 0.070 | 1.000 |
| K09739 | 0.152 | 0.088 | 0.02 | 0.194 | 0.127 | 0.070 | 0.036 | 0.092 | 0.490 |
| K09747 | 0.011 | 0.044 | 0.49 | 0.123 | 0.106 | 0.140 | 0.000 | 0.079 | 1.000 |
| K09748 | 0.000 | 0.039 | 1 | 0.000 | 0.058 | 1.000 | 0.000 | 0.080 | 1.000 |
| K09760 | 0.007 | 0.042 | 0.51 | 0.000 | 0.052 | 1.000 | 0.152 | 0.135 | 0.180 |
| K09761 | 0.040 | 0.059 | 0.38 | 0.025 | 0.066 | 0.470 | 0.055 | 0.099 | 0.310 |
| K09764 | 0.137 | 0.086 | 0.03 | 0.062 | 0.093 | 0.270 | 0.270 | 0.173 | 0.050 |
| K09765 | 0.000 | 0.037 | 1 | 0.000 | 0.055 | 1.000 | 0.000 | 0.069 | 1.000 |
| K09767 | 0.000 | 0.052 | 1 | 0.000 | 0.058 | 1.000 | 0.000 | 0.000 | 0.590 |
| K09768 | 0.041 | 0.054 | 0.34 | 0.046 | 0.076 | 0.340 | 0.069 | 0.110 | 0.190 |
| K09769 | 0.011 | 0.046 | 0.44 | 0.120 | 0.094 | 0.140 | 0.000 | 0.070 | 1.000 |
| K09772 | 0.054 | 0.064 | 0.25 | 0.145 | 0.102 | 0.050 | 0.000 | 0.059 | 1.000 |
| K09775 | 0.079 | 0.075 | 0.16 | 0.173 | 0.107 | 0.070 | 0.000 | 0.071 | 1.000 |

| | | | | | | | | | |
|---|---|---|---|---|---|---|---|---|---|
| K09777 | 0.000 | 0.034 | 1 | 0.000 | 0.058 | 1.000 | 0.000 | 0.079 | 1.000 |
| K09779 | 0.011 | 0.051 | 0.49 | 0.077 | 0.091 | 0.230 | 0.000 | 0.083 | 1.000 |
| K09780 | 0.105 | 0.078 | 0.04 | 0.094 | 0.098 | 0.150 | 0.122 | 0.116 | 0.140 |
| K09787 | 0.023 | 0.056 | 0.4 | 0.160 | 0.118 | 0.080 | 0.000 | 0.079 | 1.000 |
| K09803 | 0.088 | 0.075 | 0.11 | 0.104 | 0.097 | 0.120 | 0.023 | 0.072 | 0.490 |
| K09807 | 0.058 | 0.058 | 0.25 | 0.161 | 0.120 | 0.050 | 0.000 | 0.073 | 1.000 |
| K09809 | 0.002 | 0.046 | 0.53 | 0.000 | 0.060 | 1.000 | 0.033 | 0.101 | 0.450 |
| K09811 | 0.000 | 0.042 | 1 | 0.000 | 0.056 | 1.000 | 0.000 | 0.075 | 1.000 |
| K09812 | 0.078 | 0.071 | 0.13 | 0.000 | 0.073 | 1.000 | 0.193 | 0.141 | 0.090 |
| K09815 | 0.065 | 0.067 | 0.19 | 0.145 | 0.108 | 0.080 | 0.000 | 0.000 | 0.380 |
| K09834 | 0.038 | 0.054 | 0.29 | 0.045 | 0.080 | 0.360 | 0.006 | 0.078 | 0.460 |
| K09861 | 0.114 | 0.083 | 0.08 | 0.036 | 0.068 | 0.410 | 0.185 | 0.144 | 0.050 |
| K09888 | 0.000 | 0.043 | 1 | 0.014 | 0.059 | 0.480 | 0.000 | 0.078 | 1.000 |
| K09903 | 0.000 | 0.041 | 1 | 0.000 | 0.065 | 1.000 | 0.027 | 0.096 | 0.500 |
| K09922 | 0.143 | 0.073 | 0.02 | 0.161 | 0.111 | 0.060 | 0.103 | 0.103 | 0.210 |
| K09946 | 0.065 | 0.075 | 0.25 | 0.033 | 0.063 | 0.500 | 0.161 | 0.138 | 0.090 |
| K09951 | 0.067 | 0.065 | 0.19 | 0.116 | 0.103 | 0.160 | 0.000 | 0.078 | 1.000 |
| K09952 | 0.040 | 0.061 | 0.35 | 0.000 | 0.061 | 1.000 | 0.128 | 0.135 | 0.140 |
| K09955 | 0.087 | 0.085 | 0.1 | 0.144 | 0.106 | 0.060 | 0.012 | 0.075 | 0.520 |
| K09968 | 0.040 | 0.063 | 0.24 | 0.083 | 0.083 | 0.140 | 0.000 | 0.089 | 1.000 |
| K09973 | 0.000 | 0.044 | 1 | 0.000 | 0.000 | 0.590 | 0.000 | 0.079 | 1.000 |
| K10026 | 0.118 | 0.086 | 0.07 | 0.093 | 0.096 | 0.180 | 0.133 | 0.130 | 0.140 |
| K10112 | 0.019 | 0.059 | 0.43 | 0.006 | 0.051 | 0.470 | 0.053 | 0.099 | 0.350 |
| K10118 | 0.044 | 0.065 | 0.34 | 0.085 | 0.098 | 0.180 | 0.000 | 0.083 | 1.000 |
| K10212 | 0.044 | 0.064 | 0.27 | 0.175 | 0.108 | 0.070 | 0.000 | 0.078 | 1.000 |
| K10439 | 0.014 | 0.040 | 0.47 | 0.000 | 0.059 | 1.000 | 0.090 | 0.111 | 0.170 |
| K10532 | 0.000 | 0.038 | 1 | 0.000 | 0.059 | 1.000 | 0.038 | 0.098 | 0.430 |
| K10536 | 0.031 | 0.057 | 0.35 | 0.094 | 0.093 | 0.190 | 0.000 | 0.073 | 1.000 |
| K10563 | 0.133 | 0.088 | 0.03 | 0.158 | 0.110 | 0.070 | 0.082 | 0.112 | 0.290 |
| K10710 | 0.047 | 0.060 | 0.28 | 0.045 | 0.085 | 0.250 | 0.043 | 0.106 | 0.310 |
| K10716 | 0.094 | 0.087 | 0.06 | 0.098 | 0.108 | 0.150 | 0.005 | 0.079 | 0.500 |
| K10725 | 0.144 | 0.083 | 0.04 | 0.148 | 0.119 | 0.090 | 0.124 | 0.120 | 0.190 |
| K10761 | 0.185 | 0.104 | 0.01 | 0.179 | 0.115 | 0.060 | 0.138 | 0.126 | 0.120 |
| K10804 | 0.000 | 0.036 | 1 | 0.000 | 0.060 | 1.000 | 0.000 | 0.074 | 1.000 |
| K10822 | 0.000 | 0.040 | 1 | 0.002 | 0.057 | 0.470 | 0.023 | 0.079 | 0.440 |
| K10914 | 0.090 | 0.080 | 0.11 | 0.100 | 0.099 | 0.190 | 0.038 | 0.090 | 0.430 |
| K10947 | 0.033 | 0.064 | 0.32 | 0.034 | 0.072 | 0.450 | 0.022 | 0.079 | 0.530 |
| K11068 | 0.145 | 0.087 | 0.03 | 0.138 | 0.113 | 0.130 | 0.121 | 0.138 | 0.140 |
| K11070 | 0.000 | 0.048 | 1 | 0.000 | 0.061 | 1.000 | 0.000 | 0.074 | 1.000 |
| K11071 | 0.000 | 0.046 | 0.54 | 0.046 | 0.091 | 0.340 | 0.000 | 0.080 | 1.000 |
| K11072 | 0.010 | 0.050 | 0.43 | 0.031 | 0.082 | 0.410 | 0.000 | 0.067 | 1.000 |
| K11105 | 0.149 | 0.093 | 0.02 | 0.089 | 0.092 | 0.220 | 0.254 | 0.162 | 0.050 |
| K11145 | 0.013 | 0.044 | 0.47 | 0.121 | 0.114 | 0.060 | 0.000 | 0.072 | 1.000 |
| K11175 | 0.000 | 0.050 | 1 | 0.000 | 0.053 | 1.000 | 0.014 | 0.080 | 0.440 |
| K11176 | 0.047 | 0.056 | 0.27 | 0.137 | 0.097 | 0.110 | 0.000 | 0.080 | 1.000 |
| K11358 | 0.076 | 0.065 | 0.12 | 0.136 | 0.111 | 0.080 | 0.000 | 0.064 | 1.000 |
| K11392 | 0.019 | 0.046 | 0.44 | 0.000 | 0.056 | 1.000 | 0.114 | 0.140 | 0.190 |
| K11621 | 0.004 | 0.045 | 0.5 | 0.000 | 0.000 | 0.910 | 0.043 | 0.111 | 0.380 |
| K11717 | 0.018 | 0.036 | 0.44 | 0.000 | 0.067 | 1.000 | 0.056 | 0.105 | 0.410 |
| K11749 | 0.059 | 0.073 | 0.23 | 0.000 | 0.067 | 1.000 | 0.153 | 0.151 | 0.160 |
| K11754 | 0.028 | 0.048 | 0.35 | 0.051 | 0.060 | 0.280 | 0.039 | 0.109 | 0.380 |
| K11755 | 0.053 | 0.057 | 0.17 | 0.122 | 0.098 | 0.130 | 0.000 | 0.078 | 1.000 |
| K11928 | 0.068 | 0.069 | 0.25 | 0.066 | 0.095 | 0.260 | 0.058 | 0.110 | 0.320 |

| | | | | | | | | | |
|---|---|---|---|---|---|---|---|---|---|
| K11936 | 0.028 | 0.063 | 0.38 | 0.080 | 0.084 | 0.210 | 0.000 | 0.074 | 1.000 |
| K11941 | 0.054 | 0.078 | 0.2 | 0.093 | 0.103 | 0.170 | 0.000 | 0.086 | 1.000 |
| K11991 | 0.000 | 0.045 | 1 | 0.019 | 0.073 | 0.450 | 0.000 | 0.062 | 1.000 |
| K12251 | 0.000 | 0.044 | 1 | 0.039 | 0.069 | 0.430 | 0.000 | 0.081 | 1.000 |
| K12308 | 0.087 | 0.072 | 0.15 | 0.100 | 0.089 | 0.130 | 0.072 | 0.097 | 0.380 |
| K12340 | 0.133 | 0.080 | 0.04 | 0.136 | 0.107 | 0.080 | 0.099 | 0.120 | 0.310 |
| K12410 | 0.013 | 0.061 | 0.5 | 0.012 | 0.058 | 0.470 | 0.003 | 0.083 | 0.600 |
| K12452 | 0.189 | 0.090 | 0.03 | 0.153 | 0.123 | 0.050 | 0.223 | 0.183 | 0.090 |
| K12510 | 0.043 | 0.061 | 0.33 | 0.069 | 0.078 | 0.190 | 0.000 | 0.069 | 1.000 |
| K12511 | 0.077 | 0.075 | 0.15 | 0.182 | 0.127 | 0.050 | 0.000 | 0.077 | 1.000 |
| K12573 | 0.090 | 0.081 | 0.1 | 0.147 | 0.125 | 0.120 | 0.032 | 0.091 | 0.450 |
| K12574 | 0.109 | 0.076 | 0.06 | 0.153 | 0.107 | 0.070 | 0.083 | 0.112 | 0.320 |
| K12950 | 0.135 | 0.089 | 0.04 | 0.122 | 0.100 | 0.100 | 0.136 | 0.146 | 0.140 |
| K12960 | 0.044 | 0.058 | 0.27 | 0.022 | 0.072 | 0.430 | 0.083 | 0.110 | 0.270 |
| K12976 | 0.052 | 0.064 | 0.25 | 0.155 | 0.104 | 0.070 | 0.000 | 0.086 | 1.000 |
| K12990 | 0.012 | 0.049 | 0.5 | 0.030 | 0.074 | 0.360 | 0.000 | 0.071 | 1.000 |
| K12994 | 0.063 | 0.068 | 0.23 | 0.040 | 0.071 | 0.340 | 0.099 | 0.118 | 0.220 |
| K12995 | 0.006 | 0.047 | 0.5 | 0.000 | 0.000 | 0.930 | 0.025 | 0.086 | 0.480 |
| K12997 | 0.023 | 0.053 | 0.42 | 0.084 | 0.090 | 0.180 | 0.000 | 0.074 | 1.000 |
| K13013 | 0.023 | 0.049 | 0.4 | 0.022 | 0.072 | 0.380 | 0.029 | 0.097 | 0.370 |
| K13038 | 0.073 | 0.068 | 0.16 | 0.103 | 0.101 | 0.120 | 0.042 | 0.103 | 0.380 |
| K13043 | 0.030 | 0.050 | 0.33 | 0.102 | 0.100 | 0.200 | 0.000 | 0.071 | 1.000 |
| K13052 | 0.110 | 0.092 | 0.06 | 0.112 | 0.097 | 0.130 | 0.120 | 0.132 | 0.180 |
| K13075 | 0.013 | 0.044 | 0.44 | 0.061 | 0.087 | 0.270 | 0.000 | 0.075 | 1.000 |
| K13280 | 0.027 | 0.054 | 0.33 | 0.100 | 0.103 | 0.220 | 0.000 | 0.069 | 1.000 |
| K13444 | 0.042 | 0.059 | 0.28 | 0.035 | 0.067 | 0.400 | 0.069 | 0.088 | 0.270 |
| K13532 | 0.031 | 0.047 | 0.4 | 0.086 | 0.087 | 0.230 | 0.000 | 0.075 | 1.000 |
| K13566 | 0.026 | 0.049 | 0.38 | 0.042 | 0.078 | 0.340 | 0.010 | 0.080 | 0.500 |
| K13653 | 0.046 | 0.062 | 0.25 | 0.027 | 0.070 | 0.440 | 0.084 | 0.103 | 0.280 |
| K13684 | 0.000 | 0.047 | 1 | 0.049 | 0.068 | 0.300 | 0.000 | 0.068 | 1.000 |
| K13747 | 0.079 | 0.073 | 0.11 | 0.116 | 0.095 | 0.110 | 0.000 | 0.065 | 1.000 |
| K13787 | 0.091 | 0.089 | 0.13 | 0.011 | 0.064 | 0.450 | 0.193 | 0.151 | 0.070 |
| K13789 | 0.011 | 0.047 | 0.51 | 0.052 | 0.080 | 0.360 | 0.000 | 0.000 | 0.830 |
| K13985 | 0.158 | 0.088 | 0.02 | 0.151 | 0.110 | 0.060 | 0.217 | 0.151 | 0.060 |
| K14058 | 0.022 | 0.050 | 0.4 | 0.000 | 0.056 | 1.000 | 0.196 | 0.151 | 0.080 |
| K14059 | 0.172 | 0.089 | 0.01 | 0.176 | 0.117 | 0.050 | 0.199 | 0.175 | 0.050 |
| K14060 | 0.000 | 0.039 | 1 | 0.000 | 0.000 | 0.550 | 0.000 | 0.078 | 1.000 |
| K14089 | 0.055 | 0.063 | 0.22 | 0.058 | 0.079 | 0.250 | 0.026 | 0.086 | 0.390 |
| K14092 | 0.072 | 0.077 | 0.11 | 0.095 | 0.109 | 0.160 | 0.000 | 0.000 | 0.500 |
| K14095 | 0.154 | 0.087 | 0.02 | 0.152 | 0.103 | 0.100 | 0.131 | 0.135 | 0.170 |
| K14096 | 0.130 | 0.075 | 0.03 | 0.179 | 0.114 | 0.050 | 0.035 | 0.101 | 0.400 |
| K14110 | 0.128 | 0.090 | 0.06 | 0.109 | 0.101 | 0.150 | 0.148 | 0.144 | 0.100 |
| K14113 | 0.063 | 0.067 | 0.17 | 0.054 | 0.075 | 0.320 | 0.086 | 0.111 | 0.300 |
| K14117 | 0.118 | 0.069 | 0.05 | 0.166 | 0.105 | 0.110 | 0.026 | 0.075 | 0.450 |
| K14119 | 0.108 | 0.083 | 0.1 | 0.131 | 0.110 | 0.070 | 0.049 | 0.089 | 0.360 |
| K14127 | 0.048 | 0.059 | 0.26 | 0.088 | 0.101 | 0.160 | 0.006 | 0.087 | 0.500 |
| K14136 | 0.100 | 0.079 | 0.05 | 0.124 | 0.110 | 0.150 | 0.000 | 0.062 | 1.000 |
| K14188 | 0.002 | 0.046 | 0.59 | 0.027 | 0.064 | 0.400 | 0.000 | 0.066 | 1.000 |
| K14415 | 0.033 | 0.053 | 0.27 | 0.039 | 0.074 | 0.330 | 0.014 | 0.065 | 0.450 |
| K14441 | 0.051 | 0.062 | 0.26 | 0.000 | 0.056 | 1.000 | 0.174 | 0.131 | 0.050 |
| K14445 | 0.089 | 0.073 | 0.09 | 0.082 | 0.090 | 0.220 | 0.078 | 0.119 | 0.210 |
| K14623 | 0.000 | 0.036 | 1 | 0.000 | 0.063 | 1.000 | 0.000 | 0.072 | 1.000 |
| K14731 | 0.000 | 0.043 | 1 | 0.000 | 0.053 | 1.000 | 0.000 | 0.066 | 1.000 |

| | | | | | | | | | |
|---|---|---|---|---|---|---|---|---|---|
| K14742 | 0.014 | 0.052 | 0.46 | 0.054 | 0.079 | 0.210 | 0.000 | 0.082 | 1.000 |
| K14982 | 0.102 | 0.079 | 0.1 | 0.121 | 0.110 | 0.090 | 0.095 | 0.124 | 0.200 |
| K15024 | 0.051 | 0.062 | 0.27 | 0.118 | 0.106 | 0.160 | 0.000 | 0.083 | 1.000 |
| K15255 | 0.022 | 0.049 | 0.34 | 0.000 | 0.051 | 1.000 | 0.067 | 0.122 | 0.300 |
| K15256 | 0.050 | 0.069 | 0.22 | 0.070 | 0.087 | 0.280 | 0.000 | 0.000 | 0.780 |
| K15270 | 0.000 | 0.034 | 1 | 0.071 | 0.087 | 0.270 | 0.000 | 0.082 | 1.000 |
| K15342 | 0.100 | 0.074 | 0.09 | 0.147 | 0.100 | 0.080 | 0.000 | 0.068 | 1.000 |
| K15383 | 0.119 | 0.080 | 0.07 | 0.103 | 0.096 | 0.150 | 0.230 | 0.144 | 0.060 |
| K15460 | 0.078 | 0.076 | 0.25 | 0.114 | 0.109 | 0.130 | 0.046 | 0.083 | 0.400 |
| K15519 | 0.062 | 0.076 | 0.21 | 0.000 | 0.052 | 0.510 | 0.211 | 0.146 | 0.060 |
| K15532 | 0.061 | 0.071 | 0.23 | 0.119 | 0.097 | 0.140 | 0.000 | 0.067 | 1.000 |
| K15633 | 0.000 | 0.041 | 0.53 | 0.017 | 0.069 | 0.530 | 0.000 | 0.081 | 1.000 |
| K15635 | 0.152 | 0.087 | 0.02 | 0.108 | 0.098 | 0.120 | 0.181 | 0.144 | 0.070 |
| K15738 | 0.075 | 0.075 | 0.18 | 0.144 | 0.109 | 0.050 | 0.000 | 0.069 | 1.000 |
| K15771 | 0.026 | 0.052 | 0.39 | 0.140 | 0.109 | 0.100 | 0.000 | 0.067 | 1.000 |
| K15772 | 0.013 | 0.050 | 0.49 | 0.089 | 0.093 | 0.140 | 0.000 | 0.066 | 1.000 |
| K15773 | 0.076 | 0.071 | 0.17 | 0.086 | 0.098 | 0.200 | 0.037 | 0.095 | 0.380 |
| K15789 | 0.088 | 0.071 | 0.1 | 0.062 | 0.084 | 0.300 | 0.148 | 0.134 | 0.140 |
| K15897 | 0.000 | 0.000 | 0.24 | 0.000 | 0.057 | 1.000 | 0.180 | 0.143 | 0.090 |
| K15898 | 0.000 | 0.041 | 1 | 0.037 | 0.069 | 0.270 | 0.000 | 0.059 | 1.000 |
| K15899 | 0.000 | 0.043 | 1 | 0.000 | 0.054 | 1.000 | 0.000 | 0.076 | 1.000 |
| K15921 | 0.010 | 0.054 | 0.51 | 0.044 | 0.070 | 0.410 | 0.000 | 0.070 | 1.000 |
| K15923 | 0.054 | 0.060 | 0.21 | 0.109 | 0.105 | 0.170 | 0.000 | 0.090 | 1.000 |
| K15924 | 0.096 | 0.076 | 0.12 | 0.053 | 0.086 | 0.290 | 0.172 | 0.146 | 0.140 |
| K15984 | 0.095 | 0.073 | 0.11 | 0.041 | 0.076 | 0.460 | 0.162 | 0.145 | 0.050 |
| K15986 | 0.018 | 0.052 | 0.42 | 0.016 | 0.058 | 0.500 | 0.033 | 0.097 | 0.450 |
| K15987 | 0.012 | 0.042 | 0.56 | 0.091 | 0.090 | 0.220 | 0.000 | 0.064 | 1.000 |
| K16066 | 0.090 | 0.073 | 0.15 | 0.058 | 0.079 | 0.320 | 0.166 | 0.146 | 0.090 |
| K16214 | 0.059 | 0.075 | 0.18 | 0.000 | 0.059 | 1.000 | 0.160 | 0.126 | 0.130 |
| K16264 | 0.089 | 0.075 | 0.19 | 0.061 | 0.088 | 0.290 | 0.173 | 0.147 | 0.150 |
| K16328 | 0.025 | 0.061 | 0.42 | 0.008 | 0.064 | 0.560 | 0.097 | 0.116 | 0.220 |
| K16511 | 0.085 | 0.082 | 0.13 | 0.104 | 0.092 | 0.180 | 0.006 | 0.083 | 0.550 |
| K16568 | 0.110 | 0.073 | 0.04 | 0.153 | 0.103 | 0.100 | 0.000 | 0.075 | 1.000 |
| K16692 | 0.000 | 0.047 | 1 | 0.000 | 0.049 | 1.000 | 0.022 | 0.094 | 0.480 |
| K16694 | 0.022 | 0.057 | 0.33 | 0.020 | 0.072 | 0.370 | 0.034 | 0.092 | 0.450 |
| K16698 | 0.053 | 0.072 | 0.34 | 0.000 | 0.000 | 0.510 | 0.106 | 0.114 | 0.180 |
| K16710 | 0.072 | 0.066 | 0.23 | 0.000 | 0.059 | 1.000 | 0.176 | 0.126 | 0.070 |
| K16785 | 0.160 | 0.097 | 0.03 | 0.231 | 0.127 | 0.050 | 0.102 | 0.115 | 0.170 |
| K16786 | 0.108 | 0.075 | 0.07 | 0.164 | 0.116 | 0.070 | 0.020 | 0.087 | 0.610 |
| K16787 | 0.174 | 0.088 | 0.03 | 0.201 | 0.111 | 0.050 | 0.180 | 0.129 | 0.100 |
| K16870 | 0.075 | 0.068 | 0.21 | 0.145 | 0.105 | 0.090 | 0.000 | 0.082 | 1.000 |
| K16899 | 0.070 | 0.072 | 0.16 | 0.027 | 0.060 | 0.320 | 0.110 | 0.124 | 0.210 |
| K17103 | 0.000 | 0.038 | 1 | 0.000 | 0.066 | 1.000 | 0.028 | 0.094 | 0.410 |
| K17319 | 0.026 | 0.057 | 0.42 | 0.075 | 0.090 | 0.240 | 0.000 | 0.067 | 1.000 |
| K17320 | 0.032 | 0.059 | 0.3 | 0.112 | 0.099 | 0.170 | 0.000 | 0.064 | 1.000 |
| K17609 | 0.103 | 0.077 | 0.11 | 0.155 | 0.120 | 0.060 | 0.077 | 0.109 | 0.300 |
| K17723 | 0.072 | 0.069 | 0.1 | 0.078 | 0.098 | 0.290 | 0.034 | 0.091 | 0.440 |
| K17752 | 0.000 | 0.044 | 1 | 0.000 | 0.057 | 1.000 | 0.000 | 0.067 | 1.000 |
| K17810 | 0.000 | 0.046 | 1 | 0.000 | 0.059 | 1.000 | 0.000 | 0.080 | 1.000 |
| K17884 | 0.013 | 0.045 | 0.48 | 0.007 | 0.060 | 0.490 | 0.007 | 0.079 | 0.580 |
| K17992 | 0.000 | 0.038 | 1 | 0.000 | 0.064 | 1.000 | 0.000 | 0.073 | 1.000 |
| K18014 | 0.000 | 0.036 | 1 | 0.000 | 0.057 | 1.000 | 0.161 | 0.149 | 0.080 |
| K18122 | 0.082 | 0.084 | 0.14 | 0.108 | 0.095 | 0.100 | 0.121 | 0.121 | 0.170 |

| | | | | | | | | | |
|---|---|---|---|---|---|---|---|---|---|
| K18189 | 0.000 | 0.045 | 1 | 0.019 | 0.069 | 0.430 | 0.000 | 0.088 | 1.000 |
| K18197 | 0.000 | 0.038 | 1 | 0.035 | 0.069 | 0.350 | 0.000 | 0.085 | 1.000 |
| K18218 | 0.038 | 0.059 | 0.34 | 0.000 | 0.057 | 1.000 | 0.156 | 0.138 | 0.080 |
| K18234 | 0.035 | 0.056 | 0.36 | 0.030 | 0.064 | 0.400 | 0.066 | 0.110 | 0.260 |
| K18353 | 0.061 | 0.074 | 0.23 | 0.153 | 0.112 | 0.100 | 0.000 | 0.071 | 1.000 |
| K18475 | 0.081 | 0.064 | 0.16 | 0.077 | 0.104 | 0.230 | 0.087 | 0.130 | 0.260 |
| K18581 | 0.086 | 0.076 | 0.13 | 0.089 | 0.086 | 0.190 | 0.100 | 0.132 | 0.260 |
| K18640 | 0.000 | 0.042 | 1 | 0.000 | 0.064 | 1.000 | 0.000 | 0.077 | 1.000 |
| K18672 | 0.000 | 0.044 | 1 | 0.000 | 0.056 | 1.000 | 0.000 | 0.068 | 1.000 |
| K18677 | 0.035 | 0.058 | 0.24 | 0.035 | 0.070 | 0.380 | 0.014 | 0.081 | 0.470 |
| K18678 | 0.013 | 0.046 | 0.46 | 0.047 | 0.077 | 0.370 | 0.000 | 0.091 | 1.000 |
| K18682 | 0.013 | 0.046 | 0.35 | 0.000 | 0.056 | 1.000 | 0.147 | 0.141 | 0.180 |
| K18691 | 0.087 | 0.073 | 0.18 | 0.145 | 0.114 | 0.070 | 0.000 | 0.074 | 1.000 |
| K18697 | 0.022 | 0.048 | 0.47 | 0.000 | 0.049 | 1.000 | 0.104 | 0.124 | 0.250 |
| K18707 | 0.023 | 0.066 | 0.45 | 0.052 | 0.073 | 0.270 | 0.001 | 0.072 | 0.470 |
| K18815 | 0.000 | 0.038 | 1 | 0.000 | 0.054 | 1.000 | 0.029 | 0.085 | 0.530 |
| K18828 | 0.068 | 0.076 | 0.2 | 0.054 | 0.080 | 0.280 | 0.087 | 0.113 | 0.260 |
| K18829 | 0.086 | 0.082 | 0.05 | 0.018 | 0.058 | 0.440 | 0.164 | 0.142 | 0.150 |
| K18831 | 0.029 | 0.055 | 0.41 | 0.058 | 0.089 | 0.260 | 0.000 | 0.069 | 1.000 |
| K18843 | 0.067 | 0.070 | 0.17 | 0.094 | 0.091 | 0.110 | 0.000 | 0.064 | 1.000 |
| K18929 | 0.095 | 0.066 | 0.07 | 0.066 | 0.082 | 0.320 | 0.143 | 0.129 | 0.130 |
| K18930 | 0.000 | 0.039 | 1 | 0.000 | 0.064 | 1.000 | 0.000 | 0.079 | 1.000 |
| K18954 | 0.098 | 0.071 | 0.09 | 0.097 | 0.095 | 0.130 | 0.111 | 0.099 | 0.240 |
| K19002 | 0.067 | 0.066 | 0.14 | 0.065 | 0.079 | 0.300 | 0.041 | 0.090 | 0.330 |
| K19005 | 0.012 | 0.050 | 0.54 | 0.000 | 0.058 | 1.000 | 0.027 | 0.086 | 0.430 |
| K19055 | 0.043 | 0.059 | 0.25 | 0.000 | 0.000 | 0.440 | 0.123 | 0.130 | 0.180 |
| K19068 | 0.076 | 0.073 | 0.14 | 0.106 | 0.094 | 0.100 | 0.000 | 0.060 | 1.000 |
| K19091 | 0.020 | 0.057 | 0.41 | 0.087 | 0.107 | 0.210 | 0.000 | 0.070 | 1.000 |
| K19092 | 0.000 | 0.048 | 1 | 0.004 | 0.057 | 0.500 | 0.000 | 0.085 | 1.000 |
| K19140 | 0.047 | 0.067 | 0.26 | 0.043 | 0.069 | 0.400 | 0.055 | 0.100 | 0.280 |
| K19147 | 0.019 | 0.059 | 0.4 | 0.060 | 0.074 | 0.310 | 0.000 | 0.083 | 1.000 |
| K19156 | 0.105 | 0.075 | 0.09 | 0.148 | 0.108 | 0.100 | 0.000 | 0.086 | 1.000 |
| K19157 | 0.076 | 0.078 | 0.15 | 0.012 | 0.060 | 0.530 | 0.196 | 0.144 | 0.090 |
| K19159 | 0.043 | 0.059 | 0.28 | 0.052 | 0.085 | 0.270 | 0.002 | 0.061 | 0.510 |
| K19167 | 0.000 | 0.043 | 1 | 0.044 | 0.082 | 0.390 | 0.000 | 0.081 | 1.000 |
| K19171 | 0.055 | 0.071 | 0.21 | 0.007 | 0.060 | 0.510 | 0.083 | 0.120 | 0.270 |
| K19172 | 0.051 | 0.062 | 0.25 | 0.049 | 0.083 | 0.290 | 0.117 | 0.118 | 0.240 |
| K19181 | 0.066 | 0.065 | 0.22 | 0.114 | 0.097 | 0.120 | 0.000 | 0.072 | 1.000 |
| K19221 | 0.117 | 0.082 | 0.06 | 0.164 | 0.116 | 0.050 | 0.062 | 0.106 | 0.320 |
| K19222 | 0.108 | 0.080 | 0.07 | 0.106 | 0.093 | 0.130 | 0.139 | 0.123 | 0.180 |
| K19225 | 0.054 | 0.066 | 0.2 | 0.077 | 0.089 | 0.170 | 0.000 | 0.082 | 1.000 |
| K19265 | 0.096 | 0.083 | 0.12 | 0.131 | 0.107 | 0.120 | 0.030 | 0.093 | 0.400 |
| K19294 | 0.031 | 0.060 | 0.37 | 0.127 | 0.099 | 0.120 | 0.000 | 0.085 | 1.000 |
| K19295 | 0.007 | 0.057 | 0.46 | 0.117 | 0.089 | 0.100 | 0.000 | 0.077 | 1.000 |
| K19302 | 0.000 | 0.048 | 1 | 0.000 | 0.050 | 1.000 | 0.000 | 0.074 | 1.000 |
| K19334 | 0.080 | 0.078 | 0.17 | 0.083 | 0.090 | 0.190 | 0.106 | 0.122 | 0.230 |
| K19350 | 0.048 | 0.051 | 0.26 | 0.131 | 0.094 | 0.090 | 0.000 | 0.074 | 1.000 |
| K19353 | 0.112 | 0.072 | 0.05 | 0.082 | 0.091 | 0.140 | 0.155 | 0.141 | 0.120 |
| K19405 | 0.008 | 0.042 | 0.42 | 0.071 | 0.081 | 0.240 | 0.000 | 0.092 | 1.000 |
| K19411 | 0.000 | 0.041 | 1 | 0.009 | 0.070 | 0.440 | 0.000 | 0.086 | 1.000 |
| K19545 | 0.000 | 0.051 | 1 | 0.000 | 0.060 | 1.000 | 0.042 | 0.099 | 0.380 |
| K19693 | 0.066 | 0.062 | 0.14 | 0.008 | 0.056 | 0.520 | 0.134 | 0.130 | 0.190 |
| K19784 | 0.059 | 0.063 | 0.19 | 0.074 | 0.098 | 0.220 | 0.000 | 0.000 | 0.970 |

| | | | | | | | | | |
|---|---|---|---|---|---|---|---|---|---|
| K19802 | 0.000 | 0.039 | 1 | 0.000 | 0.050 | 1.000 | 0.000 | 0.079 | 1.000 |
| K19824 | 0.000 | 0.049 | 1 | 0.000 | 0.056 | 1.000 | 0.026 | 0.076 | 0.410 |
| K19955 | 0.067 | 0.070 | 0.19 | 0.085 | 0.097 | 0.210 | 0.031 | 0.099 | 0.370 |
| K20074 | 0.025 | 0.050 | 0.4 | 0.000 | 0.000 | 0.520 | 0.060 | 0.101 | 0.360 |
| K20151 | 0.110 | 0.086 | 0.09 | 0.112 | 0.094 | 0.080 | 0.110 | 0.116 | 0.180 |
| K20276 | 0.000 | 0.043 | 1 | 0.000 | 0.053 | 1.000 | 0.199 | 0.146 | 0.080 |
| K20509 | 0.004 | 0.038 | 0.54 | 0.000 | 0.056 | 1.000 | 0.037 | 0.110 | 0.390 |
| K20534 | 0.013 | 0.052 | 0.45 | 0.003 | 0.042 | 0.620 | 0.021 | 0.076 | 0.490 |
| K21023 | 0.011 | 0.056 | 0.53 | 0.125 | 0.099 | 0.110 | 0.000 | 0.072 | 1.000 |
| K21029 | 0.054 | 0.063 | 0.2 | 0.048 | 0.081 | 0.300 | 0.089 | 0.129 | 0.170 |
| K21064 | 0.101 | 0.073 | 0.09 | 0.136 | 0.106 | 0.100 | 0.064 | 0.095 | 0.380 |
| K21140 | 0.092 | 0.078 | 0.14 | 0.059 | 0.082 | 0.220 | 0.198 | 0.147 | 0.060 |
| K21394 | 0.087 | 0.078 | 0.08 | 0.083 | 0.102 | 0.210 | 0.091 | 0.117 | 0.320 |
| K21470 | 0.078 | 0.071 | 0.13 | 0.111 | 0.110 | 0.130 | 0.000 | 0.075 | 1.000 |
| K21471 | 0.000 | 0.039 | 1 | 0.102 | 0.097 | 0.160 | 0.000 | 0.073 | 1.000 |
| K21556 | 0.089 | 0.077 | 0.15 | 0.112 | 0.100 | 0.150 | 0.000 | 0.063 | 1.000 |
| K21572 | 0.046 | 0.059 | 0.3 | 0.066 | 0.084 | 0.210 | 0.000 | 0.073 | 1.000 |
| K21573 | 0.042 | 0.057 | 0.27 | 0.062 | 0.083 | 0.270 | 0.010 | 0.064 | 0.520 |
| K21574 | 0.069 | 0.070 | 0.17 | 0.122 | 0.105 | 0.080 | 0.006 | 0.082 | 0.520 |
| K21636 | 0.069 | 0.075 | 0.15 | 0.000 | 0.050 | 1.000 | 0.178 | 0.152 | 0.070 |
| K21744 | 0.095 | 0.080 | 0.09 | 0.143 | 0.121 | 0.050 | 0.000 | 0.056 | 1.000 |
| K21745 | 0.000 | 0.046 | 1 | 0.000 | 0.059 | 1.000 | 0.000 | 0.065 | 1.000 |
| K21929 | 0.000 | 0.042 | 1 | 0.097 | 0.093 | 0.050 | 0.000 | 0.081 | 1.000 |
| K21993 | 0.062 | 0.064 | 0.2 | 0.005 | 0.061 | 0.520 | 0.170 | 0.143 | 0.070 |
| K22024 | 0.000 | 0.048 | 1 | 0.000 | 0.061 | 1.000 | 0.000 | 0.078 | 1.000 |
| K22162 | 0.129 | 0.080 | 0.02 | 0.147 | 0.112 | 0.060 | 0.124 | 0.115 | 0.170 |
| K22210 | 0.022 | 0.057 | 0.39 | 0.013 | 0.061 | 0.510 | 0.041 | 0.089 | 0.400 |
| K22213 | 0.048 | 0.061 | 0.21 | 0.045 | 0.078 | 0.300 | 0.043 | 0.078 | 0.450 |
| K22214 | 0.082 | 0.069 | 0.15 | 0.146 | 0.101 | 0.100 | 0.000 | 0.064 | 1.000 |
| K22278 | 0.000 | 0.042 | 1 | 0.000 | 0.070 | 1.000 | 0.000 | 0.075 | 1.000 |
| K22300 | 0.031 | 0.060 | 0.39 | 0.098 | 0.099 | 0.190 | 0.000 | 0.085 | 1.000 |
| K22390 | 0.118 | 0.092 | 0.08 | 0.136 | 0.104 | 0.070 | 0.071 | 0.111 | 0.290 |
| K22391 | 0.000 | 0.035 | 1 | 0.000 | 0.054 | 1.000 | 0.178 | 0.142 | 0.150 |
| K22405 | 0.000 | 0.040 | 1 | 0.000 | 0.060 | 1.000 | 0.017 | 0.076 | 0.450 |
| K22441 | 0.044 | 0.065 | 0.27 | 0.035 | 0.069 | 0.380 | 0.053 | 0.095 | 0.350 |
| K22477 | 0.000 | 0.038 | 1 | 0.013 | 0.077 | 0.440 | 0.000 | 0.064 | 1.000 |
| K22522 | 0.000 | 0.035 | 1 | 0.000 | 0.075 | 1.000 | 0.000 | 0.076 | 1.000 |
| K22719 | 0.000 | 0.043 | 1 | 0.000 | 0.061 | 1.000 | 0.000 | 0.077 | 1.000 |
| K22900 | 0.064 | 0.068 | 0.23 | 0.101 | 0.095 | 0.160 | 0.000 | 0.062 | 1.000 |
| K22927 | 0.099 | 0.074 | 0.1 | 0.160 | 0.116 | 0.060 | 0.031 | 0.088 | 0.500 |
| K22928 | 0.000 | 0.045 | 1 | 0.000 | 0.058 | 1.000 | 0.000 | 0.075 | 1.000 |
| K23004 | 0.020 | 0.053 | 0.44 | 0.003 | 0.045 | 0.590 | 0.086 | 0.112 | 0.320 |
| K23005 | 0.044 | 0.061 | 0.36 | 0.044 | 0.081 | 0.310 | 0.012 | 0.082 | 0.480 |
| K23010 | 0.000 | 0.046 | 1 | 0.000 | 0.059 | 1.000 | 0.000 | 0.083 | 1.000 |
| K23107 | 0.092 | 0.087 | 0.1 | 0.049 | 0.077 | 0.330 | 0.194 | 0.137 | 0.060 |
| K23237 | 0.000 | 0.044 | 1 | 0.030 | 0.066 | 0.430 | 0.000 | 0.064 | 1.000 |
| K23242 | 0.084 | 0.074 | 0.08 | 0.104 | 0.096 | 0.200 | 0.046 | 0.088 | 0.380 |
| K23264 | 0.066 | 0.063 | 0.17 | 0.057 | 0.074 | 0.250 | 0.094 | 0.127 | 0.230 |
| K23265 | 0.042 | 0.061 | 0.28 | 0.013 | 0.061 | 0.440 | 0.077 | 0.104 | 0.300 |
| K23352 | 0.026 | 0.059 | 0.35 | 0.120 | 0.096 | 0.100 | 0.000 | 0.071 | 1.000 |
| K23356 | 0.099 | 0.076 | 0.07 | 0.105 | 0.091 | 0.140 | 0.088 | 0.103 | 0.270 |
| K23518 | 0.074 | 0.062 | 0.16 | 0.120 | 0.112 | 0.120 | 0.000 | 0.083 | 1.000 |
| K23684 | 0.024 | 0.052 | 0.4 | 0.086 | 0.094 | 0.160 | 0.000 | 0.075 | 1.000 |

| | | | | | | | | | |
|---|---|---|---|---|---|---|---|---|---|
| K23775 | 0.112 | 0.076 | 0.06 | 0.111 | 0.118 | 0.090 | 0.087 | 0.125 | 0.260 |
| K23999 | 0.000 | 0.041 | 1 | 0.000 | 0.053 | 1.000 | 0.117 | 0.119 | 0.240 |
| K24131 | 0.165 | 0.098 | 0.03 | 0.162 | 0.105 | 0.060 | 0.166 | 0.146 | 0.100 |
| K24258 | 0.090 | 0.074 | 0.14 | 0.106 | 0.087 | 0.130 | 0.000 | 0.078 | 1.000 |

Abundances of microbial genera and genes were transformed using the centred logratio or the additive logratio (reference K01783), respectively. Subscripts BOTH, CON and FOR correspond to microbial genera or genes with significant repeatability in both diets, exclusively concentrate-fed, or exclusively forage-fed animals, respectively.

**Supplementary Table 2. Pearson correlations of centred logratios of 1050 microbial genera abundances (above diagonal) and additive logratios of 1901 microbial genes abundances (below the diagonal).**

| | T1 | T2 | T3 | T4 | T5 | T6 |
|---|---|---|---|---|---|---|
| T1 | | 0.322 | 0.117 | 0.164 | 0.242 | 0.200 |
| T2 | 0.157 | | 0.329 | 0.344 | 0.401 | 0.303 |
| T3 | 0.109 | 0.248 | | 0.593 | 0.375 | 0.351 |
| T4 | 0.120 | 0.250 | 0.472 | | 0.465 | 0.367 |
| T5 | 0.138 | 0.268 | 0.175 | 0.260 | | 0.373 |
| T6 | 0.089 | 0.176 | 0.206 | 0.213 | 0.247 | |

**Supplementary table 3 - A. Variable importance in projection (VIP) and regression coefficients from partial least squares analyses of significantly repeatable microbial genera with stable associations with host performance traits.**

**Trait: Feed Conversion Ratio (FCR); Predictors: 224 microbial genera (MT)**

| Microbial genera | VIP | | | | | | Regression coefficient | | | | | |
|---|---|---|---|---|---|---|---|---|---|---|---|---|
| | T1 | T2 | T3 | T4 | T5 | T6 | T1 | T2 | T3 | T4 | T5 | T6 |
| Methanolacinia$_{VIP\geq0.8\ (+)}$ | 1.82 | 0.51 | 0.81 | 1.42 | 1.31 | 1.27 | 0.04 | 0.03 | 0.00 | 0.02 | 0.03 | 0.03 |
| Corynebacterium$_{VIP\geq0.8\ (+)}$ | 0.61 | 0.42 | 1.39 | 0.85 | 1.04 | 1.37 | 0.01 | 0.03 | 0.02 | 0.00 | -0.01 | 0.02 |
| Magnetospira$_{VIP\geq0.8\ (+)}$ | 1.49 | 1.07 | 1.43 | 1.85 | 1.31 | 1.34 | 0.03 | -0.02 | 0.03 | 0.03 | 0.03 | 0.03 |
| Serpula$_{VIP\geq0.8\ (+)}$ | 0.90 | 0.38 | 1.47 | 1.54 | 2.36 | 0.99 | -0.02 | -0.01 | 0.04 | 0.03 | 0.05 | 0.02 |
| Paucibacter$_{VIP\geq0.8\ (+)}$ | 0.25 | 0.82 | 1.65 | 1.72 | 0.99 | 0.83 | 0.00 | 0.05 | 0.04 | 0.03 | 0.02 | 0.02 |
| Natrialba$_{VIP\geq0.8\ (+)}$ | 2.03 | 1.58 | 1.28 | 0.90 | 0.67 | 0.20 | 0.04 | 0.07 | 0.03 | 0.01 | 0.01 | 0.00 |
| Sulfitobacter$_{VIP\geq0.8\ (+)}$ | 1.36 | 1.88 | 0.75 | 0.37 | 0.91 | 0.91 | 0.03 | 0.09 | 0.02 | -0.01 | 0.02 | 0.02 |
| Moorella$_{VIP\geq0.8\ (+)}$ | 0.67 | 0.82 | 1.84 | 1.53 | 0.57 | 1.28 | 0.01 | -0.01 | 0.04 | 0.03 | -0.01 | 0.03 |
| Laccaria$_{VIP\geq0.8\ (+)}$ | 1.14 | 0.53 | 1.12 | 0.94 | 0.75 | 1.45 | 0.02 | -0.03 | 0.04 | 0.02 | -0.01 | 0.03 |
| Niastella$_{VIP\geq0.8\ (+)}$ | 0.94 | 0.89 | 0.94 | 1.01 | 1.29 | 0.81 | 0.01 | -0.02 | 0.03 | 0.02 | 0.03 | -0.01 |
| Thielavia$_{VIP\geq0.8\ (+)}$ | 0.89 | 1.02 | 1.00 | 0.81 | 0.92 | 0.53 | 0.02 | 0.01 | 0.03 | 0.01 | 0.01 | 0.00 |
| Methanoculleus$_{VIP\geq0.8\ (+)}$ | 0.92 | 0.60 | 2.25 | 0.61 | 1.18 | 1.40 | 0.02 | 0.00 | 0.05 | -0.01 | 0.03 | 0.03 |
| Methanolobus$_{VIP\geq0.8\ (+)}$ | 2.31 | 1.13 | 1.71 | 0.48 | 1.22 | 0.59 | 0.05 | 0.02 | 0.05 | 0.01 | -0.03 | 0.01 |
| Fonsecaea$_{VIP\geq0.8\ (+)}$ | 0.91 | 1.00 | 0.60 | 0.90 | 0.41 | 1.02 | 0.02 | 0.02 | 0.01 | 0.02 | 0.00 | 0.02 |
| Hoyosella$_{VIP\geq0.8\ (+)}$ | 0.86 | 0.80 | 0.90 | 1.85 | 1.05 | 1.12 | 0.02 | 0.04 | 0.01 | 0.03 | 0.02 | -0.02 |
| Acetomicrobium$_{VIP\geq0.8\ (+)}$ | 0.68 | 0.92 | 1.06 | 1.03 | 0.88 | 1.65 | -0.01 | -0.02 | 0.03 | 0.02 | 0.02 | 0.03 |
| Gemmatimonas$_{VIP\geq0.8\ (+)}$ | 0.97 | 0.80 | 1.45 | 0.96 | 0.44 | 1.09 | 0.02 | -0.02 | 0.03 | 0.01 | 0.01 | 0.02 |
| Kingella$_{VIP\geq0.8\ (+)}$ | 1.08 | 0.73 | 1.19 | 1.07 | 0.99 | 0.52 | -0.02 | 0.03 | 0.03 | 0.02 | 0.02 | 0.01 |
| Hammondia$_{VIP\geq0.8\ (+)}$ | 1.40 | 1.30 | 0.68 | 0.80 | 1.30 | 1.02 | -0.03 | 0.02 | 0.02 | 0.01 | 0.02 | 0.02 |
| Chloroflexus$_{VIP\geq0.8\ (+)}$ | 1.50 | 0.72 | 1.31 | 1.25 | 1.03 | 0.42 | 0.03 | -0.01 | 0.03 | 0.02 | 0.02 | -0.01 |
| Pelagibaca$_{VIP\geq0.8\ (+)}$ | 2.04 | 1.06 | 1.65 | 1.05 | 0.74 | 0.59 | 0.04 | -0.02 | 0.04 | 0.01 | 0.01 | -0.01 |
| Sphaerochaeta$_{VIP\geq0.8\ (+)}$ | 0.40 | 0.74 | 0.85 | 1.34 | 0.82 | 1.10 | 0.01 | 0.00 | 0.01 | 0.02 | 0.01 | 0.02 |
| Sphaerulina$_{VIP\geq0.8\ (+)}$ | 0.58 | 0.47 | 0.95 | 0.81 | 0.80 | 0.91 | 0.01 | -0.03 | 0.03 | 0.00 | 0.01 | 0.01 |

| | | | | | | | | | | | | |
|---|---|---|---|---|---|---|---|---|---|---|---|---|
| Ruminiclostridium$_{VIP\geq0.8\ (+)}$ | 0.74 | 1.33 | 0.90 | 1.55 | 0.32 | 0.84 | 0.01 | -0.05 | 0.02 | 0.03 | -0.01 | 0.02 |
| Thalassospira$_{VIP\geq0.8\ (+)}$ | 1.06 | 1.40 | 0.74 | 1.11 | 1.70 | 0.92 | 0.02 | 0.06 | 0.01 | 0.02 | -0.04 | 0.01 |
| Carboxydothermus$_{VIP\geq0.8\ (+)}$ | 2.01 | 1.55 | 0.90 | 1.25 | 0.52 | 0.88 | 0.04 | -0.03 | 0.01 | 0.02 | 0.01 | -0.02 |
| Candidatus Methanomethylc | 0.36 | 0.45 | 1.15 | 0.86 | 1.19 | 1.17 | 0.00 | 0.03 | 0.01 | 0.00 | 0.02 | 0.02 |
| Wenyingzhuangia$_{VIP\geq0.8\ (+)}$ | 1.10 | 1.84 | 1.21 | 0.45 | 0.51 | 1.78 | 0.02 | 0.06 | 0.04 | 0.01 | -0.01 | -0.04 |
| Azospira$_{VIP\geq0.8\ (+)}$ | 0.87 | 0.70 | 1.03 | 0.87 | 0.75 | 0.99 | 0.00 | 0.00 | 0.00 | 0.00 | 0.00 | -0.01 |
| Tolumonas$_{VIP\geq0.8\ (+)}$ | 0.87 | 0.71 | 0.95 | 0.80 | 0.54 | 1.05 | 0.00 | 0.00 | 0.00 | 0.00 | 0.00 | -0.01 |
| Octadecabacter$_{VIP\geq0.8\ (+)}$ | 1.29 | 0.97 | 1.63 | 0.57 | 1.81 | 0.78 | 0.02 | 0.01 | 0.05 | 0.01 | -0.04 | -0.01 |
| Methanohalophilus$_{VIP\geq0.8\ (+)}$ | 0.72 | 1.69 | 1.04 | 0.88 | 0.60 | 0.91 | -0.01 | -0.05 | 0.03 | 0.01 | 0.01 | 0.02 |
| Desulfitobacterium$_{VIP\geq0.8\ (+)}$ | 0.12 | 0.85 | 0.82 | 1.23 | 1.13 | 0.35 | 0.00 | -0.01 | 0.01 | 0.02 | 0.02 | 0.00 |
| Chromobacterium$_{VIP\geq0.8\ (+)}$ | 0.92 | 0.68 | 0.93 | 0.89 | 0.70 | 0.96 | 0.01 | 0.01 | 0.00 | -0.01 | 0.00 | 0.00 |
| Shimwellia$_{VIP\geq0.8\ (+)}$ | 0.90 | 0.57 | 1.02 | 0.89 | 0.68 | 0.98 | 0.01 | 0.01 | 0.01 | -0.01 | 0.01 | -0.01 |
| Phycisphaera$_{VIP\geq0.8\ (+)}$ | 0.95 | 1.79 | 1.33 | 0.11 | 2.14 | 0.97 | 0.02 | 0.06 | 0.03 | 0.00 | -0.05 | 0.02 |
| Thiomonas$_{VIP\geq0.8\ (+)}$ | 0.80 | 0.82 | 0.98 | 1.23 | 1.27 | 0.99 | -0.02 | 0.00 | 0.01 | 0.01 | 0.03 | 0.01 |
| Moesziomyces$_{VIP\geq0.8\ (+)}$ | 0.64 | 1.37 | 1.00 | 1.35 | 0.96 | 0.99 | 0.01 | -0.03 | 0.01 | 0.02 | 0.02 | -0.01 |
| Sedimenticola$_{VIP\geq0.8\ (+)}$ | 0.88 | 0.67 | 0.89 | 0.94 | 0.53 | 0.99 | 0.01 | 0.00 | 0.00 | 0.00 | 0.00 | 0.01 |
| Sphingobium$_{VIP\geq0.8\ (+)}$ | 0.47 | 1.57 | 1.26 | 0.82 | 0.93 | 1.24 | 0.00 | -0.04 | 0.02 | 0.00 | 0.02 | 0.02 |
| Chthonomonas$_{VIP\geq0.8\ (+)}$ | 0.97 | 0.78 | 1.06 | 0.88 | 1.68 | 1.01 | 0.00 | 0.00 | 0.01 | 0.00 | 0.04 | -0.01 |
| Propionibacterium$_{VIP\geq0.8\ (+)}$ | 0.70 | 0.89 | 1.10 | 0.74 | 1.39 | 1.00 | 0.01 | 0.05 | 0.01 | -0.01 | -0.03 | 0.02 |
| Methyloversatilis$_{VIP\geq0.8\ (-)}$ | 0.80 | 1.49 | 0.81 | 1.20 | 0.73 | 2.24 | -0.01 | -0.03 | -0.02 | -0.02 | 0.01 | -0.05 |
| Pseudomonas$_{VIP\geq0.8\ (-)}$ | 1.36 | 0.87 | 0.94 | 0.85 | 0.73 | 1.02 | -0.02 | 0.00 | 0.00 | -0.01 | -0.01 | -0.01 |
| Fibrobacter$_{VIP\geq0.8\ (-)}$ | 0.95 | 0.76 | 0.63 | 1.34 | 0.92 | 1.03 | -0.02 | -0.04 | 0.00 | -0.02 | -0.02 | -0.01 |
| Gordonibacter$_{VIP\geq0.8\ (-)}$ | 0.88 | 0.52 | 0.72 | 0.84 | 2.55 | 0.92 | 0.02 | -0.01 | -0.01 | -0.01 | -0.06 | -0.02 |
| Cryptobacterium$_{VIP\geq0.8\ (-)}$ | 1.02 | 1.71 | 0.82 | 0.73 | 2.04 | 1.42 | 0.02 | -0.06 | 0.00 | -0.01 | -0.05 | -0.03 |
| Eggerthella$_{VIP\geq0.8\ (-)}$ | 0.50 | 0.82 | 0.85 | 1.04 | 2.07 | 0.92 | 0.01 | -0.02 | 0.00 | -0.02 | -0.05 | -0.02 |
| Methylococcus$_{VIP\geq0.8\ (-)}$ | 1.94 | 0.34 | 0.85 | 1.10 | 0.97 | 0.80 | -0.04 | 0.02 | 0.00 | -0.02 | -0.01 | -0.01 |
| Acidothermus$_{VIP\geq0.8\ (-)}$ | 1.22 | 1.15 | 0.97 | 0.88 | 0.88 | 0.46 | -0.02 | -0.02 | -0.03 | -0.01 | -0.01 | 0.00 |
| Acidovorax$_{VIP\geq0.8\ (-)}$ | 1.39 | 0.81 | 0.80 | 1.10 | 0.76 | 0.95 | -0.03 | 0.00 | -0.01 | -0.02 | 0.01 | -0.02 |
| Blastomonas$_{VIP\geq0.8\ (-)}$ | 1.51 | 0.49 | 0.93 | 0.82 | 0.68 | 1.06 | -0.02 | 0.03 | -0.03 | -0.01 | -0.01 | -0.02 |
| Raoultella$_{VIP\geq0.8\ (-)}$ | 1.15 | 0.51 | 0.84 | 0.81 | 0.68 | 1.01 | -0.01 | 0.02 | -0.02 | -0.01 | 0.00 | -0.01 |

| | | | | | | | | | | | | |
|---|---|---|---|---|---|---|---|---|---|---|---|---|
| Leclercia$_{VIP\geq0.8\ (-)}$ | 2.16 | 0.46 | 0.87 | 0.88 | 0.68 | 1.04 | -0.04 | 0.02 | -0.01 | 0.00 | 0.00 | -0.01 |
| Cedecea$_{VIP\geq0.8\ (-)}$ | 1.09 | 0.99 | 0.94 | 0.75 | 0.55 | 1.02 | -0.02 | -0.01 | 0.00 | 0.00 | -0.01 | -0.01 |
| Alteromonas$_{VIP\geq0.8\ (-)}$ | 1.49 | 0.81 | 1.06 | 0.67 | 0.90 | 0.94 | -0.03 | -0.01 | 0.02 | 0.00 | -0.01 | -0.01 |
| Cladophialophora$_{VIP\geq0.8\ (-)}$ | 0.76 | 2.01 | 0.52 | 1.34 | 1.59 | 1.20 | -0.01 | -0.06 | -0.02 | -0.03 | 0.04 | -0.02 |
| Ralstonia$_{VIP\geq0.8\ (-)}$ | 1.00 | 0.73 | 0.84 | 0.88 | 0.86 | 0.99 | -0.01 | 0.00 | -0.01 | -0.01 | -0.01 | 0.00 |
| Thauera$_{VIP\geq0.8\ (-)}$ | 1.19 | 0.99 | 0.81 | 0.85 | 0.72 | 0.93 | -0.01 | -0.01 | -0.02 | 0.00 | 0.00 | 0.00 |
| Salinispira$_{VIP\geq0.8\ (-)}$ | 1.37 | 1.37 | 1.01 | 0.87 | 0.76 | 1.00 | -0.02 | -0.03 | 0.00 | -0.01 | 0.00 | 0.00 |
| Rhodomicrobium$_{VIP\geq0.8\ (-)}$ | 1.43 | 0.65 | 1.24 | 0.91 | 0.69 | 0.98 | -0.02 | 0.00 | 0.01 | -0.01 | 0.00 | -0.01 |
| Defluviimonas$_{VIP\geq0.8\ (-)}$ | 0.76 | 0.89 | 0.81 | 0.92 | 0.66 | 1.00 | 0.00 | 0.00 | -0.01 | -0.02 | 0.00 | -0.01 |
| Pasteurella$_{VIP\geq0.8\ (-)}$ | 1.38 | 1.10 | 0.88 | 0.91 | 0.72 | 0.43 | -0.03 | -0.04 | -0.03 | -0.01 | 0.01 | -0.01 |
| Methanomassiliicoccus$_{VIP\geq0.8}$ | 0.75 | 1.02 | 0.92 | 0.94 | 0.78 | 0.97 | 0.00 | -0.01 | 0.00 | -0.01 | -0.01 | -0.01 |
| Zhongshania$_{VIP\geq0.8\ (-)}$ | 1.18 | 1.16 | 0.76 | 1.09 | 0.65 | 1.11 | -0.02 | -0.02 | -0.01 | 0.01 | 0.01 | -0.02 |
| Sulfurovum$_{VIP\geq0.8\ (-)}$ | 1.50 | 1.26 | 0.40 | 1.03 | 1.06 | 0.53 | -0.03 | -0.03 | -0.01 | 0.01 | -0.02 | -0.01 |
| Hyphomonas$_{VIP\geq0.8\ (-)}$ | 0.94 | 0.72 | 1.05 | 0.87 | 0.76 | 0.81 | 0.00 | 0.00 | -0.03 | -0.01 | 0.01 | 0.00 |
| Stigmatella$_{VIP\geq0.8\ (-)}$ | 0.80 | 0.65 | 1.10 | 0.85 | 1.25 | 0.95 | -0.01 | 0.01 | 0.01 | 0.00 | -0.02 | -0.01 |
| Eutypa$_{VIP\geq0.8\ (-)}$ | 1.25 | 0.61 | 1.45 | 1.28 | 0.77 | 0.87 | -0.03 | -0.01 | -0.03 | -0.01 | 0.00 | -0.01 |
| Rhodobacter$_{VIP\geq0.8\ (-)}$ | 0.81 | 1.14 | 0.89 | 0.95 | 0.77 | 1.00 | 0.00 | -0.01 | -0.01 | -0.01 | 0.00 | 0.01 |
| Methyloceanibacter$_{VIP\geq0.8\ (-)}$ | 0.91 | 1.16 | 0.82 | 0.87 | 0.69 | 0.92 | 0.00 | -0.02 | -0.02 | -0.01 | 0.01 | -0.01 |
| Adlercreutzia$_{VIP\geq0.8\ (-)}$ | 0.54 | 0.96 | 0.91 | 0.81 | 1.05 | 0.91 | 0.01 | -0.01 | 0.00 | 0.00 | -0.02 | -0.01 |
| Pannonibacter$_{VIP\geq0.8\ (-)}$ | 0.96 | 0.95 | 0.87 | 0.86 | 0.70 | 1.01 | -0.01 | -0.01 | -0.02 | 0.00 | 0.00 | 0.00 |
| Dickeya$_{VIP\geq0.8\ (-)}$ | 1.05 | 0.87 | 0.81 | 0.86 | 0.89 | 1.08 | -0.01 | 0.00 | -0.02 | 0.00 | 0.01 | -0.01 |
| Sideroxydans$_{VIP\geq0.8\ (-)}$ | 1.11 | 0.76 | 0.89 | 0.89 | 0.68 | 1.12 | -0.01 | 0.00 | 0.00 | 0.00 | 0.01 | -0.01 |
| Cyanothece$_{VIP\geq0.8\ (-)}$ | 0.91 | 0.96 | 1.45 | 0.82 | 1.03 | 1.50 | -0.02 | 0.01 | -0.02 | 0.00 | -0.02 | -0.02 |
| Dermabacter$_{VIP\geq0.8\ (-)}$ | 1.02 | 0.93 | 0.86 | 0.84 | 0.79 | 1.07 | 0.00 | -0.01 | -0.01 | -0.01 | 0.01 | -0.01 |
| Scheffersomyces$_{VIP\geq0.8\ (-)}$ | 0.82 | 1.27 | 1.08 | 0.89 | 0.74 | 1.30 | -0.02 | -0.07 | -0.01 | -0.01 | 0.00 | -0.02 |
| Nitrobacter$_{VIP\geq0.8\ (-)}$ | 0.80 | 1.10 | 0.85 | 0.87 | 0.80 | 0.99 | 0.00 | -0.01 | -0.02 | -0.01 | 0.01 | 0.00 |
| Halotalea$_{VIP\geq0.8\ (-)}$ | 0.85 | 0.77 | 0.85 | 0.84 | 0.70 | 1.02 | 0.00 | 0.00 | -0.02 | 0.00 | 0.01 | -0.01 |
| Sanguibacter$_{VIP\geq0.8\ (-)}$ | 1.20 | 0.66 | 0.85 | 0.83 | 0.83 | 0.96 | -0.02 | 0.01 | -0.01 | -0.01 | 0.01 | 0.00 |
| Moraxella$_{VIP\geq0.8\ (-)}$ | 1.24 | 1.16 | 0.90 | 0.82 | 0.39 | 1.02 | -0.02 | -0.02 | 0.00 | 0.00 | 0.01 | -0.01 |
| Acaryochloris$_{VIP\geq0.8\ (-)}$ | 1.01 | 1.39 | 0.88 | 0.89 | 0.73 | 0.95 | 0.01 | -0.03 | -0.01 | 0.00 | -0.01 | -0.01 |

| | | | | | | | | | | | | |
|---|---|---|---|---|---|---|---|---|---|---|---|---|
| Halolamina$_{VIP\geq0.8\ (-)}$ | 1.05 | 0.94 | 1.07 | 0.87 | 0.77 | 1.01 | -0.01 | -0.01 | 0.00 | -0.01 | 0.01 | -0.01 |
| Modestobacter$_{VIP\geq0.8\ (-)}$ | 0.92 | 0.80 | 0.87 | 0.88 | 0.73 | 1.03 | 0.00 | 0.00 | -0.01 | -0.01 | 0.00 | -0.01 |
| Chelativorans$_{VIP\geq0.8\ (-)}$ | 1.12 | 0.62 | 0.84 | 0.84 | 0.81 | 0.93 | -0.01 | 0.01 | -0.01 | -0.01 | 0.01 | -0.01 |
| Lachnobacterium$_{VIP\geq0.8\ (-)}$ | 1.33 | 0.90 | 0.77 | 1.07 | 0.79 | 1.52 | -0.03 | 0.04 | -0.02 | 0.02 | -0.02 | -0.03 |
| Immundisolibacter$_{VIP\geq0.8\ (-)}$ | 1.09 | 0.88 | 0.84 | 0.90 | 0.67 | 1.02 | -0.01 | -0.01 | -0.01 | 0.00 | 0.01 | 0.00 |
| Spizellomyces$_{VIP\geq0.8\ (-)}$ | 0.52 | 0.55 | 1.15 | 0.94 | 0.98 | 1.20 | -0.01 | -0.01 | -0.01 | -0.01 | -0.02 | -0.02 |
| Azoarcus$_{VIP\geq0.8\ (-)}$ | 0.83 | 0.56 | 0.82 | 0.88 | 0.65 | 1.00 | 0.00 | 0.01 | -0.01 | -0.01 | 0.00 | 0.00 |
| Burkholderia$_{VIP\geq0.8\ (-)}$ | 0.94 | 0.60 | 0.77 | 0.85 | 0.97 | 0.95 | -0.01 | 0.01 | -0.01 | 0.00 | -0.01 | 0.01 |
| Kalmanozyma$_{VIP\geq0.8\ (-)}$ | 1.01 | 1.03 | 0.85 | 0.80 | 0.67 | 1.04 | -0.01 | -0.01 | 0.00 | 0.00 | 0.01 | -0.01 |
| Mesorhizobium$_{VIP\geq0.8\ (-)}$ | 0.89 | 0.61 | 0.85 | 0.90 | 0.74 | 0.98 | -0.01 | 0.01 | -0.01 | 0.00 | 0.00 | 0.00 |
| Microbulbifer$_{VIP\geq0.8\ (-)}$ | 0.95 | 1.14 | 0.85 | 0.86 | 0.66 | 1.03 | 0.00 | -0.01 | -0.01 | -0.01 | 0.01 | -0.01 |
| Aeromonas$_{VIP\geq0.8\ (-)}$ | 1.00 | 0.77 | 0.96 | 0.87 | 0.68 | 1.03 | -0.01 | 0.00 | 0.00 | 0.00 | 0.01 | -0.01 |
| Chamaesiphon$_{VIP\geq0.8\ (-)}$ | 0.51 | 1.51 | 0.82 | 1.46 | 1.24 | 0.77 | -0.01 | 0.03 | 0.00 | -0.02 | -0.03 | -0.01 |
| Neisseria$_{VIP\geq0.8\ (-)}$ | 1.59 | 0.88 | 0.88 | 0.89 | 0.89 | 0.98 | -0.03 | 0.00 | 0.00 | 0.00 | 0.02 | -0.01 |
| Pneumocystis$_{VIP\geq0.8\ (-)}$ | 0.76 | 0.90 | 1.56 | 1.14 | 0.86 | 0.84 | -0.01 | 0.01 | -0.03 | -0.01 | -0.01 | 0.00 |
| Rhodanobacter$_{VIP\geq0.8\ (-)}$ | 0.84 | 0.47 | 0.85 | 0.83 | 0.57 | 0.97 | 0.00 | 0.03 | 0.00 | -0.01 | -0.01 | -0.01 |
| Wenzhouxiangella$_{VIP\geq0.8\ (-)}$ | 0.96 | 1.10 | 1.04 | 0.91 | 0.74 | 1.07 | 0.00 | -0.01 | 0.00 | 0.00 | 0.01 | -0.01 |
| Agrobacterium$_{VIP\geq0.8\ (-)}$ | 0.94 | 1.15 | 0.81 | 0.90 | 0.71 | 1.00 | 0.01 | -0.01 | -0.01 | 0.00 | 0.00 | 0.00 |
| Nitrosospira$_{VIP\geq0.8\ (-)}$ | 0.99 | 0.75 | 0.88 | 0.89 | 0.73 | 1.03 | 0.00 | 0.00 | -0.01 | 0.00 | 0.01 | -0.01 |
| Candidatus Cloacimonas$_{VIP\geq}$ | 1.10 | 0.90 | 0.84 | 1.09 | 0.58 | 1.21 | -0.02 | 0.02 | -0.01 | -0.02 | 0.01 | -0.02 |
| Herbaspirillum$_{VIP\geq0.8\ (-)}$ | 1.04 | 1.03 | 1.40 | 0.79 | 0.76 | 0.99 | -0.02 | -0.01 | 0.02 | 0.00 | -0.01 | 0.00 |
| Halomonas$_{VIP\geq0.8\ (-)}$ | 0.81 | 0.93 | 0.81 | 0.82 | 0.67 | 1.05 | 0.00 | -0.01 | -0.01 | -0.01 | 0.01 | 0.00 |
| Haemophilus$_{VIP\geq0.8\ (-)}$ | 1.21 | 1.82 | 0.76 | 0.70 | 0.88 | 1.03 | -0.02 | -0.09 | -0.01 | 0.01 | 0.02 | -0.02 |
| Azospirillum$_{VIP\geq0.8\ (-)}$ | 0.64 | 0.83 | 0.91 | 0.90 | 0.73 | 1.18 | 0.00 | 0.00 | 0.00 | -0.01 | 0.00 | 0.02 |
| Paraburkholderia$_{VIP\geq0.8\ (-)}$ | 0.80 | 1.36 | 0.88 | 0.77 | 0.95 | 0.88 | 0.00 | -0.03 | 0.00 | -0.01 | 0.02 | -0.01 |
| Halosimplex$_{VIP\geq0.8\ (-)}$ | 0.90 | 1.12 | 0.93 | 0.82 | 0.86 | 1.04 | 0.00 | -0.01 | 0.00 | -0.01 | 0.02 | -0.01 |
| Anabaena$_{VIP\geq0.8\ (-)}$ | 0.86 | 0.57 | 0.78 | 0.96 | 1.22 | 0.84 | -0.02 | -0.01 | 0.01 | -0.01 | -0.03 | 0.00 |
| Delftia$_{VIP\geq0.8\ (-)}$ | 0.90 | 0.72 | 0.99 | 1.10 | 0.37 | 1.11 | -0.02 | 0.00 | -0.03 | 0.01 | 0.00 | 0.01 |
| Rubrobacter$_{VIP\geq0.8\ (-)}$ | 1.07 | 0.79 | 0.80 | 0.91 | 1.36 | 0.39 | -0.01 | 0.00 | -0.01 | -0.01 | 0.03 | 0.00 |
| Tsukamurella$_{VIP\geq0.8\ (-)}$ | 0.92 | 0.95 | 0.84 | 0.86 | 1.00 | 0.94 | 0.01 | -0.01 | -0.01 | -0.01 | 0.02 | -0.01 |

| | | | | | | | | | | | | |
|---|---|---|---|---|---|---|---|---|---|---|---|---|
| Bradyrhizobium$_{VIP≥0.8\ (-)}$ | 0.61 | 0.95 | 0.86 | 0.78 | 0.86 | 1.23 | 0.01 | -0.01 | 0.00 | -0.01 | -0.01 | 0.01 |
| Trichodesmium$_{VIP≥0.8\ (-)}$ | 0.61 | 0.85 | 0.95 | 0.97 | 0.74 | 1.08 | -0.01 | 0.01 | 0.00 | -0.01 | -0.01 | -0.01 |
| Debaryomyces$_{VIP≥0.8\ (-)}$ | 1.20 | 0.66 | 1.24 | 0.92 | 0.91 | 0.58 | -0.02 | -0.01 | -0.01 | 0.00 | -0.01 | 0.00 |
| Wallemia$_{VIP≥0.8\ (-)}$ | 0.50 | 0.73 | 0.87 | 1.08 | 0.80 | 0.90 | 0.00 | 0.00 | -0.01 | -0.01 | -0.01 | -0.01 |
| Naumovozyma$_{VIP≥0.8\ (-)}$ | 0.82 | 0.67 | 1.40 | 0.97 | 1.23 | 0.82 | -0.01 | -0.04 | -0.02 | -0.01 | 0.02 | -0.01 |
| Chelatococcus$_{VIP≥0.8\ (-)}$ | 1.06 | 0.66 | 0.80 | 0.84 | 0.41 | 0.85 | 0.02 | 0.01 | 0.00 | 0.00 | 0.00 | 0.00 |
| Tetrapisispora$_{VIP≥0.8\ (-)}$ | 0.88 | 0.80 | 0.83 | 1.22 | 0.80 | 0.81 | -0.02 | 0.00 | 0.00 | -0.01 | 0.00 | 0.00 |
| Shinella$_{VIP≥0.8\ (-)}$ | 0.69 | 1.61 | 0.31 | 1.24 | 0.90 | 1.66 | -0.01 | -0.04 | 0.00 | 0.02 | -0.02 | 0.03 |
| Entamoeba$_{VIP≥0.8\ (-)}$ | 1.05 | 0.57 | 1.21 | 0.89 | 0.81 | 0.74 | -0.02 | -0.01 | -0.02 | 0.00 | 0.00 | 0.00 |
| Archangium$_{VIP≥0.8\ (-)}$ | 0.94 | 1.01 | 0.86 | 0.82 | 0.83 | 0.79 | 0.02 | -0.01 | 0.00 | 0.00 | -0.01 | 0.01 |
| Nakaseomyces$_{VIP≥0.8\ (-)}$ | 1.34 | 0.45 | 1.17 | 1.05 | 0.85 | 0.96 | 0.02 | -0.03 | -0.01 | -0.01 | -0.01 | -0.01 |
| Thermothelomyces$_{VIP≥0.8\ (-)}$ | 0.75 | 0.40 | 0.80 | 0.93 | 0.96 | 1.22 | 0.01 | -0.01 | 0.00 | -0.01 | 0.02 | -0.02 |
| Melampsora$_{VIP≥0.8\ (-)}$ | 0.86 | 0.82 | 1.10 | 1.01 | 0.69 | 0.87 | -0.02 | 0.00 | -0.01 | -0.01 | -0.01 | 0.01 |
| Aspergillus$_{VIP≥0.8\ (-)}$ | 0.36 | 0.62 | 0.91 | 0.88 | 0.88 | 0.87 | 0.00 | 0.00 | -0.01 | -0.01 | 0.01 | -0.01 |
| Blastocystis$_{VIP≥0.8\ (-)}$ | 0.56 | 0.89 | 1.02 | 1.02 | 1.02 | 0.83 | -0.01 | 0.00 | -0.01 | -0.01 | 0.01 | -0.01 |
| Anaeromyxobacter$_{VIP≥0.8}$ | 0.83 | 0.84 | 1.03 | 1.02 | 1.14 | 1.05 | 0.01 | 0.00 | 0.01 | -0.02 | -0.02 | 0.01 |
| Alcanivorax$_{VIP≥0.8}$ | 1.77 | 0.74 | 0.91 | 0.84 | 0.93 | 1.01 | -0.03 | 0.00 | 0.00 | 0.00 | 0.02 | -0.01 |
| Arsenicicoccus$_{VIP≥0.8}$ | 2.52 | 0.95 | 1.01 | 1.38 | 0.63 | 1.57 | 0.05 | -0.02 | 0.02 | -0.03 | 0.00 | 0.03 |
| Candidatus Methanoperedeı | 0.87 | 1.06 | 0.85 | 0.98 | 0.64 | 1.00 | -0.01 | -0.01 | 0.00 | 0.00 | 0.00 | -0.01 |
| Dokdonella$_{VIP≥0.8}$ | 1.00 | 0.86 | 1.02 | 0.86 | 0.75 | 0.99 | 0.01 | 0.00 | 0.00 | -0.01 | 0.01 | 0.00 |
| Haloquadratum$_{VIP≥0.8}$ | 0.70 | 2.68 | 0.93 | 1.77 | 1.29 | 1.15 | 0.01 | 0.08 | -0.01 | -0.03 | -0.03 | 0.02 |
| Halorhodospira$_{VIP≥0.8}$ | 0.88 | 0.88 | 0.98 | 0.90 | 0.70 | 1.02 | 0.01 | 0.00 | 0.00 | 0.00 | 0.00 | 0.00 |
| Thioalkalivibrio$_{VIP≥0.8}$ | 0.85 | 0.82 | 0.82 | 0.89 | 0.77 | 0.97 | 0.00 | 0.00 | -0.01 | 0.00 | 0.01 | 0.00 |
| Yamadazyma$_{VIP≥0.8}$ | 1.08 | 2.10 | 1.45 | 0.72 | 1.10 | 0.82 | -0.02 | 0.06 | -0.02 | 0.00 | 0.02 | 0.00 |
| Aromatoleum$_{VIP≥0.8}$ | 0.82 | 0.67 | 0.87 | 0.85 | 0.69 | 0.98 | 0.01 | 0.01 | 0.00 | 0.00 | 0.01 | -0.01 |
| Azorhizobium$_{VIP≥0.8}$ | 0.43 | 1.21 | 1.03 | 0.88 | 0.63 | 1.19 | 0.00 | -0.02 | 0.00 | 0.00 | 0.00 | 0.01 |
| Capronia$_{VIP≥0.8}$ | 0.14 | 0.35 | 1.35 | 1.38 | 0.97 | 0.95 | 0.00 | -0.02 | 0.04 | 0.03 | -0.02 | 0.02 |
| Deinococcus$_{VIP≥0.8}$ | 0.43 | 1.42 | 0.72 | 1.17 | 0.87 | 0.88 | 0.00 | -0.03 | 0.00 | -0.02 | -0.01 | 0.02 |
| Enterocytozoon$_{VIP≥0.8}$ | 0.16 | 2.43 | 1.02 | 0.87 | 0.88 | 0.43 | 0.00 | 0.07 | -0.02 | 0.02 | 0.01 | -0.01 |
| Geobacter$_{VIP≥0.8}$ | 1.05 | 0.69 | 0.99 | 0.86 | 0.78 | 0.96 | -0.01 | 0.00 | 0.00 | -0.01 | 0.00 | 0.01 |

| | | | | | | | | | | | | |
|---|---|---|---|---|---|---|---|---|---|---|---|---|
| Gordonia$_{VIP≥0.8}$ | 0.90 | 0.66 | 0.92 | 0.87 | 0.75 | 0.98 | 0.01 | 0.01 | 0.00 | -0.01 | 0.01 | -0.01 |
| Guillardia$_{VIP≥0.8}$ | 0.21 | 1.66 | 0.98 | 0.95 | 0.50 | 1.52 | 0.00 | 0.04 | -0.01 | -0.01 | 0.00 | 0.03 |
| Halothiobacillus$_{VIP≥0.8}$ | 0.99 | 0.84 | 1.12 | 0.89 | 0.75 | 0.76 | -0.02 | 0.00 | 0.01 | 0.00 | 0.01 | 0.00 |
| Marivirga$_{VIP≥0.8}$ | 0.74 | 0.77 | 0.87 | 1.49 | 0.97 | 1.15 | 0.01 | 0.01 | -0.01 | -0.03 | -0.02 | 0.02 |
| Microbacterium$_{VIP≥0.8}$ | 0.44 | 1.40 | 0.44 | 1.32 | 1.42 | 1.30 | 0.00 | 0.06 | -0.01 | 0.02 | -0.03 | 0.02 |
| Pantoea$_{VIP≥0.8}$ | 0.83 | 0.51 | 0.82 | 0.93 | 0.79 | 1.08 | 0.00 | 0.02 | -0.01 | 0.00 | 0.01 | -0.01 |
| Phycomyces$_{VIP≥0.8}$ | 0.57 | 0.95 | 0.69 | 0.83 | 0.97 | 0.82 | -0.01 | 0.01 | 0.01 | 0.00 | 0.01 | -0.01 |
| Porphyrobacter$_{VIP≥0.8}$ | 0.96 | 1.07 | 1.05 | 0.85 | 0.78 | 0.77 | 0.01 | -0.01 | 0.00 | -0.01 | 0.01 | 0.00 |
| Rhodopseudomonas$_{VIP≥0.8}$ | 0.83 | 1.08 | 0.77 | 0.79 | 1.05 | 1.60 | 0.02 | -0.02 | 0.00 | -0.01 | -0.02 | 0.03 |
| Sphingopyxis$_{VIP≥0.8}$ | 0.75 | 0.73 | 0.88 | 0.85 | 0.87 | 1.21 | 0.01 | 0.00 | 0.00 | 0.00 | -0.01 | 0.01 |
| Thermaerobacter$_{VIP≥0.8}$ | 0.86 | 0.69 | 1.06 | 0.96 | 1.09 | 0.75 | -0.01 | 0.00 | 0.01 | -0.02 | -0.02 | 0.00 |
| Thioflavicoccus$_{VIP≥0.8}$ | 0.83 | 0.79 | 0.97 | 0.87 | 0.75 | 1.20 | 0.01 | 0.00 | 0.00 | -0.01 | 0.01 | -0.02 |
| *Geoalkalibacter* | 0.79 | 0.59 | 1.42 | 0.97 | 0.77 | 1.20 | 0.01 | 0.01 | 0.02 | 0.01 | 0.01 | 0.01 |
| *Millerozyma* | 1.86 | 0.76 | 0.45 | 1.12 | 0.86 | 0.72 | 0.04 | 0.01 | 0.00 | 0.02 | 0.01 | 0.01 |
| *Candidatus* | 0.74 | 0.55 | 0.48 | 1.26 | 0.88 | 0.86 | 0.01 | 0.01 | -0.02 | 0.02 | 0.02 | 0.01 |
| *Cytophaga* | 0.74 | 1.12 | 0.91 | 1.64 | 0.46 | 0.53 | 0.01 | 0.06 | 0.02 | 0.03 | 0.01 | -0.01 |
| *Dehalogenimonas* | 0.76 | 2.45 | 1.35 | 0.94 | 0.63 | 0.43 | -0.02 | 0.10 | 0.03 | 0.01 | 0.00 | 0.00 |
| *Dictyoglomus* | 0.80 | 0.35 | 0.70 | 0.87 | 1.61 | 0.47 | 0.01 | -0.02 | 0.02 | 0.02 | 0.03 | 0.01 |
| *Leptomonas* | 0.31 | 1.78 | 0.69 | 1.39 | 2.23 | 0.42 | 0.00 | -0.06 | 0.02 | 0.03 | 0.05 | 0.01 |
| *Phytophthora* | 0.61 | 0.35 | 0.82 | 0.78 | 1.58 | 0.83 | 0.01 | -0.01 | 0.03 | 0.01 | 0.03 | 0.01 |
| *Rasamsonia* | 1.00 | 0.69 | 0.62 | 1.17 | 1.47 | 0.63 | 0.02 | -0.03 | 0.01 | 0.02 | 0.03 | 0.01 |
| *Agromyces* | 0.58 | 0.63 | 0.97 | 0.79 | 1.47 | 1.52 | 0.00 | 0.00 | 0.01 | -0.01 | -0.03 | 0.03 |
| *Calditerrivibrio* | 0.77 | 1.60 | 0.52 | 0.68 | 1.27 | 0.88 | -0.01 | 0.05 | 0.01 | 0.01 | 0.03 | -0.02 |
| *Chlorobaculum* | 1.61 | 0.73 | 1.01 | 0.83 | 0.78 | 0.77 | -0.03 | 0.00 | 0.00 | 0.00 | 0.01 | 0.01 |
| *Chryseobacterium* | 0.90 | 0.24 | 0.48 | 1.46 | 1.97 | 0.68 | -0.02 | 0.01 | 0.00 | 0.02 | 0.04 | -0.01 |
| *Cloacibacillus* | 0.43 | 1.43 | 1.19 | 0.78 | 0.75 | 1.42 | 0.01 | -0.03 | 0.01 | 0.00 | -0.01 | 0.02 |
| *Dorea* | 0.30 | 2.01 | 0.56 | 1.58 | 1.40 | 0.50 | -0.01 | 0.09 | -0.02 | 0.03 | 0.03 | 0.00 |
| *Edwardsiella* | 0.77 | 0.74 | 0.96 | 0.86 | 0.67 | 1.05 | 0.00 | 0.00 | 0.00 | -0.01 | 0.00 | -0.01 |
| *Fomitiporia* | 0.79 | 0.48 | 0.75 | 0.83 | 0.87 | 1.08 | 0.01 | -0.03 | 0.02 | 0.01 | 0.01 | -0.01 |
| *Pelobacter* | 0.20 | 0.43 | 1.41 | 1.38 | 0.77 | 1.72 | 0.00 | 0.00 | 0.03 | 0.02 | -0.01 | 0.04 |
| *Pseudopropionibacterium* | 2.09 | 0.64 | 0.65 | 0.76 | 1.52 | 1.69 | 0.04 | -0.01 | 0.00 | -0.02 | 0.03 | 0.04 |
| *Salimicrobium* | 2.47 | 0.79 | 1.09 | 1.68 | 0.43 | 0.78 | 0.05 | 0.00 | 0.02 | 0.03 | 0.00 | 0.00 |
| *Blautia* | 0.41 | 1.77 | 0.58 | 1.40 | 0.89 | 0.60 | -0.01 | 0.09 | -0.02 | 0.02 | 0.02 | -0.01 |

| | | | | | | | | | | | | |
|---|---|---|---|---|---|---|---|---|---|---|---|---|
| *Dermacoccus* | 0.69 | 0.15 | 0.33 | 0.91 | 1.95 | 1.04 | 0.01 | 0.01 | -0.01 | -0.02 | -0.04 | 0.02 |
| *Frankia* | 0.99 | 0.54 | 0.77 | 0.75 | 1.84 | 0.86 | 0.02 | 0.03 | -0.02 | 0.00 | -0.04 | 0.01 |
| *Klebsiella* | 1.22 | 0.61 | 0.75 | 0.92 | 0.58 | 0.99 | -0.02 | 0.01 | -0.01 | 0.00 | 0.00 | 0.00 |
| *Methanosarcina* | 1.14 | 0.45 | 0.74 | 0.77 | 0.85 | 0.96 | -0.02 | -0.03 | 0.01 | 0.01 | 0.01 | 0.00 |
| *Methanothermus* | 1.26 | 0.46 | 0.74 | 0.93 | 0.45 | 0.90 | 0.02 | -0.02 | 0.01 | 0.00 | 0.00 | 0.01 |
| *Novosphingobium* | 0.45 | 1.15 | 1.13 | 0.42 | 0.79 | 1.05 | 0.01 | -0.02 | 0.02 | -0.01 | -0.01 | 0.02 |
| *Pandoraea* | 0.63 | 0.74 | 0.76 | 0.93 | 0.90 | 1.01 | 0.00 | 0.00 | -0.01 | -0.02 | -0.01 | 0.01 |
| *Pichia* | 0.42 | 0.83 | 0.66 | 0.98 | 0.91 | 0.55 | 0.01 | 0.00 | 0.02 | -0.01 | -0.01 | 0.00 |
| *Sinorhizobium* | 0.62 | 0.71 | 0.76 | 0.85 | 1.25 | 0.85 | 0.01 | 0.00 | -0.02 | 0.00 | -0.02 | 0.00 |
| *Sporothrix* | 0.35 | 0.46 | 0.58 | 1.05 | 1.83 | 1.04 | 0.00 | -0.03 | 0.01 | -0.01 | 0.04 | 0.02 |
| *Toxoplasma* | 0.78 | 0.80 | 1.10 | 1.12 | 1.24 | 0.75 | -0.02 | 0.00 | -0.02 | -0.01 | 0.02 | 0.00 |
| *Brachyspira* | 0.51 | 0.67 | 1.20 | 0.76 | 1.40 | 0.95 | -0.01 | 0.00 | -0.02 | 0.00 | 0.03 | -0.02 |
| *Chaetomium* | 0.61 | 0.69 | 0.93 | 0.94 | 0.79 | 0.86 | -0.01 | -0.01 | 0.00 | 0.00 | 0.00 | 0.01 |
| *Coccidioides* | 0.33 | 0.70 | 1.25 | 0.96 | 0.84 | 0.76 | 0.00 | -0.01 | -0.02 | -0.01 | 0.00 | 0.00 |
| *Deferribacter* | 0.55 | 0.47 | 1.13 | 0.79 | 0.90 | 0.87 | 0.00 | 0.00 | -0.01 | 0.00 | 0.01 | 0.00 |
| *Enterobacter* | 0.99 | 0.55 | 0.79 | 0.81 | 0.58 | 1.07 | -0.01 | 0.01 | -0.02 | -0.01 | 0.00 | -0.01 |
| *Methanocella* | 0.73 | 2.84 | 0.67 | 1.29 | 2.69 | 0.58 | 0.01 | -0.10 | -0.02 | -0.03 | -0.06 | 0.01 |
| *Nostoc* | 0.84 | 0.47 | 1.47 | 1.34 | 0.75 | 0.73 | -0.02 | -0.02 | -0.02 | -0.02 | 0.00 | 0.01 |
| *Sandaracinus* | 0.83 | 0.91 | 0.71 | 0.94 | 0.66 | 0.77 | -0.01 | -0.01 | -0.02 | -0.02 | 0.01 | 0.01 |
| *Starkeya* | 0.60 | 1.02 | 0.80 | 1.02 | 0.83 | 0.76 | 0.00 | -0.01 | -0.01 | -0.02 | 0.02 | 0.02 |
| *Blastomyces* | 0.45 | 0.62 | 0.92 | 1.13 | 0.84 | 0.76 | 0.00 | -0.01 | 0.00 | -0.01 | -0.01 | 0.00 |
| *Methanoregula* | 0.79 | 0.97 | 1.25 | 0.78 | 0.76 | 0.92 | 0.00 | -0.01 | 0.01 | 0.00 | -0.01 | -0.01 |
| *Paracoccidioides* | 0.68 | 0.60 | 1.30 | 1.00 | 0.81 | 0.78 | -0.01 | -0.01 | -0.02 | -0.01 | 0.00 | 0.00 |
| *Selenomonas* | 1.11 | 0.40 | 0.76 | 0.66 | 0.82 | 1.30 | -0.02 | 0.00 | -0.02 | 0.01 | -0.01 | -0.03 |
| *Succiniclasticum* | 0.75 | 1.20 | 1.28 | 1.74 | 0.35 | 0.69 | 0.02 | -0.06 | -0.03 | -0.03 | -0.01 | -0.01 |
| *Variovorax* | 0.95 | 0.80 | 0.74 | 0.88 | 0.79 | 0.99 | -0.01 | 0.00 | -0.01 | -0.01 | -0.01 | 0.00 |
| *Dictyostelium* | 0.75 | 0.72 | 1.44 | 1.12 | 0.80 | 0.80 | -0.01 | -0.01 | -0.02 | -0.01 | 0.00 | -0.01 |
| *Paramecium* | 0.91 | 0.56 | 1.55 | 1.36 | 0.77 | 0.70 | -0.02 | -0.01 | -0.03 | -0.02 | -0.01 | 0.00 |
| *Plasmodium* | 0.47 | 0.58 | 1.13 | 1.03 | 0.80 | 0.79 | -0.01 | -0.01 | -0.01 | -0.01 | 0.00 | 0.00 |
| *Tetrahymena* | 0.67 | 0.77 | 1.43 | 1.13 | 0.79 | 0.81 | -0.01 | 0.00 | -0.02 | -0.01 | 0.00 | -0.01 |
| *Acanthamoeba* | 0.56 | 0.80 | 0.72 | 0.71 | 0.76 | 1.08 | 0.00 | 0.01 | 0.02 | 0.01 | 0.00 | 0.02 |
| *Eimeria* | 0.61 | 0.65 | 0.64 | 0.75 | 0.82 | 1.00 | 0.01 | -0.04 | 0.01 | 0.01 | 0.00 | 0.02 |
| *Malassezia* | 1.39 | 0.48 | 1.12 | 0.74 | 0.20 | 0.78 | 0.02 | 0.02 | -0.02 | 0.01 | 0.00 | 0.01 |
| *Luteipulveratus* | 1.18 | 0.75 | 1.16 | 0.23 | 0.74 | 0.74 | 0.02 | -0.02 | -0.02 | 0.00 | 0.02 | 0.02 |
| *Perkinsus* | 0.54 | 0.48 | 0.95 | 0.77 | 0.87 | 0.73 | 0.01 | -0.01 | 0.00 | 0.00 | 0.01 | 0.01 |

| | | | | | | | | | | | | |
|---|---|---|---|---|---|---|---|---|---|---|---|---|
| *Rhinocladiella* | 0.42 | 0.62 | 0.80 | 0.75 | 0.74 | 0.85 | 0.00 | -0.01 | 0.00 | 0.00 | 0.00 | 0.01 |
| *Methanococcus* | 0.67 | 0.70 | 0.97 | 0.78 | 0.71 | 0.82 | -0.01 | 0.00 | 0.00 | 0.01 | 0.00 | 0.01 |
| *Veillonella* | 0.57 | 1.10 | 0.73 | 1.22 | 0.79 | 0.77 | -0.01 | -0.01 | 0.00 | 0.01 | -0.01 | 0.00 |
| *Calothrix* | 0.74 | 0.60 | 1.29 | 1.36 | 0.70 | 0.73 | -0.01 | -0.01 | -0.02 | -0.02 | 0.00 | 0.01 |
| *Castellaniella* | 0.72 | 0.73 | 0.68 | 0.89 | 0.73 | 1.01 | 0.00 | 0.00 | -0.01 | 0.00 | 0.01 | 0.00 |
| *Chromohalobacter* | 0.44 | 0.73 | 0.76 | 0.33 | 1.55 | 1.77 | 0.00 | 0.00 | 0.00 | -0.01 | 0.03 | 0.03 |
| *Oceanimonas* | 0.99 | 0.58 | 0.97 | 0.71 | 0.64 | 0.74 | -0.01 | 0.04 | 0.00 | 0.00 | -0.01 | -0.01 |
| *Thiobacillus* | 0.70 | 0.51 | 0.75 | 0.81 | 0.58 | 0.90 | 0.00 | 0.01 | -0.01 | -0.01 | 0.00 | 0.00 |
| *Thiobacimonas* | 0.53 | 0.59 | 1.12 | 0.73 | 0.88 | 0.74 | 0.00 | 0.01 | 0.01 | 0.00 | -0.01 | -0.01 |
| *Cellvibrio* | 1.30 | 0.73 | 0.68 | 0.67 | 0.78 | 0.89 | -0.02 | -0.01 | -0.01 | -0.01 | -0.01 | 0.00 |
| *Massilia* | 0.76 | 1.12 | 0.75 | 0.81 | 0.68 | 0.62 | -0.01 | -0.02 | -0.01 | -0.01 | 0.01 | 0.00 |
| *Ichthyophthirius* | 0.78 | 0.60 | 1.33 | 1.20 | 0.79 | 0.80 | -0.01 | -0.01 | -0.02 | -0.01 | 0.00 | -0.01 |
| *Magnaporthe* | 0.74 | 0.70 | 0.97 | 0.77 | 0.72 | 0.61 | 0.01 | 0.00 | -0.01 | 0.01 | 0.00 | 0.00 |

Partial Least Squares analysis was performed with 2 latent components. Subscript $_{VIP≥0.8}$ denotes microbial genera that had VIP ≥ 0.8 in 4 or more timepoints. Subscripts $_{(+)}$ and $_{(-)}$ represent microbial genera that had positive or negative regression coefficient in 4 or more timepoints, respectively.

**Trait: Average Daily Weight Gain (ADG); Predictors: 264 microbial genera (MT)**

| Microbial genera | VIP | | | | | | Regression coefficient | | | | | |
|---|---|---|---|---|---|---|---|---|---|---|---|---|
| | T1 | T2 | T3 | T4 | T5 | T6 | T1 | T2 | T3 | T4 | T5 | T6 |
| Oribacterium$_{VIP≥0.8\ (+)}$ | 0.63 | 1.57 | 1.29 | 1.09 | 0.51 | 0.97 | 0.01 | -0.07 | 0.04 | -0.01 | 0.01 | 0.02 |
| Mitsuokella$_{VIP≥0.8\ (+)}$ | 0.56 | 1.34 | 1.03 | 1.01 | 0.87 | 0.94 | 0.01 | 0.02 | 0.01 | -0.01 | 0.00 | 0.00 |
| Acidothermus$_{VIP≥0.8\ (+)}$ | 0.83 | 1.41 | 1.50 | 1.04 | 0.92 | 0.59 | 0.02 | 0.03 | 0.04 | 0.01 | 0.03 | 0.01 |
| Cylindrospermum$_{VIP≥0.8\ (+)}$ | 1.96 | 1.22 | 0.94 | 0.89 | 0.58 | 2.94 | 0.03 | 0.04 | -0.02 | 0.01 | 0.01 | 0.05 |
| Methyloversatilis$_{VIP≥0.8\ (+)}$ | 0.78 | 1.16 | 1.06 | 1.42 | 0.87 | 1.67 | 0.01 | 0.02 | 0.03 | 0.02 | -0.02 | 0.03 |
| Methanocella$_{VIP≥0.8\ (+)}$ | 1.61 | 2.20 | 0.97 | 1.38 | 2.13 | 0.36 | -0.03 | 0.07 | 0.03 | 0.02 | 0.06 | 0.00 |
| Paracoccus$_{VIP≥0.8\ (+)}$ | 0.75 | 1.57 | 1.02 | 0.85 | 1.79 | 0.77 | 0.01 | 0.04 | 0.02 | -0.01 | 0.05 | 0.00 |
| Pseudomonas$_{VIP≥0.8\ (+)}$ | 1.33 | 0.94 | 0.94 | 1.02 | 0.79 | 0.92 | 0.03 | 0.00 | 0.00 | 0.01 | 0.01 | 0.01 |
| Sulfurovum$_{VIP≥0.8\ (+)}$ | 1.62 | 1.06 | 1.24 | 1.11 | 0.73 | 0.75 | 0.03 | 0.02 | 0.03 | -0.01 | 0.02 | 0.01 |
| Acidovorax$_{VIP≥0.8\ (+)}$ | 1.25 | 0.89 | 0.88 | 1.28 | 0.88 | 1.15 | 0.02 | 0.00 | 0.00 | 0.01 | 0.00 | 0.02 |
| Hyphomonas$_{VIP≥0.8\ (+)}$ | 0.78 | 0.91 | 1.57 | 1.00 | 0.85 | 0.80 | 0.01 | 0.00 | 0.05 | 0.01 | 0.00 | 0.01 |

| | | | | | | | | | | | | |
|---|---|---|---|---|---|---|---|---|---|---|---|---|
| Raoultella$_{VIP\geq0.8\ (+)}$ | 0.82 | 0.80 | 1.18 | 1.03 | 0.80 | 0.96 | 0.01 | -0.01 | 0.02 | 0.01 | 0.00 | 0.01 |
| Sorangium$_{VIP\geq0.8\ (+)}$ | 0.93 | 0.83 | 1.23 | 1.24 | 0.85 | 0.70 | 0.02 | -0.01 | 0.03 | 0.01 | 0.00 | 0.01 |
| Zhongshania$_{VIP\geq0.8\ (+)}$ | 0.83 | 0.88 | 1.05 | 0.83 | 0.82 | 1.35 | 0.01 | 0.00 | 0.01 | 0.00 | 0.00 | 0.02 |
| Leclercia$_{VIP\geq0.8\ (+)}$ | 1.65 | 0.75 | 0.90 | 0.92 | 0.84 | 0.90 | 0.03 | -0.01 | 0.00 | 0.00 | 0.00 | 0.01 |
| Dickeya$_{VIP\geq0.8\ (+)}$ | 0.87 | 1.17 | 1.19 | 0.92 | 1.12 | 0.96 | 0.01 | 0.01 | 0.02 | 0.01 | -0.01 | 0.01 |
| Methyloceanibacter$_{VIP\geq0.8\ (+)}$ | 0.77 | 1.09 | 1.02 | 1.04 | 0.79 | 0.85 | 0.00 | 0.01 | 0.01 | 0.01 | 0.01 | 0.01 |
| Ralstonia$_{VIP\geq0.8\ (+)}$ | 0.83 | 0.92 | 1.20 | 1.06 | 0.88 | 0.87 | 0.01 | 0.00 | 0.02 | 0.01 | 0.02 | 0.01 |
| Pannonibacter$_{VIP\geq0.8\ (+)}$ | 0.93 | 1.07 | 1.28 | 0.92 | 0.85 | 0.90 | 0.01 | 0.01 | 0.02 | 0.01 | 0.00 | 0.00 |
| Blastomonas$_{VIP\geq0.8\ (+)}$ | 1.01 | 0.70 | 1.25 | 0.98 | 0.78 | 0.84 | 0.02 | -0.02 | 0.03 | 0.01 | 0.00 | 0.01 |
| Variovorax$_{VIP\geq0.8\ (+)}$ | 0.94 | 0.91 | 0.95 | 1.05 | 0.83 | 0.88 | 0.02 | 0.00 | 0.01 | 0.01 | 0.01 | 0.00 |
| Enterobacter$_{VIP\geq0.8\ (+)}$ | 0.88 | 0.80 | 1.13 | 0.97 | 0.83 | 0.99 | 0.02 | -0.01 | 0.01 | 0.01 | 0.00 | 0.02 |
| Thermaerobacter$_{VIP\geq0.8\ (+)}$ | 0.82 | 0.98 | 0.69 | 1.11 | 1.04 | 0.78 | 0.01 | 0.01 | -0.02 | 0.01 | 0.03 | 0.00 |
| Aromatoleum$_{VIP\geq0.8\ (+)}$ | 0.82 | 1.00 | 1.23 | 0.90 | 0.86 | 1.03 | 0.00 | 0.01 | 0.02 | 0.01 | 0.00 | 0.02 |
| Sanguibacter$_{VIP\geq0.8\ (+)}$ | 1.01 | 0.92 | 0.99 | 1.01 | 0.99 | 0.87 | 0.02 | 0.00 | 0.00 | 0.01 | 0.00 | 0.01 |
| Halotalea$_{VIP\geq0.8\ (+)}$ | 0.83 | 0.94 | 1.48 | 0.89 | 0.99 | 0.89 | 0.01 | 0.00 | 0.03 | 0.00 | 0.00 | 0.01 |
| Thauera$_{VIP\geq0.8\ (+)}$ | 0.91 | 1.12 | 0.92 | 0.93 | 0.91 | 0.86 | 0.01 | 0.01 | 0.01 | 0.01 | 0.00 | 0.00 |
| Defluviimonas$_{VIP\geq0.8\ (+)}$ | 0.86 | 1.05 | 0.99 | 1.12 | 0.82 | 0.85 | 0.00 | 0.00 | 0.01 | 0.01 | 0.01 | 0.01 |
| Sinorhizobium$_{VIP\geq0.8\ (+)}$ | 1.16 | 1.16 | 1.56 | 0.91 | 1.01 | 0.86 | -0.01 | 0.01 | 0.03 | 0.00 | 0.02 | 0.00 |
| Nitrobacter$_{VIP\geq0.8\ (+)}$ | 0.90 | 1.22 | 1.22 | 1.00 | 0.94 | 0.87 | 0.00 | 0.02 | 0.02 | 0.01 | 0.00 | 0.00 |
| Cladophialophora$_{VIP\geq0.8\ (+)}$ | 0.87 | 1.33 | 0.61 | 1.45 | 1.24 | 1.45 | 0.02 | 0.04 | 0.03 | 0.02 | -0.04 | 0.02 |
| Halolamina$_{VIP\geq0.8\ (+)}$ | 0.83 | 1.03 | 0.78 | 1.02 | 0.93 | 0.86 | 0.01 | 0.00 | -0.01 | 0.01 | 0.00 | 0.01 |
| Microbulbifer$_{VIP\geq0.8\ (+)}$ | 0.78 | 1.12 | 0.97 | 0.99 | 0.91 | 0.90 | 0.01 | 0.01 | 0.01 | 0.01 | 0.00 | 0.01 |
| Pseudoxanthomonas$_{VIP\geq0.8\ (+}$ | 0.71 | 0.83 | 0.97 | 1.04 | 0.65 | 0.81 | 0.01 | -0.01 | 0.02 | 0.01 | 0.01 | 0.00 |
| Agrobacterium$_{VIP\geq0.8\ (+)}$ | 0.95 | 1.12 | 1.40 | 0.91 | 0.87 | 0.92 | 0.00 | 0.01 | 0.03 | 0.00 | 0.01 | 0.01 |
| Dermabacter$_{VIP\geq0.8\ (+)}$ | 0.80 | 1.15 | 1.00 | 0.98 | 1.09 | 0.87 | 0.01 | 0.01 | 0.01 | 0.01 | -0.01 | 0.01 |
| Sideroxydans$_{VIP\geq0.8\ (+)}$ | 0.79 | 0.88 | 1.04 | 0.96 | 0.85 | 0.95 | 0.00 | 0.00 | 0.01 | 0.00 | 0.00 | 0.01 |
| Rhodobacter$_{VIP\geq0.8\ (+)}$ | 0.81 | 1.10 | 0.95 | 1.20 | 0.91 | 0.92 | 0.01 | 0.01 | 0.00 | 0.01 | 0.00 | 0.00 |
| Brevundimonas$_{VIP\geq0.8\ (+)}$ | 0.68 | 1.01 | 0.84 | 0.92 | 0.73 | 1.04 | 0.01 | 0.01 | 0.00 | 0.01 | 0.01 | 0.00 |
| Modestobacter$_{VIP\geq0.8\ (+)}$ | 0.77 | 0.98 | 0.98 | 0.96 | 0.92 | 0.89 | 0.01 | 0.00 | 0.01 | 0.01 | 0.00 | 0.01 |
| Burkholderia$_{VIP\geq0.8\ (+)}$ | 0.90 | 0.95 | 1.15 | 0.87 | 0.90 | 0.97 | 0.01 | 0.00 | 0.01 | 0.00 | 0.01 | 0.00 |

| | | | | | | | | | | | | |
|---|---|---|---|---|---|---|---|---|---|---|---|---|
| Tsukamurella$_{VIP≥0.8\ (+)}$ | 0.83 | 1.05 | 1.17 | 1.01 | 1.10 | 0.82 | 0.00 | 0.01 | 0.02 | 0.01 | -0.01 | 0.01 |
| Halosimplex$_{VIP≥0.8\ (+)}$ | 0.83 | 1.20 | 0.82 | 0.99 | 0.95 | 0.98 | 0.00 | 0.01 | 0.00 | 0.01 | -0.01 | 0.02 |
| Arthrobacter$_{VIP≥0.8\ (+)}$ | 0.86 | 1.14 | 1.07 | 0.88 | 0.89 | 1.00 | 0.01 | 0.01 | 0.01 | 0.00 | 0.01 | 0.00 |
| Sinomonas$_{VIP≥0.8\ (+)}$ | 0.68 | 0.81 | 0.80 | 0.98 | 0.78 | 0.84 | 0.00 | -0.01 | 0.01 | 0.01 | 0.01 | 0.01 |
| Azospira$_{VIP≥0.8\ (+)}$ | 0.69 | 0.90 | 0.80 | 0.99 | 0.89 | 0.85 | 0.01 | 0.00 | -0.01 | 0.01 | 0.00 | 0.01 |
| Chelatococcus$_{VIP≥0.8\ (+)}$ | 0.93 | 1.02 | 1.09 | 0.94 | 0.55 | 0.79 | 0.00 | 0.01 | 0.02 | 0.01 | 0.01 | 0.01 |
| Nitrosospira$_{VIP≥0.8\ (+)}$ | 0.83 | 0.84 | 1.12 | 0.92 | 0.91 | 0.88 | 0.01 | -0.01 | 0.01 | 0.00 | 0.00 | 0.01 |
| Immundisolibacter$_{VIP≥0.8\ (+)}$ | 0.84 | 1.03 | 0.97 | 0.93 | 0.93 | 0.89 | 0.01 | 0.01 | 0.01 | 0.00 | -0.01 | 0.01 |
| Azoarcus$_{VIP≥0.8\ (+)}$ | 0.85 | 0.88 | 0.95 | 1.04 | 0.84 | 0.87 | 0.00 | 0.00 | 0.01 | 0.01 | 0.00 | 0.00 |
| Chelativorans$_{VIP≥0.8\ (+)}$ | 0.72 | 0.84 | 0.87 | 1.09 | 1.14 | 0.79 | 0.01 | 0.00 | 0.00 | 0.01 | -0.01 | 0.01 |
| Mesorhizobium$_{VIP≥0.8\ (+)}$ | 0.86 | 0.88 | 1.03 | 0.96 | 0.88 | 0.94 | 0.01 | -0.01 | 0.01 | 0.00 | 0.01 | 0.00 |
| Tolumonas$_{VIP≥0.8\ (+)}$ | 0.82 | 0.80 | 0.80 | 0.87 | 0.75 | 0.89 | 0.00 | 0.00 | 0.00 | 0.01 | 0.00 | 0.01 |
| Rhizobium$_{VIP≥0.8\ (+)}$ | 0.85 | 0.87 | 1.24 | 0.86 | 0.90 | 0.99 | 0.00 | -0.01 | 0.02 | 0.00 | 0.01 | 0.00 |
| Herbaspirillum$_{VIP≥0.8\ (+)}$ | 0.71 | 1.26 | 0.74 | 0.81 | 0.84 | 0.88 | 0.00 | 0.02 | -0.02 | 0.00 | 0.01 | 0.01 |
| Thioalkalivibrio$_{VIP≥0.8\ (+)}$ | 0.92 | 0.97 | 1.14 | 0.91 | 0.95 | 0.87 | 0.00 | 0.00 | 0.01 | 0.00 | 0.00 | 0.01 |
| Methanoregula$_{VIP≥0.8\ (+)}$ | 0.80 | 1.17 | 0.83 | 0.80 | 0.84 | 0.84 | 0.00 | 0.01 | -0.01 | 0.00 | 0.00 | 0.01 |
| Sphingomonas$_{VIP≥0.8\ (+)}$ | 0.78 | 0.77 | 1.27 | 0.87 | 0.92 | 1.13 | 0.01 | -0.02 | 0.02 | 0.01 | 0.02 | -0.01 |
| Castellaniella$_{VIP≥0.8\ (+)}$ | 0.90 | 0.94 | 0.95 | 0.96 | 0.83 | 0.91 | 0.00 | 0.00 | 0.01 | 0.01 | 0.00 | 0.00 |
| Klebsiella$_{VIP≥0.8\ (+)}$ | 0.77 | 0.89 | 1.39 | 0.93 | 0.80 | 0.86 | 0.01 | 0.00 | 0.03 | 0.00 | 0.00 | 0.00 |
| Acaryochloris$_{VIP≥0.8\ (+)}$ | 0.87 | 1.06 | 0.84 | 0.94 | 0.86 | 0.84 | 0.00 | 0.01 | -0.01 | 0.00 | 0.00 | 0.01 |
| Halorhodospira$_{VIP≥0.8\ (+)}$ | 1.03 | 1.02 | 0.91 | 0.95 | 0.89 | 0.89 | 0.00 | 0.00 | 0.00 | 0.00 | 0.00 | 0.01 |
| Edwardsiella$_{VIP≥0.8\ (+)}$ | 1.28 | 0.90 | 0.99 | 0.97 | 0.87 | 0.91 | -0.01 | 0.00 | 0.00 | 0.01 | 0.00 | 0.01 |
| Halomonas$_{VIP≥0.8\ (+)}$ | 1.11 | 0.98 | 0.91 | 0.94 | 0.96 | 0.90 | -0.01 | 0.00 | 0.00 | 0.01 | -0.01 | 0.01 |
| Blastochloris$_{VIP≥0.8\ (+)}$ | 1.00 | 0.82 | 1.11 | 1.01 | 0.83 | 0.75 | 0.00 | 0.00 | 0.01 | 0.01 | 0.00 | 0.00 |
| Azospirillum$_{VIP≥0.8\ (+)}$ | 0.80 | 1.03 | 0.93 | 1.08 | 0.96 | 1.25 | 0.01 | 0.01 | 0.00 | 0.01 | 0.00 | -0.01 |
| Bordetella$_{VIP≥0.8\ (+)}$ | 0.90 | 0.94 | 1.07 | 0.90 | 0.88 | 0.83 | 0.00 | 0.00 | 0.01 | 0.01 | 0.01 | 0.00 |
| Carboxydothermus$_{VIP≥0.8\ (+)}$ | 2.21 | 1.31 | 1.24 | 1.20 | 0.09 | 0.24 | -0.03 | 0.02 | 0.02 | -0.01 | 0.00 | 0.00 |
| Candidatus Methanoperede[illegible] | 0.78 | 1.07 | 0.99 | 1.06 | 0.87 | 0.82 | 0.00 | 0.01 | 0.01 | 0.00 | 0.00 | 0.01 |
| Pantoea$_{VIP≥0.8\ (+)}$ | 0.81 | 0.80 | 1.03 | 0.87 | 0.97 | 0.91 | 0.00 | -0.01 | 0.01 | 0.00 | -0.01 | 0.01 |
| Endocarpon$_{VIP≥0.8\ (+)}$ | 1.11 | 1.01 | 0.93 | 0.56 | 1.02 | 1.67 | 0.01 | 0.04 | -0.01 | 0.00 | 0.00 | 0.02 |

| | | | | | | | | | | | | |
|---|---|---|---|---|---|---|---|---|---|---|---|---|
| Marivirga$_{VIP\geq0.8\ (+)}$ | 0.87 | 0.66 | 0.39 | 0.86 | 0.89 | 1.72 | -0.02 | 0.00 | 0.02 | 0.01 | 0.01 | -0.03 |
| Eutypa$_{VIP\geq0.8\ (+)}$ | 1.76 | 0.83 | 0.82 | 1.01 | 1.14 | 0.94 | 0.02 | 0.01 | 0.02 | 0.01 | 0.01 | 0.00 |
| Anabaena$_{VIP\geq0.8\ (+)}$ | 1.49 | 0.79 | 0.99 | 0.96 | 1.58 | 1.02 | 0.02 | 0.01 | 0.00 | 0.01 | 0.03 | 0.00 |
| Rubrobacter$_{VIP\geq0.8\ (+)}$ | 1.00 | 0.93 | 1.00 | 1.08 | 1.26 | 0.98 | 0.02 | 0.00 | 0.01 | 0.01 | -0.02 | -0.01 |
| Cyanothece$_{VIP\geq0.8\ (+)}$ | 1.19 | 0.97 | 0.66 | 0.87 | 1.12 | 1.28 | 0.01 | -0.01 | 0.03 | 0.01 | 0.02 | 0.01 |
| Sphingopyxis$_{VIP\geq0.8\ (+)}$ | 1.31 | 0.92 | 1.11 | 0.88 | 0.90 | 0.99 | -0.01 | 0.00 | 0.01 | 0.00 | 0.01 | 0.00 |
| Coraliomargarita$_{VIP\geq0.8\ (+)}$ | 1.18 | 0.86 | 1.06 | 0.79 | 0.83 | 0.84 | -0.01 | 0.00 | 0.02 | 0.00 | 0.00 | 0.00 |
| Histoplasma$_{VIP\geq0.8\ (+)}$ | 0.73 | 0.86 | 1.00 | 0.87 | 1.08 | 0.81 | -0.01 | 0.01 | 0.00 | 0.00 | 0.01 | 0.00 |
| Starkeya$_{VIP\geq0.8\ (+)}$ | 0.82 | 1.04 | 1.14 | 1.28 | 0.48 | 0.78 | 0.00 | 0.01 | 0.02 | 0.02 | 0.00 | -0.01 |
| Shinella$_{VIP\geq0.8\ (+)}$ | 0.88 | 1.89 | 0.99 | 1.16 | 0.92 | 2.08 | 0.02 | 0.05 | 0.03 | -0.01 | 0.03 | -0.03 |
| Chamaesiphon$_{VIP\geq0.8\ (+)}$ | 0.95 | 0.97 | 0.82 | 1.65 | 0.85 | 0.68 | 0.01 | -0.01 | 0.00 | 0.02 | 0.01 | 0.00 |
| Zobellia$_{VIP\geq0.8\ (+)}$ | 0.29 | 0.98 | 1.14 | 0.97 | 2.52 | 0.39 | 0.00 | 0.02 | 0.05 | -0.01 | -0.06 | 0.01 |
| Trichophyton$_{VIP\geq0.8\ (+)}$ | 0.72 | 1.00 | 0.92 | 0.80 | 1.08 | 0.89 | 0.00 | 0.00 | 0.01 | 0.00 | 0.00 | 0.00 |
| Trypanosoma$_{VIP\geq0.8\ (+)}$ | 0.71 | 0.91 | 0.90 | 0.83 | 1.01 | 0.81 | -0.01 | 0.00 | 0.01 | 0.00 | 0.00 | 0.00 |
| Rhodopseudomonas$_{VIP\geq0.8\ (+)}$ | 1.23 | 0.99 | 0.69 | 0.91 | 0.86 | 1.07 | -0.01 | 0.01 | 0.00 | 0.01 | 0.02 | -0.01 |
| Talaromyces$_{VIP\geq0.8\ (+)}$ | 0.78 | 0.75 | 0.84 | 0.95 | 1.26 | 1.15 | 0.00 | 0.02 | 0.03 | 0.01 | 0.01 | 0.01 |
| Methanotorris$_{VIP\geq0.8\ (+)}$ | 0.68 | 1.53 | 1.16 | 1.59 | 0.85 | 1.08 | -0.01 | -0.03 | 0.06 | 0.02 | 0.00 | 0.01 |
| Magnaporthe$_{VIP\geq0.8\ (+)}$ | 0.83 | 0.77 | 0.81 | 0.79 | 1.01 | 0.92 | -0.02 | 0.01 | 0.01 | 0.00 | 0.01 | 0.01 |
| Marinithermus$_{VIP\geq0.8\ (+)}$ | 0.34 | 1.64 | 1.22 | 1.10 | 1.72 | 0.55 | 0.00 | 0.05 | 0.04 | -0.01 | -0.04 | 0.01 |
| Nosema$_{VIP\geq0.8\ (+)}$ | 0.80 | 0.79 | 0.86 | 1.14 | 1.09 | 0.92 | 0.00 | 0.02 | 0.04 | 0.01 | 0.01 | 0.00 |
| Methanocaldococcus$_{VIP\geq0.8\ (+}$ | 0.68 | 0.85 | 0.84 | 1.13 | 0.95 | 0.92 | -0.01 | 0.00 | 0.02 | 0.01 | 0.00 | 0.00 |
| Phialocephala$_{VIP\geq0.8\ (+)}$ | 1.24 | 0.75 | 0.98 | 0.76 | 1.03 | 1.05 | 0.01 | 0.01 | 0.00 | 0.00 | 0.01 | 0.01 |
| Dactylellina$_{VIP\geq0.8\ (+)}$ | 1.06 | 0.87 | 0.84 | 0.83 | 1.14 | 1.23 | 0.00 | 0.01 | 0.02 | 0.00 | 0.01 | 0.01 |
| Nodularia$_{VIP\geq0.8\ (+)}$ | 1.16 | 0.82 | 0.92 | 1.35 | 1.15 | 0.73 | 0.01 | 0.01 | 0.01 | 0.01 | 0.01 | -0.01 |
| Spizellomyces$_{VIP\geq0.8\ (+)}$ | 0.99 | 0.86 | 1.11 | 0.88 | 1.23 | 1.26 | 0.00 | 0.01 | -0.01 | 0.01 | 0.02 | 0.01 |
| Candida$_{VIP\geq0.8\ (+)}$ | 0.90 | 0.98 | 0.90 | 0.82 | 1.10 | 0.90 | 0.00 | 0.00 | 0.01 | 0.00 | 0.01 | 0.00 |
| Setosphaeria$_{VIP\geq0.8\ (+)}$ | 0.82 | 1.00 | 0.84 | 0.65 | 0.87 | 0.91 | 0.00 | 0.00 | 0.02 | 0.00 | 0.01 | 0.00 |
| Zygosaccharomyces$_{VIP\geq0.8\ (+)}$ | 0.85 | 0.92 | 0.93 | 0.93 | 1.24 | 0.79 | 0.00 | 0.00 | 0.01 | 0.00 | 0.01 | 0.00 |
| Naumovozyma$_{VIP\geq0.8\ (+)}$ | 1.08 | 0.72 | 0.86 | 0.74 | 0.89 | 0.86 | 0.01 | 0.01 | 0.02 | 0.00 | -0.01 | 0.00 |
| Marssonina$_{VIP\geq0.8\ (+)}$ | 0.81 | 0.93 | 0.92 | 0.86 | 0.96 | 0.92 | 0.00 | 0.00 | 0.00 | 0.01 | 0.00 | 0.00 |

| | | | | | | | | | | | | |
|---|---|---|---|---|---|---|---|---|---|---|---|---|
| Naegleria$_{VIP\geq0.8\ (+)}$ | 1.19 | 0.85 | 0.85 | 0.93 | 1.12 | 0.87 | 0.01 | 0.01 | 0.02 | 0.01 | 0.01 | 0.00 |
| Trichodesmium$_{VIP\geq0.8\ (+)}$ | 0.94 | 1.21 | 1.11 | 0.71 | 1.13 | 0.96 | 0.00 | -0.02 | -0.01 | 0.00 | 0.02 | 0.00 |
| Trichormus$_{VIP\geq0.8\ (+)}$ | 1.18 | 0.82 | 0.98 | 0.91 | 1.21 | 0.94 | 0.01 | 0.00 | 0.00 | 0.01 | 0.02 | 0.00 |
| Haemophilus$_{VIP\geq0.8\ (+)}$ | 1.16 | 1.03 | 0.30 | 0.85 | 0.71 | 0.88 | 0.01 | 0.04 | 0.00 | -0.01 | -0.02 | 0.01 |
| Arthrobotrys$_{VIP\geq0.8\ (+)}$ | 1.09 | 1.22 | 0.88 | 0.88 | 1.07 | 0.83 | 0.01 | -0.01 | 0.02 | 0.00 | 0.01 | 0.00 |
| Coccidioides$_{VIP\geq0.8\ (+)}$ | 0.80 | 0.92 | 0.91 | 0.92 | 1.04 | 0.88 | 0.00 | 0.00 | 0.01 | 0.01 | 0.00 | 0.00 |
| Isaria$_{VIP\geq0.8\ (+)}$ | 0.67 | 0.96 | 0.80 | 0.84 | 0.91 | 0.78 | 0.00 | 0.00 | 0.01 | 0.00 | 0.01 | 0.00 |
| Entamoeba$_{VIP\geq0.8\ (+)}$ | 1.53 | 0.90 | 0.98 | 0.78 | 0.98 | 0.79 | 0.02 | 0.01 | 0.00 | 0.00 | 0.00 | 0.00 |
| Nostoc$_{VIP\geq0.8\ (+)}$ | 1.38 | 0.89 | 0.90 | 1.13 | 0.98 | 0.75 | 0.01 | 0.00 | 0.01 | 0.01 | 0.01 | -0.01 |
| Nakaseomyces$_{VIP\geq0.8\ (+)}$ | 0.83 | 0.87 | 0.93 | 1.41 | 1.18 | 0.91 | -0.01 | 0.00 | 0.01 | 0.02 | 0.01 | 0.00 |
| Paramecium$_{VIP\geq0.8\ (+)}$ | 1.37 | 1.01 | 0.85 | 0.98 | 1.08 | 0.82 | 0.01 | 0.00 | 0.01 | 0.01 | 0.01 | 0.00 |
| Plasmodium$_{VIP\geq0.8\ (+)}$ | 0.94 | 0.90 | 0.97 | 0.87 | 1.17 | 0.88 | 0.00 | 0.01 | 0.01 | 0.00 | 0.01 | 0.00 |
| Microsporum$_{VIP\geq0.8\ (+)}$ | 0.97 | 0.76 | 0.95 | 0.89 | 1.20 | 1.04 | 0.00 | 0.01 | 0.01 | 0.00 | 0.01 | 0.00 |
| Cryptosporidium$_{VIP\geq0.8\ (+)}$ | 1.13 | 0.96 | 0.91 | 0.87 | 1.07 | 1.09 | 0.01 | 0.00 | 0.01 | 0.00 | 0.01 | 0.01 |
| Wickerhamomyces$_{VIP\geq0.8\ (+)}$ | 1.05 | 0.83 | 0.90 | 0.79 | 1.01 | 0.83 | 0.00 | 0.01 | 0.01 | 0.00 | 0.00 | 0.00 |
| Calothrix$_{VIP\geq0.8\ (+)}$ | 1.17 | 0.88 | 0.96 | 1.11 | 1.09 | 0.76 | 0.01 | 0.00 | 0.00 | 0.01 | 0.01 | -0.01 |
| Pneumocystis$_{VIP\geq0.8\ (+)}$ | 1.22 | 0.96 | 0.83 | 0.67 | 1.32 | 0.86 | 0.01 | 0.00 | 0.02 | 0.00 | 0.02 | 0.00 |
| Wallemia$_{VIP\geq0.8\ (+)}$ | 1.05 | 0.99 | 1.29 | 0.92 | 1.13 | 1.09 | 0.00 | 0.00 | -0.02 | 0.01 | 0.01 | 0.01 |
| Ichthyophthirius$_{VIP\geq0.8\ (+)}$ | 1.18 | 0.97 | 0.92 | 0.93 | 1.17 | 0.86 | 0.01 | 0.00 | 0.01 | 0.01 | 0.01 | 0.00 |
| Puccinia$_{VIP\geq0.8\ (+)}$ | 0.84 | 0.83 | 0.80 | 0.67 | 0.89 | 0.87 | 0.00 | 0.01 | 0.02 | 0.00 | 0.00 | 0.00 |
| Kluyveromyces$_{VIP\geq0.8\ (+)}$ | 0.99 | 0.62 | 1.09 | 0.67 | 0.94 | 1.33 | 0.01 | -0.01 | -0.02 | 0.00 | 0.01 | 0.01 |
| Bipolaris$_{VIP\geq0.8\ (+)}$ | 0.95 | 1.72 | 0.84 | 0.60 | 1.22 | 0.97 | 0.00 | -0.04 | 0.03 | 0.00 | 0.02 | 0.01 |
| Tetrapisispora$_{VIP\geq0.8\ (+)}$ | 1.46 | 0.92 | 0.98 | 0.97 | 0.94 | 0.87 | 0.01 | 0.00 | 0.00 | 0.01 | 0.00 | 0.00 |
| Tetrahymena$_{VIP\geq0.8\ (+)}$ | 1.13 | 1.03 | 0.86 | 0.87 | 1.11 | 0.87 | 0.01 | 0.00 | 0.02 | 0.00 | 0.01 | 0.00 |
| Cordyceps$_{VIP\geq0.8\ (+)}$ | 0.84 | 0.98 | 0.92 | 0.93 | 1.17 | 1.22 | 0.00 | 0.00 | 0.01 | 0.00 | 0.01 | 0.01 |
| Dictyostelium$_{VIP\geq0.8\ (+)}$ | 1.08 | 1.03 | 0.85 | 0.91 | 1.15 | 0.87 | 0.00 | 0.00 | 0.02 | 0.01 | 0.01 | 0.00 |
| Spathaspora$_{VIP\geq0.8\ (+)}$ | 0.96 | 0.76 | 0.96 | 0.79 | 1.09 | 1.02 | 0.00 | 0.02 | -0.01 | 0.00 | 0.01 | 0.01 |
| Kwoniella$_{VIP\geq0.8\ (+)}$ | 0.95 | 0.81 | 1.17 | 0.96 | 1.00 | 1.12 | 0.00 | 0.01 | -0.01 | 0.01 | 0.00 | 0.01 |
| Acidaminococcus$_{VIP\geq0.8\ (-)}$ | 0.78 | 1.13 | 0.89 | 1.06 | 0.92 | 0.87 | -0.01 | 0.01 | 0.00 | -0.01 | -0.01 | 0.00 |
| Enterococcus$_{VIP\geq0.8\ (-)}$ | 0.94 | 1.10 | 0.57 | 1.39 | 1.06 | 0.95 | -0.02 | -0.05 | 0.01 | -0.02 | -0.03 | -0.01 |

| | | | | | | | | | | | | |
|---|---|---|---|---|---|---|---|---|---|---|---|---|
| Paucibacter$_{VIP\geq0.8\ (-)}$ | 0.46 | 1.02 | 0.74 | 1.90 | 1.33 | 1.27 | 0.00 | -0.04 | -0.03 | -0.02 | -0.04 | -0.02 |
| Candidatus Paracaedibacter | 0.42 | 1.97 | 1.49 | 0.25 | 1.16 | 1.04 | 0.00 | -0.06 | -0.06 | 0.00 | -0.03 | -0.02 |
| Natronomonas$_{VIP\geq0.8\ (-)}$ | 1.11 | 1.61 | 0.97 | 1.31 | 0.69 | 2.25 | -0.02 | -0.05 | 0.03 | -0.02 | -0.01 | -0.04 |
| Phytophthora$_{VIP\geq0.8\ (-)}$ | 0.45 | 0.83 | 1.41 | 0.83 | 1.17 | 0.82 | 0.00 | -0.01 | -0.04 | -0.01 | -0.03 | -0.01 |
| Methanolacinia$_{VIP\geq0.8\ (-)}$ | 2.17 | 0.80 | 1.03 | 0.82 | 0.63 | 1.85 | -0.03 | -0.02 | 0.01 | -0.01 | -0.01 | -0.03 |
| Magnetospira$_{VIP\geq0.8\ (-)}$ | 1.86 | 1.16 | 0.78 | 1.74 | 1.43 | 1.50 | -0.03 | 0.02 | 0.00 | -0.02 | -0.03 | -0.02 |
| Methanoculleus$_{VIP\geq0.8\ (-)}$ | 1.61 | 0.64 | 1.24 | 0.64 | 1.34 | 1.12 | -0.02 | -0.01 | -0.07 | 0.00 | -0.03 | -0.02 |
| Serpula$_{VIP\geq0.8\ (-)}$ | 0.21 | 0.56 | 1.48 | 1.64 | 1.13 | 1.10 | 0.00 | 0.02 | -0.06 | -0.02 | -0.03 | -0.02 |
| Methanolobus$_{VIP\geq0.8\ (-)}$ | 2.34 | 0.84 | 2.32 | 0.62 | 1.09 | 0.34 | -0.04 | -0.01 | -0.09 | -0.01 | 0.03 | 0.00 |
| Rasamsonia$_{VIP\geq0.8\ (-)}$ | 1.42 | 0.28 | 1.11 | 1.09 | 0.88 | 0.60 | -0.03 | 0.00 | -0.01 | -0.01 | -0.02 | 0.00 |
| Pseudopropionibacterium$_{VIP}$ | 1.89 | 0.49 | 0.87 | 0.43 | 1.14 | 0.86 | -0.03 | -0.01 | -0.05 | 0.01 | -0.02 | -0.02 |
| Laccaria$_{VIP\geq0.8\ (-)}$ | 0.96 | 0.73 | 2.01 | 0.91 | 1.27 | 0.96 | -0.02 | 0.02 | -0.06 | -0.01 | 0.02 | -0.02 |
| Yangia$_{VIP\geq0.8\ (-)}$ | 1.10 | 0.71 | 0.96 | 1.46 | 0.97 | 2.02 | -0.02 | 0.00 | 0.03 | -0.02 | -0.02 | -0.03 |
| Acanthamoeba$_{VIP\geq0.8\ (-)}$ | 0.76 | 1.00 | 1.41 | 0.75 | 0.88 | 0.88 | -0.01 | -0.01 | -0.03 | 0.00 | 0.00 | -0.01 |
| Acidiphilium$_{VIP\geq0.8\ (-)}$ | 0.50 | 0.82 | 1.05 | 0.39 | 1.25 | 1.48 | -0.01 | 0.02 | -0.05 | 0.00 | -0.03 | -0.02 |
| Niastella$_{VIP\geq0.8\ (-)}$ | 1.58 | 0.70 | 0.06 | 0.96 | 0.91 | 1.05 | -0.03 | 0.01 | 0.00 | -0.01 | -0.03 | 0.01 |
| Hammondia$_{VIP\geq0.8\ (-)}$ | 1.32 | 1.29 | 1.31 | 0.87 | 1.08 | 0.76 | 0.02 | -0.02 | -0.03 | -0.01 | -0.03 | -0.01 |
| Dorea$_{VIP\geq0.8\ (-)}$ | 0.80 | 0.94 | 1.31 | 1.84 | 1.49 | 0.63 | -0.01 | -0.04 | 0.03 | -0.02 | -0.03 | 0.00 |
| Dictyoglomus$_{VIP\geq0.8\ (-)}$ | 0.51 | 0.58 | 0.95 | 1.05 | 0.94 | 0.87 | 0.00 | 0.02 | -0.02 | -0.01 | -0.02 | -0.01 |
| Moorella$_{VIP\geq0.8\ (-)}$ | 1.29 | 1.07 | 0.70 | 1.77 | 0.69 | 1.21 | -0.02 | 0.01 | -0.04 | -0.02 | 0.02 | -0.02 |
| Dehalococcoides$_{VIP\geq0.8\ (-)}$ | 0.68 | 1.09 | 1.11 | 1.67 | 1.07 | 1.12 | 0.00 | -0.05 | 0.02 | -0.02 | -0.02 | -0.01 |
| Theileria$_{VIP\geq0.8\ (-)}$ | 1.33 | 1.34 | 1.07 | 0.77 | 0.88 | 0.81 | 0.01 | -0.02 | -0.01 | -0.01 | -0.01 | -0.01 |
| Rhinocladiella$_{VIP\geq0.8\ (-)}$ | 0.83 | 1.00 | 1.14 | 0.73 | 0.95 | 0.77 | 0.00 | 0.00 | -0.01 | 0.00 | 0.00 | -0.01 |
| Pelagibaca$_{VIP\geq0.8\ (-)}$ | 1.02 | 0.98 | 0.53 | 1.09 | 1.46 | 0.44 | -0.01 | 0.01 | -0.03 | -0.01 | -0.03 | 0.00 |
| Pochonia$_{VIP\geq0.8\ (-)}$ | 0.71 | 1.12 | 1.26 | 0.83 | 0.89 | 0.89 | 0.00 | -0.02 | -0.02 | -0.01 | 0.00 | 0.01 |
| Chaetomium$_{VIP\geq0.8\ (-)}$ | 0.89 | 1.13 | 1.10 | 0.77 | 0.95 | 0.83 | 0.00 | -0.01 | 0.00 | 0.00 | 0.00 | 0.00 |
| Sedimenticola$_{VIP\geq0.8\ (-)}$ | 0.82 | 0.96 | 0.84 | 0.92 | 0.74 | 0.96 | 0.00 | 0.01 | 0.00 | 0.00 | 0.01 | 0.00 |
| Magnetospirillum$_{VIP\geq0.8\ (-)}$ | 1.04 | 0.82 | 0.75 | 0.94 | 0.48 | 1.24 | -0.01 | 0.02 | 0.01 | -0.01 | 0.00 | -0.02 |
| Methylibium$_{VIP\geq0.8\ (-)}$ | 0.87 | 0.85 | 0.68 | 1.05 | 0.91 | 0.78 | -0.01 | 0.00 | 0.00 | 0.01 | -0.01 | 0.01 |
| Botrytis$_{VIP\geq0.8\ (-)}$ | 0.77 | 0.76 | 1.11 | 0.82 | 0.87 | 0.86 | -0.01 | 0.01 | -0.01 | 0.00 | 0.00 | 0.00 |

| | | | | | | | | | | | | |
|---|---|---|---|---|---|---|---|---|---|---|---|---|
| $Pichia_{VIP≥0.8\ (-)}$ | 0.67 | 1.17 | 1.40 | 0.86 | 1.25 | 0.64 | 0.00 | -0.01 | -0.03 | 0.00 | 0.01 | 0.00 |
| $Blastocystis_{VIP≥0.8\ (-)}$ | 0.89 | 1.30 | 1.13 | 0.79 | 0.92 | 0.97 | 0.00 | -0.02 | -0.01 | 0.00 | -0.01 | 0.00 |
| $Sphaerochaeta_{VIP≥0.8\ (-)}$ | 1.12 | 0.86 | 1.11 | 1.39 | 0.80 | 1.32 | -0.01 | 0.00 | 0.02 | -0.01 | 0.00 | -0.02 |
| $Methanosarcina_{VIP≥0.8\ (-)}$ | 1.37 | 0.63 | 1.28 | 0.87 | 0.90 | 0.99 | 0.01 | -0.01 | -0.02 | -0.01 | 0.00 | 0.01 |
| $Calditerrivibrio_{VIP≥0.8\ (-)}$ | 0.96 | 0.85 | 0.51 | 0.62 | 1.26 | 1.12 | 0.01 | -0.02 | 0.00 | -0.01 | -0.04 | 0.01 |
| $Chlorobium_{VIP≥0.8\ (-)}$ | 1.03 | 0.77 | 0.80 | 0.94 | 0.88 | 0.91 | 0.00 | -0.02 | -0.01 | 0.00 | 0.00 | 0.01 |
| $Eimeria_{VIP≥0.8\ (-)}$ | 0.40 | 0.87 | 1.33 | 0.95 | 0.84 | 0.32 | -0.01 | 0.03 | -0.02 | -0.01 | -0.01 | 0.00 |
| $Methanothermococcus_{VIP≥0.8}$ | 1.18 | 1.19 | 0.88 | 0.84 | 0.91 | 0.74 | 0.01 | -0.01 | 0.00 | 0.00 | 0.00 | -0.01 |
| $Haloquadratum_{VIP≥0.8\ (-)}$ | 0.73 | 1.73 | 0.96 | 1.77 | 1.51 | 0.77 | -0.01 | -0.04 | -0.01 | 0.02 | 0.03 | -0.01 |
| $Heliobacterium_{VIP≥0.8\ (-)}$ | 0.96 | 1.14 | 0.71 | 0.74 | 0.86 | 0.93 | -0.01 | 0.02 | -0.03 | 0.00 | 0.02 | 0.00 |
| $Syntrophobotulus_{VIP≥0.8\ (-)}$ | 0.63 | 1.14 | 0.81 | 0.90 | 0.70 | 1.11 | 0.00 | -0.05 | -0.01 | -0.01 | 0.01 | -0.01 |
| $Spiribacter_{VIP≥0.8\ (-)}$ | 1.03 | 0.50 | 0.93 | 0.88 | 1.36 | 0.38 | 0.02 | -0.02 | -0.05 | -0.01 | -0.03 | 0.01 |
| $Chlorobaculum_{VIP≥0.8\ (-)}$ | 1.20 | 0.89 | 0.81 | 0.84 | 1.05 | 1.08 | 0.02 | 0.00 | -0.04 | 0.00 | -0.01 | -0.01 |
| $Shimwellia_{VIP≥0.8\ (-)}$ | 1.06 | 0.70 | 0.71 | 1.01 | 1.08 | 0.91 | -0.01 | -0.01 | -0.02 | 0.01 | -0.02 | 0.01 |
| $Corynebacterium_{VIP≥0.8\ (-)}$ | 1.01 | 0.68 | 0.84 | 0.73 | 0.95 | 1.30 | 0.00 | -0.02 | -0.01 | 0.00 | 0.02 | -0.01 |
| $Hydrogenophaga_{VIP≥0.8\ (-)}$ | 0.94 | 0.88 | 0.73 | 1.07 | 0.88 | 1.35 | 0.02 | 0.00 | -0.01 | -0.01 | -0.01 | -0.01 |
| $Gloeobacter_{VIP≥0.8\ (-)}$ | 0.98 | 0.91 | 0.80 | 0.87 | 1.06 | 0.90 | 0.00 | 0.01 | -0.01 | 0.00 | -0.01 | 0.00 |
| $Cloacibacillus_{VIP≥0.8\ (-)}$ | 0.80 | 0.98 | 0.81 | 0.80 | 0.85 | 1.37 | 0.00 | 0.00 | -0.04 | 0.00 | 0.00 | -0.01 |
| $Murdochiella_{VIP≥0.8\ (-)}$ | 0.60 | 0.87 | 0.97 | 0.80 | 1.14 | 1.19 | 0.00 | 0.00 | 0.01 | -0.01 | -0.02 | -0.01 |
| $Azorhizobium_{VIP≥0.8\ (-)}$ | 1.13 | 0.96 | 0.85 | 0.86 | 0.94 | 1.17 | -0.01 | 0.01 | 0.00 | 0.00 | -0.01 | -0.01 |
| $Geoalkalibacter_{VIP≥0.8\ (-)}$ | 0.88 | 0.79 | 0.77 | 0.86 | 0.94 | 1.09 | 0.00 | -0.01 | -0.03 | 0.00 | -0.01 | 0.00 |
| $Mageeibacillus_{VIP≥0.8\ (-)}$ | 0.85 | 0.76 | 0.86 | 0.92 | 0.79 | 0.83 | 0.00 | -0.03 | -0.01 | 0.00 | -0.01 | 0.00 |
| $Ketogulonicigenium_{VIP≥0.8\ (-)}$ | 0.72 | 1.77 | 0.72 | 1.36 | 0.96 | 1.00 | -0.01 | 0.05 | 0.00 | -0.02 | -0.01 | -0.01 |
| $Aeromicrobium_{VIP≥0.8}$ | 0.82 | 0.83 | 1.01 | 1.19 | 0.95 | 0.92 | 0.00 | -0.01 | 0.00 | 0.01 | 0.00 | 0.00 |
| $Aeromonas_{VIP≥0.8}$ | 0.89 | 0.92 | 0.93 | 0.95 | 0.92 | 0.89 | 0.02 | 0.00 | 0.00 | 0.00 | 0.00 | 0.01 |
| $Agromyces_{VIP≥0.8}$ | 0.91 | 0.92 | 0.81 | 0.95 | 1.34 | 0.93 | 0.00 | 0.00 | -0.01 | 0.01 | 0.04 | -0.01 |
| $Blastomyces_{VIP≥0.8}$ | 0.82 | 0.95 | 1.09 | 1.03 | 1.28 | 0.86 | 0.00 | 0.00 | -0.01 | 0.01 | 0.01 | 0.00 |
| $Bradyrhizobium_{VIP≥0.8}$ | 0.95 | 1.13 | 0.99 | 0.88 | 0.91 | 1.35 | 0.00 | 0.01 | 0.00 | 0.01 | 0.01 | -0.01 |
| $Chromobacterium_{VIP≥0.8}$ | 1.15 | 0.93 | 0.90 | 1.07 | 0.85 | 0.86 | -0.01 | 0.00 | 0.00 | 0.01 | 0.01 | 0.01 |
| $Cronobacter_{VIP≥0.8}$ | 0.93 | 0.86 | 0.98 | 0.94 | 0.92 | 0.91 | 0.00 | -0.01 | 0.00 | 0.00 | 0.00 | 0.01 |

| | | | | | | | | | | | | |
|---|---|---|---|---|---|---|---|---|---|---|---|---|
| $Frischella_{VIP≥0.8}$ | 0.92 | 0.93 | 0.80 | 0.94 | 1.11 | 0.85 | 0.00 | 0.00 | 0.00 | 0.00 | -0.02 | 0.01 |
| $Pandoraea_{VIP≥0.8}$ | 1.09 | 0.96 | 0.90 | 1.13 | 0.92 | 0.91 | 0.00 | 0.00 | 0.00 | 0.01 | 0.02 | 0.00 |
| $Phaeoacremonium_{VIP≥0.8}$ | 0.81 | 1.13 | 0.87 | 1.38 | 1.03 | 0.81 | -0.01 | -0.01 | 0.01 | 0.01 | 0.00 | 0.00 |
| $Aspergillus_{VIP≥0.8}$ | 0.73 | 0.88 | 0.80 | 0.85 | 0.85 | 1.14 | 0.00 | 0.00 | 0.01 | 0.01 | -0.01 | 0.01 |
| $Debaryomyces_{VIP≥0.8}$ | 1.26 | 0.96 | 0.95 | 0.83 | 1.40 | 0.68 | 0.01 | 0.00 | 0.01 | 0.00 | 0.02 | -0.01 |
| $Desulfococcus_{VIP≥0.8}$ | 1.35 | 0.92 | 0.82 | 0.77 | 1.24 | 0.83 | -0.01 | 0.00 | -0.02 | 0.00 | -0.01 | 0.00 |
| $Komagataella_{VIP≥0.8}$ | 0.67 | 1.12 | 0.87 | 0.97 | 0.83 | 1.05 | 0.00 | 0.05 | -0.01 | 0.01 | 0.00 | 0.01 |
| $Methanococcus_{VIP≥0.8}$ | 0.86 | 1.25 | 0.88 | 0.96 | 1.01 | 0.79 | 0.00 | -0.02 | 0.01 | -0.01 | 0.01 | 0.00 |
| $Paracoccidioides_{VIP≥0.8}$ | 0.84 | 1.01 | 0.90 | 0.78 | 0.97 | 0.84 | 0.00 | 0.00 | 0.01 | 0.00 | 0.00 | 0.00 |
| $Spirochaeta_{VIP≥0.8}$ | 1.04 | 0.85 | 1.41 | 1.00 | 1.05 | 0.67 | -0.01 | 0.00 | 0.03 | 0.01 | -0.01 | 0.01 |
| $Sporothrix_{VIP≥0.8}$ | 1.09 | 0.77 | 1.18 | 0.82 | 1.21 | 0.81 | 0.01 | 0.03 | -0.02 | 0.01 | -0.04 | -0.01 |
| $Trichoderma_{VIP≥0.8}$ | 0.84 | 0.89 | 1.29 | 0.69 | 1.07 | 0.88 | 0.00 | 0.01 | -0.02 | 0.00 | 0.01 | 0.00 |
| $Tuber_{VIP≥0.8}$ | 1.19 | 1.06 | 0.93 | 0.68 | 0.93 | 0.93 | 0.01 | -0.01 | 0.00 | 0.00 | 0.00 | 0.00 |
| $Adlercreutzia_{VIP≥0.8}$ | 0.89 | 0.85 | 1.15 | 0.86 | 0.79 | 0.64 | 0.00 | 0.00 | 0.01 | 0.00 | 0.02 | 0.00 |
| $Alicyclobacillus_{VIP≥0.8}$ | 0.94 | 0.94 | 0.71 | 0.78 | 0.87 | 1.31 | 0.00 | 0.00 | -0.02 | 0.00 | 0.00 | -0.01 |
| $Archangium_{VIP≥0.8}$ | 1.02 | 0.99 | 0.78 | 0.79 | 0.84 | 0.84 | 0.00 | 0.01 | -0.01 | 0.00 | 0.01 | -0.01 |
| $Dermacoccus_{VIP≥0.8}$ | 1.39 | 0.41 | 0.89 | 0.34 | 1.07 | 1.78 | -0.02 | -0.02 | 0.02 | 0.00 | 0.03 | -0.03 |
| $Frankia_{VIP≥0.8}$ | 1.15 | 0.74 | 1.24 | 0.77 | 1.70 | 1.15 | -0.01 | -0.02 | 0.02 | 0.00 | 0.05 | -0.01 |
| $Hyphomicrobium_{VIP≥0.8}$ | 0.95 | 0.50 | 0.62 | 1.18 | 1.21 | 1.44 | -0.02 | 0.02 | -0.03 | 0.02 | 0.03 | -0.02 |
| $Microbacterium_{VIP≥0.8}$ | 0.77 | 0.78 | 1.39 | 1.07 | 1.30 | 1.68 | 0.01 | -0.03 | 0.04 | -0.01 | 0.04 | -0.02 |
| $Rivularia_{VIP≥0.8}$ | 1.06 | 0.73 | 0.83 | 0.64 | 0.92 | 1.03 | 0.01 | 0.00 | 0.00 | 0.00 | 0.01 | -0.02 |
| $Sphingobium_{VIP≥0.8}$ | 0.67 | 0.99 | 0.78 | 0.96 | 0.87 | 0.97 | 0.00 | 0.01 | -0.03 | 0.01 | -0.01 | -0.01 |
| $Thermothelomyces_{VIP≥0.8}$ | 1.38 | 1.02 | 0.69 | 0.82 | 0.78 | 1.22 | -0.03 | -0.02 | 0.01 | 0.00 | 0.00 | 0.01 |
| *Achromobacter* | 0.89 | 0.79 | 1.82 | 0.68 | 0.81 | 0.48 | 0.02 | 0.01 | 0.07 | -0.01 | 0.02 | 0.00 |
| *Arsenicicoccus* | 1.79 | 0.82 | 0.61 | 0.71 | 0.69 | 1.61 | -0.03 | 0.01 | -0.02 | 0.01 | 0.01 | -0.03 |
| *Babjeviella* | 1.04 | 1.44 | 0.70 | 0.49 | 0.83 | 0.47 | 0.02 | 0.05 | 0.04 | 0.00 | 0.01 | -0.01 |
| *Candidatus Amoebophilus* | 0.75 | 1.35 | 0.83 | 0.79 | 0.54 | 0.84 | -0.01 | 0.06 | 0.00 | 0.00 | -0.02 | -0.02 |
| *Candidatus Methanomethylophilus* | 0.47 | 0.71 | 0.76 | 0.96 | 1.74 | 1.49 | 0.00 | -0.02 | -0.02 | 0.00 | -0.03 | -0.02 |
| *Colletotrichum* | 0.66 | 1.56 | 1.29 | 0.52 | 1.02 | 0.73 | 0.00 | -0.03 | -0.03 | 0.00 | 0.01 | -0.01 |
| *Dechloromonas* | 0.81 | 0.91 | 0.46 | 0.73 | 0.77 | 1.46 | 0.02 | 0.02 | 0.02 | 0.01 | 0.02 | 0.03 |

| | | | | | | | | | | | | |
|---|---|---|---|---|---|---|---|---|---|---|---|---|
| *Deinococcus* | 0.90 | 0.94 | 0.52 | 1.41 | 0.74 | 0.68 | -0.01 | 0.00 | -0.02 | 0.02 | 0.01 | -0.01 |
| *Desulfitobacterium* | 0.59 | 0.80 | 0.84 | 1.39 | 1.23 | 0.44 | 0.01 | -0.01 | -0.01 | -0.02 | -0.02 | 0.00 |
| *Dialister* | 0.62 | 0.81 | 0.97 | 1.27 | 0.68 | 0.78 | 0.01 | 0.00 | 0.02 | -0.01 | 0.01 | 0.00 |
| *Enterocytozoon* | 0.54 | 1.52 | 0.69 | 1.34 | 0.85 | 0.47 | 0.00 | -0.03 | 0.03 | -0.02 | 0.00 | 0.00 |
| *Fomitiporia* | 0.76 | 0.77 | 1.53 | 0.80 | 0.86 | 0.86 | -0.01 | 0.02 | -0.03 | 0.00 | 0.00 | 0.00 |
| *Jeongeupia* | 0.72 | 0.98 | 0.67 | 1.29 | 0.73 | 1.37 | 0.00 | 0.01 | -0.03 | -0.01 | 0.02 | -0.02 |
| *Kingella* | 0.66 | 1.26 | 1.04 | 0.80 | 0.87 | 0.71 | 0.01 | -0.05 | -0.05 | -0.01 | -0.03 | -0.01 |
| *Leptosphaeria* | 0.90 | 1.69 | 1.20 | 0.57 | 0.78 | 0.71 | 0.00 | -0.04 | -0.02 | 0.00 | -0.01 | 0.00 |
| *Luteipulveratus* | 1.84 | 0.45 | 0.92 | 0.72 | 0.80 | 1.09 | -0.03 | 0.00 | 0.05 | -0.01 | -0.01 | -0.02 |
| *Methanothermus* | 1.21 | 0.79 | 0.93 | 0.94 | 0.80 | 0.74 | -0.02 | 0.00 | 0.00 | 0.00 | 0.01 | -0.01 |
| *Methylacidiphilum* | 0.74 | 1.30 | 0.75 | 0.96 | 0.90 | 0.75 | -0.01 | 0.06 | 0.01 | 0.01 | 0.00 | 0.00 |
| *Nannizzia* | 0.54 | 0.55 | 1.19 | 0.99 | 0.80 | 1.94 | 0.00 | 0.01 | -0.04 | 0.01 | -0.02 | 0.03 |
| *Natrialba* | 1.71 | 1.57 | 0.75 | 1.05 | 0.80 | 0.23 | -0.03 | -0.06 | -0.04 | -0.01 | -0.02 | 0.00 |
| *Neospora* | 0.73 | 0.98 | 0.92 | 0.57 | 0.75 | 1.08 | -0.01 | -0.01 | 0.00 | -0.01 | -0.02 | 0.01 |
| *Pelobacter* | 0.70 | 0.38 | 0.92 | 1.57 | 0.63 | 1.41 | 0.00 | -0.01 | -0.05 | -0.02 | 0.01 | -0.02 |
| *Phaeobacter* | 1.11 | 0.80 | 0.57 | 1.51 | 0.54 | 1.03 | -0.01 | 0.01 | 0.02 | -0.02 | 0.00 | -0.01 |
| *Podospora* | 0.86 | 0.65 | 1.22 | 0.90 | 0.75 | 0.78 | 0.01 | 0.00 | -0.01 | -0.01 | 0.00 | 0.00 |
| *Polymorphum* | 1.23 | 1.78 | 0.44 | 1.42 | 0.72 | 0.37 | 0.02 | 0.05 | 0.00 | 0.02 | 0.02 | 0.01 |
| *Rhodomicrobium* | 0.91 | 0.72 | 0.77 | 1.16 | 0.77 | 0.87 | 0.02 | -0.02 | -0.04 | 0.01 | 0.00 | 0.01 |
| *Ruminiclostridium* | 0.43 | 1.25 | 0.71 | 1.57 | 0.09 | 1.66 | 0.00 | 0.04 | -0.04 | -0.02 | 0.00 | -0.03 |
| *Selenomonas* | 1.14 | 0.33 | 1.69 | 0.91 | 0.53 | 0.74 | 0.02 | 0.00 | 0.06 | -0.01 | 0.00 | 0.01 |
| *Succiniclasticum* | 0.31 | 0.87 | 0.76 | 2.07 | 0.70 | 0.85 | 0.00 | 0.04 | 0.04 | 0.03 | 0.01 | 0.01 |
| *Thermus* | 0.61 | 0.82 | 0.62 | 0.20 | 0.85 | 1.05 | 0.00 | 0.02 | -0.03 | 0.00 | 0.02 | -0.02 |
| *Thielavia* | 0.74 | 0.81 | 1.03 | 0.83 | 0.77 | 0.52 | -0.01 | 0.01 | -0.02 | -0.01 | -0.01 | 0.00 |
| *Thiobacimonas* | 0.81 | 0.94 | 0.77 | 0.77 | 0.90 | 0.76 | 0.00 | 0.00 | -0.01 | 0.01 | 0.02 | 0.00 |
| *Thiomonas* | 0.59 | 0.87 | 0.71 | 1.10 | 1.64 | 0.79 | 0.01 | 0.00 | -0.02 | -0.01 | -0.03 | 0.01 |
| *Toxoplasma* | 1.09 | 1.04 | 0.71 | 0.93 | 0.80 | 0.66 | 0.01 | -0.01 | 0.02 | 0.01 | -0.01 | -0.01 |
| *Trueperella* | 1.16 | 0.75 | 0.76 | 0.99 | 0.70 | 0.81 | -0.01 | -0.01 | -0.03 | 0.01 | 0.00 | 0.01 |
| *Veillonella* | 0.64 | 1.37 | 0.98 | 1.32 | 0.79 | 0.79 | 0.01 | 0.02 | 0.01 | -0.01 | 0.01 | 0.00 |
| *Actinomyces* | 0.95 | 0.47 | 0.73 | 0.63 | 0.62 | 1.48 | -0.01 | -0.01 | -0.03 | -0.01 | 0.01 | -0.02 |
| *Alteromonas* | 0.75 | 0.78 | 0.75 | 0.84 | 0.68 | 0.91 | 0.01 | 0.01 | -0.04 | 0.01 | 0.01 | 0.02 |
| *Brachyspira* | 0.79 | 0.92 | 0.79 | 0.72 | 1.14 | 0.74 | 0.00 | 0.00 | 0.04 | 0.00 | -0.03 | 0.01 |
| *Candidatus Sulcia* | 1.16 | 0.72 | 0.73 | 0.76 | 0.93 | 0.46 | -0.02 | 0.01 | 0.01 | -0.01 | -0.02 | -0.01 |
| *Clavispora* | 0.64 | 0.76 | 0.78 | 0.80 | 1.28 | 0.83 | 0.00 | 0.02 | 0.03 | 0.00 | 0.02 | 0.00 |
| *Deferribacter* | 0.78 | 1.01 | 0.77 | 0.90 | 0.76 | 0.74 | 0.00 | -0.02 | 0.02 | -0.01 | -0.01 | -0.01 |

| | | | | | | | | | | | | |
|---|---|---|---|---|---|---|---|---|---|---|---|---|
| *Gemmatimonas* | 0.92 | 0.50 | 0.74 | 1.10 | 0.72 | 0.75 | -0.02 | 0.00 | -0.04 | -0.01 | -0.01 | -0.01 |
| *Gregarina* | 0.75 | 0.74 | 0.76 | 0.91 | 1.10 | 0.62 | 0.00 | 0.03 | 0.02 | 0.01 | 0.02 | 0.00 |
| *Mycoplasma* | 0.68 | 0.70 | 0.79 | 1.15 | 1.12 | 0.42 | -0.01 | 0.01 | 0.03 | -0.01 | -0.03 | 0.00 |
| *Perkinsus* | 0.80 | 0.70 | 0.93 | 0.76 | 0.94 | 0.74 | 0.00 | 0.02 | 0.01 | 0.00 | -0.01 | -0.01 |
| *Sphaerulina* | 0.77 | 0.80 | 1.42 | 0.69 | 0.79 | 0.75 | -0.01 | 0.02 | -0.03 | 0.00 | -0.01 | -0.01 |
| *Thermococcus* | 0.70 | 0.67 | 0.74 | 0.85 | 0.78 | 1.01 | 0.00 | -0.01 | -0.04 | 0.01 | 0.00 | -0.01 |
| *Campylobacter* | 0.56 | 0.68 | 0.71 | 1.00 | 0.75 | 0.31 | 0.00 | 0.01 | 0.04 | -0.01 | -0.02 | 0.00 |
| *Olsenella* | 0.80 | 0.72 | 1.06 | 0.69 | 0.73 | 0.56 | -0.01 | -0.01 | 0.02 | -0.01 | 0.02 | 0.01 |

Partial Least Squares analysis was performed with 2 latent components. Subscript $_{VIP\geq0.8}$ denotes microbial genera that had VIP ≥ 0.8 in 4 or more timepoints. Subscripts $_{(+)}$ and $_{(-)}$ represent microbial genera that had positive or negative regression coefficient in 4 or more timepoints, respectively.

**Trait: Daily Feed Intake (DFI); Predictors: 270 microbial genera (MT)**

| Microbial genera | VIP | | | | | | Regression coefficient | | | | | |
|---|---|---|---|---|---|---|---|---|---|---|---|---|
| | T1 | T2 | T3 | T4 | T5 | T6 | T1 | T2 | T3 | T4 | T5 | T6 |
| Methanobrevibacter$_{VIP\geq0.8\ (+)}$ | 0.67 | 1.03 | 1.89 | 2.40 | 1.12 | 1.31 | 0.01 | 0.03 | 0.03 | 0.04 | 0.04 | 0.03 |
| Negativicoccus$_{VIP\geq0.8\ (+)}$ | 0.49 | 1.02 | 0.66 | 1.09 | 1.40 | 1.39 | 0.00 | 0.03 | 0.01 | 0.02 | 0.05 | 0.03 |
| Halapricum$_{VIP\geq0.8\ (+)}$ | 0.77 | 1.12 | 1.01 | 0.59 | 0.96 | 2.04 | 0.01 | 0.03 | 0.02 | 0.01 | 0.03 | 0.04 |
| Babjeviella$_{VIP\geq0.8\ (+)}$ | 2.06 | 1.97 | 0.28 | 1.03 | 0.82 | 1.17 | 0.03 | 0.06 | 0.00 | -0.01 | 0.01 | 0.02 |
| Candidatus Protochlamydia$_{V}$ | 2.08 | 1.58 | 0.66 | 0.74 | 0.96 | 0.85 | 0.03 | 0.04 | 0.00 | 0.00 | 0.03 | 0.01 |
| Methanothermobacter$_{VIP\geq0.8\ (}$ | 0.37 | 1.09 | 1.10 | 1.13 | 1.81 | 0.60 | 0.00 | 0.03 | 0.02 | 0.02 | 0.06 | 0.01 |
| Neofusicoccum$_{VIP\geq0.8\ (+)}$ | 1.62 | 1.76 | 1.34 | 1.46 | 0.88 | 0.68 | 0.02 | 0.05 | -0.02 | 0.03 | 0.02 | 0.01 |
| Caldisericum$_{VIP\geq0.8\ (+)}$ | 1.00 | 0.07 | 2.62 | 0.90 | 0.80 | 1.15 | 0.01 | 0.00 | 0.04 | 0.02 | 0.03 | 0.02 |
| Ustilago$_{VIP\geq0.8\ (+)}$ | 1.14 | 1.34 | 0.54 | 0.57 | 1.49 | 1.46 | 0.01 | 0.03 | 0.01 | 0.00 | 0.05 | 0.03 |
| Cylindrospermum$_{VIP\geq0.8\ (+)}$ | 1.59 | 0.26 | 1.76 | 1.74 | 0.87 | 2.21 | 0.02 | 0.01 | -0.03 | 0.03 | 0.03 | 0.05 |
| Methanobacterium$_{VIP\geq0.8\ (+)}$ | 0.62 | 1.18 | 1.02 | 1.26 | 1.59 | 0.77 | 0.01 | 0.03 | 0.02 | 0.03 | 0.05 | -0.02 |
| Nannizzia$_{VIP\geq0.8\ (+)}$ | 1.00 | 1.57 | 1.74 | 1.70 | 1.50 | 2.06 | 0.01 | 0.04 | 0.03 | 0.03 | -0.04 | 0.04 |
| Kluyvera$_{VIP\geq0.8\ (+)}$ | 1.81 | 1.20 | 0.57 | 0.95 | 0.19 | 1.15 | 0.02 | 0.02 | 0.01 | 0.02 | 0.00 | 0.03 |
| Glarea$_{VIP\geq0.8\ (+)}$ | 1.36 | 0.79 | 1.18 | 1.01 | 1.08 | 0.70 | 0.02 | 0.00 | 0.02 | 0.01 | 0.03 | 0.01 |
| Thielavia$_{VIP\geq0.8\ (+)}$ | 0.55 | 1.49 | 1.33 | 0.63 | 0.81 | 1.19 | 0.00 | 0.03 | 0.02 | 0.01 | 0.01 | 0.02 |
| Methylacidiphilum$_{VIP\geq0.8\ (+)}$ | 0.78 | 1.44 | 0.68 | 0.90 | 1.10 | 1.23 | 0.00 | 0.03 | 0.00 | 0.02 | 0.03 | 0.01 |

| | | | | | | | | | | | | |
|---|---|---|---|---|---|---|---|---|---|---|---|---|
| Eremothecium$_{VIP\geq0.8\ (+)}$ | 1.18 | 1.25 | 1.07 | 0.91 | 1.16 | 0.84 | 0.01 | 0.03 | 0.02 | 0.02 | -0.03 | 0.01 |
| Anabaena$_{VIP\geq0.8\ (+)}$ | 1.12 | 1.03 | 0.74 | 0.65 | 0.99 | 1.21 | 0.01 | 0.01 | 0.01 | 0.01 | 0.02 | 0.01 |
| Aspergillus$_{VIP\geq0.8\ (+)}$ | 0.88 | 1.00 | 0.67 | 0.87 | 0.92 | 1.37 | 0.00 | 0.01 | 0.01 | 0.02 | 0.01 | 0.02 |
| Endocarpon$_{VIP\geq0.8\ (+)}$ | 0.87 | 1.49 | 0.59 | 0.50 | 0.95 | 1.61 | 0.00 | 0.03 | 0.01 | 0.00 | 0.00 | 0.03 |
| Puccinia$_{VIP\geq0.8\ (+)}$ | 0.91 | 1.24 | 0.76 | 0.58 | 0.91 | 1.38 | 0.00 | 0.02 | 0.01 | 0.00 | 0.01 | 0.02 |
| Millerozyma$_{VIP\geq0.8\ (+)}$ | 1.27 | 1.54 | 1.54 | 0.59 | 0.94 | 1.46 | -0.02 | 0.04 | 0.03 | 0.01 | 0.01 | 0.02 |
| Trichoderma$_{VIP\geq0.8\ (+)}$ | 0.88 | 1.01 | 0.73 | 0.60 | 1.13 | 1.19 | 0.00 | 0.01 | 0.00 | 0.01 | 0.03 | 0.01 |
| Botrytis$_{VIP\geq0.8\ (+)}$ | 0.85 | 1.12 | 0.82 | 1.19 | 0.92 | 1.20 | -0.01 | 0.02 | 0.01 | 0.03 | 0.01 | 0.01 |
| Acytostelium$_{VIP\geq0.8\ (+)}$ | 0.97 | 1.19 | 1.05 | 0.88 | 0.96 | 0.98 | 0.01 | 0.02 | -0.01 | 0.02 | 0.00 | 0.01 |
| Aromatoleum$_{VIP\geq0.8\ (+)}$ | 0.87 | 0.86 | 0.97 | 0.93 | 1.11 | 0.97 | 0.01 | 0.01 | 0.01 | 0.01 | 0.02 | 0.02 |
| Saccharomyces$_{VIP\geq0.8\ (+)}$ | 1.25 | 0.84 | 0.80 | 0.61 | 1.10 | 1.17 | 0.01 | -0.01 | 0.00 | 0.01 | 0.03 | 0.01 |
| Methanofollis$_{VIP\geq0.8\ (+)}$ | 1.51 | 1.45 | 1.59 | 0.54 | 0.27 | 0.98 | -0.02 | 0.05 | 0.02 | 0.01 | 0.00 | 0.02 |
| Chelatococcus$_{VIP\geq0.8\ (+)}$ | 0.90 | 0.85 | 0.99 | 1.27 | 0.57 | 0.81 | 0.01 | 0.01 | 0.01 | 0.01 | 0.00 | 0.01 |
| Shinella$_{VIP\geq0.8\ (+)}$ | 1.02 | 1.12 | 1.03 | 0.40 | 1.07 | 1.11 | 0.01 | 0.04 | 0.01 | 0.00 | 0.02 | -0.02 |
| Dickeya$_{VIP\geq0.8\ (+)}$ | 1.15 | 0.86 | 0.68 | 0.98 | 0.94 | 0.86 | 0.01 | 0.02 | 0.00 | 0.01 | 0.00 | 0.02 |
| Phycomyces$_{VIP\geq0.8\ (+)}$ | 1.11 | 1.00 | 0.77 | 0.62 | 0.93 | 1.20 | 0.01 | 0.00 | 0.00 | 0.01 | 0.01 | 0.01 |
| Marssonina$_{VIP\geq0.8\ (+)}$ | 0.91 | 0.97 | 0.72 | 0.94 | 0.91 | 1.33 | 0.00 | 0.00 | 0.00 | 0.02 | 0.01 | 0.01 |
| Sporothrix$_{VIP\geq0.8\ (+)}$ | 1.14 | 1.35 | 0.65 | 0.69 | 0.84 | 1.07 | 0.01 | 0.02 | 0.00 | 0.00 | 0.00 | 0.01 |
| Hyphomonas$_{VIP\geq0.8\ (+)}$ | 1.05 | 0.94 | 0.73 | 0.70 | 1.06 | 0.86 | 0.01 | 0.00 | 0.00 | 0.00 | 0.02 | 0.02 |
| Sinomonas$_{VIP\geq0.8\ (+)}$ | 0.74 | 0.86 | 0.59 | 1.03 | 0.98 | 0.99 | 0.01 | 0.00 | 0.00 | 0.01 | 0.02 | 0.02 |
| Allisonella$_{VIP\geq0.8\ (+)}$ | 0.66 | 0.90 | 1.05 | 0.57 | 1.23 | 0.80 | 0.00 | 0.03 | 0.01 | -0.01 | 0.02 | 0.01 |
| Sorangium$_{VIP\geq0.8\ (+)}$ | 1.30 | 0.85 | 0.89 | 1.17 | 1.09 | 0.72 | 0.02 | 0.01 | -0.01 | 0.01 | 0.02 | 0.01 |
| Proteiniclasticum$_{VIP\geq0.8\ (+)}$ | 0.88 | 0.89 | 1.62 | 1.42 | 0.83 | 0.55 | -0.01 | 0.03 | 0.02 | 0.02 | 0.01 | 0.01 |
| Tsukamurella$_{VIP\geq0.8\ (+)}$ | 1.02 | 0.95 | 0.83 | 1.23 | 1.05 | 0.83 | 0.01 | 0.00 | 0.00 | 0.01 | 0.01 | 0.01 |
| Trypanosoma$_{VIP\geq0.8\ (+)}$ | 0.82 | 0.99 | 0.76 | 0.60 | 0.96 | 1.24 | 0.00 | 0.00 | 0.00 | 0.01 | 0.01 | 0.01 |
| Coraliomargarita$_{VIP\geq0.8\ (+)}$ | 0.96 | 0.92 | 0.64 | 1.09 | 1.35 | 0.84 | 0.01 | 0.00 | 0.00 | 0.01 | 0.03 | 0.01 |
| Neurospora$_{VIP\geq0.8\ (+)}$ | 0.96 | 0.96 | 0.75 | 0.63 | 0.96 | 1.51 | 0.00 | 0.00 | 0.00 | 0.01 | 0.00 | 0.02 |
| Microsporum$_{VIP\geq0.8\ (+)}$ | 0.93 | 1.03 | 0.77 | 0.61 | 0.96 | 1.19 | 0.00 | 0.01 | 0.00 | 0.01 | 0.01 | 0.01 |
| Schizosaccharomyces$_{VIP\geq0.8}$ | 0.92 | 1.00 | 0.99 | 0.63 | 0.87 | 0.92 | 0.00 | 0.00 | -0.01 | 0.01 | 0.01 | 0.01 |
| Microbacterium$_{VIP\geq0.8\ (+)}$ | 0.91 | 0.32 | 1.65 | 0.85 | 1.00 | 1.05 | 0.01 | 0.00 | 0.02 | 0.01 | 0.03 | -0.01 |

| | | | | | | | | | | | | |
|---|---|---|---|---|---|---|---|---|---|---|---|---|
| Thalassiosira$_{VIP≥0.8\ (+)}$ | 1.50 | 0.97 | 0.70 | 1.04 | 0.92 | 1.19 | -0.02 | 0.01 | 0.01 | 0.02 | 0.02 | 0.02 |
| Cytophaga$_{VIP≥0.8\ (+)}$ | 1.05 | 0.67 | 1.71 | 0.74 | 1.19 | 0.86 | -0.01 | 0.02 | 0.02 | 0.01 | 0.03 | 0.02 |
| Nodularia$_{VIP≥0.8\ (+)}$ | 0.94 | 0.93 | 1.10 | 0.59 | 1.11 | 0.95 | 0.00 | 0.00 | -0.01 | 0.01 | 0.03 | 0.00 |
| Methyloceanibacter$_{VIP≥0.8\ (+)}$ | 1.17 | 0.85 | 0.88 | 0.86 | 1.41 | 0.82 | 0.01 | 0.00 | -0.01 | 0.00 | 0.03 | 0.01 |
| Gordonia$_{VIP≥0.8\ (+)}$ | 0.96 | 0.97 | 0.72 | 1.31 | 1.04 | 0.84 | 0.01 | 0.00 | 0.00 | 0.01 | 0.01 | 0.02 |
| Bipolaris$_{VIP≥0.8\ (+)}$ | 0.85 | 0.93 | 0.94 | 1.37 | 0.81 | 1.48 | 0.00 | -0.03 | 0.01 | 0.03 | 0.00 | 0.02 |
| Scedosporium$_{VIP≥0.8\ (+)}$ | 0.87 | 0.87 | 0.77 | 0.62 | 0.88 | 0.82 | 0.00 | -0.01 | 0.01 | 0.01 | 0.02 | 0.00 |
| Histoplasma$_{VIP≥0.8\ (+)}$ | 0.80 | 0.91 | 0.79 | 0.60 | 0.97 | 1.30 | 0.00 | 0.00 | 0.00 | 0.01 | 0.01 | 0.01 |
| Dokdonella$_{VIP≥0.8\ (+)}$ | 1.09 | 0.93 | 0.71 | 1.05 | 1.01 | 0.85 | 0.01 | 0.00 | -0.01 | 0.01 | 0.01 | 0.01 |
| Tuber$_{VIP≥0.8\ (+)}$ | 1.30 | 0.79 | 0.86 | 0.59 | 0.92 | 1.06 | 0.01 | -0.01 | 0.00 | 0.01 | 0.01 | 0.01 |
| Halosimplex$_{VIP≥0.8\ (+)}$ | 0.96 | 0.92 | 0.69 | 1.04 | 0.79 | 0.85 | 0.01 | 0.00 | 0.00 | 0.01 | 0.00 | 0.02 |
| Perkinsus$_{VIP≥0.8\ (+)}$ | 0.95 | 1.21 | 0.75 | 0.62 | 1.00 | 0.94 | 0.00 | 0.02 | 0.00 | 0.00 | 0.00 | 0.00 |
| Talaromyces$_{VIP≥0.8\ (+)}$ | 0.89 | 1.00 | 0.82 | 0.82 | 1.00 | 1.29 | -0.01 | 0.01 | 0.01 | 0.00 | 0.01 | 0.01 |
| Cordyceps$_{VIP≥0.8\ (+)}$ | 0.84 | 0.90 | 0.77 | 0.58 | 0.98 | 1.33 | 0.00 | 0.00 | 0.00 | 0.01 | 0.01 | 0.01 |
| Arthrobacter$_{VIP≥0.8\ (+)}$ | 1.13 | 0.87 | 0.82 | 0.74 | 1.21 | 0.96 | 0.01 | 0.01 | 0.00 | 0.00 | 0.02 | 0.00 |
| Variovorax$_{VIP≥0.8\ (+)}$ | 1.12 | 0.82 | 0.79 | 0.89 | 1.03 | 0.86 | 0.01 | 0.00 | -0.01 | 0.00 | 0.01 | 0.01 |
| Plasmodium$_{VIP≥0.8\ (+)}$ | 0.89 | 0.97 | 0.89 | 0.73 | 1.03 | 1.10 | 0.00 | 0.00 | 0.00 | 0.00 | 0.02 | 0.01 |
| Sphaerulina$_{VIP≥0.8\ (+)}$ | 0.83 | 1.18 | 0.65 | 0.71 | 0.85 | 1.31 | 0.00 | 0.01 | 0.00 | 0.00 | 0.00 | 0.01 |
| Blastochloris$_{VIP≥0.8\ (+)}$ | 0.82 | 0.83 | 1.00 | 1.03 | 0.77 | 0.78 | 0.01 | 0.01 | 0.01 | 0.01 | 0.00 | 0.01 |
| Raoultella$_{VIP≥0.8\ (+)}$ | 0.86 | 1.00 | 0.70 | 0.87 | 0.87 | 0.95 | 0.01 | 0.00 | -0.01 | 0.01 | 0.01 | 0.02 |
| Methanococcus$_{VIP≥0.8\ (+)}$ | 0.78 | 0.90 | 0.92 | 0.58 | 1.12 | 1.05 | 0.00 | -0.03 | 0.01 | 0.00 | 0.03 | 0.01 |
| Nitrosospira$_{VIP≥0.8\ (+)}$ | 0.94 | 1.08 | 0.97 | 0.82 | 1.11 | 0.84 | 0.01 | -0.01 | 0.01 | 0.00 | 0.01 | 0.01 |
| Agrobacterium$_{VIP≥0.8\ (+)}$ | 1.04 | 1.00 | 1.04 | 0.76 | 1.05 | 0.92 | 0.01 | 0.00 | 0.01 | 0.00 | 0.01 | 0.01 |
| Thioflavicoccus$_{VIP≥0.8\ (+)}$ | 0.75 | 0.90 | 0.75 | 0.92 | 1.45 | 0.80 | 0.00 | 0.00 | -0.01 | 0.01 | 0.03 | 0.01 |
| Setosphaeria$_{VIP≥0.8\ (+)}$ | 0.95 | 0.89 | 0.71 | 0.58 | 0.86 | 0.92 | 0.00 | 0.00 | 0.00 | 0.01 | 0.01 | 0.00 |
| Chromobacterium$_{VIP≥0.8\ (+)}$ | 0.89 | 0.90 | 0.83 | 0.93 | 1.11 | 0.88 | 0.01 | 0.01 | -0.01 | 0.01 | 0.01 | 0.02 |
| Azospira$_{VIP≥0.8\ (+)}$ | 1.02 | 0.89 | 0.81 | 1.12 | 0.98 | 0.83 | 0.01 | 0.00 | -0.01 | 0.01 | 0.01 | 0.01 |
| Dactylellina$_{VIP≥0.8\ (+)}$ | 0.88 | 0.98 | 0.96 | 0.74 | 0.96 | 1.18 | 0.00 | 0.00 | -0.01 | 0.00 | 0.02 | 0.01 |
| Nostoc$_{VIP≥0.8\ (+)}$ | 1.00 | 0.86 | 1.19 | 0.67 | 0.92 | 0.98 | 0.01 | -0.01 | -0.01 | 0.00 | 0.02 | 0.00 |
| Ralstonia$_{VIP≥0.8\ (+)}$ | 0.91 | 0.93 | 0.76 | 0.81 | 1.14 | 0.86 | 0.01 | 0.00 | -0.01 | 0.00 | 0.02 | 0.01 |

| | | | | | | | | | | | | |
|---|---|---|---|---|---|---|---|---|---|---|---|---|
| Candidatus Ishikawaella$_{\mathrm{VIP}\geq 0}$ | 0.91 | 0.92 | 0.74 | 1.10 | 0.88 | 0.89 | 0.01 | 0.00 | 0.00 | 0.01 | 0.00 | 0.00 |
| Pannonibacter$_{\mathrm{VIP}\geq 0.8\ (+)}$ | 1.05 | 0.87 | 0.86 | 0.79 | 1.03 | 0.86 | 0.01 | 0.01 | -0.01 | 0.00 | 0.01 | 0.01 |
| Sinorhizobium$_{\mathrm{VIP}\geq 0.8\ (+)}$ | 0.99 | 0.89 | 1.00 | 0.69 | 0.97 | 0.85 | 0.00 | 0.02 | 0.01 | 0.00 | 0.01 | 0.01 |
| Halotalea$_{\mathrm{VIP}\geq 0.8\ (+)}$ | 1.08 | 0.95 | 0.82 | 0.61 | 0.91 | 0.87 | 0.01 | 0.00 | 0.00 | 0.00 | 0.00 | 0.01 |
| Nitrobacter$_{\mathrm{VIP}\geq 0.8\ (+)}$ | 0.92 | 0.86 | 0.83 | 0.74 | 1.13 | 0.83 | 0.01 | 0.01 | -0.01 | 0.00 | 0.02 | 0.01 |
| Cryptosporidium$_{\mathrm{VIP}\geq 0.8\ (+)}$ | 0.90 | 0.98 | 1.01 | 0.92 | 0.95 | 1.20 | 0.00 | 0.00 | -0.01 | 0.00 | 0.01 | 0.01 |
| Halolamina$_{\mathrm{VIP}\geq 0.8\ (+)}$ | 1.04 | 1.00 | 0.78 | 0.90 | 1.06 | 0.84 | 0.01 | 0.00 | -0.01 | 0.01 | 0.01 | 0.01 |
| Wenzhouxiangella$_{\mathrm{VIP}\geq 0.8\ (+)}$ | 0.95 | 0.89 | 0.77 | 0.82 | 1.02 | 0.85 | 0.01 | 0.00 | -0.01 | 0.00 | 0.01 | 0.01 |
| Pochonia$_{\mathrm{VIP}\geq 0.8\ (+)}$ | 1.22 | 0.78 | 1.06 | 0.65 | 0.89 | 1.03 | 0.01 | -0.02 | -0.01 | 0.00 | 0.01 | 0.01 |
| Microbulbifer$_{\mathrm{VIP}\geq 0.8\ (+)}$ | 1.12 | 0.99 | 0.86 | 0.87 | 0.93 | 0.85 | 0.01 | 0.00 | -0.01 | 0.00 | 0.00 | 0.01 |
| Natranaerobius$_{\mathrm{VIP}\geq 0.8\ (+)}$ | 0.13 | 0.90 | 0.48 | 0.89 | 1.03 | 0.92 | 0.00 | -0.02 | 0.00 | 0.01 | -0.03 | 0.02 |
| Halorhodospira$_{\mathrm{VIP}\geq 0.8\ (+)}$ | 0.90 | 0.95 | 0.75 | 0.69 | 1.12 | 0.87 | 0.00 | 0.00 | 0.00 | 0.00 | 0.01 | 0.01 |
| Sanguibacter$_{\mathrm{VIP}\geq 0.8\ (+)}$ | 1.01 | 0.95 | 0.83 | 0.88 | 1.06 | 0.86 | 0.01 | 0.00 | -0.01 | 0.00 | 0.01 | 0.01 |
| Zhongshania$_{\mathrm{VIP}\geq 0.8\ (+)}$ | 0.76 | 1.25 | 0.79 | 1.60 | 1.09 | 1.06 | 0.00 | -0.02 | -0.01 | 0.02 | 0.01 | 0.03 |
| Alkalilimnicola$_{\mathrm{VIP}\geq 0.8\ (+)}$ | 1.04 | 0.93 | 0.75 | 0.98 | 0.93 | 0.97 | 0.01 | 0.00 | -0.01 | 0.01 | 0.00 | 0.00 |
| Sideroxydans$_{\mathrm{VIP}\geq 0.8\ (+)}$ | 0.87 | 0.96 | 0.78 | 0.87 | 1.10 | 0.86 | 0.00 | 0.00 | 0.00 | 0.00 | 0.01 | 0.01 |
| Tolumonas$_{\mathrm{VIP}\geq 0.8\ (+)}$ | 0.80 | 1.04 | 0.68 | 1.17 | 0.94 | 0.75 | 0.01 | -0.01 | 0.00 | 0.01 | 0.01 | 0.01 |
| Wallemia$_{\mathrm{VIP}\geq 0.8\ (+)}$ | 0.99 | 0.93 | 1.40 | 0.62 | 0.89 | 1.21 | 0.00 | 0.00 | -0.02 | 0.01 | 0.01 | 0.01 |
| Veillonella$_{\mathrm{VIP}\geq 0.8\ (+)}$ | 0.62 | 0.84 | 0.87 | 1.03 | 1.16 | 0.74 | 0.00 | 0.02 | 0.01 | -0.02 | 0.02 | 0.01 |
| Modestobacter$_{\mathrm{VIP}\geq 0.8\ (+)}$ | 1.02 | 1.00 | 0.73 | 0.75 | 0.99 | 0.88 | 0.01 | 0.00 | -0.01 | 0.00 | 0.01 | 0.01 |
| Castellaniella$_{\mathrm{VIP}\geq 0.8\ (+)}$ | 0.84 | 0.92 | 0.67 | 0.93 | 1.08 | 0.87 | 0.00 | 0.00 | -0.01 | 0.01 | 0.01 | 0.01 |
| Thioalkalivibrio$_{\mathrm{VIP}\geq 0.8\ (+)}$ | 0.92 | 0.99 | 0.76 | 0.95 | 1.02 | 0.87 | 0.01 | 0.00 | -0.01 | 0.01 | 0.01 | 0.01 |
| Sedimenticola$_{\mathrm{VIP}\geq 0.8\ (+)}$ | 0.84 | 0.70 | 0.82 | 0.79 | 0.95 | 0.80 | 0.01 | 0.01 | -0.01 | 0.00 | 0.01 | 0.01 |
| Frischella$_{\mathrm{VIP}\geq 0.8\ (+)}$ | 0.90 | 0.95 | 0.71 | 0.85 | 0.84 | 0.87 | 0.01 | 0.00 | 0.00 | 0.01 | 0.00 | 0.01 |
| Thermothelomyces$_{\mathrm{VIP}\geq 0.8\ (+)}$ | 1.88 | 1.05 | 0.64 | 0.63 | 1.22 | 0.96 | -0.03 | -0.04 | 0.01 | 0.01 | 0.04 | 0.00 |
| Scheffersomyces$_{\mathrm{VIP}\geq 0.8\ (+)}$ | 1.14 | 0.82 | 1.06 | 0.59 | 0.91 | 1.03 | 0.01 | -0.02 | -0.01 | 0.01 | 0.01 | 0.01 |
| Bordetella$_{\mathrm{VIP}\geq 0.8\ (+)}$ | 0.82 | 0.88 | 0.83 | 0.82 | 0.98 | 0.76 | 0.00 | 0.00 | 0.00 | 0.00 | 0.01 | 0.00 |
| Kluyveromyces$_{\mathrm{VIP}\geq 0.8\ (+)}$ | 0.95 | 1.09 | 0.76 | 1.13 | 0.78 | 0.92 | 0.01 | -0.04 | 0.01 | -0.01 | 0.01 | 0.01 |
| Enterobacter$_{\mathrm{VIP}\geq 0.8\ (+)}$ | 0.97 | 1.00 | 0.75 | 0.88 | 0.84 | 0.82 | 0.01 | -0.01 | -0.01 | 0.01 | 0.00 | 0.01 |
| Rhizobium$_{\mathrm{VIP}\geq 0.8\ (+)}$ | 0.92 | 0.94 | 0.98 | 0.82 | 1.00 | 0.94 | 0.00 | 0.00 | 0.00 | 0.00 | 0.01 | 0.00 |

| | | | | | | | | | | | | |
|---|---|---|---|---|---|---|---|---|---|---|---|---|
| Aeromonas$_{VIP\geq0.8\ (+)}$ | 0.91 | 1.03 | 0.77 | 0.72 | 0.99 | 0.87 | 0.01 | 0.00 | -0.01 | 0.00 | 0.01 | 0.01 |
| Rhodomicrobium$_{VIP\geq0.8\ (+)}$ | 0.93 | 1.16 | 1.19 | 0.93 | 0.84 | 0.85 | 0.01 | -0.02 | -0.02 | 0.01 | 0.00 | 0.01 |
| Leclercia$_{VIP\geq0.8\ (+)}$ | 0.72 | 0.85 | 0.95 | 1.01 | 0.94 | 0.85 | 0.01 | 0.00 | -0.01 | 0.01 | 0.01 | 0.01 |
| Pseudomonas$_{VIP\geq0.8\ (+)}$ | 0.99 | 1.04 | 0.80 | 0.86 | 0.90 | 0.89 | 0.01 | -0.01 | -0.01 | 0.00 | 0.00 | 0.01 |
| Azoarcus$_{VIP\geq0.8\ (+)}$ | 0.90 | 0.89 | 0.97 | 0.80 | 0.89 | 0.83 | 0.01 | 0.01 | -0.01 | 0.00 | 0.00 | 0.01 |
| Chlorobium$_{VIP\geq0.8\ (+)}$ | 0.85 | 0.92 | 0.87 | 0.98 | 0.92 | 0.85 | 0.00 | 0.00 | -0.01 | 0.01 | 0.00 | 0.01 |
| Pantoea$_{VIP\geq0.8\ (+)}$ | 0.84 | 1.04 | 0.78 | 0.99 | 0.99 | 0.88 | 0.00 | -0.01 | -0.01 | 0.01 | 0.01 | 0.01 |
| Pandoraea$_{VIP\geq0.8\ (+)}$ | 0.90 | 0.87 | 1.06 | 0.72 | 1.20 | 0.80 | 0.00 | 0.00 | -0.02 | 0.00 | 0.02 | 0.01 |
| Mesorhizobium$_{VIP\geq0.8\ (+)}$ | 0.93 | 0.96 | 0.89 | 0.93 | 1.06 | 0.91 | 0.00 | 0.00 | -0.01 | 0.01 | 0.01 | 0.01 |
| Corynebacterium$_{VIP\geq0.8\ (+)}$ | 0.93 | 0.84 | 1.00 | 1.22 | 0.96 | 0.91 | 0.00 | -0.01 | 0.01 | 0.01 | 0.00 | 0.00 |
| Salinispira$_{VIP\geq0.8\ (+)}$ | 0.84 | 0.90 | 0.97 | 1.02 | 1.16 | 0.94 | 0.00 | 0.00 | -0.02 | 0.01 | 0.02 | 0.00 |
| Candidatus Koribacter$_{VIP\geq0.8}$ [illegible] | 0.76 | 0.81 | 1.09 | 0.69 | 1.30 | 0.81 | 0.00 | 0.01 | -0.02 | 0.00 | 0.03 | 0.00 |
| Porphyrobacter$_{VIP\geq0.8\ (+)}$ | 0.93 | 0.93 | 0.81 | 0.62 | 0.92 | 0.98 | 0.01 | 0.00 | 0.00 | 0.00 | 0.00 | 0.00 |
| Alcanivorax$_{VIP\geq0.8\ (+)}$ | 0.84 | 1.01 | 1.08 | 1.03 | 0.93 | 0.84 | 0.00 | 0.00 | -0.02 | 0.01 | 0.01 | 0.01 |
| Acidaminococcus$_{VIP\geq0.8\ (+)}$ | 0.83 | 0.83 | 1.19 | 0.65 | 0.94 | 0.77 | -0.01 | 0.01 | 0.01 | -0.01 | 0.01 | 0.01 |
| Geobacter$_{VIP\geq0.8\ (+)}$ | 0.90 | 0.82 | 0.83 | 0.92 | 0.94 | 0.87 | 0.01 | 0.01 | -0.01 | 0.00 | 0.00 | 0.00 |
| Desulfurispirillum$_{VIP\geq0.8\ (+)}$ | 0.88 | 0.87 | 1.30 | 0.83 | 0.92 | 0.83 | 0.01 | 0.01 | -0.02 | 0.00 | 0.00 | 0.01 |
| Cyanobium$_{VIP\geq0.8\ (+)}$ | 0.88 | 0.84 | 1.01 | 0.93 | 0.93 | 0.76 | 0.00 | 0.01 | -0.02 | 0.01 | 0.00 | 0.01 |
| Natronomonas$_{VIP\geq0.8\ (-)}$ | 1.25 | 0.66 | 0.37 | 1.31 | 1.32 | 2.13 | -0.02 | -0.02 | 0.00 | -0.03 | -0.04 | -0.05 |
| Fibrobacter$_{VIP\geq0.8\ (-)}$ | 0.96 | 0.71 | 0.49 | 0.90 | 1.99 | 0.87 | -0.01 | -0.01 | 0.00 | -0.01 | -0.06 | -0.02 |
| Cryptobacterium$_{VIP\geq0.8\ (-)}$ | 0.25 | 1.63 | 0.66 | 1.42 | 1.22 | 2.39 | 0.00 | -0.05 | 0.00 | -0.03 | -0.04 | -0.05 |
| Ammonifex$_{VIP\geq0.8\ (-)}$ | 1.75 | 0.10 | 1.18 | 2.71 | 0.29 | 1.54 | -0.02 | 0.00 | -0.02 | -0.05 | 0.00 | -0.03 |
| Dermacoccus$_{VIP\geq0.8\ (-)}$ | 1.59 | 0.86 | 0.93 | 1.37 | 0.84 | 2.03 | -0.02 | -0.03 | 0.01 | -0.03 | -0.02 | -0.04 |
| Synechocystis$_{VIP\geq0.8\ (-)}$ | 1.93 | 1.73 | 1.43 | 0.61 | 0.71 | 1.15 | -0.03 | -0.04 | -0.02 | 0.01 | -0.02 | -0.02 |
| Aerococcus$_{VIP\geq0.8\ (-)}$ | 1.44 | 1.64 | 0.43 | 1.60 | 0.92 | 0.46 | -0.02 | -0.05 | -0.01 | -0.03 | -0.03 | -0.01 |
| Magnetococcus$_{VIP\geq0.8\ (-)}$ | 1.83 | 1.89 | 1.04 | 1.23 | 0.51 | 0.80 | -0.02 | -0.06 | -0.01 | -0.02 | 0.02 | -0.02 |
| Acidiphilium$_{VIP\geq0.8\ (-)}$ | 0.61 | 0.38 | 1.05 | 1.29 | 1.08 | 1.47 | -0.01 | 0.01 | -0.02 | -0.03 | -0.04 | -0.03 |
| Malassezia$_{VIP\geq0.8\ (-)}$ | 1.54 | 1.59 | 0.81 | 2.41 | 0.55 | 0.91 | -0.02 | -0.05 | -0.01 | -0.04 | 0.02 | -0.02 |
| Croceibacter$_{VIP\geq0.8\ (-)}$ | 0.81 | 1.04 | 1.50 | 2.81 | 1.84 | 0.69 | -0.01 | 0.01 | -0.02 | -0.04 | -0.05 | -0.02 |
| Jeotgalibacillus$_{VIP\geq0.8\ (-)}$ | 1.49 | 1.70 | 0.45 | 0.15 | 0.90 | 0.98 | -0.02 | -0.05 | 0.01 | 0.00 | -0.03 | -0.02 |

| | | | | | | | | | | | | |
|---|---|---|---|---|---|---|---|---|---|---|---|---|
| Yangia$_{VIP\geq0.8\ (-)}$ | 1.31 | 1.00 | 1.08 | 1.25 | 1.11 | 1.72 | -0.02 | -0.02 | 0.02 | -0.03 | -0.04 | -0.03 |
| Rhodoferax$_{VIP\geq0.8\ (-)}$ | 1.27 | 0.92 | 1.19 | 0.65 | 1.19 | 1.28 | -0.01 | -0.01 | 0.01 | -0.01 | -0.04 | -0.03 |
| Ectothiorhodospira$_{VIP\geq0.8\ (-)}$ | 0.86 | 1.06 | 1.47 | 1.24 | 1.05 | 0.38 | -0.01 | -0.02 | -0.03 | -0.02 | 0.02 | -0.01 |
| Halopiger$_{VIP\geq0.8\ (-)}$ | 1.43 | 0.99 | 0.33 | 0.70 | 0.93 | 0.85 | -0.02 | -0.03 | 0.00 | 0.01 | -0.03 | -0.01 |
| Desulfobacter$_{VIP\geq0.8\ (-)}$ | 1.22 | 0.98 | 0.30 | 0.98 | 1.23 | 0.99 | -0.02 | -0.03 | 0.00 | -0.02 | 0.04 | -0.02 |
| Enterococcus$_{VIP\geq0.8\ (-)}$ | 1.47 | 1.30 | 1.39 | 0.72 | 0.46 | 0.87 | -0.02 | -0.03 | 0.02 | -0.01 | -0.02 | -0.01 |
| Luteipulveratus$_{VIP\geq0.8\ (-)}$ | 1.46 | 0.97 | 0.35 | 1.40 | 0.41 | 0.98 | -0.02 | -0.02 | 0.00 | -0.03 | -0.01 | -0.02 |
| Erysipelatoclostridium$_{VIP\geq0.8\ (}$ | 1.06 | 1.01 | 0.96 | 1.34 | 0.70 | 0.42 | -0.01 | -0.02 | 0.01 | -0.03 | -0.01 | 0.00 |
| Octadecabacter$_{VIP\geq0.8\ (-)}$ | 1.54 | 1.00 | 1.06 | 0.56 | 0.79 | 0.95 | -0.02 | 0.01 | -0.01 | 0.01 | -0.02 | -0.02 |
| Podospora$_{VIP\geq0.8\ (-)}$ | 0.62 | 0.84 | 0.85 | 1.20 | 1.50 | 0.73 | -0.01 | -0.03 | 0.00 | -0.01 | -0.04 | -0.02 |
| Candidatus Methanomethylc | 0.76 | 1.16 | 0.71 | 0.82 | 1.02 | 1.26 | 0.01 | -0.01 | 0.00 | -0.02 | -0.02 | -0.02 |
| Leptosphaeria$_{VIP\geq0.8\ (-)}$ | 0.85 | 0.92 | 1.51 | 1.14 | 1.52 | 1.28 | 0.00 | -0.03 | -0.02 | -0.01 | -0.04 | 0.02 |
| Heliobacterium$_{VIP\geq0.8\ (-)}$ | 1.23 | 0.81 | 0.86 | 0.60 | 0.76 | 1.35 | -0.01 | 0.02 | -0.01 | -0.01 | -0.01 | -0.02 |
| Caulobacter$_{VIP\geq0.8\ (-)}$ | 1.00 | 1.00 | 1.79 | 0.63 | 0.91 | 1.58 | -0.01 | -0.01 | 0.02 | -0.01 | 0.00 | -0.03 |
| Synechococcus$_{VIP\geq0.8\ (-)}$ | 0.88 | 1.37 | 0.75 | 0.71 | 0.91 | 1.23 | 0.00 | -0.02 | 0.00 | 0.00 | -0.01 | -0.01 |
| Thermococcus$_{VIP\geq0.8\ (-)}$ | 0.73 | 0.91 | 1.38 | 1.22 | 0.77 | 1.43 | 0.01 | 0.00 | -0.02 | 0.01 | -0.01 | -0.02 |
| Trichomonas$_{VIP\geq0.8\ (-)}$ | 1.18 | 0.86 | 0.69 | 0.89 | 1.12 | 0.78 | -0.02 | 0.00 | 0.01 | 0.00 | -0.02 | -0.02 |
| Azorhizobium$_{VIP\geq0.8\ (-)}$ | 1.21 | 1.08 | 0.74 | 0.71 | 0.93 | 0.89 | -0.01 | -0.01 | 0.00 | 0.00 | -0.01 | 0.00 |
| Deferribacter$_{VIP\geq0.8\ (-)}$ | 0.85 | 0.75 | 0.92 | 1.46 | 0.88 | 0.84 | -0.01 | -0.02 | 0.01 | -0.02 | -0.01 | -0.02 |
| Bernardetia$_{VIP\geq0.8\ (-)}$ | 0.93 | 0.62 | 0.87 | 1.26 | 1.21 | 0.18 | -0.01 | 0.00 | -0.01 | -0.02 | -0.03 | 0.00 |
| Methylobacterium$_{VIP\geq0.8\ (-)}$ | 0.86 | 0.95 | 0.71 | 0.61 | 0.92 | 1.03 | 0.00 | -0.01 | 0.00 | -0.01 | -0.01 | -0.01 |
| Rathayibacter$_{VIP\geq0.8\ (-)}$ | 0.72 | 0.97 | 1.25 | 0.44 | 0.97 | 0.97 | 0.00 | -0.01 | 0.01 | -0.01 | -0.03 | -0.01 |
| Chlorobaculum$_{VIP\geq0.8\ (-)}$ | 1.12 | 0.95 | 1.72 | 0.58 | 0.92 | 1.12 | 0.01 | 0.00 | -0.03 | -0.01 | 0.00 | -0.01 |
| Alicyclobacillus$_{VIP\geq0.8\ (-)}$ | 0.83 | 1.07 | 0.74 | 0.60 | 1.19 | 1.19 | 0.00 | -0.01 | -0.01 | -0.01 | 0.02 | -0.01 |
| Stigmatella$_{VIP\geq0.8\ (-)}$ | 1.15 | 1.03 | 0.67 | 0.60 | 0.92 | 0.88 | -0.01 | -0.01 | 0.00 | 0.00 | 0.00 | 0.00 |
| Mageeibacillus$_{VIP\geq0.8\ (-)}$ | 0.79 | 1.05 | 0.81 | 0.86 | 0.67 | 0.91 | 0.01 | -0.01 | -0.01 | 0.00 | -0.01 | -0.01 |
| Nakaseomyces$_{VIP\geq0.8\ (-)}$ | 0.72 | 0.78 | 0.84 | 1.80 | 0.93 | 0.90 | 0.00 | -0.01 | 0.00 | 0.04 | 0.01 | 0.00 |
| Trueperella$_{VIP\geq0.8\ (-)}$ | 1.19 | 0.79 | 0.89 | 1.48 | 0.49 | 1.00 | -0.01 | 0.00 | -0.01 | 0.02 | 0.00 | -0.01 |
| Acaryochloris$_{VIP\geq0.8\ (-)}$ | 0.95 | 1.12 | 0.94 | 0.56 | 0.82 | 0.89 | 0.01 | -0.01 | -0.01 | -0.01 | -0.01 | 0.00 |
| Thiomonas$_{VIP\geq0.8\ (-)}$ | 0.86 | 0.95 | 0.92 | 0.63 | 0.93 | 1.06 | 0.00 | 0.00 | -0.01 | 0.00 | -0.02 | 0.03 |

| | | | | | | | | | | | | |
|---|---|---|---|---|---|---|---|---|---|---|---|---|
| $Mannheimia_{VIP≥0.8\ (-)}$ | 0.85 | 1.21 | 0.97 | 1.92 | 0.25 | 0.27 | 0.01 | 0.03 | -0.02 | -0.03 | -0.01 | -0.01 |
| $Nitrosopumilus_{VIP≥0.8\ (-)}$ | 0.75 | 1.13 | 0.75 | 1.36 | 0.87 | 0.94 | -0.01 | 0.02 | 0.00 | -0.02 | 0.00 | -0.02 |
| $Sphingomonas_{VIP≥0.8\ (-)}$ | 0.86 | 1.05 | 0.78 | 0.63 | 0.94 | 0.96 | 0.01 | -0.01 | -0.01 | 0.00 | -0.01 | 0.00 |
| $Jeongeupia_{VIP≥0.8\ (-)}$ | 0.94 | 0.85 | 1.03 | 0.80 | 1.01 | 0.87 | 0.01 | -0.01 | -0.02 | -0.02 | 0.01 | -0.01 |
| $Acidihalobacter_{VIP≥0.8\ (-)}$ | 0.99 | 1.00 | 0.66 | 1.63 | 0.69 | 1.09 | 0.00 | -0.01 | 0.00 | 0.02 | -0.01 | -0.01 |
| $Phialocephala_{VIP≥0.8\ (-)}$ | 0.89 | 0.82 | 1.03 | 0.94 | 0.98 | 1.03 | 0.00 | 0.00 | -0.01 | -0.01 | 0.00 | 0.01 |
| $Theileria_{VIP≥0.8\ (-)}$ | 1.20 | 0.78 | 1.20 | 1.15 | 0.87 | 0.79 | 0.01 | -0.02 | -0.01 | -0.01 | 0.00 | -0.01 |
| $Debaryomyces_{VIP≥0.8\ (-)}$ | 0.77 | 0.91 | 0.93 | 0.93 | 0.98 | 0.80 | 0.00 | -0.01 | 0.00 | -0.01 | 0.02 | 0.00 |
| $Yamadazyma_{VIP≥0.8\ (-)}$ | 0.69 | 0.96 | 0.84 | 0.84 | 0.83 | 0.95 | 0.00 | -0.03 | 0.00 | 0.00 | 0.00 | 0.00 |
| $Candida_{VIP≥0.8\ (-)}$ | 0.89 | 0.89 | 0.97 | 0.80 | 0.97 | 1.11 | 0.00 | -0.01 | -0.01 | 0.00 | 0.01 | 0.01 |
| $Fusarium_{VIP≥0.8\ (-)}$ | 0.88 | 0.85 | 0.55 | 0.80 | 0.96 | 1.16 | 0.00 | 0.00 | 0.00 | -0.01 | -0.01 | 0.01 |
| $Blastocystis_{VIP≥0.8\ (-)}$ | 0.85 | 0.84 | 1.12 | 0.99 | 0.94 | 0.96 | 0.00 | -0.02 | -0.01 | -0.01 | 0.01 | 0.00 |
| $Paracoccidioides_{VIP≥0.8\ (-)}$ | 0.85 | 0.85 | 0.98 | 0.85 | 0.93 | 1.01 | -0.01 | -0.01 | -0.01 | 0.00 | 0.01 | 0.01 |
| $Dictyostelium_{VIP≥0.8\ (-)}$ | 0.88 | 0.88 | 0.93 | 0.84 | 0.94 | 1.02 | 0.00 | -0.01 | -0.01 | 0.00 | 0.01 | 0.01 |
| $Wickerhamomyces_{VIP≥0.8\ (-)}$ | 0.88 | 1.11 | 0.91 | 1.16 | 0.91 | 1.01 | 0.00 | 0.01 | -0.01 | -0.01 | 0.00 | 0.00 |
| $Pluralibacter_{VIP≥0.8\ (-)}$ | 0.93 | 0.93 | 0.72 | 0.64 | 0.89 | 1.04 | 0.01 | 0.00 | -0.01 | 0.00 | -0.01 | 0.00 |
| $Naumovozyma_{VIP≥0.8\ (-)}$ | 0.84 | 0.76 | 0.86 | 1.03 | 0.84 | 0.90 | 0.00 | -0.01 | 0.00 | -0.01 | 0.00 | 0.00 |
| $Chaetomium_{VIP≥0.8\ (-)}$ | 0.84 | 0.83 | 0.92 | 0.88 | 0.89 | 1.25 | 0.00 | -0.01 | 0.00 | 0.00 | 0.01 | 0.01 |
| $Rhinocladiella_{VIP≥0.8\ (-)}$ | 0.88 | 0.81 | 1.06 | 0.60 | 0.98 | 1.25 | 0.00 | -0.01 | -0.01 | 0.01 | 0.00 | 0.01 |
| $Spathaspora_{VIP≥0.8\ (-)}$ | 0.87 | 1.26 | 1.66 | 1.05 | 0.92 | 1.08 | 0.00 | 0.02 | -0.02 | -0.01 | 0.00 | 0.01 |
| $Sclerotinia_{VIP≥0.8\ (-)}$ | 0.92 | 0.82 | 1.31 | 0.76 | 0.91 | 1.25 | 0.00 | -0.01 | -0.01 | 0.00 | 0.00 | 0.01 |
| $Herbaspirillum_{VIP≥0.8\ (-)}$ | 1.08 | 0.85 | 0.71 | 0.60 | 0.93 | 0.88 | -0.01 | 0.01 | 0.00 | 0.00 | 0.00 | 0.02 |
| $Arthrobotrys_{VIP≥0.8}$ | 0.90 | 0.85 | 0.98 | 0.95 | 1.01 | 0.95 | 0.00 | -0.01 | -0.01 | -0.01 | 0.02 | 0.00 |
| $Dyella_{VIP≥0.8}$ | 0.84 | 1.09 | 0.83 | 0.98 | 0.94 | 1.10 | 0.01 | -0.01 | 0.00 | 0.01 | -0.01 | 0.00 |
| $Ichthyophthirius_{VIP≥0.8}$ | 0.90 | 0.88 | 1.10 | 0.91 | 0.94 | 0.95 | 0.00 | -0.01 | -0.01 | 0.00 | 0.01 | 0.00 |
| $Methanolacinia_{VIP≥0.8}$ | 1.16 | 1.14 | 1.35 | 1.59 | 1.41 | 1.22 | -0.01 | -0.01 | 0.02 | 0.02 | 0.04 | -0.02 |
| $Nosema_{VIP≥0.8}$ | 0.89 | 1.00 | 0.92 | 0.90 | 0.90 | 1.02 | -0.01 | 0.00 | -0.01 | 0.00 | 0.01 | 0.01 |
| $Tetrahymena_{VIP≥0.8}$ | 0.90 | 0.91 | 0.88 | 0.96 | 0.91 | 0.95 | 0.00 | 0.00 | 0.00 | -0.01 | 0.01 | 0.00 |
| $Asticcacaulis_{VIP≥0.8}$ | 1.52 | 0.85 | 0.88 | 0.54 | 0.89 | 0.89 | 0.02 | 0.01 | -0.01 | -0.01 | -0.01 | 0.00 |
| $Basfia_{VIP≥0.8}$ | 0.84 | 0.83 | 1.10 | 1.14 | 0.28 | 1.02 | 0.00 | 0.01 | -0.01 | -0.02 | -0.01 | 0.02 |

| | | | | | | | | | | | | |
|---|---|---|---|---|---|---|---|---|---|---|---|---|
| Clavibacter$_{VIP\geq0.8}$ | 0.87 | 0.87 | 0.80 | 0.79 | 0.85 | 1.22 | 0.00 | 0.00 | 0.00 | 0.00 | 0.01 | -0.01 |
| Dyadobacter$_{VIP\geq0.8}$ | 1.17 | 1.47 | 1.10 | 1.53 | 1.06 | 0.60 | 0.02 | 0.04 | -0.01 | -0.02 | 0.03 | -0.01 |
| Faecalibaculum$_{VIP\geq0.8}$ | 0.81 | 1.00 | 1.13 | 0.34 | 0.90 | 1.27 | 0.00 | -0.01 | 0.01 | -0.01 | 0.02 | 0.03 |
| Faecalitalea$_{VIP\geq0.8}$ | 0.97 | 1.36 | 1.61 | 0.94 | 1.23 | 0.65 | -0.01 | -0.03 | 0.02 | -0.02 | 0.03 | 0.00 |
| Hyphomicrobium$_{VIP\geq0.8}$ | 1.34 | 0.81 | 1.54 | 1.67 | 1.01 | 0.57 | -0.02 | 0.02 | -0.02 | 0.03 | 0.03 | -0.01 |
| Neisseria$_{VIP\geq0.8}$ | 1.44 | 0.92 | 1.21 | 0.67 | 0.91 | 0.85 | 0.02 | 0.00 | -0.02 | 0.00 | 0.01 | 0.02 |
| Sulfurovum$_{VIP\geq0.8}$ | 1.12 | 0.82 | 0.92 | 1.26 | 0.90 | 0.70 | 0.02 | -0.01 | 0.01 | -0.03 | -0.02 | 0.02 |
| Syntrophobotulus$_{VIP\geq0.8}$ | 0.96 | 1.85 | 0.97 | 0.49 | 1.01 | 0.82 | -0.01 | -0.05 | 0.01 | -0.01 | 0.01 | 0.00 |
| Xylona$_{VIP\geq0.8}$ | 0.89 | 0.86 | 0.84 | 0.75 | 0.90 | 1.09 | 0.00 | -0.01 | 0.00 | 0.00 | 0.01 | 0.00 |
| Zygosaccharomyces$_{VIP\geq0.8}$ | 0.87 | 0.88 | 0.94 | 0.72 | 0.93 | 1.04 | 0.00 | -0.01 | 0.00 | 0.00 | 0.01 | 0.01 |
| Agaricus$_{VIP\geq0.8}$ | 0.84 | 0.99 | 0.60 | 1.02 | 1.11 | 0.73 | 0.01 | 0.01 | 0.00 | -0.01 | -0.01 | 0.01 |
| Aminomonas$_{VIP\geq0.8}$ | 0.92 | 0.96 | 0.80 | 0.67 | 1.04 | 1.00 | 0.01 | 0.00 | 0.00 | 0.00 | 0.01 | -0.01 |
| Burkholderia$_{VIP\geq0.8}$ | 1.05 | 0.87 | 0.78 | 0.67 | 0.91 | 0.91 | 0.01 | 0.01 | -0.01 | 0.00 | 0.00 | 0.00 |
| Chromohalobacter$_{VIP\geq0.8}$ | 1.05 | 0.83 | 0.69 | 1.70 | 0.98 | 0.61 | 0.01 | 0.02 | 0.00 | -0.03 | -0.03 | 0.00 |
| Cronobacter$_{VIP\geq0.8}$ | 1.00 | 1.02 | 0.76 | 0.71 | 0.99 | 0.88 | 0.01 | 0.00 | 0.00 | 0.00 | 0.01 | 0.01 |
| Elizabethkingia$_{VIP\geq0.8}$ | 0.78 | 1.27 | 2.53 | 1.03 | 0.22 | 1.06 | -0.01 | -0.03 | 0.04 | 0.02 | 0.00 | 0.02 |
| Geoalkalibacter$_{VIP\geq0.8}$ | 0.99 | 1.00 | 0.79 | 0.72 | 0.99 | 0.86 | 0.01 | 0.00 | 0.00 | 0.00 | 0.01 | 0.01 |
| Geosporobacter$_{VIP\geq0.8}$ | 0.20 | 0.60 | 0.99 | 1.35 | 0.99 | 0.92 | 0.00 | 0.00 | 0.01 | -0.03 | 0.03 | -0.02 |
| Guillardia$_{VIP\geq0.8}$ | 0.92 | 0.90 | 1.03 | 0.85 | 0.78 | 0.71 | 0.00 | 0.00 | -0.01 | 0.00 | 0.02 | -0.02 |
| Methanocaldococcus$_{VIP\geq0.8}$ | 0.78 | 0.91 | 0.82 | 0.72 | 0.95 | 0.85 | -0.01 | -0.03 | 0.01 | 0.02 | 0.00 | -0.01 |
| Methanothermus$_{VIP\geq0.8}$ | 1.16 | 0.80 | 0.98 | 0.60 | 0.81 | 0.72 | -0.01 | -0.02 | 0.02 | 0.01 | 0.02 | -0.01 |
| Methanotorris$_{VIP\geq0.8}$ | 0.93 | 0.70 | 1.07 | 0.79 | 1.00 | 1.38 | -0.01 | -0.02 | 0.02 | 0.02 | -0.01 | 0.02 |
| Phaeoacremonium$_{VIP\geq0.8}$ | 1.23 | 0.92 | 0.78 | 1.05 | 0.93 | 0.80 | -0.02 | 0.00 | 0.00 | 0.02 | 0.02 | 0.00 |
| Tetrapisispora$_{VIP\geq0.8}$ | 1.14 | 0.87 | 0.71 | 0.78 | 0.97 | 0.97 | 0.01 | 0.00 | 0.00 | 0.00 | 0.00 | 0.00 |
| *Alicycliphilus* | 0.54 | 0.78 | 1.00 | 1.05 | 1.89 | 0.66 | -0.01 | 0.03 | -0.02 | -0.02 | 0.06 | 0.02 |
| *Arsenophonus* | 1.11 | 0.66 | 0.75 | 1.55 | 0.95 | 0.69 | -0.01 | -0.01 | 0.01 | -0.02 | -0.01 | -0.01 |
| *Bacteroides* | 0.77 | 0.47 | 1.40 | 1.70 | 0.89 | 0.39 | -0.01 | 0.01 | 0.02 | 0.02 | -0.03 | 0.01 |
| *Blattabacterium* | 0.90 | 0.65 | 0.76 | 1.37 | 0.96 | 0.65 | -0.01 | -0.02 | 0.01 | -0.01 | -0.01 | -0.01 |
| *Candidatus Paracaedibacter* | 0.95 | 2.12 | 1.22 | 0.32 | 0.72 | 0.79 | 0.01 | -0.07 | -0.02 | 0.00 | -0.02 | -0.02 |
| *Chloroflexus* | 1.44 | 0.77 | 1.38 | 1.04 | 0.48 | 0.72 | 0.02 | 0.03 | 0.02 | 0.01 | 0.00 | 0.02 |

| | | | | | | | | | | | | |
|---|---|---|---|---|---|---|---|---|---|---|---|---|
| *Clavispora* | 0.59 | 1.19 | 0.73 | 0.58 | 1.29 | 1.00 | 0.00 | 0.02 | 0.01 | 0.01 | 0.04 | 0.01 |
| *Cloacibacillus* | 0.74 | 1.09 | 0.93 | 0.59 | 0.93 | 0.66 | 0.00 | -0.01 | -0.01 | -0.01 | -0.01 | 0.00 |
| *Coriobacterium* | 0.77 | 0.88 | 1.16 | 0.63 | 1.22 | 0.66 | 0.00 | -0.01 | 0.01 | 0.00 | 0.03 | 0.00 |
| *Cyanothece* | 0.69 | 0.84 | 0.95 | 0.67 | 0.89 | 0.68 | 0.00 | 0.00 | -0.01 | 0.00 | -0.01 | -0.01 |
| *Desulfocapsa* | 0.40 | 1.60 | 2.18 | 0.78 | 0.94 | 0.65 | 0.00 | 0.06 | 0.03 | 0.01 | 0.02 | 0.01 |
| *Desulfococcus* | 1.07 | 0.82 | 0.75 | 0.76 | 0.92 | 0.77 | -0.01 | 0.01 | -0.01 | 0.00 | -0.01 | 0.01 |
| *Dialister* | 0.67 | 0.82 | 1.11 | 0.53 | 1.38 | 0.74 | 0.00 | 0.02 | 0.01 | -0.01 | 0.03 | 0.01 |
| *Dorea* | 1.28 | 0.70 | 0.82 | 1.07 | 0.62 | 0.76 | -0.01 | 0.01 | 0.01 | -0.02 | -0.01 | -0.01 |
| *Frankia* | 0.75 | 0.97 | 0.74 | 0.56 | 1.50 | 0.94 | 0.00 | -0.01 | 0.00 | -0.01 | 0.04 | -0.01 |
| *Gloeobacter* | 0.85 | 0.97 | 0.74 | 0.60 | 1.10 | 0.75 | 0.01 | -0.01 | -0.01 | 0.00 | -0.03 | 0.01 |
| *Gregarina* | 0.71 | 0.78 | 0.73 | 1.11 | 0.99 | 0.85 | 0.00 | -0.02 | 0.00 | -0.01 | 0.03 | 0.01 |
| *Isaria* | 0.56 | 1.04 | 0.77 | 0.62 | 1.09 | 1.16 | 0.00 | 0.01 | 0.01 | 0.01 | 0.03 | 0.01 |
| *Kalmanozyma* | 0.79 | 0.95 | 0.76 | 1.13 | 0.67 | 0.83 | 0.01 | 0.00 | -0.01 | 0.01 | 0.00 | 0.01 |
| *Kosakonia* | 0.35 | 1.08 | 0.84 | 0.64 | 0.94 | 0.78 | 0.00 | -0.02 | -0.01 | -0.01 | -0.03 | 0.01 |
| *Lachnospira* | 0.60 | 0.73 | 0.90 | 1.53 | 0.98 | 0.57 | 0.01 | 0.02 | 0.01 | -0.03 | 0.02 | -0.01 |
| *Marinithermus* | 0.65 | 1.08 | 0.42 | 1.31 | 1.34 | 0.75 | 0.01 | 0.04 | 0.00 | -0.03 | -0.04 | 0.02 |
| *Massilia* | 1.33 | 0.64 | 0.99 | 0.71 | 0.90 | 0.69 | 0.02 | 0.02 | -0.02 | 0.00 | 0.00 | 0.01 |
| *Methanomassiliicoccus* | 0.68 | 0.87 | 0.67 | 1.07 | 1.13 | 0.77 | 0.00 | 0.01 | 0.00 | 0.01 | 0.02 | 0.01 |
| *Methanosarcina* | 0.94 | 0.76 | 0.75 | 0.58 | 0.93 | 1.05 | 0.00 | -0.02 | 0.01 | 0.01 | 0.01 | 0.01 |
| *Mycoplasma* | 0.82 | 0.62 | 0.96 | 0.76 | 1.16 | 0.43 | -0.01 | -0.01 | 0.01 | 0.00 | -0.02 | -0.01 |
| *Paenibacillus* | 0.70 | 0.82 | 1.02 | 0.73 | 0.75 | 0.84 | 0.00 | 0.01 | 0.01 | -0.02 | 0.00 | -0.01 |
| *Planktothrix* | 0.86 | 0.78 | 1.97 | 1.66 | 0.58 | 0.72 | 0.00 | -0.01 | -0.03 | -0.02 | -0.01 | 0.00 |
| *Pseudoxanthomonas* | 0.85 | 0.88 | 0.83 | 0.78 | 0.63 | 0.80 | 0.01 | 0.00 | -0.01 | 0.00 | 0.00 | 0.01 |
| *Psychroflexus* | 0.77 | 0.99 | 0.48 | 2.20 | 0.63 | 1.34 | -0.01 | -0.03 | 0.00 | -0.03 | -0.02 | -0.03 |
| *Rhodovulum* | 0.72 | 0.58 | 1.41 | 0.54 | 1.04 | 0.85 | 0.01 | 0.00 | 0.02 | -0.01 | 0.02 | 0.00 |
| *Rivularia* | 1.19 | 0.81 | 0.76 | 1.03 | 0.62 | 0.69 | 0.01 | 0.01 | 0.01 | 0.02 | 0.00 | 0.00 |
| *Sandaracinus* | 0.70 | 0.80 | 0.90 | 1.22 | 0.73 | 0.62 | 0.00 | 0.00 | -0.02 | 0.01 | 0.00 | 0.01 |
| *Sphingobium* | 0.76 | 1.09 | 0.70 | 1.43 | 1.16 | 0.63 | 0.01 | -0.02 | -0.01 | 0.02 | 0.02 | 0.01 |
| *Spirochaeta* | 1.33 | 0.77 | 0.73 | 0.88 | 1.23 | 0.76 | -0.01 | 0.01 | 0.00 | 0.01 | -0.04 | 0.02 |
| *Starkeya* | 0.90 | 0.98 | 0.74 | 0.76 | 1.08 | 0.67 | 0.00 | 0.00 | 0.00 | 0.00 | 0.02 | -0.02 |
| *Thermoanaerobacter* | 0.92 | 0.86 | 1.52 | 0.78 | 0.66 | 0.33 | -0.01 | 0.03 | 0.02 | 0.01 | -0.02 | 0.00 |
| *Thiobacillus* | 0.81 | 0.79 | 1.31 | 0.51 | 0.78 | 0.92 | 0.01 | 0.01 | -0.02 | 0.00 | -0.01 | 0.00 |
| *Trichodesmium* | 0.74 | 0.75 | 1.00 | 1.20 | 0.85 | 0.78 | 0.00 | -0.01 | -0.01 | -0.01 | 0.02 | -0.01 |
| *Acanthamoeba* | 0.95 | 0.74 | 0.72 | 0.74 | 0.85 | 0.80 | -0.01 | -0.01 | 0.00 | 0.02 | 0.00 | 0.00 |
| *Acidothermus* | 0.62 | 0.88 | 0.78 | 0.69 | 1.27 | 0.71 | 0.00 | 0.02 | -0.01 | 0.00 | 0.03 | 0.02 |

| | | | | | | | | | | | | |
|---|---|---|---|---|---|---|---|---|---|---|---|---|
| *Brevundimonas* | 0.69 | 0.80 | 0.74 | 0.77 | 1.04 | 0.90 | 0.00 | 0.00 | 0.00 | 0.00 | 0.01 | 0.00 |
| *Capronia* | 0.93 | 0.74 | 0.47 | 0.76 | 0.84 | 0.53 | 0.01 | -0.02 | 0.00 | -0.01 | 0.02 | 0.00 |
| *Chamaesiphon* | 0.76 | 1.06 | 0.76 | 0.78 | 1.38 | 0.64 | 0.00 | 0.01 | 0.00 | 0.02 | -0.03 | -0.01 |
| *Deinococcus* | 0.69 | 1.05 | 0.80 | 0.81 | 0.77 | 0.61 | 0.00 | -0.01 | -0.01 | 0.00 | 0.00 | -0.01 |
| *Penicillium* | 0.77 | 0.75 | 1.14 | 0.65 | 0.92 | 0.78 | 0.00 | -0.01 | -0.01 | 0.00 | 0.02 | -0.01 |
| *Propionibacterium* | 0.72 | 0.71 | 0.61 | 1.01 | 1.00 | 0.41 | 0.01 | 0.02 | 0.00 | 0.01 | 0.02 | 0.00 |
| *Salinicoccus* | 0.90 | 0.77 | 1.85 | 0.71 | 0.78 | 0.65 | -0.01 | 0.01 | 0.03 | 0.00 | 0.00 | 0.01 |
| *Thermovirga* | 0.66 | 0.78 | 1.60 | 0.53 | 1.19 | 0.79 | 0.01 | 0.01 | 0.02 | -0.01 | 0.04 | 0.02 |
| *Roseburia* | 0.88 | 0.74 | 0.66 | 0.56 | 0.76 | 0.80 | -0.01 | 0.01 | 0.00 | -0.01 | 0.00 | -0.01 |

Partial Least Squares analysis was performed with 2 latent components. Subscript $_{\text{VIP}\geq 0.8}$ denotes microbial genera that had VIP ≥ 0.8 in 4 or more timepoints. Subscripts $_{(+)}$ and $_{(-)}$ represent microbial genera that had positive or negative regression coefficient in 4 or more timepoints, respectively.

**Trait: Residual Feed Intake (RFI); Predictors: 252 microbial genera (MT)**

| Microbial genera | VIP | | | | | | Regression coefficient | | | | | |
|---|---|---|---|---|---|---|---|---|---|---|---|---|
| | T1 | T2 | T3 | T4 | T5 | T6 | T1 | T2 | T3 | T4 | T5 | T6 |
| Legionella$_{\text{VIP}\geq 0.8\ (+)}$ | 0.71 | 1.40 | 0.78 | 2.10 | 1.70 | 2.59 | 0.01 | -0.03 | 0.01 | 0.03 | 0.04 | 0.05 |
| Oribacterium$_{\text{VIP}\geq 0.8\ (+)}$ | 0.65 | 0.80 | 1.18 | 1.53 | 1.20 | 1.45 | -0.01 | 0.00 | 0.01 | 0.02 | 0.02 | -0.03 |
| Dictyoglomus$_{\text{VIP}\geq 0.8\ (+)}$ | 1.57 | 0.81 | 1.48 | 0.76 | 1.62 | 1.37 | 0.02 | 0.03 | 0.02 | 0.01 | 0.04 | 0.03 |
| Candidatus Protochlamydia$_{\text{V}}$ | 2.46 | 0.75 | 0.82 | 0.91 | 0.70 | 1.83 | 0.04 | 0.03 | 0.00 | 0.01 | 0.00 | 0.04 |
| Methanobacterium$_{\text{VIP}\geq 0.8\ (+)}$ | 1.12 | 0.72 | 0.83 | 1.17 | 1.50 | 0.97 | 0.02 | 0.03 | 0.01 | 0.02 | 0.04 | -0.02 |
| Leptomonas$_{\text{VIP}\geq 0.8\ (+)}$ | 1.05 | 0.93 | 0.74 | 1.06 | 1.23 | 1.36 | 0.02 | 0.03 | 0.01 | 0.02 | 0.03 | 0.03 |
| Neofusicoccum$_{\text{VIP}\geq 0.8\ (+)}$ | 2.50 | 1.22 | 1.09 | 0.65 | 0.50 | 1.19 | 0.04 | 0.04 | -0.01 | 0.01 | 0.00 | 0.02 |
| Saccharomyces$_{\text{VIP}\geq 0.8\ (+)}$ | 0.72 | 0.87 | 0.83 | 0.82 | 0.74 | 0.91 | 0.01 | 0.00 | 0.01 | 0.00 | 0.02 | 0.01 |
| Methanococcus$_{\text{VIP}\geq 0.8\ (+)}$ | 0.58 | 1.06 | 0.83 | 0.81 | 0.94 | 0.59 | 0.00 | -0.01 | 0.01 | 0.01 | 0.03 | 0.00 |
| Moritella$_{\text{VIP}\geq 0.8\ (+)}$ | 1.04 | 0.55 | 0.92 | 1.01 | 0.62 | 1.19 | -0.01 | 0.02 | 0.01 | 0.01 | 0.02 | 0.02 |
| Terribacillus$_{\text{VIP}\geq 0.8\ (+)}$ | 0.15 | 2.19 | 0.86 | 1.86 | 0.17 | 1.61 | 0.00 | 0.06 | 0.01 | 0.02 | 0.00 | 0.03 |
| Rasamsonia$_{\text{VIP}\geq 0.8\ (+)}$ | 0.30 | 0.96 | 0.68 | 0.83 | 0.90 | 1.82 | 0.00 | -0.02 | 0.00 | 0.01 | 0.03 | 0.03 |
| Sphaerulina$_{\text{VIP}\geq 0.8\ (+)}$ | 0.74 | 0.80 | 0.72 | 0.93 | 0.99 | 1.26 | 0.01 | 0.01 | 0.01 | 0.00 | -0.01 | 0.02 |
| Chloroflexus$_{\text{VIP}\geq 0.8\ (+)}$ | 1.39 | 0.95 | 1.56 | 1.43 | 1.51 | 1.83 | 0.02 | 0.01 | 0.02 | 0.01 | 0.03 | 0.04 |
| Blastomyces$_{\text{VIP}\geq 0.8\ (+)}$ | 0.73 | 0.89 | 0.81 | 0.83 | 0.74 | 0.82 | 0.01 | 0.01 | 0.00 | 0.00 | 0.01 | 0.01 |
| Pectobacterium$_{\text{VIP}\geq 0.8\ (+)}$ | 1.17 | 0.08 | 0.97 | 1.57 | 2.08 | 0.83 | 0.02 | 0.00 | 0.01 | 0.02 | 0.05 | -0.02 |

| | | | | | | | | | | | | |
|---|---|---|---|---|---|---|---|---|---|---|---|---|
| Nannizzia$_{VIP\geq0.8\ (+)}$ | 1.01 | 0.91 | 0.87 | 0.60 | 1.35 | 0.46 | 0.01 | 0.03 | 0.01 | 0.01 | -0.02 | -0.01 |
| Mycoplasma$_{VIP\geq0.8\ (+)}$ | 0.73 | 1.06 | 0.81 | 0.71 | 0.84 | 1.19 | 0.00 | -0.01 | 0.00 | 0.01 | 0.00 | 0.02 |
| Blattabacterium$_{VIP\geq0.8\ (+)}$ | 0.92 | 0.96 | 0.85 | 0.88 | 0.58 | 0.58 | 0.01 | -0.01 | 0.01 | 0.00 | 0.01 | 0.00 |
| Gilliamella$_{VIP\geq0.8\ (+)}$ | 0.98 | 1.20 | 0.48 | 1.01 | 0.57 | 2.15 | -0.01 | -0.02 | 0.00 | 0.01 | 0.02 | 0.04 |
| Hoyosella$_{VIP\geq0.8\ (+)}$ | 0.43 | 1.79 | 1.64 | 1.81 | 2.30 | 0.80 | -0.01 | 0.04 | 0.02 | 0.02 | 0.05 | -0.02 |
| Haemophilus$_{VIP\geq0.8\ (+)}$ | 0.94 | 1.20 | 0.47 | 1.20 | 1.54 | 0.48 | -0.01 | -0.02 | 0.00 | 0.02 | 0.04 | 0.00 |
| Thermotoga$_{VIP\geq0.8\ (+)}$ | 0.21 | 0.62 | 1.35 | 2.28 | 1.25 | 0.94 | 0.00 | 0.01 | 0.02 | 0.03 | 0.02 | 0.02 |
| Shinella$_{VIP\geq0.8\ (+)}$ | 0.78 | 0.83 | 1.56 | 1.68 | 1.00 | 1.84 | 0.01 | 0.00 | 0.02 | 0.02 | 0.01 | 0.04 |
| Chromobacterium$_{VIP\geq0.8\ (+)}$ | 1.23 | 1.07 | 0.89 | 0.79 | 0.86 | 0.73 | 0.01 | 0.01 | -0.01 | -0.01 | 0.00 | 0.01 |
| Methanolobus$_{VIP\geq0.8\ (+)}$ | 1.11 | 0.94 | 0.93 | 0.82 | 0.12 | 0.46 | -0.01 | 0.03 | 0.01 | 0.01 | 0.00 | -0.01 |
| Phialocephala$_{VIP\geq0.8\ (+)}$ | 0.40 | 0.82 | 1.13 | 1.03 | 0.78 | 1.09 | 0.00 | 0.00 | -0.01 | -0.01 | 0.00 | 0.01 |
| Thalassospira$_{VIP\geq0.8\ (+)}$ | 0.53 | 2.32 | 1.37 | 1.09 | 0.44 | 1.02 | 0.01 | 0.06 | 0.02 | 0.01 | -0.01 | 0.02 |
| Planococcus$_{VIP\geq0.8\ (+)}$ | 0.86 | 0.27 | 0.95 | 0.92 | 1.08 | 1.13 | -0.01 | 0.01 | 0.01 | 0.01 | 0.02 | 0.02 |
| Desulfobacterium$_{VIP\geq0.8\ (+)}$ | 0.78 | 0.71 | 2.14 | 1.94 | 2.17 | 1.04 | -0.01 | -0.02 | 0.02 | 0.02 | 0.04 | 0.02 |
| Moesziomyces$_{VIP\geq0.8\ (+)}$ | 0.83 | 1.03 | 0.83 | 0.89 | 0.55 | 0.46 | 0.00 | 0.01 | -0.01 | 0.01 | 0.00 | 0.01 |
| Blautia$_{VIP\geq0.8\ (+)}$ | 1.86 | 1.89 | 0.80 | 1.02 | 0.85 | 0.77 | -0.03 | 0.04 | 0.00 | 0.01 | 0.01 | -0.01 |
| Roseburia$_{VIP\geq0.8\ (+)}$ | 1.52 | 2.03 | 0.65 | 0.93 | 0.86 | 0.57 | -0.02 | 0.04 | 0.00 | 0.01 | 0.01 | -0.01 |
| Dickeya$_{VIP\geq0.8\ (+)}$ | 1.07 | 0.99 | 0.93 | 0.82 | 1.00 | 0.80 | 0.01 | 0.00 | -0.01 | 0.00 | 0.01 | 0.01 |
| Brevundimonas$_{VIP\geq0.8\ (+)}$ | 0.92 | 0.84 | 1.04 | 0.79 | 1.65 | 0.71 | -0.01 | 0.00 | 0.00 | -0.01 | 0.02 | 0.01 |
| Bordetella$_{VIP\geq0.8\ (+)}$ | 0.77 | 0.94 | 0.98 | 0.79 | 1.20 | 0.87 | 0.01 | 0.00 | 0.00 | -0.01 | 0.01 | 0.01 |
| Lachnoclostridium$_{VIP\geq0.8\ (+)}$ | 1.41 | 1.41 | 1.06 | 0.87 | 0.56 | 0.93 | -0.02 | 0.02 | 0.01 | 0.01 | 0.00 | -0.02 |
| Geoalkalibacter$_{VIP\geq0.8\ (+)}$ | 1.05 | 0.73 | 0.97 | 0.86 | 1.38 | 0.72 | 0.01 | -0.01 | 0.00 | 0.00 | 0.02 | 0.00 |
| Sedimenticola$_{VIP\geq0.8\ (+)}$ | 1.05 | 1.34 | 1.20 | 0.96 | 0.83 | 1.15 | 0.01 | 0.02 | -0.02 | 0.00 | 0.00 | 0.02 |
| Clostridioides$_{VIP\geq0.8\ (+)}$ | 0.47 | 0.88 | 1.11 | 2.01 | 0.82 | 1.40 | -0.01 | 0.01 | 0.01 | 0.02 | 0.01 | 0.03 |
| Veillonella$_{VIP\geq0.8\ (+)}$ | 0.56 | 0.93 | 0.90 | 0.68 | 1.33 | 1.07 | -0.01 | 0.00 | 0.00 | 0.00 | 0.02 | 0.02 |
| Moorella$_{VIP\geq0.8\ (+)}$ | 0.64 | 1.02 | 2.47 | 1.52 | 0.87 | 0.70 | -0.01 | 0.01 | 0.03 | 0.02 | 0.01 | -0.01 |
| Nitratireductor$_{VIP\geq0.8\ (+)}$ | 0.61 | 0.99 | 1.14 | 1.17 | 1.13 | 0.53 | 0.01 | -0.03 | 0.01 | 0.01 | 0.02 | -0.01 |
| Lactobacillus$_{VIP\geq0.8\ (+)}$ | 0.93 | 0.85 | 0.87 | 1.91 | 0.44 | 0.76 | -0.01 | 0.01 | 0.01 | 0.02 | 0.01 | -0.01 |
| Spiribacter$_{VIP\geq0.8\ (+)}$ | 1.37 | 1.10 | 0.84 | 0.55 | 1.19 | 0.45 | 0.02 | 0.02 | 0.00 | 0.00 | -0.03 | 0.00 |
| Magnetospira$_{VIP\geq0.8\ (+)}$ | 0.77 | 1.02 | 2.02 | 1.20 | 1.79 | 0.41 | -0.01 | 0.01 | 0.02 | 0.01 | 0.03 | 0.00 |

| | | | | | | | | | | | | |
|---|---|---|---|---|---|---|---|---|---|---|---|---|
| Dialister$_{VIP\geq0.8\ (+)}$ | 0.42 | 1.07 | 1.03 | 0.74 | 0.87 | 1.07 | 0.00 | 0.01 | 0.01 | 0.00 | 0.01 | 0.02 |
| Salinispora$_{VIP\geq0.8\ (+)}$ | 0.77 | 0.58 | 1.35 | 1.00 | 0.93 | 0.91 | 0.01 | 0.00 | 0.01 | 0.01 | -0.03 | 0.02 |
| Hydrogenophaga$_{VIP\geq0.8\ (+)}$ | 1.11 | 0.95 | 0.82 | 0.96 | 1.49 | 1.65 | 0.01 | 0.00 | -0.01 | 0.00 | 0.02 | 0.03 |
| Methanofollis$_{VIP\geq0.8\ (+)}$ | 0.88 | 0.34 | 1.00 | 1.34 | 1.45 | 1.18 | -0.01 | 0.01 | 0.01 | 0.02 | -0.03 | 0.02 |
| Proteiniclasticum$_{VIP\geq0.8\ (+)}$ | 0.21 | 1.07 | 1.29 | 1.13 | 0.81 | 0.99 | 0.00 | 0.01 | 0.01 | 0.01 | 0.00 | 0.02 |
| Paraburkholderia$_{VIP\geq0.8\ (+)}$ | 1.59 | 1.01 | 0.73 | 0.80 | 1.58 | 0.87 | 0.02 | 0.01 | 0.00 | 0.00 | 0.02 | 0.01 |
| Exiguobacterium$_{VIP\geq0.8\ (+)}$ | 1.06 | 1.05 | 1.15 | 1.76 | 0.69 | 1.04 | -0.02 | 0.01 | 0.01 | 0.02 | 0.00 | 0.02 |
| Salimicrobium$_{VIP\geq0.8\ (+)}$ | 0.19 | 1.20 | 1.47 | 1.03 | 0.96 | 0.60 | 0.00 | 0.02 | 0.01 | 0.01 | 0.01 | 0.00 |
| Methanocella$_{VIP\geq0.8\ (-)}$ | 1.32 | 0.41 | 0.91 | 1.04 | 1.64 | 1.71 | -0.02 | -0.01 | -0.01 | -0.01 | -0.04 | -0.03 |
| Dermacoccus$_{VIP\geq0.8\ (-)}$ | 1.29 | 0.60 | 0.44 | 1.58 | 0.81 | 2.07 | -0.02 | -0.02 | 0.00 | -0.02 | -0.02 | -0.04 |
| Bifidobacterium$_{VIP\geq0.8\ (-)}$ | 1.38 | 0.96 | 0.23 | 0.42 | 1.40 | 1.85 | -0.02 | -0.03 | 0.00 | -0.01 | -0.03 | -0.04 |
| Methylocella$_{VIP\geq0.8\ (-)}$ | 1.34 | 1.91 | 0.83 | 0.42 | 0.54 | 1.05 | -0.02 | -0.06 | 0.01 | -0.01 | -0.01 | -0.02 |
| Acaryochloris$_{VIP\geq0.8\ (-)}$ | 1.00 | 0.72 | 1.26 | 0.80 | 0.96 | 0.69 | 0.01 | -0.01 | -0.02 | -0.01 | -0.03 | -0.01 |
| Vibrio$_{VIP\geq0.8\ (-)}$ | 1.11 | 1.37 | 1.21 | 0.84 | 0.71 | 0.44 | -0.02 | -0.04 | -0.02 | 0.01 | -0.02 | -0.01 |
| Mitsuokella$_{VIP\geq0.8\ (-)}$ | 1.49 | 1.14 | 0.91 | 0.97 | 0.65 | 0.83 | -0.02 | 0.01 | 0.00 | -0.01 | -0.01 | -0.01 |
| Jonquetella$_{VIP\geq0.8\ (-)}$ | 0.92 | 0.85 | 0.80 | 0.89 | 0.80 | 1.08 | 0.00 | -0.01 | -0.01 | -0.01 | 0.00 | -0.02 |
| Rhodomicrobium$_{VIP\geq0.8\ (-)}$ | 1.04 | 0.74 | 1.18 | 0.87 | 0.72 | 0.88 | 0.01 | -0.02 | -0.02 | -0.01 | 0.00 | -0.01 |
| Lachnobacterium$_{VIP\geq0.8\ (-)}$ | 1.55 | 1.80 | 0.45 | 0.55 | 1.58 | 1.87 | -0.02 | 0.05 | 0.00 | -0.01 | -0.04 | -0.04 |
| Thermaerobacter$_{VIP\geq0.8\ (-)}$ | 1.01 | 1.08 | 0.87 | 0.84 | 0.73 | 0.94 | 0.00 | 0.01 | -0.01 | -0.01 | -0.02 | -0.01 |
| Rhodanobacter$_{VIP\geq0.8\ (-)}$ | 0.89 | 0.80 | 1.00 | 0.75 | 0.94 | 0.62 | 0.00 | 0.00 | -0.01 | -0.01 | -0.03 | 0.01 |
| Starkeya$_{VIP\geq0.8\ (-)}$ | 1.08 | 0.72 | 0.83 | 0.79 | 0.95 | 0.99 | -0.01 | -0.02 | 0.00 | -0.01 | 0.01 | -0.02 |
| Sanguibacter$_{VIP\geq0.8\ (-)}$ | 0.96 | 1.04 | 0.95 | 0.92 | 0.80 | 0.74 | 0.00 | 0.00 | -0.01 | -0.01 | 0.00 | 0.00 |
| Aeromonas$_{VIP\geq0.8\ (-)}$ | 0.92 | 0.92 | 1.01 | 0.77 | 0.89 | 0.62 | 0.00 | 0.00 | -0.01 | 0.00 | 0.00 | 0.00 |
| Defluviimonas$_{VIP\geq0.8\ (-)}$ | 0.98 | 0.92 | 1.07 | 0.90 | 0.82 | 0.60 | 0.00 | 0.00 | -0.01 | -0.01 | 0.00 | 0.00 |
| Thioploca$_{VIP\geq0.8\ (-)}$ | 1.01 | 0.86 | 0.97 | 0.81 | 0.75 | 0.63 | 0.00 | 0.00 | -0.01 | -0.01 | 0.00 | 0.00 |
| Sandaracinus$_{VIP\geq0.8\ (-)}$ | 1.04 | 0.69 | 1.51 | 0.97 | 0.57 | 0.88 | 0.01 | -0.01 | -0.02 | -0.01 | -0.01 | 0.02 |
| Methanomassiliicoccus$_{VIP\geq0.8}$ | 0.83 | 0.86 | 0.93 | 0.86 | 0.70 | 0.57 | 0.00 | 0.00 | -0.01 | -0.01 | 0.00 | 0.00 |
| Cyanobium$_{VIP\geq0.8\ (-)}$ | 0.92 | 0.99 | 0.96 | 0.86 | 0.71 | 0.71 | 0.00 | 0.01 | -0.01 | -0.01 | 0.00 | 0.00 |
| Geobacter$_{VIP\geq0.8\ (-)}$ | 0.97 | 1.07 | 0.91 | 0.80 | 0.70 | 0.72 | 0.00 | 0.01 | -0.01 | -0.01 | -0.01 | 0.00 |
| Heliobacterium$_{VIP\geq0.8\ (-)}$ | 1.00 | 1.15 | 1.09 | 0.69 | 1.38 | 0.78 | -0.01 | 0.01 | 0.01 | 0.00 | -0.04 | -0.01 |

| | | | | | | | | | | | | |
|---|---|---|---|---|---|---|---|---|---|---|---|---|
| Sphingopyxis$_{VIP\geq0.8\ (-)}$ | 0.86 | 0.84 | 0.85 | 0.79 | 0.85 | 0.69 | 0.00 | 0.00 | -0.01 | -0.01 | 0.00 | 0.00 |
| Rhodobacter$_{VIP\geq0.8\ (-)}$ | 1.25 | 0.84 | 0.93 | 0.92 | 0.68 | 0.65 | 0.01 | 0.00 | -0.01 | -0.01 | -0.01 | 0.01 |
| Dyella$_{VIP\geq0.8\ (-)}$ | 0.97 | 0.82 | 0.87 | 0.79 | 0.97 | 0.90 | 0.01 | -0.01 | -0.01 | -0.01 | 0.00 | -0.01 |
| Pantoea$_{VIP\geq0.8\ (-)}$ | 0.88 | 0.90 | 0.89 | 0.84 | 0.77 | 0.62 | -0.01 | 0.00 | -0.01 | 0.00 | 0.00 | 0.00 |
| Pluralibacter$_{VIP\geq0.8\ (-)}$ | 1.06 | 0.92 | 0.83 | 0.77 | 0.67 | 1.27 | 0.01 | 0.00 | 0.00 | 0.00 | -0.01 | -0.02 |
| Mesorhizobium$_{VIP\geq0.8\ (-)}$ | 0.99 | 0.99 | 0.94 | 0.79 | 0.94 | 0.70 | 0.00 | 0.00 | -0.01 | -0.01 | 0.00 | 0.00 |
| Spirochaeta$_{VIP\geq0.8\ (-)}$ | 0.88 | 1.51 | 1.20 | 0.80 | 0.76 | 1.88 | -0.01 | 0.02 | -0.02 | -0.01 | -0.02 | 0.04 |
| Edwardsiella$_{VIP\geq0.8\ (-)}$ | 0.86 | 0.81 | 0.83 | 0.78 | 0.89 | 0.65 | 0.00 | -0.01 | 0.00 | 0.00 | 0.00 | 0.00 |
| Janibacter$_{VIP\geq0.8\ (-)}$ | 1.06 | 1.02 | 0.94 | 0.89 | 0.70 | 0.74 | -0.02 | -0.03 | -0.01 | 0.01 | 0.00 | -0.01 |
| Actinoplanes$_{VIP\geq0.8\ (-)}$ | 1.14 | 0.80 | 0.84 | 0.77 | 0.80 | 1.01 | 0.01 | -0.01 | -0.01 | -0.01 | 0.00 | -0.02 |
| Azospira$_{VIP\geq0.8\ (-)}$ | 1.05 | 0.84 | 0.94 | 0.76 | 0.88 | 0.74 | 0.01 | 0.00 | -0.01 | 0.00 | 0.00 | -0.01 |
| Desulfovibrio$_{VIP\geq0.8\ (-)}$ | 0.90 | 0.81 | 0.84 | 0.78 | 0.89 | 0.66 | 0.00 | -0.01 | 0.00 | -0.01 | 0.00 | -0.01 |
| Asticcacaulis$_{VIP\geq0.8\ (-)}$ | 1.32 | 1.00 | 1.24 | 0.82 | 0.75 | 0.49 | 0.01 | 0.01 | -0.02 | -0.01 | 0.00 | 0.00 |
| Syntrophus$_{VIP\geq0.8\ (-)}$ | 1.03 | 0.75 | 0.38 | 1.45 | 0.84 | 1.00 | -0.02 | -0.02 | -0.01 | -0.02 | 0.01 | 0.02 |
| Altererythrobacter$_{VIP\geq0.8\ (-)}$ | 0.90 | 0.81 | 0.91 | 0.81 | 0.94 | 0.70 | 0.00 | 0.00 | -0.01 | 0.00 | 0.00 | 0.00 |
| Cupriavidus$_{VIP\geq0.8\ (-)}$ | 0.92 | 1.01 | 0.88 | 0.80 | 1.31 | 0.75 | 0.00 | 0.00 | -0.01 | -0.01 | 0.01 | 0.00 |
| Thioalkalivibrio$_{VIP\geq0.8\ (-)}$ | 1.10 | 0.90 | 0.87 | 0.78 | 0.93 | 0.64 | 0.01 | 0.00 | -0.01 | 0.00 | 0.00 | 0.00 |
| Hyphomonas$_{VIP\geq0.8\ (-)}$ | 1.07 | 0.89 | 0.91 | 0.80 | 0.72 | 0.69 | 0.01 | 0.00 | -0.01 | -0.01 | 0.00 | 0.01 |
| Methanoregula$_{VIP\geq0.8\ (-)}$ | 1.16 | 1.19 | 0.86 | 0.78 | 0.72 | 0.87 | 0.01 | 0.01 | 0.00 | -0.01 | -0.02 | -0.01 |
| Gordonia$_{VIP\geq0.8\ (-)}$ | 1.08 | 0.89 | 0.87 | 0.78 | 1.08 | 0.67 | 0.01 | 0.00 | -0.01 | -0.01 | 0.01 | -0.01 |
| Agrobacterium$_{VIP\geq0.8\ (-)}$ | 1.26 | 0.82 | 0.88 | 0.80 | 0.74 | 0.73 | 0.01 | -0.01 | 0.00 | 0.00 | -0.01 | 0.00 |
| Ammonifex$_{VIP\geq0.8\ (-)}$ | 1.32 | 1.20 | 1.51 | 1.31 | 0.50 | 0.39 | -0.02 | -0.03 | 0.02 | -0.02 | -0.01 | -0.01 |
| Nakaseomyces$_{VIP\geq0.8\ (-)}$ | 1.03 | 0.95 | 1.04 | 0.76 | 1.08 | 0.74 | 0.01 | 0.00 | 0.00 | 0.01 | -0.01 | -0.01 |
| Halorhodospira$_{VIP\geq0.8\ (-)}$ | 1.15 | 0.94 | 0.87 | 0.76 | 0.86 | 0.62 | 0.01 | 0.00 | -0.01 | 0.00 | 0.00 | 0.00 |
| Thioflavicoccus$_{VIP\geq0.8\ (-)}$ | 1.21 | 0.80 | 0.85 | 0.76 | 1.58 | 1.26 | 0.01 | -0.01 | -0.01 | -0.01 | 0.02 | -0.02 |
| Dermabacter$_{VIP\geq0.8\ (-)}$ | 1.44 | 1.04 | 0.94 | 0.78 | 0.80 | 0.63 | 0.02 | 0.01 | -0.01 | -0.01 | 0.00 | -0.01 |
| Tsukamurella$_{VIP\geq0.8\ (-)}$ | 1.19 | 0.87 | 0.93 | 0.76 | 1.09 | 0.54 | 0.01 | 0.00 | -0.01 | 0.00 | 0.01 | 0.00 |
| Nitrosospira$_{VIP\geq0.8\ (-)}$ | 1.05 | 0.85 | 0.84 | 0.78 | 1.15 | 0.57 | 0.01 | 0.00 | 0.00 | 0.00 | 0.01 | 0.00 |
| Lacinutrix$_{VIP\geq0.8\ (-)}$ | 0.77 | 1.73 | 1.21 | 1.15 | 0.70 | 1.13 | -0.01 | -0.04 | -0.01 | -0.01 | 0.02 | -0.02 |
| Tetrapisispora$_{VIP\geq0.8\ (-)}$ | 0.59 | 0.84 | 0.84 | 0.97 | 0.97 | 0.70 | 0.01 | 0.00 | 0.00 | 0.00 | 0.00 | -0.01 |

| | | | | | | | | | | | | |
|---|---|---|---|---|---|---|---|---|---|---|---|---|
| Zygosaccharomyces$_{VIP≥0.8\ (-)}$ | 0.60 | 0.97 | 1.02 | 0.82 | 1.06 | 0.65 | 0.00 | 0.00 | 0.00 | 0.00 | -0.01 | 0.00 |
| Marivirga$_{VIP≥0.8\ (-)}$ | 1.02 | 0.61 | 0.77 | 2.40 | 0.90 | 1.92 | -0.01 | 0.02 | -0.01 | -0.03 | -0.01 | -0.04 |
| Theileria$_{VIP≥0.8\ (-)}$ | 0.91 | 0.77 | 1.15 | 0.91 | 0.94 | 0.79 | 0.01 | 0.01 | -0.01 | 0.00 | -0.01 | -0.01 |
| Blastocystis$_{VIP≥0.8\ (-)}$ | 0.61 | 0.93 | 0.88 | 1.06 | 0.68 | 0.97 | 0.00 | 0.00 | 0.00 | -0.01 | 0.01 | -0.02 |
| Ichthyophthirius$_{VIP≥0.8\ (-)}$ | 0.54 | 0.93 | 0.99 | 1.16 | 0.91 | 0.66 | 0.00 | 0.00 | 0.00 | -0.01 | 0.00 | 0.00 |
| Colletotrichum$_{VIP≥0.8\ (-)}$ | 1.02 | 1.07 | 0.77 | 0.82 | 0.85 | 1.08 | 0.01 | -0.01 | -0.01 | 0.00 | 0.02 | -0.02 |
| Planktothrix$_{VIP≥0.8\ (-)}$ | 0.90 | 0.96 | 1.04 | 0.83 | 0.92 | 1.17 | 0.01 | 0.00 | -0.01 | 0.00 | -0.01 | 0.02 |
| Tetrahymena$_{VIP≥0.8\ (-)}$ | 0.59 | 0.99 | 1.06 | 1.18 | 0.92 | 0.66 | 0.00 | 0.00 | -0.01 | -0.01 | 0.00 | 0.00 |
| Scheffersomyces$_{VIP≥0.8\ (-)}$ | 0.82 | 1.42 | 0.98 | 0.80 | 0.85 | 0.60 | 0.01 | -0.02 | 0.00 | 0.00 | 0.00 | -0.01 |
| Fibrobacter$_{VIP≥0.8\ (-)}$ | 1.21 | 0.98 | 0.63 | 1.08 | 1.98 | 1.12 | -0.02 | -0.01 | 0.00 | -0.01 | -0.03 | -0.02 |
| Psychroflexus$_{VIP≥0.8\ (-)}$ | 0.65 | 1.17 | 1.04 | 1.84 | 0.60 | 1.02 | 0.00 | -0.01 | -0.01 | -0.02 | -0.01 | -0.02 |
| Phaeoacremonium$_{VIP≥0.8\ (-)}$ | 1.30 | 0.82 | 0.88 | 0.80 | 0.67 | 1.03 | -0.02 | 0.01 | 0.00 | 0.00 | 0.01 | -0.02 |
| Thermothelomyces$_{VIP≥0.8\ (-)}$ | 1.12 | 1.36 | 0.69 | 0.72 | 0.97 | 1.13 | -0.02 | -0.03 | 0.00 | 0.00 | 0.03 | -0.02 |
| Trichomonas$_{VIP≥0.8\ (-)}$ | 0.93 | 0.85 | 0.88 | 1.06 | 1.39 | 0.53 | -0.01 | 0.00 | 0.00 | -0.01 | -0.02 | 0.01 |
| Nosema$_{VIP≥0.8\ (-)}$ | 0.60 | 0.85 | 1.21 | 1.24 | 1.06 | 0.67 | 0.00 | 0.01 | -0.01 | -0.01 | -0.01 | -0.01 |
| Paramecium$_{VIP≥0.8\ (-)}$ | 0.45 | 0.94 | 1.09 | 1.39 | 1.29 | 0.56 | 0.00 | 0.00 | -0.01 | -0.01 | -0.01 | 0.00 |
| Leptosphaeria$_{VIP≥0.8\ (-)}$ | 0.64 | 0.89 | 1.16 | 0.98 | 1.49 | 0.90 | 0.00 | 0.00 | -0.01 | -0.01 | -0.02 | 0.01 |
| Spizellomyces$_{VIP≥0.8\ (-)}$ | 0.53 | 0.84 | 0.96 | 0.93 | 1.58 | 0.78 | 0.00 | 0.01 | 0.00 | 0.00 | -0.02 | -0.01 |
| Gregarina$_{VIP≥0.8\ (-)}$ | 0.82 | 1.33 | 0.95 | 1.02 | 0.55 | 0.27 | -0.01 | -0.02 | 0.00 | -0.01 | 0.01 | 0.00 |
| Podospora$_{VIP≥0.8\ (-)}$ | 1.38 | 0.87 | 0.96 | 1.09 | 1.72 | 1.14 | -0.02 | -0.01 | 0.00 | -0.01 | -0.03 | -0.02 |
| Trichormus$_{VIP≥0.8\ (-)}$ | 0.63 | 1.22 | 1.18 | 1.06 | 0.61 | 1.08 | -0.01 | -0.01 | -0.01 | -0.01 | 0.01 | -0.02 |
| Candidatus Paracaedibacter | 1.09 | 1.75 | 0.35 | 0.82 | 0.90 | 0.92 | 0.02 | -0.04 | 0.00 | -0.01 | 0.02 | -0.02 |
| Chamaesiphon$_{VIP≥0.8\ (-)}$ | 0.60 | 0.70 | 1.09 | 1.00 | 1.86 | 1.27 | 0.00 | 0.02 | -0.01 | -0.01 | -0.03 | -0.03 |
| Bernardetia$_{VIP≥0.8\ (-)}$ | 0.94 | 0.90 | 1.00 | 1.22 | 0.55 | 0.26 | -0.01 | -0.01 | -0.01 | -0.01 | 0.02 | 0.01 |
| Croceibacter$_{VIP≥0.8\ (-)}$ | 0.80 | 0.72 | 0.69 | 1.08 | 1.19 | 1.47 | 0.00 | 0.00 | 0.00 | -0.01 | -0.02 | -0.03 |
| Agaricus$_{VIP≥0.8}$ | 1.10 | 0.84 | 0.89 | 0.89 | 0.98 | 1.04 | 0.02 | 0.03 | -0.01 | 0.00 | -0.01 | 0.02 |
| Alkalilimnicola$_{VIP≥0.8}$ | 1.14 | 0.97 | 0.92 | 0.80 | 0.97 | 1.02 | 0.01 | 0.00 | -0.01 | -0.01 | 0.00 | -0.01 |
| Chthonomonas$_{VIP≥0.8}$ | 1.41 | 1.02 | 0.92 | 0.84 | 0.85 | 0.84 | 0.02 | 0.01 | -0.01 | -0.01 | 0.00 | -0.01 |
| Porphyrobacter$_{VIP≥0.8}$ | 0.93 | 0.94 | 0.87 | 0.88 | 0.90 | 0.89 | 0.00 | 0.00 | 0.00 | -0.01 | 0.00 | -0.01 |
| Rubrobacter$_{VIP≥0.8}$ | 1.07 | 1.17 | 0.90 | 0.90 | 1.17 | 1.01 | 0.01 | 0.01 | -0.01 | -0.01 | 0.01 | -0.02 |

| | | | | | | | | | | | | |
|---|---|---|---|---|---|---|---|---|---|---|---|---|
| $Stereum_{VIP\geq0.8}$ | 1.24 | 1.05 | 1.31 | 0.80 | 1.71 | 1.17 | 0.02 | 0.04 | -0.01 | -0.01 | -0.03 | 0.02 |
| $Azospirillum_{VIP\geq0.8}$ | 0.95 | 0.95 | 0.90 | 0.93 | 0.74 | 0.91 | 0.00 | 0.00 | -0.01 | -0.01 | -0.01 | 0.02 |
| $Babjeviella_{VIP\geq0.8}$ | 2.07 | 1.81 | 0.69 | 0.95 | 1.18 | 2.42 | 0.03 | 0.05 | -0.01 | -0.01 | -0.01 | 0.05 |
| $Bradyrhizobium_{VIP\geq0.8}$ | 0.91 | 0.79 | 0.84 | 0.86 | 0.88 | 0.80 | 0.01 | -0.01 | 0.00 | -0.01 | 0.00 | 0.01 |
| $Jeotgalibacillus_{VIP\geq0.8}$ | 1.41 | 1.33 | 0.91 | 1.98 | 0.58 | 1.30 | -0.02 | -0.04 | 0.01 | 0.03 | 0.01 | -0.03 |
| $Klebsiella_{VIP\geq0.8}$ | 1.22 | 1.02 | 0.81 | 1.05 | 0.61 | 1.17 | -0.02 | 0.00 | 0.00 | 0.01 | -0.01 | 0.02 |
| $Methanoculleus_{VIP\geq0.8}$ | 0.86 | 0.62 | 0.98 | 1.49 | 0.94 | 1.59 | -0.01 | -0.02 | 0.01 | -0.02 | 0.01 | 0.03 |
| $Methyloceanibacter_{VIP\geq0.8}$ | 1.68 | 1.06 | 1.02 | 0.97 | 1.38 | 0.76 | 0.02 | 0.01 | -0.01 | -0.01 | 0.02 | -0.01 |
| $Puccinia_{VIP\geq0.8}$ | 0.99 | 0.80 | 0.85 | 0.94 | 0.91 | 0.97 | 0.01 | 0.02 | 0.00 | -0.01 | 0.00 | 0.01 |
| $Thiobacimonas_{VIP\geq0.8}$ | 1.04 | 1.19 | 0.88 | 0.71 | 1.13 | 0.94 | 0.01 | 0.01 | -0.01 | 0.00 | 0.01 | -0.02 |
| $Wallemia_{VIP\geq0.8}$ | 0.78 | 0.88 | 0.90 | 0.84 | 1.18 | 0.93 | 0.01 | 0.00 | 0.00 | 0.00 | -0.01 | 0.01 |
| $Anaeromyxobacter_{VIP\geq0.8}$ | 1.56 | 1.01 | 1.31 | 1.11 | 0.73 | 0.50 | 0.02 | 0.00 | -0.02 | -0.01 | -0.02 | 0.00 |
| $Aureimonas_{VIP\geq0.8}$ | 0.08 | 0.63 | 0.81 | 1.33 | 1.29 | 1.01 | 0.00 | -0.01 | 0.01 | 0.02 | -0.03 | 0.02 |
| $Azoarcus_{VIP\geq0.8}$ | 1.02 | 1.05 | 1.30 | 0.86 | 0.71 | 0.70 | 0.01 | 0.01 | -0.02 | -0.01 | -0.01 | 0.01 |
| $Blastochloris_{VIP\geq0.8}$ | 1.31 | 1.19 | 0.82 | 0.77 | 0.81 | 0.60 | 0.02 | 0.01 | 0.00 | 0.00 | 0.00 | 0.00 |
| $Candidatus\ Koribacter_{VIP\geq0.8}$ | 0.98 | 1.10 | 1.50 | 0.78 | 1.25 | 0.78 | 0.01 | 0.01 | -0.02 | -0.01 | 0.01 | 0.00 |
| $Carboxydothermus_{VIP\geq0.8}$ | 0.75 | 0.72 | 0.88 | 0.95 | 1.11 | 1.05 | -0.01 | -0.01 | 0.00 | 0.01 | 0.02 | -0.02 |
| $Desulfitobacterium_{VIP\geq0.8}$ | 0.44 | 0.74 | 0.91 | 1.59 | 0.81 | 1.10 | 0.00 | -0.02 | 0.01 | 0.02 | 0.01 | -0.02 |
| $Dictyostelium_{VIP\geq0.8}$ | 0.55 | 0.92 | 1.02 | 1.09 | 0.90 | 0.70 | 0.00 | 0.00 | -0.01 | -0.01 | 0.00 | 0.00 |
| $Entamoeba_{VIP\geq0.8}$ | 0.59 | 0.83 | 0.95 | 0.89 | 1.01 | 0.71 | 0.01 | 0.01 | 0.00 | 0.00 | -0.01 | 0.00 |
| $Eutypa_{VIP\geq0.8}$ | 0.83 | 0.80 | 1.09 | 1.19 | 0.85 | 0.66 | 0.01 | 0.01 | -0.01 | -0.01 | 0.00 | 0.00 |
| $Haloquadratum_{VIP\geq0.8}$ | 1.05 | 0.53 | 0.86 | 0.98 | 0.80 | 1.00 | 0.01 | 0.00 | 0.00 | -0.01 | -0.01 | 0.02 |
| $Methanosphaera_{VIP\geq0.8}$ | 0.48 | 0.57 | 1.54 | 1.29 | 1.31 | 0.80 | -0.01 | -0.01 | 0.02 | 0.02 | 0.03 | -0.02 |
| $Naegleria_{VIP\geq0.8}$ | 0.56 | 0.86 | 1.14 | 1.30 | 0.81 | 0.59 | 0.00 | 0.01 | -0.01 | -0.01 | 0.00 | 0.00 |
| $Pannonibacter_{VIP\geq0.8}$ | 1.04 | 0.92 | 0.92 | 0.78 | 0.70 | 0.86 | 0.01 | 0.00 | -0.01 | 0.00 | -0.01 | 0.01 |
| $Pelagibaca_{VIP\geq0.8}$ | 1.42 | 1.12 | 1.14 | 0.64 | 0.43 | 0.94 | 0.02 | -0.04 | 0.01 | 0.00 | -0.01 | -0.02 |
| $Ralstonia_{VIP\geq0.8}$ | 1.05 | 1.03 | 1.06 | 0.79 | 0.93 | 0.72 | 0.00 | 0.00 | -0.01 | -0.01 | 0.00 | 0.00 |
| $Rathayibacter_{VIP\geq0.8}$ | 1.05 | 0.81 | 0.75 | 0.93 | 0.89 | 0.44 | -0.02 | -0.03 | 0.00 | 0.01 | -0.02 | 0.00 |
| $Sideroxydans_{VIP\geq0.8}$ | 0.99 | 0.96 | 0.88 | 0.77 | 0.92 | 0.78 | 0.00 | 0.00 | -0.01 | 0.00 | 0.00 | -0.01 |
| $Sinorhizobium_{VIP\geq0.8}$ | 0.81 | 1.11 | 0.83 | 0.81 | 0.68 | 0.55 | 0.00 | 0.01 | 0.00 | 0.00 | -0.01 | 0.01 |

| | | | | | | | | | | | | |
|---|---|---|---|---|---|---|---|---|---|---|---|---|
| Sphingobium$_{VIP\geq0.8}$ | 0.81 | 0.77 | 0.85 | 0.81 | 1.89 | 0.78 | 0.00 | -0.02 | 0.00 | 0.00 | 0.03 | 0.00 |
| Syntrophomonas$_{VIP\geq0.8}$ | 1.48 | 0.95 | 0.60 | 0.98 | 0.44 | 1.03 | 0.02 | -0.01 | 0.01 | 0.01 | -0.01 | -0.02 |
| Thauera$_{VIP\geq0.8}$ | 1.16 | 0.95 | 1.04 | 0.79 | 0.66 | 1.04 | 0.01 | 0.00 | -0.01 | -0.01 | -0.01 | 0.02 |
| Variovorax$_{VIP\geq0.8}$ | 0.92 | 1.01 | 0.80 | 0.84 | 0.70 | 0.73 | 0.00 | 0.00 | -0.01 | -0.01 | -0.01 | 0.01 |
| *Eimeria* | 1.40 | 0.82 | 0.73 | 0.72 | 0.83 | 0.79 | 0.02 | 0.03 | 0.01 | 0.00 | 0.02 | 0.01 |
| *Millerozyma* | 0.65 | 0.92 | 0.42 | 1.04 | 0.77 | 1.34 | 0.00 | 0.03 | 0.00 | 0.01 | 0.02 | 0.02 |
| *Trichoderma* | 0.86 | 0.84 | 0.75 | 0.75 | 0.82 | 0.72 | 0.01 | 0.01 | 0.00 | 0.00 | 0.02 | 0.00 |
| *Enterococcus* | 2.04 | 0.78 | 1.47 | 1.16 | 0.27 | 0.18 | -0.03 | 0.00 | 0.02 | 0.02 | 0.01 | 0.00 |
| *Ethanoligenens* | 0.52 | 1.70 | 0.92 | 0.77 | 1.28 | 0.67 | 0.00 | 0.04 | 0.00 | 0.00 | -0.03 | 0.01 |
| *Methylacidiphilum* | 0.97 | 0.91 | 0.74 | 0.70 | 0.91 | 0.60 | 0.01 | 0.00 | 0.00 | 0.00 | 0.03 | 0.01 |
| *Paucibacter* | 0.42 | 0.86 | 1.23 | 0.72 | 0.98 | 0.39 | 0.00 | -0.03 | 0.01 | 0.01 | 0.02 | 0.00 |
| *Pelobacter* | 0.50 | 0.40 | 1.04 | 0.71 | 1.15 | 1.15 | 0.00 | 0.00 | 0.01 | 0.01 | 0.02 | 0.02 |
| *Pelosinus* | 0.29 | 0.75 | 1.57 | 0.75 | 1.22 | 1.11 | 0.00 | -0.01 | 0.02 | 0.00 | 0.02 | 0.02 |
| *Rhodovulum* | 0.90 | 0.63 | 0.79 | 0.85 | 1.17 | 0.67 | 0.01 | 0.00 | 0.00 | 0.00 | 0.01 | -0.01 |
| *Scedosporium* | 0.59 | 0.92 | 0.83 | 0.77 | 0.66 | 0.96 | 0.00 | 0.00 | 0.00 | 0.00 | 0.00 | -0.02 |
| *Trichophyton* | 0.61 | 0.86 | 0.96 | 0.80 | 0.77 | 0.74 | 0.00 | 0.01 | 0.00 | 0.00 | 0.00 | 0.00 |
| *Allisonella* | 0.63 | 1.69 | 0.94 | 0.82 | 0.76 | 0.71 | -0.01 | 0.04 | 0.01 | 0.00 | 0.00 | 0.01 |
| *Corynebacterium* | 0.78 | 1.23 | 1.20 | 0.99 | 0.71 | 0.74 | 0.00 | 0.02 | 0.01 | 0.00 | -0.01 | 0.00 |
| *Dorea* | 1.89 | 2.33 | 0.65 | 0.80 | 1.09 | 0.66 | -0.03 | 0.05 | 0.00 | 0.00 | 0.02 | -0.01 |
| *Faecalibaculum* | 0.88 | 0.75 | 1.22 | 0.74 | 0.88 | 0.80 | -0.01 | 0.00 | 0.01 | 0.01 | 0.01 | 0.02 |
| *Komagataella* | 1.01 | 0.72 | 0.71 | 0.79 | 1.36 | 0.92 | 0.01 | 0.02 | 0.01 | 0.00 | -0.02 | 0.01 |
| *Methanothermus* | 0.84 | 0.80 | 0.95 | 0.73 | 0.56 | 0.86 | -0.01 | 0.00 | 0.01 | 0.01 | 0.01 | -0.02 |
| *Niastella* | 0.76 | 0.49 | 1.34 | 0.78 | 1.28 | 0.90 | -0.01 | -0.01 | 0.02 | 0.01 | 0.03 | 0.02 |
| *Xenorhabdus* | 0.63 | 0.74 | 0.93 | 1.47 | 0.65 | 1.31 | 0.00 | -0.01 | 0.01 | 0.02 | 0.02 | 0.03 |
| *Alicycliphilus* | 0.44 | 0.69 | 0.78 | 1.05 | 1.61 | 1.78 | -0.01 | 0.00 | -0.01 | -0.01 | 0.03 | 0.04 |
| *Aromatoleum* | 1.21 | 0.94 | 0.80 | 0.78 | 0.92 | 0.68 | 0.01 | 0.00 | 0.00 | 0.00 | 0.00 | -0.01 |
| *Bipolaris* | 0.60 | 0.92 | 0.79 | 1.06 | 1.16 | 0.63 | 0.00 | -0.01 | 0.00 | 0.02 | -0.01 | 0.01 |
| *Burkholderia* | 1.01 | 1.00 | 0.92 | 0.79 | 0.69 | 0.62 | 0.01 | 0.00 | -0.01 | 0.00 | -0.01 | 0.00 |
| *Candida* | 0.59 | 0.88 | 0.95 | 0.91 | 0.74 | 0.67 | 0.00 | 0.00 | 0.00 | 0.00 | 0.01 | 0.00 |
| *Candidatus Ishikawaella* | 1.00 | 0.99 | 0.79 | 0.72 | 0.83 | 0.77 | 0.01 | 0.01 | 0.00 | 0.00 | 0.00 | -0.01 |
| *Capronia* | 1.00 | 1.13 | 0.58 | 1.49 | 0.79 | 0.78 | 0.01 | -0.02 | 0.01 | 0.02 | 0.00 | -0.02 |
| *Cellulomonas* | 0.73 | 0.47 | 0.97 | 0.66 | 0.82 | 0.98 | 0.01 | -0.01 | 0.01 | -0.01 | 0.00 | -0.02 |
| *Cladophialophora* | 1.11 | 0.67 | 0.80 | 0.88 | 0.76 | 1.56 | 0.02 | 0.01 | -0.01 | -0.01 | -0.01 | 0.03 |
| *Cordyceps* | 0.54 | 0.88 | 0.98 | 0.77 | 0.84 | 0.52 | 0.00 | 0.00 | 0.00 | 0.00 | 0.00 | 0.00 |

| | | | | | | | | | | | | |
|---|---|---|---|---|---|---|---|---|---|---|---|---|
| *Dactylellina* | 0.59 | 0.86 | 1.09 | 0.92 | 0.80 | 0.65 | 0.00 | 0.01 | -0.01 | 0.00 | 0.00 | 0.00 |
| *Desulfococcus* | 0.63 | 1.18 | 0.79 | 0.75 | 1.20 | 1.01 | 0.00 | 0.01 | 0.00 | -0.01 | 0.01 | 0.02 |
| *Desulfurispirillum* | 1.00 | 1.13 | 1.05 | 0.78 | 0.67 | 0.55 | 0.01 | 0.01 | -0.01 | 0.00 | -0.01 | 0.00 |
| *Microsporum* | 0.64 | 0.84 | 0.88 | 0.80 | 1.08 | 0.66 | 0.00 | 0.00 | 0.00 | 0.00 | -0.01 | 0.00 |
| *Olsenella* | 0.66 | 0.76 | 1.28 | 0.69 | 1.09 | 0.92 | -0.01 | 0.00 | 0.01 | 0.00 | 0.01 | -0.02 |
| *Paracoccidioides* | 0.53 | 0.93 | 1.07 | 0.96 | 0.78 | 0.67 | 0.00 | 0.00 | -0.01 | 0.00 | 0.00 | 0.00 |
| *Spathaspora* | 0.56 | 0.76 | 1.37 | 1.03 | 1.32 | 0.72 | 0.00 | 0.02 | -0.01 | -0.01 | -0.01 | 0.00 |
| *Alcanivorax* | 1.15 | 0.80 | 0.79 | 0.76 | 0.98 | 0.72 | -0.01 | -0.01 | -0.01 | 0.00 | 0.01 | 0.01 |
| *Archangium* | 0.84 | 0.96 | 0.80 | 0.74 | 0.70 | 0.52 | 0.01 | 0.01 | -0.01 | 0.00 | 0.00 | 0.00 |
| *Candidatus Methanomethylophilus* | 1.52 | 0.76 | 0.80 | 1.29 | 0.94 | 0.56 | 0.02 | 0.00 | 0.00 | -0.02 | 0.01 | 0.00 |
| *Gloeobacter* | 0.96 | 0.61 | 0.89 | 0.75 | 0.78 | 1.00 | 0.01 | -0.01 | -0.01 | 0.00 | -0.02 | 0.02 |
| *Halomonas* | 0.93 | 0.89 | 1.14 | 0.73 | 0.75 | 0.68 | 0.00 | 0.00 | -0.01 | 0.00 | 0.00 | 0.01 |
| *Halotalea* | 1.18 | 1.00 | 0.93 | 0.79 | 0.70 | 0.64 | 0.01 | 0.00 | -0.01 | -0.01 | -0.01 | 0.00 |
| *Herbaspirillum* | 0.93 | 1.14 | 0.79 | 0.73 | 0.72 | 0.82 | -0.01 | 0.01 | 0.00 | -0.01 | -0.01 | 0.01 |
| *Immundisolibacter* | 1.02 | 0.93 | 1.19 | 0.78 | 0.66 | 0.65 | 0.01 | 0.01 | -0.01 | 0.00 | -0.01 | 0.00 |
| *Malassezia* | 0.96 | 0.63 | 0.80 | 0.51 | 1.54 | 1.40 | -0.01 | -0.02 | -0.01 | 0.00 | 0.04 | -0.03 |
| *Nitrosopumilus* | 0.76 | 0.77 | 1.01 | 1.06 | 0.94 | 0.42 | 0.01 | 0.01 | -0.01 | -0.01 | -0.01 | 0.00 |
| *Petrimonas* | 0.72 | 0.79 | 0.40 | 1.13 | 0.95 | 1.25 | 0.01 | -0.03 | 0.00 | -0.02 | 0.01 | -0.02 |
| *Pneumocystis* | 0.47 | 0.78 | 1.13 | 1.32 | 1.01 | 0.69 | 0.00 | 0.01 | -0.01 | -0.01 | -0.01 | -0.01 |
| *Selenomonas* | 0.43 | 1.13 | 0.48 | 0.80 | 1.07 | 2.01 | -0.01 | 0.03 | 0.00 | -0.01 | -0.03 | -0.04 |
| *Thiomonas* | 0.57 | 0.85 | 0.71 | 0.77 | 1.12 | 1.27 | 0.00 | -0.01 | 0.00 | 0.00 | 0.01 | 0.02 |
| *Trueperella* | 0.86 | 1.12 | 0.71 | 0.77 | 1.07 | 0.79 | -0.01 | 0.01 | -0.01 | -0.01 | 0.01 | -0.01 |
| *Wenzhouxiangella* | 1.15 | 0.95 | 0.96 | 0.77 | 0.77 | 0.77 | 0.01 | 0.00 | -0.01 | 0.00 | 0.00 | -0.01 |
| *Wickerhamomyces* | 0.59 | 0.80 | 0.93 | 1.12 | 1.29 | 0.57 | 0.00 | 0.01 | 0.00 | -0.01 | -0.01 | 0.00 |
| *Blastomonas* | 0.90 | 0.78 | 1.30 | 0.71 | 0.75 | 0.83 | 0.00 | 0.00 | -0.02 | 0.00 | -0.02 | -0.01 |
| *Candidatus Methanoperedens* | 0.82 | 0.94 | 0.77 | 0.77 | 0.71 | 1.24 | 0.00 | 0.01 | -0.01 | -0.01 | -0.01 | -0.02 |
| *Cronobacter* | 1.19 | 0.86 | 0.86 | 0.79 | 0.76 | 0.77 | 0.01 | -0.01 | -0.01 | 0.00 | 0.00 | -0.01 |
| *Eggerthella* | 0.62 | 1.37 | 1.08 | 0.78 | 0.42 | 3.57 | -0.01 | -0.05 | 0.01 | -0.01 | -0.01 | -0.07 |
| *Modestobacter* | 1.33 | 0.87 | 0.89 | 0.80 | 0.76 | 0.71 | 0.01 | 0.00 | -0.01 | -0.01 | 0.00 | -0.01 |
| *Synechococcus* | 1.01 | 0.80 | 0.81 | 0.78 | 0.72 | 1.44 | 0.01 | -0.02 | 0.00 | 0.00 | -0.01 | -0.02 |
| *Thiobacillus* | 1.23 | 0.73 | 1.54 | 1.03 | 0.80 | 0.57 | 0.01 | 0.00 | -0.02 | -0.01 | -0.02 | 0.00 |
| *Acidovorax* | 1.18 | 0.74 | 0.88 | 0.84 | 0.66 | 0.65 | -0.02 | -0.01 | -0.01 | -0.01 | 0.00 | -0.01 |
| *Aeromicrobium* | 0.91 | 0.79 | 1.02 | 1.00 | 0.76 | 0.67 | 0.00 | -0.01 | -0.01 | -0.01 | 0.00 | -0.01 |

| | | | | | | | | | | | | |
|---|---|---|---|---|---|---|---|---|---|---|---|---|
| *Calothrix* | 0.34 | 0.96 | 1.27 | 1.30 | 0.77 | 0.53 | 0.00 | 0.00 | -0.01 | -0.01 | 0.00 | 0.00 |
| *Nostoc* | 0.37 | 1.08 | 1.31 | 1.21 | 0.75 | 0.52 | 0.00 | -0.01 | -0.01 | -0.01 | 0.00 | -0.01 |
| *Pseudomonas* | 0.98 | 0.85 | 0.95 | 0.78 | 0.64 | 0.68 | -0.01 | -0.01 | -0.01 | -0.01 | -0.01 | 0.00 |
| *Phycomyces* | 0.80 | 0.78 | 0.78 | 0.75 | 0.69 | 0.97 | 0.01 | 0.01 | 0.00 | 0.00 | 0.01 | 0.01 |
| *Aspergillus* | 0.86 | 0.78 | 0.80 | 0.64 | 0.72 | 0.66 | 0.01 | 0.00 | 0.00 | 0.00 | 0.00 | 0.00 |
| *Fomitiporia* | 1.12 | 0.76 | 0.75 | 0.82 | 0.70 | 0.58 | 0.01 | 0.01 | 0.00 | 0.01 | 0.02 | -0.01 |
| *Methanothermococcus* | 0.29 | 0.75 | 0.80 | 0.79 | 0.77 | 1.08 | 0.00 | 0.01 | 0.00 | 0.00 | 0.02 | 0.02 |
| *Botrytis* | 0.67 | 0.96 | 0.76 | 0.75 | 0.69 | 0.96 | 0.00 | 0.00 | 0.00 | 0.01 | 0.02 | 0.02 |
| *Lodderomyces* | 0.65 | 0.99 | 0.98 | 0.77 | 0.68 | 0.70 | 0.00 | 0.00 | 0.00 | 0.00 | 0.01 | 0.00 |
| *Methanocaldococcus* | 0.45 | 1.39 | 0.75 | 0.75 | 0.78 | 1.12 | 0.00 | -0.02 | 0.00 | 0.00 | 0.00 | -0.02 |
| *Guillardia* | 0.68 | 0.73 | 0.89 | 0.76 | 0.79 | 1.35 | 0.01 | 0.02 | 0.00 | 0.00 | -0.01 | 0.03 |
| *Methylomonas* | 0.98 | 0.76 | 0.93 | 0.75 | 0.75 | 0.61 | 0.00 | -0.01 | -0.01 | 0.00 | 0.00 | 0.00 |
| *Naumovozyma* | 0.51 | 0.79 | 0.93 | 0.83 | 0.75 | 0.70 | 0.00 | 0.00 | 0.00 | 0.00 | 0.00 | -0.01 |
| *Microbulbifer* | 1.35 | 0.76 | 1.21 | 0.76 | 0.78 | 0.60 | 0.02 | -0.01 | -0.02 | -0.01 | 0.00 | 0.00 |
| *Rhodoferax* | 1.73 | 0.74 | 1.07 | 0.78 | 0.64 | 0.48 | -0.03 | -0.01 | 0.01 | -0.01 | 0.00 | -0.01 |
| *Salinispira* | 0.97 | 0.79 | 1.02 | 0.76 | 0.75 | 0.67 | 0.00 | 0.00 | -0.01 | 0.00 | 0.00 | 0.00 |
| *Sphingomonas* | 0.75 | 0.81 | 0.86 | 0.77 | 0.75 | 0.60 | 0.00 | -0.01 | -0.01 | 0.00 | -0.02 | 0.00 |
| *Stigmatella* | 0.97 | 0.74 | 0.87 | 0.80 | 0.64 | 0.73 | -0.01 | -0.01 | -0.01 | -0.01 | -0.01 | -0.01 |
| *Vanderwaltozyma* | 0.57 | 0.80 | 0.75 | 0.78 | 0.69 | 0.76 | 0.00 | 0.01 | 0.01 | 0.00 | 0.01 | 0.01 |

Partial Least Squares analysis was performed with 2 latent components. Subscript $_{VIP\geq0.8}$ denotes microbial genera that had VIP ≥ 0.8 in 4 or more timepoints. Subscripts $_{(+)}$ and $_{(-)}$ represent microbial genera that had positive or negative regression coefficient in 4 or more timepoints, respectively.

**Trait: Methane Yield (g/kg DMI, CH4Y); Predictors: 162 microbial genera (MT)**

| Microbial genera | VIP | | | | | | Regression coefficient | | | | | |
|---|---|---|---|---|---|---|---|---|---|---|---|---|
| | T1 | T2 | T3 | T4 | T5 | T6 | T1 | T2 | T3 | T4 | T5 | T6 |
| Negativicoccus$_{VIP\geq0.8\ (+)}$ | 1.83 | 2.40 | 2.02 | 2.14 | 2.14 | 0.95 | 0.06 | 0.05 | 0.07 | 0.07 | 0.06 | 0.03 |
| Methanothermobacter$_{VIP\geq0.8\ (}$ | 2.73 | 2.47 | 1.77 | 1.61 | 1.74 | 0.99 | 0.08 | 0.05 | 0.07 | 0.05 | 0.05 | 0.03 |
| Fonsecaea$_{VIP\geq0.8\ (+)}$ | 2.14 | 2.16 | 1.78 | 2.08 | 2.03 | 0.05 | 0.06 | 0.04 | 0.07 | 0.07 | 0.06 | 0.00 |
| Halapricum$_{VIP\geq0.8\ (+)}$ | 1.76 | 1.92 | 0.68 | 1.33 | 1.11 | 2.39 | 0.05 | 0.03 | 0.03 | 0.04 | 0.04 | 0.08 |
| Halococcus$_{VIP\geq0.8\ (+)}$ | 1.90 | 2.20 | 1.77 | 1.92 | 1.86 | 0.53 | 0.06 | 0.04 | 0.07 | 0.06 | 0.06 | 0.02 |
| Methanobacterium$_{VIP\geq0.8\ (+)}$ | 2.09 | 2.15 | 1.30 | 1.16 | 1.25 | 0.69 | 0.06 | 0.04 | 0.05 | 0.04 | 0.04 | 0.03 |
| Moritella$_{VIP\geq0.8\ (+)}$ | 2.26 | 1.05 | 0.54 | 1.20 | 0.82 | 0.97 | 0.06 | 0.02 | 0.02 | 0.04 | 0.03 | 0.04 |

| | | | | | | | | | | | | |
|---|---|---|---|---|---|---|---|---|---|---|---|---|
| Leptolyngbya$_{VIP≥0.8\ (+)}$ | 0.27 | 2.01 | 1.62 | 0.93 | 1.91 | 0.47 | 0.00 | 0.04 | 0.06 | 0.02 | 0.05 | 0.00 |
| Xanthomonas$_{VIP≥0.8\ (+)}$ | 0.35 | 1.22 | 1.05 | 0.82 | 1.92 | 0.60 | -0.01 | 0.02 | 0.04 | 0.03 | 0.06 | 0.02 |
| Methanolobus$_{VIP≥0.8\ (+)}$ | 1.51 | 1.25 | 1.06 | 1.72 | 0.43 | 0.42 | 0.04 | 0.02 | 0.04 | 0.06 | -0.01 | 0.02 |
| Nannochloropsis$_{VIP≥0.8\ (+)}$ | 1.42 | 1.90 | 0.98 | 0.78 | 0.41 | 2.16 | 0.04 | 0.04 | 0.04 | 0.03 | 0.01 | -0.06 |
| Pelodictyon$_{VIP≥0.8\ (+)}$ | 0.36 | 1.73 | 1.40 | 0.16 | 1.09 | 1.40 | 0.00 | 0.03 | 0.04 | 0.00 | 0.03 | 0.05 |
| Agaricus$_{VIP≥0.8\ (+)}$ | 0.60 | 0.36 | 0.97 | 0.99 | 1.02 | 1.20 | -0.02 | 0.01 | 0.04 | 0.03 | -0.01 | 0.04 |
| Gemmatimonas$_{VIP≥0.8\ (+)}$ | 0.09 | 1.40 | 1.68 | 0.82 | 0.76 | 2.85 | 0.00 | 0.02 | 0.05 | 0.01 | 0.01 | 0.09 |
| Ottowia$_{VIP≥0.8\ (+)}$ | 1.46 | 1.15 | 0.30 | 0.99 | 0.82 | 0.65 | 0.04 | 0.02 | 0.01 | 0.03 | -0.02 | -0.02 |
| Lodderomyces$_{VIP≥0.8\ (+)}$ | 1.22 | 0.24 | 0.85 | 0.87 | 0.79 | 0.87 | 0.01 | 0.00 | 0.00 | 0.00 | 0.01 | 0.00 |
| Chryseobacterium$_{VIP≥0.8\ (+)}$ | 1.28 | 1.85 | 1.83 | 1.27 | 1.58 | 0.47 | -0.03 | 0.04 | 0.05 | 0.02 | 0.03 | 0.00 |
| Pichia$_{VIP≥0.8\ (+)}$ | 1.21 | 0.18 | 0.96 | 0.82 | 0.81 | 0.63 | 0.02 | 0.00 | -0.01 | 0.01 | 0.01 | 0.01 |
| Cordyceps$_{VIP≥0.8\ (+)}$ | 0.86 | 0.41 | 0.80 | 0.82 | 0.84 | 0.87 | -0.01 | -0.01 | 0.01 | 0.02 | 0.01 | 0.00 |
| Zygosaccharomyces$_{VIP≥0.8\ (+)}$ | 1.13 | 0.42 | 0.80 | 0.90 | 0.83 | 0.82 | 0.01 | -0.01 | 0.01 | 0.00 | 0.01 | 0.01 |
| Candida$_{VIP≥0.8\ (+)}$ | 0.99 | 0.11 | 0.79 | 0.90 | 0.90 | 0.84 | 0.00 | 0.00 | 0.01 | 0.00 | 0.00 | 0.01 |
| Ectothiorhodospira$_{VIP≥0.8\ (+)}$ | 0.18 | 1.05 | 1.00 | 1.20 | 1.00 | 0.52 | 0.00 | 0.02 | 0.02 | 0.03 | 0.01 | 0.02 |
| Mesorhizobium$_{VIP≥0.8\ (+)}$ | 0.83 | 0.38 | 0.71 | 0.98 | 0.93 | 1.06 | 0.00 | 0.00 | -0.02 | 0.00 | 0.00 | 0.00 |
| Jeotgalicoccus$_{VIP≥0.8\ (+)}$ | 0.89 | 1.08 | 0.48 | 1.01 | 0.83 | 0.18 | 0.03 | 0.02 | 0.00 | 0.03 | -0.02 | -0.01 |
| Janibacter$_{VIP≥0.8\ (+)}$ | 0.61 | 1.24 | 1.34 | 0.50 | 0.87 | 0.82 | 0.02 | -0.02 | -0.05 | 0.01 | 0.00 | 0.01 |
| Pelobacter$_{VIP≥0.8\ (+)}$ | 1.29 | 2.57 | 1.48 | 0.63 | 0.64 | 1.37 | -0.02 | 0.05 | 0.03 | 0.01 | -0.02 | 0.04 |
| Methanococcus$_{VIP≥0.8\ (+)}$ | 0.87 | 0.35 | 0.82 | 0.73 | 0.83 | 0.94 | 0.00 | 0.00 | 0.01 | 0.01 | 0.00 | 0.00 |
| Natrialba$_{VIP≥0.8\ (+)}$ | 1.66 | 1.38 | 1.14 | 0.36 | 0.87 | 1.00 | 0.05 | 0.03 | 0.03 | -0.01 | 0.02 | -0.04 |
| Arthrobotrys$_{VIP≥0.8\ (+)}$ | 0.95 | 0.22 | 0.85 | 0.91 | 0.90 | 0.81 | 0.00 | 0.00 | 0.00 | 0.00 | 0.00 | 0.01 |
| Scedosporium$_{VIP≥0.8\ (+)}$ | 0.93 | 0.29 | 0.82 | 0.90 | 0.70 | 1.00 | 0.00 | 0.00 | 0.01 | 0.00 | 0.01 | -0.01 |
| Puccinia$_{VIP≥0.8\ (+)}$ | 0.92 | 0.12 | 1.05 | 0.71 | 0.84 | 0.86 | 0.00 | 0.00 | -0.01 | 0.01 | 0.00 | 0.00 |
| Methylobacterium$_{VIP≥0.8\ (+)}$ | 1.13 | 0.21 | 0.93 | 0.82 | 0.90 | 1.02 | -0.01 | 0.00 | 0.00 | -0.02 | 0.00 | 0.01 |
| Cryptosporidium$_{VIP≥0.8\ (+)}$ | 0.86 | 0.17 | 0.79 | 0.87 | 0.95 | 0.87 | -0.01 | 0.00 | 0.01 | 0.00 | 0.00 | 0.00 |
| Archangium$_{VIP≥0.8\ (+)}$ | 0.89 | 0.47 | 0.94 | 0.83 | 1.18 | 0.60 | 0.02 | 0.00 | 0.01 | 0.00 | -0.04 | 0.00 |
| Geoalkalibacter$_{VIP≥0.8\ (+)}$ | 0.85 | 0.56 | 1.00 | 0.82 | 0.96 | 1.26 | 0.00 | 0.00 | 0.01 | -0.01 | 0.00 | 0.01 |
| Nitrosospira$_{VIP≥0.8\ (+)}$ | 0.76 | 0.32 | 0.86 | 0.94 | 0.97 | 0.90 | 0.00 | 0.01 | 0.00 | 0.00 | 0.00 | 0.00 |
| Cronobacter$_{VIP≥0.8\ (+)}$ | 0.78 | 0.42 | 1.05 | 1.07 | 0.85 | 0.90 | 0.01 | 0.01 | 0.01 | 0.01 | -0.01 | -0.01 |

| | | | | | | | | | | | | |
|---|---|---|---|---|---|---|---|---|---|---|---|---|
| Tetrapisispora$_{VIP\geq0.8\ (+)}$ | 0.82 | 0.80 | 0.76 | 0.87 | 0.80 | 0.87 | -0.01 | -0.02 | 0.01 | 0.00 | 0.01 | 0.00 |
| Enterobacter$_{VIP\geq0.8\ (+)}$ | 0.57 | 0.63 | 0.99 | 0.90 | 0.80 | 0.91 | 0.01 | 0.01 | 0.01 | 0.00 | 0.00 | 0.00 |
| Eutypa$_{VIP\geq0.8\ (+)}$ | 0.92 | 0.30 | 0.79 | 0.81 | 0.93 | 0.90 | -0.03 | -0.01 | 0.01 | 0.01 | 0.00 | 0.00 |
| Rathayibacter$_{VIP\geq0.8\ (+)}$ | 1.18 | 0.30 | 0.75 | 0.86 | 1.08 | 0.84 | -0.02 | -0.01 | 0.00 | 0.01 | 0.02 | 0.00 |
| Halothiobacillus$_{VIP\geq0.8\ (+)}$ | 1.09 | 0.61 | 1.00 | 0.95 | 1.02 | 1.09 | 0.03 | -0.01 | 0.01 | 0.00 | 0.01 | 0.01 |
| Shimwellia$_{VIP\geq0.8\ (+)}$ | 0.88 | 1.39 | 1.24 | 0.97 | 0.66 | 0.70 | 0.03 | 0.03 | 0.02 | 0.00 | 0.00 | -0.01 |
| Acidihalobacter$_{VIP\geq0.8\ (+)}$ | 0.74 | 0.94 | 0.92 | 0.88 | 1.02 | 0.93 | 0.01 | 0.02 | 0.01 | 0.00 | 0.01 | -0.03 |
| Alcanivorax$_{VIP\geq0.8\ (+)}$ | 0.84 | 0.46 | 1.32 | 0.98 | 1.09 | 0.85 | 0.00 | 0.01 | 0.02 | 0.00 | 0.01 | -0.01 |
| Halomonas$_{VIP\geq0.8\ (+)}$ | 0.79 | 0.31 | 1.15 | 0.95 | 0.87 | 1.01 | 0.02 | 0.00 | 0.02 | 0.00 | 0.00 | 0.00 |
| Jeongeupia$_{VIP\geq0.8\ (+)}$ | 0.68 | 0.24 | 0.87 | 0.84 | 1.17 | 0.91 | 0.02 | 0.00 | 0.01 | 0.00 | 0.01 | 0.01 |
| Fibrobacter$_{VIP\geq0.8\ (-)}$ | 0.67 | 1.10 | 1.00 | 1.18 | 0.94 | 0.71 | -0.02 | -0.02 | -0.02 | -0.02 | -0.01 | 0.02 |
| Kandleria$_{VIP\geq0.8\ (-)}$ | 0.98 | 1.91 | 2.02 | 1.57 | 0.36 | 1.99 | -0.03 | -0.04 | -0.07 | -0.05 | 0.01 | -0.07 |
| Pectobacterium$_{VIP\geq0.8\ (-)}$ | 0.58 | 1.61 | 1.45 | 1.21 | 1.25 | 0.33 | 0.00 | -0.03 | -0.06 | -0.04 | -0.03 | -0.01 |
| Alkaliphilus$_{VIP\geq0.8\ (-)}$ | 0.93 | 1.68 | 1.43 | 1.20 | 0.96 | 1.16 | -0.03 | -0.03 | -0.05 | -0.03 | -0.03 | -0.03 |
| Cutaneotrichosporon$_{VIP\geq0.8\ (-)}$ | 0.99 | 1.55 | 0.46 | 1.44 | 1.22 | 2.33 | -0.03 | -0.03 | -0.02 | -0.05 | -0.04 | 0.06 |
| Lacinutrix$_{VIP\geq0.8\ (-)}$ | 0.39 | 0.55 | 0.95 | 1.44 | 2.10 | 2.16 | 0.00 | 0.01 | -0.02 | -0.04 | -0.06 | -0.07 |
| Candidatus Carsonella$_{VIP\geq0.8}$ | 0.85 | 2.24 | 0.97 | 0.77 | 0.90 | 1.28 | -0.03 | -0.04 | -0.02 | -0.02 | -0.02 | -0.03 |
| Candidatus Koribacter$_{VIP\geq0.8}$ | 0.84 | 0.51 | 0.57 | 0.99 | 1.57 | 0.81 | 0.00 | -0.01 | -0.01 | 0.00 | -0.05 | 0.00 |
| Pannonibacter$_{VIP\geq0.8\ (-)}$ | 0.98 | 0.79 | 0.80 | 0.83 | 0.78 | 1.00 | 0.00 | -0.01 | -0.03 | -0.02 | -0.01 | 0.00 |
| Gordonibacter$_{VIP\geq0.8\ (-)}$ | 0.39 | 0.83 | 1.19 | 0.73 | 1.16 | 0.86 | 0.01 | 0.01 | -0.05 | -0.01 | -0.04 | -0.03 |
| Wenyingzhuangia$_{VIP\geq0.8\ (-)}$ | 0.51 | 1.10 | 1.36 | 1.47 | 0.37 | 1.58 | 0.00 | -0.02 | -0.04 | -0.04 | 0.01 | -0.04 |
| Arthrobacter$_{VIP\geq0.8\ (-)}$ | 0.98 | 0.31 | 0.99 | 0.82 | 1.04 | 0.86 | 0.00 | 0.00 | 0.01 | -0.01 | -0.03 | -0.01 |
| Zhongshania$_{VIP\geq0.8\ (-)}$ | 0.81 | 0.77 | 0.89 | 0.77 | 0.87 | 1.07 | 0.00 | -0.01 | 0.00 | -0.01 | 0.00 | -0.04 |
| Halotalea$_{VIP\geq0.8\ (-)}$ | 1.04 | 0.56 | 0.79 | 0.81 | 0.82 | 0.91 | -0.01 | -0.01 | 0.00 | -0.01 | -0.01 | 0.00 |
| Sphingomonas$_{VIP\geq0.8\ (-)}$ | 1.01 | 0.24 | 0.77 | 0.87 | 0.81 | 1.01 | -0.01 | 0.01 | -0.01 | -0.01 | -0.02 | 0.00 |
| Agrobacterium$_{VIP\geq0.8\ (-)}$ | 0.92 | 0.28 | 0.78 | 0.89 | 0.82 | 1.04 | 0.00 | 0.00 | -0.01 | -0.01 | -0.01 | 0.00 |
| Syntrophomonas$_{VIP\geq0.8\ (-)}$ | 0.85 | 0.87 | 1.56 | 1.08 | 0.64 | 0.59 | -0.03 | -0.02 | -0.04 | -0.02 | -0.02 | 0.02 |
| Wenzhouxiangella$_{VIP\geq0.8\ (-)}$ | 0.77 | 0.40 | 0.80 | 0.92 | 0.83 | 0.82 | 0.01 | 0.00 | 0.00 | 0.00 | -0.01 | -0.01 |
| Cloacibacillus$_{VIP\geq0.8\ (-)}$ | 0.81 | 0.34 | 0.79 | 0.81 | 0.98 | 1.37 | -0.01 | 0.00 | 0.00 | -0.02 | 0.00 | 0.02 |
| Geobacter$_{VIP\geq0.8\ (-)}$ | 0.84 | 0.56 | 0.78 | 0.84 | 0.81 | 0.83 | 0.00 | 0.01 | 0.00 | -0.01 | -0.01 | 0.00 |

| | | | | | | | | | | | | |
|---|---|---|---|---|---|---|---|---|---|---|---|---|
| Emiliania$_{VIP\geq0.8\ (-)}$ | 1.16 | 0.32 | 0.69 | 1.16 | 1.13 | 1.58 | -0.04 | -0.01 | -0.01 | -0.03 | -0.03 | 0.05 |
| Rhizobium$_{VIP\geq0.8\ (-)}$ | 0.95 | 0.47 | 0.87 | 0.86 | 0.88 | 1.11 | 0.00 | 0.01 | 0.00 | -0.01 | 0.00 | 0.00 |
| Planktothrix$_{VIP\geq0.8\ (-)}$ | 1.04 | 0.79 | 1.36 | 1.11 | 1.15 | 0.62 | 0.00 | -0.02 | -0.03 | -0.02 | -0.02 | 0.01 |
| Tetrahymena$_{VIP\geq0.8\ (-)}$ | 0.86 | 0.43 | 0.93 | 1.02 | 0.93 | 0.83 | -0.01 | 0.00 | 0.00 | -0.01 | 0.00 | 0.01 |
| Dictyostelium$_{VIP\geq0.8\ (-)}$ | 0.88 | 0.17 | 0.90 | 0.96 | 0.97 | 0.87 | -0.01 | 0.00 | 0.00 | 0.00 | 0.00 | 0.00 |
| Naegleria$_{VIP\geq0.8\ (-)}$ | 0.85 | 0.26 | 0.86 | 0.99 | 1.06 | 0.84 | -0.01 | 0.00 | 0.00 | -0.01 | 0.00 | 0.00 |
| Ichthyophthirius$_{VIP\geq0.8\ (-)}$ | 0.86 | 0.21 | 0.84 | 0.99 | 0.99 | 0.90 | -0.01 | 0.00 | 0.00 | 0.00 | 0.00 | 0.00 |
| Wickerhamomyces$_{VIP\geq0.8\ (-)}$ | 0.84 | 0.08 | 0.87 | 1.03 | 0.90 | 0.81 | -0.01 | 0.00 | 0.00 | -0.01 | 0.00 | 0.01 |
| Methanocaldococcus$_{VIP\geq0.8\ (-}$ | 0.76 | 1.15 | 0.87 | 1.14 | 0.99 | 0.78 | 0.00 | -0.02 | 0.00 | -0.01 | 0.00 | 0.02 |
| Cyanothece$_{VIP\geq0.8\ (-)}$ | 0.65 | 0.60 | 1.56 | 1.88 | 1.04 | 0.97 | 0.00 | 0.01 | -0.03 | -0.04 | -0.01 | -0.01 |
| Stereum$_{VIP\geq0.8\ (-)}$ | 1.45 | 0.25 | 1.01 | 1.69 | 1.07 | 0.67 | -0.04 | -0.01 | -0.01 | -0.04 | -0.01 | 0.02 |
| Pneumocystis$_{VIP\geq0.8\ (-)}$ | 0.82 | 0.45 | 0.88 | 0.81 | 1.11 | 0.78 | -0.01 | -0.01 | 0.00 | 0.00 | -0.01 | 0.01 |
| Mycoplasma$_{VIP\geq0.8\ (-)}$ | 1.21 | 1.14 | 0.88 | 0.83 | 0.91 | 0.45 | 0.02 | -0.02 | 0.00 | -0.01 | -0.01 | 0.00 |
| Nosema$_{VIP\geq0.8\ (-)}$ | 0.81 | 0.18 | 0.95 | 1.17 | 1.00 | 0.81 | -0.01 | 0.00 | -0.01 | -0.01 | 0.00 | 0.01 |
| Kwoniella$_{VIP\geq0.8\ (-)}$ | 0.86 | 0.70 | 0.81 | 0.99 | 0.99 | 0.94 | -0.01 | -0.01 | 0.01 | -0.01 | 0.00 | 0.00 |
| Methanotorris$_{VIP\geq0.8\ (-)}$ | 0.68 | 0.43 | 1.07 | 1.45 | 1.10 | 0.85 | -0.01 | 0.01 | -0.01 | -0.03 | -0.01 | -0.01 |
| Phialocephala$_{VIP\geq0.8\ (-)}$ | 1.23 | 0.75 | 0.90 | 1.04 | 1.25 | 0.65 | -0.04 | -0.02 | 0.00 | -0.01 | -0.01 | 0.02 |
| Candidatus Profftella$_{VIP\geq0.8\ (-)}$ | 0.80 | 0.51 | 1.15 | 0.98 | 0.59 | 0.94 | -0.02 | 0.01 | -0.02 | -0.02 | 0.01 | -0.02 |
| Candidatus Phytoplasma$_{VIP\geq}$ | 0.99 | 0.44 | 1.17 | 1.06 | 0.88 | 1.18 | 0.01 | -0.01 | -0.01 | -0.01 | 0.00 | -0.02 |
| Fusarium$_{VIP\geq0.8\ (-)}$ | 0.82 | 0.21 | 0.99 | 0.93 | 0.67 | 1.15 | -0.02 | 0.00 | -0.01 | -0.02 | 0.02 | -0.01 |
| Rhodovulum$_{VIP\geq0.8}$ | 1.17 | 0.81 | 1.06 | 0.90 | 1.03 | 0.85 | -0.02 | -0.01 | 0.02 | 0.00 | -0.03 | 0.00 |
| Chlorobaculum$_{VIP\geq0.8}$ | 0.91 | 0.17 | 0.97 | 0.86 | 0.86 | 0.87 | -0.01 | 0.00 | 0.01 | 0.00 | 0.00 | 0.00 |
| Dactylellina$_{VIP\geq0.8}$ | 0.86 | 0.17 | 0.89 | 0.86 | 0.93 | 1.04 | -0.01 | 0.00 | 0.00 | 0.00 | 0.00 | -0.01 |
| Dyella$_{VIP\geq0.8}$ | 0.80 | 0.38 | 0.85 | 0.89 | 0.85 | 1.05 | 0.00 | 0.00 | 0.00 | 0.00 | 0.00 | 0.00 |
| Novosphingobium$_{VIP\geq0.8}$ | 1.27 | 1.48 | 1.28 | 0.11 | 0.89 | 0.95 | -0.02 | -0.03 | 0.03 | 0.00 | 0.01 | 0.03 |
| Pantoea$_{VIP\geq0.8}$ | 1.05 | 0.51 | 0.81 | 1.00 | 1.07 | 0.90 | -0.01 | 0.01 | 0.00 | 0.01 | 0.01 | -0.01 |
| Paracoccidioides$_{VIP\geq0.8}$ | 0.83 | 0.50 | 0.88 | 0.88 | 0.92 | 0.87 | -0.01 | -0.01 | 0.00 | 0.00 | 0.00 | 0.00 |
| Sedimenticola$_{VIP\geq0.8}$ | 1.21 | 1.04 | 0.97 | 1.01 | 0.70 | 1.52 | -0.02 | -0.02 | 0.01 | 0.00 | -0.01 | 0.02 |
| Thioflavicoccus$_{VIP\geq0.8}$ | 0.88 | 0.62 | 0.85 | 0.85 | 0.93 | 0.80 | -0.01 | 0.01 | 0.00 | 0.00 | 0.00 | -0.01 |
| Cupriavidus$_{VIP\geq0.8}$ | 0.96 | 0.41 | 0.77 | 0.83 | 0.92 | 1.00 | -0.01 | 0.01 | -0.01 | -0.01 | 0.00 | 0.00 |

| | | | | | | | | | | | | |
|---|---|---|---|---|---|---|---|---|---|---|---|---|
| Debaryomyces$_{VIP≥0.8}$ | 0.92 | 0.29 | 0.77 | 0.81 | 1.18 | 0.83 | -0.03 | 0.00 | 0.01 | 0.01 | -0.01 | 0.03 |
| Dickeya$_{VIP≥0.8}$ | 0.92 | 0.33 | 1.50 | 1.02 | 0.78 | 0.92 | -0.01 | 0.00 | 0.03 | 0.01 | -0.02 | 0.00 |
| Exiguobacterium$_{VIP≥0.8}$ | 1.18 | 1.23 | 0.67 | 0.98 | 0.67 | 0.87 | -0.02 | -0.02 | 0.00 | 0.01 | -0.02 | 0.01 |
| Gordonia$_{VIP≥0.8}$ | 0.95 | 0.48 | 0.71 | 1.00 | 1.08 | 0.81 | 0.00 | 0.01 | -0.01 | 0.00 | 0.01 | 0.00 |
| Halorhodospira$_{VIP≥0.8}$ | 0.75 | 0.63 | 0.86 | 1.03 | 0.82 | 0.90 | 0.00 | -0.01 | 0.00 | 0.00 | -0.02 | 0.00 |
| Neisseria$_{VIP≥0.8}$ | 1.20 | 0.68 | 0.82 | 1.05 | 0.75 | 0.85 | -0.02 | 0.01 | 0.00 | 0.01 | -0.02 | 0.00 |
| Perkinsus$_{VIP≥0.8}$ | 0.92 | 0.62 | 0.92 | 0.99 | 0.90 | 0.71 | 0.00 | -0.01 | 0.00 | -0.01 | 0.00 | 0.02 |
| Spathaspora$_{VIP≥0.8}$ | 0.91 | 0.06 | 0.63 | 1.05 | 0.99 | 0.85 | 0.00 | 0.00 | 0.01 | -0.01 | -0.01 | 0.00 |
| Sugiyamaella$_{VIP≥0.8}$ | 0.72 | 0.33 | 0.87 | 0.89 | 0.90 | 1.21 | -0.01 | -0.01 | 0.00 | 0.00 | 0.00 | -0.01 |
| Thioalkalivibrio$_{VIP≥0.8}$ | 0.77 | 0.21 | 0.95 | 0.88 | 0.82 | 0.91 | 0.00 | 0.00 | 0.01 | -0.01 | 0.00 | 0.00 |
| Trichodesmium$_{VIP≥0.8}$ | 0.81 | 0.10 | 0.83 | 1.12 | 0.72 | 0.87 | 0.00 | 0.00 | 0.00 | -0.01 | 0.01 | 0.00 |
| Variovorax$_{VIP≥0.8}$ | 0.98 | 0.46 | 1.13 | 0.90 | 0.77 | 1.26 | -0.01 | 0.01 | 0.02 | 0.00 | -0.01 | 0.01 |
| *Microsporum* | 0.98 | 0.75 | 0.78 | 0.86 | 0.83 | 0.77 | 0.00 | 0.01 | 0.01 | 0.02 | 0.01 | 0.01 |
| *Olsenella* | 0.62 | 0.88 | 0.87 | 0.69 | 0.75 | 0.95 | 0.01 | 0.02 | 0.01 | 0.01 | 0.00 | 0.01 |
| *Methanobrevibacter* | 0.18 | 1.96 | 1.28 | 0.79 | 0.43 | 1.43 | 0.00 | 0.03 | 0.05 | 0.03 | 0.01 | 0.05 |
| *Methanoculleus* | 0.75 | 1.53 | 0.72 | 0.70 | 2.28 | 1.74 | 0.02 | 0.03 | 0.01 | 0.00 | 0.05 | 0.05 |
| *Nakaseomyces* | 1.34 | 0.18 | 0.74 | 0.88 | 0.89 | 0.80 | 0.02 | 0.00 | 0.01 | -0.01 | 0.00 | 0.01 |
| *Naumovozyma* | 0.93 | 0.69 | 0.74 | 0.82 | 0.79 | 0.85 | 0.00 | -0.01 | 0.01 | 0.02 | 0.00 | 0.00 |
| *Pelosinus* | 0.76 | 0.45 | 1.44 | 1.27 | 0.64 | 0.98 | 0.02 | 0.01 | 0.04 | 0.02 | -0.02 | 0.01 |
| *Pochonia* | 0.96 | 0.84 | 0.65 | 0.94 | 0.77 | 0.72 | 0.00 | 0.01 | 0.01 | 0.00 | 0.01 | 0.02 |
| *Sphingobium* | 0.69 | 0.73 | 0.87 | 0.77 | 1.36 | 0.81 | 0.02 | 0.01 | 0.00 | -0.01 | 0.02 | 0.00 |
| *Atopobium* | 0.90 | 1.87 | 0.62 | 1.03 | 0.66 | 0.48 | 0.03 | 0.03 | -0.01 | 0.01 | 0.01 | 0.00 |
| *Desulfobacterium* | 1.05 | 0.31 | 0.95 | 0.36 | 0.75 | 1.20 | -0.02 | -0.01 | 0.02 | 0.00 | 0.02 | 0.03 |
| *Leptothrix* | 0.76 | 0.46 | 0.87 | 0.98 | 0.78 | 0.93 | 0.00 | 0.00 | 0.00 | 0.00 | 0.00 | 0.00 |
| *Libanicoccus* | 0.72 | 1.01 | 0.71 | 0.70 | 0.82 | 0.98 | 0.02 | 0.02 | -0.02 | 0.00 | 0.01 | 0.01 |
| *Oceanimonas* | 0.82 | 0.72 | 1.03 | 1.22 | 0.46 | 0.59 | 0.03 | 0.01 | 0.02 | 0.02 | -0.01 | 0.00 |
| *Tolumonas* | 0.74 | 0.67 | 1.00 | 1.31 | 0.75 | 0.93 | 0.02 | -0.01 | 0.01 | 0.02 | -0.01 | 0.00 |
| *Acidaminococcus* | 0.78 | 0.26 | 1.08 | 0.89 | 0.76 | 0.97 | 0.02 | 0.00 | 0.01 | 0.00 | -0.01 | 0.00 |
| *Bibersteinia* | 1.44 | 1.38 | 0.34 | 0.84 | 0.11 | 0.79 | -0.04 | 0.02 | 0.00 | -0.02 | 0.00 | -0.01 |
| *Bradyrhizobium* | 0.79 | 0.17 | 0.85 | 0.81 | 0.79 | 0.94 | 0.01 | 0.00 | 0.00 | -0.02 | -0.01 | 0.00 |
| *Clostridioides* | 0.85 | 1.60 | 0.51 | 0.75 | 0.94 | 0.55 | -0.01 | -0.03 | 0.00 | 0.01 | 0.02 | -0.01 |
| *Deferribacter* | 0.94 | 0.77 | 0.73 | 1.00 | 1.25 | 0.56 | 0.01 | -0.02 | 0.02 | -0.01 | -0.02 | 0.01 |
| *Edwardsiella* | 0.80 | 0.32 | 0.97 | 0.87 | 0.77 | 0.92 | 0.01 | 0.00 | 0.00 | -0.01 | -0.01 | 0.00 |

| | | | | | | | | | | | | |
|---|---|---|---|---|---|---|---|---|---|---|---|---|
| *Laribacter* | 0.77 | 0.91 | 0.79 | 0.93 | 0.81 | 0.31 | -0.01 | -0.01 | 0.01 | 0.02 | 0.01 | 0.00 |
| *Microterricola* | 0.61 | 1.32 | 0.74 | 1.32 | 0.83 | 0.60 | -0.01 | 0.02 | -0.03 | 0.04 | 0.02 | -0.02 |
| *Thalassiosira* | 0.41 | 0.58 | 1.28 | 1.06 | 1.24 | 0.69 | 0.00 | 0.01 | -0.02 | 0.04 | -0.02 | -0.01 |
| *Burkholderia* | 1.21 | 0.31 | 0.72 | 0.87 | 0.78 | 0.96 | -0.01 | 0.00 | -0.01 | -0.01 | -0.01 | 0.00 |
| *Cladophialophora* | 0.95 | 1.30 | 0.08 | 0.49 | 0.85 | 0.66 | -0.03 | -0.02 | 0.00 | -0.01 | -0.02 | 0.02 |
| *Devosia* | 1.07 | 1.09 | 1.09 | 0.40 | 0.63 | 0.32 | -0.02 | -0.02 | -0.05 | 0.01 | 0.01 | -0.01 |
| *Endocarpon* | 0.98 | 0.68 | 0.77 | 0.88 | 0.73 | 0.95 | -0.03 | -0.01 | 0.00 | 0.03 | 0.01 | -0.01 |
| *Halobacillus* | 0.74 | 2.28 | 1.24 | 0.75 | 0.11 | 1.06 | 0.01 | -0.04 | -0.04 | -0.01 | 0.00 | -0.02 |
| *Halocynthiibacter* | 0.92 | 1.10 | 0.61 | 0.73 | 0.82 | 0.79 | -0.02 | -0.02 | 0.00 | -0.02 | 0.00 | 0.00 |
| *Immundisolibacter* | 0.75 | 1.54 | 0.78 | 0.99 | 0.77 | 0.88 | 0.01 | -0.03 | 0.00 | 0.00 | 0.00 | -0.01 |
| *Melioribacter* | 0.21 | 1.63 | 0.70 | 0.69 | 1.19 | 1.12 | -0.01 | -0.03 | -0.02 | 0.01 | -0.04 | 0.02 |
| *Salinispira* | 0.70 | 0.65 | 0.76 | 0.84 | 0.99 | 0.85 | 0.01 | -0.01 | 0.00 | -0.01 | 0.01 | -0.01 |
| *Spizellomyces* | 0.86 | 0.20 | 0.72 | 0.95 | 1.00 | 0.78 | -0.01 | 0.00 | 0.01 | -0.01 | -0.01 | 0.00 |
| *Blastochloris* | 0.80 | 0.89 | 0.66 | 1.11 | 0.76 | 0.71 | 0.00 | -0.01 | -0.01 | 0.01 | 0.00 | -0.02 |
| *Corynebacterium* | 0.96 | 1.10 | 0.73 | 0.93 | 0.78 | 0.74 | 0.00 | -0.02 | -0.02 | 0.00 | -0.01 | -0.01 |
| *Mesoplasma* | 1.12 | 0.72 | 0.66 | 1.22 | 0.71 | 1.15 | 0.02 | -0.01 | 0.00 | -0.02 | -0.01 | -0.03 |
| *Pluralibacter* | 0.93 | 0.75 | 0.94 | 0.85 | 0.80 | 0.77 | 0.00 | -0.01 | 0.01 | -0.01 | -0.01 | -0.02 |
| *Selenomonas* | 0.82 | 0.08 | 0.41 | 0.90 | 0.94 | 0.73 | -0.02 | 0.00 | -0.01 | -0.03 | -0.03 | 0.00 |
| *Sideroxydans* | 0.93 | 0.39 | 0.85 | 0.93 | 0.79 | 0.78 | -0.01 | 0.00 | 0.00 | 0.00 | -0.01 | -0.01 |
| *Aerococcus* | 0.63 | 0.84 | 1.00 | 1.72 | 0.52 | 0.35 | -0.01 | -0.02 | -0.04 | -0.05 | -0.01 | -0.01 |
| *Aromatoleum* | 1.09 | 0.74 | 0.90 | 0.86 | 0.78 | 0.73 | -0.01 | -0.01 | -0.04 | -0.02 | 0.00 | -0.02 |
| *Thauera* | 0.98 | 0.34 | 0.67 | 0.85 | 0.78 | 0.87 | -0.01 | 0.00 | -0.02 | -0.01 | 0.00 | 0.00 |
| *Thermosipho* | 0.41 | 1.28 | 1.92 | 0.58 | 1.34 | 0.48 | -0.01 | -0.02 | -0.06 | -0.01 | -0.04 | -0.01 |
| *Lysobacter* | 0.33 | 1.12 | 0.80 | 0.77 | 2.06 | 0.65 | 0.01 | 0.02 | 0.03 | 0.01 | 0.05 | 0.00 |
| *Guillardia* | 0.87 | 0.08 | 0.78 | 0.79 | 0.71 | 1.61 | 0.00 | 0.00 | 0.01 | 0.02 | 0.00 | 0.06 |
| *Actinomyces* | 0.75 | 1.78 | 0.66 | 0.42 | 0.78 | 1.04 | 0.00 | 0.03 | -0.01 | -0.01 | 0.01 | 0.02 |
| *Croceicoccus* | 1.09 | 0.48 | 0.74 | 0.63 | 0.77 | 1.60 | -0.02 | 0.00 | 0.01 | -0.01 | 0.02 | 0.04 |
| *Chamaesiphon* | 0.85 | 0.10 | 1.10 | 0.77 | 0.67 | 0.76 | 0.01 | 0.00 | -0.01 | -0.01 | 0.00 | -0.01 |
| *Moesziomyces* | 1.73 | 0.67 | 0.54 | 0.61 | 0.80 | 0.90 | -0.04 | -0.01 | -0.02 | -0.01 | 0.01 | 0.01 |
| *Thermoanaerobacter* | 0.85 | 1.54 | 0.78 | 0.60 | 0.47 | 0.30 | -0.01 | -0.03 | -0.03 | 0.01 | 0.00 | 0.00 |
| *Natranaerobius* | 0.59 | 0.97 | 0.79 | 0.77 | 1.60 | 0.60 | -0.02 | -0.02 | -0.03 | -0.03 | -0.05 | -0.02 |
| *Sphaerulina* | 1.01 | 0.14 | 0.64 | 0.79 | 0.76 | 0.79 | 0.00 | 0.00 | 0.02 | 0.01 | 0.00 | 0.01 |

Partial Least Squares analysis was performed with 2 latent components. Subscript $_{VIP\geq0.8}$ denotes microbial genera that had VIP ≥ 0.8 in 4 or more timepoints. Subscripts $_{(+)}$ and $_{(-)}$ represent microbial genera that had positive or negative regression coefficient in 4 or more timepoints, respectively.

**Trait: Daily Methane Production (g/day, CH4P); Predictors: 288 microbial genera (MT)**

| Microbial genera | VIP | | | | | | Regression coefficient | | | | | |
|---|---|---|---|---|---|---|---|---|---|---|---|---|
| | T1 | T2 | T3 | T4 | T5 | T6 | T1 | T2 | T3 | T4 | T5 | T6 |
| Halapricum$_{VIP\geq0.8\ (+)}$ | 1.97 | 3.11 | 1.23 | 2.44 | 2.22 | 2.50 | 0.03 | 0.05 | 0.01 | 0.03 | 0.04 | 0.03 |
| Negativicoccus$_{VIP\geq0.8\ (+)}$ | 2.06 | 3.43 | 1.89 | 2.48 | 2.73 | 1.07 | 0.03 | 0.06 | 0.02 | 0.03 | 0.06 | 0.01 |
| Methanothermobacter$_{VIP\geq0.8\ (}$ | 1.48 | 3.13 | 1.98 | 2.51 | 2.37 | 1.31 | 0.02 | 0.05 | 0.02 | 0.03 | 0.05 | 0.02 |
| Methanobacterium$_{VIP\geq0.8\ (+)}$ | 2.34 | 3.19 | 2.09 | 1.79 | 2.00 | 1.06 | 0.03 | 0.05 | 0.02 | 0.02 | 0.04 | 0.01 |
| Halococcus$_{VIP\geq0.8\ (+)}$ | 2.07 | 3.06 | 1.66 | 2.54 | 2.62 | 0.51 | 0.03 | 0.05 | 0.02 | 0.03 | 0.05 | 0.01 |
| Fonsecaea$_{VIP\geq0.8\ (+)}$ | 2.51 | 2.17 | 1.15 | 1.75 | 2.77 | 0.17 | 0.04 | 0.04 | 0.01 | 0.02 | 0.06 | 0.00 |
| Methanobrevibacter$_{VIP\geq0.8\ (+)}$ | 0.45 | 1.81 | 1.53 | 1.06 | 0.94 | 1.40 | 0.01 | 0.03 | 0.02 | 0.01 | 0.02 | 0.02 |
| Bifidobacterium$_{VIP\geq0.8\ (+)}$ | 0.21 | 0.88 | 0.91 | 1.42 | 1.27 | 0.67 | 0.00 | 0.02 | 0.01 | 0.02 | 0.03 | 0.01 |
| Niastella$_{VIP\geq0.8\ (+)}$ | 0.17 | 0.21 | 0.86 | 1.44 | 1.06 | 1.47 | 0.00 | 0.00 | 0.01 | 0.02 | 0.02 | 0.02 |
| Rasamsonia$_{VIP\geq0.8\ (+)}$ | 0.48 | 1.02 | 0.81 | 0.87 | 1.16 | 0.52 | -0.01 | 0.02 | 0.01 | 0.01 | 0.02 | 0.00 |
| Pelodictyon$_{VIP\geq0.8\ (+)}$ | 0.56 | 0.52 | 1.29 | 1.77 | 1.05 | 1.09 | -0.01 | 0.01 | 0.01 | 0.02 | 0.02 | 0.01 |
| Methanofollis$_{VIP\geq0.8\ (+)}$ | 1.50 | 2.52 | 0.93 | 1.61 | 0.41 | 1.39 | -0.02 | 0.04 | 0.01 | 0.02 | -0.01 | 0.02 |
| Parascardovia$_{VIP\geq0.8\ (+)}$ | 0.77 | 1.22 | 0.83 | 0.78 | 1.42 | 1.51 | 0.01 | 0.02 | 0.00 | 0.01 | 0.03 | 0.02 |
| Nitratireductor$_{VIP\geq0.8\ (+)}$ | 0.89 | 0.10 | 1.40 | 1.43 | 1.13 | 0.61 | 0.01 | 0.00 | 0.01 | 0.02 | 0.02 | 0.00 |
| Bacteroides$_{VIP\geq0.8\ (+)}$ | 0.30 | 1.13 | 1.90 | 1.65 | 0.43 | 1.48 | 0.00 | 0.02 | 0.02 | 0.02 | 0.00 | 0.02 |
| Natrialba$_{VIP\geq0.8\ (+)}$ | 1.45 | 0.27 | 1.08 | 0.84 | 1.75 | 0.08 | 0.02 | 0.00 | 0.01 | 0.01 | 0.03 | 0.00 |
| Chryseobacterium$_{VIP\geq0.8\ (+)}$ | 0.99 | 0.86 | 1.83 | 1.85 | 0.61 | 1.55 | -0.01 | 0.01 | 0.02 | 0.02 | 0.01 | 0.02 |
| Thermotoga$_{VIP\geq0.8\ (+)}$ | 0.86 | 0.92 | 0.92 | 0.31 | 1.30 | 1.50 | 0.01 | 0.02 | 0.01 | 0.00 | 0.02 | 0.02 |
| Histoplasma$_{VIP\geq0.8\ (+)}$ | 1.31 | 0.77 | 0.82 | 0.72 | 0.94 | 0.81 | 0.01 | 0.01 | 0.00 | 0.00 | 0.01 | 0.01 |
| Clavispora$_{VIP\geq0.8\ (+)}$ | 0.82 | 1.49 | 0.84 | 0.73 | 0.86 | 0.83 | 0.01 | 0.02 | 0.01 | 0.01 | 0.00 | 0.00 |
| Dechloromonas$_{VIP\geq0.8\ (+)}$ | 0.96 | 0.88 | 0.61 | 1.22 | 0.09 | 1.90 | -0.01 | 0.02 | 0.01 | 0.01 | 0.00 | 0.02 |
| Cytophaga$_{VIP\geq0.8\ (+)}$ | 1.29 | 2.08 | 1.85 | 0.88 | 1.21 | 1.21 | -0.02 | 0.04 | 0.02 | 0.01 | 0.02 | 0.02 |
| Sulfitobacter$_{VIP\geq0.8\ (+)}$ | 1.00 | 2.37 | 0.34 | 1.20 | 0.85 | 1.78 | -0.01 | 0.04 | 0.00 | 0.01 | 0.02 | 0.02 |

| | | | | | | | | | | | | |
|---|---|---|---|---|---|---|---|---|---|---|---|---|
| Methanococcoides$_{VIP\geq0.8\ (+)}$ | 1.45 | 0.40 | 1.52 | 1.19 | 0.21 | 1.10 | -0.02 | 0.01 | 0.02 | 0.01 | 0.00 | 0.02 |
| Coccidioides$_{VIP\geq0.8\ (+)}$ | 1.06 | 0.87 | 0.83 | 0.69 | 1.01 | 0.77 | 0.01 | 0.01 | 0.00 | 0.00 | 0.01 | 0.00 |
| Proteiniclasticum$_{VIP\geq0.8\ (+)}$ | 0.68 | 1.91 | 0.86 | 1.32 | 0.89 | 1.06 | 0.00 | 0.03 | 0.00 | 0.01 | 0.01 | 0.01 |
| Olsenella$_{VIP\geq0.8\ (+)}$ | 0.88 | 0.97 | 1.35 | 1.46 | 0.77 | 1.31 | -0.01 | 0.02 | 0.01 | 0.01 | 0.01 | 0.02 |
| Faecalibaculum$_{VIP\geq0.8\ (+)}$ | 0.91 | 0.61 | 1.08 | 0.89 | 1.06 | 1.53 | -0.01 | 0.01 | 0.01 | 0.01 | 0.02 | 0.02 |
| Curtobacterium$_{VIP\geq0.8\ (+)}$ | 0.97 | 1.84 | 0.81 | 1.62 | 1.21 | 0.89 | -0.01 | 0.03 | 0.00 | 0.02 | 0.02 | 0.01 |
| Trypanosoma$_{VIP\geq0.8\ (+)}$ | 1.18 | 0.70 | 0.81 | 0.74 | 0.93 | 0.81 | 0.01 | 0.01 | 0.00 | 0.00 | 0.01 | 0.00 |
| Desulfobacterium$_{VIP\geq0.8\ (+)}$ | 0.92 | 0.92 | 0.86 | 1.76 | 1.08 | 1.89 | -0.01 | -0.01 | 0.01 | 0.02 | 0.02 | 0.02 |
| Methanohalophilus$_{VIP\geq0.8\ (+)}$ | 0.87 | 0.20 | 1.56 | 0.53 | 1.29 | 1.58 | -0.01 | 0.00 | 0.02 | 0.00 | 0.02 | 0.02 |
| Pelosinus$_{VIP\geq0.8\ (+)}$ | 1.22 | 0.38 | 1.99 | 1.06 | 0.44 | 1.02 | 0.02 | 0.00 | 0.02 | 0.01 | 0.00 | 0.01 |
| Thalassospira$_{VIP\geq0.8\ (+)}$ | 1.00 | 1.62 | 0.65 | 1.19 | 0.55 | 1.51 | -0.01 | 0.03 | 0.00 | 0.01 | 0.01 | 0.02 |
| Nostoc$_{VIP\geq0.8\ (+)}$ | 0.89 | 0.54 | 0.80 | 0.84 | 1.02 | 0.72 | 0.00 | -0.01 | 0.00 | 0.00 | 0.02 | 0.01 |
| Atopobium$_{VIP\geq0.8\ (+)}$ | 1.11 | 1.29 | 0.66 | 0.84 | 0.42 | 1.13 | 0.02 | 0.02 | 0.00 | 0.00 | 0.01 | 0.01 |
| Actinomyces$_{VIP\geq0.8\ (+)}$ | 0.97 | 1.28 | 0.78 | 1.11 | 0.98 | 0.74 | -0.01 | 0.02 | 0.00 | 0.01 | 0.02 | 0.01 |
| Sporothrix$_{VIP\geq0.8\ (+)}$ | 0.80 | 0.61 | 0.69 | 0.96 | 0.94 | 0.89 | 0.00 | 0.00 | 0.00 | 0.00 | 0.01 | 0.01 |
| Paenibacillus$_{VIP\geq0.8\ (+)}$ | 0.86 | 0.63 | 1.12 | 1.09 | 0.75 | 1.15 | 0.00 | 0.01 | 0.01 | 0.01 | 0.01 | 0.01 |
| Saccharomyces$_{VIP\geq0.8\ (+)}$ | 0.90 | 0.60 | 0.83 | 0.97 | 0.90 | 0.79 | 0.00 | -0.01 | 0.01 | 0.00 | 0.01 | 0.00 |
| Beauveria$_{VIP\geq0.8\ (+)}$ | 0.98 | 1.12 | 0.76 | 0.76 | 0.87 | 0.89 | 0.01 | -0.02 | 0.00 | 0.01 | 0.01 | 0.00 |
| Teredinibacter$_{VIP\geq0.8\ (+)}$ | 0.89 | 0.90 | 1.38 | 0.43 | 1.36 | 0.23 | 0.01 | 0.02 | 0.02 | 0.01 | -0.03 | 0.00 |
| Plasmodium$_{VIP\geq0.8\ (+)}$ | 0.96 | 0.54 | 0.83 | 0.88 | 0.87 | 0.84 | 0.00 | 0.00 | 0.00 | 0.00 | 0.01 | 0.00 |
| Phycomyces$_{VIP\geq0.8\ (+)}$ | 0.82 | 0.51 | 0.81 | 0.76 | 0.86 | 0.82 | 0.00 | 0.00 | 0.00 | 0.00 | 0.01 | 0.00 |
| Pelobacter$_{VIP\geq0.8\ (+)}$ | 1.67 | 0.93 | 0.77 | 1.19 | 0.47 | 1.20 | -0.02 | 0.02 | 0.00 | 0.01 | 0.00 | 0.02 |
| Jonquetella$_{VIP\geq0.8\ (+)}$ | 0.82 | 0.51 | 0.87 | 0.77 | 0.93 | 0.87 | 0.01 | 0.00 | 0.00 | -0.01 | 0.00 | 0.00 |
| Gemmatimonas$_{VIP\geq0.8\ (+)}$ | 1.29 | 0.11 | 1.26 | 0.96 | 0.55 | 1.63 | -0.02 | 0.00 | 0.01 | 0.01 | 0.01 | 0.02 |
| Malassezia$_{VIP\geq0.8\ (+)}$ | 1.08 | 1.80 | 0.48 | 1.03 | 0.87 | 0.82 | -0.02 | -0.03 | 0.00 | 0.01 | 0.02 | 0.00 |
| Aminomonas$_{VIP\geq0.8\ (+)}$ | 0.84 | 0.61 | 0.88 | 0.85 | 0.88 | 0.89 | 0.01 | 0.01 | 0.00 | 0.00 | 0.00 | -0.01 |
| Tolumonas$_{VIP\geq0.8\ (+)}$ | 0.83 | 0.57 | 0.83 | 0.82 | 1.00 | 0.84 | 0.01 | 0.00 | 0.00 | 0.00 | 0.01 | 0.00 |
| Salinicoccus$_{VIP\geq0.8\ (+)}$ | 0.78 | 1.56 | 1.11 | 0.98 | 0.62 | 1.34 | -0.01 | 0.03 | 0.01 | 0.01 | -0.01 | 0.01 |
| Allisonella$_{VIP\geq0.8\ (+)}$ | 0.84 | 1.87 | 0.70 | 0.92 | 0.84 | 1.17 | -0.01 | 0.03 | 0.00 | 0.00 | 0.01 | 0.01 |
| Rathayibacter$_{VIP\geq0.8\ (+)}$ | 1.20 | 0.33 | 1.10 | 1.42 | 0.45 | 0.88 | -0.01 | 0.00 | 0.01 | 0.01 | 0.00 | 0.01 |

| | | | | | | | | | | | | |
|---|---|---|---|---|---|---|---|---|---|---|---|---|
| Geoalkalibacter$_{VIP \geq 0.8\ (+)}$ | 0.84 | 0.59 | 0.92 | 0.78 | 0.91 | 1.07 | 0.00 | 0.00 | 0.00 | 0.00 | 0.01 | 0.00 |
| Syntrophobotulus$_{VIP \geq 0.8\ (+)}$ | 0.83 | 1.26 | 0.82 | 0.92 | 1.11 | 0.77 | 0.00 | -0.02 | 0.00 | 0.01 | 0.01 | 0.00 |
| Beijerinckia$_{VIP \geq 0.8\ (+)}$ | 1.85 | 1.45 | 0.96 | 1.15 | 0.75 | 1.24 | -0.02 | -0.02 | 0.01 | 0.01 | 0.01 | 0.02 |
| Desulfovibrio$_{VIP \geq 0.8\ (-)}$ | 0.85 | 0.60 | 0.84 | 0.77 | 0.88 | 1.16 | 0.00 | -0.01 | 0.00 | -0.01 | -0.01 | -0.02 |
| Cutaneotrichosporon$_{VIP \geq 0.8\ (-)}$ | 1.77 | 2.90 | 0.94 | 1.47 | 2.74 | 1.12 | -0.03 | -0.05 | -0.01 | -0.02 | -0.06 | 0.01 |
| Cryptobacterium$_{VIP \geq 0.8\ (-)}$ | 0.78 | 1.82 | 1.14 | 1.17 | 2.54 | 1.84 | 0.01 | -0.03 | -0.01 | -0.02 | -0.05 | -0.03 |
| Methyloversatilis$_{VIP \geq 0.8\ (-)}$ | 1.68 | 1.33 | 1.07 | 1.13 | 1.15 | 1.75 | -0.02 | -0.02 | -0.01 | -0.01 | -0.02 | -0.02 |
| Emiliania$_{VIP \geq 0.8\ (-)}$ | 1.71 | 3.13 | 1.03 | 0.96 | 2.19 | 0.82 | -0.03 | -0.05 | -0.01 | -0.01 | -0.04 | 0.01 |
| Gordonibacter$_{VIP \geq 0.8\ (-)}$ | 1.13 | 1.66 | 1.06 | 0.71 | 1.87 | 1.40 | 0.02 | -0.03 | -0.01 | -0.01 | -0.04 | -0.02 |
| Candidatus Carsonella$_{VIP \geq 0.8}$ | 0.58 | 1.85 | 1.30 | 1.72 | 0.78 | 1.21 | -0.01 | -0.03 | -0.01 | -0.02 | -0.01 | -0.02 |
| Eggerthella$_{VIP \geq 0.8\ (-)}$ | 1.12 | 1.75 | 1.08 | 0.69 | 1.72 | 1.31 | 0.02 | -0.03 | -0.01 | -0.01 | -0.03 | -0.02 |
| Acidovorax$_{VIP \geq 0.8\ (-)}$ | 0.75 | 0.67 | 1.07 | 1.02 | 1.07 | 0.84 | 0.01 | -0.01 | -0.01 | -0.01 | -0.02 | -0.01 |
| Methylococcus$_{VIP \geq 0.8\ (-)}$ | 0.65 | 0.60 | 0.94 | 1.21 | 1.14 | 1.00 | 0.01 | 0.00 | -0.01 | -0.02 | -0.02 | -0.01 |
| Paraburkholderia$_{VIP \geq 0.8\ (-)}$ | 0.83 | 0.87 | 1.13 | 1.05 | 0.73 | 0.87 | -0.01 | -0.01 | -0.01 | -0.01 | 0.00 | 0.00 |
| Methanocella$_{VIP \geq 0.8\ (-)}$ | 1.00 | 0.55 | 0.38 | 1.09 | 0.97 | 1.19 | -0.01 | 0.01 | 0.00 | -0.01 | -0.02 | -0.02 |
| Stigmatella$_{VIP \geq 0.8\ (-)}$ | 1.25 | 0.94 | 0.77 | 0.85 | 0.82 | 1.08 | -0.01 | -0.01 | 0.00 | -0.01 | -0.01 | -0.01 |
| Adlercreutzia$_{VIP \geq 0.8\ (-)}$ | 0.89 | 0.75 | 0.95 | 0.74 | 1.35 | 1.25 | 0.01 | -0.01 | -0.01 | 0.00 | -0.03 | -0.02 |
| Rhodoferax$_{VIP \geq 0.8\ (-)}$ | 0.97 | 0.49 | 0.87 | 0.72 | 0.92 | 1.60 | -0.01 | -0.01 | 0.00 | 0.00 | -0.01 | -0.02 |
| Fibrobacter$_{VIP \geq 0.8\ (-)}$ | 1.20 | 1.81 | 1.04 | 1.12 | 1.40 | 0.83 | -0.02 | -0.03 | -0.01 | -0.01 | -0.03 | 0.00 |
| Pseudoxanthomonas$_{VIP \geq 0.8\ (-}$ | 1.05 | 0.59 | 1.68 | 1.09 | 0.63 | 0.97 | 0.01 | 0.00 | -0.02 | -0.01 | 0.00 | -0.01 |
| Acaryochloris$_{VIP \geq 0.8\ (-)}$ | 0.82 | 1.46 | 0.85 | 0.99 | 1.05 | 0.93 | 0.01 | -0.02 | 0.00 | -0.01 | -0.01 | 0.00 |
| Pluralibacter$_{VIP \geq 0.8\ (-)}$ | 0.89 | 0.62 | 0.88 | 0.86 | 0.93 | 1.11 | 0.00 | 0.00 | -0.01 | -0.01 | 0.00 | -0.01 |
| Nitrobacter$_{VIP \geq 0.8\ (-)}$ | 1.10 | 0.62 | 1.23 | 0.82 | 0.92 | 0.98 | -0.01 | 0.00 | -0.01 | -0.01 | 0.00 | -0.01 |
| Hyphomonas$_{VIP \geq 0.8\ (-)}$ | 0.74 | 0.61 | 1.34 | 0.94 | 0.86 | 0.95 | 0.01 | 0.00 | -0.01 | -0.01 | 0.00 | 0.00 |
| Chelativorans$_{VIP \geq 0.8\ (-)}$ | 0.86 | 0.81 | 1.02 | 0.77 | 0.88 | 1.13 | 0.01 | -0.01 | -0.01 | -0.01 | -0.01 | -0.01 |
| Dermabacter$_{VIP \geq 0.8\ (-)}$ | 0.84 | 0.62 | 1.07 | 0.78 | 0.89 | 1.02 | 0.00 | 0.00 | -0.01 | -0.01 | 0.00 | -0.01 |
| Rhodobacter$_{VIP \geq 0.8\ (-)}$ | 0.85 | 0.85 | 1.05 | 0.80 | 0.89 | 0.98 | 0.01 | -0.01 | -0.01 | -0.01 | -0.01 | -0.01 |
| Azorhizobium$_{VIP \geq 0.8\ (-)}$ | 1.02 | 0.89 | 0.91 | 0.82 | 1.01 | 0.93 | -0.01 | -0.01 | -0.01 | -0.01 | 0.01 | 0.00 |
| Blastomonas$_{VIP \geq 0.8\ (-)}$ | 0.97 | 0.81 | 0.90 | 0.76 | 0.88 | 1.08 | 0.01 | -0.01 | -0.01 | -0.01 | -0.01 | -0.01 |
| Asticcacaulis$_{VIP \geq 0.8\ (-)}$ | 0.80 | 0.76 | 1.00 | 1.03 | 0.88 | 0.85 | 0.01 | -0.01 | -0.01 | -0.01 | 0.00 | -0.01 |

| | | | | | | | | | | | | |
|---|---|---|---|---|---|---|---|---|---|---|---|---|
| Sideroxydans$_{\text{VIP}\geq 0.8\ (-)}$ | 1.02 | 0.81 | 0.84 | 0.83 | 0.92 | 0.96 | -0.01 | -0.01 | 0.00 | -0.01 | 0.00 | -0.01 |
| Coraliomargarita$_{\text{VIP}\geq 0.8\ (-)}$ | 0.78 | 0.96 | 1.10 | 0.71 | 0.82 | 0.96 | 0.01 | -0.01 | -0.01 | 0.00 | -0.01 | -0.01 |
| Spirochaeta$_{\text{VIP}\geq 0.8\ (-)}$ | 0.81 | 0.66 | 0.86 | 0.72 | 0.92 | 0.86 | -0.01 | 0.00 | -0.01 | 0.00 | -0.01 | 0.00 |
| Sphingomonas$_{\text{VIP}\geq 0.8\ (-)}$ | 1.23 | 0.78 | 0.80 | 0.81 | 1.02 | 0.89 | -0.01 | -0.01 | 0.00 | 0.00 | -0.01 | 0.00 |
| Thermaerobacter$_{\text{VIP}\geq 0.8\ (-)}$ | 0.92 | 0.61 | 1.19 | 0.87 | 0.87 | 0.96 | 0.01 | 0.01 | -0.01 | -0.01 | -0.01 | -0.01 |
| Candidatus Methanoperede | 1.37 | 0.71 | 0.80 | 0.78 | 0.82 | 0.84 | -0.01 | -0.01 | 0.00 | -0.01 | 0.00 | 0.00 |
| Cupriavidus$_{\text{VIP}\geq 0.8\ (-)}$ | 1.05 | 0.59 | 0.87 | 0.79 | 0.81 | 1.02 | -0.01 | 0.00 | 0.00 | 0.00 | 0.00 | -0.01 |
| Thauera$_{\text{VIP}\geq 0.8\ (-)}$ | 0.99 | 0.57 | 1.12 | 0.81 | 0.89 | 0.93 | 0.00 | 0.00 | -0.01 | 0.00 | -0.01 | 0.00 |
| Starkeya$_{\text{VIP}\geq 0.8\ (-)}$ | 0.89 | 0.83 | 1.01 | 0.77 | 0.47 | 1.01 | 0.00 | -0.01 | -0.01 | 0.00 | 0.00 | -0.01 |
| Variovorax$_{\text{VIP}\geq 0.8\ (-)}$ | 0.90 | 0.60 | 0.85 | 0.79 | 0.98 | 1.01 | 0.00 | -0.01 | -0.01 | 0.00 | -0.01 | 0.00 |
| Defluviimonas$_{\text{VIP}\geq 0.8\ (-)}$ | 0.89 | 0.59 | 1.11 | 0.77 | 0.90 | 1.05 | 0.01 | 0.00 | -0.01 | -0.01 | 0.00 | -0.01 |
| Azoarcus$_{\text{VIP}\geq 0.8\ (-)}$ | 0.86 | 0.58 | 0.99 | 0.86 | 0.91 | 1.02 | 0.01 | 0.00 | -0.01 | -0.01 | 0.00 | -0.01 |
| Modestobacter$_{\text{VIP}\geq 0.8\ (-)}$ | 0.82 | 0.60 | 0.98 | 0.81 | 0.92 | 0.99 | 0.01 | 0.00 | -0.01 | -0.01 | 0.00 | -0.01 |
| Anaeromyxobacter$_{\text{VIP}\geq 0.8\ (-)}$ | 0.92 | 0.54 | 0.77 | 0.86 | 1.04 | 0.92 | 0.01 | 0.00 | -0.01 | -0.01 | -0.01 | -0.01 |
| Immundisolibacter$_{\text{VIP}\geq 0.8\ (-)}$ | 0.85 | 0.93 | 0.92 | 0.78 | 0.95 | 1.01 | 0.00 | -0.01 | -0.01 | 0.00 | 0.00 | -0.01 |
| Azospirillum$_{\text{VIP}\geq 0.8\ (-)}$ | 0.85 | 0.56 | 0.95 | 0.80 | 0.90 | 0.91 | 0.00 | 0.00 | -0.01 | -0.01 | 0.00 | -0.01 |
| Dyella$_{\text{VIP}\geq 0.8\ (-)}$ | 0.86 | 0.60 | 0.85 | 0.79 | 0.87 | 0.98 | 0.00 | 0.00 | 0.00 | 0.00 | 0.00 | -0.01 |
| Ralstonia$_{\text{VIP}\geq 0.8\ (-)}$ | 0.85 | 0.58 | 0.98 | 0.83 | 0.91 | 0.99 | 0.01 | 0.00 | -0.01 | -0.01 | 0.00 | 0.00 |
| Cyanobium$_{\text{VIP}\geq 0.8\ (-)}$ | 0.85 | 0.51 | 1.14 | 0.81 | 0.87 | 0.94 | 0.01 | 0.00 | -0.01 | -0.01 | 0.00 | -0.01 |
| Klebsiella$_{\text{VIP}\geq 0.8\ (-)}$ | 1.11 | 0.56 | 0.81 | 0.91 | 0.79 | 0.99 | -0.01 | 0.00 | 0.00 | -0.01 | 0.00 | 0.00 |
| Halorhodospira$_{\text{VIP}\geq 0.8\ (-)}$ | 0.83 | 0.66 | 0.85 | 0.79 | 0.89 | 0.96 | 0.00 | -0.01 | 0.00 | -0.01 | 0.00 | 0.00 |
| Mesorhizobium$_{\text{VIP}\geq 0.8\ (-)}$ | 0.88 | 0.76 | 1.09 | 0.80 | 0.92 | 0.98 | 0.00 | -0.01 | -0.01 | -0.01 | 0.00 | 0.00 |
| Candidatus Koribacter$_{\text{VIP}\geq 0.8}$ | 0.79 | 0.93 | 0.98 | 0.80 | 0.75 | 1.03 | 0.01 | -0.01 | -0.01 | -0.01 | -0.01 | 0.01 |
| Sinomonas$_{\text{VIP}\geq 0.8\ (-)}$ | 1.02 | 0.62 | 1.21 | 0.84 | 0.80 | 0.92 | 0.01 | 0.00 | -0.01 | -0.01 | 0.00 | 0.00 |
| Geobacter$_{\text{VIP}\geq 0.8\ (-)}$ | 0.90 | 0.59 | 0.83 | 0.79 | 0.90 | 0.95 | 0.00 | 0.00 | 0.00 | 0.00 | 0.00 | 0.00 |
| Thermosipho$_{\text{VIP}\geq 0.8\ (-)}$ | 0.65 | 1.26 | 1.81 | 1.10 | 0.92 | 0.71 | -0.01 | 0.02 | -0.02 | -0.01 | -0.02 | -0.01 |
| Aeromicrobium$_{\text{VIP}\geq 0.8\ (-)}$ | 1.10 | 0.60 | 0.82 | 0.80 | 0.85 | 0.89 | -0.01 | 0.01 | 0.00 | -0.01 | 0.00 | -0.01 |
| Raoultella$_{\text{VIP}\geq 0.8\ (-)}$ | 1.01 | 0.55 | 1.30 | 0.69 | 0.82 | 0.91 | 0.01 | 0.00 | -0.01 | 0.00 | -0.01 | 0.00 |
| Halolamina$_{\text{VIP}\geq 0.8\ (-)}$ | 0.99 | 0.55 | 0.86 | 0.80 | 0.89 | 1.04 | 0.01 | 0.00 | -0.01 | -0.01 | 0.00 | -0.01 |
| Chlorobium$_{\text{VIP}\geq 0.8\ (-)}$ | 0.82 | 0.54 | 0.85 | 0.79 | 0.87 | 0.89 | 0.00 | 0.00 | -0.01 | -0.01 | -0.01 | -0.01 |

| | | | | | | | | | | | | |
|---|---|---|---|---|---|---|---|---|---|---|---|---|
| Fusarium$_{VIP\geq0.8\ (-)}$ | 0.95 | 0.72 | 1.16 | 0.89 | 0.88 | 1.02 | -0.01 | -0.01 | -0.01 | -0.01 | -0.01 | -0.01 |
| Chthonomonas$_{VIP\geq0.8\ (-)}$ | 1.08 | 0.76 | 0.79 | 0.93 | 0.80 | 0.93 | 0.01 | -0.01 | 0.00 | -0.01 | 0.00 | 0.00 |
| Frischella$_{VIP\geq0.8\ (-)}$ | 0.86 | 0.66 | 0.82 | 0.73 | 0.87 | 0.95 | 0.01 | 0.00 | 0.00 | 0.00 | 0.00 | -0.01 |
| Salinispira$_{VIP\geq0.8\ (-)}$ | 0.79 | 0.59 | 0.85 | 0.87 | 1.13 | 0.97 | 0.00 | 0.00 | -0.01 | -0.01 | 0.01 | -0.01 |
| Colletotrichum$_{VIP\geq0.8\ (-)}$ | 1.07 | 1.57 | 0.61 | 1.34 | 1.17 | 0.88 | -0.01 | -0.03 | 0.00 | -0.01 | -0.02 | 0.00 |
| Hydrogenophaga$_{VIP\geq0.8\ (-)}$ | 0.85 | 0.56 | 1.21 | 0.74 | 0.88 | 0.84 | 0.01 | 0.00 | -0.01 | 0.00 | 0.00 | 0.00 |
| Porphyrobacter$_{VIP\geq0.8\ (-)}$ | 0.95 | 0.64 | 0.93 | 0.85 | 0.99 | 0.96 | 0.01 | 0.00 | -0.01 | -0.01 | 0.01 | -0.01 |
| Wenzhouxiangella$_{VIP\geq0.8\ (-)}$ | 0.86 | 0.57 | 0.89 | 0.79 | 0.94 | 0.94 | 0.01 | 0.00 | -0.01 | -0.01 | 0.00 | 0.00 |
| Campylobacter$_{VIP\geq0.8\ (-)}$ | 0.83 | 0.45 | 0.91 | 1.30 | 1.75 | 1.02 | 0.01 | -0.01 | -0.01 | -0.01 | -0.03 | -0.01 |
| Nitrosospira$_{VIP\geq0.8\ (-)}$ | 0.82 | 0.64 | 0.83 | 0.77 | 0.97 | 0.92 | 0.00 | -0.01 | 0.00 | -0.01 | 0.00 | 0.00 |
| Sphingopyxis$_{VIP\geq0.8\ (-)}$ | 0.79 | 0.62 | 0.85 | 0.80 | 0.89 | 0.95 | 0.00 | 0.00 | 0.00 | 0.00 | 0.00 | 0.00 |
| Microbulbifer$_{VIP\geq0.8\ (-)}$ | 0.92 | 0.58 | 0.94 | 0.78 | 0.94 | 0.98 | 0.01 | 0.00 | -0.01 | -0.01 | 0.00 | 0.00 |
| Sedimenticola$_{VIP\geq0.8\ (-)}$ | 1.08 | 0.82 | 0.84 | 0.77 | 1.12 | 1.02 | -0.01 | -0.01 | 0.00 | 0.00 | 0.01 | 0.00 |
| Leptosphaeria$_{VIP\geq0.8\ (-)}$ | 1.10 | 0.92 | 1.04 | 0.68 | 1.05 | 0.76 | -0.01 | -0.02 | -0.01 | 0.00 | -0.02 | -0.01 |
| Pantoea$_{VIP\geq0.8\ (-)}$ | 0.84 | 0.52 | 0.86 | 0.77 | 0.94 | 0.96 | 0.00 | 0.00 | -0.01 | 0.00 | 0.00 | 0.00 |
| Tsukamurella$_{VIP\geq0.8\ (-)}$ | 0.92 | 0.70 | 0.85 | 0.77 | 0.88 | 0.96 | 0.01 | -0.01 | -0.01 | 0.00 | 0.00 | -0.01 |
| Methylomonas$_{VIP\geq0.8\ (-)}$ | 0.81 | 0.68 | 0.83 | 0.76 | 0.92 | 1.01 | 0.00 | -0.01 | 0.00 | -0.01 | 0.00 | 0.00 |
| Dermacoccus$_{VIP\geq0.8\ (-)}$ | 0.80 | 0.87 | 1.06 | 0.27 | 1.52 | 0.34 | -0.01 | -0.02 | 0.01 | 0.00 | -0.03 | 0.00 |
| Corynebacterium$_{VIP\geq0.8\ (-)}$ | 0.90 | 1.31 | 0.80 | 1.16 | 0.83 | 0.83 | 0.00 | -0.02 | 0.00 | 0.01 | 0.00 | -0.01 |
| Halotalea$_{VIP\geq0.8\ (-)}$ | 1.01 | 0.70 | 0.95 | 0.76 | 0.90 | 0.97 | 0.01 | -0.01 | -0.01 | 0.00 | 0.00 | 0.00 |
| Burkholderia$_{VIP\geq0.8\ (-)}$ | 0.92 | 0.51 | 0.82 | 0.83 | 0.85 | 0.91 | 0.00 | 0.00 | 0.00 | 0.00 | 0.00 | 0.00 |
| Pseudomonas$_{VIP\geq0.8\ (-)}$ | 0.99 | 0.59 | 0.87 | 0.78 | 0.90 | 0.98 | 0.01 | 0.00 | 0.00 | 0.00 | 0.00 | 0.00 |
| Altererythrobacter$_{VIP\geq0.8\ (-)}$ | 0.93 | 0.38 | 0.91 | 0.78 | 0.88 | 1.01 | 0.01 | 0.00 | -0.01 | -0.01 | -0.01 | 0.01 |
| Edwardsiella$_{VIP\geq0.8\ (-)}$ | 0.90 | 0.58 | 0.86 | 0.80 | 0.85 | 0.95 | 0.00 | 0.00 | 0.00 | 0.00 | 0.00 | 0.00 |
| Gloeobacter$_{VIP\geq0.8\ (-)}$ | 0.82 | 0.81 | 0.80 | 0.79 | 0.84 | 0.91 | 0.01 | -0.01 | 0.00 | 0.00 | 0.00 | 0.00 |
| Aeromonas$_{VIP\geq0.8\ (-)}$ | 1.01 | 0.56 | 0.87 | 0.78 | 0.92 | 0.97 | 0.01 | 0.00 | 0.00 | -0.01 | 0.00 | 0.00 |
| Archangium$_{VIP\geq0.8\ (-)}$ | 1.09 | 0.69 | 0.85 | 0.94 | 0.92 | 0.72 | 0.01 | 0.00 | -0.01 | 0.00 | -0.01 | -0.01 |
| Thalassiosira$_{VIP\geq0.8\ (-)}$ | 1.03 | 1.13 | 0.90 | 1.37 | 0.84 | 0.71 | -0.02 | 0.02 | 0.00 | 0.02 | 0.00 | -0.01 |
| Planktothrix$_{VIP\geq0.8\ (-)}$ | 0.86 | 1.38 | 0.85 | 1.86 | 0.91 | 1.09 | 0.00 | -0.02 | 0.00 | -0.02 | -0.02 | 0.01 |
| Candidatus Paracaedibacter | 2.58 | 1.38 | 1.36 | 1.66 | 0.51 | 0.62 | 0.03 | -0.02 | -0.01 | -0.02 | 0.01 | 0.00 |

| | | | | | | | | | | | | |
|---|---|---|---|---|---|---|---|---|---|---|---|---|
| Enterobacter$_{VIP≥0.8\ (-)}$ | 1.28 | 0.49 | 0.87 | 0.74 | 0.85 | 0.91 | 0.02 | 0.00 | -0.01 | 0.00 | 0.00 | 0.00 |
| Phialocephala$_{VIP≥0.8\ (-)}$ | 1.23 | 1.19 | 0.88 | 1.20 | 1.10 | 0.60 | -0.02 | -0.02 | 0.00 | -0.01 | -0.01 | 0.00 |
| Sanguibacter$_{VIP≥0.8\ (-)}$ | 1.14 | 0.66 | 0.93 | 0.78 | 0.93 | 0.96 | 0.02 | 0.00 | -0.01 | 0.00 | 0.00 | 0.00 |
| Spizellomyces$_{VIP≥0.8\ (-)}$ | 0.88 | 0.60 | 1.02 | 1.11 | 0.94 | 0.83 | -0.01 | -0.01 | -0.01 | -0.01 | -0.01 | -0.01 |
| Sugiyamaella$_{VIP≥0.8\ (-)}$ | 1.05 | 1.42 | 1.03 | 1.08 | 0.85 | 1.04 | -0.01 | -0.02 | -0.01 | -0.01 | 0.00 | -0.01 |
| Halomonas$_{VIP≥0.8\ (-)}$ | 0.92 | 0.56 | 0.81 | 0.75 | 0.95 | 0.99 | 0.01 | 0.00 | 0.00 | 0.00 | 0.00 | 0.00 |
| Podospora$_{VIP≥0.8\ (-)}$ | 1.05 | 1.04 | 0.85 | 1.08 | 0.95 | 1.31 | -0.02 | -0.02 | 0.00 | -0.01 | -0.01 | -0.01 |
| Candidatus Phytoplasma$_{VIP≥}$ | 0.82 | 0.34 | 1.07 | 1.36 | 0.93 | 1.16 | 0.00 | 0.00 | -0.01 | -0.01 | -0.01 | -0.01 |
| Arthrobacter$_{VIP≥0.8\ (-)}$ | 1.01 | 0.51 | 0.87 | 0.79 | 0.83 | 0.95 | 0.01 | 0.00 | 0.00 | 0.00 | 0.00 | 0.00 |
| Cronobacter$_{VIP≥0.8\ (-)}$ | 1.03 | 0.50 | 0.87 | 0.78 | 1.00 | 0.97 | 0.01 | 0.00 | 0.00 | 0.00 | 0.01 | 0.00 |
| Lodderomyces$_{VIP≥0.8\ (-)}$ | 1.01 | 0.50 | 0.91 | 0.91 | 0.87 | 0.90 | 0.00 | 0.00 | 0.00 | 0.00 | 0.01 | 0.00 |
| Pneumocystis$_{VIP≥0.8\ (-)}$ | 0.81 | 0.57 | 1.21 | 1.41 | 0.85 | 0.78 | -0.01 | -0.01 | -0.01 | -0.01 | -0.01 | 0.00 |
| Tuber$_{VIP≥0.8\ (-)}$ | 0.87 | 0.45 | 0.87 | 1.00 | 1.00 | 0.87 | 0.00 | 0.00 | 0.00 | -0.01 | 0.01 | 0.00 |
| Paracoccidioides$_{VIP≥0.8\ (-)}$ | 0.90 | 0.80 | 1.06 | 1.13 | 0.83 | 0.86 | -0.01 | -0.01 | -0.01 | -0.01 | 0.00 | 0.00 |
| Kwoniella$_{VIP≥0.8\ (-)}$ | 0.87 | 1.00 | 0.90 | 1.11 | 0.74 | 0.91 | -0.01 | -0.02 | 0.00 | -0.01 | 0.00 | -0.01 |
| Nosema$_{VIP≥0.8\ (-)}$ | 0.81 | 0.46 | 1.26 | 1.38 | 0.78 | 0.86 | 0.00 | 0.00 | -0.01 | -0.01 | 0.00 | 0.00 |
| Vanderwaltozyma$_{VIP≥0.8\ (-)}$ | 0.86 | 0.51 | 0.76 | 0.87 | 0.83 | 0.82 | 0.00 | 0.00 | 0.00 | 0.00 | 0.00 | 0.00 |
| Luteipulveratus$_{VIP≥0.8\ (-)}$ | 1.22 | 0.73 | 1.16 | 0.93 | 0.41 | 1.15 | -0.01 | -0.01 | -0.01 | 0.01 | 0.01 | -0.02 |
| Enterocytozoon$_{VIP≥0.8\ (-)}$ | 1.01 | 0.28 | 0.95 | 0.83 | 0.68 | 1.76 | 0.01 | 0.00 | -0.01 | 0.00 | 0.00 | -0.02 |
| Candida$_{VIP≥0.8\ (-)}$ | 0.89 | 0.47 | 0.95 | 1.02 | 0.83 | 0.87 | 0.00 | 0.00 | 0.00 | 0.00 | 0.00 | 0.00 |
| Penicillium$_{VIP≥0.8\ (-)}$ | 1.04 | 1.16 | 0.85 | 0.68 | 0.91 | 0.74 | -0.01 | -0.02 | 0.00 | 0.01 | 0.01 | 0.00 |
| Zygosaccharomyces$_{VIP≥0.8\ (-)}$ | 1.01 | 0.70 | 0.98 | 0.89 | 0.79 | 0.81 | 0.00 | -0.01 | 0.00 | 0.00 | 0.00 | 0.00 |
| Naegleria$_{VIP≥0.8\ (-)}$ | 0.87 | 0.51 | 1.16 | 1.34 | 0.85 | 0.82 | 0.00 | 0.00 | -0.01 | -0.01 | 0.00 | 0.00 |
| Methanocaldococcus$_{VIP≥0.8\ (-)}$ | 0.71 | 1.72 | 0.84 | 0.99 | 0.95 | 0.81 | -0.01 | -0.03 | 0.00 | 0.00 | -0.01 | 0.00 |
| Methanotorris$_{VIP≥0.8\ (-)}$ | 0.88 | 0.96 | 1.12 | 1.42 | 0.86 | 0.40 | -0.01 | 0.01 | -0.01 | -0.01 | 0.00 | 0.00 |
| Pichia$_{VIP≥0.8\ (-)}$ | 0.81 | 0.62 | 1.04 | 1.01 | 0.85 | 1.00 | 0.00 | -0.01 | -0.01 | 0.00 | -0.01 | -0.01 |
| Rhinocladiella$_{VIP≥0.8\ (-)}$ | 0.95 | 0.60 | 0.86 | 0.85 | 1.01 | 0.81 | -0.01 | -0.01 | 0.00 | 0.00 | -0.01 | 0.00 |
| Dictyostelium$_{VIP≥0.8\ (-)}$ | 0.86 | 0.51 | 1.06 | 1.15 | 0.81 | 0.86 | 0.00 | 0.00 | -0.01 | -0.01 | 0.00 | 0.00 |
| Blastocystis$_{VIP≥0.8\ (-)}$ | 0.96 | 0.66 | 0.97 | 1.10 | 0.83 | 0.79 | -0.01 | -0.01 | 0.00 | -0.01 | 0.01 | 0.00 |
| Ichthyophthirius$_{VIP≥0.8\ (-)}$ | 0.86 | 0.55 | 1.00 | 1.23 | 0.81 | 0.90 | 0.00 | -0.01 | 0.00 | -0.01 | 0.00 | 0.00 |

| | | | | | | | | | | | | |
|---|---|---|---|---|---|---|---|---|---|---|---|---|
| Spathaspora$_{VIP\geq0.8\ (-)}$ | 0.90 | 0.87 | 1.17 | 1.33 | 0.79 | 0.80 | 0.00 | 0.01 | -0.01 | -0.01 | 0.00 | 0.00 |
| Cyanothece$_{VIP\geq0.8\ (-)}$ | 1.29 | 0.45 | 0.82 | 1.27 | 0.61 | 1.07 | 0.01 | 0.00 | 0.00 | -0.01 | 0.00 | -0.01 |
| Cryptosporidium$_{VIP\geq0.8\ (-)}$ | 0.86 | 0.51 | 1.08 | 1.07 | 0.78 | 0.82 | -0.01 | 0.00 | -0.01 | -0.01 | 0.00 | 0.00 |
| Salegentibacter$_{VIP\geq0.8\ (-)}$ | 1.20 | 1.55 | 1.33 | 0.48 | 1.08 | 0.32 | -0.02 | 0.03 | 0.01 | 0.00 | -0.02 | 0.00 |
| Phaeoacremonium$_{VIP\geq0.8\ (-)}$ | 1.29 | 0.45 | 0.85 | 0.81 | 0.79 | 1.00 | -0.02 | 0.00 | 0.00 | 0.00 | 0.00 | -0.01 |
| Tetrahymena$_{VIP\geq0.8\ (-)}$ | 0.87 | 0.59 | 1.13 | 1.28 | 0.77 | 0.87 | 0.00 | 0.00 | -0.01 | -0.01 | 0.00 | 0.00 |
| Arthrobotrys$_{VIP\geq0.8\ (-)}$ | 0.85 | 0.54 | 0.98 | 1.09 | 0.81 | 0.80 | 0.00 | -0.01 | 0.00 | -0.01 | 0.00 | 0.00 |
| Dactylellina$_{VIP\geq0.8\ (-)}$ | 0.85 | 0.48 | 1.10 | 0.95 | 0.81 | 0.89 | -0.01 | 0.00 | -0.01 | 0.00 | 0.00 | 0.00 |
| Acytostelium$_{VIP\geq0.8\ (-)}$ | 0.74 | 0.35 | 0.90 | 0.86 | 0.83 | 0.93 | 0.00 | 0.00 | 0.00 | 0.00 | 0.00 | -0.01 |
| Mesoplasma$_{VIP\geq0.8\ (-)}$ | 0.91 | 1.09 | 0.70 | 1.47 | 1.27 | 0.22 | 0.01 | 0.02 | 0.00 | -0.01 | -0.02 | 0.00 |
| Tetrapisispora$_{VIP\geq0.8\ (-)}$ | 0.81 | 0.77 | 0.86 | 1.09 | 0.85 | 0.81 | 0.00 | -0.01 | 0.00 | -0.01 | 0.00 | 0.00 |
| Alkalilimnicola$_{VIP\geq0.8}$ | 0.83 | 0.55 | 0.86 | 0.87 | 1.00 | 0.93 | 0.00 | 0.00 | 0.00 | -0.01 | 0.01 | 0.00 |
| Chromobacterium$_{VIP\geq0.8}$ | 0.90 | 0.60 | 1.00 | 0.92 | 0.99 | 1.00 | 0.01 | 0.00 | -0.01 | -0.01 | 0.01 | 0.00 |
| Desulfurispirillum$_{VIP\geq0.8}$ | 0.84 | 0.59 | 1.01 | 0.82 | 1.05 | 0.82 | 0.00 | 0.00 | -0.01 | -0.01 | 0.01 | 0.00 |
| Gordonia$_{VIP\geq0.8}$ | 0.87 | 0.55 | 0.95 | 0.81 | 0.93 | 0.98 | 0.01 | 0.00 | -0.01 | 0.00 | 0.01 | -0.01 |
| Methanomassiliicoccus$_{VIP\geq0.8}$ | 0.81 | 0.83 | 0.83 | 0.79 | 0.83 | 0.96 | 0.01 | 0.01 | -0.01 | -0.01 | 0.00 | -0.01 |
| Pannonibacter$_{VIP\geq0.8}$ | 0.88 | 0.76 | 1.20 | 0.81 | 0.90 | 1.05 | 0.01 | -0.01 | -0.01 | -0.01 | 0.00 | 0.00 |
| Rhizobium$_{VIP\geq0.8}$ | 0.91 | 0.51 | 0.86 | 0.82 | 0.86 | 0.99 | 0.01 | 0.00 | 0.00 | 0.00 | 0.00 | 0.00 |
| Sulfurovum$_{VIP\geq0.8}$ | 0.70 | 1.18 | 1.36 | 0.87 | 0.92 | 0.83 | -0.01 | -0.02 | 0.01 | 0.01 | -0.01 | 0.01 |
| Thermothelomyces$_{VIP\geq0.8}$ | 1.41 | 1.12 | 0.98 | 0.67 | 0.97 | 0.97 | -0.02 | -0.02 | 0.01 | 0.00 | 0.01 | 0.00 |
| Actinoplanes$_{VIP\geq0.8}$ | 1.20 | 0.52 | 0.84 | 0.72 | 1.17 | 0.81 | 0.02 | 0.00 | 0.00 | 0.00 | 0.02 | 0.00 |
| Agrobacterium$_{VIP\geq0.8}$ | 0.96 | 0.57 | 0.84 | 0.79 | 0.93 | 1.00 | 0.01 | 0.00 | 0.00 | 0.00 | 0.00 | 0.00 |
| Bordetella$_{VIP\geq0.8}$ | 0.93 | 0.70 | 0.83 | 0.77 | 0.80 | 0.96 | 0.01 | 0.01 | -0.01 | 0.00 | 0.00 | 0.00 |
| Bradyrhizobium$_{VIP\geq0.8}$ | 0.85 | 0.45 | 0.84 | 0.77 | 0.91 | 0.94 | 0.01 | 0.00 | 0.00 | 0.00 | 0.01 | -0.01 |
| Cedecea$_{VIP\geq0.8}$ | 1.00 | 0.77 | 0.94 | 0.70 | 0.83 | 0.91 | 0.01 | -0.01 | -0.01 | -0.01 | 0.00 | 0.00 |
| Dokdonella$_{VIP\geq0.8}$ | 0.83 | 0.57 | 1.06 | 0.80 | 0.92 | 0.99 | 0.01 | 0.00 | -0.01 | -0.01 | 0.00 | -0.01 |
| Gregarina$_{VIP\geq0.8}$ | 0.86 | 1.16 | 0.77 | 1.16 | 0.62 | 1.00 | 0.00 | -0.02 | 0.00 | -0.01 | 0.00 | 0.01 |
| Melampsora$_{VIP\geq0.8}$ | 0.93 | 0.53 | 0.89 | 0.88 | 0.93 | 0.79 | 0.00 | 0.00 | 0.00 | 0.00 | -0.01 | 0.01 |
| Methyloceanibacter$_{VIP\geq0.8}$ | 1.16 | 0.55 | 1.11 | 0.78 | 0.80 | 0.91 | 0.02 | 0.00 | -0.01 | 0.00 | 0.00 | 0.00 |
| Novosphingobium$_{VIP\geq0.8}$ | 1.67 | 1.02 | 0.67 | 0.92 | 0.47 | 1.08 | -0.02 | -0.02 | 0.00 | 0.01 | 0.00 | 0.01 |

| | | | | | | | | | | | | |
|---|---|---|---|---|---|---|---|---|---|---|---|---|
| Schizosaccharomyces$_{VIP\geq0.8}$ | 0.82 | 0.61 | 0.94 | 0.73 | 0.81 | 0.88 | 0.00 | 0.00 | 0.00 | 0.00 | 0.01 | -0.01 |
| Shimwellia$_{VIP\geq0.8}$ | 1.14 | 0.62 | 0.83 | 0.75 | 0.91 | 0.93 | 0.02 | 0.01 | -0.01 | 0.00 | -0.01 | 0.00 |
| Sinorhizobium$_{VIP\geq0.8}$ | 1.36 | 0.53 | 0.93 | 0.78 | 0.82 | 0.91 | -0.01 | 0.00 | -0.01 | 0.00 | 0.00 | 0.00 |
| Thioalkalivibrio$_{VIP\geq0.8}$ | 0.87 | 0.50 | 0.85 | 0.79 | 0.95 | 1.00 | 0.01 | 0.00 | 0.00 | 0.00 | 0.00 | -0.01 |
| Thiobacillus$_{VIP\geq0.8}$ | 0.73 | 0.52 | 1.07 | 0.97 | 1.04 | 1.07 | 0.00 | 0.00 | -0.01 | -0.01 | 0.01 | -0.01 |
| Thioflavicoccus$_{VIP\geq0.8}$ | 0.70 | 0.53 | 0.87 | 0.84 | 0.93 | 0.83 | 0.00 | 0.01 | -0.01 | -0.01 | 0.01 | 0.00 |
| Trichormus$_{VIP\geq0.8}$ | 1.03 | 1.05 | 0.82 | 0.80 | 1.02 | 0.74 | 0.01 | -0.02 | 0.00 | 0.00 | 0.01 | 0.00 |
| Wenyingzhuangia$_{VIP\geq0.8}$ | 1.36 | 1.76 | 0.31 | 1.75 | 0.45 | 1.74 | 0.02 | 0.03 | 0.00 | -0.02 | 0.01 | -0.02 |
| *Acidothermus* | 0.97 | 0.58 | 0.78 | 0.87 | 0.97 | 0.67 | -0.01 | 0.00 | -0.01 | -0.01 | -0.01 | 0.00 |
| *Agaricus* | 0.84 | 0.56 | 0.61 | 0.70 | 0.94 | 1.08 | -0.01 | 0.01 | 0.01 | 0.00 | -0.01 | 0.01 |
| *Agromyces* | 0.76 | 0.49 | 0.84 | 0.87 | 0.45 | 1.25 | 0.00 | -0.01 | 0.00 | 0.00 | 0.00 | 0.01 |
| *Alicyclobacillus* | 0.75 | 0.93 | 0.76 | 0.73 | 1.01 | 0.83 | 0.00 | -0.01 | 0.00 | 0.00 | 0.01 | 0.00 |
| *Alteromonas* | 0.72 | 1.00 | 1.44 | 0.63 | 0.72 | 0.96 | 0.01 | 0.02 | 0.01 | 0.00 | -0.01 | 0.01 |
| *Aromatoleum* | 0.75 | 0.57 | 1.02 | 0.79 | 1.03 | 0.91 | 0.00 | 0.00 | -0.01 | -0.01 | 0.01 | 0.00 |
| *Aspergillus* | 0.81 | 1.01 | 0.70 | 0.69 | 0.76 | 0.82 | 0.00 | 0.01 | 0.00 | 0.00 | 0.00 | 0.00 |
| *Azospira* | 0.74 | 0.65 | 1.10 | 0.78 | 0.92 | 1.05 | 0.01 | 0.00 | -0.01 | -0.01 | -0.01 | -0.01 |
| *Botrytis* | 0.94 | 0.47 | 0.73 | 0.71 | 0.87 | 0.98 | -0.01 | 0.00 | 0.00 | 0.00 | 0.01 | 0.01 |
| *Calothrix* | 0.84 | 0.52 | 0.81 | 0.77 | 1.25 | 0.75 | 0.00 | 0.00 | 0.00 | 0.00 | 0.02 | 0.01 |
| *Candidatus Amoebophilus* | 0.90 | 0.97 | 0.65 | 0.67 | 0.75 | 1.50 | 0.01 | 0.01 | 0.00 | 0.00 | -0.02 | -0.02 |
| *Candidatus Ishikawaella* | 0.80 | 0.69 | 0.80 | 0.73 | 0.84 | 0.92 | 0.01 | -0.01 | 0.00 | 0.00 | 0.00 | -0.01 |
| *Candidatus Profftella* | 0.75 | 0.63 | 1.17 | 1.53 | 0.81 | 0.36 | 0.00 | 0.01 | -0.01 | -0.01 | -0.01 | 0.00 |
| *Candidatus* | 0.84 | 0.93 | 0.95 | 0.69 | 0.55 | 0.55 | 0.01 | 0.02 | 0.00 | 0.00 | 0.00 | 0.00 |
| *Castellaniella* | 0.79 | 0.68 | 0.90 | 0.80 | 0.92 | 1.03 | 0.01 | -0.01 | -0.01 | -0.01 | 0.00 | -0.01 |
| *Chelatococcus* | 0.94 | 0.54 | 0.80 | 0.73 | 0.75 | 0.96 | 0.01 | 0.00 | -0.01 | 0.00 | 0.01 | 0.00 |
| *Chlorobaculum* | 0.88 | 0.58 | 0.92 | 0.75 | 0.90 | 0.74 | 0.00 | -0.01 | -0.01 | 0.00 | 0.00 | 0.00 |
| *Clavibacter* | 0.85 | 0.64 | 0.74 | 1.02 | 0.70 | 0.89 | 0.00 | 0.01 | 0.00 | 0.00 | 0.01 | 0.00 |
| *Cloacibacillus* | 0.78 | 1.10 | 0.71 | 0.66 | 1.04 | 0.95 | 0.00 | -0.02 | 0.00 | 0.00 | 0.01 | 0.01 |
| *Cordyceps* | 0.89 | 0.62 | 0.85 | 0.72 | 0.85 | 0.75 | -0.01 | -0.01 | 0.00 | 0.00 | 0.00 | 0.00 |
| *Coriobacterium* | 0.96 | 0.76 | 0.76 | 0.87 | 0.48 | 1.38 | 0.01 | 0.01 | 0.00 | 0.00 | 0.00 | 0.02 |
| *Debaryomyces* | 1.08 | 0.55 | 0.83 | 0.77 | 0.98 | 0.61 | -0.01 | 0.00 | 0.00 | 0.00 | -0.01 | 0.00 |
| *Desulfitobacterium* | 0.76 | 0.48 | 1.17 | 0.90 | 0.70 | 1.11 | 0.01 | 0.01 | 0.01 | 0.01 | 0.01 | 0.01 |
| *Dickeya* | 1.01 | 0.55 | 0.79 | 0.77 | 0.89 | 0.94 | -0.01 | 0.00 | 0.00 | 0.00 | -0.01 | 0.00 |
| *Eimeria* | 0.80 | 0.59 | 0.74 | 0.74 | 0.83 | 1.29 | 0.01 | 0.01 | 0.00 | 0.00 | 0.00 | 0.02 |
| *Entamoeba* | 0.75 | 0.52 | 1.03 | 0.97 | 0.76 | 0.83 | 0.00 | -0.01 | -0.01 | 0.00 | 0.00 | 0.00 |

| | | | | | | | | | | | | |
|---|---|---|---|---|---|---|---|---|---|---|---|---|
| *Eutypa* | 0.78 | 0.46 | 1.07 | 0.97 | 0.80 | 0.92 | -0.01 | 0.00 | -0.01 | 0.00 | 0.00 | -0.01 |
| *Fomitiporia* | 0.99 | 1.00 | 0.77 | 0.78 | 1.02 | 0.79 | 0.00 | -0.02 | 0.00 | 0.00 | 0.01 | 0.00 |
| *Frankia* | 0.75 | 0.57 | 0.95 | 0.95 | 0.82 | 0.70 | 0.00 | 0.01 | -0.01 | 0.00 | -0.01 | -0.01 |
| *Halocynthiibacter* | 1.28 | 0.54 | 1.10 | 0.68 | 0.78 | 0.92 | -0.02 | 0.01 | 0.01 | -0.01 | 0.01 | 0.01 |
| *Herbaspirillum* | 0.78 | 0.53 | 0.84 | 0.78 | 0.87 | 1.01 | 0.00 | 0.00 | 0.00 | 0.00 | 0.00 | 0.00 |
| *Isaria* | 1.03 | 0.87 | 0.78 | 0.70 | 1.52 | 0.69 | 0.01 | 0.01 | 0.01 | 0.00 | 0.03 | 0.00 |
| *Laccaria* | 0.72 | 0.88 | 0.73 | 0.72 | 1.02 | 1.01 | 0.00 | -0.01 | 0.00 | 0.01 | 0.01 | 0.01 |
| *Leclercia* | 0.73 | 0.77 | 1.02 | 0.76 | 0.90 | 0.96 | 0.00 | -0.01 | -0.01 | 0.00 | 0.01 | 0.00 |
| *Mageeibacillus* | 1.19 | 0.57 | 1.07 | 0.77 | 0.66 | 0.90 | 0.02 | 0.00 | -0.01 | 0.00 | -0.01 | -0.01 |
| *Methanosphaera* | 0.27 | 1.33 | 1.11 | 0.48 | 0.65 | 1.72 | 0.00 | 0.02 | 0.01 | 0.00 | 0.01 | 0.02 |
| *Methanothermococcus* | 0.60 | 0.49 | 0.98 | 0.95 | 0.81 | 0.75 | 0.00 | 0.00 | -0.01 | 0.00 | 0.00 | 0.00 |
| *Methanothermus* | 0.85 | 0.87 | 0.76 | 0.72 | 0.74 | 0.91 | -0.01 | -0.01 | 0.00 | 0.00 | 0.00 | 0.01 |
| *Microterricola* | 1.09 | 0.72 | 0.76 | 1.34 | 0.84 | 0.22 | -0.01 | -0.01 | 0.01 | 0.02 | 0.02 | 0.00 |
| *Moesziomyces* | 0.86 | 0.64 | 1.02 | 0.71 | 0.70 | 0.89 | -0.01 | 0.00 | -0.01 | -0.01 | 0.00 | 0.00 |
| *Neisseria* | 0.84 | 0.41 | 0.77 | 0.75 | 0.86 | 0.85 | 0.00 | 0.00 | 0.00 | 0.00 | 0.00 | 0.00 |
| *Neurospora* | 0.99 | 0.64 | 0.83 | 0.69 | 0.81 | 0.78 | 0.00 | 0.01 | 0.00 | 0.00 | 0.00 | 0.00 |
| *Nodularia* | 1.09 | 0.46 | 0.80 | 0.79 | 1.25 | 0.71 | 0.01 | 0.00 | 0.00 | 0.00 | 0.02 | 0.01 |
| *Pandoraea* | 1.08 | 0.43 | 0.80 | 0.77 | 0.85 | 0.96 | -0.01 | 0.00 | 0.00 | 0.00 | -0.01 | 0.00 |
| *Paracoccus* | 0.60 | 1.45 | 1.23 | 1.03 | 0.58 | 0.69 | 0.00 | -0.02 | -0.01 | 0.01 | -0.01 | -0.01 |
| *Paucibacter* | 0.65 | 0.58 | 1.29 | 1.06 | 0.79 | 1.61 | 0.00 | -0.01 | 0.01 | 0.01 | 0.02 | -0.02 |
| *Petrimonas* | 0.99 | 1.20 | 1.61 | 0.70 | 0.54 | 0.70 | 0.01 | 0.02 | 0.02 | -0.01 | 0.00 | 0.01 |
| *Propionibacterium* | 0.64 | 0.93 | 0.73 | 0.75 | 0.85 | 0.95 | 0.00 | 0.01 | -0.01 | -0.01 | 0.01 | -0.01 |
| *Puccinia* | 0.89 | 0.67 | 0.97 | 0.81 | 0.76 | 0.78 | 0.00 | 0.01 | 0.00 | 0.00 | 0.00 | 0.00 |
| *Rhodomicrobium* | 0.65 | 0.80 | 0.79 | 0.82 | 1.07 | 1.02 | 0.00 | -0.01 | 0.00 | -0.01 | -0.01 | -0.01 |
| *Rhodovulum* | 1.21 | 0.48 | 1.00 | 1.09 | 0.69 | 0.76 | -0.01 | -0.01 | 0.01 | 0.01 | -0.01 | 0.00 |
| *Rivularia* | 0.74 | 1.41 | 0.87 | 0.75 | 0.57 | 0.96 | 0.00 | 0.02 | 0.01 | 0.01 | 0.00 | 0.01 |
| *Ruegeria* | 0.90 | 1.18 | 0.77 | 1.17 | 0.60 | 0.37 | -0.01 | -0.02 | 0.01 | 0.01 | 0.01 | 0.00 |
| *Sandaracinus* | 0.74 | 0.85 | 1.30 | 0.74 | 0.79 | 0.87 | 0.00 | -0.01 | -0.01 | 0.00 | 0.00 | -0.01 |
| *Scheffersomyces* | 0.90 | 1.59 | 0.76 | 0.82 | 0.76 | 0.72 | 0.00 | -0.03 | 0.00 | 0.00 | 0.00 | 0.00 |
| *Sclerotinia* | 0.89 | 0.70 | 0.87 | 0.76 | 1.01 | 0.76 | 0.00 | -0.01 | 0.00 | 0.00 | -0.01 | 0.00 |
| *Sphaerochaeta* | 1.49 | 0.57 | 1.20 | 0.70 | 1.64 | 0.40 | -0.02 | -0.01 | 0.01 | 0.00 | 0.03 | 0.00 |
| *Syntrophus* | 0.95 | 0.87 | 0.24 | 0.78 | 1.04 | 0.40 | -0.01 | 0.02 | 0.00 | -0.01 | 0.02 | 0.00 |
| *Theileria* | 0.73 | 0.77 | 1.15 | 1.10 | 0.72 | 0.84 | 0.00 | -0.01 | -0.01 | -0.01 | 0.01 | 0.00 |
| *Thiobacimonas* | 0.88 | 0.59 | 0.83 | 0.77 | 0.71 | 0.81 | 0.00 | 0.00 | 0.00 | 0.00 | -0.01 | 0.00 |
| *Thioploca* | 0.82 | 0.56 | 0.78 | 0.77 | 0.91 | 0.95 | 0.00 | 0.00 | 0.00 | -0.01 | 0.00 | -0.01 |

| | | | | | | | | | | | | |
|---|---|---|---|---|---|---|---|---|---|---|---|---|
| *Trichoderma* | 0.82 | 0.55 | 0.76 | 0.76 | 0.84 | 0.89 | -0.01 | -0.01 | 0.00 | 0.00 | 0.00 | 0.00 |
| *Trichophyton* | 0.80 | 0.56 | 0.81 | 0.78 | 0.89 | 0.81 | 0.00 | 0.00 | 0.00 | 0.00 | 0.01 | 0.00 |
| *Wickerhamomyces* | 0.83 | 0.49 | 1.22 | 1.38 | 0.78 | 0.77 | 0.00 | 0.00 | -0.01 | -0.01 | 0.00 | 0.00 |
| *Blastomyces* | 1.08 | 0.51 | 0.79 | 0.75 | 0.85 | 0.80 | 0.01 | 0.00 | 0.00 | 0.00 | 0.00 | 0.00 |
| *Deinococcus* | 0.74 | 0.91 | 0.76 | 0.69 | 0.86 | 0.63 | 0.01 | -0.01 | -0.01 | 0.00 | -0.01 | -0.01 |
| *Hammondia* | 0.98 | 0.50 | 0.75 | 0.72 | 0.65 | 0.94 | -0.01 | 0.00 | 0.01 | 0.01 | 0.00 | 0.01 |
| *Heliobacterium* | 0.98 | 0.66 | 0.76 | 0.77 | 0.68 | 0.84 | -0.01 | 0.01 | 0.00 | 0.00 | 0.00 | -0.01 |
| *Leptothrix* | 0.76 | 0.62 | 0.85 | 0.79 | 0.77 | 0.99 | 0.00 | 0.00 | 0.00 | -0.01 | 0.00 | -0.01 |
| *Libanicoccus* | 0.75 | 0.68 | 0.77 | 0.87 | 0.67 | 1.43 | 0.01 | 0.01 | 0.00 | 0.00 | 0.01 | 0.02 |
| *Magnaporthe* | 0.69 | 0.67 | 0.85 | 0.76 | 0.87 | 0.69 | 0.00 | 0.01 | 0.01 | 0.01 | 0.00 | 0.00 |
| *Massilia* | 0.79 | 0.58 | 0.77 | 0.76 | 0.90 | 0.84 | 0.00 | 0.01 | -0.01 | -0.01 | -0.01 | 0.00 |
| *Methanosarcina* | 0.80 | 0.72 | 0.81 | 0.71 | 0.82 | 0.79 | -0.01 | -0.01 | 0.00 | 0.01 | 0.00 | 0.00 |
| *Naumovozyma* | 0.86 | 0.62 | 0.95 | 0.76 | 0.73 | 0.78 | 0.00 | -0.01 | 0.00 | 0.00 | 0.00 | 0.00 |
| *Pochonia* | 0.78 | 0.73 | 1.03 | 0.94 | 0.79 | 0.74 | 0.00 | -0.01 | -0.01 | 0.00 | 0.00 | 0.00 |
| *Sorangium* | 0.73 | 0.57 | 0.80 | 0.78 | 0.85 | 0.95 | 0.00 | 0.00 | -0.01 | -0.01 | 0.00 | -0.01 |
| *Toxoplasma* | 0.72 | 0.70 | 1.19 | 1.11 | 0.66 | 0.70 | -0.01 | -0.01 | -0.01 | -0.01 | 0.00 | 0.01 |

Partial Least Squares analysis was performed with 2 latent components. Subscript $_{VIP\geq0.8}$ denotes microbial genera that had VIP ≥ 0.8 in 4 or more timepoints. Subscripts $_{(+)}$ and $_{(-)}$ represent microbial genera that had positive or negative regression coefficient in 4 or more timepoints, respectively.

**Supplementary table 3 - B. Taxonomic information of significantly repeatable microbial genera with stable associations with host performance traits.**

| Microbial genera | Taxonomic order | Taxonomic phylum |
|---|---|---|
| *Acanthamoeba* | Longamoebia | Discosea |
| *Acaryochloris* | Synechococcales | Cyanobacteria |
| *Acetomicrobium* | Synergistales | Synergistetes |
| *Achromobacter* | Burkholderiales | Proteobacteria |
| *Acidaminococcus* | Acidaminococcales | Firmicutes |
| *Acidihalobacter* | Chromatiales | Proteobacteria |
| *Acidiphilium* | Rhodospirillales | Proteobacteria |
| *Acidothermus* | Acidothermales | Actinobacteria |
| *Acidovorax* | Burkholderiales | Proteobacteria |
| *Actinomyces* | Actinomycetales | Actinobacteria |
| *Actinoplanes* | Actinomycetales | Actinobacteria |
| *Acytostelium* | Acytosteliales | Evosea |
| *Adlercreutzia* | Eggerthellales | Actinobacteria |
| *Aerococcus* | Lactobacillales | Firmicutes |
| *Aeromicrobium* | Propionibacteriales | Actinobacteria |
| *Aeromonas* | Aeromonadales | Proteobacteria |
| *Agaricus* | Agaricales | Basidiomycota |
| *Agrobacterium* | Hyphomicrobiales | Proteobacteria |
| *Agromyces* | Micrococcales | Actinobacteria |
| *Alcanivorax* | Oceanospirillales | Proteobacteria |
| *Alicycliphilus* | Burkholderiales | Proteobacteria |
| *Alicyclobacillus* | Bacillales | Firmicutes |
| *Alkalilimnicola* | Chromatiales | Proteobacteria |
| *Alkaliphilus* | Eubacteriales | Firmicutes |
| *Allisonella* | Veillonellales | Firmicutes |
| *Altererythrobacter* | Sphingomonadales | Proteobacteria |
| *Alteromonas* | Alteromonadales | Proteobacteria |
| *Aminomonas* | Synergistales | Synergistetes |

| | | |
|---|---|---|
| *Ammonifex* | Thermoanaerobacterales | Firmicutes |
| *Anabaena* | Nostocales | Cyanobacteria |
| *Anaeromyxobacter* | Myxococcales | Proteobacteria |
| *Archangium* | Myxococcales | Proteobacteria |
| *Aromatoleum* | Rhodocyclales | Proteobacteria |
| *Arsenicicoccus* | Micrococcales | Actinobacteria |
| *Arsenophonus* | Enterobacterales | Proteobacteria |
| *Arthrobacter* | Micrococcales | Actinobacteria |
| *Arthrobotrys* | Orbiliales | Ascomycota |
| *Aspergillus* | Eurotiales | Ascomycota |
| *Asticcacaulis* | Caulobacterales | Proteobacteria |
| *Atopobium* | Coriobacteriales | Actinobacteria |
| *Aureimonas* | Hyphomicrobiales | Proteobacteria |
| *Azoarcus* | Rhodocyclales | Proteobacteria |
| *Azorhizobium* | Hyphomicrobiales | Proteobacteria |
| *Azospira* | Rhodocyclales | Proteobacteria |
| *Azospirillum* | Rhodospirillales | Proteobacteria |
| *Babjeviella* | Saccharomycetales | Ascomycota |
| *Bacteroides* | Bacteroidales | Bacteroidota |
| *Basfia* | Pasteurellales | Proteobacteria |
| *Beauveria* | Hypocreales | Ascomycota |
| *Beijerinckia* | Hyphomicrobiales | Proteobacteria |
| *Bernardetia* | Cytophagales | Bacteroidota |
| *Bibersteinia* | Pasteurellales | Proteobacteria |
| *Bifidobacterium* | Bifidobacteriales | Actinobacteria |
| *Bipolaris* | Pleosporales | Ascomycota |
| *Blastochloris* | Hyphomicrobiales | Proteobacteria |
| *Blastocystis* | Opalinata | |
| *Blastomonas* | Sphingomonadales | Proteobacteria |
| *Blastomyces* | Onygenales | Ascomycota |
| *Blattabacterium* | Flavobacteriales | Bacteroidota |
| *Blautia* | Eubacteriales | Firmicutes |
| *Bordetella* | Burkholderiales | Proteobacteria |
| *Botrytis* | Helotiales | Ascomycota |
| *Brachyspira* | Brachyspirales | Spirochaetes |

| | | |
|---|---|---|
| *Bradyrhizobium* | Hyphomicrobiales | Proteobacteria |
| *Brevundimonas* | Caulobacterales | Proteobacteria |
| *Burkholderia* | Burkholderiales | Proteobacteria |
| *Caldisericum* | Caldisericales | Caldiserica |
| *Calditerrivibrio* | Deferribacterales | Deferribacteres |
| *Calothrix* | Nostocales | Cyanobacteria |
| *Campylobacter* | Campylobacterales | Proteobacteria |
| *Candida* | Saccharomycetales | Ascomycota |
| *Candidatus Amoebophilus* | Cytophagales | Bacteroidota |
| *Candidatus Carsonella* | Oceanospirillales | Proteobacteria |
| *Candidatus Cloacimonas* | | Candidatus Cloacimonetes |
| *Candidatus Ishikawaella* | Enterobacterales | Proteobacteria |
| *Candidatus Koribacter* | Acidobacteriales | Acidobacteria |
| *Candidatus Methanomethylophilus* | Methanomassiliicoccales | Candidatus Thermoplasmatota |
| *Candidatus Methanoperedens* | Methanosarcinales | Euryarchaeota |
| *Candidatus Methylopumilus* | Nitrosomonadales | Proteobacteria |
| *Candidatus Paracaedibacter* | Holosporales | Proteobacteria |
| *Candidatus Phytoplasma* | Acholeplasmatales | Tenericutes |
| *Candidatus Profftella* | | Proteobacteria |
| *Candidatus Protochlamydia* | Parachlamydiales | Chlamydiae |
| *Candidatus Sulcia* | Flavobacteriales | Bacteroidota |
| *Capronia* | Chaetothyriales | Ascomycota |
| *Carboxydothermus* | Thermoanaerobacterales | Firmicutes |
| *Castellaniella* | Burkholderiales | Proteobacteria |
| *Caulobacter* | Caulobacterales | Proteobacteria |
| *Cedecea* | Enterobacterales | Proteobacteria |
| *Cellulomonas* | Micrococcales | Actinobacteria |
| *Cellvibrio* | Cellvibrionales | Proteobacteria |
| *Chaetomium* | Sordariales | Ascomycota |
| *Chamaesiphon* | Synechococcales | Cyanobacteria |
| *Chelativorans* | Hyphomicrobiales | Proteobacteria |
| *Chelatococcus* | Hyphomicrobiales | Proteobacteria |
| *Chlorobaculum* | Chlorobiales | Chlorobi |
| *Chlorobium* | Chlorobiales | Chlorobi |
| *Chloroflexus* | Chloroflexales | Chloroflexi |

| | | |
|---|---|---|
| *Chromobacterium* | Neisseriales | Proteobacteria |
| *Chromohalobacter* | Oceanospirillales | Proteobacteria |
| *Chryseobacterium* | Flavobacteriales | Bacteroidota |
| *Chthonomonas* | Chthonomonadales | Armatimonadetes |
| *Cladophialophora* | Chaetothyriales | Ascomycota |
| *Clavibacter* | Micrococcales | Actinobacteria |
| *Clavispora* | Saccharomycetales | Ascomycota |
| *Cloacibacillus* | Synergistales | Synergistetes |
| *Clostridioides* | Eubacteriales | Firmicutes |
| *Coccidioides* | Onygenales | Ascomycota |
| *Colletotrichum* | Glomerellales | Ascomycota |
| *Coraliomargarita* | Puniceicoccales | Verrucomicrobia |
| *Cordyceps* | Hypocreales | Ascomycota |
| *Coriobacterium* | Coriobacteriales | Actinobacteria |
| *Corynebacterium* | Corynebacteriales | Actinobacteria |
| *Croceibacter* | Flavobacteriales | Bacteroidota |
| *Croceicoccus* | Sphingomonadales | Proteobacteria |
| *Cronobacter* | Enterobacterales | Proteobacteria |
| *Cryptobacterium* | Eggerthellales | Actinobacteria |
| *Cryptosporidium* | Eucoccidiorida | Apicomplexa |
| *Cupriavidus* | Burkholderiales | Proteobacteria |
| *Curtobacterium* | Micrococcales | Actinobacteria |
| *Cutaneotrichosporon* | Trichosporonales | Basidiomycota |
| *Cyanobium* | Synechococcales | Cyanobacteria |
| *Cyanothece* | Oscillatoriales | Cyanobacteria |
| *Cylindrospermum* | Nostocales | Cyanobacteria |
| *Cytophaga* | Cytophagales | Bacteroidota |
| *Dactylellina* | Orbiliales | Ascomycota |
| *Debaryomyces* | Saccharomycetales | Ascomycota |
| *Dechloromonas* | Rhodocyclales | Proteobacteria |
| *Deferribacter* | Deferribacterales | Deferribacteres |
| *Defluviimonas* | Rhodobacterales | Proteobacteria |
| *Dehalococcoides* | Dehalococcoidales | Chloroflexi |
| *Dehalogenimonas* | | Chloroflexi |
| *Deinococcus* | Deinococcales | Deinococcus-Thermus |

| | | |
|---|---|---|
| *Delftia* | Burkholderiales | Proteobacteria |
| *Dermabacter* | Micrococcales | Actinobacteria |
| *Dermacoccus* | Micrococcales | Actinobacteria |
| *Desulfitobacterium* | Eubacteriales | Firmicutes |
| *Desulfobacter* | Desulfobacterales | Proteobacteria |
| *Desulfobacterium* | Desulfobacterales | Proteobacteria |
| *Desulfocapsa* | Desulfobacterales | Proteobacteria |
| *Desulfococcus* | Desulfobacterales | Proteobacteria |
| *Desulfovibrio* | Desulfovibrionales | Proteobacteria |
| *Desulfurispirillum* | Chrysiogenales | Chrysiogenetes |
| *Devosia* | Hyphomicrobiales | Proteobacteria |
| *Dialister* | Veillonellales | Firmicutes |
| *Dickeya* | Enterobacterales | Proteobacteria |
| *Dictyoglomus* | Dictyoglomales | Dictyoglomi |
| *Dictyostelium* | Dictyosteliales | Evosea |
| *Dokdonella* | Xanthomonadales | Proteobacteria |
| *Dorea* | Eubacteriales | Firmicutes |
| *Dyadobacter* | Cytophagales | Bacteroidota |
| *Dyella* | Xanthomonadales | Proteobacteria |
| *Ectothiorhodospira* | Chromatiales | Proteobacteria |
| *Edwardsiella* | Enterobacterales | Proteobacteria |
| *Eggerthella* | Eggerthellales | Actinobacteria |
| *Eimeria* | Eucoccidiorida | Apicomplexa |
| *Elizabethkingia* | Flavobacteriales | Bacteroidota |
| *Emiliania* | Isochrysidales | Haptophyta |
| *Endocarpon* | Verrucariales | Ascomycota |
| *Entamoeba* | Mastigamoebida | Evosea |
| *Enterobacter* | Enterobacterales | Proteobacteria |
| *Enterococcus* | Lactobacillales | Firmicutes |
| *Enterocytozoon* | | Microsporidia |
| *Eremothecium* | Saccharomycetales | Ascomycota |
| *Erysipelatoclostridium* | Erysipelotrichales | Firmicutes |
| *Ethanoligenens* | Eubacteriales | Firmicutes |
| *Eutypa* | Xylariales | Ascomycota |
| *Exiguobacterium* | Bacillales | Firmicutes |

| | | |
|---|---|---|
| *Faecalibaculum* | Erysipelotrichales | Firmicutes |
| *Faecalitalea* | Erysipelotrichales | Firmicutes |
| *Fibrobacter* | Fibrobacterales | Fibrobacteres |
| *Fomitiporia* | Hymenochaetales | Basidiomycota |
| *Fonsecaea* | Chaetothyriales | Ascomycota |
| *Frankia* | Frankiales | Actinobacteria |
| *Frischella* | Orbales | Proteobacteria |
| *Fusarium* | Hypocreales | Ascomycota |
| *Gemmatimonas* | Gemmatimonadales | Gemmatimonadetes |
| *Geoalkalibacter* | Desulfuromonadales | Proteobacteria |
| *Geobacter* | Desulfuromonadales | Proteobacteria |
| *Geosporobacter* | Eubacteriales | Firmicutes |
| *Gilliamella* | Orbales | Proteobacteria |
| *Glarea* | Helotiales | Ascomycota |
| *Gloeobacter* | Gloeobacterales | Cyanobacteria |
| *Gordonia* | Corynebacteriales | Actinobacteria |
| *Gordonibacter* | Eggerthellales | Actinobacteria |
| *Gregarina* | Eugregarinorida | Apicomplexa |
| *Guillardia* | Pyrenomonadales | |
| *Haemophilus* | Pasteurellales | Proteobacteria |
| *Halapricum* | Halobacteriales | Euryarchaeota |
| *Halobacillus* | Bacillales | Firmicutes |
| *Halococcus* | Halobacteriales | Euryarchaeota |
| *Halocynthiibacter* | Rhodobacterales | Proteobacteria |
| *Halolamina* | Haloferacales | Euryarchaeota |
| *Halomonas* | Oceanospirillales | Proteobacteria |
| *Halopiger* | Natrialbales | Euryarchaeota |
| *Haloquadratum* | Haloferacales | Euryarchaeota |
| *Halorhodospira* | Chromatiales | Proteobacteria |
| *Halosimplex* | Halobacteriales | Euryarchaeota |
| *Halotalea* | Oceanospirillales | Proteobacteria |
| *Halothiobacillus* | Chromatiales | Proteobacteria |
| *Hammondia* | Eucoccidiorida | Apicomplexa |
| *Heliobacterium* | Eubacteriales | Firmicutes |
| *Herbaspirillum* | Burkholderiales | Proteobacteria |

| | | |
|---|---|---|
| *Histoplasma* | Onygenales | Ascomycota |
| *Hoyosella* | Corynebacteriales | Actinobacteria |
| *Hydrogenophaga* | Burkholderiales | Proteobacteria |
| *Hyphomicrobium* | Hyphomicrobiales | Proteobacteria |
| *Hyphomonas* | Hyphomonadales | Proteobacteria |
| *Ichthyophthirius* | Hymenostomatida | Ciliophora |
| *Immundisolibacter* | Immundisolibacterales | Proteobacteria |
| *Isaria* | Hypocreales | Ascomycota |
| *Janibacter* | Micrococcales | Actinobacteria |
| *Jeongeupia* | Neisseriales | Proteobacteria |
| *Jeotgalibacillus* | Bacillales | Firmicutes |
| *Jeotgalicoccus* | Bacillales | Firmicutes |
| *Jonquetella* | Synergistales | Synergistetes |
| *Kalmanozyma* | Ustilaginales | Basidiomycota |
| *Kandleria* | Erysipelotrichales | Firmicutes |
| *Ketogulonicigenium* | Rhodobacterales | Proteobacteria |
| *Kingella* | Neisseriales | Proteobacteria |
| *Klebsiella* | Enterobacterales | Proteobacteria |
| *Kluyvera* | Enterobacterales | Proteobacteria |
| *Kluyveromyces* | Saccharomycetales | Ascomycota |
| *Komagataella* | Saccharomycetales | Ascomycota |
| *Kosakonia* | Enterobacterales | Proteobacteria |
| *Kwoniella* | Tremellales | Basidiomycota |
| *Laccaria* | Agaricales | Basidiomycota |
| *Lachnobacterium* | Eubacteriales | Firmicutes |
| *Lachnoclostridium* | Eubacteriales | Firmicutes |
| *Lachnospira* | Eubacteriales | Firmicutes |
| *Lacinutrix* | Flavobacteriales | Bacteroidota |
| *Lactobacillus* | Lactobacillales | Firmicutes |
| *Laribacter* | Neisseriales | Proteobacteria |
| *Leclercia* | Enterobacterales | Proteobacteria |
| *Legionella* | Legionellales | Proteobacteria |
| *Leptolyngbya* | Pseudanabaenales | Cyanobacteria |
| *Leptomonas* | Trypanosomatida | Euglenozoa |
| *Leptosphaeria* | Pleosporales | Ascomycota |

| | | |
|---|---|---|
| *Leptothrix* | Burkholderiales | Proteobacteria |
| *Libanicoccus* | Coriobacteriales | Actinobacteria |
| *Lodderomyces* | Saccharomycetales | Ascomycota |
| *Luteipulveratus* | Micrococcales | Actinobacteria |
| *Lysobacter* | Xanthomonadales | Proteobacteria |
| *Mageeibacillus* | Eubacteriales | Firmicutes |
| *Magnaporthe* | Magnaporthales | Ascomycota |
| *Magnetococcus* | Magnetococcales | Proteobacteria |
| *Magnetospira* | Rhodospirillales | Proteobacteria |
| *Magnetospirillum* | Rhodospirillales | Proteobacteria |
| *Malassezia* | Malasseziales | Basidiomycota |
| *Mannheimia* | Pasteurellales | Proteobacteria |
| *Marinithermus* | Thermales | Deinococcus-Thermus |
| *Marivirga* | Cytophagales | Bacteroidota |
| *Marssonina* | Helotiales | Ascomycota |
| *Massilia* | Burkholderiales | Proteobacteria |
| *Melampsora* | Pucciniales | Basidiomycota |
| *Melioribacter* | Ignavibacteriales | Ignavibacteriae |
| *Mesoplasma* | Entomoplasmatales | Tenericutes |
| *Mesorhizobium* | Hyphomicrobiales | Proteobacteria |
| *Methanobacterium* | Methanobacteriales | Euryarchaeota |
| *Methanobrevibacter* | Methanobacteriales | Euryarchaeota |
| *Methanocaldococcus* | Methanococcales | Euryarchaeota |
| *Methanocella* | Methanocellales | Euryarchaeota |
| *Methanococcoides* | Methanosarcinales | Euryarchaeota |
| *Methanococcus* | Methanococcales | Euryarchaeota |
| *Methanoculleus* | Methanomicrobiales | Euryarchaeota |
| *Methanofollis* | Methanomicrobiales | Euryarchaeota |
| *Methanohalophilus* | Methanosarcinales | Euryarchaeota |
| *Methanolacinia* | Methanomicrobiales | Euryarchaeota |
| *Methanolobus* | Methanosarcinales | Euryarchaeota |
| *Methanomassiliicoccus* | Methanomassiliicoccales | Candidatus Thermoplasmatota |
| *Methanoregula* | Methanomicrobiales | Euryarchaeota |
| *Methanosarcina* | Methanosarcinales | Euryarchaeota |
| *Methanosphaera* | Methanobacteriales | Euryarchaeota |

| | | |
|---|---|---|
| *Methanothermobacter* | Methanobacteriales | Euryarchaeota |
| *Methanothermococcus* | Methanococcales | Euryarchaeota |
| *Methanothermus* | Methanobacteriales | Euryarchaeota |
| *Methanotorris* | Methanococcales | Euryarchaeota |
| *Methylacidiphilum* | Methylacidiphilales | Verrucomicrobia |
| *Methylibium* | Burkholderiales | Proteobacteria |
| *Methylobacterium* | Hyphomicrobiales | Proteobacteria |
| *Methyloceanibacter* | Hyphomicrobiales | Proteobacteria |
| *Methylocella* | Hyphomicrobiales | Proteobacteria |
| *Methylococcus* | Methylococcales | Proteobacteria |
| *Methylomonas* | Methylococcales | Proteobacteria |
| *Methyloversatilis* | Nitrosomonadales | Proteobacteria |
| *Microbacterium* | Micrococcales | Actinobacteria |
| *Microbulbifer* | Cellvibrionales | Proteobacteria |
| *Microsporum* | Onygenales | Ascomycota |
| *Microterricola* | Micrococcales | Actinobacteria |
| *Millerozyma* | Saccharomycetales | Ascomycota |
| *Mitsuokella* | Selenomonadales | Firmicutes |
| *Modestobacter* | Geodermatophilales | Actinobacteria |
| *Moesziomyces* | Ustilaginales | Basidiomycota |
| *Moorella* | Thermoanaerobacterales | Firmicutes |
| *Moraxella* | Moraxellales | Proteobacteria |
| *Moritella* | Alteromonadales | Proteobacteria |
| *Murdochiella* | Tissierellales | Firmicutes |
| *Mycoplasma* | Mycoplasmatales | Tenericutes |
| *Naegleria* | | Heterolobosea |
| *Nakaseomyces* | Saccharomycetales | Ascomycota |
| *Nannizzia* | Onygenales | Ascomycota |
| *Nannochloropsis* | Eustigmatales | |
| *Natranaerobius* | Natranaerobiaceae | Firmicutes |
| *Natrialba* | Natrialbales | Euryarchaeota |
| *Natronomonas* | Halobacteriales | Euryarchaeota |
| *Naumovozyma* | Saccharomycetales | Ascomycota |
| *Negativicoccus* | Veillonellales | Firmicutes |
| *Neisseria* | Neisseriales | Proteobacteria |

| | | |
|---|---|---|
| *Neofusicoccum* | Botryosphaeriales | Ascomycota |
| *Neospora* | Eucoccidiorida | Apicomplexa |
| *Neurospora* | Sordariales | Ascomycota |
| *Niastella* | Chitinophagales | Bacteroidota |
| *Nitratireductor* | Hyphomicrobiales | Proteobacteria |
| *Nitrobacter* | Hyphomicrobiales | Proteobacteria |
| *Nitrosopumilus* | Nitrosopumilales | Thaumarchaeota |
| *Nitrosospira* | Nitrosomonadales | Proteobacteria |
| *Nodularia* | Nostocales | Cyanobacteria |
| *Nosema* | | Microsporidia |
| *Nostoc* | Nostocales | Cyanobacteria |
| *Novosphingobium* | Sphingomonadales | Proteobacteria |
| *Oceanimonas* | Aeromonadales | Proteobacteria |
| *Octadecabacter* | Rhodobacterales | Proteobacteria |
| *Olsenella* | Coriobacteriales | Actinobacteria |
| *Oribacterium* | Eubacteriales | Firmicutes |
| *Ottowia* | Burkholderiales | Proteobacteria |
| *Paenibacillus* | Bacillales | Firmicutes |
| *Pandoraea* | Burkholderiales | Proteobacteria |
| *Pannonibacter* | Hyphomicrobiales | Proteobacteria |
| *Pantoea* | Enterobacterales | Proteobacteria |
| *Paraburkholderia* | Burkholderiales | Proteobacteria |
| *Paracoccidioides* | Onygenales | Ascomycota |
| *Paracoccus* | Rhodobacterales | Proteobacteria |
| *Paramecium* | Peniculida | Ciliophora |
| *Parascardovia* | Bifidobacteriales | Actinobacteria |
| *Pasteurella* | Pasteurellales | Proteobacteria |
| *Paucibacter* | Burkholderiales | Proteobacteria |
| *Pectobacterium* | Enterobacterales | Proteobacteria |
| *Pelagibaca* | Rhodobacterales | Proteobacteria |
| *Pelobacter* | Desulfuromonadales | Proteobacteria |
| *Pelodictyon* | Chlorobiales | Chlorobi |
| *Pelosinus* | Selenomonadales | Firmicutes |
| *Penicillium* | Eurotiales | Ascomycota |
| *Perkinsus* | Perkinsida | Perkinsozoa |

| | | |
|---|---|---|
| *Petrimonas* | Bacteroidales | Bacteroidota |
| *Phaeoacremonium* | Togniniales | Ascomycota |
| *Phaeobacter* | Rhodobacterales | Proteobacteria |
| *Phialocephala* | Helotiales | Ascomycota |
| *Phycisphaera* | Phycisphaerales | Planctomycetota |
| *Phycomyces* | Mucorales | Mucoromycota; |
| *Phytophthora* | Peronosporales | Oomycota |
| *Pichia* | Saccharomycetales | Ascomycota |
| *Planktothrix* | Oscillatoriales | Cyanobacteria |
| *Planococcus* | Bacillales | Firmicutes |
| *Plasmodium* | Haemosporida | Apicomplexa |
| *Pluralibacter* | Enterobacterales | Proteobacteria |
| *Pneumocystis* | Pneumocystidales | Ascomycota |
| *Pochonia* | Hypocreales | Ascomycota |
| *Podospora* | Sordariales | Ascomycota |
| *Polymorphum* | Rhodobacterales | Proteobacteria |
| *Porphyrobacter* | Sphingomonadales | Proteobacteria |
| *Propionibacterium* | Propionibacteriales | Actinobacteria |
| *Proteiniclasticum* | Eubacteriales | Firmicutes |
| *Pseudomonas* | Pseudomonadales | Proteobacteria |
| *Pseudopropionibacterium* | Propionibacteriales | Actinobacteria |
| *Pseudoxanthomonas* | Xanthomonadales | Proteobacteria |
| *Psychroflexus* | Flavobacteriales | Bacteroidota |
| *Puccinia* | Pucciniales | Basidiomycota |
| *Ralstonia* | Burkholderiales | Proteobacteria |
| *Raoultella* | Enterobacterales | Proteobacteria |
| *Rasamsonia* | Eurotiales | Ascomycota |
| *Rathayibacter* | Micrococcales | Actinobacteria |
| *Rhinocladiella* | Chaetothyriales | Ascomycota |
| *Rhizobium* | Hyphomicrobiales | Proteobacteria |
| *Rhodanobacter* | Xanthomonadales | Proteobacteria |
| *Rhodobacter* | Rhodobacterales | Proteobacteria |
| *Rhodoferax* | Burkholderiales | Proteobacteria |
| *Rhodomicrobium* | Hyphomicrobiales | Proteobacteria |
| *Rhodopseudomonas* | Hyphomicrobiales | Proteobacteria |

| | | |
|---|---|---|
| *Rhodovulum* | Rhodobacterales | Proteobacteria |
| *Rivularia* | Nostocales | Cyanobacteria |
| *Roseburia* | Eubacteriales | Firmicutes |
| *Rubrobacter* | Rubrobacterales | Actinobacteria |
| *Ruegeria* | Rhodobacterales | Proteobacteria |
| *Ruminiclostridium* | Eubacteriales | Firmicutes |
| *Saccharomyces* | Saccharomycetales | Ascomycota |
| *Salegentibacter* | Flavobacteriales | Bacteroidota |
| *Salimicrobium* | Bacillales | Firmicutes |
| *Salinicoccus* | Bacillales | Firmicutes |
| *Salinispira* | Spirochaetales | Spirochaetes |
| *Salinispora* | Micromonosporales | Actinobacteria |
| *Sandaracinus* | Myxococcales | Proteobacteria |
| *Sanguibacter* | Micrococcales | Actinobacteria |
| *Scedosporium* | Microascales | Ascomycota |
| *Scheffersomyces* | Saccharomycetales | Ascomycota |
| *Schizosaccharomyces* | Schizosaccharomycetales | Ascomycota |
| *Sclerotinia* | Helotiales | Ascomycota |
| *Sedimenticola* | | Proteobacteria |
| *Selenomonas* | Selenomonadales | Firmicutes |
| *Serpula* | Boletales | Basidiomycota |
| *Setosphaeria* | Pleosporales | Ascomycota |
| *Shimwellia* | Enterobacterales | Proteobacteria |
| *Shinella* | Hyphomicrobiales | Proteobacteria |
| *Sideroxydans* | Nitrosomonadales | Proteobacteria |
| *Sinomonas* | Micrococcales | Actinobacteria |
| *Sinorhizobium* | Hyphomicrobiales | Proteobacteria |
| *Sorangium* | Myxococcales | Proteobacteria |
| *Spathaspora* | Saccharomycetales | Ascomycota |
| *Sphaerochaeta* | Spirochaetales | Spirochaetes |
| *Sphaerulina* | Mycosphaerellales | Ascomycota |
| *Sphingobium* | Sphingomonadales | Proteobacteria |
| *Sphingomonas* | Sphingomonadales | Proteobacteria |
| *Sphingopyxis* | Sphingomonadales | Proteobacteria |
| *Spiribacter* | Chromatiales | Proteobacteria |

| | | |
|---|---|---|
| *Spirochaeta* | Spirochaetales | Spirochaetes |
| *Spizellomyces* | Spizellomycetales | Chytridiomycota |
| *Sporothrix* | Ophiostomatales | Ascomycota |
| *Starkeya* | Hyphomicrobiales | Proteobacteria |
| *Stereum* | Russulales | Basidiomycota |
| *Stigmatella* | Myxococcales | Proteobacteria |
| *Succiniclasticum* | Acidaminococcales | Firmicutes |
| *Sugiyamaella* | Saccharomycetales | Ascomycota |
| *Sulfitobacter* | Rhodobacterales | Proteobacteria |
| *Sulfurovum* | Campylobacterales | Proteobacteria |
| *Synechococcus* | Synechococcales | Cyanobacteria |
| *Synechocystis* | Synechococcales | Cyanobacteria |
| *Syntrophobotulus* | Eubacteriales | Firmicutes |
| *Syntrophomonas* | Eubacteriales | Firmicutes |
| *Syntrophus* | Syntrophales | Proteobacteria |
| *Talaromyces* | Eurotiales | Ascomycota |
| *Teredinibacter* | Cellvibrionales | Proteobacteria |
| *Terribacillus* | Bacillales | Firmicutes |
| *Tetrahymena* | Hymenostomatida | Ciliophora |
| *Tetrapisispora* | Saccharomycetales | Ascomycota |
| *Thalassiosira* | Thalassiosirales | Bacillariophyta |
| *Thalassospira* | Rhodospirillales | Proteobacteria |
| *Thauera* | Rhodocyclales | Proteobacteria |
| *Theileria* | Piroplasmida | Apicomplexa |
| *Thermaerobacter* | Eubacteriales | Firmicutes |
| *Thermoanaerobacter* | Thermoanaerobacterales | Firmicutes |
| *Thermococcus* | Thermococcales | Euryarchaeota |
| *Thermosipho* | Thermotogales | Thermotogae |
| *Thermothelomyces* | Sordariales | Ascomycota |
| *Thermotoga* | Thermotogales | Thermotogae |
| *Thermovirga* | Synergistales | Synergistetes |
| *Thermus* | Thermales | Deinococcus-Thermus |
| *Thielavia* | Sordariales | Ascomycota |
| *Thioalkalivibrio* | Chromatiales | Proteobacteria |
| *Thiobacillus* | Nitrosomonadales | Proteobacteria |

| | | |
|---|---|---|
| *Thiobacimonas* | Rhodobacterales | Proteobacteria |
| *Thioflavicoccus* | Chromatiales | Proteobacteria |
| *Thiomonas* | Burkholderiales | Proteobacteria |
| *Thioploca* | Thiotrichales | Proteobacteria |
| *Tolumonas* | Aeromonadales | Proteobacteria |
| *Toxoplasma* | Eucoccidiorida | Apicomplexa |
| *Trichoderma* | Hypocreales | Ascomycota |
| *Trichodesmium* | Oscillatoriales | Cyanobacteria |
| *Trichomonas* | Trichomonadida | Parabasalia |
| *Trichophyton* | Onygenales | Ascomycota |
| *Trichormus* | Nostocales | Cyanobacteria |
| *Trueperella* | Actinomycetales | Actinobacteria |
| *Trypanosoma* | Trypanosomatida | Euglenozoa |
| *Tsukamurella* | Corynebacteriales | Actinobacteria |
| *Tuber* | Pezizales | Ascomycota |
| *Ustilago* | Ustilaginales | Basidiomycota |
| *Vanderwaltozyma* | Saccharomycetales | Ascomycota |
| *Variovorax* | Burkholderiales | Proteobacteria |
| *Veillonella* | Veillonellales | Firmicutes |
| *Vibrio* | Vibrionales | Proteobacteria |
| *Wallemia* | Wallemiales | Basidiomycota |
| *Wenyingzhuangia* | Flavobacteriales | Bacteroidota |
| *Wenzhouxiangella* | Chromatiales | Proteobacteria |
| *Wickerhamomyces* | Saccharomycetales | Ascomycota |
| *Xanthomonas* | Xanthomonadales | Proteobacteria |
| *Xenorhabdus* | Enterobacterales | Proteobacteria |
| *Xylona* | Xylonales | Ascomycota |
| *Yamadazyma* | Saccharomycetales | Ascomycota |
| *Yangia* | Rhodobacterales | Proteobacteria |
| *Zhongshania* | Cellvibrionales | Proteobacteria |
| *Zobellia* | Flavobacteriales | Bacteroidota |
| *Zygosaccharomyces* | Saccharomycetales | Ascomycota |

**Supplementary table 3 - C. Variable importance in projection (VIP) and regression coefficients from partial least squares analyses of significantly repeatable microbial genes with stable associations with host performance traits.**

**Trait: Feed Conversion Ratio (FCR); Predictors: 144 microbial genes (MG)**

| Microbial genes | VIP | | | | | | Regression coefficient | | | | | |
|---|---|---|---|---|---|---|---|---|---|---|---|---|
| | T1 | T2 | T3 | T4 | T5 | T6 | T1 | T2 | T3 | T4 | T5 | T6 |
| K01156$_{VIP\geq0.8\ (+)}$ | 2.13 | 1.10 | 1.47 | 1.08 | 1.75 | 0.24 | 0.07 | 0.03 | 0.05 | 0.03 | 0.06 | 0.01 |
| K22162$_{VIP\geq0.8\ (+)}$ | 0.88 | 2.26 | 1.77 | 0.99 | 1.59 | 1.07 | 0.03 | 0.05 | 0.07 | 0.02 | 0.08 | -0.01 |
| K02518$_{VIP\geq0.8\ (+)}$ | 0.72 | 0.50 | 1.05 | 0.93 | 1.49 | 0.93 | 0.03 | 0.00 | -0.02 | -0.02 | 0.03 | 0.01 |
| K04062$_{VIP\geq0.8\ (+)}$ | 1.54 | 0.85 | 0.97 | 1.61 | 0.95 | 1.19 | 0.06 | 0.02 | 0.04 | 0.04 | 0.04 | 0.03 |
| K00046$_{VIP\geq0.8\ (+)}$ | 0.40 | 0.36 | 1.01 | 1.42 | 1.03 | 1.75 | 0.01 | -0.01 | 0.04 | 0.03 | 0.04 | 0.05 |
| K03816$_{VIP\geq0.8\ (+)}$ | 1.35 | 0.53 | 1.19 | 1.38 | 0.41 | 1.03 | 0.03 | 0.00 | 0.04 | 0.03 | -0.02 | 0.03 |
| K07063$_{VIP\geq0.8\ (+)}$ | 1.17 | 0.57 | 1.23 | 0.99 | 0.71 | 1.44 | 0.04 | 0.00 | 0.05 | 0.02 | -0.02 | 0.05 |
| K00003$_{VIP\geq0.8\ (+)}$ | 1.29 | 1.03 | 0.92 | 0.60 | 0.74 | 1.36 | 0.04 | 0.02 | 0.02 | 0.01 | -0.03 | 0.02 |
| K02217$_{VIP\geq0.8\ (+)}$ | 1.11 | 0.87 | 0.77 | 0.77 | 1.48 | 1.04 | 0.02 | 0.00 | -0.01 | 0.02 | 0.07 | 0.02 |
| K09816$_{VIP\geq0.8\ (+)}$ | 2.08 | 0.98 | 0.95 | 0.39 | 1.04 | 0.87 | 0.07 | -0.01 | 0.02 | 0.00 | 0.03 | 0.01 |
| K00609$_{VIP\geq0.8\ (+)}$ | 0.56 | 1.24 | 1.77 | 1.36 | 2.21 | 0.95 | -0.02 | 0.03 | 0.07 | 0.03 | -0.10 | 0.03 |
| K05919$_{VIP\geq0.8\ (+)}$ | 1.40 | 0.73 | 0.89 | 0.56 | 1.18 | 1.34 | 0.04 | 0.00 | 0.00 | 0.01 | 0.04 | 0.01 |
| K00763$_{VIP\geq0.8\ (+)}$ | 1.12 | 0.93 | 1.05 | 0.48 | 0.59 | 0.92 | 0.02 | 0.00 | 0.01 | 0.01 | 0.02 | 0.01 |
| K08600$_{VIP\geq0.8\ (+)}$ | 0.91 | 0.94 | 1.03 | 0.39 | 0.65 | 1.05 | 0.02 | 0.02 | 0.02 | 0.01 | -0.03 | 0.00 |
| K03737$_{VIP\geq0.8\ (+)}$ | 0.82 | 0.91 | 1.15 | 0.88 | 0.64 | 0.32 | 0.01 | 0.00 | 0.02 | 0.02 | 0.00 | -0.01 |
| K08999$_{VIP\geq0.8\ (+)}$ | 1.44 | 0.93 | 0.85 | 0.23 | 1.57 | 0.70 | 0.05 | 0.00 | -0.01 | 0.01 | 0.07 | 0.01 |
| K00684$_{VIP\geq0.8\ (+)}$ | 1.12 | 0.97 | 1.33 | 0.14 | 0.30 | 1.14 | 0.04 | 0.01 | 0.05 | 0.00 | 0.00 | 0.04 |
| K03753$_{VIP\geq0.8\ (+)}$ | 0.72 | 1.32 | 0.96 | 1.02 | 1.07 | 1.58 | 0.03 | 0.02 | 0.01 | 0.02 | -0.03 | 0.05 |
| K21071$_{VIP\geq0.8\ (+)}$ | 0.81 | 0.92 | 0.86 | 0.31 | 0.93 | 1.03 | 0.01 | 0.01 | 0.00 | 0.00 | 0.04 | 0.01 |
| K01997$_{VIP\geq0.8\ (+)}$ | 0.91 | 0.98 | 0.84 | 0.26 | 0.50 | 1.37 | -0.01 | 0.01 | 0.00 | 0.00 | 0.02 | 0.04 |
| K05833$_{VIP\geq0.8\ (+)}$ | 0.89 | 0.95 | 0.81 | 0.28 | 0.44 | 1.28 | 0.01 | 0.01 | 0.00 | 0.01 | 0.00 | 0.03 |
| K04070$_{VIP\geq0.8\ (+)}$ | 0.92 | 0.96 | 0.88 | 0.26 | 0.69 | 1.17 | 0.02 | 0.02 | 0.01 | 0.00 | -0.03 | 0.02 |

| | | | | | | | | | | | | |
|---|---|---|---|---|---|---|---|---|---|---|---|---|
| $K03564_{VIP≥0.8\ (+)}$ | 0.82 | 0.38 | 0.94 | 0.96 | 0.67 | 1.12 | 0.03 | 0.00 | 0.00 | 0.02 | -0.01 | 0.04 |
| $K16785_{VIP≥0.8\ (+)}$ | 1.14 | 0.95 | 0.89 | 0.27 | 0.47 | 1.06 | 0.03 | 0.00 | 0.01 | 0.01 | -0.02 | 0.00 |
| $K08963_{VIP≥0.8\ (+)}$ | 0.83 | 1.12 | 0.92 | 0.30 | 0.24 | 0.96 | 0.02 | 0.00 | 0.01 | 0.01 | 0.00 | 0.01 |
| $K03724_{VIP≥0.8\ (+)}$ | 1.12 | 0.77 | 0.91 | 0.82 | 0.89 | 0.27 | -0.03 | 0.02 | 0.01 | 0.02 | 0.04 | 0.01 |
| $K01915_{VIP≥0.8\ (+)}$ | 1.31 | 0.78 | 1.04 | 0.25 | 1.43 | 0.92 | 0.04 | 0.02 | 0.03 | 0.00 | -0.06 | 0.03 |
| $K03538_{VIP≥0.8\ (+)}$ | 0.80 | 0.98 | 1.01 | 0.32 | 0.90 | 1.08 | 0.03 | 0.01 | 0.01 | 0.00 | 0.02 | 0.02 |
| $K14112_{VIP≥0.8\ (+)}$ | 1.14 | 0.78 | 0.88 | 0.76 | 0.90 | 1.28 | 0.04 | 0.00 | 0.02 | -0.02 | 0.01 | 0.03 |
| $K14111_{VIP≥0.8\ (+)}$ | 0.83 | 0.77 | 0.88 | 0.64 | 0.86 | 1.25 | 0.03 | 0.00 | 0.01 | -0.02 | 0.01 | 0.02 |
| $K07165_{VIP≥0.8\ (+)}$ | 0.91 | 1.01 | 1.22 | 0.30 | 0.98 | 0.67 | 0.01 | -0.01 | 0.02 | 0.00 | 0.04 | -0.02 |
| $K14118_{VIP≥0.8\ (+)}$ | 0.81 | 0.71 | 0.86 | 0.59 | 0.80 | 0.83 | 0.03 | 0.01 | 0.00 | -0.01 | 0.01 | 0.02 |
| $K23351_{VIP≥0.8\ (+)}$ | 1.63 | 0.38 | 0.85 | 0.50 | 1.42 | 1.20 | 0.06 | 0.00 | -0.02 | -0.01 | 0.00 | 0.01 |
| $K11260_{VIP≥0.8\ (+)}$ | 1.07 | 0.71 | 0.92 | 0.77 | 0.83 | 1.33 | 0.04 | 0.00 | 0.02 | -0.02 | 0.02 | -0.04 |
| $K16850_{VIP≥0.8\ (-)}$ | 0.20 | 1.65 | 0.20 | 0.94 | 1.89 | 0.85 | 0.01 | -0.04 | -0.01 | -0.02 | -0.09 | -0.03 |
| $K03154_{VIP≥0.8\ (-)}$ | 0.47 | 1.24 | 1.30 | 2.14 | 1.63 | 0.16 | -0.01 | 0.03 | -0.05 | -0.05 | -0.05 | 0.00 |
| $K00940_{VIP≥0.8\ (-)}$ | 1.22 | 0.80 | 1.32 | 1.27 | 0.45 | 0.87 | -0.04 | -0.02 | -0.04 | -0.03 | 0.00 | -0.02 |
| $K12998_{VIP≥0.8\ (-)}$ | 0.99 | 0.83 | 1.24 | 1.43 | 1.05 | 0.12 | -0.04 | 0.00 | -0.03 | -0.03 | -0.02 | 0.00 |
| $K07058_{VIP≥0.8\ (-)}$ | 1.56 | 0.85 | 0.83 | 2.54 | 1.14 | 0.61 | -0.06 | 0.02 | -0.02 | -0.06 | -0.04 | 0.01 |
| $K02919_{VIP≥0.8\ (-)}$ | 0.90 | 0.19 | 1.93 | 1.94 | 1.87 | 0.87 | 0.03 | 0.00 | -0.07 | -0.04 | -0.05 | 0.02 |
| $K01092_{VIP≥0.8\ (-)}$ | 1.03 | 1.38 | 1.60 | 2.38 | 0.81 | 0.35 | -0.02 | 0.03 | -0.05 | -0.05 | -0.04 | 0.01 |
| $K13283_{VIP≥0.8\ (-)}$ | 0.63 | 0.87 | 2.07 | 1.16 | 0.90 | 1.17 | 0.02 | -0.01 | -0.08 | -0.02 | -0.02 | -0.03 |
| $K00077_{VIP≥0.8\ (-)}$ | 1.46 | 0.91 | 0.94 | 1.57 | 1.11 | 0.76 | -0.05 | 0.01 | -0.02 | -0.03 | -0.05 | -0.02 |
| $K01297_{VIP≥0.8\ (-)}$ | 0.28 | 0.97 | 1.11 | 1.42 | 0.98 | 0.38 | 0.00 | 0.01 | -0.02 | -0.03 | -0.04 | -0.01 |
| $K10563_{VIP≥0.8\ (-)}$ | 1.07 | 0.92 | 0.94 | 1.34 | 0.58 | 0.22 | -0.04 | -0.01 | -0.01 | -0.03 | -0.01 | 0.00 |
| $K09764_{VIP≥0.8\ (-)}$ | 0.28 | 0.99 | 1.18 | 1.14 | 1.07 | 0.32 | 0.01 | 0.01 | -0.03 | -0.03 | -0.02 | -0.01 |
| $K02078_{VIP≥0.8\ (-)}$ | 0.88 | 0.31 | 0.82 | 1.14 | 1.09 | 0.50 | -0.03 | 0.00 | -0.02 | -0.03 | 0.01 | 0.00 |
| $K15894_{VIP≥0.8\ (-)}$ | 1.92 | 0.57 | 1.12 | 1.08 | 0.57 | 1.23 | -0.07 | 0.01 | -0.04 | -0.02 | -0.03 | 0.04 |
| $K00057_{VIP≥0.8\ (-)}$ | 1.24 | 1.37 | 1.25 | 1.03 | 1.78 | 0.50 | -0.05 | 0.03 | -0.05 | -0.02 | -0.07 | 0.01 |
| $K01139_{VIP≥0.8\ (-)}$ | 1.40 | 0.92 | 0.89 | 1.04 | 0.64 | 1.02 | -0.05 | 0.01 | -0.01 | -0.02 | -0.03 | -0.03 |
| $K22452_{VIP≥0.8\ (-)}$ | 1.79 | 0.92 | 0.87 | 1.35 | 0.57 | 0.94 | -0.07 | 0.01 | 0.00 | -0.03 | 0.02 | -0.03 |
| $K00390_{VIP≥0.8\ (-)}$ | 0.41 | 1.03 | 1.09 | 1.06 | 0.86 | 1.21 | 0.01 | 0.00 | -0.03 | -0.02 | -0.01 | -0.04 |

| | | | | | | | | | | | |
|---|---|---|---|---|---|---|---|---|---|---|---|---|
| $K01091_{VIP≥0.8\ (-)}$ | 0.82 | 0.84 | 0.97 | 1.44 | 0.86 | 0.53 | -0.03 | 0.01 | 0.00 | -0.03 | -0.03 | 0.01 |
| $K02759_{VIP≥0.8\ (-)}$ | 0.94 | 0.88 | 0.87 | 0.79 | 0.83 | 0.68 | 0.00 | 0.00 | -0.01 | -0.01 | -0.03 | -0.02 |
| $K21030_{VIP≥0.8\ (-)}$ | 0.43 | 0.85 | 0.50 | 1.06 | 2.07 | 1.40 | 0.00 | -0.01 | -0.02 | 0.02 | -0.10 | -0.04 |
| $K03320_{VIP≥0.8\ (-)}$ | 1.16 | 0.74 | 0.89 | 0.38 | 1.08 | 1.09 | -0.04 | 0.01 | -0.03 | 0.00 | 0.05 | -0.03 |
| $K01223_{VIP≥0.8\ (-)}$ | 0.86 | 0.88 | 0.88 | 0.83 | 0.79 | 1.04 | -0.02 | 0.01 | -0.01 | -0.02 | -0.03 | -0.02 |
| $K23535_{VIP≥0.8\ (-)}$ | 0.75 | 0.90 | 0.80 | 0.37 | 0.81 | 1.43 | -0.01 | 0.01 | -0.01 | -0.01 | 0.00 | 0.04 |
| $K01759_{VIP≥0.8\ (-)}$ | 1.06 | 1.45 | 0.62 | 0.78 | 1.02 | 0.82 | 0.01 | -0.02 | -0.01 | -0.01 | -0.02 | 0.01 |
| $K03741_{VIP≥0.8\ (-)}$ | 0.63 | 1.19 | 0.93 | 0.82 | 0.47 | 0.99 | 0.02 | -0.01 | -0.01 | -0.01 | 0.01 | -0.03 |
| $K03709_{VIP≥0.8\ (-)}$ | 1.05 | 2.07 | 0.71 | 1.08 | 1.35 | 1.42 | 0.03 | 0.05 | -0.02 | -0.03 | -0.03 | -0.03 |
| $K02822_{VIP≥0.8\ (-)}$ | 0.88 | 0.88 | 1.03 | 0.79 | 0.31 | 0.96 | 0.01 | 0.02 | -0.03 | -0.02 | -0.01 | -0.02 |
| $K00651_{VIP≥0.8\ (-)}$ | 0.82 | 1.53 | 1.11 | 0.68 | 0.13 | 1.12 | -0.03 | 0.04 | -0.03 | -0.01 | 0.00 | -0.04 |
| $K00820_{VIP≥0.8\ (-)}$ | 1.35 | 0.95 | 0.93 | 0.63 | 1.47 | 0.43 | 0.04 | 0.01 | -0.03 | -0.01 | -0.07 | -0.01 |
| $K00145_{VIP≥0.8\ (-)}$ | 0.76 | 1.22 | 0.93 | 1.28 | 0.74 | 0.87 | 0.03 | 0.03 | 0.00 | -0.03 | -0.03 | -0.03 |
| $K02025_{VIP≥0.8\ (-)}$ | 0.70 | 1.00 | 0.90 | 0.42 | 0.87 | 0.80 | -0.01 | 0.00 | -0.02 | -0.01 | -0.02 | 0.01 |
| $K02034_{VIP≥0.8\ (-)}$ | 0.75 | 1.12 | 0.77 | 0.95 | 0.98 | 1.28 | 0.02 | -0.01 | -0.02 | -0.02 | -0.02 | 0.02 |
| $K07005_{VIP≥0.8\ (-)}$ | 1.41 | 1.23 | 0.85 | 1.12 | 0.31 | 1.54 | 0.05 | 0.03 | -0.03 | -0.02 | -0.01 | -0.03 |
| $K09793_{VIP≥0.8}$ | 0.88 | 1.03 | 1.00 | 0.99 | 1.79 | 1.35 | 0.03 | -0.02 | 0.02 | -0.02 | -0.06 | 0.04 |
| $K00626_{VIP≥0.8}$ | 0.85 | 0.97 | 0.97 | 0.85 | 0.83 | 1.42 | 0.02 | 0.01 | 0.00 | 0.02 | -0.03 | -0.03 |
| $K07387_{VIP≥0.8}$ | 1.56 | 0.61 | 1.42 | 0.87 | 1.23 | 0.87 | 0.06 | -0.01 | 0.05 | -0.02 | -0.05 | 0.03 |
| $K00441_{VIP≥0.8}$ | 0.16 | 1.19 | 1.68 | 0.89 | 0.86 | 0.92 | 0.00 | 0.02 | -0.06 | -0.02 | 0.03 | 0.02 |
| $K19304_{VIP≥0.8}$ | 1.06 | 1.06 | 0.11 | 2.15 | 1.15 | 1.48 | -0.04 | -0.03 | 0.00 | -0.05 | 0.02 | 0.05 |
| $K21498_{VIP≥0.8}$ | 1.18 | 0.63 | 1.20 | 1.48 | 1.05 | 1.10 | 0.04 | 0.01 | -0.05 | -0.03 | 0.04 | -0.03 |
| $K04762_{VIP≥0.8}$ | 0.19 | 0.98 | 1.14 | 1.36 | 1.15 | 0.81 | 0.01 | 0.02 | -0.04 | -0.03 | 0.01 | -0.01 |
| $K09116_{VIP≥0.8}$ | 0.96 | 0.88 | 0.97 | 0.67 | 1.32 | 0.79 | -0.03 | 0.00 | 0.00 | -0.02 | -0.04 | 0.01 |
| $K10761_{VIP≥0.8}$ | 0.91 | 1.20 | 1.17 | 0.15 | 0.72 | 0.94 | 0.03 | 0.02 | -0.03 | 0.00 | -0.01 | 0.02 |
| $K20742_{VIP≥0.8}$ | 1.32 | 1.08 | 1.42 | 0.64 | 1.37 | 0.80 | 0.05 | -0.03 | 0.05 | 0.02 | -0.02 | -0.01 |
| $K03208_{VIP≥0.8}$ | 1.89 | 1.11 | 0.57 | 1.01 | 0.58 | 0.87 | -0.07 | 0.02 | 0.00 | -0.02 | 0.02 | 0.00 |
| $K04047_{VIP≥0.8}$ | 1.07 | 1.01 | 1.23 | 0.19 | 0.66 | 1.17 | -0.02 | -0.01 | 0.03 | 0.00 | 0.03 | -0.04 |
| $K00854_{VIP≥0.8}$ | 0.66 | 0.88 | 1.02 | 0.37 | 0.98 | 1.07 | 0.00 | 0.02 | -0.03 | -0.01 | -0.03 | 0.03 |
| $K12952_{VIP≥0.8}$ | 0.92 | 0.80 | 1.00 | 0.10 | 1.33 | 0.91 | -0.02 | 0.00 | 0.02 | 0.00 | -0.06 | -0.01 |

| | | | | | | | | | | | | |
|---|---|---|---|---|---|---|---|---|---|---|---|---|
| $K16787_{VIP≥0.8}$ | 0.95 | 0.98 | 0.88 | 0.52 | 0.75 | 0.90 | 0.02 | 0.00 | 0.00 | -0.01 | -0.03 | 0.02 |
| $K09691_{VIP≥0.8}$ | 0.89 | 1.32 | 1.18 | 0.35 | 0.81 | 0.60 | 0.03 | 0.03 | -0.04 | 0.01 | -0.01 | -0.02 |
| $K02099_{VIP≥0.8}$ | 1.08 | 1.20 | 1.01 | 0.85 | 0.43 | 0.78 | 0.03 | 0.03 | -0.02 | 0.02 | -0.01 | -0.02 |
| $K02035_{VIP≥0.8}$ | 0.58 | 1.06 | 0.98 | 1.18 | 0.62 | 0.85 | 0.01 | 0.01 | -0.02 | -0.02 | -0.02 | 0.02 |
| $K07458_{VIP≥0.8}$ | 1.13 | 0.97 | 0.77 | 1.26 | 0.33 | 0.92 | -0.04 | 0.02 | -0.03 | -0.03 | 0.01 | 0.01 |
| K04796 | 0.61 | 0.79 | 0.97 | 0.99 | 0.73 | 1.16 | 0.02 | 0.01 | 0.00 | -0.02 | 0.00 | 0.03 |
| K14095 | 0.14 | 0.84 | 0.90 | 0.73 | 0.73 | 0.99 | 0.00 | 0.01 | 0.01 | -0.02 | 0.02 | 0.02 |
| K07503 | 0.80 | 0.74 | 1.26 | 0.95 | 0.41 | 0.74 | 0.03 | 0.00 | 0.04 | -0.02 | 0.01 | 0.00 |
| K03105 | 0.76 | 0.97 | 0.97 | 0.33 | 0.64 | 1.30 | 0.03 | 0.00 | 0.00 | 0.00 | 0.02 | 0.04 |
| K03057 | 1.03 | 0.72 | 0.88 | 0.69 | 0.75 | 1.07 | 0.04 | 0.00 | -0.01 | -0.02 | 0.01 | 0.02 |
| K08094 | 1.13 | 1.33 | 0.70 | 0.53 | 0.73 | 1.18 | 0.03 | 0.03 | 0.03 | 0.00 | 0.00 | 0.03 |
| K11041 | 0.84 | 1.81 | 0.21 | 0.40 | 0.63 | 1.46 | 0.02 | 0.04 | 0.01 | 0.00 | -0.03 | 0.03 |
| K08234 | 2.33 | 0.91 | 0.57 | 0.68 | 1.37 | 0.35 | 0.09 | 0.02 | -0.02 | -0.02 | -0.04 | -0.01 |
| K16927 | 0.75 | 0.70 | 0.77 | 0.88 | 1.03 | 1.10 | 0.01 | 0.00 | 0.00 | -0.02 | -0.05 | 0.02 |
| K01500 | 0.44 | 1.24 | 0.73 | 0.22 | 1.14 | 1.16 | 0.00 | 0.03 | 0.01 | 0.00 | -0.05 | 0.02 |
| K19048 | 0.77 | 1.11 | 0.87 | 0.53 | 0.88 | 0.66 | -0.01 | 0.02 | -0.03 | 0.01 | -0.04 | 0.00 |
| K07172 | 0.58 | 1.10 | 0.87 | 0.78 | 0.77 | 1.25 | 0.02 | 0.03 | 0.02 | 0.02 | 0.03 | 0.01 |
| K09780 | 0.81 | 0.74 | 0.73 | 0.99 | 0.63 | 0.92 | -0.02 | -0.01 | -0.03 | -0.02 | 0.02 | -0.03 |
| K11105 | 0.94 | 1.55 | 0.40 | 1.52 | 0.62 | 0.45 | -0.02 | -0.04 | -0.01 | -0.03 | 0.02 | 0.01 |
| K01077 | 0.76 | 0.58 | 0.87 | 0.71 | 1.82 | 1.54 | 0.02 | -0.01 | 0.03 | -0.01 | -0.08 | 0.05 |
| K23393 | 0.83 | 0.92 | 0.67 | 0.59 | 0.64 | 1.09 | 0.01 | 0.02 | 0.00 | 0.01 | -0.01 | 0.01 |
| K10117 | 0.78 | 0.98 | 1.06 | 0.25 | 0.93 | 0.78 | -0.02 | 0.01 | 0.01 | 0.00 | -0.02 | 0.02 |
| K15051 | 1.06 | 1.01 | 0.69 | 0.54 | 0.52 | 0.92 | 0.03 | 0.01 | 0.01 | 0.01 | 0.00 | 0.03 |
| K12950 | 0.76 | 1.01 | 0.90 | 1.22 | 0.79 | 0.40 | -0.03 | 0.00 | -0.01 | -0.02 | 0.03 | -0.01 |
| K02086 | 0.74 | 0.83 | 0.86 | 0.55 | 0.35 | 0.81 | 0.00 | 0.01 | 0.00 | 0.01 | -0.02 | 0.00 |
| K20866 | 0.73 | 0.80 | 1.37 | 1.31 | 1.77 | 0.40 | 0.00 | 0.00 | 0.04 | 0.03 | 0.09 | 0.00 |
| K03529 | 0.88 | 1.23 | 0.88 | 0.51 | 0.20 | 0.76 | -0.02 | 0.03 | 0.00 | 0.01 | 0.00 | -0.01 |
| K02027 | 0.73 | 0.95 | 0.94 | 0.48 | 1.44 | 0.71 | 0.01 | 0.00 | -0.02 | -0.01 | -0.05 | -0.01 |
| K08602 | 0.77 | 1.03 | 0.91 | 0.31 | 0.72 | 1.05 | 0.00 | 0.00 | 0.01 | 0.00 | 0.00 | 0.02 |
| K06885 | 0.66 | 0.83 | 1.05 | 0.31 | 0.84 | 0.45 | -0.02 | -0.02 | -0.01 | -0.01 | -0.04 | 0.01 |
| K23536 | 0.82 | 0.87 | 0.78 | 0.39 | 0.75 | 1.67 | -0.01 | 0.00 | -0.01 | -0.01 | -0.01 | 0.04 |
| K04564 | 0.90 | 0.70 | 0.98 | 2.02 | 0.67 | 0.80 | -0.02 | 0.00 | -0.02 | -0.05 | 0.03 | 0.03 |
| K07464 | 0.94 | 1.12 | 0.89 | 0.32 | 0.57 | 0.78 | 0.02 | 0.00 | -0.01 | 0.00 | 0.02 | 0.00 |
| K16568 | 0.77 | 0.90 | 0.88 | 1.29 | 0.29 | 0.58 | 0.03 | 0.00 | 0.00 | -0.02 | 0.00 | -0.02 |

| | | | | | | | | | | | | |
|---|---|---|---|---|---|---|---|---|---|---|---|---|
| K01686 | 0.68 | 0.74 | 0.87 | 1.11 | 0.72 | 2.19 | -0.01 | 0.02 | 0.02 | -0.03 | 0.03 | 0.07 |
| K03803 | 0.70 | 0.32 | 0.96 | 2.18 | 1.54 | 0.40 | 0.02 | -0.01 | -0.03 | -0.05 | -0.07 | 0.00 |
| K03972 | 0.75 | 1.06 | 0.48 | 2.73 | 1.59 | 0.74 | -0.03 | 0.03 | -0.01 | -0.06 | -0.07 | -0.02 |
| K07316 | 1.72 | 0.90 | 0.74 | 0.51 | 1.50 | 0.63 | 0.06 | 0.02 | 0.02 | 0.01 | 0.05 | 0.02 |
| K01733 | 1.20 | 1.53 | 0.83 | 0.47 | 0.48 | 0.78 | -0.04 | 0.04 | 0.00 | 0.00 | -0.02 | 0.02 |
| K00826 | 0.89 | 0.92 | 1.09 | 0.75 | 0.18 | 0.30 | -0.03 | 0.02 | 0.02 | -0.01 | 0.00 | -0.01 |
| K02115 | 0.75 | 1.18 | 1.08 | 0.81 | 0.10 | 0.69 | 0.02 | 0.02 | -0.03 | -0.01 | 0.00 | -0.02 |
| K02884 | 0.68 | 0.22 | 0.67 | 1.28 | 1.51 | 1.08 | -0.03 | 0.00 | -0.02 | -0.02 | -0.04 | 0.00 |
| K07979 | 0.75 | 1.36 | 0.37 | 0.60 | 1.33 | 1.13 | -0.01 | 0.03 | -0.01 | 0.01 | -0.01 | 0.01 |
| K02916 | 1.17 | 0.65 | 0.49 | 0.69 | 1.47 | 0.84 | 0.04 | 0.01 | 0.00 | -0.02 | -0.02 | 0.00 |
| K00067 | 1.30 | 1.80 | 0.80 | 1.30 | 0.08 | 0.43 | -0.05 | 0.05 | -0.03 | -0.03 | 0.00 | -0.01 |
| K02029 | 0.59 | 0.97 | 0.89 | 0.48 | 0.77 | 1.03 | 0.00 | 0.00 | -0.02 | -0.01 | -0.03 | 0.01 |
| K07478 | 0.78 | 0.99 | 1.06 | 0.99 | 0.32 | 0.54 | -0.03 | 0.01 | -0.03 | -0.02 | 0.01 | -0.01 |
| K03574 | 0.69 | 0.65 | 0.99 | 2.53 | 1.44 | 0.05 | 0.02 | 0.00 | -0.02 | -0.06 | -0.03 | 0.00 |
| K14122 | 0.27 | 0.73 | 1.01 | 0.67 | 0.76 | 0.81 | 0.01 | 0.00 | 0.02 | -0.02 | -0.01 | 0.01 |
| K17290 | 1.15 | 0.39 | 0.79 | 0.60 | 0.90 | 0.79 | -0.04 | 0.00 | -0.01 | 0.01 | -0.03 | 0.02 |
| K23997 | 0.59 | 1.03 | 0.65 | 0.43 | 0.76 | 1.48 | 0.01 | 0.03 | 0.01 | -0.01 | 0.03 | 0.01 |
| K19689 | 0.71 | 0.94 | 0.94 | 0.35 | 0.73 | 0.78 | 0.01 | 0.00 | -0.01 | 0.00 | -0.01 | 0.01 |
| K02775 | 0.44 | 0.28 | 0.86 | 1.19 | 0.72 | 0.72 | 0.00 | 0.00 | -0.03 | 0.03 | 0.03 | 0.00 |
| K10119 | 0.65 | 0.92 | 0.74 | 0.22 | 0.17 | 0.96 | 0.01 | 0.02 | 0.00 | 0.00 | 0.01 | 0.00 |
| K07335 | 0.78 | 0.80 | 0.90 | 0.37 | 0.73 | 0.86 | -0.02 | 0.02 | -0.01 | 0.00 | -0.03 | 0.02 |
| K07075 | 0.47 | 1.45 | 1.41 | 0.12 | 0.80 | 0.79 | 0.02 | 0.03 | 0.05 | 0.00 | -0.02 | 0.02 |
| K01952 | 0.95 | 0.87 | 0.76 | 0.20 | 0.79 | 0.63 | 0.02 | 0.01 | 0.00 | 0.00 | -0.02 | 0.02 |
| K07138 | 0.33 | 0.80 | 1.69 | 0.60 | 0.68 | 2.00 | 0.01 | 0.01 | 0.07 | 0.01 | 0.03 | 0.06 |
| K03723 | 0.87 | 0.82 | 0.76 | 0.29 | 0.62 | 0.71 | -0.03 | 0.02 | 0.00 | 0.00 | 0.03 | 0.02 |
| K01940 | 0.50 | 0.94 | 0.76 | 0.76 | 0.61 | 1.09 | 0.01 | 0.02 | 0.00 | -0.02 | -0.03 | -0.03 |
| K14155 | 0.89 | 0.80 | 0.77 | 0.16 | 0.99 | 0.66 | 0.01 | 0.01 | 0.00 | 0.00 | -0.04 | -0.02 |
| K07284 | 0.72 | 0.60 | 0.41 | 0.46 | 0.78 | 1.36 | 0.02 | 0.01 | 0.02 | 0.01 | -0.01 | -0.03 |

Partial Least Squares analysis was performed with 2 latent components. Subscript VIP≥0.8 denotes microbial genera that had VIP ≥ 0.8 in 4 or more timepoints. Subscripts (+) and (-) represent microbial genera that had positive or negative regression coefficient in 4 or more timepoints, respectively.

**Trait: Average Daily Weight Gain (ADG); Predictors: 133 microbial genes (MG)**

| | VIP | Regression coefficient |
|---|---|---|

| Microbial genes | T1 | T2 | T3 | T4 | T5 | T6 | T1 | T2 | T3 | T4 | T5 | T6 |
|---|---|---|---|---|---|---|---|---|---|---|---|---|
| K03503$_{VIP\geq0.8\ (+)}$ | 0.48 | 1.86 | 0.24 | 2.07 | 1.09 | 1.55 | 0.02 | 0.07 | 0.01 | 0.05 | 0.04 | 0.05 |
| K16850$_{VIP\geq0.8\ (+)}$ | 0.73 | 1.33 | 0.75 | 0.89 | 1.86 | 1.52 | 0.03 | 0.05 | 0.02 | 0.00 | 0.07 | 0.05 |
| K04518$_{VIP\geq0.8\ (+)}$ | 0.87 | 0.42 | 0.72 | 1.83 | 0.93 | 1.33 | 0.03 | 0.01 | 0.02 | 0.04 | 0.04 | 0.02 |
| K03972$_{VIP\geq0.8\ (+)}$ | 0.98 | 0.52 | 0.52 | 2.72 | 1.22 | 0.83 | 0.04 | -0.02 | 0.02 | 0.07 | 0.05 | 0.01 |
| K02066$_{VIP\geq0.8\ (+)}$ | 0.97 | 0.79 | 1.01 | 1.46 | 0.13 | 1.28 | 0.04 | 0.03 | 0.03 | 0.02 | -0.01 | 0.02 |
| K24131$_{VIP\geq0.8\ (+)}$ | 1.09 | 1.03 | 0.91 | 1.40 | 1.00 | 1.69 | 0.02 | 0.01 | 0.01 | 0.04 | 0.02 | 0.08 |
| K00077$_{VIP\geq0.8\ (+)}$ | 1.13 | 0.87 | 1.07 | 1.39 | 0.98 | 1.15 | 0.04 | -0.02 | 0.02 | 0.04 | 0.03 | 0.03 |
| K03709$_{VIP\geq0.8\ (+)}$ | 0.20 | 1.57 | 2.00 | 1.46 | 1.90 | 1.85 | -0.01 | -0.05 | 0.07 | 0.04 | 0.08 | 0.07 |
| K12962$_{VIP\geq0.8\ (+)}$ | 1.54 | 1.33 | 0.68 | 0.32 | 0.86 | 1.15 | 0.06 | 0.05 | 0.02 | 0.00 | 0.02 | 0.04 |
| K01703$_{VIP\geq0.8\ (+)}$ | 1.08 | 0.99 | 1.00 | 0.68 | 1.28 | 0.88 | 0.03 | 0.00 | 0.03 | 0.02 | 0.04 | 0.00 |
| K00057$_{VIP\geq0.8\ (+)}$ | 1.24 | 0.86 | 1.45 | 0.83 | 1.59 | 0.66 | 0.04 | -0.03 | 0.04 | 0.01 | 0.06 | -0.01 |
| K00067$_{VIP\geq0.8\ (+)}$ | 1.59 | 1.11 | 1.37 | 1.62 | 0.53 | 0.85 | 0.06 | -0.05 | 0.04 | 0.04 | -0.02 | 0.03 |
| K03778$_{VIP\geq0.8\ (+)}$ | 1.09 | 1.40 | 1.12 | 0.23 | 0.50 | 1.63 | 0.01 | 0.04 | 0.03 | 0.00 | 0.00 | 0.07 |
| K01092$_{VIP\geq0.8\ (+)}$ | 1.01 | 2.04 | 1.71 | 2.35 | 0.79 | 0.20 | 0.03 | -0.07 | 0.06 | 0.06 | 0.03 | 0.00 |
| K03320$_{VIP\geq0.8\ (+)}$ | 1.18 | 1.12 | 1.12 | 0.42 | 1.12 | 1.51 | 0.04 | 0.02 | 0.03 | 0.00 | -0.04 | 0.04 |
| K21030$_{VIP\geq0.8\ (+)}$ | 1.11 | 1.07 | 0.75 | 0.97 | 0.88 | 2.36 | 0.04 | 0.02 | 0.02 | -0.01 | 0.04 | 0.10 |
| K00548$_{VIP\geq0.8\ (+)}$ | 1.04 | 0.91 | 1.11 | 0.80 | 0.74 | 1.31 | 0.03 | 0.00 | 0.02 | 0.02 | -0.01 | 0.00 |
| K00390$_{VIP\geq0.8\ (+)}$ | 1.02 | 1.21 | 1.04 | 1.17 | 0.58 | 1.11 | 0.00 | 0.02 | 0.02 | 0.03 | -0.02 | 0.04 |
| K12998$_{VIP\geq0.8\ (+)}$ | 1.15 | 0.82 | 1.25 | 0.91 | 0.61 | 0.24 | 0.04 | 0.00 | 0.04 | 0.02 | 0.02 | -0.01 |
| K02107$_{VIP\geq0.8\ (+)}$ | 1.32 | 1.16 | 0.08 | 0.71 | 1.30 | 1.16 | 0.05 | 0.05 | 0.00 | 0.01 | 0.05 | 0.02 |
| K13993$_{VIP\geq0.8\ (+)}$ | 1.25 | 1.10 | 1.48 | 0.89 | 0.72 | 0.58 | 0.04 | 0.01 | 0.04 | 0.02 | -0.02 | 0.00 |
| K03585$_{VIP\geq0.8\ (+)}$ | 0.97 | 0.30 | 0.10 | 1.39 | 0.87 | 1.37 | 0.03 | 0.00 | 0.00 | 0.02 | -0.03 | 0.04 |
| K00052$_{VIP\geq0.8\ (+)}$ | 1.17 | 0.93 | 1.10 | 0.46 | 0.80 | 1.13 | 0.01 | -0.01 | 0.03 | 0.00 | 0.01 | 0.02 |
| K03394$_{VIP\geq0.8\ (+)}$ | 0.83 | 1.42 | 1.69 | 0.88 | 0.85 | 0.86 | 0.03 | 0.03 | 0.05 | 0.02 | 0.00 | -0.04 |
| K07118$_{VIP\geq0.8\ (+)}$ | 1.11 | 1.52 | 1.03 | 0.20 | 0.80 | 0.64 | 0.01 | 0.03 | 0.03 | 0.00 | 0.01 | 0.02 |
| K00766$_{VIP\geq0.8\ (+)}$ | 0.77 | 0.80 | 0.97 | 0.82 | 1.21 | 0.78 | 0.00 | -0.02 | 0.02 | 0.02 | 0.04 | 0.00 |
| K21498$_{VIP\geq0.8\ (+)}$ | 0.94 | 0.76 | 1.16 | 0.88 | 0.71 | 0.88 | -0.02 | 0.02 | 0.03 | 0.02 | -0.02 | 0.04 |
| K14155$_{VIP\geq0.8\ (+)}$ | 0.96 | 1.08 | 0.82 | 0.34 | 1.06 | 0.96 | -0.01 | 0.01 | 0.02 | 0.01 | 0.02 | 0.03 |

| | | | | | | | | | | | | |
|---|---|---|---|---|---|---|---|---|---|---|---|---|
| $K01297_{VIP≥0.8\ (+)}$ | 0.94 | 1.03 | 1.03 | 1.76 | 0.71 | 0.77 | -0.02 | -0.02 | 0.03 | 0.05 | 0.02 | 0.03 |
| $K09116_{VIP≥0.8\ (+)}$ | 1.10 | 1.13 | 0.95 | 0.78 | 1.81 | 0.94 | 0.04 | -0.02 | 0.03 | 0.02 | 0.07 | -0.01 |
| $K19271_{VIP≥0.8\ (+)}$ | 0.74 | 0.81 | 1.28 | 1.03 | 1.12 | 0.74 | -0.02 | -0.01 | 0.04 | 0.03 | 0.04 | 0.02 |
| $K02884_{VIP≥0.8\ (+)}$ | 0.84 | 0.94 | 0.33 | 0.77 | 0.90 | 0.91 | 0.03 | 0.03 | 0.01 | 0.02 | 0.04 | -0.01 |
| $K02115_{VIP≥0.8\ (+)}$ | 1.27 | 0.95 | 1.00 | 0.64 | 0.73 | 0.89 | -0.03 | -0.01 | 0.02 | 0.02 | 0.01 | 0.03 |
| $K00651_{VIP≥0.8\ (+)}$ | 1.14 | 1.01 | 1.05 | 0.44 | 0.84 | 0.78 | 0.03 | -0.03 | 0.02 | 0.01 | 0.00 | 0.02 |
| $K01223_{VIP≥0.8\ (+)}$ | 0.78 | 0.90 | 0.86 | 0.71 | 1.19 | 0.92 | 0.02 | -0.03 | 0.02 | 0.02 | 0.03 | 0.03 |
| $K00297_{VIP≥0.8\ (+)}$ | 1.04 | 1.21 | 0.83 | 0.85 | 0.97 | 0.64 | 0.02 | 0.01 | 0.01 | 0.02 | -0.03 | -0.01 |
| $K02114_{VIP≥0.8\ (+)}$ | 1.17 | 1.07 | 1.15 | 0.48 | 0.84 | 0.70 | -0.01 | 0.01 | 0.03 | -0.01 | 0.01 | 0.03 |
| $K07154_{VIP≥0.8\ (+)}$ | 0.86 | 1.40 | 0.09 | 1.68 | 0.89 | 0.98 | 0.03 | -0.05 | 0.00 | 0.03 | -0.03 | 0.04 |
| $K02837_{VIP≥0.8\ (+)}$ | 1.46 | 0.97 | 0.98 | 0.42 | 0.68 | 0.80 | 0.05 | -0.03 | -0.02 | 0.01 | 0.01 | 0.02 |
| $K01465_{VIP≥0.8\ (+)}$ | 0.96 | 1.25 | 0.65 | 0.87 | 0.95 | 1.11 | 0.00 | 0.02 | 0.00 | 0.02 | 0.01 | -0.04 |
| $K03685_{VIP≥0.8\ (+)}$ | 1.22 | 1.13 | 1.04 | 0.64 | 0.89 | 0.75 | 0.02 | 0.01 | 0.03 | 0.02 | 0.00 | -0.03 |
| $K07025_{VIP≥0.8\ (+)}$ | 1.06 | 1.08 | 0.77 | 0.49 | 1.04 | 2.03 | 0.03 | -0.03 | 0.03 | 0.01 | -0.04 | 0.08 |
| $K02034_{VIP≥0.8\ (+)}$ | 0.66 | 1.40 | 1.25 | 0.71 | 1.23 | 0.88 | -0.02 | 0.02 | 0.03 | 0.02 | 0.04 | -0.03 |
| $K23535_{VIP≥0.8\ (+)}$ | 0.66 | 0.85 | 0.91 | 0.69 | 0.94 | 1.16 | 0.02 | -0.03 | 0.02 | 0.01 | 0.01 | -0.05 |
| $K01733_{VIP≥0.8\ (+)}$ | 1.52 | 1.01 | 0.80 | 0.08 | 0.85 | 1.18 | 0.05 | -0.03 | 0.01 | 0.00 | 0.00 | -0.04 |
| $K10218_{VIP≥0.8\ (+)}$ | 1.38 | 0.46 | 0.91 | 1.26 | 0.92 | 1.98 | -0.05 | 0.00 | 0.03 | 0.03 | 0.04 | -0.09 |
| $K07496_{VIP≥0.8\ (+)}$ | 1.14 | 1.42 | 1.38 | 0.89 | 0.85 | 0.53 | -0.04 | -0.06 | 0.05 | 0.02 | 0.02 | 0.01 |
| $K02029_{VIP≥0.8\ (+)}$ | 0.84 | 0.97 | 0.97 | 0.52 | 1.09 | 0.67 | -0.01 | 0.00 | 0.02 | 0.00 | 0.03 | -0.02 |
| $K22162_{VIP≥0.8\ (-)}$ | 1.35 | 1.48 | 1.19 | 1.28 | 1.90 | 0.90 | -0.05 | -0.05 | -0.04 | -0.02 | -0.08 | 0.01 |
| $K07138_{VIP≥0.8\ (-)}$ | 0.51 | 0.87 | 1.75 | 0.43 | 1.25 | 1.80 | -0.01 | -0.02 | -0.05 | -0.01 | -0.05 | -0.07 |
| $K01156_{VIP≥0.8\ (-)}$ | 1.57 | 0.53 | 1.32 | 1.14 | 1.83 | 0.91 | -0.05 | -0.02 | -0.04 | -0.03 | -0.07 | -0.01 |
| $K00003_{VIP≥0.8\ (-)}$ | 0.96 | 0.92 | 0.61 | 1.17 | 0.99 | 1.19 | -0.03 | -0.03 | -0.02 | -0.01 | 0.03 | -0.03 |
| $K08600_{VIP≥0.8\ (-)}$ | 0.91 | 1.09 | 0.82 | 0.96 | 0.68 | 0.97 | -0.03 | -0.04 | -0.02 | -0.01 | 0.02 | -0.02 |
| $K03737_{VIP≥0.8\ (-)}$ | 1.01 | 0.90 | 1.00 | 1.25 | 0.98 | 0.24 | -0.02 | 0.00 | -0.03 | -0.03 | -0.03 | 0.01 |
| $K02647_{VIP≥0.8\ (-)}$ | 0.86 | 0.90 | 0.56 | 0.86 | 0.62 | 0.86 | -0.03 | -0.03 | 0.00 | -0.01 | 0.01 | -0.02 |
| $K07387_{VIP≥0.8\ (-)}$ | 1.97 | 0.67 | 1.87 | 0.72 | 1.17 | 0.93 | -0.08 | -0.01 | -0.06 | 0.02 | 0.04 | -0.04 |
| $K00763_{VIP≥0.8\ (-)}$ | 0.82 | 0.99 | 0.74 | 0.94 | 1.00 | 0.53 | -0.01 | 0.01 | -0.01 | -0.01 | -0.02 | -0.01 |
| $K06867_{VIP≥0.8\ (-)}$ | 0.80 | 0.81 | 0.98 | 0.40 | 1.28 | 0.95 | -0.02 | 0.00 | 0.03 | -0.01 | -0.04 | -0.02 |

| | | | | | | | | | | | | |
|---|---|---|---|---|---|---|---|---|---|---|---|---|
| $K16785_{VIP\geq0.8\ (-)}$ | 0.97 | 0.96 | 0.57 | 0.89 | 0.83 | 0.82 | -0.03 | 0.00 | -0.01 | -0.01 | 0.02 | -0.01 |
| $K02355_{VIP\geq0.8\ (-)}$ | 0.95 | 0.92 | 1.14 | 0.46 | 0.84 | 0.46 | 0.00 | 0.00 | -0.04 | 0.00 | -0.02 | -0.02 |
| $K19755_{VIP\geq0.8\ (-)}$ | 1.12 | 0.95 | 0.75 | 0.38 | 1.03 | 0.85 | -0.04 | -0.01 | -0.02 | 0.00 | 0.02 | -0.01 |
| $K15051_{VIP\geq0.8\ (-)}$ | 0.67 | 0.93 | 0.41 | 0.97 | 0.91 | 0.91 | -0.01 | 0.00 | 0.00 | -0.01 | 0.02 | -0.04 |
| $K00266_{VIP\geq0.8\ (-)}$ | 1.11 | 0.97 | 0.82 | 1.04 | 1.02 | 0.21 | 0.02 | -0.01 | -0.02 | -0.02 | -0.03 | 0.01 |
| $K05919_{VIP\geq0.8\ (-)}$ | 1.00 | 1.03 | 0.89 | 0.71 | 0.96 | 1.08 | -0.03 | 0.02 | 0.02 | 0.00 | -0.02 | -0.02 |
| $K00684_{VIP\geq0.8\ (-)}$ | 0.91 | 0.70 | 0.81 | 0.92 | 0.18 | 1.44 | -0.03 | -0.01 | -0.03 | 0.01 | 0.00 | -0.06 |
| $K00609_{VIP\geq0.8\ (-)}$ | 0.78 | 0.51 | 1.15 | 0.82 | 1.56 | 1.22 | 0.00 | -0.01 | -0.04 | -0.02 | 0.06 | -0.05 |
| $K02470_{VIP\geq0.8\ (-)}$ | 1.17 | 0.92 | 0.86 | 0.26 | 0.66 | 0.83 | 0.02 | 0.00 | -0.03 | 0.01 | -0.02 | -0.03 |
| $K02912_{VIP\geq0.8}$ | 0.84 | 0.97 | 0.89 | 0.20 | 0.81 | 1.27 | -0.02 | -0.01 | 0.02 | 0.00 | 0.01 | -0.04 |
| $K00626_{VIP\geq0.8}$ | 0.81 | 0.83 | 0.75 | 1.25 | 1.25 | 0.98 | -0.02 | -0.02 | 0.00 | -0.03 | 0.04 | 0.03 |
| $K20866_{VIP\geq0.8}$ | 0.93 | 0.80 | 1.08 | 1.20 | 1.88 | 0.17 | 0.01 | 0.01 | -0.04 | -0.03 | -0.07 | 0.01 |
| $K02035_{VIP\geq0.8}$ | 0.96 | 0.96 | 1.18 | 0.92 | 1.18 | 0.52 | -0.02 | -0.01 | 0.03 | 0.03 | 0.03 | -0.02 |
| $K21908_{VIP\geq0.8}$ | 1.16 | 1.75 | 0.93 | 0.55 | 1.36 | 1.21 | -0.05 | -0.06 | 0.03 | 0.01 | 0.05 | -0.02 |
| $K00817_{VIP\geq0.8}$ | 1.27 | 0.86 | 0.99 | 0.38 | 1.15 | 0.95 | 0.03 | -0.02 | 0.01 | 0.01 | -0.02 | -0.02 |
| $K01756_{VIP\geq0.8}$ | 0.98 | 0.82 | 0.82 | 0.13 | 0.86 | 0.92 | -0.01 | -0.02 | 0.01 | 0.00 | 0.01 | -0.03 |
| $K00145_{VIP\geq0.8}$ | 1.06 | 0.94 | 0.74 | 1.19 | 1.00 | 0.80 | -0.01 | -0.03 | 0.00 | 0.03 | 0.02 | 0.02 |
| $K07005_{VIP\geq0.8}$ | 1.34 | 1.16 | 1.55 | 1.13 | 0.44 | 1.00 | -0.05 | -0.05 | 0.04 | 0.03 | -0.01 | 0.03 |
| $K09793_{VIP\geq0.8}$ | 0.85 | 0.84 | 0.79 | 0.76 | 1.33 | 1.06 | -0.02 | 0.03 | -0.02 | 0.02 | 0.05 | -0.04 |
| $K20742_{VIP\geq0.8}$ | 0.54 | 0.89 | 1.39 | 1.13 | 0.61 | 1.13 | -0.01 | 0.04 | -0.04 | -0.03 | 0.02 | 0.03 |
| $K19048_{VIP\geq0.8}$ | 0.71 | 0.92 | 1.35 | 0.83 | 0.95 | 0.38 | 0.02 | -0.03 | 0.04 | -0.01 | 0.02 | -0.02 |
| $K16329_{VIP\geq0.8}$ | 0.94 | 0.97 | 0.92 | 0.33 | 0.52 | 0.86 | -0.03 | -0.01 | 0.03 | 0.01 | 0.01 | 0.00 |
| $K19304_{VIP\geq0.8}$ | 0.87 | 0.86 | 0.84 | 1.58 | 0.19 | 0.79 | 0.02 | 0.03 | -0.03 | 0.04 | -0.01 | -0.03 |
| $K01678_{VIP\geq0.8}$ | 1.19 | 1.02 | 0.62 | 0.79 | 0.84 | 1.15 | -0.04 | 0.02 | -0.01 | 0.00 | 0.03 | 0.01 |
| $K04564_{VIP\geq0.8}$ | 0.81 | 0.98 | 0.54 | 1.87 | 0.90 | 0.64 | 0.01 | -0.02 | 0.01 | 0.04 | -0.02 | -0.03 |
| $K03154_{VIP\geq0.8}$ | 0.90 | 0.51 | 0.82 | 1.80 | 1.20 | 0.41 | -0.03 | -0.01 | 0.03 | 0.04 | 0.05 | 0.00 |
| $K02500_{VIP\geq0.8}$ | 0.83 | 1.00 | 1.24 | 1.58 | 0.58 | 0.44 | -0.03 | -0.04 | 0.03 | 0.04 | 0.00 | 0.01 |
| $K00927_{VIP\geq0.8}$ | 0.98 | 0.93 | 0.84 | 0.41 | 0.67 | 0.83 | 0.02 | 0.02 | -0.03 | 0.00 | -0.01 | -0.04 |
| $K03924_{VIP\geq0.8}$ | 0.60 | 1.04 | 1.10 | 1.68 | 0.57 | 0.83 | -0.02 | -0.04 | 0.03 | 0.04 | -0.02 | 0.02 |
| K03638 | 1.76 | 0.60 | 1.01 | 1.34 | 0.46 | 0.63 | -0.07 | 0.02 | 0.03 | 0.04 | 0.02 | -0.02 |

| | | | | | | | | | | | | |
|---|---|---|---|---|---|---|---|---|---|---|---|---|
| K04796 | 0.60 | 0.59 | 1.09 | 1.01 | 0.70 | 1.00 | -0.01 | 0.00 | 0.03 | 0.03 | 0.03 | -0.03 |
| K02822 | 0.73 | 0.70 | 1.51 | 0.57 | 0.83 | 1.01 | -0.01 | -0.02 | 0.04 | 0.01 | 0.02 | 0.04 |
| K03208 | 1.41 | 1.87 | 0.63 | 0.85 | 0.76 | 0.68 | 0.05 | -0.06 | -0.02 | 0.01 | -0.01 | -0.02 |
| K07172 | 0.83 | 0.71 | 0.66 | 1.04 | 0.78 | 0.99 | 0.03 | -0.02 | -0.01 | -0.02 | 0.02 | -0.02 |
| K07502 | 0.74 | 1.18 | 0.73 | 0.67 | 0.83 | 1.12 | 0.01 | -0.05 | 0.01 | 0.00 | 0.00 | 0.01 |
| K06077 | 0.77 | 0.85 | 0.72 | 0.95 | 0.44 | 1.40 | 0.00 | 0.02 | -0.02 | 0.02 | -0.01 | 0.03 |
| K19166 | 0.27 | 0.70 | 0.76 | 1.51 | 0.87 | 1.02 | 0.01 | 0.03 | -0.02 | 0.04 | 0.03 | 0.04 |
| K02032 | 0.56 | 0.96 | 1.32 | 0.59 | 0.87 | 0.57 | -0.01 | 0.00 | 0.03 | 0.01 | 0.01 | 0.00 |
| K16787 | 0.86 | 0.96 | 0.61 | 0.49 | 1.01 | 0.73 | -0.03 | 0.00 | -0.01 | 0.00 | 0.03 | -0.02 |
| K16898 | 0.81 | 0.88 | 0.63 | 0.72 | 0.81 | 0.42 | 0.01 | -0.02 | 0.01 | -0.01 | 0.00 | -0.01 |
| K06973 | 1.51 | 0.51 | 0.49 | 1.25 | 0.79 | 1.62 | -0.06 | -0.02 | -0.02 | -0.02 | 0.02 | -0.04 |
| K02099 | 1.10 | 1.01 | 0.85 | 0.74 | 0.67 | 0.62 | -0.04 | -0.04 | 0.01 | 0.00 | 0.00 | 0.03 |
| K02027 | 0.58 | 1.44 | 1.47 | 0.44 | 1.55 | 0.31 | -0.01 | 0.03 | 0.04 | 0.01 | 0.05 | 0.01 |
| K06177 | 0.69 | 0.70 | 0.76 | 1.19 | 1.93 | 1.20 | 0.02 | 0.03 | 0.03 | 0.03 | -0.08 | 0.01 |
| K00940 | 1.04 | 0.40 | 0.87 | 1.07 | 0.46 | 0.45 | 0.03 | 0.02 | 0.03 | 0.02 | 0.00 | 0.02 |
| K05833 | 0.75 | 0.98 | 0.54 | 0.70 | 0.90 | 0.99 | -0.02 | 0.01 | 0.00 | 0.00 | 0.01 | -0.03 |
| K07335 | 0.71 | 0.63 | 0.95 | 0.61 | 1.01 | 0.89 | 0.02 | -0.02 | 0.02 | 0.00 | 0.02 | -0.04 |
| K12132 | 0.63 | 0.94 | 0.41 | 0.64 | 1.00 | 0.97 | -0.01 | -0.04 | -0.01 | 0.00 | -0.03 | -0.01 |
| K03816 | 0.86 | 1.10 | 1.21 | 0.78 | 0.65 | 0.62 | -0.01 | 0.03 | -0.04 | -0.02 | 0.00 | -0.03 |
| K04085 | 1.21 | 0.74 | 0.72 | 1.17 | 0.44 | 0.86 | 0.05 | 0.02 | 0.02 | 0.03 | 0.00 | 0.01 |
| K03803 | 0.31 | 0.65 | 0.75 | 2.25 | 1.78 | 0.89 | -0.01 | 0.02 | 0.02 | 0.05 | 0.07 | 0.00 |
| K01610 | 0.91 | 1.28 | 0.92 | 0.48 | 0.55 | 0.76 | -0.01 | 0.02 | -0.03 | -0.01 | 0.01 | -0.02 |
| K01696 | 1.45 | 0.74 | 0.87 | 0.12 | 0.92 | 0.74 | 0.05 | -0.02 | -0.02 | 0.00 | 0.02 | 0.03 |
| K03046 | 1.03 | 0.89 | 0.94 | 0.32 | 0.65 | 0.52 | 0.01 | 0.00 | -0.03 | 0.00 | -0.02 | 0.01 |
| K07482 | 0.90 | 0.78 | 0.39 | 0.60 | 1.05 | 1.47 | -0.03 | -0.03 | -0.01 | 0.01 | -0.04 | 0.03 |
| K01940 | 1.29 | 0.75 | 0.71 | 0.84 | 0.66 | 1.05 | 0.02 | -0.02 | 0.00 | 0.02 | 0.00 | 0.04 |
| K03555 | 1.39 | 0.88 | 0.80 | 0.38 | 0.80 | 0.55 | 0.04 | -0.01 | -0.02 | 0.01 | 0.00 | 0.00 |
| K12340 | 0.45 | 0.61 | 0.91 | 1.18 | 0.78 | 1.09 | -0.01 | -0.02 | 0.03 | 0.02 | -0.02 | 0.02 |
| K00784 | 0.44 | 0.69 | 1.33 | 1.09 | 0.98 | 0.41 | 0.01 | -0.02 | 0.04 | 0.03 | -0.03 | -0.01 |
| K00604 | 0.95 | 0.92 | 0.95 | 0.79 | 0.76 | 0.22 | 0.02 | 0.00 | 0.02 | 0.02 | -0.01 | 0.01 |
| K02529 | 1.20 | 1.04 | 1.54 | 0.11 | 0.74 | 0.59 | -0.04 | 0.02 | 0.05 | 0.00 | 0.02 | 0.03 |
| K01915 | 1.19 | 0.76 | 0.70 | 0.27 | 1.20 | 1.25 | -0.03 | -0.02 | -0.01 | 0.00 | 0.03 | -0.05 |
| K14122 | 0.47 | 0.58 | 0.67 | 0.84 | 1.20 | 0.78 | 0.01 | 0.00 | 0.01 | 0.02 | 0.05 | 0.00 |
| K07573 | 0.61 | 0.73 | 1.04 | 0.35 | 0.40 | 0.99 | -0.01 | 0.00 | 0.03 | 0.01 | 0.01 | -0.02 |
| K05937 | 0.17 | 0.78 | 0.73 | 0.65 | 1.02 | 1.30 | 0.00 | -0.02 | 0.02 | 0.02 | -0.04 | 0.02 |

| | | | | | | | | | | | | |
|---|---|---|---|---|---|---|---|---|---|---|---|---|
| K12952 | 0.71 | 0.70 | 0.63 | 0.82 | 0.70 | 0.80 | 0.01 | -0.02 | -0.02 | -0.01 | 0.02 | 0.00 |
| K06972 | 0.66 | 1.18 | 0.71 | 0.80 | 0.95 | 0.34 | 0.00 | 0.01 | -0.01 | -0.01 | 0.01 | 0.01 |
| K01938 | 0.67 | 0.91 | 0.71 | 0.97 | 0.78 | 0.59 | 0.01 | -0.01 | -0.02 | -0.01 | 0.01 | 0.01 |
| K04070 | 0.82 | 0.72 | 0.67 | 0.77 | 0.75 | 1.24 | -0.02 | -0.01 | -0.02 | -0.01 | 0.01 | -0.04 |
| K23536 | 0.74 | 0.75 | 0.79 | 0.68 | 0.98 | 1.27 | 0.02 | -0.01 | 0.01 | 0.01 | 0.02 | -0.05 |
| K01997 | 0.67 | 0.99 | 0.73 | 0.76 | 0.77 | 0.99 | 0.02 | -0.01 | 0.00 | 0.01 | -0.01 | -0.04 |
| K13283 | 0.59 | 1.42 | 2.54 | 0.75 | 0.69 | 0.50 | -0.01 | 0.04 | 0.08 | 0.02 | 0.02 | 0.02 |
| K01649 | 1.08 | 1.06 | 0.55 | 0.74 | 0.70 | 0.79 | 0.00 | 0.01 | -0.01 | 0.02 | -0.01 | 0.00 |
| K03723 | 1.19 | 0.77 | 0.48 | 0.54 | 1.14 | 0.65 | 0.03 | -0.01 | 0.00 | 0.01 | -0.03 | -0.02 |
| K00991 | 0.79 | 0.75 | 0.70 | 0.77 | 1.51 | 0.31 | -0.02 | 0.01 | -0.01 | -0.01 | -0.06 | -0.01 |

Partial Least Squares analysis was performed with 2 latent components. Subscript VIP≥0.8 denotes microbial genera that had VIP ≥ 0.8 in 4 or more timepoints. Subscripts (+) and (-) represent microbial genera that had positive or negative regression coefficient in 4 or more timepoints, respectively.

**Trait: Daily Feed Intake (DFI); Predictors: 194 microbial genes (MG)**

| Microbial genes | VIP | | | | | | Regression coefficient | | | | | |
|---|---|---|---|---|---|---|---|---|---|---|---|---|
| | T1 | T2 | T3 | T4 | T5 | T6 | T1 | T2 | T3 | T4 | T5 | T6 |
| $K03709_{VIP\geq0.8\ (+)}$ | 0.90 | 1.21 | 1.76 | 0.65 | 1.53 | 1.62 | 0.02 | 0.03 | 0.03 | 0.02 | 0.05 | 0.05 |
| $K08094_{VIP\geq0.8\ (+)}$ | 0.25 | 0.70 | 1.83 | 1.21 | 1.40 | 1.31 | 0.01 | 0.01 | 0.04 | 0.03 | 0.04 | 0.03 |
| $K11261_{VIP\geq0.8\ (+)}$ | 0.19 | 1.51 | 1.51 | 1.65 | 0.99 | 0.46 | 0.00 | 0.04 | 0.03 | 0.04 | 0.03 | -0.01 |
| $K10218_{VIP\geq0.8\ (+)}$ | 1.13 | 1.36 | 1.45 | 1.09 | 1.73 | 0.13 | -0.03 | 0.06 | 0.03 | 0.03 | 0.06 | 0.00 |
| $K19166_{VIP\geq0.8\ (+)}$ | 2.05 | 1.98 | 0.66 | 1.10 | 0.62 | 2.22 | 0.05 | 0.07 | 0.00 | 0.03 | 0.02 | 0.05 |
| $K07503_{VIP\geq0.8\ (+)}$ | 0.18 | 1.59 | 1.51 | 0.98 | 0.83 | 0.75 | 0.00 | 0.04 | 0.03 | 0.03 | 0.01 | 0.01 |
| $K07732_{VIP\geq0.8\ (+)}$ | 0.73 | 1.09 | 1.36 | 1.28 | 0.96 | 1.09 | 0.00 | 0.02 | 0.02 | 0.03 | 0.01 | 0.02 |
| $K14122_{VIP\geq0.8\ (+)}$ | 0.81 | 1.05 | 1.31 | 0.83 | 1.07 | 0.82 | 0.01 | 0.01 | 0.02 | 0.02 | 0.02 | 0.00 |
| $K10725_{VIP\geq0.8\ (+)}$ | 1.42 | 0.88 | 0.91 | 0.77 | 1.02 | 0.90 | 0.03 | 0.01 | 0.01 | 0.02 | 0.00 | 0.01 |
| $K03638_{VIP\geq0.8\ (+)}$ | 0.75 | 1.55 | 1.68 | 1.03 | 0.97 | 0.64 | -0.02 | 0.04 | 0.03 | 0.03 | 0.02 | -0.01 |
| $K09797_{VIP\geq0.8\ (+)}$ | 0.26 | 1.44 | 0.83 | 1.20 | 0.48 | 1.47 | 0.00 | 0.06 | 0.02 | 0.03 | -0.01 | 0.05 |
| $K14112_{VIP\geq0.8\ (+)}$ | 1.30 | 1.27 | 0.91 | 0.89 | 1.00 | 0.73 | 0.03 | 0.02 | 0.01 | 0.02 | 0.01 | 0.00 |
| $K07158_{VIP\geq0.8\ (+)}$ | 0.70 | 0.80 | 1.40 | 0.99 | 0.98 | 0.87 | 0.00 | 0.01 | 0.03 | 0.03 | 0.00 | 0.02 |
| $K12962_{VIP\geq0.8\ (+)}$ | 1.80 | 1.69 | 0.71 | 1.10 | 0.79 | 1.45 | 0.04 | 0.06 | 0.00 | -0.02 | -0.01 | 0.03 |

| | | | | | | | | | | | | |
|---|---|---|---|---|---|---|---|---|---|---|---|---|
| $K14096_{VIP≥0.8\ (+)}$ | 1.10 | 1.21 | 1.22 | 0.74 | 0.99 | 0.73 | 0.01 | 0.02 | 0.02 | 0.02 | 0.01 | 0.00 |
| $K00286_{VIP≥0.8\ (+)}$ | 0.69 | 0.86 | 1.09 | 1.72 | 0.34 | 1.13 | 0.01 | 0.04 | 0.02 | 0.03 | 0.00 | 0.04 |
| $K14095_{VIP≥0.8\ (+)}$ | 0.98 | 1.14 | 1.34 | 0.87 | 1.01 | 0.84 | 0.01 | 0.02 | 0.02 | 0.02 | 0.01 | -0.02 |
| $K14102_{VIP≥0.8\ (+)}$ | 0.89 | 1.34 | 1.08 | 0.77 | 1.04 | 0.67 | 0.01 | 0.03 | 0.02 | 0.02 | 0.01 | 0.00 |
| $K06961_{VIP≥0.8\ (+)}$ | 0.87 | 1.18 | 1.08 | 0.69 | 1.08 | 0.56 | 0.01 | 0.02 | 0.02 | 0.01 | 0.02 | 0.00 |
| $K14118_{VIP≥0.8\ (+)}$ | 0.89 | 1.17 | 1.52 | 0.74 | 1.19 | 0.88 | 0.01 | 0.02 | 0.03 | 0.02 | -0.01 | 0.00 |
| $K04796_{VIP≥0.8\ (+)}$ | 0.72 | 1.20 | 1.40 | 0.72 | 1.07 | 0.86 | 0.00 | 0.02 | 0.03 | 0.02 | 0.00 | 0.01 |
| $K24131_{VIP≥0.8\ (+)}$ | 1.21 | 0.68 | 1.20 | 1.61 | 0.74 | 1.59 | 0.03 | 0.01 | 0.02 | 0.03 | 0.00 | 0.05 |
| $K03181_{VIP≥0.8\ (+)}$ | 0.70 | 1.17 | 1.40 | 0.72 | 1.07 | 1.03 | 0.00 | 0.02 | 0.03 | 0.02 | 0.01 | 0.01 |
| $K00046_{VIP≥0.8\ (+)}$ | 0.91 | 0.62 | 0.95 | 0.97 | 1.00 | 0.57 | 0.02 | 0.02 | 0.02 | 0.03 | -0.02 | 0.01 |
| $K02529_{VIP≥0.8\ (+)}$ | 1.27 | 1.33 | 1.49 | 0.98 | 0.53 | 0.62 | -0.02 | 0.06 | 0.03 | 0.02 | 0.02 | 0.02 |
| $K03057_{VIP≥0.8\ (+)}$ | 0.91 | 0.89 | 1.36 | 0.94 | 1.02 | 0.76 | 0.01 | 0.01 | 0.02 | 0.02 | 0.00 | -0.01 |
| $K03540_{VIP≥0.8\ (+)}$ | 0.71 | 1.35 | 1.06 | 0.70 | 1.03 | 1.05 | 0.00 | 0.03 | 0.01 | 0.01 | 0.01 | 0.01 |
| $K07573_{VIP≥0.8\ (+)}$ | 0.83 | 1.24 | 1.22 | 0.78 | 1.10 | 0.86 | 0.00 | 0.02 | 0.02 | 0.01 | 0.00 | 0.00 |
| $K07720_{VIP≥0.8\ (+)}$ | 1.02 | 0.73 | 1.61 | 1.65 | 1.21 | 0.94 | -0.01 | 0.02 | 0.02 | 0.03 | 0.02 | 0.02 |
| $K01874_{VIP≥0.8\ (+)}$ | 0.82 | 0.63 | 1.25 | 1.53 | 1.03 | 0.87 | 0.02 | 0.01 | 0.02 | 0.03 | 0.03 | 0.00 |
| $K03606_{VIP≥0.8\ (+)}$ | 0.81 | 0.53 | 1.29 | 1.33 | 0.24 | 1.17 | 0.01 | 0.02 | 0.02 | 0.02 | -0.01 | 0.04 |
| $K02557_{VIP≥0.8\ (+)}$ | 1.23 | 1.20 | 0.80 | 0.67 | 0.70 | 0.93 | 0.03 | 0.06 | -0.01 | 0.01 | 0.01 | 0.02 |
| $K03538_{VIP≥0.8\ (+)}$ | 0.80 | 1.19 | 1.17 | 0.68 | 0.93 | 0.91 | 0.00 | 0.02 | 0.02 | 0.01 | 0.01 | 0.01 |
| $K02217_{VIP≥0.8\ (+)}$ | 0.75 | 0.87 | 0.83 | 0.95 | 1.10 | 0.82 | 0.01 | 0.04 | 0.01 | 0.01 | 0.03 | 0.03 |
| $K21030_{VIP≥0.8\ (+)}$ | 1.61 | 0.51 | 0.84 | 0.48 | 1.50 | 1.85 | 0.04 | 0.02 | 0.01 | 0.00 | -0.05 | 0.06 |
| $K03105_{VIP≥0.8\ (+)}$ | 0.85 | 1.29 | 0.99 | 0.76 | 1.05 | 1.12 | 0.00 | 0.02 | 0.01 | 0.01 | 0.01 | 0.01 |
| $K00390_{VIP≥0.8\ (+)}$ | 1.04 | 0.85 | 1.02 | 1.78 | 0.88 | 0.97 | 0.02 | 0.01 | 0.01 | 0.03 | 0.01 | 0.01 |
| $K07569_{VIP≥0.8\ (+)}$ | 0.61 | 1.33 | 0.76 | 0.84 | 1.03 | 0.81 | 0.00 | 0.03 | 0.00 | 0.02 | 0.02 | -0.01 |
| $K11260_{VIP≥0.8\ (+)}$ | 0.77 | 1.28 | 0.81 | 0.88 | 1.34 | 0.62 | 0.00 | 0.03 | 0.01 | 0.02 | -0.02 | 0.00 |
| $K07464_{VIP≥0.8\ (+)}$ | 1.06 | 0.85 | 1.11 | 1.20 | 1.00 | 0.81 | 0.02 | 0.01 | 0.01 | 0.02 | 0.02 | 0.01 |
| $K07075_{VIP≥0.8\ (+)}$ | 1.10 | 1.50 | 0.70 | 0.73 | 1.09 | 0.87 | -0.03 | 0.04 | 0.01 | 0.02 | 0.03 | 0.00 |
| $K04798_{VIP≥0.8\ (+)}$ | 0.86 | 1.10 | 1.05 | 0.81 | 1.03 | 0.95 | 0.00 | 0.01 | 0.01 | 0.00 | 0.01 | 0.00 |
| $K03264_{VIP≥0.8\ (+)}$ | 0.85 | 1.24 | 1.06 | 0.76 | 1.04 | 0.80 | -0.01 | 0.02 | 0.01 | 0.01 | 0.01 | -0.01 |
| $K05305_{VIP≥0.8\ (+)}$ | 1.18 | 0.07 | 1.32 | 0.75 | 1.12 | 1.67 | -0.03 | 0.00 | 0.03 | 0.02 | -0.04 | 0.06 |

| | | | | | | | | | | | | |
|---|---|---|---|---|---|---|---|---|---|---|---|---|
| $K03626_{VIP≥0.8\ (+)}$ | 0.89 | 1.13 | 1.01 | 0.95 | 0.98 | 0.94 | 0.00 | 0.01 | 0.01 | 0.00 | 0.01 | 0.00 |
| $K03120_{VIP≥0.8\ (+)}$ | 0.88 | 1.10 | 0.95 | 0.94 | 1.01 | 0.97 | 0.00 | 0.01 | 0.01 | 0.00 | 0.02 | 0.01 |
| $K13993_{VIP≥0.8\ (+)}$ | 1.31 | 0.73 | 1.00 | 1.12 | 0.97 | 0.71 | 0.03 | 0.02 | 0.01 | 0.01 | -0.02 | 0.01 |
| $K01703_{VIP≥0.8\ (+)}$ | 0.65 | 0.91 | 0.78 | 1.22 | 1.28 | 0.84 | 0.01 | 0.01 | 0.00 | 0.02 | 0.04 | 0.00 |
| $K07502_{VIP≥0.8\ (+)}$ | 0.82 | 0.86 | 1.23 | 0.82 | 1.17 | 0.18 | 0.01 | -0.01 | 0.02 | 0.01 | 0.03 | 0.00 |
| $K00052_{VIP≥0.8\ (+)}$ | 0.84 | 0.75 | 0.88 | 0.93 | 0.69 | 0.83 | 0.01 | 0.03 | 0.01 | 0.01 | 0.00 | 0.01 |
| $K19755_{VIP≥0.8\ (+)}$ | 0.91 | 1.16 | 0.93 | 0.74 | 0.85 | 1.01 | -0.02 | 0.01 | 0.01 | 0.01 | 0.01 | 0.00 |
| $K03147_{VIP≥0.8\ (+)}$ | 0.89 | 1.24 | 0.81 | 1.32 | 0.66 | 0.88 | 0.01 | 0.06 | 0.00 | 0.02 | -0.02 | 0.00 |
| $K03555_{VIP≥0.8\ (+)}$ | 1.21 | 1.12 | 0.78 | 1.41 | 0.87 | 1.04 | 0.03 | -0.01 | 0.00 | 0.02 | 0.00 | 0.00 |
| $K02866_{VIP≥0.8\ (+)}$ | 0.74 | 1.05 | 0.98 | 0.76 | 1.02 | 0.89 | 0.00 | 0.01 | 0.01 | 0.00 | 0.01 | -0.03 |
| $K01649_{VIP≥0.8\ (+)}$ | 0.87 | 0.75 | 0.85 | 1.16 | 0.65 | 0.88 | 0.01 | 0.01 | 0.00 | 0.02 | 0.01 | 0.01 |
| $K03263_{VIP≥0.8\ (+)}$ | 0.88 | 1.08 | 0.95 | 1.17 | 0.94 | 0.97 | 0.00 | 0.00 | 0.00 | -0.01 | 0.01 | 0.00 |
| $K02026_{VIP≥0.8\ (+)}$ | 1.52 | 0.93 | 1.15 | 0.69 | 1.36 | 0.80 | -0.03 | 0.05 | 0.02 | -0.01 | 0.03 | 0.02 |
| $K03723_{VIP≥0.8\ (+)}$ | 0.88 | 0.62 | 0.85 | 1.34 | 0.88 | 0.83 | 0.00 | 0.01 | 0.01 | 0.02 | 0.01 | 0.01 |
| $K01610_{VIP≥0.8\ (+)}$ | 0.76 | 0.73 | 0.84 | 1.16 | 1.27 | 1.10 | 0.01 | 0.01 | 0.00 | 0.01 | 0.03 | -0.01 |
| $K03698_{VIP≥0.8\ (+)}$ | 0.84 | 1.83 | 0.86 | 0.73 | 1.12 | 0.53 | 0.00 | -0.05 | 0.01 | -0.02 | 0.02 | 0.01 |
| $K03327_{VIP≥0.8\ (+)}$ | 0.95 | 0.89 | 1.14 | 1.26 | 0.84 | 0.84 | 0.00 | 0.01 | 0.01 | 0.02 | 0.01 | 0.01 |
| $K19117_{VIP≥0.8\ (+)}$ | 0.95 | 0.83 | 0.88 | 0.82 | 1.03 | 1.21 | 0.01 | 0.01 | 0.00 | 0.00 | 0.00 | 0.04 |
| $K00941_{VIP≥0.8\ (+)}$ | 1.01 | 0.66 | 0.95 | 0.89 | 1.28 | 0.78 | -0.01 | 0.02 | 0.01 | 0.00 | -0.04 | 0.00 |
| $K00651_{VIP≥0.8\ (+)}$ | 0.84 | 0.84 | 0.85 | 0.92 | 0.92 | 1.02 | 0.01 | 0.01 | -0.01 | 0.00 | 0.00 | -0.01 |
| $K02519_{VIP≥0.8\ (+)}$ | 0.74 | 0.57 | 0.88 | 0.98 | 0.91 | 1.14 | 0.01 | 0.02 | 0.01 | 0.01 | 0.02 | -0.01 |
| $K01752_{VIP≥0.8\ (+)}$ | 0.86 | 0.92 | 0.90 | 0.88 | 1.08 | 1.00 | 0.01 | 0.00 | 0.00 | 0.00 | 0.02 | 0.01 |
| $K00265_{VIP≥0.8\ (+)}$ | 1.12 | 0.82 | 0.82 | 0.71 | 0.83 | 0.99 | 0.02 | 0.01 | 0.00 | 0.00 | 0.01 | 0.00 |
| $K08963_{VIP≥0.8\ (+)}$ | 1.00 | 0.97 | 1.13 | 0.98 | 1.21 | 0.79 | -0.01 | 0.00 | 0.01 | 0.01 | 0.03 | 0.00 |
| $K11068_{VIP≥0.8\ (+)}$ | 0.88 | 0.82 | 0.89 | 0.56 | 1.41 | 0.87 | -0.02 | 0.02 | 0.01 | 0.01 | -0.03 | 0.01 |
| $K00788_{VIP≥0.8\ (+)}$ | 0.99 | 0.87 | 0.95 | 1.12 | 1.12 | 0.51 | -0.01 | 0.04 | 0.01 | 0.02 | -0.04 | 0.02 |
| $K02027_{VIP≥0.8\ (+)}$ | 1.13 | 0.89 | 1.28 | 0.75 | 0.89 | 0.96 | -0.01 | 0.04 | 0.02 | -0.01 | 0.01 | 0.00 |
| $K07496_{VIP≥0.8\ (+)}$ | 1.07 | 1.38 | 0.97 | 0.89 | 0.60 | 0.34 | -0.02 | -0.05 | 0.02 | 0.01 | 0.02 | 0.00 |
| $K01258_{VIP≥0.8\ (+)}$ | 0.95 | 1.03 | 0.90 | 0.93 | 0.87 | 1.04 | 0.01 | -0.01 | 0.00 | 0.01 | 0.01 | 0.02 |
| $K02112_{VIP≥0.8\ (+)}$ | 0.99 | 0.80 | 0.83 | 0.74 | 1.44 | 0.97 | -0.01 | 0.01 | 0.00 | 0.00 | 0.03 | 0.01 |

| | | | | | | | | | | | | |
|---|---|---|---|---|---|---|---|---|---|---|---|---|
| $K02025_{VIP≥0.8\ (+)}$ | 0.98 | 0.74 | 1.10 | 0.74 | 1.22 | 0.92 | -0.01 | 0.03 | 0.01 | -0.01 | 0.02 | 0.00 |
| $K02035_{VIP≥0.8\ (+)}$ | 1.03 | 1.03 | 1.12 | 0.80 | 1.12 | 0.72 | -0.01 | 0.00 | 0.01 | 0.00 | 0.02 | 0.01 |
| $K07165_{VIP≥0.8\ (+)}$ | 0.95 | 0.93 | 0.94 | 0.89 | 0.89 | 1.00 | 0.01 | 0.00 | 0.00 | 0.00 | -0.02 | 0.02 |
| $K09816_{VIP≥0.8\ (+)}$ | 1.18 | 0.61 | 0.91 | 0.83 | 1.50 | 0.65 | -0.02 | 0.01 | 0.01 | 0.01 | 0.04 | 0.00 |
| $K02313_{VIP≥0.8\ (+)}$ | 0.78 | 0.52 | 0.92 | 0.95 | 0.82 | 0.95 | -0.01 | 0.02 | 0.01 | 0.01 | -0.01 | 0.00 |
| $K04043_{VIP≥0.8\ (+)}$ | 0.82 | 1.04 | 0.96 | 0.84 | 0.90 | 1.23 | 0.00 | -0.01 | 0.01 | 0.00 | 0.02 | -0.02 |
| $K03741_{VIP≥0.8\ (+)}$ | 0.95 | 0.93 | 0.92 | 1.05 | 0.96 | 1.00 | 0.00 | 0.00 | 0.00 | 0.01 | 0.00 | 0.01 |
| $K06207_{VIP≥0.8\ (+)}$ | 0.81 | 0.92 | 0.84 | 1.10 | 0.72 | 0.92 | 0.01 | 0.00 | 0.00 | 0.01 | 0.00 | 0.00 |
| $K02114_{VIP≥0.8\ (+)}$ | 0.96 | 0.84 | 0.99 | 0.77 | 1.13 | 1.09 | 0.01 | 0.01 | 0.01 | -0.01 | 0.01 | -0.01 |
| $K02337_{VIP≥0.8\ (+)}$ | 0.95 | 0.73 | 0.71 | 1.13 | 0.85 | 0.89 | 0.00 | 0.00 | 0.00 | 0.01 | 0.01 | 0.00 |
| $K01955_{VIP≥0.8\ (+)}$ | 0.95 | 0.96 | 0.98 | 1.12 | 0.77 | 0.95 | 0.00 | -0.01 | 0.01 | 0.01 | 0.00 | 0.01 |
| $K00020_{VIP≥0.8\ (+)}$ | 0.81 | 1.31 | 1.06 | 0.78 | 0.92 | 0.83 | 0.02 | -0.03 | 0.01 | -0.02 | 0.01 | 0.02 |
| $K07118_{VIP≥0.8\ (+)}$ | 0.96 | 0.97 | 1.00 | 0.78 | 0.86 | 1.01 | 0.01 | 0.00 | 0.01 | -0.01 | 0.00 | 0.01 |
| $K01999_{VIP≥0.8\ (+)}$ | 0.88 | 0.86 | 0.92 | 0.78 | 0.95 | 1.06 | 0.00 | 0.01 | 0.01 | 0.00 | 0.01 | -0.01 |
| $K03798_{VIP≥0.8\ (+)}$ | 1.03 | 1.05 | 0.96 | 0.95 | 0.97 | 1.05 | 0.00 | -0.01 | 0.01 | 0.00 | 0.01 | 0.00 |
| $K17248_{VIP≥0.8\ (-)}$ | 2.29 | 1.38 | 1.17 | 0.54 | 2.44 | 1.44 | -0.06 | -0.07 | 0.02 | -0.01 | -0.08 | -0.04 |
| $K03048_{VIP≥0.8\ (-)}$ | 1.69 | 1.02 | 0.58 | 1.17 | 0.78 | 1.16 | -0.04 | -0.03 | 0.00 | -0.03 | -0.02 | -0.02 |
| $K09691_{VIP≥0.8\ (-)}$ | 1.36 | 0.71 | 0.91 | 1.79 | 1.80 | 0.80 | -0.04 | -0.03 | 0.02 | -0.04 | -0.05 | -0.03 |
| $K01734_{VIP≥0.8\ (-)}$ | 0.60 | 0.49 | 1.01 | 1.33 | 1.22 | 1.64 | 0.01 | -0.01 | -0.02 | -0.03 | -0.04 | -0.05 |
| $K20608_{VIP≥0.8\ (-)}$ | 1.91 | 0.75 | 0.76 | 0.96 | 1.20 | 1.20 | -0.05 | -0.03 | 0.00 | -0.01 | -0.02 | -0.04 |
| $K14059_{VIP≥0.8\ (-)}$ | 1.19 | 0.69 | 0.64 | 0.87 | 0.86 | 1.87 | -0.02 | -0.02 | 0.01 | -0.02 | -0.02 | -0.05 |
| $K01077_{VIP≥0.8\ (-)}$ | 0.86 | 0.49 | 1.26 | 1.42 | 0.26 | 1.32 | 0.01 | -0.01 | -0.02 | -0.04 | 0.01 | -0.03 |
| $K16927_{VIP≥0.8\ (-)}$ | 1.12 | 1.21 | 0.60 | 0.77 | 1.25 | 1.69 | -0.02 | -0.03 | 0.00 | -0.02 | 0.03 | -0.05 |
| $K22162_{VIP≥0.8\ (-)}$ | 1.92 | 1.19 | 0.96 | 1.67 | 1.13 | 0.77 | -0.05 | 0.04 | 0.02 | -0.04 | -0.04 | -0.02 |
| $K01091_{VIP≥0.8\ (-)}$ | 1.01 | 1.33 | 0.99 | 0.73 | 0.78 | 0.86 | -0.01 | -0.03 | -0.02 | 0.00 | -0.02 | 0.00 |
| $K16787_{VIP≥0.8\ (-)}$ | 0.96 | 0.99 | 0.69 | 1.08 | 0.91 | 1.01 | -0.01 | -0.01 | 0.00 | -0.03 | 0.00 | -0.01 |
| $K12132_{VIP≥0.8\ (-)}$ | 1.15 | 0.67 | 0.78 | 0.89 | 0.87 | 0.83 | -0.02 | 0.00 | 0.01 | -0.02 | -0.02 | -0.02 |
| $K04070_{VIP≥0.8\ (-)}$ | 0.97 | 0.63 | 0.65 | 0.97 | 0.88 | 1.32 | -0.01 | 0.03 | 0.00 | -0.02 | -0.01 | -0.03 |
| $K08986_{VIP≥0.8\ (-)}$ | 0.91 | 0.79 | 0.76 | 0.96 | 0.90 | 1.15 | 0.00 | 0.00 | 0.00 | -0.02 | -0.01 | -0.02 |
| $K16329_{VIP≥0.8\ (-)}$ | 0.84 | 1.26 | 0.87 | 0.92 | 0.94 | 0.87 | -0.01 | -0.06 | 0.01 | -0.01 | -0.02 | -0.02 |

| | | | | | | | | | | | | |
|---|---|---|---|---|---|---|---|---|---|---|---|---|
| K02072$_{VIP≥0.8\ (-)}$ | 1.06 | 0.69 | 0.74 | 0.81 | 0.86 | 1.07 | -0.01 | 0.01 | 0.00 | -0.02 | -0.01 | -0.01 |
| K00882$_{VIP≥0.8\ (-)}$ | 0.85 | 0.94 | 1.01 | 1.13 | 0.96 | 1.09 | 0.00 | 0.00 | -0.02 | -0.03 | 0.02 | -0.01 |
| K02099$_{VIP≥0.8\ (-)}$ | 1.16 | 0.95 | 0.84 | 0.73 | 0.70 | 0.87 | -0.02 | -0.01 | -0.01 | 0.00 | -0.01 | 0.00 |
| K01005$_{VIP≥0.8\ (-)}$ | 0.87 | 0.77 | 0.88 | 0.89 | 0.84 | 1.34 | 0.00 | 0.00 | 0.01 | -0.02 | -0.01 | -0.03 |
| K21071$_{VIP≥0.8\ (-)}$ | 1.02 | 0.89 | 0.76 | 0.76 | 0.85 | 0.99 | -0.01 | 0.00 | 0.00 | -0.01 | 0.01 | -0.02 |
| K16898$_{VIP≥0.8\ (-)}$ | 1.00 | 0.88 | 0.89 | 0.93 | 0.89 | 1.36 | 0.00 | 0.00 | 0.01 | -0.02 | 0.00 | -0.03 |
| K07335$_{VIP≥0.8\ (-)}$ | 0.85 | 0.87 | 0.91 | 0.81 | 0.86 | 1.32 | 0.00 | -0.02 | 0.01 | -0.02 | 0.00 | -0.02 |
| K01938$_{VIP≥0.8\ (-)}$ | 0.88 | 0.73 | 0.76 | 0.80 | 0.84 | 1.16 | 0.00 | 0.01 | 0.00 | -0.02 | -0.01 | -0.02 |
| K00266$_{VIP≥0.8\ (-)}$ | 1.02 | 1.05 | 0.91 | 0.75 | 1.05 | 1.11 | 0.02 | -0.01 | 0.00 | -0.01 | -0.03 | -0.01 |
| K07492$_{VIP≥0.8\ (-)}$ | 0.84 | 1.07 | 1.04 | 1.15 | 0.97 | 0.88 | 0.00 | 0.01 | -0.01 | -0.02 | -0.02 | -0.02 |
| K06867$_{VIP≥0.8\ (-)}$ | 1.06 | 0.67 | 0.84 | 1.35 | 0.69 | 0.90 | -0.03 | -0.01 | 0.01 | -0.02 | -0.01 | -0.02 |
| K07478$_{VIP≥0.8\ (-)}$ | 1.00 | 1.09 | 1.04 | 0.72 | 0.85 | 1.11 | 0.02 | -0.01 | -0.02 | -0.01 | -0.01 | 0.00 |
| K10761$_{VIP≥0.8\ (-)}$ | 1.02 | 0.77 | 0.66 | 1.03 | 1.14 | 0.84 | -0.02 | -0.03 | 0.01 | -0.01 | 0.00 | -0.02 |
| K06972$_{VIP≥0.8\ (-)}$ | 0.94 | 0.95 | 0.96 | 0.83 | 0.79 | 0.98 | 0.00 | 0.00 | 0.01 | -0.02 | 0.00 | -0.01 |
| K00856$_{VIP≥0.8\ (-)}$ | 0.98 | 0.84 | 0.94 | 1.58 | 0.99 | 0.99 | -0.02 | -0.01 | 0.00 | -0.02 | -0.01 | 0.00 |
| K02031$_{VIP≥0.8\ (-)}$ | 0.94 | 1.43 | 0.89 | 0.71 | 1.01 | 0.83 | -0.01 | -0.03 | 0.01 | -0.01 | 0.02 | 0.00 |
| K03215$_{VIP≥0.8\ (-)}$ | 1.10 | 1.37 | 0.90 | 0.77 | 1.11 | 1.14 | 0.02 | -0.02 | -0.01 | -0.01 | 0.01 | -0.01 |
| K03070$_{VIP≥0.8\ (-)}$ | 1.04 | 1.06 | 0.81 | 0.95 | 0.92 | 1.03 | -0.01 | -0.01 | 0.00 | 0.01 | 0.02 | -0.01 |
| K02036$_{VIP≥0.8\ (-)}$ | 1.09 | 0.99 | 0.87 | 0.79 | 1.26 | 1.02 | 0.00 | 0.00 | -0.01 | -0.02 | 0.02 | 0.00 |
| K02875$_{VIP≥0.8\ (-)}$ | 0.89 | 0.96 | 1.01 | 0.95 | 0.95 | 0.89 | 0.00 | 0.00 | 0.00 | 0.00 | 0.02 | 0.00 |
| K02518$_{VIP≥0.8\ (-)}$ | 0.65 | 0.99 | 0.84 | 1.07 | 0.98 | 0.72 | 0.00 | 0.02 | -0.01 | -0.02 | -0.02 | -0.02 |
| K10117$_{VIP≥0.8\ (-)}$ | 0.84 | 0.97 | 0.88 | 0.70 | 1.02 | 0.78 | 0.00 | 0.00 | 0.00 | -0.01 | 0.01 | 0.00 |
| K02908$_{VIP≥0.8}$ | 0.87 | 0.92 | 0.97 | 1.05 | 0.98 | 1.05 | -0.01 | 0.00 | 0.01 | -0.01 | 0.02 | 0.00 |
| K02995$_{VIP≥0.8}$ | 0.94 | 0.99 | 0.96 | 1.06 | 0.89 | 1.12 | 0.00 | 0.00 | 0.00 | -0.01 | 0.01 | -0.03 |
| K02921$_{VIP≥0.8}$ | 0.88 | 0.85 | 0.94 | 0.97 | 0.94 | 0.98 | -0.01 | -0.01 | 0.01 | 0.00 | 0.01 | 0.01 |
| K02032$_{VIP≥0.8}$ | 1.19 | 1.30 | 0.91 | 0.81 | 1.22 | 0.72 | -0.02 | -0.02 | 0.01 | 0.00 | 0.02 | 0.00 |
| K02039$_{VIP≥0.8}$ | 1.09 | 1.00 | 0.97 | 0.77 | 1.45 | 0.94 | 0.00 | 0.00 | 0.00 | -0.01 | 0.03 | 0.02 |
| K03753$_{VIP≥0.8}$ | 0.33 | 0.89 | 1.03 | 0.79 | 1.00 | 0.91 | -0.01 | 0.00 | 0.01 | 0.02 | 0.02 | 0.00 |
| K00441$_{VIP≥0.8}$ | 1.16 | 0.61 | 0.67 | 0.91 | 0.96 | 0.93 | -0.03 | -0.03 | 0.01 | 0.02 | -0.01 | 0.02 |
| K23997$_{VIP≥0.8}$ | 1.07 | 1.66 | 1.52 | 1.49 | 0.42 | 0.50 | -0.02 | -0.05 | 0.03 | 0.03 | 0.01 | -0.01 |

| | | | | | | | | | | | | |
|---|---|---|---|---|---|---|---|---|---|---|---|---|
| $K23876_{VIP≥0.8}$ | 1.36 | 0.83 | 1.08 | 0.96 | 0.67 | 0.57 | -0.02 | 0.00 | 0.01 | 0.01 | 0.00 | 0.01 |
| $K01223_{VIP≥0.8}$ | 0.88 | 1.61 | 0.81 | 0.70 | 0.94 | 0.60 | -0.01 | -0.04 | 0.00 | -0.01 | 0.02 | 0.01 |
| $K01989_{VIP≥0.8}$ | 0.84 | 0.80 | 0.69 | 0.80 | 0.96 | 0.91 | -0.01 | 0.00 | 0.00 | -0.02 | 0.00 | 0.00 |
| $K00656_{VIP≥0.8}$ | 1.01 | 0.79 | 0.89 | 0.77 | 0.88 | 0.89 | 0.01 | 0.03 | 0.00 | 0.00 | -0.01 | 0.01 |
| $K03778_{VIP≥0.8}$ | 1.00 | 0.85 | 0.97 | 0.79 | 0.77 | 0.90 | 0.02 | 0.00 | 0.01 | -0.02 | 0.00 | 0.01 |
| $K01297_{VIP≥0.8}$ | 1.41 | 0.66 | 0.78 | 1.14 | 1.27 | 1.03 | -0.04 | -0.01 | 0.01 | 0.03 | -0.03 | 0.01 |
| $K06413_{VIP≥0.8}$ | 1.89 | 0.99 | 0.83 | 0.62 | 0.89 | 0.64 | -0.05 | -0.05 | 0.01 | 0.01 | 0.01 | -0.01 |
| $K01953_{VIP≥0.8}$ | 0.95 | 0.77 | 0.93 | 0.99 | 0.65 | 0.96 | 0.01 | 0.01 | 0.00 | 0.01 | -0.01 | -0.01 |
| $K03924_{VIP≥0.8}$ | 0.20 | 1.14 | 0.55 | 0.93 | 0.84 | 0.81 | -0.01 | -0.02 | 0.00 | 0.01 | -0.02 | 0.03 |
| K09739 | 0.75 | 1.24 | 1.40 | 0.79 | 1.01 | 0.76 | 0.01 | 0.03 | 0.02 | 0.02 | 0.01 | 0.00 |
| K14111 | 0.69 | 1.41 | 1.46 | 0.75 | 1.02 | 0.64 | 0.00 | 0.03 | 0.03 | 0.02 | 0.00 | 0.00 |
| K06874 | 0.74 | 1.08 | 1.30 | 0.71 | 1.00 | 0.77 | 0.00 | 0.01 | 0.02 | 0.00 | 0.00 | 0.00 |
| K06875 | 0.75 | 0.73 | 0.93 | 0.73 | 1.06 | 0.88 | -0.01 | -0.01 | 0.00 | 0.01 | 0.01 | 0.00 |
| K09116 | 0.78 | 0.73 | 1.18 | 0.66 | 0.82 | 0.95 | 0.01 | -0.01 | 0.02 | 0.01 | 0.02 | -0.03 |
| K02822 | 1.03 | 1.18 | 0.64 | 0.67 | 0.90 | 0.72 | -0.01 | -0.02 | 0.00 | -0.02 | 0.02 | 0.02 |
| K20742 | 1.03 | 1.15 | 0.71 | 1.86 | 0.24 | 0.41 | 0.02 | 0.03 | 0.01 | -0.04 | -0.01 | 0.00 |
| K03208 | 0.65 | 0.80 | 1.21 | 1.03 | 0.68 | 0.66 | -0.02 | -0.04 | -0.02 | -0.02 | -0.02 | -0.02 |
| K07172 | 2.33 | 0.52 | 0.44 | 0.80 | 1.41 | 1.13 | 0.06 | 0.02 | 0.00 | -0.02 | 0.04 | -0.03 |
| K07031 | 0.32 | 1.27 | 1.59 | 1.50 | 0.69 | 0.52 | -0.01 | -0.06 | -0.03 | -0.03 | 0.02 | -0.02 |
| K07402 | 0.75 | 1.38 | 0.82 | 0.80 | 0.73 | 1.24 | 0.00 | -0.03 | 0.00 | 0.00 | 0.00 | -0.02 |
| K07334 | 1.19 | 0.68 | 1.06 | 1.03 | 0.76 | 0.57 | 0.03 | 0.00 | -0.02 | -0.03 | -0.02 | 0.01 |
| K04047 | 0.86 | 1.17 | 0.77 | 0.75 | 0.74 | 0.94 | 0.00 | -0.02 | 0.00 | -0.01 | 0.00 | 0.02 |
| K09825 | 1.03 | 0.71 | 0.80 | 0.79 | 0.86 | 0.86 | 0.00 | 0.01 | 0.00 | -0.01 | -0.02 | 0.00 |
| K05595 | 1.49 | 0.16 | 1.00 | 0.38 | 0.65 | 0.80 | 0.03 | 0.00 | -0.02 | -0.01 | -0.02 | 0.03 |
| K08999 | 1.07 | 0.71 | 0.82 | 0.80 | 1.17 | 0.45 | 0.03 | 0.02 | 0.00 | 0.01 | -0.04 | 0.00 |
| K12950 | 0.94 | 0.97 | 0.78 | 0.73 | 0.74 | 0.81 | -0.01 | 0.00 | 0.00 | 0.00 | 0.01 | 0.00 |
| K02086 | 0.91 | 0.54 | 0.71 | 1.25 | 0.78 | 1.45 | -0.01 | 0.01 | 0.00 | -0.03 | -0.02 | -0.03 |
| K20866 | 0.93 | 0.77 | 0.74 | 0.68 | 0.98 | 0.87 | 0.02 | 0.00 | 0.00 | -0.02 | -0.03 | 0.02 |
| K07741 | 0.71 | 1.33 | 1.67 | 1.65 | 0.51 | 0.77 | -0.01 | 0.06 | 0.03 | 0.03 | -0.01 | 0.02 |
| K06885 | 0.77 | 0.60 | 0.82 | 0.74 | 0.94 | 1.06 | 0.00 | 0.00 | 0.01 | 0.02 | -0.01 | 0.02 |
| K23535 | 0.74 | 1.55 | 0.84 | 0.69 | 0.86 | 0.80 | 0.00 | -0.04 | 0.01 | -0.01 | 0.01 | -0.01 |
| K06177 | 0.41 | 0.80 | 1.13 | 2.06 | 2.74 | 0.74 | 0.01 | 0.03 | 0.02 | 0.05 | -0.09 | 0.02 |
| K23536 | 0.79 | 1.46 | 0.88 | 0.74 | 0.93 | 0.63 | 0.00 | -0.04 | 0.01 | -0.02 | 0.01 | 0.00 |
| K00991 | 1.51 | 0.75 | 0.76 | 0.58 | 1.06 | 1.27 | -0.03 | 0.04 | 0.00 | -0.01 | -0.03 | -0.03 |

| | | | | | | | | | | | | |
|---|---|---|---|---|---|---|---|---|---|---|---|---|
| K01997 | 0.78 | 0.91 | 0.91 | 1.07 | 0.79 | 0.77 | 0.00 | 0.00 | 0.01 | 0.01 | 0.00 | -0.01 |
| K07105 | 0.87 | 0.88 | 0.80 | 0.69 | 0.87 | 0.76 | 0.00 | 0.00 | 0.00 | 0.00 | -0.02 | 0.01 |
| K09922 | 1.12 | 0.85 | 0.76 | 0.51 | 0.62 | 1.93 | 0.02 | -0.04 | 0.00 | 0.01 | 0.00 | 0.05 |
| K01815 | 1.43 | 0.75 | 0.80 | 1.06 | 0.24 | 1.03 | 0.04 | 0.03 | 0.02 | 0.03 | 0.00 | 0.03 |
| K01952 | 0.78 | 0.76 | 0.93 | 1.39 | 0.75 | 0.93 | 0.01 | 0.03 | 0.01 | 0.02 | 0.01 | 0.02 |
| K21908 | 0.67 | 0.62 | 1.05 | 1.02 | 1.42 | 0.73 | -0.02 | -0.03 | 0.02 | 0.03 | 0.05 | -0.02 |
| K00297 | 0.77 | 0.80 | 0.78 | 1.11 | 1.08 | 0.83 | 0.00 | 0.01 | 0.00 | 0.02 | -0.03 | 0.01 |
| K00817 | 0.90 | 0.77 | 0.74 | 1.38 | 0.69 | 1.01 | 0.01 | 0.04 | 0.00 | 0.02 | -0.02 | 0.00 |
| K01876 | 0.86 | 0.78 | 0.66 | 0.81 | 0.79 | 1.72 | 0.00 | 0.00 | 0.00 | 0.00 | -0.02 | -0.04 |
| K21571 | 0.47 | 0.31 | 0.84 | 1.80 | 1.50 | 0.79 | 0.01 | 0.00 | 0.02 | 0.04 | -0.05 | 0.03 |
| K03106 | 0.62 | 0.91 | 0.83 | 0.79 | 0.79 | 1.05 | 0.01 | -0.01 | 0.01 | 0.00 | -0.01 | 0.00 |
| K00067 | 0.83 | 0.33 | 0.53 | 1.82 | 0.66 | 1.72 | 0.02 | 0.01 | 0.01 | 0.04 | -0.02 | 0.06 |
| K14155 | 0.73 | 0.72 | 1.19 | 1.24 | 0.51 | 1.16 | 0.01 | 0.02 | 0.01 | 0.02 | 0.00 | 0.04 |
| K01740 | 0.83 | 0.34 | 0.86 | 1.22 | 0.79 | 0.44 | -0.01 | 0.00 | 0.01 | 0.02 | -0.03 | 0.01 |
| K07495 | 1.16 | 0.44 | 0.87 | 0.63 | 0.77 | 1.43 | 0.03 | 0.02 | -0.01 | 0.00 | 0.00 | 0.05 |
| K02056 | 0.59 | 1.79 | 0.48 | 0.68 | 0.77 | 1.07 | 0.01 | 0.08 | 0.01 | 0.00 | 0.01 | 0.04 |
| K00878 | 0.85 | 0.78 | 0.95 | 0.75 | 0.61 | 0.38 | -0.01 | 0.00 | 0.01 | 0.00 | 0.01 | 0.00 |
| K22452 | 0.76 | 0.80 | 0.80 | 0.71 | 0.88 | 0.71 | 0.01 | 0.01 | -0.01 | -0.01 | -0.01 | 0.00 |
| K15051 | 0.65 | 0.71 | 0.60 | 0.64 | 0.85 | 1.05 | 0.01 | 0.02 | 0.00 | -0.01 | 0.01 | -0.02 |
| K02647 | 0.92 | 0.61 | 0.74 | 0.69 | 0.69 | 1.21 | -0.01 | 0.00 | -0.01 | -0.02 | -0.02 | -0.03 |
| K12984 | 0.79 | 0.75 | 1.15 | 0.41 | 0.58 | 1.06 | -0.02 | -0.01 | -0.02 | 0.01 | -0.01 | 0.03 |
| K01733 | 0.80 | 0.67 | 0.85 | 0.78 | 0.75 | 1.05 | 0.01 | 0.03 | 0.00 | 0.00 | -0.01 | 0.00 |
| K00604 | 0.58 | 0.67 | 0.77 | 0.76 | 1.00 | 0.93 | 0.01 | 0.02 | 0.00 | 0.00 | -0.02 | 0.01 |
| K02836 | 0.72 | 0.76 | 0.85 | 0.71 | 0.69 | 0.91 | 0.00 | 0.02 | 0.00 | 0.00 | 0.00 | 0.00 |
| K03601 | 0.56 | 0.78 | 0.68 | 0.78 | 0.78 | 1.00 | 0.00 | 0.01 | 0.00 | 0.00 | -0.01 | 0.01 |
| K07484 | 0.76 | 0.62 | 1.10 | 0.55 | 0.73 | 0.73 | 0.01 | 0.03 | 0.01 | 0.00 | -0.01 | 0.02 |

Partial Least Squares analysis was performed with 2 latent components. Subscript VIP≥0.8 denotes microbial genera that had VIP ≥ 0.8 in 4 or more timepoints. Subscripts (+) and (-) represent microbial genera that had positive or negative regression coefficient in 4 or more timepoints, respectively.

**Trait: Residual Feed Intake (RFI); Predictors: 214 microbial genes (MG)**

| Microbial genes | VIP | | | | | | Regression coefficient | | | | | |
|---|---|---|---|---|---|---|---|---|---|---|---|---|
| | T1 | T2 | T3 | T4 | T5 | T6 | T1 | T2 | T3 | T4 | T5 | T6 |

| | | | | | | | | | | | | |
|---|---|---|---|---|---|---|---|---|---|---|---|---|
| $K08094_{VIP\geq0.8\ (+)}$ | 1.02 | 0.53 | 1.52 | 1.14 | 0.83 | 0.75 | 0.03 | 0.01 | 0.03 | 0.02 | 0.02 | 0.03 |
| $K03709_{VIP\geq0.8\ (+)}$ | 1.60 | 1.86 | 0.91 | 0.35 | 0.41 | 1.26 | 0.05 | 0.05 | 0.02 | 0.00 | 0.01 | 0.04 |
| $K11261_{VIP\geq0.8\ (+)}$ | 0.89 | 1.23 | 1.42 | 1.22 | 0.39 | 0.45 | 0.03 | 0.03 | 0.03 | 0.02 | 0.01 | -0.01 |
| $K03105_{VIP\geq0.8\ (+)}$ | 0.98 | 0.67 | 1.04 | 0.83 | 0.91 | 0.71 | 0.01 | 0.02 | 0.01 | 0.00 | 0.02 | 0.02 |
| $K01823_{VIP\geq0.8\ (+)}$ | 0.76 | 0.99 | 1.04 | 0.76 | 0.91 | 0.93 | -0.01 | 0.02 | 0.02 | 0.01 | 0.02 | 0.03 |
| $K03540_{VIP\geq0.8\ (+)}$ | 0.87 | 0.82 | 1.32 | 0.81 | 0.82 | 0.52 | 0.01 | 0.02 | 0.02 | 0.00 | 0.01 | 0.00 |
| $K03264_{VIP\geq0.8\ (+)}$ | 0.93 | 0.83 | 1.24 | 0.80 | 0.91 | 0.91 | 0.01 | 0.02 | 0.02 | 0.01 | 0.01 | -0.02 |
| $K02866_{VIP\geq0.8\ (+)}$ | 0.89 | 1.09 | 1.07 | 0.78 | 1.03 | 0.90 | 0.01 | 0.03 | 0.01 | 0.00 | 0.02 | -0.02 |
| $K07573_{VIP\geq0.8\ (+)}$ | 0.96 | 0.55 | 1.14 | 0.81 | 0.84 | 0.61 | 0.02 | 0.01 | 0.01 | 0.00 | 0.01 | -0.01 |
| $K01815_{VIP\geq0.8\ (+)}$ | 1.63 | 1.31 | 0.65 | 0.63 | 0.98 | 1.16 | 0.05 | 0.02 | 0.01 | 0.01 | 0.03 | 0.03 |
| $K03626_{VIP\geq0.8\ (+)}$ | 0.97 | 0.57 | 1.04 | 0.90 | 0.90 | 0.51 | 0.01 | 0.01 | 0.01 | 0.00 | 0.01 | 0.01 |
| $K04798_{VIP\geq0.8\ (+)}$ | 0.97 | 0.24 | 1.11 | 0.83 | 0.92 | 0.80 | 0.01 | 0.00 | 0.01 | 0.00 | 0.02 | -0.01 |
| $K02908_{VIP\geq0.8\ (+)}$ | 0.98 | 0.56 | 1.03 | 0.94 | 0.92 | 0.71 | 0.00 | 0.01 | 0.01 | 0.00 | 0.01 | 0.00 |
| $K02889_{VIP\geq0.8\ (+)}$ | 0.96 | 0.54 | 1.01 | 0.88 | 0.92 | 0.77 | 0.01 | 0.01 | 0.01 | 0.00 | 0.01 | -0.01 |
| $K03236_{VIP\geq0.8\ (+)}$ | 1.01 | 0.20 | 1.02 | 0.83 | 0.88 | 0.55 | 0.00 | 0.00 | 0.01 | 0.00 | 0.01 | 0.00 |
| $K03120_{VIP\geq0.8\ (+)}$ | 0.97 | 0.52 | 1.03 | 1.00 | 0.93 | 0.55 | 0.00 | 0.01 | 0.01 | 0.00 | 0.02 | 0.00 |
| $K19755_{VIP\geq0.8\ (+)}$ | 0.95 | 0.60 | 1.03 | 0.81 | 0.87 | 0.78 | -0.01 | 0.02 | 0.01 | 0.00 | 0.01 | 0.00 |
| $K07075_{VIP\geq0.8\ (+)}$ | 0.82 | 1.67 | 1.04 | 0.87 | 0.75 | 0.83 | -0.01 | 0.04 | 0.02 | -0.01 | 0.00 | 0.01 |
| $K02875_{VIP\geq0.8\ (+)}$ | 1.02 | 0.51 | 0.95 | 0.91 | 0.89 | 0.72 | 0.00 | 0.01 | 0.00 | 0.00 | 0.01 | -0.01 |
| $K03263_{VIP\geq0.8\ (+)}$ | 1.01 | 0.48 | 1.00 | 0.95 | 0.91 | 0.76 | 0.00 | 0.01 | 0.01 | 0.00 | 0.01 | -0.01 |
| $K11260_{VIP\geq0.8\ (+)}$ | 0.85 | 0.78 | 1.19 | 0.71 | 0.94 | 1.56 | 0.01 | 0.01 | 0.02 | 0.01 | 0.00 | -0.04 |
| $K03564_{VIP\geq0.8\ (+)}$ | 0.88 | 0.94 | 0.97 | 0.92 | 0.64 | 0.83 | 0.00 | -0.03 | 0.01 | 0.01 | 0.00 | 0.03 |
| $K02966_{VIP\geq0.8\ (+)}$ | 1.00 | 0.75 | 0.99 | 1.04 | 0.89 | 1.06 | 0.00 | 0.02 | 0.01 | 0.00 | 0.01 | -0.02 |
| $K02912_{VIP\geq0.8\ (+)}$ | 1.08 | 0.35 | 0.94 | 0.89 | 0.91 | 0.48 | 0.00 | 0.01 | 0.01 | 0.00 | 0.00 | 0.01 |
| $K02991_{VIP\geq0.8\ (+)}$ | 0.99 | 0.52 | 0.97 | 0.93 | 0.91 | 1.05 | 0.00 | 0.01 | 0.00 | 0.00 | 0.01 | -0.02 |
| $K02337_{VIP\geq0.8\ (+)}$ | 0.83 | 1.51 | 0.72 | 0.87 | 0.89 | 1.30 | -0.01 | -0.04 | 0.00 | 0.00 | 0.01 | 0.02 |
| $K04047_{VIP\geq0.8\ (+)}$ | 0.76 | 0.91 | 0.82 | 1.01 | 0.85 | 0.84 | 0.00 | -0.02 | 0.00 | 0.01 | 0.00 | -0.02 |
| $K07484_{VIP\geq0.8\ (+)}$ | 0.96 | 1.09 | 1.33 | 1.96 | 0.82 | 1.58 | 0.01 | 0.02 | 0.02 | 0.03 | 0.00 | 0.04 |
| $K02032_{VIP\geq0.8\ (+)}$ | 0.98 | 1.38 | 0.84 | 0.89 | 1.21 | 1.05 | -0.02 | -0.03 | 0.01 | 0.00 | 0.01 | 0.00 |
| $K01156_{VIP\geq0.8\ (+)}$ | 1.95 | 1.23 | 1.17 | 0.42 | 1.24 | 0.91 | 0.05 | 0.03 | 0.02 | 0.00 | 0.02 | 0.01 |

| | | | | | | | | | | | | |
|---|---|---|---|---|---|---|---|---|---|---|---|---|
| $K07492_{VIP\geq0.8\ (+)}$ | 0.86 | 0.65 | 0.82 | 0.72 | 1.19 | 1.01 | 0.01 | 0.01 | 0.00 | 0.01 | -0.02 | -0.02 |
| $K17290_{VIP\geq0.8\ (+)}$ | 0.67 | 1.22 | 0.93 | 1.67 | 0.95 | 1.04 | 0.00 | 0.03 | 0.01 | -0.02 | -0.01 | 0.03 |
| $K02035_{VIP\geq0.8\ (+)}$ | 0.95 | 0.72 | 0.84 | 0.90 | 1.12 | 0.93 | 0.00 | 0.00 | 0.00 | -0.01 | 0.01 | 0.00 |
| $K02031_{VIP\geq0.8\ (+)}$ | 1.00 | 1.48 | 0.96 | 1.02 | 0.95 | 1.06 | -0.02 | -0.03 | 0.01 | 0.01 | 0.00 | 0.00 |
| $K01258_{VIP\geq0.8\ (+)}$ | 1.02 | 0.82 | 0.92 | 0.88 | 1.37 | 1.21 | 0.01 | -0.02 | 0.00 | -0.01 | 0.02 | 0.01 |
| $K02995_{VIP\geq0.8\ (+)}$ | 1.01 | 0.34 | 0.96 | 0.97 | 0.90 | 1.59 | 0.00 | 0.01 | 0.00 | 0.00 | 0.01 | -0.04 |
| $K02217_{VIP\geq0.8\ (+)}$ | 1.27 | 0.94 | 0.68 | 1.35 | 1.91 | 1.70 | 0.03 | 0.02 | -0.01 | 0.02 | 0.04 | 0.04 |
| $K23535_{VIP\geq0.8\ (+)}$ | 0.91 | 1.44 | 0.90 | 0.79 | 0.97 | 1.09 | -0.01 | -0.03 | 0.01 | 0.00 | 0.00 | 0.01 |
| $K09825_{VIP\geq0.8\ (+)}$ | 1.00 | 0.91 | 0.82 | 0.83 | 1.03 | 0.82 | 0.00 | 0.02 | 0.00 | 0.00 | 0.01 | 0.00 |
| $K07402_{VIP\geq0.8\ (+)}$ | 0.98 | 1.23 | 1.11 | 0.91 | 1.06 | 1.07 | -0.02 | -0.03 | 0.01 | 0.01 | 0.01 | 0.01 |
| $K01999_{VIP\geq0.8\ (+)}$ | 1.01 | 0.84 | 0.82 | 0.84 | 1.09 | 1.11 | 0.00 | 0.02 | 0.00 | 0.00 | 0.01 | -0.01 |
| $K03816_{VIP\geq0.8\ (+)}$ | 1.00 | 0.75 | 1.08 | 1.00 | 1.48 | 1.30 | 0.02 | -0.01 | 0.02 | 0.01 | 0.03 | 0.02 |
| $K03723_{VIP\geq0.8\ (+)}$ | 0.86 | 0.61 | 0.73 | 0.81 | 1.30 | 1.36 | -0.01 | 0.01 | 0.00 | 0.00 | 0.02 | 0.02 |
| $K07172_{VIP\geq0.8\ (+)}$ | 2.17 | 1.42 | 0.64 | 0.48 | 1.27 | 0.80 | 0.06 | 0.02 | 0.01 | 0.00 | 0.02 | -0.02 |
| $K00020_{VIP\geq0.8\ (+)}$ | 1.29 | 1.51 | 0.85 | 0.97 | 0.80 | 1.09 | 0.02 | -0.03 | 0.00 | 0.01 | 0.00 | 0.01 |
| $K02025_{VIP\geq0.8\ (+)}$ | 1.05 | 1.12 | 1.14 | 0.87 | 0.87 | 1.21 | -0.02 | 0.01 | 0.01 | 0.00 | 0.00 | 0.01 |
| $K07316_{VIP\geq0.8\ (+)}$ | 1.99 | 1.16 | 0.94 | 0.60 | 1.03 | 0.87 | 0.05 | 0.03 | 0.01 | 0.00 | 0.02 | 0.02 |
| $K02470_{VIP\geq0.8\ (+)}$ | 0.91 | 0.57 | 1.06 | 0.77 | 0.95 | 1.08 | 0.01 | -0.01 | 0.01 | -0.01 | 0.01 | 0.01 |
| $K02056_{VIP\geq0.8\ (+)}$ | 0.87 | 2.12 | 0.38 | 2.03 | 0.61 | 0.95 | 0.01 | 0.05 | 0.01 | 0.03 | -0.01 | 0.01 |
| $K05832_{VIP\geq0.8\ (+)}$ | 0.80 | 1.27 | 0.82 | 0.75 | 0.99 | 1.12 | -0.01 | 0.02 | 0.01 | 0.00 | 0.00 | 0.01 |
| $K01610_{VIP\geq0.8\ (+)}$ | 0.58 | 0.75 | 0.94 | 1.15 | 1.04 | 0.96 | 0.01 | -0.02 | 0.01 | 0.01 | 0.01 | 0.01 |
| $K08963_{VIP\geq0.8\ (+)}$ | 0.97 | 0.66 | 1.06 | 1.09 | 1.26 | 1.16 | -0.01 | -0.02 | 0.01 | 0.01 | 0.02 | 0.02 |
| $K04043_{VIP\geq0.8\ (+)}$ | 0.83 | 0.43 | 0.85 | 0.88 | 1.24 | 1.02 | 0.01 | -0.01 | 0.00 | 0.00 | 0.02 | 0.00 |
| $K00626_{VIP\geq0.8\ (+)}$ | 0.89 | 1.20 | 0.91 | 1.15 | 0.96 | 0.80 | -0.01 | 0.02 | 0.01 | 0.01 | 0.00 | -0.01 |
| $K23675_{VIP\geq0.8\ (+)}$ | 0.97 | 1.53 | 0.85 | 0.84 | 0.64 | 0.83 | 0.00 | 0.02 | 0.01 | 0.01 | -0.01 | -0.01 |
| $K21071_{VIP\geq0.8\ (+)}$ | 0.99 | 1.45 | 0.80 | 0.75 | 1.21 | 1.01 | -0.01 | 0.02 | 0.00 | 0.00 | 0.02 | 0.01 |
| $K00927_{VIP\geq0.8\ (+)}$ | 0.84 | 1.17 | 0.99 | 0.92 | 0.59 | 1.37 | 0.01 | -0.03 | 0.01 | 0.00 | 0.00 | 0.03 |
| $K02026_{VIP\geq0.8\ (+)}$ | 1.13 | 1.25 | 1.09 | 0.96 | 0.76 | 1.31 | -0.03 | 0.01 | 0.02 | 0.01 | -0.01 | 0.02 |
| $K03798_{VIP\geq0.8\ (+)}$ | 0.94 | 0.57 | 0.94 | 0.95 | 1.76 | 1.32 | -0.01 | 0.02 | 0.00 | 0.00 | 0.03 | 0.02 |
| $K01997_{VIP\geq0.8\ (+)}$ | 1.05 | 0.58 | 0.98 | 0.83 | 0.94 | 0.97 | 0.01 | 0.01 | 0.01 | 0.00 | 0.01 | -0.01 |

| | | | | | | | | | | | |
|---|---|---|---|---|---|---|---|---|---|---|---|---|
| $K10119_{VIP\geq0.8\ (+)}$ | 1.06 | 1.63 | 1.41 | 0.78 | 0.60 | 0.96 | -0.02 | 0.02 | 0.02 | 0.00 | -0.01 | 0.00 |
| $K00763_{VIP\geq0.8\ (+)}$ | 1.15 | 1.07 | 0.85 | 0.90 | 0.99 | 1.03 | 0.02 | 0.02 | 0.00 | 0.00 | 0.01 | 0.00 |
| $K01874_{VIP\geq0.8\ (+)}$ | 0.91 | 0.70 | 1.22 | 1.06 | 1.27 | 1.08 | 0.02 | -0.01 | 0.02 | 0.01 | 0.03 | 0.01 |
| $K01752_{VIP\geq0.8\ (+)}$ | 1.05 | 0.08 | 0.86 | 0.97 | 1.65 | 1.19 | 0.01 | 0.00 | 0.00 | 0.00 | 0.03 | 0.01 |
| $K06177_{VIP\geq0.8\ (+)}$ | 0.49 | 0.97 | 1.04 | 1.22 | 1.91 | 0.35 | 0.01 | 0.00 | 0.02 | 0.02 | -0.05 | 0.01 |
| $K05919_{VIP\geq0.8\ (+)}$ | 1.18 | 1.52 | 0.75 | 1.35 | 0.45 | 0.81 | 0.01 | 0.01 | 0.00 | 0.02 | -0.01 | -0.02 |
| $K07502_{VIP\geq0.8\ (+)}$ | 0.97 | 1.52 | 1.03 | 0.88 | 1.75 | 0.63 | -0.01 | 0.02 | 0.01 | 0.01 | 0.03 | 0.00 |
| $K06959_{VIP\geq0.8\ (+)}$ | 0.96 | 0.41 | 1.40 | 0.95 | 0.25 | 0.95 | -0.03 | 0.01 | 0.02 | 0.01 | 0.00 | 0.01 |
| $K03327_{VIP\geq0.8\ (+)}$ | 0.91 | 0.40 | 1.09 | 1.02 | 1.43 | 1.31 | -0.01 | 0.00 | 0.01 | 0.01 | 0.02 | 0.01 |
| $K00887_{VIP\geq0.8\ (+)}$ | 1.07 | 1.32 | 0.78 | 1.40 | 0.68 | 0.94 | 0.02 | 0.01 | 0.01 | 0.02 | 0.01 | -0.01 |
| $K14155_{VIP\geq0.8\ (+)}$ | 0.89 | 0.15 | 1.09 | 0.85 | 0.62 | 1.03 | 0.02 | 0.00 | 0.01 | 0.00 | 0.00 | 0.01 |
| $K15051_{VIP\geq0.8\ (+)}$ | 0.79 | 1.78 | 1.05 | 0.99 | 0.60 | 1.18 | 0.00 | 0.04 | 0.01 | 0.01 | -0.01 | 0.02 |
| $K23393_{VIP\geq0.8\ (+)}$ | 0.85 | 1.93 | 1.30 | 0.63 | 0.71 | 0.89 | -0.01 | 0.04 | 0.02 | 0.00 | 0.01 | -0.02 |
| $K07720_{VIP\geq0.8\ (+)}$ | 0.87 | 0.57 | 1.18 | 1.01 | 1.52 | 1.35 | 0.00 | 0.01 | 0.01 | 0.01 | 0.02 | 0.01 |
| $K01952_{VIP\geq0.8\ (+)}$ | 0.88 | 0.66 | 0.85 | 0.84 | 1.26 | 1.32 | 0.02 | 0.02 | 0.00 | 0.00 | 0.02 | 0.02 |
| $K11753_{VIP\geq0.8\ (-)}$ | 0.08 | 2.08 | 1.31 | 0.96 | 0.84 | 1.27 | 0.00 | -0.06 | -0.02 | -0.01 | -0.02 | -0.04 |
| $K01091_{VIP\geq0.8\ (-)}$ | 0.78 | 1.33 | 0.90 | 1.05 | 1.23 | 0.92 | -0.01 | -0.03 | -0.01 | -0.02 | -0.03 | -0.01 |
| $K22452_{VIP\geq0.8\ (-)}$ | 0.97 | 1.02 | 1.12 | 1.11 | 0.94 | 0.91 | -0.03 | 0.02 | -0.02 | -0.02 | -0.02 | -0.02 |
| $K00297_{VIP\geq0.8\ (-)}$ | 0.61 | 0.90 | 0.85 | 1.21 | 1.22 | 1.03 | -0.01 | -0.02 | 0.00 | -0.02 | -0.03 | -0.03 |
| $K13283_{VIP\geq0.8\ (-)}$ | 0.33 | 0.84 | 2.26 | 0.85 | 1.04 | 0.61 | 0.01 | -0.02 | -0.04 | -0.01 | -0.03 | -0.02 |
| $K06020_{VIP\geq0.8\ (-)}$ | 1.49 | 1.65 | 1.09 | 1.90 | 0.90 | 1.00 | -0.04 | -0.03 | -0.02 | -0.03 | -0.02 | -0.03 |
| $K01139_{VIP\geq0.8\ (-)}$ | 0.91 | 0.58 | 0.89 | 0.89 | 1.20 | 1.18 | -0.02 | -0.01 | 0.00 | -0.01 | -0.03 | -0.03 |
| $K07478_{VIP\geq0.8\ (-)}$ | 0.60 | 0.45 | 1.33 | 1.15 | 0.88 | 0.97 | 0.00 | -0.01 | -0.02 | -0.02 | -0.01 | -0.01 |
| $K00651_{VIP\geq0.8\ (-)}$ | 0.65 | 0.98 | 1.03 | 0.94 | 0.89 | 1.63 | -0.01 | 0.02 | -0.01 | -0.01 | -0.01 | -0.05 |
| $K01738_{VIP\geq0.8\ (-)}$ | 0.83 | 0.90 | 0.77 | 0.97 | 0.57 | 1.32 | -0.03 | 0.01 | -0.01 | -0.01 | -0.01 | -0.04 |
| $K00057_{VIP\geq0.8\ (-)}$ | 0.50 | 0.41 | 1.01 | 0.94 | 1.41 | 1.23 | -0.01 | 0.01 | -0.02 | -0.01 | -0.04 | 0.01 |
| $K00145_{VIP\geq0.8\ (-)}$ | 0.33 | 0.24 | 0.90 | 0.99 | 0.98 | 1.31 | 0.00 | 0.00 | 0.00 | -0.01 | -0.02 | -0.04 |
| $K03601_{VIP\geq0.8\ (-)}$ | 0.46 | 0.33 | 0.99 | 1.16 | 0.86 | 1.03 | -0.01 | -0.01 | -0.01 | -0.02 | 0.00 | 0.00 |
| $K07058_{VIP\geq0.8\ (-)}$ | 1.45 | 0.26 | 1.43 | 1.99 | 1.94 | 0.22 | -0.04 | 0.00 | -0.02 | -0.03 | -0.04 | -0.01 |
| $K02500_{VIP\geq0.8\ (-)}$ | 0.60 | 0.91 | 0.27 | 1.34 | 1.15 | 1.70 | -0.02 | 0.02 | 0.00 | -0.02 | -0.03 | -0.06 |

| | | | | | | | | | | | | |
|---|---|---|---|---|---|---|---|---|---|---|---|---|
| K02072 $_{VIP\geq0.8\ (-)}$ | 1.03 | 1.09 | 0.77 | 0.84 | 1.01 | 1.32 | -0.02 | 0.02 | 0.00 | -0.01 | -0.02 | -0.04 |
| K08307 $_{VIP\geq0.8\ (-)}$ | 0.83 | 0.68 | 1.34 | 1.64 | 1.02 | 0.17 | -0.01 | -0.01 | -0.02 | -0.03 | -0.03 | 0.00 |
| K03208 $_{VIP\geq0.8\ (-)}$ | 1.62 | 0.35 | 1.07 | 1.99 | 1.22 | 1.22 | -0.05 | -0.01 | -0.02 | -0.03 | -0.02 | -0.04 |
| K16787 $_{VIP\geq0.8\ (-)}$ | 1.07 | 1.22 | 0.70 | 0.77 | 0.97 | 0.93 | -0.03 | 0.00 | 0.00 | -0.01 | -0.02 | -0.01 |
| K01092 $_{VIP\geq0.8\ (-)}$ | 1.25 | 0.20 | 0.80 | 1.92 | 1.69 | 0.82 | -0.03 | 0.00 | -0.01 | -0.03 | -0.04 | -0.03 |
| K00077 $_{VIP\geq0.8\ (-)}$ | 1.17 | 0.31 | 0.92 | 0.85 | 0.98 | 0.89 | -0.03 | -0.01 | -0.01 | -0.01 | 0.01 | 0.00 |
| K00820 $_{VIP\geq0.8\ (-)}$ | 1.06 | 0.20 | 1.05 | 0.85 | 0.91 | 0.91 | 0.02 | -0.01 | -0.02 | 0.00 | -0.02 | -0.02 |
| K00088 $_{VIP\geq0.8\ (-)}$ | 0.41 | 0.26 | 0.85 | 0.89 | 0.85 | 0.97 | -0.01 | -0.01 | 0.01 | -0.01 | -0.02 | -0.03 |
| K03596 $_{VIP\geq0.8\ (-)}$ | 0.58 | 0.39 | 0.80 | 1.18 | 1.08 | 1.02 | -0.01 | -0.01 | 0.00 | -0.02 | 0.01 | 0.00 |
| K06987 $_{VIP\geq0.8\ (-)}$ | 1.69 | 1.10 | 0.43 | 0.78 | 1.26 | 1.83 | -0.04 | -0.03 | 0.00 | -0.01 | -0.03 | -0.05 |
| K02996 $_{VIP\geq0.8\ (-)}$ | 0.56 | 1.04 | 0.87 | 1.26 | 1.46 | 0.16 | 0.02 | -0.02 | -0.02 | -0.02 | -0.04 | 0.00 |
| K03741 $_{VIP\geq0.8\ (-)}$ | 0.88 | 0.21 | 0.90 | 0.89 | 0.86 | 0.86 | 0.01 | 0.00 | -0.01 | -0.01 | 0.00 | -0.02 |
| K01696 $_{VIP\geq0.8\ (-)}$ | 0.21 | 1.32 | 0.88 | 0.98 | 0.76 | 1.05 | 0.00 | 0.04 | -0.01 | -0.02 | -0.02 | -0.02 |
| K03154 $_{VIP\geq0.8\ (-)}$ | 1.46 | 1.33 | 1.75 | 1.55 | 1.44 | 0.69 | -0.04 | 0.03 | -0.03 | -0.02 | -0.03 | -0.01 |
| K19304 $_{VIP\geq0.8\ (-)}$ | 0.39 | 1.17 | 1.83 | 1.44 | 0.30 | 1.08 | -0.01 | -0.03 | -0.03 | -0.02 | -0.01 | 0.04 |
| K06207 $_{VIP\geq0.8\ (-)}$ | 0.59 | 1.28 | 0.85 | 0.85 | 0.75 | 1.07 | -0.01 | -0.03 | 0.00 | 0.00 | 0.00 | 0.00 |
| K07118 $_{VIP\geq0.8\ (-)}$ | 0.89 | 0.63 | 0.83 | 0.88 | 0.90 | 0.87 | 0.01 | -0.01 | -0.01 | 0.00 | -0.01 | -0.01 |
| K02029 $_{VIP\geq0.8\ (-)}$ | 1.04 | 1.21 | 0.81 | 0.82 | 0.91 | 1.05 | -0.02 | 0.01 | 0.01 | -0.01 | -0.02 | 0.00 |
| K03215 $_{VIP\geq0.8\ (-)}$ | 1.08 | 0.17 | 0.95 | 0.94 | 0.97 | 1.10 | 0.01 | 0.00 | -0.01 | -0.01 | 0.00 | 0.00 |
| K00940 $_{VIP\geq0.8\ (-)}$ | 1.07 | 1.56 | 1.16 | 1.54 | 1.45 | 0.70 | -0.02 | -0.04 | -0.01 | -0.02 | -0.03 | -0.02 |
| K14059 $_{VIP\geq0.8\ (-)}$ | 0.92 | 1.60 | 0.28 | 0.67 | 0.86 | 0.98 | -0.02 | 0.04 | -0.01 | -0.01 | -0.02 | -0.02 |
| K01733 $_{VIP\geq0.8\ (-)}$ | 0.77 | 1.13 | 0.76 | 0.93 | 0.99 | 1.08 | 0.01 | 0.03 | 0.00 | -0.01 | -0.02 | 0.00 |
| K16898 $_{VIP\geq0.8\ (-)}$ | 1.07 | 1.20 | 0.81 | 0.76 | 0.83 | 0.97 | -0.02 | 0.02 | 0.00 | -0.01 | -0.01 | -0.01 |
| K03685 $_{VIP\geq0.8\ (-)}$ | 1.05 | 0.71 | 0.66 | 0.84 | 0.96 | 1.07 | 0.02 | -0.01 | -0.01 | -0.01 | -0.02 | 0.01 |
| K03581 $_{VIP\geq0.8\ (-)}$ | 0.94 | 1.10 | 0.82 | 0.84 | 1.05 | 0.97 | -0.02 | 0.01 | 0.00 | 0.00 | 0.01 | -0.01 |
| K02837 $_{VIP\geq0.8\ (-)}$ | 0.31 | 1.03 | 0.85 | 0.89 | 0.79 | 0.96 | 0.00 | 0.03 | 0.00 | 0.00 | -0.01 | -0.01 |
| K01005 $_{VIP\geq0.8\ (-)}$ | 1.07 | 1.52 | 0.89 | 0.71 | 0.86 | 0.99 | -0.02 | 0.02 | 0.01 | 0.00 | -0.02 | -0.01 |
| K02469 $_{VIP\geq0.8\ (-)}$ | 0.92 | 0.99 | 0.79 | 0.84 | 1.45 | 1.01 | -0.01 | -0.03 | 0.00 | 0.00 | 0.02 | 0.00 |
| K00817 $_{VIP\geq0.8\ (-)}$ | 0.90 | 0.89 | 0.82 | 0.78 | 0.77 | 1.14 | 0.01 | 0.02 | 0.00 | -0.01 | 0.00 | -0.03 |
| K02099 $_{VIP\geq0.8\ (-)}$ | 0.88 | 1.10 | 0.82 | 1.33 | 0.68 | 1.00 | 0.00 | 0.01 | -0.01 | 0.02 | -0.02 | -0.03 |

| | | | | | | | | | | | | |
|---|---|---|---|---|---|---|---|---|---|---|---|---|
| K07495$_{VIP≥0.8\ (-)}$ | 1.38 | 0.83 | 0.93 | 0.73 | 0.79 | 0.91 | 0.04 | -0.02 | -0.01 | 0.00 | -0.02 | 0.01 |
| K02086$_{VIP≥0.8\ (-)}$ | 0.95 | 1.60 | 0.81 | 0.61 | 0.82 | 0.85 | -0.01 | 0.02 | 0.01 | 0.00 | -0.02 | -0.01 |
| K02358$_{VIP≥0.8\ (-)}$ | 0.67 | 0.30 | 0.81 | 0.85 | 1.34 | 0.94 | 0.00 | -0.01 | 0.00 | -0.01 | 0.02 | -0.01 |
| K02884$_{VIP≥0.8\ (-)}$ | 0.64 | 1.02 | 0.86 | 1.24 | 1.34 | 0.92 | 0.01 | -0.01 | -0.01 | -0.02 | -0.03 | 0.03 |
| K16785$_{VIP≥0.8\ (-)}$ | 0.94 | 1.26 | 0.94 | 0.66 | 0.73 | 1.04 | -0.01 | 0.01 | 0.01 | 0.00 | -0.02 | -0.02 |
| K04085$_{VIP≥0.8\ (-)}$ | 1.35 | 0.62 | 1.39 | 1.32 | 0.85 | 0.47 | 0.04 | -0.01 | -0.03 | -0.02 | 0.01 | -0.01 |
| K03070$_{VIP≥0.8\ (-)}$ | 0.82 | 0.46 | 0.84 | 0.88 | 1.43 | 1.12 | -0.01 | -0.01 | 0.00 | 0.00 | 0.02 | 0.01 |
| K03555$_{VIP≥0.8\ (-)}$ | 1.16 | 0.50 | 0.76 | 0.83 | 0.85 | 1.01 | 0.03 | -0.01 | 0.00 | 0.00 | 0.00 | 0.00 |
| K09157$_{VIP≥0.8\ (-)}$ | 1.03 | 1.45 | 0.93 | 0.77 | 0.75 | 1.06 | -0.01 | 0.03 | 0.00 | 0.00 | -0.01 | 0.00 |
| K07025$_{VIP≥0.8\ (-)}$ | 1.92 | 1.22 | 1.20 | 0.32 | 1.32 | 0.96 | -0.05 | -0.03 | 0.02 | 0.00 | -0.03 | -0.03 |
| K01771$_{VIP≥0.8\ (-)}$ | 0.84 | 0.83 | 0.97 | 1.36 | 1.19 | 1.35 | 0.00 | 0.02 | 0.02 | -0.01 | -0.01 | -0.03 |
| K05595$_{VIP≥0.8\ (-)}$ | 0.86 | 0.55 | 1.86 | 1.09 | 0.40 | 1.19 | 0.02 | -0.01 | -0.03 | -0.02 | 0.00 | 0.04 |
| K09131$_{VIP≥0.8\ (-)}$ | 0.81 | 0.45 | 0.85 | 0.77 | 1.25 | 0.87 | -0.02 | -0.01 | 0.00 | 0.00 | -0.02 | 0.03 |
| K10761$_{VIP≥0.8\ (-)}$ | 1.00 | 0.36 | 0.86 | 1.11 | 0.97 | 1.01 | -0.02 | -0.01 | 0.01 | -0.01 | 0.00 | -0.02 |
| K00856$_{VIP≥0.8\ (-)}$ | 1.37 | 0.19 | 0.95 | 1.09 | 1.05 | 1.10 | -0.02 | 0.00 | 0.00 | -0.01 | -0.01 | -0.02 |
| K04564$_{VIP≥0.8\ (-)}$ | 1.51 | 0.58 | 0.90 | 1.63 | 1.08 | 0.81 | -0.03 | -0.02 | -0.01 | -0.02 | -0.01 | 0.01 |
| K23876$_{VIP≥0.8\ (-)}$ | 0.76 | 1.30 | 1.03 | 1.51 | 0.78 | 0.85 | -0.01 | -0.01 | 0.01 | 0.02 | -0.02 | 0.00 |
| K02233$_{VIP≥0.8\ (-)}$ | 1.41 | 0.94 | 0.96 | 1.61 | 0.71 | 1.64 | -0.04 | 0.02 | 0.02 | -0.02 | 0.00 | -0.05 |
| K16329$_{VIP≥0.8\ (-)}$ | 0.90 | 1.00 | 0.88 | 0.62 | 0.57 | 0.94 | -0.01 | -0.03 | 0.01 | 0.00 | 0.00 | -0.02 |
| K12998$_{VIP≥0.8\ (-)}$ | 0.81 | 0.86 | 0.82 | 0.69 | 0.86 | 0.73 | -0.01 | -0.02 | 0.00 | -0.01 | -0.01 | -0.01 |
| K23997$_{VIP≥0.8}$ | 0.89 | 0.88 | 1.13 | 0.96 | 1.08 | 1.05 | -0.02 | -0.01 | 0.02 | 0.01 | 0.02 | -0.03 |
| K19118$_{VIP≥0.8}$ | 1.32 | 0.91 | 0.93 | 0.83 | 0.88 | 1.50 | 0.02 | 0.01 | -0.01 | 0.00 | 0.00 | 0.03 |
| K08234$_{VIP≥0.8}$ | 1.73 | 1.49 | 0.93 | 0.67 | 1.49 | 1.06 | 0.05 | 0.03 | -0.01 | 0.01 | -0.03 | -0.02 |
| K12962$_{VIP≥0.8}$ | 0.89 | 1.93 | 0.73 | 1.16 | 1.10 | 0.92 | 0.03 | 0.05 | 0.00 | -0.01 | -0.02 | 0.03 |
| K03698$_{VIP≥0.8}$ | 1.06 | 1.42 | 0.85 | 0.64 | 0.92 | 1.13 | -0.02 | -0.03 | 0.01 | -0.01 | 0.00 | 0.02 |
| K19117$_{VIP≥0.8}$ | 1.11 | 0.64 | 0.88 | 0.85 | 0.84 | 1.66 | 0.01 | 0.01 | -0.01 | 0.00 | -0.01 | 0.04 |
| K08602$_{VIP≥0.8}$ | 0.93 | 1.51 | 0.81 | 0.71 | 1.04 | 0.99 | -0.01 | -0.03 | 0.00 | -0.01 | 0.01 | 0.00 |
| K23536$_{VIP≥0.8}$ | 0.91 | 1.84 | 0.91 | 0.70 | 1.07 | 1.24 | -0.01 | -0.04 | 0.01 | 0.00 | 0.01 | 0.02 |
| K00941$_{VIP≥0.8}$ | 1.04 | 1.81 | 0.73 | 1.18 | 0.97 | 0.93 | 0.01 | 0.04 | 0.00 | 0.01 | -0.03 | -0.02 |
| K05833$_{VIP≥0.8}$ | 0.92 | 1.17 | 0.82 | 0.73 | 0.91 | 1.19 | -0.01 | 0.02 | 0.01 | 0.00 | 0.00 | 0.01 |

| | | | | | | | | | | | |
|---|---|---|---|---|---|---|---|---|---|---|---|---|
| $K02037_{VIP≥0.8}$ | 0.97 | 0.56 | 0.88 | 0.91 | 0.98 | 0.80 | 0.00 | 0.00 | -0.01 | -0.01 | 0.00 | -0.01 |
| $K02036_{VIP≥0.8}$ | 0.99 | 0.54 | 0.87 | 1.00 | 1.05 | 0.86 | 0.00 | 0.01 | 0.00 | -0.01 | 0.01 | -0.01 |
| $K07335_{VIP≥0.8}$ | 1.00 | 0.95 | 0.81 | 0.79 | 0.99 | 0.99 | -0.01 | -0.02 | 0.00 | 0.00 | 0.00 | -0.01 |
| $K03778_{VIP≥0.8}$ | 1.05 | 0.18 | 0.81 | 0.88 | 0.97 | 1.38 | 0.01 | 0.00 | 0.00 | -0.01 | 0.01 | -0.04 |
| $K00265_{VIP≥0.8}$ | 1.15 | 0.14 | 0.82 | 0.82 | 0.84 | 0.90 | 0.02 | 0.00 | -0.01 | -0.01 | 0.00 | -0.01 |
| $K02519_{VIP≥0.8}$ | 1.06 | 0.83 | 0.82 | 0.77 | 1.30 | 1.02 | 0.02 | -0.02 | 0.00 | -0.01 | 0.02 | 0.00 |
| $K06985_{VIP≥0.8}$ | 0.29 | 1.84 | 0.98 | 1.62 | 0.70 | 0.82 | 0.00 | 0.05 | -0.02 | -0.03 | 0.02 | 0.02 |
| $K01759_{VIP≥0.8}$ | 1.35 | 1.02 | 1.09 | 0.71 | 0.78 | 1.11 | 0.03 | 0.00 | 0.02 | 0.00 | 0.00 | 0.02 |
| $K21030_{VIP≥0.8}$ | 1.28 | 0.49 | 0.94 | 1.06 | 1.65 | 0.30 | 0.03 | -0.01 | 0.01 | 0.01 | -0.04 | -0.01 |
| $K00573_{VIP≥0.8}$ | 1.13 | 1.11 | 1.23 | 0.73 | 0.72 | 0.87 | 0.03 | 0.02 | -0.02 | -0.01 | -0.02 | 0.03 |
| $K10563_{VIP≥0.8}$ | 1.04 | 0.50 | 1.46 | 0.98 | 0.78 | 0.97 | 0.01 | 0.01 | -0.02 | -0.02 | -0.01 | 0.02 |
| $K01223_{VIP≥0.8}$ | 0.95 | 0.56 | 0.84 | 0.89 | 0.99 | 0.70 | -0.02 | -0.01 | 0.01 | 0.00 | 0.01 | -0.02 |
| $K18908_{VIP≥0.8}$ | 0.93 | 0.40 | 0.83 | 0.80 | 1.49 | 0.76 | -0.02 | 0.00 | 0.00 | 0.00 | 0.03 | -0.01 |
| $K00003_{VIP≥0.8}$ | 0.84 | 1.77 | 1.19 | 0.65 | 0.46 | 0.83 | 0.00 | 0.03 | 0.02 | 0.01 | -0.01 | -0.02 |
| $K03147_{VIP≥0.8}$ | 1.02 | 2.32 | 0.78 | 0.79 | 1.59 | 0.90 | 0.02 | 0.06 | -0.01 | 0.00 | -0.04 | 0.00 |
| $K01687_{VIP≥0.8}$ | 0.94 | 0.28 | 0.92 | 0.84 | 0.74 | 0.96 | 0.00 | -0.01 | 0.00 | 0.00 | -0.01 | -0.02 |
| $K00604_{VIP≥0.8}$ | 0.56 | 0.65 | 1.17 | 1.20 | 1.00 | 1.26 | 0.00 | 0.01 | -0.02 | -0.02 | -0.02 | 0.01 |
| $K07137_{VIP≥0.8}$ | 0.81 | 0.55 | 1.06 | 0.68 | 1.13 | 1.13 | -0.01 | -0.01 | 0.01 | -0.01 | 0.01 | 0.00 |
| $K00558_{VIP≥0.8}$ | 0.82 | 0.84 | 0.64 | 0.96 | 0.42 | 1.45 | 0.00 | 0.02 | -0.01 | -0.02 | 0.00 | 0.03 |
| K14095 | 0.81 | 0.62 | 1.41 | 0.66 | 0.78 | 0.92 | 0.02 | 0.01 | 0.02 | 0.01 | 0.01 | -0.02 |
| K14102 | 0.94 | 0.60 | 1.10 | 0.65 | 0.81 | 0.74 | 0.03 | 0.01 | 0.02 | 0.01 | 0.01 | -0.01 |
| K06875 | 0.77 | 0.54 | 1.05 | 0.81 | 0.90 | 0.72 | 0.01 | 0.01 | 0.01 | 0.00 | 0.02 | -0.01 |
| K07387 | 1.31 | 0.46 | 1.04 | 0.97 | 0.53 | 0.71 | -0.03 | 0.01 | 0.02 | -0.01 | 0.00 | 0.00 |
| K03753 | 0.50 | 0.59 | 1.24 | 1.34 | 1.08 | 0.69 | 0.01 | 0.02 | 0.02 | 0.02 | -0.01 | 0.00 |
| K09764 | 0.79 | 0.44 | 0.84 | 0.59 | 1.61 | 1.23 | 0.00 | 0.00 | 0.01 | 0.01 | -0.03 | -0.03 |
| K09793 | 0.77 | 0.40 | 0.84 | 0.97 | 0.31 | 0.81 | 0.00 | 0.01 | 0.01 | -0.01 | 0.00 | 0.02 |
| K03710 | 0.80 | 1.34 | 1.43 | 0.63 | 0.84 | 0.61 | 0.00 | -0.03 | 0.02 | 0.01 | 0.01 | -0.02 |
| K19048 | 0.87 | 1.11 | 0.71 | 0.60 | 0.77 | 1.03 | 0.01 | 0.02 | 0.01 | 0.00 | -0.01 | 0.02 |
| K09816 | 0.65 | 1.47 | 0.86 | 0.76 | 0.62 | 0.84 | 0.01 | -0.03 | 0.01 | 0.00 | 0.00 | -0.01 |
| K07334 | 1.71 | 0.92 | 0.74 | 0.57 | 0.76 | 0.85 | 0.04 | 0.00 | -0.01 | 0.00 | -0.02 | 0.02 |
| K00882 | 0.95 | 0.88 | 0.77 | 0.77 | 0.68 | 1.02 | -0.01 | 0.01 | 0.00 | -0.01 | -0.01 | -0.02 |
| K03724 | 0.77 | 0.32 | 0.67 | 1.10 | 1.13 | 1.13 | 0.00 | 0.01 | -0.01 | 0.01 | 0.02 | 0.02 |

| | | | | | | | | | | | | |
|---|---|---|---|---|---|---|---|---|---|---|---|---|
| K01709 | 0.59 | 1.32 | 0.44 | 1.27 | 0.79 | 1.32 | -0.02 | -0.03 | 0.01 | -0.02 | -0.02 | -0.04 |
| K01897 | 0.76 | 1.56 | 1.27 | 1.20 | 0.32 | 0.33 | 0.02 | 0.04 | -0.02 | -0.02 | -0.01 | -0.01 |
| K19689 | 1.10 | 0.61 | 0.80 | 0.78 | 1.06 | 1.07 | -0.02 | -0.01 | 0.00 | -0.01 | 0.00 | 0.00 |
| K12950 | 1.02 | 0.73 | 1.18 | 0.85 | 0.72 | 0.74 | -0.03 | 0.02 | -0.02 | -0.01 | 0.00 | 0.00 |
| K01433 | 1.54 | 1.08 | 0.69 | 0.51 | 0.69 | 0.95 | -0.03 | 0.01 | -0.01 | -0.01 | -0.01 | -0.02 |
| K07105 | 0.85 | 1.01 | 0.72 | 0.74 | 0.82 | 0.71 | 0.00 | 0.01 | 0.00 | -0.01 | -0.01 | -0.01 |
| K12132 | 1.11 | 0.88 | 0.77 | 0.73 | 0.70 | 0.81 | -0.03 | 0.01 | 0.01 | -0.01 | -0.01 | -0.02 |
| K00864 | 0.84 | 0.49 | 1.07 | 0.68 | 0.71 | 0.96 | -0.01 | -0.01 | 0.01 | -0.01 | 0.00 | -0.02 |
| K07464 | 1.52 | 0.57 | 0.77 | 0.81 | 0.84 | 0.78 | 0.03 | 0.00 | 0.00 | 0.00 | 0.00 | 0.00 |
| K04751 | 1.19 | 1.56 | 1.45 | 0.79 | 0.74 | 0.62 | 0.03 | 0.04 | -0.03 | 0.00 | 0.00 | -0.01 |
| K09774 | 0.73 | 1.42 | 0.81 | 0.83 | 0.58 | 0.39 | 0.02 | -0.03 | -0.02 | -0.01 | -0.02 | -0.01 |
| K02536 | 0.72 | 1.22 | 1.31 | 1.56 | 0.38 | 0.74 | 0.02 | -0.02 | -0.02 | -0.03 | -0.01 | 0.02 |
| K03972 | 0.77 | 0.08 | 0.30 | 0.96 | 1.49 | 0.91 | 0.02 | 0.00 | 0.00 | -0.02 | -0.03 | -0.03 |
| K24131 | 0.96 | 1.06 | 0.76 | 0.83 | 0.75 | 0.41 | 0.02 | -0.03 | 0.00 | 0.00 | -0.01 | 0.00 |
| K01876 | 0.95 | 0.80 | 0.68 | 0.77 | 0.85 | 1.02 | 0.01 | 0.02 | 0.00 | 0.00 | -0.02 | -0.03 |
| K01756 | 0.61 | 0.20 | 0.79 | 0.94 | 0.82 | 0.92 | -0.01 | 0.00 | -0.01 | -0.01 | 0.01 | -0.01 |
| K03702 | 0.83 | 0.63 | 0.56 | 1.14 | 0.75 | 1.41 | -0.02 | 0.02 | 0.00 | -0.02 | -0.01 | 0.02 |
| K21571 | 1.12 | 0.76 | 0.99 | 1.55 | 0.62 | 0.45 | 0.03 | 0.00 | 0.02 | 0.02 | -0.02 | -0.02 |
| K00826 | 0.82 | 0.34 | 0.77 | 0.82 | 0.67 | 1.28 | -0.02 | 0.00 | 0.00 | -0.01 | -0.01 | -0.04 |
| K02836 | 0.54 | 0.66 | 0.86 | 1.20 | 0.76 | 1.08 | -0.01 | -0.02 | 0.00 | -0.02 | -0.01 | -0.01 |
| K02913 | 0.74 | 0.66 | 0.83 | 1.38 | 1.11 | 0.42 | 0.02 | 0.00 | -0.01 | -0.02 | -0.03 | 0.00 |
| K13993 | 0.98 | 0.29 | 0.68 | 0.82 | 0.89 | 0.78 | 0.02 | 0.01 | 0.00 | 0.00 | -0.01 | 0.00 |
| K07569 | 0.72 | 1.00 | 1.00 | 0.73 | 0.76 | 0.77 | 0.00 | 0.02 | 0.01 | 0.01 | 0.01 | -0.01 |
| K09739 | 0.80 | 0.30 | 1.51 | 1.04 | 0.76 | 0.76 | 0.01 | 0.00 | 0.03 | 0.02 | 0.01 | -0.01 |
| K03538 | 0.96 | 0.60 | 1.27 | 0.73 | 0.78 | 0.47 | 0.00 | 0.01 | 0.02 | 0.01 | 0.01 | 0.00 |
| K06961 | 0.76 | 0.50 | 1.34 | 0.73 | 0.87 | 0.37 | 0.01 | 0.01 | 0.02 | 0.00 | 0.02 | 0.00 |
| K14111 | 0.83 | 0.66 | 1.44 | 0.71 | 0.76 | 0.36 | 0.02 | 0.02 | 0.02 | 0.01 | 0.01 | 0.00 |
| K06874 | 0.84 | 0.75 | 1.29 | 0.75 | 0.75 | 0.49 | 0.01 | 0.02 | 0.02 | 0.00 | 0.01 | 0.00 |
| K03181 | 0.69 | 0.58 | 1.28 | 0.74 | 0.84 | 0.71 | 0.01 | 0.02 | 0.02 | 0.01 | 0.01 | -0.01 |
| K00652 | 1.04 | 0.70 | 0.66 | 1.28 | 0.78 | 0.66 | 0.03 | -0.01 | -0.01 | -0.02 | -0.01 | 0.01 |
| K06204 | 0.78 | 0.30 | 0.99 | 0.99 | 0.75 | 0.77 | 0.00 | 0.01 | -0.01 | -0.01 | -0.01 | 0.01 |
| K01940 | 1.06 | 0.44 | 0.79 | 0.80 | 0.75 | 1.24 | 0.02 | 0.00 | -0.01 | -0.01 | -0.02 | -0.04 |

Partial Least Squares analysis was performed with 2 latent components. Subscript VIP≥0.8 denotes microbial genera that had VIP ≥ 0.8 in 4 or more timepoints. Subscripts (+) and (-) represent microbial genera that had positive or negative regression coefficient in 4 or more timepoints, respectively.

**Trait: Methane Yield (g/kg DMI, CH4Y); Predictors: 223 microbial genes (MG)**

| Microbial genes | VIP | | | | | | Regression coefficient | | | | | |
|---|---|---|---|---|---|---|---|---|---|---|---|---|
| | T1 | T2 | T3 | T4 | T5 | T6 | T1 | T2 | T3 | T4 | T5 | T6 |
| $K10218_{VIP\geq0.8\ (+)}$ | 1.92 | 2.47 | 0.67 | 0.53 | 2.31 | 2.46 | 0.04 | 0.08 | 0.02 | 0.01 | 0.04 | 0.06 |
| $K00441_{VIP\geq0.8\ (+)}$ | 1.44 | 1.95 | 1.39 | 1.67 | 0.48 | 1.92 | 0.03 | 0.06 | 0.03 | 0.03 | 0.01 | 0.04 |
| $K01156_{VIP\geq0.8\ (+)}$ | 0.60 | 1.54 | 1.86 | 1.50 | 1.92 | 0.97 | 0.01 | 0.01 | 0.04 | 0.03 | 0.03 | 0.02 |
| $K00641_{VIP\geq0.8\ (+)}$ | 0.21 | 1.19 | 1.67 | 1.11 | 0.56 | 2.09 | 0.00 | 0.02 | 0.04 | 0.02 | 0.00 | 0.05 |
| $K07316_{VIP\geq0.8\ (+)}$ | 1.24 | 1.25 | 1.50 | 1.26 | 1.95 | 0.90 | 0.03 | 0.02 | 0.03 | 0.02 | 0.03 | 0.02 |
| $K00558_{VIP\geq0.8\ (+)}$ | 0.70 | 2.25 | 1.16 | 0.57 | 1.55 | 1.57 | 0.00 | 0.06 | 0.03 | 0.00 | 0.02 | 0.03 |
| $K12452_{VIP\geq0.8\ (+)}$ | 0.79 | 1.42 | 0.13 | 0.98 | 0.95 | 1.78 | 0.02 | 0.02 | 0.00 | 0.02 | 0.02 | 0.04 |
| $K00125_{VIP\geq0.8\ (+)}$ | 0.94 | 1.08 | 1.24 | 2.08 | 1.33 | 1.42 | -0.01 | 0.01 | 0.02 | 0.04 | 0.02 | 0.03 |
| $K10725_{VIP\geq0.8\ (+)}$ | 0.94 | 1.23 | 1.42 | 0.51 | 0.99 | 1.30 | 0.02 | 0.03 | 0.03 | 0.01 | -0.01 | 0.02 |
| $K03181_{VIP\geq0.8\ (+)}$ | 0.96 | 0.68 | 1.09 | 0.90 | 0.89 | 1.48 | 0.01 | 0.01 | 0.02 | 0.02 | 0.00 | 0.03 |
| $K20608_{VIP\geq0.8\ (+)}$ | 1.28 | 1.12 | 0.91 | 1.11 | 0.79 | 0.75 | 0.02 | 0.04 | 0.02 | 0.02 | 0.01 | 0.01 |
| $K00046_{VIP\geq0.8\ (+)}$ | 0.53 | 1.78 | 0.83 | 0.37 | 1.09 | 0.83 | -0.01 | 0.01 | 0.01 | 0.00 | 0.02 | 0.02 |
| $K08094_{VIP\geq0.8\ (+)}$ | 0.80 | 1.08 | 0.48 | 1.18 | 0.41 | 1.40 | -0.02 | 0.03 | 0.01 | 0.02 | 0.00 | 0.03 |
| $K03702_{VIP\geq0.8\ (+)}$ | 1.10 | 1.70 | 1.17 | 1.05 | 0.86 | 1.06 | 0.02 | 0.03 | 0.02 | 0.00 | 0.00 | 0.01 |
| $K00088_{VIP\geq0.8\ (+)}$ | 0.57 | 1.52 | 1.05 | 0.86 | 0.89 | 1.58 | -0.01 | 0.01 | 0.02 | 0.00 | 0.01 | 0.03 |
| $K14102_{VIP\geq0.8\ (+)}$ | 1.42 | 0.33 | 1.16 | 0.80 | 1.15 | 0.92 | 0.02 | 0.00 | 0.02 | 0.01 | -0.01 | 0.00 |
| $K04796_{VIP\geq0.8\ (+)}$ | 1.29 | 0.55 | 0.94 | 0.62 | 1.16 | 1.60 | 0.02 | 0.01 | 0.02 | 0.00 | -0.01 | 0.03 |
| $K09116_{VIP\geq0.8\ (+)}$ | 1.34 | 0.86 | 1.04 | 0.68 | 0.98 | 1.08 | 0.02 | 0.03 | 0.02 | 0.00 | -0.01 | 0.01 |
| $K01771_{VIP\geq0.8\ (+)}$ | 1.09 | 0.40 | 0.93 | 0.82 | 1.17 | 0.90 | 0.01 | 0.01 | 0.01 | 0.01 | -0.01 | 0.02 |
| $K00826_{VIP\geq0.8\ (+)}$ | 0.78 | 1.45 | 1.13 | 1.06 | 0.60 | 0.89 | 0.02 | 0.02 | 0.02 | 0.01 | 0.00 | 0.01 |
| $K14059_{VIP\geq0.8\ (+)}$ | 0.77 | 1.68 | 0.51 | 1.45 | 1.21 | 0.85 | 0.01 | 0.06 | 0.00 | 0.02 | 0.01 | 0.01 |
| $K01915_{VIP\geq0.8\ (+)}$ | 0.66 | 1.54 | 0.82 | 1.51 | 0.77 | 1.02 | 0.00 | 0.01 | 0.01 | 0.02 | -0.01 | 0.02 |
| $K03638_{VIP\geq0.8\ (+)}$ | 1.47 | 0.61 | 0.81 | 0.73 | 1.01 | 0.99 | 0.03 | 0.01 | 0.02 | 0.00 | -0.01 | 0.01 |
| $K03723_{VIP\geq0.8\ (+)}$ | 0.83 | 1.36 | 1.30 | 1.36 | 0.90 | 0.91 | 0.00 | 0.01 | 0.02 | 0.01 | 0.00 | 0.00 |
| $K01874_{VIP\geq0.8\ (+)}$ | 0.57 | 1.13 | 1.06 | 1.00 | 1.15 | 1.05 | 0.00 | 0.00 | 0.02 | 0.01 | 0.02 | 0.01 |

| | | | | | | | | | | | | |
|---|---|---|---|---|---|---|---|---|---|---|---|---|
| K02337$_{VIP\geq0.8\ (+)}$ | 0.87 | 1.38 | 1.06 | 1.25 | 0.66 | 1.14 | 0.00 | 0.01 | 0.02 | 0.01 | 0.00 | 0.01 |
| K01876$_{VIP\geq0.8\ (+)}$ | 0.89 | 0.77 | 1.12 | 1.45 | 1.09 | 1.19 | 0.00 | 0.00 | 0.02 | 0.02 | 0.01 | 0.02 |
| K03057$_{VIP\geq0.8\ (+)}$ | 0.76 | 0.88 | 0.85 | 0.61 | 1.16 | 1.05 | 0.00 | 0.03 | 0.02 | 0.01 | -0.01 | 0.01 |
| K07458$_{VIP\geq0.8\ (+)}$ | 1.29 | 1.62 | 1.30 | 1.03 | 0.73 | 0.62 | -0.02 | 0.04 | 0.03 | -0.02 | 0.01 | 0.01 |
| K06885$_{VIP\geq0.8\ (+)}$ | 0.97 | 1.22 | 0.89 | 0.63 | 0.89 | 0.85 | -0.02 | 0.01 | 0.01 | 0.00 | 0.00 | 0.01 |
| K01756$_{VIP\geq0.8\ (+)}$ | 0.79 | 1.58 | 1.00 | 0.82 | 0.87 | 0.99 | -0.01 | 0.03 | 0.01 | 0.00 | 0.01 | 0.02 |
| K12132$_{VIP\geq0.8\ (+)}$ | 1.39 | 0.32 | 0.79 | 0.99 | 0.99 | 1.00 | 0.03 | 0.01 | 0.00 | 0.01 | 0.01 | 0.02 |
| K12373$_{VIP\geq0.8\ (+)}$ | 0.84 | 1.24 | 0.51 | 0.88 | 1.23 | 1.96 | -0.01 | 0.03 | 0.00 | -0.02 | 0.02 | 0.05 |
| K00927$_{VIP\geq0.8\ (+)}$ | 1.09 | 1.38 | 1.08 | 0.97 | 0.65 | 0.77 | 0.02 | -0.03 | 0.02 | 0.01 | 0.00 | 0.01 |
| K02313$_{VIP\geq0.8\ (+)}$ | 0.55 | 1.39 | 1.04 | 1.22 | 0.81 | 1.08 | 0.01 | 0.03 | 0.02 | 0.01 | -0.01 | 0.01 |
| K03540$_{VIP\geq0.8\ (+)}$ | 0.96 | 0.28 | 0.84 | 0.91 | 1.07 | 1.37 | 0.01 | 0.01 | 0.00 | 0.00 | -0.01 | 0.03 |
| K04043$_{VIP\geq0.8\ (+)}$ | 0.99 | 0.92 | 1.05 | 1.68 | 0.72 | 1.04 | -0.01 | 0.01 | 0.01 | 0.02 | -0.01 | 0.01 |
| K01465$_{VIP\geq0.8\ (+)}$ | 0.95 | 1.29 | 0.70 | 1.01 | 0.62 | 1.06 | -0.01 | 0.02 | 0.01 | 0.01 | -0.01 | 0.02 |
| K02470$_{VIP\geq0.8\ (+)}$ | 0.92 | 1.40 | 0.75 | 0.92 | 0.58 | 1.23 | -0.01 | 0.03 | 0.00 | 0.01 | -0.01 | 0.02 |
| K00937$_{VIP\geq0.8\ (+)}$ | 0.87 | 0.79 | 0.94 | 1.20 | 0.97 | 1.01 | 0.00 | -0.01 | 0.00 | 0.01 | -0.01 | 0.00 |
| K04564$_{VIP\geq0.8\ (+)}$ | 1.03 | 0.76 | 0.84 | 1.02 | 0.71 | 1.00 | 0.01 | 0.02 | 0.00 | -0.01 | 0.00 | 0.01 |
| K07464$_{VIP\geq0.8\ (+)}$ | 0.86 | 0.03 | 0.96 | 1.35 | 1.02 | 1.05 | 0.00 | 0.00 | 0.01 | 0.02 | 0.01 | 0.02 |
| K01952$_{VIP\geq0.8\ (+)}$ | 0.73 | 1.16 | 1.25 | 0.96 | 0.68 | 1.05 | 0.00 | -0.02 | 0.02 | 0.01 | 0.00 | 0.00 |
| K08963$_{VIP\geq0.8\ (+)}$ | 0.87 | 0.53 | 0.90 | 1.06 | 0.91 | 1.15 | 0.01 | 0.01 | 0.00 | 0.01 | 0.00 | 0.02 |
| K02519$_{VIP\geq0.8\ (+)}$ | 1.35 | 1.14 | 1.15 | 0.86 | 0.91 | 0.92 | -0.02 | 0.00 | 0.02 | 0.00 | 0.01 | 0.00 |
| K01687$_{VIP\geq0.8\ (+)}$ | 0.84 | 0.60 | 1.00 | 1.09 | 0.70 | 0.88 | 0.01 | 0.01 | 0.01 | 0.01 | -0.01 | -0.01 |
| K02908$_{VIP\geq0.8\ (+)}$ | 0.90 | 0.36 | 0.89 | 1.10 | 1.09 | 0.95 | 0.00 | 0.01 | 0.00 | -0.01 | -0.01 | 0.01 |
| K03070$_{VIP\geq0.8\ (+)}$ | 0.98 | 0.95 | 0.99 | 0.86 | 0.92 | 0.92 | 0.00 | 0.00 | 0.01 | 0.00 | 0.00 | 0.00 |
| K02469$_{VIP\geq0.8\ (+)}$ | 0.87 | 0.37 | 1.00 | 0.82 | 0.81 | 1.05 | 0.01 | 0.00 | 0.01 | 0.00 | 0.00 | 0.01 |
| K12998$_{VIP\geq0.8\ (+)}$ | 0.81 | 0.46 | 0.76 | 1.09 | 1.02 | 0.91 | 0.01 | 0.01 | 0.00 | -0.02 | -0.01 | 0.02 |
| K01953$_{VIP\geq0.8\ (+)}$ | 0.81 | 0.50 | 1.02 | 0.94 | 0.86 | 0.76 | 0.01 | 0.00 | 0.01 | 0.00 | 0.01 | 0.00 |
| K07137$_{VIP\geq0.8\ (+)}$ | 0.95 | 1.15 | 0.88 | 0.79 | 0.95 | 0.84 | -0.01 | 0.02 | 0.01 | -0.01 | 0.00 | 0.01 |
| K04070$_{VIP\geq0.8\ (+)}$ | 1.02 | 1.00 | 0.85 | 0.87 | 0.93 | 0.58 | 0.02 | 0.02 | -0.01 | 0.00 | 0.00 | 0.01 |
| K03215$_{VIP\geq0.8\ (+)}$ | 0.96 | 0.56 | 1.00 | 1.00 | 0.90 | 0.92 | 0.02 | 0.01 | 0.00 | 0.00 | 0.00 | -0.01 |
| K02217$_{VIP\geq0.8\ (+)}$ | 0.94 | 0.84 | 0.93 | 1.04 | 0.76 | 0.72 | -0.01 | 0.03 | 0.01 | 0.01 | -0.01 | 0.01 |

| | | | | | | | | | | | | |
|---|---|---|---|---|---|---|---|---|---|---|---|---|
| $K01999_{VIP≥0.8\ (+)}$ | 0.88 | 0.37 | 0.94 | 0.88 | 0.90 | 1.00 | 0.01 | 0.01 | 0.00 | 0.00 | 0.00 | 0.01 |
| $K03529_{VIP≥0.8\ (+)}$ | 0.93 | 0.44 | 0.88 | 1.22 | 0.97 | 0.80 | 0.01 | 0.00 | 0.00 | 0.01 | 0.00 | 0.00 |
| $K14475_{VIP≥0.8\ (+)}$ | 0.89 | 1.32 | 0.93 | 0.82 | 0.31 | 0.97 | 0.01 | -0.01 | -0.01 | 0.01 | 0.00 | 0.02 |
| $K00013_{VIP≥0.8\ (+)}$ | 1.43 | 1.14 | 0.48 | 0.91 | 0.69 | 0.91 | -0.02 | 0.01 | -0.01 | 0.00 | 0.00 | 0.01 |
| $K09157_{VIP≥0.8\ (+)}$ | 0.91 | 0.51 | 0.90 | 1.28 | 0.90 | 0.84 | 0.01 | 0.01 | 0.00 | 0.01 | 0.00 | 0.00 |
| $K01488_{VIP≥0.8\ (-)}$ | 1.49 | 1.42 | 1.51 | 1.09 | 1.53 | 0.93 | -0.03 | -0.02 | -0.03 | -0.02 | -0.03 | -0.02 |
| $K05337_{VIP≥0.8\ (-)}$ | 1.12 | 1.67 | 1.84 | 0.84 | 1.51 | 1.81 | 0.03 | 0.00 | -0.04 | -0.02 | -0.03 | -0.04 |
| $K02884_{VIP≥0.8\ (-)}$ | 0.85 | 1.06 | 1.30 | 1.28 | 1.57 | 0.18 | -0.02 | -0.03 | -0.03 | -0.03 | -0.03 | 0.00 |
| $K06416_{VIP≥0.8\ (-)}$ | 0.75 | 1.41 | 1.29 | 0.89 | 1.19 | 0.56 | -0.01 | -0.01 | -0.03 | -0.02 | -0.02 | -0.01 |
| $K07005_{VIP≥0.8\ (-)}$ | 1.82 | 0.92 | 1.76 | 1.50 | 1.22 | 1.01 | -0.04 | 0.03 | -0.04 | -0.03 | -0.02 | -0.02 |
| $K15876_{VIP≥0.8\ (-)}$ | 1.48 | 1.71 | 0.36 | 1.06 | 1.31 | 2.00 | -0.03 | -0.06 | -0.01 | -0.02 | -0.03 | -0.05 |
| $K09705_{VIP≥0.8\ (-)}$ | 0.82 | 1.44 | 1.11 | 1.38 | 0.72 | 1.15 | -0.02 | -0.04 | -0.02 | -0.03 | -0.01 | -0.02 |
| $K07495_{VIP≥0.8\ (-)}$ | 1.74 | 2.01 | 1.10 | 0.81 | 0.95 | 0.83 | -0.03 | -0.06 | -0.02 | -0.01 | -0.02 | 0.00 |
| $K21498_{VIP≥0.8\ (-)}$ | 0.45 | 0.82 | 1.07 | 1.52 | 1.28 | 0.70 | -0.01 | -0.02 | -0.02 | -0.03 | -0.02 | -0.02 |
| $K01759_{VIP≥0.8\ (-)}$ | 0.61 | 1.07 | 1.57 | 0.86 | 1.32 | 0.70 | 0.00 | -0.02 | -0.03 | -0.01 | -0.02 | -0.01 |
| $K02996_{VIP≥0.8\ (-)}$ | 0.94 | 0.24 | 1.39 | 1.05 | 1.30 | 0.43 | -0.02 | 0.00 | -0.03 | -0.02 | -0.03 | -0.01 |
| $K13985_{VIP≥0.8\ (-)}$ | 1.24 | 0.82 | 0.58 | 0.80 | 1.01 | 1.02 | -0.03 | 0.00 | -0.01 | -0.01 | -0.02 | -0.02 |
| $K01734_{VIP≥0.8\ (-)}$ | 1.49 | 1.21 | 0.73 | 1.03 | 0.35 | 1.28 | -0.03 | -0.03 | -0.02 | -0.02 | -0.01 | -0.03 |
| $K07450_{VIP≥0.8\ (-)}$ | 1.12 | 2.52 | 1.08 | 0.33 | 1.01 | 0.56 | -0.02 | -0.08 | -0.02 | -0.01 | -0.02 | -0.01 |
| $K02913_{VIP≥0.8\ (-)}$ | 0.60 | 0.82 | 1.46 | 1.58 | 1.42 | 1.27 | 0.00 | 0.02 | -0.03 | -0.03 | -0.03 | -0.02 |
| $K13993_{VIP≥0.8\ (-)}$ | 1.60 | 0.69 | 1.31 | 1.23 | 1.11 | 0.96 | -0.03 | 0.02 | -0.03 | -0.02 | -0.02 | -0.02 |
| $K02916_{VIP≥0.8\ (-)}$ | 0.75 | 0.67 | 1.65 | 1.61 | 1.40 | 0.80 | 0.01 | -0.01 | -0.03 | -0.03 | -0.03 | -0.01 |
| $K08364_{VIP≥0.8\ (-)}$ | 1.46 | 1.01 | 0.80 | 0.76 | 1.20 | 0.75 | -0.03 | 0.00 | -0.02 | -0.01 | -0.02 | 0.00 |
| $K02822_{VIP≥0.8\ (-)}$ | 1.22 | 0.26 | 1.09 | 1.08 | 0.85 | 1.03 | -0.02 | -0.01 | -0.02 | -0.02 | -0.01 | -0.02 |
| $K09976_{VIP≥0.8\ (-)}$ | 0.92 | 1.50 | 0.69 | 1.06 | 1.49 | 0.17 | 0.02 | -0.02 | -0.02 | -0.02 | -0.03 | 0.00 |
| $K04761_{VIP≥0.8\ (-)}$ | 1.39 | 0.81 | 0.81 | 1.37 | 0.27 | 0.89 | -0.03 | 0.01 | -0.02 | -0.03 | 0.00 | -0.02 |
| $K04751_{VIP≥0.8\ (-)}$ | 1.10 | 0.54 | 1.13 | 0.82 | 1.03 | 0.67 | -0.02 | 0.01 | -0.02 | -0.01 | -0.02 | 0.00 |
| $K05832_{VIP≥0.8\ (-)}$ | 1.27 | 0.89 | 1.24 | 0.87 | 0.93 | 0.70 | -0.02 | -0.01 | -0.02 | 0.00 | -0.01 | -0.01 |
| $K06987_{VIP≥0.8\ (-)}$ | 1.35 | 1.32 | 0.89 | 1.51 | 1.03 | 1.00 | -0.03 | -0.04 | -0.02 | -0.03 | -0.02 | -0.02 |
| $K01989_{VIP≥0.8\ (-)}$ | 1.17 | 0.78 | 1.30 | 0.84 | 0.96 | 0.78 | -0.01 | -0.01 | -0.03 | -0.01 | 0.00 | 0.00 |

| | | | | | | | | | | | | |
|---|---|---|---|---|---|---|---|---|---|---|---|---|
| $K18908_{VIP≥0.8\ (-)}$ | 0.89 | 0.78 | 0.95 | 1.27 | 0.74 | 0.98 | 0.00 | -0.02 | 0.00 | -0.03 | -0.01 | -0.02 |
| $K03778_{VIP≥0.8\ (-)}$ | 0.92 | 0.79 | 0.91 | 1.02 | 1.16 | 1.03 | 0.00 | -0.02 | -0.01 | 0.00 | -0.02 | -0.02 |
| $K02056_{VIP≥0.8\ (-)}$ | 0.61 | 1.16 | 1.42 | 0.69 | 1.34 | 1.09 | 0.00 | -0.04 | -0.03 | -0.01 | -0.03 | 0.02 |
| $K01193_{VIP≥0.8\ (-)}$ | 0.79 | 0.86 | 1.32 | 0.88 | 0.87 | 0.39 | 0.01 | -0.03 | -0.03 | -0.02 | -0.02 | 0.00 |
| $K00784_{VIP≥0.8\ (-)}$ | 1.27 | 1.05 | 0.81 | 1.04 | 1.37 | 0.35 | -0.03 | 0.01 | -0.02 | -0.02 | -0.03 | 0.01 |
| $K07484_{VIP≥0.8\ (-)}$ | 1.94 | 0.94 | 0.75 | 0.83 | 0.77 | 0.81 | -0.03 | -0.03 | 0.00 | 0.01 | -0.01 | -0.01 |
| $K02114_{VIP≥0.8\ (-)}$ | 0.74 | 0.57 | 0.89 | 0.85 | 1.22 | 1.05 | 0.01 | -0.01 | -0.01 | 0.00 | -0.02 | -0.02 |
| $K22452_{VIP≥0.8\ (-)}$ | 0.84 | 0.87 | 0.85 | 0.96 | 0.67 | 0.81 | 0.02 | 0.00 | -0.01 | -0.02 | -0.01 | -0.02 |
| $K02759_{VIP≥0.8\ (-)}$ | 1.03 | 0.31 | 1.05 | 1.01 | 0.94 | 0.89 | -0.01 | 0.00 | -0.02 | -0.02 | -0.01 | 0.00 |
| $K08986_{VIP≥0.8\ (-)}$ | 0.89 | 1.00 | 0.98 | 0.84 | 0.92 | 0.66 | 0.01 | -0.01 | -0.01 | -0.01 | -0.01 | -0.01 |
| $K00941_{VIP≥0.8\ (-)}$ | 0.79 | 0.87 | 0.99 | 0.96 | 0.95 | 0.71 | 0.00 | 0.00 | -0.02 | 0.00 | -0.02 | -0.01 |
| $K14155_{VIP≥0.8\ (-)}$ | 1.08 | 0.80 | 0.80 | 0.80 | 1.18 | 0.96 | -0.02 | -0.02 | 0.00 | -0.01 | -0.02 | -0.02 |
| $K00656_{VIP≥0.8\ (-)}$ | 0.87 | 0.66 | 0.92 | 0.91 | 0.98 | 1.47 | 0.01 | -0.01 | 0.00 | 0.00 | -0.02 | -0.03 |
| $K09764_{VIP≥0.8\ (-)}$ | 0.82 | 0.40 | 1.10 | 1.10 | 1.04 | 0.86 | -0.02 | -0.01 | -0.02 | -0.02 | -0.01 | 0.00 |
| $K07118_{VIP≥0.8\ (-)}$ | 1.00 | 0.73 | 0.90 | 0.90 | 1.02 | 1.00 | -0.01 | -0.02 | 0.00 | -0.01 | -0.01 | -0.01 |
| $K02037_{VIP≥0.8\ (-)}$ | 0.91 | 0.46 | 1.00 | 0.92 | 0.80 | 0.95 | 0.00 | -0.01 | -0.01 | -0.01 | -0.01 | -0.01 |
| $K03855_{VIP≥0.8\ (-)}$ | 0.70 | 1.09 | 1.46 | 0.81 | 0.89 | 0.50 | 0.01 | 0.01 | -0.03 | -0.01 | -0.01 | -0.01 |
| $K03741_{VIP≥0.8\ (-)}$ | 0.81 | 0.64 | 0.90 | 0.91 | 0.86 | 0.94 | 0.00 | -0.02 | 0.00 | -0.02 | -0.01 | 0.00 |
| $K02072_{VIP≥0.8\ (-)}$ | 0.94 | 1.23 | 0.94 | 0.89 | 0.81 | 0.67 | 0.02 | -0.03 | -0.01 | -0.01 | -0.01 | -0.01 |
| $K10563_{VIP≥0.8\ (-)}$ | 0.91 | 1.51 | 0.81 | 0.80 | 0.86 | 0.84 | 0.02 | -0.04 | 0.00 | 0.00 | -0.01 | -0.01 |
| $K08602_{VIP≥0.8\ (-)}$ | 0.89 | 0.75 | 0.85 | 0.92 | 0.96 | 0.50 | 0.00 | -0.02 | 0.00 | -0.01 | -0.01 | 0.00 |
| $K09816_{VIP≥0.8\ (-)}$ | 0.43 | 0.85 | 0.85 | 0.89 | 0.93 | 0.50 | 0.00 | -0.01 | -0.01 | -0.02 | 0.01 | -0.01 |
| $K02025_{VIP≥0.8\ (-)}$ | 0.85 | 0.75 | 0.98 | 0.90 | 0.92 | 0.81 | 0.01 | -0.02 | -0.01 | -0.01 | -0.01 | 0.01 |
| $K02036_{VIP≥0.8\ (-)}$ | 0.92 | 0.44 | 1.00 | 0.89 | 0.80 | 0.94 | 0.00 | 0.00 | -0.01 | -0.01 | -0.01 | -0.01 |
| $K23351_{VIP≥0.8\ (-)}$ | 1.03 | 0.74 | 0.88 | 1.07 | 0.99 | 0.68 | 0.02 | -0.02 | -0.01 | -0.02 | -0.02 | 0.01 |
| $K02026_{VIP≥0.8\ (-)}$ | 0.74 | 0.89 | 1.15 | 0.82 | 0.85 | 1.01 | 0.01 | -0.03 | -0.02 | -0.01 | -0.01 | 0.02 |
| $K02032_{VIP≥0.8\ (-)}$ | 0.85 | 0.73 | 1.04 | 0.97 | 0.91 | 0.61 | 0.00 | 0.02 | -0.02 | -0.02 | -0.01 | 0.00 |
| $K08234_{VIP≥0.8\ (-)}$ | 0.39 | 1.74 | 1.16 | 1.08 | 0.73 | 0.95 | 0.01 | 0.04 | -0.02 | -0.02 | -0.01 | -0.01 |
| $K02027_{VIP≥0.8\ (-)}$ | 0.86 | 0.56 | 1.00 | 0.90 | 0.95 | 1.03 | 0.01 | -0.01 | -0.02 | -0.01 | -0.01 | 0.01 |
| $K01613_{VIP≥0.8\ (-)}$ | 0.75 | 1.13 | 0.94 | 1.69 | 0.96 | 0.53 | -0.01 | 0.01 | -0.01 | -0.03 | -0.02 | -0.01 |

| | | | | | | | | | | | |
|---|---|---|---|---|---|---|---|---|---|---|---|---|
| $K21071_{VIP≥0.8\ (-)}$ | 1.04 | 0.92 | 0.99 | 0.87 | 0.83 | 0.48 | 0.02 | -0.01 | -0.01 | -0.01 | 0.00 | 0.00 |
| $K02500_{VIP≥0.8\ (-)}$ | 0.23 | 1.09 | 1.46 | 1.10 | 0.80 | 0.32 | 0.00 | 0.01 | -0.03 | -0.02 | -0.01 | 0.01 |
| $K19118_{VIP≥0.8\ (-)}$ | 0.97 | 0.69 | 0.88 | 0.86 | 0.91 | 1.12 | 0.02 | -0.01 | 0.00 | 0.00 | -0.01 | -0.02 |
| $K00950_{VIP≥0.8\ (-)}$ | 1.27 | 1.30 | 1.13 | 1.06 | 0.72 | 0.37 | -0.03 | -0.01 | -0.02 | -0.02 | -0.01 | 0.00 |
| $K07154_{VIP≥0.8\ (-)}$ | 1.52 | 1.43 | 0.41 | 1.45 | 0.99 | 0.77 | -0.04 | 0.00 | 0.00 | -0.03 | -0.02 | -0.01 |
| $K01258_{VIP≥0.8\ (-)}$ | 1.68 | 0.82 | 0.90 | 1.00 | 0.93 | 0.83 | -0.02 | 0.00 | 0.00 | -0.02 | -0.01 | 0.00 |
| $K02837_{VIP≥0.8\ (-)}$ | 2.33 | 0.96 | 1.05 | 0.79 | 0.73 | 0.94 | -0.05 | -0.01 | 0.01 | 0.00 | -0.01 | -0.01 |
| $K00020_{VIP≥0.8\ (-)}$ | 1.02 | 0.96 | 0.92 | 1.08 | 0.76 | 0.88 | 0.02 | -0.03 | -0.01 | -0.02 | 0.00 | 0.01 |
| $K07741_{VIP≥0.8\ (-)}$ | 1.33 | 1.30 | 0.46 | 0.91 | 0.81 | 0.93 | -0.02 | 0.04 | -0.01 | -0.01 | -0.02 | 0.02 |
| $K19689_{VIP≥0.8\ (-)}$ | 1.14 | 0.51 | 0.97 | 0.86 | 0.99 | 0.83 | 0.02 | -0.01 | -0.01 | -0.01 | -0.01 | 0.01 |
| $K03698_{VIP≥0.8\ (-)}$ | 1.01 | 0.31 | 0.86 | 0.89 | 0.93 | 0.48 | 0.02 | 0.00 | -0.01 | -0.02 | -0.01 | 0.00 |
| $K00854_{VIP≥0.8\ (-)}$ | 0.87 | 1.06 | 0.86 | 0.83 | 1.03 | 1.29 | 0.01 | -0.01 | -0.01 | 0.00 | -0.02 | 0.03 |
| $K06213_{VIP≥0.8\ (-)}$ | 0.90 | 1.50 | 0.85 | 0.69 | 1.01 | 0.59 | 0.00 | 0.02 | -0.01 | 0.00 | 0.01 | -0.01 |
| $K02039_{VIP≥0.8\ (-)}$ | 0.98 | 0.42 | 0.95 | 0.92 | 0.92 | 0.86 | 0.00 | -0.01 | 0.00 | 0.00 | 0.01 | 0.00 |
| $K06204_{VIP≥0.8\ (-)}$ | 0.83 | 0.40 | 1.03 | 1.03 | 1.55 | 0.89 | -0.02 | 0.00 | -0.01 | -0.02 | -0.02 | 0.00 |
| $K17290_{VIP≥0.8\ (-)}$ | 0.86 | 1.24 | 0.69 | 0.47 | 1.28 | 1.52 | -0.02 | 0.00 | 0.00 | 0.00 | -0.02 | 0.04 |
| $K02078_{VIP≥0.8\ (-)}$ | 1.05 | 0.52 | 0.87 | 0.88 | 0.66 | 0.84 | -0.02 | 0.00 | -0.01 | -0.02 | -0.01 | 0.01 |
| $K06867_{VIP≥0.8\ (-)}$ | 0.57 | 0.88 | 0.91 | 1.06 | 0.52 | 0.94 | -0.01 | -0.01 | -0.01 | -0.01 | 0.00 | 0.00 |
| $K06207_{VIP≥0.8\ (-)}$ | 0.94 | 1.01 | 0.79 | 0.80 | 0.67 | 0.98 | -0.01 | -0.01 | 0.00 | -0.01 | -0.01 | -0.01 |
| $K19117_{VIP≥0.8\ (-)}$ | 0.91 | 0.63 | 0.86 | 0.84 | 1.06 | 0.88 | 0.02 | -0.02 | 0.00 | 0.00 | 0.01 | -0.01 |
| $K01752_{VIP≥0.8\ (-)}$ | 0.98 | 0.85 | 0.99 | 0.94 | 0.90 | 1.04 | 0.00 | 0.02 | -0.01 | -0.01 | -0.01 | 0.01 |
| $K01938_{VIP≥0.8\ (-)}$ | 1.08 | 0.40 | 0.87 | 0.92 | 0.84 | 0.73 | 0.02 | 0.00 | -0.01 | 0.00 | 0.00 | 0.00 |
| $K03269_{VIP≥0.8\ (-)}$ | 1.23 | 1.63 | 1.10 | 0.48 | 1.15 | 0.42 | -0.03 | 0.02 | -0.02 | -0.01 | -0.02 | 0.00 |
| $K01955_{VIP≥0.8\ (-)}$ | 1.38 | 0.95 | 0.96 | 0.76 | 0.79 | 0.83 | -0.02 | -0.01 | 0.01 | -0.01 | -0.01 | -0.01 |
| $K03120_{VIP≥0.8\ (-)}$ | 0.83 | 0.39 | 0.88 | 1.03 | 1.03 | 1.24 | 0.00 | -0.01 | 0.00 | 0.00 | -0.01 | 0.02 |
| $K19755_{VIP≥0.8\ (-)}$ | 1.15 | 0.50 | 0.89 | 1.03 | 1.19 | 0.95 | 0.01 | -0.02 | 0.00 | -0.01 | -0.01 | 0.01 |
| $K03626_{VIP≥0.8\ (-)}$ | 0.88 | 0.26 | 0.90 | 1.08 | 1.12 | 1.18 | 0.00 | -0.01 | 0.00 | -0.01 | -0.01 | 0.02 |
| $K02936_{VIP≥0.8\ (-)}$ | 0.83 | 0.53 | 0.87 | 1.08 | 1.08 | 0.73 | -0.01 | -0.02 | 0.00 | -0.01 | -0.01 | 0.01 |
| $K00266_{VIP≥0.8\ (-)}$ | 0.79 | 0.92 | 0.91 | 1.25 | 0.89 | 1.13 | 0.00 | -0.01 | 0.00 | 0.01 | 0.00 | -0.02 |
| $K02889_{VIP≥0.8\ (-)}$ | 0.82 | 0.26 | 0.88 | 1.07 | 1.07 | 1.01 | 0.00 | 0.00 | 0.00 | 0.00 | -0.01 | 0.01 |

| | | | | | | | | | | | | |
|---|---|---|---|---|---|---|---|---|---|---|---|---|
| K07075$_{VIP≥0.8\ (-)}$ | 0.70 | 1.07 | 0.90 | 0.79 | 1.28 | 0.96 | -0.01 | 0.00 | -0.01 | 0.00 | -0.01 | 0.01 |
| K18928$_{VIP≥0.8\ (-)}$ | 1.02 | 1.21 | 0.77 | 0.93 | 0.89 | 0.61 | -0.02 | 0.00 | -0.01 | -0.01 | -0.01 | 0.00 |
| K19166$_{VIP≥0.8\ (-)}$ | 0.50 | 0.41 | 0.90 | 0.88 | 0.86 | 0.80 | -0.01 | 0.01 | -0.01 | -0.01 | -0.01 | 0.00 |
| K02035$_{VIP≥0.8\ (-)}$ | 1.18 | 0.03 | 0.92 | 0.82 | 0.96 | 1.94 | 0.03 | 0.00 | -0.01 | 0.00 | -0.01 | 0.04 |
| K02979$_{VIP≥0.8\ (-)}$ | 0.86 | 0.33 | 0.87 | 1.25 | 0.99 | 1.10 | 0.00 | -0.01 | 0.00 | -0.01 | 0.00 | 0.02 |
| K02529$_{VIP≥0.8\ (-)}$ | 0.85 | 1.39 | 0.85 | 0.97 | 0.27 | 1.25 | -0.01 | -0.01 | -0.02 | -0.02 | 0.00 | 0.03 |
| K01997$_{VIP≥0.8\ (-)}$ | 1.04 | 0.82 | 0.83 | 0.79 | 0.86 | 1.08 | -0.01 | 0.02 | 0.00 | 0.00 | -0.01 | 0.02 |
| K02912$_{VIP≥0.8\ (-)}$ | 0.95 | 0.32 | 0.92 | 1.21 | 1.01 | 0.89 | 0.00 | -0.01 | 0.00 | -0.01 | -0.01 | 0.01 |
| K01823$_{VIP≥0.8\ (-)}$ | 0.98 | 0.59 | 0.62 | 0.83 | 1.05 | 1.08 | -0.02 | -0.02 | 0.00 | -0.01 | -0.01 | 0.02 |
| K02991$_{VIP≥0.8\ (-)}$ | 0.91 | 0.39 | 0.91 | 1.14 | 1.07 | 0.95 | 0.00 | -0.01 | 0.00 | -0.01 | -0.01 | 0.01 |
| K03564$_{VIP≥0.8\ (-)}$ | 1.37 | 0.98 | 0.88 | 0.54 | 0.91 | 0.88 | -0.03 | 0.01 | -0.01 | 0.00 | -0.01 | 0.00 |
| K02987$_{VIP≥0.8\ (-)}$ | 0.84 | 0.38 | 0.86 | 1.22 | 1.22 | 1.05 | 0.00 | -0.01 | 0.00 | -0.01 | -0.01 | 0.02 |
| K01183$_{VIP≥0.8\ (-)}$ | 0.98 | 0.70 | 0.85 | 0.90 | 0.63 | 1.21 | -0.02 | -0.02 | -0.01 | -0.01 | 0.01 | 0.03 |
| K03236$_{VIP≥0.8\ (-)}$ | 0.83 | 0.52 | 0.90 | 1.20 | 0.96 | 1.06 | 0.00 | -0.02 | 0.00 | -0.01 | -0.01 | 0.01 |
| K04047$_{VIP≥0.8}$ | 1.65 | 1.09 | 0.93 | 0.89 | 1.20 | 0.88 | -0.03 | -0.03 | 0.00 | 0.00 | 0.01 | 0.00 |
| K03555$_{VIP≥0.8}$ | 0.89 | 1.10 | 1.41 | 1.11 | 0.84 | 1.11 | -0.01 | -0.01 | 0.03 | 0.01 | -0.01 | 0.01 |
| K04798$_{VIP≥0.8}$ | 0.94 | 0.43 | 0.85 | 0.93 | 1.07 | 1.17 | 0.00 | -0.01 | 0.00 | 0.00 | -0.01 | 0.02 |
| K03264$_{VIP≥0.8}$ | 1.18 | 0.48 | 0.84 | 0.97 | 1.00 | 1.11 | 0.01 | -0.02 | 0.00 | 0.00 | -0.01 | 0.02 |
| K09131$_{VIP≥0.8}$ | 1.05 | 1.17 | 0.83 | 0.77 | 1.15 | 1.00 | 0.02 | 0.02 | -0.01 | 0.00 | -0.01 | 0.02 |
| K02921$_{VIP≥0.8}$ | 0.98 | 0.35 | 0.91 | 1.11 | 1.03 | 0.98 | 0.00 | -0.01 | 0.00 | -0.01 | -0.01 | 0.01 |
| K02875$_{VIP≥0.8}$ | 0.98 | 0.17 | 0.85 | 1.14 | 1.05 | 1.03 | 0.00 | 0.00 | 0.01 | -0.01 | -0.01 | 0.01 |
| K10117$_{VIP≥0.8}$ | 0.89 | 0.42 | 0.95 | 0.94 | 1.43 | 1.22 | 0.00 | -0.01 | 0.00 | 0.00 | 0.02 | 0.02 |
| K07335$_{VIP≥0.8}$ | 0.89 | 0.49 | 0.86 | 0.88 | 1.04 | 0.84 | 0.01 | 0.00 | -0.01 | -0.01 | -0.02 | 0.01 |
| K00265$_{VIP≥0.8}$ | 0.89 | 0.68 | 1.01 | 0.97 | 0.82 | 0.92 | 0.00 | -0.02 | 0.01 | 0.01 | 0.00 | -0.01 |
| K02112$_{VIP≥0.8}$ | 0.64 | 0.94 | 1.21 | 1.09 | 0.82 | 0.92 | 0.00 | -0.01 | 0.02 | 0.01 | -0.01 | 0.00 |
| K07720$_{VIP≥0.8}$ | 1.44 | 0.79 | 0.88 | 0.86 | 0.85 | 1.02 | -0.02 | 0.02 | 0.01 | 0.00 | -0.01 | 0.01 |
| K03798$_{VIP≥0.8}$ | 1.18 | 0.44 | 1.04 | 0.94 | 0.91 | 1.09 | -0.01 | 0.00 | 0.01 | 0.00 | 0.00 | 0.01 |
| K02355$_{VIP≥0.8}$ | 0.84 | 1.02 | 0.98 | 1.04 | 0.69 | 0.99 | 0.00 | -0.01 | 0.01 | 0.01 | 0.00 | 0.00 |
| K07573$_{VIP≥0.8}$ | 0.73 | 0.20 | 0.84 | 0.84 | 1.17 | 1.05 | 0.00 | -0.01 | 0.00 | 0.01 | -0.01 | 0.01 |
| K07172$_{VIP≥0.8}$ | 1.26 | 1.32 | 0.91 | 0.82 | 0.65 | 0.31 | 0.03 | 0.03 | -0.02 | -0.02 | 0.00 | 0.00 |

| | | | | | | | | | | | | |
|---|---|---|---|---|---|---|---|---|---|---|---|---|
| $K07402_{VIP\geq0.8}$ | 0.82 | 0.84 | 0.84 | 0.78 | 0.79 | 1.05 | 0.01 | 0.01 | 0.00 | 0.00 | 0.00 | 0.02 |
| $K03724_{VIP\geq0.8}$ | 1.52 | 0.56 | 0.86 | 0.82 | 0.82 | 0.61 | -0.02 | -0.01 | 0.00 | 0.00 | -0.01 | 0.00 |
| $K06972_{VIP\geq0.8}$ | 0.93 | 0.63 | 0.93 | 1.05 | 0.96 | 0.78 | 0.01 | -0.02 | 0.00 | 0.00 | -0.01 | 0.00 |
| $K02086_{VIP\geq0.8}$ | 0.85 | 1.01 | 0.94 | 0.74 | 0.96 | 0.58 | 0.01 | 0.01 | -0.01 | -0.01 | 0.01 | 0.00 |
| $K02031_{VIP\geq0.8}$ | 0.84 | 0.48 | 1.14 | 1.15 | 0.96 | 0.73 | 0.01 | 0.01 | -0.02 | -0.02 | -0.01 | 0.01 |
| $K23536_{VIP\geq0.8}$ | 0.82 | 0.72 | 0.86 | 0.93 | 1.03 | 0.67 | 0.01 | 0.01 | -0.01 | -0.02 | -0.02 | 0.01 |
| $K05833_{VIP\geq0.8}$ | 0.88 | 0.66 | 0.87 | 0.98 | 0.94 | 0.63 | 0.00 | -0.01 | -0.01 | 0.00 | 0.00 | 0.00 |
| $K01139_{VIP\geq0.8}$ | 0.74 | 0.08 | 0.86 | 0.85 | 1.06 | 1.19 | 0.01 | 0.00 | -0.01 | 0.00 | 0.01 | -0.02 |
| $K02034_{VIP\geq0.8}$ | 0.80 | 0.42 | 1.00 | 0.93 | 1.12 | 1.50 | 0.01 | 0.00 | -0.02 | -0.02 | -0.02 | 0.03 |
| $K00864_{VIP\geq0.8}$ | 0.89 | 0.86 | 0.77 | 0.89 | 0.73 | 0.80 | 0.00 | 0.01 | -0.01 | 0.00 | 0.00 | 0.01 |
| $K05801_{VIP\geq0.8}$ | 0.97 | 1.31 | 0.72 | 0.28 | 1.12 | 1.18 | -0.02 | 0.01 | -0.01 | 0.00 | -0.02 | 0.03 |
| $K06959_{VIP\geq0.8}$ | 0.96 | 1.59 | 0.44 | 0.70 | 1.02 | 0.96 | -0.01 | 0.02 | 0.00 | -0.01 | 0.02 | 0.02 |
| $K03601_{VIP\geq0.8}$ | 0.38 | 0.70 | 0.91 | 0.87 | 0.93 | 0.97 | 0.00 | 0.01 | 0.01 | -0.01 | -0.02 | 0.01 |
| $K03574_{VIP\geq0.8}$ | 0.74 | 1.21 | 0.81 | 1.28 | 1.47 | 0.75 | -0.02 | 0.01 | 0.00 | -0.02 | -0.03 | 0.00 |
| K14122 | 0.79 | 0.45 | 1.18 | 0.65 | 1.01 | 0.96 | 0.01 | 0.00 | 0.02 | 0.01 | -0.01 | 0.00 |
| K14111 | 0.88 | 0.34 | 0.64 | 0.77 | 1.26 | 1.39 | 0.01 | 0.00 | 0.00 | 0.00 | -0.02 | 0.03 |
| K07492 | 0.77 | 0.77 | 1.05 | 0.71 | 1.14 | 1.45 | -0.01 | -0.01 | -0.01 | 0.01 | -0.02 | -0.02 |
| K01500 | 0.76 | 0.55 | 0.83 | 1.32 | 1.35 | 0.52 | 0.01 | 0.01 | -0.01 | -0.03 | -0.03 | 0.00 |
| K07148 | 0.67 | 0.83 | 0.69 | 0.98 | 0.91 | 0.66 | 0.00 | 0.01 | 0.00 | -0.02 | 0.00 | 0.00 |
| K00878 | 0.85 | 0.25 | 1.05 | 1.06 | 0.76 | 0.16 | 0.02 | 0.00 | 0.01 | 0.01 | 0.00 | 0.00 |
| K08999 | 1.02 | 0.67 | 0.97 | 0.79 | 0.96 | 0.36 | 0.02 | 0.00 | -0.01 | -0.01 | -0.01 | 0.00 |
| K01267 | 0.72 | 0.97 | 1.37 | 0.86 | 0.78 | 0.61 | 0.01 | -0.01 | -0.03 | -0.01 | -0.01 | -0.01 |
| K16787 | 0.77 | 0.44 | 0.91 | 0.83 | 0.91 | 0.74 | 0.01 | 0.00 | -0.01 | -0.01 | 0.00 | 0.01 |
| K06998 | 1.35 | 1.23 | 1.39 | 0.53 | 0.74 | 0.75 | 0.03 | 0.03 | -0.03 | -0.01 | -0.01 | -0.02 |
| K23535 | 0.80 | 0.57 | 0.90 | 0.87 | 0.96 | 0.78 | 0.01 | 0.02 | -0.01 | -0.01 | -0.01 | 0.01 |
| K19271 | 0.77 | 0.54 | 0.89 | 0.57 | 1.08 | 1.04 | -0.01 | 0.00 | 0.00 | 0.00 | -0.01 | 0.00 |
| K00003 | 1.56 | 0.80 | 0.74 | 0.75 | 1.23 | 1.09 | 0.04 | 0.02 | 0.00 | 0.01 | 0.02 | 0.02 |
| K00991 | 0.76 | 1.33 | 0.97 | 0.83 | 0.78 | 0.67 | 0.00 | 0.04 | -0.01 | 0.00 | 0.00 | -0.01 |
| K02233 | 1.00 | 1.70 | 0.70 | 0.63 | 0.74 | 1.46 | 0.02 | 0.04 | 0.01 | -0.01 | 0.01 | 0.03 |
| K05919 | 1.06 | 1.16 | 1.06 | 0.79 | 0.68 | 0.28 | 0.02 | 0.00 | -0.02 | 0.00 | -0.01 | 0.00 |
| K03803 | 0.73 | 1.58 | 0.73 | 1.76 | 1.52 | 0.78 | 0.00 | 0.03 | 0.00 | -0.03 | -0.03 | -0.01 |
| K07058 | 0.43 | 0.84 | 0.91 | 1.26 | 0.36 | 0.70 | -0.01 | 0.00 | -0.01 | -0.02 | 0.00 | 0.00 |
| K01696 | 0.87 | 0.89 | 0.90 | 0.71 | 0.54 | 0.74 | -0.02 | 0.01 | -0.01 | 0.00 | -0.01 | 0.00 |

| | | | | | | | | | | | | |
|---|---|---|---|---|---|---|---|---|---|---|---|---|
| K01649 | 0.61 | 1.28 | 1.50 | 0.74 | 0.70 | 0.85 | 0.00 | 0.01 | 0.03 | 0.00 | 0.00 | 0.01 |
| K01297 | 0.68 | 0.94 | 0.81 | 0.51 | 0.84 | 0.78 | -0.01 | 0.01 | 0.00 | 0.00 | 0.00 | 0.00 |
| K02358 | 0.73 | 0.39 | 0.95 | 0.96 | 0.79 | 0.94 | 0.01 | 0.01 | 0.01 | 0.00 | -0.01 | 0.00 |
| K00817 | 0.93 | 0.79 | 0.80 | 0.83 | 0.79 | 1.19 | -0.01 | 0.01 | -0.01 | 0.00 | 0.00 | 0.02 |
| K03596 | 0.71 | 0.88 | 0.93 | 0.80 | 0.78 | 0.97 | 0.00 | 0.02 | 0.01 | -0.01 | -0.01 | -0.01 |
| K00651 | 0.65 | 0.48 | 1.02 | 0.84 | 0.88 | 0.79 | 0.00 | -0.01 | -0.02 | -0.01 | -0.01 | -0.01 |
| K00820 | 0.48 | 1.57 | 1.10 | 0.79 | 0.84 | 0.71 | 0.00 | 0.04 | 0.02 | -0.01 | -0.01 | 0.00 |
| K03685 | 1.01 | 0.77 | 0.71 | 0.76 | 1.00 | 0.94 | -0.01 | -0.01 | -0.01 | 0.00 | -0.01 | 0.02 |
| K02836 | 0.59 | 0.75 | 0.75 | 0.84 | 1.09 | 0.92 | 0.00 | -0.02 | 0.00 | 0.00 | 0.01 | 0.01 |
| K07478 | 0.61 | 0.50 | 0.78 | 0.85 | 0.85 | 0.97 | 0.01 | 0.01 | 0.00 | -0.01 | -0.01 | 0.00 |
| K02838 | 0.46 | 1.09 | 0.79 | 1.17 | 0.71 | 0.95 | 0.00 | -0.03 | 0.00 | -0.02 | -0.01 | 0.02 |
| K02518 | 0.65 | 0.78 | 1.58 | 1.33 | 1.18 | 0.74 | -0.01 | 0.01 | -0.03 | -0.02 | -0.02 | 0.00 |
| K01091 | 0.91 | 0.44 | 0.95 | 0.83 | 0.73 | 0.71 | 0.02 | 0.00 | -0.02 | -0.01 | -0.01 | 0.00 |
| K06875 | 0.73 | 0.54 | 0.76 | 0.76 | 1.05 | 1.20 | 0.00 | 0.02 | 0.00 | 0.01 | -0.01 | 0.02 |
| K07105 | 0.76 | 0.74 | 0.93 | 0.76 | 0.75 | 0.69 | 0.01 | 0.00 | -0.01 | -0.01 | 0.00 | 0.00 |

Partial Least Squares analysis was performed with 2 latent components. Subscript VIP≥0.8 denotes microbial genera that had VIP ≥ 0.8 in 4 or more timepoints. Subscripts (+) and (-) represent microbial genera that had positive or negative regression coefficient in 4 or more timepoints, respectively.

**Trait: Daily Methane Production (g/day, CH4P); Predictors: 204 microbial genes (MG)**

| Microbial genes | VIP | | | | | | Regression coefficient | | | | | |
|---|---|---|---|---|---|---|---|---|---|---|---|---|
| | T1 | T2 | T3 | T4 | T5 | T6 | T1 | T2 | T3 | T4 | T5 | T6 |
| $K15635_{VIP≥0.8\ (+)}$ | 0.61 | 1.59 | 1.87 | 1.56 | 0.83 | 2.49 | 0.02 | 0.02 | 0.03 | 0.03 | 0.02 | 0.03 |
| $K00441_{VIP≥0.8\ (+)}$ | 0.03 | 1.08 | 1.39 | 1.77 | 0.58 | 1.88 | 0.00 | 0.02 | 0.02 | 0.03 | 0.00 | 0.03 |
| $K00343_{VIP≥0.8\ (+)}$ | 0.59 | 1.27 | 1.55 | 1.46 | 0.28 | 2.20 | -0.02 | 0.01 | 0.03 | 0.03 | 0.00 | 0.03 |
| $K21908_{VIP≥0.8\ (+)}$ | 0.94 | 0.64 | 0.87 | 1.58 | 1.48 | 1.09 | 0.03 | 0.01 | 0.01 | 0.02 | 0.04 | 0.01 |
| $K05305_{VIP≥0.8\ (+)}$ | 0.55 | 1.03 | 1.64 | 0.76 | 1.50 | 1.91 | 0.01 | 0.01 | 0.03 | 0.01 | 0.04 | 0.03 |
| $K00046_{VIP≥0.8\ (+)}$ | 0.66 | 1.05 | 0.87 | 1.19 | 1.30 | 1.96 | 0.01 | 0.01 | 0.01 | 0.02 | 0.03 | 0.03 |
| $K10725_{VIP≥0.8\ (+)}$ | 1.46 | 1.54 | 1.62 | 0.79 | 0.80 | 1.35 | 0.03 | 0.03 | 0.02 | 0.01 | -0.01 | 0.02 |
| $K00609_{VIP≥0.8\ (+)}$ | 0.69 | 1.53 | 1.48 | 1.37 | 0.65 | 1.36 | 0.00 | 0.03 | 0.02 | 0.02 | -0.01 | 0.02 |
| $K08094_{VIP≥0.8\ (+)}$ | 0.50 | 1.07 | 1.26 | 1.22 | 0.76 | 1.88 | -0.01 | 0.02 | 0.02 | 0.02 | 0.02 | 0.03 |
| $K07503_{VIP≥0.8\ (+)}$ | 0.80 | 0.66 | 1.42 | 0.89 | 0.83 | 1.20 | 0.02 | 0.01 | 0.02 | 0.01 | 0.01 | 0.02 |

| | | | | | | | | | | | | |
|---|---|---|---|---|---|---|---|---|---|---|---|---|
| $K02233_{VIP≥0.8\ (+)}$ | 1.22 | 1.03 | 1.34 | 0.61 | 1.15 | 1.01 | 0.03 | 0.02 | 0.02 | 0.00 | 0.02 | 0.01 |
| $K02529_{VIP≥0.8\ (+)}$ | 0.82 | 1.32 | 0.48 | 1.00 | 1.37 | 1.58 | -0.01 | 0.01 | 0.01 | 0.02 | 0.04 | 0.02 |
| $K13002_{VIP≥0.8\ (+)}$ | 0.68 | 1.56 | 1.44 | 1.29 | 0.37 | 1.35 | -0.02 | 0.03 | 0.02 | 0.02 | 0.00 | 0.02 |
| $K07316_{VIP≥0.8\ (+)}$ | 1.01 | 1.01 | 1.04 | 0.76 | 1.07 | 1.70 | 0.03 | 0.01 | 0.02 | 0.01 | 0.02 | 0.02 |
| $K21571_{VIP≥0.8\ (+)}$ | 0.38 | 1.34 | 0.98 | 1.36 | 0.35 | 1.41 | 0.01 | 0.02 | 0.02 | 0.02 | -0.01 | 0.02 |
| $K04062_{VIP≥0.8\ (+)}$ | 1.01 | 1.96 | 1.59 | 0.91 | 0.52 | 0.97 | 0.03 | 0.04 | 0.03 | 0.02 | 0.00 | -0.02 |
| $K00757_{VIP≥0.8\ (+)}$ | 0.37 | 1.07 | 1.20 | 1.32 | 0.65 | 1.96 | -0.01 | 0.01 | 0.02 | 0.02 | 0.01 | 0.03 |
| $K01686_{VIP≥0.8\ (+)}$ | 0.63 | 1.08 | 0.80 | 1.12 | 0.56 | 1.93 | 0.00 | 0.01 | 0.01 | 0.02 | 0.01 | 0.03 |
| $K00286_{VIP≥0.8\ (+)}$ | 0.68 | 1.05 | 0.63 | 1.14 | 0.80 | 1.89 | 0.00 | 0.01 | 0.01 | 0.02 | 0.02 | 0.02 |
| $K00259_{VIP≥0.8\ (+)}$ | 0.51 | 1.11 | 0.89 | 1.63 | 0.64 | 1.40 | -0.01 | 0.01 | 0.01 | 0.03 | 0.01 | 0.02 |
| $K14112_{VIP≥0.8\ (+)}$ | 1.00 | 1.04 | 1.59 | 1.10 | 0.83 | 0.97 | 0.02 | 0.02 | 0.02 | 0.01 | -0.01 | 0.01 |
| $K14102_{VIP≥0.8\ (+)}$ | 1.25 | 1.04 | 1.70 | 0.95 | 0.89 | 0.78 | 0.02 | 0.02 | 0.02 | 0.01 | -0.01 | 0.01 |
| $K00125_{VIP≥0.8\ (+)}$ | 1.07 | 0.98 | 1.54 | 1.43 | 0.77 | 0.94 | -0.01 | 0.00 | 0.03 | 0.02 | 0.02 | 0.01 |
| $K03181_{VIP≥0.8\ (+)}$ | 1.03 | 1.13 | 1.48 | 0.92 | 0.88 | 1.25 | 0.01 | 0.02 | 0.02 | 0.01 | -0.01 | 0.02 |
| $K12340_{VIP≥0.8\ (+)}$ | 0.65 | 0.83 | 1.14 | 1.43 | 0.71 | 1.62 | -0.02 | 0.01 | 0.01 | 0.02 | 0.00 | 0.02 |
| $K03426_{VIP≥0.8\ (+)}$ | 0.82 | 1.44 | 1.07 | 0.58 | 1.07 | 1.89 | 0.00 | 0.02 | 0.02 | 0.01 | -0.03 | 0.02 |
| $K07158_{VIP≥0.8\ (+)}$ | 1.00 | 0.92 | 1.29 | 0.87 | 0.87 | 1.78 | 0.02 | 0.02 | 0.02 | 0.01 | -0.02 | 0.03 |
| $K03394_{VIP≥0.8\ (+)}$ | 0.99 | 2.25 | 1.50 | 0.42 | 1.17 | 0.77 | 0.03 | 0.04 | 0.02 | 0.00 | -0.02 | 0.01 |
| $K14122_{VIP≥0.8\ (+)}$ | 1.00 | 0.94 | 1.65 | 0.94 | 0.75 | 0.83 | 0.01 | 0.02 | 0.02 | 0.01 | -0.01 | 0.01 |
| $K09739_{VIP≥0.8\ (+)}$ | 0.73 | 1.08 | 1.40 | 1.17 | 0.77 | 1.05 | 0.00 | 0.02 | 0.02 | 0.02 | -0.01 | 0.02 |
| $K07732_{VIP≥0.8\ (+)}$ | 0.86 | 0.96 | 1.45 | 0.68 | 0.75 | 1.44 | 0.01 | 0.02 | 0.02 | 0.01 | -0.01 | 0.02 |
| $K03057_{VIP≥0.8\ (+)}$ | 1.11 | 1.23 | 1.54 | 0.90 | 0.96 | 0.89 | 0.02 | 0.02 | 0.02 | 0.01 | -0.02 | 0.01 |
| $K07063_{VIP≥0.8\ (+)}$ | 0.97 | 0.57 | 1.16 | 1.38 | 0.47 | 0.94 | 0.02 | 0.01 | 0.02 | 0.02 | -0.01 | 0.02 |
| $K04796_{VIP≥0.8\ (+)}$ | 1.00 | 1.14 | 1.48 | 0.70 | 0.94 | 1.45 | 0.01 | 0.02 | 0.02 | 0.00 | -0.02 | 0.02 |
| $K01079_{VIP≥0.8\ (+)}$ | 0.70 | 1.07 | 0.80 | 0.61 | 0.82 | 1.05 | 0.01 | 0.02 | 0.01 | 0.01 | 0.02 | 0.01 |
| $K21575_{VIP≥0.8\ (+)}$ | 0.60 | 0.81 | 1.19 | 1.07 | 0.61 | 1.78 | -0.01 | 0.01 | 0.02 | 0.01 | -0.01 | 0.02 |
| $K03638_{VIP≥0.8\ (+)}$ | 0.89 | 1.37 | 1.40 | 0.71 | 0.80 | 1.09 | 0.01 | 0.03 | 0.02 | 0.01 | -0.02 | 0.01 |
| $K06885_{VIP≥0.8\ (+)}$ | 1.19 | 0.52 | 1.21 | 1.61 | 0.86 | 1.43 | -0.03 | 0.01 | 0.01 | 0.02 | 0.01 | 0.02 |
| $K14118_{VIP≥0.8\ (+)}$ | 0.88 | 0.92 | 1.65 | 0.86 | 1.14 | 0.99 | 0.01 | 0.02 | 0.02 | 0.01 | -0.02 | 0.02 |
| $K01823_{VIP≥0.8\ (+)}$ | 1.59 | 0.77 | 1.21 | 1.22 | 0.73 | 1.93 | -0.04 | 0.01 | 0.02 | 0.02 | 0.00 | 0.03 |

| | | | | | | | | | | | | |
|---|---|---|---|---|---|---|---|---|---|---|---|---|
| $K14111_{VIP≥0.8\ (+)}$ | 0.90 | 0.90 | 1.28 | 0.90 | 0.94 | 1.18 | 0.01 | 0.02 | 0.02 | 0.01 | -0.02 | 0.02 |
| $K06959_{VIP≥0.8\ (+)}$ | 1.23 | 1.22 | 0.80 | 0.73 | 0.54 | 1.13 | -0.02 | 0.00 | 0.01 | 0.01 | 0.01 | 0.01 |
| $K02775_{VIP≥0.8\ (+)}$ | 0.88 | 1.23 | 0.64 | 0.82 | 0.18 | 1.48 | -0.02 | 0.01 | 0.01 | 0.01 | 0.00 | 0.02 |
| $K01756_{VIP≥0.8\ (+)}$ | 1.17 | 1.44 | 0.90 | 0.74 | 1.11 | 1.14 | -0.01 | 0.02 | 0.01 | 0.01 | 0.03 | 0.01 |
| $K00558_{VIP≥0.8\ (+)}$ | 0.87 | 1.29 | 1.26 | 0.55 | 0.75 | 1.09 | 0.00 | 0.01 | 0.02 | -0.01 | -0.01 | 0.01 |
| $K19166_{VIP≥0.8\ (+)}$ | 0.89 | 1.17 | 0.76 | 0.91 | 1.12 | 0.44 | 0.01 | 0.02 | 0.00 | 0.01 | 0.03 | 0.01 |
| $K07720_{VIP≥0.8\ (+)}$ | 1.30 | 0.71 | 0.96 | 1.01 | 0.76 | 1.20 | -0.01 | 0.01 | 0.01 | 0.02 | 0.01 | 0.02 |
| $K00817_{VIP≥0.8\ (+)}$ | 0.98 | 1.04 | 0.73 | 1.00 | 0.62 | 0.94 | 0.02 | 0.01 | 0.00 | 0.01 | 0.01 | 0.01 |
| $K01915_{VIP≥0.8\ (+)}$ | 0.79 | 1.33 | 1.06 | 0.96 | 0.88 | 1.01 | 0.01 | 0.00 | 0.02 | 0.01 | -0.01 | 0.01 |
| $K03723_{VIP≥0.8\ (+)}$ | 1.07 | 1.30 | 0.71 | 1.04 | 0.83 | 0.72 | 0.02 | -0.01 | 0.01 | 0.01 | 0.01 | 0.01 |
| $K10117_{VIP≥0.8\ (+)}$ | 1.00 | 0.76 | 0.80 | 0.68 | 1.26 | 1.23 | 0.00 | 0.01 | 0.00 | 0.01 | 0.03 | 0.02 |
| $K03183_{VIP≥0.8\ (+)}$ | 0.80 | 0.76 | 1.04 | 1.26 | 1.12 | 1.32 | -0.02 | 0.01 | 0.01 | 0.02 | -0.03 | 0.02 |
| $K03702_{VIP≥0.8\ (+)}$ | 0.98 | 1.31 | 0.66 | 0.82 | 0.97 | 0.48 | 0.02 | 0.01 | 0.01 | 0.01 | -0.01 | 0.00 |
| $K12373_{VIP≥0.8\ (+)}$ | 0.86 | 1.33 | 0.88 | 0.43 | 0.91 | 0.85 | -0.02 | 0.03 | 0.01 | 0.00 | 0.02 | 0.01 |
| $K06413_{VIP≥0.8\ (+)}$ | 0.81 | 1.09 | 1.51 | 0.87 | 0.92 | 0.89 | -0.02 | -0.02 | 0.02 | 0.01 | 0.01 | 0.01 |
| $K24131_{VIP≥0.8\ (+)}$ | 1.71 | 1.11 | 0.89 | 0.66 | 0.85 | 0.45 | 0.05 | 0.02 | 0.01 | 0.00 | -0.01 | -0.01 |
| $K01733_{VIP≥0.8\ (+)}$ | 0.95 | 1.25 | 0.89 | 0.74 | 0.98 | 0.90 | 0.02 | 0.02 | 0.01 | 0.00 | -0.01 | 0.01 |
| $K03327_{VIP≥0.8\ (+)}$ | 1.10 | 0.91 | 0.71 | 0.99 | 0.86 | 0.83 | -0.01 | 0.01 | 0.00 | 0.01 | 0.01 | 0.01 |
| $K03540_{VIP≥0.8\ (+)}$ | 0.73 | 1.30 | 0.98 | 0.82 | 0.80 | 1.18 | 0.00 | 0.03 | 0.00 | 0.00 | -0.01 | 0.02 |
| $K03564_{VIP≥0.8\ (+)}$ | 1.54 | 0.75 | 1.07 | 1.32 | 0.57 | 0.84 | -0.04 | 0.01 | 0.01 | 0.02 | 0.00 | 0.01 |
| $K01465_{VIP≥0.8\ (+)}$ | 1.09 | 1.16 | 0.57 | 1.09 | 1.04 | 1.34 | -0.01 | 0.01 | 0.00 | 0.02 | -0.02 | 0.01 |
| $K02337_{VIP≥0.8\ (+)}$ | 0.95 | 1.36 | 0.74 | 0.96 | 0.80 | 0.89 | 0.01 | -0.01 | 0.01 | 0.01 | -0.01 | 0.01 |
| $K07573_{VIP≥0.8\ (+)}$ | 0.83 | 0.62 | 1.16 | 0.90 | 0.96 | 0.76 | 0.00 | 0.01 | 0.01 | 0.01 | -0.02 | 0.01 |
| $K00864_{VIP≥0.8\ (+)}$ | 0.98 | 1.15 | 0.65 | 0.73 | 1.19 | 0.95 | -0.01 | -0.01 | 0.00 | 0.01 | 0.03 | 0.01 |
| $K00766_{VIP≥0.8\ (+)}$ | 1.07 | 1.04 | 0.56 | 1.03 | 0.30 | 1.25 | -0.02 | 0.01 | 0.00 | 0.01 | -0.01 | 0.01 |
| $K01952_{VIP≥0.8\ (+)}$ | 0.80 | 1.00 | 0.95 | 0.83 | 0.62 | 0.45 | 0.01 | 0.00 | 0.01 | 0.01 | -0.01 | 0.01 |
| $K01657_{VIP≥0.8\ (+)}$ | 0.86 | 1.29 | 0.58 | 0.65 | 1.10 | 1.32 | 0.02 | 0.02 | 0.00 | 0.00 | -0.03 | 0.01 |
| $K11260_{VIP≥0.8\ (+)}$ | 0.96 | 0.86 | 0.84 | 0.92 | 0.83 | 0.32 | 0.01 | 0.02 | 0.00 | 0.01 | -0.01 | 0.00 |
| $K01940_{VIP≥0.8\ (+)}$ | 0.91 | 0.94 | 0.59 | 0.82 | 0.96 | 0.97 | 0.02 | 0.00 | 0.00 | 0.01 | -0.02 | 0.01 |
| $K03105_{VIP≥0.8\ (+)}$ | 0.92 | 0.63 | 1.00 | 0.84 | 0.75 | 1.03 | -0.01 | 0.01 | 0.01 | 0.00 | 0.00 | 0.02 |

| | | | | | | | | | | | | |
|---|---|---|---|---|---|---|---|---|---|---|---|---|
| $K06875_{VIP≥0.8\ (+)}$ | 0.95 | 0.64 | 1.03 | 0.78 | 1.00 | 0.85 | -0.02 | 0.01 | 0.01 | 0.00 | -0.02 | 0.01 |
| $K02313_{VIP≥0.8\ (+)}$ | 0.72 | 0.94 | 0.90 | 0.91 | 1.15 | 0.25 | 0.01 | 0.01 | 0.01 | 0.00 | -0.02 | 0.00 |
| $K03555_{VIP≥0.8\ (+)}$ | 0.86 | 1.20 | 0.80 | 0.82 | 1.01 | 0.30 | 0.02 | -0.01 | 0.01 | 0.01 | -0.01 | 0.00 |
| $K01955_{VIP≥0.8\ (+)}$ | 1.00 | 0.83 | 0.80 | 0.73 | 0.84 | 0.56 | 0.00 | 0.00 | 0.01 | 0.00 | -0.01 | 0.00 |
| $K14155_{VIP≥0.8\ (+)}$ | 1.44 | 0.83 | 0.72 | 0.83 | 0.87 | 0.55 | -0.03 | 0.01 | 0.01 | 0.01 | -0.02 | 0.00 |
| $K01258_{VIP≥0.8\ (+)}$ | 1.02 | 0.95 | 0.85 | 0.66 | 0.94 | 0.62 | 0.00 | -0.01 | 0.01 | 0.00 | -0.01 | 0.01 |
| $K03798_{VIP≥0.8\ (+)}$ | 1.22 | 0.94 | 0.82 | 0.81 | 0.80 | 0.47 | -0.01 | -0.01 | 0.00 | 0.00 | 0.00 | 0.01 |
| $K07464_{VIP≥0.8\ (+)}$ | 1.14 | 0.48 | 0.81 | 0.88 | 1.01 | 0.29 | -0.01 | 0.01 | 0.00 | 0.00 | 0.02 | 0.00 |
| $K19304_{VIP≥0.8\ (-)}$ | 0.44 | 1.04 | 0.83 | 1.70 | 1.86 | 0.98 | -0.01 | -0.01 | -0.01 | -0.03 | -0.05 | -0.01 |
| $K13985_{VIP≥0.8\ (-)}$ | 1.68 | 0.72 | 0.52 | 1.79 | 1.59 | 1.14 | -0.04 | -0.01 | -0.01 | -0.03 | -0.03 | -0.02 |
| $K05595_{VIP≥0.8\ (-)}$ | 1.33 | 1.18 | 1.40 | 1.55 | 1.04 | 1.28 | 0.02 | -0.01 | -0.02 | -0.02 | -0.02 | -0.02 |
| $K02884_{VIP≥0.8\ (-)}$ | 0.94 | 0.37 | 1.29 | 1.01 | 1.77 | 0.71 | -0.03 | 0.00 | -0.02 | -0.01 | -0.04 | -0.01 |
| $K05337_{VIP≥0.8\ (-)}$ | 0.43 | 1.09 | 1.19 | 0.88 | 1.74 | 1.62 | 0.00 | 0.01 | -0.02 | -0.01 | -0.04 | -0.02 |
| $K11105_{VIP≥0.8\ (-)}$ | 0.99 | 1.01 | 0.78 | 1.43 | 0.86 | 0.62 | 0.02 | -0.02 | -0.01 | -0.02 | -0.01 | -0.01 |
| $K00573_{VIP≥0.8\ (-)}$ | 0.96 | 1.00 | 1.09 | 1.16 | 0.64 | 1.25 | 0.01 | 0.00 | -0.02 | -0.02 | -0.02 | -0.02 |
| $K06416_{VIP≥0.8\ (-)}$ | 0.15 | 0.97 | 1.25 | 0.91 | 1.32 | 0.65 | 0.00 | 0.01 | -0.02 | -0.01 | -0.03 | -0.01 |
| $K09780_{VIP≥0.8\ (-)}$ | 2.39 | 0.63 | 1.80 | 1.42 | 0.70 | 0.92 | 0.06 | -0.01 | -0.03 | -0.02 | -0.01 | -0.01 |
| $K03820_{VIP≥0.8\ (-)}$ | 0.62 | 1.08 | 1.21 | 1.01 | 1.04 | 0.82 | 0.02 | -0.02 | -0.02 | -0.01 | -0.01 | -0.01 |
| $K02913_{VIP≥0.8\ (-)}$ | 0.72 | 0.64 | 0.99 | 0.80 | 1.60 | 1.04 | 0.00 | 0.01 | -0.01 | -0.01 | -0.04 | -0.02 |
| $K17290_{VIP≥0.8\ (-)}$ | 1.33 | 0.99 | 0.64 | 0.83 | 1.84 | 0.66 | -0.04 | 0.00 | 0.00 | -0.01 | -0.05 | 0.00 |
| $K06998_{VIP≥0.8\ (-)}$ | 1.51 | 0.63 | 1.48 | 1.56 | 1.17 | 1.47 | 0.04 | 0.01 | -0.02 | -0.02 | -0.02 | -0.02 |
| $K00940_{VIP≥0.8\ (-)}$ | 0.88 | 1.25 | 0.64 | 1.53 | 1.42 | 0.32 | 0.01 | -0.03 | 0.00 | -0.03 | -0.04 | 0.00 |
| $K21498_{VIP≥0.8\ (-)}$ | 0.86 | 0.38 | 1.11 | 1.77 | 1.51 | 0.28 | 0.02 | 0.01 | -0.02 | -0.03 | -0.03 | 0.00 |
| $K03855_{VIP≥0.8\ (-)}$ | 1.27 | 0.95 | 1.08 | 0.63 | 1.33 | 0.80 | 0.03 | -0.01 | -0.02 | -0.01 | -0.03 | -0.01 |
| $K03154_{VIP≥0.8\ (-)}$ | 0.99 | 1.87 | 1.50 | 0.51 | 1.64 | 0.18 | -0.03 | 0.04 | -0.03 | -0.01 | -0.04 | 0.00 |
| $K00652_{VIP≥0.8\ (-)}$ | 1.05 | 0.52 | 0.52 | 1.83 | 1.23 | 0.87 | 0.03 | 0.00 | 0.00 | -0.03 | -0.02 | -0.01 |
| $K16927_{VIP≥0.8\ (-)}$ | 1.04 | 1.27 | 0.87 | 0.59 | 0.53 | 0.98 | -0.02 | -0.03 | -0.01 | 0.00 | -0.01 | -0.02 |
| $K01470_{VIP≥0.8\ (-)}$ | 1.02 | 0.19 | 1.37 | 1.37 | 1.01 | 0.82 | 0.03 | 0.00 | -0.02 | -0.02 | 0.00 | -0.01 |
| $K16329_{VIP≥0.8\ (-)}$ | 0.83 | 1.40 | 0.94 | 0.54 | 1.40 | 0.97 | 0.00 | -0.03 | 0.01 | 0.00 | -0.04 | -0.01 |
| $K04751_{VIP≥0.8\ (-)}$ | 0.94 | 0.72 | 1.09 | 1.00 | 0.99 | 0.28 | -0.02 | 0.01 | -0.01 | -0.01 | -0.01 | 0.00 |

| | | | | | | | | | | | | |
|---|---|---|---|---|---|---|---|---|---|---|---|---|
| K19118$_{VIP≥0.8\ (-)}$ | 0.97 | 0.24 | 0.95 | 1.30 | 1.01 | 0.57 | 0.00 | 0.00 | -0.01 | -0.01 | -0.01 | -0.01 |
| K07492$_{VIP≥0.8\ (-)}$ | 0.93 | 0.64 | 1.00 | 0.65 | 0.95 | 1.01 | -0.01 | 0.00 | -0.01 | 0.00 | -0.02 | -0.02 |
| K07105$_{VIP≥0.8\ (-)}$ | 0.90 | 0.19 | 0.94 | 1.31 | 0.85 | 0.74 | 0.02 | 0.00 | -0.01 | -0.02 | -0.01 | -0.01 |
| K10563$_{VIP≥0.8\ (-)}$ | 1.14 | 0.61 | 1.29 | 1.12 | 1.05 | 0.69 | 0.03 | 0.00 | -0.01 | -0.01 | 0.00 | -0.01 |
| K01091$_{VIP≥0.8\ (-)}$ | 0.96 | 0.57 | 1.06 | 1.10 | 1.49 | 0.45 | 0.02 | 0.00 | -0.01 | -0.01 | -0.03 | -0.01 |
| K02072$_{VIP≥0.8\ (-)}$ | 0.95 | 0.82 | 0.90 | 1.12 | 0.84 | 0.40 | 0.02 | -0.02 | -0.01 | -0.01 | -0.01 | -0.01 |
| K07334$_{VIP≥0.8\ (-)}$ | 1.38 | 0.43 | 0.93 | 1.15 | 1.42 | 0.17 | 0.04 | 0.01 | -0.01 | -0.02 | -0.03 | 0.00 |
| K02358$_{VIP≥0.8\ (-)}$ | 0.84 | 0.75 | 0.81 | 0.96 | 1.16 | 0.48 | 0.01 | -0.01 | 0.00 | 0.00 | -0.02 | -0.01 |
| K07478$_{VIP≥0.8\ (-)}$ | 1.54 | 0.92 | 1.14 | 1.10 | 1.32 | 0.33 | 0.04 | -0.01 | -0.01 | -0.01 | -0.02 | -0.01 |
| K12950$_{VIP≥0.8\ (-)}$ | 0.96 | 0.53 | 0.90 | 1.24 | 0.80 | 0.88 | 0.02 | 0.00 | 0.00 | -0.01 | -0.01 | -0.01 |
| K02114$_{VIP≥0.8\ (-)}$ | 1.09 | 0.12 | 0.95 | 1.19 | 1.06 | 0.67 | 0.02 | 0.00 | -0.01 | -0.01 | -0.01 | -0.01 |
| K01845$_{VIP≥0.8\ (-)}$ | 0.93 | 0.38 | 0.87 | 1.06 | 1.00 | 0.47 | 0.03 | 0.00 | -0.01 | -0.01 | -0.02 | -0.01 |
| K13993$_{VIP≥0.8\ (-)}$ | 0.70 | 1.11 | 0.82 | 0.71 | 1.17 | 0.89 | 0.00 | 0.02 | -0.01 | 0.00 | -0.02 | -0.02 |
| K00604$_{VIP≥0.8\ (-)}$ | 0.91 | 0.71 | 0.82 | 0.92 | 1.35 | 0.18 | 0.02 | 0.00 | -0.01 | -0.01 | -0.02 | 0.00 |
| K07118$_{VIP≥0.8\ (-)}$ | 1.04 | 0.46 | 0.89 | 0.97 | 0.98 | 0.23 | 0.00 | -0.01 | 0.01 | -0.01 | 0.00 | 0.00 |
| K03215$_{VIP≥0.8\ (-)}$ | 1.42 | 0.74 | 0.94 | 0.92 | 0.94 | 0.38 | 0.04 | 0.00 | 0.00 | 0.00 | 0.01 | -0.01 |
| K02115$_{VIP≥0.8\ (-)}$ | 0.85 | 0.81 | 1.01 | 0.72 | 0.88 | 0.22 | 0.00 | 0.01 | -0.01 | 0.00 | -0.01 | 0.00 |
| K04043$_{VIP≥0.8\ (-)}$ | 1.13 | 1.04 | 0.84 | 0.97 | 0.94 | 0.34 | -0.01 | -0.01 | 0.01 | 0.01 | -0.02 | 0.00 |
| K19689$_{VIP≥0.8\ (-)}$ | 0.98 | 0.92 | 0.76 | 0.87 | 1.01 | 0.21 | 0.02 | -0.02 | 0.00 | -0.01 | 0.01 | 0.00 |
| K00266$_{VIP≥0.8\ (-)}$ | 0.96 | 1.04 | 0.93 | 0.77 | 0.94 | 0.27 | 0.01 | -0.01 | -0.01 | 0.00 | -0.01 | 0.00 |
| K00937$_{VIP≥0.8\ (-)}$ | 1.06 | 0.38 | 0.85 | 0.93 | 1.12 | 0.22 | 0.01 | 0.01 | 0.00 | 0.00 | -0.02 | 0.00 |
| K03626$_{VIP≥0.8}$ | 0.94 | 0.37 | 0.98 | 0.86 | 0.84 | 0.97 | 0.00 | 0.00 | 0.01 | 0.00 | -0.01 | 0.02 |
| K16850$_{VIP≥0.8}$ | 1.86 | 0.76 | 0.89 | 0.95 | 0.84 | 1.73 | 0.05 | -0.01 | 0.01 | -0.02 | -0.02 | 0.02 |
| K02377$_{VIP≥0.8}$ | 0.85 | 1.09 | 1.47 | 1.97 | 0.37 | 0.92 | -0.02 | -0.01 | 0.02 | 0.03 | -0.01 | 0.01 |
| K07221$_{VIP≥0.8}$ | 0.68 | 1.29 | 1.30 | 1.27 | 0.91 | 1.64 | -0.02 | -0.01 | 0.02 | 0.02 | -0.02 | 0.02 |
| K07172$_{VIP≥0.8}$ | 2.29 | 0.49 | 0.94 | 1.02 | 1.17 | 0.27 | 0.06 | 0.01 | -0.01 | -0.02 | 0.02 | 0.00 |
| K04047$_{VIP≥0.8}$ | 2.00 | 0.17 | 0.83 | 0.88 | 1.04 | 0.62 | -0.04 | 0.00 | 0.00 | -0.01 | 0.02 | 0.01 |
| K07741$_{VIP≥0.8}$ | 1.63 | 1.70 | 0.84 | 0.65 | 1.29 | 0.69 | -0.03 | 0.03 | 0.01 | 0.00 | -0.03 | 0.01 |
| K00788$_{VIP≥0.8}$ | 1.44 | 1.02 | 0.64 | 1.01 | 0.42 | 1.07 | -0.02 | 0.02 | -0.01 | 0.02 | -0.01 | 0.02 |
| K03816$_{VIP≥0.8}$ | 0.85 | 0.67 | 0.87 | 0.50 | 1.04 | 0.93 | 0.00 | 0.01 | -0.01 | 0.01 | -0.03 | 0.01 |

| | | | | | | | | | | | | |
|---|---|---|---|---|---|---|---|---|---|---|---|---|
| K04085$_{VIP≥0.8}$ | 0.58 | 0.75 | 1.71 | 1.64 | 0.84 | 1.42 | 0.01 | 0.01 | -0.03 | -0.03 | 0.01 | -0.02 |
| K03046$_{VIP≥0.8}$ | 0.99 | 0.96 | 0.72 | 0.90 | 1.32 | 0.34 | 0.00 | -0.01 | 0.00 | 0.01 | -0.03 | -0.01 |
| K01876$_{VIP≥0.8}$ | 1.09 | 0.99 | 0.60 | 0.95 | 1.07 | 0.38 | 0.00 | -0.01 | 0.00 | 0.01 | -0.02 | 0.00 |
| K02355$_{VIP≥0.8}$ | 0.94 | 1.20 | 0.65 | 0.85 | 0.89 | 0.22 | 0.00 | -0.01 | 0.00 | 0.01 | -0.01 | 0.00 |
| K07495$_{VIP≥0.8}$ | 0.97 | 0.46 | 1.08 | 1.61 | 1.00 | 0.29 | -0.01 | 0.00 | -0.01 | -0.02 | 0.01 | 0.00 |
| K14095 | 0.85 | 0.52 | 1.20 | 0.89 | 0.79 | 0.64 | 0.01 | 0.01 | 0.01 | 0.01 | -0.01 | 0.01 |
| K14096 | 0.93 | 0.64 | 1.25 | 0.71 | 0.86 | 0.79 | 0.01 | 0.01 | 0.01 | 0.01 | -0.01 | 0.01 |
| K06961 | 0.82 | 0.59 | 0.88 | 0.73 | 0.62 | 0.92 | 0.01 | 0.01 | 0.00 | 0.00 | 0.00 | 0.01 |
| K02107 | 1.71 | 1.06 | 0.23 | 0.79 | 0.48 | 1.03 | -0.05 | 0.01 | 0.00 | 0.01 | 0.01 | 0.01 |
| K10218 | 0.46 | 1.80 | 1.31 | 0.57 | 1.86 | 0.71 | 0.01 | 0.04 | 0.02 | 0.01 | 0.05 | 0.01 |
| K11041 | 0.41 | 0.76 | 1.21 | 1.65 | 0.74 | 1.08 | -0.01 | 0.01 | 0.02 | 0.03 | -0.02 | 0.01 |
| K03048 | 0.78 | 0.56 | 0.60 | 1.29 | 1.72 | 0.87 | -0.02 | 0.00 | 0.01 | -0.02 | -0.04 | -0.01 |
| K01488 | 0.69 | 1.44 | 0.35 | 1.28 | 1.68 | 0.72 | -0.01 | 0.02 | 0.00 | -0.02 | -0.04 | -0.01 |
| K02759 | 0.92 | 0.46 | 0.71 | 1.10 | 1.05 | 0.33 | -0.01 | 0.00 | 0.00 | -0.01 | -0.01 | 0.00 |
| K12452 | 0.42 | 1.05 | 0.04 | 1.49 | 0.75 | 1.68 | 0.00 | 0.00 | 0.00 | 0.03 | 0.02 | 0.02 |
| K01771 | 0.94 | 0.47 | 0.79 | 0.81 | 1.44 | 0.17 | 0.01 | 0.01 | 0.00 | 0.01 | -0.03 | 0.00 |
| K00854 | 0.91 | 0.65 | 0.80 | 0.72 | 1.00 | 0.81 | 0.01 | 0.01 | 0.01 | 0.00 | 0.01 | 0.01 |
| K09825 | 1.30 | 0.46 | 0.83 | 0.75 | 1.04 | 0.21 | -0.01 | -0.01 | 0.00 | 0.00 | -0.02 | 0.00 |
| K07450 | 1.16 | 0.85 | 0.86 | 0.73 | 0.78 | 0.24 | -0.03 | -0.02 | -0.01 | -0.01 | 0.02 | 0.00 |
| K00662 | 0.78 | 1.31 | 0.97 | 0.77 | 0.32 | 1.08 | -0.01 | -0.03 | 0.01 | -0.01 | 0.00 | 0.02 |
| K01897 | 0.20 | 0.78 | 0.97 | 1.37 | 0.37 | 1.04 | 0.00 | 0.00 | -0.02 | -0.02 | 0.00 | -0.02 |
| K00978 | 0.29 | 1.20 | 0.71 | 0.60 | 1.14 | 1.58 | 0.01 | 0.01 | -0.01 | 0.01 | 0.03 | 0.02 |
| K01223 | 0.83 | 1.08 | 0.71 | 0.77 | 0.94 | 0.53 | 0.01 | -0.01 | 0.00 | -0.01 | 0.02 | -0.01 |
| K06867 | 1.11 | 0.80 | 0.72 | 0.99 | 0.25 | 0.64 | -0.03 | 0.00 | 0.00 | -0.01 | 0.00 | -0.01 |
| K01179 | 0.57 | 1.51 | 0.77 | 0.83 | 1.67 | 0.73 | -0.01 | -0.03 | 0.01 | 0.01 | -0.04 | 0.01 |
| K02217 | 1.18 | 1.07 | 0.71 | 0.79 | 0.72 | 0.89 | -0.02 | 0.02 | 0.00 | 0.01 | 0.01 | 0.01 |
| K08602 | 0.96 | 0.96 | 0.75 | 0.77 | 0.95 | 0.26 | 0.01 | -0.02 | 0.00 | -0.01 | 0.00 | 0.00 |
| K06177 | 0.38 | 0.73 | 0.97 | 1.66 | 0.13 | 1.31 | 0.01 | 0.00 | 0.01 | 0.03 | 0.00 | 0.02 |
| K21071 | 1.01 | 0.31 | 0.83 | 0.67 | 0.99 | 0.30 | 0.01 | -0.01 | -0.01 | 0.00 | 0.02 | 0.00 |
| K01997 | 0.94 | 0.82 | 0.69 | 0.63 | 0.81 | 0.65 | 0.00 | 0.01 | 0.00 | 0.00 | 0.01 | 0.01 |
| K01092 | 1.14 | 1.54 | 0.84 | 0.39 | 0.26 | 0.38 | 0.02 | -0.03 | -0.01 | -0.01 | 0.01 | -0.01 |
| K00941 | 1.26 | 0.54 | 0.90 | 0.74 | 0.81 | 0.28 | -0.01 | 0.01 | -0.01 | 0.01 | -0.02 | 0.00 |
| K01999 | 0.98 | 0.50 | 0.85 | 0.77 | 0.99 | 0.49 | 0.01 | 0.00 | 0.01 | 0.00 | 0.02 | 0.00 |
| K00390 | 0.83 | 0.34 | 0.89 | 0.75 | 1.08 | 0.44 | 0.02 | 0.00 | 0.00 | 0.00 | -0.02 | -0.01 |

| | | | | | | | | | | | | |
|---|---|---|---|---|---|---|---|---|---|---|---|---|
| K02035 | 0.95 | 0.55 | 0.74 | 0.88 | 0.94 | 0.62 | 0.01 | 0.00 | 0.00 | 0.00 | 0.00 | 0.01 |
| K09922 | 0.81 | 0.93 | 0.77 | 0.48 | 0.40 | 0.93 | 0.00 | -0.02 | 0.00 | 0.00 | 0.00 | 0.02 |
| K03803 | 0.85 | 0.85 | 0.62 | 0.70 | 1.05 | 0.15 | 0.01 | 0.01 | 0.00 | -0.01 | -0.03 | 0.00 |
| K01156 | 0.59 | 1.13 | 1.21 | 0.79 | 0.79 | 1.79 | 0.00 | 0.00 | 0.02 | 0.01 | 0.02 | 0.03 |
| K03972 | 1.21 | 1.03 | 0.88 | 0.46 | 0.35 | 0.38 | 0.03 | 0.01 | -0.01 | -0.01 | -0.01 | -0.01 |
| K01649 | 0.84 | 1.12 | 0.77 | 0.73 | 0.65 | 0.88 | 0.01 | 0.01 | 0.00 | 0.01 | 0.01 | 0.01 |
| K00927 | 1.02 | 1.50 | 0.49 | 0.78 | 1.33 | 0.58 | 0.03 | -0.02 | 0.00 | 0.01 | -0.03 | -0.01 |
| K02469 | 1.03 | 1.10 | 0.70 | 0.73 | 1.01 | 0.31 | 0.02 | -0.02 | 0.00 | 0.00 | -0.01 | 0.00 |
| K01613 | 0.77 | 0.99 | 0.77 | 1.04 | 0.97 | 0.36 | -0.01 | -0.01 | -0.01 | -0.02 | -0.02 | 0.00 |
| K00088 | 1.12 | 1.25 | 0.60 | 0.77 | 0.47 | 1.70 | -0.02 | 0.00 | 0.01 | 0.01 | 0.01 | 0.02 |
| K02112 | 1.36 | 1.03 | 0.76 | 0.79 | 0.81 | 0.21 | -0.02 | -0.01 | 0.00 | 0.00 | 0.00 | 0.00 |
| K06207 | 0.90 | 1.12 | 0.71 | 0.78 | 1.27 | 0.35 | 0.02 | -0.01 | 0.00 | 0.00 | -0.03 | 0.00 |
| K03070 | 1.14 | 1.07 | 0.77 | 0.73 | 0.95 | 0.65 | -0.01 | -0.01 | 0.00 | 0.00 | 0.01 | 0.01 |
| K00057 | 0.51 | 0.79 | 0.69 | 0.82 | 1.29 | 1.11 | 0.00 | 0.00 | -0.01 | -0.01 | -0.03 | 0.01 |
| K00826 | 1.02 | 1.19 | 0.71 | 0.87 | 0.74 | 0.59 | 0.03 | 0.00 | 0.00 | 0.01 | 0.00 | 0.00 |
| K01953 | 1.17 | 0.99 | 0.91 | 0.77 | 0.60 | 0.78 | 0.03 | -0.01 | 0.00 | 0.00 | 0.01 | 0.01 |
| K07137 | 0.88 | 1.04 | 0.91 | 0.64 | 0.76 | 0.63 | 0.00 | 0.01 | 0.01 | 0.01 | -0.01 | 0.01 |
| K03601 | 0.78 | 0.87 | 0.68 | 1.06 | 1.25 | 0.33 | 0.02 | -0.01 | 0.00 | -0.01 | -0.02 | 0.00 |
| K01740 | 0.55 | 1.51 | 0.65 | 1.03 | 0.38 | 1.76 | 0.00 | 0.02 | 0.01 | 0.02 | -0.01 | 0.02 |
| K06400 | 0.91 | 1.17 | 0.66 | 0.79 | 1.19 | 0.61 | 0.02 | -0.02 | 0.01 | 0.00 | 0.03 | -0.01 |
| K09131 | 0.90 | 0.77 | 0.64 | 0.68 | 1.25 | 0.33 | 0.01 | 0.00 | 0.00 | 0.00 | -0.03 | 0.00 |
| K08963 | 0.98 | 0.68 | 0.76 | 0.75 | 1.01 | 0.30 | 0.00 | 0.00 | 0.00 | 0.00 | 0.02 | 0.00 |
| K02032 | 0.94 | 0.46 | 0.68 | 0.73 | 1.38 | 0.24 | 0.00 | 0.01 | 0.00 | 0.00 | 0.03 | 0.00 |
| K02031 | 0.89 | 0.26 | 0.70 | 0.76 | 1.07 | 0.27 | 0.01 | 0.00 | 0.00 | -0.01 | 0.02 | 0.00 |
| K02027 | 0.99 | 0.60 | 0.66 | 0.74 | 0.93 | 0.77 | 0.00 | 0.01 | 0.00 | 0.00 | 0.01 | 0.01 |
| K02025 | 1.00 | 0.22 | 0.64 | 0.69 | 1.11 | 0.60 | 0.00 | 0.00 | 0.00 | 0.00 | 0.02 | 0.01 |
| K05919 | 0.94 | 0.86 | 0.75 | 0.61 | 0.52 | 0.39 | 0.02 | 0.01 | 0.00 | 0.00 | -0.01 | -0.01 |
| K07025 | 0.71 | 0.78 | 0.90 | 0.56 | 1.10 | 0.31 | -0.02 | -0.02 | 0.01 | 0.01 | 0.03 | 0.00 |
| K01627 | 0.67 | 0.87 | 0.41 | 0.78 | 0.27 | 1.28 | 0.01 | 0.01 | 0.00 | 0.01 | 0.01 | 0.02 |
| K01687 | 1.55 | 0.88 | 0.78 | 0.79 | 0.73 | 0.56 | 0.04 | 0.01 | 0.00 | 0.00 | 0.00 | 0.00 |
| K03320 | 0.87 | 1.44 | 0.64 | 0.71 | 0.64 | 0.64 | -0.02 | 0.02 | 0.00 | 0.01 | 0.00 | 0.01 |
| K02519 | 0.95 | 1.06 | 0.67 | 0.70 | 0.73 | 0.28 | -0.01 | -0.01 | 0.00 | 0.00 | 0.01 | 0.00 |
| K01874 | 0.66 | 1.10 | 0.72 | 1.14 | 0.68 | 0.61 | 0.01 | -0.01 | 0.01 | 0.02 | 0.01 | 0.01 |
| K00067 | 0.75 | 1.08 | 0.39 | 0.61 | 0.79 | 1.41 | -0.01 | 0.02 | 0.01 | 0.01 | 0.02 | 0.02 |
| K00548 | 0.62 | 1.16 | 0.77 | 0.63 | 0.44 | 0.92 | -0.01 | 0.01 | 0.01 | 0.01 | 0.00 | 0.01 |

| | | | | | | | | | | | | |
|---|---|---|---|---|---|---|---|---|---|---|---|---|
| K02838 | 0.41 | 0.78 | 0.82 | 0.56 | 1.63 | 0.42 | -0.01 | -0.01 | 0.00 | -0.01 | -0.04 | 0.00 |
| K03208 | 0.62 | 0.24 | 0.45 | 0.74 | 1.56 | 0.59 | 0.01 | 0.00 | 0.00 | -0.01 | 0.04 | 0.01 |
| K23997 | 0.74 | 0.55 | 0.58 | 0.79 | 0.98 | 0.71 | 0.01 | 0.01 | 0.01 | 0.01 | 0.02 | 0.00 |
| K07031 | 0.77 | 0.57 | 0.76 | 0.36 | 0.71 | 1.11 | 0.02 | -0.01 | -0.01 | 0.00 | -0.02 | 0.01 |
| K02837 | 0.68 | 0.89 | 0.75 | 0.77 | 0.78 | 0.33 | 0.00 | 0.00 | 0.00 | 0.00 | -0.01 | 0.00 |

Partial Least Squares analysis was performed with 2 latent components. Subscript VIP≥0.8 denotes microbial genera that had VIP ≥ 0.8 in 4 or more timepoints. Subscripts (+) and (-) represent microbial genera that had positive or negative regression coefficient in 4 or more timepoints, respectively.

**Supplementary table 3 - D. Functional information of significantly repeatable microbial genes with stable associations with host performance traits.**

| Microbial genes | Name (KEGG) | Definition (KEGG) | Function (COG) | KEGG orthology (KO) | BRITE hierarchies |
|---|---|---|---|---|---|
| K00003 | *hom* | homoserine dehydrogenase [EC:1.1.1.3] | Amino acid transport and metabolism | Metabolism - Amino acid metabolism (Glycine, serine and threonine metabolism; Cysteine and methionine metabolism; Lysine biosynthesis) | KO only |
| K00013 | *hisD* | histidinol dehydrogenase [EC:1.1.1.23] | Amino acid transport and metabolism | Metabolism - Amino acid metabolism (Histidine metabolism) | KO only |
| K00020 | *HIBADH* | 3-hydroxyisobutyrate dehydrogenase [EC:1.1.1.31] | Lipid transport and metabolism | Metabolism - Amino acid metabolism (Valine, leucine and isoleucine degradation) | KO only |
| K00046 | *idnO* | gluconate 5-dehydrogenase [EC:1.1.1.69] | Lipid transport and metabolism, Secondary metabolites biosynthesis, transport and catabolism, General function prediction only | Not Included in Pathway or Brite (Unclassified: metabolism) | |
| K00052 | *leuB* | 3-isopropylmalate dehydrogenase [EC:1.1.1.85] | Energy production and conversion, Amino acid transport and metabolism | Metabolism - Carbohydrate metabolism (C5-Branched dibasic acid metabolism), Amino acid metabolism (Valine, leucine and isoleucine biosynthesis) | KO only |
| K00057 | *gpsA* | glycerol-3-phosphate dehydrogenase (NAD(P)+) [EC:1.1.1.94] | Energy production and conversion | Metabolism - Lipid metabolism (Glycerophospholipid metabolism) | KO only |
| K00067 | *rfbD* | dTDP-4-dehydrorhamnose reductase [EC:1.1.1.133] | Cell wall/membrane/envelope biogenesis | Metabolism - Glycan biosynthesis and metabolism (O-Antigen nucleotide sugar biosynthesis), Metabolism of terpenoids and polyketides (Polyketide sugar unit biosynthesis), Biosynthesis of other secondary metabolites (Streptomycin biosynthesis) | KO only |

| | | | | | |
|---|---|---|---|---|---|
| K00077 | *panE* | 2-dehydropantoate 2-reductase [EC:1.1.1.169] | Coenzyme transport and metabolism | Metabolism - Metabolism of cofactors and vitamins (Pantothenate and CoA biosynthesis) | KO only |
| K00088 | *IMPDH* | IMP dehydrogenase [EC:1.1.1.205] | Nucleotide transport and metabolism; Signal transduction mechanisms | Metabolism - Nucleotide metabolism(Purine metabolism), Xenobiotics biodegradation and metabolism (Drug metabolism - other enzymes) | Protein families: signaling and cellular processes - Exosome. |
| K00125 | *fdhB* | formate dehydrogenase (coenzyme F420) beta subunit [EC:1.17.98.3 | Energy production and conversion | Metabolism - Energy metabolism (Methane metabolism) | KO only |
| K00145 | *argC* | N-acetyl-gamma-glutamyl-phosphate reductase [EC:1.2.1.38] | Amino acid transport and metabolism | Metabolism - Amino acid metabolism (Arginine biosynthesis) | KO only |
| K00259 | *ald* | alanine dehydrogenase [EC:1.4.1.1] | Amino acid transport and metabolism | Metabolism - Amino acid metabolism (Alanine, aspartate and glutamate metabolism), Metabolism of other amino acids (Taurine and hypotaurine metabolism) | KO only |
| K00265 | *gltB* | glutamate synthase (NADPH) large chain [EC:1.4.1.13] | Amino acid transport and metabolism | Metabolism - Energy metabolism (Nitrogen metabolism), Amino acid metabolism (Alanine, aspartate and glutamate metabolism) | Protein families: metabolism - Peptidases and inhibitors. |
| K00266 | *gltD* | glutamate synthase (NADPH) small chain [EC:1.4.1.13] | Amino acid transport and metabolism, General function prediction only | Metabolism - Energy metabolism (Nitrogen metabolism), Amino acid metabolism (Alanine, aspartate and glutamate metabolism) | KO only |
| K00286 | *proC* | pyrroline-5-carboxylate reductase [EC:1.5.1.2] | Amino acid transport and metabolism | Metabolism - Amino acid metabolism (Arginine and proline metabolism) | KO only |
| K00297 | *metF* | methylenetetrahydrofolate reductase (NADH) [EC:1.5.1.54] | Amino acid transport and metabolism | Metabolism - Energy metabolism (Carbon fixation pathways in prokaryotes), Metabolism of cofactors and vitamins (One carbon pool by folate) | KO only |

| | | | | | |
|---|---|---|---|---|---|
| K00343 | *nuoN* | NADH-quinone oxidoreductase subunit N [EC:7.1.1.2] | Energy production and conversion | Metabolism - Energy metabolism (Oxidative phosphorylation) | KO only |
| K00390 | *cysH* | phosphoadenosine phosphosulfate reductase [EC:1.8.4.8 1.8.4.10] | Amino acid transport and metabolism, Coenzyme transport and metabolism | Metabolism - Energy metabolism (Sulfur metabolism) | KO only |
| K00441 | *frhB* | coenzyme F420 hydrogenase subunit beta [EC:1.12.98.1] | Energy production and conversion | Metabolism - Energy metabolism (Methane metabolism) | KO only |
| K00548 | *metH* | 5-methyltetrahydrofolate--homocysteine methyltransferase [EC:2.1. | Amino acid transport and metabolism | Metabolism - Amino acid metabolism (Cysteine and methionine metabolism). Metabolism of other amino acids (Selenocompound metabolism). Metabolism of cofactors and vitamins (One carbon pool by folate) | KO only |
| K00558 | *DNMT1* | DNA (cytosine-5)-methyltransferase 1 [EC:2.1.1.37] | Replication, recombination and repair | Metabolism - Amino acid metabolism (Cysteine and methionine metabolism). Human Diseases - Cancer: overview (MicroRNAs in cancer) | Protein families: genetic information processing - Transcription factors; Chromosome and associated proteins. Protein families: signaling and cellular processes - Prokaryotic defense system. |
| K00573 | *pcm* | protein-L-isoaspartate(D-aspartate) O-methyltransferase [EC:2.1.1.77] | Posttranslational modification, protein turnover, chaperones | Not Included in Pathway or Brite (Unclassified: metabolism) | |
| K00604 | *MTFMT* | methionyl-tRNA formyltransferase [EC:2.1.2.9] | Translation, ribosomal structure and biogenesis | Metabolism - Metabolism of cofactors and vitamins (One carbon pool by folate). Genetic Information Processing - Translation (Aminoacyl-tRNA biosynthesis) | KO only |

| | | | | | |
|---|---|---|---|---|---|
| K00609 | *pyrB* | aspartate carbamoyltransferase catalytic subunit [EC:2.1.3.2] | Nucleotide transport and metabolism | Metabolism - Nucleotide metabolism (Pyrimidine metabolism), Amino acid metabolism (Alanine, aspartate and glutamate metabolism) | KO only |
| K00626 | *ACAT* | acetyl-CoA C-acetyltransferase [EC:2.3.1.9] | Lipid transport and metabolism | Metabolism: Carbohydrate metabolism (Pyruvate metabolism, Glyoxylate and dicarboxylate metabolism; Butanoate metabolism). Energy metabolism (Carbon fixation pathways in prokaryotes). Lipid metabolism (Fatty acid degradation). Amino acid metabolism (Valine, leucine and isoleucine degradation; Lysine degradation; Tryptophan metabolism). Metabolism of terpenoids and polyketides (Terpenoid backbone biosynthesis). Xenobiotics biodegradation and metabolism (Benzoate degradation). Environmental Information Processing: Signal transduction (Two-component system). Organismal Systems: Digestive system (Fat digestion and absorption). | Protein families: signaling and cellular processes - Exosome. |
| K00641 | *metX* | homoserine O-acetyltransferase/O-succinyltransferase [EC:2.3.1.31 | Amino acid transport and metabolism | Metabolism - Energy metabolism (Sulfur metabolism), Amino acid metabolism (Cysteine and methionine metabolism) | KO only |
| K00651 | *metA* | homoserine O-succinyltransferase/O-acetyltransferase [EC:2.3.1.46 | Amino acid transport and metabolism | Metabolism - Energy metabolism (Sulfur metabolism), Amino acid metabolism (Cysteine and methionine metabolism) | KO only |
| K00652 | *bioF* | 8-amino-7-oxononanoate synthase [EC:2.3.1.47] | Coenzyme transport and metabolism | Metabolism - Metabolism of cofactors and vitamins (Biotin metabolism) | Protein families: metabolism - Amino acid related enzymes. |
| K00656 | *E2.3.1.54* | formate C-acetyltransferase [EC:2.3.1.54] | Energy production and conversion | Metabolism - Carbohydrate metabolism (Pyruvate metabolism; Propanoate metabolism; Butanoate metabolism) | KO only |

| | | | | | |
|---|---|---|---|---|---|
| K00662 | *aacC* | aminoglycoside 3-N-acetyltransferase [EC:2.3.1.81] | | BRITE only | Protein families: signaling and cellular processes - Antimicrobial resistance genes. |
| K00684 | *aat* | leucyl/phenylalanyl-tRNA---protein transferase [EC:2.3.2.6] | Posttranslational modification, protein turnover, chaperones | Not Included in Pathway or Brite (Unclassified: metabolism) | |
| K00757 | *udp* | uridine phosphorylase [EC:2.4.2.3] | Nucleotide transport and metabolism | Metabolism - Nucleotide metabolism (Pyrimidine metabolism), Xenobiotics biodegradation and metabolism (Drug metabolism - other enzymes) | KO only |
| K00763 | *pncB* | nicotinate phosphoribosyltransferase [EC:6.3.4.21] | Coenzyme transport and metabolism | Metabolism - Metabolism of cofactors and vitamins (Nicotinate and nicotinamide metabolism) | KO only |
| K00766 | *trpD* | anthranilate phosphoribosyltransferase [EC:2.4.2.18] | Amino acid transport and metabolism | Metabolism - Amino acid metabolism (Phenylalanine, tyrosine and tryptophan biosynthesis) | KO only |
| K00784 | *rnz* | ribonuclease Z [EC:3.1.26.11] | Translation, ribosomal structure and biogenesis | BRITE only | Protein families: genetic information processing - Transfer RNA biogenesis. |
| K00788 | *thiE* | thiamine-phosphate pyrophosphorylase [EC:2.5.1.3] | Coenzyme transport and metabolism | Metabolism - Metabolism of cofactors and vitamins (Thiamine metabolism) | KO only |
| K00817 | *hisC* | histidinol-phosphate aminotransferase [EC:2.6.1.9] | Amino acid transport and metabolism | Metabolism - Amino acid metabolism (Histidine metabolism; Tyrosine metabolism; Phenylalanine metabolism; Phenylalanine, tyrosine and tryptophan biosynthesis), Biosynthesis of other secondary metabolites (Tropane, piperidine and pyridine alkaloid biosynthesis; Novobiocin biosynthesis) | Protein families: metabolism - Amino acid related enzymes. |

| | | | | | |
|---|---|---|---|---|---|
| K00820 | *glmS* | glutamine---fructose-6-phosphate transaminase (isomerizing) [EC:2 | Cell wall/membrane/envelope biogenesis | Metabolism - Carbohydrate metabolism (Amino sugar and nucleotide sugar metabolism), Amino acid metabolism (Alanine, aspartate and glutamate metabolism). Human Diseases - Cardiovascular disease (Diabetic cardiomyopathy), Endocrine and metabolic disease (Insulin resistance) | Protein families: metabolism - Peptidases and inhibitors. |
| K00826 | *E2.6.1.42* | branched-chain amino acid aminotransferase [EC:2.6.1.42] | Amino acid transport and metabolism, Coenzyme transport and metabolism | Metabolism - Amino acid metabolism (Cysteine and methionine metabolism; Valine, leucine and isoleucine degradation; Valine, leucine and isoleucine biosynthesis), Metabolism of cofactors and vitamins (Pantothenate and CoA biosynthesis), Biosynthesis of other secondary metabolites (Glucosinolate biosynthesis) | Protein families: metabolism - Amino acid related enzymes. |
| K00854 | *xylB* | xylulokinase [EC:2.7.1.17] | Carbohydrate transport and metabolism | Metabolism - Carbohydrate metabolism (Pentose and glucuronate interconversions) | KO only |
| K00856 | *ADK* | adenosine kinase [EC:2.7.1.20] | Carbohydrate transport and metabolism | Metabolism - Nucleotide metabolism (Purine metabolism) | KO only |
| K00864 | *glpK* | glycerol kinase [EC:2.7.1.30] | Energy production and conversion | Metabolism - Lipid metabolism (Glycerolipid metabolism). Organismal Systems - Endocrine system (PPAR signaling pathway), Environmental adaptation (Plant-pathogen interaction) | Protein families: signaling and cellular processes - Exosome. |
| K00878 | *thiM* | hydroxyethylthiazole kinase [EC:2.7.1.50] | Coenzyme transport and metabolism | Metabolism - Metabolism of cofactors and vitamins (Thiamine metabolism; Riboflavin metabolism) | KO only |
| K00882 | *fruK* | 1-phosphofructokinase [EC:2.7.1.56] | Carbohydrate transport and metabolism | Metabolism - Carbohydrate metabolism (Fructose and mannose metabolism). Environmental Information Processing - Membrane transport (Phosphotransferase system (PTS)) | KO only |

| | | | | | |
|---|---|---|---|---|---|
| K00887 | *dgkA* | undecaprenol kinase [EC:2.7.1.66] | Lipid transport and metabolism | Metabolism - Glycan biosynthesis and metabolism (Peptidoglycan biosynthesis) | KO only |
| K00927 | *PGK* | phosphoglycerate kinase [EC:2.7.2.3] | Carbohydrate transport and metabolism | Metabolism - Carbohydrate metabolism(Glycolysis / Gluconeogenesis), Energy metabolism (Carbon fixation in photosynthetic organisms). Environmental Information Processing - Signal transduction (HIF-1 signaling pathway) | Protein families: signaling and cellular processes - Exosome. |
| K00937 | *ppk1* | polyphosphate kinase [EC:2.7.4.1] | Inorganic ion transport and metabolism | Metabolism - Energy metabolism (Oxidative phosphorylation). Genetic Information Processing - Folding, sorting and degradation (RNA degradation) | Protein families: genetic information processing - Messenger RNA biogenesis. |
| K00940 | *ndk* | nucleoside-diphosphate kinase [EC:2.7.4.6] | Nucleotide transport and metabolism | Metabolism - Nucleotide metabolism (Purine metabolism, Pyrimidine metabolism), Xenobiotics biodegradation and metabolism (Drug metabolism - other enzymes). Environmental Information Processing - Signal transduction (MAPK signaling pathway - plant) | Protein families: genetic information processing - Membrane trafficking. |
| K00941 | *thiD* | hydroxymethylpyrimidine/phosphomethylpyrimidine kinase [EC:2.7.1. | Coenzyme transport and metabolism | Metabolism - Metabolism of cofactors and vitamins (Thiamine metabolism) | KO only |
| K00950 | *folK* | 2-amino-4-hydroxy-6-hydroxymethyldihydropteridine diphosphokinase | Coenzyme transport and metabolism | Metabolism - Metabolism of cofactors and vitamins (Folate biosynthesis) | KO only |
| K00978 | *rfbF* | glucose-1-phosphate cytidylyltransferase [EC:2.7.7.33] | Translation, ribosomal structure and biogenesis, Cell wall/membrane/envelope biogenesis | Metabolism - Carbohydrate metabolism (Starch and sucrose metabolism; Amino sugar and nucleotide sugar metabolism), Glycan biosynthesis and metabolism (O-Antigen nucleotide sugar biosynthesis) | KO only |

| | | | | | |
|---|---|---|---|---|---|
| K00991 | *ispD* | 2-C-methyl-D-erythritol 4-phosphate cytidylyltransferase [EC:2.7.7.60] | Lipid transport and metabolism | Metabolism - Metabolism of terpenoids and polyketides (Terpenoid backbone biosynthesis) | KO only |
| K00991 | *ispD* | 2-C-methyl-D-erythritol 4-phosphate cytidylyltransferase [EC:2.7.7.60] | Lipid transport and metabolism | Metabolism - Metabolism of terpenoids and polyketides (Terpenoid backbone biosynthesis) | KO only |
| K01005 | *tagT_U_V* | polyisoprenyl-teichoic acid--peptidoglycan teichoic acid tran | Cell wall/membrane/envelope biogenesis | Metabolism - Glycan biosynthesis and metabolism (Teichoic acid biosynthesis) | KO only |
| K01077 | *E3.1.3.1* | alkaline phosphatase [EC:3.1.3.1] | Inorganic ion transport and metabolism, General function prediction only | Metabolism - Metabolism of cofactors and vitamins (Thiamine metabolism, Folate biosynthesis). Environmental Information Processing - Signal transduction (Two-component system) | Protein families: signaling and cellular processes - Exosome; Glycosylphosphatidylinositol (GPI)-anchored proteins. |
| K01079 | *serB* | phosphoserine phosphatase [EC:3.1.3.3] | Amino acid transport and metabolism | Metabolism - Energy metabolism (Methane metabolism), Amino acid metabolism (Glycine, serine and threonine metabolism) | Protein families: metabolism - Protein phosphatases and associated proteins. |
| K01091 | *gph* | phosphoglycolate phosphatase [EC:3.1.3.18] | Energy production and conversion | Metabolism - Carbohydrate metabolism (Glyoxylate and dicarboxylate metabolism) | KO only |
| K01092 | *E3.1.3.25* | myo-inositol-1(or 4)-monophosphatase [EC:3.1.3.25] | Carbohydrate transport and metabolism | Metabolism - Carbohydrate metabolism(Inositol phosphate metabolism), Biosynthesis of other secondary metabolites (Streptomycin biosynthesis). Environmental Information Processing - Signal transduction (Phosphatidylinositol signaling system) | KO only |
| K01139 | *spoT* | GTP diphosphokinase / guanosine-3',5'-bis(diphosphate) 3'-diphosphatase [EC:2.7.6.5 3.1.7.2] | Signal transduction mechanisms, Transcription | Metabolism - Nucleotide metabolism (Purine metabolism) | Protein families: genetic information processing - Ribosome biogenesis. |

| | | | | | |
|---|---|---|---|---|---|
| K01156 | *res* | type III restriction enzyme [EC:3.1.21.5] | Defense mechanisms | BRITE only | Protein families: signaling and cellular processes - Prokaryotic defense system. |
| K01179 | *E3.2.1.4* | endoglucanase [EC:3.2.1.4] | Carbohydrate transport and metabolism | Metabolism - Carbohydrate metabolism (Starch and sucrose metabolism). Environmental Information Processing - Signal transduction (Two-component system) | KO only |
| K01183 | *E3.2.1.14* | chitinase [EC:3.2.1.14] | Carbohydrate transport and metabolism | Metabolism - Carbohydrate metabolism (Amino sugar and nucleotide sugar metabolism) | KO only |
| K01193 | *INV* | beta-fructofuranosidase [EC:3.2.1.26] | Carbohydrate transport and metabolism | Metabolism - Carbohydrate metabolism (Galactose metabolism; Starch and sucrose metabolism) | KO only |
| K01223 | *E3.2.1.86B* | 6-phospho-beta-glucosidase [EC:3.2.1.86] | Carbohydrate transport and metabolism | Metabolism - Carbohydrate metabolism (Glycolysis / Gluconeogenesis; Starch and sucrose metabolism) | KO only |
| K01258 | *pepT* | tripeptide aminopeptidase [EC:3.4.11.4] | Amino acid transport and metabolism | BRITE only | Protein families: metabolism - Peptidases and inhibitors. |
| K01267 | *DNPEP* | aspartyl aminopeptidase [EC:3.4.11.21] | Amino acid transport and metabolism | BRITE only | Protein families: metabolism - Peptidases and inhibitors. Protein families: genetic information processing - Membrane trafficking. |

| | | | | | |
|---|---|---|---|---|---|
| K01297 | *ldcA* | muramoyltetrapeptide carboxypeptidase [EC:3.4.17.13] | Cell wall/membrane/envelope biogenesis | BRITE only | Protein families: metabolism - Peptidases and inhibitors; Peptidoglycan biosynthesis and degradation proteins. |
| K01433 | *purU* | formyltetrahydrofolate deformylase [EC:3.5.1.10] | Nucleotide transport and metabolism | Metabolism - Nucleotide metabolism (Pyrimidine metabolism), Metabolism of cofactors and vitamins (One carbon pool by folate) | KO only |
| K01465 | *URA4* | dihydroorotase [EC:3.5.2.3] | Nucleotide transport and metabolism | Metabolism - Nucleotide metabolism (Pyrimidine metabolism) | KO only |
| K01470 | *E3.5.2.10* | creatinine amidohydrolase [EC:3.5.2.10] | Coenzyme transport and metabolism, Secondary metabolites biosynthesis, transport and catabolism | Metabolism - Amino acid metabolism (Arginine and proline metabolism) | KO only |
| K01488 | *add* | adenosine deaminase [EC:3.5.4.4] | Nucleotide transport and metabolism | Metabolism - Nucleotide metabolism (Purine metabolism). Human Diseases - Immune disease (Primary immunodeficiency) | KO only |
| K01500 | *fchA* | methenyltetrahydrofolate cyclohydrolase [EC:3.5.4.9] | Amino acid transport and metabolism | Metabolism - Energy metabolism (Carbon fixation pathways in prokaryotes), Metabolism of cofactors and vitamins (One carbon pool by folate) | KO only |
| K01610 | *E4.1.1.49* | phosphoenolpyruvate carboxykinase (ATP) [EC:4.1.1.49] | Energy production and conversion | Metabolism - Carbohydrate metabolism (Glycolysis / Gluconeogenesis; Citrate cycle (TCA cycle); Pyruvate metabolism), Energy metabolism(Carbon fixation in photosynthetic organisms) | KO only |
| K01613 | *psd* | phosphatidylserine decarboxylase [EC:4.1.1.65] | Lipid transport and metabolism | Metabolism - Lipid metabolism (Glycerophospholipid metabolism) | KO only |

| | | | | | |
|---|---|---|---|---|---|
| K01627 | *kdsA* | 2-dehydro-3-deoxyphosphooctonate aldolase (KDO 8-P synthase) [EC: | Cell wall/membrane/envelope biogenesis | Metabolism - Glycan biosynthesis and metabolism (Lipopolysaccharide biosynthesis) | Protein families: metabolism - Lipopolysaccharide biosynthesis proteins. |
| K01649 | *leuA* | 2-isopropylmalate synthase [EC:2.3.3.13] | Amino acid transport and metabolism | Metabolism - Carbohydrate metabolism (Pyruvate metabolism), Amino acid metabolism (Valine, leucine and isoleucine biosynthesis) | KO only |
| K01657 | *trpE* | anthranilate synthase component I [EC:4.1.3.27] | Amino acid transport and metabolism, Coenzyme transport and metabolism | Metabolism - Amino acid metabolism (Phenylalanine, tyrosine and tryptophan biosynthesis), Biosynthesis of other secondary metabolites (Phenazine biosynthesis), Cellular Processes - Cellular community - prokaryotes (Quorum sensing; Biofilm formation - Pseudomonas aeruginosa) | KO only |
| K01678 | *E4.2.1.2 AB* | fumarate hydratase subunit beta [EC:4.2.1.2] | Energy production and conversion | Metabolism - Carbohydrate metabolism (Citrate cycle (TCA cycle); Pyruvate metabolism), Energy metabolism (Carbon fixation pathways in prokaryotes) | KO only |
| K01686 | *uxuA* | mannonate dehydratase [EC:4.2.1.8] | Carbohydrate transport and metabolism | Metabolism - Carbohydrate metabolism (Pentose and glucuronate interconversions) | KO only |
| K01687 | *ilvD* | dihydroxy-acid dehydratase [EC:4.2.1.9] | Amino acid transport and metabolism, Carbohydrate transport and metabolism | Metabolism - Amino acid metabolism (Valine, leucine and isoleucine biosynthesis), Metabolism of cofactors and vitamins (Pantothenate and CoA biosynthesis) | KO only |
| K01696 | *trpB* | tryptophan synthase beta chain [EC:4.2.1.20] | Amino acid transport and metabolism | Metabolism - Amino acid metabolism (Glycine, serine and threonine metabolism; Phenylalanine, tyrosine and tryptophan biosynthesis) | KO only |

| | | | | | |
|---|---|---|---|---|---|
| K01703 | *leuC* | 3-isopropylmalate/(R)-2-methylmalate dehydratase large subunit [E | Amino acid transport and metabolism | Metabolism - Carbohydrate metabolism (C5-Branched dibasic acid metabolism), Amino acid metabolism (Valine, leucine and isoleucine biosynthesis), Biosynthesis of other secondary metabolites (Glucosinolate biosynthesis) | KO only |
| K01709 | *rfbG* | CDP-glucose 4,6-dehydratase [EC:4.2.1.45] | Cell wall/membrane/envelope biogenesis | Metabolism - Carbohydrate metabolism (Amino sugar and nucleotide sugar metabolism), Glycan biosynthesis and metabolism (O-Antigen nucleotide sugar biosynthesis) | KO only |
| K01733 | *thrC* | threonine synthase [EC:4.2.3.1] | Amino acid transport and metabolism | Metabolism - Amino acid metabolism (Glycine, serine and threonine metabolism), Metabolism of cofactors and vitamins (Vitamin B6 metabolism) | KO only |
| K01734 | *mgsA* | methylglyoxal synthase [EC:4.2.3.3] | Carbohydrate transport and metabolism | Metabolism - Carbohydrate metabolism (Propanoate metabolism) | KO only |
| K01738 | *cysK* | cysteine synthase [EC:2.5.1.47] | Amino acid transport and metabolism | Metabolism - Energy metabolism (Sulfur metabolism), Amino acid metabolism (Cysteine and methionine metabolism) | KO only |
| K01740 | *metY* | O-acetylhomoserine (thiol)-lyase [EC:2.5.1.49] | Amino acid transport and metabolism | Metabolism - Amino acid metabolism (Cysteine and methionine metabolism) | KO only |
| K01752 | *E4.3.1.17* | L-serine dehydratase [EC:4.3.1.17] | Amino acid transport and metabolism | Metabolism - Amino acid metabolism (Glycine, serine and threonine metabolism; Cysteine and methionine metabolism) | KO only |
| K01756 | *purB* | adenylosuccinate lyase [EC:4.3.2.2] | Nucleotide transport and metabolism | Metabolism - Nucleotide metabolism (Purine metabolism), Amino acid metabolism (Alanine, aspartate and glutamate metabolism) | KO only |
| K01759 | *GLO1* | lactoylglutathione lyase [EC:4.4.1.5] | Secondary metabolites biosynthesis, transport and catabolism | Metabolism - Carbohydrate metabolism (Pyruvate metabolism) | KO only |
| K01771 | *plc* | 1-phosphatidylinositol phosphodiesterase [EC:4.6.1.13] | | Metabolism - Carbohydrate metabolism (Inositol phosphate metabolism) | KO only |

| | | | | | |
|---|---|---|---|---|---|
| K01815 | *kduI* | 4-deoxy-L-threo-5-hexosulose-uronate ketol-isomerase [EC:5.3.1.17 | Carbohydrate transport and metabolism | Metabolism - Carbohydrate metabolism (Pentose and glucuronate interconversions) | KO only |
| K01823 | *idi* | isopentenyl-diphosphate Delta-isomerase [EC:5.3.3.2] | Energy production and conversion, Lipid transport and metabolism, General function prediction only | Metabolism - Metabolism of terpenoids and polyketides (Terpenoid backbone biosynthesis) | KO only |
| K01845 | *hemL* | glutamate-1-semialdehyde 2,1-aminomutase [EC:5.4.3.8] | Coenzyme transport and metabolism | Metabolism - Metabolism of cofactors and vitamins (Porphyrin metabolism) | Protein families: metabolism - Amino acid related enzymes. |
| K01874 | *MARS* | methionyl-tRNA synthetase [EC:6.1.1.10] | Translation, ribosomal structure and biogenesis | Metabolism - Metabolism of other amino acids (Selenocompound metabolism). Genetic Information Processing - Translation (Aminoacyl-tRNA biosynthesis) | Protein families: metabolism - Amino acid related enzymes. Protein families: genetic information processing - Transfer RNA biogenesis. |
| K01876 | *DARS2* | aspartyl-tRNA synthetase [EC:6.1.1.12] | Translation, ribosomal structure and biogenesis | Genetic Information Processing - Translation (Aminoacyl-tRNA biosynthesis) | Protein families: metabolism - Amino acid related enzymes. Protein families: genetic information processing - Transfer RNA biogenesis; Mitochondrial biogenesis. |

| | | | | | |
|---|---|---|---|---|---|
| K01897 | *ACSL* | long-chain acyl-CoA synthetase [EC:6.2.1.3] | Lipid transport and metabolism, Secondary metabolites biosynthesis, transport and catabolism | Metabolism - Lipid metabolism (Fatty acid biosynthesis; Fatty acid degradation). Cellular Processes - Transport and catabolism (Peroxisome); Cell growth and death (Ferroptosis); Cellular community - prokaryotes (Quorum sensing). Organismal Systems - Endocrine system (Adipocytokine signaling pathway; PPAR signaling pathway), Environmental adaptation(Thermogenesis) | Protein families: metabolism - Lipid biosynthesis proteins. Protein families: signaling and cellular processes - Exosome. |
| K01915 | *glnA* | glutamine synthetase [EC:6.3.1.2] | Amino acid transport and metabolism | Metabolism - Carbohydrate metabolism (Glyoxylate and dicarboxylate metabolism), Energy metabolism (Nitrogen metabolism), Amino acid metabolism (Alanine, aspartate and glutamate metabolism; Arginine biosynthesis). Environmental Information Processing - Signal transduction (Two-component system). Cellular Processes - Cell growth and death (Necroptosis). Organismal Systems - Nervous system (Glutamatergic synapse; GABAergic synapse) | Protein families: signaling and cellular processes - Exosome. |
| K01938 | *fhs* | formate--tetrahydrofolate ligase [EC:6.3.4.3] | Nucleotide transport and metabolism | Metabolism - Energy metabolism (Carbon fixation pathways in prokaryotes), Metabolism of cofactors and vitamins (One carbon pool by folate) | KO only |
| K01940 | *argG* | argininosuccinate synthase [EC:6.3.4.5] | Amino acid transport and metabolism | Metabolism - Amino acid metabolism (Alanine, aspartate and glutamate metabolism; Arginine biosynthesis). Human Diseases - Cardiovascular disease (Fluid shear stress and atherosclerosis) | Protein families: signaling and cellular processes - Exosome. |
| K01952 | *PFAS* | phosphoribosylformylglycinamidine synthase [EC:6.3.5.3] | Nucleotide transport and metabolism | Metabolism - Nucleotide metabolism (Purine metabolism) | KO only |

| | | | | | |
|---|---|---|---|---|---|
| K01953 | *asnB* | asparagine synthase (glutamine-hydrolysing) [EC:6.3.5.4] | Amino acid transport and metabolism | Metabolism - Amino acid metabolism (Alanine, aspartate and glutamate metabolism) | Protein families: metabolism - Peptidases and inhibitors. |
| K01955 | *carB* | carbamoyl-phosphate synthase large subunit [EC:6.3.5.5] | Amino acid transport and metabolism, Nucleotide transport and metabolism | Metabolism - Nucleotide metabolism (Pyrimidine metabolism), Amino acid metabolism (Alanine, aspartate and glutamate metabolism) | KO only |
| K01989 | | putative tryptophan/tyrosine transport system substrate-binding protein | ABC-type uncharacterized transport system, periplasmic component [General function prediction only] | BRITE only | Protein families: signaling and cellular processes - Transporters. |
| K01997 | *livH* | branched-chain amino acid transport system permease protein | Amino acid transport and metabolism | Environmental Information Processing - Membrane transport (ABC transporters). Cellular Processes - Cellular community; prokaryotes (Quorum sensing). | Protein families: signaling and cellular processes - Transporters. |
| K01999 | *livK* | branched-chain amino acid transport system substrate-binding prot | Amino acid transport and metabolism | Environmental Information Processing - Membrane transport (ABC transporters). Cellular Processes - Cellular community; prokaryotes (Quorum sensing). | Protein families: signaling and cellular processes - Transporters. |
| K02025 | *ABC.MS.P* | multiple sugar transport system permease protein | Carbohydrate transport and metabolism | BRITE only | Protein families: signaling and cellular processes - Transporters. |
| K02026 | *ABC.MS.P1* | multiple sugar transport system permease protein | Carbohydrate transport and metabolism | BRITE only | Protein families: signaling and cellular processes - Transporters. |
| K02027 | *ABC.MS.S* | multiple sugar transport system substrate-binding protein | Carbohydrate transport and metabolism | BRITE only | Protein families: signaling and cellular processes - Transporters. |

| | | | | | |
|---|---|---|---|---|---|
| K02029 | *ABC.PA.P* | polar amino acid transport system permease protein | Amino acid transport and metabolism | BRITE only | Protein families: signaling and cellular processes - Transporters. |
| K02031 | *ddpD* | peptide/nickel transport system ATP-binding protein | Amino acid transport and metabolism, Inorganic ion transport and metabolism | Cellular Processes - Cellular community - prokaryotes (Quorum sensing) | Protein families: signaling and cellular processes - Transporters. |
| K02032 | *ddpF* | peptide/nickel transport system ATP-binding protein | Amino acid transport and metabolism, Inorganic ion transport and metabolism | Cellular Processes - Cellular community - prokaryotes (Quorum sensing) | Protein families: signaling and cellular processes - Transporters. |
| K02034 | *ABC.PE.P1* | peptide/nickel transport system permease protein | Amino acid transport and metabolism, Inorganic ion transport and metabolism | Cellular Processes - Cellular community - prokaryotes (Quorum sensing) | Protein families: signaling and cellular processes - Transporters. |
| K02035 | *ABC.PE.S* | peptide/nickel transport system substrate-binding protein | Amino acid transport and metabolism | Cellular Processes - Cellular community - prokaryotes (Quorum sensing) | Protein families: signaling and cellular processes - Transporters. |
| K02036 | *pstB* | phosphate transport system ATP-binding protein [EC:7.3.2.1] | Inorganic ion transport and metabolism | Environmental Information Processing - Membrane transport (ABC transporters) | Protein families: signaling and cellular processes - Transporters. |
| K02037 | *pstC* | phosphate transport system permease protein | Inorganic ion transport and metabolism | Environmental Information Processing - Membrane transport (ABC transporters) | Protein families: signaling and cellular processes - Transporters. |
| K02039 | *phoU* | phosphate transport system protein | Inorganic ion transport and metabolism | Not Included in Pathway or Brite (Unclassified: signaling and cellular processes) | |
| K02056 | *ABC.SS.A* | simple sugar transport system ATP-binding protein [EC:7.5.2.-] | Carbohydrate transport and metabolism | BRITE only | Protein families: signaling and cellular processes - Transporters. |

| | | | | | |
|---|---|---|---|---|---|
| K02066 | *mlaE* | phospholipid/cholesterol/gamma-HCH transport system permease prot | Cell wall/membrane/envelope biogenesis | Environmental Information Processing - Membrane transport (ABC transporters) | Protein families: signaling and cellular processes - Transporters. |
| K02072 | *metI* | D-methionine transport system permease protein | Amino acid transport and metabolism | Environmental Information Processing - Membrane transport (ABC transporters) | Protein families: signaling and cellular processes - Transporters. |
| K02078 | *acpP* | acyl carrier protein | Lipid transport and metabolism, Secondary metabolites biosynthesis, transport and catabolism | Metabolism - Lipid metabolism (Fatty acid biosynthesis), Biosynthesis of other secondary metabolites (Biosynthesis of various antibiotics) | KO only |
| K02086 | *dnaD* | DNA replication protein | Replication, recombination and repair | Not Included in Pathway or Brite (Unclassified: genetic information processing) | |
| K02099 | *araC* | AraC family transcriptional regulator, arabinose operon regulatory protein | Transcription | BRITE only | Protein families: genetic information processing - Transcription factors. |
| K02107 | *ATPVG* | V/A-type H+/Na+-transporting ATPase subunit G/H | Energy production and conversion | Metabolism - Energy metabolism (Oxidative phosphorylation) | KO only |
| K02112 | *ATPF1B* | F-type H+/Na+-transporting ATPase subunit beta [EC:7.1.2.2 7.2.2.1] | Energy production and conversion | Metabolism - Energy metabolism (Oxidative phosphorylation; Photosynthesis) | Protein families: metabolism - Photosynthesis proteins. |
| K02114 | *ATPF1E* | F-type H+-transporting ATPase subunit epsilon | Energy production and conversion | Metabolism - Energy metabolism (Oxidative phosphorylation; Photosynthesis) | Protein families: metabolism - Photosynthesis proteins. |
| K02115 | *ATPF1G* | F-type H+-transporting ATPase subunit gamma | Energy production and conversion | Metabolism - Energy metabolism (Oxidative phosphorylation; Photosynthesis) | Protein families: metabolism - Photosynthesis proteins. |

| | | | | | |
|---|---|---|---|---|---|
| K02217 | *ftnA* | ferritin [EC:1.16.3.2] | Inorganic ion transport and metabolism | Not Included in Pathway or Brite (Unclassified: metabolism) | |
| K02233 | *E2.7.8.26* | adenosylcobinamide-GDP ribazoletransferase [EC:2.7.8.26] | Coenzyme transport and metabolism | Metabolism - Metabolism of cofactors and vitamins (Porphyrin metabolism) | KO only |
| K02313 | *dnaA* | chromosomal replication initiator protein | Replication, recombination and repair | Environmental Information Processing - Signal transduction (Two-component system). Cellular Processes - Cell growth and death (Cell cycle - Caulobacter) | Protein families: genetic information processing - DNA replication proteins; Chromosome and associated proteins. |
| K02337 | *dnaE* | DNA polymerase III subunit alpha [EC:2.7.7.7] | Replication, recombination and repair | Genetic Information Processing - Replication and repair (DNA replication; Mismatch repair; Homologous recombination) | Protein families: genetic information processing - DNA replication proteins; DNA repair and recombination proteins. |
| K02355 | *fusA* | elongation factor G | Translation, ribosomal structure and biogenesis | BRITE only | Protein families: genetic information processing - Translation factors; Mitochondrial biogenesis. |
| K02358 | *tuf* | elongation factor Tu | Translation, ribosomal structure and biogenesis | Organismal Systems - Environmental adaptation (Plant-pathogen interaction) | Protein families: genetic information processing - Translation factors; Mitochondrial biogenesis. Protein families: signaling and cellular processes - Exosome. |

| | | | | | |
|---|---|---|---|---|---|
| K02377 | *TSTA3* | GDP-L-fucose synthase [EC:1.1.1.271] | Cell wall/membrane/envelope biogenesis | Metabolism - Carbohydrate metabolism (Fructose and mannose metabolism; Amino sugar and nucleotide sugar metabolism), Glycan biosynthesis and metabolism (O-Antigen nucleotide sugar biosynthesis) | KO only |
| K02469 | *gyrA* | DNA gyrase subunit A [EC:5.6.2.2] | Replication, recombination and repair | BRITE only | Protein families: genetic information processing - DNA replication proteins; DNA repair and recombination proteins. |
| K02470 | *gyrB* | DNA gyrase subunit B [EC:5.6.2.2] | Replication, recombination and repair | BRITE only | Protein families: genetic information processing - DNA replication proteins; DNA repair and recombination proteins. |
| K02500 | *hisF* | imidazole glycerol-phosphate synthase subunit HisF [EC:4.3.2.10] | Amino acid transport and metabolism | Metabolism - Amino acid metabolism (Histidine metabolism) | KO only |
| K02518 | *infA* | translation initiation factor IF-1 | Translation, ribosomal structure and biogenesis | BRITE only | Protein families: genetic information processing - Translation factors. |
| K02519 | *infB* | translation initiation factor IF-2 | Translation, ribosomal structure and biogenesis | BRITE only | Protein families: genetic information processing - Translation factors; Mitochondrial biogenesis. |

| | | | | | |
|---|---|---|---|---|---|
| K02529 | *lacI* | LacI family transcriptional regulator | Transcription | BRITE only | Protein families: genetic information processing - Transcription factors. |
| K02536 | *lpxD* | UDP-3-O-[3-hydroxymyristoyl] glucosamine N-acyltransferase [EC:2. | Cell wall/membrane/envelope biogenesis | Metabolism - Glycan biosynthesis and metabolism (Lipopolysaccharide biosynthesis) | Protein families: metabolism - Lipopolysaccharide biosynthesis proteins. |
| K02557 | *motB* | chemotaxis protein MotB | Cell motility | Cellular Processes - Cell motility (Bacterial chemotaxis; Flagellar assembly) | Protein families: signaling and cellular processes - Transporters; Bacterial motility proteins. |
| K02647 | *cdaR* | carbohydrate diacid regulator | Transcription, Signal transduction mechanisms | BRITE only | Protein families: genetic information processing - Transcription factors. |
| K02759 | *celC* | cellobiose PTS system EIIA component [EC:2.7.1.196 2.7.1.205] | Carbohydrate transport and metabolism | Metabolism - Carbohydrate metabolism (Starch and sucrose metabolism). Environmental Information Processing - Membrane transport (Phosphotransferase system (PTS)) | Protein families: signaling and cellular processes - Transporters. |
| K02775 | *gatC* | galactitol PTS system EIIC component | Carbohydrate transport and metabolism | Metabolism - Carbohydrate metabolism (Galactose metabolism). Environmental Information Processing - Membrane transport (Phosphotransferase system (PTS)) | Protein families: signaling and cellular processes - Transporters. |
| K02822 | *ulaB* | ascorbate PTS system EIIB component [EC:2.7.1.194] | Carbohydrate transport and metabolism | Metabolism - Carbohydrate metabolism (Ascorbate and aldarate metabolism). Environmental Information Processing - Membrane transport (Phosphotransferase system (PTS)) | Protein families: signaling and cellular processes - Transporters. |

| | | | | | |
|---|---|---|---|---|---|
| K02836 | *prfB* | peptide chain release factor 2 | Translation, ribosomal structure and biogenesis | BRITE only | Protein families: genetic information processing - Translation factors. |
| K02837 | *prfC* | peptide chain release factor 3 | Translation, ribosomal structure and biogenesis | BRITE only | Protein families: genetic information processing - Translation factors. |
| K02838 | *frr* | ribosome recycling factor | Translation, ribosomal structure and biogenesis | BRITE only | Protein families: genetic information processing - Translation factors. |
| K02866 | *RP-L10e* | large subunit ribosomal protein L10e | Translation, ribosomal structure and biogenesis | Genetic Information Processing - Translation (Ribosome). Human Diseases - Infectious disease: viral (Coronavirus disease - COVID-19) | Protein families: genetic information processing - Ribosome. |
| K02875 | *RP-L14e* | large subunit ribosomal protein L14e | Translation, ribosomal structure and biogenesis | Genetic Information Processing - Translation (Ribosome). Human Diseases - Infectious disease: viral (Coronavirus disease - COVID-19) | Protein families: genetic information processing - Ribosome. |
| K02884 | *RP-L19* | large subunit ribosomal protein L19 | Translation, ribosomal structure and biogenesis | Genetic Information Processing - Translation (Ribosome) | Protein families: genetic information processing - Ribosome. |
| K02889 | *RP-L21e* | large subunit ribosomal protein L21e | Translation, ribosomal structure and biogenesis | Genetic Information Processing - Translation (Ribosome). Human Diseases - Infectious disease: viral (Coronavirus disease - COVID-19) | Protein families: genetic information processing - Ribosome. |
| K02908 | *RP-L30e* | large subunit ribosomal protein L30e | Translation, ribosomal structure and biogenesis | Genetic Information Processing - Translation (Ribosome). Human Diseases - Infectious disease: viral (Coronavirus disease - COVID-19) | Protein families: genetic information processing - Ribosome. |

| | | | | | |
|---|---|---|---|---|---|
| K02912 | *RP-L32e* | large subunit ribosomal protein L32e | Translation, ribosomal structure and biogenesis | Genetic Information Processing - Translation (Ribosome). Human Diseases - Infectious disease: viral (Coronavirus disease - COVID-19) | Protein families: genetic information processing - Ribosome. |
| K02913 | *RP-L33* | large subunit ribosomal protein L33 | Translation, ribosomal structure and biogenesis | Genetic Information Processing - Translation (Ribosome) | Protein families: genetic information processing - Ribosome. |
| K02916 | *RP-L35* | large subunit ribosomal protein L35 | Translation, ribosomal structure and biogenesis | Genetic Information Processing - Translation (Ribosome) | Protein families: genetic information processing - Ribosome. |
| K02919 | *RP-L36* | large subunit ribosomal protein L36 | Translation, ribosomal structure and biogenesis | Genetic Information Processing - Translation (Ribosome) | Protein families: genetic information processing - Ribosome. |
| K02921 | *RP-L37Ae* | large subunit ribosomal protein L37Ae | Translation, ribosomal structure and biogenesis | Genetic Information Processing - Translation (Ribosome). Human Diseases - Infectious disease: viral (Coronavirus disease - COVID-19) | Protein families: genetic information processing - Ribosome. |
| K02936 | *RP-L7Ae* | large subunit ribosomal protein L7Ae | Translation, ribosomal structure and biogenesis | Genetic Information Processing - Translation (Ribosome). Human Diseases - Infectious disease: viral (Coronavirus disease - COVID-19) | Protein families: genetic information processing - Ribosome; Ribosome biogenesis. |
| K02966 | *RP-S19e* | small subunit ribosomal protein S19e | Translation, ribosomal structure and biogenesis | Genetic Information Processing - Translation (Ribosome). Human Diseases - Infectious disease: viral (Coronavirus disease - COVID-19) | Protein families: genetic information processing - Ribosome. |
| K02979 | *RP-S28e* | small subunit ribosomal protein S28e | Translation, ribosomal structure and biogenesis | Genetic Information Processing - Translation (Ribosome). Human Diseases - Infectious disease: viral (Coronavirus disease - COVID-19) | Protein families: genetic information processing - Ribosome. |

| | | | | | |
|---|---|---|---|---|---|
| K02987 | *RP-S4e* | small subunit ribosomal protein S4e | Translation, ribosomal structure and biogenesis | Genetic Information Processing - Translation (Ribosome). Human Diseases - Infectious disease: viral (Coronavirus disease - COVID-19) | Protein families: genetic information processing - Ribosome. |
| K02991 | *RP-S6e* | small subunit ribosomal protein S6e | Translation, ribosomal structure and biogenesis | Genetic Information Processing - Translation (Ribosome). Environmental Information Processing - Signal transduction (Apelin signaling pathway, HIF-1 signaling pathway, PI3K-Akt signaling pathway, mTOR signaling pathway),Organismal Systems - Endocrine system (Insulin signaling pathway), Environmental adaptation (Thermogenesis). Human Diseases - Cancer: overview (Proteoglycans in cancer), Infectious disease: viral (Coronavirus disease - COVID-19), Drug resistance: antineoplastic (EGFR tyrosine kinase inhibitor resistance) | Protein families: genetic information processing - Ribosome. |
| K02995 | *RP-S8e* | small subunit ribosomal protein S8e | Translation, ribosomal structure and biogenesis | Genetic Information Processing - Translation (Ribosome). Human Diseases - Infectious disease: viral (Coronavirus disease - COVID-19) | Protein families: genetic information processing - Ribosome. |
| K02996 | *RP-S9* | small subunit ribosomal protein S9 | Translation, ribosomal structure and biogenesis | Genetic Information Processing - Translation (Ribosome) | Protein families: genetic information processing - Ribosome. |
| K03046 | *rpoC* | DNA-directed RNA polymerase subunit beta' [EC:2.7.7.6] | Transcription | Genetic Information Processing - Transcription (RNA polymerase) | Protein families: genetic information processing - Transcription machinery; DNA repair and recombination proteins. |

| | | | | | |
|---|---|---|---|---|---|
| K03048 | *rpoE* | DNA-directed RNA polymerase subunit delta | Transcription | Genetic Information Processing - Transcription (RNA polymerase) | Protein families: genetic information processing - Transcription machinery; DNA repair and recombination proteins. |
| K03057 | *tfs* | transcription factor S | Transcription | BRITE only | Protein families: genetic information processing - Transcription machinery. |
| K03070 | *secA* | preprotein translocase subunit SecA [EC:7.4.2.8] | Intracellular trafficking, secretion, and vesicular transport | Genetic Information Processing - Folding, sorting and degradation (Protein export). Environmental Information Processing - Membrane transport (Bacterial secretion system), Cellular Processes - Cellular community - prokaryotes (Quorum sensing) | Protein families: signaling and cellular processes - Secretion system. |
| K03105 | *SRP19* | signal recognition particle subunit SRP19 | Intracellular trafficking, secretion, and vesicular transport | Genetic Information Processing - Folding, sorting and degradation (Protein export) | Protein families: signaling and cellular processes - Secretion system. |
| K03106 | *SRP54* | signal recognition particle subunit SRP54 [EC:3.6.5.4] | Intracellular trafficking, secretion, and vesicular transport | Genetic Information Processing - Folding, sorting and degradation (Protein export). Environmental Information Processing - Membrane transport (Bacterial secretion system), Cellular Processes - Cellular community - prokaryotes (Quorum sensing) | Protein families: signaling and cellular processes - Secretion system. |

| | | | | | |
|---|---|---|---|---|---|
| K03120 | *TBP* | transcription initiation factor TFIID TATA-box-binding protein | | Genetic Information Processing - Transcription (Basal transcription factors). Human Diseases - Cancer: overview (Viral carcinogenesis), Infectious disease: viral (Human T-cell leukemia virus 1 infection, Human papillomavirus infection), Neurodegenerative disease (Huntington disease, Spinocerebellar ataxia) | Protein families: genetic information processing - Transcription factors; Transcription machinery. |
| K03147 | *thiC* | phosphomethylpyrimidine synthase [EC:4.1.99.17] | Coenzyme transport and metabolism | Metabolism - Metabolism of cofactors and vitamins (Thiamine metabolism) | KO only |
| K03154 | *thiS* | sulfur carrier protein | Coenzyme transport and metabolism | Genetic Information Processing - Folding, sorting and degradation (Sulfur relay system) | KO only |
| K03181 | *ubiC* | chorismate lyase [EC:4.1.3.40] | Coenzyme transport and metabolism | Metabolism - Metabolism of cofactors and vitamins (Ubiquinone and other terpenoid-quinone biosynthesis) | KO only |
| K03183 | *ubiE* | demethylmenaquinone methyltransferase / 2-methoxy-6-polyprenyl-1, | Coenzyme transport and metabolism | Metabolism - Metabolism of cofactors and vitamins (Ubiquinone and other terpenoid-quinone biosynthesis) | KO only |
| K03208 | *wcaI* | putative colanic acid biosynthesis glycosyltransferase WcaI | Cell wall/membrane/envelope biogenesis | Metabolism - Glycan biosynthesis and metabolism (Exopolysaccharide biosynthesis) | KO only |
| K03215 | *rumA* | 23S rRNA (uracil1939-C5)-methyltransferase [EC:2.1.1.190] | Translation, ribosomal structure and biogenesis | BRITE only | Protein families: genetic information processing - Ribosome biogenesis. |
| K03236 | *EIF1A* | translation initiation factor 1A | Translation, ribosomal structure and biogenesis | BRITE only | Protein families: genetic information processing - Translation factors. |
| K03263 | *EIF5A* | translation initiation factor 5A | Translation, ribosomal structure and biogenesis | BRITE only | Protein families: genetic information processing - Translation factors. |

| | | | | | |
|---|---|---|---|---|---|
| K03264 | *EIF6* | translation initiation factor 6 | Translation, ribosomal structure and biogenesis | Genetic Information Processing - Translation (Ribosome biogenesis in eukaryotes) | Protein families: genetic information processing - Ribosome biogenesis; Translation factors. |
| K03269 | *lpxH* | UDP-2,3-diacylglucosamine hydrolase [EC:3.6.1.54] | Cell wall/membrane/envelope biogenesis | Metabolism - Glycan biosynthesis and metabolism (Lipopolysaccharide biosynthesis) | Protein families: metabolism - Lipopolysaccharide biosynthesis proteins. |
| K03320 | *amt* | ammonium transporter, Amt family | Inorganic ion transport and metabolism | BRITE only | Protein families: signaling and cellular processes - Transporters. |
| K03327 | *TC.MAT E* | multidrug resistance protein, MATE family | Defense mechanisms | BRITE only | Protein families: signaling and cellular processes - Transporters. |
| K03394 | *cobI-cbiL* | precorrin-2/cobalt-factor-2 C20-methyltransferase [EC:2.1.1. | Coenzyme transport and metabolism | Metabolism - Metabolism of cofactors and vitamins (Porphyrin metabolism) | KO only |
| K03426 | *E3.6.1.2 2* | NAD+ diphosphatase [EC:3.6.1.22] | Nucleotide transport and metabolism | Metabolism - Metabolism of cofactors and vitamins (Nicotinate and nicotinamide metabolism). Cellular Processes - Transport and catabolism (Peroxisome) | KO only |
| K03503 | *umuD* | DNA polymerase V [EC:3.4.21.-] | Transcription, Signal transduction mechanisms | BRITE only | Protein families: metabolism - Peptidases and inhibitors. Protein families: genetic information processing - DNA repair and recombination proteins. |

| | | | | | |
|---|---|---|---|---|---|
| K03529 | *smc* | chromosome segregation protein | Cell cycle control, cell division, chromosome partitioning | BRITE only | Protein families: genetic information processing - Chromosome and associated proteins. |
| K03538 | *POP4* | ribonuclease P protein subunit POP4 [EC:3.1.26.5] | Coenzyme transport and metabolism | Genetic Information Processing - Translation (Ribosome biogenesis in eukaryotes) | Protein families: genetic information processing - Ribosome biogenesis; Transfer RNA biogenesis; Mitochondrial biogenesis. |
| K03540 | *RPR2* | ribonuclease P protein subunit RPR2 [EC:3.1.26.5] | Translation, ribosomal structure and biogenesis | BRITE only | Protein families: genetic information processing - Transfer RNA biogenesis. |
| K03555 | *mutS* | DNA mismatch repair protein MutS | Nucleotide transport and metabolism | Genetic Information Processing - Replication and repair (Mismatch repair) | Protein families: genetic information processing - DNA repair and recombination proteins. |
| K03564 | *BCP* | thioredoxin-dependent peroxiredoxin [EC:1.11.1.24] | Posttranslational modification, protein turnover, chaperones | Not Included in Pathway or Brite (Unclassified: metabolism) | |
| K03574 | *mutT* | 8-oxo-dGTP diphosphatase [EC:3.6.1.55] | Defense mechanisms; Nucleotide transport and metabolism | BRITE only | Protein families: genetic information processing - DNA repair and recombination proteins. |

| | | | | | |
|---|---|---|---|---|---|
| K03581 | *recD* | exodeoxyribonuclease V alpha subunit [EC:3.1.11.5] | Replication, recombination and repair | Genetic Information Processing - Replication and repair (Homologous recombination) | Protein families: genetic information processing - DNA repair and recombination proteins. |
| K03585 | *acrA* | membrane fusion protein, multidrug efflux system | Translation, ribosomal structure and biogenesis | Human Diseases - Drug resistance: antimicrobial (beta-Lactam resistance; Cationic antimicrobial peptide (CAMP) resistance) | Protein families: genetic information processing - Chromosome and associated proteins. Protein families: signaling and cellular processes - Transporters; Antimicrobial resistance genes. |
| K03596 | *lepA* | GTP-binding protein LepA | Replication, recombination and repair | Human Diseases - Infectious disease: bacterial (Legionellosis) | KO only |
| K03601 | *xseA* | exodeoxyribonuclease VII large subunit [EC:3.1.11.6] | Replication, recombination and repair | Genetic Information Processing - Replication and repair (Mismatch repair) | Protein families: genetic information processing - DNA repair and recombination proteins. |
| K03606 | *wcaJ* | undecaprenyl-phosphate glucose phosphotransferase [EC:2.7.8.31] | Replication, recombination and repair | Metabolism - Glycan biosynthesis and metabolism (Exopolysaccharide biosynthesis). Cellular Processes - Cellular community - prokaryotes (Biofilm formation - Vibrio cholerae) | KO only |
| K03626 | *EGD2* | nascent polypeptide-associated complex subunit alpha | Transcription | Organismal Systems - Endocrine system (Parathyroid hormone synthesis, secretion and action) | KO only |

| | | | | | |
|---|---|---|---|---|---|
| K03638 | *moaB* | molybdopterin adenylyltransferase [EC:2.7.7.75] | Coenzyme transport and metabolism | Metabolism - Metabolism of cofactors and vitamins (Folate biosynthesis). Genetic Information Processing - Folding, sorting and degradation (Sulfur relay system) | KO only |
| K03685 | *rnc* | ribonuclease III [EC:3.1.26.3] | Transcription | Genetic Information Processing - Translation (Ribosome biogenesis in eukaryotes). Human Diseases - Cancer: overview (Proteoglycans in cancer) | Protein families: genetic information processing - Messenger RNA biogenesis; Ribosome biogenesis; Chromosome and associated proteins. |
| K03698 | *cbf* | 3'-5' exoribonuclease [EC:3.1.-.-] | Translation, ribosomal structure and biogenesis | BRITE only | Protein families: genetic information processing - Messenger RNA biogenesis. |
| K03702 | *uvrB* | excinuclease ABC subunit B | Replication, recombination and repair | Genetic Information Processing - Replication and repair (Nucleotide excision repair) | Protein families: genetic information processing - DNA repair and recombination proteins. |
| K03709 | *troR* | DtxR family transcriptional regulator, Mn-dependent transcriptional regulator | Transcription | BRITE only | Protein families: genetic information processing - Transcription factors. |
| K03710 | | GntR family transcriptional regulator | Transcription | BRITE only | Protein families: genetic information processing - Transcription factors. |

| | | | | | |
|---|---|---|---|---|---|
| K03723 | *mfd* | transcription-repair coupling factor (superfamily II helicase) [EC | Replication, recombination and repair, Transcription | Genetic Information Processing - Replication and repair (Nucleotide excision repair) | Protein families: genetic information processing - DNA repair and recombination proteins. |
| K03724 | *lhr* | ATP-dependent helicase Lhr and Lhr-like helicase [EC:3.6.4.13 5.6.2.4] | Replication, recombination and repair | BRITE only | Protein families: genetic information processing - DNA repair and recombination proteins. |
| K03737 | *por* | pyruvate-ferredoxin/flavodoxin oxidoreductase [EC:1.2.7.1 1.2.7.-] | Energy production and conversion | Metabolism - Carbohydrate metabolism (Glycolysis / Gluconeogenesis; Citrate cycle (TCA cycle); Pyruvate metabolism; Butanoate metabolism). Energy metabolism (Carbon fixation pathways in prokaryotes) | KO only |
| K03741 | *arsC* | arsenate reductase (thioredoxin) [EC:1.20.4.4] | Signal transduction mechanisms | Not Included in Pathway or Brite (Unclassified: metabolism) | |
| K03753 | *mobB* | molybdopterin-guanine dinucleotide biosynthesis adapter protein | Coenzyme transport and metabolism | Not Included in Pathway or Brite (Unclassified: metabolism) | |
| K03778 | *ldhA* | D-lactate dehydrogenase [EC:1.1.1.28] | Energy production and conversion, Coenzyme transport and metabolism, General function prediction only | Metabolism - Carbohydrate metabolism (Pyruvate metabolism) | KO only |

| | | | | | |
|---|---|---|---|---|---|
| K03798 | *ftsH* | cell division protease FtsH [EC:3.4.24.-] | Posttranslational modification, protein turnover, chaperones | BRITE only | Protein families: metabolism - Peptidases and inhibitors. Protein families: genetic information processing - Chaperones and folding catalysts. |
| K03803 | *rseC* | sigma-E factor negative regulatory protein RseC | Signal transduction mechanisms | BRITE only | Protein families: genetic information processing - Transcription machinery. |
| K03816 | *xpt* | xanthine phosphoribosyltransferase [EC:2.4.2.22] | Nucleotide transport and metabolism | Metabolism - Nucleotide metabolism (Purine metabolism) | KO only |
| K03820 | *lnt* | apolipoprotein N-acyltransferase [EC:2.3.1.269] | Cell wall/membrane/envelope biogenesis | Not Included in Pathway or Brite (Unclassified: metabolism) | |
| K03855 | *fixX* | ferredoxin like protein | Energy production and conversion | Not Included in Pathway or Brite (Unclassified: metabolism) | |
| K03924 | *moxR* | MoxR-like ATPase [EC:3.6.3.-] | MoxR-like ATPase [General function prediction only] | Not Included in Pathway or Brite (Unclassified: metabolism) | |
| K03972 | *pspE* | phage shock protein E | Inorganic ion transport and metabolism | Not Included in Pathway or Brite (Unclassified: genetic information processing) | |

| | | | | | |
|---|---|---|---|---|---|
| K04043 | *dnaK* | molecular chaperone DnaK | Posttranslational modification, protein turnover, chaperones | Genetic Information Processing - Folding, sorting and degradation (RNA degradation). Organismal Systems - Aging (Longevity regulating pathway - worm). Human Diseases - Infectious disease: bacterial (Tuberculosis) | Protein families: genetic information processing - Messenger RNA biogenesis; Chaperones and folding catalysts; Mitochondrial biogenesis. Protein families: signaling and cellular processes - Exosome. |
| K04047 | *dps* | starvation-inducible DNA-binding protein | Inorganic ion transport and metabolism, Defense mechanisms | BRITE only | Protein families: genetic information processing - Chromosome and associated proteins. |
| K04062 | *osmB* | osmotically inducible lipoprotein OsmB | | Not Included in Pathway or Brite (Unclassified: signaling and cellular processes) | |
| K04070 | *pflX* | putative pyruvate formate lyase activating enzyme [EC:1.97.1.4] | Uncharacterized Fe-S protein PflX, radical SAM superfamily [General function prediction only] | Not Included in Pathway or Brite (Unclassified: metabolism) | |
| K04085 | *tusA* | tRNA 2-thiouridine synthesizing protein A [EC:2.8.1.-] | Posttranslational modification, protein turnover, chaperones | Genetic Information Processing - Folding, sorting and degradation (Sulfur relay system) | Protein families: genetic information processing - Transfer RNA biogenesis. |
| K04518 | *pheA2* | prephenate dehydratase [EC:4.2.1.51] | Amino acid transport and metabolism | Metabolism - Amino acid metabolism (Phenylalanine, tyrosine and tryptophan biosynthesis) | KO only |

| | | | | | |
|---|---|---|---|---|---|
| K04564 | *SOD2* | superoxide dismutase, Fe-Mn family [EC:1.15.1.1] | Inorganic ion transport and metabolism | Environmental Information Processing - Signal transduction (MAPK signaling pathway - fly; FoxO signaling pathway). Cellular Processes - Transport and catabolism (Peroxisome). Organismal Systems - Aging (Longevity regulating pathway; Longevity regulating pathway - worm; Longevity regulating pathway - multiple species). Human Diseases - Cancer: overview (Chemical carcinogenesis - reactive oxygen species), Neurodegenerative disease (Huntington disease), Cardiovascular disease (Lipid and atherosclerosis) | KO only |
| K04751 | *glnB* | nitrogen regulatory protein P-II 1 | Signal transduction mechanisms, Amino acid transport and metabolism | Environmental Information Processing - Signal transduction (Two-component system) | KO only |
| K04761 | *oxyR* | LysR family transcriptional regulator, hydrogen peroxide-inducibl | Transcription | Cellular Processes - Cellular community - prokaryotes (Biofilm formation - Escherichia coli) | Protein families: genetic information processing - Transcription factors. |
| K04762 | *hslR* | ribosome-associated heat shock protein Hsp15 | Translation, ribosomal structure and biogenesis | BRITE only | Protein families: genetic information processing - Chaperones and folding catalysts. |
| K04796 | *RUXX* | small nuclear ribonucleoprotein | Transcription | Not Included in Pathway or Brite (Unclassified: genetic information processing) | |
| K04798 | *pfdB* | prefoldin beta subunit | Posttranslational modification, protein turnover, chaperones | BRITE only | Protein families: genetic information processing - Chaperones and folding catalysts. |

| | | | | | |
|---|---|---|---|---|---|
| K05305 | *FUK* | fucokinase [EC:2.7.1.52] | | Metabolism - Carbohydrate metabolism (Fructose and mannose metabolism; Amino sugar and nucleotide sugar metabolism) | KO only |
| K05337 | *fer* | ferredoxin | Energy production and conversion | Not Included in Pathway or Brite (Unclassified: metabolism) | |
| K05595 | *marC* | multiple antibiotic resistance protein | Amino acid transport and metabolism | BRITE only | Protein families: signaling and cellular processes - Transporters. |
| K05801 | *djlA* | DnaJ like chaperone protein | Posttranslational modification, protein turnover, chaperones | BRITE only | Protein families: genetic information processing - Chaperones and folding catalysts. |
| K05832 | | putative tryptophan/tyrosine transport system permease protein | ABC-type uncharacterized transport system, permease component [General function prediction only] | BRITE only | Protein families: signaling and cellular processes - Transporters. |
| K05833 | | putative tryptophan/tyrosine transport system ATP-binding protein | ABC-type uncharacterized transport system, ATPase component [General function prediction only] | BRITE only | Protein families: signaling and cellular processes - Transporters. |
| K05919 | *dfx* | superoxide reductase [EC:1.15.1.2] | Energy production and conversion | Not Included in Pathway or Brite (Unclassified: metabolism) | |
| K05937 | | uncharacterized protein | Uncharacterized conserved protein YdhG, YjbR/CyaY-like superfamily, DUF1801 family [Function unknown] | Not Included in Pathway or Brite (Poorly characterized) | |
| K06020 | *ettA* | energy-dependent translational throttle protein EttA | General function prediction only | BRITE only | Protein families: genetic information processing - Transfer RNA biogenesis. |

| | | | | | |
|---|---|---|---|---|---|
| K06077 | *slyB* | outer membrane lipoprotein SlyB | Cell wall/membrane/envelope biogenesis | Not Included in Pathway or Brite (Unclassified: signaling and cellular processes) | |
| K06177 | *rluA* | tRNA pseudouridine32 synthase / 23S rRNA pseudouridine746 synthase [EC:5.4.99.28 5.4.99.29] | Translation, ribosomal structure and biogenesis | BRITE only | Protein families: genetic information processing - Ribosome biogenesis; Transfer RNA biogenesis. |
| K06204 | *dksA* | DnaK suppressor protein | Translation, ribosomal structure and biogenesis | Cellular Processes - Cellular community - prokaryotes (Biofilm formation - Escherichia coli) | Protein families: genetic information processing - Transcription factors; Transcription machinery; Ribosome biogenesis. |
| K06207 | *typA* | GTP-binding protein | Signal transduction mechanisms | Not Included in Pathway or Brite (Unclassified: signaling and cellular processes) | |
| K06213 | *mgtE* | magnesium transporter | Inorganic ion transport and metabolism | BRITE only | Protein families: signaling and cellular processes - Transporters. |
| K06400 | *spoIVCA* | site-specific DNA recombinase | Replication, recombination and repair | Not Included in Pathway or Brite (Unclassified: signaling and cellular processes) | |
| K06413 | *spoVK* | stage V sporulation protein K | Cell wall/membrane/envelope biogenesis, Cell cycle control, cell division, chromosome partitioning, Signal transduction mechanisms | Not Included in Pathway or Brite (Unclassified: signaling and cellular processes) | |
| K06416 | *spoVS* | stage V sporulation protein S | Stage V sporulation protein SpoVS (function unknown) [Function unknown] | Not Included in Pathway or Brite (Unclassified: signaling and cellular processes) | |

| | | | | | |
|---|---|---|---|---|---|
| K06867 | | uncharacterized protein | Signal transduction mechanisms | Not Included in Pathway or Brite (Poorly characterized) | |
| K06874 | | zinc finger protein | C4-type Zn-finger protein [General function prediction only] | Not Included in Pathway or Brite (Poorly characterized) | |
| K06875 | *PDCD5* | programmed cell death protein 5 | General function prediction only | Not Included in Pathway or Brite (Unclassified: signaling and cellular processes) | |
| K06885 | | uncharacterized protein | HD superfamily phosphohydrolase [General function prediction only] | Not Included in Pathway or Brite (Poorly characterized) | |
| K06959 | *tex* | protein Tex | Transcription | Not Included in Pathway or Brite (Unclassified: genetic information | |
| K06961 | *KRR1* | ribosomal RNA assembly protein | Translation, ribosomal structure and biogenesis | BRITE only | Protein families: genetic information processing - Ribosome biogenesis. |
| K06972 | *PITRM1* | presequence protease [EC:3.4.24.-] | Posttranslational modification, protein turnover, chaperones | BRITE only | Protein families: metabolism - Peptidases and inhibitors. |
| K06973 | | uncharacterized protein | Posttranslational modification, protein turnover, chaperones | Not Included in Pathway or Brite (Poorly characterized) | |
| K06985 | | aspartyl protease family protein | Predicted aspartyl protease [General function prediction only] | Cellular Processes - Cell growth and death (Cell cycle - Caulobacter) | KO only |
| K06987 | | uncharacterized protein | Predicted deacylase [General function prediction only] | Not Included in Pathway or Brite (Poorly characterized) | |
| K06998 | *phzF* | trans-2,3-dihydro-3-hydroxyanthranilate isomerase [EC:5.3.3.17] | Predicted epimerase YddE/YHI9, PhzF superfamily [General function prediction only] | Metabolism - Biosynthesis of other secondary metabolites (Phenazine biosynthesis). Cellular Processes - Cellular community - prokaryotes (Quorum sensing) | KO only |
| K07005 | | uncharacterized protein | Defense mechanisms | Not Included in Pathway or Brite (Poorly characterized) | |

| | | | | | |
|---|---|---|---|---|---|
| K07025 | | putative hydrolase of the HAD superfamily | Coenzyme transport and metabolism | Not Included in Pathway or Brite (Poorly characterized) | |
| K07031 | *hddA* | D-glycero-alpha-D-manno-heptose-7-phosphate kinase [EC:2.7.1.168] | Predicted kinase related to galactokinase and mevalonate kinase [General function prediction only] | Metabolism - Glycan biosynthesis and metabolism (Lipopolysaccharide biosynthesis) | KO only |
| K07058 | | membrane protein | Uncharacterized membrane protein, BrkB/YihY/UPF0761 family (not an RNase) [Function unknown] | Not Included in Pathway or Brite (Poorly characterized) | |
| K07063 | | uncharacterized protein | Predicted nucleic acid-binding protein, contains PIN domain [General function prediction only] | Not Included in Pathway or Brite (Poorly characterized) | |
| K07075 | | uncharacterized protein | Predicted nucleotidyltransferase [General function prediction only] | Not Included in Pathway or Brite (Poorly characterized) | |
| K07105 | | uncharacterized protein | Transcription, Coenzyme transport and metabolism | Not Included in Pathway or Brite (Poorly characterized) | |
| K07118 | | uncharacterized protein | Putative NADH-flavin reductase [General function prediction only] | Not Included in Pathway or Brite (Poorly characterized) | |
| K07137 | | uncharacterized protein | FAD-dependent dehydrogenase [General function prediction only] | Not Included in Pathway or Brite (Poorly characterized) | |
| K07138 | | uncharacterized protein | Uncharacterized Fe-S cluster protein [Function unknown] | Not Included in Pathway or Brite (Poorly characterized) | |
| K07148 | | uncharacterized protein | Uncharacterized membrane protein YeiB [Function unknown] | Not Included in Pathway or Brite (Poorly characterized) | |

| | | | | | |
|---|---|---|---|---|---|
| K07154 | *hipA* | serine/threonine-protein kinase HipA [EC:2.7.11.1] | Signal transduction mechanisms | BRITE only | Protein families: metabolism - Protein kinases. Protein families: signaling and cellular processes - Prokaryotic defense system. |
| K07158 | | uncharacterized protein | Translation, ribosomal structure and biogenesis | Not Included in Pathway or Brite (Poorly characterized) | |
| K07165 | *fecR* | transmembrane sensor | Inorganic ion transport and metabolism, Signal transduction mechanisms | Environmental Information Processing - Signal transduction (Two-component system) | KO only |
| K07172 | *mazE* | antitoxin MazE | Signal transduction mechanisms | BRITE only | Protein families: signaling and cellular processes - Prokaryotic defense system. |
| K07221 | *oprO_P* | phosphate-selective porin OprO and OprP | Inorganic ion transport and metabolism | BRITE only | Protein families: signaling and cellular processes - Transporters. |
| K07284 | *srtA* | sortase A [EC:3.4.22.70] | Cell wall/membrane/envelope biogenesis | BRITE only | Protein families: metabolism - Peptidases and inhibitors; Peptidoglycan biosynthesis and degradation proteins. |
| K07316 | *mod* | adenine-specific DNA-methyltransferase [EC:2.1.1.72] | Replication, recombination and repair | BRITE only | Protein families: signaling and cellular processes - Prokaryotic defense system. |

| | | | | | |
|---|---|---|---|---|---|
| K07334 | *higB-1* | toxin HigB-1 | Defense mechanisms | BRITE only | Protein families: signaling and cellular processes - Prokaryotic defense system. |
| K07335 | *bmpA* | basic membrane protein A and related proteins | Cell wall/membrane/envelope biogenesis | Environmental Information Processing - Membrane transport (ABC transporters) | Protein families: signaling and cellular processes - Transporters. |
| K07387 | | metalloprotease [EC:3.4.24.-] | Posttranslational modification, protein turnover, chaperones | BRITE only | Protein families: metabolism - Peptidases and inhibitors. |
| K07402 | *xdhC* | xanthine dehydrogenase accessory factor | Posttranslational modification, protein turnover, chaperones | Not Included in Pathway or Brite (Unclassified: genetic information processing) | |
| K07450 | | putative resolvase | Mobilome: prophages, transposons | Not Included in Pathway or Brite (Unclassified: genetic information processing) | |
| K07458 | *vsr* | DNA mismatch endonuclease, patch repair protein [EC:3.1.-.-] | Replication, recombination and repair | BRITE only | Protein families: genetic information processing - DNA repair and recombination proteins. |
| K07464 | *cas4* | CRISPR-associated exonuclease Cas4 [EC:3.1.12.1] | Defense mechanisms | BRITE only | Protein families: signaling and cellular processes - Prokaryotic defense system. |
| K07478 | *ycaJ* | putative ATPase | Replication, recombination and repair | Not Included in Pathway or Brite (Unclassified: genetic information processing) | |
| K07482 | | transposase | Mobilome: prophages, transposons | Not Included in Pathway or Brite (Unclassified: genetic information processing) | |
| K07484 | | transposase | Mobilome: prophages, transposons | Not Included in Pathway or Brite (Unclassified: genetic information processing) | |

| | | | | | |
|---|---|---|---|---|---|
| K07492 | | putative transposase | Mobilome: prophages, transposons | Not Included in Pathway or Brite (Unclassified: genetic information processing) | |
| K07495 | | putative transposase | Mobilome: prophages, transposons | Not Included in Pathway or Brite (Unclassified: genetic information processing) | |
| K07496 | | putative transposase | Mobilome: prophages, transposons | Not Included in Pathway or Brite (Unclassified: genetic information processing) | |
| K07502 | *yprB* | uncharacterized protein | Uncharacterized conserved protein YprB, contains RNaseH-like and TPR domains [General function prediction only] | Not Included in Pathway or Brite (Poorly characterized) | |
| K07503 | *nucS* | endonuclease [EC:3.1.-.-] | Replication, recombination and repair | Not Included in Pathway or Brite (Unclassified: metabolism) | |
| K07569 | *gar1* | RNA-binding protein | Translation, ribosomal structure and biogenesis | BRITE only | Protein families: genetic information processing - Ribosome biogenesis. |
| K07573 | *CSL4* | exosome complex component CSL4 | Translation, ribosomal structure and biogenesis | Genetic Information Processing - Folding, sorting and degradation (RNA degradation) | Protein families: genetic information processing - Messenger RNA biogenesis. |
| K07720 | *yesN* | two-component system, response regulator YesN | Signal transduction mechanisms, Transcription | Environmental Information Processing - Signal transduction (Two-component system) | Protein families: signaling and cellular processes - Two-component system. |
| K07732 | *rfk* | riboflavin kinase, archaea type [EC:2.7.1.161] | Coenzyme transport and metabolism | Metabolism - Metabolism of cofactors and vitamins (Riboflavin metabolism) | KO only |
| K07741 | *antB* | anti-repressor protein | Mobilome: prophages, transposons | Not Included in Pathway or Brite (Unclassified: genetic information processing) | |
| K07979 | *ytrA* | GntR family transcriptional regulator | Transcription | BRITE only | Protein families: genetic information processing - Transcription factors. |

| | | | | | |
|---|---|---|---|---|---|
| K08094 | *hxlB* | 6-phospho-3-hexuloisomerase [EC:5.3.1.27] | Carbohydrate transport and metabolism, Cell wall/membrane/envelope biogenesis | Metabolism - Carbohydrate metabolism (Pentose phosphate pathway), Energy metabolism (Methane metabolism) | KO only |
| K08234 | *yaeR* | glyoxylase I family protein | Secondary metabolites biosynthesis, transport and catabolism | Not Included in Pathway or Brite (Unclassified: metabolism) | |
| K08307 | *mltD* | membrane-bound lytic murein transglycosylase D [EC:4.2.2.-] | Cell wall/membrane/envelope biogenesis | BRITE only | Protein families: metabolism - Peptidoglycan biosynthesis and degradation proteins. |
| K08364 | *merP* | periplasmic mercuric ion binding protein | Inorganic ion transport and metabolism | BRITE only | Protein families: signaling and cellular processes - Transporters. |
| K08600 | *srtB* | sortase B [EC:3.4.22.71] | Cell wall/membrane/envelope biogenesis | BRITE only | Protein families: metabolism - Peptidases and inhibitors; Peptidoglycan biosynthesis and degradation proteins. |
| K08602 | *pepF* | oligoendopeptidase F [EC:3.4.24.-] | Amino acid transport and metabolism | BRITE only | Protein families: metabolism - Peptidases and inhibitors. |
| K08963 | *mtnA* | methylthioribose-1-phosphate isomerase [EC:5.3.1.23] | | Metabolism - Amino acid metabolism (Cysteine and methionine metabolism) | KO only |
| K08986 | *ycgQ* | putative membrane protein | Uncharacterized membrane protein YcgQ, UPF0703/DUF1980 family [Function unknown] | Not Included in Pathway or Brite (Poorly characterized) | |

| | | | | | |
|---|---|---|---|---|---|
| K08999 | | uncharacterized protein | Bifunctional DNase/RNase [General function prediction only] | Not Included in Pathway or Brite (Poorly characterized) | |
| K09116 | | damage-control phosphatase, subfamily I [EC:3.1.3.-] | Uncharacterized conserved protein, contains ATP-grasp and redox domains [Function unknown] | Not Included in Pathway or Brite (Unclassified: metabolism) | |
| K09131 | | uncharacterized protein | Uncharacterized conserved protein YggU, UPF0235/DUF167 family [Function unknown] | Not Included in Pathway or Brite (Poorly characterized) | |
| K09157 | | uncharacterized protein | Cell cycle control, cell division, chromosome partitioning | Not Included in Pathway or Brite (Poorly characterized) | |
| K09691 | *wzt* | lipopolysaccharide transport system ATP-binding protein | Carbohydrate transport and metabolism, Cell wall/membrane/envelope biogenesis | Environmental Information Processing - Membrane transport (ABC transporters) | Protein families: signaling and cellular processes - Transporters. |
| K09705 | | uncharacterized protein | Predicted sugar epimerase, cupin superfamily [General function prediction only] | Not Included in Pathway or Brite (Poorly characterized) | |
| K09739 | *mptD* | dihydroneopterin aldolase [EC:4.1.2.25] | Uncharacterized protein [Function unknown] | Metabolism - Metabolism of cofactors and vitamins (Folate biosynthesis) | KO only |
| K09764 | | uncharacterized protein | Uncharacterized conserved protein YlxP, DUF503 family [Function unknown] | Not Included in Pathway or Brite (Poorly characterized) | |
| K09774 | *lptA* | lipopolysaccharide export system protein LptA | Cell wall/membrane/envelope biogenesis | BRITE only | Protein families: signaling and cellular processes - Transporters. |
| K09780 | | uncharacterized protein | Uncharacterized conserved protein YciI, contains a putative active-site phosphohistidine [General function prediction only] | Not Included in Pathway or Brite (Poorly characterized) | |

| | | | | | |
|---|---|---|---|---|---|
| K09793 | | uncharacterized protein | Uncharacterized conserved protein YqgC, DUF456 family [Function unknown] | Not Included in Pathway or Brite (Poorly characterized) | |
| K09797 | | uncharacterized protein | Uncharacterized protein [Function unknown] | Not Included in Pathway or Brite (Poorly characterized) | |
| K09816 | *znuB* | zinc transport system permease protein | Inorganic ion transport and metabolism | Environmental Information Processing - Membrane transport (ABC transporters) | Protein families: signaling and cellular processes - Transporters. |
| K09825 | *perR* | Fur family transcriptional regulator, peroxide stress response regulator | Inorganic ion transport and metabolism | BRITE only | Protein families: genetic information processing - Transcription factors. |
| K09922 | | uncharacterized protein | Uncharacterized conserved protein, DUF486 family [Function unknown] | Not Included in Pathway or Brite (Poorly characterized) | |
| K09976 | | uncharacterized protein | Uncharacterized protein YneF, UPF0154 family [Function unknown] | Not Included in Pathway or Brite (Poorly characterized) | |
| K10117 | *msmE* | raffinose/stachyose/melibiose transport system substrate-binding protein | Carbohydrate transport and metabolism | Environmental Information Processing - Membrane transport (ABC transporters) | Protein families: signaling and cellular processes - Transporters. |
| K10119 | *msmG* | raffinose/stachyose/melibiose transport system permease protein | Carbohydrate transport and metabolism | Environmental Information Processing - Membrane transport (ABC transporters) | Protein families: signaling and cellular processes - Transporters. |
| K10218 | *ligK* | 4-hydroxy-4-methyl-2-oxoglutarate aldolase [EC:4.1.3.17] | Translation, ribosomal structure and biogenesis | Metabolism - Carbohydrate metabolism (C5-Branched dibasic acid metabolism), Xenobiotics biodegradation and metabolism (Benzoate degradation) | KO only |

| | | | | | |
|---|---|---|---|---|---|
| K10563 | *mutM* | formamidopyrimidine-DNA glycosylase [EC:3.2.2.23 4.2.99.18] | Replication, recombination and repair | Genetic Information Processing - Replication and repair (Base excision repair) | Protein families: genetic information processing - DNA repair and recombination proteins. |
| K10725 | *cdc6A* | archaeal cell division control protein 6 | Replication, recombination and repair | BRITE only | Protein families: genetic information processing - DNA replication proteins. |
| K10761 | *THG1* | tRNA(His) guanylyltransferase [EC:2.7.7.79] | Translation, ribosomal structure and biogenesis | BRITE only | Protein families: genetic information processing - Transfer RNA biogenesis. |
| K11041 | *eta* | exfoliative toxin A/B | Defense mechanisms | Human Diseases - Infectious disease: bacterial (Staphylococcus aureus infection) | Protein families: signaling and cellular processes - Bacterial toxins. |
| K11068 | *hlyIII* | hemolysin III | Intracellular trafficking, secretion, and vesicular transport | BRITE only | Protein families: signaling and cellular processes - Bacterial toxins. |
| K11105 | *cvrA* | potassium/hydrogen antiporter | Energy production and conversion, Inorganic ion transport and metabolism | BRITE only | Protein families: signaling and cellular processes - Transporters. |
| K11260 | *fwdG* | 4Fe-4S ferredoxin | Energy production and conversion | Metabolism - Energy metabolism (Methane metabolism) | KO only |
| K11261 | *fwdE* | formylmethanofuran dehydrogenase subunit E [EC:1.2.7.12] | Energy production and conversion | Metabolism - Energy metabolism (Methane metabolism) | KO only |
| K11753 | *ribF* | riboflavin kinase / FMN adenylyltransferase [EC:2.7.1.26 2.7.7.2] | Coenzyme transport and metabolism | Metabolism - Metabolism of cofactors and vitamins (Riboflavin metabolism) | KO only |

| | | | | | |
|---|---|---|---|---|---|
| K12132 | *prkC* | eukaryotic-like serine/threonine-protein kinase [EC:2.7.11.1] | Signal transduction mechanisms; Cell wall/membrane/envelope biogenesis | BRITE only | Protein families: metabolism - Protein kinases. |
| K12340 | *tolC* | outer membrane protein | Cell wall/membrane/envelope biogenesis | Environmental Information Processing - Membrane transport (Bacterial secretion system), Signal transduction (Two-component system). Organismal Systems - Environmental adaptation (Plant-pathogen interaction). Human Diseases - Infectious disease: bacterial (Pertussis), Drug resistance: antimicrobial (beta-Lactam resistance, Cationic antimicrobial peptide (CAMP) resistance) | Protein families: signaling and cellular processes - Transporters; Secretion system; Antimicrobial resistance genes. |
| K12373 | *HEXA_B* | hexosaminidase [EC:3.2.1.52] | Carbohydrate transport and metabolism | Metabolism - Carbohydrate metabolism (Amino sugar and nucleotide sugar metabolism), Lipid metabolism (Sphingolipid metabolism), Glycan biosynthesis and metabolism (Various types of N-glycan biosynthesis; Glycosaminoglycan degradation; Glycosphingolipid biosynthesis - globo and isoglobo series; Glycosphingolipid biosynthesis - ganglio series; Other glycan degradation). Cellular Processes - Transport and catabolism (Lysosome) | Protein families: genetic information processing - Chaperones and folding catalysts. |
| K12452 | *ascC* | CDP-4-dehydro-6-deoxyglucose reductase, E1 [EC:1.17.1.1] | Cell wall/membrane/envelope biogenesis | Metabolism - Carbohydrate metabolism (Amino sugar and nucleotide sugar metabolism), Glycan biosynthesis and metabolism (O-Antigen nucleotide sugar biosynthesis) | KO only |
| K12950 | *ctpC* | manganese-transporting P-type ATPase C [EC:7.2.2.22] | Inorganic ion transport and metabolism | Not Included in Pathway or Brite (Unclassified: metabolism) | |

| | | | | | |
|---|---|---|---|---|---|
| K12952 | *ctpE* | cation-transporting P-type ATPase E [EC:7.2.2.-] | Inorganic ion transport and metabolism | Not Included in Pathway or Brite (Unclassified: metabolism) | |
| K12962 | *arnE* | undecaprenyl phosphate-alpha-L-ara4N flippase subunit ArnE | Carbohydrate transport and metabolism, Amino acid transport and metabolism, General function prediction only | Human Diseases - Drug resistance: antimicrobial (Cationic antimicrobial peptide (CAMP) resistance) | Protein families: signaling and cellular processes - Transporters. |
| K12984 | *waaE* | (heptosyl)LPS beta-1,4-glucosyltransferase [EC:2.4.1.-] | Cell wall/membrane/envelope biogenesis | BRITE only | Protein families: metabolism - Lipopolysaccharide biosynthesis proteins. |
| K12998 | *rgpE* | glucosyltransferase [EC:2.4.1.-] | Cell wall/membrane/envelope biogenesis | BRITE only | Protein families: metabolism - Lipopolysaccharide biosynthesis proteins. |
| K13002 | *wbyL* | glycosyltransferase [EC:2.4.1.-] | Cell wall/membrane/envelope biogenesis | BRITE only | Protein families: metabolism - Lipopolysaccharide biosynthesis proteins. |
| K13283 | *fieF* | ferrous-iron efflux pump FieF | Inorganic ion transport and metabolism | BRITE only | Protein families: signaling and cellular processes - Transporters. |
| K13985 | *NAPEPLD* | N-acyl-phosphatidylethanolamine-hydrolysing phospholipase D [EC:3.1.4.54] | | Organismal Systems - Nervous system (Retrograde endocannabinoid signaling) | KO only |
| K13993 | *HSP20* | HSP20 family protein | Posttranslational modification, protein turnover, chaperones | Genetic Information Processing - Folding, sorting and degradation (Protein processing in endoplasmic reticulum) | Protein families: genetic information processing - Chaperones and folding catalysts. |
| K14059 | *int* | integrase | Replication, recombination and repair, Mobilome: prophages, transposons | Not Included in Pathway or Brite (Unclassified: genetic information processing) | |

| | | | | | |
|---|---|---|---|---|---|
| K14095 | *ehaD* | energy-converting hydrogenase A subunit D | Energy production and conversion | Not Included in Pathway or Brite (Unclassified: metabolism) | |
| K14096 | *ehaE* | energy-converting hydrogenase A subunit E | Energy production and conversion | Not Included in Pathway or Brite (Unclassified: metabolism) | |
| K14102 | *ehaK* | energy-converting hydrogenase A subunit K | | Not Included in Pathway or Brite (Unclassified: metabolism) | |
| K14111 | *ehbB* | energy-converting hydrogenase B subunit B | Inorganic ion transport and metabolism | Not Included in Pathway or Brite (Unclassified: metabolism) | |
| K14112 | *ehbC* | energy-converting hydrogenase B subunit C | Inorganic ion transport and metabolism | Not Included in Pathway or Brite (Unclassified: metabolism) | |
| K14118 | *ehbI* | energy-converting hydrogenase B subunit I | Inorganic ion transport and metabolism | Not Included in Pathway or Brite (Unclassified: metabolism) | |
| K14122 | *ehbM* | energy-converting hydrogenase B subunit M | Energy production and conversion | Not Included in Pathway or Brite (Unclassified: metabolism) | |
| K14155 | *patB* | cysteine-S-conjugate beta-lyase [EC:4.4.1.13] | Amino acid transport and metabolism, General function prediction only | Metabolism - Amino acid metabolism (Cysteine and methionine metabolism). Metabolism of other amino acids (Selenocompound metabolism) | Protein families: metabolism - Amino acid related enzymes. |
| K14475 | *ICP* | inhibitor of cysteine peptidase | Predicted secreted protein [Function unknown] | Human Diseases - Infectious disease: parasitic (African trypanosomiasis) | KO only |
| K15051 | *endA* | DNA-entry nuclease | Nucleotide transport and metabolism | Not Included in Pathway or Brite (Unclassified: metabolism) | |
| K15635 | *apgM* | 2,3-bisphosphoglycerate-independent phosphoglycerate mutase [EC:5.4.2.12] | Carbohydrate transport and metabolism | Metabolism - Carbohydrate metabolism (Glycolysis / Gluconeogenesis), Energy metabolism (Methane metabolism), Amino acid metabolism (Glycine, serine and threonine metabolism) | KO only |
| K15876 | *nrfH* | cytochrome c nitrite reductase small subunit | Energy production and conversion | Metabolism - Energy metabolism (Nitrogen metabolism) | KO only |
| K15894 | *pseB* | UDP-N-acetylglucosamine 4,6-dehydratase/5-epimerase [EC:4.2.1.115 5.1.3.-] | Cell wall/membrane/envelope biogenesis, Posttranslational modification, protein turnover, chaperones | Metabolism - Carbohydrate metabolism (Amino sugar and nucleotide sugar metabolism), Glycan biosynthesis and metabolism (O-Antigen nucleotide sugar biosynthesis) | KO only |

| | | | | | |
|---|---|---|---|---|---|
| K16329 | *psuG* | pseudouridylate synthase [EC:4.2.1.70] | Nucleotide transport and metabolism | Metabolism - Nucleotide metabolism (Pyrimidine metabolism) | KO only |
| K16568 | *exoZ* | exopolysaccharide production protein ExoZ | Cell wall/membrane/envelope biogenesis | Metabolism - Glycan biosynthesis and metabolism (Exopolysaccharide biosynthesis) | KO only |
| K16785 | *ecfT* | energy-coupling factor transport system permease protein | Coenzyme transport and metabolism | Environmental Information Processing - Membrane transport (ABC transporters) | Protein families: signaling and cellular processes - Transporters. |
| K16787 | *ecfA2* | energy-coupling factor transport system ATP-binding protein [EC:7.-.-.-] | Inorganic ion transport and metabolism, General function prediction only | Environmental Information Processing - Membrane transport (ABC transporters) | Protein families: signaling and cellular processes - Transporters. |
| K16850 | *uxaA2* | altronate dehydratase large subunit [EC:4.2.1.7] | Carbohydrate transport and metabolism | Metabolism - Carbohydrate metabolism (Pentose and glucuronate interconversions) | KO only |
| K16898 | *addA* | ATP-dependent helicase/nuclease subunit A [EC:5.6.2.4 3.1.-.-] | Replication, recombination and repair | BRITE only | Protein families: genetic information processing - DNA repair and recombination proteins. |
| K16927 | *cbrT* | energy-coupling factor transport system substrate-specific component | | BRITE only | Protein families: signaling and cellular processes - Transporters. |
| K17248 | *pglJ* | N-acetylgalactosamine-N,N'-diacetylbacillosaminyl-diphospho-undecaprenol 4-alpha-N-acetylgalactosaminyltransferase [EC:2.4.1.291] | Cell wall/membrane/envelope biogenesis | BRITE only | Protein families: metabolism - Glycosyltransferases. |

| | | | | | |
|---|---|---|---|---|---|
| K17290 | *HTATIP2* | oxidoreductase [EC:1.1.1.-] | Uncharacterized conserved protein YbjT, contains NAD(P)-binding and DUF2867 domains [General function prediction only] | BRITE only | Protein families: signaling and cellular processes - Exosome. |
| K18908 | *mepA* | multidrug efflux pump | Defense mechanisms | BRITE only | Protein families: signaling and cellular processes - Transporters; Antimicrobial resistance genes. |
| K18928 | *lldE* | L-lactate dehydrogenase complex protein LldE | Energy production and conversion | Not Included in Pathway or Brite (Unclassified: metabolism) | |
| K19048 | *symE* | toxic protein SymE | | BRITE only | Protein families: signaling and cellular processes - Prokaryotic defense system. |
| K19117 | *csd1* | CRISPR-associated protein Csd1 | | BRITE only | Protein families: signaling and cellular processes - Prokaryotic defense system. |
| K19118 | *csd2* | CRISPR-associated protein Csd2 | Defense mechanisms | BRITE only | Protein families: signaling and cellular processes - Prokaryotic defense system. |
| K19166 | *higB* | mRNA interferase HigB [EC:3.1.-.-] | mRNA-degrading endonuclease (mRNA interferase) HigB, toxic component of the HigAB toxin-antitoxin module [Translation, ribosomal structure and biogenesis] | BRITE only | Protein families: signaling and cellular processes - Prokaryotic defense system. |

| | | | | | |
|---|---|---|---|---|---|
| K19271 | *catA* | chloramphenicol O-acetyltransferase type A [EC:2.3.1.28] | Defense mechanisms | BRITE only | Protein families: signaling and cellular processes - Antimicrobial resistance genes. |
| K19304 | *mepM* | murein DD-endopeptidase [EC:3.4.24.-] | Defense mechanisms | BRITE only | Protein families: metabolism - Peptidases and inhibitors; Peptidoglycan biosynthesis and degradation proteins. |
| K19689 | *ampS* | aminopeptidase [EC:3.4.11.-] | Amino acid transport and metabolism | BRITE only | Protein families: metabolism - Peptidases and inhibitors. |
| K19755 | *RSPH1* | radial spoke head protein 1 | | BRITE only | Protein families: signaling and cellular processes - Cilium and associated proteins; Cytoskeleton proteins. |
| K20608 | *tet* | tetrahedral aminopeptidase [EC:3.4.11.-] | Amino acid transport and metabolism, Carbohydrate transport and metabolism | BRITE only | Protein families: metabolism - Peptidases and inhibitors. |
| K20742 | *ykfC* | gamma-D-glutamyl-L-lysine dipeptidyl-peptidase [EC:3.4.14.13] | Cell wall/membrane/envelope biogenesis | BRITE only | Protein families: metabolism - Peptidases and inhibitors. |
| K20866 | *yihX* | glucose-1-phosphatase [EC:3.1.3.10] | Coenzyme transport and metabolism | Metabolism - Carbohydrate metabolism (Glycolysis / Gluconeogenesis) | KO only |
| K21030 | *tarI* | D-ribitol-5-phosphate cytidylyltransferase [EC:2.7.7.40] | Lipid transport and metabolism | Metabolism - Carbohydrate metabolism (Pentose and glucuronate interconversions) | KO only |

| | | | | | |
|---|---|---|---|---|---|
| K21071 | *pfk* | ATP-dependent phosphofructokinase / diphosphate-dependent phosphofructokinase [EC:2.7.1.11 2.7.1.90] | Carbohydrate transport and metabolism | Metabolism - Carbohydrate metabolism (Glycolysis / Gluconeogenesis; Pentose phosphate pathway; Fructose and mannose metabolism; Galactose metabolism). Energy metabolism (Methane metabolism) | KO only |
| K21498 | *higA-1* | antitoxin HigA-1 | | BRITE only | Protein families: signaling and cellular processes - Prokaryotic defense system. |
| K21571 | *susE_F* | starch-binding outer membrane protein SusE/F | | Not Included in Pathway or Brite (Unclassified: signaling and cellular processes) | |
| K21575 | *susA* | neopullulanase [EC:3.2.1.135] | Carbohydrate transport and metabolism | Not Included in Pathway or Brite (Unclassified: metabolism) | |
| K21908 | *hdeD* | membrane protein HdeD | Uncharacterized membrane protein HdeD, DUF308 family [Function unknown] | Not Included in Pathway or Brite (Unclassified: signaling and cellular processes) | |
| K22162 | *fpoF* | F420H2 dehydrogenase subunit F [EC:1.5.7.2 1.5.98.3] | Energy production and conversion | Not Included in Pathway or Brite (Unclassified: metabolism) | |
| K22452 | *tgpA* | protein-glutamine gamma-glutamyltransferase [EC:2.3.2.13] | Posttranslational modification, protein turnover, chaperones | Not Included in Pathway or Brite (Unclassified: metabolism) | |
| K23351 | *gcdC* | glutaconyl-CoA/methylmalonyl-CoA decarboxylase subunit gamma | Coenzyme transport and metabolism, Lipid transport and metabolism | Metabolism - Carbohydrate metabolism (Butanoate metabolism), Xenobiotics biodegradation and metabolism (Benzoate degradation) | Protein families: signaling and cellular processes - Transporters. |
| K23393 | *murT* | lipid II isoglutaminyl synthase (glutamine-hydrolysing) [EC:6.3.5.13] | Cell wall/membrane/envelope biogenesis | Metabolism - Glycan biosynthesis and metabolism (Peptidoglycan biosynthesis) | KO only |
| K23535 | *nupB* | general nucleoside transport system permease protein | ABC-type uncharacterized transport system, permease component [General function prediction only] | Environmental Information Processing - Membrane transport (ABC transporters) | Protein families: signaling and cellular processes - Transporters. |

| | | | | | |
|---|---|---|---|---|---|
| K23536 | *nupC* | general nucleoside transport system permease protein | ABC-type uncharacterized transport system, permease component [General function prediction only] | Environmental Information Processing - Membrane transport (ABC transporters) | Protein families: signaling and cellular processes - Transporters. |
| K23675 | *fmnP* | riboflavin transporter | Coenzyme transport and metabolism | BRITE only | Protein families: signaling and cellular processes - Transporters. |
| K23876 | *hgdC* | (R)-2-hydroxyacyl-CoA dehydratese activating ATPase [EC:5.6.1.9] | | Not Included in Pathway or Brite (Unclassified: metabolism) | |
| K23997 | *nnr* | ADP-dependent NAD(P)H-hydrate dehydratase / NAD(P)H-hydrate epimerase [EC:4.2.1.136 5.1.99.6] | Nucleotide transport and metabolism | Not Included in Pathway or Brite (Unclassified: metabolism) | |
| K24131 | *prsW* | protease PrsW [EC:3.4.-.-] | Signal transduction mechanisms | BRITE only | Protein families: metabolism - Peptidases and inhibitors. |

**Supplementary table 4 - A. Proportion of variation of the host traits explained by abundances of previously identified biomarkers generated from each timepoint during the growing-finishing phase**

| Trait | Biomarkers previously identified in: | Number of explanatory variables | T1 | T2 | T3 | T4 | T5 | T6 | Average ± SD |
|---|---|---|---|---|---|---|---|---|---|
| FCR | Lima (2019) | 14 genes[1] | 39.62 | 29.7 | 39.16 | 72.21 | 61.58 | 30.71 | 45.5 ± 17.41 |
| | Roehe (2016) | 40 genes[2] | 64.46 | 53.71 | 51.97 | 63.91 | 62.49 | 38.07 | 55.77 ± 10.19 |
| ADG | Lima (2019) | 13 genes[3] | 43.45 | 34.23 | 53.91 | 40.19 | 59.66 | 37.29 | 44.79 ± 9.95 |
| DFI | Lima (2019) | 14 genes[4] | 47.21 | 23.13 | 46.08 | 26.93 | 34.16 | 27.3 | 34.13 ± 10.33 |
| RFI | Lima (2019) | 9 genes[5] | 38.83 | 31.51 | 42.36 | 30.3 | 69.04 | 23.39 | 39.24 ± 16.06 |
| $CH_4$ yield | Martínez-Álvaro (2020) | 15 genera[6] | 58.03 | 69.04 | 52.31 | 27.3 | 24.77 | 17.37 | 41.47 ± 21.03 |
| | | 14 genes[7] | 46.32 | 50.25 | 51.03 | 27.45 | 54.52 | 69.47 | 49.84 ± 13.58 |
| | Roehe (2016) | 4 genes[8] | 14.95 | 50.12 | 27.88 | 22.82 | 4.78 | 33.11 | 25.61 ± 15.60 |

FCR, ADG, DFI, RFI, and $CH_4$ yield refer to feed conversion ratio, average daily weight gain, daily feed intake, residual feed intake, and methane yield (g/Kg DMI) respectively, previously corrected by the animals' weights at the start of the trial and basal diet. The percent variation of the trait was obtained from Partial Least Squares models calculated with 2 components and using centred and additive logratio transformed abundances of microbial genera and genes previously identified as biomarkers of these traits, respectively. SD refers to standard deviation. Lima (2019), Roehe (2016) and Martínez-Álvaro (2020) refer to Lima J, Auffret MD, Stewart RD, et al. Identification of rumen microbial genes involved in pathways linked to appetite, growth, and feed conversion efficiency in cattle. Front Genet. 2019;10:701(August), Roehe R, Dewhurst RJ, Duthie CA, et al. Bovine host genetic variation influences rumen microbial methane production with best selection criterion for low methane emitting and efficiently feed converting hosts based on metagenomic gene abundance. PLoS Genet. 2016;12(2):e1005846. doi:10.1371/journal.pgen.1005846, and Martínez-Álvaro M, Auffret MD, Stewart RD, et al. Identification of complex rumen microbiome interaction within diverse functional niches as mechanisms affecting the variation of methane emissions in bovine. Front Microbiol. 2020;11(April):1-13. doi:10.3389/fmicb.2020.00659, respectively.

[1]K01759, K03303, K02600, K08138, K00046, K00849, K02437, K02518, K03783, K03530, K00677, K01925, K01195, K01235

[2]K01924, K03581, K03657, K02313, K01876, K03501, K02343, K02907, K04517, K01814, K08602, K07588, K00941, K01129, K03631, K07139, K01358, K00179, K00868, K02377, K01613, K03111, K11752, K09811, K01928, K01784, K03615, K00075, K00278, K06921, K00766, K01104, K01493, K00974, K03426, K00773, K02008, K07082, K01818, K01195

[3]K02437, K01912, K02919, K02113, K00283, K02600, K02518, K01448, K00133, K03775, K03530, K02879, K01925

[4]K12340, K03043, K14113, K04751, K03210, K03685, K05515, K01709, K02428, K02428, K01858, K05349, K06178, K03625, K00978

[5]K01534, K07258, K07301, K14113, K00595, K02009, K04720, K01709, K00978

[6] *Pochonia, Tremella, Niastella, Bacillus, Fomitiporia, Selenomonas, Tolumonas, Leclercia, Moraxella, Fibrobacter, Prevotella, Butyrivibrio, Salinibacter, Alloactinosynnema, Sediminispirochaeta*

[7]K00016, K03564, K00651, K01998, K09702, K00091, K01181, K02585, K00639, K02600, K05989, K06950, K00873, K02049

[8]K00441, K00125, K00170, K00169

**Supplementary table 4 - B. Importance of microbial genera and microbial genes previously identified biomarkers measured in each timepoint for prediction of host traits.**

**Microbial genera previously identified in Martínez-Álvaro et al. (2020) et al. for the prediction of methane yield based on slaughter samples-derived data are highly associated to methane yield in all timepoints in the present work.**

| | VIP | | | | | | Regression coefficients | | | | | |
|---|---|---|---|---|---|---|---|---|---|---|---|---|
| Microbial genus | T1 | T2 | T3 | T4 | T5 | T6 | T1 | T2 | T3 | T4 | T5 | T6 |
| *Pochonia* STABLE | 0.90* | 0.97* | 1.17* | 1.16* | 1.26* | 1.24* | 7.43E-04 | 2.38E-03 | 2.50E-03 | 2.22E-03 | 3.26E-03 | 3.20E-03 |
| *Fibrobacter* | 0.80* | 0.87* | 1.06* | 1.00* | 0.97* | 1.35* | -1.01E-03 | -6.14E-04 | 3.16E-03 | 1.98E-03 | 2.05E-03 | 2.39E-03 |
| *Selenomonas* STABLE | 0.36 | 1.21* | 1.65* | 1.53* | 1.47* | 1.02* | -1.52E-03 | -4.97E-03 | -6.78E-03 | -5.63E-03 | -7.02E-03 | -2.53E-03 |
| *Tolumonas* STABLE | 0.81* | 1.12* | 1.00* | 0.94* | 1.14* | 1.19* | -8.00E-04 | -2.44E-03 | -7.30E-04 | -6.60E-04 | -2.67E-03 | -2.28E-03 |
| *Fomitiporia* | 1.24* | 1.13* | 1.25* | 1.07* | 1.32* | 0.97* | -2.69E-03 | -9.70E-04 | 2.58E-03 | -5.17E-04 | 3.58E-03 | 1.43E-03 |
| *Leclercia* | 0.74 | 1.11* | 1.14* | 1.03* | 1.11* | 1.34* | -2.13E-03 | -2.71E-03 | -1.87E-03 | -4.10E-05 | 2.95E-05 | -2.24E-03 |
| *Moraxella* STABLE | 0.85* | 1.12* | 1.00* | 0.84* | 0.89* | 1.27* | -2.22E-03 | -2.59E-03 | -1.05E-03 | -4.82E-04 | -3.09E-03 | -2.85E-03 |
| *Tremella* | 1.14* | 0.63 | 0.97* | 0.88* | 1.01* | 1.21* | 3.88E-03 | -7.26E-04 | 1.07E-03 | 2.06E-03 | 5.75E-04 | 3.56E-03 |
| *Bacillus* | 0.85* | 1.13* | 1.13* | 0.95* | 1.12* | 0.31 | 1.46E-03 | 4.39E-03 | -1.95E-03 | -8.75E-04 | 3.62E-03 | 1.07E-03 |
| *Niastella* | 0.29 | 0.60 | 0.54 | 0.51 | 0.28 | 1.28* | 1.41E-03 | -1.77E-03 | -2.15E-03 | 1.41E-03 | -1.38E-03 | 4.44E-03 |
| *Alloactinosynnema* | 0.17 | 0.91* | 0.85* | 0.77 | 0.75 | 0.36 | 9.25E-05 | 3.73E-03 | -3.32E-03 | 2.86E-03 | 1.24E-03 | 4.07E-05 |
| *Prevotella* | 1.39* | 1.41* | 0.82* | 0.34 | 0.48 | 0.22 | -6.94E-03 | 5.64E-03 | 3.48E-03 | 1.30E-03 | -2.53E-03 | -6.79E-04 |
| *Butyrivibrio* | 1.08* | 0.65 | 0.60 | 0.55 | 0.23 | 0.96* | 5.23E-03 | 7.29E-04 | 1.28E-03 | -9.95E-04 | 7.44E-04 | 2.96E-03 |
| *Sediminispirochaeta* | 0.67 | 0.47 | 0.79 | 0.78 | 0.53 | 0.26 | -3.35E-03 | -1.94E-03 | -3.12E-03 | -2.76E-03 | 1.78E-03 | 9.66E-04 |
| *Salinibacter* | 1.30* | 1.85* | 0.8* | 0.45 | 0.31 | 1.20* | -5.33E-03 | 7.46E-03 | 8.47E-04 | -7.98E-05 | -6.84E-04 | 4.59E-03 |

Martínez-Álvaro et al. (2020) refers to Martínez-Álvaro M, Auffret MD, Stewart RD, et al. Identification of complex rumen microbiome interaction within diverse functional niches as mechanisms affecting the variation of methane emissions in bovine. Front Microbiol. 2020;11(April):1-13. doi:10.3389/fmicb.2020.00659. T refers to Timepoint. Variable importance in projection (VIP) and regression coefficients were obtained from partial least squares analysis performed with 2 latent components. The * denotes VIP≥0.8 and the subscript "STABLE" highlights the same signal of regression coefficient in all timepoints. *Hydrogenobacter* and *Tsuchiyaea* were removed by abundance/zeros threshold. *Candidatus azobacteroides*, *Polaromonas*, *Acidobacterium*, *Pseudobutyrivibrio*, *Saccharomonospora*, *Aphanomyces*, *Methanocaldococcus and Candidatus methanomethylophilus* were not identified in the current database.

**Microbial genes previously identified in Roehe et al. (2016) et al. for the prediction of Feed Conversion Ratio (FCR) based on slaughter samples-derived data are highly associated to FCR in all timepoints in the present work.**

| | VIP | | | | | | Regression coefficients | | | | | |
|---|---|---|---|---|---|---|---|---|---|---|---|---|
| Microbial gene | T1 | T2 | T3 | T4 | T5 | T6 | T1 | T2 | T3 | T4 | T5 | T6 |
| K03657 | 1.35* | 1.39* | 1.09* | 0.75 | 1.18* | 1.01* | -4.88E-04 | 1.29E-03 | 1.22E-03 | 5.74E-04 | 5.15E-04 | 6.29E-04 |
| K03581 | 1.22* | 1.10* | 0.95* | 0.69 | 1.04* | 1.15* | 2.21E-03 | 1.53E-04 | -5.47E-04 | -6.54E-04 | 1.19E-03 | 1.06E-03 |
| K01876 | 1.19* | 1.42* | 1.05* | 0.86* | 0.86* | 0.87* | 1.93E-03 | 2.04E-03 | -5.25E-04 | 1.04E-03 | -1.41E-03 | 6.19E-04 |
| K02907 | 1.44* | 0.52 | 1.32* | 2.23* | 1.66* | 0.12 | 3.59E-03 | -1.06E-03 | -1.17E-03 | -3.88E-03 | -3.49E-03 | -3.71E-04 |
| $K02313_{STABLE}$ | 0.82* | 1.16* | 0.91* | 0.68 | 1.07* | 1.16* | -1.30E-03 | -5.08E-04 | -2.13E-04 | -1.25E-03 | -1.27E-03 | -2.86E-04 |
| K08602 | 1.34* | 1.18* | 0.94* | 0.57 | 0.78 | 1.16* | 4.29E-04 | 3.49E-04 | 1.07E-03 | 1.39E-05 | -3.17E-04 | 1.97E-03 |
| K03631 | 0.83* | 1.35* | 0.75 | 0.60 | 1.07* | 1.12* | 8.74E-04 | 2.08E-03 | -1.08E-03 | -1.22E-03 | 4.97E-04 | -6.72E-04 |
| K01493 | 0.54 | 0.86* | 0.87* | 0.62 | 1.17* | 0.72 | -8.30E-04 | 1.20E-03 | -7.01E-04 | -5.46E-04 | -1.56E-03 | 2.07E-03 |
| K00941 | 1.48* | 0.99* | 1.23* | 1.24* | 0.45 | 1.34* | 4.26E-03 | 6.26E-04 | -2.80E-03 | 1.72E-03 | 4.52E-04 | -1.29E-03 |
| K00766 | 0.68 | 1.35* | 0.66 | 0.37 | 1.47* | 0.89* | 1.17E-03 | 2.62E-03 | -1.36E-03 | -7.57E-04 | -3.32E-03 | -1.17E-03 |
| K07588 | 1.17* | 0.69 | 0.67 | 1.68* | 1.10* | 1.06* | -1.43E-04 | -1.09E-03 | -1.17E-03 | -2.85E-03 | -2.24E-03 | 1.81E-03 |
| K01928 | 0.65 | 1.16* | 0.89* | 0.51 | 0.73 | 1.02* | -4.63E-04 | 2.13E-03 | -1.86E-03 | 8.38E-04 | -2.64E-04 | 2.39E-03 |
| K07139 | 1.25* | 1.80* | 0.68 | 0.75 | 0.82* | 1.19* | 1.68E-03 | 3.93E-03 | -9.32E-04 | 1.02E-03 | -2.05E-03 | -9.36E-05 |
| K01129 | 1.06* | 0.80* | 0.84* | 0.94* | 0.91* | 0.97* | -1.65E-03 | 4.33E-04 | -9.99E-04 | 1.84E-03 | 1.90E-03 | 1.19E-04 |
| K01613 | 1.13* | 0.48 | 0.71 | 1.73* | 0.22 | 0.47 | -3.73E-04 | -1.16E-03 | 7.69E-04 | -2.91E-03 | 2.50E-04 | 2.32E-04 |
| K03426 | 1.01* | 1.09* | 0.75 | 0.08 | 0.66 | 0.48 | -1.53E-03 | 2.43E-03 | -1.77E-03 | -5.31E-05 | 2.91E-05 | 4.47E-04 |
| K03501 | 0.56 | 1.12* | 1.4* | 1.01* | 0.77 | 1.36* | -1.46E-03 | 8.12E-04 | -3.80E-03 | -2.06E-03 | -4.88E-04 | -8.71E-04 |
| K11752 | 0.68 | 1.43* | 0.19 | 1.21* | 0.86* | 0.15 | -3.08E-03 | -1.63E-03 | 1.09E-05 | -2.41E-03 | 1.27E-03 | 3.44E-04 |
| K00974 | 0.47 | 0.79 | 0.45 | 1.09* | 0.38 | 1.06* | 1.64E-03 | -2.69E-04 | -8.70E-04 | -2.25E-03 | -7.71E-04 | -8.91E-04 |
| K01784 | 0.66 | 1.63* | 0.51 | 0.65 | 1.11* | 1.08* | -2.30E-04 | 3.59E-03 | 4.93E-04 | -1.31E-03 | -1.62E-03 | -3.77E-04 |
| K00868 | 0.67 | 1.35* | 0.95* | 1.44* | 1.01* | 0.87* | 7.68E-04 | -1.63E-03 | -2.00E-03 | 2.73E-03 | -2.69E-03 | 1.02E-04 |
| K02377 | 0.66 | 0.22 | 1.34* | 0.9* | 0.10 | 1.86* | -2.73E-03 | 5.22E-04 | 3.48E-03 | 1.88E-03 | 2.39E-04 | 4.65E-03 |
| K01104 | 0.69 | 0.67 | 1.52* | 0.01 | 0.61 | 0.16 | 2.11E-03 | 1.45E-03 | 4.19E-03 | -9.54E-06 | -1.38E-03 | -3.79E-04 |
| K02008 | 0.58 | 0.98* | 0.47 | 0.97* | 1.41* | 0.35 | -2.61E-03 | 8.59E-04 | -1.22E-03 | -1.82E-03 | -2.38E-03 | 1.11E-03 |
| K04517 | 1.23* | 0.96* | 1.16* | 0.69 | 0.55 | 1.13* | 1.16E-03 | 1.43E-03 | 2.78E-03 | 9.83E-05 | -1.41E-03 | 3.15E-04 |
| K00075 | 0.53 | 1.57* | 0.73 | 0.87* | 0.96* | 1.17* | -1.83E-04 | 3.01E-03 | 1.09E-03 | -1.79E-03 | -1.51E-03 | -3.16E-04 |
| K03615 | 0.72 | 0.86* | 1.26* | 0.35 | 1.12* | 1.28* | 3.25E-03 | 1.88E-03 | -3.29E-03 | -2.71E-05 | -2.79E-03 | 4.13E-03 |
| K00179 | 0.98* | 0.90* | 0.51 | 1.64* | 2.24* | 0.37 | -5.62E-04 | 4.11E-04 | 1.41E-03 | -2.83E-03 | -5.57E-03 | 4.22E-04 |
| K01924 | 0.79 | 0.94* | 1.27* | 0.21 | 1.12* | 1.07* | -2.17E-03 | 1.30E-03 | -3.46E-03 | -2.94E-04 | 1.94E-03 | -8.06E-04 |
| K02343 | 0.58 | 0.97* | 0.93* | 0.41 | 0.52 | 1.36* | -2.26E-03 | 2.00E-03 | 1.67E-03 | 7.62E-04 | 1.34E-03 | 4.23E-03 |
| K01814 | 0.57 | 0.61 | 0.92* | 2.25* | 1.31* | 0.34 | -1.89E-03 | 1.40E-03 | 2.23E-03 | -4.05E-03 | -3.53E-03 | -2.50E-05 |

| | | | | | | | | | | | | |
|---|---|---|---|---|---|---|---|---|---|---|---|---|
| K06921 | 1.27* | 0.87* | 0.20 | 0.97* | 0.94* | 0.39 | 5.39E-03 | -3.18E-05 | 5.22E-04 | -1.82E-03 | 1.58E-03 | 1.16E-03 |
| K07082 | 0.39 | 0.41 | 1.24* | 1.28* | 0.90* | 1.19* | 1.38E-03 | 5.44E-05 | -3.28E-03 | -2.47E-03 | 1.24E-03 | 3.30E-03 |
| K00278 | 0.99* | 0.75 | 0.28 | 0.42 | 0.97* | 0.60 | 3.24E-03 | 2.91E-04 | 4.38E-04 | -8.04E-04 | -2.23E-03 | -1.84E-03 |
| K09811 | 1.49* | 0.92* | 0.22 | 1.06* | 0.93* | 1.06* | -6.57E-03 | 4.29E-04 | -4.55E-05 | -1.90E-03 | -2.51E-04 | 3.18E-03 |
| K01195 | 0.60 | 0.46 | 0.14 | 1.14* | 0.34 | 1.01* | 1.43E-03 | 7.82E-04 | -3.62E-04 | -2.11E-03 | -7.65E-04 | 1.32E-03 |
| K01358 | 0.73 | 0.89* | 0.49 | 1.09* | 0.49 | 1.40* | -3.03E-03 | 1.94E-03 | -9.82E-04 | -2.05E-03 | 2.02E-04 | 4.44E-03 |
| K03111 | 1.44* | 1.32* | 0.44 | 0.38 | 0.69 | 0.90* | 5.91E-03 | 2.92E-03 | -7.25E-04 | -6.12E-04 | -1.66E-03 | -4.55E-04 |
| K00773 | 0.15 | 1.15* | 0.33 | 0.81* | 0.61 | 1.21* | 1.97E-04 | -1.82E-03 | -8.14E-04 | -1.67E-03 | 9.11E-04 | -5.14E-04 |
| K01818 | 0.32 | 0.71 | 0.72 | 0.76 | 0.49 | 0.21 | -7.45E-04 | 1.60E-03 | 1.94E-03 | -1.50E-03 | -1.11E-03 | 5.55E-04 |

Roehe et al. (2016) refers to Roehe R, Dewhurst RJ, Duthie CA, et al. Bovine host genetic variation influences rumen microbial methane production with best selection criterion for low methane emitting and efficiently feed converting hosts based on metagenomic gene abundance. PLoS Genet. 2016;12(2):e1005846. doi:10.1371/journal.pgen.1005846. T refers to Timepoint. Variable importance in projection (VIP) and regression coefficients were obtained from partial least squares analysis performed with 2 latent components. The * denotes VIP≥0.8 and the subscript "STABLE" highlights the same signal of regression coefficient in all timepoints. K03694, K00956, K03458, K00394, K00375, K08483, K02006 were removed by abundance/zeros threshold. K01269 and K13542 were not identified in the current database.

**Microbial genes previously identified in Lima et al. (2019) et al. for the prediction of Feed Conversion Ratio (FCR) based on slaughter samples-derived data are highly associated to FCR in all timepoints in the present work.**

| | VIP | | | | | | Regression coefficients | | | | | |
|---|---|---|---|---|---|---|---|---|---|---|---|---|
| Microbial gene | T1 | T2 | T3 | T4 | T5 | T6 | T1 | T2 | T3 | T4 | T5 | T6 |
| K02518 | 1.02* | 0.50 | 1.50* | 1.53* | 1.16* | 0.18 | 6.39E-04 | -6.47E-04 | -2.47E-03 | -2.42E-03 | -1.27E-03 | 5.47E-04 |
| K03530 | 0.48 | 0.44 | 1.75* | 0.42 | 1.12* | 0.23 | -2.11E-03 | 1.54E-04 | -4.04E-03 | -1.19E-04 | -1.83E-03 | -4.72E-04 |
| K02437 | 1.24* | 0.73 | 0.68 | 0.85* | 0.64 | 0.81* | -3.33E-03 | -1.12E-03 | 4.89E-04 | -9.96E-04 | 4.77E-04 | 6.50E-04 |
| K01759 | 0.99* | 1.40* | 0.35 | 0.64 | 1.09* | 0.91* | 1.19E-03 | -1.89E-03 | -1.37E-04 | -1.09E-03 | -2.16E-03 | 1.69E-03 |
| K00046 | 0.64 | 0.69 | 1.27* | 1.28* | 0.97* | 1.78* | 5.34E-04 | -1.66E-03 | 3.40E-03 | 2.64E-03 | 2.35E-03 | 4.53E-03 |
| K02600 | 1.04* | 1.27* | 1.18* | 0.44 | 0.69 | 1.22* | 4.36E-03 | 2.17E-03 | 2.33E-03 | -4.63E-04 | -1.43E-03 | -2.84E-04 |
| K03303 | 1.30* | 0.63 | 1.10* | 1.85* | 1.87* | 0.95* | 4.21E-03 | -1.31E-03 | 3.02E-03 | -3.46E-03 | -4.86E-03 | -2.70E-03 |
| K08138 | 0.22 | 0.69 | 1.61* | 0.61 | 0.27 | 0.57 | -8.05E-04 | 8.81E-04 | 3.78E-03 | 1.28E-03 | -4.40E-04 | 1.25E-03 |
| K00677 | 0.67 | 0.27 | 0.36 | 1.13* | 0.31 | 0.39 | -6.73E-04 | 1.66E-04 | 1.48E-04 | -1.51E-03 | 1.69E-04 | -8.80E-05 |
| K03783 | 1.04* | 0.67 | 0.69 | 0.59 | 0.67 | 0.51 | 4.56E-03 | 1.61E-03 | 1.78E-03 | 5.21E-04 | -1.72E-03 | 1.43E-03 |
| $K01235_{STABLE}$ | 0.59 | 0.16 | 0.41 | 0.43 | 1.01* | 0.25 | -1.11E-03 | -1.20E-04 | -5.65E-04 | -5.88E-04 | -2.70E-03 | -8.73E-06 |
| K00849 | 0.48 | 0.57 | 1.53* | 0.95* | 0.77 | 0.89* | 1.28E-03 | 1.37E-03 | 3.94E-03 | 1.98E-03 | -2.07E-03 | 2.68E-03 |
| K01195 | 0.60 | 0.46 | 0.14 | 1.14* | 0.34 | 1.01* | 1.43E-03 | 7.82E-04 | -3.62E-04 | -2.11E-03 | -7.65E-04 | 1.32E-03 |
| K01925 | 0.94* | 0.66 | 0.63 | 0.62 | 0.28 | 0.93* | -4.16E-03 | 9.80E-04 | -1.20E-03 | -1.30E-03 | 7.45E-04 | -1.11E-03 |

Lima et al. (2019) refers to Lima J, Auffret MD, Stewart RD, et al. Identification of rumen microbial genes involved in pathways linked to appetite, growth, and feed conversion efficiency in cattle. Front Genet. 2019;10:701(August). doi:10.3389/fgene.2019.00701. T refers to Timepoint. Variable importance in projection (VIP) and regression coefficients were obtained from partial least squares analysis performed with 2 latent components. The * denotes VIP≥0.8 and the subscript "STABLE" highlights the same signal of regression coefficient in all timepoints. K00634 and K07561 were removed by abundance/zeros threshold. K00040, K14220, K01188 and K07214 were not identified in the current database.

**Microbial genes previously identified in Lima et al. (2019) et al. for the prediction of Average Daily Gain (ADG) based on slaughter samples-derived data are highly associated to ADG in all timepoints in the present work.**

| | VIP | | | | | | Regression coefficients | | | | | |
|---|---|---|---|---|---|---|---|---|---|---|---|---|
| Microbial gene | T1 | T2 | T3 | T4 | T5 | T6 | T1 | T2 | T3 | T4 | T5 | T6 |
| K02518 | 1.26* | 0.89* | 1.04* | 1.33* | 0.98* | 0.16 | 9.03E-04 | 2.24E-03 | 2.68E-03 | 2.27E-03 | 2.23E-03 | -9.38E-06 |
| $K03530_{STABLE}$ | 1.35* | 1.06* | 1.55* | 0.51 | 1.16* | 0.20 | 3.47E-03 | 2.86E-03 | 5.07E-03 | 7.65E-04 | 3.18E-03 | 6.25E-04 |
| K02437 | 1.51* | 0.73 | 1.27* | 1.29* | 0.47 | 0.84* | 3.46E-03 | 1.85E-04 | -8.80E-04 | 1.88E-03 | -3.52E-05 | 1.31E-03 |
| $K02919_{STABLE}$ | 1.23* | 1.00* | 2.02* | 1.72* | 1.31* | 0.49 | 8.06E-04 | 2.81E-03 | 6.61E-03 | 3.51E-03 | 3.79E-03 | 1.34E-03 |
| K01912 | 0.89* | 1.48* | 0.90* | 1.26* | 1.29* | 0.91* | -1.07E-03 | -2.41E-03 | 9.23E-04 | 1.92E-03 | 2.80E-03 | -1.61E-03 |
| K00283 | 0.88* | 1.43* | 0.92* | 0.82* | 1.08* | 0.78 | 3.04E-03 | -3.63E-03 | -2.80E-03 | -3.91E-04 | -1.29E-03 | -1.83E-03 |
| K02600 | 0.75 | 1.24* | 0.86* | 0.95* | 0.74 | 1.29* | -2.02E-03 | -2.90E-03 | -2.65E-03 | 1.58E-04 | 7.42E-06 | 1.47E-03 |
| K00133 | 0.56 | 1.43* | 1.38* | 0.96* | 1.35* | 0.88* | 1.13E-03 | -3.42E-03 | -4.51E-03 | 4.09E-04 | -3.88E-03 | 3.62E-04 |
| K02113 | 0.59 | 1.05* | 0.91* | 0.87* | 1.22* | 1.95* | 6.90E-04 | -9.91E-04 | 7.91E-04 | 1.22E-03 | 1.86E-03 | 5.51E-03 |
| K02879 | 1.30* | 1.94* | 0.32 | 0.96* | 0.89* | 0.79 | -2.43E-03 | 4.99E-03 | -2.49E-04 | 1.71E-03 | 1.25E-03 | 1.33E-03 |
| K01448 | 0.95* | 0.96* | 0.47 | 0.95* | 0.91* | 1.06* | 2.23E-03 | -2.54E-03 | -7.84E-04 | -1.79E-04 | -2.11E-03 | -1.02E-03 |
| K03775 | 1.31* | 0.73 | 1.58* | 1.27* | 1.74* | 0.65 | -4.22E-03 | -3.93E-04 | -3.08E-03 | 2.35E-03 | -4.96E-03 | -1.19E-03 |
| K01925 | 1.48* | 0.61 | 1.15* | 0.64 | 0.62 | 0.83* | 4.30E-03 | -1.43E-03 | 3.25E-03 | 1.13E-03 | -1.78E-03 | 1.21E-03 |

Lima et al. (2019) refers to Lima J, Auffret MD, Stewart RD, et al. Identification of rumen microbial genes involved in pathways linked to appetite, growth, and feed conversion efficiency in cattle. Front Genet. 2019;10:701(August). doi:10.3389/fgene.2019.00701. T refers to Timepoint. Variable importance in projection (VIP) and regression coefficients were obtained from partial least squares analysis performed with 2 latent components. The * denotes VIP≥0.8 and the subscript "STABLE" highlights the same signal of regression coefficient in all timepoints. K07561 was removed by abundance/zeros threshold.

**Microbial genes previously identified in Lima et al. (2019) et al. for the prediction of Daily Feed Intake (DFI) based on slaughter samples-derived data are highly associated to DFI in all timepoints in the present work.**

| | VIP | | | | | | Regression coefficients | | | | | |
|---|---|---|---|---|---|---|---|---|---|---|---|---|
| Microbial gene | T1 | T2 | T3 | T4 | T5 | T6 | T1 | T2 | T3 | T4 | T5 | T6 |

| | | | | | | | | | | | | |
|---|---|---|---|---|---|---|---|---|---|---|---|---|
| K03043$_{STABLE}$ | 0.53 | 1.06* | 1.22* | 1.42* | 0.91* | 1.13* | -1.48E-03 | -1.70E-04 | -1.41E-03 | -1.61E-03 | -1.44E-03 | -1.14E-03 |
| K14113 | 0.12 | 1.54* | 1.37* | 1.03* | 0.95* | 0.81* | 1.07E-04 | -1.25E-03 | 1.96E-03 | 1.17E-03 | 2.64E-03 | -1.05E-04 |
| K03685 | 0.91* | 1.09* | 0.96* | 0.99* | 1.17* | 1.18* | 1.18E-03 | 1.94E-03 | -9.09E-04 | -5.71E-04 | -1.47E-03 | 1.15E-03 |
| K03210 | 0.52 | 0.97* | 1.09* | 0.52 | 0.35 | 1.23* | -5.91E-05 | 3.62E-03 | -8.19E-05 | 8.94E-04 | 1.18E-03 | 3.46E-03 |
| K12340 | 1.01* | 1.02* | 0.98* | 1.49* | 1.46* | 0.88* | -2.86E-03 | -1.84E-03 | 4.58E-04 | 2.54E-03 | -3.04E-03 | 5.41E-05 |
| K04751 | 1.03* | 1.61* | 0.82* | 1.05* | 1.36* | 1.27* | -2.35E-03 | 4.27E-03 | 3.70E-04 | 1.32E-05 | 2.06E-03 | 2.27E-03 |
| K05515 | 0.71 | 0.64 | 0.83* | 0.92* | 1.58* | 1.09* | 4.42E-04 | 1.30E-03 | -1.86E-03 | 4.08E-04 | -4.93E-03 | -3.46E-04 |
| K00978 | 0.69 | 0.44 | 0.33 | 0.43 | 0.21 | 0.60 | -2.99E-04 | 1.12E-03 | 5.86E-04 | -2.08E-05 | 6.59E-04 | -6.68E-04 |
| K01709 | 1.25* | 0.78 | 0.30 | 0.63 | 0.59 | 0.81* | -2.71E-03 | -1.58E-03 | -1.39E-04 | -4.07E-04 | 1.95E-03 | -1.52E-03 |
| K05349 | 0.41 | 0.42 | 0.80* | 1.15* | 0.68 | 1.18* | -8.74E-04 | -1.74E-03 | 1.80E-03 | 2.22E-03 | -2.07E-03 | 2.79E-03 |
| K02428 | 1.31* | 0.68 | 0.52 | 1.03* | 0.91* | 1.06* | 1.55E-03 | -2.43E-03 | -6.67E-04 | -4.86E-04 | 2.23E-03 | 1.40E-03 |
| K01858 | 1.36* | 0.29 | 1.34* | 1.30* | 0.15 | 0.78 | 3.02E-03 | -9.41E-04 | 2.93E-03 | 2.19E-03 | 2.08E-04 | 2.02E-03 |
| K03625 | 0.77 | 0.16 | 0.34 | 0.69 | 0.88* | 1.47* | -1.89E-05 | 6.54E-04 | 7.15E-04 | 4.95E-04 | -5.55E-04 | 2.88E-03 |
| K06178 | 1.02* | 0.23 | 0.48 | 0.74 | 0.93* | 0.86* | 1.84E-03 | 6.29E-04 | -1.05E-03 | 9.09E-04 | -2.79E-03 | 1.55E-03 |

Lima et al. (2019) refers to Lima J, Auffret MD, Stewart RD, et al. Identification of rumen microbial genes involved in pathways linked to appetite, growth, and feed conversion efficiency in cattle. Front Genet. 2019;10:701(August). doi:10.3389/fgene.2019.00701. T refers to Timepoint. Variable importance in projection (VIP) and regression coefficients were obtained from partial least squares analysis performed with 2 latent components. The * denotes VIP≥0.8 and the subscript "STABLE" highlights the same signal of regression coefficient in all timepoints. K00370, K00613 and K04764 were removed by abundance/zeros threshold.

**Microbial genes previously identified in Lima et al. (2019) et al. for the prediction of Residual Feed Intake (RFI) based on slaughter samples-derived data are highly associated to RFI in all timepoints in the present work.**

| | VIP | | | | | | Regression coefficients | | | | | |
|---|---|---|---|---|---|---|---|---|---|---|---|---|
| Microbial gene | T1 | T2 | T3 | T4 | T5 | T6 | T1 | T2 | T3 | T4 | T5 | T6 |
| K01534 | 0.89* | 1.48* | 1.18* | 1.09* | 1.17* | 1.2* | 2.85E-04 | 2.42E-03 | -3.55E-04 | -5.63E-04 | -1.75E-03 | -8.76E-04 |
| K07258 | 1.38* | 1.58* | 0.88* | 1.22* | 2.01* | 1.00* | -3.59E-03 | 5.03E-03 | -4.12E-04 | -1.15E-03 | -5.41E-03 | -9.08E-04 |
| K07301 | 1.03* | 0.75 | 1.12* | 1.17* | 0.53 | 0.84* | 1.88E-03 | -8.88E-04 | 6.26E-04 | 2.01E-03 | -8.18E-04 | 1.39E-03 |
| K14113 | 1.01* | 1.35* | 1.26* | 0.88* | 0.99* | 0.92* | 1.46E-03 | 8.27E-05 | 1.58E-03 | 2.72E-04 | -1.41E-03 | -1.96E-04 |
| K00595 | 1.42* | 0.57 | 1.08* | 0.78 | 0.82* | 1.83* | -4.22E-03 | -1.51E-03 | 1.97E-03 | -1.57E-03 | 1.55E-03 | -4.76E-03 |
| K02009 | 0.95* | 1.21* | 1.33* | 0.83* | 0.88* | 0.93* | 2.23E-03 | 2.29E-03 | 2.18E-03 | 4.47E-04 | 5.95E-04 | -7.32E-04 |
| K04720 | 0.68 | 0.91* | 1.02* | 1.63* | 0.75 | 1.01* | -4.55E-04 | 3.85E-04 | -1.92E-04 | -3.48E-03 | 1.49E-03 | -1.93E-03 |
| K01709$_{STABLE}$ | 0.83* | 0.57 | 0.27 | 1.41* | 1.36* | 1.64* | -2.61E-03 | -1.77E-03 | -6.27E-05 | -2.80E-03 | -3.22E-03 | -4.94E-03 |
| K00978$_{STABLE}$ | 0.47 | 0.19 | 0.27 | 1.32* | 1.17* | 1.65* | -1.46E-03 | -6.22E-04 | -3.44E-04 | -2.76E-03 | -3.01E-03 | -5.03E-03 |

Lima et al. (2019) refers to Lima J, Auffret MD, Stewart RD, et al. Identification of rumen microbial genes involved in pathways linked to appetite, growth, and feed conversion efficiency in cattle. Front Genet. 2019;10:701(August). doi:10.3389/fgene.2019.00701. T refers to Timepoint. Variable importance in projection (VIP) and regression coefficients were obtained from partial least squares analysis performed with 2 latent components. The * denotes VIP≥0.8 and the subscript "STABLE" highlights the same signal of regression coefficient in all timepoints. K03406, K03413, K03407, K01571, K02057, K02390, K02417 and K03738 were removed by abundance/zeros threshold.

**Microbial genes previously identified in Roehe et al. (2016) et al. for the prediction of Methane Yield based on slaughter samples-derived data are highly associated to Methane Yield in all timepoints in the present work.**

| | VIP | | | | | | Regression coefficients | | | | | |
|---|---|---|---|---|---|---|---|---|---|---|---|---|
| Microbial gene | T1 | T2 | T3 | T4 | T5 | T6 | T1 | T2 | T3 | T4 | T5 | T6 |
| K00441 | 0.83* | 1.26* | 0.67 | 0.87* | 0.65 | 1.61* | 2.48E-03 | 2.38E-03 | 1.11E-03 | 2.94E-03 | -1.44E-04 | 2.71E-03 |
| K00125 | 1.58* | 0.64 | 0.78 | 1.07* | 0.72 | 0.43 | -4.76E-03 | -1.86E-03 | -2.99E-04 | 2.20E-03 | -4.49E-04 | 6.23E-05 |
| K00170 | 0.55 | 0.60 | 0.61 | 1.24* | 0.68 | 0.65 | 1.28E-03 | 1.15E-03 | -7.36E-04 | -3.85E-03 | 8.19E-04 | 1.16E-03 |
| K00169 | 0.30 | 0.59 | 0.46 | 0.79 | 0.46 | 0.19 | -7.45E-04 | -1.10E-03 | -2.69E-04 | -2.69E-03 | 4.24E-04 | 3.31E-04 |

Roehe et al. (2016) refers to Roehe R, Dewhurst RJ, Duthie CA, et al. Bovine host genetic variation influences rumen microbial methane production with best selection criterion for low methane emitting and efficiently feed converting hosts based on metagenomic gene abundance. PLoS Genet. 2016;12(2):e1005846. doi:10.1371/journal.pgen.1005846. T refers to Timepoint. Variable importance in projection (VIP) and regression coefficients were obtained from partial least squares analysis performed with 2 latent components. The * denotes VIP≥0.8 and the subscript "STABLE" highlights the same signal of regression coefficient in all timepoints. K00400, K00577, K13812, K00672, K14123, K00205, K01499K00200, K00580, K00203, K00584, K00581, K00123, K14128, K00399 and K00201 were removed by abundance/zeros threshold.

**Microbial genes previously identified in Martínez-Álvaro et al. (2020) et al. for the prediction of Methane Yield based on slaughter samples-derived data are highly associated to Methane Yield in all timepoints in the present work.**

| | VIP | | | | | | Regression coefficients | | | | | |
|---|---|---|---|---|---|---|---|---|---|---|---|---|
| Microbial gene | T1 | T2 | T3 | T4 | T5 | T6 | T1 | T2 | T3 | T4 | T5 | T6 |
| K00016 | 1.02* | 1.20* | 1.24* | 1.08* | 0.87* | 1.38* | -2.75E-03 | -9.10E-04 | -5.32E-04 | -4.52E-04 | 9.95E-04 | -1.78E-03 |
| K03564 | 0.84* | 1.09* | 1.14* | 1.46* | 0.85* | 1.14* | -1.53E-03 | -4.48E-04 | 6.83E-05 | 4.22E-03 | 9.42E-04 | 8.63E-04 |
| K00651 | 0.43 | 1.16* | 1.27* | 1.13* | 1.34* | 1.24* | -2.15E-04 | 1.77E-03 | -1.87E-03 | -1.14E-03 | -8.80E-04 | -1.43E-03 |
| K01998 | 0.69 | 1.10* | 1.48* | 1.15* | 1.22* | 1.08* | -2.02E-03 | -2.06E-03 | -2.14E-03 | -1.46E-03 | -9.75E-04 | 4.93E-04 |
| K09702 | 0.65 | 0.92* | 0.62 | 0.36 | 0.87* | 0.92* | -1.89E-03 | -2.78E-03 | 1.27E-03 | -1.15E-03 | -1.04E-03 | -1.27E-03 |
| K00091 | 0.59 | 1.16* | 0.72 | 0.85* | 1.20* | 0.73 | -1.06E-03 | 1.30E-03 | -3.27E-04 | 1.51E-03 | 2.35E-03 | 1.02E-03 |
| K01181 | 0.42 | 1.05* | 0.94* | 1.69* | 0.26 | 0.39 | -2.18E-05 | 1.84E-03 | -1.26E-04 | 5.59E-03 | 4.08E-04 | 7.17E-04 |
| K02585 | 0.19 | 0.89* | 0.62 | 0.62 | 0.82* | 0.96* | -2.06E-04 | 8.63E-04 | -8.66E-04 | -6.77E-04 | -1.44E-03 | 1.30E-03 |
| K00639 | 0.41 | 1.03* | 0.58 | 0.50 | 1.42* | 0.42 | -1.25E-03 | -1.28E-03 | -2.19E-04 | -1.25E-03 | -2.92E-03 | 7.59E-04 |

| | | | | | | | | | | | | |
|---|---|---|---|---|---|---|---|---|---|---|---|---|
| K02600 | 0.14 | 0.32 | 1.42* | 1.17* | 1.22* | 0.85* | 1.50E-04 | 6.73E-04 | -2.81E-03 | -8.38E-04 | -2.00E-03 | 9.82E-04 |
| K05989 | 0.81* | 0.59 | 1.04* | 0.84* | 1.20* | 1.18* | -1.96E-03 | 1.72E-03 | -1.77E-03 | 6.28E-04 | -8.62E-04 | 1.93E-03 |
| K06950 | 0.89* | 0.82* | 0.95* | 1.13* | 0.50 | 0.87* | 1.88E-03 | 2.15E-05 | -1.74E-03 | 3.69E-03 | 1.08E-03 | 1.45E-03 |
| K00873 | 0.72 | 0.78 | 1.31* | 0.80* | 0.84* | 1.29* | -2.13E-03 | -1.89E-04 | -2.31E-03 | 3.42E-04 | 9.31E-04 | -9.87E-04 |
| K02049 | 0.71 | 0.73 | 0.25 | 0.66 | 0.24 | 0.47 | -1.78E-03 | 1.59E-03 | -5.50E-04 | -1.26E-03 | 1.87E-04 | 2.81E-04 |

Martínez-Álvaro et al. (2020) refers to Martínez-Álvaro M, Auffret MD, Stewart RD, et al. Identification of complex rumen microbiome interaction within diverse functional niches as mechanisms affecting The * denotes VIP≥0.8 and the subscript "STABLE" highlights the same signal of regression coefficient in all timepoints. K00164, K00281, K01995, K02588, K03735, K05350, K10108, K10110 and K14260 were removed by abundance/zeros threshold.

**Supplementary table 5 - A. Proportion of variation of the host traits' estimated breeding values explained by abundances of 673 heritable microbial genes previously identified.**

| Trait | T1 | T2 | T3 | T4 | T5 | T6 | Average ± SD |
|---|---|---|---|---|---|---|---|
| FCR | 76.48 | 71.49 | 83.99 | 79.33 | 65.97 | 66.98 | 74.04 ± 6.51 |
| ADG | 63.92 | 69.71 | 66.62 | 68.51 | 77.11 | 61.09 | 67.83 ± 5.04 |
| DFI | 67.07 | 82.09 | 66.69 | 65.66 | 74.10 | 66.13 | 70.29 ± 6.00 |
| RFI | 67.96 | 63.41 | 53.95 | 66.89 | 82.94 | 70.16 | 67.55 ± 8.62 |
| CH4 yield | 63.55 | 58.32 | 86.88 | 65.36 | 71.77 | 64.30 | 68.36 ± 9.16 |

FCR, ADG, DFI, RFI, and CH4 yield refer to estimated breeding values of feed conversion ratio, average daily weight gain, daily feed intake, residual feed intake, and methane yield (g/Kg DMI) respectively. The percent variation of the trait was obtained from Partial Least Squares models calculated with 2 components and using centred and additive logratio transformed abundances of microbial genes previously identified as significantly heritable in Martínez-Álvaro, M. et al. Microbiome-driven breeding strategy potentially improves beef fatty acid profile benefiting human health and reduces methane emissions. Microbiome 10, 166 (2022). SD refers to standard deviation.

**Supplementary table 5 - B. Variable importance in projection (VIP) and regression coefficients from partial least squares analyses of 673 significantly heritable microbial genes predicting estimated breeding values of host traits. Subscript VIP≥0.8 denotes microbial genera that had VIP ≥ 0.8 in the prediction of estimated breeding values in 4 or more timepoints. Subscripts (+) and (-) represent microbial genera that had positive or negative association with the estimated breeding values in 4 or more timepoints, respectively.**

**Trait: Feed Conversion Ratio (FCR); Predictors: 673 heritable microbial genes**

| Microbial gene | VIP | | | | | | Regression coefficient | | | | | |
|---|---|---|---|---|---|---|---|---|---|---|---|---|
| | T1 | T2 | T3 | T4 | T5 | T6 | T1 | T2 | T3 | T4 | T5 | T6 |
| $K06894_{VIP≥0.8\ (+)}$ | 1.55 | 1.21 | 0.92 | 1.02 | 1.03 | 1.21 | 1.55E-02 | 7.59E-03 | 6.48E-03 | 7.13E-03 | 1.35E-03 | 8.66E-03 |
| $K13002_{VIP≥0.8\ (+)}$ | 1.37 | 0.82 | 1.24 | 1.15 | 1.29 | 1.68 | 8.97E-03 | 2.46E-03 | 1.32E-02 | 5.35E-03 | 7.82E-03 | 1.18E-02 |
| $K02379_{VIP≥0.8\ (+)}$ | 0.87 | 0.86 | 0.88 | 0.83 | 0.27 | 1.47 | 7.76E-03 | 6.40E-03 | 9.37E-03 | 3.58E-03 | 1.48E-03 | 1.10E-02 |
| $K23997_{VIP≥0.8\ (+)}$ | 0.82 | 1.76 | 1.31 | 0.61 | 1.08 | 1.25 | 5.21E-03 | 1.37E-02 | 7.23E-03 | 9.19E-04 | 6.28E-03 | 7.06E-03 |
| $K04744_{VIP≥0.8\ (+)}$ | 1.28 | 0.49 | 1.27 | 0.92 | 0.81 | 0.82 | 5.16E-03 | 9.51E-04 | 1.29E-02 | 4.13E-03 | 2.46E-03 | 5.12E-03 |
| $K09015_{VIP≥0.8\ (+)}$ | 0.73 | 1.69 | 0.85 | 0.88 | 1.42 | 1.08 | 9.00E-04 | 1.14E-02 | 9.60E-03 | 4.69E-03 | 8.74E-03 | 6.85E-03 |
| $K00012_{VIP≥0.8\ (+)}$ | 1.17 | 0.49 | 2.86 | 0.85 | 0.91 | 1.15 | 1.07E-02 | 3.61E-03 | 3.18E-02 | 5.86E-03 | 5.93E-03 | 8.82E-03 |
| $K02429_{VIP≥0.8\ (+)}$ | 1.14 | 1.07 | 0.75 | 1.03 | 0.98 | 0.81 | 7.30E-03 | 1.13E-03 | 7.41E-03 | 2.99E-03 | 4.58E-03 | 5.97E-03 |
| $K00549_{VIP≥0.8\ (+)}$ | 1.05 | 0.98 | 0.49 | 0.46 | 0.97 | 0.80 | 1.16E-02 | 5.80E-03 | 2.61E-03 | 3.24E-03 | 6.38E-03 | 2.98E-03 |
| $K04516_{VIP≥0.8\ (+)}$ | 0.92 | 1.06 | 1.16 | 0.58 | 0.79 | 1.85 | 2.47E-03 | 4.59E-03 | 1.15E-02 | 1.23E-03 | 3.40E-03 | 1.27E-02 |
| $K00241_{VIP≥0.8\ (+)}$ | 1.03 | 0.78 | 1.03 | 0.59 | 1.00 | 0.99 | 4.45E-03 | 8.85E-04 | 7.67E-03 | 3.39E-03 | 6.83E-04 | 5.12E-03 |
| $K07322_{VIP≥0.8\ (+)}$ | 0.73 | 1.21 | 0.74 | 1.18 | 0.89 | 1.14 | 5.54E-03 | 3.94E-03 | 8.36E-03 | 6.73E-03 | 5.93E-03 | 8.68E-03 |
| $K01803_{VIP≥0.8\ (+)}$ | 0.20 | 1.14 | 1.64 | 1.40 | 0.76 | 1.62 | 7.06E-04 | 5.82E-03 | 1.36E-02 | 7.75E-03 | 4.07E-03 | 1.01E-02 |
| $K03797_{VIP≥0.8\ (+)}$ | 0.91 | 1.17 | 0.26 | 0.74 | 0.84 | 1.31 | 5.47E-03 | 2.80E-03 | 8.66E-04 | 9.36E-04 | 1.63E-03 | 9.93E-03 |
| $K21572_{VIP≥0.8\ (+)}$ | 0.95 | 1.37 | 0.62 | 0.97 | 0.98 | 0.77 | 8.16E-03 | 4.60E-03 | 6.57E-03 | 1.61E-03 | 6.25E-03 | 5.59E-03 |
| $K02018_{VIP≥0.8\ (+)}$ | 1.08 | 1.82 | 1.37 | 0.88 | 1.61 | 1.79 | 1.63E-02 | 1.45E-02 | 4.26E-04 | 4.26E-03 | -1.07E-02 | 1.38E-02 |
| $K23876_{VIP≥0.8\ (+)}$ | 1.33 | 0.95 | 1.03 | 1.38 | 1.03 | 1.14 | 1.96E-02 | 6.46E-03 | 1.15E-03 | 9.17E-03 | -2.07E-03 | 3.55E-03 |
| $K23393_{VIP≥0.8\ (+)}$ | 1.00 | 1.19 | 1.19 | 1.16 | 0.94 | 1.11 | 3.53E-03 | 8.96E-03 | 5.20E-03 | 4.49E-03 | -1.44E-03 | 5.39E-03 |
| $K16053_{VIP≥0.8\ (+)}$ | 1.26 | 1.01 | 1.58 | 1.46 | 0.95 | 0.84 | -1.89E-02 | 3.22E-03 | 1.79E-02 | 1.03E-02 | 4.72E-03 | 3.71E-03 |
| $K11176_{VIP≥0.8\ (+)}$ | 1.03 | 1.20 | 0.90 | 0.83 | 1.35 | 0.96 | 2.05E-03 | 9.54E-03 | 1.86E-03 | 1.58E-03 | -3.48E-03 | 7.20E-04 |
| $K00620_{VIP≥0.8\ (+)}$ | 0.94 | 0.83 | 1.13 | 0.94 | 1.42 | 0.87 | 7.36E-03 | 5.43E-03 | 1.01E-02 | 2.72E-03 | -6.28E-03 | 2.88E-03 |

| | | | | | | | | | | | | |
|---|---|---|---|---|---|---|---|---|---|---|---|---|
| $K14117_{VIP≥0.8\ (+)}$ | 0.80 | 0.87 | 1.13 | 1.14 | 0.46 | 1.06 | -6.33E-03 | 5.13E-03 | 6.20E-03 | 4.03E-03 | 2.50E-03 | 7.93E-03 |
| $K07503_{VIP≥0.8\ (+)}$ | 1.80 | 0.97 | 2.30 | 1.00 | 0.53 | 1.20 | 2.54E-02 | 6.39E-03 | 2.13E-02 | -5.26E-03 | 3.60E-03 | 9.10E-03 |
| $K03785_{VIP≥0.8\ (+)}$ | 1.07 | 1.61 | 0.64 | 0.80 | 1.14 | 1.04 | 1.35E-02 | 1.27E-02 | 6.99E-03 | 5.24E-03 | 5.70E-03 | -3.06E-03 |
| $K00878_{VIP≥0.8\ (+)}$ | 1.17 | 1.05 | 0.90 | 0.67 | 0.88 | 1.21 | 1.59E-02 | 7.08E-03 | 5.53E-04 | 4.41E-03 | -2.00E-05 | 6.42E-03 |
| $K04062_{VIP≥0.8\ (+)}$ | 0.83 | 1.65 | 2.04 | 2.10 | 0.94 | 0.55 | 1.03E-02 | 1.17E-02 | 1.95E-02 | 1.49E-02 | 4.80E-03 | -2.06E-05 |
| $K01858_{VIP≥0.8\ (+)}$ | 1.70 | 0.43 | 0.83 | 1.03 | 0.95 | 2.37 | 1.80E-02 | 2.48E-03 | 9.03E-03 | 7.27E-03 | -6.37E-03 | 1.83E-02 |
| $K06024_{VIP≥0.8\ (+)}$ | 1.03 | 0.44 | 0.95 | 0.82 | 1.68 | 1.14 | 1.06E-02 | 3.07E-03 | 7.55E-03 | 3.36E-03 | -5.54E-03 | 1.40E-03 |
| $K13532_{VIP≥0.8\ (+)}$ | 1.18 | 0.48 | 0.88 | 1.02 | 0.96 | 1.12 | 5.03E-03 | -1.73E-03 | 1.01E-02 | 5.86E-03 | 5.43E-03 | 7.47E-03 |
| $K00046_{VIP≥0.8\ (+)}$ | 0.85 | 0.69 | 1.35 | 1.36 | 1.39 | 1.65 | 3.20E-03 | -7.32E-04 | 1.52E-02 | 9.09E-03 | 7.88E-03 | 1.08E-02 |
| $K01685_{VIP≥0.8\ (+)}$ | 1.53 | 1.10 | 1.75 | 0.86 | 1.04 | 0.31 | -2.28E-02 | 2.40E-03 | 1.85E-02 | 2.85E-03 | 4.99E-03 | 2.08E-03 |
| $K01854_{VIP≥0.8\ (+)}$ | 0.79 | 1.28 | 2.33 | 1.33 | 0.80 | 1.52 | -4.70E-03 | 5.08E-03 | 2.46E-02 | 9.42E-03 | 5.18E-03 | 1.11E-02 |
| $K07484_{VIP≥0.8\ (+)}$ | 1.22 | 0.44 | 0.90 | 1.37 | 1.44 | 1.02 | -5.67E-03 | 1.51E-03 | 1.02E-02 | 9.31E-03 | 6.11E-03 | 4.63E-03 |
| $K03049_{VIP≥0.8\ (+)}$ | 0.97 | 0.80 | 1.27 | 1.23 | 0.75 | 1.12 | -1.46E-02 | 6.30E-03 | 8.65E-03 | 7.08E-03 | 4.96E-03 | 8.67E-03 |
| $K07569_{VIP≥0.8\ (+)}$ | 0.69 | 1.04 | 1.31 | 0.97 | 0.57 | 1.06 | -8.36E-03 | 7.19E-03 | 1.03E-02 | 2.47E-03 | 3.58E-03 | 7.43E-03 |
| $K14118_{VIP≥0.8\ (+)}$ | 0.59 | 1.03 | 1.37 | 0.96 | 0.42 | 0.84 | -3.44E-04 | 7.55E-03 | 9.15E-03 | 2.24E-03 | 2.62E-03 | 5.86E-03 |
| $K14119_{VIP≥0.8\ (+)}$ | 0.91 | 1.19 | 1.27 | 0.86 | 0.23 | 0.76 | -1.18E-02 | 8.12E-03 | 1.01E-02 | 2.17E-03 | 1.49E-03 | 5.53E-03 |
| $K00578_{VIP≥0.8\ (+)}$ | 0.88 | 0.56 | 1.03 | 0.87 | 0.17 | 1.03 | -1.29E-02 | 3.13E-03 | 6.40E-03 | 1.69E-03 | 3.63E-04 | 7.79E-03 |
| $K17884_{VIP≥0.8\ (+)}$ | 0.21 | 1.43 | 0.88 | 2.03 | 0.45 | 1.69 | -2.15E-03 | 1.14E-02 | 8.58E-03 | 1.44E-02 | 3.01E-03 | 1.30E-02 |
| $K02009_{VIP≥0.8\ (+)}$ | 0.61 | 0.98 | 1.32 | 1.04 | 0.40 | 1.20 | -5.58E-03 | 5.95E-03 | 1.04E-02 | 7.79E-04 | 2.57E-03 | 9.16E-03 |
| $K03057_{VIP≥0.8\ (+)}$ | 0.85 | 0.71 | 0.94 | 0.80 | 0.36 | 1.11 | -1.30E-03 | 4.48E-03 | 3.02E-03 | 1.44E-03 | 2.27E-03 | 8.34E-03 |
| $K03791_{VIP≥0.8\ (+)}$ | 0.40 | 1.08 | 0.93 | 0.28 | 0.90 | 1.73 | 1.72E-03 | 7.82E-03 | 9.15E-03 | 1.96E-03 | -5.27E-03 | 1.34E-02 |
| $K03750_{VIP≥0.8\ (+)}$ | 2.09 | 0.56 | 0.59 | 1.35 | 1.36 | 1.22 | 2.63E-02 | 9.13E-04 | 6.75E-03 | 6.19E-03 | -8.76E-03 | 9.20E-03 |
| $K01095_{VIP≥0.8\ (+)}$ | 1.38 | 0.58 | 0.42 | 1.75 | 0.84 | 1.31 | 2.04E-02 | 1.22E-03 | -8.32E-04 | 1.16E-02 | 3.29E-03 | 9.79E-03 |
| $K00626_{VIP≥0.8\ (+)}$ | 1.16 | 0.99 | 0.27 | 0.95 | 0.53 | 1.21 | 4.57E-03 | 5.91E-03 | 1.27E-03 | 5.50E-03 | -1.13E-03 | 5.80E-04 |
| $K07404_{VIP≥0.8\ (+)}$ | 1.06 | 0.95 | 0.68 | 0.67 | 1.66 | 1.46 | 6.11E-03 | 4.25E-03 | -3.12E-03 | 4.65E-03 | 1.06E-02 | 6.35E-03 |
| $K16511_{VIP≥0.8\ (+)}$ | 1.15 | 1.43 | 0.30 | 0.73 | 0.90 | 1.25 | 9.22E-03 | 1.11E-02 | 3.49E-03 | 4.60E-03 | 2.64E-03 | -5.43E-03 |
| $K20866_{VIP≥0.8\ (+)}$ | 0.54 | 0.80 | 1.51 | 1.19 | 0.80 | 1.38 | 3.21E-03 | -4.56E-03 | 1.70E-02 | 8.05E-03 | 3.13E-03 | 8.14E-03 |
| $K06023_{VIP≥0.8\ (+)}$ | 1.09 | 0.62 | 0.86 | 0.54 | 1.49 | 1.00 | 1.12E-02 | 4.91E-03 | 4.66E-03 | 1.18E-03 | -6.99E-04 | 1.18E-03 |
| $K06442_{VIP≥0.8\ (+)}$ | 1.08 | 0.69 | 0.67 | 0.91 | 1.72 | 1.13 | 5.97E-03 | 4.44E-03 | 5.95E-04 | 3.37E-03 | -7.91E-03 | 3.92E-03 |
| $K07464_{VIP≥0.8\ (+)}$ | 0.85 | 1.07 | 0.32 | 0.50 | 0.96 | 1.54 | 6.37E-03 | 3.26E-03 | -3.52E-03 | 1.59E-03 | 6.44E-03 | 9.19E-03 |

| | | | | | | | | | | | | |
|---|---|---|---|---|---|---|---|---|---|---|---|---|
| $K23675_{VIP≥0.8\ (+)}$ | 1.14 | 1.00 | 0.46 | 0.72 | 1.26 | 1.27 | 1.17E-02 | 7.23E-03 | 1.70E-03 | 2.56E-03 | -7.45E-04 | 5.68E-03 |
| $K08591_{VIP≥0.8\ (+)}$ | 1.06 | 0.45 | 0.77 | 1.00 | 1.40 | 1.32 | 9.23E-03 | 3.14E-03 | 3.15E-03 | 5.15E-03 | -2.46E-03 | 4.65E-03 |
| $K01156_{VIP≥0.8\ (+)}$ | 2.31 | 1.43 | 1.85 | 0.44 | 1.79 | 0.53 | 3.36E-02 | 9.06E-03 | 1.88E-02 | 2.97E-03 | 1.18E-02 | -1.07E-04 |
| $K01515_{VIP≥0.8\ (+)}$ | 0.89 | 0.44 | 1.15 | 0.74 | 0.98 | 1.22 | 9.73E-03 | -3.25E-03 | 1.32E-02 | 4.12E-03 | 1.77E-03 | 4.28E-03 |
| $K01284_{VIP≥0.8\ (+)}$ | 1.02 | 0.86 | 0.68 | 0.62 | 0.97 | 1.22 | -6.27E-04 | 4.79E-03 | 2.96E-03 | 3.24E-03 | 2.50E-03 | 8.14E-03 |
| $K04043_{VIP≥0.8\ (+)}$ | 0.84 | 1.43 | 0.83 | 0.41 | 0.74 | 0.92 | 1.39E-03 | 3.80E-03 | 4.13E-03 | 2.89E-03 | -4.86E-03 | 1.56E-03 |
| $K07221_{VIP≥0.8\ (+)}$ | 1.44 | 0.76 | 0.97 | 1.08 | 0.80 | 0.29 | 9.49E-03 | 2.00E-03 | 1.07E-02 | 4.84E-03 | 2.77E-03 | -4.20E-04 |
| $K06131_{VIP≥0.8\ (+)}$ | 1.36 | 1.25 | 1.02 | 0.78 | 0.93 | 0.28 | -1.53E-02 | 3.63E-03 | 8.84E-03 | 1.57E-03 | 4.65E-03 | 2.09E-03 |
| $K00971_{VIP≥0.8\ (+)}$ | 0.94 | 0.84 | 0.94 | 0.74 | 0.71 | 1.88 | 3.34E-03 | 1.08E-03 | 9.01E-03 | -1.46E-04 | 2.36E-03 | 1.33E-02 |
| $K01790_{VIP≥0.8\ (+)}$ | 0.64 | 1.05 | 0.46 | 1.21 | 1.16 | 1.13 | 4.01E-03 | 5.03E-03 | 4.23E-03 | -6.88E-03 | 7.82E-03 | 8.58E-03 |
| $K00558_{VIP≥0.8\ (+)}$ | 0.67 | 1.44 | 1.03 | 0.73 | 1.62 | 1.76 | 4.37E-03 | 7.58E-03 | -1.07E-02 | 2.67E-04 | 1.09E-02 | 1.22E-02 |
| $K02119_{VIP≥0.8\ (+)}$ | 0.95 | 0.89 | 1.25 | 1.09 | 1.02 | 0.92 | -7.36E-03 | 7.08E-03 | 5.92E-03 | 4.40E-03 | -3.31E-03 | 3.68E-03 |
| $K07030_{VIP≥0.8\ (+)}$ | 1.02 | 0.86 | 1.08 | 1.32 | 1.91 | 1.42 | 1.39E-03 | 6.88E-03 | 7.13E-03 | 6.38E-03 | -9.12E-03 | -7.72E-03 |
| $K20742_{VIP≥0.8\ (+)}$ | 1.17 | 1.09 | 1.64 | 0.89 | 0.91 | 0.21 | 6.31E-03 | -8.40E-03 | 1.89E-02 | 4.56E-03 | -6.16E-03 | 1.54E-03 |
| $K07035_{VIP≥0.8\ (+)}$ | 0.88 | 0.80 | 1.70 | 1.20 | 1.43 | 0.77 | 2.85E-03 | 1.27E-04 | -9.70E-03 | 5.87E-03 | -5.78E-03 | 1.30E-03 |
| $K07045_{VIP≥0.8\ (+)}$ | 0.82 | 1.03 | 1.43 | 0.96 | 1.27 | 0.70 | -1.11E-02 | 7.49E-03 | 1.56E-02 | 2.39E-03 | -8.59E-03 | 1.72E-03 |
| $K02007_{VIP≥0.8\ (+)}$ | 1.15 | 1.72 | 0.98 | 1.71 | 0.42 | 1.27 | 1.48E-03 | 1.37E-02 | -5.03E-03 | 1.12E-02 | -2.02E-03 | 7.16E-03 |
| $K02492_{VIP≥0.8\ (+)}$ | 0.57 | 0.86 | 1.18 | 0.85 | 1.16 | 1.12 | 7.26E-03 | 5.02E-04 | -7.28E-03 | -4.50E-03 | 7.82E-03 | 6.66E-03 |
| $K02203_{VIP≥0.8\ (+)}$ | 0.91 | 0.93 | 0.29 | 0.85 | 1.73 | 0.81 | 1.23E-02 | 6.72E-03 | -9.35E-04 | 1.36E-03 | -6.93E-03 | 3.40E-03 |
| $K07023_{VIP≥0.8\ (+)}$ | 0.82 | 0.24 | 0.94 | 0.84 | 1.89 | 0.90 | 1.05E-02 | 1.88E-03 | -3.21E-03 | 1.12E-03 | -9.09E-03 | 2.34E-05 |
| $K01845_{VIP≥0.8\ (+)}$ | 0.74 | 0.92 | 1.05 | 0.82 | 1.25 | 1.59 | 7.94E-03 | 2.99E-03 | -6.63E-03 | -3.77E-03 | 8.39E-03 | 1.11E-02 |
| $K06213_{VIP≥0.8\ (+)}$ | 1.10 | 0.67 | 1.19 | 1.11 | 1.39 | 1.20 | 2.39E-03 | 5.15E-03 | 4.85E-03 | 6.05E-03 | -2.30E-03 | -7.41E-04 |
| $K16870_{VIP≥0.8\ (+)}$ | 1.22 | 1.07 | 0.22 | 0.93 | 0.93 | 0.96 | 5.85E-03 | 5.35E-03 | -1.47E-03 | -2.33E-03 | 2.69E-03 | 5.69E-03 |
| $K00240_{VIP≥0.8\ (+)}$ | 1.57 | 1.18 | 1.85 | 0.49 | 1.17 | 1.11 | 1.29E-02 | -4.56E-03 | 1.97E-02 | 2.11E-03 | -4.30E-03 | 8.32E-03 |
| $K03770_{VIP≥0.8\ (+)}$ | 0.89 | 0.99 | 0.33 | 1.15 | 1.24 | 0.95 | -3.45E-04 | -4.13E-03 | 3.81E-03 | 8.51E-05 | 3.41E-03 | 6.34E-03 |
| $K04486_{VIP≥0.8\ (+)}$ | 0.92 | 1.15 | 1.08 | 0.54 | 1.74 | 1.08 | 3.93E-03 | 8.86E-03 | -5.79E-03 | 1.68E-03 | -6.99E-03 | 4.72E-04 |
| $K01144_{VIP≥0.8\ (+)}$ | 1.20 | 0.85 | 0.33 | 0.86 | 1.02 | 1.61 | 7.35E-03 | -1.34E-03 | 1.36E-03 | 4.91E-03 | -3.71E-03 | 1.18E-02 |
| $K00992_{VIP≥0.8\ (+)}$ | 1.11 | 1.09 | 0.91 | 1.78 | 1.28 | 0.51 | 3.41E-03 | 7.30E-03 | 2.71E-03 | -6.53E-03 | 5.54E-03 | -2.86E-03 |
| $K02897_{VIP≥0.8\ (+)}$ | 1.35 | 0.93 | 0.36 | 1.91 | 1.10 | 1.04 | 4.81E-03 | -6.11E-03 | 4.00E-03 | -8.89E-03 | 5.92E-03 | 6.35E-03 |
| $K19353_{VIP≥0.8\ (+)}$ | 1.19 | 1.05 | 1.78 | 0.84 | 0.88 | 0.77 | 1.30E-02 | -1.37E-03 | 2.01E-02 | 3.11E-03 | 2.95E-03 | -3.38E-03 |

| | | | | | | | | | | | | |
|---|---|---|---|---|---|---|---|---|---|---|---|---|
| $K17828_{VIP\geq0.8\ (+)}$ | 0.87 | 1.33 | 1.09 | 0.28 | 0.85 | 1.06 | -1.05E-02 | 4.59E-03 | 1.11E-03 | 1.98E-03 | -5.24E-03 | 7.25E-03 |
| $K03593_{VIP\geq0.8\ (+)}$ | 0.97 | 0.81 | 1.59 | 0.78 | 1.19 | 0.93 | 2.27E-03 | -2.92E-03 | 1.53E-02 | 3.32E-03 | -7.56E-03 | 7.20E-03 |
| $K18831_{VIP\geq0.8\ (+)}$ | 1.01 | 1.05 | 1.55 | 2.10 | 1.09 | 0.64 | 9.84E-03 | -9.70E-05 | 6.74E-03 | -1.23E-02 | 7.37E-03 | 2.62E-03 |
| $K01588_{VIP\geq0.8\ (+)}$ | 0.42 | 1.10 | 1.38 | 1.45 | 1.00 | 1.13 | 3.55E-03 | 5.41E-03 | -1.18E-02 | -5.74E-03 | 6.15E-03 | 8.17E-03 |
| $K07052_{VIP\geq0.8\ (+)}$ | 0.97 | 1.01 | 2.46 | 0.90 | 1.30 | 0.47 | 8.90E-03 | 7.92E-03 | 2.45E-02 | 1.24E-03 | -6.59E-03 | -5.83E-04 |
| $K07387_{VIP\geq0.8\ (+)}$ | 1.20 | 0.31 | 2.13 | 0.53 | 1.76 | 1.02 | 1.05E-02 | 1.53E-03 | 2.42E-02 | -1.99E-03 | -1.17E-02 | 5.82E-03 |
| $K19048_{VIP\geq0.8\ (+)}$ | 1.35 | 1.12 | 0.66 | 1.19 | 0.71 | 1.03 | -6.44E-03 | 7.57E-03 | -7.59E-03 | 8.11E-03 | 2.41E-04 | 2.28E-03 |
| $K01749_{VIP\geq0.8\ (+)}$ | 1.24 | 1.42 | 0.86 | 0.55 | 0.44 | 1.95 | 1.84E-02 | 1.07E-02 | -3.54E-03 | 1.61E-03 | -2.70E-03 | 1.48E-02 |
| $K02770_{VIP\geq0.8\ (+)}$ | 1.08 | 1.44 | 0.18 | 0.55 | 0.84 | 1.22 | 4.45E-03 | 8.59E-03 | 2.08E-03 | 2.60E-03 | -3.68E-03 | -6.36E-03 |
| $K01512_{VIP\geq0.8\ (+)}$ | 0.53 | 1.01 | 0.94 | 1.13 | 1.70 | 0.44 | 5.75E-03 | 7.32E-03 | -7.90E-04 | 5.04E-03 | -8.66E-03 | 3.55E-04 |
| $K07726_{VIP\geq0.8\ (+)}$ | 2.54 | 0.83 | 0.75 | 0.68 | 1.67 | 0.90 | 3.57E-02 | 6.41E-03 | -6.48E-03 | 2.20E-03 | -8.32E-03 | 4.14E-03 |
| $K01609_{VIP\geq0.8\ (+)}$ | 0.98 | 0.42 | 0.90 | 0.64 | 1.28 | 1.07 | 1.26E-02 | 4.26E-04 | 1.02E-02 | 2.37E-03 | -4.77E-03 | -3.20E-03 |
| $K04654_{VIP\geq0.8\ (+)}$ | 1.19 | 1.53 | 0.46 | 0.41 | 0.83 | 1.38 | 1.48E-02 | 1.17E-02 | -3.61E-03 | 1.73E-03 | -3.22E-03 | 6.37E-03 |
| $K04653_{VIP\geq0.8\ (+)}$ | 1.16 | 0.91 | 0.20 | 0.60 | 1.28 | 1.42 | 1.70E-02 | 7.26E-03 | -2.26E-03 | 3.05E-03 | -7.29E-03 | 7.97E-03 |
| $K01926_{VIP\geq0.8\ (+)}$ | 0.81 | 0.43 | 0.90 | 0.82 | 1.77 | 0.71 | 9.15E-03 | 2.98E-03 | -4.92E-03 | 2.11E-03 | -6.57E-03 | 2.57E-03 |
| $K18828_{VIP\geq0.8\ (+)}$ | 0.26 | 1.48 | 1.51 | 1.39 | 1.15 | 0.30 | -3.76E-03 | 1.18E-02 | 7.94E-03 | -8.84E-03 | 7.80E-03 | 2.17E-03 |
| $K03705_{VIP\geq0.8\ (+)}$ | 1.09 | 0.36 | 0.91 | 0.52 | 1.50 | 1.08 | 4.52E-03 | -9.69E-04 | 4.34E-03 | 7.07E-04 | -4.06E-03 | 2.01E-03 |
| $K09787_{VIP\geq0.8\ (+)}$ | 0.84 | 0.50 | 0.50 | 0.83 | 1.70 | 1.04 | 5.94E-03 | -3.93E-03 | 5.49E-04 | 9.46E-04 | -6.53E-03 | 3.40E-03 |
| $K03816_{VIP\geq0.8\ (+)}$ | 0.68 | 0.89 | 1.90 | 1.21 | 0.28 | 1.27 | 3.09E-03 | -4.02E-03 | 2.20E-02 | 8.57E-03 | -1.39E-03 | 8.50E-03 |
| $K07075_{VIP\geq0.8\ (+)}$ | 0.99 | 1.19 | 1.93 | 0.19 | 0.77 | 0.95 | -1.02E-03 | 9.38E-03 | 2.23E-02 | 1.16E-03 | -3.56E-03 | 2.04E-03 |
| $K03281_{VIP\geq0.8\ (+)}$ | 1.66 | 1.32 | 0.55 | 1.44 | 1.02 | 0.38 | 1.41E-02 | -2.73E-03 | 2.18E-03 | -1.81E-03 | 5.09E-03 | 2.91E-03 |
| $K00946_{VIP\geq0.8\ (+)}$ | 1.10 | 1.41 | 0.10 | 1.71 | 1.60 | 0.68 | 7.63E-03 | -1.11E-02 | 7.25E-04 | -5.17E-03 | 9.26E-03 | 4.47E-03 |
| $K06872_{VIP\geq0.8\ (+)}$ | 0.90 | 0.64 | 2.83 | 1.02 | 1.55 | 0.31 | 1.23E-02 | 2.99E-03 | 2.78E-02 | 5.78E-03 | -8.28E-03 | -2.10E-03 |
| $K02517_{VIP\geq0.8\ (+)}$ | 0.31 | 0.99 | 1.44 | 0.99 | 0.34 | 0.81 | 1.27E-03 | -3.27E-03 | -1.14E-02 | 1.74E-03 | 1.04E-03 | 2.32E-03 |
| $K06919_{VIP\geq0.8\ (+)}$ | 1.97 | 0.85 | 1.12 | 0.45 | 0.33 | 1.30 | -2.42E-02 | 6.78E-03 | -1.13E-02 | 2.39E-03 | 3.41E-04 | 9.55E-03 |
| $K07063_{VIP\geq0.8\ (+)}$ | 0.68 | 0.90 | 1.59 | 0.94 | 0.78 | 1.10 | -1.39E-04 | -6.89E-03 | 1.83E-02 | 6.66E-03 | 1.43E-03 | 7.80E-03 |
| $K06223_{VIP\geq0.8\ (+)}$ | 1.53 | 0.91 | 1.82 | 0.42 | 1.49 | 0.62 | 2.08E-02 | 6.89E-03 | -1.97E-02 | -3.07E-04 | 1.01E-02 | 9.23E-04 |
| $K01613_{VIP\geq0.8\ (+)}$ | 1.25 | 1.06 | 0.76 | 2.14 | 0.84 | 0.37 | 1.71E-03 | -6.88E-03 | 1.90E-03 | -1.17E-02 | 1.54E-03 | 1.44E-03 |
| $K07076_{VIP\geq0.8\ (+)}$ | 1.01 | 0.29 | 1.09 | 1.30 | 0.42 | 1.13 | 2.44E-03 | -2.34E-03 | 1.01E-02 | -9.17E-03 | 1.86E-03 | 8.54E-03 |
| $K01005_{VIP\geq0.8\ (+)}$ | 1.24 | 1.04 | 0.73 | 0.55 | 1.18 | 1.15 | 7.43E-04 | 6.25E-03 | 7.93E-04 | 1.78E-03 | -2.28E-03 | -2.35E-03 |

| | | | | | | | | | | | | |
|---|---|---|---|---|---|---|---|---|---|---|---|---|
| K19157$_{VIP\geq0.8\ (+)}$ | 0.93 | 1.16 | 1.66 | 2.54 | 0.42 | 0.69 | 1.80E-03 | -8.96E-03 | 8.40E-03 | -1.58E-02 | 6.86E-05 | 3.39E-03 |
| K01991$_{VIP\geq0.8\ (+)}$ | 1.57 | 0.22 | 0.32 | 0.90 | 1.00 | 1.08 | 9.27E-03 | 6.72E-04 | 4.78E-04 | -4.34E-03 | -5.64E-03 | 6.12E-03 |
| K06142$_{VIP\geq0.8\ (+)}$ | 1.16 | 0.32 | 0.22 | 0.96 | 0.91 | 1.07 | 3.31E-03 | 4.79E-04 | 2.59E-03 | -8.91E-04 | -5.88E-05 | 7.21E-03 |
| K06406$_{VIP\geq0.8\ (-)}$ | 1.41 | 0.87 | 1.16 | 0.87 | 1.05 | 0.90 | -1.76E-02 | 6.68E-03 | -1.93E-03 | 2.13E-03 | -5.51E-03 | -2.77E-03 |
| K06204$_{VIP\geq0.8\ (-)}$ | 1.48 | 0.87 | 1.11 | 1.54 | 1.01 | 0.89 | 7.74E-03 | -6.54E-03 | -1.05E-02 | -7.38E-03 | -5.88E-03 | 4.40E-03 |
| K02548$_{VIP\geq0.8\ (-)}$ | 1.13 | 0.99 | 1.59 | 2.09 | 0.96 | 0.94 | -6.45E-04 | -3.87E-04 | -1.02E-02 | -1.19E-02 | 2.13E-03 | 6.09E-03 |
| K01304$_{VIP\geq0.8\ (-)}$ | 0.90 | 0.96 | 1.01 | 0.88 | 0.84 | 0.79 | -1.30E-03 | -4.87E-03 | 3.10E-03 | -1.78E-03 | 1.83E-03 | -5.75E-04 |
| K01118$_{VIP\geq0.8\ (-)}$ | 1.08 | 1.05 | 0.93 | 0.78 | 1.36 | 1.40 | 2.91E-03 | 4.97E-03 | -6.17E-03 | -4.64E-03 | -8.15E-03 | -1.08E-02 |
| K01759$_{VIP\geq0.8\ (-)}$ | 1.10 | 1.18 | 0.88 | 0.31 | 0.98 | 1.21 | 1.61E-02 | -8.33E-03 | -7.86E-03 | -1.88E-03 | -4.42E-03 | 5.35E-03 |
| K19689$_{VIP\geq0.8\ (-)}$ | 1.20 | 0.81 | 0.92 | 0.50 | 0.91 | 1.11 | -4.12E-04 | 3.26E-03 | -9.10E-03 | -3.32E-03 | -2.01E-03 | 3.06E-03 |
| K03303$_{VIP\geq0.8\ (-)}$ | 1.09 | 0.80 | 0.78 | 1.32 | 1.20 | 1.91 | 6.54E-03 | -6.25E-03 | 7.67E-03 | -7.23E-03 | -5.88E-03 | -1.47E-02 |
| K03473$_{VIP\geq0.8\ (-)}$ | 1.16 | 0.59 | 1.17 | 1.84 | 0.98 | 1.66 | -1.50E-02 | -4.69E-03 | 2.17E-03 | -1.17E-02 | 2.17E-03 | -1.26E-02 |
| K05832$_{VIP\geq0.8\ (-)}$ | 1.14 | 0.93 | 0.87 | 0.37 | 1.05 | 1.17 | -3.26E-03 | -5.94E-03 | -6.21E-03 | 2.00E-04 | -1.49E-03 | 4.87E-03 |
| K02574$_{VIP\geq0.8\ (-)}$ | 1.03 | 0.10 | 1.05 | 1.06 | 1.11 | 1.43 | 1.94E-03 | 3.53E-04 | -1.10E-02 | -7.52E-03 | -7.36E-03 | -1.04E-02 |
| K00350$_{VIP\geq0.8\ (-)}$ | 0.90 | 0.90 | 1.02 | 1.26 | 1.13 | 0.44 | -2.02E-03 | 6.93E-04 | -7.56E-03 | -2.11E-04 | 6.72E-03 | -3.37E-03 |
| K01633$_{VIP\geq0.8\ (-)}$ | 1.08 | 1.09 | 1.08 | 1.77 | 0.96 | 0.24 | 5.17E-03 | -4.43E-03 | -5.81E-03 | -5.77E-03 | 4.35E-03 | -1.80E-03 |
| K03284$_{VIP\geq0.8\ (-)}$ | 0.95 | 1.39 | 1.03 | 1.13 | 1.34 | 0.54 | -1.43E-02 | -7.71E-03 | -1.11E-02 | -5.70E-03 | 6.60E-03 | 2.24E-03 |
| K00794$_{VIP\geq0.8\ (-)}$ | 0.81 | 1.06 | 1.26 | 1.58 | 0.76 | 1.38 | -5.80E-03 | -8.44E-03 | -9.07E-03 | -8.43E-03 | 1.32E-03 | 1.03E-02 |
| K01262$_{VIP\geq0.8\ (-)}$ | 0.86 | 0.95 | 0.29 | 1.61 | 0.95 | 1.30 | -1.10E-02 | -2.65E-03 | 2.82E-03 | -6.87E-03 | 5.16E-03 | -8.95E-03 |
| K03613$_{VIP\geq0.8\ (-)}$ | 1.21 | 1.45 | 1.31 | 1.56 | 1.40 | 0.75 | 3.90E-03 | -1.09E-02 | -1.45E-02 | -1.02E-02 | -9.32E-03 | 5.34E-03 |
| K03585$_{VIP\geq0.8\ (-)}$ | 0.97 | 1.10 | 1.16 | 1.20 | 1.38 | 0.65 | -8.98E-03 | -2.87E-03 | -6.54E-03 | -1.10E-03 | 7.01E-03 | 2.18E-04 |
| K01972$_{VIP\geq0.8\ (-)}$ | 0.21 | 1.20 | 1.17 | 0.91 | 1.09 | 1.21 | 2.85E-03 | 4.80E-03 | -9.45E-03 | -5.90E-03 | -7.36E-03 | -6.46E-03 |
| K03499$_{VIP\geq0.8\ (-)}$ | 1.11 | 1.52 | 0.84 | 0.48 | 0.90 | 1.12 | -9.02E-03 | -8.79E-03 | -6.65E-03 | 2.98E-03 | -9.97E-04 | 4.73E-03 |
| K07107$_{VIP\geq0.8\ (-)}$ | 1.26 | 1.03 | 1.80 | 1.66 | 0.89 | 0.67 | 6.12E-03 | -5.99E-03 | -1.63E-02 | -7.69E-03 | 4.73E-03 | -4.65E-03 |
| K02004$_{VIP\geq0.8\ (-)}$ | 0.76 | 1.15 | 1.05 | 1.66 | 1.32 | 0.97 | -1.04E-02 | 6.45E-03 | 6.24E-03 | -1.11E-02 | -7.49E-03 | -7.39E-03 |
| K10112$_{VIP\geq0.8\ (-)}$ | 0.04 | 1.30 | 1.55 | 1.17 | 1.83 | 0.17 | 5.18E-04 | 9.35E-03 | -6.83E-03 | -5.96E-03 | -1.01E-02 | -1.28E-03 |
| K19304$_{VIP\geq0.8\ (-)}$ | 0.90 | 1.80 | 1.60 | 1.97 | 0.47 | 0.43 | 1.16E-02 | -1.42E-02 | -3.95E-04 | -1.34E-02 | -2.84E-03 | 5.66E-04 |
| K16787$_{VIP\geq0.8\ (-)}$ | 1.07 | 0.90 | 0.58 | 0.36 | 1.45 | 1.03 | 2.26E-03 | -9.65E-04 | -1.96E-03 | -2.07E-03 | -5.77E-03 | 2.60E-03 |
| K00974$_{VIP\geq0.8\ (-)}$ | 1.17 | 0.97 | 0.64 | 0.92 | 0.64 | 1.24 | 1.77E-02 | -7.68E-03 | -5.87E-03 | -6.39E-03 | 2.85E-03 | -6.50E-03 |
| K07461$_{VIP\geq0.8\ (-)}$ | 0.40 | 0.60 | 1.63 | 1.10 | 1.77 | 1.40 | -1.68E-03 | -3.19E-03 | 1.85E-02 | -6.89E-03 | -9.42E-03 | 1.08E-02 |

| | | | | | | | | | | | | |
|---|---|---|---|---|---|---|---|---|---|---|---|---|
| $K01235_{VIP \geq 0.8\ (-)}$ | 0.44 | 0.83 | 0.96 | 1.09 | 0.83 | 0.50 | -1.97E-03 | 2.83E-03 | -9.78E-03 | -2.20E-03 | -1.95E-03 | 2.58E-03 |
| $K02031_{VIP \geq 0.8\ (-)}$ | 1.43 | 0.88 | 0.85 | 0.19 | 0.77 | 1.40 | -5.81E-03 | -8.07E-04 | -8.03E-03 | -1.29E-03 | 2.37E-04 | 6.32E-03 |
| $K22927_{VIP \geq 0.8\ (-)}$ | 1.14 | 0.87 | 0.72 | 0.53 | 1.10 | 1.19 | 1.06E-02 | -1.45E-03 | -3.79E-03 | 2.53E-03 | -2.91E-03 | -3.73E-03 |
| $K02027_{VIP \geq 0.8\ (-)}$ | 1.09 | 0.79 | 1.04 | 0.16 | 0.93 | 1.12 | 6.49E-03 | -1.44E-03 | -9.88E-03 | -1.07E-03 | -5.21E-03 | 1.42E-03 |
| $K08602_{VIP \geq 0.8\ (-)}$ | 1.29 | 0.80 | 0.84 | 0.22 | 0.94 | 0.95 | -3.47E-03 | -1.33E-03 | -4.78E-03 | 3.38E-05 | -2.98E-03 | 1.70E-03 |
| $K03442_{VIP \geq 0.8\ (-)}$ | 0.56 | 0.85 | 0.98 | 0.84 | 0.97 | 0.50 | 1.31E-03 | -6.39E-03 | 1.08E-02 | -5.84E-03 | -1.48E-03 | -3.10E-03 |
| $K07335_{VIP \geq 0.8\ (-)}$ | 1.46 | 0.46 | 0.86 | 0.31 | 1.02 | 1.06 | -6.55E-03 | 3.06E-03 | -8.68E-03 | -1.06E-03 | -5.78E-03 | 3.98E-03 |
| $K00179_{VIP \geq 0.8\ (-)}$ | 1.01 | 0.22 | 1.10 | 0.80 | 1.48 | 0.85 | -1.44E-03 | 4.27E-04 | 1.61E-03 | -5.66E-03 | -9.99E-03 | -6.09E-03 |
| $K07574_{VIP \geq 0.8\ (-)}$ | 0.86 | 0.29 | 0.99 | 0.54 | 1.40 | 0.98 | 2.31E-03 | -9.41E-04 | -1.13E-02 | -3.80E-03 | -4.87E-03 | 4.95E-05 |
| $K01246_{VIP \geq 0.8\ (-)}$ | 1.22 | 0.95 | 0.43 | 0.53 | 1.05 | 1.33 | -6.44E-03 | -4.68E-03 | -2.65E-03 | -3.60E-04 | 6.48E-03 | 7.98E-03 |
| $K03784_{VIP \geq 0.8\ (-)}$ | 0.67 | 1.25 | 1.12 | 0.57 | 0.85 | 1.31 | 2.31E-03 | -9.82E-03 | -2.01E-03 | -2.39E-03 | -3.84E-03 | 8.22E-03 |
| $K23351_{VIP \geq 0.8\ (-)}$ | 1.93 | 0.42 | 1.23 | 0.22 | 1.35 | 0.85 | 2.03E-02 | -2.84E-03 | -1.42E-02 | -1.45E-03 | -6.66E-03 | 6.29E-03 |
| $K21064_{VIP \geq 0.8\ (-)}$ | 0.79 | 0.51 | 0.80 | 1.27 | 1.46 | 1.20 | 9.39E-03 | -4.07E-03 | -6.08E-04 | 7.98E-03 | -7.29E-03 | -6.28E-04 |
| $K03581_{VIP \geq 0.8\ (-)}$ | 1.02 | 0.81 | 0.55 | 0.23 | 0.83 | 1.10 | 3.37E-03 | -3.96E-03 | -4.38E-03 | -1.58E-03 | 1.55E-03 | -3.15E-04 |
| $K02067_{VIP \geq 0.8\ (-)}$ | 0.90 | 1.29 | 1.35 | 1.91 | 0.69 | 0.33 | 1.08E-03 | -7.22E-03 | -3.83E-03 | -6.43E-03 | -2.37E-03 | 2.58E-03 |
| $K00796_{VIP \geq 0.8\ (-)}$ | 0.53 | 1.25 | 1.99 | 1.48 | 0.80 | 0.24 | -7.97E-03 | 2.78E-03 | -2.05E-02 | -2.95E-03 | 5.23E-03 | -5.86E-04 |
| $K07259_{VIP \geq 0.8\ (-)}$ | 0.48 | 1.42 | 1.70 | 1.30 | 0.67 | 1.02 | -4.15E-03 | -6.96E-03 | 1.80E-02 | -1.33E-03 | -7.60E-04 | 7.87E-03 |
| $K00931_{VIP \geq 0.8\ (-)}$ | 0.96 | 1.07 | 0.81 | 1.10 | 0.71 | 0.41 | -8.23E-03 | 4.43E-03 | 4.80E-04 | -4.00E-03 | -4.33E-03 | -5.91E-04 |
| $K02501_{VIP \geq 0.8\ (-)}$ | 0.22 | 1.14 | 0.62 | 1.40 | 0.97 | 0.81 | 2.46E-03 | 4.14E-03 | -3.21E-03 | -4.80E-03 | -5.01E-03 | -2.98E-03 |
| $K01933_{VIP \geq 0.8\ (-)}$ | 0.79 | 1.67 | 0.97 | 0.97 | 0.48 | 1.04 | -2.09E-03 | 8.62E-03 | -1.10E-02 | -5.06E-03 | -3.03E-03 | 6.14E-03 |
| $K04095_{VIP \geq 0.8\ (-)}$ | 0.08 | 1.77 | 1.16 | 1.83 | 0.43 | 1.20 | 1.23E-03 | -1.04E-02 | -5.46E-03 | -1.15E-02 | 1.28E-03 | -7.15E-03 |
| $K03596_{VIP \geq 0.8\ (-)}$ | 1.29 | 1.28 | 0.61 | 0.81 | 0.44 | 1.15 | -1.22E-02 | 2.09E-03 | -5.46E-03 | -3.42E-03 | 2.98E-03 | -4.29E-03 |
| $K02115_{VIP \geq 0.8\ (-)}$ | 0.85 | 1.15 | 0.47 | 1.33 | 0.16 | 0.89 | 1.25E-02 | 3.53E-03 | -3.63E-03 | -6.79E-03 | -1.08E-03 | -4.10E-04 |
| $K09748_{VIP \geq 0.8\ (-)}$ | 0.63 | 0.89 | 0.27 | 1.68 | 1.46 | 1.38 | -6.94E-03 | -4.44E-03 | 1.85E-03 | -1.05E-02 | -9.11E-03 | 9.23E-03 |
| $K00067_{VIP \geq 0.8\ (-)}$ | 0.96 | 1.05 | 1.75 | 1.45 | 0.52 | 0.57 | -1.31E-02 | 3.99E-03 | -1.97E-02 | -7.32E-03 | -5.04E-04 | 1.94E-03 |
| $K00764_{VIP \geq 0.8\ (-)}$ | 0.74 | 1.39 | 0.98 | 1.59 | 1.19 | 0.13 | 3.28E-03 | 7.53E-03 | -9.52E-03 | -9.08E-03 | -7.97E-03 | -1.75E-04 |
| $K02838_{VIP \geq 0.8\ (-)}$ | 1.05 | 1.22 | 0.71 | 0.89 | 0.96 | 0.23 | 6.87E-03 | -9.72E-03 | -8.07E-03 | -6.32E-03 | -6.11E-03 | 1.76E-03 |
| $K03561_{VIP \geq 0.8\ (-)}$ | 1.84 | 1.09 | 0.35 | 1.75 | 0.31 | 1.32 | 1.54E-02 | -4.21E-04 | -1.12E-03 | -7.16E-03 | -1.85E-04 | 1.02E-02 |
| $K15921_{VIP \geq 0.8\ (-)}$ | 1.02 | 0.56 | 0.54 | 1.09 | 1.13 | 1.10 | -4.45E-03 | 4.36E-03 | 3.82E-04 | -7.29E-03 | -3.87E-03 | -7.79E-03 |
| $K03630_{VIP \geq 0.8\ (-)}$ | 0.08 | 0.98 | 1.02 | 0.98 | 1.31 | 0.18 | -1.11E-03 | 1.88E-03 | -3.31E-03 | -6.84E-03 | -8.66E-03 | 6.84E-04 |

| | | | | | | | | | | | | |
|---|---|---|---|---|---|---|---|---|---|---|---|---|
| $K07154_{VIP \geq 0.8\ (-)}$ | 0.97 | 1.13 | 0.92 | 1.81 | 0.39 | 0.49 | -2.42E-03 | 7.11E-03 | 7.63E-03 | -7.32E-03 | -6.47E-05 | -1.07E-03 |
| $K08678_{VIP \geq 0.8\ (-)}$ | 1.02 | 1.22 | 1.49 | 1.59 | 1.68 | 1.74 | -1.49E-02 | -9.59E-03 | -1.44E-02 | -1.06E-02 | 1.10E-02 | -1.31E-02 |
| $K13599_{VIP \geq 0.8\ (-)}$ | 1.06 | 1.18 | 0.84 | 0.88 | 0.84 | 0.53 | -2.84E-03 | -6.96E-03 | 9.70E-03 | -3.17E-03 | -5.42E-03 | -1.10E-04 |
| $K02173_{VIP \geq 0.8\ (-)}$ | 1.29 | 0.02 | 1.17 | 1.25 | 1.28 | 0.96 | -1.27E-02 | -9.17E-05 | 2.35E-03 | -8.47E-03 | -7.40E-03 | -2.64E-03 |
| $K23004_{VIP \geq 0.8\ (-)}$ | 1.27 | 0.29 | 1.15 | 1.31 | 1.70 | 1.26 | 7.32E-03 | -6.66E-04 | -1.28E-02 | -8.51E-03 | -1.11E-02 | -9.54E-03 |
| $K23010_{VIP \geq 0.8\ (-)}$ | 0.85 | 0.23 | 0.95 | 1.39 | 0.85 | 1.22 | -1.04E-02 | 5.44E-04 | -8.69E-03 | -9.44E-03 | -4.83E-04 | -7.88E-03 |
| $K06934_{VIP \geq 0.8\ (-)}$ | 1.11 | 0.32 | 0.98 | 1.66 | 1.52 | 1.23 | -1.61E-02 | 1.38E-03 | -1.12E-02 | -1.18E-02 | -1.02E-02 | -9.41E-03 |
| $K03474_{VIP \geq 0.8\ (-)}$ | 0.87 | 0.86 | 1.29 | 1.80 | 0.89 | 0.79 | -9.39E-03 | -6.88E-03 | -5.52E-03 | -1.13E-02 | 1.62E-03 | -4.96E-03 |
| $K07588_{VIP \geq 0.8\ (-)}$ | 1.14 | 0.32 | 0.93 | 0.95 | 1.32 | 1.10 | 1.08E-04 | -2.32E-03 | -1.05E-02 | -6.73E-03 | -6.07E-03 | -7.15E-03 |
| $K18700_{VIP \geq 0.8\ (-)}$ | 0.92 | 0.59 | 1.51 | 1.78 | 1.34 | 1.00 | -1.36E-02 | 3.70E-03 | -1.75E-02 | -1.26E-02 | -9.01E-03 | -7.49E-03 |
| $K03634_{VIP \geq 0.8\ (-)}$ | 1.26 | 0.92 | 1.30 | 1.67 | 1.04 | 0.60 | 7.99E-03 | -7.17E-03 | -1.07E-02 | -8.95E-03 | -6.94E-03 | -2.71E-03 |
| $K03642_{VIP \geq 0.8\ (-)}$ | 0.90 | 0.89 | 2.00 | 2.09 | 1.23 | 0.28 | -9.01E-04 | -1.76E-03 | -1.89E-02 | -1.05E-02 | 5.34E-03 | -1.14E-03 |
| $K03778_{VIP \geq 0.8\ (-)}$ | 0.97 | 1.24 | 1.19 | 0.43 | 0.96 | 1.54 | 2.51E-03 | -8.34E-03 | -1.37E-02 | -9.25E-04 | -6.48E-03 | -9.04E-03 |
| $K01934_{VIP \geq 0.8\ (-)}$ | 0.53 | 1.33 | 1.70 | 1.23 | 1.13 | 1.17 | -6.71E-03 | -6.61E-03 | -1.90E-02 | -6.93E-03 | -3.73E-03 | 2.68E-03 |
| $K01883_{VIP \geq 0.8\ (-)}$ | 1.08 | 1.38 | 1.78 | 0.44 | 1.04 | 0.95 | -1.28E-02 | 1.79E-03 | -1.69E-02 | -2.06E-03 | -6.54E-03 | -2.71E-03 |
| $K07005_{VIP \geq 0.8\ (-)}$ | 0.35 | 1.59 | 1.67 | 0.89 | 1.27 | 1.57 | -5.00E-03 | 1.13E-02 | -1.90E-02 | -4.86E-03 | -7.53E-03 | -8.45E-03 |
| $K20074_{VIP \geq 0.8\ (-)}$ | 0.93 | 1.30 | 0.26 | 1.11 | 1.29 | 1.21 | -1.40E-02 | 1.01E-02 | -2.95E-04 | -7.78E-03 | -8.32E-03 | -9.21E-03 |
| $K01921_{VIP \geq 0.8\ (-)}$ | 0.79 | 1.36 | 1.24 | 1.30 | 1.45 | 0.82 | -9.44E-03 | -8.23E-04 | -1.43E-02 | -7.84E-03 | -9.73E-03 | 2.80E-03 |
| $K03545_{VIP \geq 0.8\ (-)}$ | 1.36 | 1.27 | 0.96 | 0.63 | 1.34 | 1.24 | -1.19E-02 | -7.19E-04 | -7.93E-03 | 4.44E-03 | -6.11E-03 | -5.46E-03 |
| $K02601_{VIP \geq 0.8\ (-)}$ | 0.90 | 0.87 | 0.86 | 1.13 | 1.34 | 0.50 | 6.82E-03 | -6.22E-03 | -9.93E-03 | -7.78E-03 | -8.74E-03 | -1.13E-03 |
| $K07126_{VIP \geq 0.8\ (-)}$ | 0.54 | 1.08 | 1.40 | 1.62 | 0.81 | 0.82 | -1.67E-03 | -4.32E-03 | -5.10E-03 | -8.61E-03 | 5.49E-03 | -4.16E-03 |
| $K02466_{VIP \geq 0.8\ (-)}$ | 1.85 | 0.03 | 0.95 | 0.86 | 0.89 | 0.77 | 1.99E-02 | -1.37E-04 | -5.82E-03 | -5.95E-03 | -5.97E-03 | -5.63E-03 |
| $K02759_{VIP \geq 0.8\ (-)}$ | 1.10 | 1.11 | 0.92 | 0.72 | 0.53 | 1.06 | -7.16E-03 | 5.13E-03 | -9.31E-03 | -4.95E-03 | -2.05E-03 | -2.78E-03 |
| $K00756_{VIP \geq 0.8\ (-)}$ | 0.51 | 0.85 | 0.81 | 1.08 | 0.79 | 0.96 | -5.01E-03 | -5.48E-03 | -5.15E-03 | -1.76E-03 | 1.29E-03 | -3.17E-03 |
| $K03658_{VIP \geq 0.8\ (-)}$ | 0.38 | 1.28 | 1.34 | 1.45 | 0.92 | 0.48 | -5.71E-03 | -9.95E-03 | -1.25E-02 | -7.78E-03 | 5.22E-03 | -3.27E-03 |
| $K07054_{VIP \geq 0.8\ (-)}$ | 1.10 | 0.80 | 0.60 | 1.06 | 0.89 | 0.92 | -2.21E-04 | 4.76E-03 | -3.97E-03 | -7.32E-03 | -2.80E-03 | -3.27E-03 |
| $K09807_{VIP \geq 0.8\ (-)}$ | 1.10 | 0.83 | 0.80 | 0.60 | 0.62 | 0.96 | -2.94E-03 | -5.36E-03 | -6.64E-04 | -2.42E-03 | -1.21E-03 | 1.05E-03 |
| $K06020_{VIP \geq 0.8\ (-)}$ | 1.05 | 0.48 | 1.00 | 2.11 | 0.26 | 1.20 | 8.88E-03 | -7.72E-04 | -8.31E-03 | -1.29E-02 | -1.51E-03 | -9.28E-03 |
| $K01139_{VIP \geq 0.8\ (-)}$ | 1.71 | 1.28 | 0.63 | 0.87 | 0.77 | 1.30 | -1.49E-02 | 9.26E-03 | -6.66E-03 | -5.75E-03 | -3.06E-03 | -7.64E-03 |
| $K03118_{VIP \geq 0.8\ (-)}$ | 1.22 | 0.25 | 0.53 | 1.35 | 1.25 | 1.00 | 4.24E-03 | -1.50E-03 | -5.55E-03 | -9.36E-03 | -6.07E-03 | -2.56E-03 |

| | | | | | | | | | | | | |
|---|---|---|---|---|---|---|---|---|---|---|---|---|
| $K00566_{VIP≥0.8\ (-)}$ | 1.11 | 1.14 | 0.34 | 0.17 | 1.03 | 1.07 | -3.63E-03 | -8.20E-03 | 3.90E-03 | -1.22E-03 | -4.47E-03 | -1.28E-03 |
| $K02065_{VIP≥0.8\ (-)}$ | 0.97 | 1.38 | 1.43 | 1.87 | 0.66 | 0.22 | -1.03E-03 | -1.03E-02 | -6.22E-03 | -7.16E-03 | 1.47E-03 | -9.99E-04 |
| $K02527_{VIP≥0.8\ (-)}$ | 0.80 | 1.02 | 0.54 | 1.48 | 1.29 | 0.44 | -4.47E-04 | -5.83E-03 | -2.37E-03 | -3.64E-03 | 4.60E-03 | -3.37E-03 |
| $K09774_{VIP≥0.8\ (-)}$ | 1.37 | 1.46 | 1.61 | 1.82 | 0.76 | 0.53 | 1.02E-02 | -6.73E-03 | -1.15E-02 | -5.11E-03 | -9.35E-04 | -4.09E-03 |
| $K23775_{VIP≥0.8\ (-)}$ | 1.17 | 0.80 | 1.11 | 0.70 | 2.38 | 0.56 | 1.15E-02 | -5.67E-03 | -7.43E-03 | -4.03E-03 | -1.55E-02 | -3.14E-03 |
| $K03612_{VIP≥0.8\ (-)}$ | 1.51 | 1.03 | 0.97 | 0.76 | 1.59 | 0.24 | 1.08E-02 | -7.34E-03 | -6.23E-03 | -3.53E-03 | -1.08E-02 | -9.67E-04 |
| $K01297_{VIP≥0.8\ (-)}$ | 0.77 | 0.14 | 1.19 | 0.99 | 1.16 | 1.44 | -6.64E-03 | 6.00E-04 | -1.27E-02 | -7.04E-03 | -6.75E-03 | -1.02E-02 |
| $K06901_{VIP≥0.8\ (-)}$ | 0.32 | 1.41 | 1.19 | 0.99 | 0.77 | 1.14 | -4.35E-03 | 8.88E-03 | -4.69E-03 | -6.36E-03 | -3.63E-03 | -8.64E-03 |
| $K02970_{VIP≥0.8\ (-)}$ | 0.90 | 1.76 | 0.72 | 1.16 | 1.57 | 0.57 | -1.23E-02 | -1.41E-02 | -4.11E-03 | -7.47E-03 | -1.04E-02 | 3.60E-03 |
| $K08316_{VIP≥0.8\ (-)}$ | 0.36 | 1.42 | 0.92 | 1.74 | 0.54 | 0.80 | 3.16E-03 | -9.47E-03 | -5.69E-03 | -1.07E-02 | -5.29E-04 | -2.72E-03 |
| $K07568_{VIP≥0.8\ (-)}$ | 0.62 | 1.32 | 0.39 | 1.59 | 0.85 | 0.82 | -2.28E-03 | -4.97E-03 | -4.51E-03 | -8.07E-03 | -5.55E-03 | 4.45E-03 |
| $K03574_{VIP≥0.8\ (-)}$ | 1.18 | 0.49 | 1.53 | 2.32 | 1.77 | 0.51 | 5.09E-03 | -3.36E-03 | -1.68E-02 | -1.60E-02 | -1.20E-02 | -1.51E-03 |
| $K00275_{VIP≥0.8\ (-)}$ | 0.26 | 1.04 | 1.62 | 1.15 | 0.83 | 1.07 | -2.93E-03 | -8.17E-03 | -2.97E-03 | -5.11E-03 | -2.93E-04 | -5.12E-03 |
| $K07260_{VIP≥0.8\ (-)}$ | 1.09 | 0.54 | 1.73 | 1.58 | 1.37 | 0.82 | -1.48E-02 | -2.33E-03 | -1.71E-02 | -1.08E-02 | -5.57E-03 | -4.39E-03 |
| $K00748_{VIP≥0.8\ (-)}$ | 0.90 | 1.45 | 1.12 | 2.20 | 0.89 | 0.21 | -7.20E-03 | -9.54E-03 | -9.56E-03 | -1.05E-02 | -2.49E-03 | -1.03E-03 |
| $K06207_{VIP≥0.8\ (-)}$ | 1.76 | 1.49 | 0.68 | 0.86 | 0.88 | 0.86 | -1.85E-02 | -2.77E-03 | -6.49E-03 | -4.28E-03 | -5.74E-03 | -1.34E-03 |
| $K01091_{VIP≥0.8\ (-)}$ | 1.58 | 1.28 | 0.91 | 1.14 | 0.62 | 1.00 | -1.25E-02 | -3.67E-03 | -5.99E-03 | -6.46E-03 | -7.65E-04 | -7.65E-04 |
| $K09764_{VIP≥0.8\ (-)}$ | 0.95 | 0.82 | 0.05 | 0.33 | 1.36 | 0.93 | -9.24E-03 | -2.70E-03 | -1.19E-04 | -2.27E-03 | -8.75E-03 | -6.70E-03 |
| $K16329_{VIP≥0.8\ (-)}$ | 0.93 | 1.10 | 0.70 | 0.77 | 1.11 | 1.07 | -6.63E-03 | -7.10E-03 | -3.23E-03 | -2.59E-03 | -7.33E-03 | -8.11E-03 |
| $K02805_{VIP≥0.8\ (-)}$ | 2.35 | 0.75 | 1.51 | 1.73 | 0.77 | 0.89 | -3.01E-02 | -2.88E-03 | -1.71E-02 | -1.17E-02 | -2.48E-03 | -3.98E-03 |
| $K19271_{VIP≥0.8\ (-)}$ | 1.07 | 0.28 | 1.30 | 0.61 | 1.12 | 1.17 | -1.33E-03 | -7.20E-04 | -1.43E-02 | -3.94E-03 | -7.55E-03 | -7.64E-03 |
| $K01726_{VIP≥0.8\ (-)}$ | 1.05 | 0.28 | 0.95 | 1.06 | 1.00 | 0.61 | -1.46E-02 | -1.53E-03 | -1.09E-02 | -7.53E-03 | -3.52E-03 | -1.14E-03 |
| $K07058_{VIP≥0.8\ (-)}$ | 1.49 | 0.57 | 0.68 | 2.59 | 1.31 | 0.89 | -2.26E-02 | -4.07E-03 | -3.42E-03 | -1.72E-02 | -8.51E-03 | -6.76E-03 |
| $K00979_{VIP≥0.8\ (-)}$ | 0.86 | 1.24 | 1.29 | 1.63 | 0.32 | 0.44 | -3.51E-03 | -5.21E-03 | -9.32E-03 | -2.75E-03 | -8.82E-04 | -3.31E-03 |
| $K19824_{VIP≥0.8\ (-)}$ | 0.52 | 0.89 | 1.00 | 1.25 | 0.93 | 0.56 | -5.14E-03 | -5.72E-03 | -2.45E-03 | -8.85E-03 | -6.27E-03 | -4.05E-03 |
| $K01679_{VIP≥0.8}$ | 0.89 | 1.33 | 1.29 | 1.46 | 1.40 | 1.22 | 6.14E-03 | 9.76E-03 | -5.52E-03 | -9.63E-03 | -6.50E-03 | 9.21E-03 |
| $K21556_{VIP≥0.8}$ | 0.93 | 1.18 | 1.99 | 0.85 | 1.13 | 1.43 | 1.20E-03 | -9.29E-03 | 2.23E-02 | 5.26E-03 | -3.42E-03 | -1.07E-02 |
| $K08640_{VIP≥0.8}$ | 1.07 | 0.93 | 1.30 | 1.41 | 0.93 | 0.87 | -1.68E-03 | 7.44E-03 | 1.50E-02 | -8.71E-03 | 6.26E-03 | -4.67E-03 |
| $K01575_{VIP≥0.8}$ | 1.28 | 0.81 | 0.33 | 1.28 | 1.03 | 0.96 | 1.62E-02 | 5.84E-03 | -3.55E-03 | 7.79E-03 | -6.02E-03 | -7.21E-03 |
| $K06012_{VIP≥0.8}$ | 0.98 | 0.56 | 1.25 | 1.17 | 1.16 | 0.90 | -1.32E-02 | 4.13E-03 | 2.62E-03 | 2.93E-03 | -6.18E-03 | -3.89E-03 |

| | | | | | | | | | | | | |
|---|---|---|---|---|---|---|---|---|---|---|---|---|
| $K02279_{VIP≥0.8}$ | 0.93 | 1.16 | 1.48 | 0.56 | 0.96 | 1.74 | -1.55E-03 | 8.96E-03 | -1.27E-02 | 2.94E-03 | -5.00E-03 | 1.23E-02 |
| $K01193_{VIP≥0.8}$ | 0.95 | 1.37 | 0.93 | 0.39 | 1.39 | 0.99 | 2.80E-03 | 1.08E-02 | -6.72E-03 | 2.47E-03 | -8.69E-03 | -3.10E-04 |
| $K21744_{VIP≥0.8}$ | 1.77 | 0.69 | 0.82 | 0.91 | 1.68 | 1.66 | -1.53E-02 | 5.26E-03 | 2.06E-03 | 2.51E-03 | -9.27E-03 | -1.18E-02 |
| $K03775_{VIP≥0.8}$ | 1.97 | 0.85 | 1.15 | 1.44 | 0.16 | 1.37 | 2.05E-02 | -3.57E-03 | 9.13E-03 | -7.93E-03 | -2.58E-04 | 1.04E-02 |
| $K06898_{VIP≥0.8}$ | 1.20 | 2.30 | 0.81 | 0.37 | 1.22 | 1.57 | 1.36E-03 | 1.81E-02 | -5.36E-03 | -1.44E-03 | -6.77E-03 | 1.00E-02 |
| $K01966_{VIP≥0.8}$ | 0.81 | 1.20 | 1.75 | 1.34 | 0.43 | 0.90 | 6.31E-04 | -7.49E-03 | 1.89E-02 | 9.14E-03 | -2.32E-03 | -3.92E-03 |
| $K23535_{VIP≥0.8}$ | 1.47 | 1.07 | 1.01 | 0.31 | 0.83 | 1.50 | -8.41E-03 | 6.88E-03 | -9.49E-03 | 6.29E-04 | -1.41E-03 | 9.09E-03 |
| $K02315_{VIP≥0.8}$ | 0.67 | 1.18 | 1.47 | 1.32 | 1.21 | 1.11 | 5.27E-03 | 9.00E-03 | -1.28E-02 | 7.18E-03 | -4.94E-03 | -2.17E-03 |
| $K00912_{VIP≥0.8}$ | 1.09 | 1.06 | 0.62 | 2.16 | 0.88 | 0.80 | 2.22E-03 | -7.46E-03 | -2.72E-03 | -1.11E-02 | 1.45E-03 | 5.99E-03 |
| $K01918_{VIP≥0.8}$ | 1.56 | 0.91 | 0.75 | 1.65 | 0.83 | 1.03 | 1.17E-02 | 5.41E-03 | -6.18E-03 | -5.20E-03 | 1.25E-04 | -7.86E-03 |
| $K03269_{VIP≥0.8}$ | 1.50 | 0.83 | 0.67 | 1.50 | 1.16 | 0.85 | 8.61E-03 | -1.78E-03 | -1.65E-03 | -3.32E-03 | 3.32E-04 | 5.60E-03 |
| $K01627_{VIP≥0.8}$ | 1.21 | 1.41 | 1.54 | 1.42 | 1.21 | 0.68 | 6.69E-03 | -9.38E-03 | -9.50E-03 | -8.94E-04 | 7.96E-03 | 5.07E-03 |
| $K03975_{VIP≥0.8}$ | 1.03 | 1.35 | 1.90 | 1.89 | 1.14 | 0.36 | 5.22E-04 | -8.80E-03 | -1.80E-02 | -8.47E-03 | 6.10E-03 | 2.18E-03 |
| $K05837_{VIP≥0.8}$ | 1.39 | 1.27 | 0.84 | 1.98 | 1.40 | 0.31 | 8.53E-03 | -5.95E-03 | -3.62E-03 | -8.12E-03 | 7.91E-03 | 1.25E-03 |
| $K02536_{VIP≥0.8}$ | 1.23 | 1.39 | 0.95 | 1.84 | 0.96 | 0.41 | 4.16E-03 | -9.56E-03 | 4.22E-04 | -9.48E-03 | -2.71E-03 | 8.83E-04 |
| $K00950_{VIP≥0.8}$ | 1.00 | 0.85 | 1.17 | 1.75 | 1.05 | 0.71 | 6.48E-04 | -2.62E-03 | -4.98E-03 | -6.51E-03 | 4.56E-03 | 3.84E-03 |
| $K00973_{VIP≥0.8}$ | 1.11 | 1.04 | 1.32 | 1.39 | 0.42 | 0.87 | -1.55E-02 | 5.07E-03 | 1.39E-02 | -8.71E-03 | -1.28E-03 | 6.77E-03 |
| $K03497_{VIP≥0.8}$ | 0.81 | 1.12 | 1.27 | 0.54 | 1.10 | 1.09 | 3.74E-03 | 1.31E-03 | -1.45E-02 | -3.84E-03 | 5.42E-03 | -1.29E-03 |
| $K02781_{VIP≥0.8}$ | 1.76 | 1.22 | 1.03 | 0.41 | 0.95 | 0.76 | 1.96E-02 | 9.70E-03 | -7.37E-03 | 1.10E-03 | -4.98E-03 | -3.55E-03 |
| $K01780_{VIP≥0.8}$ | 1.22 | 0.53 | 0.90 | 0.99 | 1.32 | 0.78 | 1.16E-02 | -3.71E-03 | 2.04E-03 | -4.76E-03 | -7.70E-03 | 5.81E-03 |
| $K23356_{VIP≥0.8}$ | 1.83 | 1.08 | 0.60 | 0.96 | 0.53 | 1.09 | -1.87E-02 | -5.30E-03 | -5.03E-03 | 6.29E-03 | 2.40E-03 | 7.85E-03 |
| $K08234_{VIP≥0.8}$ | 2.53 | 0.86 | 0.34 | 0.66 | 1.48 | 0.88 | 3.30E-02 | 5.86E-04 | -1.73E-03 | 3.43E-03 | -9.64E-03 | -5.72E-03 |
| $K22928_{VIP≥0.8}$ | 1.08 | 1.05 | 0.42 | 1.11 | 0.89 | 0.75 | -9.97E-03 | -7.53E-03 | -4.65E-03 | 7.24E-03 | 3.03E-03 | 1.93E-03 |
| $K01500_{VIP≥0.8}$ | 1.28 | 0.95 | 0.54 | 0.28 | 1.20 | 1.35 | -1.00E-03 | 7.45E-03 | -2.24E-04 | 4.72E-04 | -7.73E-03 | 6.51E-03 |
| $K19411_{VIP≥0.8}$ | 2.55 | 0.20 | 0.38 | 1.20 | 1.68 | 0.93 | 3.40E-02 | -5.38E-04 | -7.89E-05 | 5.02E-03 | -9.17E-03 | 6.18E-04 |
| $K14415_{VIP≥0.8}$ | 0.31 | 1.27 | 0.81 | 0.59 | 1.09 | 1.06 | -4.62E-03 | 9.41E-03 | 5.41E-04 | -4.16E-03 | -7.33E-03 | 8.17E-03 |
| $K09516_{VIP≥0.8}$ | 1.37 | 1.06 | 1.37 | 0.61 | 0.68 | 1.03 | 6.31E-03 | -7.04E-03 | 1.58E-02 | 4.09E-03 | -4.35E-03 | -7.43E-03 |
| $K20276_{VIP≥0.8}$ | 1.00 | 0.50 | 0.86 | 0.94 | 1.25 | 0.53 | 5.05E-03 | -9.06E-04 | 9.21E-03 | -6.40E-03 | -8.45E-03 | 1.22E-03 |
| $K01255_{VIP≥0.8}$ | 0.68 | 1.26 | 0.87 | 1.50 | 1.02 | 0.51 | 7.09E-03 | -8.88E-03 | -7.30E-03 | -9.31E-03 | 6.17E-03 | 8.79E-04 |
| $K15771_{VIP≥0.8}$ | 1.02 | 0.39 | 0.50 | 0.94 | 1.05 | 1.25 | 7.71E-03 | 1.44E-03 | 5.75E-03 | -1.00E-03 | -2.07E-03 | -8.69E-03 |

| | | | | | | | | | | | | |
|---|---|---|---|---|---|---|---|---|---|---|---|---|
| K00965$_{VIP\geq0.8}$ | 1.18 | 1.34 | 0.61 | 0.69 | 1.53 | 1.21 | 1.98E-03 | 1.06E-02 | -2.18E-03 | 3.15E-03 | -8.25E-03 | -5.32E-03 |
| K03741$_{VIP\geq0.8}$ | 0.87 | 1.02 | 0.27 | 1.44 | 0.42 | 1.14 | 8.04E-03 | -5.09E-03 | 2.89E-03 | -7.15E-03 | 2.60E-03 | -3.10E-03 |
| K10117$_{VIP\geq0.8}$ | 1.42 | 1.00 | 0.88 | 0.09 | 0.64 | 0.83 | -4.41E-03 | 4.00E-03 | 8.86E-03 | 1.94E-04 | -2.54E-03 | -3.63E-05 |
| K06864$_{VIP\geq0.8}$ | 1.10 | 1.91 | 0.78 | 0.53 | 1.00 | 1.50 | 2.78E-03 | 1.51E-02 | -6.47E-03 | -3.27E-03 | -5.88E-03 | 9.38E-03 |
| K01698$_{VIP\geq0.8}$ | 0.74 | 0.76 | 1.49 | 1.18 | 0.89 | 0.85 | 2.23E-03 | -4.68E-04 | -1.24E-02 | -6.77E-03 | 6.03E-03 | 2.60E-03 |
| K12976$_{VIP\geq0.8}$ | 0.02 | 1.12 | 1.75 | 1.26 | 0.52 | 0.81 | -3.17E-04 | -6.25E-03 | 2.02E-02 | 6.91E-03 | -2.37E-03 | 2.80E-03 |
| K02039$_{VIP\geq0.8}$ | 1.20 | 0.83 | 0.28 | 0.56 | 0.90 | 1.09 | -6.42E-04 | 3.30E-03 | -2.42E-03 | -2.67E-03 | 6.05E-03 | 2.41E-04 |
| K06940$_{VIP\geq0.8}$ | 1.52 | 0.55 | 1.30 | 1.05 | 0.69 | 0.92 | -1.14E-02 | 2.25E-03 | -1.45E-02 | -6.53E-03 | 2.55E-03 | 2.89E-03 |
| K00941$_{VIP\geq0.8}$ | 1.23 | 0.62 | 0.77 | 0.82 | 1.14 | 1.16 | 1.75E-02 | 3.83E-03 | -7.49E-03 | 5.79E-03 | -3.03E-03 | -8.56E-04 |
| K02037$_{VIP\geq0.8}$ | 1.00 | 0.85 | 0.36 | 0.59 | 0.89 | 0.99 | 3.88E-03 | -6.55E-04 | 3.28E-03 | -3.03E-03 | 5.87E-03 | -9.58E-04 |
| K02026$_{VIP\geq0.8}$ | 0.90 | 0.73 | 1.05 | 0.24 | 1.02 | 1.36 | 3.21E-03 | 7.79E-04 | -4.07E-03 | -6.83E-04 | -4.62E-03 | 6.72E-03 |
| K03643$_{VIP\geq0.8}$ | 0.33 | 1.30 | 1.29 | 1.18 | 1.17 | 0.26 | -1.98E-03 | 4.64E-03 | -7.29E-05 | -1.42E-04 | 6.93E-03 | 1.60E-03 |
| K04751$_{VIP\geq0.8}$ | 0.83 | 0.46 | 0.99 | 0.77 | 0.92 | 0.99 | 1.24E-02 | 1.74E-04 | -7.72E-03 | -3.95E-04 | 5.71E-03 | -2.79E-04 |
| K16785$_{VIP\geq0.8}$ | 1.09 | 0.83 | 0.72 | 0.71 | 1.25 | 1.11 | 8.81E-03 | -3.13E-04 | 2.94E-04 | 2.73E-03 | -4.73E-03 | -3.57E-04 |
| K12994$_{VIP\geq0.8}$ | 1.04 | 1.36 | 0.27 | 0.76 | 0.81 | 0.87 | 2.09E-03 | 1.09E-02 | -1.26E-03 | -4.47E-03 | -1.64E-03 | 5.28E-04 |
| K07444$_{VIP\geq0.8}$ | 0.58 | 0.86 | 1.01 | 0.82 | 0.53 | 1.01 | -2.94E-03 | 4.15E-05 | -1.06E-02 | -1.47E-04 | 2.32E-03 | 7.83E-03 |
| K11720$_{VIP\geq0.8}$ | 1.17 | 1.41 | 1.54 | 2.07 | 0.63 | 0.30 | 3.20E-03 | -9.55E-03 | -1.50E-02 | -9.30E-03 | 4.68E-04 | 1.34E-03 |
| K03973$_{VIP\geq0.8}$ | 1.27 | 0.47 | 0.22 | 1.04 | 0.99 | 1.36 | 3.10E-03 | -3.58E-03 | 2.06E-03 | -7.26E-03 | -5.74E-03 | 9.53E-03 |
| K10536$_{VIP\geq0.8}$ | 1.54 | 1.07 | 0.14 | 0.87 | 0.72 | 1.14 | 1.24E-02 | 5.91E-03 | -1.53E-03 | -9.40E-04 | -2.05E-03 | 7.03E-03 |
| K06076$_{VIP\geq0.8}$ | 1.16 | 0.85 | 0.56 | 0.77 | 0.95 | 1.07 | -9.78E-04 | 5.83E-03 | 5.66E-03 | 9.29E-05 | -5.68E-05 | -6.66E-03 |
| K01218$_{VIP\geq0.8}$ | 0.93 | 0.93 | 0.45 | 0.84 | 1.04 | 0.42 | -5.59E-03 | 6.66E-03 | -4.33E-03 | -1.62E-04 | 3.97E-03 | 3.93E-04 |
| K21908$_{VIP\geq0.8}$ | 0.71 | 1.17 | 0.89 | 1.15 | 0.56 | 1.30 | -7.22E-03 | 9.34E-03 | -8.81E-04 | 6.42E-03 | -3.43E-03 | 1.00E-02 |
| K00962$_{VIP\geq0.8}$ | 1.87 | 0.98 | 1.08 | 0.29 | 0.48 | 0.85 | -1.90E-02 | 4.59E-04 | 1.06E-02 | -1.54E-03 | -2.59E-03 | 4.13E-03 |
| K04079$_{VIP\geq0.8}$ | 0.46 | 0.94 | 1.11 | 1.08 | 0.93 | 0.58 | -6.92E-03 | -2.15E-04 | 4.50E-03 | 6.94E-03 | -2.44E-03 | 3.10E-04 |
| K22719$_{VIP\geq0.8}$ | 0.55 | 1.29 | 0.94 | 1.53 | 0.76 | 1.04 | -5.81E-04 | 4.34E-03 | 1.07E-02 | -3.43E-03 | 4.34E-03 | -6.89E-03 |
| K00765$_{VIP\geq0.8}$ | 0.22 | 1.11 | 0.97 | 0.51 | 1.42 | 1.12 | -2.91E-03 | 4.31E-04 | 4.90E-03 | -3.22E-03 | -8.97E-03 | 6.57E-03 |
| K03286$_{VIP\geq0.8}$ | 1.47 | 1.61 | 0.63 | 1.85 | 0.80 | 0.72 | 7.10E-03 | -1.28E-02 | -3.10E-03 | -1.19E-02 | 2.44E-03 | 1.54E-04 |
| K00927$_{VIP\geq0.8}$ | 0.92 | 1.37 | 0.89 | 0.05 | 0.46 | 1.17 | -4.09E-03 | -5.38E-03 | 3.81E-03 | 1.63E-04 | -3.08E-03 | 5.07E-03 |
| K01890$_{VIP\geq0.8}$ | 1.09 | 1.51 | 0.99 | 0.33 | 0.33 | 0.91 | -8.30E-03 | 3.73E-03 | 7.34E-03 | -1.35E-03 | 2.21E-03 | -6.16E-04 |
| K03654$_{VIP\geq0.8}$ | 0.71 | 1.11 | 1.01 | 0.67 | 0.81 | 0.87 | -1.84E-03 | 5.48E-03 | 7.79E-03 | 1.42E-03 | -2.38E-03 | -6.47E-03 |

| | | | | | | | | | | | | |
|---|---|---|---|---|---|---|---|---|---|---|---|---|
| $K02493_{VIP≥0.8}$ | 0.76 | 1.06 | 0.64 | 1.51 | 1.08 | 1.38 | 1.02E-02 | 7.86E-03 | -3.42E-03 | -1.01E-02 | -4.12E-03 | 9.68E-03 |
| $K19334_{VIP≥0.8}$ | 1.10 | 1.33 | 1.25 | 1.49 | 0.71 | 0.27 | -1.62E-02 | 1.95E-03 | -1.07E-05 | 1.54E-03 | 3.98E-03 | -7.15E-04 |
| $K07391_{VIP≥0.8}$ | 0.30 | 1.33 | 1.03 | 0.22 | 1.35 | 1.16 | -1.84E-03 | 5.51E-03 | 4.96E-03 | -1.68E-06 | -5.75E-03 | 6.10E-03 |
| $K03572_{VIP≥0.8}$ | 0.85 | 1.24 | 0.86 | 0.28 | 0.65 | 0.93 | -7.90E-03 | -8.74E-04 | 9.34E-03 | 1.75E-03 | 3.62E-03 | -1.39E-03 |
| $K00969_{VIP≥0.8}$ | 0.60 | 1.50 | 0.88 | 1.80 | 0.47 | 0.81 | 2.35E-03 | 6.86E-03 | -9.91E-03 | -1.16E-02 | 3.06E-03 | -3.60E-03 |
| $K00677_{VIP≥0.8}$ | 0.94 | 0.95 | 0.64 | 1.39 | 0.97 | 0.62 | 3.39E-05 | -1.74E-04 | -1.55E-03 | -1.98E-03 | 3.13E-03 | 4.41E-03 |
| $K00705_{VIP≥0.8}$ | 1.02 | 1.60 | 2.02 | 0.47 | 1.22 | 0.33 | -1.46E-02 | 1.16E-02 | 1.78E-02 | 3.14E-03 | -6.89E-03 | -1.64E-03 |
| $K02904_{VIP≥0.8}$ | 0.86 | 1.20 | 0.88 | 0.68 | 0.74 | 0.88 | 1.02E-02 | -9.52E-03 | 5.32E-03 | -4.22E-03 | -1.00E-03 | 3.06E-03 |
| $K03531_{VIP≥0.8}$ | 0.21 | 1.01 | 0.81 | 1.07 | 0.54 | 0.99 | -1.59E-03 | 4.16E-03 | 6.54E-03 | -4.26E-03 | 2.63E-03 | -6.46E-03 |
| $K07001_{VIP≥0.8}$ | 0.75 | 0.99 | 1.37 | 0.48 | 0.86 | 1.42 | -1.13E-02 | 5.82E-03 | -1.57E-02 | -1.49E-03 | 3.75E-04 | 1.08E-02 |
| $K19092_{VIP≥0.8}$ | 0.31 | 0.47 | 1.19 | 1.20 | 0.99 | 0.98 | 3.44E-03 | 4.73E-05 | -7.32E-03 | -8.02E-03 | -1.21E-03 | 1.51E-03 |
| $K02030_{VIP≥0.8}$ | 1.10 | 1.56 | 0.60 | 0.46 | 1.29 | 1.37 | 2.58E-03 | 1.24E-02 | -3.57E-03 | -3.28E-03 | -5.12E-03 | 6.40E-03 |
| $K07133_{VIP≥0.8}$ | 0.28 | 1.08 | 1.01 | 1.16 | 0.59 | 0.99 | -1.36E-03 | -1.13E-04 | 8.46E-03 | -5.56E-03 | 3.91E-03 | 3.38E-03 |
| K14092 | 0.99 | 1.06 | 1.45 | 0.42 | 0.30 | 0.78 | -1.47E-02 | 7.33E-03 | 1.20E-02 | -1.66E-03 | 1.56E-03 | 5.44E-03 |
| K14095 | 0.41 | 0.95 | 0.92 | 0.80 | 0.75 | 0.54 | -2.83E-03 | 6.11E-03 | 3.19E-03 | -3.79E-03 | 4.94E-03 | 3.60E-03 |
| K03053 | 0.76 | 0.83 | 1.28 | 0.99 | 0.20 | 0.76 | -8.84E-03 | 6.35E-03 | 1.15E-02 | 4.13E-03 | 9.61E-04 | 5.76E-03 |
| K05566 | 0.57 | 0.89 | 0.43 | 0.88 | 1.91 | 0.49 | -7.60E-03 | -3.42E-03 | 4.65E-03 | -5.91E-03 | -1.25E-02 | 3.23E-03 |
| K03050 | 0.90 | 0.55 | 0.76 | 1.09 | 0.28 | 0.84 | -3.75E-03 | 1.63E-03 | 5.07E-03 | 6.00E-03 | 1.85E-03 | 6.10E-03 |
| K10212 | 0.53 | 0.52 | 1.43 | 1.90 | 0.72 | 1.23 | 4.89E-03 | 2.64E-03 | 1.24E-02 | 1.20E-02 | 2.90E-03 | 7.88E-03 |
| K21993 | 0.88 | 0.87 | 0.69 | 0.93 | 0.65 | 0.22 | 4.84E-03 | 5.73E-03 | -2.85E-03 | 1.08E-04 | 1.81E-03 | -1.56E-03 |
| K00441 | 0.63 | 1.23 | 0.92 | 0.48 | 0.71 | 2.04 | 9.16E-03 | 9.50E-03 | -9.87E-03 | -3.71E-04 | 4.36E-03 | 1.57E-02 |
| K13653 | 0.92 | 0.55 | 0.63 | 0.61 | 1.70 | 0.95 | -4.08E-03 | -2.77E-04 | 5.03E-03 | -1.69E-03 | -1.15E-02 | -7.21E-03 |
| K02822 | 0.68 | 1.03 | 0.96 | 0.57 | 0.79 | 1.08 | 7.10E-03 | 7.05E-03 | -8.94E-03 | -4.00E-03 | -3.74E-03 | -2.66E-03 |
| K02019 | 0.73 | 0.74 | 0.88 | 0.97 | 1.76 | 0.27 | 8.20E-03 | -3.82E-03 | -5.98E-03 | -5.07E-03 | -1.18E-02 | 1.60E-03 |
| K06962 | 0.71 | 1.11 | 1.03 | 0.73 | 0.73 | 0.93 | 6.00E-04 | 4.61E-03 | 1.09E-02 | 4.69E-03 | -1.89E-03 | 5.85E-03 |
| K01518 | 0.27 | 0.43 | 1.00 | 0.87 | 0.44 | 0.96 | -3.55E-03 | 1.10E-03 | 4.76E-03 | -4.36E-03 | -3.95E-04 | -6.36E-03 |
| K00641 | 0.64 | 0.61 | 1.45 | 0.87 | 0.40 | 1.13 | -9.48E-03 | 4.85E-03 | 1.46E-02 | 1.40E-03 | 1.46E-04 | 8.67E-03 |
| K09706 | 1.07 | 0.55 | 0.72 | 1.33 | 0.65 | 1.43 | -6.00E-03 | 9.51E-05 | 7.92E-03 | 7.35E-03 | -3.46E-03 | 1.03E-02 |
| K06295 | 0.65 | 0.66 | 1.29 | 0.79 | 1.04 | 1.20 | -8.29E-03 | 5.01E-03 | 1.03E-02 | -1.34E-03 | -5.24E-03 | -7.57E-03 |
| K11936 | 0.26 | 1.33 | 1.52 | 1.60 | 0.79 | 0.53 | 1.21E-03 | 4.77E-03 | 1.76E-02 | 1.04E-02 | -1.12E-03 | 3.12E-03 |
| K06396 | 0.61 | 0.71 | 1.26 | 1.41 | 1.11 | 0.72 | -8.62E-03 | 4.77E-03 | 1.18E-02 | 5.21E-03 | -2.98E-03 | 5.74E-05 |
| K16927 | 0.80 | 0.63 | 1.23 | 1.06 | 0.72 | 1.09 | 1.07E-02 | 4.62E-03 | 1.25E-02 | -7.55E-03 | -3.72E-03 | 5.83E-03 |

| | | | | | | | | | | | | |
|---|---|---|---|---|---|---|---|---|---|---|---|---|
| K04072 | 0.09 | 1.14 | 2.32 | 0.78 | 1.40 | 0.44 | 1.09E-03 | 8.91E-03 | 2.14E-02 | 1.23E-03 | -7.76E-03 | -3.36E-03 |
| K00887 | 0.88 | 1.05 | 0.35 | 0.34 | 0.50 | 1.14 | 4.45E-03 | 7.67E-03 | 1.18E-03 | 1.75E-03 | 1.66E-03 | -1.15E-03 |
| K02651 | 0.24 | 0.64 | 0.28 | 1.00 | 2.05 | 1.01 | 2.51E-03 | 4.83E-03 | -2.12E-03 | 6.72E-03 | -1.36E-02 | 7.81E-03 |
| K01975 | 0.85 | 0.64 | 0.54 | 0.88 | 1.57 | 0.80 | -5.25E-05 | 2.91E-04 | 5.38E-03 | -2.52E-03 | -1.02E-02 | 6.17E-03 |
| K05341 | 0.62 | 0.77 | 1.53 | 0.64 | 1.59 | 1.09 | 3.43E-03 | 6.10E-03 | 1.52E-02 | 3.34E-03 | -7.70E-03 | 3.39E-03 |
| K01496 | 0.33 | 1.49 | 1.78 | 1.14 | 0.45 | 0.52 | 4.83E-03 | 1.14E-02 | 1.88E-02 | 4.20E-03 | 9.70E-04 | -2.73E-03 |
| K04034 | 1.06 | 2.18 | 0.40 | 0.72 | 0.26 | 1.32 | -2.56E-03 | 1.68E-02 | 2.82E-04 | -1.43E-03 | 6.14E-04 | 9.42E-03 |
| K06198 | 1.65 | 1.33 | 0.98 | 0.64 | 0.53 | 0.57 | 2.13E-02 | 1.04E-02 | 9.00E-03 | -4.46E-03 | -1.24E-03 | 1.16E-03 |
| K01646 | 0.26 | 0.64 | 1.06 | 0.27 | 0.98 | 1.72 | -3.38E-03 | 4.94E-03 | -1.19E-02 | -4.35E-04 | 5.03E-03 | 1.30E-02 |
| K03708 | 0.96 | 0.68 | 0.66 | 0.80 | 1.67 | 0.85 | 1.19E-02 | -5.45E-03 | 7.66E-03 | 9.81E-04 | -9.66E-03 | -1.49E-03 |
| K07502 | 1.24 | 1.73 | 0.33 | 0.75 | 0.70 | 0.98 | 2.59E-04 | 1.35E-02 | 2.25E-03 | 4.45E-03 | 3.92E-03 | 3.85E-03 |
| K13684 | 0.56 | 2.00 | 0.63 | 0.13 | 2.77 | 0.90 | 3.60E-04 | 1.55E-02 | 1.67E-03 | -6.27E-04 | 1.87E-02 | 6.89E-03 |
| K04088 | 0.49 | 1.34 | 1.98 | 1.08 | 0.53 | 0.55 | 5.87E-03 | -1.06E-02 | -1.71E-02 | -6.67E-03 | 3.13E-03 | -3.10E-03 |
| K07794 | 0.67 | 1.12 | 0.74 | 0.90 | 0.67 | 1.29 | -8.28E-03 | -7.99E-03 | -7.85E-03 | -5.38E-03 | -9.00E-04 | 9.94E-03 |
| K22300 | 0.40 | 0.56 | 2.04 | 1.01 | 1.68 | 0.32 | -5.61E-03 | 2.97E-03 | -2.18E-02 | -4.34E-03 | -8.47E-03 | -4.02E-04 |
| K08972 | 0.37 | 2.45 | 1.35 | 0.86 | 0.56 | 0.73 | -2.22E-04 | 1.81E-02 | -2.94E-03 | -3.62E-03 | -2.03E-03 | 4.70E-03 |
| K06407 | 0.30 | 0.55 | 0.96 | 1.05 | 0.84 | 0.65 | -4.38E-03 | 5.60E-04 | 5.47E-03 | 4.18E-04 | -2.90E-03 | -1.38E-03 |
| K09124 | 0.66 | 0.52 | 1.25 | 1.21 | 0.42 | 2.51 | -6.88E-03 | 3.27E-03 | 1.45E-02 | 7.29E-03 | 2.44E-03 | 1.90E-02 |
| K01085 | 1.70 | 0.77 | 0.40 | 1.49 | 0.40 | 1.04 | 1.46E-02 | -1.23E-03 | 6.99E-04 | -1.01E-02 | -2.21E-03 | -6.50E-03 |
| K06933 | 0.94 | 0.53 | 0.72 | 0.58 | 0.92 | 1.22 | -1.70E-03 | -4.17E-03 | -1.24E-03 | 3.07E-03 | -4.53E-03 | 5.15E-03 |
| K07148 | 1.30 | 0.72 | 1.02 | 0.62 | 0.69 | 0.93 | 6.27E-03 | 5.47E-03 | 9.49E-03 | -3.50E-04 | -2.60E-03 | -5.59E-03 |
| K04047 | 0.78 | 0.51 | 1.07 | 0.15 | 1.31 | 0.90 | 2.68E-04 | 9.83E-04 | 9.62E-03 | 9.87E-05 | 7.85E-03 | -1.21E-03 |
| K13018 | 1.40 | 0.95 | 0.41 | 0.79 | 0.53 | 0.94 | 5.73E-03 | -5.72E-03 | -4.57E-03 | -4.77E-03 | -3.60E-03 | 3.43E-03 |
| K05364 | 1.27 | 1.48 | 0.20 | 0.24 | 0.71 | 1.28 | -2.49E-04 | 1.11E-02 | 5.70E-04 | 1.48E-03 | 2.00E-03 | -6.78E-03 |
| K07080 | 1.12 | 0.57 | 1.17 | 0.33 | 0.56 | 1.01 | 1.53E-02 | 4.58E-04 | -1.26E-02 | -1.79E-03 | -2.05E-03 | 4.09E-03 |
| K09769 | 0.39 | 0.59 | 1.36 | 0.78 | 1.63 | 0.91 | 2.87E-03 | -4.57E-03 | -8.80E-03 | 2.04E-03 | -7.91E-03 | 4.05E-03 |
| K22477 | 0.30 | 0.38 | 1.14 | 1.08 | 0.84 | 0.68 | 2.96E-03 | 8.18E-04 | 3.95E-03 | 3.95E-04 | -2.12E-03 | -4.97E-03 |
| K04656 | 1.17 | 1.61 | 0.33 | 0.47 | 0.75 | 1.35 | 1.42E-02 | 1.08E-02 | -2.62E-03 | 3.08E-03 | -2.49E-04 | 5.88E-03 |
| K03639 | 1.07 | 1.04 | 0.69 | 0.62 | 0.58 | 1.56 | 1.15E-02 | 8.35E-03 | 7.44E-03 | 1.56E-03 | -7.03E-04 | 1.13E-02 |
| K04094 | 0.73 | 0.74 | 1.00 | 0.34 | 1.67 | 0.89 | 2.48E-03 | -3.99E-03 | 3.93E-03 | 2.62E-04 | -7.84E-03 | 1.78E-03 |
| K05794 | 0.93 | 0.88 | 0.60 | 1.54 | 0.67 | 0.76 | 5.55E-03 | 2.96E-03 | -4.39E-03 | -9.02E-03 | -3.89E-03 | -8.36E-04 |
| K07192 | 0.80 | 0.62 | 0.32 | 0.93 | 1.07 | 0.83 | 8.14E-03 | 4.58E-03 | -6.46E-05 | -5.12E-03 | -7.20E-03 | -6.28E-03 |
| K09803 | 0.35 | 1.76 | 1.02 | 0.91 | 0.51 | 0.72 | 5.04E-03 | 1.24E-02 | 9.13E-04 | 2.31E-03 | 1.05E-03 | 4.84E-03 |
| K02283 | 1.16 | 2.06 | 0.53 | 0.68 | 0.65 | 0.96 | 1.19E-02 | 1.63E-02 | -4.71E-03 | 4.39E-03 | 1.74E-03 | -1.40E-03 |

| | | | | | | | | | | | | |
|---|---|---|---|---|---|---|---|---|---|---|---|---|
| K10532 | 0.76 | 0.71 | 1.46 | 1.07 | 0.45 | 0.99 | 6.97E-03 | -2.32E-03 | -1.57E-02 | -5.94E-03 | -2.50E-03 | 3.77E-03 |
| K09707 | 1.48 | 0.41 | 0.87 | 0.60 | 1.56 | 0.19 | 1.63E-02 | -7.45E-04 | -3.55E-03 | -4.20E-03 | -6.45E-03 | -1.44E-03 |
| K02456 | 0.49 | 1.32 | 1.44 | 0.94 | 0.74 | 0.66 | 6.50E-03 | -9.91E-03 | -7.80E-03 | -4.70E-03 | 2.08E-03 | 1.81E-03 |
| K06972 | 1.18 | 0.88 | 0.61 | 0.24 | 0.69 | 1.18 | 1.29E-03 | 3.15E-03 | 3.24E-03 | 1.57E-03 | -1.04E-03 | -3.50E-03 |
| K06209 | 1.12 | 0.39 | 0.26 | 0.41 | 0.96 | 1.04 | 6.96E-03 | -2.30E-04 | -2.63E-03 | 1.74E-03 | -6.72E-04 | 1.03E-03 |
| K15772 | 0.86 | 0.29 | 0.38 | 0.97 | 1.08 | 0.60 | 2.30E-03 | 2.17E-04 | 1.59E-03 | 1.99E-03 | -4.78E-04 | -2.32E-03 |
| K01919 | 0.16 | 1.16 | 0.92 | 0.72 | 0.79 | 1.04 | 1.97E-03 | 8.59E-03 | -1.04E-02 | -3.91E-03 | 2.11E-03 | -6.42E-03 |
| K08963 | 1.16 | 1.22 | 0.64 | 0.37 | 0.78 | 1.46 | 3.45E-03 | 3.67E-03 | 8.91E-04 | 2.55E-03 | 3.96E-03 | 7.63E-03 |
| K01267 | 0.87 | 0.42 | 0.47 | 0.12 | 0.92 | 1.04 | 7.89E-03 | -8.57E-04 | -6.81E-04 | -8.29E-04 | 1.40E-03 | -2.55E-03 |
| K22452 | 1.63 | 0.46 | 0.61 | 1.13 | 0.61 | 1.22 | -1.31E-02 | -1.38E-04 | -5.74E-04 | -6.63E-03 | 1.65E-03 | -7.08E-03 |
| K07089 | 2.02 | 0.51 | 0.59 | 0.84 | 1.36 | 0.74 | 2.42E-02 | 2.58E-03 | -3.68E-03 | -5.98E-03 | -9.17E-03 | -3.45E-03 |
| K09121 | 1.13 | 1.47 | 0.64 | 0.33 | 0.50 | 1.31 | 2.45E-03 | 9.15E-03 | -1.36E-03 | 1.49E-03 | 7.93E-04 | 6.34E-03 |
| K07736 | 1.08 | 0.41 | 0.70 | 0.79 | 1.44 | 1.03 | 1.14E-02 | -3.30E-03 | 9.47E-04 | 2.17E-04 | -2.18E-03 | 3.92E-03 |
| K03742 | 0.07 | 1.23 | 1.54 | 1.25 | 0.43 | 0.57 | 1.08E-03 | -7.88E-03 | 7.49E-03 | -8.23E-03 | 2.78E-03 | 1.83E-03 |
| K01155 | 1.32 | 1.45 | 0.88 | 0.36 | 0.38 | 0.40 | -1.76E-02 | 9.98E-03 | -8.73E-03 | 1.19E-03 | -2.52E-03 | -2.38E-03 |
| K00283 | 1.14 | 1.58 | 0.73 | 0.15 | 0.50 | 1.12 | -1.38E-02 | 9.07E-03 | -7.71E-03 | 1.04E-03 | -2.01E-03 | 6.91E-03 |
| K16786 | 1.03 | 0.72 | 0.55 | 0.60 | 1.15 | 1.08 | 1.13E-02 | 3.08E-03 | -4.63E-03 | 9.17E-04 | -1.18E-04 | -1.84E-04 |
| K07099 | 1.02 | 0.33 | 0.73 | 0.78 | 1.84 | 1.04 | 1.92E-03 | 1.04E-03 | 4.94E-03 | 1.85E-05 | -8.75E-03 | 1.92E-03 |
| K19225 | 0.81 | 0.42 | 0.63 | 1.10 | 0.65 | 0.93 | 3.60E-03 | -4.46E-04 | -6.78E-03 | 6.88E-03 | 1.85E-03 | -1.34E-03 |
| K19117 | 0.98 | 0.89 | 0.66 | 0.66 | 0.45 | 1.14 | 7.93E-03 | -5.02E-05 | -6.43E-03 | -1.65E-03 | 1.38E-03 | 5.31E-03 |
| K04655 | 1.12 | 1.63 | 0.26 | 0.54 | 0.71 | 1.31 | 2.61E-03 | 1.24E-02 | -1.36E-03 | 2.76E-03 | -1.66E-03 | 4.86E-03 |
| K01728 | 0.96 | 0.93 | 0.47 | 1.01 | 0.77 | 0.41 | 3.50E-03 | 2.44E-04 | -4.72E-03 | 6.12E-03 | 3.71E-04 | -3.06E-03 |
| K08309 | 1.05 | 0.53 | 0.24 | 0.15 | 1.30 | 1.09 | -4.49E-03 | -3.87E-03 | 3.37E-04 | -9.34E-04 | 4.41E-03 | -2.25E-03 |
| K05521 | 0.58 | 0.67 | 1.85 | 0.58 | 1.14 | 1.36 | 6.86E-03 | -3.40E-03 | -2.13E-02 | 2.86E-03 | -3.31E-03 | 8.50E-03 |
| K02217 | 0.71 | 0.71 | 0.77 | 1.04 | 0.85 | 1.90 | 8.59E-03 | 2.57E-03 | 8.03E-03 | 7.36E-03 | 5.51E-03 | 1.31E-02 |
| K03929 | 0.78 | 0.91 | 0.48 | 0.90 | 0.50 | 0.83 | 4.86E-03 | 6.71E-04 | -5.42E-03 | -2.46E-03 | 2.03E-03 | 6.37E-03 |
| K08722 | 0.96 | 0.39 | 0.51 | 0.33 | 1.04 | 1.00 | 1.88E-03 | 8.41E-05 | 1.76E-03 | -1.87E-03 | -1.52E-03 | -2.70E-03 |
| K01744 | 0.01 | 0.91 | 0.78 | 0.83 | 0.58 | 1.39 | -1.07E-04 | -5.92E-03 | 8.21E-03 | 5.65E-03 | 3.90E-03 | 1.02E-02 |
| K04042 | 0.98 | 0.83 | 0.30 | 0.70 | 0.46 | 0.82 | 1.46E-03 | -6.03E-03 | -1.93E-03 | -4.01E-03 | 1.37E-03 | -2.27E-03 |
| K21903 | 0.95 | 0.53 | 0.06 | 0.94 | 0.69 | 1.14 | 7.00E-03 | -3.35E-03 | -1.51E-04 | -5.41E-03 | 1.20E-03 | 8.75E-04 |
| K18928 | 1.27 | 0.87 | 0.23 | 1.23 | 0.75 | 0.72 | 6.53E-03 | 6.73E-03 | -2.28E-03 | -4.79E-03 | -7.35E-04 | -3.56E-03 |
| K03321 | 0.68 | 0.57 | 1.10 | 0.95 | 0.71 | 1.32 | -2.58E-03 | 2.62E-03 | 9.00E-03 | -6.68E-03 | 1.10E-03 | -9.27E-03 |
| K19159 | 0.48 | 0.70 | 1.28 | 0.86 | 0.80 | 0.96 | -4.58E-03 | -1.51E-03 | 4.44E-04 | -3.96E-03 | 4.70E-03 | -3.97E-03 |
| K06885 | 1.08 | 0.63 | 1.04 | 0.18 | 1.07 | 0.79 | -2.22E-03 | -4.94E-03 | -1.10E-02 | -8.31E-04 | -3.31E-03 | 4.37E-04 |

| | | | | | | | | | | | | |
|---|---|---|---|---|---|---|---|---|---|---|---|---|
| K21395 | 0.91 | 0.41 | 1.09 | 0.45 | 0.63 | 1.15 | 5.22E-03 | 1.38E-03 | -5.71E-03 | 2.97E-03 | -3.39E-03 | 6.03E-03 |
| K03606 | 0.75 | 1.32 | 0.97 | 0.55 | 0.34 | 1.57 | 3.83E-04 | 8.42E-03 | 7.85E-03 | 2.61E-03 | 1.84E-03 | 1.06E-02 |
| K07792 | 0.90 | 0.61 | 0.05 | 0.80 | 1.39 | 0.29 | -5.58E-04 | -4.31E-03 | 4.63E-04 | 1.83E-03 | 4.71E-03 | -2.10E-04 |
| K21029 | 0.87 | 0.70 | 0.28 | 0.69 | 1.58 | 1.39 | 7.15E-04 | 5.26E-03 | -3.22E-03 | -1.13E-03 | 1.07E-02 | -1.04E-02 |
| K01119 | 0.11 | 1.43 | 2.25 | 1.30 | 0.39 | 0.49 | -1.05E-03 | 7.75E-03 | -2.57E-02 | 6.32E-04 | 2.24E-03 | 1.93E-03 |
| K18908 | 1.51 | 0.61 | 0.29 | 0.33 | 1.00 | 1.41 | -8.13E-03 | -4.81E-04 | -7.79E-04 | -2.33E-03 | 6.19E-03 | -5.51E-03 |
| K06177 | 0.81 | 0.86 | 0.34 | 0.92 | 0.39 | 0.64 | 7.19E-03 | -5.70E-03 | 1.87E-03 | 2.75E-03 | -6.50E-04 | -1.54E-03 |
| K00027 | 0.04 | 0.73 | 0.62 | 1.51 | 0.87 | 1.07 | 3.13E-05 | -4.44E-03 | 3.67E-03 | 5.67E-03 | 4.51E-04 | 2.01E-03 |
| K23536 | 1.56 | 0.62 | 0.86 | 0.31 | 0.73 | 1.54 | -9.52E-03 | 2.05E-03 | -6.38E-03 | -1.61E-04 | -2.23E-03 | 9.60E-03 |
| K03216 | 1.00 | 0.41 | 0.51 | 0.87 | 1.94 | 0.74 | 4.55E-03 | -1.34E-03 | 3.80E-04 | 1.19E-03 | -8.69E-03 | 3.49E-04 |
| K00991 | 0.99 | 0.44 | 0.63 | 0.64 | 1.04 | 1.11 | 1.24E-02 | 2.24E-03 | 4.13E-03 | 3.35E-03 | 2.30E-03 | -2.31E-03 |
| K00788 | 0.76 | 1.81 | 0.66 | 0.52 | 1.18 | 1.50 | 1.02E-02 | 1.45E-02 | -4.59E-03 | 3.00E-03 | -2.21E-03 | 9.43E-03 |
| K11145 | 1.01 | 0.21 | 0.54 | 0.72 | 1.56 | 1.14 | 2.93E-03 | 1.10E-03 | -1.17E-03 | 9.01E-04 | -3.26E-03 | 6.69E-05 |
| K06975 | 0.61 | 1.33 | 0.71 | 0.11 | 0.81 | 1.33 | 9.21E-03 | -1.06E-02 | 7.26E-03 | -4.72E-04 | 3.77E-03 | 8.91E-03 |
| K07742 | 0.38 | 0.57 | 0.81 | 0.76 | 1.97 | 0.93 | -1.65E-04 | -3.75E-03 | -1.07E-03 | 6.27E-04 | -9.95E-03 | -1.71E-03 |
| K15894 | 2.05 | 0.49 | 2.13 | 1.38 | 0.72 | 0.14 | -2.87E-02 | 8.13E-04 | -2.27E-02 | -6.72E-03 | 4.68E-03 | -8.58E-04 |
| K03789 | 0.94 | 0.46 | 0.55 | 0.99 | 1.76 | 0.59 | 8.98E-03 | -2.32E-03 | -2.44E-03 | 3.50E-03 | -6.68E-03 | 1.64E-03 |
| K03151 | 1.21 | 0.70 | 0.19 | 0.15 | 1.02 | 0.94 | -1.95E-03 | 4.15E-03 | -1.05E-03 | 3.69E-04 | 3.40E-04 | 2.73E-03 |
| K00058 | 1.02 | 0.59 | 0.65 | 0.70 | 1.29 | 1.12 | 1.95E-03 | 8.68E-04 | -7.54E-03 | -4.36E-03 | 7.62E-03 | -3.49E-03 |
| K03976 | 0.25 | 0.71 | 0.88 | 0.88 | 1.68 | 0.37 | 2.08E-03 | -4.93E-03 | 7.66E-03 | 1.73E-03 | -1.12E-02 | -1.21E-03 |
| K05601 | 1.04 | 1.01 | 0.63 | 0.06 | 0.38 | 0.99 | 5.94E-03 | 4.82E-03 | -7.08E-03 | -4.55E-04 | -2.55E-03 | 3.46E-03 |
| K00340 | 1.37 | 0.81 | 0.54 | 0.86 | 0.71 | 0.68 | 6.98E-03 | 4.61E-03 | 5.71E-03 | 5.16E-04 | 2.69E-03 | 3.34E-03 |
| K02036 | 1.01 | 0.77 | 0.15 | 0.51 | 0.98 | 1.06 | 3.75E-03 | 1.33E-03 | 1.62E-03 | -1.81E-03 | 6.41E-03 | -1.12E-03 |
| K24180 | 0.23 | 1.06 | 1.97 | 0.72 | 0.76 | 1.06 | -3.48E-03 | 7.32E-03 | -2.24E-02 | -5.06E-03 | -4.57E-03 | 7.02E-03 |
| K07105 | 1.06 | 0.76 | 0.12 | 0.43 | 1.33 | 1.10 | 4.01E-03 | 4.57E-03 | -4.09E-04 | 1.80E-04 | 5.65E-03 | -5.06E-04 |
| K09772 | 0.97 | 0.31 | 0.56 | 0.63 | 1.53 | 1.14 | 5.91E-03 | -6.66E-04 | 2.00E-03 | 9.54E-04 | -5.26E-03 | 9.91E-04 |
| K00980 | 0.55 | 0.85 | 1.02 | 0.10 | 0.97 | 0.59 | -3.86E-04 | 3.92E-03 | 1.01E-02 | -2.84E-04 | -5.27E-03 | -3.83E-03 |
| K00761 | 0.99 | 0.67 | 0.73 | 0.79 | 1.37 | 1.19 | 2.21E-04 | 5.27E-03 | 1.79E-04 | 1.11E-03 | -3.80E-03 | 7.05E-03 |
| K03523 | 1.05 | 0.71 | 0.61 | 0.52 | 1.51 | 1.25 | 5.67E-03 | 3.77E-04 | -1.37E-03 | 8.26E-04 | -4.44E-03 | 3.04E-03 |
| K03091 | 0.62 | 0.50 | 1.26 | 1.12 | 1.34 | 0.78 | -7.44E-03 | 2.73E-03 | 6.60E-03 | 1.59E-03 | -5.85E-03 | -1.40E-03 |
| K00567 | 1.11 | 1.26 | 0.55 | 0.34 | 0.73 | 1.09 | -6.68E-03 | -8.67E-03 | 4.87E-03 | -2.18E-03 | 4.90E-03 | 4.10E-03 |
| K06167 | 1.43 | 0.96 | 0.65 | 0.89 | 0.78 | 0.67 | 9.70E-03 | 6.68E-03 | 7.30E-03 | -2.08E-03 | -3.05E-03 | 2.74E-03 |
| K02072 | 1.08 | 0.46 | 0.42 | 0.58 | 1.23 | 1.42 | 7.68E-03 | -1.26E-03 | -4.91E-03 | -4.10E-03 | -4.88E-03 | -5.50E-03 |
| K03621 | 1.01 | 0.54 | 0.27 | 0.49 | 1.32 | 1.15 | 1.01E-02 | -3.48E-03 | 3.07E-03 | -3.47E-03 | -5.37E-03 | -1.96E-03 |

| | | | | | | | | | | | | |
|---|---|---|---|---|---|---|---|---|---|---|---|---|
| K03704 | 0.88 | 0.49 | 0.62 | 0.87 | 0.52 | 1.23 | 2.19E-03 | -8.34E-04 | -5.29E-03 | -5.23E-03 | 2.41E-05 | -2.60E-03 |
| K07102 | 0.87 | 0.77 | 0.70 | 1.66 | 1.54 | 0.40 | -2.80E-03 | -7.86E-05 | 6.61E-03 | -1.98E-03 | 6.73E-03 | 2.27E-03 |
| K00703 | 1.14 | 0.72 | 0.24 | 0.54 | 1.07 | 1.31 | -5.35E-03 | -5.73E-03 | -1.13E-03 | -3.10E-03 | 2.07E-03 | 7.52E-03 |
| K04083 | 1.02 | 0.60 | 0.35 | 0.58 | 0.89 | 1.13 | 5.55E-03 | -3.21E-03 | -3.96E-03 | -3.76E-03 | 8.03E-06 | -3.16E-03 |
| K06958 | 1.07 | 0.69 | 0.15 | 0.39 | 0.82 | 1.17 | -8.17E-04 | -4.42E-03 | 1.32E-04 | -2.75E-03 | -2.87E-03 | -1.46E-03 |
| K07037 | 0.94 | 0.43 | 0.46 | 0.36 | 1.31 | 1.13 | 1.74E-03 | 4.84E-04 | 3.21E-03 | 8.35E-04 | -7.11E-03 | 7.47E-03 |
| K00683 | 1.36 | 0.55 | 0.56 | 0.78 | 0.99 | 2.03 | 6.79E-03 | -6.54E-04 | 4.62E-03 | 5.37E-03 | 4.91E-03 | 1.46E-02 |
| K07164 | 1.34 | 0.26 | 1.12 | 0.25 | 0.73 | 1.18 | 4.83E-03 | -7.22E-04 | 1.22E-02 | 4.19E-04 | -4.12E-03 | 4.58E-03 |
| K08679 | 0.65 | 1.31 | 0.85 | 1.31 | 0.66 | 0.53 | -9.82E-03 | -7.57E-03 | -4.45E-03 | 9.28E-03 | -1.56E-03 | 4.03E-03 |
| K02073 | 1.19 | 0.79 | 0.52 | 0.79 | 1.24 | 1.48 | 1.41E-02 | -5.91E-03 | -5.92E-03 | -5.35E-03 | -7.14E-03 | -6.49E-03 |
| K01009 | 1.08 | 0.21 | 0.47 | 1.00 | 0.62 | 0.86 | -4.01E-04 | 1.31E-03 | 4.32E-03 | -4.30E-03 | 4.50E-04 | -1.12E-03 |
| K01524 | 0.95 | 0.98 | 0.10 | 0.34 | 0.62 | 0.95 | 4.00E-03 | 2.13E-03 | -1.18E-03 | 3.15E-04 | 6.45E-04 | 1.44E-03 |
| K05808 | 1.23 | 0.40 | 0.16 | 0.40 | 1.16 | 1.16 | -3.47E-03 | -2.84E-03 | -1.44E-03 | -2.80E-03 | -2.86E-03 | -7.42E-04 |
| K00346 | 0.74 | 1.13 | 0.64 | 1.53 | 1.01 | 0.27 | -2.61E-03 | -4.80E-03 | -4.09E-03 | -4.85E-03 | -4.52E-04 | 9.47E-04 |
| K03783 | 0.73 | 0.86 | 0.47 | 0.61 | 0.93 | 0.95 | 4.31E-03 | 4.10E-03 | 5.25E-03 | -1.24E-03 | -4.52E-03 | 7.23E-03 |
| K09922 | 1.58 | 0.47 | 0.90 | 1.90 | 0.75 | 0.72 | 8.38E-03 | -3.58E-03 | -7.16E-03 | -1.10E-02 | 3.66E-03 | 1.94E-03 |
| K04085 | 0.35 | 0.35 | 1.70 | 1.36 | 1.67 | 0.48 | 2.53E-03 | -2.64E-03 | -7.37E-03 | -7.12E-03 | 1.12E-02 | -2.86E-04 |
| K02557 | 0.39 | 1.36 | 1.35 | 1.03 | 0.51 | 0.35 | 2.32E-03 | 5.60E-03 | -3.70E-03 | 6.82E-04 | 3.23E-03 | 1.44E-04 |
| K03060 | 0.53 | 0.75 | 1.19 | 0.55 | 1.19 | 1.05 | 2.82E-03 | -5.65E-03 | -8.52E-03 | -3.92E-03 | -3.23E-03 | 1.06E-03 |
| K05807 | 1.26 | 0.35 | 0.62 | 1.58 | 1.11 | 0.73 | 3.13E-03 | -1.71E-03 | 7.41E-04 | -7.18E-03 | 3.71E-03 | 2.99E-03 |
| K04773 | 1.54 | 1.28 | 0.43 | 2.30 | 0.47 | 0.21 | 9.48E-03 | -6.61E-03 | -3.02E-03 | -1.26E-02 | -3.04E-03 | -1.48E-03 |
| K16694 | 0.80 | 0.72 | 0.51 | 1.29 | 0.91 | 1.26 | 8.11E-03 | 2.96E-03 | -1.54E-03 | -5.97E-03 | -1.57E-03 | 8.51E-03 |
| K01775 | 1.00 | 0.23 | 0.35 | 0.51 | 1.10 | 1.02 | 1.64E-03 | 6.32E-04 | -3.35E-03 | -3.54E-03 | -2.34E-03 | -1.51E-04 |
| K03614 | 0.83 | 0.55 | 0.06 | 0.58 | 1.22 | 0.83 | -6.03E-04 | -3.98E-03 | -7.47E-04 | 2.89E-03 | -8.26E-03 | 5.83E-03 |
| K02199 | 0.75 | 0.48 | 1.09 | 1.34 | 1.77 | 0.11 | -3.03E-03 | 1.18E-03 | -1.17E-02 | -8.70E-03 | -1.15E-02 | -8.10E-04 |
| K02784 | 0.88 | 0.77 | 0.35 | 0.61 | 1.55 | 0.91 | 7.90E-03 | 6.09E-03 | -4.10E-04 | 1.49E-03 | -5.98E-03 | 6.98E-04 |
| K00265 | 1.14 | 1.12 | 0.28 | 0.47 | 0.53 | 0.80 | -3.62E-03 | -2.53E-03 | -3.22E-03 | 7.48E-04 | 3.48E-03 | -3.82E-03 |
| K03521 | 0.42 | 2.10 | 1.10 | 0.45 | 0.31 | 0.91 | 4.17E-03 | 1.48E-02 | 8.11E-03 | 3.07E-03 | -1.30E-03 | 2.37E-03 |
| K15738 | 0.85 | 1.46 | 0.13 | 1.10 | 0.42 | 0.67 | -9.02E-03 | 7.08E-03 | -1.13E-03 | -1.87E-03 | -1.70E-04 | -4.75E-03 |
| K09747 | 0.64 | 0.71 | 0.89 | 0.57 | 1.46 | 0.96 | 5.92E-03 | -5.22E-03 | -1.03E-02 | -4.05E-03 | -3.83E-03 | 1.20E-03 |
| K01206 | 0.69 | 0.81 | 0.63 | 0.64 | 0.96 | 2.59 | -9.21E-03 | 1.28E-03 | 2.27E-03 | -3.37E-03 | 5.75E-03 | 1.99E-02 |
| K23242 | 0.20 | 0.23 | 1.08 | 1.26 | 1.53 | 0.60 | 3.05E-03 | -1.31E-03 | -1.04E-02 | -8.45E-03 | -7.83E-03 | 4.31E-03 |
| K00793 | 0.67 | 1.07 | 1.98 | 1.73 | 0.72 | 0.18 | -9.57E-04 | -6.19E-03 | -1.71E-02 | -8.25E-03 | 2.11E-03 | -5.16E-04 |
| K03771 | 1.28 | 0.57 | 0.68 | 1.94 | 0.98 | 0.73 | 3.51E-03 | -3.34E-03 | -5.06E-03 | -1.00E-02 | -1.22E-03 | 3.06E-03 |

| | | | | | | | | | | | | |
|---|---|---|---|---|---|---|---|---|---|---|---|---|
| K03811 | 1.80 | 0.06 | 0.27 | 0.16 | 0.82 | 1.03 | 1.25E-02 | -4.83E-04 | 1.03E-03 | -9.87E-04 | -3.97E-03 | 4.44E-03 |
| K01176 | 0.45 | 1.45 | 1.98 | 0.76 | 0.24 | 1.45 | -5.70E-03 | -1.16E-02 | -2.16E-02 | -5.02E-03 | 7.88E-04 | -9.31E-03 |
| K03616 | 0.38 | 0.58 | 1.51 | 1.10 | 1.02 | 0.70 | -8.91E-04 | 3.39E-04 | -1.59E-02 | -6.13E-03 | -6.90E-03 | 5.18E-03 |
| K00053 | 0.71 | 1.42 | 0.48 | 0.82 | 2.05 | 0.55 | 8.35E-03 | 3.61E-03 | 5.57E-03 | -3.23E-03 | 1.37E-02 | -1.58E-03 |
| K00865 | 0.09 | 1.44 | 0.93 | 1.14 | 0.54 | 0.67 | 8.59E-04 | 8.14E-03 | -9.08E-03 | -4.59E-03 | 2.34E-03 | -1.62E-04 |
| K03092 | 1.35 | 1.43 | 0.69 | 1.97 | 0.71 | 0.25 | 8.28E-03 | -6.91E-03 | -1.82E-03 | -8.66E-03 | -4.89E-04 | -1.90E-03 |
| K18843 | 1.00 | 0.62 | 0.67 | 0.61 | 1.01 | 0.95 | -2.48E-03 | 2.11E-03 | -6.72E-03 | -4.04E-03 | 5.75E-03 | 1.12E-03 |
| K11752 | 0.13 | 1.66 | 1.68 | 1.68 | 0.20 | 0.47 | 8.16E-04 | -7.36E-03 | -8.88E-03 | -7.54E-03 | 3.31E-04 | 3.11E-03 |
| K09014 | 0.46 | 1.30 | 1.32 | 0.41 | 1.29 | 0.76 | -3.79E-03 | 2.79E-03 | 1.02E-02 | 5.70E-04 | 8.66E-03 | -7.00E-04 |
| K06041 | 0.79 | 1.49 | 1.97 | 2.00 | 0.72 | 0.20 | -1.71E-03 | -1.02E-02 | -1.73E-02 | -7.69E-03 | 3.55E-03 | -5.94E-04 |
| K18682 | 0.21 | 1.38 | 1.36 | 0.73 | 0.52 | 0.82 | 1.31E-03 | -8.94E-04 | 9.34E-03 | 4.47E-03 | -3.45E-03 | -2.16E-03 |
| K00645 | 0.66 | 0.85 | 1.71 | 1.62 | 0.11 | 0.60 | -8.62E-03 | 1.04E-03 | 1.89E-02 | -8.27E-03 | 1.91E-04 | 2.12E-03 |
| K03790 | 1.26 | 0.26 | 0.69 | 0.87 | 1.21 | 0.28 | 1.31E-02 | -1.53E-03 | -7.80E-03 | -4.42E-03 | -7.70E-03 | -1.75E-03 |
| K05970 | 0.60 | 0.89 | 0.29 | 1.11 | 0.79 | 0.84 | -7.02E-03 | 1.10E-03 | 1.40E-03 | -2.23E-03 | -1.88E-03 | 4.61E-03 |
| K03205 | 1.11 | 1.12 | 0.47 | 0.09 | 0.73 | 1.17 | 2.27E-03 | 6.53E-03 | -3.78E-03 | -4.76E-04 | 3.00E-03 | 6.07E-03 |
| K01912 | 1.05 | 0.46 | 0.73 | 0.71 | 1.81 | 0.92 | -5.99E-04 | 2.25E-03 | -3.59E-03 | -5.05E-03 | -1.22E-02 | -5.67E-03 |
| K02337 | 1.03 | 1.36 | 0.67 | 0.16 | 1.08 | 0.63 | -2.65E-03 | -1.87E-04 | 5.93E-04 | 5.02E-04 | -7.05E-03 | 2.71E-03 |
| K00817 | 0.91 | 1.51 | 0.66 | 0.52 | 1.04 | 0.67 | 1.11E-03 | 9.59E-03 | 2.65E-03 | 5.52E-05 | 6.31E-03 | 1.56E-03 |
| K01940 | 0.55 | 1.42 | 0.86 | 1.09 | 0.25 | 0.77 | 2.53E-03 | 8.58E-03 | 5.12E-03 | -4.12E-03 | -5.04E-04 | -2.94E-03 |
| K02040 | 0.69 | 1.57 | 0.51 | 0.75 | 1.08 | 1.12 | 3.04E-03 | 7.59E-03 | 5.65E-03 | -1.85E-04 | 7.10E-03 | -3.30E-03 |
| K01869 | 0.54 | 1.32 | 1.02 | 0.16 | 0.29 | 1.24 | 1.72E-03 | 5.09E-03 | 9.59E-03 | -2.06E-04 | -1.88E-03 | 6.57E-03 |
| K01224 | 0.82 | 0.82 | 0.91 | 0.62 | 0.66 | 0.24 | -5.26E-03 | 2.20E-03 | 3.85E-03 | 3.12E-03 | 3.96E-03 | 1.76E-03 |
| K20534 | 0.45 | 0.71 | 0.22 | 0.98 | 1.40 | 0.83 | 3.31E-03 | -2.19E-03 | -2.48E-03 | -3.51E-03 | -9.42E-03 | -1.88E-05 |
| K00174 | 0.52 | 0.94 | 1.15 | 0.46 | 0.65 | 1.36 | -1.66E-03 | 1.26E-03 | -4.51E-03 | 3.17E-03 | -2.60E-03 | 1.05E-02 |
| K07729 | 0.47 | 0.76 | 1.09 | 1.22 | 1.87 | 0.46 | 6.98E-03 | 3.40E-03 | -1.16E-02 | 4.05E-03 | -1.10E-02 | 2.09E-04 |
| K07460 | 1.01 | 0.13 | 0.20 | 0.78 | 1.06 | 1.33 | 5.41E-03 | 5.84E-04 | 2.25E-03 | -5.14E-03 | -7.02E-03 | 1.03E-02 |
| K11754 | 0.39 | 1.29 | 0.20 | 1.87 | 0.34 | 1.26 | 3.80E-03 | 2.95E-03 | -1.97E-03 | -1.19E-02 | 1.42E-03 | -6.94E-03 |
| K01173 | 0.96 | 0.72 | 0.42 | 1.04 | 0.96 | 0.56 | 4.67E-03 | 3.19E-03 | 9.35E-04 | -3.06E-03 | -5.34E-03 | 2.26E-03 |
| K21571 | 1.39 | 1.50 | 0.73 | 0.99 | 0.45 | 0.12 | 1.56E-02 | 9.40E-03 | 4.14E-03 | 4.82E-03 | 2.86E-03 | -8.91E-04 |
| K00648 | 0.86 | 0.58 | 1.91 | 1.19 | 0.50 | 0.52 | -1.27E-02 | 1.37E-04 | -2.05E-02 | -3.73E-03 | 2.79E-03 | 3.97E-03 |
| K06133 | 1.16 | 0.45 | 0.62 | 2.19 | 1.36 | 0.48 | 5.95E-03 | 1.94E-03 | -3.16E-03 | -1.30E-02 | -8.31E-03 | -2.14E-03 |
| K01889 | 0.19 | 0.86 | 1.54 | 0.17 | 0.20 | 1.17 | -1.64E-04 | -2.62E-03 | 1.31E-02 | -9.11E-04 | -1.32E-03 | 2.48E-03 |
| K19302 | 1.45 | 0.59 | 1.05 | 0.41 | 1.67 | 0.72 | 1.24E-02 | -1.07E-03 | 6.64E-03 | -3.21E-04 | -1.01E-02 | 4.68E-03 |
| K12340 | 0.76 | 0.37 | 1.12 | 0.81 | 1.00 | 0.65 | 1.14E-03 | -1.47E-04 | -1.26E-02 | 4.68E-04 | -1.70E-04 | -2.24E-03 |

| | | | | | | | | | | | | |
|---|---|---|---|---|---|---|---|---|---|---|---|---|
| K02967 | 0.23 | 1.34 | 1.00 | 0.21 | 0.69 | 1.06 | -3.04E-03 | -3.29E-03 | 8.93E-03 | -4.24E-04 | -4.66E-03 | -1.74E-03 |
| K03106 | 0.73 | 1.47 | 0.35 | 0.88 | 0.27 | 1.03 | -6.80E-03 | -2.14E-03 | -2.41E-03 | -5.52E-03 | 4.61E-04 | 7.41E-05 |
| K06942 | 0.63 | 1.09 | 1.11 | 0.63 | 1.15 | 0.74 | 9.04E-03 | -2.81E-03 | 8.14E-03 | -1.65E-03 | -7.71E-03 | 2.09E-03 |
| K01810 | 0.69 | 1.38 | 0.55 | 0.58 | 0.85 | 0.83 | -5.53E-03 | -1.17E-03 | -3.82E-03 | -7.65E-04 | -5.76E-03 | 3.25E-03 |
| K03168 | 0.13 | 1.47 | 0.71 | 0.77 | 1.03 | 0.95 | 2.03E-03 | 1.44E-03 | -6.46E-03 | -3.64E-03 | 6.93E-03 | 1.91E-03 |
| K01875 | 0.29 | 1.21 | 1.10 | 0.31 | 0.49 | 1.17 | 4.26E-03 | 1.25E-03 | 8.75E-03 | -1.97E-03 | -3.14E-03 | 1.73E-04 |
| K02528 | 1.27 | 2.37 | 0.14 | 0.49 | 0.49 | 1.17 | -1.49E-02 | -1.57E-02 | 6.91E-04 | -2.12E-03 | 2.24E-03 | 5.49E-04 |
| K01258 | 1.30 | 1.23 | 0.26 | 0.80 | 0.21 | 1.02 | -6.10E-03 | 1.46E-03 | 1.87E-03 | -2.18E-03 | 1.38E-03 | 3.55E-03 |
| K03470 | 0.77 | 0.76 | 1.52 | 0.45 | 1.15 | 1.02 | 6.84E-03 | -3.12E-03 | -1.74E-02 | -1.98E-03 | -7.02E-03 | 1.70E-03 |
| K02916 | 1.12 | 1.18 | 0.24 | 0.66 | 1.89 | 0.45 | 6.83E-03 | -8.99E-03 | 7.34E-04 | -4.32E-03 | -1.23E-02 | 3.02E-03 |
| K03437 | 0.75 | 0.84 | 0.37 | 1.03 | 0.85 | 0.60 | -7.64E-03 | 5.48E-03 | -3.85E-03 | -7.00E-03 | -5.73E-03 | 4.34E-03 |
| K00975 | 1.12 | 0.62 | 0.38 | 0.04 | 1.15 | 1.27 | 5.14E-03 | -2.81E-03 | -2.87E-03 | 8.21E-05 | -4.79E-03 | -4.04E-03 |
| K00075 | 0.77 | 1.08 | 0.63 | 1.57 | 0.47 | 1.29 | -7.38E-03 | 1.57E-04 | 6.60E-03 | -1.06E-02 | -2.33E-03 | -5.30E-03 |
| K01738 | 0.41 | 1.08 | 0.50 | 0.62 | 1.16 | 1.03 | -1.04E-03 | -6.89E-03 | -5.76E-03 | -3.20E-03 | -7.17E-03 | -4.71E-03 |
| K00266 | 1.16 | 1.40 | 0.74 | 0.56 | 0.54 | 1.08 | -4.56E-03 | 3.32E-03 | 8.52E-03 | 3.88E-03 | -2.35E-03 | -4.21E-03 |
| K02029 | 1.22 | 0.61 | 0.53 | 0.21 | 1.24 | 1.27 | 3.86E-03 | 1.54E-03 | -3.92E-03 | -1.06E-03 | -4.46E-03 | 2.47E-03 |
| K04487 | 1.50 | 0.47 | 0.64 | 0.63 | 0.83 | 1.19 | -7.99E-03 | -1.96E-03 | -7.41E-03 | -3.78E-03 | -2.80E-03 | -4.93E-04 |
| K03544 | 0.46 | 1.16 | 1.20 | 0.38 | 0.62 | 1.13 | 3.41E-03 | 4.89E-03 | -1.35E-02 | -2.43E-03 | -3.28E-03 | -3.79E-05 |
| K02483 | 1.13 | 0.69 | 0.63 | 0.24 | 0.88 | 1.17 | 3.94E-03 | 1.35E-04 | -7.22E-03 | -1.68E-03 | -2.81E-03 | 7.03E-04 |
| K07727 | 0.58 | 0.26 | 0.82 | 1.01 | 1.18 | 0.44 | -7.33E-03 | 1.20E-03 | -9.13E-03 | -4.91E-03 | 1.33E-04 | 2.35E-04 |
| K07481 | 0.20 | 0.48 | 1.11 | 0.35 | 1.60 | 1.31 | -2.98E-03 | -3.33E-03 | 6.14E-03 | -1.75E-03 | 7.27E-03 | -4.56E-03 |
| K21023 | 0.93 | 0.79 | 0.52 | 0.58 | 1.13 | 1.22 | 7.28E-03 | -1.83E-03 | 3.68E-03 | -3.79E-03 | -5.07E-03 | -1.71E-03 |
| K00615 | 0.65 | 1.26 | 1.06 | 0.18 | 0.63 | 1.07 | 8.07E-03 | 3.11E-03 | 9.10E-03 | 1.05E-03 | -4.70E-04 | 3.79E-04 |
| K03498 | 0.85 | 1.25 | 1.50 | 0.43 | 0.56 | 0.42 | -1.16E-02 | -1.13E-03 | -1.25E-02 | 8.14E-04 | 3.73E-03 | -1.07E-03 |
| K01209 | 0.55 | 1.44 | 0.80 | 1.05 | 1.18 | 0.54 | -8.05E-03 | 6.07E-03 | 8.90E-03 | 2.89E-03 | 7.59E-03 | 4.07E-03 |
| K00432 | 1.33 | 0.74 | 0.24 | 0.90 | 1.06 | 0.64 | 3.39E-03 | -5.63E-03 | 3.50E-04 | -4.13E-03 | -6.90E-03 | 2.15E-03 |
| K01992 | 0.44 | 0.89 | 0.97 | 0.60 | 0.57 | 0.92 | -6.24E-03 | 6.46E-03 | -5.90E-03 | -1.89E-03 | -3.80E-03 | 5.95E-03 |
| K03699 | 0.56 | 1.60 | 0.33 | 1.25 | 0.46 | 1.02 | -7.77E-04 | 6.70E-03 | -1.92E-03 | -4.83E-03 | -1.21E-03 | 1.84E-03 |
| K07483 | 0.68 | 0.45 | 1.31 | 0.86 | 1.62 | 0.68 | -1.02E-02 | 2.01E-04 | -5.89E-03 | -7.30E-04 | -1.03E-02 | -2.69E-03 |
| K07263 | 0.65 | 1.10 | 0.64 | 0.86 | 0.96 | 0.65 | -4.84E-03 | 6.02E-03 | -4.78E-05 | 2.88E-03 | 4.04E-04 | 1.75E-03 |
| K03310 | 0.68 | 1.34 | 0.47 | 0.64 | 0.91 | 1.05 | 6.61E-03 | 4.42E-03 | -5.17E-03 | -7.44E-04 | -3.16E-03 | -3.30E-03 |
| K07098 | 0.38 | 1.14 | 0.61 | 0.87 | 0.60 | 1.31 | -3.82E-03 | -1.05E-04 | 3.82E-03 | 8.36E-04 | -3.96E-03 | 8.66E-03 |
| K07271 | 1.07 | 1.94 | 0.62 | 0.72 | 1.24 | 0.54 | 1.13E-02 | 1.40E-02 | 5.78E-03 | -3.09E-03 | -8.33E-03 | -3.98E-03 |
| K03308 | 0.67 | 1.36 | 0.08 | 1.04 | 0.74 | 1.85 | -4.35E-03 | 3.35E-03 | -4.47E-05 | -7.90E-04 | 4.93E-03 | 1.42E-02 |

| | | | | | | | | | | | | |
|---|---|---|---|---|---|---|---|---|---|---|---|---|
| K07493 | 0.94 | 1.03 | 0.58 | 0.50 | 0.23 | 1.37 | -1.12E-05 | 7.80E-04 | -6.61E-03 | -2.60E-03 | -1.36E-03 | -5.84E-03 |
| K03559 | 1.24 | 0.73 | 0.65 | 1.41 | 0.73 | 1.01 | 3.76E-03 | -1.60E-03 | -6.69E-03 | -4.51E-03 | 2.57E-03 | 6.80E-03 |
| K01154 | 1.22 | 1.41 | 0.55 | 0.51 | 0.41 | 0.90 | -9.91E-03 | 4.70E-03 | -3.12E-03 | -3.43E-03 | 1.85E-04 | -1.66E-03 |
| K07473 | 0.42 | 0.77 | 1.33 | 1.20 | 0.55 | 0.93 | 6.16E-03 | -4.19E-03 | -5.70E-03 | -6.78E-03 | 1.38E-03 | -2.71E-04 |
| K03111 | 2.11 | 0.21 | 0.76 | 0.20 | 1.29 | 0.91 | 2.35E-02 | 1.55E-03 | -7.75E-03 | 4.95E-04 | -5.98E-03 | 3.28E-03 |
| K03327 | 1.64 | 1.46 | 0.67 | 0.47 | 0.60 | 1.41 | -1.05E-02 | 4.34E-03 | 3.98E-03 | 3.32E-03 | 2.69E-03 | 7.96E-03 |
| K04763 | 1.01 | 1.26 | 0.51 | 0.48 | 0.04 | 1.02 | -2.66E-03 | 3.18E-03 | -2.91E-03 | -4.06E-04 | -2.83E-04 | -9.71E-04 |
| K14102 | 0.55 | 0.91 | 1.52 | 0.77 | 0.25 | 0.71 | 1.70E-03 | 5.65E-03 | 1.48E-02 | 2.06E-03 | 1.12E-03 | 5.29E-03 |
| K14096 | 0.61 | 0.35 | 1.15 | 0.91 | 0.23 | 0.60 | -6.14E-03 | 8.02E-04 | 9.54E-03 | 2.10E-03 | 1.50E-03 | 4.62E-03 |
| K02626 | 0.66 | 0.38 | 1.10 | 0.85 | 0.23 | 0.38 | -8.82E-03 | 6.24E-04 | 2.93E-03 | -6.65E-05 | 1.15E-03 | 1.90E-03 |
| K23264 | 0.46 | 0.65 | 1.11 | 0.58 | 0.15 | 1.01 | -1.30E-03 | 4.19E-03 | 8.47E-03 | 1.30E-03 | 7.16E-04 | 7.79E-03 |
| K07270 | 0.89 | 0.80 | 0.33 | 0.57 | 0.41 | 0.61 | -1.22E-02 | 4.83E-03 | -3.79E-03 | -3.97E-03 | 1.19E-03 | -3.13E-03 |
| K00563 | 0.35 | 0.28 | 1.24 | 0.75 | 1.10 | 0.29 | 5.28E-03 | 1.43E-03 | -3.65E-03 | 1.70E-03 | -4.65E-03 | -1.42E-04 |
| K04769 | 1.51 | 0.35 | 0.67 | 1.05 | 0.48 | 0.62 | -1.74E-02 | 1.41E-03 | -3.53E-03 | -1.57E-03 | 3.11E-03 | -4.90E-04 |
| K11621 | 0.27 | 0.25 | 0.64 | 1.72 | 0.99 | 0.47 | 1.84E-03 | 1.49E-03 | 2.64E-04 | -1.14E-02 | -6.21E-03 | 1.35E-03 |
| K00616 | 1.11 | 0.05 | 0.54 | 0.25 | 0.51 | 0.85 | 1.38E-02 | -2.01E-04 | 5.57E-03 | 1.05E-04 | 4.38E-04 | 4.93E-03 |
| K03623 | 1.20 | 0.66 | 0.62 | 0.85 | 0.47 | 0.25 | 7.71E-03 | 4.54E-03 | 4.48E-03 | 2.55E-03 | -3.05E-03 | 1.86E-03 |
| K22210 | 0.79 | 0.08 | 0.30 | 0.24 | 0.85 | 1.31 | 1.68E-03 | 6.70E-04 | 3.42E-03 | 1.62E-03 | -4.61E-03 | -4.62E-03 |
| K07402 | 1.11 | 0.67 | 0.27 | 0.35 | 0.53 | 1.41 | 1.25E-03 | -4.29E-03 | 7.24E-04 | -1.81E-04 | 2.34E-04 | 7.78E-03 |
| K05937 | 0.39 | 1.21 | 0.47 | 0.35 | 0.95 | 0.63 | -2.94E-03 | -8.74E-03 | 4.99E-03 | 3.65E-04 | -4.91E-04 | -2.56E-03 |
| K06985 | 0.50 | 0.49 | 1.33 | 2.07 | 0.73 | 0.48 | 1.87E-03 | -3.17E-03 | -1.52E-02 | -1.40E-02 | 2.62E-03 | 2.78E-03 |
| K03299 | 0.55 | 0.71 | 1.53 | 1.42 | 0.72 | 0.46 | -2.70E-03 | 3.47E-03 | -1.74E-02 | 5.82E-03 | -1.75E-03 | 2.16E-04 |
| K09767 | 1.04 | 0.78 | 0.71 | 0.98 | 0.55 | 0.69 | -1.74E-03 | -6.19E-03 | -3.36E-03 | -5.68E-03 | 2.09E-03 | -4.49E-03 |
| K18122 | 0.15 | 0.59 | 0.64 | 0.99 | 0.79 | 0.91 | -5.43E-04 | -4.18E-04 | -7.34E-03 | -6.01E-03 | -2.02E-03 | 6.47E-03 |
| K05305 | 0.55 | 0.78 | 2.05 | 0.52 | 0.75 | 1.07 | 2.60E-03 | 2.18E-03 | -2.28E-02 | 1.41E-04 | 5.02E-03 | 7.99E-03 |
| K09779 | 0.72 | 0.73 | 0.54 | 1.04 | 0.93 | 0.60 | -1.05E-02 | -2.68E-03 | 1.83E-04 | -1.46E-04 | -4.33E-03 | -1.60E-04 |
| K16214 | 0.71 | 1.16 | 0.70 | 0.21 | 0.89 | 0.60 | 1.02E-02 | -8.81E-03 | 7.95E-03 | -1.39E-03 | 5.97E-03 | 2.57E-03 |
| K01486 | 0.59 | 0.86 | 0.72 | 0.37 | 1.56 | 0.61 | 8.60E-03 | 4.11E-03 | -1.90E-03 | -2.33E-03 | -9.81E-03 | 3.01E-03 |
| K05799 | 0.39 | 0.44 | 1.40 | 0.80 | 0.86 | 0.67 | -2.06E-03 | 2.15E-03 | 2.82E-03 | 4.37E-03 | -2.29E-03 | 3.82E-03 |
| K02413 | 0.98 | 0.29 | 0.84 | 0.50 | 0.75 | 0.78 | 1.28E-03 | -2.06E-03 | -1.67E-03 | -1.64E-03 | 3.21E-04 | 2.40E-04 |
| K13075 | 0.69 | 0.79 | 0.79 | 0.38 | 1.28 | 1.18 | -1.85E-03 | 6.26E-03 | -4.04E-03 | -1.80E-04 | -7.39E-03 | -9.00E-03 |
| K02398 | 0.86 | 0.51 | 1.13 | 0.64 | 0.74 | 0.77 | -4.34E-04 | -3.99E-03 | -3.81E-03 | -3.23E-03 | 5.34E-04 | -1.22E-03 |
| K16698 | 0.69 | 0.86 | 0.46 | 0.22 | 1.05 | 0.43 | -1.04E-02 | -6.91E-03 | -7.12E-04 | -8.80E-04 | -5.11E-03 | -1.05E-03 |
| K03820 | 0.48 | 0.26 | 1.18 | 1.30 | 0.73 | 0.35 | 7.33E-03 | -8.39E-04 | 4.73E-03 | -7.51E-03 | 4.94E-03 | -2.33E-03 |

| | | | | | | | | | | | | |
|---|---|---|---|---|---|---|---|---|---|---|---|---|
| K05337 | 1.12 | 0.55 | 0.65 | 0.62 | 1.36 | 0.41 | 1.69E-02 | -2.42E-03 | -4.18E-03 | -8.66E-05 | -6.71E-03 | 1.25E-03 |
| K02435 | 0.29 | 0.59 | 0.59 | 0.87 | 1.44 | 0.76 | 4.28E-03 | -3.88E-03 | 2.15E-04 | -1.65E-03 | -5.83E-03 | 1.25E-04 |
| K00872 | 0.94 | 0.51 | 0.49 | 1.03 | 0.51 | 0.76 | 6.59E-03 | -3.33E-03 | -4.34E-03 | -6.89E-03 | 3.45E-03 | 1.20E-04 |
| K11941 | 1.11 | 0.51 | 0.39 | 0.87 | 0.19 | 0.19 | -1.48E-02 | 3.67E-03 | -1.37E-03 | 6.96E-04 | -1.11E-04 | 1.34E-03 |
| K00797 | 0.38 | 0.62 | 0.49 | 0.39 | 0.98 | 1.15 | 1.44E-03 | -4.49E-03 | -2.15E-04 | -2.42E-03 | 1.31E-04 | -4.18E-03 |
| K01902 | 0.80 | 0.54 | 0.72 | 0.39 | 0.47 | 0.86 | 3.29E-03 | -9.57E-04 | -6.00E-03 | 1.60E-03 | 2.50E-03 | 3.95E-03 |
| K07488 | 0.79 | 1.13 | 0.73 | 0.78 | 0.60 | 1.65 | 8.90E-03 | -6.34E-03 | -1.74E-03 | -5.41E-03 | 1.64E-03 | 1.27E-02 |
| K19119 | 0.99 | 0.67 | 0.65 | 0.66 | 0.69 | 1.20 | 1.03E-02 | 2.25E-05 | -3.35E-03 | -2.79E-03 | 1.54E-03 | 5.76E-03 |
| K19118 | 1.04 | 0.75 | 0.68 | 0.62 | 0.55 | 0.95 | 1.10E-02 | -1.80E-04 | -6.55E-03 | -1.69E-03 | -1.06E-03 | 1.87E-03 |
| K07741 | 0.69 | 1.01 | 0.06 | 0.57 | 0.54 | 1.51 | 1.04E-02 | 7.71E-03 | -6.64E-04 | 1.59E-03 | -2.56E-04 | 9.47E-03 |
| K02038 | 1.07 | 0.73 | 0.15 | 0.63 | 0.67 | 1.01 | 3.57E-03 | -9.41E-04 | -1.42E-03 | -3.58E-03 | 4.42E-03 | -4.81E-04 |
| K06990 | 0.87 | 0.69 | 0.21 | 0.34 | 0.12 | 1.30 | 1.02E-02 | 1.60E-03 | -2.12E-03 | -1.85E-03 | 5.90E-04 | 6.87E-03 |
| K14136 | 0.20 | 1.16 | 0.40 | 1.38 | 0.21 | 0.74 | 1.17E-03 | 2.82E-03 | -7.19E-04 | -4.10E-03 | -9.00E-04 | -4.98E-03 |
| K00351 | 0.62 | 1.12 | 1.33 | 0.70 | 0.79 | 0.74 | -3.29E-03 | -7.16E-03 | 1.11E-02 | 3.87E-03 | -1.55E-03 | 5.69E-03 |
| K01626 | 1.12 | 0.56 | 0.25 | 0.36 | 0.64 | 1.44 | 2.83E-03 | -3.17E-03 | 1.55E-03 | -1.97E-03 | -1.35E-03 | -7.74E-03 |
| K06215 | 0.69 | 1.78 | 0.46 | 0.73 | 0.63 | 0.87 | -4.13E-03 | -9.58E-03 | -5.28E-03 | 2.11E-03 | -3.72E-03 | -1.50E-03 |
| K11358 | 0.87 | 0.44 | 0.28 | 0.54 | 0.55 | 1.03 | 5.27E-03 | -3.27E-03 | -2.87E-03 | -3.64E-03 | 2.31E-03 | -9.00E-04 |
| K00995 | 0.75 | 0.71 | 0.76 | 0.59 | 1.10 | 1.32 | 6.01E-04 | -5.59E-03 | -7.76E-03 | -4.03E-03 | -2.13E-03 | -4.39E-03 |
| K08681 | 0.40 | 1.29 | 0.68 | 0.95 | 0.28 | 0.78 | -3.21E-03 | -4.81E-03 | -7.79E-03 | 6.21E-03 | 8.40E-04 | 2.74E-03 |
| K18346 | 0.20 | 0.59 | 0.32 | 0.57 | 1.06 | 1.01 | -2.75E-03 | -6.23E-05 | -4.75E-04 | 1.69E-03 | -6.93E-03 | -6.33E-03 |
| K02025 | 1.28 | 0.70 | 0.73 | 0.08 | 0.70 | 1.28 | -2.17E-03 | 1.51E-03 | -6.73E-03 | -5.80E-04 | -1.34E-03 | 5.18E-03 |
| K06960 | 0.77 | 0.49 | 0.56 | 0.92 | 1.93 | 0.74 | 1.17E-02 | -1.76E-03 | 5.92E-04 | -2.78E-03 | -8.90E-03 | -8.39E-04 |
| K03183 | 1.22 | 0.27 | 0.72 | 0.75 | 0.60 | 1.21 | 6.63E-03 | 5.70E-04 | 8.22E-03 | 3.42E-04 | 5.44E-04 | 6.97E-03 |
| K01754 | 1.04 | 0.77 | 0.45 | 0.20 | 0.63 | 1.21 | 1.54E-02 | -6.15E-03 | 4.43E-03 | 1.27E-03 | 1.98E-03 | -4.18E-03 |
| K06188 | 1.18 | 0.69 | 0.70 | 0.30 | 1.06 | 0.66 | 1.43E-03 | -5.10E-03 | 8.11E-03 | -2.05E-03 | -6.86E-03 | -2.45E-04 |
| K00937 | 1.01 | 0.74 | 0.56 | 0.18 | 0.49 | 1.02 | 6.94E-03 | -3.05E-03 | 5.70E-03 | 3.85E-05 | -6.57E-04 | -1.14E-03 |
| K09474 | 1.37 | 0.10 | 0.44 | 0.69 | 0.74 | 0.96 | 4.03E-03 | 4.36E-05 | 5.09E-03 | -4.80E-03 | -6.58E-04 | -5.76E-03 |
| K00721 | 1.32 | 0.25 | 0.78 | 0.50 | 0.52 | 1.58 | 3.50E-03 | -1.95E-03 | 8.41E-03 | 2.42E-03 | 1.26E-04 | 1.04E-02 |
| K00812 | 1.25 | 0.73 | 0.43 | 0.84 | 0.76 | 0.75 | 6.49E-03 | 6.82E-04 | -1.12E-05 | 4.04E-03 | 1.91E-03 | 2.37E-03 |
| K00798 | 1.13 | 0.26 | 0.34 | 0.71 | 0.62 | 0.86 | -1.91E-03 | 1.97E-03 | 3.25E-03 | -4.93E-03 | -7.40E-04 | -1.93E-03 |
| K03431 | 1.16 | 0.50 | 0.25 | 0.09 | 0.48 | 1.13 | 4.97E-03 | 2.39E-03 | -9.81E-04 | -5.86E-04 | -5.35E-04 | 3.20E-03 |
| K01817 | 0.69 | 1.35 | 1.04 | 0.62 | 0.74 | 0.80 | 1.02E-02 | 7.21E-04 | 1.15E-02 | -4.33E-03 | -2.01E-03 | 5.25E-03 |
| K03500 | 1.15 | 0.56 | 0.76 | 0.53 | 0.75 | 1.12 | -2.40E-03 | -3.93E-03 | -6.81E-03 | -2.86E-03 | 3.65E-04 | -5.07E-03 |
| K00180 | 1.10 | 0.45 | 0.68 | 0.65 | 1.32 | 0.62 | 3.78E-04 | 4.83E-04 | -1.12E-03 | -4.53E-03 | -8.89E-03 | -2.59E-03 |

| | | | | | | | | | | | | |
|---|---|---|---|---|---|---|---|---|---|---|---|---|
| K02426 | 1.32 | 0.30 | 0.45 | 0.31 | 0.60 | 0.86 | 5.11E-03 | -8.68E-04 | 5.21E-03 | 1.93E-03 | -1.21E-03 | 3.71E-03 |
| K03086 | 1.23 | 0.76 | 0.16 | 0.48 | 0.80 | 1.12 | -2.08E-03 | -4.83E-03 | 1.48E-04 | -1.96E-03 | 2.80E-03 | -3.16E-03 |
| K01338 | 0.47 | 0.89 | 1.40 | 0.57 | 0.41 | 0.62 | -6.90E-03 | -3.48E-03 | 8.43E-03 | 3.83E-03 | 2.61E-03 | -4.73E-03 |
| K09765 | 0.29 | 0.41 | 0.80 | 0.59 | 1.57 | 0.65 | -1.67E-03 | -3.25E-03 | 1.31E-03 | -2.61E-03 | -7.77E-03 | -9.52E-04 |
| K21498 | 0.78 | 0.68 | 1.79 | 1.56 | 0.63 | 0.59 | 1.06E-02 | -4.98E-03 | -1.01E-02 | -8.73E-03 | 1.30E-03 | -1.43E-03 |
| K01610 | 0.75 | 1.33 | 1.18 | 0.55 | 0.32 | 0.75 | -2.12E-03 | -3.06E-03 | 1.32E-02 | 2.15E-03 | -4.65E-04 | -2.51E-03 |
| K02346 | 1.25 | 1.49 | 0.32 | 0.43 | 0.37 | 0.58 | -7.63E-03 | 9.28E-03 | 1.16E-03 | 2.94E-03 | 2.37E-03 | -1.90E-03 |
| K02114 | 0.91 | 0.68 | 0.36 | 0.50 | 0.67 | 1.24 | 2.37E-03 | 2.70E-03 | -1.03E-03 | 1.31E-03 | 1.63E-03 | -4.78E-03 |
| K07277 | 0.71 | 0.95 | 0.31 | 1.19 | 0.75 | 0.58 | -1.72E-03 | -2.13E-03 | -3.25E-03 | -2.98E-03 | -7.31E-05 | 4.42E-03 |
| K17103 | 1.17 | 0.76 | 0.45 | 0.44 | 0.97 | 0.79 | 4.69E-03 | -3.93E-03 | 4.27E-03 | -2.85E-03 | -4.44E-03 | 4.73E-03 |
| K03525 | 0.77 | 0.33 | 1.09 | 0.66 | 1.02 | 0.69 | 1.17E-02 | -2.17E-03 | 3.30E-03 | 1.08E-04 | -4.35E-03 | 7.60E-04 |
| K14445 | 0.73 | 0.97 | 0.62 | 1.14 | 0.79 | 0.69 | -3.76E-03 | 1.76E-03 | 6.35E-03 | 5.22E-03 | -1.36E-03 | 2.96E-03 |
| K03787 | 0.77 | 0.36 | 0.61 | 0.45 | 0.84 | 1.81 | 6.55E-05 | 3.60E-04 | -5.37E-03 | 8.34E-04 | -5.18E-03 | 1.36E-02 |
| K09117 | 0.45 | 0.76 | 1.10 | 0.71 | 0.39 | 0.94 | 2.62E-03 | -4.30E-03 | -1.07E-02 | -1.64E-03 | 6.68E-04 | 5.52E-03 |
| K04771 | 0.60 | 1.31 | 0.70 | 1.03 | 0.44 | 0.71 | 4.31E-03 | -2.30E-03 | 7.66E-03 | -1.50E-03 | -2.11E-03 | -1.62E-03 |
| K19693 | 0.71 | 1.42 | 0.23 | 0.78 | 0.85 | 0.30 | 7.71E-03 | 6.57E-03 | 1.19E-04 | -1.67E-03 | 4.27E-03 | -2.34E-03 |
| K03517 | 0.52 | 1.42 | 1.15 | 0.62 | 0.44 | 0.65 | 2.70E-03 | 5.66E-03 | 1.23E-02 | 2.60E-03 | 2.88E-03 | -5.01E-03 |
| K13038 | 0.47 | 1.12 | 0.40 | 0.99 | 0.57 | 0.36 | 6.15E-03 | 3.56E-03 | 4.18E-03 | -4.77E-03 | 2.96E-03 | 2.28E-03 |
| K19158 | 0.89 | 0.75 | 1.92 | 0.27 | 0.72 | 0.61 | 3.23E-03 | -4.25E-03 | 1.54E-02 | -9.09E-04 | 4.61E-03 | 3.35E-03 |
| K01873 | 1.00 | 1.64 | 0.70 | 0.44 | 0.49 | 0.69 | -5.90E-03 | 8.53E-03 | 7.53E-04 | -2.16E-03 | 3.30E-03 | 5.27E-04 |
| K03043 | 0.97 | 1.35 | 0.56 | 0.19 | 0.36 | 0.70 | -8.50E-04 | 2.21E-03 | -9.16E-04 | 8.79E-04 | 1.04E-04 | 1.63E-03 |
| K03046 | 1.59 | 1.21 | 0.63 | 0.28 | 0.20 | 0.80 | -1.36E-02 | 8.97E-04 | 2.95E-03 | 7.39E-04 | -9.97E-04 | -3.43E-03 |
| K03615 | 0.63 | 0.98 | 0.74 | 0.35 | 0.74 | 0.93 | -7.31E-03 | -1.50E-04 | -8.22E-03 | -4.40E-04 | -4.31E-03 | 7.20E-03 |
| K01495 | 1.28 | 0.11 | 0.65 | 1.63 | 0.12 | 0.78 | 3.28E-03 | -8.81E-04 | -2.20E-03 | -1.02E-02 | -6.07E-04 | 5.35E-03 |
| K06909 | 0.38 | 0.53 | 1.15 | 0.33 | 0.60 | 0.92 | 2.22E-03 | 3.98E-03 | -1.09E-02 | -6.81E-04 | 4.05E-03 | 6.25E-03 |
| K07317 | 0.30 | 1.54 | 1.34 | 0.37 | 0.55 | 0.57 | -3.77E-03 | 1.07E-02 | 1.08E-02 | -2.62E-03 | -2.51E-03 | -3.93E-03 |
| K02519 | 0.69 | 1.39 | 0.57 | 0.39 | 0.45 | 0.83 | -2.95E-03 | -2.36E-03 | -1.77E-04 | -1.10E-03 | 3.03E-03 | -2.50E-03 |
| K04077 | 0.32 | 1.46 | 0.60 | 0.34 | 0.30 | 0.81 | -3.07E-04 | 2.22E-03 | -4.80E-05 | -1.57E-03 | -1.55E-03 | -3.18E-03 |
| K19005 | 0.77 | 0.61 | 0.11 | 1.62 | 0.99 | 0.79 | -5.47E-03 | 4.63E-03 | -1.24E-03 | -8.81E-03 | -2.34E-03 | -2.95E-03 |
| K06113 | 0.77 | 1.39 | 0.39 | 0.77 | 0.90 | 0.31 | -7.34E-03 | 8.77E-03 | 4.04E-03 | -5.93E-04 | 2.75E-03 | -1.31E-03 |
| K02112 | 0.68 | 1.36 | 0.53 | 0.55 | 0.11 | 1.05 | 1.65E-04 | 4.13E-03 | 4.67E-03 | 2.20E-03 | 4.68E-05 | -2.78E-03 |
| K01929 | 0.22 | 1.35 | 0.53 | 0.68 | 0.44 | 0.94 | 2.52E-03 | -1.59E-03 | 1.63E-03 | -3.69E-03 | 2.89E-03 | -1.26E-03 |
| K00760 | 0.71 | 0.41 | 0.93 | 0.30 | 1.50 | 0.09 | 4.98E-03 | 9.99E-04 | -1.32E-03 | -2.87E-04 | -1.01E-02 | -4.58E-04 |
| K03977 | 0.53 | 1.12 | 0.66 | 0.33 | 0.62 | 1.16 | 7.56E-03 | -2.24E-03 | -2.57E-03 | 1.04E-03 | -1.99E-03 | 6.20E-03 |

| | | | | | | | | | | | | |
|---|---|---|---|---|---|---|---|---|---|---|---|---|
| K03655 | 0.59 | 1.17 | 0.22 | 0.61 | 0.39 | 0.86 | 4.12E-03 | 9.72E-04 | 5.54E-04 | -1.86E-03 | 1.14E-03 | -2.69E-03 |
| K01808 | 0.24 | 0.20 | 0.52 | 0.33 | 1.22 | 1.56 | 1.71E-03 | -6.78E-04 | 4.86E-03 | -1.28E-03 | -5.89E-03 | 1.19E-02 |
| K02520 | 1.67 | 0.65 | 0.57 | 0.57 | 1.92 | 0.77 | 1.88E-02 | -2.65E-03 | -5.61E-03 | -3.53E-03 | -1.26E-02 | -1.66E-03 |
| K07979 | 1.01 | 0.79 | 0.99 | 0.77 | 0.74 | 0.54 | -5.84E-03 | 6.24E-03 | -3.41E-06 | 4.85E-03 | -1.70E-03 | 7.54E-04 |
| K03602 | 0.97 | 0.46 | 0.34 | 1.06 | 0.68 | 0.56 | 2.32E-03 | -3.56E-03 | 1.03E-03 | -6.56E-03 | -2.58E-03 | 4.15E-03 |
| K02963 | 0.64 | 0.57 | 1.23 | 0.74 | 0.26 | 1.04 | -5.51E-03 | -4.03E-03 | 1.42E-02 | -5.13E-03 | 5.22E-04 | 7.43E-03 |
| K07114 | 1.49 | 0.60 | 0.46 | 1.28 | 0.73 | 0.59 | 8.04E-03 | -4.39E-03 | 5.30E-03 | -5.25E-03 | -2.09E-03 | 1.74E-03 |
| K00059 | 0.73 | 0.43 | 0.59 | 0.90 | 1.15 | 0.25 | -5.36E-03 | -1.11E-03 | 6.84E-03 | -5.77E-03 | -7.65E-03 | 1.91E-03 |
| K01153 | 1.71 | 1.42 | 0.47 | 0.58 | 0.51 | 0.77 | -1.87E-02 | 6.00E-03 | -3.64E-03 | -3.89E-03 | 2.63E-03 | -3.58E-03 |
| K02316 | 0.54 | 1.18 | 0.78 | 0.73 | 0.70 | 0.84 | -7.74E-03 | 5.21E-03 | 7.04E-03 | -4.23E-03 | 4.61E-03 | 3.18E-03 |
| K03427 | 1.45 | 1.55 | 0.64 | 0.69 | 0.70 | 0.75 | -1.58E-02 | 9.12E-03 | 1.50E-03 | -3.56E-03 | 3.68E-03 | -3.65E-04 |
| K07273 | 1.58 | 1.08 | 0.75 | 0.32 | 0.47 | 0.41 | 1.79E-02 | 8.53E-03 | 1.90E-03 | 7.87E-04 | -1.88E-03 | 3.60E-04 |
| K00986 | 0.67 | 0.79 | 0.70 | 0.32 | 0.97 | 1.26 | 4.74E-03 | 3.70E-03 | -5.69E-03 | 2.15E-03 | -5.21E-03 | 8.02E-03 |
| K07485 | 0.97 | 0.65 | 0.72 | 0.37 | 0.50 | 0.97 | -2.77E-03 | -2.49E-04 | -6.28E-03 | -1.94E-03 | 1.11E-03 | 1.14E-03 |
| K15024 | 0.63 | 0.64 | 1.16 | 0.43 | 0.19 | 0.53 | -9.42E-03 | -4.52E-03 | 8.02E-03 | 7.73E-04 | 3.93E-04 | 2.40E-03 |
| K02499 | 0.69 | 0.15 | 1.87 | 0.69 | 0.54 | 0.70 | 9.30E-03 | 8.34E-05 | 2.17E-02 | -3.62E-03 | 1.55E-03 | -3.09E-03 |
| K13566 | 0.52 | 0.70 | 0.55 | 1.74 | 0.53 | 0.46 | 6.93E-03 | 3.34E-03 | -5.49E-03 | 9.02E-03 | 2.59E-03 | -1.59E-03 |
| K07248 | 0.69 | 0.64 | 1.45 | 0.43 | 0.15 | 0.66 | -1.87E-03 | -3.67E-03 | -1.47E-02 | -2.34E-03 | 4.46E-04 | -4.00E-03 |
| K01666 | 0.05 | 0.27 | 1.24 | 0.52 | 0.36 | 0.62 | -5.57E-04 | 6.85E-04 | 1.12E-02 | -1.11E-03 | 1.55E-03 | -4.46E-03 |
| K02420 | 0.71 | 0.35 | 1.15 | 0.77 | 0.78 | 0.68 | 2.72E-03 | -2.73E-03 | -3.94E-03 | -4.12E-03 | 1.26E-03 | 6.01E-04 |
| K02614 | 1.07 | 0.41 | 0.55 | 0.12 | 0.61 | 0.75 | -6.27E-04 | 3.98E-04 | -1.86E-03 | -5.72E-04 | -1.38E-03 | -3.65E-03 |
| K09680 | 1.02 | 0.58 | 0.13 | 0.70 | 0.47 | 0.65 | 1.01E-03 | 4.63E-03 | -6.95E-04 | 3.07E-03 | -4.96E-04 | 4.62E-03 |
| K09760 | 0.19 | 0.98 | 0.39 | 0.48 | 0.22 | 0.33 | -2.73E-03 | 3.30E-03 | -3.22E-03 | -3.39E-03 | 1.47E-03 | 2.56E-03 |
| K01719 | 1.15 | 0.58 | 0.36 | 0.40 | 0.70 | 0.76 | 3.12E-03 | 4.09E-03 | -1.38E-03 | -2.31E-04 | -2.24E-03 | 2.35E-03 |
| K20509 | 0.60 | 0.64 | 1.02 | 0.32 | 0.70 | 0.78 | -8.16E-03 | 4.58E-03 | 5.29E-03 | -3.38E-04 | -2.85E-03 | -5.94E-03 |
| K06199 | 0.73 | 0.76 | 0.77 | 0.87 | 0.60 | 0.73 | 8.11E-03 | -4.94E-03 | 2.83E-03 | -1.47E-03 | 2.38E-05 | -1.52E-03 |
| K07386 | 0.54 | 0.44 | 0.76 | 0.65 | 0.32 | 0.98 | -4.32E-03 | 5.63E-04 | -5.21E-04 | 4.12E-04 | 1.63E-03 | 7.14E-03 |
| K01952 | 0.77 | 1.48 | 0.45 | 0.43 | 0.43 | 0.53 | -1.81E-03 | 7.57E-03 | -2.33E-03 | -1.16E-03 | -1.49E-03 | -1.50E-05 |
| K13444 | 1.47 | 0.21 | 0.26 | 0.80 | 0.37 | 0.53 | 5.78E-03 | -1.01E-03 | -2.51E-03 | -5.33E-03 | 1.52E-03 | -2.49E-05 |
| K22522 | 0.75 | 0.76 | 1.17 | 0.46 | 0.16 | 0.44 | -4.61E-04 | -5.99E-03 | 6.31E-03 | 2.61E-03 | 9.95E-04 | 3.34E-03 |
| K07816 | 0.30 | 0.62 | 0.81 | 0.71 | 0.62 | 0.64 | 2.53E-03 | 4.96E-03 | 7.53E-03 | -4.93E-03 | -1.06E-03 | -4.93E-03 |
| K04567 | 0.77 | 1.30 | 0.45 | 0.11 | 0.35 | 0.76 | -7.53E-03 | -2.40E-03 | 4.37E-03 | 1.15E-04 | -1.86E-03 | -2.46E-03 |
| K11717 | 0.74 | 1.15 | 0.63 | 0.78 | 0.74 | 0.38 | 4.00E-03 | 5.98E-03 | 2.27E-04 | 5.51E-03 | 2.09E-03 | -2.83E-03 |
| K09710 | 0.74 | 0.62 | 0.60 | 0.32 | 0.47 | 1.08 | -5.51E-03 | -4.93E-03 | 4.46E-03 | 2.17E-03 | -2.27E-03 | 8.20E-03 |

| | | | | | | | | | | | | |
|---|---|---|---|---|---|---|---|---|---|---|---|---|
| K04066 | 0.47 | 1.29 | 0.51 | 0.64 | 0.62 | 0.79 | 1.74E-03 | -1.67E-03 | -2.76E-03 | -2.87E-03 | -4.17E-03 | 1.38E-03 |
| K06904 | 0.63 | 0.53 | 0.83 | 0.30 | 0.42 | 0.41 | -5.83E-03 | 4.21E-03 | -8.68E-03 | 1.22E-03 | -2.41E-03 | -2.83E-03 |
| K00287 | 0.78 | 0.57 | 0.57 | 1.35 | 0.43 | 0.34 | 3.00E-03 | 4.57E-03 | -6.56E-03 | -7.15E-03 | -2.75E-03 | -2.59E-03 |
| K01662 | 0.63 | 1.39 | 0.20 | 0.57 | 0.30 | 0.69 | 7.43E-05 | 3.07E-03 | -1.33E-03 | 2.18E-04 | 1.88E-03 | 4.16E-03 |
| K03502 | 0.51 | 1.31 | 0.43 | 0.63 | 0.53 | 0.53 | 7.75E-03 | -4.05E-03 | -2.68E-03 | -3.74E-03 | -2.66E-03 | 9.38E-04 |
| K02965 | 1.38 | 0.80 | 0.44 | 0.64 | 0.72 | 0.45 | 1.05E-02 | -5.37E-03 | -1.06E-03 | -5.44E-04 | -2.67E-03 | 3.41E-03 |
| K22278 | 0.68 | 0.49 | 0.43 | 0.67 | 0.45 | 0.90 | -5.49E-03 | -9.00E-04 | -1.93E-03 | -2.95E-03 | -2.91E-03 | 6.91E-03 |
| K01201 | 0.35 | 0.66 | 0.65 | 0.10 | 0.42 | 0.55 | 5.02E-03 | 2.71E-03 | 7.41E-03 | -2.95E-04 | -2.81E-03 | 3.90E-03 |
| K14188 | 0.32 | 0.41 | 0.48 | 0.56 | 0.67 | 0.39 | -4.90E-03 | -2.49E-03 | 5.42E-03 | -2.17E-03 | -8.37E-04 | -3.74E-04 |
| K01809 | 0.66 | 0.43 | 0.31 | 0.64 | 0.20 | 0.73 | -7.67E-03 | 2.34E-03 | -3.11E-03 | -1.84E-03 | -1.15E-04 | 5.18E-03 |

**Trait: Average Daily Gain (ADG); Predictors: 673 heritable microbial genes**

| Microbial gene | VIP | | | | | | Regression coefficient | | | | | |
|---|---|---|---|---|---|---|---|---|---|---|---|---|
| | T1 | T2 | T3 | T4 | T5 | T6 | T1 | T2 | T3 | T4 | T5 | T6 |
| K01726$_{VIP\geq0.8\ (+)}$ | 1.114 | 0.856 | 0.93 | 0.919 | 1.253 | 0.546 | 8.31E-03 | 5.12E-03 | 3.38E-03 | 3.33E-03 | 1.11E-02 | 3.59E-04 |
| K02065$_{VIP\geq0.8\ (+)}$ | 1.502 | 1.856 | 0.734 | 1.488 | 1.144 | 1.341 | 6.63E-03 | 1.55E-02 | 7.32E-03 | 6.70E-03 | 4.75E-03 | 8.82E-03 |
| K09774$_{VIP\geq0.8\ (+)}$ | 1.025 | 1.558 | 0.474 | 1.122 | 1.421 | 1.11 | 2.83E-03 | 9.20E-03 | 4.87E-03 | 4.87E-03 | 9.98E-04 | 5.75E-03 |
| K13038$_{VIP\geq0.8\ (+)}$ | 1.254 | 1.015 | 0.941 | 0.976 | 1.689 | 0.612 | 5.50E-03 | 1.76E-03 | 1.38E-05 | 4.73E-03 | 1.43E-02 | 4.52E-03 |
| K05970$_{VIP\geq0.8\ (+)}$ | 1.481 | 1.299 | 0.585 | 1.528 | 1.08 | 1.088 | 9.74E-03 | 4.10E-03 | 2.75E-04 | 6.39E-03 | 7.50E-03 | 1.91E-03 |
| K09764$_{VIP\geq0.8\ (+)}$ | 0.84 | 0.675 | 0.928 | 0.471 | 0.964 | 0.981 | 5.68E-03 | 3.93E-03 | 3.67E-04 | 1.71E-03 | 5.62E-03 | 7.23E-03 |
| K06985$_{VIP\geq0.8\ (+)}$ | 1.437 | 0.554 | 1.232 | 1.988 | 0.888 | 0.12 | 8.86E-03 | 2.47E-03 | 1.35E-02 | 1.04E-02 | 1.14E-03 | 8.22E-04 |
| K06885$_{VIP\geq0.8\ (+)}$ | 0.861 | 0.514 | 0.916 | 0.956 | 0.925 | 0.689 | 4.30E-03 | 2.49E-03 | 3.87E-03 | 1.76E-03 | 6.97E-03 | 1.71E-03 |
| K00995$_{VIP\geq0.8\ (+)}$ | 1.043 | 0.678 | 0.999 | 0.94 | 0.748 | 1.329 | 8.07E-03 | 5.19E-03 | 8.36E-04 | 1.82E-03 | 3.42E-03 | 5.01E-03 |
| K03634$_{VIP\geq0.8\ (+)}$ | 0.959 | 1.235 | 0.798 | 1.5 | 0.554 | 1.182 | 1.33E-03 | 1.00E-02 | 4.31E-03 | 6.66E-03 | 3.05E-05 | 6.76E-03 |
| K00912$_{VIP\geq0.8\ (+)}$ | 0.991 | 1.106 | 0.78 | 1.94 | 1.299 | 0.567 | 2.27E-03 | 7.27E-03 | 2.02E-03 | 9.02E-03 | 1.70E-03 | 1.80E-04 |
| K04487$_{VIP\geq0.8\ (+)}$ | 1.028 | 0.212 | 1.271 | 0.961 | 0.61 | 0.946 | 6.91E-03 | 1.77E-03 | 4.71E-03 | 2.71E-03 | 6.15E-03 | 1.20E-03 |
| K02483$_{VIP\geq0.8\ (+)}$ | 0.903 | 0.571 | 1.127 | 0.897 | 0.465 | 0.878 | 6.81E-03 | 3.77E-03 | 7.44E-04 | 1.36E-04 | 4.82E-03 | 1.31E-03 |
| K01235$_{VIP\geq0.8\ (+)}$ | 1.203 | 0.997 | 0.888 | 1.403 | 1.774 | 0.963 | 9.10E-03 | -2.60E-05 | 9.48E-03 | 6.66E-03 | 1.43E-02 | 3.45E-03 |
| K02527$_{VIP\geq0.8\ (+)}$ | 1.417 | 1.125 | 0.825 | 1.189 | 1.514 | 1.196 | 5.61E-03 | 6.53E-03 | 9.79E-04 | 4.33E-03 | -1.16E-03 | 6.69E-03 |
| K07058$_{VIP\geq0.8\ (+)}$ | 1.76 | 0.944 | 0.897 | 1.732 | 0.871 | 1.172 | 1.35E-02 | 7.06E-03 | -4.30E-04 | 8.78E-03 | 7.58E-03 | 8.30E-03 |
| K00793$_{VIP\geq0.8\ (+)}$ | 0.958 | 1.24 | 1.134 | 1.643 | 1.34 | 1.148 | 3.83E-03 | 8.35E-03 | 1.18E-02 | 7.81E-03 | -5.48E-03 | 7.82E-03 |
| K00748$_{VIP\geq0.8\ (+)}$ | 1.153 | 1.183 | 0.82 | 1.827 | 1.56 | 1.223 | 4.79E-03 | 7.13E-03 | -7.20E-04 | 8.79E-03 | 5.92E-03 | 6.56E-03 |

| | | | | | | | | | | | | |
|---|---|---|---|---|---|---|---|---|---|---|---|---|
| K03975$_{VIP\geq0.8\ (+)}$ | 1.154 | 1.191 | 0.986 | 1.476 | 1.612 | 1.058 | 3.89E-03 | 7.68E-03 | 7.87E-03 | 6.50E-03 | -1.13E-02 | 8.70E-05 |
| K02536$_{VIP\geq0.8\ (+)}$ | 1.085 | 1.434 | 1.111 | 1.638 | 1.369 | 1.211 | 1.92E-03 | 1.03E-02 | -7.09E-03 | 8.00E-03 | 1.74E-03 | 5.72E-03 |
| K00865$_{VIP\geq0.8\ (+)}$ | 1.186 | 1.044 | 1.317 | 1.141 | 1.141 | 0.807 | 4.53E-03 | -1.41E-03 | 1.02E-02 | 5.88E-03 | 2.68E-03 | 5.86E-03 |
| K01173$_{VIP\geq0.8\ (+)}$ | 1.199 | 0.986 | 0.95 | 1.358 | 1.231 | 1.086 | 2.73E-03 | -3.42E-04 | 1.04E-02 | 6.09E-03 | 4.75E-03 | 6.09E-03 |
| K00677$_{VIP\geq0.8\ (+)}$ | 1.12 | 1.177 | 0.842 | 1.569 | 1.443 | 1.084 | 2.41E-03 | 2.55E-03 | 4.28E-03 | 5.97E-03 | -3.91E-03 | 2.68E-03 |
| K00067$_{VIP\geq0.8\ (+)}$ | 1.83 | 1.066 | 1.251 | 1.892 | 1.236 | 1.136 | 1.24E-02 | -4.55E-03 | 1.26E-02 | 9.95E-03 | 3.96E-03 | 8.32E-03 |
| K07493$_{VIP\geq0.8\ (+)}$ | 0.833 | 0.871 | 1.326 | 0.984 | 1.229 | 1.17 | 6.35E-03 | -5.40E-03 | 2.84E-03 | 3.41E-03 | 1.10E-02 | 6.39E-03 |
| K02466$_{VIP\geq0.8\ (+)}$ | 0.856 | 0.879 | 1.662 | 0.909 | 0.7 | 1.162 | -5.25E-03 | 7.51E-03 | 1.81E-02 | 4.44E-03 | 7.82E-04 | 8.32E-03 |
| K06934$_{VIP\geq0.8\ (+)}$ | 0.671 | 1.22 | 0.825 | 0.9 | 1.702 | 1.091 | 5.17E-03 | -1.05E-02 | 2.03E-04 | 4.65E-03 | 1.76E-02 | 7.91E-03 |
| K02027$_{VIP\geq0.8\ (+)}$ | 1.099 | 0.809 | 1.527 | 0.796 | 1.266 | 0.903 | -4.97E-03 | 5.72E-03 | 9.82E-03 | 3.63E-04 | 1.31E-02 | 3.75E-03 |
| K02805$_{VIP\geq0.8\ (+)}$ | 1.793 | 1.041 | 1.426 | 1.173 | 0.568 | 1.894 | 1.23E-02 | -8.68E-03 | 1.21E-02 | 5.74E-03 | 6.00E-03 | 1.37E-02 |
| K06177$_{VIP\geq0.8\ (+)}$ | 0.907 | 0.995 | 0.67 | 0.972 | 1.494 | 1.199 | 3.53E-03 | 6.29E-03 | 3.67E-03 | 3.95E-03 | -1.52E-02 | 3.81E-03 |
| K00351$_{VIP\geq0.8\ (+)}$ | 1.226 | 1.659 | 1.585 | 0.465 | 1.43 | 0.845 | 4.23E-03 | 1.13E-02 | -1.68E-02 | 1.71E-03 | 1.80E-03 | 3.04E-03 |
| K07037$_{VIP\geq0.8\ (+)}$ | 0.889 | 0.852 | 1.068 | 0.903 | 0.975 | 0.795 | 1.18E-03 | 2.29E-03 | -2.91E-03 | 2.12E-03 | 5.84E-03 | 1.17E-03 |
| K03642$_{VIP\geq0.8\ (+)}$ | 1.203 | 1.025 | 0.722 | 1.611 | 1.409 | 1.378 | 9.86E-04 | 4.21E-03 | 2.10E-03 | 6.66E-03 | -1.54E-03 | 6.99E-03 |
| K00946$_{VIP\geq0.8\ (+)}$ | 1.058 | 1.845 | 0.591 | 1.286 | 1.825 | 1.056 | 2.66E-03 | 1.55E-02 | 1.97E-03 | 4.97E-03 | -1.54E-02 | 6.48E-06 |
| K02548$_{VIP\geq0.8\ (+)}$ | 1.325 | 1.117 | 0.515 | 1.168 | 1.447 | 1.143 | 5.10E-03 | 9.04E-04 | 2.70E-03 | 4.97E-03 | -3.69E-03 | 7.48E-04 |
| K11720$_{VIP\geq0.8\ (+)}$ | 1.139 | 1.261 | 0.75 | 1.513 | 1.244 | 1.1 | 1.95E-03 | 8.77E-03 | 5.49E-03 | 6.78E-03 | -6.49E-03 | 4.74E-03 |
| K00979$_{VIP\geq0.8\ (+)}$ | 1.017 | 1.1 | 0.588 | 1.078 | 0.94 | 1.367 | 3.30E-03 | 4.96E-03 | 3.13E-03 | 3.87E-03 | -9.58E-04 | 8.69E-03 |
| K03269$_{VIP\geq0.8\ (+)}$ | 1.083 | 1.041 | 0.775 | 1.318 | 1.557 | 0.953 | -4.31E-04 | 3.33E-03 | 1.04E-03 | 3.62E-03 | 1.25E-03 | 2.89E-04 |
| K03771$_{VIP\geq0.8\ (+)}$ | 1.027 | 0.613 | 0.972 | 1.554 | 1.316 | 1.034 | 6.72E-04 | 1.13E-03 | 2.91E-03 | 6.04E-03 | -1.32E-04 | 3.06E-04 |
| K02067$_{VIP\geq0.8\ (+)}$ | 0.936 | 1.219 | 0.728 | 1.323 | 1.281 | 0.935 | 6.48E-04 | 7.90E-03 | 5.50E-03 | 5.81E-03 | -9.75E-04 | 2.66E-03 |
| K00794$_{VIP\geq0.8\ (+)}$ | 1.019 | 2.042 | 0.913 | 1.615 | 0.977 | 0.435 | 4.23E-03 | 1.73E-02 | 3.90E-03 | 6.91E-03 | -2.24E-03 | 8.48E-04 |
| K00796$_{VIP\geq0.8\ (+)}$ | 1.192 | 1.211 | 0.8 | 0.662 | 1.607 | 1.176 | 6.36E-03 | 1.29E-03 | 8.77E-03 | 2.15E-03 | -1.45E-02 | 7.42E-03 |
| K06041$_{VIP\geq0.8\ (+)}$ | 1.411 | 1.729 | 0.746 | 1.511 | 1.274 | 0.912 | 4.28E-03 | 1.42E-02 | 8.10E-03 | 7.52E-03 | -6.18E-03 | 3.82E-03 |
| K00648$_{VIP\geq0.8\ (+)}$ | 1.199 | 1.204 | 2.007 | 0.922 | 0.953 | 0.68 | 7.39E-03 | 7.46E-03 | 2.18E-02 | 1.95E-03 | -5.20E-03 | 3.17E-03 |
| K01883$_{VIP\geq0.8\ (+)}$ | 0.899 | 1.461 | 1.172 | 0.704 | 0.887 | 0.833 | 6.51E-03 | -7.10E-03 | 5.73E-03 | 2.65E-03 | 8.92E-03 | 4.40E-03 |
| K01921$_{VIP\geq0.8\ (+)}$ | 1.251 | 1.413 | 0.836 | 1.102 | 1.582 | 0.529 | 8.86E-03 | 4.56E-03 | 5.74E-03 | 5.11E-03 | 1.30E-02 | -3.40E-03 |
| K00950$_{VIP\geq0.8\ (+)}$ | 1.182 | 1.134 | 0.732 | 1.515 | 1.522 | 1.1 | 7.17E-04 | 6.18E-03 | 1.47E-03 | 5.81E-03 | -1.17E-02 | 1.86E-03 |
| K02601$_{VIP\geq0.8\ (+)}$ | 0.82 | 1.113 | 0.874 | 0.853 | 0.377 | 1.042 | -4.58E-03 | 8.85E-03 | 8.88E-03 | 4.47E-03 | 3.34E-03 | 2.92E-03 |

| | | | | | | | | | | | | |
|---|---|---|---|---|---|---|---|---|---|---|---|---|
| K07107$_{VIP≥0.8\ (+)}$ | 1.529 | 0.786 | 1.205 | 1.509 | 0.941 | 1.31 | 6.29E-03 | 2.28E-03 | 1.13E-02 | 6.90E-03 | -9.06E-03 | 9.29E-03 |
| K09767$_{VIP≥0.8\ (+)}$ | 0.88 | 0.735 | 0.943 | 1.003 | 0.272 | 0.808 | 6.79E-03 | 3.88E-03 | -4.10E-03 | 4.62E-03 | 1.56E-03 | 5.88E-03 |
| K23004$_{VIP≥0.8\ (+)}$ | 0.706 | 0.989 | 1.008 | 1.351 | 0.774 | 1.575 | -5.39E-03 | 8.49E-03 | 7.00E-03 | 6.09E-03 | 6.74E-03 | 1.15E-02 |
| K05794$_{VIP≥0.8\ (+)}$ | 1.995 | 0.518 | 0.887 | 1.447 | 1.129 | 0.682 | -1.53E-02 | 1.43E-03 | 5.78E-03 | 6.10E-03 | 1.18E-02 | 1.17E-03 |
| K03658$_{VIP≥0.8\ (+)}$ | 1.522 | 1.069 | 0.68 | 1.217 | 0.228 | 0.861 | 1.07E-02 | 9.10E-03 | 2.53E-03 | 6.26E-03 | -2.40E-03 | 5.05E-03 |
| K13075$_{VIP≥0.8\ (+)}$ | 0.208 | 1.025 | 0.862 | 0.618 | 0.968 | 1.024 | 1.27E-03 | -8.63E-03 | 4.04E-03 | 3.20E-03 | 9.90E-03 | 7.17E-03 |
| K07488$_{VIP≥0.8\ (+)}$ | 0.817 | 1.301 | 0.499 | 1.016 | 0.296 | 0.947 | 4.13E-03 | 6.48E-03 | 2.11E-03 | 4.61E-03 | 1.92E-03 | -6.40E-03 |
| K09807$_{VIP≥0.8\ (+)}$ | 0.96 | 1.089 | 1.024 | 0.801 | 0.392 | 0.455 | 7.26E-03 | 9.34E-03 | 2.01E-03 | 1.98E-03 | 3.77E-03 | -2.00E-03 |
| K18700$_{VIP≥0.8\ (+)}$ | 0.53 | 0.899 | 0.952 | 1.163 | 1.012 | 0.087 | 4.06E-03 | -7.60E-03 | 8.15E-03 | 5.81E-03 | 1.05E-02 | 1.46E-04 |
| K24180$_{VIP≥0.8\ (+)}$ | 0.859 | 0.974 | 1.717 | 0.477 | 1.677 | 0.64 | 4.41E-03 | -3.90E-03 | 1.88E-02 | 2.45E-03 | 1.71E-02 | 2.61E-04 |
| K06215$_{VIP≥0.8\ (+)}$ | 1.88 | 1.141 | 1.055 | 0.131 | 0.621 | 1.8 | 1.39E-02 | 4.37E-03 | 5.69E-03 | -6.14E-04 | 4.77E-03 | 1.29E-02 |
| K07574$_{VIP≥0.8\ (+)}$ | 0.695 | 0.757 | 1.078 | 0.997 | 1.249 | 1.271 | -3.79E-04 | 5.05E-03 | 6.56E-03 | 2.03E-03 | 8.88E-03 | 2.59E-04 |
| K02072$_{VIP≥0.8\ (+)}$ | 0.901 | 0.053 | 1.09 | 0.971 | 0.761 | 1.652 | -5.10E-03 | 2.85E-04 | 6.57E-04 | 1.28E-03 | 5.58E-03 | 8.76E-03 |
| K06188$_{VIP≥0.8\ (+)}$ | 1.231 | 0.967 | 1.301 | 0.886 | 0.17 | 0.37 | 6.54E-03 | 6.18E-03 | -4.38E-03 | 7.83E-04 | 1.12E-03 | 5.24E-04 |
| K06958$_{VIP≥0.8\ (+)}$ | 0.906 | 0.482 | 1.023 | 0.965 | 0.476 | 0.904 | 5.92E-03 | 4.15E-03 | -1.89E-03 | 1.68E-04 | 4.88E-03 | 2.02E-03 |
| K05808$_{VIP≥0.8\ (+)}$ | 1.033 | 0.5 | 0.995 | 0.964 | 0.72 | 1.021 | 7.26E-03 | 3.87E-03 | -4.94E-03 | 1.22E-03 | 3.04E-03 | 4.56E-04 |
| K00346$_{VIP≥0.8\ (+)}$ | 1.263 | 1.232 | 0.709 | 1.201 | 1.658 | 0.793 | 3.26E-03 | 6.27E-03 | -7.78E-03 | 6.12E-03 | 1.37E-03 | 2.22E-03 |
| K04085$_{VIP≥0.8\ (+)}$ | 0.717 | 1.331 | 0.813 | 1.182 | 0.89 | 0.301 | 5.46E-03 | 1.14E-02 | 7.84E-03 | 6.17E-03 | -4.92E-03 | 2.22E-03 |
| K16694$_{VIP≥0.8\ (+)}$ | 0.778 | 1.128 | 0.296 | 1.713 | 1.175 | 1.038 | 1.22E-03 | 3.99E-03 | -1.53E-03 | 7.78E-03 | 3.36E-03 | 4.48E-03 |
| K01176$_{VIP≥0.8\ (+)}$ | 1.404 | 1.318 | 1.598 | 0.186 | 0.591 | 1.21 | 1.08E-02 | 1.07E-02 | 1.67E-02 | -6.69E-04 | 4.61E-03 | 8.14E-03 |
| K11752$_{VIP≥0.8\ (+)}$ | 2.239 | 1.613 | 0.042 | 1.299 | 0.691 | 0.817 | 1.54E-02 | 9.02E-03 | 4.48E-04 | 6.80E-03 | -5.36E-03 | 4.34E-03 |
| K02501$_{VIP≥0.8\ (+)}$ | 0.58 | 1.077 | 1.129 | 1.141 | 0.602 | 1.067 | -1.61E-03 | 2.12E-04 | 1.18E-02 | 5.82E-03 | 2.92E-03 | 6.87E-03 |
| K01934$_{VIP≥0.8\ (+)}$ | 1.362 | 1.303 | 2.017 | 0.793 | 0.397 | 1.142 | 8.33E-03 | 9.17E-03 | 1.94E-02 | 3.47E-03 | -2.37E-03 | 5.43E-03 |
| K06207$_{VIP≥0.8\ (+)}$ | 1.776 | 1.256 | 1.067 | 0.797 | 1.117 | 0.571 | 1.35E-02 | 2.69E-03 | -2.85E-03 | 3.21E-03 | 4.78E-03 | 2.77E-03 |
| K18831$_{VIP≥0.8\ (+)}$ | 1.419 | 0.871 | 0.581 | 1.593 | 0.91 | 0.461 | 6.54E-03 | 4.11E-04 | -4.76E-03 | 8.37E-03 | 3.48E-03 | 3.32E-03 |
| K06898$_{VIP≥0.8\ (+)}$ | 0.898 | 1.282 | 1.401 | 0.866 | 1.397 | 1.318 | 5.07E-04 | -1.02E-02 | 9.07E-03 | 1.17E-03 | 1.47E-02 | -8.48E-03 |
| K00340$_{VIP≥0.8\ (+)}$ | 1.183 | 0.97 | 0.962 | 1.305 | 0.932 | 0.841 | -1.14E-03 | 2.11E-03 | 5.75E-04 | 3.59E-03 | -8.11E-03 | 1.63E-03 |
| K00350$_{VIP≥0.8\ (+)}$ | 1.129 | 1.259 | 1.125 | 1.148 | 1.174 | 1.458 | 1.38E-03 | 5.10E-03 | -1.19E-02 | 4.55E-03 | -5.51E-04 | 9.40E-03 |
| K01633$_{VIP≥0.8\ (+)}$ | 1.241 | 1.259 | 0.904 | 1.248 | 1.33 | 0.996 | -4.25E-03 | 6.36E-03 | 3.91E-04 | 5.51E-03 | -1.08E-02 | 4.01E-03 |
| K21064$_{VIP≥0.8\ (+)}$ | 1.07 | 0.911 | 1.432 | 1.698 | 0.856 | 1.09 | -8.26E-03 | 7.51E-03 | 1.04E-02 | -7.88E-03 | 2.73E-03 | 2.62E-03 |

| | | | | | | | | | | | | |
|---|---|---|---|---|---|---|---|---|---|---|---|---|
| K01218$_{\text{VIP}\geq 0.8\ (+)}$ | 1.11 | 1.068 | 0.928 | 1.003 | 0.823 | 0.909 | 4.68E-03 | -5.74E-03 | 1.36E-03 | 1.06E-03 | -4.35E-03 | 3.67E-03 |
| K01627$_{\text{VIP}\geq 0.8\ (+)}$ | 1.231 | 1.736 | 0.841 | 1.362 | 1.136 | 1.002 | 4.09E-03 | 1.31E-02 | 3.90E-03 | 4.94E-03 | -1.00E-02 | -1.80E-03 |
| K07259$_{\text{VIP}\geq 0.8\ (+)}$ | 1.618 | 1.526 | 1.258 | 1.003 | 1.388 | 0.999 | 8.05E-03 | 8.83E-03 | -1.32E-02 | 3.98E-03 | 3.64E-03 | -9.53E-04 |
| K01613$_{\text{VIP}\geq 0.8\ (+)}$ | 0.966 | 1.123 | 1.153 | 1.382 | 1.407 | 0.836 | 2.89E-03 | 7.12E-03 | -3.79E-03 | 6.18E-03 | -3.28E-03 | 2.91E-03 |
| K07076$_{\text{VIP}\geq 0.8\ (+)}$ | 1.139 | 1.115 | 0.911 | 1.599 | 1.158 | 1.031 | 3.56E-03 | 9.49E-03 | -4.03E-03 | 8.11E-03 | 1.14E-02 | -7.61E-03 |
| K02115$_{\text{VIP}\geq 0.8\ (+)}$ | 0.981 | 0.815 | 1.03 | 1.168 | 0.962 | 0.988 | -6.65E-03 | -9.17E-04 | 1.34E-04 | 6.00E-03 | 5.78E-03 | 7.00E-03 |
| K07221$_{\text{VIP}\geq 0.8\ (+)}$ | 1.037 | 1.031 | 1.092 | 1.112 | 1.285 | 0.894 | 1.58E-04 | 8.84E-04 | -3.41E-03 | 9.59E-04 | -1.16E-02 | 2.03E-03 |
| K03585$_{\text{VIP}\geq 0.8\ (+)}$ | 1.277 | 1.278 | 0.971 | 1.213 | 1.693 | 1.229 | 8.65E-03 | 3.81E-03 | -2.56E-03 | 4.47E-03 | -1.42E-02 | 4.90E-03 |
| K20074$_{\text{VIP}\geq 0.8\ (+)}$ | 1.459 | 1.066 | 0.935 | 1.357 | 1.131 | 1.111 | 1.07E-02 | -6.19E-03 | -2.33E-03 | 7.13E-03 | 1.09E-02 | 7.84E-03 |
| K02970$_{\text{VIP}\geq 0.8\ (+)}$ | 1.24 | 1.749 | 1.035 | 1.114 | 1.254 | 0.931 | 7.76E-03 | 1.50E-02 | -2.43E-03 | 5.70E-03 | 1.33E-02 | -4.25E-03 |
| K01759$_{\text{VIP}\geq 0.8\ (+)}$ | 0.501 | 1.095 | 1.249 | 0.907 | 1.174 | 0.888 | -3.43E-03 | 9.34E-03 | 9.36E-03 | 8.26E-05 | 1.24E-02 | -4.35E-03 |
| K03303$_{\text{VIP}\geq 0.8\ (+)}$ | 0.945 | 0.623 | 0.954 | 2.032 | 1.247 | 1.842 | -7.11E-03 | 4.29E-03 | -3.02E-03 | 1.06E-02 | 1.13E-02 | 1.36E-02 |
| K03473$_{\text{VIP}\geq 0.8\ (+)}$ | 1.268 | 0.69 | 1.426 | 1.634 | 0.838 | 1.191 | 9.05E-03 | 5.17E-03 | -1.01E-02 | 8.43E-03 | -3.52E-03 | 6.86E-03 |
| K09680$_{\text{VIP}\geq 0.8\ (+)}$ | 0.988 | 0.4 | 1.012 | 0.978 | 0.944 | 0.807 | 3.59E-04 | -2.71E-03 | 5.84E-03 | 6.11E-04 | 7.90E-03 | -1.19E-03 |
| K07260$_{\text{VIP}\geq 0.8\ (+)}$ | 1.163 | 0.73 | 1.341 | 0.896 | 1.038 | 0.967 | 8.66E-03 | -2.81E-03 | 1.26E-02 | 2.96E-03 | -4.48E-05 | 2.11E-03 |
| K06204$_{\text{VIP}\geq 0.8\ (+)}$ | 1.269 | 1.081 | 1.074 | 1.562 | 1.076 | 0.733 | -5.90E-03 | 9.28E-03 | 1.92E-03 | 6.02E-03 | 9.82E-03 | -3.19E-03 |
| K00992$_{\text{VIP}\geq 0.8\ (+)}$ | 1.244 | 0.817 | 0.793 | 1.708 | 1.427 | 1.366 | 2.79E-03 | 6.27E-04 | -5.20E-03 | 7.63E-03 | -5.05E-03 | 6.52E-03 |
| K03281$_{\text{VIP}\geq 0.8\ (+)}$ | 1.276 | 1.482 | 0.175 | 1.008 | 1.276 | 0.888 | -4.01E-03 | 5.63E-03 | 6.11E-04 | 4.93E-03 | -6.92E-04 | 3.04E-04 |
| K17103$_{\text{VIP}\geq 0.8\ (+)}$ | 1.063 | 0.942 | 1.024 | 0.885 | 0.969 | 0.492 | -4.23E-03 | 4.57E-03 | 4.65E-05 | 3.25E-03 | 3.82E-03 | -1.91E-03 |
| K03612$_{\text{VIP}\geq 0.8\ (+)}$ | 0.979 | 0.907 | 0.782 | 1.239 | 2.06 | 0.823 | -5.74E-03 | 5.31E-03 | 7.66E-04 | 6.31E-03 | 2.16E-02 | -6.07E-03 |
| K03092$_{\text{VIP}\geq 0.8\ (+)}$ | 1.23 | 1.39 | 0.123 | 1.565 | 1.383 | 1.295 | -2.45E-04 | 6.23E-03 | -1.26E-03 | 7.77E-03 | 3.22E-03 | 8.58E-03 |
| K00927$_{\text{VIP}\geq 0.8\ (+)}$ | 1.1 | 1.082 | 1.16 | 0.808 | 0.759 | 1.079 | 8.52E-03 | 2.36E-03 | -1.04E-02 | 8.79E-04 | 4.03E-03 | -6.22E-03 |
| K01940$_{\text{VIP}\geq 0.8\ (+)}$ | 1.144 | 1.048 | 0.627 | 1.106 | 1.061 | 0.948 | 6.80E-03 | -4.84E-03 | -2.68E-03 | 5.66E-03 | 4.77E-03 | 6.93E-03 |
| K19334$_{\text{VIP}\geq 0.8\ (+)}$ | 1.747 | 1.313 | 0.613 | 0.983 | 1.119 | 1.273 | 1.19E-02 | 6.90E-04 | -1.05E-03 | 3.21E-03 | -2.78E-04 | 8.34E-03 |
| K06133$_{\text{VIP}\geq 0.8\ (+)}$ | 1.066 | 1.024 | 0.858 | 2.186 | 2.081 | 0.647 | -7.71E-03 | -7.81E-03 | 2.22E-04 | 1.15E-02 | 1.83E-02 | 3.68E-04 |
| K00705$_{\text{VIP}\geq 0.8\ (+)}$ | 1.347 | 1.357 | 0.628 | 0.95 | 1.933 | 1.138 | 1.04E-02 | -8.35E-03 | -6.88E-03 | 4.73E-03 | 1.79E-02 | 8.32E-03 |
| K01972$_{\text{VIP}\geq 0.8\ (+)}$ | 0.493 | 1.198 | 0.823 | 0.967 | 1.296 | 0.995 | -3.57E-03 | -2.95E-03 | 3.75E-03 | 4.12E-03 | 1.14E-02 | 5.97E-03 |
| K15921$_{\text{VIP}\geq 0.8\ (+)}$ | 0.406 | 0.879 | 1.068 | 1.549 | 1.278 | 1.073 | 7.73E-04 | -6.11E-03 | -2.23E-03 | 7.04E-03 | 1.04E-02 | 7.14E-03 |
| K02030$_{\text{VIP}\geq 0.8\ (+)}$ | 0.818 | 0.212 | 1.117 | 0.994 | 1.031 | 0.895 | -2.74E-03 | -1.14E-03 | 4.80E-03 | 2.00E-03 | 9.68E-03 | 7.19E-04 |
| K03559$_{\text{VIP}\geq 0.8\ (+)}$ | 1.005 | 1.072 | 0.965 | 1.653 | 1.451 | 0.657 | -1.76E-03 | 4.62E-03 | 5.29E-03 | 6.04E-03 | -1.16E-02 | 1.33E-04 |

| | | | | | | | | | | | | |
|---|---|---|---|---|---|---|---|---|---|---|---|---|
| $K04763_{VIP \geq 0.8\ (+)}$ | 0.94 | 1.296 | 0.684 | 0.874 | 0.842 | 1.341 | 7.16E-03 | -7.83E-03 | -2.94E-03 | 3.40E-03 | 3.24E-03 | 9.42E-03 |
| $K23356_{VIP \geq 0.8\ (+)}$ | 1.33 | 0.924 | 0.602 | 0.434 | 1.02 | 1.225 | 8.59E-03 | 5.81E-03 | 2.32E-03 | -1.75E-03 | 1.02E-02 | -8.57E-03 |
| $K22928_{VIP \geq 0.8\ (+)}$ | 0.596 | 1.43 | 1.196 | 1.166 | 1.319 | 0.772 | 4.58E-03 | 1.09E-02 | 1.07E-02 | -5.54E-03 | -1.32E-02 | 2.10E-04 |
| $K01679_{VIP \geq 0.8\ (+)}$ | 0.675 | 1.287 | 1.255 | 0.992 | 0.685 | 1.343 | 2.66E-03 | -1.03E-02 | 8.01E-03 | 1.15E-03 | 3.12E-03 | -9.91E-03 |
| $K07502_{VIP \geq 0.8\ (+)}$ | 0.945 | 1.282 | 0.982 | 0.781 | 0.197 | 1.115 | 5.67E-03 | -1.08E-02 | 4.81E-03 | -9.85E-04 | 1.70E-03 | 1.37E-03 |
| $K07794_{VIP \geq 0.8\ (+)}$ | 0.854 | 0.903 | 0.415 | 0.951 | 0.639 | 1.32 | -6.29E-03 | 7.64E-03 | 1.08E-03 | 3.37E-04 | 6.69E-03 | -9.61E-03 |
| $K21556_{VIP \geq 0.8\ (+)}$ | 1.071 | 1.495 | 1.164 | 0.373 | 0.661 | 0.807 | -7.97E-03 | 1.29E-02 | -6.42E-03 | 2.96E-04 | 3.33E-03 | 5.94E-03 |
| $K06933_{VIP \geq 0.8\ (+)}$ | 1.318 | 0.547 | 1.42 | 0.801 | 0.586 | 1.213 | -6.99E-03 | 3.85E-03 | 1.17E-02 | 7.24E-04 | 5.27E-03 | -5.13E-03 |
| $K23010_{VIP \geq 0.8\ (+)}$ | 0.047 | 0.805 | 0.773 | 1.547 | 0.846 | 0.933 | -9.19E-05 | -6.89E-03 | 2.04E-03 | 7.72E-03 | 6.62E-03 | 5.93E-03 |
| $K00756_{VIP \geq 0.8\ (+)}$ | 0.261 | 1.079 | 0.979 | 1.035 | 0.394 | 1.144 | 1.36E-03 | 6.49E-03 | 7.21E-03 | -3.77E-03 | -1.50E-03 | 6.03E-03 |
| $K01486_{VIP \geq 0.8\ (+)}$ | 1.245 | 0.881 | 0.847 | 0.563 | 1.842 | 0.473 | -9.03E-03 | 5.67E-03 | 9.15E-03 | 2.52E-03 | 1.95E-02 | -2.91E-03 |
| $K06209_{VIP \geq 0.8\ (+)}$ | 0.832 | 0.259 | 1.113 | 1.026 | 0.395 | 0.955 | -6.73E-04 | 2.21E-03 | 1.59E-03 | -2.20E-03 | 1.76E-03 | 9.37E-05 |
| $K01919_{VIP \geq 0.8\ (+)}$ | 0.416 | 1.123 | 1.012 | 0.535 | 0.996 | 0.91 | 7.19E-04 | -9.44E-03 | 8.37E-03 | -1.34E-03 | 2.54E-03 | 2.21E-03 |
| $K19689_{VIP \geq 0.8\ (+)}$ | 0.752 | 1.135 | 1.312 | 0.99 | 0.546 | 0.812 | 2.54E-03 | -9.25E-03 | 4.81E-03 | 8.31E-04 | 5.31E-03 | -3.13E-04 |
| $K01267_{VIP \geq 0.8\ (+)}$ | 1.392 | 0.054 | 1.563 | 0.957 | 0.536 | 1.167 | -1.06E-02 | 4.24E-04 | 1.26E-02 | 3.51E-03 | -4.94E-03 | 4.63E-03 |
| $K07054_{VIP \geq 0.8\ (+)}$ | 0.614 | 1.009 | 1.031 | 1.111 | 0.803 | 0.527 | -4.72E-03 | -8.56E-03 | 8.11E-04 | 3.92E-03 | 6.72E-03 | 1.56E-03 |
| $K03929_{VIP \geq 0.8\ (+)}$ | 1.077 | 0.957 | 1.242 | 1.266 | 0.659 | 0.62 | 3.32E-03 | -2.53E-03 | 1.33E-02 | 6.64E-03 | -6.55E-03 | 1.44E-03 |
| $K08722_{VIP \geq 0.8\ (+)}$ | 0.392 | 0.072 | 0.826 | 0.845 | 0.992 | 0.946 | 1.89E-03 | 5.70E-04 | -5.29E-03 | 9.99E-04 | 5.43E-03 | -1.45E-04 |
| $K02031_{VIP \geq 0.8\ (+)}$ | 0.923 | 0.556 | 1.19 | 0.818 | 0.489 | 1.022 | 2.74E-03 | -2.25E-03 | 4.71E-03 | 4.82E-04 | 5.06E-03 | -3.27E-03 |
| $K06940_{VIP \geq 0.8\ (+)}$ | 1.06 | 0.157 | 1.68 | 0.905 | 0.165 | 0.934 | 6.35E-03 | -8.19E-04 | 1.32E-02 | 3.31E-03 | -1.37E-03 | 2.21E-03 |
| $K07792_{VIP \geq 0.8\ (+)}$ | 0.342 | 0.605 | 0.807 | 0.843 | 1.229 | 0.91 | -2.64E-03 | 2.47E-03 | 5.08E-04 | -6.85E-04 | 1.69E-03 | 3.00E-03 |
| $K01119_{VIP \geq 0.8\ (+)}$ | 0.869 | 1.261 | 2.291 | 0.696 | 0.736 | 1.197 | 5.16E-03 | -3.94E-03 | 2.30E-02 | 2.09E-03 | -6.59E-03 | 8.58E-03 |
| $K18908_{VIP \geq 0.8\ (+)}$ | 0.9 | 0.374 | 1.068 | 0.679 | 1.013 | 1.885 | 4.77E-03 | 1.06E-03 | -4.00E-03 | 5.31E-04 | -1.07E-02 | 9.81E-03 |
| $K03474_{VIP \geq 0.8\ (+)}$ | 0.652 | 0.799 | 1.069 | 1.628 | 0.813 | 0.803 | 4.65E-03 | 6.81E-03 | -6.96E-03 | 8.46E-03 | -3.62E-03 | 3.02E-03 |
| $K02315_{VIP \geq 0.8\ (+)}$ | 0.63 | 0.627 | 1.416 | 1.33 | 0.946 | 1.163 | 1.45E-03 | -4.62E-03 | 9.99E-03 | -4.70E-03 | 4.44E-03 | 2.66E-03 |
| $K01139_{VIP \geq 0.8\ (+)}$ | 1.735 | 0.71 | 1.101 | 0.862 | 0.518 | 1.424 | 1.34E-02 | -5.03E-03 | -3.71E-03 | 1.91E-03 | 5.39E-03 | 9.80E-03 |
| $K08681_{VIP \geq 0.8\ (+)}$ | 1.299 | 1.043 | 1.187 | 0.309 | 0.598 | 1.003 | 8.45E-03 | 4.11E-03 | 9.73E-03 | -1.60E-03 | -5.22E-03 | 7.09E-03 |
| $K03118_{VIP \geq 0.8\ (+)}$ | 0.753 | 0.188 | 0.955 | 1.618 | 0.932 | 0.844 | -5.54E-03 | -1.08E-03 | 4.62E-03 | 7.96E-03 | 2.97E-03 | 2.29E-03 |
| $K03770_{VIP \geq 0.8\ (+)}$ | 1.261 | 1.235 | 0.444 | 0.671 | 1.606 | 1.241 | 2.43E-03 | 5.67E-03 | -4.70E-03 | 1.76E-03 | -2.98E-03 | 1.07E-03 |
| $K02073_{VIP \geq 0.8\ (+)}$ | 1.232 | 0.657 | 1.104 | 0.902 | 0.748 | 1.443 | -8.56E-03 | 5.52E-03 | -1.65E-03 | 1.77E-03 | 7.90E-03 | 8.12E-03 |

| | | | | | | | | | | | | |
|---|---|---|---|---|---|---|---|---|---|---|---|---|
| $K02426_{VIP\geq0.8\ (+)}$ | 1.035 | 0.82 | 1.194 | 0.926 | 0.322 | 0.438 | -3.31E-03 | 4.23E-03 | -1.23E-03 | 1.72E-03 | 2.53E-04 | 2.47E-04 |
| $K04773_{VIP\geq0.8\ (+)}$ | 1.19 | 1.172 | 0.564 | 1.727 | 0.777 | 1.044 | -2.92E-04 | 3.69E-03 | 2.33E-03 | 8.44E-03 | -1.30E-03 | 4.38E-03 |
| $K03284_{VIP\geq0.8\ (+)}$ | 2.091 | 1.327 | 0.704 | 0.967 | 1.291 | 0.568 | 1.58E-02 | 5.58E-03 | -5.07E-03 | 5.07E-03 | -9.14E-03 | 8.62E-04 |
| $K15738_{VIP\geq0.8\ (+)}$ | 0.58 | 1.403 | 0.917 | 0.539 | 1.165 | 1.012 | 1.47E-03 | -7.22E-03 | 2.42E-03 | 2.84E-03 | -6.80E-04 | 5.50E-03 |
| $K07386_{VIP\geq0.8\ (+)}$ | 0.955 | 0.903 | 0.389 | 1.056 | 0.642 | 1.073 | 1.78E-03 | 3.70E-03 | 1.12E-03 | 4.73E-03 | -2.22E-03 | -1.27E-03 |
| $K03616_{VIP\geq0.8\ (+)}$ | 0.822 | 0.765 | 0.662 | 1.266 | 1.445 | 1.052 | 4.33E-03 | 5.45E-04 | -3.11E-03 | 6.68E-03 | 1.52E-02 | -7.59E-03 |
| $K01297_{VIP\geq0.8\ (+)}$ | 0.453 | 0.405 | 0.934 | 1.522 | 0.977 | 1.2 | -3.04E-03 | -2.90E-03 | 6.00E-03 | 7.64E-03 | 9.33E-03 | 8.82E-03 |
| $K03613_{VIP\geq0.8\ (+)}$ | 0.605 | 0.953 | 0.741 | 1.779 | 1.405 | 0.81 | -2.65E-03 | 6.12E-03 | 2.95E-03 | 9.40E-03 | 1.41E-02 | -5.94E-03 |
| $K06901_{VIP\geq0.8\ (+)}$ | 0.515 | 1.44 | 0.392 | 1.222 | 1.641 | 1.718 | -3.98E-03 | -7.15E-03 | 1.08E-03 | 6.45E-03 | 1.52E-02 | 1.26E-02 |
| $K01912_{VIP\geq0.8\ (+)}$ | 0.512 | 0.908 | 0.915 | 1.039 | 1.392 | 0.205 | -3.22E-03 | -7.81E-03 | 2.61E-03 | 4.18E-03 | 1.47E-02 | 1.28E-03 |
| $K02337_{VIP\geq0.8\ (+)}$ | 0.872 | 1.292 | 0.992 | 0.695 | 1.49 | 0.166 | 6.74E-03 | 6.18E-04 | -5.90E-03 | 2.77E-04 | 1.09E-02 | -1.02E-03 |
| $K01809_{VIP\geq0.8\ (+)}$ | 0.907 | 0.812 | 0.959 | 1.11 | 0.542 | 0.532 | 5.45E-03 | -4.17E-03 | -1.76E-03 | 4.24E-03 | 1.52E-04 | 4.25E-05 |
| $K04066_{VIP\geq0.8\ (+)}$ | 0.665 | 1.042 | 0.836 | 0.859 | 0.899 | 0.458 | 4.53E-03 | -2.18E-03 | -2.49E-03 | 3.45E-03 | 3.35E-03 | 1.23E-03 |
| $K06904_{VIP\geq0.8\ (+)}$ | 1.279 | 0.452 | 1.192 | 0.283 | 0.981 | 1.105 | -9.82E-03 | -3.85E-03 | 1.27E-02 | 9.35E-04 | 1.00E-02 | 7.54E-03 |
| $K03106_{VIP\geq0.8\ (+)}$ | 0.908 | 1.293 | 0.894 | 0.817 | 0.672 | 0.563 | 5.85E-03 | 6.02E-04 | -1.74E-03 | 2.85E-03 | -5.75E-03 | 1.64E-03 |
| $K07005_{VIP\geq0.8\ (+)}$ | 0.575 | 1.786 | 1.617 | 1.138 | 0.479 | 1.494 | -3.42E-03 | -1.39E-02 | 1.59E-02 | 5.95E-03 | 5.06E-03 | 7.05E-03 |
| $K01991_{VIP\geq0.8\ (+)}$ | 1.024 | 0.681 | 1.004 | 1.386 | 1.138 | 0.786 | -2.83E-03 | 2.29E-03 | -9.75E-04 | 5.02E-03 | 8.82E-03 | 5.14E-04 |
| $K01588_{VIP\geq0.8\ (+)}$ | 1.092 | 0.845 | 1.004 | 1.109 | 0.553 | 0.407 | 2.23E-03 | 1.48E-03 | 1.07E-02 | 5.80E-03 | -5.82E-03 | -7.01E-05 |
| $K07114_{VIP\geq0.8\ (+)}$ | 1.043 | 0.491 | 1.068 | 1.537 | 0.968 | 0.752 | -1.75E-03 | 1.51E-03 | 2.85E-04 | 5.29E-03 | -4.03E-03 | 2.91E-03 |
| $K00973_{VIP\geq0.8\ (+)}$ | 1.387 | 1.059 | 0.132 | 1.768 | 0.841 | 0.169 | 1.01E-02 | -2.28E-03 | 9.56E-04 | 9.20E-03 | 8.99E-04 | -1.09E-03 |
| $K00615_{VIP\geq0.8\ (+)}$ | 0.294 | 1.33 | 1.046 | 0.847 | 0.742 | 1.001 | 2.20E-03 | -4.14E-03 | -9.45E-03 | 1.44E-03 | 7.75E-03 | 9.18E-04 |
| $K07568_{VIP\geq0.8\ (+)}$ | 1.008 | 1.4 | 0.539 | 1.527 | 0.949 | 0.146 | 4.20E-03 | 5.01E-03 | 1.52E-03 | 7.75E-03 | -3.04E-03 | -2.89E-04 |
| $K03699_{VIP\geq0.8\ (+)}$ | 1.006 | 1.407 | 0.941 | 1.054 | 0.077 | 0.137 | 5.93E-03 | -2.94E-03 | 2.30E-03 | 5.57E-03 | 2.71E-04 | -1.49E-04 |
| $K01154_{VIP\geq0.8\ (+)}$ | 1.563 | 1.507 | 0.92 | 0.764 | 0.427 | 1.126 | 1.18E-02 | -8.16E-03 | -4.89E-03 | 1.11E-03 | 2.24E-03 | 7.90E-03 |
| $K07133_{VIP\geq0.8\ (+)}$ | 1.185 | 0.825 | 0.835 | 0.943 | 0.659 | 0.355 | 6.48E-03 | 1.96E-03 | -1.48E-03 | 4.37E-03 | -7.62E-04 | 1.91E-03 |
| $K01666_{VIP\geq0.8\ (-)}$ | 1.701 | 0.82 | 1.457 | 0.904 | 1.046 | 0.947 | -1.27E-02 | -6.31E-03 | -1.54E-02 | -3.72E-03 | -8.43E-03 | -5.68E-03 |
| $K00620_{VIP\geq0.8\ (-)}$ | 0.965 | 1.126 | 0.993 | 1.204 | 1.251 | 1.376 | -2.07E-03 | -9.48E-03 | -8.10E-03 | -2.14E-03 | -4.05E-04 | -7.01E-03 |
| $K07404_{VIP\geq0.8\ (-)}$ | 0.962 | 1.311 | 1.036 | 0.644 | 0.949 | 0.847 | -6.15E-03 | -1.03E-02 | -1.86E-03 | -1.41E-03 | -9.52E-03 | -2.47E-03 |
| $K06023_{VIP\geq0.8\ (-)}$ | 1.511 | 0.81 | 0.786 | 0.939 | 1.393 | 1.299 | -8.70E-03 | -6.88E-03 | -2.98E-03 | -2.09E-03 | -2.48E-03 | -1.60E-03 |
| $K01156_{VIP\geq0.8\ (-)}$ | 1.972 | 1.224 | 1.392 | 0.791 | 2.081 | 0.322 | -1.50E-02 | -5.35E-03 | -1.51E-02 | -4.01E-03 | -2.13E-02 | -2.55E-04 |

| | | | | | | | | | | | | |
|---|---|---|---|---|---|---|---|---|---|---|---|---|
| $K03775_{VIP\geq0.8\ (-)}$ | 2.04 | 0.978 | 1.45 | 1.246 | 1.92 | 0.846 | -1.57E-02 | -4.81E-03 | -8.69E-03 | 5.63E-03 | -2.03E-02 | -5.76E-03 |
| $K23393_{VIP\geq0.8\ (-)}$ | 1.093 | 1.139 | 0.939 | 1.236 | 1.264 | 1.021 | -5.52E-03 | -9.10E-03 | -2.07E-03 | -3.59E-03 | 1.02E-02 | -2.64E-03 |
| $K06213_{VIP\geq0.8\ (-)}$ | 1.077 | 0.871 | 0.936 | 1.077 | 1.272 | 1.184 | -5.56E-03 | -6.66E-03 | -4.46E-03 | -3.40E-03 | -9.41E-04 | 5.15E-04 |
| $K13532_{VIP\geq0.8\ (-)}$ | 1.122 | 1.401 | 1.158 | 1.027 | 1.092 | 0.887 | -9.68E-04 | 1.01E-02 | -4.50E-03 | -1.62E-03 | -1.11E-02 | -1.85E-03 |
| $K00971_{VIP\geq0.8\ (-)}$ | 0.938 | 1.101 | 0.929 | 0.894 | 0.828 | 1.582 | -4.06E-03 | -1.27E-04 | -4.69E-03 | 1.50E-03 | -3.86E-03 | -7.79E-03 |
| $K10212_{VIP\geq0.8\ (-)}$ | 0.898 | 0.821 | 1.2 | 1.037 | 0.781 | 1.118 | -6.81E-03 | 6.77E-03 | -1.26E-02 | -4.23E-03 | -5.45E-04 | -4.37E-03 |
| $K06012_{VIP\geq0.8\ (-)}$ | 0.371 | 1.079 | 0.825 | 1.329 | 1.21 | 1.074 | 1.03E-03 | -9.05E-03 | -9.05E-03 | -5.40E-03 | -5.87E-03 | -1.34E-03 |
| $K05341_{VIP\geq0.8\ (-)}$ | 0.682 | 1.416 | 1.268 | 1.027 | 0.912 | 0.999 | -2.55E-03 | -1.20E-02 | -1.28E-02 | -2.67E-03 | 5.84E-03 | -4.82E-03 |
| $K03708_{VIP\geq0.8\ (-)}$ | 0.821 | 0.997 | 0.726 | 1.245 | 1.294 | 1.356 | -5.87E-03 | 8.44E-03 | -6.81E-03 | -5.04E-03 | -4.13E-03 | -3.00E-03 |
| $K22477_{VIP\geq0.8\ (-)}$ | 0.945 | 0.507 | 0.965 | 0.94 | 1.338 | 0.899 | 2.04E-03 | -1.87E-03 | -8.71E-03 | -3.13E-03 | -2.06E-03 | -1.86E-03 |
| $K07736_{VIP\geq0.8\ (-)}$ | 1.196 | 0.903 | 0.752 | 1.283 | 1.303 | 1.338 | -5.44E-03 | 7.57E-03 | -2.61E-03 | -3.37E-03 | -3.05E-03 | -5.77E-03 |
| $K16787_{VIP\geq0.8\ (-)}$ | 0.951 | 0.601 | 1.039 | 0.926 | 1.038 | 0.995 | -2.11E-03 | -1.73E-03 | -1.31E-03 | -5.01E-04 | 8.53E-03 | -2.65E-03 |
| $K16786_{VIP\geq0.8\ (-)}$ | 1.228 | 0.899 | 0.913 | 1.156 | 0.767 | 1.151 | -6.28E-03 | -7.28E-03 | -2.88E-03 | -2.53E-03 | -1.03E-03 | 1.54E-03 |
| $K06442_{VIP\geq0.8\ (-)}$ | 1.001 | 0.391 | 0.999 | 1.353 | 1.42 | 1.384 | -1.87E-03 | -2.93E-03 | -3.51E-04 | -4.17E-03 | 9.41E-03 | -5.20E-03 |
| $K06024_{VIP\geq0.8\ (-)}$ | 1.015 | 0.128 | 0.86 | 1.194 | 1.317 | 1.34 | -2.86E-03 | -1.09E-03 | -3.90E-03 | -2.81E-03 | 1.92E-03 | -1.89E-03 |
| $K00761_{VIP\geq0.8\ (-)}$ | 1.006 | 0.759 | 0.92 | 1.028 | 1.289 | 1.489 | -3.71E-03 | -6.53E-03 | -6.28E-03 | -1.53E-03 | 5.03E-03 | -7.25E-03 |
| $K23675_{VIP\geq0.8\ (-)}$ | 0.992 | 0.573 | 0.971 | 1.034 | 1.093 | 1.154 | -4.33E-03 | -4.33E-03 | -9.39E-04 | -1.65E-03 | 1.50E-03 | -3.00E-03 |
| $K08591_{VIP\geq0.8\ (-)}$ | 1.027 | 0.193 | 0.929 | 1.281 | 1.16 | 1.308 | -3.04E-03 | -1.62E-03 | -2.58E-03 | -4.04E-03 | 7.46E-04 | -4.74E-03 |
| $K03205_{VIP\geq0.8\ (-)}$ | 1.023 | 1.274 | 1.145 | 0.992 | 0.613 | 1.049 | 6.99E-03 | -9.93E-03 | -2.22E-03 | -1.32E-03 | -5.60E-03 | -7.27E-03 |
| $K01854_{VIP\geq0.8\ (-)}$ | 1.456 | 1.247 | 0.602 | 1.262 | 0.918 | 0.905 | 1.12E-02 | -4.28E-03 | -5.34E-03 | -6.53E-03 | -8.95E-03 | -6.41E-03 |
| $K01803_{VIP\geq0.8\ (-)}$ | 0.816 | 1.346 | 1.169 | 0.882 | 0.782 | 1.198 | 3.12E-03 | -9.13E-03 | -1.27E-02 | -3.46E-03 | -8.15E-03 | -5.77E-03 |
| $K11936_{VIP\geq0.8\ (-)}$ | 1.337 | 1.258 | 1.459 | 1.246 | 0.593 | 0.433 | -9.56E-03 | -4.41E-03 | -1.60E-02 | -5.32E-03 | -1.01E-03 | 2.30E-03 |
| $K06396_{VIP\geq0.8\ (-)}$ | 0.269 | 0.456 | 1.495 | 1.65 | 1.799 | 1.261 | 7.44E-04 | -2.67E-03 | -1.61E-02 | -7.81E-03 | -1.03E-02 | -3.20E-03 |
| $K06406_{VIP\geq0.8\ (-)}$ | 0.862 | 0.771 | 0.51 | 1.335 | 1.146 | 1.23 | 6.03E-03 | -6.47E-03 | -1.68E-03 | -5.48E-03 | -8.19E-03 | -2.82E-03 |
| $K06407_{VIP\geq0.8\ (-)}$ | 0.459 | 0.526 | 1.276 | 0.88 | 1.475 | 1.325 | -6.04E-04 | 1.10E-04 | -1.31E-02 | -3.09E-03 | -8.89E-03 | -5.03E-03 |
| $K04047_{VIP\geq0.8\ (-)}$ | 0.273 | 0.481 | 1.104 | 0.835 | 0.889 | 1.163 | -1.57E-03 | -3.98E-03 | -6.77E-03 | -2.54E-03 | -8.93E-03 | 7.76E-03 |
| $K23876_{VIP\geq0.8\ (-)}$ | 2.035 | 0.509 | 0.955 | 0.951 | 0.665 | 0.965 | -1.51E-02 | -3.72E-03 | -6.18E-04 | -2.89E-03 | -2.01E-03 | 1.56E-03 |
| $K04094_{VIP\geq0.8\ (-)}$ | 0.712 | 0.263 | 0.869 | 1.017 | 1.277 | 1.11 | -2.16E-03 | -8.77E-04 | -2.95E-03 | -2.08E-03 | 6.16E-03 | -4.58E-03 |
| $K04062_{VIP\geq0.8\ (-)}$ | 1.47 | 1.486 | 0.308 | 0.543 | 1.476 | 1.376 | -1.14E-02 | -1.04E-02 | 1.37E-03 | -2.73E-03 | -1.56E-02 | -6.93E-03 |
| $K07023_{VIP\geq0.8\ (-)}$ | 1.685 | 0.362 | 0.637 | 0.95 | 1.303 | 1.2 | -1.02E-02 | -2.53E-03 | -4.36E-04 | -1.79E-03 | 7.84E-03 | -2.34E-03 |

| | | | | | | | | | | | | |
|---|---|---|---|---|---|---|---|---|---|---|---|---|
| K16511$_{VIP≥0.8\ (-)}$ | 0.642 | 1.107 | 0.994 | 1.046 | 0.6 | 1.399 | -3.33E-04 | -9.46E-03 | -4.44E-03 | -3.57E-03 | -2.73E-03 | 7.20E-03 |
| K06872$_{VIP≥0.8\ (-)}$ | 1.507 | 0.56 | 1.812 | 0.528 | 1.088 | 0.917 | -1.15E-02 | -5.80E-04 | -1.82E-02 | -2.71E-03 | 1.42E-03 | -4.14E-03 |
| K03525$_{VIP≥0.8\ (-)}$ | 1.373 | 1.521 | 0.286 | 0.825 | 0.6 | 1.255 | -8.47E-03 | -1.31E-02 | -6.27E-04 | -6.59E-04 | -3.12E-03 | 1.47E-03 |
| K04043$_{VIP≥0.8\ (-)}$ | 0.143 | 1.269 | 1.009 | 0.822 | 1.188 | 0.525 | -6.20E-04 | -4.92E-03 | -5.10E-03 | -1.48E-03 | 9.52E-03 | -1.94E-03 |
| K06909$_{VIP≥0.8\ (-)}$ | 0.941 | 0.478 | 1.251 | 0.496 | 0.94 | 1.338 | -7.26E-03 | -4.10E-03 | 1.37E-02 | -2.55E-03 | -6.49E-03 | -6.40E-03 |
| K03593$_{VIP≥0.8\ (-)}$ | 0.754 | 0.94 | 1.609 | 0.169 | 1.184 | 0.871 | -5.13E-03 | -2.29E-04 | -1.19E-02 | -7.14E-04 | 1.19E-02 | -5.43E-03 |
| K09710$_{VIP≥0.8\ (-)}$ | 0.388 | 0.921 | 0.975 | 0.492 | 1.273 | 0.937 | -4.69E-04 | 7.62E-03 | -1.56E-04 | -1.26E-03 | -1.25E-02 | -6.62E-03 |
| K07035$_{VIP≥0.8\ (-)}$ | 0.936 | 1.417 | 0.943 | 1.199 | 1.054 | 1.065 | -3.89E-03 | -9.48E-03 | 7.01E-03 | -4.52E-03 | -1.76E-03 | 6.10E-06 |
| K15771$_{VIP≥0.8\ (-)}$ | 1.006 | 1.017 | 1.297 | 1.211 | 1.471 | 1.032 | 1.31E-03 | -8.75E-03 | -1.36E-02 | -4.28E-03 | -6.08E-03 | 1.08E-04 |
| K16053$_{VIP≥0.8\ (-)}$ | 2.315 | 1.181 | 1.064 | 1.356 | 1.372 | 0.947 | 1.66E-02 | -4.11E-03 | -1.04E-02 | -6.46E-03 | -1.41E-02 | 6.50E-04 |
| K11176$_{VIP≥0.8\ (-)}$ | 1.108 | 1.104 | 0.839 | 1.116 | 1.353 | 1.374 | -1.64E-04 | -9.49E-03 | 1.84E-03 | -1.40E-03 | 3.39E-03 | -2.53E-04 |
| K01966$_{VIP≥0.8\ (-)}$ | 1.061 | 0.944 | 1.482 | 1.105 | 0.848 | 1.816 | -3.56E-03 | 5.45E-03 | -1.57E-02 | -5.64E-03 | -4.98E-03 | 1.31E-02 |
| K16870$_{VIP≥0.8\ (-)}$ | 1.038 | 1.231 | 0.861 | 1.341 | 1.047 | 1.071 | -1.95E-03 | -3.38E-03 | 3.89E-04 | 4.41E-03 | -9.24E-03 | -2.67E-03 |
| K04744$_{VIP≥0.8\ (-)}$ | 0.974 | 0.835 | 1.505 | 1.006 | 1.16 | 1.081 | -1.25E-03 | 1.09E-03 | -1.27E-02 | 1.53E-03 | -4.60E-03 | -1.08E-03 |
| K00046$_{VIP≥0.8\ (-)}$ | 1.19 | 1.013 | 1.129 | 0.823 | 1.573 | 1.289 | 5.76E-03 | 2.74E-03 | -5.38E-03 | -9.47E-04 | -1.62E-02 | -6.11E-03 |
| K04486$_{VIP≥0.8\ (-)}$ | 1.207 | 0.835 | 0.978 | 1.106 | 1.169 | 1.385 | -5.48E-03 | -7.08E-03 | 1.09E-03 | -2.64E-03 | 4.65E-04 | -1.84E-03 |
| K07030$_{VIP≥0.8\ (-)}$ | 1.202 | 1.329 | 1.094 | 1.36 | 1.498 | 1.598 | -6.00E-03 | -1.14E-02 | -1.15E-02 | -5.31E-03 | 5.74E-03 | 7.06E-03 |
| K01284$_{VIP≥0.8\ (-)}$ | 0.834 | 0.974 | 0.837 | 0.873 | 1.405 | 1.069 | 6.20E-04 | -7.99E-04 | -3.50E-03 | 2.78E-03 | -2.58E-03 | -1.32E-03 |
| K04072$_{VIP≥0.8\ (-)}$ | 1.404 | 1.609 | 1.57 | 1.142 | 1.292 | 0.733 | -9.97E-03 | -1.37E-02 | -1.71E-02 | -5.27E-03 | 3.25E-03 | 1.59E-03 |
| K03750$_{VIP≥0.8\ (-)}$ | 1.195 | 0.22 | 0.944 | 0.925 | 1.899 | 1.932 | -9.17E-03 | 1.56E-03 | -2.40E-03 | -3.45E-03 | 1.93E-02 | -1.41E-02 |
| K19048$_{VIP≥0.8\ (-)}$ | 1.024 | 1.21 | 1.372 | 1.057 | 0.489 | 0.808 | 5.71E-03 | -9.97E-03 | 1.06E-02 | -3.86E-03 | -2.96E-03 | -4.08E-03 |
| K02770$_{VIP≥0.8\ (-)}$ | 0.733 | 1.206 | 1.166 | 1.098 | 0.857 | 1.085 | -9.03E-05 | -6.88E-03 | -8.55E-03 | -3.42E-03 | 7.61E-03 | 7.22E-03 |
| K02007$_{VIP≥0.8\ (-)}$ | 0.74 | 0.801 | 1.597 | 0.973 | 1.243 | 0.977 | -6.11E-05 | -6.53E-03 | 1.16E-02 | -3.31E-03 | 1.29E-02 | -3.93E-03 |
| K15772$_{VIP≥0.8\ (-)}$ | 0.803 | 0.323 | 1.262 | 1.319 | 1.197 | 1.08 | 6.26E-04 | -2.75E-03 | -1.29E-02 | -4.16E-03 | 2.83E-04 | -3.62E-03 |
| K07726$_{VIP≥0.8\ (-)}$ | 1.213 | 1.252 | 0.644 | 1.173 | 0.991 | 1.11 | -9.33E-03 | -1.01E-02 | 6.80E-03 | -4.53E-03 | 2.79E-03 | -6.02E-03 |
| K07099$_{VIP≥0.8\ (-)}$ | 0.884 | 0.51 | 0.905 | 1.124 | 1.64 | 1.277 | -8.46E-04 | 4.13E-03 | -8.09E-03 | -2.11E-03 | 1.31E-02 | -3.36E-03 |
| K00027$_{VIP≥0.8\ (-)}$ | 0.883 | 0.976 | 0.238 | 1.449 | 1.356 | 1.256 | -5.49E-03 | 6.92E-03 | 2.31E-03 | -7.39E-03 | -6.63E-03 | -4.48E-03 |
| K03216$_{VIP≥0.8\ (-)}$ | 1.164 | 0.815 | 0.623 | 1.233 | 1.492 | 1.477 | -3.34E-03 | 5.38E-03 | -2.28E-03 | -4.07E-03 | 4.89E-03 | -7.12E-03 |
| K00991$_{VIP≥0.8\ (-)}$ | 1.314 | 0.41 | 1.027 | 0.818 | 1.304 | 0.883 | -9.62E-03 | 3.45E-03 | -2.57E-03 | -9.68E-04 | -1.11E-02 | 6.05E-04 |
| K09772$_{VIP≥0.8\ (-)}$ | 0.849 | 0.172 | 0.906 | 1.201 | 0.993 | 1.132 | -8.38E-04 | 1.47E-03 | -2.37E-03 | -3.05E-03 | 4.05E-03 | -1.67E-03 |

| | | | | | | | | | | | | |
|---|---|---|---|---|---|---|---|---|---|---|---|---|
| K06167$_{VIP\geq0.8\ (-)}$ | 1.367 | 0.876 | 1.119 | 1.365 | 0.869 | 0.613 | -7.25E-03 | -2.32E-03 | -2.67E-03 | 4.20E-03 | 7.19E-03 | -1.58E-04 |
| K03183$_{VIP\geq0.8\ (-)}$ | 0.993 | 0.621 | 1.311 | 1.238 | 1.046 | 0.983 | -1.66E-03 | 2.64E-04 | -6.46E-03 | 2.89E-03 | -9.84E-03 | -1.27E-03 |
| K07075$_{VIP\geq0.8\ (-)}$ | 1.006 | 0.402 | 1.461 | 0.853 | 1.499 | 0.972 | -7.39E-03 | -1.86E-03 | -8.54E-03 | 1.80E-03 | 1.52E-02 | -3.87E-03 |
| K16785$_{VIP\geq0.8\ (-)}$ | 0.946 | 0.555 | 1.055 | 1.226 | 1.34 | 1.208 | -4.84E-03 | -2.60E-03 | -2.46E-03 | -3.63E-03 | 1.14E-02 | 1.31E-03 |
| K06223$_{VIP\geq0.8\ (-)}$ | 1.395 | 1.54 | 1.138 | 0.448 | 1.268 | 1.346 | -1.08E-02 | -1.25E-02 | 1.23E-02 | 2.07E-03 | -1.34E-02 | -9.23E-03 |
| K13002$_{VIP\geq0.8\ (-)}$ | 1.006 | 1.168 | 0.791 | 0.975 | 0.907 | 1.537 | -1.55E-03 | 3.41E-03 | -5.78E-04 | 2.03E-03 | -7.68E-03 | -6.77E-03 |
| K08640$_{VIP\geq0.8\ (-)}$ | 0.652 | 1.075 | 1.211 | 1.552 | 1.515 | 1.102 | -6.07E-04 | -8.92E-03 | -5.26E-03 | 6.39E-03 | -1.60E-02 | 5.89E-03 |
| K03797$_{VIP\geq0.8\ (-)}$ | 0.937 | 1.35 | 0.323 | 0.924 | 1.357 | 1.077 | 3.18E-04 | -2.50E-03 | -2.51E-03 | 3.11E-03 | -3.47E-03 | -3.90E-03 |
| K07052$_{VIP\geq0.8\ (-)}$ | 0.627 | 1.029 | 0.879 | 0.894 | 0.952 | 1.123 | 1.99E-03 | -8.36E-03 | -8.77E-03 | -4.43E-03 | -3.75E-03 | 1.84E-03 |
| K00986$_{VIP\geq0.8\ (-)}$ | 0.686 | 1.451 | 1.064 | 0.835 | 0.829 | 1.001 | -5.31E-03 | -1.22E-02 | 3.91E-03 | -1.59E-03 | 8.76E-03 | -7.17E-03 |
| K07387$_{VIP\geq0.8\ (-)}$ | 1.79 | 0.366 | 2.478 | 0.195 | 1.355 | 0.98 | -1.37E-02 | -3.06E-03 | -2.20E-02 | 7.45E-04 | 1.38E-02 | -6.92E-03 |
| K00441$_{VIP\geq0.8\ (-)}$ | 1.556 | 1.417 | 1.072 | 0.74 | 0.225 | 1.292 | -1.17E-02 | -1.22E-02 | 1.18E-02 | 3.80E-03 | -2.31E-03 | -9.54E-03 |
| K00563$_{VIP\geq0.8\ (-)}$ | 0.675 | 1.046 | 0.505 | 1.013 | 1.162 | 1.091 | -1.76E-03 | -8.93E-03 | 1.71E-03 | -4.22E-03 | -5.21E-04 | 6.12E-03 |
| K04769$_{VIP\geq0.8\ (-)}$ | 1.586 | 0.289 | 0.595 | 1.404 | 1.067 | 1.167 | 1.20E-02 | -1.23E-03 | 5.23E-03 | -6.02E-03 | -1.02E-02 | -4.66E-03 |
| K06295$_{VIP\geq0.8\ (-)}$ | 0.589 | 0.306 | 1.128 | 1.003 | 0.883 | 1.094 | 4.48E-03 | -2.11E-03 | -1.20E-02 | -2.21E-03 | -1.54E-03 | 1.99E-03 |
| K01575$_{VIP\geq0.8\ (-)}$ | 1.175 | 0.941 | 0.505 | 0.971 | 1.006 | 0.396 | -7.59E-03 | -7.98E-03 | -2.54E-03 | -4.04E-03 | 6.65E-03 | 2.58E-03 |
| K02018$_{VIP\geq0.8\ (-)}$ | 0.712 | 1.311 | 0.398 | 1.055 | 1.036 | 2.331 | -4.49E-03 | -1.08E-02 | 1.75E-03 | -2.71E-03 | 1.09E-02 | -1.72E-02 |
| K03623$_{VIP\geq0.8\ (-)}$ | 0.953 | 0.385 | 0.865 | 0.607 | 0.812 | 0.908 | -7.36E-03 | -3.12E-03 | 4.79E-03 | -2.98E-03 | 4.98E-03 | -3.47E-03 |
| K01496$_{VIP\geq0.8\ (-)}$ | 1.421 | 1.55 | 0.763 | 0.66 | 0.987 | 0.9 | -9.82E-03 | -1.30E-02 | -8.36E-03 | 1.01E-03 | -6.25E-04 | 5.37E-04 |
| K23997$_{VIP\geq0.8\ (-)}$ | 0.928 | 2.314 | 0.692 | 1.007 | 0.563 | 1.066 | -3.95E-03 | -1.97E-02 | 4.54E-04 | 1.78E-03 | -3.45E-03 | -2.52E-03 |
| K07402$_{VIP\geq0.8\ (-)}$ | 0.826 | 0.236 | 1.013 | 0.986 | 0.336 | 1.611 | 2.38E-03 | -2.02E-03 | -4.05E-03 | 3.58E-04 | -3.35E-03 | -1.12E-02 |
| K19411$_{VIP\geq0.8\ (-)}$ | 2.612 | 0.506 | 0.148 | 1.38 | 1.307 | 1.299 | -2.02E-02 | 3.94E-03 | -1.44E-03 | -5.12E-03 | 5.40E-04 | -1.57E-03 |
| K06198$_{VIP\geq0.8\ (-)}$ | 1.523 | 1.472 | 1.099 | 1.393 | 0.641 | 0.739 | -1.18E-02 | -1.22E-02 | -9.53E-03 | 7.07E-03 | -2.26E-03 | 4.08E-03 |
| K01749$_{VIP\geq0.8\ (-)}$ | 1.308 | 0.643 | 1.212 | 0.997 | 0.326 | 1.859 | -8.95E-03 | -3.97E-03 | 6.52E-03 | -1.52E-03 | 2.89E-03 | -1.36E-02 |
| K05364$_{VIP\geq0.8\ (-)}$ | 0.479 | 1.248 | 1.1 | 0.937 | 0.289 | 1.02 | 4.31E-04 | -1.04E-02 | -1.78E-03 | -2.10E-03 | -2.40E-03 | 6.59E-03 |
| K04656$_{VIP\geq0.8\ (-)}$ | 0.574 | 1.384 | 1.288 | 1.15 | 0.437 | 1.109 | -1.40E-03 | -1.02E-02 | 4.29E-03 | -3.82E-03 | 4.01E-03 | -5.22E-03 |
| K03639$_{VIP\geq0.8\ (-)}$ | 1.009 | 0.831 | 0.698 | 1.033 | 0.692 | 2.162 | -4.54E-03 | -7.09E-03 | 1.52E-03 | -2.27E-03 | 6.92E-03 | -1.59E-02 |
| K09707$_{VIP\geq0.8\ (-)}$ | 1.917 | 0.42 | 0.802 | 0.246 | 1.218 | 0.894 | -1.44E-02 | -3.47E-03 | 4.82E-03 | -6.47E-04 | -6.87E-04 | 6.06E-03 |
| K08963$_{VIP\geq0.8\ (-)}$ | 0.758 | 0.978 | 1.183 | 0.882 | 0.212 | 1.117 | -2.07E-03 | -1.24E-03 | 2.38E-03 | -9.61E-04 | 2.24E-03 | -6.30E-03 |
| K02203$_{VIP\geq0.8\ (-)}$ | 1.256 | 0.475 | 0.51 | 1.147 | 1.408 | 1.239 | -5.23E-03 | -1.72E-03 | 2.29E-04 | -2.63E-03 | 3.69E-03 | -3.44E-03 |

| | | | | | | | | | | | | |
|---|---|---|---|---|---|---|---|---|---|---|---|---|
| K02119$_{VIP\geq0.8\ (-)}$ | 0.609 | 1.014 | 0.631 | 0.907 | 1.342 | 1.245 | 9.41E-04 | -8.56E-03 | -4.33E-03 | -3.66E-03 | 5.94E-03 | -6.29E-03 |
| K04654$_{VIP\geq0.8\ (-)}$ | 0.705 | 0.758 | 1.576 | 1.005 | 0.872 | 1.091 | -1.95E-03 | -5.51E-03 | 9.15E-03 | -1.66E-03 | 9.08E-03 | -5.81E-03 |
| K22927$_{VIP\geq0.8\ (-)}$ | 0.87 | 0.541 | 1.094 | 1.153 | 0.753 | 1.006 | -4.73E-03 | -1.66E-03 | -3.97E-03 | -2.77E-03 | 4.34E-03 | 2.09E-03 |
| K01926$_{VIP\geq0.8\ (-)}$ | 1.691 | 0.62 | 0.659 | 1.15 | 1.414 | 1.501 | -1.13E-02 | -4.06E-03 | 1.15E-04 | -2.59E-03 | 2.27E-03 | -7.29E-03 |
| K03321$_{VIP\geq0.8\ (-)}$ | 1.068 | 0.198 | 1.148 | 1.034 | 0.299 | 0.998 | -8.02E-03 | -1.63E-03 | -5.39E-03 | 4.23E-03 | -1.77E-03 | 6.95E-03 |
| K00941$_{VIP\geq0.8\ (-)}$ | 1.319 | 0.39 | 1.573 | 0.857 | 0.662 | 1.5 | -1.01E-02 | -3.27E-03 | 8.53E-03 | -2.49E-03 | -1.67E-03 | 7.98E-03 |
| K09787$_{VIP\geq0.8\ (-)}$ | 0.899 | 0.603 | 0.558 | 1.127 | 1.451 | 1.372 | -1.71E-03 | 4.17E-03 | -3.24E-03 | -2.98E-03 | 3.24E-03 | -1.88E-03 |
| K03789$_{VIP\geq0.8\ (-)}$ | 1.218 | 0.685 | 0.545 | 1.303 | 1.579 | 1.313 | -4.37E-03 | 3.92E-03 | -9.70E-05 | -4.45E-03 | 5.85E-03 | -5.78E-03 |
| K03151$_{VIP\geq0.8\ (-)}$ | 0.667 | 0.599 | 1.089 | 0.995 | 0.936 | 0.824 | 1.29E-03 | -4.92E-03 | -7.48E-03 | -6.35E-04 | 7.47E-03 | -1.19E-03 |
| K05601$_{VIP\geq0.8\ (-)}$ | 0.349 | 0.816 | 1.34 | 0.835 | 0.75 | 0.952 | -1.98E-03 | -5.60E-03 | 4.65E-03 | -2.13E-03 | 5.65E-03 | -3.98E-03 |
| K18346$_{VIP\geq0.8\ (-)}$ | 1 | 0.793 | 0.819 | 0.958 | 0.521 | 0.901 | 7.74E-03 | -3.79E-03 | -8.78E-03 | -4.66E-03 | -2.08E-04 | 1.88E-04 |
| K03643$_{VIP\geq0.8\ (-)}$ | 1.251 | 1.426 | 0.341 | 0.413 | 1.226 | 0.868 | 2.75E-05 | -9.70E-03 | -3.71E-03 | 8.88E-04 | -8.04E-03 | -1.18E-03 |
| K07164$_{VIP\geq0.8\ (-)}$ | 0.887 | 0.769 | 1.514 | 0.97 | 0.379 | 0.872 | 1.97E-04 | 3.33E-03 | -8.09E-03 | -8.04E-04 | -7.56E-04 | -3.48E-03 |
| K03783$_{VIP\geq0.8\ (-)}$ | 0.788 | 1.169 | 1.133 | 1.195 | 1.1 | 0.478 | -2.46E-03 | -5.83E-03 | -8.78E-03 | 5.22E-03 | 8.81E-03 | -3.43E-03 |
| K09760$_{VIP\geq0.8\ (-)}$ | 0.325 | 1.228 | 0.855 | 0.987 | 0.864 | 0.399 | 1.45E-03 | -2.75E-03 | -4.73E-03 | 4.80E-03 | -9.04E-03 | -2.75E-03 |
| K02784$_{VIP\geq0.8\ (-)}$ | 1.734 | 0.641 | 0.61 | 0.891 | 1.439 | 1.301 | -9.56E-03 | -5.06E-03 | 1.60E-03 | -1.71E-03 | 4.57E-03 | -1.63E-03 |
| K19353$_{VIP\geq0.8\ (-)}$ | 1.843 | 1.088 | 1.193 | 0.45 | 1.29 | 0.668 | -1.40E-02 | -4.17E-03 | -1.29E-02 | 2.38E-03 | -1.55E-03 | 4.69E-03 |
| K07063$_{VIP\geq0.8\ (-)}$ | 0.33 | 0.552 | 1.201 | 0.89 | 1.249 | 0.876 | -8.71E-04 | 4.43E-03 | -5.90E-03 | -2.36E-03 | 1.08E-02 | -6.39E-03 |
| K00012$_{VIP\geq0.8\ (-)}$ | 0.974 | 1.138 | 1.204 | 0.12 | 0.978 | 0.656 | -6.24E-03 | 8.50E-03 | -1.23E-02 | 5.98E-04 | -1.03E-02 | -3.36E-03 |
| K01869$_{VIP\geq0.8\ (-)}$ | 1.246 | 1.276 | 1.336 | 0.627 | 0.71 | 0.862 | 9.23E-03 | -6.62E-03 | -1.29E-02 | -3.65E-04 | 1.23E-03 | -5.11E-03 |
| K03545$_{VIP\geq0.8\ (-)}$ | 0.607 | 1.132 | 0.924 | 0.95 | 0.53 | 1.513 | 4.67E-03 | -3.48E-03 | -2.65E-03 | -3.79E-03 | -2.59E-03 | 8.56E-03 |
| K01790$_{VIP\geq0.8\ (-)}$ | 1.038 | 1.077 | 0.713 | 1.818 | 0.898 | 0.56 | -3.47E-03 | -2.01E-03 | 5.97E-03 | 9.31E-03 | -9.42E-03 | -3.74E-03 |
| K00558$_{VIP\geq0.8\ (-)}$ | 0.078 | 1.344 | 1.326 | 0.102 | 1.528 | 1.103 | 4.70E-04 | -3.96E-03 | 1.41E-02 | -5.28E-04 | -1.62E-02 | -7.82E-03 |
| K03111$_{VIP\geq0.8\ (-)}$ | 1.686 | 0.212 | 1.496 | 0.364 | 0.862 | 0.946 | -1.29E-02 | -9.70E-04 | 1.60E-02 | 4.56E-04 | -4.87E-03 | -2.37E-04 |
| K06864$_{VIP\geq0.8}$ | 0.88 | 1.018 | 1.369 | 0.834 | 0.906 | 1.222 | -5.58E-04 | -8.02E-03 | 6.37E-03 | 1.09E-03 | 9.53E-03 | -8.39E-03 |
| K05521$_{VIP\geq0.8}$ | 1.041 | 1.264 | 1.336 | 0.913 | 0.889 | 1.383 | 3.35E-03 | 9.87E-03 | 1.07E-02 | -1.83E-03 | -4.26E-03 | -9.48E-03 |
| K02026$_{VIP\geq0.8}$ | 1.133 | 0.921 | 0.91 | 0.816 | 1.337 | 0.876 | -5.34E-03 | 6.34E-03 | 5.74E-03 | -4.27E-04 | 1.41E-02 | -4.51E-04 |
| K04516$_{VIP\geq0.8}$ | 1.039 | 1.229 | 1.016 | 0.834 | 1.445 | 1.525 | 1.10E-03 | 1.78E-05 | -7.73E-03 | 1.21E-03 | -1.09E-02 | -5.82E-03 |
| K01685$_{VIP\geq0.8}$ | 1.934 | 1.286 | 1.087 | 0.934 | 1.019 | 0.803 | 1.45E-02 | -1.01E-03 | -8.28E-03 | 3.08E-03 | -3.28E-04 | 4.27E-03 |
| K00812$_{VIP\geq0.8}$ | 1.057 | 1.071 | 0.888 | 1.083 | 0.876 | 1.044 | 1.07E-04 | 3.35E-03 | -2.24E-03 | 1.96E-03 | -6.89E-03 | -8.04E-04 |

| | | | | | | | | | | | | |
|---|---|---|---|---|---|---|---|---|---|---|---|---|
| $K00683_{VIP≥0.8}$ | 1.064 | 1.319 | 0.906 | 0.842 | 1.189 | 1.289 | -1.50E-03 | 8.00E-03 | 1.66E-03 | 1.69E-03 | -1.02E-02 | -6.85E-03 |
| $K10536_{VIP≥0.8}$ | 1.264 | 1.16 | 1.042 | 1.255 | 1.716 | 1.483 | -4.12E-03 | -3.63E-03 | 2.44E-04 | 3.80E-03 | 1.42E-02 | -7.39E-03 |
| $K06076_{VIP≥0.8}$ | 0.815 | 1.03 | 1.103 | 1.303 | 0.812 | 1.363 | -3.19E-04 | -5.38E-03 | -3.66E-03 | 3.00E-03 | 3.97E-03 | 6.85E-03 |
| $K14445_{VIP≥0.8}$ | 0.938 | 1.324 | 1.028 | 0.915 | 1.005 | 1.371 | 3.97E-03 | 3.55E-03 | -8.49E-03 | -1.57E-03 | 3.22E-03 | -1.37E-03 |
| $K00645_{VIP≥0.8}$ | 0.864 | 1.072 | 1.869 | 1.501 | 1.264 | 0.859 | -4.55E-03 | 3.32E-03 | -1.82E-02 | 7.93E-03 | -1.29E-02 | 6.27E-03 |
| $K07317_{VIP≥0.8}$ | 1.086 | 1.34 | 0.826 | 1.031 | 0.827 | 1.038 | 7.81E-03 | -7.75E-03 | -6.93E-03 | 4.80E-03 | -9.93E-04 | 6.45E-03 |
| $K01005_{VIP≥0.8}$ | 0.926 | 1.063 | 1.124 | 1.12 | 0.832 | 1.059 | 2.49E-03 | -8.24E-03 | 9.78E-05 | -2.24E-03 | -1.20E-03 | 2.20E-03 |
| $K06142_{VIP≥0.8}$ | 1.011 | 1.086 | 1.035 | 1.302 | 1.203 | 0.969 | -1.90E-03 | 7.28E-03 | 1.10E-03 | 3.18E-03 | -4.93E-03 | -2.16E-04 |
| $K03785_{VIP≥0.8}$ | 0.522 | 1.025 | 1.108 | 1.172 | 1.01 | 1.062 | 3.38E-03 | -8.77E-03 | 5.08E-03 | -5.40E-03 | -4.80E-03 | 5.67E-03 |
| $K03299_{VIP≥0.8}$ | 1.242 | 0.54 | 0.915 | 0.957 | 0.851 | 0.871 | -3.56E-03 | 9.18E-04 | 9.00E-03 | -4.15E-03 | 4.63E-03 | -3.95E-03 |
| $K05305_{VIP≥0.8}$ | 0.825 | 0.994 | 2.068 | 0.91 | 0.94 | 0.491 | -6.38E-03 | -5.13E-03 | 2.26E-02 | 2.20E-03 | -9.29E-03 | 3.49E-03 |
| $K01512_{VIP≥0.8}$ | 1.598 | 0.873 | 0.553 | 0.845 | 1.193 | 0.805 | -1.09E-02 | -6.26E-03 | 5.31E-03 | -3.52E-03 | 3.54E-03 | 4.35E-03 |
| $K21744_{VIP≥0.8}$ | 1.782 | 0.897 | 0.382 | 1.069 | 1.225 | 1.85 | 1.22E-02 | -7.61E-03 | 3.77E-05 | -5.13E-03 | -3.30E-03 | 1.06E-02 |
| $K00878_{VIP≥0.8}$ | 1.228 | 0.965 | 1.159 | 0.945 | 0.61 | 0.955 | -8.88E-03 | -7.83E-03 | 5.06E-03 | -2.93E-03 | 1.98E-03 | 6.06E-04 |
| $K00965_{VIP≥0.8}$ | 0.958 | 0.182 | 1.085 | 1.312 | 1.06 | 1.005 | 3.08E-04 | -9.78E-04 | -1.26E-03 | -4.38E-03 | 1.04E-02 | 6.26E-03 |
| $K09121_{VIP≥0.8}$ | 0.813 | 1.149 | 1.356 | 0.926 | 0.266 | 1.133 | 9.94E-04 | -7.04E-03 | 7.20E-03 | -1.35E-04 | 2.70E-03 | -7.00E-03 |
| $K00626_{VIP≥0.8}$ | 0.978 | 0.902 | 1.161 | 1.372 | 0.536 | 1.199 | -4.14E-03 | -7.34E-03 | 8.74E-04 | -5.37E-03 | 5.59E-03 | 2.60E-03 |
| $K04655_{VIP≥0.8}$ | 0.992 | 1.314 | 1.29 | 1.051 | 0.434 | 0.966 | 6.26E-03 | -1.10E-02 | 4.24E-03 | -1.93E-03 | 4.16E-03 | -4.98E-03 |
| $K04653_{VIP≥0.8}$ | 0.901 | 0.057 | 1.404 | 0.935 | 1.542 | 1.251 | -5.60E-03 | 4.37E-04 | 1.07E-02 | -1.61E-03 | 1.63E-02 | -6.69E-03 |
| $K21395_{VIP≥0.8}$ | 0.998 | 0.073 | 1.028 | 0.976 | 1.242 | 1.142 | -6.15E-03 | 2.76E-04 | 4.09E-03 | -2.28E-03 | 1.24E-02 | -7.98E-03 |
| $K23535_{VIP≥0.8}$ | 1.115 | 1.221 | 1.134 | 0.947 | 0.338 | 1.217 | 6.58E-03 | -9.98E-03 | 5.16E-03 | -3.62E-04 | 3.26E-03 | -7.04E-03 |
| $K03705_{VIP≥0.8}$ | 0.813 | 0.047 | 1.108 | 1.102 | 1.129 | 1.212 | 4.79E-04 | 1.65E-04 | -7.37E-03 | -1.99E-03 | 1.64E-03 | -1.26E-03 |
| $K00788_{VIP≥0.8}$ | 0.899 | 0.995 | 1.624 | 0.374 | 1.238 | 0.991 | -6.83E-03 | -8.55E-03 | 1.23E-02 | 2.77E-04 | -4.93E-03 | 2.89E-04 |
| $K11145_{VIP≥0.8}$ | 0.948 | 0.166 | 0.964 | 1.147 | 1.326 | 1.274 | 1.43E-03 | 1.21E-03 | 1.83E-03 | -1.96E-03 | -4.07E-04 | -6.29E-04 |
| $K03976_{VIP≥0.8}$ | 1.659 | 1.015 | 0.462 | 0.918 | 1.296 | 1.108 | -1.15E-02 | 8.55E-03 | -2.50E-03 | -4.03E-03 | 1.33E-02 | 4.55E-03 |
| $K03523_{VIP≥0.8}$ | 0.869 | 0.515 | 1.043 | 1.135 | 1.339 | 1.203 | -1.02E-03 | 2.28E-03 | 2.07E-03 | -2.04E-03 | 5.51E-03 | -1.32E-03 |
| $K09015_{VIP≥0.8}$ | 1.104 | 1.312 | 0.366 | 0.898 | 1.355 | 1.189 | 2.67E-04 | -4.90E-03 | 9.75E-04 | 1.75E-04 | -1.22E-02 | -1.31E-03 |
| $K06960_{VIP≥0.8}$ | 1.146 | 0.802 | 0.244 | 0.883 | 1.424 | 1.331 | -5.93E-03 | 4.77E-03 | 9.24E-04 | -4.31E-04 | 3.98E-03 | -1.34E-03 |
| $K07102_{VIP≥0.8}$ | 1.478 | 0.92 | 0.515 | 1.359 | 1.451 | 1.225 | 7.46E-03 | -3.31E-05 | -2.38E-03 | 5.67E-03 | -2.72E-04 | 4.66E-03 |
| $K00721_{VIP≥0.8}$ | 1.06 | 1.242 | 1.071 | 1.024 | 0.563 | 0.992 | -2.00E-03 | 1.01E-02 | 5.74E-05 | 1.19E-03 | -3.64E-03 | -4.43E-03 |

| | | | | | | | | | | | | |
|---|---|---|---|---|---|---|---|---|---|---|---|---|
| $K01144_{VIP≥0.8}$ | 1.065 | 1.21 | 0.77 | 0.995 | 1.639 | 1.106 | 1.92E-03 | 3.84E-03 | -2.75E-03 | -1.64E-03 | 1.19E-02 | -2.87E-03 |
| $K00241_{VIP≥0.8}$ | 0.989 | 1.059 | 0.896 | 0.721 | 1.253 | 1.108 | 4.34E-04 | 4.48E-04 | -1.71E-03 | -3.46E-06 | -5.18E-03 | 8.04E-04 |
| $K12994_{VIP≥0.8}$ | 0.373 | 1.045 | 1.134 | 1.655 | 1.006 | 1.11 | -1.78E-03 | -8.21E-03 | -2.38E-03 | 7.41E-03 | 7.65E-03 | 5.83E-05 |
| $K05807_{VIP≥0.8}$ | 1.07 | 0.776 | 1.163 | 1.775 | 1.643 | 1.169 | -6.94E-04 | 4.89E-03 | -3.64E-03 | 7.28E-03 | -1.12E-02 | 3.46E-03 |
| $K09747_{VIP≥0.8}$ | 0.627 | 0.951 | 0.924 | 0.869 | 1.081 | 1.307 | -1.18E-03 | 6.21E-03 | 3.78E-03 | 6.12E-04 | -6.38E-04 | -1.66E-03 |
| $K01918_{VIP≥0.8}$ | 1.451 | 0.85 | 0.562 | 1.329 | 1.106 | 0.873 | -8.86E-03 | -6.77E-06 | 2.05E-03 | 6.24E-03 | -6.58E-03 | 3.24E-03 |
| $K07277_{VIP≥0.8}$ | 1.411 | 1.174 | 0.67 | 0.832 | 1.332 | 0.988 | 6.41E-03 | 5.15E-03 | -7.28E-03 | 4.31E-03 | -2.10E-03 | -6.91E-04 |
| $K06919_{VIP≥0.8}$ | 1.069 | 1.317 | 1.024 | 0.604 | 0.932 | 1.593 | 6.54E-03 | -1.13E-02 | 1.11E-02 | 5.56E-04 | -7.61E-03 | -1.17E-02 |
| $K23242_{VIP≥0.8}$ | 0.916 | 0.861 | 0.52 | 0.877 | 1.159 | 0.959 | -2.89E-03 | -7.32E-03 | 5.67E-03 | 4.28E-03 | 7.82E-03 | -3.37E-03 |
| $K02897_{VIP≥0.8}$ | 1.164 | 0.749 | 1.159 | 1.887 | 1.871 | 1.055 | 2.94E-04 | 4.18E-03 | -4.81E-03 | 8.44E-03 | -1.80E-02 | -4.18E-03 |
| $K05837_{VIP≥0.8}$ | 1.097 | 1.139 | 0.661 | 1.353 | 1.853 | 1.029 | -1.22E-03 | 3.58E-03 | -3.52E-03 | 5.97E-03 | -1.47E-02 | 6.68E-03 |
| $K01262_{VIP≥0.8}$ | 0.268 | 0.941 | 0.933 | 1.646 | 1.267 | 1.538 | 2.05E-06 | -6.02E-03 | -9.67E-03 | 8.68E-03 | -3.97E-04 | 1.12E-02 |
| $K09014_{VIP≥0.8}$ | 1.133 | 1.3 | 0.916 | 0.239 | 1.003 | 1.016 | 8.45E-03 | -3.12E-03 | -7.59E-03 | 3.27E-05 | -7.98E-03 | 6.92E-03 |
| $K06113_{VIP≥0.8}$ | 1.348 | 1.258 | 0.61 | 1.375 | 0.872 | 1.385 | 8.18E-03 | -3.07E-03 | -1.06E-03 | 6.19E-03 | -3.56E-03 | 8.84E-03 |
| $K07729_{VIP≥0.8}$ | 1.236 | 0.605 | 1.015 | 1.154 | 1.668 | 1.071 | -6.21E-03 | 2.11E-03 | 1.11E-02 | -5.27E-03 | 1.20E-02 | -2.52E-04 |
| $K12340_{VIP≥0.8}$ | 0.924 | 0.704 | 0.817 | 1.327 | 0.841 | 1.144 | -6.83E-03 | -2.74E-03 | 5.29E-03 | 4.72E-03 | -2.80E-03 | 3.26E-03 |
| $K00969_{VIP≥0.8}$ | 0.92 | 1.414 | 1.118 | 1.737 | 0.787 | 1.082 | -5.89E-04 | -6.35E-03 | 9.72E-03 | 9.09E-03 | -2.68E-04 | 7.07E-03 |
| $K00075_{VIP≥0.8}$ | 1.009 | 0.817 | 0.986 | 0.991 | 0.502 | 1.095 | 7.69E-03 | -4.20E-04 | -7.48E-03 | 5.05E-03 | -8.25E-04 | 5.60E-03 |
| $K06131_{VIP≥0.8}$ | 1.159 | 1.449 | 0.345 | 0.887 | 1.233 | 0.934 | 8.67E-03 | -5.22E-03 | -3.71E-03 | 3.42E-03 | -9.02E-03 | 3.95E-03 |
| $K03561_{VIP≥0.8}$ | 1.562 | 1.391 | 0.864 | 1.212 | 0.885 | 0.788 | -9.01E-03 | 4.46E-03 | 2.29E-03 | 4.69E-03 | -3.36E-03 | -5.53E-03 |
| $K07263_{VIP≥0.8}$ | 1.086 | 1.167 | 0.44 | 0.837 | 1.297 | 1.27 | 3.82E-03 | -3.64E-03 | 2.31E-03 | 1.87E-03 | -1.51E-03 | -9.72E-04 |
| $K02429_{VIP≥0.8}$ | 1.162 | 1.279 | 0.46 | 0.939 | 1.315 | 1.158 | -4.66E-03 | 2.88E-04 | -2.37E-03 | 1.69E-03 | -9.88E-03 | 5.80E-03 |
| $K07154_{VIP≥0.8}$ | 1.307 | 1.246 | 0.938 | 1.693 | 0.676 | 0.936 | 8.48E-03 | -7.51E-03 | -6.25E-03 | 7.88E-03 | -4.50E-03 | 4.77E-03 |
| $K21572_{VIP≥0.8}$ | 1.01 | 1.343 | 0.324 | 1.057 | 1.391 | 1.111 | -6.76E-04 | -1.38E-03 | 2.83E-03 | 4.99E-03 | -1.25E-02 | 7.74E-03 |
| $K05566_{VIP≥0.8}$ | 1.446 | 0.698 | 1.132 | 0.942 | 1.582 | 0.441 | 1.11E-02 | -7.78E-04 | -8.91E-04 | 4.50E-03 | 1.58E-02 | -2.36E-04 |
| $K07503_{VIP≥0.8}$ | 1.238 | 0.264 | 1.005 | 0.957 | 0.565 | 0.843 | -8.56E-03 | 2.27E-03 | -4.54E-03 | 5.05E-03 | 2.43E-03 | -6.16E-03 |
| $K02019_{VIP≥0.8}$ | 0.208 | 0.878 | 0.872 | 0.012 | 2.241 | 0.871 | -7.64E-05 | 7.12E-03 | 8.76E-03 | -6.19E-05 | 2.36E-02 | -4.76E-03 |
| $K01304_{VIP≥0.8}$ | 1.159 | 1.119 | 0.755 | 0.215 | 1.09 | 1.718 | -3.69E-03 | 4.67E-03 | 7.49E-03 | -5.50E-05 | -8.89E-03 | 1.07E-02 |
| $K02499_{VIP≥0.8}$ | 0.785 | 1.049 | 1 | 0.4 | 0.987 | 1.275 | -4.21E-03 | 9.03E-03 | -9.82E-03 | 2.01E-03 | -2.88E-03 | 6.01E-03 |
| $K13566_{VIP≥0.8}$ | 1.085 | 1.239 | 1.184 | 0.996 | 0.783 | 0.794 | -7.83E-03 | -9.71E-03 | 1.10E-02 | -3.63E-03 | 8.05E-03 | 6.32E-04 |

| | | | | | | | | | | | | |
|---|---|---|---|---|---|---|---|---|---|---|---|---|
| $K01500_{VIP\geq0.8}$ | 0.939 | 0.548 | 0.81 | 0.573 | 1.966 | 1 | 2.93E-03 | -4.65E-03 | -2.10E-03 | 1.07E-03 | 2.08E-02 | -3.53E-03 |
| $K02279_{VIP\geq0.8}$ | 0.594 | 0.752 | 1.777 | 1.038 | 0.845 | 1.697 | 9.62E-04 | -6.08E-03 | 1.28E-02 | -1.67E-03 | 8.88E-03 | -1.17E-02 |
| $K01193_{VIP\geq0.8}$ | 0.859 | 0.503 | 1.107 | 0.547 | 1.584 | 0.938 | -2.12E-03 | -2.65E-03 | 8.68E-03 | -3.88E-04 | 1.68E-02 | 3.85E-03 |
| $K01085_{VIP\geq0.8}$ | 0.824 | 0.52 | 0.726 | 1.365 | 1.495 | 0.912 | -6.35E-03 | -6.98E-04 | 4.63E-03 | 6.27E-03 | -1.53E-02 | 6.26E-03 |
| $K07080_{VIP\geq0.8}$ | 1.477 | 0.289 | 0.918 | 0.945 | 0.667 | 0.966 | -1.02E-02 | -1.48E-03 | 2.94E-04 | 1.81E-04 | 6.72E-03 | -3.77E-03 |
| $K09769_{VIP\geq0.8}$ | 1.02 | 0.335 | 0.723 | 0.994 | 1.338 | 1.529 | -7.51E-03 | 1.09E-03 | 6.06E-03 | -2.49E-03 | 5.45E-03 | -7.68E-03 |
| $K09516_{VIP\geq0.8}$ | 1.155 | 0.86 | 1.229 | 1.209 | 0.38 | 0.669 | 6.49E-03 | 3.81E-03 | -5.92E-03 | -4.88E-03 | -2.91E-03 | 3.96E-03 |
| $K20276_{VIP\geq0.8}$ | 1.044 | 0.329 | 1.107 | 0.786 | 1.418 | 1.028 | -7.92E-03 | 2.34E-03 | -8.15E-03 | 4.15E-03 | 1.40E-02 | -7.48E-03 |
| $K02283_{VIP\geq0.8}$ | 0.581 | 2.304 | 1.285 | 0.887 | 0.106 | 0.861 | -7.73E-04 | -1.98E-02 | 2.60E-03 | -2.21E-03 | 1.66E-04 | 2.45E-03 |
| $K22452_{VIP\geq0.8}$ | 1.628 | 0.113 | 0.936 | 0.972 | 0.143 | 1.4 | 1.26E-02 | -9.75E-04 | -1.67E-04 | 4.17E-03 | -1.16E-03 | 9.76E-03 |
| $K01609_{VIP\geq0.8}$ | 0.63 | 1.138 | 0.583 | 0.934 | 1.321 | 1.511 | -8.06E-04 | 9.35E-03 | -1.58E-03 | -9.80E-04 | 2.12E-04 | 3.38E-03 |
| $K01728_{VIP\geq0.8}$ | 1.085 | 1.192 | 0.802 | 1.001 | 0.769 | 0.42 | -1.09E-03 | -1.71E-03 | 2.68E-03 | -1.85E-03 | 3.72E-03 | 9.94E-04 |
| $K08602_{VIP\geq0.8}$ | 1.061 | 0.528 | 1.14 | 0.891 | 0.353 | 0.939 | 6.23E-03 | -3.40E-03 | 1.54E-04 | -1.24E-03 | 2.95E-03 | -8.65E-04 |
| $K00549_{VIP\geq0.8}$ | 0.982 | 0.835 | 1.145 | 0.92 | 0.263 | 0.49 | -7.31E-03 | -6.23E-03 | 3.05E-03 | -3.35E-03 | 1.13E-03 | 1.85E-03 |
| $K06894_{VIP\geq0.8}$ | 0.992 | 1.062 | 0.453 | 0.205 | 1.908 | 0.894 | -1.19E-03 | -1.26E-03 | -4.76E-03 | 1.88E-04 | 1.41E-02 | 1.49E-03 |
| $K23536_{VIP\geq0.8}$ | 1.161 | 0.615 | 1.116 | 0.913 | 0.606 | 1.351 | 7.24E-03 | -4.93E-03 | 3.97E-03 | -2.16E-04 | 6.38E-03 | -7.48E-03 |
| $K06975_{VIP\geq0.8}$ | 0.963 | 1.882 | 0.817 | 0.984 | 0.647 | 0.462 | 6.86E-03 | 1.60E-02 | -6.07E-03 | -3.55E-03 | -6.83E-03 | 1.95E-03 |
| $K07742_{VIP\geq0.8}$ | 1.03 | 0.684 | 0.341 | 1.028 | 1.406 | 1.355 | -3.53E-03 | 4.07E-03 | 1.56E-03 | -2.85E-03 | 4.23E-03 | -3.38E-04 |
| $K07335_{VIP\geq0.8}$ | 0.908 | 0.123 | 1.326 | 0.957 | 0.658 | 0.848 | 4.59E-03 | -1.04E-03 | 5.91E-03 | -1.00E-03 | 6.94E-03 | -4.15E-03 |
| $K00240_{VIP\geq0.8}$ | 1.153 | 1.586 | 0.881 | 0.62 | 0.936 | 0.327 | -4.66E-03 | 7.20E-03 | -8.63E-03 | -2.64E-03 | 4.23E-03 | 2.10E-03 |
| $K01754_{VIP\geq0.8}$ | 1.786 | 1.086 | 0.738 | 0.863 | 0.217 | 0.958 | -1.38E-02 | 8.79E-03 | -2.96E-04 | -1.33E-03 | 8.84E-04 | 4.86E-03 |
| $K00566_{VIP\geq0.8}$ | 1.257 | 0.622 | 1.252 | 0.951 | 0.755 | 0.908 | 8.64E-03 | 5.18E-03 | -1.06E-02 | -4.64E-04 | 7.80E-03 | -1.17E-03 |
| $K21908_{VIP\geq0.8}$ | 0.865 | 1.263 | 0.682 | 0.261 | 1.671 | 1.454 | -1.71E-03 | -1.06E-02 | 4.95E-03 | 1.38E-03 | 1.74E-02 | -1.03E-02 |
| $K04771_{VIP\geq0.8}$ | 0.92 | 1.228 | 1.016 | 0.689 | 1.259 | 0.446 | 1.64E-04 | 2.31E-04 | -1.11E-02 | 3.64E-03 | -1.16E-02 | -2.33E-03 |
| $K19693_{VIP\geq0.8}$ | 0.645 | 1.418 | 0.339 | 1.252 | 0.962 | 1.646 | -7.91E-04 | -3.99E-03 | 2.12E-03 | 6.37E-03 | -6.01E-03 | 1.15E-02 |
| $K19158_{VIP\geq0.8}$ | 1.391 | 1.321 | 1.587 | 0.466 | 0.871 | 0.379 | 9.85E-03 | 1.13E-02 | -1.23E-02 | -1.44E-03 | -8.78E-03 | 2.79E-03 |
| $K03286_{VIP\geq0.8}$ | 1.238 | 0.902 | 1.038 | 1.976 | 0.622 | 0.577 | -8.81E-03 | 7.55E-03 | -2.93E-03 | 9.80E-03 | 2.69E-03 | -2.34E-03 |
| $K04567_{VIP\geq0.8}$ | 0.811 | 1.416 | 0.802 | 0.612 | 1.056 | 0.118 | 5.60E-03 | -8.36E-03 | -5.43E-03 | -1.48E-03 | 3.85E-03 | 8.68E-04 |
| $K01224_{VIP\geq0.8}$ | 2.032 | 1.102 | 1.1 | 0.576 | 1.393 | 0.373 | 1.36E-02 | 1.40E-03 | -7.54E-03 | -8.49E-04 | -1.27E-02 | 1.08E-03 |
| $K03596_{VIP\geq0.8}$ | 1.296 | 1.138 | 1.071 | 0.787 | 0.781 | 0.852 | 9.77E-03 | -2.71E-03 | -1.87E-03 | 3.37E-03 | -2.39E-03 | 4.88E-03 |

| | | | | | | | | | | | | |
|---|---|---|---|---|---|---|---|---|---|---|---|---|
| $K00174_{VIP\geq0.8}$ | 1.084 | 1.2 | 0.261 | 0.153 | 0.978 | 1.508 | 4.26E-03 | 2.60E-03 | -1.58E-03 | -8.11E-04 | 3.26E-03 | -9.71E-03 |
| $K11754_{VIP\geq0.8}$ | 0.65 | 1.207 | 0.738 | 1.222 | 1.232 | 1.007 | 1.77E-03 | -2.77E-03 | -7.13E-03 | 6.26E-03 | -1.26E-02 | 7.25E-03 |
| $K07391_{VIP\geq0.8}$ | 0.677 | 1.429 | 0.921 | 0.491 | 0.955 | 0.995 | 4.62E-03 | -6.68E-03 | 2.78E-03 | 3.98E-04 | -6.81E-03 | -5.97E-03 |
| $K21571_{VIP\geq0.8}$ | 0.867 | 1.454 | 0.302 | 0.729 | 1.293 | 1.1 | -2.58E-03 | -7.97E-03 | 2.11E-03 | 2.47E-03 | -1.34E-02 | 6.20E-03 |
| $K19302_{VIP\geq0.8}$ | 1.242 | 1.29 | 0.667 | 0.064 | 0.895 | 1.044 | -7.55E-03 | 8.58E-03 | -9.50E-04 | 6.75E-05 | 3.26E-03 | -4.72E-03 |
| $K19157_{VIP\geq0.8}$ | 0.927 | 2.012 | 0.733 | 2.029 | 1.023 | 0.052 | -4.48E-03 | 1.72E-02 | -4.03E-03 | 1.06E-02 | 5.74E-04 | -9.71E-05 |
| $K01875_{VIP\geq0.8}$ | 0.55 | 1.218 | 1.197 | 0.789 | 0.873 | 0.93 | -3.76E-03 | -4.82E-03 | -1.18E-02 | 1.75E-03 | 1.16E-03 | 2.21E-03 |
| $K00975_{VIP\geq0.8}$ | 0.704 | 0.154 | 1.125 | 0.987 | 0.895 | 1.008 | -2.90E-03 | 1.18E-03 | -1.91E-03 | -7.20E-04 | 8.89E-03 | 3.42E-03 |
| $K02029_{VIP\geq0.8}$ | 0.74 | 0.315 | 1.213 | 1.026 | 0.86 | 1.047 | -2.13E-03 | -1.69E-03 | 3.85E-03 | 6.22E-04 | 6.86E-03 | -5.11E-04 |
| $K03602_{VIP\geq0.8}$ | 0.809 | 0.872 | 0.963 | 1.059 | 0.714 | 0.699 | -5.13E-03 | 7.50E-03 | 4.81E-03 | 5.35E-03 | -2.98E-03 | -2.16E-04 |
| $K02963_{VIP\geq0.8}$ | 0.47 | 1.284 | 1.373 | 0.349 | 0.901 | 1.054 | 1.75E-03 | 1.06E-02 | -1.23E-02 | 1.51E-03 | -9.08E-03 | -5.73E-03 |
| $K07001_{VIP\geq0.8}$ | 2.063 | 0.954 | 0.86 | 0.575 | 1.465 | 0.44 | 1.56E-02 | -2.42E-03 | 9.06E-03 | 1.81E-03 | -1.43E-02 | -1.88E-03 |
| $K01209_{VIP\geq0.8}$ | 1.715 | 1.406 | 0.543 | 0.938 | 0.878 | 0.633 | 1.21E-02 | -6.42E-04 | -3.69E-03 | 3.64E-03 | -5.47E-03 | 3.34E-03 |
| $K07271_{VIP\geq0.8}$ | 0.824 | 1.517 | 0.244 | 1.128 | 0.759 | 1.292 | -1.65E-03 | -8.43E-03 | -1.09E-03 | 5.52E-03 | 7.80E-03 | 8.49E-03 |
| $K01153_{VIP\geq0.8}$ | 1.252 | 1.505 | 0.818 | 0.716 | 0.511 | 0.931 | 9.57E-03 | -7.81E-03 | -2.06E-03 | 1.12E-03 | -1.29E-03 | 6.88E-03 |
| $K03497_{VIP\geq0.8}$ | 0.331 | 1.153 | 1.52 | 0.989 | 1.501 | 0.742 | 1.49E-03 | -7.46E-03 | 9.70E-03 | 1.58E-03 | -1.46E-02 | -3.49E-03 |
| $K02004_{VIP\geq0.8}$ | 0.751 | 1.583 | 0.565 | 1.809 | 1.226 | 1.155 | -3.99E-03 | -1.02E-02 | -4.97E-03 | 9.56E-03 | 9.21E-03 | 8.50E-03 |
| K02781 | 0.55 | 0.04 | 1.825 | 0.769 | 0.912 | 0.929 | -3.47E-03 | -4.78E-05 | 1.92E-02 | -2.28E-03 | -1.46E-03 | 4.75E-03 |
| K01780 | 1.086 | 0.635 | 0.898 | 0.36 | 1.305 | 0.66 | -8.36E-03 | 5.46E-03 | 4.65E-04 | 1.88E-03 | 1.26E-02 | -3.00E-03 |
| K07270 | 0.323 | 0.662 | 0.959 | 0.714 | 0.851 | 0.995 | 2.47E-03 | 5.69E-03 | 6.27E-04 | 2.67E-04 | 8.50E-03 | -4.20E-03 |
| K02822 | 0.499 | 0.578 | 1.217 | 0.495 | 1.054 | 1.378 | -3.67E-03 | -3.90E-03 | 7.76E-03 | 8.22E-04 | 1.11E-02 | 8.32E-03 |
| K06962 | 0.206 | 0.913 | 0.999 | 1.161 | 0.768 | 0.459 | 3.37E-05 | -3.25E-03 | -5.59E-03 | -3.83E-03 | 8.12E-03 | -2.81E-03 |
| K08234 | 1.421 | 0.912 | 1.025 | 0.288 | 0.664 | 0.134 | -1.08E-02 | 1.11E-03 | 1.10E-02 | -8.08E-04 | -3.42E-04 | 9.91E-04 |
| K01518 | 1.382 | 0.254 | 0.57 | 0.363 | 0.989 | 1.083 | -8.93E-03 | -1.00E-03 | -5.95E-03 | 1.36E-03 | -2.82E-03 | 9.46E-04 |
| K09706 | 0.632 | 0.394 | 0.672 | 1.114 | 1.347 | 0.948 | 1.52E-03 | 4.03E-04 | 3.68E-03 | -4.31E-03 | 1.26E-02 | -3.20E-03 |
| K16927 | 0.738 | 0.508 | 0.998 | 0.775 | 0.956 | 1.228 | -4.25E-03 | -4.24E-03 | -8.56E-03 | 1.84E-03 | 9.92E-03 | -7.82E-03 |
| K00887 | 0.661 | 0.884 | 0.814 | 0.608 | 0.233 | 1.18 | -2.84E-03 | -7.44E-03 | -3.88E-03 | -5.07E-04 | -1.74E-03 | 4.78E-03 |
| K20742 | 0.391 | 1.037 | 1.518 | 1.167 | 0.715 | 0.402 | 6.45E-04 | 8.87E-03 | -1.07E-02 | -6.06E-03 | 4.51E-03 | -8.35E-05 |
| K02651 | 0.144 | 0.273 | 0.765 | 0.802 | 2.218 | 1.06 | 9.70E-04 | -2.05E-03 | 7.97E-03 | -1.77E-03 | 2.33E-02 | -7.58E-03 |
| K01975 | 0.303 | 0.531 | 0.847 | 0.351 | 1.567 | 1.126 | 2.25E-03 | 3.38E-03 | 4.46E-03 | 7.18E-04 | 1.59E-02 | -7.99E-03 |
| K01095 | 1.79 | 1.181 | 0.787 | 0.197 | 0.619 | 0.927 | -1.39E-02 | 7.99E-03 | 6.92E-03 | -5.84E-04 | -6.19E-03 | 5.31E-03 |
| K02759 | 0.439 | 1.036 | 1.397 | 0.867 | 0.635 | 0.753 | 2.51E-03 | -6.28E-03 | 6.92E-03 | 1.64E-03 | 5.89E-03 | 4.76E-03 |

| | | | | | | | | | | | | |
|---|---|---|---|---|---|---|---|---|---|---|---|---|
| K22210 | 1.02 | 0.729 | 0.86 | 0.465 | 0.577 | 1.101 | -5.80E-03 | -5.38E-03 | 3.22E-03 | -1.55E-03 | 6.03E-03 | 4.04E-03 |
| K05937 | 0.262 | 0.826 | 0.53 | 0.405 | 1.225 | 1.354 | 2.00E-03 | 4.88E-04 | 5.79E-03 | 1.68E-03 | -7.89E-03 | 6.19E-03 |
| K01646 | 1.307 | 0.414 | 0.483 | 0.604 | 1.453 | 1.599 | 8.29E-03 | -1.81E-03 | -5.16E-03 | -3.13E-03 | -1.40E-02 | -1.18E-02 |
| K22300 | 0.148 | 1.871 | 1.468 | 0.401 | 1.546 | 0.725 | -7.96E-04 | -1.52E-02 | 1.61E-02 | -6.18E-04 | -3.43E-03 | -1.35E-03 |
| K08972 | 0.402 | 1.343 | 0.676 | 0.426 | 0.988 | 1.227 | -3.09E-04 | -1.07E-02 | 4.44E-03 | 1.61E-03 | 1.03E-02 | -7.30E-03 |
| K09124 | 0.529 | 1.027 | 0.976 | 0.484 | 0.384 | 1.742 | -3.20E-03 | -8.19E-03 | -7.31E-03 | -2.52E-03 | 4.05E-03 | -1.21E-02 |
| K07148 | 1.482 | 0.267 | 0.985 | 0.882 | 0.756 | 0.703 | -1.15E-02 | -2.02E-03 | -2.89E-03 | 2.46E-03 | 7.17E-03 | 4.31E-03 |
| K01201 | 1.079 | 0.982 | 0.385 | 0.922 | 0.447 | 0.721 | -8.35E-03 | -6.74E-03 | 1.00E-03 | 4.85E-03 | -4.39E-03 | 5.28E-03 |
| K09779 | 0.696 | 0.731 | 0.794 | 0.808 | 1.243 | 1.314 | 2.97E-03 | 2.31E-03 | -7.40E-03 | -3.70E-03 | -7.27E-03 | -5.54E-03 |
| K07045 | 0.415 | 0.796 | 0.968 | 0.276 | 1.051 | 1.391 | -3.13E-03 | -6.80E-03 | -3.59E-03 | -1.40E-03 | 9.26E-03 | -1.02E-02 |
| K14415 | 0.328 | 1.714 | 0.748 | 0.495 | 1.475 | 1.772 | -1.36E-03 | -1.46E-02 | -5.17E-04 | -2.58E-03 | 1.54E-02 | -1.31E-02 |
| K05799 | 1.776 | 0.578 | 0.336 | 0.314 | 1.282 | 0.825 | -1.35E-02 | -3.60E-03 | -2.14E-04 | -1.69E-04 | 1.18E-02 | -2.82E-05 |
| K09803 | 0.539 | 1.981 | 0.367 | 0.936 | 0.202 | 1.647 | 4.16E-03 | -1.55E-02 | -3.88E-03 | -3.35E-03 | 3.71E-04 | -1.21E-02 |
| K02492 | 0.429 | 0.483 | 0.971 | 0.87 | 0.447 | 1.26 | -2.93E-03 | -5.93E-04 | 5.77E-03 | 3.16E-03 | -4.59E-03 | -9.29E-03 |
| K10532 | 0.583 | 0.614 | 1.176 | 1.082 | 0.486 | 1.443 | 2.13E-03 | -7.87E-04 | 1.26E-02 | 5.68E-03 | 6.46E-04 | 7.01E-03 |
| K19304 | 1.07 | 1.429 | 0.726 | 1.495 | 0.326 | 0.15 | -7.15E-03 | 1.12E-02 | -4.75E-03 | 7.69E-03 | -3.19E-03 | -1.11E-03 |
| K03741 | 0.637 | 0.331 | 1.223 | 1.129 | 0.546 | 0.887 | -4.77E-03 | -9.89E-06 | 3.00E-03 | 5.82E-03 | -1.41E-03 | 5.56E-03 |
| K10117 | 0.902 | 0.79 | 0.993 | 0.648 | 0.45 | 0.866 | 2.34E-03 | -4.49E-03 | -4.45E-03 | 1.06E-04 | 3.89E-03 | 4.08E-03 |
| K14188 | 1.148 | 0.74 | 0.646 | 0.351 | 1.244 | 0.894 | 8.68E-03 | -6.04E-03 | -5.51E-03 | -2.43E-04 | -3.25E-03 | -2.03E-04 |
| K03820 | 0.58 | 0.76 | 0.881 | 1.263 | 0.849 | 0.088 | -3.72E-03 | -6.37E-03 | -8.69E-03 | 6.16E-03 | -7.79E-03 | -2.79E-04 |
| K08678 | 0.756 | 0.945 | 0.706 | 1.451 | 0.724 | 1.573 | 5.84E-03 | 8.13E-03 | 1.76E-03 | 7.15E-03 | -7.21E-03 | 1.15E-02 |
| K06972 | 0.646 | 0.655 | 1.118 | 0.968 | 0.242 | 1.146 | 1.92E-03 | -3.68E-03 | -1.95E-03 | -1.75E-03 | 2.54E-03 | 5.13E-03 |
| K05337 | 1.162 | 0.956 | 0.656 | 0.754 | 0.567 | 0.985 | -8.51E-03 | 5.22E-03 | 3.77E-03 | -1.42E-03 | 3.57E-03 | -6.16E-03 |
| K02435 | 0.558 | 0.72 | 0.479 | 0.889 | 1.314 | 1.295 | -2.22E-03 | 4.08E-03 | 2.30E-03 | -2.27E-03 | -4.61E-03 | -2.06E-03 |
| K07089 | 0.909 | 0.064 | 0.52 | 1.143 | 1.073 | 0.044 | -7.01E-03 | -5.25E-04 | -1.88E-03 | 5.26E-03 | 1.14E-02 | -3.15E-05 |
| K03742 | 1.238 | 0.495 | 0.454 | 1.143 | 0.8 | 0.114 | 9.47E-03 | 2.74E-03 | -4.59E-03 | 4.80E-03 | 1.99E-04 | 3.78E-04 |
| K01155 | 1.266 | 1.32 | 0.861 | 0.111 | 0.723 | 0.362 | 9.40E-03 | -6.81E-03 | 9.44E-03 | 4.70E-04 | 4.03E-03 | 1.85E-03 |
| K11941 | 1.03 | 0.154 | 1.053 | 0.51 | 0.204 | 1.071 | 6.11E-03 | -9.43E-04 | -9.30E-03 | 6.52E-04 | -1.06E-03 | -7.31E-03 |
| K20866 | 0.479 | 0.16 | 0.945 | 1.068 | 1.485 | 0.668 | 6.07E-04 | 1.08E-03 | -6.65E-03 | -5.51E-03 | -1.56E-02 | -3.33E-03 |
| K00974 | 0.297 | 0.519 | 1.036 | 0.881 | 0.307 | 0.963 | -1.60E-03 | 3.63E-03 | -7.42E-03 | 2.59E-03 | 1.82E-03 | 4.44E-03 |
| K19225 | 0.417 | 0.776 | 1.667 | 0.692 | 1.23 | 1.372 | 1.17E-03 | 2.42E-03 | 1.54E-02 | -3.64E-03 | -1.04E-02 | 7.56E-03 |
| K07461 | 0.643 | 1.228 | 0.511 | 0.678 | 1.238 | 1.536 | 3.36E-03 | 9.80E-03 | -5.02E-03 | 6.53E-04 | 4.71E-03 | -1.11E-02 |
| K08309 | 0.663 | 0.594 | 1.03 | 0.907 | 1.092 | 0.687 | 4.39E-03 | 4.96E-03 | -6.19E-03 | 1.30E-04 | -5.94E-03 | -1.40E-03 |
| K02217 | 0.151 | 0.347 | 0.961 | 0.849 | 0.289 | 1.09 | -6.87E-04 | 5.73E-04 | 5.84E-04 | -3.26E-03 | -1.58E-03 | -6.00E-03 |

| | | | | | | | | | | | | |
|---|---|---|---|---|---|---|---|---|---|---|---|---|
| K01858 | 1.088 | 0.671 | 0.825 | 0.63 | 0.69 | 1.495 | -1.74E-04 | 5.58E-03 | 8.74E-03 | -9.93E-04 | 5.33E-03 | -1.08E-02 |
| K01744 | 0.994 | 0.613 | 0.326 | 0.531 | 0.836 | 0.862 | -7.68E-03 | -4.58E-03 | -1.73E-03 | -1.45E-03 | -7.56E-03 | -6.24E-03 |
| K21903 | 0.571 | 0.631 | 1.12 | 1.276 | 0.542 | 0.871 | -3.38E-03 | 5.39E-03 | 1.76E-03 | 5.56E-03 | 5.19E-03 | -9.53E-04 |
| K18928 | 0.943 | 0.656 | 0.799 | 1.793 | 0.62 | 1.077 | -5.47E-03 | -1.96E-03 | 2.18E-03 | 7.40E-03 | 1.02E-03 | 4.72E-03 |
| K02614 | 0.581 | 0.78 | 0.963 | 0.942 | 0.851 | 0.317 | -4.07E-03 | -6.61E-03 | 1.26E-03 | 1.45E-03 | 8.75E-03 | 2.16E-03 |
| K07588 | 0.463 | 0.473 | 1.027 | 1.145 | 1.235 | 0.728 | -3.17E-03 | -1.55E-03 | 5.69E-03 | 5.02E-03 | 1.02E-02 | 3.49E-03 |
| K06990 | 0.687 | 0.427 | 1.103 | 0.94 | 0.95 | 0.713 | -5.27E-03 | -2.85E-03 | 3.93E-03 | 3.00E-03 | 7.74E-03 | -4.64E-03 |
| K06020 | 0.434 | 1.108 | 0.229 | 1.988 | 0.567 | 1.044 | -3.16E-03 | -9.20E-03 | 1.61E-03 | 1.05E-02 | -1.79E-03 | 6.94E-03 |
| K14136 | 0.666 | 1.003 | 0.548 | 1.106 | 0.483 | 1.218 | -3.72E-03 | -8.91E-04 | -6.60E-04 | 5.75E-03 | 1.86E-03 | 7.89E-03 |
| K05832 | 0.748 | 0.397 | 0.967 | 0.98 | 0.628 | 0.958 | -1.69E-03 | 3.31E-03 | -1.42E-03 | 5.12E-05 | 4.51E-03 | -2.36E-03 |
| K00058 | 0.463 | 0.184 | 1.12 | 0.861 | 0.246 | 0.93 | 2.36E-03 | -1.54E-04 | -5.77E-04 | 1.89E-03 | 2.00E-03 | 3.24E-03 |
| K01626 | 0.53 | 0.292 | 1.103 | 0.887 | 0.493 | 1.37 | 2.44E-03 | 2.42E-03 | -3.81E-03 | 1.08E-03 | 4.97E-03 | 9.12E-03 |
| K07105 | 0.584 | 0.485 | 1.248 | 0.685 | 1.592 | 1.261 | -3.71E-03 | -3.98E-03 | 3.90E-03 | 1.68E-03 | -1.39E-02 | 6.28E-03 |
| K03091 | 0.416 | 0.344 | 0.729 | 1.299 | 1.394 | 1.228 | -1.97E-04 | 4.71E-05 | -7.96E-03 | -4.99E-03 | -6.54E-03 | -3.68E-03 |
| K00179 | 0.461 | 0.762 | 0.973 | 0.996 | 1.604 | 0.385 | -3.47E-03 | -6.49E-03 | -3.07E-03 | 4.47E-03 | 1.70E-02 | 1.84E-03 |
| K02574 | 1.298 | 0.554 | 0.863 | 1.301 | 0.416 | 0.328 | -1.00E-02 | -3.49E-03 | 1.75E-03 | 6.47E-03 | 4.12E-03 | 2.42E-03 |
| K02025 | 0.869 | 0.519 | 1.246 | 0.795 | 0.829 | 0.757 | 3.78E-04 | 2.83E-03 | 6.31E-03 | 1.56E-04 | 8.74E-03 | -1.81E-03 |
| K03621 | 0.757 | 0.248 | 1.085 | 1.001 | 0.696 | 1.066 | -4.08E-03 | -1.66E-03 | -8.11E-03 | 1.12E-03 | 4.08E-03 | 2.18E-03 |
| K03704 | 0.718 | 0.039 | 1.088 | 0.878 | 0.524 | 1.372 | 5.51E-03 | 9.60E-05 | 1.39E-04 | 3.03E-03 | 5.04E-03 | 8.07E-03 |
| K00703 | 0.861 | 0.73 | 0.786 | 0.847 | 0.672 | 1.153 | -2.42E-03 | 5.50E-03 | 1.54E-03 | 1.50E-07 | -1.83E-04 | -4.79E-03 |
| K00798 | 0.5 | 0.321 | 1.244 | 1.079 | 1.007 | 0.674 | -2.05E-03 | -1.60E-03 | -1.93E-03 | 3.69E-03 | -9.18E-03 | -4.91E-04 |
| K04083 | 0.396 | 0.437 | 1.185 | 0.916 | 0.49 | 0.848 | 1.95E-03 | 3.76E-03 | 9.61E-05 | 1.47E-03 | 2.96E-03 | 5.98E-04 |
| K03431 | 0.556 | 0.244 | 1.13 | 1 | 0.492 | 0.843 | -2.27E-03 | -2.07E-03 | -2.01E-03 | 4.31E-04 | 4.93E-03 | -1.18E-03 |
| K04751 | 1.309 | 0.701 | 1.612 | 0.437 | 0.344 | 1.089 | -6.87E-03 | 5.77E-03 | 1.34E-02 | 1.07E-03 | -2.59E-03 | 6.39E-03 |
| K01817 | 0.804 | 1.329 | 0.592 | 0.853 | 0.609 | 0.289 | 5.96E-03 | 1.59E-03 | -6.02E-03 | 3.75E-03 | -4.17E-03 | -2.06E-03 |
| K03500 | 0.904 | 0.55 | 1.076 | 0.868 | 0.393 | 0.527 | 6.63E-03 | 4.46E-03 | -2.10E-03 | 1.89E-03 | 3.34E-03 | 1.67E-03 |
| K00180 | 0.557 | 0.578 | 1.056 | 0.802 | 1.085 | 0.149 | -4.21E-03 | -4.82E-03 | 6.83E-04 | 2.99E-03 | 1.12E-02 | -5.23E-04 |
| K09922 | 1.08 | 0.315 | 1.015 | 1.756 | 0.49 | 0.667 | 1.46E-03 | -1.66E-03 | 3.58E-03 | 7.10E-03 | -8.89E-04 | 3.47E-03 |
| K03778 | 0.466 | 0.561 | 1.755 | 0.691 | 1.13 | 1.955 | 3.58E-03 | 4.22E-03 | 1.11E-02 | 1.30E-03 | 8.81E-03 | 1.37E-02 |
| K03060 | 0.75 | 1.222 | 0.624 | 0.927 | 0.761 | 1.195 | 5.03E-03 | 9.26E-03 | 3.15E-03 | 1.68E-03 | 3.40E-03 | -9.76E-04 |
| K03784 | 1.411 | 0.546 | 0.884 | 0.604 | 0.491 | 0.982 | -1.06E-02 | 4.19E-03 | 7.31E-03 | -2.35E-03 | -1.85E-03 | -4.75E-03 |
| K23351 | 1.458 | 0.446 | 0.979 | 0.134 | 0.725 | 0.816 | -1.09E-02 | -3.05E-03 | 8.09E-03 | -6.79E-04 | 1.78E-06 | -1.10E-03 |
| K01775 | 0.599 | 0.302 | 1.021 | 0.993 | 0.658 | 1.094 | -4.69E-04 | -2.49E-03 | -3.41E-03 | 2.10E-04 | 2.67E-03 | -3.28E-03 |
| K03614 | 0.518 | 0.667 | 0.989 | 0.109 | 1.039 | 1.245 | -3.26E-03 | 3.11E-03 | -3.74E-03 | 2.72E-04 | 1.08E-02 | -9.19E-03 |

| | | | | | | | | | | | | |
|---|---|---|---|---|---|---|---|---|---|---|---|---|
| K09765 | 0.826 | 0.262 | 0.552 | 0.47 | 1.055 | 1.162 | -4.54E-03 | 1.22E-03 | 2.25E-03 | 1.78E-03 | 2.58E-03 | 1.73E-03 |
| K21498 | 0.9 | 1.367 | 1.094 | 0.475 | 0.512 | 0.789 | 3.79E-04 | 1.17E-02 | 1.07E-02 | 2.25E-03 | -5.41E-03 | 5.61E-03 |
| K02199 | 0.884 | 0.664 | 1.34 | 1.36 | 0.567 | 0.7 | 3.34E-03 | 1.12E-03 | 1.44E-02 | 6.32E-03 | 5.99E-03 | 5.15E-03 |
| K01719 | 0.95 | 0.691 | 1.016 | 1.085 | 0.626 | 0.678 | -3.33E-03 | 1.26E-03 | 2.32E-03 | 3.00E-03 | -3.45E-03 | 2.83E-03 |
| K03521 | 0.976 | 1.869 | 1.088 | 0.707 | 0.763 | 0.333 | -7.03E-03 | -1.36E-02 | -8.86E-03 | -2.20E-03 | 8.04E-04 | -1.19E-04 |
| K01610 | 0.543 | 1.192 | 1.188 | 0.571 | 1.102 | 0.134 | 3.87E-03 | 3.80E-03 | -1.00E-02 | -8.18E-04 | 6.91E-03 | -5.17E-04 |
| K20509 | 1.203 | 1.014 | 0.683 | 0.927 | 0.445 | 0.445 | -6.15E-03 | -7.46E-03 | -3.39E-03 | 4.87E-03 | -1.10E-03 | 2.77E-03 |
| K01206 | 0.097 | 0.788 | 0.169 | 1.143 | 1.367 | 1.566 | 1.12E-04 | 1.85E-04 | 1.71E-03 | 5.97E-03 | -1.45E-02 | -1.16E-02 |
| K01952 | 0.271 | 1.164 | 1.065 | 0.636 | 0.892 | 0.312 | 1.04E-03 | -3.22E-03 | -4.44E-05 | 2.10E-03 | 8.89E-04 | 2.23E-03 |
| K01515 | 0.571 | 0.79 | 0.908 | 1.071 | 0.761 | 1.14 | -2.81E-03 | 6.72E-03 | -1.67E-03 | -2.62E-03 | -3.83E-03 | -1.58E-03 |
| K03581 | 0.285 | 0.502 | 1.129 | 0.955 | 0.235 | 0.863 | -6.36E-04 | 3.68E-03 | -4.56E-04 | 8.65E-04 | 1.87E-03 | -1.35E-03 |
| K07322 | 1.191 | 1.185 | 0.569 | 0.686 | 1.658 | 0.521 | 3.66E-03 | 4.35E-04 | -5.87E-03 | 9.07E-05 | -1.70E-02 | 1.26E-03 |
| K00053 | 1.155 | 1.246 | 1.009 | 0.693 | 0.6 | 0.648 | -8.86E-03 | 1.10E-04 | -4.00E-03 | 2.99E-03 | -6.27E-03 | 4.78E-03 |
| K03517 | 1.776 | 1.446 | 0.681 | 0.447 | 0.296 | 0.905 | 1.17E-02 | -6.02E-03 | -6.15E-03 | -3.42E-04 | -1.93E-03 | 6.25E-03 |
| K13444 | 1.009 | 0.669 | 1.053 | 0.947 | 0.759 | 0.557 | -2.15E-03 | 5.20E-03 | 4.81E-03 | 2.18E-03 | -6.29E-03 | -2.68E-03 |
| K18843 | 0.349 | 0.237 | 1.205 | 0.948 | 0.86 | 0.4 | 2.64E-03 | -1.26E-03 | 2.67E-03 | 2.12E-03 | -9.06E-03 | -6.51E-04 |
| K00962 | 2.131 | 0.7 | 1.236 | 0.718 | 1.088 | 0.589 | 1.65E-02 | -1.61E-03 | -1.13E-02 | 6.70E-04 | 6.80E-03 | -3.76E-03 |
| K07816 | 1.187 | 1.453 | 0.688 | 0.452 | 0.896 | 0.583 | -6.03E-03 | -1.25E-02 | -3.59E-03 | 2.25E-03 | -1.21E-03 | 3.28E-03 |
| K19824 | 1.182 | 1.127 | 0.729 | 1.082 | 0.11 | 0.718 | -9.13E-03 | 6.98E-03 | 5.75E-03 | 5.62E-03 | 8.28E-04 | 2.25E-03 |
| K03043 | 0.452 | 1.244 | 1.1 | 0.788 | 1.039 | 0.197 | 3.40E-03 | -8.12E-04 | -5.45E-03 | -1.10E-03 | 7.54E-04 | -6.75E-04 |
| K03046 | 1.036 | 1.038 | 1.111 | 0.695 | 0.775 | 0.42 | 8.00E-03 | -2.39E-03 | -8.03E-03 | 9.53E-04 | 1.03E-03 | 2.73E-03 |
| K04079 | 0.348 | 1.109 | 0.891 | 1.057 | 0.433 | 0.509 | 8.91E-05 | -4.97E-03 | -9.74E-03 | -5.44E-03 | -2.04E-03 | 1.72E-03 |
| K03790 | 1.17 | 0.624 | 0.899 | 0.234 | 1.222 | 0.515 | -8.91E-03 | -5.26E-03 | 2.36E-03 | 8.91E-04 | 9.68E-03 | -1.87E-04 |
| K22719 | 1.283 | 1.341 | 0.33 | 1.228 | 0.754 | 0.623 | 4.77E-03 | -2.74E-03 | -3.24E-03 | 6.37E-03 | -3.62E-03 | 4.58E-03 |
| K00765 | 1.271 | 1.302 | 0.348 | 0.773 | 1.206 | 0.49 | 6.79E-03 | 5.83E-03 | 3.80E-03 | 3.31E-03 | 1.26E-02 | -7.60E-04 |
| K03615 | 0.69 | 1.047 | 0.403 | 0.787 | 0.88 | 1.474 | -6.63E-04 | -2.02E-03 | 7.51E-04 | 2.66E-03 | 9.24E-03 | -1.07E-02 |
| K01495 | 1.056 | 0.781 | 1.044 | 1.541 | 0.773 | 0.455 | -4.56E-03 | 6.10E-03 | 4.84E-03 | 6.91E-03 | -8.14E-03 | 1.26E-03 |
| K00931 | 1.087 | 0.746 | 0.715 | 0.933 | 0.728 | 0.882 | 7.97E-03 | -6.72E-04 | 4.12E-03 | 4.75E-03 | 7.28E-03 | 6.50E-03 |
| K01890 | 0.961 | 1.483 | 1.184 | 0.626 | 0.636 | 0.584 | 7.37E-03 | -6.01E-03 | -1.03E-02 | 9.76E-04 | -3.44E-03 | 6.58E-04 |
| K00817 | 0.477 | 0.955 | 0.722 | 0.838 | 0.855 | 0.067 | 3.52E-03 | -2.89E-03 | -2.99E-04 | 3.65E-03 | -8.85E-03 | 4.89E-04 |
| K03654 | 0.619 | 1.296 | 0.573 | 0.653 | 0.877 | 1.439 | -2.89E-03 | -4.35E-03 | -2.40E-03 | 1.42E-04 | -5.17E-03 | 9.75E-03 |
| K11717 | 0.391 | 1.266 | 0.38 | 0.361 | 1.213 | 0.887 | -5.20E-04 | -3.46E-03 | -1.06E-03 | -1.42E-03 | 5.57E-03 | 5.14E-03 |
| K04077 | 0.409 | 1.467 | 1.032 | 0.652 | 0.908 | 0.405 | 2.90E-04 | -1.97E-03 | -6.07E-03 | 4.53E-04 | -2.88E-03 | 2.41E-03 |
| K01933 | 0.217 | 1.39 | 1.396 | 0.991 | 0.521 | 0.035 | -1.12E-03 | -5.40E-03 | 8.08E-03 | 4.66E-03 | 2.56E-03 | -2.58E-04 |

| | | | | | | | | | | | | |
|---|---|---|---|---|---|---|---|---|---|---|---|---|
| K04095 | 0.467 | 1.349 | 0.38 | 1.164 | 0.148 | 1.019 | -2.48E-03 | 8.03E-03 | -3.98E-03 | 5.85E-03 | -1.25E-03 | 7.52E-03 |
| K02040 | 0.287 | 1.495 | 0.979 | 0.464 | 0.842 | 0.791 | 9.64E-04 | -8.87E-03 | -1.54E-03 | 2.45E-03 | -3.56E-04 | 5.11E-03 |
| K02112 | 0.594 | 1.134 | 1.091 | 0.558 | 0.571 | 0.893 | -3.60E-03 | -3.47E-03 | -5.83E-03 | -1.15E-03 | 2.01E-03 | 5.80E-03 |
| K01929 | 0.758 | 1.282 | 0.33 | 1.028 | 0.786 | 0.853 | -4.98E-03 | -1.57E-03 | -8.75E-04 | 4.96E-03 | -4.59E-04 | 3.45E-03 |
| K03572 | 0.422 | 1.291 | 0.778 | 0.925 | 0.416 | 0.898 | 2.76E-03 | 2.32E-03 | -8.01E-03 | -4.51E-03 | -3.65E-03 | 4.94E-03 |
| K03977 | 0.39 | 1.08 | 0.828 | 0.671 | 0.229 | 0.931 | 1.10E-03 | 4.51E-03 | -4.14E-03 | 1.26E-03 | -2.01E-03 | -5.18E-03 |
| K01889 | 0.417 | 0.98 | 1.481 | 0.674 | 0.512 | 1.019 | -2.40E-03 | 3.83E-03 | -1.61E-02 | 1.94E-04 | -2.53E-03 | -1.27E-03 |
| K02967 | 0.526 | 1.088 | 1.531 | 0.609 | 1.327 | 0.736 | -1.33E-03 | -4.00E-04 | -1.62E-02 | 7.60E-04 | 1.04E-02 | 2.08E-03 |
| K06942 | 0.63 | 1.076 | 1.281 | 0.312 | 1.421 | 0.502 | -8.12E-04 | 2.46E-03 | -1.29E-02 | 3.24E-04 | 1.40E-02 | 2.34E-03 |
| K01810 | 0.682 | 1.179 | 1.237 | 0.656 | 1.444 | 0.678 | 4.52E-03 | -5.50E-04 | 4.06E-03 | 1.44E-03 | 1.08E-02 | -4.69E-03 |
| K01662 | 0.771 | 1.322 | 1.008 | 0.29 | 0.705 | 0.859 | 4.93E-03 | -2.78E-03 | -2.98E-03 | -1.20E-04 | 2.19E-04 | -6.29E-03 |
| K02528 | 1.066 | 2.322 | 0.411 | 0.787 | 0.437 | 0.808 | 8.21E-03 | 1.66E-02 | -3.90E-03 | 3.78E-03 | 3.23E-03 | 6.88E-04 |
| K01258 | 0.83 | 1.045 | 1.065 | 0.779 | 0.721 | 0.254 | 6.39E-03 | -3.24E-03 | -1.20E-04 | 3.61E-03 | -4.21E-04 | 1.07E-03 |
| K03470 | 0.655 | 1.153 | 1.551 | 0.467 | 0.671 | 1.146 | -1.47E-03 | 7.58E-03 | 1.68E-02 | 2.22E-03 | 7.06E-03 | 3.99E-03 |
| K02916 | 0.971 | 1.206 | 0.603 | 0.549 | 0.965 | 0.776 | -7.49E-03 | 9.77E-03 | 1.50E-03 | 2.50E-03 | 9.86E-03 | -1.31E-03 |
| K03437 | 0.352 | 0.915 | 1.007 | 1.214 | 0.578 | 0.471 | 1.84E-03 | -2.43E-03 | -4.79E-04 | 5.74E-03 | 5.02E-03 | -3.47E-03 |
| K01738 | 0.636 | 1.072 | 1.088 | 0.757 | 0.71 | 1.018 | -4.88E-03 | 8.62E-03 | 7.33E-03 | 2.69E-03 | 6.40E-03 | 4.89E-03 |
| K03502 | 1.074 | 1.038 | 0.976 | 0.728 | 0.385 | 0.34 | 8.04E-03 | 2.74E-03 | 2.50E-04 | 1.35E-03 | 8.80E-04 | 2.45E-03 |
| K00266 | 0.747 | 1.269 | 1.159 | 0.927 | 0.295 | 0.794 | 5.77E-03 | -5.37E-03 | -7.76E-03 | -3.19E-03 | -1.53E-03 | 4.01E-03 |
| K08316 | 0.733 | 1.135 | 0.151 | 0.886 | 0.08 | 1.218 | -4.33E-03 | 8.42E-03 | 7.74E-04 | 4.60E-03 | -4.04E-04 | 6.43E-03 |
| K02965 | 1.053 | 0.929 | 0.983 | 0.484 | 0.666 | 0.789 | -7.51E-03 | 7.70E-03 | 2.66E-03 | -2.34E-03 | -1.45E-03 | -1.72E-03 |
| K00764 | 0.895 | 1.369 | 0.228 | 1.69 | 0.777 | 0.272 | -4.33E-03 | -4.23E-03 | 2.39E-03 | 8.91E-03 | 7.84E-03 | -2.00E-03 |
| K22278 | 0.6 | 0.919 | 0.9 | 0.278 | 0.16 | 1.518 | 2.85E-03 | 4.00E-03 | 2.09E-03 | 6.32E-04 | 4.48E-04 | -1.11E-02 |
| K07727 | 0.844 | 0.614 | 0.645 | 0.554 | 1.714 | 1.105 | -3.06E-03 | -4.89E-03 | 6.60E-03 | 1.13E-03 | -8.49E-03 | -4.18E-03 |
| K03531 | 0.34 | 1.107 | 0.229 | 0.995 | 0.767 | 1.012 | 1.74E-03 | -1.46E-03 | -2.50E-03 | 5.10E-03 | -2.03E-03 | 7.23E-03 |
| K07481 | 0.742 | 0.208 | 0.901 | 0.664 | 1.356 | 0.961 | 4.16E-03 | 1.42E-03 | -9.08E-03 | 6.33E-04 | -1.01E-02 | 3.03E-04 |
| K19092 | 0.597 | 0.335 | 0.904 | 1.102 | 0.493 | 0.822 | 4.56E-03 | 1.96E-03 | 5.81E-03 | 4.65E-03 | 1.48E-04 | 1.09E-03 |
| K00432 | 0.926 | 0.606 | 1.111 | 1.16 | 0.694 | 0.409 | -3.21E-03 | 3.96E-03 | -5.62E-05 | 3.90E-03 | 7.24E-03 | 3.99E-04 |
| K00059 | 0.651 | 0.806 | 1.215 | 0.889 | 0.116 | 0.735 | 8.18E-04 | 3.10E-03 | -6.52E-03 | 3.47E-03 | 1.08E-03 | -4.17E-03 |
| K07483 | 0.473 | 0.988 | 0.602 | 0.626 | 0.961 | 1.034 | 2.98E-03 | -7.68E-03 | 2.98E-03 | -2.31E-03 | 4.39E-03 | 4.26E-03 |
| K03310 | 0.446 | 1.118 | 1.193 | 0.194 | 0.187 | 0.836 | -3.36E-03 | -3.98E-03 | 5.58E-03 | 1.27E-04 | -1.84E-03 | 5.97E-03 |
| K07098 | 0.869 | 1.108 | 0.463 | 1.092 | 0.593 | 0.071 | 6.36E-04 | 5.86E-05 | 4.96E-03 | 5.17E-03 | 3.26E-03 | 3.03E-04 |
| K03308 | 1.302 | 1.417 | 0.573 | 0.645 | 0.634 | 0.885 | 8.91E-03 | -3.55E-03 | -2.61E-04 | 3.32E-03 | -4.97E-03 | -5.10E-03 |
| K01091 | 0.436 | 0.881 | 0.842 | 1.071 | 0.172 | 0.424 | 3.12E-03 | 6.78E-04 | -2.99E-03 | 4.91E-03 | -1.19E-03 | -2.13E-05 |

| | | | | | | | | | | | | |
|---|---|---|---|---|---|---|---|---|---|---|---|---|
| K03574 | 1.485 | 0.719 | 0.951 | 2.199 | 0.666 | 0.62 | 9.05E-03 | 3.07E-03 | 2.36E-03 | 1.14E-02 | 6.22E-03 | 2.97E-03 |
| K03427 | 1.897 | 1.641 | 0.85 | 0.722 | 0.777 | 0.655 | 1.40E-02 | -1.06E-02 | -6.40E-03 | 2.99E-03 | -8.17E-03 | 4.80E-03 |
| K07273 | 1.19 | 0.946 | 1.351 | 0.462 | 0.61 | 0.699 | -8.91E-03 | -7.46E-03 | 1.47E-02 | 3.89E-04 | -1.68E-03 | 1.02E-03 |
| K03327 | 0.891 | 1.228 | 1.084 | 0.727 | 0.14 | 0.758 | 5.39E-03 | -3.14E-03 | 1.82E-03 | 8.51E-04 | -1.35E-03 | -3.15E-03 |
| K07126 | 1.101 | 0.553 | 0.717 | 1.165 | 0.753 | 0.893 | 7.89E-03 | 2.05E-03 | -1.66E-04 | 6.01E-03 | -2.60E-03 | 6.42E-03 |
| K03049 | 0.652 | 0.611 | 0.841 | 0.498 | 0.704 | 0.964 | 4.78E-03 | 3.69E-03 | 4.86E-03 | -2.58E-03 | -4.74E-03 | -7.04E-03 |
| K14092 | 0.922 | 0.009 | 0.835 | 0.684 | 0.518 | 0.745 | 6.93E-03 | -1.86E-05 | 1.22E-03 | 2.43E-03 | 3.62E-03 | -5.42E-03 |
| K14096 | 0.48 | 0.569 | 0.93 | 0.153 | 0.807 | 0.706 | 3.71E-03 | 4.85E-03 | 1.68E-03 | -7.79E-04 | 6.51E-03 | -4.93E-03 |
| K03053 | 0.483 | 0.423 | 0.858 | 0.354 | 0.639 | 1.108 | 3.27E-03 | 2.89E-03 | 2.55E-03 | -1.73E-03 | 3.63E-03 | -8.09E-03 |
| K07569 | 0.418 | 0.029 | 0.941 | 0.097 | 0.637 | 0.856 | 3.15E-03 | 1.28E-04 | -4.23E-03 | -3.56E-04 | 6.29E-03 | -6.31E-03 |
| K02626 | 0.241 | 0.163 | 0.827 | 0.161 | 1.199 | 0.266 | 6.28E-04 | 1.37E-03 | 5.38E-03 | 7.55E-04 | 1.25E-02 | -7.39E-04 |
| K23264 | 0.778 | 0.146 | 0.843 | 0.364 | 0.352 | 1.268 | -5.68E-03 | 1.11E-03 | 3.83E-03 | -1.88E-03 | 3.29E-03 | -9.10E-03 |
| K14117 | 0.524 | 0.261 | 0.841 | 0.238 | 0.592 | 1.096 | -3.89E-03 | 2.07E-03 | 5.28E-03 | -1.25E-03 | 5.68E-03 | -8.04E-03 |
| K14119 | 0.566 | 0.127 | 0.853 | 0.286 | 0.657 | 0.862 | 3.94E-03 | 1.16E-04 | 3.22E-03 | 1.30E-03 | 4.90E-03 | -6.29E-03 |
| K00578 | 0.732 | 0.256 | 0.911 | 0.179 | 0.846 | 0.769 | 5.61E-03 | 2.20E-03 | 4.18E-03 | 8.09E-04 | 7.83E-03 | -5.53E-03 |
| K17884 | 0.276 | 0.332 | 0.748 | 0.877 | 0.183 | 1.179 | 9.17E-04 | -2.52E-03 | -7.65E-03 | -4.47E-03 | 4.21E-05 | -8.62E-03 |
| K15024 | 1.305 | 0.323 | 0.446 | 0.577 | 0.452 | 1.149 | 9.99E-03 | -1.05E-03 | 1.69E-05 | 2.64E-03 | -1.85E-03 | 3.06E-03 |
| K02009 | 0.13 | 0.191 | 0.932 | 0.184 | 0.446 | 1.31 | 9.81E-04 | -1.02E-03 | 2.77E-04 | -9.57E-04 | 2.12E-03 | -9.67E-03 |
| K03057 | 0.349 | 0.019 | 0.934 | 0.298 | 0.582 | 1.099 | 1.25E-03 | -1.59E-04 | 9.22E-03 | 1.41E-03 | 3.69E-03 | -8.11E-03 |
| K13599 | 0.291 | 0.812 | 1.591 | 0.348 | 0.492 | 0.319 | 8.53E-04 | 5.36E-03 | -8.84E-03 | 1.80E-03 | 3.79E-03 | 2.35E-03 |
| K02173 | 1.086 | 0.72 | 0.726 | 0.728 | 0.484 | 1.279 | 5.93E-03 | 6.06E-03 | 7.96E-03 | -8.78E-04 | 3.10E-03 | 6.52E-03 |
| K21993 | 0.315 | 0.95 | 0.852 | 0.187 | 0.324 | 0.727 | -1.26E-03 | -8.05E-03 | 8.14E-03 | -9.38E-04 | -2.99E-03 | -1.44E-03 |
| K13653 | 0.327 | 0.642 | 0.886 | 0.236 | 1.194 | 0.762 | 4.48E-04 | -4.15E-03 | 3.35E-03 | -6.81E-04 | 1.18E-02 | -1.64E-03 |
| K01118 | 0.511 | 1.076 | 0.713 | 0.214 | 1.368 | 0.607 | 2.89E-03 | -7.56E-03 | 4.99E-04 | 2.30E-04 | 1.04E-02 | 3.48E-03 |
| K07248 | 0.776 | 0.95 | 0.746 | 0.47 | 0.81 | 0.371 | 5.48E-03 | 5.59E-03 | -2.42E-03 | 7.52E-04 | 8.57E-03 | 1.06E-03 |
| K03791 | 0.741 | 0.366 | 1.161 | 0.59 | 0.574 | 1.592 | -5.47E-03 | -3.13E-03 | -6.82E-03 | 2.07E-03 | 5.59E-03 | -1.04E-02 |
| K11621 | 0.504 | 0.231 | 0.508 | 0.631 | 1.513 | 1.11 | -1.94E-03 | -1.91E-03 | 5.55E-03 | 1.10E-03 | 1.53E-02 | -2.51E-03 |
| K00616 | 0.715 | 0.233 | 1.038 | 0.898 | 0.473 | 0.322 | -4.40E-03 | -1.30E-03 | -8.17E-03 | -1.46E-03 | 4.97E-03 | -8.55E-04 |
| K04034 | 0.781 | 1.446 | 0.617 | 0.39 | 0.409 | 1.277 | 5.72E-03 | -1.16E-02 | 3.49E-04 | -1.43E-03 | 3.75E-03 | -8.29E-03 |
| K13684 | 0.654 | 2.104 | 0.218 | 0.589 | 2.367 | 0.53 | -4.92E-03 | -1.72E-02 | -2.03E-03 | 2.99E-03 | -2.48E-02 | 3.38E-03 |
| K04088 | 0.069 | 1.186 | 0.859 | 0.696 | 0.227 | 0.58 | -4.90E-04 | 8.12E-03 | 5.43E-03 | 3.04E-03 | 1.55E-03 | 4.28E-03 |
| K13018 | 0.799 | 0.373 | 1.114 | 0.631 | 0.775 | 1.05 | -6.08E-03 | 1.16E-03 | 6.60E-04 | 3.08E-03 | 7.82E-03 | -7.22E-03 |
| K10112 | 0.52 | 2.201 | 0.262 | 0.41 | 1.12 | 0.296 | -9.01E-05 | -1.83E-02 | 2.87E-04 | 7.00E-04 | 3.43E-03 | -3.00E-04 |
| K00275 | 1.192 | 1.218 | 0.34 | 0.789 | 0.268 | 0.541 | 8.90E-03 | 8.74E-03 | -3.15E-03 | 3.88E-03 | 1.32E-03 | 3.60E-03 |

| | | | | | | | | | | | | |
|---|---|---|---|---|---|---|---|---|---|---|---|---|
| K01255 | 0.177 | 0.649 | 0.995 | 0.976 | 0.566 | 0.607 | -1.33E-03 | 5.54E-03 | -2.47E-03 | 4.88E-03 | -5.50E-03 | -4.34E-03 |
| K02420 | 0.544 | 0.525 | 1.077 | 0.87 | 0.406 | 0.274 | 4.03E-03 | 3.45E-03 | 4.85E-03 | 2.92E-03 | -3.50E-03 | -2.03E-03 |
| K07192 | 1.439 | 1.794 | 0.702 | 0.044 | 0.329 | 0.537 | -1.07E-02 | -1.54E-02 | 1.94E-03 | 2.34E-04 | -2.78E-03 | 3.36E-03 |
| K02413 | 0.887 | 0.365 | 1.142 | 0.709 | 0.247 | 0.376 | 6.59E-03 | 2.08E-03 | 4.37E-03 | 5.87E-04 | -2.24E-03 | -2.60E-03 |
| K02398 | 0.984 | 0.658 | 1.046 | 0.792 | 0.201 | 0.049 | 7.62E-03 | 4.88E-03 | 4.36E-03 | 1.83E-03 | -1.61E-03 | -3.53E-05 |
| K02456 | 0.262 | 0.924 | 0.736 | 1.112 | 0.256 | 0.08 | 3.57E-04 | 7.92E-03 | 3.89E-03 | 5.24E-03 | -2.53E-03 | -2.13E-04 |
| K01698 | 0.299 | 0.357 | 1.502 | 1.151 | 0.558 | 0.52 | 2.93E-04 | 1.86E-04 | 1.18E-02 | 5.22E-03 | -2.84E-03 | -3.35E-03 |
| K00872 | 0.127 | 0.661 | 1.108 | 1.088 | 0.547 | 0.379 | 7.52E-05 | 5.60E-03 | -7.16E-04 | 4.01E-03 | -2.78E-04 | 2.77E-03 |
| K12976 | 1.004 | 1.04 | 0.791 | 0.532 | 0.708 | 0.666 | -2.28E-03 | 7.92E-03 | -8.27E-03 | -2.07E-03 | -7.41E-03 | 3.99E-03 |
| K00283 | 1.85 | 1.801 | 0.718 | 0.651 | 0.377 | 0.694 | 1.38E-02 | -1.24E-02 | -1.06E-03 | -7.06E-04 | -3.53E-03 | -4.96E-03 |
| K01845 | 0.27 | 0.611 | 1.089 | 0.768 | 0.497 | 1.19 | -8.07E-04 | -2.94E-03 | 7.16E-03 | 2.72E-03 | -4.89E-03 | -8.78E-03 |
| K00797 | 0.77 | 0.604 | 0.887 | 0.726 | 0.611 | 1.408 | 5.59E-03 | 4.99E-03 | -9.75E-04 | 3.52E-04 | 3.30E-04 | 8.13E-03 |
| K02039 | 0.233 | 0.716 | 1.285 | 0.733 | 0.327 | 0.956 | -1.14E-04 | -5.22E-03 | 2.46E-03 | 1.51E-03 | -1.51E-04 | 4.34E-03 |
| K04042 | 0.549 | 0.48 | 1.121 | 0.848 | 0.31 | 0.499 | 4.23E-03 | 3.96E-03 | -4.43E-03 | 2.15E-03 | -3.40E-04 | 3.29E-03 |
| K02038 | 0.27 | 0.382 | 1.172 | 0.873 | 0.441 | 0.699 | -1.53E-03 | -1.16E-03 | -1.39E-03 | 2.51E-03 | 2.25E-03 | 2.73E-03 |
| K03606 | 0.462 | 1.079 | 0.874 | 0.676 | 0.362 | 0.707 | 3.55E-03 | -6.61E-03 | 3.47E-03 | 3.37E-03 | -2.89E-03 | 1.19E-04 |
| K18828 | 1.945 | 0.668 | 0.438 | 1.987 | 0.502 | 0.387 | 1.39E-02 | 5.41E-03 | -2.24E-03 | 1.04E-02 | -4.51E-03 | -1.91E-03 |
| K21029 | 0.774 | 1.342 | 0.654 | 0.746 | 0.734 | 2.026 | -5.92E-03 | 1.14E-02 | 6.77E-03 | 2.39E-03 | 3.93E-03 | 1.49E-02 |
| K19271 | 0.294 | 0.232 | 1.128 | 0.669 | 0.866 | 0.716 | -1.11E-03 | -1.97E-03 | 9.27E-03 | 1.76E-03 | 8.94E-03 | 5.18E-03 |
| K15894 | 1.649 | 1.646 | 0.432 | 0.622 | 0.073 | 0.259 | 1.22E-02 | -1.35E-02 | 4.73E-03 | 3.11E-03 | 7.70E-04 | 1.87E-03 |
| K03442 | 0.221 | 1.529 | 0.526 | 0.872 | 0.722 | 0.732 | 5.80E-05 | 1.25E-02 | -3.16E-03 | 3.34E-03 | 4.00E-03 | -2.61E-03 |
| K00567 | 0.185 | 0.792 | 0.878 | 0.948 | 0.599 | 0.592 | 1.42E-03 | 6.51E-03 | -1.77E-03 | 1.58E-03 | -3.61E-03 | -1.36E-03 |
| K03816 | 0.367 | 0.979 | 1.197 | 0.612 | 0.371 | 0.364 | 2.81E-03 | 6.64E-03 | -1.31E-02 | -3.22E-03 | -3.19E-03 | -1.44E-03 |
| K01246 | 1.048 | 0.476 | 1.14 | 0.653 | 0.277 | 0.56 | 8.01E-03 | 3.08E-03 | 1.43E-03 | 1.22E-03 | -5.07E-04 | -3.77E-03 |
| K00937 | 0.112 | 0.683 | 1.074 | 0.816 | 0.171 | 0.573 | -8.05E-04 | 5.47E-03 | -2.60E-03 | -1.75E-04 | -7.83E-06 | 2.66E-03 |
| K07464 | 0.227 | 0.765 | 1.457 | 0.619 | 0.46 | 0.886 | -1.69E-03 | -8.56E-04 | 7.89E-03 | 7.48E-04 | -4.09E-03 | -5.15E-03 |
| K09474 | 0.661 | 0.469 | 1.248 | 1.115 | 0.637 | 0.401 | -2.25E-03 | -3.85E-03 | -3.24E-03 | 3.59E-03 | -6.23E-03 | 2.12E-03 |
| K01009 | 0.77 | 0.548 | 1.177 | 1.358 | 0.422 | 0.756 | -6.68E-04 | -7.85E-04 | -5.09E-03 | 4.55E-03 | 2.12E-03 | 9.54E-04 |
| K02557 | 1.332 | 1.083 | 0.508 | 0.425 | 0.685 | 0.676 | 2.99E-03 | -3.92E-04 | 2.77E-03 | 1.64E-03 | -2.16E-03 | 1.72E-03 |
| K07444 | 1.125 | 1.118 | 0.572 | 0.74 | 0.676 | 0.32 | 1.56E-03 | 3.44E-03 | 4.67E-03 | 2.44E-03 | 4.92E-04 | 4.78E-05 |
| K01338 | 0.949 | 1.16 | 0.574 | 0.411 | 0.789 | 0.674 | 6.18E-03 | 4.23E-03 | -5.92E-03 | -1.71E-03 | -7.02E-03 | 4.92E-03 |
| K03973 | 0.687 | 0.27 | 1.192 | 1.369 | 0.397 | 0.43 | -3.98E-03 | 2.01E-03 | -3.43E-04 | 6.26E-03 | -2.33E-04 | -2.44E-03 |
| K02517 | 1.212 | 0.644 | 1.776 | 0.217 | 0.539 | 0.472 | 1.94E-03 | 2.69E-03 | 1.73E-02 | 9.94E-04 | -1.20E-03 | 3.48E-03 |
| K00265 | 1.026 | 0.739 | 1.075 | 0.522 | 0.641 | 0.369 | 7.71E-03 | 1.51E-03 | 1.12E-03 | 4.45E-04 | 2.36E-04 | 2.72E-03 |

| | | | | | | | | | | | | |
|---|---|---|---|---|---|---|---|---|---|---|---|---|
| K02346 | 0.477 | 1.887 | 0.912 | 0.503 | 0.492 | 0.138 | 3.06E-03 | -1.43E-02 | -4.31E-03 | 8.51E-04 | -1.45E-03 | 2.99E-04 |
| K02114 | 0.393 | 0.335 | 1.244 | 0.681 | 0.275 | 1.132 | 2.57E-03 | 1.43E-03 | 4.55E-03 | -1.11E-03 | 2.75E-03 | 7.16E-03 |
| K06199 | 0.586 | 0.569 | 1.036 | 0.507 | 0.147 | 1.077 | -2.49E-03 | 4.90E-03 | 1.94E-03 | 2.02E-03 | -5.69E-04 | 7.90E-03 |
| K03811 | 0.953 | 0.705 | 1.257 | 0.741 | 0.753 | 0.515 | -5.68E-03 | 4.89E-03 | -3.87E-03 | 2.05E-03 | 5.82E-03 | -1.32E-04 |
| K23775 | 2.448 | 0.254 | 0.728 | 0.318 | 1.841 | 0.454 | -1.85E-02 | 6.89E-04 | 1.77E-03 | 1.66E-03 | 1.86E-02 | 2.22E-03 |
| K03787 | 0.703 | 0.7 | 1.025 | 0.791 | 0.516 | 1.025 | 1.32E-03 | 8.09E-04 | 7.48E-03 | 8.10E-04 | 3.08E-03 | -6.59E-03 |
| K09117 | 0.448 | 0.558 | 1.134 | 0.464 | 1.25 | 0.129 | 3.26E-03 | 4.66E-03 | 8.12E-03 | 1.70E-03 | 2.96E-03 | -7.57E-04 |
| K01873 | 0.539 | 1.399 | 1.023 | 0.526 | 0.608 | 0.518 | 4.13E-03 | -5.17E-03 | -6.46E-03 | 9.00E-04 | -1.97E-03 | -3.57E-03 |
| K18682 | 0.321 | 1.414 | 0.701 | 0.563 | 0.72 | 0.959 | -2.45E-03 | -1.97E-03 | -5.16E-03 | -1.96E-03 | -7.46E-03 | 2.86E-03 |
| K02519 | 0.352 | 1.408 | 1.023 | 0.603 | 0.537 | 0.364 | 1.20E-03 | 3.79E-03 | -4.52E-03 | 1.36E-03 | 1.66E-05 | 8.49E-04 |
| K19005 | 0.3 | 0.651 | 0.765 | 2.03 | 1.658 | 0.731 | -2.17E-03 | -1.81E-03 | -7.89E-05 | 9.99E-03 | 1.35E-02 | -7.48E-04 |
| K20534 | 0.743 | 0.636 | 0.338 | 1.043 | 1.255 | 0.795 | -5.74E-03 | -2.80E-03 | 3.52E-03 | 5.49E-03 | 1.14E-02 | 3.32E-03 |
| K07460 | 0.664 | 0.538 | 0.997 | 0.339 | 0.276 | 1.183 | -2.06E-03 | 3.10E-03 | 1.66E-03 | 4.84E-04 | 6.40E-04 | -7.54E-03 |
| K00760 | 0.603 | 0.606 | 0.742 | 0.15 | 0.922 | 0.87 | -4.13E-03 | -3.23E-04 | 3.81E-03 | 3.26E-04 | 9.68E-03 | 3.75E-03 |
| K00287 | 0.775 | 0.642 | 1.16 | 1.715 | 0.449 | 0.714 | -5.43E-03 | -4.31E-03 | 1.03E-02 | 8.70E-03 | -1.73E-03 | 5.20E-03 |
| K03655 | 0.527 | 0.955 | 1.197 | 0.692 | 0.546 | 0.619 | 2.86E-03 | -1.71E-03 | -1.04E-02 | 1.84E-03 | -4.70E-03 | 3.86E-03 |
| K03168 | 0.727 | 1.561 | 0.668 | 0.682 | 1.185 | 0.424 | -5.20E-03 | -6.95E-03 | -1.29E-03 | 2.97E-03 | -1.04E-02 | -1.81E-03 |
| K02520 | 1.66 | 0.719 | 0.54 | 0.349 | 0.398 | 1.06 | -1.26E-02 | 3.87E-03 | 5.82E-03 | 1.48E-03 | 4.19E-03 | 2.14E-03 |
| K02904 | 0.472 | 1.458 | 0.276 | 0.439 | 0.449 | 1.004 | -2.66E-04 | 1.18E-02 | 4.13E-04 | 2.31E-03 | 8.85E-04 | -8.81E-04 |
| K09748 | 0.782 | 0.589 | 0.479 | 1.483 | 0.309 | 0.888 | 2.42E-03 | 9.21E-04 | -4.24E-04 | 7.77E-03 | -1.48E-03 | -2.03E-03 |
| K03544 | 0.414 | 1.202 | 1.17 | 0.616 | 0.384 | 0.742 | 3.18E-03 | -5.90E-03 | 8.85E-03 | -1.31E-04 | -1.65E-03 | 5.77E-04 |
| K02838 | 0.591 | 1.013 | 0.947 | 0.767 | 0.426 | 0.428 | -3.26E-03 | 8.71E-03 | 4.19E-04 | 3.12E-03 | -3.12E-03 | -2.85E-03 |
| K21023 | 0.262 | 0.314 | 0.888 | 1.043 | 0.566 | 0.762 | 1.01E-03 | -1.02E-04 | -3.13E-03 | 3.00E-03 | 5.36E-03 | -2.76E-04 |
| K01992 | 0.532 | 0.72 | 1.393 | 0.155 | 0.895 | 0.739 | -3.97E-03 | -5.08E-03 | 1.50E-02 | -4.38E-04 | 4.92E-03 | -3.36E-03 |
| K07473 | 0.673 | 0.372 | 0.88 | 1.039 | 0.518 | 0.544 | 3.37E-03 | 3.08E-03 | 2.44E-03 | 4.59E-03 | 5.24E-03 | 2.50E-03 |
| K07484 | 0.445 | 0.438 | 0.715 | 1.307 | 1.265 | 0.519 | 2.52E-03 | 3.47E-03 | -6.84E-04 | -6.23E-03 | -1.06E-02 | -2.13E-03 |
| K07485 | 1.032 | 0.468 | 0.925 | 0.738 | 0.471 | 0.298 | 7.42E-03 | 2.97E-03 | -7.71E-04 | 1.19E-04 | 3.23E-03 | -1.53E-03 |
| K14095 | 0.206 | 0.114 | 0.87 | 0.481 | 0.475 | 0.767 | 9.75E-04 | -5.22E-04 | 5.77E-03 | 1.97E-03 | 1.46E-03 | -5.62E-03 |
| K14102 | 0.159 | 0.211 | 0.948 | 0.187 | 0.713 | 0.679 | -6.84E-04 | 1.71E-03 | -1.53E-03 | 1.49E-05 | 6.35E-03 | -5.01E-03 |
| K03050 | 0.298 | 0.426 | 1 | 0.228 | 0.452 | 0.756 | 1.08E-03 | 3.52E-03 | 4.32E-03 | -8.10E-04 | 8.78E-04 | -5.50E-03 |
| K02379 | 0.794 | 0.224 | 0.508 | 0.689 | 0.499 | 1.056 | -5.41E-03 | -1.52E-03 | 4.79E-03 | -2.16E-03 | 5.20E-03 | -7.49E-03 |
| K16329 | 0.092 | 0.568 | 0.951 | 0.099 | 0.567 | 0.33 | 5.65E-04 | -1.73E-03 | 1.28E-03 | 6.48E-05 | 5.36E-03 | 1.33E-03 |
| K18122 | 0.397 | 0.742 | 0.445 | 0.909 | 0.491 | 0.557 | 2.72E-03 | -5.12E-03 | -4.08E-03 | 1.20E-04 | 5.09E-03 | -1.63E-03 |
| K16698 | 0.37 | 0.782 | 0.54 | 0.31 | 0.481 | 0.94 | -1.86E-03 | 6.60E-03 | 2.93E-03 | -1.40E-03 | 3.15E-03 | 3.78E-03 |

| | | | | | | | | | | | | |
|---|---|---|---|---|---|---|---|---|---|---|---|---|
| K01902 | 0.348 | 0.357 | 1.142 | 0.748 | 0.416 | 0.231 | 1.81E-03 | 2.74E-03 | 3.33E-03 | 6.44E-04 | 3.94E-04 | -1.21E-03 |
| K19117 | 0.126 | 0.61 | 1.399 | 0.656 | 0.321 | 0.561 | -6.66E-04 | 1.35E-03 | 6.47E-03 | 1.56E-03 | -2.71E-03 | 8.21E-04 |
| K19119 | 0.11 | 0.433 | 1.198 | 0.654 | 0.554 | 0.719 | -7.64E-04 | 2.29E-03 | 3.95E-03 | 6.92E-04 | -5.66E-03 | -4.25E-03 |
| K19118 | 0.198 | 0.468 | 1.367 | 0.677 | 0.104 | 0.651 | -1.23E-03 | 1.96E-03 | 6.05E-03 | 1.33E-03 | 4.98E-04 | 2.95E-03 |
| K07741 | 0.715 | 0.281 | 1.599 | 0.758 | 0.411 | 0.786 | -5.00E-03 | -1.14E-04 | 1.55E-02 | 3.03E-03 | -4.18E-03 | -3.67E-03 |
| K19159 | 1.257 | 0.573 | 0.638 | 0.655 | 0.624 | 0.676 | 8.82E-03 | -4.57E-03 | -2.92E-03 | 2.12E-03 | -6.48E-03 | 4.78E-03 |
| K02037 | 0.321 | 0.487 | 1.033 | 0.704 | 0.44 | 0.675 | -2.49E-03 | -8.87E-05 | -2.95E-03 | 1.19E-03 | -2.64E-03 | 2.98E-03 |
| K02036 | 0.289 | 0.513 | 1.104 | 0.592 | 0.418 | 0.691 | -2.17E-03 | -3.16E-03 | -1.72E-03 | 5.25E-04 | -2.91E-03 | 2.94E-03 |
| K11358 | 0.314 | 0.614 | 1.289 | 0.786 | 0.251 | 0.635 | -7.91E-04 | 4.82E-03 | 6.44E-03 | 8.42E-04 | 7.50E-04 | 2.00E-03 |
| K00980 | 0.506 | 0.754 | 0.314 | 0.728 | 0.632 | 0.976 | -1.66E-04 | -1.98E-03 | 2.42E-03 | 2.54E-03 | 6.45E-03 | 3.97E-03 |
| K08679 | 0.613 | 1.541 | 0.147 | 0.387 | 0.363 | 0.63 | -1.76E-03 | 1.03E-02 | -5.69E-05 | -1.91E-03 | -3.51E-04 | 2.75E-03 |
| K01524 | 0.22 | 0.647 | 1.171 | 0.788 | 0.094 | 0.442 | 1.21E-03 | -1.32E-03 | -8.96E-05 | 1.99E-04 | 3.79E-04 | 2.48E-03 |
| K03086 | 0.781 | 0.345 | 1.184 | 0.754 | 0.292 | 0.688 | 5.63E-03 | 2.96E-03 | -2.05E-03 | 1.33E-04 | -2.99E-03 | 8.49E-04 |
| K17828 | 0.56 | 1.344 | 0.525 | 0.152 | 0.607 | 0.666 | 3.15E-03 | -1.78E-03 | 5.52E-03 | 6.19E-04 | 4.69E-03 | -3.56E-03 |
| K22522 | 0.748 | 0.916 | 0.79 | 0.506 | 0.694 | 0.397 | -3.42E-03 | 7.12E-03 | 2.62E-03 | 7.38E-04 | -7.07E-03 | -1.78E-03 |
| K02493 | 0.557 | 0.53 | 0.66 | 1.591 | 0.507 | 0.788 | -4.03E-03 | 1.44E-03 | 1.88E-03 | 8.03E-03 | 1.33E-03 | -4.56E-03 |
| K01808 | 0.142 | 0.288 | 0.698 | 0.269 | 0.73 | 1.473 | -1.10E-03 | 8.59E-04 | -5.55E-04 | 1.26E-03 | 8.42E-04 | -9.43E-03 |
| K07979 | 0.411 | 0.763 | 0.393 | 0.315 | 0.762 | 1.073 | 7.88E-06 | -6.25E-03 | 2.45E-03 | -1.30E-03 | 6.94E-03 | 1.04E-03 |
| K03499 | 0.46 | 1.462 | 0.569 | 0.52 | 0.775 | 0.666 | 3.40E-03 | 1.02E-02 | 1.79E-03 | 2.32E-04 | -2.86E-03 | -1.08E-03 |
| K03498 | 0.329 | 1.373 | 0.399 | 0.627 | 0.244 | 0.149 | 2.20E-03 | -1.28E-03 | 1.30E-03 | 2.60E-03 | -2.58E-03 | 1.09E-03 |
| K03630 | 0.471 | 1.275 | 0.265 | 0.707 | 0.562 | 0.503 | -3.63E-03 | -5.71E-03 | -1.82E-03 | 3.13E-03 | 3.54E-03 | -2.78E-03 |
| K02316 | 0.67 | 1.305 | 0.177 | 0.339 | 0.77 | 0.316 | 4.69E-03 | -8.21E-03 | -1.61E-03 | 9.70E-04 | -5.76E-03 | 6.37E-06 |
| K14118 | 0.294 | 0.111 | 0.697 | 0.084 | 0.432 | 0.638 | -2.23E-03 | -1.92E-04 | 5.25E-03 | 2.96E-04 | 1.17E-03 | -4.71E-03 |
| K00641 | 0.218 | 0.703 | 0.769 | 0.273 | 0.663 | 0.743 | -3.20E-04 | -4.62E-03 | -4.56E-03 | 1.44E-03 | 7.01E-03 | -5.38E-03 |
| K16214 | 0.089 | 0.438 | 0.748 | 0.528 | 0.481 | 0.553 | -1.46E-05 | 3.42E-03 | -2.05E-03 | -7.10E-05 | -3.12E-03 | -3.50E-03 |

**Trait: Daily Feed Intake (DFI); Predictors: 673 heritable microbial genes**

| Microbial gene | VIP | | | | | | Regression coefficient | | | | | |
|---|---|---|---|---|---|---|---|---|---|---|---|---|
| | T1 | T2 | T3 | T4 | T5 | T6 | T1 | T2 | T3 | T4 | T5 | T6 |
| K02217$_{VIP\geq0.8\ (+)}$ | 0.896 | 1.28 | 1.588 | 1.147 | 0.938 | 1.548 | 5.03E-03 | 1.03E-02 | 7.55E-03 | 4.53E-03 | 5.98E-03 | 1.05E-02 |
| K06990$_{VIP\geq0.8\ (+)}$ | 1.004 | 1.039 | 0.982 | 1.435 | 1.019 | 0.991 | 1.80E-03 | 5.64E-05 | 7.62E-04 | 5.90E-03 | 5.83E-03 | 2.51E-03 |
| K07464$_{VIP\geq0.8\ (+)}$ | 1.108 | 1.258 | 1.287 | 1.406 | 0.889 | 1.124 | 3.39E-03 | 6.00E-03 | 3.93E-03 | 5.41E-03 | 4.14E-03 | 6.03E-03 |
| K13038$_{VIP\geq0.8\ (+)}$ | 1.545 | 1.156 | 0.978 | 1 | 1.634 | 0.932 | 1.04E-02 | 4.90E-03 | 2.53E-03 | 1.61E-03 | 1.37E-02 | 6.08E-03 |

| | | | | | | | | | | | | |
|---|---|---|---|---|---|---|---|---|---|---|---|---|
| $K07503_{VIP\geq0.8\ (+)}$ | 0.738 | 1.909 | 1.899 | 1.541 | 1.199 | 0.995 | 4.48E-03 | 1.91E-02 | 1.36E-02 | 1.35E-02 | 8.98E-03 | 6.54E-03 |
| $K02379_{VIP\geq0.8\ (+)}$ | 0.518 | 1.28 | 1.406 | 1.205 | 1.153 | 0.817 | 1.62E-03 | 1.23E-02 | 1.02E-02 | 8.96E-03 | 8.94E-03 | 5.65E-03 |
| $K02007_{VIP\geq0.8\ (+)}$ | 0.84 | 1.437 | 1.318 | 2.153 | 1.29 | 0.622 | 4.90E-04 | 1.66E-02 | 6.65E-03 | 1.47E-02 | 1.01E-02 | 4.29E-03 |
| $K00878_{VIP\geq0.8\ (+)}$ | 0.772 | 0.958 | 1.109 | 0.977 | 0.879 | 1.093 | 2.55E-03 | 2.70E-03 | 4.81E-03 | 1.23E-03 | 1.71E-03 | 7.56E-03 |
| $K06898_{VIP\geq0.8\ (+)}$ | 0.805 | 1.701 | 1.034 | 1.28 | 0.928 | 0.38 | 1.10E-03 | 1.89E-02 | 3.92E-03 | 5.81E-03 | 2.32E-03 | 1.85E-03 |
| $K04654_{VIP\geq0.8\ (+)}$ | 1.236 | 1.518 | 1.339 | 1.035 | 1.061 | 0.508 | 6.49E-03 | 1.66E-02 | 4.80E-03 | 6.61E-04 | 4.30E-03 | 2.02E-04 |
| $K04653_{VIP\geq0.8\ (+)}$ | 0.959 | 1.528 | 1.213 | 1.112 | 1.006 | 0.437 | 5.24E-03 | 1.82E-02 | 5.35E-03 | 3.57E-03 | 4.08E-03 | 2.75E-03 |
| $K06894_{VIP\geq0.8\ (+)}$ | 1.152 | 1.169 | 0.426 | 1.502 | 1.9 | 1.643 | 7.01E-03 | 1.18E-02 | 2.99E-03 | 1.20E-02 | 1.57E-02 | 1.06E-02 |
| $K02426_{VIP\geq0.8\ (+)}$ | 0.853 | 0.62 | 1.049 | 0.815 | 0.828 | 0.987 | 3.74E-04 | 6.09E-03 | 2.66E-03 | 3.96E-03 | 2.92E-04 | 6.08E-03 |
| $K07502_{VIP\geq0.8\ (+)}$ | 1.147 | 0.775 | 1.214 | 1.557 | 0.971 | 0.788 | 4.62E-03 | 4.82E-03 | 5.58E-03 | 8.84E-03 | 6.60E-03 | 4.84E-03 |
| $K01235_{VIP\geq0.8\ (+)}$ | 1.292 | 0.568 | 0.813 | 0.567 | 1.161 | 1.01 | 8.00E-03 | 3.30E-03 | 4.80E-03 | 4.72E-03 | 9.42E-03 | 5.63E-03 |
| $K23675_{VIP\geq0.8\ (+)}$ | 0.858 | 0.743 | 0.982 | 0.955 | 1.123 | 0.188 | 2.06E-03 | 6.81E-03 | 1.25E-03 | 1.31E-03 | 2.93E-03 | 1.30E-03 |
| $K03281_{VIP\geq0.8\ (+)}$ | 1.06 | 1.119 | 0.169 | 0.673 | 0.838 | 1.306 | 5.79E-03 | 6.16E-03 | 7.98E-04 | 3.17E-03 | 7.19E-03 | 3.10E-03 |
| $K00012_{VIP\geq0.8\ (+)}$ | 0.529 | 2.18 | 1.257 | 1.346 | 0.275 | 1.157 | 7.02E-05 | 2.57E-02 | 9.03E-03 | 1.10E-02 | 1.44E-03 | 5.68E-03 |
| $K13002_{VIP\geq0.8\ (+)}$ | 0.802 | 1.13 | 1.338 | 0.624 | 0.67 | 0.981 | 2.47E-03 | 1.30E-02 | 9.13E-03 | 5.25E-03 | 4.01E-03 | 4.79E-03 |
| $K03050_{VIP\geq0.8\ (+)}$ | 0.951 | 1.311 | 1.478 | 2.208 | 0.877 | 0.843 | -2.29E-03 | 1.01E-02 | 9.61E-03 | 1.92E-02 | 4.12E-03 | 2.86E-03 |
| $K02626_{VIP\geq0.8\ (+)}$ | 1.049 | 0.912 | 1.4 | 1.644 | 1.829 | 1.084 | -5.57E-03 | 3.15E-03 | 9.26E-03 | 1.43E-02 | 1.48E-02 | 4.37E-03 |
| $K00578_{VIP\geq0.8\ (+)}$ | 0.91 | 0.973 | 1.53 | 1.792 | 1.291 | 0.986 | -5.18E-03 | 6.98E-03 | 1.03E-02 | 1.57E-02 | 8.49E-03 | 4.81E-03 |
| $K01902_{VIP\geq0.8\ (+)}$ | 1.156 | 1 | 1.033 | 1.402 | 0.936 | 1.428 | 3.23E-03 | 2.75E-03 | -3.99E-03 | 4.90E-03 | 1.62E-03 | 3.29E-03 |
| $K07133_{VIP\geq0.8\ (+)}$ | 0.953 | 1.295 | 1.082 | 0.956 | 0.972 | 1.478 | 4.73E-03 | 4.09E-03 | 1.98E-03 | -1.74E-03 | 3.44E-03 | 6.64E-03 |
| $K03049_{VIP\geq0.8\ (+)}$ | 0.842 | 1.792 | 1.727 | 2.004 | 0.939 | 0.775 | -4.56E-03 | 1.99E-02 | 1.21E-02 | 1.74E-02 | 3.50E-03 | 5.11E-03 |
| $K07569_{VIP\geq0.8\ (+)}$ | 0.791 | 1.476 | 0.848 | 1.694 | 1.618 | 0.823 | -3.61E-03 | 1.36E-02 | 3.99E-03 | 1.48E-02 | 1.24E-02 | 2.85E-03 |
| $K14118_{VIP\geq0.8\ (+)}$ | 0.718 | 1.531 | 1.911 | 1.717 | 1.019 | 0.928 | -2.95E-03 | 1.50E-02 | 1.38E-02 | 1.50E-02 | 5.06E-03 | 4.50E-03 |
| $K14117_{VIP\geq0.8\ (+)}$ | 1.406 | 1.677 | 1.714 | 1.77 | 1.415 | 0.746 | -8.30E-03 | 1.55E-02 | 1.20E-02 | 1.54E-02 | 9.74E-03 | 1.44E-03 |
| $K14119_{VIP\geq0.8\ (+)}$ | 0.944 | 1.651 | 1.707 | 2.035 | 1.179 | 0.756 | -4.33E-03 | 1.55E-02 | 1.19E-02 | 1.78E-02 | 7.74E-03 | 7.26E-04 |
| $K02009_{VIP\geq0.8\ (+)}$ | 0.868 | 1.309 | 1.422 | 0.982 | 1.056 | 0.455 | -3.46E-03 | 9.69E-03 | 9.33E-03 | 8.03E-03 | 6.28E-03 | 1.33E-03 |
| $K00641_{VIP\geq0.8\ (+)}$ | 0.949 | 0.637 | 1.088 | 1.828 | 1.394 | 1.041 | -5.89E-03 | 3.77E-03 | 7.66E-03 | 1.59E-02 | 1.00E-02 | 6.21E-03 |
| $K04656_{VIP\geq0.8\ (+)}$ | 1.281 | 1.237 | 1.009 | 0.963 | 1.133 | 0.398 | 6.54E-03 | 3.80E-03 | 3.51E-04 | -3.08E-03 | 4.97E-03 | 1.64E-04 |
| $K08963_{VIP\geq0.8\ (+)}$ | 1.012 | 1.312 | 1.202 | 1.287 | 1.299 | 0.599 | -1.53E-03 | 5.46E-03 | 3.67E-03 | 3.41E-03 | 7.94E-03 | 2.09E-03 |
| $K09121_{VIP\geq0.8\ (+)}$ | 0.82 | 1.228 | 1.309 | 1.476 | 0.914 | 0.704 | 2.82E-03 | 7.26E-03 | 5.61E-03 | 6.21E-03 | 2.89E-03 | -3.71E-03 |

| | | | | | | | | | | | | |
|---|---|---|---|---|---|---|---|---|---|---|---|---|
| $K04655_{VIP \geq 0.8\ (+)}$ | 1.369 | 1.129 | 1.109 | 1.063 | 0.95 | 0.705 | 7.57E-03 | 9.07E-03 | 1.41E-03 | 8.78E-04 | 1.61E-03 | -1.55E-03 |
| $K03606_{VIP \geq 0.8\ (+)}$ | 0.825 | 1.162 | 1.966 | 1.537 | 0.386 | 2.148 | 9.31E-04 | 7.64E-03 | 1.21E-02 | 9.63E-03 | -8.34E-04 | 1.48E-02 |
| $K00788_{VIP \geq 0.8\ (+)}$ | 0.649 | 1.324 | 1.73 | 1.033 | 1.198 | 1.646 | 5.51E-04 | 1.46E-02 | 9.72E-03 | 3.63E-03 | -8.94E-03 | 1.14E-02 |
| $K00941_{VIP \geq 0.8\ (+)}$ | 0.86 | 0.734 | 1.327 | 1.169 | 1.495 | 0.882 | 1.14E-03 | 2.85E-03 | 4.73E-03 | 2.88E-03 | -9.81E-03 | 5.42E-03 |
| $K00340_{VIP \geq 0.8\ (+)}$ | 0.867 | 0.882 | 0.843 | 0.715 | 0.827 | 0.948 | 3.01E-03 | 1.02E-02 | 3.19E-04 | 3.39E-03 | -4.83E-03 | 5.90E-03 |
| $K00721_{VIP \geq 0.8\ (+)}$ | 0.861 | 1.531 | 1.168 | 0.824 | 0.662 | 1.241 | 3.47E-04 | 1.67E-02 | 5.86E-03 | 1.88E-03 | -7.85E-04 | 8.56E-03 |
| $K04751_{VIP \geq 0.8\ (+)}$ | 0.775 | 0.97 | 1.189 | 0.914 | 1.009 | 1.433 | 2.38E-03 | 9.03E-03 | 4.91E-03 | -1.70E-03 | 3.92E-03 | 8.41E-03 |
| $K00683_{VIP \geq 0.8\ (+)}$ | 0.902 | 0.918 | 1.092 | 1.193 | 0.63 | 1.572 | 2.77E-03 | 1.09E-02 | 5.45E-03 | 1.03E-02 | -2.58E-03 | 1.08E-02 |
| $K02040_{VIP \geq 0.8\ (+)}$ | 0.867 | 1.403 | 1.046 | 0.683 | 1.097 | 1.107 | 2.87E-03 | -4.80E-04 | 5.48E-04 | 1.61E-03 | 8.24E-03 | 2.83E-04 |
| $K21571_{VIP \geq 0.8\ (+)}$ | 0.754 | 0.913 | 0.95 | 0.855 | 1.547 | 1.292 | 4.67E-03 | 3.36E-03 | 6.85E-03 | 7.14E-03 | -1.26E-02 | 4.16E-03 |
| $K00615_{VIP \geq 0.8\ (+)}$ | 0.879 | 0.83 | 0.815 | 1.365 | 1.397 | 0.185 | 5.54E-03 | 2.25E-04 | 2.22E-03 | 5.61E-03 | 9.12E-03 | -1.11E-03 |
| $K14092_{VIP \geq 0.8\ (+)}$ | 0.693 | 1.471 | 1.605 | 1.376 | 1.122 | 0.51 | -3.01E-03 | 1.36E-02 | 1.11E-02 | 1.16E-02 | 6.23E-03 | 1.36E-03 |
| $K14102_{VIP \geq 0.8\ (+)}$ | 0.605 | 1.559 | 1.444 | 1.611 | 1.337 | 0.528 | -4.12E-04 | 1.41E-02 | 9.78E-03 | 1.41E-02 | 8.78E-03 | 2.39E-03 |
| $K14096_{VIP \geq 0.8\ (+)}$ | 0.687 | 1.263 | 1.429 | 1.521 | 1.402 | 0.627 | -7.12E-04 | 1.07E-02 | 9.40E-03 | 1.33E-02 | 1.03E-02 | 2.03E-03 |
| $K02466_{VIP \geq 0.8\ (+)}$ | 0.857 | 0.504 | 1.352 | 0.598 | 1.1 | 0.846 | 4.22E-03 | 5.99E-03 | 9.63E-03 | 1.83E-03 | -8.15E-03 | 3.14E-03 |
| $K15024_{VIP \geq 0.8\ (+)}$ | 0.585 | 1.29 | 1.288 | 1.178 | 0.144 | 1.328 | 2.94E-03 | -1.38E-02 | 9.32E-03 | 9.08E-03 | 1.22E-03 | 7.63E-03 |
| $K03057_{VIP \geq 0.8\ (+)}$ | 0.79 | 1.094 | 1.676 | 1.947 | 1.19 | 0.763 | -1.39E-03 | 8.06E-03 | 1.20E-02 | 1.70E-02 | 7.54E-03 | 2.80E-03 |
| $K02781_{VIP \geq 0.8\ (+)}$ | 1.043 | 1.431 | 1.294 | 0.511 | 1.052 | 0.147 | 5.82E-03 | 1.70E-02 | 8.58E-03 | 4.41E-04 | -9.01E-03 | 9.41E-04 |
| $K01780_{VIP \geq 0.8\ (+)}$ | 0.81 | 1.034 | 1.2 | 0.589 | 1.098 | 0.568 | -3.05E-03 | 6.43E-03 | 6.72E-03 | 4.47E-03 | 8.66E-03 | 1.24E-03 |
| $K02019_{VIP \geq 0.8\ (+)}$ | 0.736 | 1.263 | 1.066 | 0.423 | 0.987 | 0.816 | 2.57E-03 | 7.78E-03 | 7.26E-03 | 1.34E-03 | 8.36E-03 | -2.30E-03 |
| $K03750_{VIP \geq 0.8\ (+)}$ | 0.989 | 1.124 | 0.786 | 1.352 | 0.822 | 0.49 | 5.94E-03 | 5.35E-03 | 3.03E-03 | 1.06E-02 | 5.60E-03 | -2.93E-03 |
| $K03785_{VIP \geq 0.8\ (+)}$ | 1.814 | 1.1 | 1.52 | 0.587 | 0.978 | 0.372 | 1.14E-02 | 1.13E-02 | 7.17E-03 | -2.61E-03 | 7.14E-03 | 9.58E-04 |
| $K01749_{VIP \geq 0.8\ (+)}$ | 0.523 | 1.252 | 1.098 | 0.823 | 0.296 | 0.813 | 2.26E-03 | 1.24E-02 | 4.70E-03 | 4.30E-04 | -2.09E-03 | 2.55E-03 |
| $K23393_{VIP \geq 0.8\ (+)}$ | 1.239 | 0.441 | 1.106 | 0.939 | 1.423 | 0.495 | -6.24E-03 | 4.77E-03 | 4.47E-03 | 3.12E-03 | 1.08E-02 | 3.25E-03 |
| $K01609_{VIP \geq 0.8\ (+)}$ | 1.031 | 1.411 | 0.904 | 1.348 | 0.739 | 0.658 | 6.05E-03 | 1.65E-02 | 2.70E-03 | 6.68E-03 | -5.38E-03 | 5.43E-05 |
| $K00797_{VIP \geq 0.8\ (+)}$ | 1.346 | 0.471 | 0.996 | 0.954 | 1.144 | 0.482 | 8.51E-03 | 3.32E-04 | -3.45E-03 | 2.73E-04 | 2.70E-03 | 1.76E-03 |
| $K01858_{VIP \geq 0.8\ (+)}$ | 1.469 | 0.681 | 1.784 | 0.807 | 0.698 | 1.656 | 9.62E-03 | 8.02E-03 | 1.30E-02 | 6.86E-03 | -5.99E-03 | 1.11E-02 |
| $K07741_{VIP \geq 0.8\ (+)}$ | 0.335 | 1.079 | 2.211 | 1.792 | 0.736 | 1.176 | 6.27E-04 | 1.19E-02 | 1.46E-02 | 9.55E-03 | -3.30E-03 | 7.48E-03 |
| $K18828_{VIP \geq 0.8\ (+)}$ | 1.902 | 2.793 | 0.256 | 1.355 | 1.07 | 0.354 | 1.29E-02 | 3.31E-02 | -1.74E-03 | 1.11E-02 | 9.11E-03 | 1.38E-03 |
| $K21029_{VIP \geq 0.8\ (+)}$ | 1.226 | 2.434 | 0.547 | 0.27 | 2.432 | 0.825 | -5.88E-03 | 2.83E-02 | 3.58E-03 | 4.37E-05 | 2.05E-02 | 3.91E-05 |

| | | | | | | | | | | | | |
|---|---|---|---|---|---|---|---|---|---|---|---|---|
| K23536$_{VIP\geq0.8\ (+)}$ | 0.759 | 0.925 | 0.9 | 0.961 | 1.079 | 0.171 | 5.19E-04 | -6.24E-03 | 2.09E-03 | 3.57E-04 | 2.75E-03 | 1.17E-03 |
| K00046$_{VIP\geq0.8\ (+)}$ | 1.246 | 0.585 | 0.911 | 0.81 | 0.835 | 0.636 | 7.31E-03 | 5.56E-03 | 5.44E-03 | 6.87E-03 | -2.08E-03 | 4.40E-03 |
| K01817$_{VIP\geq0.8\ (+)}$ | 1.612 | 1.05 | 0.053 | 0.749 | 1 | 0.844 | 1.09E-02 | 4.34E-03 | 3.86E-04 | 2.25E-03 | -7.30E-03 | 5.06E-03 |
| K01144$_{VIP\geq0.8\ (+)}$ | 1.249 | 0.632 | 0.715 | 0.889 | 0.893 | 1.661 | 7.11E-03 | 6.06E-03 | 1.64E-03 | -3.40E-03 | 6.07E-03 | 9.97E-03 |
| K04085$_{VIP\geq0.8\ (+)}$ | 1.531 | 1.155 | 0.615 | 0.635 | 1.52 | 1.013 | 9.99E-03 | 1.31E-02 | -3.63E-03 | 6.15E-04 | 1.20E-02 | 3.91E-03 |
| K00992$_{VIP\geq0.8\ (+)}$ | 1.073 | 1.139 | 1.101 | 0.193 | 0.315 | 1.079 | 5.84E-03 | 1.12E-02 | -6.62E-03 | 1.31E-03 | 8.30E-05 | 1.67E-03 |
| K03811$_{VIP\geq0.8\ (+)}$ | 1.168 | 0.945 | 1.131 | 0.716 | 0.754 | 0.886 | 1.15E-03 | 8.32E-03 | -1.06E-04 | 2.55E-03 | 1.43E-03 | 4.94E-03 |
| K00865$_{VIP\geq0.8\ (+)}$ | 0.827 | 1.656 | 0.697 | 0.846 | 0.573 | 1.446 | 4.11E-03 | 1.47E-02 | 6.12E-04 | -5.09E-04 | 3.09E-03 | 3.70E-03 |
| K09014$_{VIP\geq0.8\ (+)}$ | 1.088 | 1.121 | 0.933 | 0.767 | 0.69 | 0.934 | 7.35E-03 | 8.15E-04 | 4.32E-03 | -5.76E-03 | 4.51E-03 | 4.59E-03 |
| K01940$_{VIP\geq0.8\ (+)}$ | 1.334 | 1.415 | 0.729 | 1.159 | 0.543 | 0.849 | 6.63E-03 | 8.50E-03 | -8.88E-04 | 4.03E-03 | 2.47E-03 | 1.56E-03 |
| K08234$_{VIP\geq0.8\ (+)}$ | 1.334 | 0.854 | 0.932 | 0.837 | 1.789 | 1.095 | 8.74E-03 | 1.78E-04 | 6.76E-03 | 7.05E-03 | -1.50E-02 | -4.98E-03 |
| K09767$_{VIP\geq0.8\ (+)}$ | 1.208 | 0.873 | 1.315 | 0.855 | 1.136 | 0.974 | 4.51E-03 | -8.40E-03 | -7.77E-03 | 1.00E-03 | 4.85E-03 | 2.72E-03 |
| K02456$_{VIP\geq0.8\ (+)}$ | 1.179 | 1.066 | 0.991 | 1.389 | 1.069 | 1.104 | 6.40E-03 | -8.81E-03 | -6.17E-03 | 6.44E-03 | 2.63E-04 | 2.13E-03 |
| K01845$_{VIP\geq0.8\ (+)}$ | 1.087 | 1.189 | 0.801 | 0.94 | 0.974 | 1.1 | 6.33E-03 | 1.78E-03 | -1.80E-03 | -1.22E-03 | 6.65E-03 | 2.26E-03 |
| K00549$_{VIP\geq0.8\ (+)}$ | 1.053 | 1.084 | 1.412 | 0.865 | 1.409 | 1.071 | -6.99E-04 | 3.96E-03 | 5.90E-03 | -1.14E-03 | 1.08E-02 | 6.55E-03 |
| K06975$_{VIP\geq0.8\ (+)}$ | 2.078 | 0.859 | 0.84 | 1.356 | 0.855 | 2.124 | 1.39E-02 | 1.02E-02 | 1.99E-04 | -1.14E-02 | -3.35E-04 | 1.38E-02 |
| K02037$_{VIP\geq0.8\ (+)}$ | 1.192 | 1.02 | 1.074 | 1.04 | 1.07 | 0.851 | 2.96E-04 | 4.98E-04 | -2.18E-03 | -5.86E-03 | 5.37E-03 | 1.60E-03 |
| K02026$_{VIP\geq0.8\ (+)}$ | 0.91 | 1.16 | 0.993 | 0.838 | 1.405 | 0.957 | -4.78E-03 | 1.13E-02 | 5.79E-03 | -2.28E-03 | 7.50E-03 | 6.20E-03 |
| K07105$_{VIP\geq0.8\ (+)}$ | 1.203 | 0.926 | 1.138 | 1.37 | 1.103 | 1.156 | -5.92E-04 | 2.82E-03 | 1.56E-03 | 5.32E-03 | -4.56E-03 | 7.13E-03 |
| K01246$_{VIP\geq0.8\ (+)}$ | 1.215 | 1.163 | 1.086 | 1.104 | 1.451 | 1.259 | 3.84E-03 | -5.71E-03 | -1.37E-03 | 1.53E-03 | 8.53E-03 | 5.38E-03 |
| K02114$_{VIP\geq0.8\ (+)}$ | 1.219 | 1.131 | 1.265 | 1.023 | 1.221 | 0.866 | 2.63E-03 | 6.72E-03 | 3.01E-03 | -3.85E-03 | 3.16E-03 | -2.14E-04 |
| K02115$_{VIP\geq0.8\ (+)}$ | 0.824 | 1.33 | 0.964 | 0.815 | 0.872 | 1.265 | 1.50E-03 | 2.62E-03 | -1.64E-03 | -1.40E-03 | 3.08E-03 | 4.67E-03 |
| K02030$_{VIP\geq0.8\ (+)}$ | 0.934 | 1.923 | 1.087 | 1.059 | 1.195 | 1.128 | -2.62E-03 | 2.25E-02 | 3.92E-03 | -5.60E-04 | 7.56E-04 | 7.30E-03 |
| K04763$_{VIP\geq0.8\ (+)}$ | 1.221 | 1.36 | 0.854 | 1.519 | 0.816 | 1.731 | 5.10E-03 | -5.27E-03 | -4.77E-03 | 6.50E-03 | 2.83E-04 | 9.11E-03 |
| K00441$_{VIP\geq0.8\ (+)}$ | 0.945 | 0.382 | 1.006 | 1.989 | 0.936 | 1.66 | -6.39E-03 | -2.28E-03 | 7.18E-03 | 1.65E-02 | 4.99E-03 | 1.14E-02 |
| K06962$_{VIP\geq0.8\ (+)}$ | 0.589 | 1.17 | 1.017 | 0.903 | 1.237 | 1.287 | -9.42E-04 | 6.82E-03 | 1.25E-03 | -2.18E-03 | 6.18E-03 | 3.14E-03 |
| K09706$_{VIP\geq0.8\ (+)}$ | 0.95 | 0.51 | 1.293 | 0.95 | 1.051 | 1.669 | -4.28E-03 | -8.43E-04 | 7.25E-03 | 4.96E-03 | 9.01E-03 | 1.13E-02 |
| K06972$_{VIP\geq0.8\ (+)}$ | 0.926 | 0.977 | 1.073 | 1.029 | 1.021 | 0.451 | 1.46E-03 | 1.85E-03 | 1.33E-03 | -2.39E-03 | 3.44E-04 | -1.86E-03 |
| K00872$_{VIP\geq0.8\ (+)}$ | 1.121 | 0.661 | 1.155 | 1.007 | 0.974 | 1.197 | 2.61E-03 | 2.41E-03 | -4.83E-03 | -9.62E-04 | 4.16E-03 | 1.65E-03 |
| K06940$_{VIP\geq0.8\ (+)}$ | 0.849 | 0.746 | 0.964 | 1.01 | 1.017 | 1.177 | -1.94E-03 | 1.32E-03 | 1.90E-03 | -5.72E-03 | 1.09E-03 | 8.12E-03 |

| | | | | | | | | | | | | |
|---|---|---|---|---|---|---|---|---|---|---|---|---|
| $K02027_{VIP≥0.8\ (+)}$ | 0.816 | 0.963 | 1.106 | 0.978 | 1.052 | 0.79 | -1.39E-03 | 5.41E-03 | 4.23E-03 | -1.58E-03 | 3.70E-03 | 3.30E-03 |
| $K00240_{VIP≥0.8\ (+)}$ | 0.918 | 0.709 | 1.324 | 1.324 | 0.955 | 1.608 | 3.73E-03 | 7.77E-03 | 9.61E-03 | -1.07E-02 | -3.48E-03 | 9.58E-03 |
| $K07075_{VIP≥0.8\ (+)}$ | 1.446 | 1.379 | 0.975 | 1.025 | 1.392 | 0.501 | -7.66E-03 | 1.51E-02 | 5.00E-03 | 7.64E-03 | 1.02E-02 | -2.66E-03 |
| $K01610_{VIP≥0.8\ (+)}$ | 0.898 | 1.251 | 1.124 | 0.983 | 0.519 | 1.181 | 1.51E-03 | 2.73E-03 | 2.80E-03 | -3.31E-04 | 2.70E-03 | -5.96E-03 |
| $K18831_{VIP≥0.8\ (+)}$ | 1.927 | 1.028 | 0.839 | 0.423 | 1.784 | 1.461 | 1.30E-02 | 3.63E-03 | -5.97E-03 | -3.44E-03 | 1.38E-02 | 7.09E-03 |
| $K02965_{VIP≥0.8\ (+)}$ | 0.87 | 0.932 | 0.987 | 1.144 | 0.296 | 1.092 | 1.35E-04 | 1.37E-03 | 2.89E-03 | -6.30E-03 | -2.50E-03 | 4.07E-03 |
| $K03561_{VIP≥0.8\ (+)}$ | 0.812 | 0.895 | 0.821 | 1.449 | 0.544 | 0.908 | 1.78E-03 | 7.19E-03 | 1.45E-03 | -1.15E-02 | -4.62E-03 | 6.13E-03 |
| $K07493_{VIP≥0.8\ (+)}$ | 1.342 | 1.505 | 1.113 | 1.457 | 1.223 | 0.661 | 5.27E-03 | -8.91E-03 | -2.25E-03 | 5.50E-03 | 6.63E-03 | 7.22E-04 |
| $K06142_{VIP≥0.8\ (+)}$ | 0.797 | 1.03 | 0.998 | 0.808 | 0.916 | 1.245 | 3.46E-04 | 1.22E-02 | 2.22E-03 | -1.35E-03 | -5.67E-03 | 7.19E-03 |
| $K03327_{VIP≥0.8\ (+)}$ | 0.961 | 1.393 | 1.351 | 1.389 | 0.813 | 0.704 | -2.13E-03 | 1.74E-03 | 5.58E-03 | 5.73E-03 | -2.55E-05 | 3.52E-03 |
| $K14095_{VIP≥0.8\ (+)}$ | 0.526 | 1.369 | 1.41 | 1.008 | 1.419 | 0.735 | -1.07E-03 | 1.14E-02 | 9.45E-03 | 7.98E-03 | 9.82E-03 | -1.10E-03 |
| $K03053_{VIP≥0.8\ (+)}$ | 0.756 | 1.639 | 1.83 | 1.504 | 1.13 | 0.361 | -2.42E-03 | 1.73E-02 | 1.30E-02 | 1.32E-02 | 6.14E-03 | -2.02E-03 |
| $K23264_{VIP≥0.8\ (+)}$ | 1.007 | 1.146 | 1.536 | 0.982 | 0.729 | 0.545 | -5.70E-03 | 8.80E-03 | 1.06E-02 | 8.53E-03 | 4.40E-03 | -2.63E-04 |
| $K21993_{VIP≥0.8\ (+)}$ | 0.747 | 0.93 | 1.099 | 0.789 | 1.155 | 1.129 | 1.48E-03 | -4.61E-03 | 7.28E-03 | 5.72E-03 | 1.89E-03 | -2.14E-03 |
| $K01095_{VIP≥0.8\ (+)}$ | 0.324 | 1.427 | 0.634 | 2.414 | 0.8 | 3.242 | -8.99E-04 | 1.68E-02 | 3.14E-03 | 1.68E-02 | -1.93E-03 | 2.24E-02 |
| $K00616_{VIP≥0.8\ (+)}$ | 1.056 | 0.323 | 1.049 | 0.887 | 1.144 | 0.718 | 5.31E-03 | 5.94E-04 | -4.96E-03 | -5.33E-03 | 6.30E-03 | 3.33E-03 |
| $K23876_{VIP≥0.8\ (+)}$ | 1.002 | 0.758 | 1.135 | 2.003 | 0.958 | 0.724 | -4.34E-03 | 4.05E-03 | 4.59E-03 | 1.27E-02 | -2.16E-04 | 4.77E-03 |
| $K05799_{VIP≥0.8\ (+)}$ | 2.625 | 0.585 | 1.087 | 1.73 | 1.417 | 0.636 | -1.75E-02 | -4.28E-03 | 7.75E-03 | 1.37E-02 | 1.07E-02 | 2.07E-03 |
| $K19689_{VIP≥0.8\ (+)}$ | 0.8 | 1.18 | 0.919 | 1.001 | 1.164 | 0.409 | 9.22E-04 | -7.40E-03 | 2.29E-04 | -4.96E-03 | 1.35E-03 | 7.99E-04 |
| $K03929_{VIP≥0.8\ (+)}$ | 0.684 | 0.993 | 0.757 | 1.372 | 1.023 | 1.532 | 4.18E-03 | -5.89E-03 | 5.50E-03 | 9.67E-03 | -8.43E-03 | 7.71E-03 |
| $K08722_{VIP≥0.8\ (+)}$ | 1.078 | 0.544 | 0.981 | 1.092 | 1.111 | 0.784 | 3.04E-03 | 6.88E-04 | -4.51E-03 | 1.08E-03 | 3.93E-03 | -5.42E-03 |
| $K21903_{VIP≥0.8\ (+)}$ | 1.043 | 0.63 | 1.143 | 1.467 | 1.111 | 0.432 | 3.81E-04 | -4.25E-04 | 1.82E-03 | 5.03E-03 | 5.26E-03 | -4.33E-05 |
| $K06885_{VIP≥0.8\ (+)}$ | 1.143 | 0.499 | 1.1 | 0.891 | 1.09 | 0.47 | 3.10E-03 | -4.24E-03 | -5.48E-04 | 1.64E-03 | 1.51E-03 | 2.20E-03 |
| $K06177_{VIP≥0.8\ (+)}$ | 0.962 | 0.063 | 0.915 | 1.429 | 2.262 | 0.621 | 6.14E-03 | 2.43E-04 | 5.30E-03 | 1.19E-02 | -1.93E-02 | -2.15E-05 |
| $K11358_{VIP≥0.8\ (+)}$ | 0.999 | 0.468 | 1.2 | 0.936 | 1.053 | 0.759 | 2.34E-03 | 5.35E-04 | 3.37E-03 | -4.31E-03 | 2.73E-03 | -1.22E-03 |
| $K02025_{VIP≥0.8\ (+)}$ | 0.792 | 0.977 | 0.991 | 0.982 | 1.347 | 0.66 | -9.67E-04 | 6.34E-03 | 2.94E-03 | -9.34E-04 | 6.55E-03 | 1.99E-03 |
| $K03183_{VIP≥0.8\ (+)}$ | 0.864 | 0.293 | 1 | 0.797 | 1.217 | 0.95 | 3.21E-03 | 2.51E-03 | -6.53E-04 | 3.32E-04 | -8.21E-03 | 6.40E-03 |
| $K01754_{VIP≥0.8\ (+)}$ | 1.147 | 0.248 | 0.92 | 1.089 | 1.069 | 0.677 | -4.60E-03 | 1.12E-03 | 1.08E-03 | 2.77E-03 | 4.65E-03 | -6.57E-04 |
| $K06188_{VIP≥0.8\ (+)}$ | 1.415 | 0.476 | 1.018 | 1.147 | 1.165 | 0.516 | 6.90E-03 | 7.33E-04 | 1.36E-03 | -2.45E-03 | -8.05E-03 | 2.76E-04 |
| $K03431_{VIP≥0.8\ (+)}$ | 1.19 | 0.74 | 1.017 | 1.224 | 1.058 | 0.459 | -1.03E-04 | 5.01E-04 | -2.10E-03 | 1.53E-03 | 2.63E-03 | 2.06E-03 |

| | | | | | | | | | | | | |
|---|---|---|---|---|---|---|---|---|---|---|---|---|
| K07164$_{VIP\geq 0.8\ (+)}$ | 1.134 | 0.525 | 1.053 | 1.183 | 0.886 | 0.563 | 3.41E-03 | 4.66E-03 | 2.70E-03 | -3.89E-03 | -5.40E-03 | 1.54E-03 |
| K00350$_{VIP\geq 0.8\ (+)}$ | 0.541 | 1.041 | 2.226 | 0.766 | 1.186 | 1.241 | -2.06E-04 | 1.07E-02 | -1.57E-02 | 6.56E-03 | 1.00E-02 | 2.98E-03 |
| K02557$_{VIP\geq 0.8\ (+)}$ | 1 | 1.494 | 0.985 | 0.265 | 0.55 | 1.515 | 6.63E-03 | 1.11E-02 | -6.86E-03 | -2.78E-05 | 2.85E-03 | 1.57E-04 |
| K03060$_{VIP\geq 0.8\ (+)}$ | 1.105 | 0.336 | 0.897 | 1.073 | 1.125 | 0.204 | 7.04E-03 | 3.42E-03 | -5.57E-03 | 1.93E-03 | -1.12E-03 | 1.08E-03 |
| K05807$_{VIP\geq 0.8\ (+)}$ | 0.887 | 0.482 | 1.21 | 0.661 | 0.874 | 1.227 | 2.06E-03 | 5.50E-03 | -4.14E-03 | 2.99E-04 | -6.40E-03 | 6.74E-03 |
| K01719$_{VIP\geq 0.8\ (+)}$ | 0.855 | 0.762 | 1.069 | 0.752 | 1.058 | 1.093 | -1.76E-03 | 9.00E-03 | 2.18E-03 | 3.02E-03 | -6.56E-03 | 7.41E-03 |
| K01952$_{VIP\geq 0.8\ (+)}$ | 0.923 | 1.516 | 0.881 | 1.062 | 0.461 | 0.737 | -1.72E-03 | 9.82E-03 | 4.96E-04 | 2.54E-03 | -2.95E-03 | 5.57E-04 |
| K21908$_{VIP\geq 0.8\ (+)}$ | 1.312 | 0.437 | 1.003 | 2.235 | 1.64 | 0.624 | -8.88E-03 | -2.34E-03 | 6.45E-03 | 1.90E-02 | 1.40E-02 | 1.07E-03 |
| K07322$_{VIP\geq 0.8\ (+)}$ | 1.161 | 1.275 | 0.201 | 0.26 | 0.813 | 2.01 | 7.66E-03 | 1.04E-02 | -1.26E-03 | 2.08E-03 | -6.83E-03 | 1.14E-02 |
| K04043$_{VIP\geq 0.8\ (+)}$ | 0.867 | 1.461 | 0.866 | 1.053 | 0.43 | 0.727 | 2.27E-05 | -8.42E-04 | 1.14E-03 | 1.06E-03 | 4.11E-04 | -2.88E-03 |
| K07076$_{VIP\geq 0.8\ (+)}$ | 1.168 | 0.873 | 0.581 | 0.953 | 1.976 | 0.336 | 6.51E-03 | 9.86E-03 | -1.04E-03 | 7.99E-03 | 1.69E-02 | -2.45E-04 |
| K06113$_{VIP\geq 0.8\ (+)}$ | 1.044 | 0.961 | 0.63 | 1.272 | 0.625 | 1.103 | 5.26E-03 | 8.75E-03 | -7.80E-04 | 1.10E-02 | -6.70E-04 | 6.06E-03 |
| K07460$_{VIP\geq 0.8\ (+)}$ | 0.836 | 0.752 | 0.918 | 1.386 | 0.731 | 1.18 | 1.67E-03 | 6.71E-03 | 2.59E-03 | -8.77E-03 | -5.81E-03 | 5.11E-03 |
| K02029$_{VIP\geq 0.8\ (+)}$ | 1.102 | 0.734 | 1.036 | 1.23 | 1.173 | 0.29 | -1.58E-03 | 4.93E-04 | 1.72E-03 | 1.80E-03 | -6.06E-04 | 4.33E-04 |
| K03559$_{VIP\geq 0.8\ (+)}$ | 0.859 | 0.403 | 1.048 | 0.643 | 1.004 | 1.124 | 6.50E-04 | 3.86E-03 | -1.36E-03 | 1.40E-03 | -8.19E-03 | 7.55E-03 |
| K01666$_{VIP\geq 0.8\ (-)}$ | 1.904 | 1.104 | 0.312 | 1.329 | 0.831 | 2.352 | -1.29E-02 | -1.26E-02 | -1.70E-03 | -1.15E-02 | -6.87E-03 | -1.55E-02 |
| K06934$_{VIP\geq 0.8\ (-)}$ | 1.319 | 1.782 | 1.235 | 0.852 | 0.328 | 0.822 | -6.65E-03 | -2.05E-02 | -4.50E-03 | -5.40E-03 | -1.91E-03 | -3.25E-03 |
| K18700$_{VIP\geq 0.8\ (-)}$ | 1.168 | 1.129 | 1.043 | 1.229 | 0.441 | 1.509 | -5.91E-03 | -1.18E-02 | -2.41E-03 | -7.16E-03 | -3.35E-03 | -9.55E-03 |
| K04095$_{VIP\geq 0.8\ (-)}$ | 0.503 | 1.508 | 1.029 | 1.003 | 0.876 | 1.28 | -2.67E-03 | -1.33E-02 | -7.41E-03 | -7.92E-03 | -1.12E-03 | -3.26E-03 |
| K00975$_{VIP\geq 0.8\ (-)}$ | 1.112 | 0.92 | 1.021 | 1.106 | 1.218 | 0.673 | -1.13E-03 | -6.39E-03 | -2.25E-03 | -1.18E-03 | -1.19E-03 | -3.60E-03 |
| K09779$_{VIP\geq 0.8\ (-)}$ | 0.872 | 0.854 | 0.696 | 0.45 | 1.477 | 1.227 | -4.70E-03 | -1.66E-03 | -3.94E-03 | -2.87E-03 | -1.27E-02 | -6.25E-03 |
| K16787$_{VIP\geq 0.8\ (-)}$ | 0.738 | 0.976 | 0.908 | 0.949 | 1.235 | 0.181 | -1.65E-03 | -5.61E-03 | -1.26E-03 | -5.18E-03 | -1.43E-03 | -1.14E-03 |
| K02072$_{VIP\geq 0.8\ (-)}$ | 1.028 | 0.67 | 0.999 | 1.053 | 1.18 | 0.278 | -1.19E-03 | -1.01E-03 | -2.56E-03 | -3.41E-03 | -4.03E-03 | -9.58E-05 |
| K00798$_{VIP\geq 0.8\ (-)}$ | 1.214 | 0.531 | 1.174 | 1.029 | 1.371 | 0.54 | -2.93E-03 | -2.15E-03 | -1.39E-04 | -1.43E-03 | -1.02E-02 | -2.82E-03 |
| K02073$_{VIP\geq 0.8\ (-)}$ | 1.022 | 0.628 | 1.194 | 1.044 | 1.211 | 0.646 | -3.22E-04 | -3.60E-03 | -6.39E-03 | -5.11E-03 | -6.15E-03 | -2.13E-03 |
| K06207$_{VIP\geq 0.8\ (-)}$ | 0.851 | 1.434 | 0.89 | 0.945 | 0.756 | 0.665 | -8.21E-04 | -1.02E-03 | -3.47E-03 | -1.42E-03 | -4.32E-03 | -1.53E-03 |
| K03545$_{VIP\geq 0.8\ (-)}$ | 0.913 | 1.405 | 0.783 | 0.902 | 1.708 | 0.15 | -4.23E-03 | -9.63E-03 | -4.24E-03 | -5.00E-03 | -1.15E-02 | -3.87E-04 |
| K02759$_{VIP\geq 0.8\ (-)}$ | 0.878 | 1.1 | 0.858 | 1.052 | 0.984 | 1.025 | -4.19E-03 | -2.78E-04 | -4.07E-04 | -6.46E-03 | 1.30E-03 | -1.45E-03 |
| K15771$_{VIP\geq 0.8\ (-)}$ | 0.879 | 1.098 | 1.068 | 0.997 | 1.037 | 2.224 | 4.32E-03 | -1.22E-02 | -6.89E-03 | -8.61E-03 | -8.24E-03 | -1.44E-02 |
| K09474$_{VIP\geq 0.8\ (-)}$ | 1.094 | 0.949 | 1.146 | 0.982 | 1.379 | 0.864 | -6.31E-04 | -9.61E-03 | -1.32E-03 | 1.70E-03 | -7.76E-03 | -4.06E-03 |

| | | | | | | | | | | | | |
|---|---|---|---|---|---|---|---|---|---|---|---|---|
| $K03086_{VIP\geq0.8\ (-)}$ | 1.172 | 0.859 | 1.104 | 1.061 | 1.172 | 0.939 | 2.96E-03 | -5.90E-03 | -1.41E-03 | -4.49E-03 | -3.79E-04 | -5.43E-03 |
| $K03205_{VIP\geq0.8\ (-)}$ | 1.456 | 0.988 | 1.02 | 1.076 | 0.848 | 0.88 | 8.28E-03 | -2.51E-03 | -3.75E-03 | -5.39E-03 | -1.54E-03 | -3.74E-03 |
| $K01091_{VIP\geq0.8\ (-)}$ | 1.195 | 1.421 | 1.476 | 1.05 | 0.903 | 0.958 | -4.35E-03 | -9.57E-03 | -1.00E-02 | 1.56E-03 | -1.66E-03 | -3.72E-03 |
| $K03497_{VIP\geq0.8\ (-)}$ | 0.811 | 1.584 | 0.84 | 1.029 | 1.063 | 1.255 | 3.93E-03 | -1.30E-02 | -3.14E-04 | -3.21E-03 | -5.82E-03 | -7.45E-03 |
| $K02420_{VIP\geq0.8\ (-)}$ | 1.247 | 0.419 | 0.993 | 0.959 | 1.137 | 1.088 | 6.82E-03 | -8.70E-04 | -3.54E-03 | -1.81E-03 | -3.01E-03 | -3.27E-03 |
| $K07192_{VIP\geq0.8\ (-)}$ | 1.088 | 1.698 | 0.794 | 1.02 | 1.326 | 1.065 | -6.05E-03 | -1.98E-02 | 3.77E-03 | -5.77E-03 | -1.13E-02 | -5.30E-03 |
| $K02413_{VIP\geq0.8\ (-)}$ | 1.404 | 0.474 | 1.057 | 1.001 | 1.169 | 1.146 | 7.34E-03 | -1.35E-03 | -9.02E-04 | -3.26E-03 | -3.09E-03 | -4.65E-03 |
| $K02398_{VIP\geq0.8\ (-)}$ | 1.502 | 0.529 | 1 | 0.985 | 1.153 | 1.174 | 8.38E-03 | -1.80E-03 | -3.22E-03 | -4.12E-03 | -2.51E-03 | -3.63E-03 |
| $K22452_{VIP\geq0.8\ (-)}$ | 1.122 | 0.899 | 1.008 | 0.944 | 1.03 | 0.637 | 4.21E-03 | -2.42E-03 | -3.01E-03 | -2.46E-03 | -1.80E-03 | -1.37E-03 |
| $K07054_{VIP\geq0.8\ (-)}$ | 1.403 | 1.216 | 1.175 | 1.044 | 0.939 | 0.732 | -5.91E-03 | -1.12E-02 | -3.74E-04 | -1.80E-03 | 1.86E-03 | -2.70E-03 |
| $K22927_{VIP\geq0.8\ (-)}$ | 0.968 | 0.893 | 0.958 | 0.999 | 1.084 | 0.718 | 1.20E-03 | -3.59E-03 | -3.69E-03 | -1.57E-03 | -5.16E-04 | -4.76E-03 |
| $K08602_{VIP\geq0.8\ (-)}$ | 0.813 | 1.361 | 0.891 | 0.899 | 1.105 | 0.381 | 1.59E-03 | -1.11E-02 | -1.19E-03 | -4.51E-03 | -2.84E-03 | -2.63E-03 |
| $K19271_{VIP\geq0.8\ (-)}$ | 1.226 | 0.878 | 1.046 | 0.921 | 0.527 | 0.878 | -4.06E-03 | -6.10E-03 | 1.75E-03 | -6.40E-04 | -2.51E-03 | -2.64E-03 |
| $K07588_{VIP\geq0.8\ (-)}$ | 1.293 | 0.839 | 1.125 | 0.759 | 0.986 | 1.02 | -4.05E-03 | -9.00E-03 | -4.68E-05 | 1.57E-03 | -1.25E-04 | -6.85E-03 |
| $K15894_{VIP\geq0.8\ (-)}$ | 1.049 | 1.753 | 1.494 | 1.218 | 0.964 | 0.247 | -6.95E-03 | -2.05E-02 | -1.02E-02 | -1.05E-02 | 6.98E-03 | -1.43E-03 |
| $K02574_{VIP\geq0.8\ (-)}$ | 1.861 | 0.475 | 1.287 | 1.041 | 1.251 | 1.819 | -1.08E-02 | -2.92E-03 | -3.73E-03 | 8.81E-03 | -8.67E-03 | -1.07E-02 |
| $K18346_{VIP\geq0.8\ (-)}$ | 0.577 | 0.909 | 1.079 | 0.81 | 1.229 | 1.236 | 3.50E-03 | -1.06E-02 | -7.38E-03 | -6.98E-03 | -1.05E-02 | -7.59E-03 |
| $K03621_{VIP\geq0.8\ (-)}$ | 0.926 | 0.933 | 0.991 | 1.036 | 1.196 | 0.343 | 1.51E-03 | -9.30E-03 | -3.85E-03 | -7.98E-04 | -2.81E-03 | -1.83E-03 |
| $K16785_{VIP\geq0.8\ (-)}$ | 0.881 | 0.977 | 0.917 | 0.876 | 0.96 | 0.24 | -2.59E-04 | -5.18E-03 | -3.24E-04 | -1.75E-03 | 2.14E-03 | -1.53E-03 |
| $K01775_{VIP\geq0.8\ (-)}$ | 0.943 | 0.3 | 0.988 | 0.984 | 1.175 | 0.813 | -1.79E-03 | -1.57E-03 | -3.84E-03 | -1.99E-03 | 2.96E-04 | -5.63E-03 |
| $K02346_{VIP\geq0.8\ (-)}$ | 0.953 | 1.224 | 0.83 | 1.284 | 0.343 | 1.309 | -3.10E-03 | -3.35E-03 | -3.03E-03 | 6.17E-03 | -1.68E-03 | -6.83E-03 |
| $K03581_{VIP\geq0.8\ (-)}$ | 1.027 | 0.818 | 0.958 | 1.121 | 1.23 | 0.792 | -1.40E-04 | -3.89E-03 | -4.16E-04 | -2.01E-04 | 3.06E-03 | -4.85E-03 |
| $K01297_{VIP\geq0.8\ (-)}$ | 1.589 | 0.722 | 1.137 | 1.296 | 0.882 | 0.843 | -8.46E-03 | -5.10E-03 | -1.12E-03 | 1.13E-02 | -2.74E-03 | -2.15E-03 |
| $K11754_{VIP\geq0.8\ (-)}$ | 0.206 | 1.126 | 0.814 | 1.124 | 1.337 | 1.179 | 1.32E-03 | -1.52E-03 | -5.68E-03 | -9.36E-03 | -9.45E-03 | -5.04E-03 |
| $K03655_{VIP\geq0.8\ (-)}$ | 0.954 | 1.216 | 0.943 | 1.066 | 1.122 | 0.747 | 4.45E-03 | -2.48E-04 | -4.99E-03 | -2.25E-04 | -4.59E-03 | -1.15E-03 |
| $K00266_{VIP\geq0.8\ (-)}$ | 1.003 | 1.489 | 1.07 | 0.978 | 1.299 | 0.782 | 3.00E-03 | -3.44E-03 | -8.58E-05 | -5.20E-03 | -8.35E-03 | -3.62E-03 |
| $K22210_{VIP\geq0.8\ (-)}$ | 1.01 | 0.914 | 0.965 | 0.459 | 0.919 | 0.506 | -5.12E-03 | -1.01E-02 | 2.72E-03 | -2.73E-03 | -4.71E-03 | -2.24E-03 |
| $K22300_{VIP\geq0.8\ (-)}$ | 1.083 | 2.131 | 0.589 | 0.676 | 1.434 | 0.809 | -6.72E-03 | -2.52E-02 | 2.24E-03 | -5.21E-03 | -1.22E-02 | -3.15E-03 |
| $K07726_{VIP\geq0.8\ (-)}$ | 1.637 | 1.013 | 0.41 | 0.804 | 1.213 | 0.506 | 1.10E-02 | -1.21E-02 | -2.05E-03 | -6.56E-03 | -7.91E-03 | -3.49E-03 |
| $K16786_{VIP\geq0.8\ (-)}$ | 0.562 | 0.826 | 0.966 | 0.882 | 1.161 | 0.04 | 7.36E-04 | -5.79E-03 | -5.09E-03 | -2.72E-03 | -4.16E-04 | -2.73E-04 |

| | | | | | | | | | | | | |
|---|---|---|---|---|---|---|---|---|---|---|---|---|
| $K01926_{VIP≥0.8\ (-)}$ | 0.955 | 0.249 | 0.482 | 0.986 | 1.14 | 1.269 | -5.62E-03 | -5.84E-04 | -3.94E-04 | 2.69E-03 | -4.96E-03 | -6.69E-03 |
| $K06958_{VIP≥0.8\ (-)}$ | 1.015 | 0.606 | 1.081 | 1.02 | 1.116 | 0.571 | 3.17E-03 | -1.91E-03 | -1.56E-04 | -2.94E-03 | -5.67E-04 | -2.02E-03 |
| $K00566_{VIP≥0.8\ (-)}$ | 1.036 | 0.639 | 1.097 | 1.06 | 1.153 | 0.749 | 5.51E-03 | -6.14E-03 | -5.01E-03 | -8.63E-04 | -2.13E-04 | -4.89E-03 |
| $K23775_{VIP≥0.8\ (-)}$ | 1.981 | 1.386 | 0.875 | 0.354 | 0.785 | 1.198 | -1.25E-02 | -1.29E-02 | 1.49E-03 | -2.19E-04 | -6.72E-03 | -1.53E-03 |
| $K19824_{VIP≥0.8\ (-)}$ | 1.87 | 0.253 | 1.029 | 0.402 | 0.891 | 0.966 | -1.21E-02 | -1.76E-03 | 6.02E-03 | -1.74E-03 | -6.67E-03 | -4.02E-03 |
| $K12340_{VIP≥0.8\ (-)}$ | 1.343 | 0.714 | 1.076 | 1.068 | 1.054 | 0.647 | -7.81E-03 | -8.49E-03 | -2.05E-03 | 9.16E-03 | -3.70E-03 | -1.65E-03 |
| $K03106_{VIP≥0.8\ (-)}$ | 0.514 | 1.344 | 0.724 | 0.948 | 0.95 | 0.813 | 4.41E-04 | -5.56E-03 | -1.39E-03 | -7.03E-04 | -4.20E-03 | -3.64E-04 |
| $K03168_{VIP≥0.8\ (-)}$ | 0.984 | 1.492 | 0.443 | 0.806 | 0.563 | 0.954 | -6.64E-03 | -8.82E-03 | -3.06E-04 | -1.91E-03 | 1.60E-03 | -3.79E-03 |
| $K00075_{VIP≥0.8\ (-)}$ | 0.328 | 1.131 | 0.818 | 1.087 | 1.047 | 0.652 | 1.22E-03 | -4.73E-03 | -8.60E-04 | -9.51E-03 | -3.61E-03 | -3.24E-03 |
| $K02838_{VIP≥0.8\ (-)}$ | 0.846 | 0.798 | 1.203 | 1.171 | 1.094 | 0.492 | 1.23E-03 | -7.71E-03 | -3.59E-03 | -6.06E-03 | -9.38E-03 | -1.95E-03 |
| $K07727_{VIP≥0.8\ (-)}$ | 1.242 | 0.899 | 0.409 | 0.188 | 0.858 | 1.218 | -8.16E-03 | -9.20E-03 | 2.30E-04 | -1.52E-03 | -6.30E-03 | -5.68E-03 |
| $K07270_{VIP≥0.8\ (-)}$ | 1.126 | 2.204 | 1.11 | 1.602 | 1.702 | 1.743 | -5.03E-03 | 2.35E-02 | -2.91E-03 | -7.75E-03 | 1.38E-02 | -1.19E-02 |
| $K07402_{VIP≥0.8\ (-)}$ | 0.801 | 1.274 | 0.912 | 1.271 | 0.816 | 0.942 | 2.80E-03 | -1.27E-02 | -2.11E-03 | 4.08E-03 | -1.61E-03 | -5.63E-03 |
| $K05364_{VIP≥0.8\ (-)}$ | 1.102 | 1.027 | 1.028 | 0.976 | 0.97 | 0.984 | -6.61E-04 | 6.87E-03 | -1.60E-03 | -4.76E-03 | 1.76E-03 | -4.82E-03 |
| $K03303_{VIP≥0.8\ (-)}$ | 1.03 | 0.979 | 0.858 | 1.889 | 0.877 | 1.062 | -4.50E-03 | -1.15E-02 | 1.93E-03 | 1.53E-02 | -1.79E-04 | -7.13E-03 |
| $K04042_{VIP≥0.8\ (-)}$ | 1.178 | 0.887 | 1.212 | 0.998 | 1.042 | 0.912 | 3.58E-03 | -5.66E-03 | -5.74E-03 | -2.68E-03 | 1.46E-03 | -1.49E-04 |
| $K01626_{VIP≥0.8\ (-)}$ | 1.141 | 0.874 | 1.135 | 1.101 | 1.203 | 0.848 | 3.17E-03 | -4.15E-03 | -3.57E-03 | -1.25E-03 | 2.24E-03 | -2.22E-03 |
| $K02036_{VIP≥0.8\ (-)}$ | 1.159 | 1.055 | 1.105 | 0.969 | 1.188 | 0.871 | -5.80E-05 | -1.56E-03 | -9.74E-04 | -4.87E-03 | 6.73E-03 | 8.81E-04 |
| $K01912_{VIP≥0.8\ (-)}$ | 1.278 | 1.306 | 1.064 | 0.826 | 0.827 | 1.395 | -5.10E-03 | -1.29E-02 | 2.63E-03 | 2.75E-03 | -3.66E-03 | -7.60E-03 |
| $K02112_{VIP≥0.8\ (-)}$ | 1.014 | 1.407 | 0.936 | 0.925 | 0.841 | 0.928 | -2.90E-03 | 2.04E-03 | -4.46E-04 | -1.21E-03 | 2.14E-03 | -2.21E-04 |
| $K21023_{VIP≥0.8\ (-)}$ | 1.173 | 1.026 | 0.997 | 1.131 | 1.089 | 0.884 | 4.72E-03 | -3.12E-03 | -1.27E-03 | 7.99E-05 | -4.17E-03 | -4.23E-03 |
| $K13653_{VIP≥0.8\ (-)}$ | 1.088 | 1.302 | 1.324 | 0.634 | 0.867 | 1.862 | -3.33E-03 | -1.17E-02 | 8.80E-03 | 2.48E-04 | -4.25E-03 | -1.08E-02 |
| $K16329_{VIP≥0.8\ (-)}$ | 1.333 | 1.848 | 1.038 | 0.563 | 0.877 | 1.468 | -5.73E-03 | -1.81E-02 | 3.37E-03 | 1.54E-03 | -6.75E-03 | -8.31E-03 |
| $K13018_{VIP≥0.8\ (-)}$ | 1.354 | 1.378 | 1.207 | 0.708 | 0.88 | 1.089 | -4.24E-03 | -9.66E-03 | -1.54E-03 | 4.55E-03 | 6.70E-03 | -4.71E-03 |
| $K23010_{VIP≥0.8\ (-)}$ | 1.483 | 1.411 | 1.011 | 0.856 | 1.081 | 0.477 | -8.55E-03 | -1.56E-02 | -8.64E-04 | 7.30E-03 | 4.61E-03 | -2.20E-03 |
| $K14415_{VIP≥0.8\ (-)}$ | 0.703 | 1.321 | 0.819 | 1.645 | 0.903 | 1.384 | -4.61E-03 | -1.42E-02 | 3.07E-03 | -1.30E-02 | 7.32E-03 | -9.30E-03 |
| $K09516_{VIP≥0.8\ (-)}$ | 1.633 | 0.63 | 0.926 | 1.851 | 1.058 | 1.051 | 8.95E-03 | -7.44E-03 | 4.87E-03 | -1.18E-02 | -9.06E-03 | -7.20E-03 |
| $K01255_{VIP≥0.8\ (-)}$ | 1.008 | 1.165 | 1.423 | 0.685 | 1.183 | 0.999 | 4.25E-03 | -8.98E-03 | -9.11E-03 | -4.27E-03 | 5.54E-03 | -4.52E-03 |
| $K09707_{VIP≥0.8\ (-)}$ | 1.402 | 0.963 | 0.983 | 1.364 | 1.333 | 0.797 | -8.64E-03 | -7.81E-03 | 4.26E-03 | -1.02E-02 | -1.01E-02 | 3.79E-03 |
| $K03775_{VIP≥0.8\ (-)}$ | 0.862 | 1.594 | 1.179 | 0.379 | 2.978 | 0.874 | -3.55E-03 | -1.70E-02 | -5.27E-03 | 2.36E-03 | -2.55E-02 | 6.02E-03 |

| | | | | | | | | | | | | |
|---|---|---|---|---|---|---|---|---|---|---|---|---|
| K00965$_{VIP≥0.8\ (-)}$ | 0.797 | 1.542 | 0.895 | 0.928 | 0.935 | 0.951 | 4.59E-04 | 1.83E-02 | -2.68E-03 | -4.64E-03 | -2.32E-03 | -4.25E-03 |
| K01698$_{VIP≥0.8\ (-)}$ | 0.823 | 1.089 | 0.853 | 0.995 | 0.849 | 0.758 | 3.56E-03 | -1.82E-03 | -2.43E-03 | -2.14E-04 | 4.87E-03 | -1.08E-03 |
| K09807$_{VIP≥0.8\ (-)}$ | 1.098 | 0.792 | 1.043 | 1.036 | 1.089 | 0.9 | 4.00E-03 | 3.92E-03 | -1.41E-03 | -1.40E-03 | -1.57E-04 | -2.20E-03 |
| K00058$_{VIP≥0.8\ (-)}$ | 0.958 | 0.901 | 1.11 | 1.006 | 1.788 | 0.612 | 1.45E-03 | -9.16E-04 | -5.39E-03 | -4.67E-03 | 1.26E-02 | -3.61E-03 |
| K06215$_{VIP≥0.8\ (-)}$ | 1.577 | 1.665 | 0.697 | 1.126 | 0.823 | 1.79 | 1.02E-02 | -1.37E-02 | -1.21E-03 | -9.86E-03 | -3.78E-03 | 1.09E-02 |
| K04083$_{VIP≥0.8\ (-)}$ | 1.182 | 0.677 | 1.104 | 1.055 | 1.192 | 0.878 | 4.15E-03 | -1.83E-03 | -2.22E-03 | -2.36E-03 | 1.77E-03 | -5.72E-03 |
| K03500$_{VIP≥0.8\ (-)}$ | 1.123 | 0.63 | 1.342 | 1.056 | 1.191 | 1.132 | 3.77E-03 | -1.75E-03 | -7.61E-03 | -6.02E-04 | 2.87E-03 | -6.53E-03 |
| K04773$_{VIP≥0.8\ (-)}$ | 1.042 | 0.822 | 0.59 | 0.894 | 1.053 | 1.118 | 5.03E-03 | -6.93E-03 | 8.12E-04 | -7.37E-03 | -8.34E-03 | -1.08E-03 |
| K00265$_{VIP≥0.8\ (-)}$ | 1.286 | 1.359 | 0.892 | 0.885 | 0.609 | 1.096 | 4.67E-03 | -4.20E-03 | -1.13E-03 | -2.27E-03 | 2.49E-03 | -4.37E-03 |
| K06199$_{VIP≥0.8\ (-)}$ | 1.14 | 0.714 | 1.102 | 0.857 | 1.169 | 1.608 | 2.21E-03 | -3.81E-04 | -1.82E-06 | -3.30E-03 | -3.27E-03 | 7.91E-03 |
| K05837$_{VIP≥0.8\ (-)}$ | 0.848 | 0.856 | 1.153 | 1.151 | 0.328 | 1.625 | 3.27E-03 | -6.81E-03 | -6.72E-03 | -9.48E-03 | -2.81E-03 | 7.11E-03 |
| K01262$_{VIP≥0.8\ (-)}$ | 1.454 | 2 | 1.074 | 0.873 | 0.645 | 1.278 | -9.81E-03 | -1.95E-02 | -7.66E-03 | 3.83E-03 | 4.84E-03 | -1.82E-03 |
| K03046$_{VIP≥0.8\ (-)}$ | 1.057 | 1.316 | 0.817 | 1.161 | 0.578 | 0.939 | -2.61E-03 | -1.45E-03 | -2.48E-03 | 2.51E-03 | 4.28E-04 | -3.82E-03 |
| K03286$_{VIP≥0.8\ (-)}$ | 1.192 | 1.029 | 1.458 | 0.627 | 1.184 | 0.832 | -4.44E-03 | -1.06E-02 | -6.83E-03 | 4.46E-03 | 8.02E-03 | -4.23E-03 |
| K03502$_{VIP≥0.8\ (-)}$ | 1.649 | 1.223 | 0.797 | 1.007 | 0.968 | 0.919 | 1.12E-02 | -5.82E-03 | 9.06E-04 | -5.26E-03 | -4.06E-03 | -1.14E-03 |
| K02483$_{VIP≥0.8\ (-)}$ | 1.662 | 1.051 | 1.034 | 1.032 | 1.186 | 0.558 | 9.01E-03 | 6.58E-03 | -3.70E-03 | -3.95E-03 | -2.83E-04 | -1.00E-03 |
| K03310$_{VIP≥0.8\ (-)}$ | 0.703 | 1.412 | 0.898 | 0.909 | 1.509 | 1.145 | -6.52E-04 | 2.14E-03 | 1.29E-03 | -7.22E-03 | -8.94E-03 | -8.05E-04 |
| K01153$_{VIP≥0.8\ (-)}$ | 1.011 | 1.161 | 0.68 | 0.914 | 0.996 | 0.955 | -4.57E-03 | -1.94E-04 | -3.36E-03 | -5.03E-03 | 2.87E-03 | 2.92E-05 |
| K09764$_{VIP≥0.8\ (-)}$ | 0.962 | 1.167 | 0.81 | 0.484 | 0.878 | 0.618 | -2.12E-03 | -2.75E-03 | 9.71E-04 | 3.11E-03 | -6.55E-03 | -1.44E-04 |
| K01118$_{VIP≥0.8\ (-)}$ | 0.976 | 1.147 | 0.914 | 0.691 | 0.106 | 1.856 | 1.85E-03 | -5.22E-03 | -1.29E-03 | -1.31E-03 | 6.98E-05 | -1.09E-02 |
| K06406$_{VIP≥0.8\ (-)}$ | 0.954 | 0.459 | 0.508 | 0.801 | 1.881 | 1.232 | -6.46E-03 | 3.16E-03 | 2.57E-03 | -6.66E-03 | -1.60E-02 | -7.50E-03 |
| K11621$_{VIP≥0.8\ (-)}$ | 0.793 | 0.129 | 1.207 | 1.852 | 1.115 | 1.055 | -4.02E-03 | -1.45E-03 | 8.68E-03 | -1.55E-02 | 9.47E-03 | -1.95E-03 |
| K06407$_{VIP≥0.8\ (-)}$ | 0.926 | 0.795 | 0.832 | 0.195 | 1.405 | 1.359 | -5.73E-03 | 7.35E-04 | -5.20E-03 | 6.96E-04 | -1.20E-02 | -7.18E-03 |
| K01085$_{VIP≥0.8\ (-)}$ | 1.015 | 1.027 | 0.938 | 0.668 | 2.41 | 0.464 | -5.88E-04 | -7.60E-03 | 5.54E-03 | -9.18E-04 | -2.06E-02 | 1.28E-03 |
| K23004$_{VIP≥0.8\ (-)}$ | 1.065 | 0.97 | 1.156 | 0.693 | 1.645 | 0.471 | -2.68E-03 | 1.01E-02 | -2.86E-03 | -4.36E-04 | -1.34E-02 | 3.25E-03 |
| K01267$_{VIP≥0.8\ (-)}$ | 1.296 | 0.561 | 1.777 | 1.35 | 1.187 | 0.12 | -6.15E-03 | -2.12E-03 | 1.04E-02 | 6.08E-03 | -1.77E-03 | -3.06E-04 |
| K11941$_{VIP≥0.8\ (-)}$ | 0.923 | 0.614 | 1.487 | 0.898 | 0.155 | 1.364 | -6.00E-03 | 5.34E-04 | -9.66E-03 | 7.24E-03 | -1.28E-03 | -7.33E-03 |
| K00974$_{VIP≥0.8\ (-)}$ | 1.36 | 0.935 | 1.521 | 0.786 | 1.059 | 0.637 | 8.40E-03 | -1.03E-02 | -1.02E-02 | -2.15E-03 | 6.53E-03 | -3.88E-03 |
| K03321$_{VIP≥0.8\ (-)}$ | 1.875 | 0.73 | 1.013 | 0.744 | 0.945 | 0.98 | -1.13E-02 | -1.43E-03 | 3.46E-03 | 5.79E-04 | -1.47E-03 | -5.83E-03 |
| K00980$_{VIP≥0.8\ (-)}$ | 0.508 | 0.464 | 1.43 | 1.234 | 1.037 | 0.835 | -7.79E-04 | -1.50E-03 | 9.87E-03 | 5.80E-03 | -4.55E-03 | -1.84E-03 |

| | | | | | | | | | | | | |
|---|---|---|---|---|---|---|---|---|---|---|---|---|
| $K07335_{VIP\geq0.8\ (-)}$ | 0.826 | 0.589 | 0.937 | 0.917 | 0.999 | 0.623 | 6.98E-05 | -2.43E-03 | 1.08E-03 | -4.18E-03 | -3.91E-03 | -3.45E-03 |
| $K00995_{VIP\geq0.8\ (-)}$ | 1.248 | 0.454 | 1.023 | 1.037 | 1.173 | 0.211 | 7.19E-03 | -4.13E-03 | -4.23E-03 | -5.20E-04 | 2.23E-05 | -9.21E-04 |
| $K03118_{VIP\geq0.8\ (-)}$ | 1.221 | 0.765 | 1.02 | 0.812 | 1.205 | 0.388 | -3.53E-03 | -6.87E-03 | 1.85E-03 | 6.36E-03 | -7.56E-03 | -1.28E-03 |
| $K01726_{VIP\geq0.8\ (-)}$ | 0.938 | 0.537 | 1.196 | 1.188 | 1.148 | 0.324 | -1.66E-04 | 3.72E-03 | -3.36E-03 | -4.75E-03 | 5.98E-03 | -1.84E-03 |
| $K03634_{VIP\geq0.8\ (-)}$ | 1.339 | 0.26 | 1.007 | 0.875 | 1.381 | 0.658 | 8.08E-03 | -3.03E-03 | -3.98E-03 | -4.75E-03 | -1.18E-02 | 3.40E-03 |
| $K05808_{VIP\geq0.8\ (-)}$ | 1.056 | 0.417 | 1.116 | 1.16 | 1.218 | 0.327 | 3.87E-03 | -3.34E-04 | -5.54E-03 | 8.93E-04 | -6.99E-04 | -1.12E-03 |
| $K03784_{VIP\geq0.8\ (-)}$ | 1.556 | 1.358 | 1.38 | 1.541 | 0.787 | 0.227 | -9.51E-03 | -1.43E-02 | 9.21E-03 | -8.58E-03 | -5.88E-03 | 1.57E-03 |
| $K21064_{VIP\geq0.8\ (-)}$ | 0.845 | 0.48 | 1.852 | 0.855 | 1.537 | 0.336 | -2.71E-03 | 5.44E-03 | 1.10E-02 | -6.48E-03 | -1.15E-02 | -5.54E-04 |
| $K03614_{VIP\geq0.8\ (-)}$ | 1.058 | 0.492 | 0.93 | 0.846 | 0.457 | 1.354 | -4.41E-03 | -2.09E-03 | -1.35E-03 | 7.39E-03 | 1.17E-03 | -7.90E-03 |
| $K11720_{VIP\geq0.8\ (-)}$ | 0.945 | 0.574 | 1.09 | 0.858 | 0.607 | 1.279 | 4.30E-03 | -4.30E-03 | -4.94E-03 | -6.75E-03 | -5.14E-03 | 5.15E-03 |
| $K15738_{VIP\geq0.8\ (-)}$ | 1.108 | 1.29 | 0.827 | 0.701 | 0.599 | 1.518 | -5.83E-03 | 8.06E-04 | 1.94E-03 | -1.76E-03 | -3.22E-03 | -3.00E-03 |
| $K20509_{VIP\geq0.8\ (-)}$ | 1.756 | 0.591 | 0.728 | 1.726 | 0.823 | 1.226 | -1.16E-02 | -6.99E-03 | 3.10E-03 | 1.43E-02 | -4.89E-03 | -8.09E-03 |
| $K07058_{VIP\geq0.8\ (-)}$ | 0.661 | 0.257 | 1.089 | 1.205 | 0.838 | 0.819 | -2.28E-03 | 7.25E-04 | -4.01E-03 | -8.59E-03 | -6.57E-03 | 1.62E-03 |
| $K06919_{VIP\geq0.8\ (-)}$ | 1.159 | 0.901 | 0.897 | 1.415 | 0.518 | 0.782 | -7.79E-03 | -1.07E-02 | 6.53E-03 | 8.73E-03 | -4.27E-03 | -4.38E-03 |
| $K09774_{VIP\geq0.8\ (-)}$ | 1.567 | 1.037 | 1.006 | 0.685 | 0.343 | 1.184 | 9.66E-03 | 2.47E-03 | -6.62E-03 | -5.98E-03 | -2.84E-03 | -7.57E-04 |
| $K03975_{VIP\geq0.8\ (-)}$ | 1.059 | 0.684 | 1.518 | 0.833 | 0.203 | 1.241 | 4.63E-03 | -6.10E-03 | -7.92E-03 | -6.06E-03 | -1.68E-03 | 1.73E-03 |
| $K19353_{VIP\geq0.8\ (-)}$ | 0.802 | 1.261 | 0.142 | 1.06 | 0.036 | 1.302 | -5.09E-03 | -1.02E-02 | -7.89E-04 | 6.22E-03 | 2.23E-04 | -2.28E-03 |
| $K04771_{VIP\geq0.8\ (-)}$ | 0.124 | 1.26 | 0.522 | 0.835 | 2.27 | 0.895 | 4.47E-04 | -8.40E-03 | -3.69E-03 | 3.61E-03 | -1.95E-02 | -5.32E-03 |
| $K03613_{VIP\geq0.8\ (-)}$ | 0.949 | 1.018 | 1.113 | 0.786 | 0.273 | 0.942 | -7.00E-04 | -1.18E-02 | -4.56E-03 | 5.69E-03 | 6.10E-04 | -3.65E-03 |
| $K00962_{VIP\geq0.8\ (-)}$ | 0.817 | 1.194 | 0.815 | 0.939 | 0.41 | 0.502 | 2.45E-03 | -1.95E-03 | -1.50E-04 | -1.85E-03 | 1.33E-03 | -6.88E-04 |
| $K00645_{VIP\geq0.8\ (-)}$ | 1.512 | 0.66 | 1.003 | 0.523 | 1.79 | 1.321 | -1.01E-02 | 5.54E-03 | -6.40E-03 | -1.09E-03 | -1.49E-02 | 7.51E-03 |
| $K01005_{VIP\geq0.8\ (-)}$ | 0.829 | 0.717 | 1.044 | 0.961 | 1.168 | 0.509 | 1.16E-03 | -4.18E-04 | 1.77E-03 | -1.02E-03 | -3.72E-03 | -3.36E-03 |
| $K06133_{VIP\geq0.8\ (-)}$ | 1.062 | 1.171 | 0.846 | 0.887 | 0.471 | 0.617 | -4.90E-03 | -1.39E-02 | -1.49E-03 | 6.08E-03 | 4.03E-03 | -2.93E-03 |
| $K15921_{VIP\geq0.8\ (-)}$ | 1.116 | 0.583 | 1.113 | 0.857 | 1.076 | 0.649 | -2.17E-03 | -6.37E-03 | -5.13E-05 | 5.52E-03 | 4.36E-03 | -4.39E-03 |
| $K08640_{VIP\geq0.8\ (-)}$ | 1.074 | 0.616 | 0.836 | 1.12 | 1.035 | 0.355 | -3.22E-03 | -6.38E-03 | 3.36E-03 | -4.29E-03 | -8.86E-03 | 2.09E-03 |
| $K07483_{VIP\geq0.8\ (-)}$ | 0.891 | 1.436 | 0.856 | 0.226 | 1.231 | 0.472 | -3.93E-03 | -1.54E-02 | 3.60E-03 | -1.93E-03 | -1.06E-02 | 1.70E-03 |
| $K03427_{VIP\geq0.8\ (-)}$ | 0.889 | 1.124 | 0.634 | 0.792 | 0.847 | 1.113 | 2.13E-03 | -2.53E-03 | -3.51E-03 | -1.43E-03 | -2.30E-03 | 1.44E-03 |
| $K03658_{VIP\geq0.8}$ | 0.805 | 0.937 | 0.949 | 0.813 | 1.137 | 1.068 | 5.39E-03 | -8.98E-03 | -6.58E-03 | 6.67E-04 | 6.25E-03 | -1.39E-03 |
| $K04062_{VIP\geq0.8}$ | 0.901 | 1.023 | 1.919 | 2.179 | 1.334 | 1.436 | -5.93E-03 | 5.69E-03 | 1.40E-02 | 1.77E-02 | -9.00E-03 | -9.30E-03 |
| $K03741_{VIP\geq0.8}$ | 1.183 | 1.257 | 1.307 | 0.835 | 0.916 | 1.09 | 2.59E-04 | -7.67E-03 | 3.33E-03 | -2.94E-03 | -1.98E-04 | 1.77E-03 |

| | | | | | | | | | | | | |
|---|---|---|---|---|---|---|---|---|---|---|---|---|
| $K07404_{VIP≥0.8}$ | 1.1 | 1.096 | 0.867 | 1.089 | 1.339 | 0.814 | -2.95E-03 | -6.86E-03 | -2.78E-03 | 4.59E-03 | 9.67E-03 | 3.29E-03 |
| $K20866_{VIP≥0.8}$ | 0.957 | 1.031 | 1.257 | 0.983 | 1.643 | 1.339 | 3.77E-03 | -6.23E-03 | 4.83E-03 | -8.16E-03 | -1.18E-02 | 7.59E-03 |
| $K19117_{VIP≥0.8}$ | 1.221 | 1.07 | 1.049 | 1.002 | 1.075 | 1.312 | 4.45E-03 | 3.52E-03 | -8.49E-04 | -1.78E-03 | -3.08E-03 | 7.67E-03 |
| $K19119_{VIP≥0.8}$ | 1.406 | 0.95 | 1.06 | 0.999 | 1.204 | 0.832 | 6.92E-03 | 4.56E-03 | -1.64E-03 | -3.96E-03 | -3.88E-03 | 1.17E-03 |
| $K02039_{VIP≥0.8}$ | 1.178 | 1.01 | 1.117 | 1.001 | 1.239 | 0.919 | -5.23E-04 | -9.36E-04 | 4.78E-04 | -3.38E-03 | 8.27E-03 | 4.27E-03 |
| $K19118_{VIP≥0.8}$ | 1.396 | 0.989 | 1.033 | 1.005 | 1.104 | 1.085 | 6.79E-03 | 3.69E-03 | -1.06E-03 | -2.10E-03 | -3.02E-03 | 5.23E-03 |
| $K19159_{VIP≥0.8}$ | 1.17 | 1.254 | 1.099 | 0.904 | 0.927 | 1.078 | 6.91E-03 | -9.62E-03 | -6.49E-03 | -1.53E-03 | 1.15E-03 | 6.25E-04 |
| $K02038_{VIP≥0.8}$ | 1.185 | 0.97 | 1.088 | 1.037 | 1.265 | 0.803 | 5.63E-04 | -2.83E-03 | -2.26E-03 | -2.07E-03 | 7.95E-03 | 1.79E-03 |
| $K02614_{VIP≥0.8}$ | 1.307 | 1.529 | 1.044 | 0.914 | 1.134 | 0.913 | -5.16E-03 | -1.61E-02 | 2.23E-03 | 1.92E-03 | 6.45E-03 | -2.89E-03 |
| $K03704_{VIP≥0.8}$ | 1.38 | 0.862 | 1.169 | 0.977 | 1.213 | 1.016 | 7.12E-03 | -1.44E-03 | -5.21E-03 | -1.83E-03 | 3.21E-03 | 4.64E-03 |
| $K00937_{VIP≥0.8}$ | 1.279 | 0.886 | 1.111 | 1.017 | 1.095 | 0.891 | 2.95E-03 | 2.35E-03 | 1.01E-03 | -3.52E-03 | -3.74E-03 | -1.03E-03 |
| $K01524_{VIP≥0.8}$ | 1.252 | 1.237 | 1.108 | 1.132 | 1.083 | 1.144 | 2.97E-03 | 3.45E-03 | -4.81E-04 | -6.27E-04 | -5.82E-04 | 3.10E-03 |
| $K09922_{VIP≥0.8}$ | 1.416 | 1.337 | 1.058 | 1.394 | 0.909 | 1.27 | 7.09E-03 | -1.57E-02 | -2.03E-03 | -7.18E-03 | 6.17E-03 | 8.62E-03 |
| $K03778_{VIP≥0.8}$ | 1.233 | 1.235 | 0.968 | 0.968 | 1.001 | 0.912 | 3.81E-03 | -1.08E-02 | 2.20E-04 | -2.01E-03 | -7.29E-03 | 2.63E-03 |
| $K00053_{VIP≥0.8}$ | 0.969 | 1.46 | 0.962 | 0.828 | 1.822 | 0.933 | -2.85E-03 | 6.13E-03 | -4.82E-04 | -2.45E-03 | 1.47E-02 | 2.04E-03 |
| $K18843_{VIP≥0.8}$ | 1.086 | 0.857 | 1.095 | 1.057 | 0.956 | 0.918 | 1.86E-03 | 3.05E-03 | -3.47E-03 | -1.45E-03 | 3.52E-04 | -7.67E-04 |
| $K19158_{VIP≥0.8}$ | 2.196 | 1.404 | 0.998 | 1.121 | 0.824 | 1.36 | 1.35E-02 | 1.00E-02 | -6.00E-03 | -5.98E-03 | -4.30E-03 | 7.90E-03 |
| $K07473_{VIP≥0.8}$ | 1.348 | 0.894 | 1.081 | 1.011 | 1.346 | 1.013 | 8.13E-03 | -4.08E-03 | -5.43E-03 | -2.18E-05 | 6.41E-03 | 3.07E-03 |
| $K07485_{VIP≥0.8}$ | 1.361 | 1.016 | 1.21 | 1.026 | 1.171 | 1.174 | 6.34E-03 | 4.41E-03 | -7.03E-03 | -5.63E-03 | 2.56E-03 | -1.32E-03 |
| $K02173_{VIP≥0.8}$ | 0.922 | 1.544 | 1.268 | 1.729 | 1.319 | 0.315 | -5.71E-03 | 1.77E-02 | 9.24E-03 | -1.51E-02 | -1.03E-02 | 2.15E-03 |
| $K13566_{VIP≥0.8}$ | 0.813 | 1.157 | 1.158 | 1.894 | 1.418 | 0.722 | -4.00E-03 | -1.17E-02 | 7.30E-03 | 1.46E-02 | 1.18E-02 | -1.83E-03 |
| $K05937_{VIP≥0.8}$ | 0.598 | 1.968 | 0.852 | 1.09 | 1.165 | 1.071 | -2.70E-03 | -2.27E-02 | 5.78E-03 | 8.29E-03 | -8.97E-03 | 5.49E-03 |
| $K21556_{VIP≥0.8}$ | 1.034 | 0.589 | 1.226 | 1.32 | 1.24 | 1.2 | -5.19E-03 | 3.13E-03 | 8.11E-03 | 1.15E-02 | -3.43E-03 | -6.37E-03 |
| $K02770_{VIP≥0.8}$ | 0.826 | 1.156 | 1.274 | 0.817 | 0.615 | 0.884 | 1.79E-03 | 7.86E-03 | -7.41E-03 | -1.28E-03 | 1.93E-05 | -3.72E-03 |
| $K04047_{VIP≥0.8}$ | 0.646 | 0.997 | 0.954 | 1.112 | 0.979 | 1.308 | -5.53E-04 | -4.55E-03 | 2.14E-04 | -8.01E-03 | 3.43E-03 | 7.07E-03 |
| $K01201_{VIP≥0.8}$ | 1.369 | 0.45 | 0.884 | 1.869 | 0.942 | 1.618 | -9.17E-03 | -2.79E-03 | 6.29E-03 | 1.45E-02 | -7.97E-03 | 1.10E-02 |
| $K07045_{VIP≥0.8}$ | 1.427 | 0.834 | 1.185 | 1.218 | 0.64 | 1.639 | -8.40E-03 | 4.23E-03 | 7.73E-03 | 1.07E-02 | -7.64E-04 | -9.10E-03 |
| $K02492_{VIP≥0.8}$ | 0.864 | 1.228 | 0.854 | 1.024 | 0.719 | 1.093 | 5.12E-03 | -2.28E-03 | -3.59E-03 | 4.60E-04 | 5.28E-03 | -2.65E-03 |
| $K00626_{VIP≥0.8}$ | 1.041 | 1.053 | 1.124 | 0.884 | 0.917 | 0.381 | -2.19E-03 | -8.11E-04 | 1.52E-03 | -7.33E-04 | 1.17E-03 | 2.15E-03 |
| $K16511_{VIP≥0.8}$ | 1.206 | 0.83 | 1.01 | 0.831 | 0.893 | 0.306 | 4.81E-03 | 5.19E-03 | -3.81E-03 | -3.20E-03 | 4.29E-04 | -1.88E-03 |

| | | | | | | | | | | | | |
|---|---|---|---|---|---|---|---|---|---|---|---|---|
| $K02031_{VIP≥0.8}$ | 0.876 | 1.29 | 0.861 | 0.915 | 1.266 | 0.528 | -1.85E-03 | -9.06E-03 | 5.65E-04 | -1.95E-03 | 5.01E-03 | 2.75E-03 |
| $K21395_{VIP≥0.8}$ | 1.07 | 0.645 | 0.802 | 0.985 | 1.011 | 0.864 | -4.03E-03 | 3.65E-03 | 3.04E-03 | -1.14E-03 | 2.78E-03 | -4.22E-03 |
| $K02805_{VIP≥0.8}$ | 0.885 | 2.433 | 0.667 | 1.194 | 0.944 | 1.665 | -5.99E-03 | -2.71E-02 | 2.70E-03 | -1.02E-02 | 1.83E-03 | 1.04E-02 |
| $K00991_{VIP≥0.8}$ | 1.019 | 0.87 | 0.958 | 1.105 | 1.302 | 0.494 | -3.83E-03 | 8.26E-03 | 4.61E-04 | 4.32E-03 | -8.58E-03 | -2.60E-03 |
| $K01139_{VIP≥0.8}$ | 0.852 | 1.29 | 1.074 | 1.018 | 1.164 | 0.606 | 2.89E-03 | 1.09E-02 | -5.16E-03 | -4.58E-03 | 6.86E-04 | -1.08E-03 |
| $K00567_{VIP≥0.8}$ | 1 | 0.804 | 0.952 | 1.332 | 0.823 | 0.731 | -2.12E-03 | -4.09E-03 | -5.61E-04 | 3.44E-03 | 2.12E-03 | 2.21E-03 |
| $K23351_{VIP≥0.8}$ | 0.925 | 1.307 | 0.901 | 0.66 | 1.287 | 1.281 | 1.97E-03 | -1.47E-02 | 3.72E-04 | -5.27E-03 | -1.01E-02 | 7.29E-03 |
| $K03973_{VIP≥0.8}$ | 1.186 | 0.764 | 1.089 | 0.804 | 1.046 | 1.57 | -2.74E-03 | -4.39E-03 | 5.82E-04 | 4.20E-03 | -6.41E-03 | 1.04E-02 |
| $K00796_{VIP≥0.8}$ | 0.149 | 1.206 | 1.213 | 1.409 | 0.901 | 1.49 | 1.01E-03 | 4.78E-03 | -7.91E-03 | -1.20E-02 | -6.72E-03 | 4.49E-03 |
| $K11752_{VIP≥0.8}$ | 2.485 | 1.207 | 1.429 | 0.51 | 1.306 | 1.554 | 1.68E-02 | 4.77E-04 | -1.03E-02 | -2.85E-03 | -8.82E-03 | 5.39E-03 |
| $K01495_{VIP≥0.8}$ | 0.937 | 0.73 | 0.897 | 0.83 | 1.009 | 1.059 | -1.81E-03 | 6.34E-03 | 1.26E-03 | -2.60E-03 | -8.44E-03 | 7.28E-03 |
| $K02519_{VIP≥0.8}$ | 0.771 | 1.186 | 0.84 | 0.902 | 0.828 | 1.008 | -1.56E-03 | 5.32E-03 | 2.04E-04 | -4.83E-04 | 4.87E-03 | -5.71E-03 |
| $K19005_{VIP≥0.8}$ | 1.304 | 0.291 | 0.974 | 1.046 | 1.311 | 1.01 | -6.12E-03 | 3.23E-03 | -2.36E-03 | 9.15E-03 | 9.32E-03 | -6.86E-03 |
| $K01934_{VIP≥0.8}$ | 0.517 | 0.961 | 1.086 | 1.164 | 1.666 | 1.282 | 2.72E-03 | -2.13E-04 | 6.20E-03 | -9.12E-03 | -9.97E-03 | 8.09E-03 |
| $K01662_{VIP≥0.8}$ | 0.949 | 1.21 | 0.894 | 0.963 | 0.495 | 0.969 | 4.46E-03 | 3.20E-03 | -7.67E-04 | -7.53E-03 | 2.33E-03 | -5.12E-03 |
| $K01258_{VIP≥0.8}$ | 0.984 | 1.442 | 1.038 | 0.927 | 0.786 | 1.214 | 1.54E-03 | -1.54E-03 | 1.53E-03 | -3.81E-04 | -1.02E-03 | 3.24E-03 |
| $K04487_{VIP≥0.8}$ | 1.036 | 0.901 | 0.98 | 1.242 | 1.111 | 0.437 | 1.07E-03 | -6.02E-03 | -5.00E-04 | 2.11E-03 | 9.70E-05 | -7.96E-04 |
| $K01154_{VIP≥0.8}$ | 1.025 | 1.398 | 0.781 | 1.008 | 0.956 | 1.087 | 4.26E-03 | -5.71E-03 | -4.05E-03 | -6.24E-03 | 2.12E-03 | 4.41E-03 |
| $K07126_{VIP≥0.8}$ | 1.303 | 1.385 | 1.235 | 0.786 | 0.936 | 1.353 | 7.00E-03 | -6.28E-03 | -7.98E-03 | -2.04E-03 | 3.90E-03 | 1.65E-03 |
| $K13599_{VIP≥0.8}$ | 1.176 | 1.255 | 1.002 | 0.526 | 0.518 | 0.948 | -1.90E-03 | -1.10E-03 | 8.39E-04 | 3.80E-03 | -3.09E-03 | 3.43E-03 |
| $K23356_{VIP≥0.8}$ | 0.949 | 1.005 | 0.568 | 1.509 | 1.509 | 0.176 | -5.66E-03 | -5.36E-03 | -3.75E-03 | 1.16E-02 | 1.29E-02 | 8.44E-04 |
| $K11936_{VIP≥0.8}$ | 1.079 | 1.21 | 0.495 | 1.208 | 0.654 | 1.258 | -7.30E-03 | 5.42E-03 | -2.86E-03 | 7.05E-03 | -1.20E-03 | 4.41E-03 |
| $K07248_{VIP≥0.8}$ | 1.054 | 0.533 | 2.111 | 0.7 | 1.048 | 1.007 | 5.08E-03 | 6.33E-03 | -1.32E-02 | -3.21E-03 | 8.85E-03 | -6.71E-03 |
| $K01679_{VIP≥0.8}$ | 0.801 | 0.724 | 1.287 | 1.317 | 0.963 | 0.438 | 4.20E-03 | 4.72E-03 | 7.25E-03 | -1.14E-02 | -4.16E-03 | -2.80E-03 |
| $K19048_{VIP≥0.8}$ | 0.779 | 1.099 | 0.916 | 1.214 | 0.936 | 0.721 | 4.72E-04 | -6.97E-03 | 3.23E-03 | 4.85E-03 | -3.43E-03 | -2.92E-03 |
| $K01496_{VIP≥0.8}$ | 1.262 | 0.532 | 1.027 | 2.503 | 0.426 | 0.856 | -8.48E-03 | -1.76E-03 | 6.39E-03 | 1.97E-02 | 2.80E-03 | -1.02E-03 |
| $K01646_{VIP≥0.8}$ | 0.967 | 1.217 | 1.599 | 0.953 | 0.513 | 0.769 | 6.33E-03 | 1.42E-02 | -1.13E-02 | -7.73E-03 | -2.67E-03 | 5.01E-03 |
| $K04088_{VIP≥0.8}$ | 0.849 | 1.159 | 1.09 | 0.576 | 1.023 | 0.665 | 4.13E-03 | -1.36E-02 | -7.29E-03 | -2.36E-03 | 6.19E-03 | 1.25E-03 |
| $K05305_{VIP≥0.8}$ | 1.048 | 0.963 | 0.933 | 0.524 | 0.528 | 2.133 | -6.29E-03 | -9.55E-03 | 5.91E-03 | 1.29E-03 | -3.05E-03 | 1.39E-02 |
| $K07080_{VIP≥0.8}$ | 0.854 | 0.711 | 0.915 | 0.959 | 0.897 | 0.039 | -2.62E-03 | 1.94E-03 | -5.61E-03 | -1.12E-03 | 1.54E-03 | 2.26E-04 |

| | | | | | | | | | | | | |
|---|---|---|---|---|---|---|---|---|---|---|---|---|
| K00275$_{VIP\geq0.8}$ | 1.255 | 0.334 | 1.331 | 0.771 | 1.137 | 1.035 | 8.30E-03 | -3.12E-03 | -9.57E-03 | 9.98E-05 | 4.40E-04 | -2.46E-03 |
| K05794$_{VIP\geq0.8}$ | 2.277 | 0.809 | 0.959 | 1.022 | 0.705 | 0.282 | -1.47E-02 | 3.12E-03 | 3.37E-03 | -5.01E-03 | 4.34E-03 | -7.93E-04 |
| K02283$_{VIP\geq0.8}$ | 1.237 | 0.642 | 1.025 | 0.899 | 1.002 | 0.485 | 6.10E-03 | -2.74E-05 | -8.23E-04 | 2.41E-04 | 3.87E-03 | -2.94E-03 |
| K10117$_{VIP\geq0.8}$ | 0.834 | 1.115 | 1.141 | 0.891 | 0.735 | 0.283 | 3.22E-04 | -3.24E-03 | 2.70E-03 | -8.12E-04 | -6.94E-04 | 6.86E-04 |
| K06209$_{VIP\geq0.8}$ | 0.934 | 0.556 | 0.974 | 0.914 | 1.203 | 0.451 | 2.91E-03 | 4.70E-04 | -1.43E-03 | -3.12E-03 | 4.93E-04 | -2.96E-03 |
| K03742$_{VIP\geq0.8}$ | 1.517 | 1.01 | 0.597 | 0.949 | 0.76 | 0.991 | 1.03E-02 | -7.41E-03 | -6.36E-04 | -5.19E-04 | 3.77E-03 | 9.18E-04 |
| K00620$_{VIP\geq0.8}$ | 0.6 | 0.508 | 0.876 | 1.375 | 1.077 | 1.007 | 1.15E-03 | -1.84E-03 | 1.53E-03 | 6.41E-03 | -8.10E-03 | -6.08E-03 |
| K08309$_{VIP\geq0.8}$ | 0.79 | 0.354 | 1.221 | 1.059 | 1.066 | 0.87 | 1.30E-03 | 8.28E-04 | -6.83E-03 | -1.05E-04 | 1.16E-03 | -4.57E-03 |
| K05521$_{VIP\geq0.8}$ | 1.03 | 0.978 | 0.547 | 1.014 | 1.146 | 0.415 | 6.90E-03 | 1.16E-02 | -3.12E-03 | 2.93E-03 | -7.31E-03 | -2.44E-03 |
| K01744$_{VIP\geq0.8}$ | 1.007 | 1.519 | 0.548 | 1.048 | 0.595 | 1.037 | -6.77E-03 | -1.73E-02 | 2.92E-03 | 6.09E-03 | -2.38E-03 | 3.39E-03 |
| K07792$_{VIP\geq0.8}$ | 1.192 | 0.474 | 0.822 | 1.004 | 1.118 | 0.707 | -3.37E-03 | -5.34E-03 | 1.85E-03 | -6.29E-03 | 6.97E-03 | 7.58E-04 |
| K06213$_{VIP\geq0.8}$ | 1.208 | 0.091 | 0.9 | 1.245 | 1.006 | 0.38 | -5.23E-03 | 1.03E-03 | 2.33E-03 | 6.23E-03 | -2.58E-03 | -2.62E-03 |
| K14136$_{VIP\geq0.8}$ | 0.618 | 1.196 | 0.924 | 0.897 | 0.782 | 1.497 | -4.14E-03 | 1.94E-03 | -6.35E-03 | 4.20E-03 | -3.00E-03 | 1.45E-03 |
| K03151$_{VIP\geq0.8}$ | 1.049 | 0.635 | 1.206 | 1.202 | 1.609 | 0.065 | -2.16E-03 | -9.23E-04 | -7.04E-03 | 2.33E-03 | 9.72E-03 | 3.90E-04 |
| K05832$_{VIP\geq0.8}$ | 1.023 | 0.957 | 0.787 | 1.105 | 1.196 | 0.293 | -4.64E-03 | -8.02E-03 | -2.02E-03 | 1.90E-03 | 1.51E-03 | 1.67E-03 |
| K05601$_{VIP\geq0.8}$ | 1.05 | 1.176 | 1.019 | 1.156 | 0.393 | 0.221 | 1.56E-03 | 2.91E-04 | -1.14E-03 | -8.81E-03 | -1.56E-03 | 1.31E-04 |
| K00179$_{VIP\geq0.8}$ | 1.401 | 1.262 | 1.022 | 0.664 | 0.639 | 1.417 | -6.20E-03 | -1.29E-02 | 2.10E-03 | 2.72E-03 | 3.94E-03 | -9.66E-03 |
| K08681$_{VIP\geq0.8}$ | 1.195 | 1.251 | 0.737 | 0.441 | 1.148 | 1.612 | 7.80E-03 | -5.09E-03 | 2.70E-03 | -1.75E-03 | -8.06E-03 | 9.76E-03 |
| K00180$_{VIP\geq0.8}$ | 1.336 | 1.241 | 1.123 | 0.8 | 0.662 | 1.07 | -4.92E-03 | -1.07E-02 | 2.30E-03 | 3.22E-03 | 5.47E-04 | -5.48E-03 |
| K12994$_{VIP\geq0.8}$ | 1.034 | 0.407 | 1.268 | 1.321 | 1.001 | 0.321 | -8.34E-04 | 3.25E-03 | -2.66E-03 | 1.12E-02 | 5.36E-03 | -1.83E-03 |
| K02548$_{VIP\geq0.8}$ | 1.089 | 0.684 | 1.26 | 2.215 | 0.334 | 1.59 | 5.18E-03 | 2.38E-03 | -7.76E-03 | -1.74E-02 | -2.73E-03 | 8.66E-03 |
| K21498$_{VIP\geq0.8}$ | 1.092 | 0.368 | 0.483 | 1.632 | 1.106 | 0.934 | 7.18E-03 | 2.37E-03 | -8.44E-04 | -1.39E-02 | -4.40E-03 | 4.15E-03 |
| K02517$_{VIP\geq0.8}$ | 0.625 | 1.122 | 0.858 | 0.73 | 1.012 | 1.47 | 4.01E-03 | -5.27E-03 | 4.19E-03 | -2.73E-03 | -2.55E-03 | 4.82E-03 |
| K01156$_{VIP\geq0.8}$ | 0.975 | 0.991 | 0.565 | 0.914 | 0.138 | 1.212 | 5.95E-03 | 7.23E-03 | 1.94E-03 | -7.97E-03 | -3.95E-04 | -3.38E-03 |
| K03269$_{VIP\geq0.8}$ | 1.123 | 0.556 | 0.809 | 1.057 | 0.529 | 1.043 | 5.07E-03 | 2.48E-03 | -2.04E-03 | -6.29E-03 | -1.08E-03 | 5.21E-03 |
| K06076$_{VIP\geq0.8}$ | 0.992 | 0.197 | 0.971 | 0.811 | 1.088 | 0.575 | -1.80E-03 | -1.56E-03 | 8.11E-04 | 2.01E-03 | 4.21E-03 | -2.56E-03 |
| K03525$_{VIP\geq0.8}$ | 0.439 | 2.799 | 0.931 | 0.716 | 1.128 | 0.843 | -2.92E-03 | -3.33E-02 | 6.42E-03 | 1.77E-03 | -8.11E-03 | 1.67E-03 |
| K03612$_{VIP\geq0.8}$ | 0.86 | 0.514 | 0.901 | 1.237 | 0.651 | 1.725 | 2.07E-03 | -5.22E-03 | -3.27E-03 | 1.05E-02 | 5.21E-03 | -1.11E-02 |
| K00794$_{VIP\geq0.8}$ | 0.757 | 1.163 | 1.381 | 0.873 | 0.448 | 1.943 | 1.53E-03 | 1.26E-02 | -6.69E-03 | -3.61E-03 | -1.47E-03 | 1.34E-02 |
| K13444$_{VIP\geq0.8}$ | 0.983 | 1.085 | 1.111 | 1.454 | 0.364 | 0.621 | 1.31E-03 | 9.27E-03 | 4.58E-03 | -7.30E-03 | -2.17E-03 | -3.90E-03 |

| | | | | | | | | | | | | |
|---|---|---|---|---|---|---|---|---|---|---|---|---|
| $K03043_{VIP \geq 0.8}$ | 1.026 | 1.349 | 0.883 | 0.971 | 0.446 | 0.771 | 6.80E-04 | 6.40E-03 | -3.12E-03 | -4.47E-03 | 4.54E-04 | -8.28E-04 |
| $K01854_{VIP \geq 0.8}$ | 1.25 | 1.02 | 2.081 | 0.779 | 0.77 | 1.274 | 8.21E-03 | -7.40E-04 | 1.36E-02 | -6.64E-03 | -1.71E-04 | 5.11E-03 |
| $K00817_{VIP \geq 0.8}$ | 1.13 | 1.399 | 0.791 | 1.356 | 0.668 | 0.882 | 4.33E-03 | 1.13E-02 | -1.08E-03 | 5.71E-03 | -1.09E-03 | -6.68E-04 |
| $K01224_{VIP \geq 0.8}$ | 1.984 | 0.977 | 0.826 | 0.508 | 1.001 | 0.755 | 1.22E-02 | 1.09E-02 | -3.93E-03 | -3.18E-03 | -8.57E-03 | 2.50E-03 |
| $K03596_{VIP \geq 0.8}$ | 0.692 | 1.269 | 0.843 | 0.921 | 0.731 | 0.824 | 4.57E-04 | 4.37E-04 | -2.32E-03 | -7.81E-04 | 7.53E-04 | -2.30E-03 |
| $K09710_{VIP \geq 0.8}$ | 0.98 | 0.725 | 0.972 | 0.508 | 1.68 | 0.837 | -3.71E-03 | 3.47E-03 | 3.65E-03 | -6.10E-04 | -1.43E-02 | 2.54E-03 |
| $K03977_{VIP \geq 0.8}$ | 0.915 | 0.94 | 0.597 | 1.277 | 0.999 | 0.326 | 5.85E-03 | 2.46E-03 | -2.01E-03 | 4.20E-03 | -4.50E-03 | -1.23E-03 |
| $K01810_{VIP \geq 0.8}$ | 0.47 | 1.316 | 0.997 | 1.009 | 0.79 | 0.807 | 2.28E-04 | -2.59E-03 | 2.46E-03 | -1.91E-03 | 7.80E-04 | -3.35E-03 |
| $K07114_{VIP \geq 0.8}$ | 0.996 | 0.621 | 1.056 | 0.942 | 0.944 | 0.553 | 3.06E-03 | -7.03E-03 | 4.20E-03 | -2.61E-03 | -6.99E-03 | 3.80E-03 |
| $K22278_{VIP \geq 0.8}$ | 0.815 | 0.388 | 0.967 | 0.804 | 0.56 | 1.255 | -1.23E-03 | 3.86E-03 | 9.99E-04 | -2.54E-03 | 5.30E-04 | -6.80E-03 |
| $K07052_{VIP \geq 0.8}$ | 1.076 | 0.373 | 1.36 | 0.744 | 1.411 | 1.016 | 6.08E-03 | -4.36E-03 | 9.87E-03 | -5.91E-03 | -1.19E-02 | 2.14E-03 |
| $K00986_{VIP \geq 0.8}$ | 0.982 | 1.751 | 0.754 | 0.887 | 1.03 | 0.666 | -3.67E-03 | -1.82E-02 | 8.73E-05 | -2.45E-03 | 2.18E-03 | 1.38E-04 |
| $K07484_{VIP \geq 0.8}$ | 0.979 | 0.726 | 1.377 | 0.589 | 1.14 | 0.975 | -2.64E-04 | 5.52E-03 | 6.69E-03 | -2.59E-03 | -3.20E-04 | 4.49E-03 |
| K05566 | 0.742 | 1.451 | 1.012 | 0.742 | 0.508 | 1.189 | 2.40E-03 | -1.22E-02 | 3.64E-03 | 3.83E-03 | -4.30E-03 | 3.66E-03 |
| K17884 | 0.348 | 1.182 | 0.419 | 2.594 | 0.651 | 1.122 | 1.96E-03 | 1.37E-02 | -3.05E-03 | 2.15E-02 | 3.63E-03 | 7.74E-03 |
| K10212 | 0.517 | 1.747 | 0.092 | 2.424 | 0.567 | 0.979 | -1.60E-03 | 1.92E-02 | 2.98E-04 | 1.83E-02 | 4.30E-03 | 6.36E-03 |
| K01518 | 1.883 | 0.848 | 0.142 | 0.461 | 0.277 | 1.159 | -1.23E-02 | 7.36E-04 | -4.26E-04 | 4.03E-03 | -1.63E-03 | -4.45E-03 |
| K01304 | 1.103 | 0.643 | 1.371 | 0.287 | 0.656 | 1.604 | -6.70E-03 | -2.42E-03 | 9.60E-03 | -8.28E-04 | -2.57E-04 | 1.11E-02 |
| K16927 | 0.303 | 0.399 | 0.886 | 0.994 | 0.951 | 0.787 | 5.81E-04 | 2.23E-03 | 2.00E-03 | -7.92E-03 | 5.91E-03 | -5.31E-03 |
| K04072 | 1.738 | 0.188 | 0.646 | 1.125 | 0.559 | 0.888 | -1.17E-02 | -2.01E-03 | 4.28E-03 | -9.67E-03 | -4.77E-03 | 6.18E-04 |
| K03791 | 0.729 | 0.921 | 0.755 | 1.625 | 0.746 | 1.197 | -3.21E-03 | 6.05E-03 | -2.21E-03 | 1.42E-02 | 1.21E-03 | 4.09E-03 |
| K20742 | 1.23 | 1.004 | 0.857 | 0.67 | 0.448 | 0.632 | 4.79E-03 | 6.31E-03 | 4.53E-03 | -5.25E-03 | -3.17E-03 | 3.23E-03 |
| K01975 | 0.984 | 1.156 | 1.266 | 0.676 | 0.371 | 0.797 | -5.85E-04 | 5.48E-03 | 8.61E-03 | 5.89E-03 | 3.05E-03 | -3.07E-03 |
| K06012 | 1.449 | 0.635 | 0.447 | 0.356 | 1.663 | 1.228 | -9.79E-03 | -6.07E-03 | -3.16E-03 | -4.46E-04 | -1.42E-02 | -7.61E-03 |
| K05341 | 0.631 | 0.799 | 0.885 | 0.872 | 1.15 | 0.8 | -3.25E-03 | -8.66E-03 | 2.55E-03 | -4.60E-04 | -6.03E-03 | -5.17E-03 |
| K03623 | 1.227 | 0.732 | 1.187 | 0.718 | 0.759 | 1.132 | -6.24E-03 | 3.40E-03 | 7.73E-03 | 3.90E-03 | 3.41E-03 | -1.31E-04 |
| K23997 | 0.759 | 1.017 | 1.554 | 2.259 | 0.782 | 0.758 | -3.12E-03 | -8.03E-03 | 9.37E-03 | 1.49E-02 | 4.87E-03 | 4.96E-03 |
| K02279 | 0.438 | 0.697 | 1.211 | 1.099 | 1.016 | 0.329 | 9.21E-04 | 7.68E-03 | 5.79E-03 | 1.65E-03 | 2.02E-03 | 2.22E-03 |
| K19411 | 0.347 | 0.925 | 0.04 | 0.909 | 1.309 | 0.468 | 9.19E-05 | 9.28E-03 | -1.01E-04 | 4.29E-03 | -1.10E-02 | 1.04E-03 |
| K06198 | 0.408 | 0.783 | 0.576 | 1.898 | 0.959 | 1.184 | -2.70E-05 | -6.48E-03 | 2.48E-04 | 1.37E-02 | -7.99E-03 | 8.10E-03 |
| K03708 | 0.575 | 0.603 | 0.198 | 0.803 | 2.022 | 0.867 | -5.26E-04 | 6.17E-03 | -1.24E-03 | -6.93E-03 | -1.73E-02 | -3.33E-03 |
| K13684 | 0.949 | 0.708 | 0.281 | 0.934 | 0.562 | 1.56 | -5.65E-03 | 6.17E-03 | 1.93E-03 | 8.05E-03 | 4.79E-03 | 1.08E-02 |

| | | | | | | | | | | | | |
|---|---|---|---|---|---|---|---|---|---|---|---|---|
| K09124 | 1.073 | 0.739 | 0.638 | 1.107 | 0.988 | 0.738 | -6.63E-03 | -7.43E-03 | 4.23E-03 | 9.57E-03 | 7.75E-03 | 5.10E-03 |
| K06933 | 1.495 | 0.434 | 1.494 | 1.725 | 0.761 | 0.256 | -9.16E-03 | -3.92E-03 | 8.90E-03 | 1.04E-02 | -3.02E-03 | -1.77E-03 |
| K07148 | 1.639 | 0.409 | 0.912 | 0.812 | 0.707 | 0.499 | -9.00E-03 | 7.85E-04 | 1.84E-03 | 7.00E-03 | 4.52E-04 | -2.16E-03 |
| K01759 | 1.03 | 0.643 | 0.777 | 0.924 | 1.26 | 0.545 | 6.31E-03 | -1.10E-03 | 3.04E-03 | -1.46E-03 | 4.84E-03 | 6.31E-04 |
| K18122 | 0.569 | 0.626 | 0.955 | 0.898 | 1.081 | 0.479 | 1.95E-03 | -5.00E-03 | -6.96E-03 | -7.22E-03 | 3.56E-03 | 3.28E-03 |
| K07035 | 0.789 | 1.821 | 0.827 | 0.687 | 1.193 | 0.531 | -3.35E-03 | -2.01E-02 | 5.42E-03 | 1.99E-03 | -7.91E-03 | 1.40E-03 |
| K16214 | 1.368 | 1.329 | 0.925 | 0.597 | 0.799 | 0.372 | 8.07E-03 | -1.36E-02 | 1.68E-03 | -1.17E-03 | 5.39E-03 | 4.74E-04 |
| K00756 | 0.396 | 0.384 | 0.907 | 0.911 | 0.981 | 0.148 | -2.67E-03 | -1.92E-04 | 5.01E-03 | -7.87E-03 | 2.69E-03 | -2.50E-04 |
| K10112 | 0.46 | 1.408 | 0.341 | 0.314 | 0.931 | 0.893 | -2.09E-03 | -1.57E-02 | 2.47E-03 | -2.07E-03 | -7.00E-03 | -4.80E-03 |
| K01486 | 0.772 | 1.092 | 1.315 | 0.402 | 0.804 | 0.252 | -4.98E-03 | 1.15E-02 | 9.54E-03 | 2.27E-03 | 2.30E-03 | 5.17E-04 |
| K20276 | 1.289 | 0.827 | 0.654 | 0.506 | 0.292 | 1.521 | -7.20E-03 | 2.20E-03 | 6.18E-04 | 4.36E-03 | 2.44E-03 | -9.54E-03 |
| K03639 | 0.73 | 0.521 | 1.303 | 0.821 | 1.05 | 0.722 | 1.74E-03 | 6.20E-03 | 6.89E-03 | 1.38E-03 | 6.99E-03 | -4.89E-03 |
| K04094 | 0.576 | 0.585 | 0.812 | 0.857 | 0.946 | 0.685 | -3.03E-03 | -5.08E-03 | 1.68E-03 | -1.38E-03 | -3.34E-03 | -4.65E-03 |
| K09803 | 1.367 | 0.652 | 0.873 | 0.236 | 0.717 | 1.761 | 9.25E-03 | -4.21E-03 | -6.36E-03 | -5.52E-04 | 5.57E-04 | -1.19E-02 |
| K10532 | 0.857 | 0.549 | 0.234 | 0.515 | 0.92 | 2.113 | 5.03E-03 | -3.95E-03 | -1.18E-03 | 2.55E-03 | -4.96E-03 | 1.46E-02 |
| K13075 | 0.889 | 0.618 | 0.985 | 1.15 | 0.767 | 0.747 | -9.53E-04 | -6.70E-03 | 3.19E-03 | 9.55E-03 | -3.18E-03 | -4.95E-03 |
| K16698 | 1.283 | 0.504 | 0.644 | 1.441 | 0.718 | 0.838 | -8.38E-03 | -1.44E-03 | 2.28E-03 | -1.16E-02 | -3.65E-03 | 4.53E-03 |
| K14188 | 0.698 | 1.669 | 0.211 | 0.937 | 0.241 | 0.838 | 3.92E-03 | -1.88E-02 | -8.28E-04 | -2.70E-03 | -1.35E-03 | -9.34E-04 |
| K03820 | 0.677 | 1.245 | 1.086 | 1.071 | 0.579 | 0.628 | 3.06E-03 | -1.21E-02 | -7.00E-03 | 2.95E-03 | 9.38E-05 | -3.68E-03 |
| K08678 | 0.734 | 0.718 | 1.716 | 0.475 | 1.519 | 0.862 | -1.40E-03 | -5.10E-03 | -9.97E-03 | 2.26E-03 | 1.14E-02 | 1.04E-03 |
| K16053 | 0.475 | 0.806 | 0.224 | 0.573 | 0.874 | 1.047 | 3.10E-03 | 2.53E-03 | 1.31E-04 | -2.03E-03 | -5.16E-03 | 6.88E-03 |
| K12976 | 0.569 | 0.79 | 0.991 | 0.218 | 1.894 | 0.979 | -1.55E-03 | 3.85E-04 | 5.17E-03 | -1.38E-03 | -1.52E-02 | 5.38E-03 |
| K19225 | 1.046 | 0.152 | 1.78 | 0.227 | 0.65 | 0.807 | 5.33E-03 | 1.69E-03 | 1.11E-02 | 1.68E-03 | -5.16E-03 | 5.43E-03 |
| K07488 | 1.58 | 0.656 | 0.31 | 0.934 | 0.705 | 1.391 | 1.06E-02 | -2.70E-03 | 9.16E-04 | 7.76E-03 | 3.93E-03 | 9.55E-03 |
| K03473 | 0.93 | 0.541 | 1.729 | 0.459 | 0.687 | 1.461 | 8.93E-04 | 3.86E-03 | -1.04E-02 | 4.01E-03 | 7.60E-04 | -9.37E-03 |
| K06442 | 0.683 | 0.57 | 0.906 | 0.882 | 0.96 | 0.549 | 1.34E-03 | 4.81E-03 | 9.00E-04 | -6.42E-04 | -3.16E-04 | -3.23E-03 |
| K18928 | 0.931 | 0.488 | 0.973 | 0.775 | 0.816 | 0.382 | -2.19E-03 | 5.70E-03 | -2.10E-03 | 4.91E-03 | -1.73E-03 | -6.09E-04 |
| K01119 | 0.311 | 1.224 | 0.785 | 0.64 | 0.945 | 2.035 | 1.11E-03 | 7.98E-03 | 5.38E-03 | -5.60E-03 | -6.08E-03 | 1.12E-02 |
| K23535 | 0.71 | 0.963 | 0.763 | 1.178 | 1.11 | 0.218 | 1.67E-04 | -4.77E-03 | 8.41E-04 | 2.80E-03 | 1.37E-03 | 8.83E-04 |
| K03705 | 0.784 | 0.294 | 0.877 | 0.933 | 1.236 | 0.176 | 2.36E-03 | -3.12E-04 | -8.16E-04 | -8.95E-04 | -3.09E-03 | -4.25E-04 |
| K11145 | 0.603 | 0.358 | 0.875 | 0.937 | 1.208 | 0.35 | 1.64E-03 | 3.80E-03 | 1.33E-03 | 9.89E-04 | -3.75E-03 | -2.08E-03 |
| K06024 | 0.71 | 0.436 | 1.006 | 1.114 | 1.19 | 0.308 | 3.32E-03 | 4.58E-03 | 2.99E-03 | 3.07E-03 | -4.45E-03 | -9.18E-04 |
| K03976 | 1.741 | 1.027 | 0.697 | 0.654 | 0.604 | 1.24 | -1.17E-02 | 6.14E-03 | 5.00E-03 | -5.56E-03 | -2.60E-03 | 6.72E-03 |
| K03442 | 0.439 | 0.717 | 0.931 | 0.707 | 0.855 | 1.274 | 4.79E-04 | 7.19E-03 | 6.77E-03 | 4.41E-04 | 2.40E-03 | -7.46E-03 |

| | | | | | | | | | | | | |
|---|---|---|---|---|---|---|---|---|---|---|---|---|
| K09680 | 0.687 | 0.345 | 1.002 | 0.673 | 0.913 | 0.906 | 1.14E-03 | 1.82E-03 | 3.99E-03 | 1.35E-03 | 6.93E-03 | 4.15E-03 |
| K13532 | 0.723 | 1.18 | 0.86 | 0.752 | 0.574 | 1.064 | 2.47E-03 | 1.40E-02 | 1.90E-03 | -1.51E-03 | -5.30E-05 | 6.78E-03 |
| K24180 | 0.131 | 0.281 | 0.835 | 0.342 | 1.099 | 1.136 | -6.32E-04 | 3.21E-03 | 5.41E-03 | -2.46E-03 | 9.41E-03 | 7.81E-03 |
| K09772 | 0.651 | 0.438 | 0.854 | 0.847 | 1.297 | 0.342 | 1.94E-03 | 3.80E-03 | -1.29E-04 | -2.75E-03 | -3.01E-03 | -2.33E-03 |
| K00761 | 0.818 | 0.088 | 0.741 | 0.913 | 0.991 | 0.575 | -4.10E-03 | 1.04E-03 | -3.10E-03 | 2.43E-03 | 4.20E-03 | -3.86E-04 |
| K03523 | 0.738 | 0.772 | 0.934 | 0.944 | 1.029 | 0.095 | 1.65E-03 | 4.99E-03 | 1.59E-03 | -9.13E-04 | -1.82E-04 | -5.30E-04 |
| K03091 | 1.033 | 0.729 | 0.097 | 0.403 | 1.742 | 1.118 | -6.91E-03 | 5.53E-03 | 7.03E-04 | -2.44E-03 | -1.44E-02 | -6.40E-03 |
| K06167 | 0.655 | 0.714 | 0.979 | 0.822 | 0.849 | 0.221 | -1.12E-03 | 8.43E-03 | 2.76E-03 | -5.26E-04 | 3.34E-03 | 1.42E-03 |
| K09015 | 0.354 | 1.319 | 0.981 | 0.502 | 0.348 | 1.081 | 2.50E-04 | 1.24E-02 | 7.07E-03 | 1.24E-03 | 1.92E-03 | 5.42E-03 |
| K03816 | 0.84 | 1.071 | 0.708 | 0.446 | 0.214 | 0.981 | 3.43E-03 | 8.99E-03 | 1.20E-03 | 1.78E-03 | -1.64E-03 | 5.75E-03 |
| K07102 | 1.022 | 0.621 | 0.368 | 0.255 | 1.177 | 1.412 | 5.68E-03 | -2.46E-03 | 9.56E-04 | 1.98E-03 | 9.34E-03 | 5.28E-03 |
| K03643 | 0.206 | 1.484 | 1.242 | 0.425 | 0.283 | 1.441 | 7.67E-04 | -6.73E-03 | -9.05E-03 | -3.62E-03 | 2.03E-03 | 4.65E-05 |
| K07260 | 0.703 | 1.362 | 0.631 | 0.519 | 1.14 | 0.905 | -4.63E-03 | -1.52E-02 | 3.97E-03 | -4.26E-03 | -7.13E-03 | -5.02E-03 |
| K07037 | 0.759 | 0.48 | 0.998 | 0.637 | 1.001 | 1.311 | 1.16E-03 | 5.65E-03 | 1.66E-03 | 2.60E-04 | -4.82E-03 | 8.95E-03 |
| K08679 | 1.22 | 0.395 | 0.413 | 1.113 | 0.871 | 0.759 | -8.26E-03 | 1.05E-03 | -6.61E-04 | 7.70E-03 | -4.88E-03 | 3.55E-03 |
| K04486 | 0.961 | 0.573 | 0.706 | 0.92 | 1.454 | 0.589 | -5.11E-03 | 4.59E-03 | -3.38E-05 | -4.06E-04 | -8.54E-03 | -2.79E-03 |
| K01009 | 0.894 | 0.379 | 0.976 | 0.736 | 0.949 | 0.366 | -1.66E-03 | -2.22E-03 | 2.15E-04 | 7.07E-04 | 3.60E-03 | -1.29E-03 |
| K08591 | 0.75 | 0.394 | 0.878 | 1.138 | 1.097 | 0.228 | 2.25E-03 | 3.40E-03 | 8.17E-04 | 3.47E-03 | -6.21E-04 | -1.56E-03 |
| K00346 | 0.375 | 0.893 | 1.415 | 0.504 | 0.105 | 1.466 | 1.23E-03 | 1.28E-03 | -1.02E-02 | 1.76E-03 | -3.27E-05 | 1.39E-03 |
| K03642 | 0.511 | 0.678 | 2.096 | 1.59 | 0.424 | 1.382 | 1.03E-03 | -2.68E-04 | -1.29E-02 | -1.13E-02 | 3.36E-03 | 5.01E-03 |
| K16694 | 0.896 | 0.87 | 0.52 | 0.658 | 0.6 | 2.212 | 5.85E-03 | 9.98E-03 | -1.58E-03 | 5.48E-03 | -1.81E-04 | 1.50E-02 |
| K09765 | 1.273 | 0.744 | 0.9 | 0.421 | 0.954 | 0.798 | -7.49E-03 | -6.67E-03 | 5.53E-03 | 2.96E-03 | -7.31E-03 | -3.82E-04 |
| K02065 | 1.112 | 0.499 | 0.405 | 0.445 | 0.939 | 1.707 | 6.09E-03 | 3.45E-03 | -1.32E-03 | -3.46E-03 | 7.87E-03 | 7.48E-03 |
| K02527 | 0.847 | 0.498 | 0.88 | 0.656 | 0.563 | 1.197 | 4.67E-03 | -2.75E-03 | -1.59E-03 | -4.42E-03 | 3.54E-03 | 9.39E-04 |
| K01918 | 0.736 | 0.836 | 0.683 | 0.432 | 1.617 | 1.314 | -7.41E-04 | 8.88E-03 | -1.72E-03 | -3.77E-03 | -1.27E-02 | -7.73E-03 |
| K07277 | 0.907 | 1.043 | 0.767 | 0.479 | 0.472 | 1.35 | 5.03E-03 | 8.29E-03 | -5.58E-03 | -1.26E-03 | -4.05E-03 | 2.56E-03 |
| K00979 | 0.69 | 0.839 | 1.14 | 0.333 | 0.584 | 1.24 | 8.05E-04 | -1.53E-03 | -6.25E-03 | -2.82E-03 | -4.41E-03 | 4.77E-03 |
| K01515 | 0.774 | 0.75 | 1.632 | 1.102 | 0.824 | 0.469 | 3.20E-03 | 8.77E-03 | 7.53E-03 | 2.99E-03 | -8.41E-04 | 3.24E-03 |
| K02897 | 0.956 | 0.265 | 0.974 | 0.605 | 1.311 | 0.528 | 3.66E-03 | -2.51E-03 | -1.46E-03 | -2.41E-03 | -1.06E-02 | 2.60E-03 |
| K01218 | 0.794 | 0.275 | 1.041 | 0.912 | 0.865 | 0.401 | 8.05E-04 | 3.27E-03 | -5.78E-04 | -4.53E-03 | 2.61E-03 | 8.56E-04 |
| K00793 | 0.933 | 0.461 | 0.987 | 0.152 | 0.647 | 1.265 | 4.91E-03 | -9.15E-04 | -5.02E-03 | -5.73E-04 | -5.55E-03 | 5.05E-03 |
| K03771 | 0.971 | 0.374 | 1.025 | 1.453 | 0.485 | 0.772 | 2.84E-03 | -4.44E-03 | -1.55E-03 | -9.73E-03 | -1.31E-03 | 3.48E-03 |
| K01627 | 1.572 | 0.411 | 1.343 | 0.412 | 0.37 | 1.117 | 9.42E-03 | 2.21E-03 | -7.06E-03 | 3.20E-03 | 3.13E-03 | 1.64E-03 |
| K02536 | 1.103 | 0.294 | 1.476 | 0.148 | 0.465 | 1.224 | 4.67E-03 | 2.01E-03 | -8.53E-03 | -5.25E-04 | -2.79E-03 | 5.73E-03 |

| | | | | | | | | | | | | |
|---|---|---|---|---|---|---|---|---|---|---|---|---|
| K03787 | 0.839 | 0.176 | 1.069 | 0.521 | 0.711 | 1.201 | 2.18E-03 | 1.78E-03 | 4.29E-03 | 8.45E-04 | -3.79E-03 | 8.31E-03 |
| K07030 | 1.11 | 0.565 | 0.492 | 0.877 | 1.098 | 0.72 | -5.82E-03 | -6.72E-03 | -1.79E-03 | 3.41E-03 | -6.99E-03 | -4.42E-03 |
| K02067 | 0.76 | 0.811 | 0.611 | 0.821 | 0.719 | 1.524 | 2.88E-03 | -1.73E-03 | -2.22E-03 | -6.89E-03 | -6.04E-03 | 5.24E-03 |
| K07063 | 0.682 | 1.162 | 1.012 | 0.677 | 1.787 | 0.747 | -9.11E-04 | -1.18E-02 | 5.99E-03 | 1.58E-03 | 1.43E-02 | 3.37E-03 |
| K19693 | 0.399 | 1.119 | 0.245 | 1.247 | 0.284 | 1.696 | 2.40E-03 | 5.35E-03 | 1.51E-03 | 9.69E-03 | -1.82E-03 | 9.93E-03 |
| K06041 | 0.662 | 0.839 | 1.081 | 0.417 | 0.394 | 1.512 | 3.52E-03 | 8.62E-04 | -7.56E-03 | -3.33E-03 | -1.40E-03 | 2.80E-03 |
| K07259 | 0.814 | 0.877 | 0.151 | 0.339 | 0.107 | 1.799 | 5.45E-03 | 4.38E-03 | -6.63E-04 | -2.85E-03 | 4.71E-04 | 7.23E-03 |
| K18682 | 0.324 | 1.101 | 1.019 | 0.612 | 1.136 | 0.186 | -2.16E-03 | -5.83E-03 | 6.03E-03 | 2.30E-03 | -9.64E-03 | -9.80E-04 |
| K07816 | 1.037 | 1.37 | 0.514 | 0.464 | 0.087 | 0.957 | -5.63E-03 | -1.58E-02 | 1.64E-03 | -2.54E-03 | -6.78E-04 | -3.98E-03 |
| K04079 | 0.845 | 1.153 | 0.086 | 0.29 | 1.093 | 0.248 | -5.70E-03 | -1.08E-02 | 5.38E-04 | 1.15E-03 | -4.55E-03 | -1.05E-03 |
| K03790 | 0.763 | 1.521 | 1.148 | 0.454 | 0.298 | 1.214 | -1.58E-03 | -1.69E-02 | -3.57E-03 | -2.43E-03 | 6.89E-04 | -3.11E-03 |
| K05970 | 1.059 | 0.888 | 0.57 | 0.702 | 0.728 | 1.008 | 6.54E-03 | 8.91E-03 | 2.80E-04 | 6.05E-03 | 3.90E-03 | 5.56E-03 |
| K22719 | 0.318 | 0.975 | 0.772 | 0.823 | 0.746 | 1.372 | 1.79E-03 | 4.21E-03 | 5.50E-03 | 3.16E-03 | 6.36E-03 | -8.58E-03 |
| K00927 | 0.915 | 0.935 | 0.696 | 1.296 | 0.472 | 0.553 | 4.85E-03 | -4.62E-04 | -1.68E-03 | 4.30E-03 | -6.65E-05 | -2.73E-03 |
| K03615 | 1.114 | 0.786 | 0.357 | 1.345 | 0.626 | 1.037 | -7.54E-03 | -5.72E-03 | 1.60E-03 | 5.11E-03 | 2.46E-03 | -6.83E-03 |
| K00931 | 0.718 | 1.099 | 0.71 | 0.862 | 0.72 | 0.947 | 1.29E-03 | 4.92E-03 | 5.81E-04 | -5.92E-04 | -2.06E-03 | 5.40E-03 |
| K02337 | 0.901 | 1.255 | 0.712 | 1.046 | 0.549 | 0.621 | 2.87E-03 | 5.21E-04 | -6.47E-04 | 1.18E-03 | -2.10E-03 | -1.08E-03 |
| K06909 | 0.775 | 0.534 | 1.002 | 0.604 | 0.832 | 0.929 | -4.89E-03 | -4.53E-03 | 7.09E-03 | -4.82E-03 | 3.07E-03 | 2.29E-03 |
| K03654 | 1.07 | 0.532 | 0.826 | 0.504 | 1.405 | 0.461 | -5.72E-03 | 2.06E-03 | 4.99E-03 | -2.98E-03 | -1.05E-02 | 8.65E-04 |
| K07317 | 0.744 | 0.928 | 0.799 | 1.404 | 0.876 | 0.527 | 5.03E-03 | 6.20E-03 | 3.64E-03 | 8.45E-03 | -7.51E-03 | 2.55E-03 |
| K02493 | 0.374 | 1.488 | 0.458 | 0.974 | 1.059 | 0.473 | -4.38E-04 | 1.72E-02 | 1.42E-03 | 2.95E-03 | -5.22E-03 | 3.22E-03 |
| K04077 | 0.397 | 1.216 | 0.77 | 0.888 | 0.762 | 0.952 | 2.10E-04 | 3.19E-03 | -2.88E-03 | -2.59E-03 | -5.08E-03 | -4.75E-03 |
| K01933 | 0.795 | 1.363 | 0.752 | 0.87 | 0.75 | 0.98 | -2.01E-03 | 3.67E-03 | -3.37E-04 | -1.17E-03 | -3.48E-03 | 2.20E-03 |
| K03593 | 1.145 | 0.256 | 0.925 | 0.641 | 0.361 | 0.87 | -4.92E-03 | -3.05E-03 | 2.67E-03 | 5.49E-03 | 3.08E-03 | 3.35E-04 |
| K01613 | 1.148 | 0.019 | 1.041 | 1.276 | 0.442 | 0.752 | 3.75E-03 | -2.19E-04 | -3.14E-03 | -9.84E-03 | -3.71E-03 | 2.89E-03 |
| K01869 | 1.704 | 1.304 | 0.788 | 0.851 | 0.6 | 0.546 | 1.11E-02 | -6.95E-04 | -2.28E-03 | -3.16E-03 | -2.73E-03 | -1.73E-04 |
| K01929 | 0.86 | 1.101 | 0.628 | 0.948 | 0.643 | 0.314 | -5.82E-03 | -5.68E-03 | 3.87E-03 | 2.32E-03 | 2.76E-03 | -1.86E-05 |
| K01173 | 0.777 | 0.749 | 1.419 | 0.674 | 0.927 | 1.049 | 4.17E-03 | 8.66E-03 | 1.03E-02 | 5.89E-03 | -7.87E-03 | 6.68E-03 |
| K07391 | 0.548 | 0.882 | 1.523 | 0.575 | 2.059 | 0.555 | 3.69E-03 | 1.67E-03 | 8.97E-03 | 2.42E-04 | -1.59E-02 | -3.29E-03 |
| K03572 | 0.608 | 0.978 | 0.356 | 1.722 | 0.874 | 0.522 | -3.08E-03 | 5.88E-03 | -1.73E-04 | -1.50E-02 | 4.37E-03 | 7.30E-04 |
| K04066 | 0.706 | 1.279 | 0.625 | 0.979 | 0.817 | 0.595 | 3.48E-03 | -5.78E-03 | -3.24E-04 | -1.43E-04 | -5.01E-03 | 1.01E-03 |
| K19302 | 0.686 | 1.356 | 0.835 | 0.227 | 1.247 | 0.776 | -5.77E-04 | 1.61E-02 | 4.30E-03 | 9.14E-04 | -1.07E-02 | 1.22E-03 |
| K06904 | 1.983 | 0.119 | 1.008 | 0.596 | 0.895 | 0.71 | -1.30E-02 | -1.15E-03 | 6.75E-03 | 5.02E-03 | 7.64E-03 | 4.74E-03 |
| K07221 | 1.218 | 0.458 | 0.93 | 0.637 | 1.1 | 0.749 | 6.17E-03 | 3.18E-03 | 2.53E-03 | 2.75E-03 | -7.97E-03 | -1.36E-03 |

| | | | | | | | | | | | | |
|---|---|---|---|---|---|---|---|---|---|---|---|---|
| K03585 | 1.082 | 0.589 | 1.492 | 0.44 | 0.527 | 1.035 | 2.68E-03 | 4.81E-04 | -7.92E-03 | 3.35E-03 | -3.23E-03 | 5.78E-03 |
| K02916 | 1.001 | 0.787 | 0.485 | 0.522 | 0.933 | 0.97 | -2.52E-03 | -4.39E-03 | -1.94E-04 | 1.80E-03 | -7.81E-03 | 4.68E-03 |
| K07005 | 1.075 | 0.791 | 0.428 | 1.18 | 1.756 | 0.725 | -7.11E-03 | -4.05E-03 | -2.88E-03 | 6.97E-03 | -1.31E-02 | -5.00E-03 |
| K01991 | 0.977 | 0.602 | 1.014 | 0.761 | 0.509 | 1.186 | 1.88E-03 | 6.16E-03 | 1.89E-03 | 5.54E-04 | 7.87E-04 | 8.20E-03 |
| K20074 | 0.593 | 0.894 | 0.956 | 1.13 | 0.156 | 0.549 | 1.73E-03 | 1.03E-02 | -1.26E-03 | 9.87E-03 | -1.26E-03 | -3.34E-03 |
| K01921 | 0.299 | 1.054 | 0.424 | 0.933 | 0.665 | 0.81 | 8.96E-04 | 4.40E-03 | -2.49E-03 | -3.10E-03 | -3.71E-03 | -3.80E-03 |
| K09748 | 0.557 | 0.984 | 0.382 | 0.392 | 1.753 | 1.235 | -1.93E-03 | -1.11E-02 | 1.70E-03 | -3.42E-03 | -1.47E-02 | 8.46E-03 |
| K03602 | 0.934 | 0.684 | 0.848 | 0.142 | 0.609 | 1.251 | -2.90E-03 | 4.94E-03 | 3.83E-03 | 9.43E-04 | -5.19E-03 | 6.84E-03 |
| K00950 | 0.605 | 0.474 | 1.278 | 0.864 | 0.778 | 1.2 | 1.39E-03 | 5.82E-04 | -7.02E-03 | -5.09E-03 | -6.00E-03 | 5.65E-03 |
| K03544 | 0.841 | 0.711 | 0.573 | 1.116 | 1.126 | 0.686 | 5.46E-03 | 1.08E-04 | 1.91E-03 | -8.96E-03 | -7.15E-03 | -2.97E-03 |
| K07001 | 1.361 | 0.598 | 0.571 | 0.676 | 1.752 | 1.64 | 8.26E-03 | 5.67E-03 | 1.21E-03 | -4.70E-03 | -1.39E-02 | 1.13E-02 |
| K07481 | 0.323 | 0.609 | 1.057 | 0.997 | 1.086 | 0.734 | 5.64E-04 | -6.29E-03 | -6.46E-03 | 1.94E-03 | 2.85E-03 | -4.56E-03 |
| K19092 | 1.051 | 0.526 | 0.76 | 0.966 | 1.128 | 0.743 | 6.71E-03 | 3.92E-03 | -2.66E-03 | 5.83E-04 | -2.03E-03 | 4.33E-03 |
| K03498 | 1.151 | 0.753 | 0.215 | 1.512 | 0.717 | 0.858 | -7.77E-03 | -2.96E-03 | -1.51E-03 | 8.80E-03 | 6.14E-03 | -4.91E-03 |
| K01209 | 1.123 | 1.335 | 0.31 | 0.569 | 0.476 | 1.167 | 7.32E-03 | 1.01E-02 | 1.18E-03 | 4.73E-03 | 4.04E-03 | 5.60E-03 |
| K01992 | 0.921 | 0.667 | 1.292 | 1.231 | 0.79 | 0.439 | -5.90E-03 | -5.02E-03 | 9.41E-03 | -1.08E-02 | -1.58E-03 | 2.94E-03 |
| K03630 | 0.613 | 1.02 | 0.465 | 0.755 | 1.412 | 0.858 | -3.87E-03 | -9.53E-03 | -7.79E-04 | -5.99E-03 | -1.12E-02 | -5.93E-03 |
| K00059 | 0.742 | 0.305 | 0.872 | 0.774 | 1.1 | 1.026 | -2.35E-03 | 2.43E-03 | 6.54E-04 | -3.57E-03 | -7.91E-03 | -3.50E-03 |
| K03699 | 0.931 | 1.418 | 0.79 | 0.652 | 0.784 | 1.262 | 4.08E-03 | 8.60E-03 | -2.51E-03 | -7.63E-04 | -2.58E-03 | -2.26E-03 |
| K07098 | 0.259 | 0.838 | 0.737 | 1.37 | 0.664 | 1.573 | -1.54E-03 | -8.53E-04 | 5.31E-03 | 1.11E-02 | -3.36E-03 | 8.57E-03 |
| K07107 | 1.648 | 1.177 | 1.005 | 0.545 | 0.572 | 0.719 | 1.02E-02 | -1.36E-02 | -3.72E-03 | -3.42E-03 | -2.31E-03 | 4.94E-03 |
| K03308 | 0.932 | 1.131 | 0.512 | 0.569 | 0.734 | 1.853 | 4.67E-03 | -5.19E-04 | 1.22E-03 | 6.17E-05 | 5.67E-04 | 9.93E-03 |
| K02316 | 0.222 | 1.245 | 0.822 | 1.097 | 0.393 | 0.618 | -1.10E-03 | -7.91E-03 | 5.96E-03 | -9.42E-03 | 1.59E-03 | -3.50E-04 |
| K00558 | 0.735 | 1.072 | 1.044 | 0.662 | 0.701 | 0.978 | 2.88E-03 | 7.66E-03 | 7.59E-03 | -5.14E-03 | 4.95E-03 | 5.38E-03 |
| K03574 | 1.986 | 0.455 | 1.753 | 0.301 | 1.544 | 0.057 | 1.19E-02 | -2.03E-03 | -8.65E-03 | 1.87E-04 | -1.31E-02 | 1.93E-04 |
| K03111 | 0.894 | 0.431 | 1.299 | 0.553 | 1.406 | 0.727 | 1.96E-03 | -4.94E-03 | 8.77E-03 | 8.17E-04 | -1.06E-02 | 4.89E-03 |
| K02004 | 1.89 | 0.852 | 0.473 | 0.963 | 0.778 | 0.444 | -1.27E-02 | -8.20E-03 | 2.38E-03 | 6.80E-03 | -5.11E-03 | -2.44E-04 |
| K21572 | 0.607 | 1.301 | 0.785 | 1.158 | 0.538 | 2.469 | 3.60E-03 | 8.58E-03 | 5.17E-03 | 8.93E-03 | -4.44E-03 | 1.52E-02 |
| K07387 | 1.041 | 0.788 | 0.872 | 0.385 | 0.552 | 0.765 | -6.03E-03 | 2.52E-03 | -1.80E-03 | 3.35E-03 | -2.30E-03 | 4.01E-04 |
| K02822 | 0.589 | 0.923 | 0.693 | 0.69 | 1.023 | 0.792 | -1.21E-03 | 8.59E-04 | 1.44E-03 | -5.43E-03 | 3.73E-03 | 4.10E-03 |
| K00563 | 0.327 | 1.284 | 0.413 | 0.342 | 0.442 | 1.253 | -1.93E-03 | -1.53E-02 | 2.35E-03 | -8.56E-04 | -3.40E-03 | 8.07E-03 |
| K04769 | 0.025 | 0.599 | 0.422 | 1.65 | 0.612 | 1.156 | -3.61E-05 | -2.17E-03 | 2.70E-03 | -1.42E-02 | -5.23E-03 | -6.63E-03 |
| K06295 | 0.355 | 0.66 | 0.424 | 0.685 | 1.34 | 1.278 | -2.32E-03 | 6.64E-03 | -1.70E-04 | -4.66E-03 | -1.11E-02 | -7.79E-03 |
| K06396 | 1.049 | 0.739 | 0.716 | 0.495 | 1.482 | 0.76 | -6.94E-03 | 5.74E-03 | -5.11E-03 | -4.33E-03 | -1.26E-02 | -2.29E-03 |

| | | | | | | | | | | | | |
|---|---|---|---|---|---|---|---|---|---|---|---|---|
| K01575 | 0.722 | 0.629 | 0.634 | 1.181 | 0.164 | 1.29 | 2.18E-03 | -1.17E-03 | -3.38E-03 | 8.38E-03 | -1.33E-03 | -8.00E-03 |
| K00887 | 0.936 | 0.682 | 0.76 | 0.87 | 0.58 | 0.475 | -2.13E-03 | -7.34E-04 | -2.83E-03 | 2.84E-03 | -4.07E-04 | 2.68E-03 |
| K22928 | 0.799 | 0.666 | 0.834 | 0.48 | 1.183 | 0.751 | -1.40E-03 | 6.03E-03 | 3.99E-03 | -3.49E-03 | -7.54E-03 | 5.17E-03 |
| K02018 | 0.712 | 0.723 | 1.041 | 1.354 | 0.689 | 0.386 | 4.59E-03 | 8.59E-03 | 7.16E-03 | 6.99E-03 | -5.37E-03 | -1.99E-03 |
| K04034 | 0.84 | 1.332 | 0.483 | 0.498 | 0.559 | 0.227 | 1.83E-03 | 1.58E-02 | -1.74E-03 | -3.80E-03 | -3.71E-04 | -4.69E-04 |
| K03299 | 1.254 | 1.017 | 0.611 | 0.753 | 0.435 | 0.7 | -8.48E-03 | 9.34E-03 | -4.42E-03 | 6.18E-03 | 2.69E-03 | -3.95E-03 |
| K07794 | 1.994 | 0.455 | 0.282 | 0.717 | 1.186 | 0.195 | -1.34E-02 | -1.10E-03 | -1.32E-03 | -3.45E-03 | 4.81E-03 | 1.32E-04 |
| K08972 | 0.338 | 1.561 | 0.569 | 0.764 | 1.283 | 0.574 | -2.27E-03 | 1.51E-02 | 3.64E-03 | 6.53E-03 | 1.05E-02 | 8.54E-04 |
| K01193 | 0.737 | 1.252 | 0.877 | 0.789 | 0.669 | 0.417 | -2.58E-03 | 1.48E-02 | 4.74E-03 | 2.00E-03 | -9.67E-04 | 9.49E-04 |
| K09769 | 1.178 | 0.595 | 0.461 | 1.139 | 0.609 | 0.779 | -7.47E-03 | -5.41E-03 | 3.10E-03 | 6.51E-03 | -9.94E-04 | -3.37E-03 |
| K01512 | 1.435 | 0.376 | 0.692 | 0.393 | 0.917 | 0.342 | -9.34E-03 | 1.39E-03 | 4.83E-03 | 2.54E-03 | -6.29E-03 | 2.33E-03 |
| K22477 | 0.406 | 0.537 | 1.311 | 0.26 | 0.685 | 1.456 | 2.61E-03 | -2.06E-03 | -9.23E-03 | -2.07E-03 | -5.84E-03 | -6.91E-03 |
| K21744 | 0.608 | 0.694 | 0.481 | 0.678 | 1.653 | 0.895 | 2.02E-03 | -6.31E-03 | 2.20E-03 | -5.77E-03 | -1.41E-02 | -4.02E-03 |
| K19304 | 0.506 | 0.798 | 1.317 | 0.454 | 1.182 | 0.666 | 1.13E-03 | -8.22E-03 | -8.92E-03 | -2.03E-03 | -9.83E-03 | 9.25E-04 |
| K06864 | 0.722 | 1.777 | 0.93 | 0.774 | 0.74 | 0.546 | 7.26E-04 | 2.05E-02 | 6.58E-04 | 9.14E-05 | -1.23E-03 | 8.18E-04 |
| K05337 | 0.403 | 0.496 | 0.646 | 0.449 | 1.104 | 0.989 | 2.70E-03 | 5.20E-03 | 3.09E-03 | -7.14E-04 | -5.91E-03 | -6.67E-03 |
| K15772 | 0.584 | 0.466 | 1.743 | 0.765 | 0.694 | 1.658 | 2.01E-03 | -4.31E-03 | -1.23E-02 | -4.77E-03 | -6.00E-04 | -1.03E-02 |
| K01919 | 0.072 | 0.553 | 0.491 | 0.439 | 1.277 | 0.975 | 4.88E-04 | -9.51E-04 | 1.57E-03 | -3.82E-03 | 1.07E-02 | -6.21E-03 |
| K07089 | 1.144 | 0.781 | 0.73 | 0.751 | 0.285 | 1.173 | 5.95E-03 | -9.00E-04 | -1.76E-03 | 4.14E-03 | -9.33E-06 | -4.65E-03 |
| K02203 | 0.496 | 0.824 | 0.497 | 0.721 | 0.993 | 0.758 | 2.81E-03 | 8.76E-03 | -8.85E-04 | -5.23E-04 | -5.34E-03 | -6.13E-04 |
| K02119 | 0.878 | 0.305 | 0.656 | 0.952 | 0.494 | 0.642 | -4.87E-03 | -9.33E-05 | 2.59E-03 | 6.89E-03 | 3.94E-03 | -4.03E-03 |
| K07023 | 1.054 | 0.107 | 0.517 | 0.607 | 1.23 | 0.79 | -6.40E-03 | -9.12E-04 | 9.37E-04 | 1.42E-04 | -6.84E-03 | -4.60E-03 |
| K00283 | 0.598 | 1.199 | 0.573 | 0.768 | 1.176 | 0.714 | 3.01E-03 | 6.95E-04 | -1.81E-03 | -4.31E-03 | -7.79E-03 | 2.20E-04 |
| K07099 | 0.764 | 0.951 | 0.66 | 0.779 | 0.952 | 0.546 | -1.56E-03 | 1.13E-02 | -2.24E-03 | -6.97E-04 | 1.49E-03 | -3.13E-03 |
| K07461 | 0.414 | 1.121 | 1.156 | 0.768 | 0.739 | 0.766 | 2.68E-03 | 1.12E-02 | 8.14E-03 | -6.70E-03 | -4.82E-03 | -1.84E-03 |
| K11176 | 0.537 | 0.04 | 0.91 | 1.281 | 0.652 | 0.592 | -1.67E-04 | 4.35E-04 | 3.57E-03 | 5.66E-03 | -1.52E-04 | -6.79E-06 |
| K01728 | 0.659 | 0.391 | 0.768 | 0.672 | 0.885 | 0.978 | 2.58E-03 | -1.59E-03 | 1.39E-03 | 1.68E-03 | 4.73E-03 | -6.57E-03 |
| K01966 | 0.597 | 0.655 | 0.382 | 0.438 | 1.204 | 1.131 | -3.00E-03 | 7.12E-04 | 2.78E-03 | -3.78E-03 | -1.03E-02 | 6.54E-03 |
| K18908 | 0.933 | 0.729 | 0.999 | 0.766 | 0.761 | 0.387 | -1.42E-03 | -3.74E-04 | -3.40E-03 | -1.51E-03 | -2.02E-03 | 2.65E-03 |
| K03474 | 0.912 | 0.595 | 1.887 | 0.506 | 0.651 | 0.555 | 6.94E-04 | 1.03E-03 | -1.19E-02 | 4.31E-03 | -1.04E-03 | -3.12E-03 |
| K03216 | 0.611 | 0.619 | 0.567 | 0.687 | 1.085 | 1.526 | -1.32E-03 | 4.92E-03 | -1.80E-03 | -4.81E-03 | -5.46E-03 | -9.18E-03 |
| K02315 | 0.818 | 0.751 | 0.55 | 0.889 | 0.789 | 0.242 | 4.59E-03 | 8.19E-03 | 3.53E-04 | 1.94E-03 | -2.23E-03 | -1.66E-03 |
| K06020 | 0.53 | 2.139 | 0.428 | 0.546 | 0.787 | 1.047 | 6.97E-04 | -2.36E-02 | -2.28E-03 | 5.48E-04 | -6.42E-03 | -6.39E-03 |
| K07742 | 0.766 | 0.527 | 0.326 | 0.569 | 1.281 | 0.896 | -5.14E-03 | -1.60E-03 | 1.68E-03 | -1.24E-03 | -9.28E-03 | -3.92E-03 |

| | | | | | | | | | | | | |
|---|---|---|---|---|---|---|---|---|---|---|---|---|
| K03789 | 0.458 | 0.472 | 0.466 | 0.651 | 0.845 | 1.263 | 4.03E-04 | -4.16E-05 | -7.44E-04 | -1.02E-03 | -1.97E-03 | -6.01E-03 |
| K16870 | 0.779 | 0.58 | 0.999 | 0.638 | 1.079 | 0.777 | 2.05E-03 | 4.99E-03 | -1.38E-03 | 1.61E-03 | -4.36E-03 | 4.14E-03 |
| K04516 | 0.696 | 0.912 | 0.774 | 0.585 | 0.454 | 1.187 | 2.41E-03 | 9.31E-03 | 4.45E-03 | -1.70E-03 | -3.72E-03 | 7.09E-03 |
| K07574 | 0.667 | 0.428 | 0.72 | 1.17 | 1.08 | 0.374 | 5.82E-04 | 4.44E-03 | -1.01E-03 | 2.67E-03 | 1.76E-03 | -1.71E-04 |
| K06960 | 0.267 | 0.64 | 0.398 | 0.486 | 1.179 | 0.923 | 1.24E-03 | 3.64E-03 | 2.16E-03 | -2.53E-04 | -7.49E-03 | -2.31E-03 |
| K00703 | 1.322 | 0.592 | 0.796 | 0.68 | 1.185 | 0.411 | -7.20E-03 | -5.80E-03 | 1.52E-03 | -3.26E-03 | 4.42E-03 | 2.71E-03 |
| K00812 | 0.927 | 0.557 | 0.975 | 0.8 | 0.735 | 0.359 | 3.60E-03 | 5.00E-03 | -8.51E-04 | 5.80E-03 | -1.32E-03 | -1.13E-05 |
| K00241 | 0.747 | 0.37 | 1.161 | 0.487 | 0.756 | 0.857 | 2.36E-03 | 1.76E-03 | 6.66E-03 | -1.27E-04 | -3.32E-03 | 4.77E-03 |
| K06204 | 0.784 | 0.786 | 1.609 | 0.9 | 0.58 | 0.332 | -5.04E-04 | 1.82E-03 | -7.15E-03 | -2.94E-03 | 1.05E-03 | 1.93E-03 |
| K01633 | 0.483 | 0.542 | 1.386 | 0.766 | 0.653 | 1.299 | 6.21E-04 | -2.54E-03 | -6.84E-03 | -6.48E-03 | -5.14E-03 | 1.14E-04 |
| K00946 | 1.305 | 0.522 | 0.66 | 0.745 | 0.297 | 1.197 | 7.91E-03 | 6.16E-03 | 2.19E-03 | -5.66E-03 | -9.97E-04 | 4.69E-03 |
| K00912 | 0.981 | 0.208 | 0.798 | 0.766 | 0.53 | 1.099 | 3.62E-03 | -2.47E-03 | -8.75E-04 | -4.93E-03 | 3.97E-03 | 6.81E-03 |
| K06872 | 0.509 | 0.61 | 0.771 | 0.779 | 1.138 | 1.647 | -3.44E-03 | 6.93E-03 | 5.49E-03 | 6.64E-03 | -9.45E-03 | -9.08E-03 |
| K03284 | 1.291 | 0.434 | 1.364 | 0.248 | 0.506 | 0.213 | 7.58E-03 | -3.34E-03 | -8.35E-03 | 3.28E-04 | -1.82E-04 | 1.27E-03 |
| K10536 | 0.746 | 0.623 | 1.031 | 0.619 | 1.392 | 0.473 | 3.20E-03 | 5.50E-03 | 8.48E-04 | 3.59E-03 | 1.18E-02 | -1.72E-03 |
| K03521 | 0.702 | 1.421 | 0.725 | 0.724 | 0.37 | 0.89 | -2.79E-03 | 8.15E-03 | 1.80E-04 | -3.33E-03 | -1.44E-03 | -2.63E-03 |
| K09747 | 0.56 | 0.231 | 0.77 | 0.884 | 1.288 | 0.468 | 3.25E-03 | -1.64E-03 | -3.19E-03 | -3.20E-03 | -5.39E-03 | 7.94E-05 |
| K01206 | 0.787 | 0.799 | 0.369 | 0.772 | 0.859 | 1.706 | -5.32E-03 | 6.76E-03 | 2.34E-03 | 5.91E-03 | -3.68E-03 | 1.15E-02 |
| K23242 | 0.724 | 1.772 | 0.33 | 0.137 | 0.602 | 0.993 | -4.00E-03 | -2.10E-02 | -1.85E-05 | -1.20E-03 | -2.84E-03 | 4.02E-04 |
| K14445 | 0.739 | 1.153 | 0.59 | 0.518 | 0.478 | 1.061 | 1.49E-03 | 1.24E-02 | -1.19E-03 | -2.16E-03 | -3.16E-04 | 2.88E-04 |
| K00748 | 0.763 | 0.788 | 1.73 | 0.651 | 0.123 | 1.3 | 5.58E-04 | -7.68E-03 | -9.67E-03 | -5.38E-03 | 3.85E-04 | 5.19E-03 |
| K01176 | 1.107 | 0.703 | 0.139 | 1.025 | 0.491 | 0.588 | 7.41E-03 | -8.27E-03 | 7.91E-04 | -8.91E-03 | 4.04E-03 | -8.29E-04 |
| K09117 | 1.224 | 0.704 | 0.798 | 0.797 | 0.484 | 1.234 | 7.79E-03 | 5.14E-04 | 1.25E-03 | -3.88E-03 | 1.06E-03 | 5.98E-03 |
| K03616 | 0.402 | 0.289 | 1.758 | 0.741 | 0.754 | 0.912 | 2.22E-03 | -2.96E-03 | -1.10E-02 | 4.96E-03 | 6.47E-03 | -6.13E-03 |
| K01284 | 0.847 | 0.713 | 0.798 | 0.768 | 0.355 | 0.941 | -3.67E-04 | 7.95E-03 | 1.70E-03 | 6.63E-03 | -1.50E-03 | 5.22E-03 |
| K03092 | 0.848 | 0.788 | 0.411 | 0.539 | 0.207 | 1.445 | 4.19E-03 | -1.84E-03 | -2.87E-03 | -4.71E-03 | 1.77E-03 | 6.77E-03 |
| K03517 | 1.778 | 0.963 | 0.471 | 0.068 | 0.399 | 0.3 | 1.18E-02 | 1.40E-03 | 3.43E-03 | 1.92E-05 | 3.08E-03 | 9.82E-04 |
| K01873 | 0.796 | 1.514 | 0.787 | 0.784 | 0.777 | 1.129 | -1.79E-03 | 1.01E-02 | -3.14E-03 | -3.17E-03 | 4.17E-03 | -6.55E-03 |
| K17828 | 0.825 | 0.869 | 0.792 | 0.282 | 0.112 | 0.219 | -5.19E-03 | 5.53E-03 | 5.77E-03 | 2.18E-03 | -7.68E-04 | 1.43E-03 |
| K22522 | 0.903 | 0.525 | 1.428 | 0.772 | 0.627 | 0.737 | -4.64E-03 | 1.68E-03 | 9.49E-03 | 5.92E-03 | -2.88E-03 | 2.92E-03 |
| K06223 | 0.304 | 0.794 | 0.683 | 1.337 | 0.6 | 1.281 | 1.79E-03 | -9.30E-03 | -2.82E-03 | 1.11E-02 | 4.94E-03 | -8.86E-03 |
| K06901 | 1.368 | 0.705 | 0.382 | 0.543 | 0.924 | 0.69 | -9.26E-03 | 2.11E-03 | 2.73E-03 | 3.30E-03 | 7.48E-03 | 5.75E-04 |
| K00765 | 0.494 | 1.109 | 0.438 | 1.023 | 0.552 | 0.662 | 2.18E-03 | 9.09E-03 | 2.84E-03 | 4.28E-03 | -2.80E-03 | 4.18E-03 |
| K04567 | 0.466 | 2.012 | 0.707 | 0.747 | 0.601 | 1.263 | -8.43E-04 | -1.94E-02 | 1.24E-03 | -6.86E-04 | 1.42E-04 | -5.96E-03 |

| | | | | | | | | | | | | |
|---|---|---|---|---|---|---|---|---|---|---|---|---|
| K01890 | 0.694 | 1.396 | 0.761 | 0.893 | 0.661 | 0.634 | -2.42E-04 | -1.83E-03 | -4.37E-04 | -6.64E-04 | 6.76E-04 | -2.56E-03 |
| K11717 | 0.624 | 0.834 | 0.52 | 0.05 | 0.997 | 0.647 | -1.19E-04 | 7.92E-03 | 2.10E-03 | 2.90E-04 | 8.33E-03 | 1.35E-03 |
| K19334 | 0.299 | 1.225 | 0.547 | 0.075 | 0.454 | 1.689 | 2.03E-03 | 3.64E-03 | -2.62E-03 | 2.65E-04 | 1.79E-03 | 6.94E-03 |
| K00174 | 0.594 | 1.068 | 0.155 | 0.476 | 0.22 | 1.053 | 3.24E-03 | 9.81E-03 | 1.08E-03 | 2.53E-03 | -1.83E-03 | -2.73E-03 |
| K07729 | 0.301 | 1.016 | 0.272 | 0.13 | 0.383 | 0.937 | -1.80E-03 | 7.46E-03 | 1.54E-03 | -8.60E-04 | -3.11E-03 | 7.30E-04 |
| K00648 | 0.412 | 0.633 | 0.569 | 1.204 | 0.239 | 1.207 | -1.60E-03 | 7.35E-03 | 3.86E-03 | -8.60E-03 | -4.44E-04 | 6.60E-03 |
| K00760 | 0.64 | 0.181 | 0.969 | 0.248 | 0.444 | 0.983 | -1.49E-03 | 8.69E-04 | 4.65E-03 | 7.67E-04 | -3.45E-03 | 1.75E-03 |
| K02967 | 0.714 | 1.365 | 0.719 | 0.889 | 0.638 | 0.674 | -4.56E-03 | -8.75E-03 | -3.72E-03 | -5.96E-05 | 1.80E-03 | -2.87E-03 |
| K01883 | 0.649 | 1.457 | 0.474 | 0.911 | 0.699 | 0.446 | -3.55E-03 | -7.78E-03 | -8.71E-04 | 1.02E-03 | 2.09E-04 | 1.14E-04 |
| K01803 | 0.563 | 0.835 | 0.02 | 1.013 | 0.788 | 0.765 | 3.80E-03 | -6.23E-03 | -8.08E-05 | 6.91E-03 | 4.21E-03 | 5.29E-03 |
| K19157 | 0.61 | 1.259 | 0.593 | 0.254 | 0.451 | 0.974 | -1.22E-03 | 1.50E-02 | -3.65E-03 | -2.21E-03 | -2.68E-03 | 3.28E-03 |
| K01875 | 0.594 | 1.165 | 0.627 | 1.069 | 0.52 | 0.478 | -3.60E-03 | -7.01E-03 | -1.16E-03 | 1.78E-03 | -2.27E-03 | 2.47E-04 |
| K02528 | 0.498 | 0.539 | 0.335 | 0.892 | 0.871 | 0.535 | -2.94E-03 | 1.28E-03 | -1.90E-03 | 3.15E-03 | 3.85E-03 | -1.33E-03 |
| K00969 | 0.349 | 1.142 | 0.879 | 0.753 | 0.633 | 0.363 | 8.92E-04 | 5.85E-04 | 5.13E-03 | 1.20E-03 | 4.23E-03 | 1.74E-03 |
| K00705 | 0.176 | 0.594 | 1.281 | 2.249 | 0.647 | 0.686 | -5.55E-04 | 4.16E-03 | 8.98E-03 | 1.88E-02 | 4.25E-03 | 4.69E-03 |
| K07979 | 0.73 | 0.386 | 0.596 | 0.789 | 0.888 | 0.82 | -3.81E-03 | -4.51E-03 | 3.95E-03 | 5.64E-03 | 5.91E-03 | 1.02E-03 |
| K03437 | 1.011 | 0.395 | 1.135 | 0.473 | 0.184 | 0.534 | -3.32E-03 | 4.69E-03 | -1.73E-03 | 1.24E-03 | -1.10E-03 | -1.26E-03 |
| K01738 | 0.728 | 0.692 | 0.953 | 0.925 | 0.534 | 0.473 | -3.97E-03 | -5.56E-03 | 4.07E-03 | -1.71E-04 | -3.88E-03 | -3.21E-03 |
| K06131 | 0.4 | 0.947 | 0.558 | 0.541 | 0.299 | 1.202 | -2.97E-04 | -2.88E-03 | 4.06E-03 | 4.67E-03 | -1.52E-03 | 4.62E-03 |
| K00067 | 0.594 | 0.796 | 0.371 | 1.248 | 0.436 | 1.69 | 3.65E-03 | -2.92E-03 | -2.64E-03 | 8.67E-03 | -3.34E-03 | 1.08E-02 |
| K01588 | 0.73 | 1.019 | 0.499 | 0.481 | 0.756 | 1.386 | 4.94E-03 | 8.09E-03 | -3.62E-03 | -2.06E-03 | 3.37E-03 | 9.57E-03 |
| K08316 | 0.234 | 0.736 | 0.532 | 1.16 | 0.877 | 0.585 | -1.54E-03 | -7.43E-03 | -3.76E-03 | -1.01E-02 | 1.36E-03 | 3.98E-03 |
| K02963 | 0.717 | 0.858 | 0.335 | 0.434 | 1.057 | 0.635 | 1.70E-04 | 8.59E-03 | 1.28E-03 | -3.73E-03 | -7.99E-03 | 3.11E-03 |
| K01790 | 0.187 | 0.668 | 0.963 | 0.79 | 0.327 | 0.896 | 4.62E-05 | 6.51E-03 | 6.62E-03 | 6.77E-03 | 2.48E-03 | 6.15E-03 |
| K00764 | 0.662 | 0.788 | 0.547 | 0.917 | 0.525 | 1.021 | -3.75E-03 | 4.25E-03 | -2.09E-03 | 5.51E-03 | -4.18E-03 | -7.05E-03 |
| K02601 | 0.549 | 0.187 | 0.568 | 0.514 | 1.196 | 0.814 | 5.99E-05 | 9.96E-04 | 2.13E-03 | -4.13E-03 | -9.64E-03 | 2.85E-03 |
| K03499 | 0.642 | 0.801 | 0.333 | 1.604 | 0.662 | 0.419 | -2.66E-03 | -5.51E-03 | -1.48E-05 | 8.85E-03 | -2.09E-03 | 1.27E-03 |
| K00432 | 0.976 | 0.573 | 1.106 | 0.651 | 0.632 | 0.51 | 2.75E-05 | -5.45E-03 | -4.17E-05 | 1.67E-03 | -3.75E-04 | 3.13E-03 |
| K07568 | 0.633 | 0.616 | 0.68 | 0.28 | 1.392 | 1.032 | 1.89E-03 | -8.94E-04 | -5.88E-04 | -2.41E-03 | -1.17E-02 | 1.05E-03 |
| K07263 | 0.499 | 0.869 | 0.767 | 0.366 | 0.458 | 0.887 | 7.68E-04 | 8.27E-03 | 4.25E-03 | 2.95E-03 | -3.12E-04 | -5.40E-04 |
| K07271 | 0.731 | 1.217 | 0.768 | 0.565 | 0.797 | 1.055 | 4.50E-03 | 1.15E-02 | 5.58E-03 | 4.82E-03 | -6.83E-03 | 6.05E-03 |
| K07154 | 1.189 | 0.45 | 0.74 | 0.202 | 0.823 | 0.645 | 5.27E-03 | -6.14E-04 | -3.39E-03 | 1.77E-03 | -6.91E-03 | 3.79E-03 |
| K02499 | 0.482 | 1.104 | 0.793 | 0.612 | 0.395 | 0.626 | 9.73E-05 | 1.29E-02 | 5.62E-03 | 5.04E-03 | 3.18E-03 | 2.25E-03 |
| K02651 | 0.615 | 0.746 | 0.705 | 1.881 | 0.715 | 0.737 | 3.62E-03 | 7.17E-03 | 4.86E-03 | 1.18E-02 | 3.30E-03 | 4.53E-03 |

| | | | | | | | | | | | | |
|---|---|---|---|---|---|---|---|---|---|---|---|---|
| K01500 | 0.914 | 0.443 | 0.724 | 0.796 | 0.714 | 0.272 | 1.56E-03 | 2.70E-04 | -2.51E-03 | 3.46E-03 | 4.59E-03 | 5.51E-04 |
| K06985 | 1.045 | 0.307 | 0.543 | 0.231 | 0.335 | 0.48 | 6.85E-03 | -3.54E-03 | 3.52E-03 | 1.97E-03 | 1.89E-03 | 1.61E-03 |
| K02435 | 0.371 | 0.463 | 0.464 | 0.477 | 1.414 | 0.775 | 6.39E-04 | 1.92E-03 | 1.95E-03 | -3.62E-03 | -1.10E-02 | 4.29E-04 |
| K07736 | 0.649 | 0.507 | 0.671 | 0.783 | 1.083 | 0.562 | 9.71E-04 | 6.03E-03 | 4.82E-04 | -3.83E-03 | -3.39E-03 | -3.16E-03 |
| K01155 | 0.291 | 0.736 | 1.101 | 0.243 | 0.468 | 0.758 | -1.58E-03 | 6.05E-03 | 8.01E-03 | -6.09E-05 | -3.01E-03 | -2.43E-03 |
| K06023 | 0.799 | 0.382 | 0.741 | 0.774 | 1.077 | 0.609 | -2.03E-03 | -4.20E-03 | 7.00E-04 | 1.59E-04 | -1.65E-03 | -2.68E-03 |
| K09787 | 0.435 | 0.19 | 0.514 | 0.647 | 0.981 | 0.634 | 8.98E-04 | -4.34E-04 | -2.77E-04 | -9.10E-04 | -3.90E-03 | 2.11E-03 |
| K00351 | 0.259 | 0.599 | 0.617 | 0.719 | 0.329 | 1.643 | 1.25E-03 | 7.11E-03 | -3.89E-03 | 5.80E-03 | -2.61E-03 | 8.72E-03 |
| K04744 | 0.854 | 0.633 | 0.728 | 0.691 | 0.268 | 0.753 | 1.35E-03 | 7.48E-03 | 2.79E-04 | 5.28E-03 | 6.91E-04 | 1.34E-03 |
| K01685 | 0.376 | 0.727 | 0.451 | 0.46 | 0.579 | 0.814 | 8.19E-04 | 2.57E-03 | 2.69E-03 | 3.95E-03 | 4.12E-03 | 4.28E-03 |
| K03770 | 0.571 | 0.506 | 0.137 | 0.098 | 0.385 | 1.541 | 2.18E-03 | 2.94E-03 | 7.94E-04 | -8.10E-04 | 1.43E-03 | 7.15E-03 |
| K03783 | 0.437 | 0.26 | 0.821 | 0.552 | 0.797 | 0.751 | -1.50E-04 | 7.59E-04 | -3.24E-03 | 4.54E-03 | 4.86E-03 | 4.90E-03 |
| K09760 | 0.336 | 0.466 | 1.257 | 0.74 | 0.538 | 0.588 | -2.89E-04 | 1.82E-03 | -5.83E-03 | 6.41E-03 | -3.46E-03 | -4.01E-03 |
| K01338 | 0.425 | 0.385 | 0.8 | 0.236 | 0.226 | 0.652 | 1.50E-03 | 3.79E-03 | 5.79E-03 | -1.70E-03 | -1.90E-03 | -4.50E-03 |
| K02199 | 0.688 | 0.368 | 0.789 | 0.67 | 1.545 | 0.426 | 5.60E-04 | 3.99E-03 | 4.61E-03 | -2.01E-03 | -1.25E-02 | 2.75E-03 |
| K02784 | 0.808 | 0.124 | 0.544 | 0.788 | 0.793 | 0.542 | -4.31E-03 | -2.05E-05 | -2.20E-04 | 1.31E-03 | -4.24E-03 | -8.96E-04 |
| K17103 | 0.762 | 0.009 | 1.061 | 0.62 | 0.636 | 0.445 | -1.27E-03 | -6.16E-05 | 3.97E-03 | -3.99E-04 | -2.14E-03 | 2.67E-03 |
| K07386 | 0.413 | 0.536 | 0.668 | 0.687 | 0.293 | 1.201 | -2.08E-03 | 6.20E-03 | 3.25E-03 | 5.95E-03 | -2.47E-03 | 4.92E-03 |
| K01809 | 0.671 | 0.348 | 1.134 | 0.481 | 0.328 | 0.428 | 8.32E-05 | -3.43E-03 | -3.39E-03 | 1.07E-03 | 2.81E-03 | 2.27E-03 |
| K02501 | 0.336 | 0.867 | 0.423 | 0.347 | 0.48 | 0.533 | -4.09E-04 | 4.86E-03 | 2.62E-03 | -1.78E-03 | -2.73E-03 | 3.25E-03 |
| K20534 | 0.663 | 0.926 | 0.612 | 0.548 | 0.481 | 0.471 | -3.67E-03 | -1.04E-02 | 4.31E-03 | 1.78E-03 | -4.01E-03 | 1.02E-03 |
| K01889 | 0.446 | 0.331 | 0.355 | 1.037 | 0.483 | 0.15 | -2.64E-03 | 2.41E-03 | -9.16E-04 | 4.06E-03 | -2.41E-03 | 2.65E-04 |
| K00287 | 0.752 | 0.323 | 0.906 | 0.672 | 0.602 | 0.307 | -2.00E-03 | 2.16E-03 | 3.76E-03 | 5.73E-03 | -4.29E-03 | 1.10E-03 |
| K06942 | 0.783 | 0.744 | 0.586 | 0.899 | 0.589 | 0.507 | 5.26E-03 | 1.99E-03 | -1.24E-03 | -6.56E-03 | 2.85E-03 | 1.96E-03 |
| K01808 | 0.461 | 0.319 | 0.767 | 0.555 | 0.671 | 0.877 | 1.02E-03 | 1.23E-03 | 4.37E-03 | 4.55E-03 | -4.73E-03 | 3.36E-03 |
| K02520 | 0.53 | 0.08 | 0.64 | 0.409 | 1.604 | 0.424 | -7.61E-04 | -7.51E-05 | 4.66E-03 | -1.10E-03 | -1.31E-02 | 3.58E-04 |
| K00677 | 0.757 | 0.662 | 0.767 | 0.424 | 0.171 | 1.512 | 2.11E-03 | 3.32E-03 | 9.93E-06 | 2.14E-03 | -1.35E-04 | 6.62E-03 |
| K01972 | 0.352 | 0.798 | 0.466 | 0.831 | 0.518 | 0.788 | -2.09E-03 | 1.97E-03 | 1.11E-03 | -3.66E-04 | -1.54E-03 | -4.44E-03 |
| K02904 | 1.366 | 0.224 | 0.306 | 0.285 | 0.676 | 0.699 | 9.14E-03 | 1.76E-04 | 2.14E-03 | -2.41E-03 | 8.32E-04 | 4.66E-03 |
| K02970 | 0.617 | 0.502 | 1.382 | 0.204 | 0.286 | 0.661 | 1.49E-04 | -3.04E-03 | -6.95E-03 | 1.68E-03 | -2.25E-03 | 1.38E-03 |
| K00971 | 0.685 | 0.413 | 0.787 | 0.799 | 0.515 | 1.151 | -2.09E-03 | 2.75E-03 | 2.74E-03 | -4.30E-03 | 1.34E-03 | 7.26E-03 |
| K00973 | 0.076 | 0.707 | 1.932 | 0.679 | 0.613 | 0.586 | -2.73E-04 | 6.61E-03 | 1.36E-02 | 5.50E-03 | -5.24E-03 | 4.05E-03 |
| K02429 | 0.518 | 0.738 | 0.492 | 0.298 | 0.551 | 2.053 | -2.60E-04 | 2.53E-03 | 2.48E-03 | 6.66E-04 | -3.37E-03 | 1.25E-02 |
| K03797 | 0.733 | 0.8 | 0.325 | 0.282 | 0.506 | 1.044 | 3.53E-03 | 3.60E-03 | 1.13E-03 | 1.96E-04 | -3.98E-03 | 4.01E-03 |

| | | | | | | | | | | | | |
|---|---|---|---|---|---|---|---|---|---|---|---|---|
| K07273 | 0.559 | 0.061 | 1.897 | 0.597 | 0.359 | 0.486 | 2.46E-04 | -4.96E-04 | 1.38E-02 | 2.21E-03 | -2.89E-03 | 1.50E-03 |
| K00027 | 0.662 | 0.699 | 0.648 | 0.686 | 0.286 | 0.569 | -4.37E-03 | 1.43E-03 | 4.72E-03 | -4.38E-03 | -8.02E-04 | -3.76E-03 |
| K07444 | 0.263 | 0.594 | 0.687 | 0.438 | 0.657 | 0.684 | -7.03E-04 | 3.49E-03 | 9.13E-04 | -3.35E-03 | 5.26E-03 | 4.26E-03 |
| K03470 | 0.402 | 0.794 | 0.685 | 0.617 | 0.69 | 0.739 | 1.07E-03 | 9.29E-03 | 4.87E-03 | 1.58E-03 | -3.30E-03 | 5.10E-03 |
| K03531 | 0.41 | 0.601 | 0.729 | 0.387 | 0.376 | 0.359 | -7.16E-04 | 4.44E-03 | 5.28E-03 | 1.51E-03 | 3.10E-03 | -9.68E-04 |

**Trait: Residual Feed Intake (RFI); Predictors: 673 heritable microbial genes**

| Microbial gene | VIP | | | | | | Regression coefficient | | | | | |
|---|---|---|---|---|---|---|---|---|---|---|---|---|
| | T1 | T2 | T3 | T4 | T5 | T6 | T1 | T2 | T3 | T4 | T5 | T6 |
| $K02426_{VIP\geq0.8\ (+)}$ | 1.098 | 0.942 | 0.809 | 0.804 | 0.974 | 1.055 | 7.71E-04 | 2.43E-04 | 1.16E-03 | 2.25E-03 | 1.87E-03 | 6.00E-03 |
| $K00721_{VIP\geq0.8\ (+)}$ | 1.213 | 1.226 | 0.769 | 0.836 | 0.942 | 1.032 | 2.93E-03 | 3.79E-03 | 7.12E-04 | 1.16E-03 | 4.46E-03 | 2.97E-03 |
| $K03049_{VIP\geq0.8\ (+)}$ | 1.026 | 1.018 | 1.576 | 0.534 | 1.144 | 0.522 | 6.45E-03 | 6.24E-03 | 5.83E-03 | 3.85E-04 | 7.34E-03 | 1.89E-03 |
| $K03521_{VIP\geq0.8\ (+)}$ | 0.198 | 0.971 | 0.565 | 0.998 | 0.881 | 0.878 | 1.22E-03 | 7.58E-03 | 1.69E-04 | 1.47E-03 | 6.77E-03 | 7.77E-03 |
| $K07502_{VIP\geq0.8\ (+)}$ | 1.062 | 0.872 | 1.142 | 1.235 | 1.534 | 1.06 | -4.07E-03 | 3.42E-03 | 5.25E-03 | 4.29E-03 | 1.14E-02 | 8.04E-03 |
| $K01304_{VIP\geq0.8\ (+)}$ | 1.188 | 1.054 | 1.656 | 0.558 | 1.313 | 1.315 | -4.66E-03 | 7.85E-03 | 7.90E-03 | 3.51E-03 | 8.88E-03 | 1.17E-02 |
| $K01095_{VIP\geq0.8\ (+)}$ | 0.654 | 1.183 | 1.062 | 1.307 | 1.178 | 1.788 | 3.61E-03 | 1.09E-02 | -4.43E-03 | 6.15E-03 | 7.66E-03 | 1.59E-02 |
| $K00616_{VIP\geq0.8\ (+)}$ | 0.851 | 0.52 | 0.963 | 1.008 | 1.566 | 1.106 | 1.03E-03 | -2.60E-04 | 2.90E-03 | 1.66E-03 | 1.06E-02 | 9.79E-03 |
| $K21395_{VIP\geq0.8\ (+)}$ | 0.917 | 1.617 | 1.139 | 1.285 | 0.951 | 0.675 | -1.79E-03 | 1.60E-02 | 5.50E-03 | 2.64E-03 | 3.39E-03 | 5.59E-03 |
| $K06894_{VIP\geq0.8\ (+)}$ | 0.926 | 1.472 | 0.198 | 1.307 | 1.371 | 1.807 | 3.43E-03 | 6.06E-03 | -9.75E-04 | 7.92E-03 | 9.39E-03 | 1.55E-02 |
| $K00240_{VIP\geq0.8\ (+)}$ | 1.191 | 1.145 | 0.66 | 0.876 | 0.938 | 1.886 | 5.53E-03 | 3.83E-03 | 3.05E-03 | -5.39E-03 | 3.26E-03 | 1.51E-02 |
| $K07037_{VIP\geq0.8\ (+)}$ | 0.909 | 1.315 | 0.834 | 0.614 | 0.998 | 1.087 | 1.22E-03 | 2.25E-03 | 1.53E-03 | 2.48E-03 | -2.02E-03 | 6.22E-03 |
| $K01515_{VIP\geq0.8\ (+)}$ | 1.114 | 1.106 | 0.759 | 1.011 | 1.308 | 0.868 | 4.59E-03 | 3.00E-03 | 1.99E-03 | -4.34E-04 | 7.96E-03 | 4.86E-03 |
| $K14092_{VIP\geq0.8\ (+)}$ | 0.672 | 1.054 | 1.79 | 0.915 | 1.23 | 0.571 | 3.73E-03 | 7.57E-03 | 7.17E-03 | 7.75E-04 | 7.98E-03 | -3.69E-03 |
| $K14095_{VIP\geq0.8\ (+)}$ | 0.395 | 0.973 | 1.799 | 0.814 | 1.26 | 0.294 | 2.50E-03 | 7.65E-03 | 7.11E-03 | 2.13E-03 | 8.22E-03 | -1.28E-03 |
| $K14117_{VIP\geq0.8\ (+)}$ | 0.475 | 1.08 | 1.275 | 0.813 | 1.183 | 0.26 | 2.56E-04 | 8.82E-03 | 3.70E-03 | 4.41E-03 | 7.02E-03 | -1.11E-03 |
| $K17884_{VIP\geq0.8\ (+)}$ | 1.442 | 1.441 | 1.762 | 0.254 | 1.253 | 0.644 | 9.33E-03 | 1.36E-02 | -8.62E-03 | 1.14E-03 | 9.52E-03 | 4.78E-03 |
| $K20742_{VIP\geq0.8\ (+)}$ | 0.951 | 0.173 | 1.785 | 0.972 | 0.498 | 2.014 | 5.13E-03 | 2.66E-04 | 8.10E-03 | -6.05E-03 | 3.90E-03 | 1.71E-02 |
| $K04034_{VIP\geq0.8\ (+)}$ | 1.189 | 1.277 | 1.767 | 0.06 | 0.67 | 0.995 | -7.11E-03 | 1.25E-02 | 8.52E-03 | 1.92E-05 | 2.72E-03 | 8.68E-03 |
| $K08972_{VIP\geq0.8\ (+)}$ | 0.57 | 1.336 | 0.623 | 1.11 | 1.054 | 0.964 | 1.01E-03 | 1.29E-02 | 2.45E-03 | -6.81E-03 | 8.24E-03 | 8.55E-03 |
| $K07080_{VIP\geq0.8\ (+)}$ | 0.995 | 0.843 | 0.424 | 1.374 | 1.007 | 0.536 | -2.55E-03 | 7.45E-03 | 1.17E-03 | 3.37E-03 | 5.36E-03 | 4.18E-03 |
| $K02283_{VIP\geq0.8\ (+)}$ | 0.969 | 0.561 | 1.092 | 1.039 | 1.211 | 0.554 | -9.33E-04 | 3.10E-03 | 3.81E-03 | 1.63E-03 | 6.89E-03 | 3.53E-03 |

| | | | | | | | | | | | | |
|---|---|---|---|---|---|---|---|---|---|---|---|---|
| $K05337_{VIP≥0.8\ (+)}$ | 0.46 | 1.163 | 1.065 | 0.655 | 0.909 | 0.912 | -3.63E-04 | 4.61E-04 | 5.22E-03 | 7.26E-04 | 2.25E-03 | 8.05E-03 |
| $K01155_{VIP≥0.8\ (+)}$ | 0.417 | 0.899 | 0.85 | 1.009 | 0.907 | 0.425 | 2.47E-03 | -2.21E-03 | 3.31E-03 | 6.35E-03 | 7.14E-03 | 1.71E-03 |
| $K05521_{VIP≥0.8\ (+)}$ | 1.21 | 0.613 | 0.833 | 1.23 | 0.835 | 0.51 | 7.39E-03 | 4.64E-03 | -3.86E-03 | 4.71E-03 | 1.47E-03 | 3.89E-03 |
| $K02217_{VIP≥0.8\ (+)}$ | 1.03 | 0.617 | 0.736 | 1.215 | 1.431 | 1.475 | 5.54E-03 | 5.17E-03 | -1.38E-03 | 4.36E-03 | 1.08E-02 | 1.30E-02 |
| $K00351_{VIP≥0.8\ (+)}$ | 1.362 | 1.304 | 0.204 | 0.811 | 0.284 | 1.083 | 7.43E-03 | 1.34E-03 | -9.54E-04 | 4.94E-03 | 2.32E-03 | 8.24E-03 |
| $K00980_{VIP≥0.8\ (+)}$ | 0.515 | 0.673 | 2.042 | 0.922 | 0.805 | 0.824 | 1.68E-04 | 6.56E-03 | 1.00E-02 | -1.64E-03 | 2.16E-03 | 6.09E-03 |
| $K00683_{VIP≥0.8\ (+)}$ | 1.129 | 1.011 | 0.747 | 0.903 | 0.77 | 0.969 | 2.16E-03 | -1.10E-03 | 1.54E-03 | 4.66E-03 | 2.66E-03 | 2.46E-03 |
| $K07164_{VIP≥0.8\ (+)}$ | 1.099 | 1.227 | 0.916 | 1.106 | 0.79 | 0.791 | 2.18E-03 | 2.97E-03 | 1.02E-03 | -3.46E-03 | 1.70E-03 | 3.20E-03 |
| $K07317_{VIP≥0.8\ (+)}$ | 0.47 | 0.801 | 1.236 | 1.463 | 0.528 | 1.136 | 2.93E-03 | -1.72E-03 | 5.67E-03 | 7.13E-03 | 4.19E-03 | 9.92E-03 |
| $K23393_{VIP≥0.8\ (+)}$ | 1.246 | 1.098 | 0.986 | 0.857 | 1.105 | 0.88 | -5.11E-03 | 1.06E-02 | 4.64E-03 | 8.24E-04 | 6.90E-03 | -2.36E-03 |
| $K07099_{VIP≥0.8\ (+)}$ | 1.127 | 1.228 | 1.14 | 0.942 | 1.569 | 0.951 | 9.25E-04 | 9.75E-03 | 5.52E-03 | -1.72E-03 | -1.08E-02 | 7.70E-04 |
| $K16511_{VIP≥0.8\ (+)}$ | 1.019 | 0.916 | 0.862 | 1.103 | 0.921 | 0.952 | -2.08E-03 | 3.65E-03 | 2.27E-03 | 2.10E-03 | 1.10E-03 | -3.72E-03 |
| $K06975_{VIP≥0.8\ (+)}$ | 1.672 | 0.838 | 1.13 | 0.996 | 1.165 | 1.487 | 1.07E-02 | 1.10E-03 | -3.36E-03 | -1.89E-04 | 7.07E-03 | 1.32E-02 |
| $K02026_{VIP≥0.8\ (+)}$ | 1.442 | 0.808 | 1.02 | 1.074 | 0.977 | 0.83 | -7.54E-03 | 5.55E-03 | 5.02E-03 | 8.52E-05 | -2.19E-03 | 5.01E-03 |
| $K07075_{VIP≥0.8\ (+)}$ | 1.035 | 1.858 | 1.075 | 1.029 | 1.1 | 0.93 | -4.12E-03 | 1.23E-02 | 4.15E-03 | -2.67E-03 | 5.98E-03 | 4.43E-03 |
| $K18831_{VIP≥0.8\ (+)}$ | 2.449 | 1.259 | 0.886 | 1.104 | 1.198 | 2.293 | 1.56E-02 | 1.13E-02 | -3.62E-03 | -6.93E-03 | 8.98E-03 | 1.89E-02 |
| $K09706_{VIP≥0.8\ (+)}$ | 0.852 | 0.836 | 0.978 | 1.346 | 0.425 | 0.858 | -2.99E-03 | -2.22E-03 | 4.79E-03 | 6.82E-03 | 3.41E-03 | 7.55E-03 |
| $K19048_{VIP≥0.8\ (+)}$ | 0.929 | 0.591 | 0.893 | 0.905 | 1.052 | 1.026 | -7.13E-04 | 2.12E-03 | 4.11E-03 | 7.69E-05 | -4.61E-03 | 9.12E-03 |
| $K02279_{VIP≥0.8\ (+)}$ | 1.066 | 0.876 | 1.161 | 1.039 | 1.09 | 0.488 | -5.75E-03 | 7.31E-03 | 5.34E-03 | -4.93E-04 | 4.87E-03 | 3.50E-03 |
| $K09124_{VIP≥0.8\ (+)}$ | 1.841 | 0.97 | 0.12 | 0.913 | 1.804 | 0.853 | -1.05E-02 | -6.54E-03 | 7.39E-05 | 5.69E-03 | 1.44E-02 | 7.57E-03 |
| $K09121_{VIP≥0.8\ (+)}$ | 1.1 | 0.466 | 0.896 | 1.141 | 0.86 | 0.934 | -3.58E-03 | 2.46E-03 | 3.63E-03 | 2.59E-04 | 3.08E-03 | -7.18E-03 |
| $K07488_{VIP≥0.8\ (+)}$ | 2.446 | 0.801 | 0.709 | 0.814 | 1.312 | 2.054 | 1.58E-02 | 6.32E-04 | -3.48E-03 | -2.60E-03 | 9.94E-03 | 1.82E-02 |
| $K19118_{VIP≥0.8\ (+)}$ | 0.907 | 0.808 | 1.044 | 1.036 | 1.035 | 0.667 | 3.59E-03 | 9.08E-04 | -3.44E-04 | -1.28E-03 | 7.44E-04 | 5.62E-03 |
| $K00567_{VIP≥0.8\ (+)}$ | 1.08 | 1.41 | 0.866 | 1.147 | 0.791 | 0.88 | 5.75E-03 | -8.88E-03 | 9.90E-04 | -2.80E-03 | 2.49E-03 | 7.82E-03 |
| $K00937_{VIP≥0.8\ (+)}$ | 0.819 | 0.851 | 0.937 | 1.05 | 1.084 | 0.559 | 3.13E-03 | 2.40E-03 | -1.79E-03 | -1.40E-03 | 3.21E-03 | 3.09E-03 |
| $K00241_{VIP≥0.8\ (+)}$ | 0.975 | 1.099 | 1.02 | 0.499 | 0.915 | 1.411 | 2.37E-03 | -7.57E-03 | 3.13E-03 | -1.40E-03 | 3.04E-03 | 8.12E-03 |
| $K01719_{VIP≥0.8\ (+)}$ | 1.05 | 0.99 | 0.986 | 0.741 | 0.856 | 0.912 | -1.38E-03 | -1.78E-03 | 1.80E-03 | 8.35E-04 | 9.31E-04 | 2.69E-03 |
| $K03811_{VIP≥0.8\ (+)}$ | 0.918 | 0.934 | 1.011 | 0.7 | 0.924 | 1.249 | -3.46E-04 | 9.13E-04 | 2.14E-03 | -1.09E-03 | 3.36E-04 | 8.89E-03 |
| $K01297_{VIP≥0.8\ (+)}$ | 1.771 | 0.909 | 0.962 | 0.534 | 1.136 | 0.909 | -1.09E-02 | 1.48E-03 | 1.19E-03 | 1.77E-04 | -5.58E-03 | 7.89E-03 |
| $K13444_{VIP≥0.8\ (+)}$ | 1.174 | 1.024 | 0.839 | 1.016 | 0.466 | 0.856 | 2.66E-03 | 4.68E-04 | -3.96E-04 | -3.52E-03 | 9.93E-04 | 8.43E-04 |

| | | | | | | | | | | | | |
|---|---|---|---|---|---|---|---|---|---|---|---|---|
| $K19158_{VIP≥0.8\ (+)}$ | 1.747 | 1.478 | 0.642 | 0.919 | 0.918 | 1.4 | 1.10E-02 | 1.17E-02 | -3.15E-03 | -4.13E-03 | 5.15E-03 | 1.07E-02 |
| $K06904_{VIP≥0.8\ (+)}$ | 1.737 | 0.826 | 1.088 | 0.53 | 0.898 | 1.023 | -1.03E-02 | -7.90E-03 | 3.97E-03 | 3.26E-03 | 7.15E-03 | 8.95E-03 |
| $K19092_{VIP≥0.8\ (+)}$ | 1.508 | 0.861 | 0.722 | 1.26 | 1.118 | 1.165 | 9.43E-03 | 4.57E-03 | 2.24E-03 | -6.09E-03 | -1.21E-03 | 9.83E-03 |
| $K00432_{VIP≥0.8\ (+)}$ | 1.084 | 0.648 | 1.34 | 1.254 | 0.974 | 0.966 | 8.38E-04 | 6.94E-04 | 4.68E-03 | -5.71E-03 | -5.31E-03 | 2.53E-03 |
| $K06142_{VIP≥0.8\ (+)}$ | 1.069 | 1.25 | 0.77 | 0.928 | 1.089 | 1.385 | 1.33E-03 | 2.89E-03 | 1.47E-03 | -3.01E-03 | -5.27E-03 | 5.51E-03 |
| $K15024_{VIP≥0.8\ (+)}$ | 0.827 | 2.293 | 0.866 | 0.757 | 0.383 | 0.937 | -4.73E-03 | -1.89E-02 | 1.43E-03 | 3.94E-03 | 3.07E-03 | 7.33E-03 |
| $K02379_{VIP≥0.8\ (+)}$ | 0.517 | 1.133 | 1.099 | 0.714 | 0.904 | 0.933 | -1.99E-04 | 8.07E-03 | 4.90E-03 | -4.45E-03 | 6.35E-03 | 7.57E-03 |
| $K00641_{VIP≥0.8\ (+)}$ | 0.347 | 1.014 | 1.275 | 0.597 | 1.838 | 1.176 | -2.05E-04 | 4.74E-03 | 5.51E-03 | -6.92E-04 | 1.39E-02 | 9.89E-03 |
| $K22210_{VIP≥0.8\ (+)}$ | 0.862 | 1.458 | 0.606 | 0.821 | 0.753 | 1.172 | 4.28E-04 | -5.28E-03 | 2.31E-03 | 3.81E-03 | 9.00E-04 | -8.64E-03 |
| $K05937_{VIP≥0.8\ (+)}$ | 0.36 | 1.266 | 1.425 | 0.855 | 0.989 | 0.371 | 2.09E-03 | -6.48E-03 | 6.99E-03 | 4.35E-03 | -6.92E-03 | 1.69E-03 |
| $K07794_{VIP≥0.8\ (+)}$ | 0.277 | 1.289 | 0.384 | 0.937 | 1.003 | 2.259 | -5.39E-04 | 1.27E-02 | 1.88E-03 | -2.28E-03 | 2.53E-03 | 2.01E-02 |
| $K01759_{VIP≥0.8\ (+)}$ | 0.641 | 1.108 | 1.032 | 1.169 | 1.009 | 0.319 | 1.55E-03 | -2.22E-03 | 4.99E-03 | -3.65E-03 | 1.41E-03 | 2.56E-03 |
| $K00878_{VIP≥0.8\ (+)}$ | 0.888 | 0.542 | 0.594 | 0.974 | 1.243 | 1.099 | -3.59E-03 | 2.09E-03 | 2.40E-03 | -1.61E-04 | 6.57E-03 | 9.32E-03 |
| $K06209_{VIP≥0.8\ (+)}$ | 1.162 | 1.115 | 0.622 | 1.149 | 1.13 | 0.757 | -3.66E-03 | 5.04E-03 | 1.20E-03 | -3.95E-03 | 8.52E-04 | 3.53E-03 |
| $K08963_{VIP≥0.8\ (+)}$ | 1.193 | 0.496 | 1.152 | 1.331 | 0.992 | 0.625 | -4.71E-03 | -3.33E-03 | 4.73E-03 | 3.04E-03 | 4.67E-03 | 4.80E-03 |
| $K00626_{VIP≥0.8\ (+)}$ | 1.256 | 0.795 | 0.861 | 1.126 | 0.988 | 0.508 | -4.81E-03 | 9.01E-04 | 2.55E-03 | 2.36E-03 | 3.40E-03 | -6.73E-04 |
| $K20866_{VIP≥0.8\ (+)}$ | 0.941 | 1.144 | 0.846 | 0.766 | 0.963 | 0.708 | 5.98E-03 | -8.02E-03 | 1.10E-03 | -3.43E-03 | 2.02E-03 | 6.28E-03 |
| $K02039_{VIP≥0.8\ (+)}$ | 0.836 | 0.598 | 0.934 | 1.114 | 0.882 | 0.508 | -3.37E-03 | 2.15E-03 | 1.34E-03 | -2.29E-03 | 3.80E-03 | 3.04E-03 |
| $K02031_{VIP≥0.8\ (+)}$ | 1.253 | 1.482 | 0.775 | 1.306 | 0.992 | 0.619 | -5.31E-03 | -9.23E-03 | 3.14E-03 | 2.70E-03 | 1.24E-03 | 1.36E-03 |
| $K06177_{VIP≥0.8\ (+)}$ | 0.822 | 1.102 | 0.794 | 1.056 | 1.776 | 0.779 | 4.14E-03 | 2.48E-03 | 2.93E-03 | 6.53E-03 | -1.44E-02 | -1.23E-03 |
| $K06990_{VIP≥0.8\ (+)}$ | 1.284 | 0.993 | 0.764 | 0.947 | 0.848 | 0.212 | 6.65E-03 | -3.20E-03 | 3.22E-04 | -1.97E-03 | 4.49E-03 | 1.65E-03 |
| $K04744_{VIP≥0.8\ (+)}$ | 1.066 | 1.088 | 0.68 | 0.554 | 1.074 | 1.171 | 1.20E-03 | -2.84E-03 | -2.51E-03 | 1.87E-04 | 8.01E-03 | 6.50E-03 |
| $K03704_{VIP≥0.8\ (+)}$ | 0.694 | 0.843 | 0.957 | 1.156 | 1.134 | 0.11 | 2.22E-03 | 1.99E-03 | -1.75E-03 | -4.06E-03 | 1.94E-03 | 9.73E-04 |
| $K02517_{VIP≥0.8\ (+)}$ | 1.12 | 0.814 | 0.905 | 0.771 | 0.84 | 0.549 | 5.50E-03 | 7.93E-03 | -2.98E-03 | 4.97E-04 | -1.69E-03 | 2.93E-03 |
| $K00927_{VIP≥0.8\ (+)}$ | 1.244 | 1.185 | 0.656 | 1.013 | 0.668 | 1.31 | 6.98E-03 | -1.14E-02 | 2.18E-03 | -5.72E-04 | 4.53E-03 | 1.13E-02 |
| $K07076_{VIP≥0.8\ (+)}$ | 1.328 | 1.726 | 0.333 | 1.013 | 0.767 | 1.536 | 6.23E-03 | 1.45E-02 | -7.08E-04 | -4.30E-03 | 5.82E-03 | 1.14E-02 |
| $K02115_{VIP≥0.8\ (+)}$ | 0.171 | 0.471 | 0.973 | 1.141 | 0.867 | 0.917 | 7.97E-04 | 4.64E-03 | -2.08E-03 | -5.34E-03 | 4.58E-03 | 7.71E-03 |
| $K02970_{VIP≥0.8\ (+)}$ | 0.91 | 0.428 | 0.844 | 0.849 | 0.227 | 1.097 | 3.29E-03 | 1.39E-03 | -1.78E-03 | -5.17E-03 | 1.77E-03 | 9.55E-03 |
| $K00971_{VIP≥0.8\ (+)}$ | 0.847 | 1.285 | 0.587 | 0.389 | 0.839 | 0.836 | -3.13E-05 | 4.02E-03 | 1.25E-03 | -1.02E-03 | 3.74E-03 | 2.47E-03 |
| $K02965_{VIP≥0.8\ (+)}$ | 0.877 | 0.069 | 1.2 | 0.935 | 0.235 | 1.337 | 4.54E-03 | 6.59E-04 | 1.63E-03 | -4.59E-03 | -1.82E-03 | 1.19E-02 |

| | | | | | | | | | | | | |
|---|---|---|---|---|---|---|---|---|---|---|---|---|
| $K07114_{VIP≥0.8\ (+)}$ | 1.141 | 0.957 | 0.656 | 0.878 | 0.665 | 1.151 | 2.33E-03 | -3.29E-03 | 2.75E-04 | -2.21E-03 | 2.36E-03 | 6.77E-03 |
| $K02601_{VIP≥0.8\ (+)}$ | 0.58 | 0.114 | 1.175 | 1.436 | 0.827 | 1.411 | 1.47E-03 | 1.08E-03 | 4.67E-03 | -9.04E-03 | -6.46E-03 | 1.25E-02 |
| $K00615_{VIP≥0.8\ (+)}$ | 0.408 | 0.186 | 1.314 | 1.144 | 1.012 | 0.915 | -4.18E-04 | -4.68E-04 | 6.08E-03 | 1.37E-03 | 5.11E-03 | 1.39E-03 |
| $K03559_{VIP≥0.8\ (+)}$ | 1.088 | 1.026 | 0.619 | 1.018 | 0.515 | 1.3 | 1.50E-03 | 4.04E-04 | -1.35E-03 | -3.89E-03 | 7.72E-04 | 5.53E-03 |
| $K01154_{VIP≥0.8\ (+)}$ | 0.237 | 0.615 | 0.895 | 1.033 | 0.952 | 0.899 | 1.12E-03 | 4.29E-03 | -2.82E-03 | -1.60E-03 | 4.44E-03 | 7.86E-03 |
| $K03327_{VIP≥0.8\ (+)}$ | 0.893 | 0.387 | 1.031 | 1.164 | 0.966 | 0.729 | -2.57E-03 | -2.45E-03 | 4.24E-03 | 2.14E-03 | 4.66E-03 | 3.06E-03 |
| $K03151_{VIP≥0.8\ (-)}$ | 1.058 | 1.105 | 0.895 | 1.094 | 1.077 | 0.927 | -3.45E-03 | -6.51E-04 | -3.31E-03 | -3.34E-03 | -3.25E-03 | -4.32E-03 |
| $K16927_{VIP≥0.8\ (-)}$ | 1.027 | 1.844 | 0.659 | 0.989 | 0.892 | 1.505 | -5.68E-03 | -1.27E-02 | -2.55E-03 | -1.85E-03 | -5.87E-03 | -1.29E-02 |
| $K02770_{VIP≥0.8\ (-)}$ | 1.071 | 0.439 | 0.908 | 1.027 | 1.77 | 1.726 | -2.73E-03 | -2.52E-03 | -1.48E-03 | -4.16E-03 | -1.39E-02 | -1.51E-02 |
| $K04094_{VIP≥0.8\ (-)}$ | 0.908 | 1.265 | 0.245 | 1.291 | 1.289 | 1.33 | -3.28E-03 | -4.74E-03 | -4.78E-04 | -6.37E-03 | -6.81E-03 | -6.23E-03 |
| $K16787_{VIP≥0.8\ (-)}$ | 1.469 | 1.198 | 0.498 | 1.169 | 1.315 | 1.125 | -7.44E-03 | -4.49E-03 | -7.09E-05 | -3.82E-03 | -4.39E-03 | -4.98E-03 |
| $K01139_{VIP≥0.8\ (-)}$ | 0.869 | 0.704 | 0.959 | 1.137 | 1.343 | 1.253 | -4.82E-03 | -3.37E-03 | -2.41E-03 | -2.84E-03 | -6.78E-03 | -8.85E-03 |
| $K00975_{VIP≥0.8\ (-)}$ | 1.024 | 1.132 | 0.723 | 1.173 | 1.462 | 1.109 | -1.77E-03 | -5.27E-04 | -1.39E-03 | -1.75E-03 | -7.00E-03 | -6.73E-03 |
| $K15771_{VIP≥0.8\ (-)}$ | 1.183 | 1.168 | 0.58 | 0.735 | 1.261 | 1.457 | -1.10E-03 | -3.58E-03 | -6.47E-04 | -2.62E-03 | -9.57E-03 | -8.72E-03 |
| $K07192_{VIP≥0.8\ (-)}$ | 1.081 | 1.528 | 0.979 | 0.648 | 1.13 | 0.718 | -6.99E-03 | -1.48E-02 | -1.69E-03 | -2.52E-03 | -8.98E-03 | -6.36E-03 |
| $K03775_{VIP≥0.8\ (-)}$ | 0.534 | 1.889 | 1.529 | 0.84 | 1.233 | 0.728 | -7.54E-04 | -1.68E-02 | -7.25E-03 | -4.24E-03 | -1.01E-02 | -1.05E-03 |
| $K06020_{VIP≥0.8\ (-)}$ | 0.912 | 0.976 | 0.936 | 1.069 | 0.361 | 0.675 | -5.17E-03 | -8.28E-03 | -4.55E-03 | -6.47E-03 | -2.55E-03 | -2.05E-03 |
| $K07058_{VIP≥0.8\ (-)}$ | 0.91 | 0.534 | 1.417 | 1.644 | 1.555 | 0.29 | -4.40E-03 | -2.96E-04 | -6.58E-03 | -9.77E-03 | -1.22E-02 | -9.00E-04 |
| $K19353_{VIP≥0.8\ (-)}$ | 0.635 | 1.163 | 0.216 | 0.818 | 1.061 | 1.694 | -2.67E-03 | -1.14E-02 | -5.80E-05 | -4.53E-03 | -8.18E-03 | -1.51E-02 |
| $K01091_{VIP≥0.8\ (-)}$ | 0.605 | 1.439 | 1.012 | 1.441 | 1.537 | 0.222 | -2.51E-03 | -1.21E-02 | -2.23E-03 | -7.53E-03 | -1.07E-02 | -1.09E-03 |
| $K23010_{VIP≥0.8\ (-)}$ | 1.737 | 0.802 | 1.093 | 0.907 | 1.082 | 0.967 | -1.05E-02 | -1.68E-03 | 3.17E-03 | -4.15E-03 | -2.67E-03 | -7.98E-03 |
| $K10117_{VIP≥0.8\ (-)}$ | 1.249 | 0.849 | 0.927 | 1.013 | 0.917 | 0.983 | -5.30E-03 | -1.98E-03 | 3.47E-03 | -1.68E-03 | -4.07E-03 | -3.39E-03 |
| $K03581_{VIP≥0.8\ (-)}$ | 1.006 | 0.965 | 0.864 | 1.165 | 1.069 | 0.964 | -5.07E-03 | -8.79E-05 | -2.47E-03 | -6.08E-04 | 1.20E-03 | -5.47E-03 |
| $K03975_{VIP≥0.8\ (-)}$ | 1.118 | 0.88 | 1.132 | 1.256 | 0.869 | 1.302 | 1.16E-03 | -2.19E-03 | -5.06E-03 | -7.53E-03 | -6.86E-03 | -1.78E-03 |
| $K09707_{VIP≥0.8\ (-)}$ | 1.24 | 1.196 | 1.116 | 0.906 | 2.036 | 0.039 | -8.02E-03 | -1.18E-02 | 2.34E-03 | -5.48E-03 | -1.59E-02 | -1.58E-04 |
| $K15772_{VIP≥0.8\ (-)}$ | 0.941 | 1.042 | 0.65 | 0.839 | 1.407 | 1.001 | -2.25E-03 | 2.25E-03 | -9.77E-04 | -1.40E-03 | -1.00E-02 | -2.68E-03 |
| $K16786_{VIP≥0.8\ (-)}$ | 1.261 | 0.974 | 0.441 | 1.062 | 1.152 | 1.286 | -5.13E-03 | 2.33E-04 | -9.70E-04 | -2.27E-03 | -2.28E-05 | -7.46E-03 |
| $K00620_{VIP≥0.8\ (-)}$ | 1.076 | 0.977 | 0.821 | 0.916 | 0.768 | 0.822 | -1.65E-03 | -2.87E-04 | 3.74E-03 | -9.19E-04 | -1.90E-04 | -1.00E-03 |
| $K22927_{VIP≥0.8\ (-)}$ | 1.009 | 0.964 | 0.596 | 1.137 | 1.202 | 1.065 | -1.24E-03 | -1.65E-03 | 6.32E-04 | -1.59E-03 | -5.15E-03 | -3.17E-03 |
| $K21903_{VIP≥0.8\ (-)}$ | 1.016 | 1.106 | 0.83 | 1.132 | 0.999 | 0.45 | 2.94E-03 | -1.39E-03 | -2.17E-03 | -2.00E-04 | -2.28E-03 | -2.41E-03 |

| | | | | | | | | | | | | |
|---|---|---|---|---|---|---|---|---|---|---|---|---|
| K03321$_{VIP\geq0.8\ (-)}$ | 1.766 | 0.932 | 1.168 | 1.182 | 0.898 | 0.199 | -1.10E-02 | -1.18E-03 | 3.38E-03 | -5.10E-03 | -1.05E-03 | -1.77E-03 |
| K07792$_{VIP\geq0.8\ (-)}$ | 1.283 | 0.98 | 0.543 | 1.041 | 1.106 | 0.949 | -7.17E-03 | -2.48E-03 | -7.46E-04 | -5.74E-03 | 5.67E-03 | -6.53E-03 |
| K07588$_{VIP\geq0.8\ (-)}$ | 1.272 | 0.981 | 1.054 | 1.13 | 1.175 | 0.661 | -6.22E-03 | -2.43E-03 | 2.42E-03 | -4.75E-03 | -3.96E-03 | -3.18E-03 |
| K00991$_{VIP\geq0.8\ (-)}$ | 1.159 | 1.063 | 0.711 | 0.893 | 1.006 | 1.3 | -6.41E-03 | 9.83E-03 | -1.57E-03 | -2.60E-03 | -4.49E-03 | -1.05E-02 |
| K16870$_{VIP\geq0.8\ (-)}$ | 1.146 | 1.271 | 0.875 | 0.729 | 1.137 | 1.171 | -1.48E-03 | 3.36E-03 | -3.01E-03 | -1.79E-03 | -2.85E-03 | -5.33E-03 |
| K07335$_{VIP\geq0.8\ (-)}$ | 1.112 | 1.161 | 0.544 | 1.136 | 0.907 | 1.162 | -4.48E-03 | -7.19E-03 | 1.13E-03 | -2.03E-04 | -1.02E-03 | -7.59E-03 |
| K06167$_{VIP\geq0.8\ (-)}$ | 1.113 | 1.157 | 0.742 | 1.077 | 0.946 | 0.926 | -1.81E-03 | 3.19E-03 | -7.36E-04 | -4.04E-03 | -2.17E-04 | -4.91E-03 |
| K00179$_{VIP\geq0.8\ (-)}$ | 1.62 | 0.724 | 1.436 | 1.348 | 1.703 | 1.613 | -9.17E-03 | -2.60E-03 | 4.41E-03 | -6.86E-03 | -1.29E-02 | -1.43E-02 |
| K02574$_{VIP\geq0.8\ (-)}$ | 1.665 | 0.697 | 1.124 | 1.093 | 1.165 | 1.158 | -1.00E-02 | -1.41E-03 | 2.52E-03 | -5.02E-03 | -7.56E-03 | -1.03E-02 |
| K00798$_{VIP\geq0.8\ (-)}$ | 1.217 | 0.834 | 1.075 | 1.607 | 1.568 | 0.757 | -4.79E-03 | -3.18E-03 | 2.42E-03 | -7.36E-03 | -1.09E-02 | -3.20E-04 |
| K04083$_{VIP\geq0.8\ (-)}$ | 0.781 | 1.142 | 0.919 | 1.219 | 1.311 | 1.123 | -4.11E-04 | 7.37E-04 | -1.83E-03 | -3.82E-03 | -4.98E-03 | -6.83E-03 |
| K03431$_{VIP\geq0.8\ (-)}$ | 0.948 | 1.141 | 1.04 | 1.126 | 0.966 | 0.777 | -1.07E-03 | -1.10E-03 | -3.64E-03 | -1.37E-03 | -2.15E-03 | 6.97E-04 |
| K01009$_{VIP\geq0.8\ (-)}$ | 1.197 | 1.028 | 0.747 | 1.062 | 1.11 | 1.345 | -3.05E-03 | -1.48E-03 | 7.07E-04 | -4.09E-03 | -3.55E-03 | -1.20E-02 |
| K16785$_{VIP\geq0.8\ (-)}$ | 1.068 | 0.94 | 0.607 | 1.025 | 1.099 | 1.266 | -2.78E-03 | -2.19E-04 | 1.86E-03 | -2.19E-03 | -4.14E-03 | -6.05E-03 |
| K00180$_{VIP\geq0.8\ (-)}$ | 1.421 | 0.512 | 1.346 | 1.329 | 1.074 | 1.166 | -7.60E-03 | -4.76E-04 | 3.11E-03 | -5.88E-03 | -6.48E-03 | -1.03E-02 |
| K12994$_{VIP\geq0.8\ (-)}$ | 1.224 | 1.107 | 0.857 | 0.792 | 1.072 | 0.91 | -6.01E-03 | 1.79E-03 | -6.55E-04 | -8.31E-04 | -3.18E-03 | -5.38E-03 |
| K01775$_{VIP\geq0.8\ (-)}$ | 0.943 | 1.106 | 0.651 | 1.178 | 1.283 | 1.029 | -1.18E-04 | 3.63E-03 | -1.33E-03 | -4.37E-03 | -3.29E-03 | -4.78E-03 |
| K01912$_{VIP\geq0.8\ (-)}$ | 1.535 | 0.659 | 1.399 | 1.149 | 1.478 | 1.462 | -8.13E-03 | -3.36E-03 | 4.51E-03 | -4.33E-03 | -1.04E-02 | -1.28E-02 |
| K04487$_{VIP\geq0.8\ (-)}$ | 0.846 | 0.981 | 0.93 | 1.14 | 1.272 | 0.671 | -2.59E-04 | -2.50E-04 | -3.19E-03 | -2.34E-03 | -5.38E-03 | 1.80E-03 |
| K07126$_{VIP\geq0.8\ (-)}$ | 1.203 | 0.809 | 1.877 | 0.851 | 0.835 | 0.554 | 7.67E-03 | -5.60E-03 | -7.33E-03 | -2.73E-03 | -4.21E-03 | -2.34E-03 |
| K01666$_{VIP\geq0.8\ (-)}$ | 0.894 | 0.489 | 0.206 | 1.873 | 0.911 | 2.338 | -5.74E-03 | 2.74E-03 | -3.12E-04 | -1.18E-02 | -7.35E-03 | -1.86E-02 |
| K22300$_{VIP\geq0.8\ (-)}$ | 0.847 | 0.946 | 0.976 | 0.599 | 0.756 | 0.912 | -5.38E-03 | -5.84E-03 | 1.82E-03 | -3.77E-03 | -5.29E-03 | -8.04E-03 |
| K10112$_{VIP\geq0.8\ (-)}$ | 0.419 | 1.339 | 1.311 | 1.009 | 1.161 | 0.534 | -1.29E-03 | -1.03E-02 | 5.96E-03 | -6.24E-03 | -8.72E-03 | -3.65E-03 |
| K03639$_{VIP\geq0.8\ (-)}$ | 1.134 | 1.645 | 1.74 | 0.877 | 0.567 | 0.613 | -3.19E-03 | -1.36E-02 | 8.46E-03 | -3.21E-03 | -1.86E-03 | -5.38E-03 |
| K03741$_{VIP\geq0.8\ (-)}$ | 0.45 | 0.994 | 1.062 | 0.952 | 0.84 | 0.569 | 2.87E-04 | -6.03E-03 | -2.54E-03 | -2.53E-03 | -1.56E-03 | -5.04E-03 |
| K07054$_{VIP\geq0.8\ (-)}$ | 1.228 | 0.686 | 1.047 | 1.518 | 1.146 | 0.48 | -6.42E-03 | -7.22E-04 | 1.87E-03 | -6.63E-03 | -4.80E-03 | -1.40E-03 |
| K03473$_{VIP\geq0.8\ (-)}$ | 0.885 | 0.862 | 0.8 | 1.014 | 0.887 | 0.75 | 9.40E-05 | -7.55E-03 | -3.82E-03 | -5.96E-03 | -4.49E-03 | -1.19E-03 |
| K18928$_{VIP\geq0.8\ (-)}$ | 1.263 | 1.079 | 0.441 | 0.751 | 1.073 | 1.368 | -5.19E-03 | 2.85E-03 | -6.30E-04 | -8.63E-04 | -4.18E-03 | -9.97E-03 |
| K23535$_{VIP\geq0.8\ (-)}$ | 0.998 | 1.947 | 0.772 | 1.15 | 0.964 | 0.771 | -3.03E-03 | -1.43E-02 | 3.53E-03 | -4.69E-05 | -2.43E-05 | -5.29E-04 |
| K03474$_{VIP\geq0.8\ (-)}$ | 0.723 | 0.589 | 0.92 | 0.99 | 0.807 | 1.334 | -1.56E-03 | -4.97E-03 | -4.46E-03 | -5.89E-03 | -3.94E-03 | 7.50E-03 |

| | | | | | | | | | | | | |
|---|---|---|---|---|---|---|---|---|---|---|---|---|
| $K15894_{VIP≥0.8\ (-)}$ | 1.079 | 0.972 | 0.509 | 2.184 | 0.71 | 1.748 | -6.98E-03 | -8.51E-03 | -2.44E-03 | -1.37E-02 | -4.60E-03 | 1.48E-02 |
| $K02036_{VIP≥0.8\ (-)}$ | 0.631 | 0.822 | 0.983 | 1.13 | 0.859 | 0.366 | -1.13E-03 | -2.77E-03 | -1.97E-03 | -4.14E-03 | 2.98E-03 | -1.82E-03 |
| $K13532_{VIP≥0.8\ (-)}$ | 1.174 | 0.962 | 0.66 | 0.885 | 0.666 | 1.033 | -3.05E-04 | -3.76E-03 | -2.13E-03 | -2.80E-03 | 1.31E-03 | -1.56E-03 |
| $K07574_{VIP≥0.8\ (-)}$ | 0.939 | 1.198 | 0.323 | 1.161 | 1.402 | 0.566 | -2.85E-03 | 6.87E-03 | -9.12E-04 | -4.13E-03 | -7.00E-03 | -9.88E-04 |
| $K02072_{VIP≥0.8\ (-)}$ | 1.194 | 0.894 | 0.646 | 1.237 | 1.241 | 0.78 | -5.31E-03 | 3.94E-03 | -2.45E-06 | -3.25E-03 | -3.38E-03 | -2.63E-03 |
| $K02073_{VIP≥0.8\ (-)}$ | 1.131 | 0.769 | 0.81 | 1.2 | 1.055 | 0.648 | -4.91E-03 | 3.71E-03 | -1.65E-03 | -1.87E-03 | -1.53E-03 | -2.15E-03 |
| $K03500_{VIP≥0.8\ (-)}$ | 0.708 | 1.032 | 1.47 | 1.127 | 1.374 | 0.791 | -1.43E-03 | 6.98E-04 | -5.75E-03 | -3.66E-03 | -6.65E-03 | -6.16E-03 |
| $K03642_{VIP≥0.8\ (-)}$ | 1.177 | 0.68 | 1.973 | 1.011 | 0.797 | 1.152 | 1.20E-04 | -2.61E-03 | -9.60E-03 | -5.13E-03 | -5.64E-03 | -2.44E-03 |
| $K04773_{VIP≥0.8\ (-)}$ | 1.206 | 1.137 | 0.867 | 0.573 | 0.677 | 0.977 | 1.72E-03 | -6.90E-03 | -4.19E-03 | -3.61E-03 | -5.13E-03 | -6.74E-04 |
| $K03086_{VIP≥0.8\ (-)}$ | 0.777 | 1.066 | 0.877 | 1.169 | 1.147 | 0.475 | -1.26E-04 | 3.14E-04 | -1.54E-03 | -2.40E-03 | -8.21E-05 | -5.23E-04 |
| $K02199_{VIP≥0.8\ (-)}$ | 0.832 | 1.301 | 0.456 | 0.808 | 0.674 | 0.829 | -6.43E-05 | 7.75E-03 | -1.52E-03 | -3.61E-03 | -4.45E-03 | -3.37E-03 |
| $K02527_{VIP≥0.8\ (-)}$ | 1.322 | 1.063 | 0.865 | 0.559 | 0.734 | 1.437 | 5.95E-03 | -1.53E-03 | -3.85E-03 | -2.33E-03 | -2.88E-03 | -7.22E-03 |
| $K14445_{VIP≥0.8\ (-)}$ | 1.244 | 1.463 | 0.39 | 1.212 | 0.649 | 1.079 | -4.50E-03 | 6.89E-03 | -1.32E-03 | -6.81E-03 | -8.74E-04 | -2.41E-03 |
| $K07030_{VIP≥0.8\ (-)}$ | 1.284 | 1.018 | 0.609 | 0.722 | 1.645 | 1.584 | -5.66E-03 | -1.18E-03 | 3.00E-03 | -9.21E-04 | -1.17E-02 | -9.94E-03 |
| $K02067_{VIP≥0.8\ (-)}$ | 1.023 | 0.693 | 0.942 | 1.074 | 0.466 | 1.143 | -3.77E-05 | -3.41E-03 | -4.64E-03 | -6.71E-03 | -3.73E-03 | 2.35E-03 |
| $K19824_{VIP≥0.8\ (-)}$ | 1.542 | 0.614 | 1.851 | 1.175 | 1.303 | 0.238 | -9.31E-03 | -1.08E-03 | 7.41E-03 | -7.20E-03 | -9.43E-03 | -9.97E-04 |
| $K03286_{VIP≥0.8\ (-)}$ | 0.962 | 0.943 | 0.699 | 1.28 | 0.929 | 0.672 | -2.41E-03 | -8.45E-03 | -2.63E-03 | -6.59E-03 | 2.15E-03 | -5.43E-03 |
| $K00266_{VIP≥0.8\ (-)}$ | 0.885 | 0.318 | 0.923 | 0.971 | 1.125 | 0.775 | 4.25E-03 | -3.06E-03 | -8.68E-04 | -1.56E-03 | -6.88E-03 | -2.36E-03 |
| $K09516_{VIP≥0.8\ (-)}$ | 1.774 | 0.875 | 0.983 | 1.465 | 2.149 | 1.122 | 1.05E-02 | -3.25E-03 | 3.81E-03 | -6.78E-03 | -1.75E-02 | -9.47E-03 |
| $K01609_{VIP≥0.8\ (-)}$ | 0.84 | 1.393 | 1.004 | 0.94 | 0.882 | 1.074 | -1.83E-03 | 1.34E-02 | 4.52E-03 | -1.58E-03 | -6.14E-03 | -3.79E-03 |
| $K06442_{VIP≥0.8\ (-)}$ | 1.207 | 1.074 | 1.26 | 1.012 | 1.19 | 0.889 | -3.24E-03 | 5.35E-03 | 5.85E-03 | -3.01E-03 | -4.50E-03 | -2.37E-03 |
| $K11145_{VIP≥0.8\ (-)}$ | 1.129 | 1.315 | 1.129 | 1.017 | 1.408 | 0.862 | -2.63E-03 | 9.79E-03 | 5.38E-03 | -2.50E-03 | -6.49E-03 | -4.98E-05 |
| $K07742_{VIP≥0.8\ (-)}$ | 1.074 | 1.071 | 1.276 | 0.885 | 1.424 | 1.322 | -5.79E-03 | 5.46E-03 | 5.95E-03 | -3.65E-03 | -9.69E-03 | -7.83E-03 |
| $K00058_{VIP≥0.8\ (-)}$ | 0.854 | 0.877 | 0.921 | 1.081 | 1.213 | 0.826 | -2.88E-03 | 2.17E-03 | -2.82E-03 | -1.67E-03 | 6.54E-03 | -2.09E-03 |
| $K18700_{VIP≥0.8\ (-)}$ | 1.41 | 1.001 | 0.96 | 0.928 | 0.975 | 0.872 | -8.61E-03 | 8.05E-03 | 2.34E-03 | -4.61E-03 | -7.64E-03 | -7.70E-03 |
| $K03118_{VIP≥0.8\ (-)}$ | 1.306 | 0.942 | 1.215 | 1.026 | 1.342 | 0.973 | -6.72E-03 | -4.74E-03 | 4.01E-03 | -4.74E-03 | -7.69E-03 | 5.94E-03 |
| $K01726_{VIP≥0.8\ (-)}$ | 0.916 | 1.376 | 1.224 | 1.366 | 1.147 | 0.867 | -3.32E-03 | 6.09E-03 | 4.24E-03 | -6.45E-03 | -3.30E-03 | -1.83E-03 |
| $K01633_{VIP≥0.8\ (-)}$ | 1.073 | 0.961 | 1.046 | 1.308 | 1.683 | 1.142 | 4.52E-04 | 4.42E-03 | -4.84E-03 | -8.21E-03 | -1.33E-02 | -1.99E-03 |
| $K02784_{VIP≥0.8\ (-)}$ | 1.43 | 1.081 | 1.074 | 0.883 | 1.082 | 1.153 | -6.31E-03 | 2.62E-03 | 5.28E-03 | -1.63E-03 | -6.84E-03 | -8.09E-03 |
| $K00748_{VIP≥0.8\ (-)}$ | 1.13 | 0.967 | 1.547 | 1.493 | 0.873 | 1.593 | 3.42E-03 | -4.53E-03 | -7.38E-03 | -9.32E-03 | -6.21E-03 | 8.19E-03 |

| | | | | | | | | | | | | |
|---|---|---|---|---|---|---|---|---|---|---|---|---|
| $K07481_{VIP≥0.8\ (-)}$ | 1.457 | 1.009 | 0.998 | 0.89 | 0.971 | 1.084 | 9.43E-03 | 2.17E-03 | -2.18E-03 | -2.42E-03 | -1.83E-03 | -8.84E-03 |
| $K15921_{VIP≥0.8\ (-)}$ | 1.213 | 1.346 | 0.894 | 0.926 | 1.162 | 1.134 | -5.04E-03 | 4.42E-03 | 1.57E-03 | -2.38E-03 | -3.57E-03 | -1.01E-02 |
| $K13653_{VIP≥0.8\ (-)}$ | 1.185 | 0.12 | 1.564 | 0.991 | 1.025 | 2.527 | -5.41E-03 | 6.96E-04 | 6.00E-03 | -4.51E-03 | -6.70E-03 | -2.13E-02 |
| $K07248_{VIP≥0.8\ (-)}$ | 0.806 | 1.372 | 1.559 | 1.799 | 0.842 | 0.712 | -2.66E-03 | 5.87E-03 | -7.58E-03 | -1.03E-02 | 6.76E-03 | -6.33E-03 |
| $K05341_{VIP≥0.8\ (-)}$ | 0.758 | 1.036 | 0.871 | 0.958 | 1.679 | 0.876 | 3.51E-04 | -1.18E-03 | 3.80E-03 | -1.06E-03 | -1.20E-02 | -5.14E-03 |
| $K00974_{VIP≥0.8\ (-)}$ | 1.508 | 1.325 | 1.607 | 0.873 | 0.933 | 0.587 | 9.31E-03 | -5.84E-03 | -6.94E-03 | -3.33E-03 | -4.36E-03 | 1.17E-03 |
| $K06023_{VIP≥0.8\ (-)}$ | 1.387 | 1.053 | 0.732 | 0.942 | 1.139 | 1.051 | -5.62E-03 | 3.77E-03 | 3.54E-03 | -2.12E-03 | -3.45E-03 | -2.99E-03 |
| $K08309_{VIP≥0.8\ (-)}$ | 0.756 | 1.174 | 1.287 | 1.099 | 1.396 | 0.866 | 1.94E-03 | 7.47E-03 | -5.18E-03 | -3.40E-03 | -7.85E-03 | -6.50E-03 |
| $K08722_{VIP≥0.8\ (-)}$ | 0.993 | 1.066 | 0.747 | 1.101 | 1.366 | 0.912 | 3.72E-03 | 2.42E-03 | -2.04E-03 | -3.36E-03 | -6.82E-03 | -2.16E-03 |
| $K01926_{VIP≥0.8\ (-)}$ | 1.289 | 1.214 | 1.046 | 0.989 | 1.191 | 0.727 | -6.40E-03 | 9.52E-03 | 5.15E-03 | -3.47E-03 | -3.32E-03 | -9.24E-04 |
| $K03789_{VIP≥0.8\ (-)}$ | 1.391 | 1.043 | 0.743 | 0.832 | 1.051 | 1.174 | -6.21E-03 | 4.88E-03 | 3.61E-03 | -2.56E-03 | -4.39E-03 | -6.77E-03 |
| $K05832_{VIP≥0.8\ (-)}$ | 1.559 | 0.938 | 0.417 | 1.111 | 1.23 | 1.005 | -9.14E-03 | 7.54E-04 | -8.39E-04 | -3.98E-04 | 2.92E-03 | -3.40E-03 |
| $K00340_{VIP≥0.8\ (-)}$ | 1.256 | 1.57 | 0.617 | 0.804 | 0.945 | 1.293 | 4.00E-03 | 7.64E-03 | -1.38E-03 | -1.29E-03 | -4.55E-03 | -7.77E-03 |
| $K01626_{VIP≥0.8\ (-)}$ | 0.899 | 1.067 | 0.914 | 1.151 | 1.152 | 0.643 | -2.80E-03 | -2.57E-03 | -1.12E-04 | -1.70E-03 | 2.49E-03 | 4.87E-03 |
| $K06215_{VIP≥0.8\ (-)}$ | 0.492 | 1.369 | 1.088 | 0.967 | 1.362 | 0.859 | 3.19E-03 | -1.32E-02 | -4.49E-03 | -5.70E-03 | -1.06E-02 | 7.43E-03 |
| $K00761_{VIP≥0.8\ (-)}$ | 1.331 | 0.927 | 0.783 | 0.901 | 0.889 | 0.923 | -6.46E-03 | 5.59E-03 | 3.69E-03 | -2.32E-03 | -2.05E-03 | -2.19E-03 |
| $K03621_{VIP≥0.8\ (-)}$ | 1.178 | 1.275 | 0.65 | 1.147 | 1.48 | 0.913 | 3.57E-03 | -2.07E-03 | -4.21E-05 | -3.61E-03 | -7.66E-03 | 4.99E-03 |
| $K06188_{VIP≥0.8\ (-)}$ | 1.108 | 0.873 | 0.911 | 1.318 | 1.549 | 0.698 | 2.28E-03 | -5.15E-04 | 2.21E-03 | -4.46E-03 | -1.08E-02 | -2.81E-03 |
| $K04486_{VIP≥0.8\ (-)}$ | 1.317 | 1.142 | 0.435 | 1.117 | 1.607 | 0.919 | -5.47E-03 | 8.26E-03 | 1.91E-03 | -3.78E-03 | -9.62E-03 | -1.96E-03 |
| $K21498_{VIP≥0.8\ (-)}$ | 1.574 | 0.766 | 1.182 | 1.023 | 0.932 | 0.916 | 1.01E-02 | 7.45E-03 | -5.04E-03 | -6.35E-03 | -1.65E-03 | -1.93E-03 |
| $K03973_{VIP≥0.8\ (-)}$ | 0.927 | 0.624 | 1.109 | 0.969 | 0.912 | 0.986 | -2.27E-03 | -8.08E-05 | 3.17E-03 | -3.18E-03 | -3.08E-03 | 6.35E-03 |
| $K09774_{VIP≥0.8\ (-)}$ | 1.059 | 0.893 | 1.108 | 0.965 | 0.682 | 1.141 | 3.57E-03 | -7.68E-03 | -5.29E-03 | -6.08E-03 | -5.47E-03 | 1.63E-03 |
| $K00793_{VIP≥0.8\ (-)}$ | 0.873 | 0.811 | 0.975 | 1.253 | 0.153 | 1.628 | 1.92E-04 | -7.27E-04 | -4.72E-03 | -7.75E-03 | -8.00E-05 | 1.09E-02 |
| $K03771_{VIP≥0.8\ (-)}$ | 1.172 | 1.112 | 0.762 | 1.019 | 0.944 | 1.894 | 2.08E-03 | -7.33E-03 | -2.22E-03 | -5.08E-03 | -5.02E-03 | 1.11E-02 |
| $K01627_{VIP≥0.8\ (-)}$ | 1.426 | 0.706 | 0.821 | 1.052 | 0.95 | 1.316 | 6.91E-03 | -2.47E-03 | -3.80E-03 | -5.74E-03 | -7.34E-03 | 8.31E-04 |
| $K02536_{VIP≥0.8\ (-)}$ | 1.265 | 0.862 | 1.454 | 1.585 | 0.673 | 1.988 | 4.94E-03 | -3.28E-03 | -7.10E-03 | -9.72E-03 | -2.88E-03 | 1.29E-02 |
| $K11752_{VIP≥0.8\ (-)}$ | 1.445 | 0.732 | 1.561 | 1.286 | 1.029 | 1.976 | 8.85E-03 | -7.14E-03 | -6.98E-03 | -7.91E-03 | -6.44E-03 | 1.62E-02 |
| $K06041_{VIP≥0.8\ (-)}$ | 1.261 | 1.255 | 0.729 | 1.216 | 0.834 | 1.064 | 4.29E-03 | -1.22E-02 | -3.11E-03 | -7.66E-03 | -6.75E-03 | 5.18E-04 |
| $K03205_{VIP≥0.8\ (-)}$ | 1.132 | 1.028 | 0.804 | 1.165 | 1.15 | 0.618 | 3.98E-03 | -5.55E-03 | -8.83E-04 | -1.56E-03 | -6.33E-03 | 3.40E-03 |
| $K04095_{VIP≥0.8\ (-)}$ | 0.903 | 1.369 | 1.237 | 1.049 | 0.876 | 0.547 | 5.83E-03 | -1.34E-02 | -5.89E-03 | -5.07E-03 | -2.50E-03 | 4.46E-03 |

| | | | | | | | | | | | | |
|---|---|---|---|---|---|---|---|---|---|---|---|---|
| K01934$_{VIP\geq0.8\ (-)}$ | 0.983 | 1.283 | 0.857 | 1.182 | 1.403 | 0.492 | 4.93E-03 | -1.02E-02 | -3.65E-03 | -4.90E-03 | -6.43E-03 | 4.28E-03 |
| K01005$_{VIP\geq0.8\ (-)}$ | 1.221 | 0.851 | 0.582 | 1.098 | 1.352 | 1.094 | -3.96E-03 | 2.31E-03 | 1.21E-03 | -2.03E-03 | -6.58E-03 | -4.12E-03 |
| K00287$_{VIP\geq0.8\ (-)}$ | 1.375 | 1.222 | 1.055 | 1.015 | 0.216 | 1.275 | -7.44E-03 | -1.20E-02 | 3.02E-03 | -6.26E-03 | -1.48E-03 | 9.91E-03 |
| K03437$_{VIP\geq0.8\ (-)}$ | 1.156 | 1.453 | 1.066 | 0.9 | 0.747 | 0.806 | -5.91E-03 | 8.01E-03 | 3.22E-03 | -4.89E-03 | -5.09E-03 | -4.84E-03 |
| K20074$_{VIP\geq0.8\ (-)}$ | 0.81 | 1.574 | 0.995 | 0.492 | 1.776 | 1.471 | -2.45E-03 | 1.22E-02 | 2.30E-03 | -2.56E-03 | -1.44E-02 | -1.31E-02 |
| K00764$_{VIP\geq0.8\ (-)}$ | 1.14 | 0.97 | 0.792 | 1.319 | 1.722 | 2.148 | -5.42E-03 | 3.18E-03 | 3.75E-03 | -8.08E-03 | -1.37E-02 | -1.80E-02 |
| K22278$_{VIP\geq0.8\ (-)}$ | 1.01 | 0.717 | 1.029 | 1.101 | 0.897 | 1.52 | -4.23E-03 | 1.86E-03 | 2.06E-03 | -5.60E-03 | -4.77E-03 | -1.07E-02 |
| K08640$_{VIP\geq0.8\ (-)}$ | 1.16 | 0.921 | 0.569 | 0.835 | 1.131 | 0.93 | -3.81E-03 | 1.50E-04 | -8.96E-04 | -1.52E-03 | -9.10E-03 | 3.64E-04 |
| K07483$_{VIP\geq0.8\ (-)}$ | 1.584 | 1.05 | 1.722 | 0.904 | 0.946 | 0.457 | -9.94E-03 | -8.77E-03 | 6.50E-03 | -5.68E-03 | -7.68E-03 | 3.77E-03 |
| K07493$_{VIP\geq0.8\ (-)}$ | 1.149 | 1.186 | 1.122 | 1.082 | 0.856 | 0.564 | 6.39E-03 | -7.82E-03 | -2.58E-03 | -1.30E-03 | 1.05E-04 | -3.10E-03 |
| K07473$_{VIP\geq0.8\ (-)}$ | 1.627 | 0.95 | 1.154 | 1.016 | 1.045 | 0.564 | 1.05E-02 | -7.19E-05 | -3.33E-03 | -2.89E-03 | -8.17E-04 | 3.98E-03 |
| K02173$_{VIP\geq0.8\ (-)}$ | 1.096 | 1.281 | 2.46 | 1.458 | 0.661 | 0.671 | -5.93E-03 | 1.25E-02 | 1.17E-02 | -8.71E-03 | -6.12E-04 | -2.55E-03 |
| K02822$_{VIP\geq0.8\ (-)}$ | 0.899 | 1.848 | 0.236 | 0.8 | 0.831 | 1.21 | -5.20E-03 | -1.43E-02 | 7.03E-05 | 7.62E-04 | -3.21E-03 | -1.00E-02 |
| K06295$_{VIP\geq0.8\ (-)}$ | 0.666 | 1.299 | 0.127 | 1.177 | 1.514 | 1.068 | -4.30E-03 | 1.25E-02 | -5.11E-04 | -6.55E-03 | -1.17E-02 | 5.18E-03 |
| K06396$_{VIP\geq0.8\ (-)}$ | 0.47 | 1.061 | 0.288 | 0.989 | 1.979 | 0.909 | -3.00E-03 | 9.10E-03 | 7.83E-04 | -6.24E-03 | -1.59E-02 | -3.74E-04 |
| K04072$_{VIP\geq0.8\ (-)}$ | 0.608 | 0.418 | 1.62 | 0.885 | 1.201 | 0.988 | -3.24E-03 | 2.43E-03 | 7.81E-03 | -5.53E-03 | -9.80E-03 | -2.74E-04 |
| K06406$_{VIP\geq0.8\ (-)}$ | 0.64 | 1.008 | 0.473 | 1.144 | 2.036 | 1.011 | -3.92E-03 | 9.52E-03 | -2.19E-03 | -6.66E-03 | -1.64E-02 | 1.60E-03 |
| K02759$_{VIP\geq0.8\ (-)}$ | 0.836 | 0.961 | 0.696 | 1.274 | 0.893 | 0.143 | -4.47E-03 | -7.46E-03 | 1.60E-03 | -4.73E-03 | -1.93E-03 | 5.11E-04 |
| K07402$_{VIP\geq0.8\ (-)}$ | 1.088 | 1.482 | 1.208 | 1.148 | 0.751 | 0.509 | -3.96E-03 | -9.28E-03 | 4.67E-03 | 1.40E-03 | -1.20E-03 | -1.80E-03 |
| K03708$_{VIP\geq0.8\ (-)}$ | 0.17 | 1.489 | 0.799 | 1.667 | 2.281 | 1.007 | -3.25E-04 | 1.41E-02 | 3.57E-03 | -1.04E-02 | -1.85E-02 | -2.20E-03 |
| K09767$_{VIP\geq0.8\ (-)}$ | 0.708 | 1.037 | 1.695 | 0.773 | 0.847 | 1.057 | 2.12E-03 | -5.61E-03 | -7.32E-03 | -2.29E-03 | -4.64E-04 | 7.35E-03 |
| K07148$_{VIP\geq0.8\ (-)}$ | 1.531 | 0.809 | 1.042 | 0.409 | 0.839 | 0.788 | -8.83E-03 | -3.09E-04 | 3.63E-03 | 5.14E-04 | -2.64E-03 | -6.80E-03 |
| K13018$_{VIP\geq0.8\ (-)}$ | 0.866 | 0.499 | 1.048 | 1.15 | 1.268 | 0.277 | -3.42E-03 | -2.61E-03 | 1.05E-03 | -5.22E-03 | 9.58E-03 | -1.43E-03 |
| K07045$_{VIP\geq0.8\ (-)}$ | 1.437 | 0.794 | 0.841 | 0.703 | 1.364 | 2.107 | -8.12E-03 | 6.87E-03 | 1.08E-03 | -2.69E-03 | -1.01E-02 | -1.84E-02 |
| K02420$_{VIP\geq0.8\ (-)}$ | 0.416 | 0.99 | 1.325 | 1.049 | 1.069 | 0.535 | 1.82E-04 | 2.97E-03 | -4.24E-03 | -1.41E-03 | -8.58E-04 | -4.76E-03 |
| K09803$_{VIP\geq0.8\ (-)}$ | 1.631 | 0.73 | 1.123 | 0.457 | 0.808 | 0.867 | 1.02E-02 | -3.39E-04 | 5.48E-03 | -2.64E-03 | -6.94E-04 | -7.66E-03 |
| K02413$_{VIP\geq0.8\ (-)}$ | 0.631 | 0.985 | 1.061 | 1.108 | 1.062 | 0.201 | 2.39E-04 | 1.79E-03 | -2.45E-03 | -2.20E-03 | -5.82E-04 | -1.74E-03 |
| K02398$_{VIP\geq0.8\ (-)}$ | 0.594 | 0.914 | 1.177 | 1.088 | 1.085 | 0.489 | 2.62E-03 | 4.02E-03 | -3.48E-03 | -2.07E-03 | -8.26E-04 | -4.34E-03 |
| K00965$_{VIP\geq0.8\ (-)}$ | 1.198 | 0.91 | 0.64 | 1.057 | 1.205 | 0.665 | -3.26E-03 | 5.28E-03 | 1.37E-03 | -1.55E-03 | -7.12E-03 | -4.11E-03 |
| K22452$_{VIP\geq0.8\ (-)}$ | 0.559 | 0.906 | 1.371 | 1.29 | 1.222 | 0.285 | -2.56E-03 | 1.72E-03 | -5.21E-03 | -5.65E-03 | -5.84E-03 | 1.98E-04 |

| | | | | | | | | | | | | |
|---|---|---|---|---|---|---|---|---|---|---|---|---|
| K01902$_{VIP\geq0.8\ (-)}$ | 0.621 | 0.888 | 1.553 | 0.986 | 0.98 | 0.619 | 2.97E-03 | -4.32E-03 | -5.80E-03 | -1.05E-03 | 3.60E-03 | -2.10E-03 |
| K04655$_{VIP\geq0.8\ (-)}$ | 0.898 | 0.756 | 0.925 | 1.088 | 0.976 | 0.617 | -9.87E-04 | 4.19E-03 | 2.72E-03 | -1.97E-03 | -3.25E-03 | -5.32E-03 |
| K04042$_{VIP\geq0.8\ (-)}$ | 0.608 | 1.144 | 1.456 | 1.071 | 0.923 | 0.483 | 5.16E-04 | -4.86E-03 | -5.42E-03 | -1.98E-03 | -2.44E-04 | 4.28E-03 |
| K02805$_{VIP\geq0.8\ (-)}$ | 1.236 | 1.901 | 0.604 | 0.818 | 0.798 | 1.598 | -7.68E-03 | -1.69E-02 | -1.65E-03 | -1.81E-03 | 1.72E-03 | 1.42E-02 |
| K02614$_{VIP\geq0.8\ (-)}$ | 1.395 | 0.586 | 1.412 | 0.869 | 0.907 | 0.721 | -7.22E-03 | -2.15E-03 | 4.61E-03 | 1.13E-03 | -3.85E-04 | -6.40E-03 |
| K00046$_{VIP\geq0.8\ (-)}$ | 0.986 | 1.173 | 0.813 | 0.584 | 1.118 | 0.443 | 3.55E-03 | -3.04E-03 | 3.31E-03 | -7.25E-04 | -5.29E-03 | -8.33E-04 |
| K18346$_{VIP\geq0.8\ (-)}$ | 0.813 | 0.629 | 0.675 | 1.843 | 2.306 | 1.194 | -5.21E-03 | 5.11E-03 | 3.18E-03 | -1.16E-02 | -1.88E-02 | -4.81E-03 |
| K06960$_{VIP\geq0.8\ (-)}$ | 0.707 | 1.059 | 1.48 | 0.897 | 1.043 | 0.694 | -3.01E-04 | 6.67E-03 | 7.04E-03 | -4.65E-03 | -4.25E-03 | -1.07E-03 |
| K01754$_{VIP\geq0.8\ (-)}$ | 0.884 | 1.175 | 0.789 | 0.83 | 0.966 | 0.357 | 3.96E-03 | 5.86E-03 | -2.37E-03 | -6.44E-04 | -2.95E-03 | -5.32E-04 |
| K03643$_{VIP\geq0.8\ (-)}$ | 0.99 | 0.087 | 1.659 | 0.899 | 0.706 | 1.326 | 3.55E-03 | 8.48E-04 | -7.55E-03 | -5.67E-03 | -5.76E-03 | -9.42E-03 |
| K06958$_{VIP\geq0.8\ (-)}$ | 0.825 | 1.238 | 0.772 | 1.137 | 1.291 | 0.586 | -2.07E-03 | -1.71E-03 | 4.07E-04 | -2.79E-03 | -5.26E-03 | 8.75E-04 |
| K00566$_{VIP\geq0.8\ (-)}$ | 1.135 | 1.371 | 0.756 | 1.238 | 1.314 | 0.693 | 4.23E-03 | -6.50E-03 | -4.08E-04 | -4.49E-03 | -5.63E-03 | 1.46E-03 |
| K00346$_{VIP\geq0.8\ (-)}$ | 1.095 | 0.985 | 1.39 | 0.617 | 0.345 | 0.956 | 2.66E-03 | -7.40E-03 | -6.15E-03 | -3.79E-03 | -2.90E-05 | 3.60E-03 |
| K04085$_{VIP\geq0.8\ (-)}$ | 1.235 | 0.819 | 1.086 | 1.008 | 0.731 | 0.477 | 7.99E-03 | -3.07E-03 | -4.32E-03 | -5.86E-03 | 4.69E-03 | -3.34E-03 |
| K21064$_{VIP\geq0.8\ (-)}$ | 0.497 | 1.178 | 0.367 | 0.947 | 1.344 | 0.905 | -1.55E-03 | 5.43E-03 | 1.00E-03 | -4.83E-03 | -8.49E-03 | -3.84E-03 |
| K11720$_{VIP\geq0.8\ (-)}$ | 1.181 | 0.858 | 1.039 | 0.749 | 0.591 | 1.512 | 1.26E-03 | -1.75E-03 | -4.90E-03 | -4.50E-03 | -4.15E-03 | 6.90E-03 |
| K03269$_{VIP\geq0.8\ (-)}$ | 1.221 | 1.106 | 0.407 | 0.806 | 0.702 | 1.192 | 3.47E-03 | -1.81E-03 | -1.18E-03 | -2.95E-03 | 1.78E-03 | -2.18E-03 |
| K02897$_{VIP\geq0.8\ (-)}$ | 1.509 | 0.822 | 0.742 | 0.881 | 0.582 | 1.323 | 7.00E-03 | -4.43E-03 | -2.71E-03 | -4.00E-03 | -2.19E-03 | 3.29E-03 |
| K00796$_{VIP\geq0.8\ (-)}$ | 0.711 | 1.115 | 1.067 | 1.461 | 0.754 | 1.06 | -1.48E-03 | 6.19E-03 | -5.24E-03 | -9.20E-03 | -5.84E-03 | 1.30E-03 |
| K17828$_{VIP\geq0.8\ (-)}$ | 1.037 | 0.88 | 1.072 | 0.633 | 0.544 | 0.92 | -6.44E-03 | 7.02E-03 | 4.77E-03 | -3.96E-03 | -4.34E-03 | -6.62E-03 |
| K22522$_{VIP\geq0.8\ (-)}$ | 0.734 | 1.043 | 1.444 | 0.838 | 0.767 | 1.021 | -3.62E-04 | -9.62E-03 | 5.19E-03 | 4.81E-03 | -3.19E-03 | -9.07E-03 |
| K04079$_{VIP\geq0.8\ (-)}$ | 0.245 | 1.205 | 0.175 | 1.236 | 0.854 | 0.899 | 4.61E-04 | -1.19E-02 | -3.64E-04 | 6.98E-03 | -3.19E-03 | -4.08E-03 |
| K22719$_{VIP\geq0.8\ (-)}$ | 1.465 | 0.919 | 0.591 | 1.063 | 0.823 | 0.453 | 8.56E-03 | 1.74E-03 | -2.63E-03 | -6.10E-03 | -5.74E-03 | -2.60E-03 |
| K01495$_{VIP\geq0.8\ (-)}$ | 1.129 | 1.066 | 0.697 | 1.676 | 0.407 | 1.334 | -1.04E-03 | 5.72E-03 | -1.58E-03 | -9.13E-03 | -2.91E-03 | 6.55E-03 |
| K01809$_{VIP\geq0.8\ (-)}$ | 0.745 | 0.866 | 0.706 | 1.019 | 1.464 | 0.878 | -1.16E-03 | -5.30E-03 | 6.56E-05 | -5.41E-03 | 1.19E-02 | -4.68E-03 |
| K01940$_{VIP\geq0.8\ (-)}$ | 1.029 | 0.792 | 1.497 | 0.853 | 0.703 | 1.609 | 6.63E-03 | 5.58E-03 | -6.03E-03 | -5.33E-04 | -5.57E-03 | -1.37E-02 |
| K03654$_{VIP\geq0.8\ (-)}$ | 0.87 | 1.173 | 0.612 | 0.857 | 1.77 | 0.6 | -3.25E-03 | 1.40E-03 | 2.50E-03 | -5.07E-03 | -1.27E-02 | -5.20E-03 |
| K02501$_{VIP\geq0.8\ (-)}$ | 0.18 | 0.842 | 0.202 | 1.337 | 1.173 | 1.062 | -5.91E-04 | 6.78E-03 | 9.69E-04 | -8.31E-03 | -9.13E-03 | -9.37E-03 |
| K20534$_{VIP\geq0.8\ (-)}$ | 1.15 | 0.614 | 1.026 | 1.366 | 1.47 | 0.467 | -7.17E-03 | 5.85E-03 | 5.05E-03 | -8.40E-03 | -1.20E-02 | -3.23E-03 |
| K03977$_{VIP\geq0.8\ (-)}$ | 0.53 | 0.966 | 0.473 | 1.095 | 1.14 | 1.01 | 3.31E-03 | -8.76E-03 | -1.50E-03 | -2.83E-03 | -7.50E-03 | 6.08E-03 |

| | | | | | | | | | | | | |
|---|---|---|---|---|---|---|---|---|---|---|---|---|
| K03655$_{VIP≥0.8\ (-)}$ | 0.584 | 1.15 | 0.875 | 1.119 | 0.929 | 0.656 | 3.36E-03 | -9.46E-03 | -2.19E-03 | -2.96E-03 | -1.71E-03 | 3.81E-03 |
| K07221$_{VIP≥0.8\ (-)}$ | 1.117 | 1.147 | 0.647 | 0.705 | 1.502 | 1.188 | 2.38E-03 | -4.45E-03 | 6.11E-04 | -1.43E-04 | -1.05E-02 | -8.20E-03 |
| K02029$_{VIP≥0.8\ (-)}$ | 1.15 | 1.104 | 0.593 | 1.197 | 1.237 | 0.744 | -3.58E-03 | -6.34E-04 | 1.56E-03 | -2.36E-03 | -2.22E-03 | 2.27E-03 |
| K03544$_{VIP≥0.8\ (-)}$ | 0.274 | 0.107 | 1.394 | 1.488 | 1.032 | 0.808 | -1.14E-03 | -6.02E-04 | 6.76E-03 | -8.14E-03 | -7.22E-03 | 6.23E-03 |
| K03531$_{VIP≥0.8\ (-)}$ | 0.668 | 1.187 | 1.547 | 0.984 | 1.069 | 0.254 | -3.78E-03 | 5.41E-03 | 7.58E-03 | -6.06E-03 | -7.68E-03 | -2.25E-03 |
| K07568$_{VIP≥0.8\ (-)}$ | 0.828 | 0.925 | 0.546 | 1.288 | 0.883 | 0.41 | 1.97E-03 | -7.39E-03 | 1.11E-03 | -8.12E-03 | -7.17E-03 | -1.69E-03 |
| K11621$_{VIP≥0.8}$ | 0.867 | 1.099 | 1.529 | 1.111 | 1.049 | 1.197 | -5.60E-03 | 9.25E-03 | 7.08E-03 | -6.97E-03 | 8.40E-03 | -3.07E-03 |
| K07035$_{VIP≥0.8}$ | 1.059 | 2.028 | 1.576 | 0.816 | 0.917 | 0.85 | -3.66E-03 | -1.59E-02 | 7.62E-03 | 2.32E-03 | -3.17E-03 | 5.68E-03 |
| K23876$_{VIP≥0.8}$ | 1.158 | 1.007 | 0.815 | 1.401 | 1.399 | 1.187 | -5.77E-03 | -1.22E-03 | 3.63E-03 | 5.76E-03 | -9.49E-03 | 9.92E-03 |
| K07736$_{VIP≥0.8}$ | 1.17 | 1.351 | 0.9 | 1.003 | 1.088 | 0.813 | -2.50E-03 | 1.14E-02 | 4.39E-03 | -3.93E-03 | -3.61E-04 | 9.05E-05 |
| K11176$_{VIP≥0.8}$ | 1.286 | 0.938 | 1.396 | 1.034 | 0.85 | 0.867 | -4.63E-03 | 7.69E-04 | 6.84E-03 | 1.05E-03 | -3.90E-03 | -3.29E-03 |
| K23536$_{VIP≥0.8}$ | 0.949 | 2.138 | 0.865 | 1.089 | 0.878 | 0.917 | -2.20E-03 | -1.48E-02 | 3.98E-03 | -2.92E-04 | 6.90E-04 | 4.63E-03 |
| K09787$_{VIP≥0.8}$ | 1.006 | 1.145 | 0.868 | 0.99 | 1.672 | 0.86 | -1.52E-03 | 5.76E-03 | 4.26E-03 | -4.58E-03 | -1.15E-02 | 3.38E-03 |
| K09772$_{VIP≥0.8}$ | 1.043 | 1.187 | 0.865 | 1.162 | 1.587 | 0.866 | -1.31E-03 | 7.81E-03 | 3.89E-03 | -4.89E-03 | -8.34E-03 | 2.18E-03 |
| K03523$_{VIP≥0.8}$ | 1.155 | 0.988 | 0.832 | 1.183 | 1.28 | 0.814 | -3.68E-03 | 4.90E-03 | 3.69E-03 | -4.81E-03 | -5.19E-03 | 2.37E-03 |
| K03091$_{VIP≥0.8}$ | 0.94 | 1.326 | 0.834 | 1.113 | 2.146 | 0.997 | -5.95E-03 | 1.24E-02 | 3.49E-03 | -6.81E-03 | -1.69E-02 | 2.89E-03 |
| K03634$_{VIP≥0.8}$ | 1.106 | 1.581 | 1.393 | 1.437 | 1.505 | 1.557 | 4.16E-03 | 7.81E-03 | -6.73E-03 | -7.62E-03 | -1.23E-02 | 8.48E-03 |
| K05807$_{VIP≥0.8}$ | 1.19 | 1.123 | 0.952 | 1.064 | 0.887 | 1.533 | 1.06E-03 | 3.04E-03 | -4.06E-03 | -4.18E-03 | -4.59E-03 | 6.90E-03 |
| K03561$_{VIP≥0.8}$ | 1.161 | 0.862 | 1.02 | 1.348 | 0.825 | 0.909 | 4.21E-03 | -4.11E-03 | -4.41E-03 | -8.33E-03 | 6.73E-03 | 2.00E-04 |
| K07270$_{VIP≥0.8}$ | 1.686 | 1.363 | 0.962 | 1.563 | 0.786 | 3.043 | -1.00E-02 | 1.01E-02 | 5.98E-04 | -6.55E-03 | 2.63E-04 | -2.70E-02 |
| K01679$_{VIP≥0.8}$ | 1.058 | 0.922 | 1.229 | 0.938 | 0.863 | 0.682 | -5.06E-03 | -7.42E-03 | 6.03E-03 | 1.41E-03 | -2.50E-03 | 6.06E-03 |
| K01749$_{VIP≥0.8}$ | 1.086 | 1.116 | 0.261 | 0.916 | 0.923 | 0.851 | -5.49E-03 | 1.09E-02 | 5.19E-04 | -3.01E-03 | -7.40E-03 | 3.95E-03 |
| K06933$_{VIP≥0.8}$ | 1.549 | 0.935 | 1.155 | 0.926 | 0.818 | 0.339 | -8.75E-03 | 3.25E-03 | 5.68E-03 | 3.89E-04 | -4.28E-03 | -1.13E-03 |
| K21744$_{VIP≥0.8}$ | 0.947 | 1.101 | 1.111 | 0.27 | 1.774 | 2.145 | -2.00E-03 | 1.06E-02 | 5.35E-03 | 4.87E-04 | -1.44E-02 | -1.64E-02 |
| K03658$_{VIP≥0.8}$ | 1.248 | 0.583 | 1.662 | 1.311 | 0.89 | 0.893 | 7.86E-03 | 1.46E-03 | -7.28E-03 | -7.75E-03 | 3.73E-03 | -7.39E-03 |
| K06898$_{VIP≥0.8}$ | 1.16 | 0.959 | 0.929 | 0.965 | 0.949 | 0.454 | -2.78E-03 | 9.26E-03 | 4.29E-03 | -1.04E-03 | -3.72E-03 | 3.88E-03 |
| K06972$_{VIP≥0.8}$ | 1.191 | 0.846 | 0.913 | 1.13 | 0.929 | 0.784 | -5.69E-03 | -2.25E-03 | 2.90E-03 | -1.92E-03 | 4.85E-04 | 2.62E-03 |
| K19689$_{VIP≥0.8}$ | 1.159 | 1.197 | 0.574 | 1.157 | 1.151 | 0.81 | -4.88E-03 | -7.10E-03 | 7.15E-04 | -7.58E-04 | 2.20E-03 | 4.95E-03 |
| K01267$_{VIP≥0.8}$ | 0.874 | 1.047 | 1.396 | 1.117 | 1.082 | 0.525 | -4.02E-03 | 7.14E-03 | 6.60E-03 | 2.05E-03 | -4.07E-03 | -2.88E-03 |
| K02203$_{VIP≥0.8}$ | 1.05 | 1.051 | 1.598 | 0.841 | 1.087 | 0.673 | -2.78E-03 | 3.33E-03 | 7.87E-03 | -1.61E-03 | -5.29E-03 | 3.30E-03 |

| | | | | | | | | | | | | |
|---|---|---|---|---|---|---|---|---|---|---|---|---|
| K07023$_{VIP\geq0.8}$ | 1.263 | 1.384 | 0.637 | 0.93 | 1.255 | 1.269 | -5.46E-03 | 1.25E-02 | 3.14E-03 | 2.63E-03 | -6.27E-03 | -8.59E-03 |
| K03742$_{VIP\geq0.8}$ | 1.885 | 1.287 | 0.896 | 0.96 | 0.468 | 1.329 | 1.21E-02 | -9.76E-03 | -1.95E-03 | -2.12E-03 | 1.17E-03 | 1.13E-02 |
| K04654$_{VIP\geq0.8}$ | 1.046 | 0.858 | 0.887 | 1.103 | 0.961 | 0.525 | 1.16E-03 | 6.18E-03 | 2.94E-03 | -2.35E-03 | -3.74E-03 | -4.41E-03 |
| K00797$_{VIP\geq0.8}$ | 1.546 | 1.085 | 0.951 | 0.954 | 1.215 | 0.669 | 9.82E-03 | 6.17E-03 | -2.33E-03 | -3.41E-03 | -4.05E-03 | 5.83E-03 |
| K19119$_{VIP\geq0.8}$ | 1.04 | 0.83 | 1.1 | 1.077 | 1.178 | 0.167 | 5.08E-03 | 1.99E-03 | -1.42E-03 | -1.98E-03 | -3.32E-03 | 4.45E-04 |
| K04653$_{VIP\geq0.8}$ | 0.951 | 1.029 | 1.08 | 1.048 | 0.87 | 0.167 | 2.80E-03 | 6.79E-03 | 4.97E-03 | -3.62E-03 | -3.08E-03 | -1.48E-03 |
| K02027$_{VIP\geq0.8}$ | 1.301 | 0.831 | 0.878 | 1.176 | 0.897 | 0.727 | -5.28E-03 | 6.30E-03 | 3.74E-03 | 1.68E-04 | -3.40E-03 | -3.68E-03 |
| K08602$_{VIP\geq0.8}$ | 0.997 | 1.601 | 0.532 | 1.063 | 1.019 | 0.968 | -1.42E-03 | -9.32E-03 | 3.70E-04 | -2.14E-03 | 1.15E-03 | 5.06E-03 |
| K19159$_{VIP\geq0.8}$ | 1.933 | 0.955 | 1.292 | 0.951 | 0.919 | 0.78 | 1.25E-02 | -3.25E-03 | -4.17E-03 | -2.96E-03 | 7.72E-04 | 5.20E-03 |
| K06885$_{VIP\geq0.8}$ | 1.036 | 1.314 | 1.097 | 0.863 | 1.216 | 0.453 | -1.18E-03 | 2.91E-03 | 2.28E-03 | 2.51E-03 | -2.61E-03 | -1.31E-03 |
| K00549$_{VIP\geq0.8}$ | 0.814 | 0.655 | 0.838 | 0.822 | 1.381 | 0.82 | -2.66E-03 | 6.46E-03 | 1.59E-03 | -1.30E-03 | 1.06E-02 | -5.81E-03 |
| K21029$_{VIP\geq0.8}$ | 1.466 | 1.1 | 0.8 | 1.289 | 1.141 | 0.324 | -8.39E-03 | 9.14E-03 | -3.82E-03 | -7.59E-03 | 9.21E-03 | 1.93E-03 |
| K03705$_{VIP\geq0.8}$ | 1.078 | 1.101 | 0.77 | 1.138 | 1.606 | 0.94 | -1.85E-03 | 5.52E-03 | 3.13E-03 | -3.64E-03 | -9.15E-03 | 1.77E-03 |
| K03216$_{VIP\geq0.8}$ | 1.291 | 1.143 | 0.612 | 0.962 | 1.332 | 0.915 | -4.42E-03 | 6.38E-03 | 3.01E-03 | -4.72E-03 | -7.76E-03 | 9.01E-04 |
| K06213$_{VIP\geq0.8}$ | 1.087 | 1.028 | 0.608 | 0.939 | 1.242 | 0.852 | -1.17E-03 | 4.28E-03 | 2.75E-03 | 1.38E-03 | -5.63E-03 | -1.02E-03 |
| K02315$_{VIP\geq0.8}$ | 0.917 | 0.764 | 0.917 | 0.993 | 1.551 | 0.997 | -4.79E-03 | 5.62E-03 | 4.49E-03 | 2.37E-03 | -1.13E-02 | -4.37E-03 |
| K06024$_{VIP\geq0.8}$ | 1.121 | 1.176 | 1.197 | 1.025 | 1.256 | 0.766 | -1.09E-03 | 7.66E-03 | 5.74E-03 | -2.81E-03 | -4.06E-03 | 4.17E-04 |
| K03183$_{VIP\geq0.8}$ | 1.022 | 1.208 | 0.7 | 0.801 | 0.874 | 1.203 | 1.61E-03 | 1.33E-03 | -1.51E-03 | -4.65E-04 | -8.96E-04 | 4.88E-03 |
| K01246$_{VIP\geq0.8}$ | 1.071 | 0.925 | 1.282 | 0.968 | 0.882 | 0.783 | 5.95E-03 | -3.81E-03 | -4.26E-03 | -5.12E-05 | 9.03E-04 | 6.93E-03 |
| K00703$_{VIP\geq0.8}$ | 1.184 | 1.323 | 0.473 | 0.929 | 1.074 | 0.978 | -5.54E-03 | -1.16E-03 | 1.33E-03 | -4.89E-03 | 3.07E-03 | 5.49E-03 |
| K09474$_{VIP\geq0.8}$ | 0.893 | 0.883 | 0.99 | 1.241 | 1.477 | 0.497 | 3.57E-04 | 2.95E-03 | 3.28E-03 | -3.71E-03 | -8.79E-03 | -1.33E-03 |
| K04751$_{VIP\geq0.8}$ | 0.832 | 1.099 | 1.501 | 1.006 | 0.859 | 0.138 | 5.06E-03 | 1.05E-02 | -6.19E-03 | -2.86E-03 | 2.34E-03 | -8.90E-04 |
| K23675$_{VIP\geq0.8}$ | 1.121 | 0.937 | 0.727 | 1.161 | 1.108 | 0.972 | -2.10E-03 | 2.96E-03 | 2.84E-03 | 2.41E-03 | -2.41E-03 | -4.29E-03 |
| K05808$_{VIP\geq0.8}$ | 0.843 | 1.159 | 0.749 | 1.217 | 1.37 | 0.894 | 4.14E-04 | 2.65E-03 | -1.83E-03 | -3.79E-03 | -4.14E-03 | 6.74E-03 |
| K06204$_{VIP\geq0.8}$ | 1.179 | 1.124 | 1.042 | 1.233 | 0.682 | 1.221 | -8.02E-04 | 8.13E-03 | -3.64E-03 | -5.47E-03 | 1.60E-03 | 5.40E-03 |
| K09922$_{VIP\geq0.8}$ | 1.374 | 0.837 | 0.652 | 1.139 | 0.819 | 1.535 | 5.55E-03 | -7.34E-03 | -1.08E-03 | -4.25E-03 | 1.60E-03 | 1.07E-02 |
| K00992$_{VIP\geq0.8}$ | 1.301 | 1.113 | 1.438 | 0.44 | 0.873 | 1.114 | 4.67E-03 | 2.79E-03 | -7.07E-03 | -2.22E-03 | -5.37E-03 | 3.58E-04 |
| K02548$_{VIP\geq0.8}$ | 1.336 | 0.999 | 1.457 | 2.074 | 0.72 | 1.472 | 4.36E-03 | 2.38E-03 | -7.15E-03 | -1.26E-02 | -5.43E-03 | 9.01E-03 |
| K06199$_{VIP\geq0.8}$ | 0.733 | 1.072 | 1.163 | 0.925 | 1.069 | 0.857 | 4.40E-03 | -3.05E-03 | -2.67E-03 | 6.95E-04 | -1.54E-03 | 5.26E-03 |
| K00979$_{VIP\geq0.8}$ | 1.122 | 0.538 | 1.062 | 1.291 | 1.38 | 1.439 | 4.40E-03 | 2.22E-04 | -5.22E-03 | -8.04E-03 | -1.13E-02 | 4.78E-03 |

| | | | | | | | | | | | | |
|---|---|---|---|---|---|---|---|---|---|---|---|---|
| $K06076_{VIP≥0.8}$ | 1.267 | 1.35 | 0.646 | 0.96 | 1.065 | 1.062 | -3.51E-03 | 2.03E-03 | 3.21E-04 | -1.77E-03 | 1.23E-03 | -7.98E-03 |
| $K03525_{VIP≥0.8}$ | 0.94 | 2.006 | 1.811 | 0.801 | 0.649 | 1.71 | -3.78E-03 | -1.89E-02 | 8.75E-03 | -3.01E-03 | 2.17E-03 | 1.35E-02 |
| $K01218_{VIP≥0.8}$ | 1.08 | 1.08 | 0.987 | 0.542 | 1.326 | 1.496 | 1.71E-03 | -1.02E-03 | 2.29E-03 | 7.35E-04 | -6.65E-03 | -1.32E-02 |
| $K05837_{VIP≥0.8}$ | 1.104 | 1.009 | 0.986 | 0.879 | 0.246 | 1.48 | 6.41E-04 | -7.88E-03 | -4.85E-03 | -5.52E-03 | 1.40E-03 | 1.09E-02 |
| $K18843_{VIP≥0.8}$ | 1.047 | 0.916 | 0.919 | 1.133 | 1.048 | 0.534 | 5.26E-03 | 5.95E-03 | -6.72E-04 | -2.76E-03 | -4.10E-03 | 4.29E-03 |
| $K21571_{VIP≥0.8}$ | 0.977 | 0.824 | 0.443 | 1.368 | 0.901 | 1.421 | 5.65E-03 | -2.22E-03 | 1.79E-03 | 8.48E-03 | -6.83E-03 | -1.17E-02 |
| $K12340_{VIP≥0.8}$ | 1.184 | 1.175 | 0.76 | 0.803 | 1.461 | 0.91 | -6.49E-03 | 1.40E-03 | 8.59E-04 | -2.93E-03 | -7.50E-03 | 1.77E-04 |
| $K02483_{VIP≥0.8}$ | 0.879 | 0.892 | 0.85 | 1.21 | 1.155 | 0.511 | 2.49E-03 | 1.57E-03 | -1.68E-03 | -2.79E-03 | -2.24E-03 | 2.74E-04 |
| $K02030_{VIP≥0.8}$ | 1.65 | 0.996 | 0.559 | 1.17 | 1.258 | 0.904 | -9.34E-03 | 8.05E-03 | 2.18E-03 | -1.45E-03 | -3.64E-03 | 6.48E-03 |
| $K07485_{VIP≥0.8}$ | 0.892 | 0.932 | 1.125 | 1.299 | 1.136 | 0.389 | 5.40E-03 | -3.69E-03 | -3.51E-03 | 2.82E-03 | 3.80E-03 | -3.36E-03 |
| $K07133_{VIP≥0.8}$ | 1.786 | 0.399 | 1.188 | 0.992 | 1.027 | 1.075 | 1.16E-02 | -2.32E-03 | -1.86E-03 | -2.25E-03 | 5.40E-03 | 8.44E-03 |
| $K13599_{VIP≥0.8}$ | 0.853 | 0.524 | 0.981 | 0.545 | 0.978 | 1.457 | -2.41E-03 | -5.16E-03 | 1.66E-03 | -2.17E-03 | 7.30E-03 | 1.25E-02 |
| $K23356_{VIP≥0.8}$ | 1.065 | 1.294 | 0.119 | 0.202 | 1.582 | 1.563 | -5.48E-03 | -9.96E-03 | 5.82E-04 | -4.47E-05 | 1.29E-02 | 1.36E-02 |
| $K03791_{VIP≥0.8}$ | 1.469 | 0.702 | 1.538 | 0.666 | 1.079 | 1.058 | -9.31E-03 | 4.75E-04 | -7.36E-03 | 9.96E-04 | -5.74E-03 | 3.19E-03 |
| $K23997_{VIP≥0.8}$ | 1.136 | 0.566 | 1.023 | 1.369 | 0.98 | 0.716 | -4.74E-03 | -3.09E-03 | 5.01E-03 | 5.18E-03 | 6.21E-03 | -4.32E-03 |
| $K19411_{VIP≥0.8}$ | 0.35 | 1.306 | 0.627 | 1.044 | 1.897 | 0.82 | 1.94E-03 | 1.26E-02 | 2.19E-03 | -6.21E-03 | -1.52E-02 | -2.91E-03 |
| $K01193_{VIP≥0.8}$ | 1.469 | 0.809 | 0.454 | 0.87 | 1.211 | 0.44 | -7.92E-03 | 7.43E-03 | 2.23E-03 | 1.01E-03 | -8.88E-03 | -2.66E-03 |
| $K06407_{VIP≥0.8}$ | 0.663 | 0.84 | 0.521 | 1.175 | 2.042 | 0.856 | -4.14E-03 | 6.05E-03 | 8.51E-04 | -7.41E-03 | -1.66E-02 | 1.61E-03 |
| $K23004_{VIP≥0.8}$ | 1.185 | 1.013 | 0.645 | 1.16 | 1.953 | 0.566 | -6.55E-03 | 4.84E-03 | 5.33E-04 | -5.31E-03 | -1.52E-02 | 8.08E-04 |
| $K18122_{VIP≥0.8}$ | 0.388 | 1.796 | 0.588 | 0.858 | 1.014 | 2.078 | -2.49E-03 | -1.59E-02 | 2.84E-03 | -8.93E-04 | 3.94E-03 | 1.85E-02 |
| $K05364_{VIP≥0.8}$ | 1.252 | 0.623 | 0.856 | 1.09 | 1.125 | 0.511 | -6.53E-03 | 1.39E-03 | 1.90E-03 | -2.34E-03 | 4.76E-03 | -9.19E-04 |
| $K09779_{VIP≥0.8}$ | 0.611 | 0.885 | 0.585 | 1.33 | 1.559 | 0.846 | -3.77E-03 | 7.60E-03 | 2.23E-04 | -8.09E-03 | -1.27E-02 | 4.63E-03 |
| $K09769_{VIP≥0.8}$ | 0.413 | 0.819 | 0.496 | 1 | 1.732 | 0.962 | 2.22E-03 | 9.00E-04 | -2.07E-04 | -5.60E-03 | -1.33E-02 | 2.83E-03 |
| $K01512_{VIP≥0.8}$ | 0.846 | 0.914 | 0.685 | 0.833 | 1.658 | 0.191 | -2.86E-03 | 3.87E-03 | 2.62E-03 | -5.25E-03 | -1.27E-02 | 1.69E-03 |
| $K02456_{VIP≥0.8}$ | 1.128 | 0.792 | 1.706 | 0.868 | 1.036 | 0.685 | 7.13E-03 | -2.64E-04 | -7.55E-03 | -2.54E-03 | 2.65E-04 | 6.78E-04 |
| $K06864_{VIP≥0.8}$ | 1.215 | 0.816 | 0.881 | 1.062 | 0.662 | 0.256 | -4.49E-03 | 7.98E-03 | 3.27E-03 | -4.52E-03 | -7.00E-05 | 9.19E-05 |
| $K16053_{VIP≥0.8}$ | 0.806 | 1.196 | 0.399 | 1.054 | 1.596 | 0.792 | 1.48E-03 | 9.58E-04 | 1.96E-03 | -5.05E-03 | -1.18E-02 | -1.66E-03 |
| $K00872_{VIP≥0.8}$ | 0.756 | 0.863 | 1.065 | 1.088 | 0.743 | 1.106 | 1.56E-03 | 2.26E-03 | -2.57E-03 | -3.09E-03 | -2.29E-04 | 9.07E-03 |
| $K01728_{VIP≥0.8}$ | 1.028 | 1.082 | 0.515 | 0.805 | 1.228 | 0.581 | 2.02E-03 | 2.94E-03 | -1.49E-04 | -5.18E-04 | 8.40E-03 | -5.01E-03 |
| $K06940_{VIP≥0.8}$ | 0.888 | 0.726 | 0.532 | 1.096 | 1.017 | 1.771 | -8.68E-04 | 2.75E-03 | -1.50E-03 | -3.28E-03 | 1.66E-04 | 1.56E-02 |

| | | | | | | | | | | | | |
|---|---|---|---|---|---|---|---|---|---|---|---|---|
| K09807$_{VIP\geq0.8}$ | 0.634 | 0.843 | 1.182 | 1.152 | 1.003 | 0.289 | 3.61E-05 | 3.69E-03 | -3.57E-03 | -3.87E-03 | -9.64E-04 | 2.55E-03 |
| K00995$_{VIP\geq0.8}$ | 0.949 | 1.261 | 0.796 | 1.18 | 1.505 | 0.8 | 4.84E-03 | 1.28E-03 | -2.21E-03 | -4.40E-03 | -7.37E-03 | 1.42E-03 |
| K02025$_{VIP\geq0.8}$ | 1.336 | 0.8 | 1.089 | 1.164 | 0.943 | 0.579 | -6.18E-03 | 2.89E-03 | 4.84E-03 | -1.39E-04 | -1.60E-03 | 8.22E-04 |
| K03770$_{VIP\geq0.8}$ | 1.223 | 1.021 | 0.627 | 0.441 | 0.834 | 1.331 | 3.85E-03 | -3.05E-03 | -2.70E-03 | -2.63E-03 | 5.48E-03 | 8.47E-03 |
| K01817$_{VIP\geq0.8}$ | 1.115 | 1.16 | 0.487 | 1.217 | 1.01 | 0.545 | 7.17E-03 | 9.79E-03 | 2.09E-03 | -6.82E-03 | -7.49E-03 | -4.22E-03 |
| K01144$_{VIP\geq0.8}$ | 1.289 | 1.564 | 0.447 | 0.641 | 0.954 | 1.182 | 5.96E-03 | 5.47E-03 | -4.19E-04 | -1.11E-04 | -2.44E-03 | 6.99E-03 |
| K00350$_{VIP\geq0.8}$ | 1.079 | 1.157 | 1.771 | 0.601 | 0.247 | 1.25 | 1.74E-03 | 4.65E-03 | -8.43E-03 | -3.10E-03 | -1.05E-03 | 7.79E-03 |
| K08591$_{VIP\geq0.8}$ | 1.139 | 1.22 | 0.71 | 0.977 | 1.365 | 0.7 | -9.99E-04 | 1.56E-03 | 3.13E-03 | -1.13E-03 | -6.57E-03 | 5.66E-04 |
| K03060$_{VIP\geq0.8}$ | 0.907 | 1.234 | 0.478 | 1.108 | 1.325 | 0.775 | 4.26E-03 | 6.72E-03 | -1.46E-03 | -4.39E-03 | -5.36E-03 | 5.94E-03 |
| K00912$_{VIP\geq0.8}$ | 1.029 | 1.23 | 0.763 | 1.06 | 0.472 | 1.572 | 1.51E-03 | 5.39E-04 | -3.16E-03 | -5.96E-03 | -2.29E-04 | 9.70E-03 |
| K02065$_{VIP\geq0.8}$ | 1.392 | 0.994 | 1.159 | 0.55 | 0.554 | 1.325 | 5.76E-03 | -9.33E-03 | -5.62E-03 | -3.11E-03 | 9.72E-04 | 7.19E-03 |
| K01610$_{VIP\geq0.8}$ | 0.965 | 0.846 | 0.816 | 0.961 | 0.339 | 0.509 | 5.72E-03 | -8.17E-03 | 2.85E-05 | -9.63E-05 | 1.38E-03 | -1.53E-03 |
| K02346$_{VIP\geq0.8}$ | 1.017 | 0.796 | 1 | 1.01 | 0.243 | 0.981 | -5.87E-03 | -7.64E-03 | 3.58E-03 | 2.15E-03 | 7.74E-04 | -7.74E-03 |
| K17103$_{VIP\geq0.8}$ | 1.063 | 0.749 | 0.874 | 0.468 | 0.9 | 1.348 | -1.18E-03 | -1.96E-03 | 1.91E-03 | -1.10E-03 | 3.99E-03 | 9.26E-03 |
| K01176$_{VIP\geq0.8}$ | 1.079 | 0.958 | 0.728 | 0.824 | 0.227 | 0.976 | 6.95E-03 | 3.87E-03 | -3.38E-03 | -5.19E-03 | 1.55E-03 | -8.61E-03 |
| K03787$_{VIP\geq0.8}$ | 0.784 | 1.042 | 1.153 | 0.996 | 0.792 | 1.131 | 1.18E-03 | -3.82E-03 | 3.22E-03 | -5.14E-03 | -2.33E-03 | 4.90E-03 |
| K09117$_{VIP\geq0.8}$ | 0.773 | 1.171 | 0.578 | 0.864 | 0.916 | 1.325 | 4.80E-03 | -6.42E-03 | -9.44E-05 | -3.12E-03 | 7.18E-03 | 1.09E-02 |
| K03092$_{VIP\geq0.8}$ | 1.316 | 1.094 | 1.339 | 0.328 | 0.276 | 1.362 | 5.47E-03 | 8.07E-04 | -6.41E-03 | -1.62E-03 | -1.62E-03 | 5.40E-03 |
| K19005$_{VIP\geq0.8}$ | 1.232 | 1.183 | 0.764 | 0.961 | 1.01 | 0.563 | -6.73E-03 | 2.22E-04 | -3.05E-03 | -5.19E-03 | 2.27E-03 | 3.92E-03 |
| K19157$_{VIP\geq0.8}$ | 1.013 | 0.357 | 0.334 | 0.952 | 1.097 | 1.179 | 4.55E-03 | 1.74E-03 | -1.46E-03 | -6.00E-03 | -8.83E-03 | 6.94E-03 |
| K03585$_{VIP\geq0.8}$ | 0.921 | 1.209 | 0.556 | 1.117 | 0.609 | 1.325 | 5.19E-05 | 2.28E-03 | -2.37E-03 | -5.93E-03 | -3.29E-03 | 5.55E-03 |
| K01808$_{VIP\geq0.8}$ | 0.112 | 0.805 | 1.792 | 1.145 | 1.086 | 0.261 | 6.34E-04 | -6.77E-03 | 7.79E-03 | -7.21E-03 | -8.63E-03 | 1.75E-03 |
| K13002$_{VIP\geq0.8}$ | 1.01 | 1.18 | 0.806 | 0.442 | 0.722 | 1.045 | 1.23E-03 | 2.07E-03 | 3.13E-03 | -3.55E-04 | -3.17E-04 | -7.92E-03 |
| K00950$_{VIP\geq0.8}$ | 1.184 | 1.13 | 0.561 | 0.779 | 1.131 | 1.471 | 1.55E-03 | 6.15E-03 | -2.60E-03 | -3.39E-03 | -8.10E-03 | 5.36E-03 |
| K07727$_{VIP\geq0.8}$ | 1.226 | 1.033 | 1.545 | 0.67 | 0.623 | 0.897 | -7.66E-03 | 1.02E-02 | 6.42E-03 | -3.93E-03 | -2.71E-03 | 6.70E-03 |
| K03630$_{VIP\geq0.8}$ | 0.636 | 0.887 | 0.784 | 0.942 | 1.408 | 1.337 | -4.10E-03 | 3.12E-05 | 3.07E-03 | -4.30E-03 | -1.13E-02 | 1.18E-02 |
| K03574$_{VIP\geq0.8}$ | 1.291 | 1.555 | 0.795 | 0.98 | 0.47 | 1.231 | 6.46E-03 | 8.82E-03 | -1.68E-03 | -5.87E-03 | -3.34E-03 | 6.49E-03 |
| K03111$_{VIP\geq0.8}$ | 0.538 | 0.406 | 1.248 | 1.356 | 1.187 | 1.772 | 2.90E-03 | -6.60E-04 | 4.38E-03 | -8.23E-03 | -7.65E-03 | 1.57E-02 |
| K14102 | 0.484 | 0.925 | 1.14 | 0.676 | 1.203 | 0.46 | 3.11E-03 | 7.01E-03 | 2.98E-03 | 7.04E-04 | 7.37E-03 | -1.36E-03 |
| K03053 | 0.732 | 0.723 | 1.506 | 0.636 | 0.875 | 1.117 | 3.95E-03 | 4.37E-03 | 5.43E-03 | 6.20E-05 | 3.90E-03 | -9.30E-03 |
| K07569 | 0.379 | 1.268 | 1.049 | 0.787 | 1.35 | 0.415 | 7.56E-04 | 1.08E-02 | 1.55E-03 | 3.76E-03 | 9.24E-03 | -2.70E-03 |

| | | | | | | | | | | | | |
|---|---|---|---|---|---|---|---|---|---|---|---|---|
| K03050 | 0.643 | 0.946 | 1.602 | 0.614 | 0.967 | 0.319 | 1.98E-03 | 7.75E-03 | 5.90E-03 | 1.45E-03 | 5.55E-03 | 4.93E-04 |
| K14118 | 0.574 | 1.188 | 1.161 | 0.697 | 0.902 | 0.32 | 3.57E-03 | 9.43E-03 | 3.82E-03 | 2.55E-03 | 3.67E-03 | 1.02E-03 |
| K23264 | 0.088 | 1.464 | 1.827 | 0.749 | 1 | 0.533 | 4.23E-04 | 1.19E-02 | 7.25E-03 | -3.75E-03 | 7.21E-03 | 3.62E-03 |
| K14119 | 0.687 | 1.273 | 1.534 | 0.702 | 0.964 | 0.38 | 3.21E-03 | 1.09E-02 | 5.74E-03 | 2.39E-03 | 5.20E-03 | -3.00E-03 |
| K07503 | 0.716 | 1.903 | 1.893 | 0.679 | 1.295 | 0.603 | -3.87E-03 | 1.57E-02 | 8.04E-03 | 2.60E-03 | 9.93E-03 | 8.82E-04 |
| K07387 | 1.3 | 0.797 | 1.172 | 0.882 | 0.649 | 0.501 | -7.39E-03 | 4.63E-03 | 4.10E-03 | -5.41E-03 | 3.04E-03 | 2.31E-03 |
| K02009 | 0.348 | 1.021 | 1.471 | 0.684 | 0.914 | 0.453 | -6.25E-04 | 7.54E-03 | 4.66E-03 | 1.22E-03 | 4.67E-03 | -1.35E-03 |
| K02781 | 0.718 | 0.958 | 0.269 | 0.587 | 1.16 | 1.195 | 4.31E-03 | 7.52E-03 | 1.12E-03 | -1.70E-03 | -9.34E-03 | -1.06E-02 |
| K01780 | 0.911 | 0.429 | 1.258 | 0.529 | 0.606 | 0.911 | -5.33E-03 | 3.97E-03 | 3.45E-03 | -1.66E-03 | 3.62E-03 | 7.09E-03 |
| K21993 | 0.477 | 0.378 | 1.153 | 0.677 | 1.33 | 1.111 | 3.07E-04 | 2.92E-03 | 4.06E-03 | 1.10E-03 | 6.93E-03 | -8.01E-03 |
| K00441 | 0.314 | 0.596 | 1.152 | 0.621 | 0.928 | 0.836 | 2.19E-04 | -7.91E-04 | -5.40E-03 | -3.61E-03 | 4.15E-03 | 6.24E-03 |
| K00563 | 0.726 | 0.917 | 0.507 | 0.588 | 1.734 | 0.867 | 1.92E-03 | -4.35E-03 | 2.34E-03 | 1.19E-03 | -1.41E-02 | -7.26E-03 |
| K02499 | 0.393 | 0.889 | 2.007 | 0.537 | 0.405 | 1.046 | 2.68E-04 | 8.76E-03 | 9.82E-03 | -2.41E-03 | 3.25E-03 | 9.06E-03 |
| K01118 | 0.64 | 0.252 | 0.957 | 1.405 | 0.696 | 1.427 | 2.00E-03 | 2.39E-03 | 1.34E-03 | -7.38E-03 | -5.67E-03 | -1.23E-02 |
| K13566 | 0.839 | 0.854 | 0.374 | 1.129 | 0.75 | 0.683 | 3.51E-03 | -4.86E-03 | 1.84E-03 | 6.33E-03 | 5.99E-03 | -4.73E-03 |
| K01575 | 0.971 | 0.236 | 0.797 | 1.042 | 0.658 | 2.577 | -6.22E-03 | 2.33E-03 | -3.08E-03 | -6.54E-03 | -5.35E-03 | -2.23E-02 |
| K00887 | 0.958 | 0.966 | 0.509 | 1.601 | 0.642 | 0.768 | 1.51E-03 | 1.68E-03 | 1.06E-03 | 7.23E-03 | -3.14E-03 | -9.94E-04 |
| K22928 | 1.179 | 1.373 | 0.669 | 0.533 | 0.768 | 0.881 | 7.22E-03 | 1.26E-02 | -3.19E-03 | -2.44E-03 | -2.70E-03 | 7.57E-03 |
| K03750 | 0.354 | 1.31 | 0.966 | 0.369 | 0.883 | 0.711 | -1.64E-03 | 9.59E-03 | 2.63E-03 | -2.33E-03 | 5.55E-03 | -2.48E-03 |
| K06012 | 0.669 | 0.698 | 0.657 | 0.958 | 1.988 | 1.132 | -4.21E-03 | 5.15E-03 | -2.74E-03 | -5.81E-03 | -1.61E-02 | 4.18E-03 |
| K03623 | 0.624 | 1.038 | 1.743 | 0.719 | 0.753 | 0.929 | -2.81E-03 | 1.02E-02 | 7.20E-03 | -8.73E-04 | 4.07E-03 | -6.88E-03 |
| K01496 | 0.478 | 0.87 | 1.305 | 0.602 | 0.214 | 1.128 | -1.27E-04 | 8.41E-03 | 6.40E-03 | 3.09E-03 | 1.21E-03 | -7.34E-03 |
| K06985 | 0.755 | 1.391 | 0.285 | 0.515 | 0.95 | 1.831 | -5.98E-04 | 9.99E-03 | -3.41E-04 | -3.11E-03 | 7.06E-03 | 1.60E-02 |
| K01646 | 1.197 | 1.593 | 0.38 | 0.854 | 0.7 | 0.711 | 7.53E-03 | 1.53E-02 | -1.87E-03 | -5.37E-03 | -5.07E-03 | -4.86E-03 |
| K04088 | 0.245 | 1.383 | 2.067 | 0.566 | 0.958 | 0.563 | 4.99E-04 | -7.97E-03 | -9.64E-03 | -1.16E-03 | -5.93E-03 | -4.30E-03 |
| K03299 | 1.285 | 1.304 | 0.375 | 0.503 | 0.292 | 1.36 | -6.60E-03 | 1.25E-02 | 8.17E-04 | 2.89E-03 | -1.50E-03 | -1.21E-02 |
| K01085 | 0.643 | 1.24 | 0.696 | 0.605 | 1.219 | 1.032 | -2.80E-03 | -1.22E-02 | 2.70E-03 | -9.91E-04 | -9.89E-03 | -8.79E-03 |
| K04047 | 0.517 | 1.462 | 0.724 | 1.113 | 0.858 | 0.648 | -1.70E-03 | -1.02E-02 | -6.28E-04 | 1.14E-03 | 4.81E-04 | -5.47E-03 |
| K01201 | 0.206 | 0.901 | 0.526 | 2.076 | 0.777 | 1.644 | 5.20E-04 | -8.37E-03 | 1.72E-03 | 1.23E-02 | -6.35E-03 | 1.46E-02 |
| K05305 | 1.059 | 1.318 | 0.767 | 0.689 | 1.192 | 0.446 | -6.63E-03 | 9.81E-03 | 2.67E-03 | 3.42E-03 | -9.57E-03 | -3.71E-03 |
| K00756 | 0.571 | 1.976 | 0.492 | 0.815 | 1.008 | 0.58 | -3.52E-03 | -1.05E-02 | 2.40E-03 | -5.02E-03 | 4.57E-03 | 2.91E-03 |
| K00275 | 0.742 | 0.998 | 1.523 | 0.754 | 1.134 | 0.423 | 4.76E-03 | 5.34E-04 | -6.87E-03 | -1.18E-03 | -2.84E-03 | -2.02E-03 |
| K22477 | 0.925 | 0.902 | 0.14 | 0.67 | 0.266 | 1.408 | -4.70E-03 | 5.60E-03 | 4.10E-04 | -4.10E-03 | 1.62E-03 | -1.16E-02 |
| K04656 | 0.906 | 0.486 | 0.755 | 1.093 | 0.886 | 0.405 | -1.19E-03 | -1.16E-03 | -8.30E-04 | -1.45E-03 | 4.26E-04 | 1.69E-03 |

| | | | | | | | | | | | | |
|---|---|---|---|---|---|---|---|---|---|---|---|---|
| K01255 | 1.043 | 0.69 | 2.059 | 0.848 | 0.76 | 0.757 | 5.77E-03 | -1.02E-03 | -8.98E-03 | -4.14E-03 | 6.96E-04 | -4.99E-03 |
| K05799 | 1.094 | 0.725 | 1.743 | 1.123 | 0.653 | 0.78 | -6.89E-03 | 2.35E-03 | 7.51E-03 | 6.31E-03 | -2.81E-03 | -1.88E-03 |
| K05794 | 2.144 | 0.545 | 0.82 | 1.066 | 0.544 | 0.587 | -1.36E-02 | -2.44E-03 | 4.54E-04 | -4.98E-03 | -5.05E-04 | 7.34E-04 |
| K02007 | 1.048 | 0.846 | 0.656 | 0.747 | 0.695 | 0.887 | -1.70E-03 | -5.37E-03 | 2.91E-03 | 8.83E-04 | 4.95E-03 | 7.88E-03 |
| K10532 | 0.414 | 1.457 | 0.705 | 0.996 | 0.784 | 1.17 | 1.07E-03 | 1.21E-02 | 3.33E-03 | 4.63E-03 | -4.89E-03 | 1.04E-02 |
| K13075 | 1.533 | 0.826 | 1.257 | 0.211 | 0.66 | 0.35 | -8.89E-03 | -7.50E-03 | 3.35E-03 | 1.17E-03 | -1.11E-03 | 2.86E-03 |
| K16698 | 1.607 | 0.68 | 1.651 | 1.398 | 0.469 | 0.402 | -1.02E-02 | 3.47E-03 | 7.30E-03 | -8.74E-03 | 8.21E-04 | 2.52E-03 |
| K04062 | 0.2 | 0.616 | 1.467 | 0.757 | 1.265 | 2.489 | -1.26E-03 | -2.91E-03 | 6.86E-03 | 4.76E-03 | -8.49E-03 | -2.17E-02 |
| K19304 | 0.321 | 1.149 | 1.281 | 0.704 | 0.37 | 1.202 | -1.72E-03 | -5.81E-03 | -6.21E-03 | -3.76E-03 | 1.53E-03 | 1.05E-02 |
| K14188 | 0.169 | 0.512 | 1.688 | 0.915 | 1.5 | 0.766 | 7.09E-04 | -4.98E-03 | 8.15E-03 | -3.27E-03 | -1.22E-02 | -3.76E-03 |
| K08678 | 0.581 | 1.091 | 0.603 | 1.084 | 0.938 | 0.363 | -1.76E-03 | -1.07E-02 | -2.45E-03 | -5.06E-03 | 4.89E-03 | 2.96E-03 |
| K02435 | 0.332 | 1.069 | 0.609 | 1.323 | 1.51 | 0.793 | -9.52E-04 | 7.28E-03 | 2.77E-03 | -8.17E-03 | -1.12E-02 | 3.95E-03 |
| K01919 | 0.616 | 0.835 | 0.684 | 2.084 | 0.456 | 2.141 | 2.66E-03 | 8.23E-03 | -3.34E-03 | -1.31E-02 | -3.42E-03 | -1.77E-02 |
| K01698 | 0.532 | 1.065 | 1.679 | 1.308 | 0.519 | 0.525 | 4.19E-04 | -3.72E-03 | -7.37E-03 | -6.35E-03 | -1.76E-03 | 3.64E-03 |
| K02119 | 0.763 | 0.406 | 1.484 | 0.412 | 1.566 | 2.052 | 1.80E-03 | 4.77E-04 | 7.30E-03 | -2.10E-03 | -1.27E-02 | -1.57E-02 |
| K03303 | 1.339 | 0.8 | 0.688 | 0.271 | 1.84 | 2.171 | -7.55E-03 | -6.33E-03 | -6.49E-05 | -1.42E-03 | -1.25E-02 | -1.91E-02 |
| K07726 | 0.404 | 0.942 | 0.599 | 1.203 | 1.63 | 0.248 | 8.49E-04 | 6.13E-03 | 2.07E-03 | -7.02E-03 | -1.14E-02 | -2.20E-03 |
| K01845 | 0.645 | 0.893 | 1.451 | 1.279 | 0.523 | 0.671 | 2.06E-03 | -4.69E-03 | -6.46E-03 | -6.38E-03 | -1.02E-03 | -5.11E-03 |
| K19225 | 0.794 | 1.145 | 1.189 | 1.17 | 0.589 | 0.303 | 2.51E-03 | 5.41E-03 | 5.80E-03 | 7.37E-03 | 4.01E-03 | -1.16E-04 |
| K07461 | 0.479 | 0.581 | 2.68 | 1.265 | 0.631 | 1.116 | -1.85E-03 | 2.74E-03 | 1.31E-02 | -7.73E-03 | -2.62E-03 | 9.92E-03 |
| K19117 | 0.754 | 0.749 | 1.074 | 1.029 | 0.993 | 0.651 | 2.28E-03 | -1.87E-03 | 3.90E-04 | -9.63E-04 | -1.44E-03 | 5.28E-03 |
| K03929 | 0.889 | 0.726 | 0.349 | 1.517 | 1.006 | 0.222 | 2.83E-03 | 4.05E-04 | 1.64E-03 | 8.28E-03 | -8.09E-03 | -1.09E-03 |
| K06934 | 1.02 | 1.147 | 0.985 | 0.206 | 0.47 | 0.309 | -5.46E-03 | -1.05E-02 | 2.86E-03 | -5.27E-04 | -2.61E-03 | -2.56E-03 |
| K02038 | 0.703 | 0.674 | 0.933 | 1.13 | 0.886 | 0.331 | -9.61E-04 | -5.55E-04 | 7.65E-04 | -9.05E-04 | 3.16E-03 | 1.92E-03 |
| K03606 | 0.652 | 0.205 | 0.628 | 0.842 | 0.974 | 1.463 | 2.94E-03 | -1.88E-03 | 1.35E-03 | 2.54E-03 | 7.63E-03 | 1.30E-02 |
| K18828 | 1.896 | 0.846 | 0.581 | 0.48 | 0.278 | 1.047 | 1.22E-02 | 8.09E-03 | -2.32E-03 | -3.02E-03 | 2.17E-03 | -5.51E-03 |
| K18908 | 0.988 | 0.675 | 0.721 | 1.21 | 0.826 | 0.719 | -2.65E-03 | -2.87E-03 | 4.89E-04 | 3.29E-03 | 4.12E-03 | 3.21E-03 |
| K19271 | 1.062 | 0.587 | 1.255 | 0.796 | 0.802 | 0.552 | -4.61E-03 | 8.53E-05 | 3.26E-03 | 4.22E-04 | -5.28E-03 | -3.50E-03 |
| K00027 | 0.636 | 0.986 | 1.061 | 0.285 | 0.29 | 1.624 | 2.93E-03 | 5.33E-03 | 4.71E-03 | -6.94E-05 | 8.37E-04 | -1.21E-02 |
| K00788 | 0.292 | 1.797 | 0.564 | 0.872 | 0.691 | 1.045 | -1.30E-03 | 1.75E-02 | 1.99E-03 | 5.48E-04 | -3.59E-04 | 9.03E-03 |
| K00941 | 0.632 | 0.968 | 0.694 | 1.145 | 1.136 | 0.541 | -3.87E-05 | 5.24E-03 | -4.47E-04 | 2.20E-03 | -5.46E-03 | 3.52E-04 |
| K03976 | 1.15 | 0.578 | 1.929 | 0.657 | 0.517 | 1.942 | -6.60E-03 | 4.51E-03 | 8.74E-03 | -4.07E-03 | -1.84E-03 | 1.71E-02 |
| K03442 | 0.353 | 0.851 | 1.682 | 1.036 | 0.788 | 0.298 | 7.92E-04 | 5.31E-03 | 7.90E-03 | -5.53E-03 | -2.63E-03 | -1.85E-03 |
| K09680 | 1.102 | 1.207 | 0.736 | 0.731 | 0.661 | 1.205 | 1.59E-05 | 9.35E-03 | -1.02E-03 | 2.56E-03 | 1.81E-03 | -8.52E-03 |

| | | | | | | | | | | | | |
|---|---|---|---|---|---|---|---|---|---|---|---|---|
| K07105 | 0.779 | 0.912 | 0.784 | 0.944 | 1.195 | 0.18 | -1.29E-03 | 1.75E-03 | 1.28E-04 | -2.14E-03 | -3.69E-03 | -6.65E-05 |
| K11358 | 0.724 | 1.391 | 0.776 | 0.97 | 0.867 | 0.448 | 1.06E-03 | -2.64E-03 | -1.86E-03 | -1.94E-03 | 4.78E-04 | 2.68E-03 |
| K04516 | 1.011 | 1.324 | 0.538 | 0.471 | 0.522 | 1.044 | 2.12E-03 | 4.00E-03 | 2.27E-03 | -8.94E-04 | -2.38E-03 | 3.20E-03 |
| K08681 | 1.007 | 1.132 | 0.667 | 0.476 | 1.097 | 0.456 | 6.44E-03 | -1.11E-02 | -2.69E-03 | -7.14E-04 | -8.13E-03 | 3.77E-03 |
| K07464 | 1.158 | 0.537 | 0.95 | 0.958 | 0.785 | 0.248 | 6.35E-03 | 2.82E-04 | -3.06E-04 | 8.63E-04 | 1.28E-03 | -1.91E-03 |
| K00812 | 1.078 | 1.111 | 0.798 | 0.689 | 0.886 | 0.616 | 1.83E-03 | -1.19E-03 | 2.64E-03 | 1.06E-03 | 1.77E-03 | -1.44E-03 |
| K07260 | 0.438 | 1.119 | 0.355 | 0.844 | 1.365 | 0.447 | -2.51E-03 | -2.21E-03 | 6.77E-04 | -4.83E-03 | -8.59E-03 | -1.06E-03 |
| K08679 | 1.121 | 0.869 | 0.476 | 0.803 | 0.634 | 0.723 | -5.47E-03 | 5.45E-03 | 2.03E-03 | 3.75E-03 | -1.71E-04 | 6.16E-03 |
| K01524 | 0.539 | 0.569 | 1.025 | 1.139 | 1.068 | 0.114 | 1.38E-03 | 9.84E-04 | -2.04E-03 | -1.35E-03 | 1.35E-03 | 3.24E-04 |
| K03783 | 0.72 | 1.3 | 0.538 | 0.282 | 0.8 | 0.909 | -1.41E-03 | 5.39E-03 | 1.68E-03 | -3.50E-04 | 6.70E-04 | 5.49E-03 |
| K03778 | 0.801 | 0.53 | 0.798 | 1.061 | 0.554 | 1.377 | 3.20E-03 | -1.41E-03 | -2.68E-05 | -2.91E-03 | -5.15E-04 | -1.19E-02 |
| K02557 | 1.344 | 1.653 | 1.54 | 0.702 | 0.464 | 0.799 | 7.35E-03 | 1.48E-02 | -6.57E-03 | -4.40E-03 | -2.31E-03 | -1.25E-03 |
| K07444 | 0.963 | 0.909 | 0.615 | 0.365 | 0.645 | 1.348 | 2.06E-03 | -5.08E-03 | 5.77E-04 | -2.30E-03 | 3.59E-03 | 1.18E-02 |
| K03281 | 1.356 | 0.804 | 0.258 | 0.527 | 0.505 | 1.433 | 6.16E-03 | -3.04E-03 | -3.04E-04 | -2.70E-03 | 3.35E-03 | 9.45E-03 |
| K00946 | 1.083 | 1.4 | 0.342 | 0.633 | 0.516 | 1.344 | 4.60E-03 | -1.30E-02 | -9.91E-04 | -3.64E-03 | -2.01E-03 | 3.45E-04 |
| K16694 | 0.637 | 2.012 | 0.422 | 0.4 | 1.177 | 1.969 | 2.16E-03 | 1.19E-02 | -1.82E-03 | 2.86E-04 | -5.93E-03 | 1.49E-02 |
| K09765 | 0.875 | 0.83 | 1.896 | 0.378 | 0.777 | 0.736 | -5.64E-03 | -8.19E-03 | 8.09E-03 | 3.61E-04 | -5.55E-03 | 1.73E-03 |
| K00265 | 0.548 | 0.549 | 1.104 | 0.956 | 0.45 | 1.051 | 2.29E-03 | -4.09E-03 | -3.64E-03 | -3.10E-03 | -4.53E-04 | -8.07E-03 |
| K10536 | 1.194 | 1.426 | 0.768 | 0.63 | 0.779 | 1.049 | 4.51E-03 | 4.86E-03 | 5.49E-05 | -9.40E-04 | 4.57E-03 | 2.44E-03 |
| K15738 | 1.091 | 0.823 | 0.807 | 0.782 | 0.619 | 0.635 | -6.69E-03 | 4.17E-03 | -1.63E-03 | -4.33E-04 | 4.14E-03 | 1.83E-03 |
| K09747 | 0.741 | 1.249 | 0.315 | 1.292 | 1.383 | 0.751 | 7.69E-05 | 1.99E-03 | 7.87E-04 | -5.93E-03 | -5.53E-03 | 5.27E-03 |
| K01918 | 0.942 | 1.418 | 0.554 | 1.107 | 0.584 | 0.565 | 1.14E-03 | 8.00E-03 | -2.38E-03 | -6.97E-03 | -7.24E-04 | -4.77E-03 |
| K02114 | 0.65 | 0.79 | 0.963 | 1.08 | 1.137 | 0.382 | 2.94E-03 | -2.35E-03 | -1.64E-03 | -6.78E-04 | 2.52E-03 | -2.46E-03 |
| K01206 | 1.153 | 1.573 | 0.648 | 0.642 | 0.684 | 0.893 | -7.41E-03 | 1.30E-02 | 2.47E-03 | -3.91E-03 | -3.94E-03 | 6.79E-03 |
| K01156 | 1.588 | 1.721 | 0.891 | 0.582 | 0.156 | 0.065 | 9.92E-03 | 1.13E-02 | 3.81E-03 | -2.99E-03 | -1.20E-03 | -1.91E-04 |
| K07386 | 0.986 | 1.268 | 0.322 | 0.909 | 0.379 | 0.608 | -2.40E-03 | -1.04E-04 | -5.26E-04 | 5.72E-03 | -2.38E-03 | 2.78E-03 |
| K06919 | 2.073 | 0.453 | 1.026 | 1.056 | 0.417 | 0.422 | -1.34E-02 | 3.17E-03 | 4.28E-03 | 4.07E-03 | 1.93E-03 | 2.80E-03 |
| K23242 | 1.224 | 0.86 | 1.73 | 0.419 | 0.458 | 0.689 | -7.43E-03 | -7.32E-03 | 7.82E-03 | -2.23E-03 | 6.10E-04 | 5.21E-03 |
| K21908 | 0.816 | 0.405 | 2.343 | 0.644 | 0.994 | 0.353 | -3.25E-03 | -3.08E-03 | 1.05E-02 | 4.02E-03 | 7.98E-03 | -2.32E-03 |
| K01284 | 0.991 | 1.05 | 0.559 | 0.749 | 0.598 | 1.033 | 2.99E-04 | -5.69E-03 | 6.35E-04 | 4.55E-03 | 1.34E-03 | 6.24E-03 |
| K04771 | 0.676 | 0.595 | 0.754 | 0.997 | 1.127 | 0.812 | 1.65E-03 | -1.67E-03 | -2.59E-03 | -5.94E-03 | -9.19E-03 | -4.03E-03 |
| K00053 | 0.414 | 0.41 | 0.971 | 0.885 | 0.967 | 0.181 | -1.58E-03 | 3.19E-03 | -1.86E-03 | -6.90E-05 | 7.10E-03 | -1.49E-03 |
| K07063 | 0.582 | 0.751 | 1.695 | 0.636 | 1.715 | 1.435 | 2.57E-03 | 6.02E-03 | 7.42E-03 | 7.80E-04 | 1.23E-02 | 1.15E-02 |
| K00865 | 0.757 | 0.584 | 1.316 | 1.021 | 0.912 | 0.791 | 3.99E-03 | 4.15E-03 | -5.73E-03 | -4.08E-03 | -7.23E-03 | 5.79E-03 |

| | | | | | | | | | | | | |
|---|---|---|---|---|---|---|---|---|---|---|---|---|
| K00794 | 0.977 | 0.781 | 0.598 | 1.424 | 0.797 | 1.777 | 3.25E-04 | 3.52E-03 | -1.87E-03 | -7.64E-03 | -4.24E-03 | 1.35E-02 |
| K01262 | 0.826 | 1.447 | 1.03 | 0.553 | 0.228 | 0.305 | -5.25E-03 | -1.43E-02 | -4.01E-03 | -1.08E-03 | -1.82E-03 | -1.61E-03 |
| K13038 | 1.349 | 0.633 | 0.624 | 0.94 | 1.338 | 0.532 | 8.38E-03 | 4.14E-03 | -2.45E-04 | -2.41E-03 | 1.09E-02 | -8.14E-04 |
| K07259 | 1.801 | 0.828 | 1.011 | 0.028 | 0.187 | 0.739 | 1.07E-02 | -5.13E-03 | -4.28E-03 | 9.20E-05 | 1.36E-03 | 1.37E-03 |
| K00645 | 0.682 | 0.896 | 0.734 | 1.593 | 1.957 | 0.44 | -8.43E-04 | 3.70E-03 | -3.61E-03 | -9.81E-03 | -1.58E-02 | -2.69E-04 |
| K05970 | 0.845 | 1.129 | 0.392 | 0.321 | 0.841 | 0.5 | 1.63E-03 | -2.90E-03 | -1.87E-03 | 5.27E-04 | 3.85E-03 | -3.14E-03 |
| K00012 | 0.673 | 0.585 | 0.553 | 1.235 | 1.162 | 0.846 | 2.65E-03 | 2.07E-03 | 2.70E-03 | 7.48E-03 | 9.44E-03 | 4.26E-03 |
| K06901 | 1.789 | 1.053 | 0.743 | 0.44 | 0.584 | 1.128 | -1.15E-02 | -8.85E-04 | 3.65E-03 | 1.41E-03 | 2.63E-03 | -1.00E-02 |
| K04043 | 0.585 | 0.653 | 0.63 | 0.948 | 0.836 | 0.91 | 4.24E-04 | -6.15E-03 | 2.98E-04 | -9.61E-04 | 6.02E-03 | -5.20E-03 |
| K00817 | 0.376 | 1.229 | 0.763 | 0.992 | 0.708 | 1.509 | -5.19E-04 | 1.16E-02 | 6.83E-04 | -2.29E-03 | -2.23E-03 | -1.27E-02 |
| K02493 | 0.293 | 0.321 | 0.897 | 1.297 | 0.806 | 0.585 | -1.51E-03 | -2.68E-03 | 4.33E-03 | -6.87E-03 | -3.83E-04 | 4.42E-03 |
| K04077 | 0.869 | 0.802 | 0.772 | 1.146 | 0.319 | 0.773 | 5.59E-03 | 3.11E-03 | -2.11E-03 | -4.76E-03 | -1.21E-03 | -3.16E-03 |
| K01613 | 1.113 | 0.867 | 0.757 | 0.623 | 0.243 | 1.469 | 1.56E-03 | -7.11E-03 | -3.08E-03 | -3.77E-03 | -1.77E-04 | 7.91E-03 |
| K19334 | 1.193 | 0.69 | 1.394 | 0.368 | 0.68 | 1.149 | 6.97E-03 | -3.88E-03 | -6.86E-03 | -2.31E-03 | 5.21E-03 | -7.50E-03 |
| K02112 | 0.174 | 0.27 | 0.84 | 0.903 | 0.982 | 0.465 | -2.42E-04 | 2.03E-03 | 6.43E-05 | -1.97E-03 | 5.18E-03 | 3.58E-03 |
| K00174 | 1.015 | 1.002 | 0.127 | 0.406 | 0.235 | 0.933 | 4.81E-03 | -6.24E-03 | 6.22E-04 | 9.18E-04 | -9.60E-04 | -8.28E-03 |
| K01173 | 1.198 | 1.325 | 0.576 | 0.387 | 0.706 | 0.88 | 5.33E-03 | 2.67E-03 | -2.66E-03 | -2.24E-03 | -4.82E-03 | 2.31E-03 |
| K07391 | 0.469 | 0.577 | 1.068 | 1.049 | 1.196 | 0.724 | 3.04E-03 | -5.02E-03 | 4.79E-03 | 3.49E-03 | -6.71E-03 | 3.39E-03 |
| K09710 | 0.765 | 0.28 | 1.599 | 1.17 | 0.864 | 0.142 | -1.73E-03 | -1.49E-04 | 5.71E-03 | -6.54E-03 | -6.71E-03 | 1.51E-04 |
| K04066 | 0.991 | 0.155 | 0.514 | 1.083 | 0.573 | 1.219 | 6.09E-03 | -1.50E-03 | 1.04E-04 | -2.66E-03 | 2.26E-03 | 9.97E-03 |
| K06133 | 0.93 | 0.634 | 0.595 | 1.544 | 1.387 | 0.506 | -4.04E-03 | -6.05E-03 | -8.99E-04 | -9.73E-03 | -1.12E-02 | -4.31E-03 |
| K02967 | 0.768 | 0.525 | 0.847 | 1.002 | 0.643 | 0.82 | 4.95E-03 | -4.89E-03 | -3.31E-03 | -3.61E-03 | 4.16E-03 | -4.69E-03 |
| K01883 | 0.499 | 1.13 | 0.345 | 0.921 | 0.925 | 0.582 | 3.21E-03 | -1.11E-02 | 1.15E-03 | -2.44E-03 | 5.31E-03 | 1.83E-03 |
| K02520 | 0.533 | 0.229 | 1.143 | 1.452 | 0.708 | 1.136 | 2.98E-03 | -1.22E-03 | 5.36E-03 | -8.89E-03 | -4.61E-03 | 1.01E-02 |
| K00677 | 1.095 | 1.191 | 0.661 | 0.662 | 0.729 | 1.227 | 1.93E-03 | 1.75E-03 | -2.86E-03 | -2.68E-03 | -4.34E-03 | -1.46E-03 |
| K01258 | 0.562 | 0.813 | 0.756 | 1.049 | 0.807 | 0.754 | -8.94E-06 | -7.95E-03 | 9.08E-04 | -2.82E-03 | 3.57E-03 | 6.70E-03 |
| K00705 | 0.275 | 1.18 | 1.819 | 1.22 | 0.624 | 0.663 | 1.73E-03 | 7.92E-03 | 8.93E-03 | 7.67E-03 | 2.33E-03 | -3.45E-03 |
| K03470 | 0.905 | 0.346 | 1.376 | 0.905 | 0.519 | 0.263 | 5.43E-03 | -2.73E-03 | 6.45E-03 | -4.20E-03 | -2.30E-03 | 2.00E-03 |
| K07979 | 1.144 | 0.352 | 1.127 | 0.38 | 0.495 | 0.883 | -6.92E-03 | 1.16E-03 | 5.32E-03 | -2.83E-05 | 1.82E-03 | 4.28E-03 |
| K02904 | 1.476 | 1.024 | 0.345 | 1.268 | 0.719 | 0.121 | 9.51E-03 | 3.79E-03 | 1.47E-03 | -7.99E-03 | -2.58E-03 | 1.07E-03 |
| K01991 | 1.082 | 1.403 | 0.749 | 0.677 | 0.747 | 0.936 | 1.36E-03 | 4.02E-03 | -1.68E-04 | 7.23E-05 | 2.81E-04 | 4.56E-03 |
| K03502 | 0.869 | 0.893 | 0.729 | 1.144 | 0.76 | 0.786 | 5.41E-03 | -8.11E-03 | 4.53E-04 | -2.66E-03 | -1.79E-03 | 6.70E-03 |
| K09748 | 0.792 | 0.652 | 0.792 | 1.721 | 2.054 | 0.803 | 2.85E-03 | -5.58E-03 | 2.78E-03 | -1.08E-02 | -1.66E-02 | 6.32E-03 |
| K00067 | 1.017 | 1.243 | 0.415 | 0.54 | 0.075 | 2.108 | 6.19E-03 | 9.97E-03 | -1.42E-03 | 1.50E-03 | 1.72E-04 | 1.81E-02 |

| | | | | | | | | | | | | |
|---|---|---|---|---|---|---|---|---|---|---|---|---|
| K08316 | 0.648 | 0.792 | 0.327 | 1.577 | 1.203 | 1.351 | 3.93E-03 | -4.70E-03 | 1.43E-03 | -9.46E-03 | -8.11E-03 | 1.20E-02 |
| K03545 | 0.649 | 0.342 | 0.616 | 1.164 | 1.274 | 0.942 | -3.62E-03 | -3.32E-03 | 2.45E-03 | -5.20E-03 | -6.46E-03 | -3.23E-03 |
| K02963 | 0.737 | 0.908 | 1.613 | 0.895 | 0.563 | 0.611 | 3.25E-03 | -2.51E-03 | 7.65E-03 | -5.48E-03 | 3.14E-03 | 5.25E-03 |
| K00973 | 0.543 | 1.609 | 1.305 | 0.199 | 0.58 | 1.422 | -2.33E-03 | 1.19E-02 | 6.42E-03 | -9.42E-04 | -4.20E-03 | 1.25E-02 |
| K02838 | 0.561 | 0.93 | 1 | 1.258 | 0.412 | 0.374 | 1.12E-04 | -9.04E-03 | 3.23E-03 | -6.35E-03 | -3.33E-03 | 1.95E-03 |
| K07001 | 1.301 | 0.913 | 0.479 | 0.699 | 1.049 | 0.391 | 8.15E-03 | 2.81E-03 | -7.15E-04 | -4.24E-03 | -7.10E-03 | 3.41E-03 |
| K21023 | 0.669 | 0.795 | 0.81 | 1.182 | 0.978 | 0.627 | -1.63E-03 | 1.34E-03 | -1.30E-03 | -1.69E-03 | -5.70E-04 | -3.87E-03 |
| K01209 | 0.699 | 0.935 | 0.908 | 0.282 | 0.146 | 1.154 | -1.33E-04 | 1.65E-03 | -4.44E-03 | -1.70E-03 | -1.49E-04 | -1.01E-02 |
| K01992 | 0.349 | 0.348 | 1.608 | 0.914 | 0.691 | 2.575 | -1.39E-03 | -2.35E-03 | 7.56E-03 | -5.33E-03 | 3.01E-03 | 2.29E-02 |
| K00059 | 0.828 | 0.711 | 0.669 | 1.773 | 0.927 | 0.717 | 3.00E-03 | -8.40E-04 | -1.28E-03 | -1.02E-02 | -6.33E-03 | -3.52E-03 |
| K03699 | 0.248 | 0.906 | 0.783 | 1.214 | 1.028 | 0.658 | -1.27E-03 | 6.94E-03 | -2.86E-04 | -6.70E-03 | -6.65E-03 | -5.85E-03 |
| K07263 | 0.936 | 1.084 | 0.433 | 0.311 | 0.711 | 1.161 | 2.51E-03 | -6.72E-04 | -4.14E-04 | 1.70E-03 | 2.00E-03 | 1.24E-03 |
| K03310 | 0.802 | 0.652 | 0.634 | 1.237 | 1.257 | 0.344 | -4.99E-03 | 5.71E-03 | -1.08E-03 | -6.75E-03 | -6.49E-03 | 2.91E-03 |
| K07271 | 0.612 | 0.978 | 0.376 | 0.112 | 1.379 | 2.098 | 8.35E-04 | 2.86E-03 | 1.28E-03 | -7.07E-04 | -1.11E-02 | -1.74E-02 |
| K02429 | 0.973 | 1.156 | 0.145 | 0.349 | 0.653 | 1.062 | -5.48E-04 | -1.43E-04 | 2.36E-04 | 1.62E-03 | -1.90E-03 | 3.73E-03 |
| K07154 | 0.991 | 1.038 | 0.613 | 0.394 | 0.769 | 1.239 | 3.53E-03 | 4.06E-03 | -2.98E-03 | -2.32E-03 | -5.71E-03 | 4.65E-03 |
| K01153 | 0.436 | 0.675 | 1.072 | 1.058 | 0.813 | 0.776 | -2.73E-03 | 3.53E-03 | -4.12E-03 | -3.42E-03 | 3.40E-03 | 6.84E-03 |
| K03797 | 0.877 | 1.065 | 0.206 | 0.519 | 0.522 | 1.28 | 1.87E-03 | -2.39E-04 | 9.45E-04 | 3.21E-03 | 2.62E-03 | 6.75E-03 |
| K00558 | 0.381 | 1.334 | 0.316 | 1.23 | 0.326 | 0.813 | -3.17E-05 | 7.73E-03 | 6.80E-04 | -7.49E-03 | -1.79E-03 | 7.19E-03 |
| K03497 | 0.749 | 0.54 | 0.451 | 1.25 | 1.597 | 0.887 | 2.07E-03 | -2.57E-03 | 7.32E-04 | -3.90E-03 | -1.12E-02 | -6.53E-03 |
| K00986 | 0.554 | 1.523 | 0.451 | 1.093 | 1.045 | 0.546 | 1.59E-03 | -1.18E-02 | 4.48E-04 | 1.10E-03 | 1.94E-03 | 4.50E-03 |
| K02004 | 2.129 | 0.763 | 1.215 | 0.742 | 1.014 | 0.604 | -1.38E-02 | -3.28E-03 | 5.76E-03 | -4.44E-03 | -6.32E-03 | -1.39E-03 |
| K07484 | 0.682 | 0.755 | 1.195 | 1.198 | 1.058 | 0.406 | 1.23E-03 | 3.56E-03 | 5.23E-03 | 5.17E-03 | -6.35E-04 | 3.59E-03 |
| K04763 | 1.593 | 0.681 | 1.257 | 1.45 | 0.741 | 0.654 | 9.50E-03 | -5.71E-03 | -4.91E-03 | 4.74E-03 | -2.49E-04 | 5.78E-03 |
| K14096 | 0.505 | 0.779 | 1.506 | 0.606 | 0.893 | 0.691 | 2.86E-03 | 5.53E-03 | 5.17E-03 | 2.15E-03 | 4.76E-03 | 5.35E-03 |
| K05566 | 0.371 | 0.576 | 1.378 | 0.701 | 0.16 | 0.824 | -3.45E-04 | 5.62E-03 | 4.52E-03 | 2.54E-03 | 1.29E-03 | 7.00E-03 |
| K02626 | 0.415 | 0.469 | 1.483 | 0.427 | 1.333 | 0.614 | -2.26E-03 | -4.26E-03 | 5.22E-03 | 8.21E-04 | 9.51E-03 | 5.15E-03 |
| K00578 | 0.333 | 0.303 | 1.506 | 0.699 | 0.999 | 0.287 | 3.13E-04 | 1.21E-03 | 5.34E-03 | 2.75E-03 | 5.35E-03 | 1.03E-03 |
| K10212 | 0.396 | 1.968 | 0.539 | 1.239 | 0.299 | 0.653 | -2.38E-03 | 1.94E-02 | 3.29E-04 | 6.67E-03 | -1.89E-04 | 2.92E-03 |
| K03057 | 0.671 | 0.774 | 0.991 | 0.682 | 1.048 | 0.257 | 2.99E-03 | 5.49E-03 | 2.26E-03 | 2.53E-03 | 6.32E-03 | -2.66E-04 |
| K09764 | 0.73 | 0.178 | 0.983 | 0.417 | 0.994 | 0.58 | 1.53E-03 | 1.56E-03 | 2.56E-03 | 2.33E-03 | -7.42E-03 | -4.45E-03 |
| K06962 | 0.72 | 0.271 | 0.667 | 0.985 | 0.946 | 0.664 | 2.67E-03 | -2.09E-04 | 1.01E-03 | -2.46E-03 | 2.67E-03 | 3.17E-03 |
| K08234 | 1.095 | 0.775 | 0.27 | 0.579 | 1.019 | 0.352 | 7.08E-03 | 1.43E-03 | -2.92E-04 | 2.79E-03 | -7.95E-03 | -2.15E-03 |
| K01518 | 1.142 | 0.512 | 0.736 | 1.275 | 0.792 | 0.676 | -7.27E-03 | 4.59E-03 | 2.76E-03 | -7.79E-03 | -6.40E-03 | -2.55E-03 |

| | | | | | | | | | | | | |
|---|---|---|---|---|---|---|---|---|---|---|---|---|
| K04769 | 0.528 | 0.766 | 0.38 | 1.188 | 0.277 | 1.219 | -2.79E-03 | 3.16E-03 | -7.00E-04 | -7.35E-03 | -2.24E-03 | 7.02E-03 |
| K02651 | 1.4 | 0.692 | 0.351 | 0.842 | 0.421 | 0.558 | 8.94E-03 | 6.50E-03 | 1.34E-03 | -1.59E-03 | 5.80E-04 | -3.29E-03 |
| K01500 | 1.132 | 0.583 | 0.381 | 0.853 | 0.437 | 0.719 | -2.89E-03 | 4.24E-03 | -1.13E-03 | 2.14E-03 | -5.96E-04 | -3.16E-03 |
| K01975 | 0.434 | 0.311 | 1.13 | 0.082 | 0.321 | 0.807 | -2.99E-04 | -9.86E-04 | 3.65E-03 | 4.76E-04 | -2.03E-03 | -7.15E-03 |
| K16329 | 0.5 | 0.93 | 1.284 | 0.683 | 0.467 | 0.365 | 4.41E-04 | -8.99E-03 | 3.21E-03 | -2.37E-03 | 1.46E-03 | 2.66E-03 |
| K03785 | 0.791 | 0.951 | 1.459 | 0.692 | 0.737 | 0.254 | 1.69E-03 | 3.83E-03 | 6.67E-03 | 1.52E-04 | 3.18E-03 | 1.95E-03 |
| K06198 | 0.564 | 0.6 | 0.399 | 1.182 | 0.228 | 2.691 | 3.42E-03 | -3.61E-03 | -9.55E-04 | 5.98E-03 | -9.73E-05 | 2.39E-02 |
| K13684 | 0.341 | 1.522 | 0.764 | 0.23 | 0.46 | 1.8 | 9.54E-04 | 1.04E-02 | 3.73E-03 | -1.03E-03 | -3.74E-03 | 1.60E-02 |
| K21556 | 0.687 | 0.777 | 1.364 | 0.692 | 1.132 | 0.667 | -9.63E-04 | 4.57E-03 | 6.05E-03 | 1.23E-04 | -1.48E-03 | -5.77E-03 |
| K16214 | 1.376 | 0.738 | 0.88 | 0.48 | 0.665 | 0.355 | 8.43E-03 | 1.62E-03 | 1.44E-03 | -9.66E-05 | 3.88E-03 | 2.80E-03 |
| K14415 | 0.543 | 0.651 | 0.986 | 1.007 | 0.677 | 0.352 | -2.77E-03 | -6.36E-03 | -2.92E-04 | -6.30E-03 | 4.24E-03 | -3.13E-03 |
| K01486 | 1.618 | 0.778 | 1.01 | 0.667 | 0.628 | 0.274 | -1.05E-02 | 7.55E-03 | 3.29E-03 | -3.95E-03 | -2.59E-03 | -5.49E-04 |
| K20276 | 0.415 | 0.366 | 0.565 | 0.158 | 0.842 | 0.991 | 1.03E-03 | 1.99E-04 | -1.21E-05 | -3.83E-04 | 6.85E-03 | 8.81E-03 |
| K02492 | 0.367 | 0.797 | 1.74 | 1.164 | 0.268 | 0.53 | 8.47E-04 | -2.22E-03 | -7.86E-03 | -5.03E-03 | -2.85E-04 | -2.64E-03 |
| K03820 | 0.646 | 0.77 | 1.661 | 0.933 | 0.491 | 0.783 | 4.18E-03 | -4.84E-03 | -6.69E-03 | -2.81E-03 | 8.86E-04 | -5.92E-03 |
| K07089 | 0.179 | 0.876 | 1.033 | 0.644 | 0.635 | 0.071 | 6.61E-04 | 2.70E-03 | 4.39E-03 | 7.86E-04 | -4.09E-03 | 2.22E-05 |
| K12976 | 0.74 | 1.039 | 0.531 | 0.323 | 2.313 | 0.643 | 3.41E-03 | 1.02E-02 | -1.64E-03 | -1.80E-03 | -1.83E-02 | -5.54E-03 |
| K00283 | 0.234 | 0.674 | 0.58 | 0.858 | 0.807 | 0.495 | 1.25E-03 | 3.24E-03 | -1.44E-03 | -2.75E-03 | 2.49E-03 | 4.23E-03 |
| K11941 | 0.556 | 0.998 | 0.471 | 0.649 | 0.832 | 0.314 | -2.63E-03 | 7.28E-03 | 9.60E-04 | 3.71E-03 | 6.78E-03 | -1.51E-03 |
| K07404 | 1.14 | 0.428 | 0.731 | 1.144 | 0.691 | 0.595 | -5.19E-03 | -4.16E-04 | 2.39E-03 | 3.89E-03 | 3.36E-03 | 4.85E-03 |
| K01235 | 0.518 | 0.847 | 0.493 | 0.249 | 0.731 | 1.241 | -9.34E-04 | -3.76E-03 | 1.82E-03 | 1.47E-03 | 3.51E-03 | -1.09E-02 |
| K01966 | 0.942 | 0.853 | 0.23 | 0.026 | 0.369 | 0.614 | 1.22E-03 | -5.50E-03 | -8.96E-04 | -1.56E-04 | 3.02E-03 | 5.45E-03 |
| K01858 | 2.416 | 0.467 | 0.159 | 1.756 | 0.281 | 0.197 | 1.53E-02 | -4.51E-03 | 2.19E-04 | 1.10E-02 | 2.22E-03 | 1.63E-03 |
| K07741 | 0.357 | 1.675 | 0.46 | 1.25 | 0.664 | 0.437 | -2.30E-03 | 1.63E-02 | 1.80E-03 | 3.22E-03 | 2.99E-03 | 2.70E-03 |
| K14136 | 0.47 | 0.334 | 1.203 | 0.614 | 0.736 | 1.144 | 2.69E-03 | -2.91E-03 | -4.25E-03 | -2.95E-04 | -4.07E-03 | -8.65E-03 |
| K02037 | 0.576 | 0.746 | 1.162 | 1.121 | 0.795 | 0.258 | -3.08E-04 | -1.76E-03 | -3.45E-03 | -3.00E-03 | 1.90E-03 | -2.40E-05 |
| K05601 | 0.744 | 0.573 | 0.81 | 1.07 | 0.685 | 0.498 | -1.55E-03 | -1.30E-03 | -5.55E-04 | -1.45E-04 | 5.23E-03 | 4.10E-03 |
| K24180 | 0.986 | 1.366 | 0.536 | 0.411 | 0.372 | 0.651 | -5.23E-03 | 1.10E-02 | -1.12E-03 | -2.58E-03 | -2.70E-03 | -4.52E-03 |
| K09015 | 1.003 | 1.562 | 0.292 | 0.641 | 0.63 | 0.701 | 2.81E-03 | 8.71E-03 | 1.43E-03 | 3.01E-03 | -2.93E-03 | -3.42E-03 |
| K03816 | 0.563 | 0.854 | 0.944 | 0.512 | 0.398 | 0.452 | 2.58E-03 | -8.33E-03 | 4.01E-03 | 1.42E-03 | 3.06E-03 | 1.85E-03 |
| K07102 | 1.298 | 1.101 | 0.203 | 0.312 | 0.736 | 0.665 | 4.94E-03 | 7.56E-04 | -9.89E-04 | -1.88E-03 | 3.39E-03 | 1.32E-03 |
| K03784 | 0.111 | 0.697 | 1.58 | 1.39 | 0.3 | 0.609 | -6.44E-04 | -6.00E-03 | 5.78E-03 | -6.63E-03 | -2.19E-04 | 4.91E-03 |
| K09760 | 0.256 | 1.05 | 0.522 | 0.683 | 0.601 | 1.273 | -7.93E-04 | 5.44E-05 | -1.38E-03 | -3.56E-03 | -3.11E-03 | -1.08E-02 |
| K23351 | 0.492 | 0.509 | 1.291 | 0.556 | 0.812 | 0.71 | 1.20E-03 | -3.59E-03 | 4.97E-03 | -3.49E-03 | -5.38E-03 | 6.28E-03 |

| | | | | | | | | | | | | |
|---|---|---|---|---|---|---|---|---|---|---|---|---|
| K01338 | 0.484 | 1.098 | 0.364 | 0.943 | 0.323 | 0.741 | 1.24E-03 | -6.34E-04 | 1.49E-03 | -5.88E-03 | -1.69E-03 | 2.98E-03 |
| K03614 | 1.013 | 0.838 | 0.737 | 0.561 | 0.55 | 0.473 | -4.62E-03 | 4.61E-04 | 1.47E-03 | -3.28E-03 | -5.77E-04 | -8.89E-04 |
| K03284 | 0.575 | 0.722 | 0.338 | 1.072 | 0.669 | 1.354 | 1.01E-03 | -3.18E-03 | -5.62E-04 | -6.75E-03 | -2.50E-03 | 1.09E-02 |
| K20509 | 1.592 | 0.75 | 2.176 | 0.599 | 0.686 | 0.775 | -1.01E-02 | -1.82E-03 | 1.00E-02 | -3.70E-03 | -1.25E-04 | -5.52E-03 |
| K07277 | 1.216 | 0.936 | 0.616 | 0.705 | 0.18 | 0.792 | 5.18E-03 | -5.06E-03 | -1.95E-03 | -3.75E-03 | -8.03E-04 | 4.47E-03 |
| K01952 | 0.557 | 0.743 | 0.808 | 0.944 | 0.394 | 0.661 | 2.76E-03 | 6.14E-03 | -1.10E-03 | -2.05E-04 | 2.44E-03 | 4.27E-03 |
| K23775 | 1.825 | 0.51 | 1.741 | 0.427 | 0.287 | 0.462 | -1.18E-02 | -4.84E-03 | 6.67E-03 | -2.29E-03 | -2.32E-03 | -3.18E-03 |
| K07322 | 1.138 | 0.656 | 0.608 | 0.175 | 0.021 | 0.816 | 5.56E-03 | -4.57E-03 | -2.83E-03 | 1.17E-04 | -1.64E-04 | -6.84E-05 |
| K03612 | 0.871 | 0.559 | 0.466 | 1.04 | 0.371 | 0.059 | -3.15E-04 | -1.53E-03 | -1.05E-04 | -6.32E-03 | -7.20E-04 | 2.94E-04 |
| K19693 | 0.431 | 0.885 | 0.445 | 0.963 | 0.537 | 0.336 | 6.60E-04 | -4.14E-03 | -1.25E-03 | 5.55E-03 | -3.29E-03 | -2.54E-03 |
| K03517 | 0.856 | 0.333 | 0.144 | 1.039 | 0.23 | 0.325 | 3.71E-03 | -5.48E-04 | -1.48E-04 | 6.50E-03 | -9.30E-04 | 5.31E-04 |
| K09014 | 0.526 | 0.677 | 0.552 | 1.084 | 0.9 | 0.665 | 3.41E-03 | -3.86E-03 | 1.80E-03 | -5.83E-03 | 7.11E-03 | 4.27E-03 |
| K07816 | 1.492 | 0.593 | 1.958 | 0.715 | 0.241 | 0.65 | -9.52E-03 | -5.78E-03 | 8.84E-03 | -4.40E-03 | -1.85E-03 | 2.70E-03 |
| K03046 | 0.416 | 1.119 | 0.721 | 0.963 | 0.43 | 0.642 | 3.26E-05 | -1.05E-02 | -7.35E-04 | -1.43E-03 | -5.09E-04 | 4.49E-03 |
| K03790 | 0.681 | 0.156 | 1.086 | 0.976 | 0.308 | 0.16 | -4.30E-03 | -4.99E-04 | 2.91E-03 | -5.95E-03 | 1.89E-03 | -2.29E-04 |
| K06223 | 0.105 | 0.902 | 0.783 | 0.855 | 0.734 | 0.337 | -4.06E-04 | -7.59E-03 | -3.25E-03 | -5.04E-03 | 5.88E-03 | 8.91E-04 |
| K00765 | 0.495 | 0.824 | 0.364 | 0.998 | 0.546 | 0.499 | 1.56E-03 | 7.40E-03 | 1.71E-03 | -4.66E-03 | -7.25E-05 | -3.71E-03 |
| K03615 | 1.442 | 0.532 | 0.118 | 1.231 | 0.619 | 0.746 | -9.20E-03 | 1.23E-03 | -5.45E-04 | -5.19E-03 | -3.89E-03 | -4.62E-03 |
| K00931 | 0.255 | 0.476 | 0.533 | 0.992 | 1.079 | 0.489 | -1.65E-03 | 4.69E-03 | -7.96E-04 | -3.23E-03 | -7.31E-03 | 1.14E-03 |
| K06909 | 0.163 | 0.422 | 0.866 | 1.325 | 0.657 | 0.72 | 1.73E-04 | -4.08E-03 | 1.27E-03 | -8.29E-03 | -1.96E-03 | 3.54E-03 |
| K01854 | 0.323 | 0.636 | 1.604 | 0.526 | 0.632 | 1.061 | -7.41E-04 | -6.23E-03 | 7.69E-03 | 2.79E-04 | 2.34E-03 | 9.22E-03 |
| K01933 | 0.934 | 0.588 | 0.663 | 1.216 | 0.489 | 0.192 | -6.01E-03 | 4.51E-03 | 4.05E-05 | -5.50E-03 | -6.38E-04 | -1.69E-03 |
| K01869 | 0.753 | 0.41 | 0.712 | 0.936 | 0.693 | 0.897 | 4.68E-03 | -3.88E-03 | -6.13E-04 | 1.63E-04 | 4.56E-03 | 6.58E-03 |
| K01224 | 1.626 | 1.038 | 0.585 | 0.625 | 0.205 | 0.576 | 8.79E-03 | -9.19E-04 | -2.88E-03 | 3.94E-03 | 1.42E-03 | 4.63E-03 |
| K06113 | 0.94 | 1.356 | 0.724 | 0.41 | 0.797 | 0.382 | 1.38E-03 | 4.66E-03 | -3.54E-03 | -1.42E-03 | -1.65E-03 | 2.01E-03 |
| K01929 | 0.466 | 0.503 | 0.173 | 0.938 | 0.352 | 0.803 | -2.53E-03 | -1.74E-03 | 8.46E-04 | -3.04E-03 | -1.76E-05 | -5.20E-04 |
| K07729 | 0.78 | 1.28 | 1.839 | 0.354 | 0.327 | 0.664 | -2.45E-03 | 1.18E-02 | 8.48E-03 | -1.76E-03 | -1.23E-03 | -4.63E-03 |
| K07460 | 0.773 | 0.651 | 1.268 | 0.909 | 0.45 | 0.622 | 2.61E-03 | 3.90E-03 | 4.01E-03 | -4.82E-03 | -1.84E-03 | 5.37E-03 |
| K11754 | 0.535 | 0.399 | 0.492 | 1.293 | 0.789 | 0.972 | -1.68E-03 | 8.00E-04 | -1.55E-03 | -6.59E-03 | -3.91E-03 | -8.44E-03 |
| K06207 | 0.227 | 0.847 | 0.767 | 1.052 | 0.462 | 0.607 | -1.09E-03 | -8.14E-03 | -1.44E-03 | -2.12E-03 | -1.82E-03 | -4.29E-04 |
| K00648 | 0.706 | 0.759 | 0.141 | 0.981 | 0.054 | 1.39 | 1.90E-03 | 2.72E-03 | 6.43E-04 | -5.80E-03 | -2.05E-04 | 4.65E-03 |
| K01889 | 0.825 | 0.366 | 0.481 | 1.115 | 0.233 | 0.493 | 5.33E-03 | 1.83E-03 | 2.32E-03 | -6.02E-03 | -3.44E-05 | -9.67E-04 |
| K19302 | 0.796 | 0.658 | 0.755 | 0.28 | 1.416 | 1.286 | 8.69E-04 | 4.18E-03 | -1.00E-03 | -1.70E-03 | -1.15E-02 | -8.69E-03 |
| K06942 | 0.901 | 0.538 | 0.496 | 1.075 | 0.44 | 0.69 | 5.80E-03 | -5.29E-03 | -4.07E-05 | -4.91E-03 | 2.90E-03 | 1.36E-03 |

| | | | | | | | | | | | | |
|---|---|---|---|---|---|---|---|---|---|---|---|---|
| K01662 | 0.697 | 0.269 | 0.994 | 0.953 | 0.703 | 0.422 | 4.30E-03 | -3.76E-04 | -2.68E-03 | -4.23E-03 | 5.31E-03 | -1.59E-03 |
| K03168 | 0.689 | 0.999 | 0.367 | 1.3 | 0.346 | 0.582 | -4.26E-03 | -9.86E-03 | 7.73E-04 | -6.64E-03 | 1.18E-03 | 2.49E-03 |
| K01875 | 0.064 | 0.772 | 0.458 | 1.019 | 0.469 | 1.056 | -6.99E-05 | -6.77E-03 | 1.18E-03 | -2.37E-03 | 3.37E-03 | 8.56E-03 |
| K02528 | 0.188 | 1.581 | 0.488 | 1.108 | 0.681 | 0.463 | 9.06E-04 | -1.49E-02 | 2.38E-03 | -5.59E-03 | 2.50E-03 | -3.10E-03 |
| K00969 | 0.926 | 0.378 | 0.658 | 0.827 | 0.048 | 0.478 | 4.53E-03 | 1.51E-03 | -2.96E-03 | -2.82E-03 | 3.24E-04 | 3.94E-03 |
| K01972 | 0.238 | 0.623 | 0.441 | 1.294 | 0.424 | 0.974 | 1.50E-03 | -6.00E-03 | 2.17E-03 | -6.62E-03 | -1.74E-04 | -5.85E-03 |
| K00075 | 0.754 | 0.288 | 0.767 | 0.815 | 1.045 | 0.451 | 4.56E-03 | 6.35E-04 | 2.77E-03 | -4.79E-03 | -5.55E-03 | 3.32E-03 |
| K01738 | 0.619 | 0.518 | 0.579 | 1.524 | 0.646 | 2.143 | -4.01E-03 | 2.18E-03 | -2.55E-03 | -8.39E-03 | -4.31E-03 | -1.72E-02 |
| K01921 | 0.684 | 0.58 | 0.263 | 1.296 | 0.494 | 0.957 | 4.42E-03 | 4.78E-03 | 7.42E-04 | -5.45E-03 | -3.19E-03 | 8.45E-03 |
| K01588 | 0.744 | 0.677 | 0.107 | 1.833 | 0.657 | 0.938 | 1.63E-03 | 6.67E-03 | -9.45E-05 | -1.15E-02 | -2.39E-03 | 7.50E-03 |
| K03602 | 0.725 | 0.272 | 1.063 | 1.591 | 0.368 | 0.503 | -4.87E-04 | 2.46E-03 | 2.86E-03 | -9.87E-03 | -2.85E-03 | 4.40E-03 |
| K03499 | 0.64 | 0.713 | 0.419 | 1.439 | 1.56 | 0.338 | -3.52E-03 | -4.46E-03 | 2.04E-03 | -8.13E-03 | -1.19E-02 | -2.75E-03 |
| K03498 | 0.789 | 0.693 | 0.544 | 1.572 | 1.104 | 0.787 | -5.02E-03 | -4.79E-03 | 2.38E-03 | -9.42E-03 | -8.68E-03 | -6.06E-03 |
| K07107 | 1.652 | 0.614 | 0.667 | 1.146 | 0.693 | 0.626 | 8.45E-03 | -1.42E-03 | -2.69E-03 | -6.67E-03 | -3.75E-03 | -2.35E-03 |
| K03308 | 0.773 | 0.735 | 0.497 | 0.643 | 0.939 | 1.471 | 4.99E-03 | -3.91E-03 | -4.26E-04 | -1.34E-03 | -6.47E-03 | 9.59E-03 |
| K02316 | 0.235 | 0.603 | 0.858 | 0.96 | 0.665 | 0.655 | -4.44E-04 | -4.56E-03 | 4.21E-03 | -4.79E-03 | 4.80E-03 | -2.54E-03 |
| K07052 | 0.646 | 0.442 | 1.431 | 0.188 | 0.414 | 0.863 | 2.87E-03 | 4.19E-03 | 6.73E-03 | -7.44E-04 | -2.72E-03 | -4.71E-03 |
| K03427 | 0.746 | 0.741 | 0.715 | 1.087 | 0.758 | 1.096 | 4.81E-03 | 4.37E-03 | -2.25E-03 | -4.62E-03 | 2.48E-03 | 9.59E-03 |
| K07273 | 0.445 | 0.474 | 1.136 | 0.62 | 0.12 | 2.122 | -1.75E-03 | 1.97E-03 | 4.18E-03 | -1.42E-03 | 6.86E-04 | -1.68E-02 |
| K21572 | 0.977 | 0.669 | 0.539 | 0.816 | 0.114 | 0.405 | 4.59E-03 | -1.53E-03 | -1.15E-03 | 4.59E-03 | -7.78E-04 | 2.17E-04 |
| K02466 | 0.709 | 0.913 | 0.496 | 0.539 | 0.494 | 0.684 | 3.83E-03 | -2.48E-03 | 2.44E-03 | 1.85E-03 | -2.46E-03 | -6.05E-03 |
| K02019 | 0.615 | 0.586 | 1.885 | 0.403 | 0.691 | 0.5 | -3.97E-03 | 5.40E-03 | 8.07E-03 | -7.38E-04 | 5.53E-03 | -4.43E-03 |
| K11936 | 0.467 | 0.707 | 0.469 | 0.62 | 0.737 | 1.791 | -1.64E-03 | -6.92E-03 | -1.47E-03 | 1.79E-03 | -2.16E-03 | 1.59E-02 |
| K02018 | 0.715 | 0.229 | 0.563 | 1.094 | 0.65 | 0.601 | -3.32E-03 | -1.60E-03 | 1.19E-03 | -5.64E-03 | -5.08E-03 | 1.46E-03 |
| K01119 | 0.764 | 1.027 | 0.16 | 0.394 | 0.489 | 0.624 | -4.25E-03 | 3.57E-03 | -3.32E-04 | -2.21E-03 | -1.87E-03 | -1.74E-03 |
| K01685 | 0.62 | 1.024 | 0.348 | 0.485 | 0.569 | 0.564 | 1.24E-03 | -2.34E-03 | 1.71E-03 | 3.01E-03 | 1.68E-04 | -4.95E-03 |
| K06872 | 0.731 | 0.295 | 0.729 | 0.882 | 0.635 | 0.737 | -4.51E-03 | 2.45E-03 | 1.89E-03 | -4.32E-03 | -3.63E-03 | -4.84E-03 |
| K03616 | 0.587 | 1.621 | 0.607 | 0.481 | 0.299 | 0.226 | -1.01E-03 | 1.20E-02 | -2.33E-03 | -1.37E-03 | -2.05E-03 | -1.89E-03 |
| K01873 | 0.441 | 0.597 | 0.675 | 0.846 | 0.638 | 0.575 | 9.18E-04 | 3.37E-03 | 2.17E-03 | -1.72E-03 | 4.26E-03 | 3.04E-03 |
| K18682 | 0.135 | 0.72 | 0.761 | 0.719 | 0.117 | 0.889 | -4.16E-04 | -5.57E-03 | 3.62E-03 | 1.85E-03 | -5.00E-04 | 1.01E-05 |
| K03613 | 0.757 | 0.496 | 0.699 | 1.386 | 0.394 | 0.424 | 1.08E-03 | -3.78E-03 | 9.36E-04 | -8.72E-03 | -1.05E-03 | 3.45E-03 |
| K00962 | 0.479 | 0.223 | 0.735 | 1.022 | 0.693 | 0.64 | 1.32E-03 | -7.86E-04 | -6.77E-04 | -5.08E-04 | 5.17E-03 | 3.16E-03 |
| K03043 | 0.778 | 0.374 | 0.786 | 0.987 | 0.406 | 0.654 | 3.47E-03 | -3.64E-03 | -8.21E-04 | -2.32E-03 | 2.74E-03 | 3.87E-03 |
| K04567 | 0.448 | 0.978 | 0.657 | 0.755 | 0.541 | 0.055 | 2.81E-03 | -9.56E-03 | -2.11E-05 | 8.36E-04 | 2.12E-03 | 1.20E-04 |

| | | | | | | | | | | | | |
|---|---|---|---|---|---|---|---|---|---|---|---|---|
| K02337 | 0.505 | 0.595 | 0.618 | 0.986 | 0.378 | 0.669 | -1.27E-03 | -4.62E-03 | -1.52E-03 | -1.56E-03 | 1.05E-03 | 2.44E-03 |
| K01890 | 0.267 | 0.667 | 0.591 | 1.014 | 0.515 | 0.781 | -8.64E-05 | -6.58E-03 | 4.29E-04 | -3.76E-03 | 3.65E-04 | 4.83E-03 |
| K11717 | 0.639 | 1.022 | 0.759 | 0.465 | 0.731 | 0.603 | -2.19E-03 | -5.13E-03 | 3.19E-03 | -2.91E-03 | 4.54E-03 | -5.34E-03 |
| K02519 | 0.59 | 0.473 | 0.734 | 0.941 | 0.512 | 0.685 | 3.35E-03 | -2.54E-03 | -1.61E-03 | -2.43E-03 | 2.14E-03 | 7.48E-04 |
| K03593 | 0.732 | 0.521 | 1.667 | 0.294 | 0.372 | 0.644 | -2.10E-03 | 1.67E-04 | 6.92E-03 | -7.69E-04 | 2.81E-03 | -3.89E-03 |
| K02040 | 0.325 | 0.268 | 0.944 | 0.597 | 0.32 | 0.312 | 1.12E-03 | 1.73E-03 | -3.58E-04 | -1.15E-03 | 4.18E-04 | -2.75E-03 |
| K03596 | 0.248 | 0.719 | 0.766 | 1.165 | 0.669 | 0.518 | 9.85E-04 | -6.81E-03 | -1.76E-03 | -5.20E-03 | 2.16E-03 | 1.47E-03 |
| K03572 | 0.434 | 0.535 | 0.617 | 0.738 | 0.489 | 1.235 | -2.80E-03 | 5.90E-04 | 2.85E-03 | -3.26E-03 | -2.27E-03 | 9.84E-03 |
| K00760 | 0.75 | 0.765 | 1.405 | 0.696 | 0.497 | 0.574 | -3.75E-03 | 4.47E-03 | 4.48E-03 | -4.23E-03 | -3.21E-03 | 8.00E-04 |
| K03106 | 0.514 | 0.696 | 0.705 | 1.058 | 0.765 | 0.538 | 3.32E-03 | -6.81E-03 | 3.07E-04 | -3.23E-03 | -2.48E-03 | 2.64E-03 |
| K01803 | 1.654 | 0.636 | 0.678 | 0.399 | 0.517 | 0.606 | 1.06E-02 | -5.35E-03 | 2.79E-03 | 8.97E-04 | -1.66E-03 | 5.01E-03 |
| K01810 | 0.433 | 0.628 | 0.643 | 1.277 | 0.573 | 0.775 | 2.68E-03 | -6.19E-03 | 3.55E-04 | -5.81E-03 | -4.93E-04 | 4.67E-03 |
| K02916 | 0.468 | 0.386 | 1.194 | 0.748 | 0.245 | 0.797 | -8.45E-04 | 2.07E-03 | 4.95E-03 | -3.18E-03 | -1.38E-03 | 7.02E-03 |
| K07005 | 0.404 | 0.249 | 0.517 | 0.745 | 1.208 | 0.576 | -2.26E-03 | -2.01E-03 | -2.52E-03 | -2.78E-03 | -6.46E-03 | -4.27E-03 |
| K06131 | 0.061 | 0.938 | 0.644 | 0.142 | 0.474 | 0.506 | -3.59E-04 | -2.31E-03 | 3.17E-03 | -8.59E-04 | -7.26E-04 | 5.61E-04 |
| K01790 | 0.746 | 1.189 | 0.471 | 0.505 | 0.678 | 0.313 | 6.22E-04 | 8.38E-03 | 1.60E-03 | -3.08E-03 | -4.10E-03 | -2.61E-03 |
| K01744 | 0.035 | 0.663 | 0.754 | 0.541 | 0.469 | 0.396 | 1.69E-04 | -6.10E-03 | 3.05E-03 | -1.28E-03 | 2.48E-03 | 3.37E-03 |
| K07098 | 0.681 | 0.729 | 0.332 | 0.072 | 0.641 | 0.574 | 1.00E-03 | 5.68E-03 | 1.63E-03 | -4.46E-04 | -3.90E-03 | -4.60E-03 |

**Trait: Methane Yield (g/kg DMI, CH4Y); Predictors: 673 heritable microbial genes**

| Microbial gene | VIP | | | | | | Regression coefficient | | | | | |
|---|---|---|---|---|---|---|---|---|---|---|---|---|
| | T1 | T2 | T3 | T4 | T5 | T6 | T1 | T2 | T3 | T4 | T5 | T6 |
| K01156$_{VIP\geq0.8\ (+)}$ | 1.015 | 1.622 | 2.24 | 2.17 | 2.156 | 1.234 | 6.95E-03 | 7.00E-03 | 1.54E-02 | 1.15E-02 | 1.56E-02 | 5.90E-03 |
| K01728$_{VIP\geq0.8\ (+)}$ | 1.125 | 1.512 | 1.287 | 0.76 | 1.315 | 0.954 | 1.53E-03 | 5.03E-03 | 9.15E-03 | 1.88E-03 | 1.05E-02 | 6.77E-03 |
| K00615$_{VIP\geq0.8\ (+)}$ | 0.696 | 0.94 | 0.993 | 1.161 | 1.54 | 1.027 | 4.75E-03 | 3.31E-03 | 4.27E-03 | 1.15E-03 | 7.69E-03 | 4.40E-03 |
| K00620$_{VIP\geq0.8\ (+)}$ | 0.971 | 0.592 | 1.263 | 1.062 | 0.444 | 1.584 | 7.78E-04 | 6.39E-04 | 4.18E-03 | 1.63E-03 | 8.52E-04 | 1.12E-02 |
| K00046$_{VIP\geq0.8\ (+)}$ | 0.93 | 1.568 | 0.899 | 0.445 | 1.323 | 0.582 | 5.70E-04 | 5.29E-03 | 4.93E-03 | 1.78E-04 | 1.02E-02 | 1.95E-03 |
| K07464$_{VIP\geq0.8\ (+)}$ | 0.582 | 0.442 | 1.072 | 1.071 | 1.123 | 0.924 | 1.37E-03 | 8.19E-05 | 2.48E-03 | 2.73E-03 | 2.77E-03 | 1.47E-03 |
| K01952$_{VIP\geq0.8\ (+)}$ | 0.424 | 0.396 | 1.101 | 1.09 | 0.86 | 1.291 | 7.64E-04 | 1.69E-03 | 4.73E-03 | 1.69E-03 | 1.62E-03 | 9.46E-04 |
| K01875$_{VIP\geq0.8\ (+)}$ | 0.313 | 0.516 | 1.614 | 1.193 | 0.966 | 1.021 | 1.88E-03 | 2.11E-03 | 9.11E-03 | 2.01E-03 | 2.66E-03 | 2.31E-03 |
| K02379$_{VIP\geq0.8\ (+)}$ | 1.065 | 0.972 | 1.267 | 1.057 | 1.788 | 1.665 | 4.15E-03 | 3.59E-03 | 9.64E-03 | 6.05E-03 | -1.31E-02 | 1.19E-02 |
| K11936$_{VIP\geq0.8\ (+)}$ | 1.683 | 1.344 | 0.889 | 2.193 | 1.08 | 1.085 | 1.12E-02 | 6.27E-03 | -6.77E-03 | 1.17E-02 | 5.22E-03 | 1.41E-04 |

| | | | | | | | | | | | | |
|---|---|---|---|---|---|---|---|---|---|---|---|---|
| K04567$_{VIP\geq0.8\ (+)}$ | 0.941 | 1.327 | 1.752 | 1.06 | 1.117 | 1.099 | 6.26E-03 | 5.79E-03 | 1.12E-02 | 3.87E-03 | -5.30E-03 | 1.92E-03 |
| K00641$_{VIP\geq0.8\ (+)}$ | 0.96 | 1.031 | 1.974 | 0.839 | 0.587 | 2.27 | 4.29E-03 | 2.56E-03 | 1.50E-02 | 4.28E-03 | -1.30E-04 | 1.61E-02 |
| K08963$_{VIP\geq0.8\ (+)}$ | 0.954 | 0.11 | 1.166 | 1.199 | 1.133 | 1.322 | 1.22E-03 | 6.38E-05 | 2.22E-03 | 7.57E-04 | -2.72E-03 | 5.72E-03 |
| K00053$_{VIP\geq0.8\ (+)}$ | 0.548 | 0.895 | 1.215 | 1.084 | 0.978 | 1.097 | 2.93E-03 | 4.24E-03 | 5.28E-03 | 2.80E-03 | 3.88E-03 | -4.13E-03 |
| K01873$_{VIP\geq0.8\ (+)}$ | 0.476 | 0.947 | 1.357 | 0.905 | 0.905 | 1.22 | -6.99E-04 | 4.16E-03 | 6.37E-03 | 6.02E-04 | 7.91E-04 | 3.23E-03 |
| K02337$_{VIP\geq0.8\ (+)}$ | 0.77 | 1.018 | 1.025 | 1.349 | 0.911 | 1.265 | -3.04E-03 | 4.22E-03 | 4.66E-03 | 3.82E-03 | 3.02E-04 | 3.59E-03 |
| K02519$_{VIP\geq0.8\ (+)}$ | 1.049 | 0.616 | 1.219 | 1.002 | 1.012 | 1.199 | -5.89E-03 | 1.74E-03 | 5.79E-03 | 7.13E-04 | 1.56E-03 | 1.57E-03 |
| K02967$_{VIP\geq0.8\ (+)}$ | 0.64 | 1.339 | 1.117 | 1.057 | 0.968 | 1.197 | 3.34E-03 | 6.00E-03 | 5.88E-03 | 1.15E-03 | -4.40E-03 | 3.17E-03 |
| K14118$_{VIP\geq0.8\ (+)}$ | 1.078 | 0.595 | 0.919 | 0.701 | 1.831 | 1.177 | 6.42E-03 | 3.87E-04 | 6.57E-03 | 7.05E-04 | -1.12E-02 | 4.43E-03 |
| K17884$_{VIP\geq0.8\ (+)}$ | 1.582 | 0.347 | 1 | 0.476 | 1.808 | 1.229 | 9.85E-03 | 1.06E-03 | 6.97E-03 | 2.64E-03 | -1.32E-02 | 8.23E-03 |
| K00441$_{VIP\geq0.8\ (+)}$ | 1.371 | 1.385 | 0.733 | 0.898 | 0.58 | 1.654 | 9.15E-03 | 6.16E-03 | 5.22E-03 | 5.20E-03 | -3.11E-04 | 1.16E-02 |
| K16214$_{VIP\geq0.8\ (+)}$ | 0.763 | 0.806 | 1.609 | 0.92 | 0.619 | 1.034 | 4.08E-03 | 1.07E-04 | 8.72E-03 | 4.11E-03 | -6.09E-06 | 1.87E-03 |
| K07045$_{VIP\geq0.8\ (+)}$ | 0.693 | 1.013 | 0.781 | 1.154 | 1.017 | 1.497 | 3.69E-04 | 4.67E-03 | 3.54E-03 | 6.62E-03 | -2.52E-03 | 9.56E-03 |
| K03639$_{VIP\geq0.8\ (+)}$ | 0.918 | 0.575 | 0.715 | 1 | 0.806 | 2.017 | 3.09E-03 | 2.76E-03 | 6.32E-04 | 2.76E-03 | -3.34E-03 | 1.39E-02 |
| K07089$_{VIP\geq0.8\ (+)}$ | 1.197 | 0.851 | 1.423 | 0.695 | 0.462 | 1.251 | 6.68E-03 | 5.56E-04 | 1.08E-02 | -8.07E-04 | 9.54E-04 | 6.91E-03 |
| K03614$_{VIP\geq0.8\ (+)}$ | 1.248 | 1.233 | 1.037 | 0.055 | 0.702 | 1.181 | 4.24E-03 | 3.10E-03 | 6.27E-03 | 2.30E-04 | -2.15E-03 | 7.04E-03 |
| K06919$_{VIP\geq0.8\ (+)}$ | 1.72 | 0.681 | 0.671 | 1.177 | 1.791 | 2.866 | -1.02E-02 | 2.62E-03 | 5.03E-03 | 4.61E-03 | 1.36E-02 | 1.86E-02 |
| K04771$_{VIP\geq0.8\ (+)}$ | 0.993 | 1.481 | 0.59 | 1.002 | 0.453 | 1.501 | -6.39E-03 | 5.95E-03 | 4.37E-03 | 4.45E-03 | 1.23E-03 | 6.66E-03 |
| K03517$_{VIP\geq0.8\ (+)}$ | 0.83 | 1.07 | 0.511 | 1.256 | 0.324 | 0.829 | 1.92E-03 | 3.63E-03 | 3.64E-03 | 7.21E-03 | 5.98E-04 | -4.95E-03 |
| K00927$_{VIP\geq0.8\ (+)}$ | 0.771 | 0.558 | 1.495 | 1.111 | 0.954 | 1.177 | 5.04E-03 | -1.76E-03 | 8.12E-03 | 1.84E-03 | 1.58E-03 | 4.22E-03 |
| K03572$_{VIP\geq0.8\ (+)}$ | 0.047 | 1.328 | 0.403 | 0.91 | 0.863 | 1.165 | 2.11E-04 | 5.72E-03 | 1.41E-03 | 1.80E-03 | -1.51E-03 | 3.16E-03 |
| K01889$_{VIP\geq0.8\ (+)}$ | 0.674 | 1.048 | 1.24 | 1.08 | 0.738 | 1.274 | 3.40E-03 | 3.79E-03 | 7.51E-03 | 3.22E-03 | -1.58E-03 | 7.15E-03 |
| K00059$_{VIP\geq0.8\ (+)}$ | 0.854 | 1.187 | 1.07 | 0.33 | 0.458 | 1.568 | -3.28E-04 | 1.73E-03 | 6.37E-03 | 1.69E-03 | 7.90E-04 | 1.09E-02 |
| K03308$_{VIP\geq0.8\ (+)}$ | 0.193 | 1.412 | 1.033 | 0.925 | 0.861 | 0.742 | 1.33E-03 | 5.56E-03 | 6.79E-03 | -4.17E-03 | 3.24E-04 | 4.67E-03 |
| K00558$_{VIP\geq0.8\ (+)}$ | 0.629 | 1.886 | 0.947 | 0.763 | 1.404 | 1.585 | -2.92E-03 | 8.32E-03 | 7.17E-03 | 1.60E-03 | 8.03E-03 | 9.22E-03 |
| K04656$_{VIP\geq0.8\ (+)}$ | 0.989 | 1.222 | 1.018 | 1.144 | 1.134 | 1.187 | 4.24E-03 | 5.74E-03 | 8.61E-04 | -1.76E-03 | -4.25E-03 | 5.24E-03 |
| K23393$_{VIP\geq0.8\ (+)}$ | 1.228 | 1.272 | 0.869 | 0.903 | 0.859 | 1.306 | -3.62E-03 | 5.98E-03 | -6.13E-04 | 7.53E-04 | 4.85E-03 | 7.95E-03 |
| K09121$_{VIP\geq0.8\ (+)}$ | 0.84 | 0.898 | 1.023 | 1.185 | 1.197 | 1.054 | 2.26E-03 | 4.12E-03 | 1.16E-03 | -3.63E-03 | -4.47E-03 | 5.61E-03 |
| K00283$_{VIP\geq0.8\ (+)}$ | 1.271 | 1.414 | 0.881 | 0.915 | 1.103 | 1.627 | -8.41E-03 | 6.73E-03 | 3.96E-03 | 4.99E-04 | -4.23E-04 | 8.90E-03 |
| K08309$_{VIP\geq0.8\ (+)}$ | 0.818 | 1.043 | 1.002 | 1.092 | 1.175 | 1.183 | 8.26E-04 | -1.42E-03 | -4.05E-03 | 1.40E-03 | 6.28E-03 | 1.91E-03 |

| | | | | | | | | | | | | |
|---|---|---|---|---|---|---|---|---|---|---|---|---|
| $K21395_{VIP \geq 0.8\ (+)}$ | 0.889 | 0.916 | 0.937 | 1.102 | 1.198 | 1.945 | -6.79E-04 | 3.26E-03 | 3.43E-03 | -8.75E-04 | 2.01E-03 | 1.07E-02 |
| $K00058_{VIP \geq 0.8\ (+)}$ | 0.824 | 0.856 | 0.995 | 1.047 | 1.273 | 1.052 | 2.92E-03 | 1.57E-03 | -3.51E-03 | -2.57E-03 | 4.34E-03 | 2.28E-03 |
| $K03118_{VIP \geq 0.8\ (+)}$ | 1.003 | 1.085 | 1.014 | 0.936 | 0.864 | 1.11 | 4.92E-04 | 1.92E-03 | 2.61E-04 | -4.12E-03 | -2.72E-03 | 5.62E-03 |
| $K09474_{VIP \geq 0.8\ (+)}$ | 1.228 | 0.978 | 1.015 | 0.951 | 1.032 | 1.35 | 2.60E-03 | -1.03E-04 | 1.24E-03 | -8.20E-04 | 1.33E-03 | 7.00E-03 |
| $K03431_{VIP \geq 0.8\ (+)}$ | 1.068 | 1.008 | 1.124 | 1.166 | 1.205 | 1.209 | 6.91E-04 | -9.50E-04 | 1.41E-03 | 1.16E-03 | -5.32E-03 | 3.26E-03 |
| $K04487_{VIP \geq 0.8\ (+)}$ | 1.057 | 0.847 | 1.039 | 1.134 | 1.03 | 1.025 | 1.13E-04 | 1.64E-03 | -9.18E-05 | 5.71E-04 | -4.87E-03 | 5.09E-04 |
| $K14092_{VIP \geq 0.8\ (+)}$ | 0.829 | 0.709 | 0.983 | 0.989 | 1.912 | 1.263 | 4.25E-03 | -9.09E-04 | 7.00E-03 | 5.28E-04 | -1.16E-02 | 6.19E-03 |
| $K14119_{VIP \geq 0.8\ (+)}$ | 1.052 | 0.477 | 0.901 | 0.886 | 1.976 | 1.38 | 4.96E-03 | 8.31E-04 | 5.97E-03 | -2.23E-03 | -1.21E-02 | 7.04E-03 |
| $K00887_{VIP \geq 0.8\ (+)}$ | 0.948 | 0.94 | 1.095 | 1.641 | 0.84 | 0.796 | 1.60E-04 | 2.69E-03 | -7.33E-03 | -9.06E-03 | 3.45E-03 | 1.97E-03 |
| $K02217_{VIP \geq 0.8\ (+)}$ | 0.358 | 0.831 | 0.866 | 0.985 | 0.966 | 0.808 | -2.35E-04 | 2.86E-03 | -9.71E-04 | 9.36E-04 | 2.03E-04 | 3.28E-03 |
| $K06885_{VIP \geq 0.8\ (+)}$ | 1.093 | 1.098 | 0.971 | 0.757 | 1.26 | 1.015 | -3.93E-03 | 1.73E-03 | 4.40E-03 | 1.19E-03 | -2.49E-03 | 1.60E-03 |
| $K02614_{VIP \geq 0.8\ (+)}$ | 0.985 | 0.786 | 0.949 | 0.96 | 1.786 | 1.143 | 1.14E-03 | 2.25E-04 | 4.20E-03 | -2.29E-03 | -8.42E-03 | 3.15E-03 |
| $K06894_{VIP \geq 0.8\ (+)}$ | 0.824 | 1.247 | 1.42 | 0.845 | 0.534 | 1.236 | -2.42E-03 | 3.33E-03 | 1.08E-02 | 4.63E-03 | -3.36E-03 | 8.55E-03 |
| $K04744_{VIP \geq 0.8\ (+)}$ | 1.23 | 1.101 | 0.827 | 0.485 | 1.405 | 0.988 | -5.34E-03 | 1.34E-03 | 4.09E-03 | -9.15E-04 | 1.02E-02 | 7.09E-03 |
| $K03183_{VIP \geq 0.8\ (+)}$ | 1.093 | 1.409 | 0.965 | 0.829 | 0.44 | 0.916 | -1.68E-03 | 3.41E-03 | 2.02E-04 | -2.15E-03 | 1.38E-03 | 5.55E-04 |
| $K06188_{VIP \geq 0.8\ (+)}$ | 1.234 | 1.17 | 0.997 | 0.973 | 0.788 | 1.193 | -6.62E-03 | -9.12E-05 | 2.76E-03 | 3.93E-03 | 4.46E-05 | 1.57E-03 |
| $K00812_{VIP \geq 0.8\ (+)}$ | 1.121 | 1.475 | 0.888 | 0.691 | 0.82 | 1.097 | -2.56E-03 | 3.73E-03 | 3.04E-03 | -1.63E-03 | 6.19E-03 | 7.45E-03 |
| $K00912_{VIP \geq 0.8\ (+)}$ | 1.36 | 1.47 | 0.915 | 1.027 | 1.441 | 0.604 | 3.45E-03 | 4.55E-03 | 1.63E-04 | -5.50E-03 | -1.11E-02 | 4.03E-03 |
| $K03973_{VIP \geq 0.8\ (+)}$ | 1.276 | 0.896 | 1.105 | 1.122 | 0.594 | 1.109 | 2.80E-03 | 6.37E-04 | -9.57E-04 | -4.49E-03 | 2.22E-03 | 1.98E-03 |
| $K03616_{VIP \geq 0.8\ (+)}$ | 0.982 | 1.747 | 0.467 | 1.391 | 1.404 | 0.965 | 4.67E-03 | 7.48E-03 | 4.19E-04 | -8.11E-03 | -1.09E-02 | 6.50E-03 |
| $K03613_{VIP \geq 0.8\ (+)}$ | 1.962 | 0.905 | 0.655 | 1.29 | 1.244 | 1.069 | 9.79E-03 | 1.60E-03 | 2.48E-03 | -7.51E-03 | -8.87E-03 | 5.35E-03 |
| $K03205_{VIP \geq 0.8\ (+)}$ | 0.838 | 0.525 | 1.155 | 1.182 | 1.062 | 1.293 | 2.28E-03 | -1.19E-03 | 2.27E-03 | -1.38E-03 | 1.15E-03 | 3.01E-03 |
| $K01912_{VIP \geq 0.8\ (+)}$ | 0.991 | 0.874 | 0.959 | 0.708 | 1.539 | 1.104 | 3.62E-04 | -4.94E-05 | 4.14E-03 | 7.16E-05 | -7.32E-03 | 4.01E-03 |
| $K04043_{VIP \geq 0.8\ (+)}$ | 1.065 | 0.8 | 1.31 | 1.622 | 0.919 | 1.227 | -4.13E-03 | 3.84E-03 | 6.15E-03 | 6.24E-03 | -3.93E-03 | 2.51E-03 |
| $K01890_{VIP \geq 0.8\ (+)}$ | 0.552 | 0.848 | 1.5 | 1.078 | 1.049 | 1.222 | -2.35E-03 | 3.53E-03 | 8.05E-03 | 1.58E-03 | -1.32E-05 | 2.13E-03 |
| $K04077_{VIP \geq 0.8\ (+)}$ | 0.513 | 1.293 | 1.03 | 1.302 | 1.037 | 1.144 | -3.37E-03 | 5.18E-03 | 4.59E-03 | 3.30E-03 | 2.48E-03 | -2.60E-03 |
| $K00760_{VIP \geq 0.8\ (+)}$ | 0.967 | 1.003 | 1.461 | 1.173 | 0.139 | 0.81 | 2.98E-03 | 2.05E-03 | -8.28E-03 | -6.83E-03 | 2.77E-04 | 2.43E-03 |
| $K03437_{VIP \geq 0.8\ (+)}$ | 0.82 | 1.377 | 0.972 | 1.02 | 0.175 | 0.958 | -2.09E-03 | 3.53E-03 | 3.20E-05 | -5.58E-03 | 1.01E-03 | 6.30E-03 |
| $K02838_{VIP \geq 0.8\ (+)}$ | 0.949 | 1.053 | 0.889 | 1.222 | 0.182 | 1.427 | 2.01E-03 | -3.39E-03 | 3.38E-03 | -5.75E-03 | 4.63E-04 | 9.20E-03 |
| $K03497_{VIP \geq 0.8\ (+)}$ | 0.519 | 1.056 | 0.997 | 1.253 | 0.97 | 1.355 | 4.94E-04 | 5.17E-03 | -4.77E-03 | -2.86E-03 | 3.13E-04 | 4.09E-03 |

| | | | | | | | | | | | | |
|---|---|---|---|---|---|---|---|---|---|---|---|---|
| K03049$_{VIP\geq0.8\ (+)}$ | 2.585 | 0.857 | 0.751 | 0.629 | 1.424 | 1.401 | 1.57E-02 | -8.98E-04 | 4.14E-03 | 1.64E-03 | -8.04E-03 | 9.35E-03 |
| K14095$_{VIP\geq0.8\ (+)}$ | 0.87 | 0.633 | 0.608 | 0.829 | 1.719 | 1.409 | 5.43E-03 | -1.14E-03 | 2.62E-03 | 1.52E-03 | -9.49E-03 | 7.37E-03 |
| K14102$_{VIP\geq0.8\ (+)}$ | 1.681 | 0.669 | 1.031 | 0.751 | 1.66 | 1.065 | 1.06E-02 | -1.40E-03 | 7.40E-03 | 2.02E-03 | -8.89E-03 | 9.99E-04 |
| K14117$_{VIP\geq0.8\ (+)}$ | 1.147 | 0.489 | 0.889 | 0.645 | 1.77 | 1.271 | 5.82E-03 | 1.24E-04 | 5.85E-03 | -1.10E-04 | -1.03E-02 | 4.91E-03 |
| K07248$_{VIP\geq0.8\ (+)}$ | 0.996 | 0.945 | 0.7 | 0.378 | 1.074 | 1.043 | 4.42E-03 | 9.82E-04 | -1.34E-03 | 1.42E-03 | -7.88E-03 | 6.67E-03 |
| K02018$_{VIP\geq0.8\ (+)}$ | 0.555 | 1.205 | 0.383 | 0.913 | 1.576 | 1.461 | 3.58E-03 | 5.32E-03 | -1.29E-05 | 2.13E-04 | -1.26E-02 | 1.05E-02 |
| K07502$_{VIP\geq0.8\ (+)}$ | 0.941 | 0.645 | 1.134 | 1.31 | 0.913 | 0.642 | 2.53E-03 | 5.52E-04 | 4.07E-03 | -6.64E-03 | -5.39E-03 | 4.60E-03 |
| K13684$_{VIP\geq0.8\ (+)}$ | 0.68 | 2.068 | 1.497 | 0.841 | 1.684 | 0.686 | 2.74E-03 | 9.52E-03 | -1.10E-02 | -4.50E-03 | 1.32E-02 | 4.58E-03 |
| K07794$_{VIP\geq0.8\ (+)}$ | 1.531 | 0.531 | 0.789 | 1.226 | 1.773 | 1.716 | 1.03E-02 | 1.58E-03 | -5.95E-03 | -5.96E-03 | 1.00E-02 | 1.15E-02 |
| K09516$_{VIP\geq0.8\ (+)}$ | 0.862 | 1.112 | 0.806 | 0.755 | 0.706 | 1.001 | -2.16E-03 | -4.53E-04 | 3.86E-03 | 2.88E-03 | 5.66E-03 | 3.14E-03 |
| K01255$_{VIP\geq0.8\ (+)}$ | 1.837 | 0.656 | 0.777 | 0.885 | 1.099 | 1.213 | 1.27E-02 | -1.01E-03 | -1.32E-03 | 3.01E-03 | 9.36E-04 | 7.10E-03 |
| K00549$_{VIP\geq0.8\ (+)}$ | 0.98 | 0.494 | 0.939 | 0.791 | 0.822 | 1.083 | -2.39E-03 | 1.60E-03 | 2.31E-04 | -1.38E-03 | 1.48E-03 | 4.09E-03 |
| K15894$_{VIP\geq0.8\ (+)}$ | 0.276 | 1.228 | 1.088 | 0.324 | 1.01 | 1.658 | 1.90E-03 | 4.55E-03 | -7.38E-03 | -6.20E-04 | 6.07E-03 | 1.19E-02 |
| K03151$_{VIP\geq0.8\ (+)}$ | 1.094 | 0.708 | 1.012 | 1.062 | 0.963 | 0.756 | 8.76E-04 | -3.46E-04 | -2.22E-03 | 7.82E-04 | 2.18E-03 | 4.23E-03 |
| K02574$_{VIP\geq0.8\ (+)}$ | 0.767 | 1.096 | 0.952 | 0.643 | 1.414 | 1.157 | 4.72E-04 | 2.53E-03 | 4.88E-03 | -1.74E-03 | -6.31E-03 | 2.60E-03 |
| K00703$_{VIP\geq0.8\ (+)}$ | 1.031 | 1.228 | 0.884 | 0.783 | 0.789 | 1.338 | -8.58E-04 | -3.29E-03 | 7.09E-04 | 1.96E-03 | 4.19E-04 | 7.84E-03 |
| K23675$_{VIP\geq0.8\ (+)}$ | 1.096 | 0.76 | 1.106 | 1.1 | 0.937 | 0.643 | 4.75E-03 | 1.77E-03 | -5.56E-03 | -3.23E-03 | 1.67E-03 | 2.13E-03 |
| K01144$_{VIP\geq0.8\ (+)}$ | 1.04 | 1.437 | 0.842 | 0.672 | 1.613 | 0.248 | -3.45E-03 | 4.31E-03 | 4.64E-03 | 3.32E-03 | -1.13E-02 | 1.52E-03 |
| K00241$_{VIP\geq0.8\ (+)}$ | 1.056 | 1.288 | 0.891 | 0.579 | 0.847 | 0.583 | -1.88E-03 | 1.21E-03 | 7.92E-04 | -2.80E-03 | 6.79E-03 | 2.53E-03 |
| K01515$_{VIP\geq0.8\ (+)}$ | 0.644 | 0.925 | 1.051 | 1.022 | 0.854 | 0.749 | 2.22E-03 | 5.35E-04 | -5.58E-03 | -1.46E-03 | 4.76E-04 | 2.05E-03 |
| K03525$_{VIP\geq0.8\ (+)}$ | 1.414 | 1.077 | 0.551 | 0.868 | 1.179 | 0.739 | 9.75E-03 | -4.73E-03 | 3.33E-03 | -2.62E-03 | 5.98E-03 | 5.30E-03 |
| K03612$_{VIP\geq0.8\ (+)}$ | 1.119 | 1.132 | 0.716 | 0.733 | 2.462 | 0.948 | 2.95E-03 | 3.03E-03 | 2.40E-04 | -4.10E-03 | -1.82E-02 | 4.35E-03 |
| K13444$_{VIP\geq0.8\ (+)}$ | 1.331 | 1.103 | 1.02 | 0.577 | 0.333 | 0.982 | 1.43E-03 | -7.33E-04 | 9.17E-04 | 1.19E-03 | -6.19E-04 | 3.44E-03 |
| K18682$_{VIP\geq0.8\ (+)}$ | 0.361 | 1.45 | 0.805 | 0.836 | 1.63 | 0.663 | -2.47E-03 | 5.45E-03 | 4.66E-03 | 2.13E-03 | 1.05E-02 | -2.80E-03 |
| K00962$_{VIP\geq0.8\ (+)}$ | 0.792 | 0.556 | 1.65 | 1.095 | 0.934 | 1.241 | -3.17E-03 | 2.57E-03 | 9.36E-03 | 1.22E-03 | -5.84E-03 | 2.71E-03 |
| K19824$_{VIP\geq0.8\ (+)}$ | 1.019 | 1.071 | 0.748 | 1.15 | 0.635 | 0.895 | 3.45E-03 | 5.29E-04 | -2.40E-03 | -6.68E-03 | 4.06E-03 | 2.08E-03 |
| K03043$_{VIP\geq0.8\ (+)}$ | 0.65 | 0.96 | 1.412 | 1.221 | 0.778 | 1.318 | -9.37E-04 | 4.57E-03 | 6.79E-03 | 2.89E-03 | -1.94E-03 | 3.68E-04 |
| K01854$_{VIP\geq0.8\ (+)}$ | 1.435 | 0.714 | 0.904 | 0.659 | 1.174 | 1.667 | -8.25E-03 | 1.84E-03 | 4.81E-03 | -1.15E-03 | 2.47E-03 | 9.22E-03 |
| K01869$_{VIP\geq0.8\ (+)}$ | 0.7 | 0.474 | 1.865 | 1.212 | 0.945 | 1.357 | -3.81E-03 | 2.30E-03 | 1.10E-02 | 3.29E-03 | -4.16E-03 | 4.56E-03 |
| K07460$_{VIP\geq0.8\ (+)}$ | 1.223 | 1.022 | 1.455 | 0.788 | 0.393 | 1.463 | 3.56E-03 | 3.20E-03 | -7.39E-03 | -4.21E-03 | 3.30E-04 | 9.54E-03 |

| | | | | | | | | | | | | |
|---|---|---|---|---|---|---|---|---|---|---|---|---|
| K07391$_{VIP\geq0.8\ (+)}$ | 0.71 | 1.525 | 0.748 | 0.818 | 1.107 | 1.425 | 3.92E-03 | 6.62E-03 | -5.32E-03 | -1.24E-03 | 5.45E-03 | 8.37E-03 |
| K01883$_{VIP\geq0.8\ (+)}$ | 0.58 | 0.75 | 1.16 | 1.14 | 1.258 | 1.295 | -3.85E-03 | 2.87E-03 | 6.42E-03 | 1.91E-03 | -8.40E-03 | 4.65E-03 |
| K01810$_{VIP\geq0.8\ (+)}$ | 0.254 | 0.684 | 1.092 | 1.202 | 1.019 | 1.307 | -1.40E-03 | 2.57E-03 | 4.19E-03 | 1.27E-03 | -2.53E-03 | 2.84E-03 |
| K03655$_{VIP\geq0.8\ (+)}$ | 0.482 | 0.547 | 1.183 | 1.134 | 1.129 | 1.273 | 3.08E-03 | -2.65E-03 | 5.85E-03 | 1.06E-04 | -4.17E-04 | 1.71E-04 |
| K03168$_{VIP\geq0.8\ (+)}$ | 0.142 | 1.22 | 0.476 | 1.12 | 0.975 | 1.492 | 9.45E-04 | 5.16E-03 | 1.71E-03 | -3.20E-03 | -1.62E-03 | 6.31E-03 |
| K00705$_{VIP\geq0.8\ (+)}$ | 1.236 | 1.669 | 1.492 | 0.104 | 1.362 | 0.419 | -8.26E-03 | 7.05E-03 | 1.05E-02 | 2.11E-04 | -9.66E-03 | 2.77E-03 |
| K00075$_{VIP\geq0.8\ (+)}$ | 0.137 | 1.142 | 0.991 | 0.845 | 1.172 | 0.706 | -5.16E-04 | 5.58E-03 | 1.94E-03 | -4.68E-03 | 5.24E-04 | 1.37E-03 |
| K02316$_{VIP\geq0.8\ (+)}$ | 0.659 | 1.326 | 1.548 | 1.127 | 0.739 | 0.973 | -4.20E-03 | 5.33E-03 | 1.15E-02 | 3.42E-03 | -7.63E-04 | 1.08E-03 |
| K04042$_{VIP\geq0.8\ (-)}$ | 0.971 | 1.21 | 1.005 | 1.02 | 1.185 | 1.261 | -2.16E-03 | -3.85E-03 | -4.32E-03 | -4.03E-04 | -5.20E-03 | -3.08E-03 |
| K18928$_{VIP\geq0.8\ (-)}$ | 1.259 | 1.182 | 1.162 | 1.025 | 1.051 | 0.805 | -6.91E-03 | -2.46E-04 | -3.67E-03 | -3.89E-03 | -5.30E-03 | -9.90E-04 |
| K07105$_{VIP\geq0.8\ (-)}$ | 1.15 | 0.883 | 1.035 | 1.332 | 0.945 | 0.878 | -1.80E-03 | -1.07E-03 | -2.81E-03 | -6.61E-03 | -3.89E-03 | -3.35E-03 |
| K03086$_{VIP\geq0.8\ (-)}$ | 1.001 | 1.069 | 1.068 | 1.146 | 1.186 | 1.007 | -5.53E-04 | -2.11E-03 | -3.22E-03 | -1.24E-03 | -4.41E-03 | -2.58E-03 |
| K00975$_{VIP\geq0.8\ (-)}$ | 1.158 | 0.913 | 1.069 | 1.174 | 1.023 | 0.974 | -5.77E-04 | -1.17E-03 | -1.54E-03 | -6.93E-04 | -3.43E-03 | -1.73E-03 |
| K07485$_{VIP\geq0.8\ (-)}$ | 0.886 | 0.989 | 1.096 | 1.081 | 1.301 | 1.185 | -4.06E-03 | -3.75E-03 | -6.59E-03 | -1.69E-03 | -6.09E-03 | -3.75E-03 |
| K02822$_{VIP\geq0.8\ (-)}$ | 1.198 | 0.522 | 1.12 | 1.235 | 0.987 | 1.108 | -6.87E-03 | -1.86E-03 | -7.86E-03 | -6.34E-03 | -4.60E-03 | -7.47E-03 |
| K07148$_{VIP\geq0.8\ (-)}$ | 0.739 | 0.964 | 0.854 | 1.06 | 1.348 | 0.851 | -7.63E-04 | -2.20E-04 | -5.19E-04 | -5.36E-03 | -4.86E-03 | -1.54E-03 |
| K05364$_{VIP\geq0.8\ (-)}$ | 1.171 | 0.573 | 1.059 | 1.171 | 1.15 | 1.027 | -3.13E-03 | -6.58E-04 | -4.41E-03 | -2.36E-03 | -1.43E-03 | -3.32E-03 |
| K03741$_{VIP\geq0.8\ (-)}$ | 1.129 | 0.602 | 0.949 | 1.171 | 1.093 | 1.286 | -4.62E-03 | -1.18E-03 | -1.45E-03 | -4.98E-03 | -2.20E-03 | -5.20E-03 |
| K22452$_{VIP\geq0.8\ (-)}$ | 0.769 | 0.897 | 0.96 | 1.379 | 0.829 | 0.923 | -1.03E-03 | -2.30E-04 | -4.72E-03 | -6.06E-03 | -4.39E-03 | -4.98E-03 |
| K02027$_{VIP\geq0.8\ (-)}$ | 0.962 | 0.527 | 0.997 | 1.254 | 1.383 | 0.957 | -4.70E-04 | -5.55E-04 | -5.95E-03 | -3.65E-03 | -8.92E-03 | -1.20E-04 |
| K02038$_{VIP\geq0.8\ (-)}$ | 1.137 | 0.661 | 1.044 | 1.159 | 0.975 | 1.149 | -2.99E-03 | -1.32E-03 | -2.17E-03 | -2.33E-03 | -3.53E-04 | -2.88E-03 |
| K18908$_{VIP\geq0.8\ (-)}$ | 1.364 | 0.651 | 1.001 | 1.536 | 0.862 | 1.088 | -4.74E-03 | -2.57E-03 | -1.87E-03 | -7.96E-03 | -1.20E-03 | -7.54E-03 |
| K00941$_{VIP\geq0.8\ (-)}$ | 0.616 | 0.902 | 1.1 | 1.073 | 1.162 | 1.088 | -1.03E-03 | -8.49E-04 | -6.48E-03 | -1.27E-04 | -8.36E-03 | -6.96E-03 |
| K05832$_{VIP\geq0.8\ (-)}$ | 1.678 | 0.722 | 1.168 | 1.189 | 1.252 | 0.815 | -8.25E-03 | -1.44E-03 | -7.59E-03 | -2.94E-03 | -7.18E-03 | -1.06E-03 |
| K02037$_{VIP\geq0.8\ (-)}$ | 1.13 | 0.581 | 1.077 | 1.27 | 1 | 1.26 | -4.03E-03 | -1.10E-03 | -5.56E-03 | -4.52E-03 | -2.36E-03 | -4.88E-03 |
| K02036$_{VIP\geq0.8\ (-)}$ | 1.165 | 0.578 | 1.073 | 1.18 | 1.023 | 1.236 | -4.16E-03 | -4.65E-04 | -4.68E-03 | -3.39E-03 | -2.14E-03 | -4.75E-03 |
| K06215$_{VIP\geq0.8\ (-)}$ | 1.455 | 0.53 | 1.147 | 1.29 | 1.566 | 0.925 | -9.55E-03 | -1.17E-03 | -8.39E-03 | -7.11E-03 | -1.22E-02 | -6.51E-03 |
| K03778$_{VIP\geq0.8\ (-)}$ | 1.129 | 0.536 | 0.912 | 1.1 | 1.117 | 1.758 | -3.97E-03 | -9.53E-04 | -4.05E-03 | -1.57E-03 | -7.26E-03 | -1.19E-02 |
| K21498$_{VIP\geq0.8\ (-)}$ | 0.231 | 0.836 | 1.595 | 1.449 | 1.059 | 1.373 | -2.35E-04 | -3.94E-03 | -1.21E-02 | -8.44E-03 | -4.24E-03 | -9.67E-03 |
| K06199$_{VIP\geq0.8\ (-)}$ | 0.738 | 0.979 | 1.343 | 1.107 | 1.079 | 0.981 | -2.99E-03 | -2.30E-03 | -9.42E-03 | -5.00E-03 | -3.63E-03 | -4.19E-03 |

| | | | | | | | | | | | | |
|---|---|---|---|---|---|---|---|---|---|---|---|---|
| K00794$_{VIP≥0.8\ (-)}$ | 0.982 | 0.957 | 1.233 | 1.599 | 0.777 | 0.955 | -2.45E-03 | -2.23E-03 | -4.93E-03 | -8.30E-03 | -5.46E-03 | -2.09E-03 |
| K01193$_{VIP≥0.8\ (-)}$ | 0.821 | 0.677 | 1.404 | 1.176 | 1.397 | 0.441 | -9.99E-04 | -3.00E-03 | -1.06E-02 | -5.30E-03 | -1.10E-02 | -1.66E-03 |
| K00275$_{VIP≥0.8\ (-)}$ | 0.371 | 1.219 | 1.546 | 0.783 | 1.082 | 0.827 | -1.40E-03 | -2.87E-03 | -1.18E-02 | -3.43E-03 | -6.04E-03 | -3.03E-03 |
| K05601$_{VIP≥0.8\ (-)}$ | 1.029 | 0.458 | 1.142 | 1.131 | 1.537 | 0.58 | -3.20E-03 | -4.30E-05 | -6.36E-03 | -1.85E-03 | -1.21E-02 | -2.43E-03 |
| K04085$_{VIP≥0.8\ (-)}$ | 0.314 | 0.861 | 1.776 | 0.992 | 0.743 | 0.928 | -2.08E-03 | -3.09E-03 | -1.35E-02 | -5.51E-03 | -1.70E-03 | -4.24E-03 |
| K02199$_{VIP≥0.8\ (-)}$ | 1.093 | 0.976 | 1.304 | 1.198 | 0.483 | 0.595 | -5.11E-03 | -1.32E-03 | -6.20E-03 | -6.25E-03 | -3.18E-03 | -6.41E-04 |
| K07979$_{VIP≥0.8\ (-)}$ | 0.982 | 0.46 | 1.232 | 1.023 | 0.887 | 0.526 | -5.38E-03 | -1.74E-03 | -9.43E-03 | -5.64E-03 | -7.05E-03 | -1.70E-03 |
| K05337$_{VIP≥0.8\ (-)}$ | 1.121 | 1.514 | 1.588 | 1.231 | 1.064 | 1.322 | 7.62E-03 | -5.24E-03 | -1.21E-02 | -6.81E-03 | -7.57E-03 | -8.92E-03 |
| K03303$_{VIP≥0.8\ (-)}$ | 1.051 | 1.114 | 1.066 | 0.933 | 2.291 | 0.855 | 3.68E-03 | -3.53E-03 | -3.03E-03 | -5.44E-03 | -1.46E-02 | -5.72E-03 |
| K00797$_{VIP≥0.8\ (-)}$ | 1.174 | 1.089 | 0.95 | 0.866 | 0.85 | 1.353 | 8.10E-03 | -2.68E-03 | -3.80E-03 | -1.60E-03 | -4.63E-03 | -9.32E-03 |
| K01902$_{VIP≥0.8\ (-)}$ | 1.096 | 0.831 | 0.901 | 1.24 | 1.236 | 1.028 | -4.90E-03 | -2.39E-03 | -3.98E-03 | 3.67E-03 | -5.00E-03 | -1.10E-04 |
| K09807$_{VIP≥0.8\ (-)}$ | 0.902 | 0.975 | 0.951 | 1.038 | 1.405 | 1.176 | -1.47E-03 | -2.45E-03 | -4.29E-03 | -7.93E-04 | -8.80E-03 | 3.07E-03 |
| K01626$_{VIP≥0.8\ (-)}$ | 0.951 | 1.008 | 1.06 | 1.141 | 1.224 | 1.214 | 8.35E-04 | -2.22E-03 | -2.88E-03 | -6.09E-04 | -4.29E-03 | -5.09E-03 |
| K11358$_{VIP≥0.8\ (-)}$ | 1.001 | 1.402 | 0.962 | 1.139 | 1.119 | 1.102 | -1.07E-03 | -4.40E-03 | 3.64E-05 | -4.88E-03 | -4.86E-03 | -3.33E-03 |
| K00995$_{VIP≥0.8\ (-)}$ | 0.881 | 1.171 | 1.29 | 1.048 | 0.91 | 0.927 | 5.12E-03 | -2.29E-03 | -8.40E-03 | -2.18E-03 | -4.03E-03 | -3.30E-04 |
| K02072$_{VIP≥0.8\ (-)}$ | 1.01 | 0.863 | 1.049 | 1.298 | 1.034 | 0.841 | 1.77E-04 | -7.89E-04 | -2.14E-03 | -4.26E-03 | -5.01E-03 | -4.23E-03 |
| K02025$_{VIP≥0.8\ (-)}$ | 1.033 | 0.803 | 0.946 | 1.268 | 1.233 | 0.926 | -1.81E-03 | -2.80E-03 | -5.20E-03 | -3.89E-03 | -5.96E-03 | 1.64E-04 |
| K03704$_{VIP≥0.8\ (-)}$ | 0.837 | 0.913 | 1.239 | 1.49 | 1.213 | 1.375 | 2.94E-03 | -1.50E-03 | -7.46E-03 | -7.12E-03 | -5.70E-03 | -8.79E-03 |
| K04083$_{VIP≥0.8\ (-)}$ | 0.921 | 1.041 | 1.102 | 1.134 | 1.085 | 0.928 | 2.68E-03 | -2.23E-03 | -4.37E-03 | -2.07E-03 | -4.88E-03 | -1.01E-03 |
| K06958$_{VIP≥0.8\ (-)}$ | 1.017 | 1.171 | 1.038 | 1.086 | 1.164 | 1.028 | -4.78E-05 | -3.35E-03 | -3.41E-03 | -1.70E-03 | -5.51E-03 | 2.05E-04 |
| K04751$_{VIP≥0.8\ (-)}$ | 0.897 | 0.836 | 1.604 | 1.062 | 1.104 | 0.921 | -6.11E-03 | 3.15E-03 | -1.22E-02 | -3.34E-03 | -5.51E-03 | -4.44E-03 |
| K02073$_{VIP≥0.8\ (-)}$ | 0.909 | 0.896 | 1.039 | 1.303 | 1.044 | 1.097 | 1.70E-03 | -1.82E-03 | -2.40E-03 | -4.54E-03 | -3.66E-03 | -5.04E-03 |
| K05808$_{VIP≥0.8\ (-)}$ | 0.949 | 1.092 | 1.146 | 1.304 | 1.001 | 0.926 | 2.87E-03 | -1.58E-03 | -6.14E-03 | -4.93E-03 | -4.06E-03 | -1.85E-03 |
| K02040$_{VIP≥0.8\ (-)}$ | 0.857 | 1.252 | 0.92 | 1.226 | 0.821 | 1.291 | -4.72E-03 | 6.01E-03 | -4.01E-03 | -6.59E-03 | -3.56E-03 | -6.91E-03 |
| K02483$_{VIP≥0.8\ (-)}$ | 0.847 | 0.845 | 0.985 | 1.378 | 1.217 | 0.951 | 2.68E-03 | -1.30E-03 | -3.40E-03 | -5.11E-03 | -3.76E-03 | -2.95E-03 |
| K03310$_{VIP≥0.8\ (-)}$ | 0.841 | 1.24 | 1.113 | 1.199 | 1.094 | 0.928 | -4.82E-03 | 6.04E-03 | -7.40E-03 | -5.19E-03 | -2.05E-04 | -1.67E-03 |
| K07154$_{VIP≥0.8\ (-)}$ | 1.24 | 1.325 | 0.941 | 1.549 | 1.322 | 0.937 | -7.48E-03 | 4.01E-03 | -3.75E-03 | -8.87E-03 | -9.50E-03 | -3.33E-03 |
| K06962$_{VIP≥0.8\ (-)}$ | 1.265 | 0.305 | 0.993 | 1.041 | 1.357 | 1.193 | -7.04E-03 | -1.14E-03 | -1.13E-03 | -1.38E-04 | -8.10E-03 | 4.61E-03 |
| K02759$_{VIP≥0.8\ (-)}$ | 0.825 | 0.437 | 0.98 | 1.33 | 1.251 | 1.099 | -3.66E-03 | 1.65E-03 | -4.77E-03 | -5.08E-03 | -4.02E-03 | -3.62E-03 |
| K00965$_{VIP≥0.8\ (-)}$ | 1.036 | 0.77 | 0.937 | 1.116 | 1.298 | 1.125 | 3.27E-03 | -1.70E-03 | -2.88E-03 | -8.08E-04 | -9.35E-03 | -6.73E-03 |

| | | | | | | | | | | | | |
|---|---|---|---|---|---|---|---|---|---|---|---|---|
| $K06209_{VIP≥0.8\ (-)}$ | 1.158 | 0.861 | 1.046 | 1.304 | 1.293 | 0.621 | 5.71E-03 | -9.16E-04 | -3.71E-03 | -4.97E-03 | -6.79E-03 | -2.74E-03 |
| $K00626_{VIP≥0.8\ (-)}$ | 1.073 | 0.611 | 1.059 | 1.031 | 1.379 | 1.043 | 8.83E-04 | -5.28E-04 | -4.00E-04 | -2.65E-03 | -8.93E-03 | -7.06E-03 |
| $K02039_{VIP≥0.8\ (-)}$ | 1.454 | 0.609 | 1.036 | 1.176 | 0.943 | 1.126 | -5.96E-03 | -6.19E-04 | -9.03E-04 | -2.54E-03 | 5.27E-04 | -4.13E-03 |
| $K03929_{VIP≥0.8\ (-)}$ | 0.808 | 0.697 | 1.216 | 1.296 | 0.827 | 0.805 | -2.34E-03 | 5.39E-04 | -8.26E-03 | -7.45E-03 | -6.58E-03 | -4.25E-03 |
| $K02031_{VIP≥0.8\ (-)}$ | 1.123 | 0.499 | 1.034 | 1.5 | 1.292 | 0.933 | -2.47E-03 | -1.43E-04 | -5.38E-03 | -7.10E-03 | -6.95E-03 | 2.15E-03 |
| $K22927_{VIP≥0.8\ (-)}$ | 0.94 | 0.564 | 1.026 | 1.204 | 0.982 | 0.964 | 1.69E-03 | -3.30E-04 | -8.41E-04 | -1.53E-04 | -1.17E-03 | -3.46E-03 |
| $K08602_{VIP≥0.8\ (-)}$ | 1.043 | 0.815 | 1.011 | 1.341 | 1.225 | 0.636 | -1.69E-03 | -2.51E-03 | -2.75E-05 | -5.44E-03 | -6.17E-03 | 1.08E-03 |
| $K23535_{VIP≥0.8\ (-)}$ | 0.846 | 0.485 | 0.866 | 1.33 | 1.222 | 1.125 | -3.80E-04 | -5.54E-04 | -2.88E-03 | -5.19E-03 | -4.61E-03 | 5.65E-03 |
| $K23536_{VIP≥0.8\ (-)}$ | 0.89 | 0.789 | 0.896 | 1.425 | 1.262 | 1.153 | -7.89E-04 | -2.58E-03 | -2.01E-03 | -6.43E-03 | -6.30E-03 | 6.54E-03 |
| $K06975_{VIP≥0.8\ (-)}$ | 0.166 | 1.228 | 0.867 | 1.03 | 1.096 | 1.096 | -7.32E-04 | -3.96E-03 | 2.53E-03 | -9.95E-04 | -4.61E-03 | -5.68E-03 |
| $K00340_{VIP≥0.8\ (-)}$ | 1.151 | 1.535 | 1.187 | 0.895 | 0.654 | 0.956 | -1.74E-03 | 4.69E-03 | -4.62E-03 | -3.09E-03 | -3.13E-03 | -3.00E-03 |
| $K02026_{VIP≥0.8\ (-)}$ | 0.789 | 0.86 | 1.081 | 1.145 | 1.143 | 1.077 | -1.08E-03 | -3.76E-03 | -8.05E-03 | -2.91E-03 | -5.50E-03 | 4.10E-03 |
| $K07574_{VIP≥0.8\ (-)}$ | 0.983 | 0.948 | 1.028 | 1.107 | 0.952 | 0.298 | 3.47E-03 | -1.21E-03 | -5.90E-03 | -3.90E-03 | -7.03E-03 | -2.13E-03 |
| $K00992_{VIP≥0.8\ (-)}$ | 1.08 | 1.237 | 1.024 | 1.132 | 0.921 | 0.73 | -2.73E-03 | 4.14E-03 | -4.94E-03 | -6.28E-03 | -7.34E-03 | -5.04E-03 |
| $K21064_{VIP≥0.8\ (-)}$ | 0.891 | 1.031 | 0.857 | 0.809 | 0.788 | 0.813 | -4.49E-03 | -1.67E-03 | -4.50E-03 | 1.03E-03 | -5.67E-03 | -1.10E-03 |
| $K02114_{VIP≥0.8\ (-)}$ | 0.67 | 0.837 | 0.941 | 1.056 | 1.408 | 1.409 | 5.91E-04 | -2.54E-03 | -3.39E-03 | -1.44E-03 | -8.47E-03 | -7.87E-03 |
| $K00793_{VIP≥0.8\ (-)}$ | 1.279 | 1.433 | 1.576 | 1.5 | 0.691 | 1.506 | -8.09E-03 | 5.42E-03 | -8.75E-03 | -8.71E-03 | -5.30E-03 | -1.04E-02 |
| $K09117_{VIP≥0.8\ (-)}$ | 1.563 | 0.627 | 0.941 | 0.877 | 0.891 | 0.835 | -9.64E-03 | 1.44E-03 | -6.47E-03 | -2.55E-03 | -3.86E-03 | -4.43E-03 |
| $K00865_{VIP≥0.8\ (-)}$ | 1.144 | 1.377 | 1.528 | 1.622 | 0.754 | 1.045 | -7.87E-03 | 6.60E-03 | -1.17E-02 | -8.64E-03 | -3.21E-03 | -4.88E-03 |
| $K01495_{VIP≥0.8\ (-)}$ | 1.171 | 1.006 | 1.673 | 1.657 | 0.558 | 1.217 | -2.92E-03 | 2.36E-04 | -8.01E-03 | -8.54E-03 | -2.44E-03 | -4.41E-03 |
| $K07076_{VIP≥0.8\ (-)}$ | 0.961 | 0.874 | 1.612 | 1.781 | 0.633 | 0.987 | -8.38E-04 | 3.56E-03 | -9.94E-03 | -8.82E-03 | -3.85E-03 | -6.25E-03 |
| $K01005_{VIP≥0.8\ (-)}$ | 1.03 | 0.595 | 1.03 | 1.147 | 1.003 | 0.859 | -1.50E-04 | 1.13E-03 | -6.08E-04 | -1.95E-03 | -8.62E-05 | -7.05E-05 |
| $K00287_{VIP≥0.8\ (-)}$ | 1.168 | 0.767 | 0.937 | 2.137 | 1.251 | 1.117 | 4.63E-03 | -3.52E-04 | -3.39E-03 | -1.23E-02 | -9.69E-03 | -4.52E-03 |
| $K01258_{VIP≥0.8\ (-)}$ | 1.668 | 0.253 | 0.865 | 1.295 | 1.193 | 1.104 | -9.01E-03 | 8.68E-04 | -3.04E-03 | -5.29E-03 | -1.63E-03 | -3.12E-03 |
| $K02916_{VIP≥0.8\ (-)}$ | 1.155 | 0.251 | 1.491 | 1.99 | 1.227 | 0.819 | 4.99E-03 | -1.23E-03 | -1.01E-02 | -1.06E-02 | -9.44E-03 | -2.16E-03 |
| $K07005_{VIP≥0.8\ (-)}$ | 1.311 | 0.449 | 1.908 | 2.13 | 1.236 | 0.963 | -8.80E-03 | 2.01E-03 | -1.45E-02 | -1.23E-02 | -9.09E-03 | -6.82E-03 |
| $K03559_{VIP≥0.8\ (-)}$ | 1.169 | 1.465 | 1.111 | 0.929 | 0.196 | 0.929 | -2.04E-03 | 3.83E-03 | -9.18E-04 | -3.45E-03 | -1.53E-03 | -1.06E-03 |
| $K07473_{VIP≥0.8\ (-)}$ | 0.701 | 0.871 | 1.163 | 0.98 | 0.95 | 0.942 | 4.66E-03 | -1.34E-03 | -8.06E-03 | -3.17E-03 | -2.44E-03 | -2.35E-03 |
| $K02781_{VIP≥0.8\ (-)}$ | 1.358 | 0.541 | 1.677 | 1.76 | 1.113 | 0.232 | 7.94E-03 | -4.50E-05 | -1.26E-02 | -1.03E-02 | -8.41E-03 | -1.59E-03 |
| $K09764_{VIP≥0.8\ (-)}$ | 0.796 | 0.65 | 1.122 | 0.995 | 1.134 | 1.047 | -1.33E-03 | -2.71E-03 | -5.08E-03 | -5.13E-03 | -5.08E-03 | 7.58E-05 |

| | | | | | | | | | | | | |
|---|---|---|---|---|---|---|---|---|---|---|---|---|
| K08234$_{VIP≥0.8\ (-)}$ | 0.765 | 0.517 | 1.665 | 1.459 | 1.121 | 1.266 | 4.65E-03 | -3.86E-04 | -1.13E-02 | -7.29E-03 | -6.43E-03 | -2.43E-03 |
| K02770$_{VIP≥0.8\ (-)}$ | 1.085 | 0.053 | 0.944 | 1.005 | 0.511 | 1.274 | 5.30E-03 | -1.84E-04 | -3.33E-03 | -3.65E-05 | -2.92E-03 | -8.80E-03 |
| K06933$_{VIP≥0.8\ (-)}$ | 0.77 | 1.09 | 0.855 | 1.619 | 1.089 | 0.405 | -5.37E-04 | -3.96E-03 | -4.80E-03 | -8.78E-03 | -7.86E-03 | 2.10E-03 |
| K01759$_{VIP≥0.8\ (-)}$ | 0.414 | 0.985 | 1.392 | 1.28 | 1.426 | 0.701 | 4.02E-04 | -3.11E-03 | -1.04E-02 | -4.88E-03 | -8.59E-03 | -3.06E-03 |
| K01512$_{VIP≥0.8\ (-)}$ | 1.055 | 0.936 | 0.961 | 1.248 | 0.51 | 0.715 | 7.03E-03 | -3.62E-03 | -7.33E-03 | -7.31E-03 | -4.08E-03 | -4.71E-03 |
| K01698$_{VIP≥0.8\ (-)}$ | 0.544 | 0.808 | 1.565 | 1.034 | 0.949 | 0.661 | 1.73E-03 | -2.29E-03 | -1.18E-02 | -2.99E-03 | -5.67E-03 | -1.49E-03 |
| K19118$_{VIP≥0.8\ (-)}$ | 0.78 | 0.786 | 0.888 | 0.996 | 1.095 | 1.162 | 3.83E-03 | -1.77E-03 | -1.17E-03 | -9.07E-04 | -4.74E-04 | -4.15E-03 |
| K09680$_{VIP≥0.8\ (-)}$ | 1.308 | 0.749 | 1.106 | 0.91 | 1.593 | 0.484 | -7.31E-03 | 1.69E-03 | -3.00E-03 | -3.34E-03 | -1.12E-02 | -1.12E-03 |
| K24180$_{VIP≥0.8\ (-)}$ | 0.767 | 0.907 | 0.963 | 0.608 | 1.74 | 0.977 | -4.83E-03 | -2.09E-03 | -4.54E-03 | -3.52E-03 | -1.21E-02 | 7.01E-03 |
| K08681$_{VIP≥0.8\ (-)}$ | 0.485 | 0.531 | 1.861 | 0.852 | 0.955 | 0.838 | -3.28E-03 | 1.25E-03 | -1.42E-02 | -4.07E-03 | -5.40E-03 | -6.02E-03 |
| K00721$_{VIP≥0.8\ (-)}$ | 1.188 | 1.233 | 1.102 | 0.74 | 0.589 | 1.058 | -2.40E-03 | -2.56E-03 | -8.39E-04 | -3.06E-04 | -1.84E-05 | 2.89E-03 |
| K07444$_{VIP≥0.8\ (-)}$ | 1.24 | 1.214 | 0.828 | 0.563 | 0.912 | 0.555 | -7.57E-03 | 1.06E-04 | -1.43E-03 | -3.18E-03 | -7.13E-03 | -1.74E-03 |
| K02527$_{VIP≥0.8\ (-)}$ | 1.114 | 1.338 | 0.985 | 0.639 | 0.864 | 0.798 | -5.46E-03 | 4.01E-03 | -1.23E-03 | -2.71E-03 | -6.43E-03 | -5.64E-03 |
| K03771$_{VIP≥0.8\ (-)}$ | 1.181 | 1.189 | 1.07 | 0.98 | 0.533 | 0.452 | -3.12E-03 | -4.87E-04 | -9.43E-04 | -4.83E-03 | -3.84E-03 | 5.88E-04 |
| K01613$_{VIP≥0.8\ (-)}$ | 1.144 | 1.12 | 1.094 | 1.331 | 0.489 | 0.543 | -1.17E-03 | 1.22E-03 | -2.49E-03 | -7.54E-03 | -3.83E-03 | -2.06E-03 |
| K19005$_{VIP≥0.8\ (-)}$ | 0.817 | 1.204 | 0.909 | 0.664 | 1.962 | 0.784 | -2.00E-03 | -1.95E-04 | -1.74E-04 | -3.21E-03 | -1.31E-02 | 2.70E-03 |
| K01934$_{VIP≥0.8\ (-)}$ | 1.183 | 0.646 | 1.226 | 1.381 | 1.119 | 0.708 | -7.87E-03 | -3.16E-03 | -9.38E-03 | -6.34E-03 | -4.58E-03 | 2.98E-03 |
| K09710$_{VIP≥0.8\ (-)}$ | 0.885 | 0.906 | 1.255 | 0.57 | 0.273 | 1.188 | -3.16E-03 | -2.59E-03 | -5.86E-03 | -3.06E-03 | 1.27E-03 | -1.23E-03 |
| K02904$_{VIP≥0.8\ (-)}$ | 0.292 | 0.942 | 1.256 | 1.503 | 1.508 | 0.453 | 7.85E-04 | -2.70E-03 | -9.37E-03 | -8.79E-03 | -1.21E-02 | -3.12E-03 |
| K00950$_{VIP≥0.8\ (-)}$ | 1.19 | 1.226 | 1.628 | 1.166 | 0.798 | 0.625 | -5.53E-03 | 3.91E-03 | -9.27E-03 | -5.69E-03 | -5.81E-03 | -3.48E-03 |
| K07484$_{VIP≥0.8\ (-)}$ | 1.958 | 0.61 | 0.796 | 1.093 | 0.885 | 1.013 | -1.04E-02 | -1.51E-03 | -1.03E-03 | 3.73E-03 | -1.82E-03 | -4.81E-03 |
| K00616$_{VIP≥0.8\ (-)}$ | 1.349 | 1.19 | 0.969 | 1.205 | 1.133 | 1.007 | 9.03E-03 | -4.98E-03 | -4.21E-03 | 3.46E-03 | -3.01E-03 | -3.00E-03 |
| K22210$_{VIP≥0.8\ (-)}$ | 1.024 | 1.085 | 0.891 | 0.871 | 0.8 | 1.148 | -2.89E-03 | 2.29E-03 | -2.36E-03 | -4.83E-03 | 3.46E-03 | -6.65E-03 |
| K07402$_{VIP≥0.8\ (-)}$ | 0.915 | 0.908 | 1.099 | 1.096 | 1.012 | 1.548 | -1.16E-03 | -2.66E-03 | 2.34E-03 | -2.78E-03 | -1.90E-03 | 8.14E-03 |
| K09767$_{VIP≥0.8\ (-)}$ | 0.838 | 0.973 | 1.165 | 0.968 | 1.115 | 0.893 | 1.52E-03 | -2.33E-03 | -7.33E-03 | 3.27E-03 | -5.98E-03 | -3.17E-03 |
| K04047$_{VIP≥0.8\ (-)}$ | 1.532 | 1.127 | 1.029 | 1.084 | 1.061 | 1.153 | -9.01E-03 | -4.67E-03 | 1.64E-03 | -1.22E-04 | 9.01E-04 | -5.96E-03 |
| K02413$_{VIP≥0.8\ (-)}$ | 0.944 | 1.236 | 0.938 | 1.052 | 1.069 | 1.231 | 4.27E-03 | -3.81E-03 | -4.08E-03 | -4.29E-04 | -3.12E-03 | 1.54E-03 |
| K02398$_{VIP≥0.8\ (-)}$ | 0.911 | 1.044 | 0.908 | 1.01 | 1.062 | 1.114 | 5.04E-03 | -1.52E-03 | -4.80E-03 | -1.87E-03 | -3.09E-03 | 3.56E-04 |
| K00872$_{VIP≥0.8\ (-)}$ | 0.939 | 1.052 | 1.083 | 1.014 | 1.055 | 1.145 | -1.05E-03 | -2.37E-03 | -5.29E-03 | -1.82E-03 | 1.85E-03 | 4.36E-04 |
| K01744$_{VIP≥0.8\ (-)}$ | 1.052 | 0.878 | 0.825 | 0.912 | 0.872 | 1.421 | -7.19E-03 | -4.19E-03 | 5.14E-03 | -4.54E-03 | -1.39E-03 | 6.52E-03 |

| | | | | | | | | | | | | |
|---|---|---|---|---|---|---|---|---|---|---|---|---|
| K21903$_{VIP\geq0.8\ (-)}$ | 0.957 | 1.099 | 1.026 | 1.258 | 0.926 | 0.872 | 2.57E-03 | -3.11E-03 | -1.84E-03 | -5.22E-03 | -4.43E-03 | 1.30E-03 |
| K00567$_{VIP\geq0.8\ (-)}$ | 1.127 | 1.087 | 0.87 | 1.116 | 1.075 | 1.101 | 7.37E-03 | -4.27E-03 | -1.86E-03 | -2.57E-03 | -3.56E-03 | 3.21E-03 |
| K06167$_{VIP\geq0.8\ (-)}$ | 1.204 | 1.406 | 1.143 | 0.855 | 1.208 | 1.226 | -5.68E-03 | 3.21E-03 | -2.14E-03 | -2.81E-03 | -5.35E-03 | 5.71E-03 |
| K03621$_{VIP\geq0.8\ (-)}$ | 1.355 | 0.935 | 0.99 | 1.039 | 1.112 | 1.139 | 8.04E-03 | -5.62E-04 | -6.35E-04 | -1.53E-03 | -4.95E-03 | 3.08E-03 |
| K00566$_{VIP\geq0.8\ (-)}$ | 1.032 | 0.991 | 1.04 | 1.137 | 1.174 | 0.982 | 4.92E-03 | -3.65E-03 | 8.55E-04 | -2.50E-03 | -5.44E-03 | -5.68E-04 |
| K03500$_{VIP\geq0.8\ (-)}$ | 0.856 | 1.124 | 1.057 | 1.033 | 0.959 | 0.973 | 2.05E-03 | -2.52E-03 | -4.49E-03 | 1.90E-04 | -2.92E-03 | -1.48E-03 |
| K02426$_{VIP\geq0.8\ (-)}$ | 1.18 | 1.24 | 1.213 | 0.816 | 0.957 | 1.204 | -2.15E-03 | 3.95E-04 | -2.35E-03 | -1.92E-03 | -1.32E-03 | 2.67E-03 |
| K01775$_{VIP\geq0.8\ (-)}$ | 1.028 | 0.851 | 1.041 | 1.023 | 0.897 | 0.87 | 3.85E-03 | -4.68E-04 | -3.12E-04 | -1.54E-03 | -3.39E-03 | 2.12E-03 |
| K03787$_{VIP\geq0.8\ (-)}$ | 0.971 | 1.326 | 0.955 | 0.855 | 0.811 | 1.057 | -1.54E-03 | 1.49E-03 | -7.88E-04 | -4.41E-03 | -4.98E-03 | 4.93E-03 |
| K00931$_{VIP\geq0.8\ (-)}$ | 1.444 | 1.767 | 1.528 | 0.993 | 0.825 | 1.187 | -8.91E-03 | 8.64E-03 | -1.16E-02 | -1.71E-03 | -5.34E-03 | 6.26E-03 |
| K00817$_{VIP\geq0.8\ (-)}$ | 1.09 | 1.304 | 0.806 | 1.035 | 1.025 | 1.113 | -5.24E-03 | 6.37E-03 | -4.86E-03 | -6.07E-04 | -4.91E-03 | 2.57E-03 |
| K02029$_{VIP\geq0.8\ (-)}$ | 1.024 | 0.878 | 1.122 | 1.275 | 1.056 | 1.229 | 1.52E-03 | -1.35E-03 | -5.99E-03 | -3.82E-03 | -1.94E-03 | 5.48E-03 |
| K00432$_{VIP\geq0.8\ (-)}$ | 1.255 | 1.009 | 0.963 | 1.071 | 1.411 | 1.008 | 1.26E-03 | 6.79E-04 | -2.34E-04 | -4.34E-03 | -7.94E-03 | -2.35E-04 |
| K01153$_{VIP\geq0.8\ (-)}$ | 1.133 | 0.84 | 1.191 | 1.036 | 1.22 | 1.026 | -6.48E-03 | 3.64E-03 | 6.96E-03 | -1.39E-04 | -5.11E-03 | -3.41E-03 |
| K00986$_{VIP\geq0.8\ (-)}$ | 0.807 | 1.134 | 0.846 | 1.091 | 1.036 | 1.544 | -3.64E-03 | 5.48E-03 | -2.82E-03 | -1.74E-03 | -1.65E-03 | 8.18E-03 |
| K05566$_{VIP\geq0.8\ (-)}$ | 0.605 | 0.948 | 0.876 | 1.11 | 1.502 | 1.573 | 1.03E-03 | -4.60E-03 | -1.07E-03 | -4.17E-03 | -1.04E-02 | 1.03E-02 |
| K13599$_{VIP\geq0.8\ (-)}$ | 0.88 | 1.289 | 0.916 | 1.15 | 0.265 | 0.997 | -2.46E-03 | -6.15E-03 | 3.90E-03 | -5.94E-03 | -1.44E-03 | 8.71E-04 |
| K02173$_{VIP\geq0.8\ (-)}$ | 1.596 | 0.975 | 0.893 | 0.854 | 0.797 | 1.353 | -8.31E-03 | 4.62E-03 | -6.53E-03 | -7.32E-05 | -5.46E-03 | 8.53E-03 |
| K01975$_{VIP\geq0.8\ (-)}$ | 0.976 | 0.49 | 1.076 | 1.276 | 1.214 | 0.905 | 3.70E-03 | -2.31E-03 | -5.48E-03 | -7.14E-03 | -8.53E-03 | 1.53E-03 |
| K02420$_{VIP\geq0.8\ (-)}$ | 0.969 | 1.175 | 0.778 | 0.97 | 1.006 | 1.285 | 5.85E-03 | -3.18E-03 | -3.37E-03 | -2.45E-03 | -1.92E-03 | 2.88E-03 |
| K05794$_{VIP\geq0.8\ (-)}$ | 0.524 | 1.377 | 1.396 | 0.845 | 1.407 | 0.965 | 4.37E-04 | -6.24E-03 | -6.14E-03 | -3.67E-03 | -6.75E-03 | 1.10E-03 |
| K06972$_{VIP\geq0.8\ (-)}$ | 0.853 | 0.692 | 1.181 | 1.2 | 1.311 | 0.936 | 5.46E-04 | -1.93E-03 | 2.29E-03 | -6.24E-04 | -6.85E-03 | -1.37E-03 |
| K06864$_{VIP\geq0.8\ (-)}$ | 0.911 | 0.234 | 0.99 | 1.223 | 1.309 | 1.271 | 3.01E-03 | -4.48E-04 | -3.98E-04 | -5.61E-03 | -8.48E-03 | 8.31E-03 |
| K19689$_{VIP\geq0.8\ (-)}$ | 0.857 | 0.637 | 0.935 | 1.194 | 1.264 | 1.057 | 3.57E-03 | -8.03E-04 | -2.99E-03 | -2.78E-03 | -5.40E-03 | 2.01E-03 |
| K02203$_{VIP\geq0.8\ (-)}$ | 1.193 | 0.888 | 0.826 | 1.028 | 0.911 | 0.662 | 7.09E-03 | -2.26E-03 | -4.21E-03 | -4.25E-03 | -7.11E-03 | 4.34E-03 |
| K16511$_{VIP\geq0.8\ (-)}$ | 1.056 | 0.759 | 0.968 | 1.117 | 0.936 | 0.909 | 2.72E-03 | 6.66E-04 | -2.25E-03 | -3.74E-03 | -4.37E-03 | -5.65E-03 |
| K19119$_{VIP\geq0.8\ (-)}$ | 0.748 | 0.882 | 0.934 | 1.052 | 1.468 | 1.07 | 3.70E-03 | -2.33E-03 | -3.86E-03 | -1.30E-03 | 6.25E-03 | -2.04E-03 |
| K19159$_{VIP\geq0.8\ (-)}$ | 0.909 | 0.664 | 1.03 | 0.863 | 1.08 | 1.149 | 6.26E-03 | -2.71E-04 | -7.36E-03 | -1.92E-03 | 1.91E-03 | -6.38E-03 |
| K00991$_{VIP\geq0.8\ (-)}$ | 1.066 | 0.837 | 0.986 | 1.013 | 0.991 | 0.689 | -4.50E-03 | 2.88E-03 | -4.05E-03 | -2.56E-03 | 3.53E-03 | -1.95E-03 |
| K01139$_{VIP\geq0.8\ (-)}$ | 0.943 | 0.626 | 0.907 | 1.109 | 1.158 | 1.351 | -3.57E-03 | 1.69E-03 | -1.78E-03 | -2.59E-03 | 2.34E-03 | -8.23E-03 |

| | | | | | | | | | | | | |
|---|---|---|---|---|---|---|---|---|---|---|---|---|
| K09772$_{VIP\geq0.8\ (-)}$ | 0.886 | 0.824 | 1.086 | 1.187 | 0.913 | 0.778 | 1.72E-03 | 3.03E-05 | -5.48E-03 | -4.42E-03 | -2.87E-03 | -1.56E-03 |
| K07335$_{VIP\geq0.8\ (-)}$ | 0.875 | 0.451 | 0.907 | 1.22 | 1.212 | 0.939 | 7.01E-05 | -2.21E-04 | -2.20E-03 | -4.12E-03 | -5.91E-03 | 1.87E-03 |
| K01754$_{VIP\geq0.8\ (-)}$ | 0.912 | 1.094 | 0.886 | 0.831 | 0.671 | 1.122 | 5.18E-03 | -1.81E-03 | -1.30E-03 | 6.52E-04 | -2.03E-04 | -4.48E-03 |
| K00798$_{VIP\geq0.8\ (-)}$ | 1.052 | 1.214 | 1.064 | 0.801 | 0.643 | 1.097 | -2.32E-03 | -1.58E-03 | -5.85E-05 | -1.32E-03 | 1.97E-03 | 9.94E-04 |
| K03634$_{VIP\geq0.8\ (-)}$ | 1.01 | 0.929 | 0.888 | 1.482 | 0.935 | 0.625 | -5.95E-03 | 1.11E-04 | 3.83E-03 | -7.19E-03 | -7.42E-03 | -3.94E-04 |
| K06204$_{VIP\geq0.8\ (-)}$ | 1.139 | 0.699 | 1.256 | 1.178 | 1.563 | 1.036 | -2.79E-03 | 1.70E-03 | -3.85E-03 | -4.76E-03 | -9.16E-03 | 8.46E-04 |
| K00180$_{VIP\geq0.8\ (-)}$ | 0.942 | 0.778 | 0.972 | 0.953 | 1.377 | 1.168 | 5.28E-04 | -5.55E-04 | -3.99E-04 | -2.99E-03 | -5.86E-03 | 1.29E-03 |
| K12994$_{VIP\geq0.8\ (-)}$ | 0.973 | 1.221 | 1.08 | 0.717 | 0.8 | 0.836 | -3.72E-03 | -3.39E-04 | -3.19E-04 | -1.06E-03 | 2.80E-03 | 4.16E-03 |
| K23351$_{VIP\geq0.8\ (-)}$ | 1.494 | 0.728 | 0.927 | 1.073 | 0.925 | 0.849 | 6.96E-03 | -3.40E-03 | -2.01E-03 | -6.26E-03 | -7.38E-03 | 2.59E-03 |
| K09747$_{VIP\geq0.8\ (-)}$ | 1.019 | 1.239 | 1.356 | 1.379 | 0.964 | 0.268 | 6.41E-03 | -3.66E-03 | -9.63E-03 | -6.43E-03 | -6.34E-03 | 1.68E-03 |
| K17103$_{VIP\geq0.8\ (-)}$ | 1.146 | 1.203 | 1.117 | 0.986 | 0.522 | 1.096 | -1.06E-03 | 2.09E-03 | -9.58E-04 | -5.11E-03 | -1.75E-03 | 4.20E-03 |
| K00979$_{VIP\geq0.8\ (-)}$ | 1.003 | 1.203 | 0.742 | 0.99 | 1.502 | 1.035 | -3.16E-04 | 4.72E-03 | 5.93E-04 | -5.78E-03 | -1.20E-02 | -7.23E-03 |
| K03581$_{VIP\geq0.8\ (-)}$ | 1.095 | 0.739 | 0.996 | 1.152 | 1.129 | 0.939 | -3.40E-03 | 3.73E-04 | 1.19E-03 | -3.95E-04 | -1.11E-03 | -1.56E-03 |
| K03811$_{VIP\geq0.8\ (-)}$ | 1.085 | 1.122 | 0.994 | 0.714 | 0.926 | 1.191 | -1.85E-03 | -8.04E-05 | 2.81E-03 | -2.25E-03 | -2.66E-03 | 2.13E-04 |
| K00748$_{VIP\geq0.8\ (-)}$ | 1.149 | 1.208 | 0.991 | 0.996 | 1.261 | 0.145 | -3.94E-03 | 2.70E-03 | -2.17E-03 | -5.83E-03 | -1.01E-02 | 1.21E-04 |
| K23775$_{VIP\geq0.8\ (-)}$ | 0.827 | 0.492 | 0.829 | 0.951 | 0.805 | 1.206 | 4.18E-03 | -1.13E-03 | -3.28E-03 | -5.35E-03 | -5.08E-03 | 3.56E-03 |
| K00765$_{VIP\geq0.8\ (-)}$ | 0.95 | 1.192 | 0.783 | 1.223 | 0.886 | 1.789 | -6.55E-03 | 5.50E-03 | -5.84E-03 | -6.00E-03 | -6.15E-03 | 1.25E-02 |
| K01809$_{VIP\geq0.8\ (-)}$ | 1.021 | 1.158 | 0.896 | 0.876 | 0.285 | 1.105 | -5.35E-03 | -2.79E-04 | 2.63E-03 | -4.70E-03 | -8.13E-04 | 6.94E-03 |
| K03596$_{VIP\geq0.8\ (-)}$ | 0.624 | 0.925 | 0.875 | 1.051 | 1.047 | 1.177 | -3.20E-03 | 4.51E-03 | 2.25E-03 | -2.78E-03 | -1.51E-03 | -1.89E-03 |
| K11754$_{VIP\geq0.8\ (-)}$ | 0.417 | 0.913 | 1.023 | 1.225 | 1.193 | 0.908 | -9.91E-04 | 3.32E-03 | -7.83E-03 | -5.17E-03 | 1.81E-03 | -5.03E-03 |
| K06207$_{VIP\geq0.8\ (-)}$ | 0.846 | 0.518 | 0.874 | 1.052 | 0.935 | 1.081 | -4.42E-03 | 2.24E-03 | 1.59E-03 | -1.97E-03 | -5.99E-04 | -2.94E-03 |
| K02115$_{VIP\geq0.8\ (-)}$ | 0.23 | 0.835 | 0.925 | 1.335 | 1.072 | 0.961 | 5.09E-04 | 4.06E-03 | -5.30E-03 | -6.25E-03 | -5.33E-03 | -3.00E-03 |
| K19157$_{VIP\geq0.8\ (-)}$ | 1.137 | 1.014 | 0.942 | 1.086 | 0.474 | 0.86 | 4.24E-03 | 4.81E-03 | -5.56E-03 | -6.30E-03 | -2.07E-03 | -6.16E-03 |
| K02965$_{VIP\geq0.8\ (-)}$ | 1.076 | 0.435 | 1.533 | 0.97 | 1.316 | 1.011 | 3.27E-03 | -1.19E-03 | -8.24E-03 | -4.86E-03 | -9.72E-03 | 4.83E-03 |
| K21023$_{VIP\geq0.8\ (-)}$ | 0.809 | 0.687 | 1.262 | 1.218 | 1.242 | 1.069 | 2.82E-03 | 8.99E-04 | -8.20E-03 | -3.88E-03 | -7.77E-03 | -2.51E-04 |
| K03630$_{VIP\geq0.8\ (-)}$ | 1.027 | 1.228 | 0.455 | 0.98 | 1.137 | 1.045 | -7.08E-03 | 2.11E-03 | -1.66E-03 | -1.05E-04 | -8.71E-03 | 7.47E-03 |
| K03427$_{VIP\geq0.8\ (-)}$ | 0.815 | 0.505 | 1.405 | 0.993 | 1.127 | 1.258 | -4.42E-03 | 1.12E-03 | 8.66E-03 | -5.56E-04 | -4.99E-03 | -7.38E-03 |
| K03327$_{VIP\geq0.8\ (-)}$ | 0.867 | 0.591 | 0.949 | 1.087 | 1.072 | 1.231 | -1.37E-03 | 2.87E-03 | -3.42E-03 | -2.81E-04 | -1.90E-03 | 5.16E-03 |
| K01118$_{VIP\geq0.8\ (-)}$ | 1.066 | 0.148 | 0.977 | 0.508 | 1.143 | 1.046 | 4.11E-03 | -5.48E-04 | -4.65E-03 | 1.18E-03 | -5.81E-03 | -2.47E-03 |
| K11621$_{VIP\geq0.8\ (-)}$ | 0.912 | 0.731 | 1.261 | 0.648 | 0.859 | 1.098 | 5.46E-03 | -3.00E-03 | -9.48E-03 | -3.48E-03 | -5.65E-03 | 7.87E-03 |

| | | | | | | | | | | | | |
|---|---|---|---|---|---|---|---|---|---|---|---|---|
| K01679$_{VIP\geq0.8\ (-)}$ | 1.065 | 0.419 | 0.74 | 0.911 | 0.804 | 2.129 | -4.53E-03 | 2.05E-03 | -2.10E-03 | -3.55E-03 | -2.48E-03 | 1.48E-02 |
| K19048$_{VIP\geq0.8\ (-)}$ | 1.537 | 0.678 | 0.843 | 1.011 | 1.327 | 0.772 | -6.04E-03 | 2.43E-03 | -4.92E-03 | -3.22E-03 | -8.88E-03 | 1.20E-03 |
| K03785$_{VIP\geq0.8\ (-)}$ | 1.24 | 0.748 | 0.931 | 0.823 | 0.648 | 0.877 | 8.10E-03 | 4.72E-04 | -5.40E-04 | -3.37E-03 | -3.56E-03 | -6.21E-03 |
| K02492$_{VIP\geq0.8\ (-)}$ | 0.421 | 0.587 | 1.112 | 0.921 | 0.994 | 0.845 | 8.27E-04 | -7.87E-06 | -7.87E-03 | -1.98E-03 | -7.34E-03 | 9.19E-04 |
| K20866$_{VIP\geq0.8\ (-)}$ | 0.886 | 0.757 | 0.867 | 0.73 | 1.494 | 0.926 | -5.18E-03 | -3.01E-03 | 4.83E-04 | -1.68E-03 | 6.42E-03 | -8.42E-04 |
| K19117$_{VIP\geq0.8\ (-)}$ | 0.787 | 0.652 | 0.895 | 0.995 | 1.473 | 1.035 | 4.16E-03 | -1.95E-03 | -1.42E-03 | -1.39E-03 | 5.59E-03 | -2.29E-03 |
| K07741$_{VIP\geq0.8\ (-)}$ | 0.685 | 1.464 | 0.883 | 1.297 | 0.739 | 1.217 | -4.63E-03 | 7.06E-03 | -6.74E-03 | -5.93E-03 | -2.96E-03 | 6.32E-03 |
| K00788$_{VIP\geq0.8\ (-)}$ | 0.441 | 0.842 | 0.962 | 0.971 | 0.813 | 0.286 | -2.40E-03 | 7.67E-04 | -5.82E-03 | -8.96E-05 | -6.41E-03 | 1.78E-03 |
| K11145$_{VIP\geq0.8\ (-)}$ | 0.949 | 0.865 | 1.009 | 1.071 | 0.737 | 0.497 | 3.63E-03 | 7.98E-04 | -4.34E-03 | -3.06E-03 | -1.05E-03 | -1.05E-03 |
| K03789$_{VIP\geq0.8\ (-)}$ | 1.13 | 0.706 | 0.856 | 0.848 | 0.81 | 0.662 | 6.30E-03 | -5.33E-04 | -4.82E-03 | -2.85E-03 | -6.44E-03 | 4.34E-03 |
| K00179$_{VIP\geq0.8\ (-)}$ | 0.841 | 0.942 | 0.928 | 0.553 | 0.981 | 0.766 | -8.47E-04 | -2.85E-04 | 5.19E-03 | -1.12E-03 | -4.48E-03 | 1.15E-03 |
| K08591$_{VIP\geq0.8\ (-)}$ | 1.225 | 0.922 | 1.006 | 1.038 | 0.746 | 0.729 | 6.59E-03 | -1.96E-03 | -1.56E-03 | -1.73E-03 | -1.94E-03 | 2.51E-03 |
| K01524$_{VIP\geq0.8\ (-)}$ | 0.767 | 0.559 | 1.014 | 1.127 | 1.281 | 1.281 | -1.24E-03 | 6.37E-04 | -3.44E-03 | -3.15E-04 | -4.37E-03 | 6.15E-04 |
| K03642$_{VIP\geq0.8\ (-)}$ | 1.251 | 1.594 | 1.133 | 0.809 | 0.119 | 0.394 | -6.60E-03 | 6.78E-03 | -4.54E-03 | -3.61E-03 | 1.56E-04 | -2.83E-03 |
| K03060$_{VIP\geq0.8\ (-)}$ | 0.779 | 1.14 | 1.543 | 1.179 | 0.97 | 0.541 | 3.39E-03 | -2.35E-03 | -1.16E-02 | -5.41E-03 | -6.28E-03 | 1.15E-03 |
| K02346$_{VIP\geq0.8\ (-)}$ | 1.373 | 0.531 | 0.844 | 0.96 | 0.77 | 1.061 | -7.24E-03 | 1.71E-03 | -3.63E-03 | -9.73E-04 | -4.47E-03 | 2.31E-03 |
| K06076$_{VIP\geq0.8\ (-)}$ | 1.228 | 1.494 | 0.981 | 0.787 | 1.077 | 0.748 | -5.32E-03 | 3.59E-03 | 3.11E-03 | -1.10E-03 | -2.30E-03 | -3.11E-03 |
| K02897$_{VIP\geq0.8\ (-)}$ | 1.197 | 1.069 | 0.885 | 1.124 | 0.536 | 0.705 | -5.27E-03 | 1.69E-03 | 2.12E-03 | -5.38E-03 | -3.78E-03 | -8.82E-04 |
| K02067$_{VIP\geq0.8\ (-)}$ | 1.037 | 1.177 | 1.232 | 1.04 | 0.58 | 0.468 | 3.60E-04 | 4.60E-03 | -6.25E-03 | -6.05E-03 | -4.08E-03 | -4.03E-04 |
| K22522$_{VIP\geq0.8\ (-)}$ | 1.18 | 1.104 | 0.924 | 0.513 | 0.531 | 0.999 | -6.87E-03 | -1.60E-03 | -3.09E-03 | -1.27E-03 | 8.22E-04 | 3.10E-04 |
| K06901$_{VIP\geq0.8\ (-)}$ | 1.029 | 1.727 | 0.259 | 0.91 | 0.82 | 0.47 | -7.09E-03 | 6.48E-03 | 1.97E-03 | -5.20E-03 | -5.43E-03 | -3.38E-03 |
| K01933$_{VIP\geq0.8\ (-)}$ | 0.626 | 1.201 | 0.637 | 1.098 | 1.015 | 0.966 | -3.25E-03 | 5.47E-03 | -1.54E-03 | -3.44E-03 | -3.71E-03 | 4.86E-03 |
| K18831$_{VIP\geq0.8\ (-)}$ | 0.432 | 1.519 | 0.397 | 1.458 | 0.801 | 0.946 | -1.89E-04 | 7.42E-03 | -1.90E-03 | -8.53E-03 | -2.15E-03 | 5.03E-03 |
| K01808$_{VIP\geq0.8\ (-)}$ | 0.884 | 0.596 | 1.327 | 1.251 | 0.439 | 0.937 | 4.81E-03 | -1.98E-03 | -8.34E-03 | -7.31E-03 | -3.43E-03 | 5.07E-03 |
| K00677$_{VIP\geq0.8\ (-)}$ | 1.135 | 1.672 | 1.047 | 1.207 | 0.667 | 0.144 | -3.57E-03 | 5.76E-03 | -2.21E-03 | -6.25E-03 | -5.34E-03 | 9.10E-04 |
| K09748$_{VIP\geq0.8\ (-)}$ | 0.761 | 0.88 | 1.811 | 1.701 | 0.88 | 0.695 | -1.65E-04 | 3.71E-03 | -1.26E-02 | -9.95E-03 | -7.04E-03 | 4.91E-03 |
| K00266$_{VIP\geq0.8\ (-)}$ | 0.643 | 0.687 | 0.908 | 1.32 | 1.087 | 1.301 | -8.85E-04 | 3.28E-03 | -2.99E-03 | 3.50E-03 | -2.24E-03 | -6.38E-03 |
| K03602$_{VIP\geq0.8\ (-)}$ | 1.047 | 0.316 | 1.796 | 1.709 | 0.76 | 0.994 | 2.13E-03 | -5.16E-04 | -1.09E-02 | -9.92E-03 | -5.48E-03 | 4.30E-03 |
| K22278$_{VIP\geq0.8\ (-)}$ | 0.915 | 1.156 | 1.023 | 0.366 | 0.754 | 2.048 | -2.08E-03 | 2.35E-03 | -2.93E-03 | -1.39E-04 | -3.76E-03 | 1.47E-02 |
| K07481$_{VIP\geq0.8\ (-)}$ | 0.731 | 0.938 | 0.906 | 0.849 | 1.119 | 0.656 | -4.52E-03 | -2.53E-03 | -6.27E-03 | -2.16E-04 | 5.58E-03 | 1.30E-03 |

| | | | | | | | | | | | | |
|---|---|---|---|---|---|---|---|---|---|---|---|---|
| K19092$_{VIP\geq0.8\ (-)}$ | 1.564 | 0.714 | 1.446 | 1.382 | 0.883 | 0.547 | 1.08E-02 | 1.68E-03 | -1.08E-02 | -6.72E-03 | -1.70E-03 | -2.43E-03 |
| K06142$_{VIP\geq0.8\ (-)}$ | 1.158 | 1.204 | 1.199 | 0.832 | 0.373 | 0.743 | -2.53E-03 | 1.53E-04 | -2.79E-03 | -3.00E-03 | 1.71E-04 | -2.31E-03 |
| K04654$_{VIP\geq0.8}$ | 1.041 | 1.072 | 0.985 | 1.306 | 1.084 | 1.035 | 4.47E-03 | 4.31E-03 | -2.62E-03 | -5.04E-03 | -4.58E-03 | 4.82E-03 |
| K07054$_{VIP\geq0.8}$ | 0.83 | 1.014 | 1.015 | 0.826 | 1.502 | 1.199 | 7.59E-04 | -2.70E-03 | 2.82E-03 | -5.27E-04 | -6.15E-03 | 2.86E-03 |
| K04655$_{VIP\geq0.8}$ | 0.908 | 1.153 | 1.027 | 1.299 | 1.143 | 0.884 | 1.47E-03 | 4.62E-03 | -1.43E-03 | -5.08E-03 | -4.69E-03 | 1.50E-03 |
| K07588$_{VIP\geq0.8}$ | 0.962 | 1.203 | 1.044 | 0.933 | 1.469 | 0.95 | 3.58E-05 | -6.64E-05 | 4.36E-03 | -3.33E-03 | -6.46E-03 | 5.82E-04 |
| K01246$_{VIP\geq0.8}$ | 0.836 | 0.827 | 1.025 | 0.967 | 1.156 | 1.21 | -1.52E-03 | -2.55E-03 | 1.15E-04 | 1.49E-03 | -3.51E-03 | 1.88E-03 |
| K00937$_{VIP\geq0.8}$ | 0.96 | 0.859 | 1.094 | 1.222 | 1.175 | 1.328 | -2.21E-03 | -1.28E-03 | 1.13E-03 | 2.34E-03 | -1.33E-03 | 2.09E-03 |
| K01726$_{VIP\geq0.8}$ | 0.887 | 1.224 | 0.847 | 0.851 | 1.874 | 1.141 | 6.70E-04 | 2.49E-04 | 2.29E-03 | -2.95E-03 | -1.03E-02 | -1.68E-04 |
| K09922$_{VIP\geq0.8}$ | 1.119 | 0.988 | 0.975 | 0.986 | 0.936 | 1.013 | -1.45E-03 | 1.88E-04 | 1.17E-03 | -3.05E-03 | -3.89E-03 | 3.62E-03 |
| K18843$_{VIP\geq0.8}$ | 1.009 | 1.048 | 1.035 | 1.094 | 1.103 | 1.075 | 5.22E-03 | 3.05E-03 | -3.34E-03 | -2.47E-03 | 8.67E-05 | -4.59E-03 |
| K03286$_{VIP\geq0.8}$ | 1.409 | 1.032 | 0.921 | 1.248 | 1.167 | 1.06 | 4.72E-03 | -2.51E-03 | 9.13E-04 | -5.81E-03 | -5.35E-03 | 2.51E-03 |
| K03574$_{VIP\geq0.8}$ | 0.954 | 1.248 | 0.886 | 1.838 | 1.19 | 0.901 | -8.93E-04 | 3.21E-03 | 2.36E-03 | -1.05E-02 | -8.80E-03 | 8.94E-04 |
| K01154$_{VIP\geq0.8}$ | 1.065 | 0.905 | 1.292 | 1.086 | 1.223 | 1.014 | -5.83E-03 | 4.17E-03 | 7.13E-03 | 3.00E-04 | -6.71E-03 | -6.21E-03 |
| K02626$_{VIP\geq0.8}$ | 0.911 | 0.81 | 0.823 | 0.582 | 1.584 | 1.188 | 5.18E-03 | -2.38E-03 | 5.47E-03 | -1.27E-03 | -9.59E-03 | 5.13E-03 |
| K02466$_{VIP\geq0.8}$ | 1.711 | 0.863 | 1.744 | 1.6 | 0.778 | 0.903 | 1.00E-02 | -3.78E-03 | -1.24E-02 | -7.88E-03 | 4.58E-03 | 1.01E-03 |
| K23356$_{VIP\geq0.8}$ | 1.308 | 0.557 | 0.998 | 1.187 | 1.135 | 0.978 | -6.34E-03 | -2.61E-03 | 5.12E-03 | 6.42E-03 | -8.99E-03 | 6.90E-03 |
| K01500$_{VIP\geq0.8}$ | 1.066 | 0.906 | 0.885 | 1.735 | 1.502 | 0.658 | 5.22E-04 | 3.77E-03 | -4.08E-03 | -9.84E-03 | -1.20E-02 | 1.50E-03 |
| K06198$_{VIP\geq0.8}$ | 1 | 0.846 | 1.698 | 0.691 | 0.813 | 1.667 | 6.22E-03 | -3.16E-03 | 1.30E-02 | -3.69E-03 | -5.81E-03 | 1.03E-02 |
| K13018$_{VIP\geq0.8}$ | 1.04 | 0.958 | 0.954 | 1.074 | 0.72 | 1.177 | 1.77E-03 | -3.51E-03 | 7.91E-04 | -4.28E-03 | -2.66E-03 | 3.84E-03 |
| K23010$_{VIP\geq0.8}$ | 0.875 | 0.899 | 0.991 | 0.496 | 2.441 | 0.993 | -5.63E-03 | 1.08E-03 | 5.60E-03 | 1.98E-03 | -1.54E-02 | -2.68E-03 |
| K07080$_{VIP\geq0.8}$ | 1.146 | 0.544 | 0.859 | 1.11 | 1.069 | 1.842 | 7.09E-03 | -6.73E-04 | 3.61E-04 | -2.26E-03 | -2.84E-03 | 1.23E-02 |
| K23876$_{VIP\geq0.8}$ | 0.727 | 0.861 | 0.91 | 0.959 | 1.127 | 0.822 | 1.57E-04 | -3.05E-03 | -1.28E-03 | -2.99E-03 | 3.78E-03 | 3.70E-03 |
| K04094$_{VIP\geq0.8}$ | 1.023 | 0.677 | 0.858 | 1.015 | 0.88 | 0.896 | 6.94E-03 | -9.68E-04 | -1.72E-03 | -1.10E-03 | 3.27E-03 | 2.57E-03 |
| K10117$_{VIP\geq0.8}$ | 1.225 | 0.451 | 1.005 | 1.133 | 1.278 | 1.179 | -3.42E-03 | 3.24E-05 | -1.10E-03 | -1.80E-03 | 6.87E-03 | 5.69E-03 |
| K06898$_{VIP\geq0.8}$ | 1.055 | 0.379 | 0.915 | 1.212 | 1.417 | 1.238 | 4.75E-03 | 1.64E-03 | -7.61E-04 | -5.23E-03 | -9.75E-03 | 8.39E-03 |
| K16787$_{VIP\geq0.8}$ | 0.913 | 0.54 | 1.06 | 1.228 | 1.047 | 0.983 | 1.03E-03 | 3.76E-04 | -7.75E-04 | -4.04E-03 | -3.55E-03 | 2.97E-03 |
| K07404$_{VIP\geq0.8}$ | 0.917 | 0.446 | 0.946 | 1.019 | 1.039 | 1.3 | -4.88E-04 | 1.82E-03 | -2.20E-03 | -3.83E-03 | 3.17E-03 | 5.89E-03 |
| K04653$_{VIP\geq0.8}$ | 1.238 | 0.805 | 1.254 | 1.476 | 1.386 | 0.719 | 7.81E-03 | 2.22E-03 | -8.70E-03 | -7.26E-03 | -9.96E-03 | 4.40E-03 |
| K06934$_{VIP\geq0.8}$ | 0.678 | 0.946 | 0.848 | 0.927 | 1.656 | 1.093 | 1.05E-04 | -1.88E-03 | 3.96E-03 | -5.01E-03 | -1.14E-02 | 3.95E-03 |

| | | | | | | | | | | | | |
|---|---|---|---|---|---|---|---|---|---|---|---|---|
| $K06940_{VIP≥0.8}$ | 0.988 | 0.69 | 1.175 | 1.442 | 1.077 | 0.84 | 3.08E-03 | 1.73E-03 | -7.66E-03 | -6.89E-03 | -5.86E-03 | 1.42E-03 |
| $K03606_{VIP≥0.8}$ | 1.125 | 1.967 | 0.529 | 1.046 | 0.886 | 0.933 | -5.91E-03 | 9.62E-03 | 9.13E-04 | -5.10E-03 | -5.26E-03 | 6.39E-03 |
| $K07792_{VIP≥0.8}$ | 0.818 | 1.119 | 0.939 | 0.163 | 1.28 | 0.923 | -2.82E-03 | 1.80E-03 | 4.18E-03 | -2.56E-04 | -9.07E-03 | 6.62E-03 |
| $K19271_{VIP≥0.8}$ | 0.947 | 0.81 | 0.925 | 0.75 | 1.568 | 1.218 | 8.02E-04 | 3.89E-05 | -2.42E-04 | 2.19E-04 | -8.74E-03 | -3.58E-04 |
| $K03705_{VIP≥0.8}$ | 0.914 | 0.845 | 1.009 | 1.144 | 0.877 | 0.63 | 1.07E-03 | 1.20E-03 | -3.68E-04 | -2.19E-03 | 9.21E-04 | -1.12E-03 |
| $K03216_{VIP≥0.8}$ | 1.183 | 1.004 | 1.248 | 0.908 | 0.373 | 1.012 | 6.64E-03 | -3.13E-03 | -8.91E-03 | 1.81E-03 | -1.66E-03 | 7.21E-03 |
| $K06990_{VIP≥0.8}$ | 0.801 | 0.777 | 1.005 | 1.067 | 1.519 | 1.346 | 1.62E-03 | -1.23E-03 | 5.84E-04 | -4.27E-03 | -1.08E-02 | 5.21E-03 |
| $K16870_{VIP≥0.8}$ | 1.167 | 1.638 | 1.017 | 0.902 | 0.554 | 1.016 | -4.81E-03 | 5.27E-03 | -1.74E-03 | -3.67E-03 | 1.03E-03 | 6.69E-03 |
| $K00761_{VIP≥0.8}$ | 1.053 | 0.436 | 0.951 | 1.184 | 1.063 | 1.052 | 6.53E-03 | -8.98E-05 | -4.67E-03 | -5.18E-03 | 5.47E-03 | 7.15E-03 |
| $K07075_{VIP≥0.8}$ | 0.951 | 1.113 | 1.147 | 0.779 | 1.44 | 1.155 | -1.29E-04 | 1.40E-03 | -4.41E-03 | 1.89E-04 | -6.51E-03 | 3.09E-03 |
| $K01009_{VIP≥0.8}$ | 1.427 | 1.265 | 0.935 | 0.701 | 1.182 | 0.837 | -8.29E-03 | -1.06E-03 | 3.12E-03 | 3.06E-03 | -5.03E-03 | 3.37E-03 |
| $K01719_{VIP≥0.8}$ | 1.141 | 1.281 | 1.179 | 0.955 | 0.58 | 1.098 | -2.51E-03 | 2.38E-03 | -2.60E-03 | -3.36E-03 | 1.65E-03 | 1.64E-03 |
| $K00265_{VIP≥0.8}$ | 0.99 | 0.298 | 0.968 | 0.996 | 0.888 | 1.144 | -3.95E-03 | 1.24E-03 | 2.74E-03 | 3.39E-04 | -1.33E-03 | -2.80E-03 |
| $K15738_{VIP≥0.8}$ | 2.12 | 1.241 | 0.509 | 0.808 | 1.031 | 1.142 | -1.41E-02 | 5.69E-03 | 3.14E-04 | -1.07E-03 | 2.68E-03 | -6.46E-03 |
| $K23242_{VIP≥0.8}$ | 1.097 | 0.942 | 1.103 | 0.871 | 0.242 | 0.986 | 7.44E-03 | -4.17E-03 | -7.95E-03 | -4.91E-03 | 1.26E-03 | 4.64E-03 |
| $K03790_{VIP≥0.8}$ | 1.646 | 0.872 | 0.751 | 1.269 | 1.081 | 1.108 | 1.00E-02 | -3.84E-03 | 3.47E-04 | -7.29E-03 | -5.16E-03 | 4.10E-03 |
| $K02112_{VIP≥0.8}$ | 0.315 | 0.83 | 1.443 | 1.109 | 1.054 | 1.145 | -2.13E-04 | 4.00E-03 | 6.87E-03 | 2.37E-03 | -2.34E-03 | -1.52E-03 |
| $K06133_{VIP≥0.8}$ | 0.838 | 1.007 | 0.981 | 1.318 | 1.847 | 0.69 | 7.09E-04 | 2.85E-03 | -2.70E-03 | -7.67E-03 | -1.44E-02 | 3.71E-03 |
| $K01662_{VIP≥0.8}$ | 0.827 | 0.945 | 0.767 | 1.262 | 1.029 | 1.404 | -4.94E-03 | 4.00E-03 | -6.13E-04 | 4.00E-03 | -7.09E-03 | 6.47E-03 |
| $K01991_{VIP≥0.8}$ | 1.201 | 1.282 | 1.026 | 0.785 | 0.902 | 1.419 | -3.99E-03 | 2.51E-04 | 3.19E-03 | -2.59E-03 | -5.90E-03 | 8.91E-03 |
| $K07114_{VIP≥0.8}$ | 1.187 | 1.285 | 1.055 | 0.99 | 0.338 | 0.872 | -3.50E-03 | 2.97E-03 | 3.64E-03 | -3.61E-03 | -1.70E-03 | 2.00E-03 |
| $K03561_{VIP≥0.8}$ | 1.163 | 1.26 | 0.996 | 1.254 | 1.021 | 0.766 | -1.01E-04 | 2.80E-03 | 5.22E-03 | -7.31E-03 | -8.00E-03 | 3.85E-03 |
| $K15921_{VIP≥0.8}$ | 1.068 | 1.188 | 1.06 | 0.961 | 1.397 | 0.762 | -4.80E-03 | 5.72E-04 | 4.31E-03 | -2.95E-03 | -5.51E-03 | 4.21E-03 |
| $K08640_{VIP≥0.8}$ | 1.143 | 1.155 | 1.032 | 0.926 | 0.953 | 0.732 | -4.25E-03 | 1.01E-03 | -3.32E-03 | -3.12E-03 | 7.60E-03 | 3.15E-03 |
| $K02030_{VIP≥0.8}$ | 0.863 | 0.677 | 1.183 | 1.208 | 1.07 | 1.313 | 4.89E-04 | 1.12E-03 | -7.56E-03 | -3.03E-03 | -2.94E-03 | 6.10E-03 |
| $K07493_{VIP≥0.8}$ | 1.067 | 0.488 | 1.023 | 1.124 | 0.959 | 1.143 | -3.31E-03 | 1.39E-03 | -2.88E-03 | 4.69E-04 | 1.88E-04 | -2.57E-03 |
| $K21572_{VIP≥0.8}$ | 1.013 | 1.104 | 0.983 | 0.804 | 0.808 | 0.587 | -5.53E-03 | 3.59E-03 | -6.99E-03 | -4.61E-03 | 3.18E-03 | 6.29E-04 |
| $K03050_{VIP≥0.8}$ | 1.125 | 0.536 | 0.775 | 0.838 | 1.883 | 1.278 | 5.00E-03 | -1.11E-03 | 1.44E-03 | -1.92E-03 | -1.19E-02 | 4.80E-03 |
| $K21993_{VIP≥0.8}$ | 0.634 | 0.781 | 0.973 | 1.006 | 1.178 | 0.96 | -2.34E-05 | -3.03E-03 | 7.14E-03 | 4.75E-03 | -2.60E-04 | 5.57E-03 |
| $K02651_{VIP≥0.8}$ | 0.621 | 0.729 | 1.381 | 1.243 | 1.513 | 1.289 | 3.75E-03 | 3.53E-03 | -1.05E-02 | -6.15E-03 | -1.20E-02 | 8.88E-03 |

| | | | | | | | | | | | | |
|---|---|---|---|---|---|---|---|---|---|---|---|---|
| $K16329_{VIP≥0.8}$ | 0.854 | 0.976 | 0.777 | 0.562 | 1.182 | 0.902 | 2.23E-03 | -4.43E-03 | 2.34E-03 | -1.36E-03 | -7.78E-03 | 3.61E-03 |
| $K02279_{VIP≥0.8}$ | 0.599 | 0.508 | 0.856 | 1.092 | 1.065 | 1.65 | -1.68E-03 | 5.27E-04 | 4.24E-04 | -2.98E-03 | -2.87E-03 | 1.05E-02 |
| $K03299_{VIP≥0.8}$ | 0.822 | 0.653 | 1.476 | 1.27 | 0.019 | 0.808 | 4.42E-03 | 1.10E-03 | -1.13E-02 | -7.33E-03 | -6.26E-05 | 5.40E-03 |
| $K07035_{VIP≥0.8}$ | 0.868 | 1.103 | 0.783 | 0.813 | 0.693 | 1.006 | 4.59E-03 | -4.90E-03 | 3.63E-03 | -3.69E-03 | -2.33E-03 | 7.23E-03 |
| $K15771_{VIP≥0.8}$ | 1.314 | 0.57 | 0.729 | 1.075 | 0.84 | 0.827 | 7.35E-03 | -5.79E-04 | 3.77E-04 | -5.25E-03 | 6.67E-03 | -5.70E-03 |
| $K02283_{VIP≥0.8}$ | 1.004 | 0.779 | 1.045 | 1.166 | 0.996 | 0.41 | 4.75E-03 | 3.01E-03 | -1.02E-03 | -4.61E-03 | -2.56E-03 | 7.88E-04 |
| $K10532_{VIP≥0.8}$ | 0.664 | 0.954 | 0.442 | 1.223 | 0.826 | 1.005 | 1.51E-03 | 4.31E-03 | -2.18E-03 | -6.87E-03 | -2.11E-03 | 6.90E-03 |
| $K07736_{VIP≥0.8}$ | 1.21 | 0.803 | 0.876 | 0.902 | 0.753 | 0.601 | 6.86E-03 | -1.51E-03 | -4.50E-03 | -1.42E-03 | 2.30E-03 | 3.05E-03 |
| $K03742_{VIP≥0.8}$ | 1.918 | 0.581 | 0.522 | 0.935 | 1.142 | 1.043 | 1.30E-02 | -2.05E-03 | 2.22E-03 | -3.24E-03 | -7.43E-03 | 6.56E-04 |
| $K16786_{VIP≥0.8}$ | 1.381 | 0.643 | 1.031 | 1.168 | 1.11 | 0.696 | 9.01E-03 | -7.86E-04 | -8.06E-04 | -3.51E-03 | 7.94E-04 | 8.15E-04 |
| $K03473_{VIP≥0.8}$ | 0.789 | 0.963 | 0.881 | 0.231 | 0.833 | 0.994 | -2.88E-03 | 3.01E-03 | 5.62E-03 | 3.91E-04 | -3.84E-03 | -7.02E-03 |
| $K01235_{VIP≥0.8}$ | 0.872 | 1.101 | 0.72 | 0.311 | 1.099 | 0.823 | -5.89E-03 | 2.48E-03 | 1.61E-03 | -1.76E-03 | -8.32E-03 | 5.83E-03 |
| $K08722_{VIP≥0.8}$ | 1.201 | 0.915 | 0.99 | 1.076 | 0.655 | 0.712 | 6.69E-03 | 2.08E-03 | -5.13E-03 | -3.66E-03 | -5.31E-04 | 1.97E-03 |
| $K03321_{VIP≥0.8}$ | 0.598 | 1.187 | 0.901 | 0.609 | 1.152 | 0.825 | -1.62E-03 | -2.83E-03 | 1.89E-03 | 6.90E-04 | -2.10E-03 | 2.21E-04 |
| $K06024_{VIP≥0.8}$ | 1.101 | 0.824 | 0.978 | 1.094 | 0.722 | 0.621 | 5.95E-03 | 1.49E-03 | -3.41E-03 | -3.22E-03 | -2.51E-03 | 2.60E-03 |
| $K00980_{VIP≥0.8}$ | 1.015 | 0.269 | 0.25 | 1.591 | 1.079 | 0.9 | 4.22E-03 | -1.28E-03 | 1.34E-03 | -8.26E-03 | -7.52E-03 | 5.40E-03 |
| $K07037_{VIP≥0.8}$ | 1.063 | 1.407 | 1.014 | 0.558 | 0.737 | 1.162 | -2.99E-03 | 3.12E-03 | 2.55E-04 | -1.59E-03 | -1.81E-03 | 7.28E-03 |
| $K00683_{VIP≥0.8}$ | 1.161 | 1.375 | 1.072 | 0.56 | 0.406 | 0.835 | -1.96E-03 | -2.23E-03 | -1.39E-03 | 1.30E-03 | 2.56E-03 | 3.45E-03 |
| $K07164_{VIP≥0.8}$ | 1.176 | 1.282 | 1.088 | 0.712 | 0.456 | 1.23 | -2.87E-03 | 2.21E-03 | -1.50E-03 | -4.12E-04 | 2.29E-03 | 4.51E-03 |
| $K01817_{VIP≥0.8}$ | 0.873 | 1.223 | 0.422 | 1.344 | 0.587 | 0.929 | -5.54E-03 | 5.29E-03 | 3.18E-03 | -6.95E-03 | -9.69E-05 | 6.12E-03 |
| $K01633_{VIP≥0.8}$ | 1.067 | 1.263 | 1.373 | 0.853 | 0.177 | 0.458 | 1.76E-04 | 4.41E-03 | -5.98E-03 | -4.99E-03 | 4.88E-05 | -3.04E-03 |
| $K02557_{VIP≥0.8}$ | 0.978 | 1.751 | 1.196 | 0.25 | 0.75 | 1.028 | 4.29E-03 | 8.51E-03 | -9.14E-03 | 8.20E-04 | -3.38E-03 | -3.62E-03 |
| $K05807_{VIP≥0.8}$ | 1.183 | 1.31 | 1.002 | 1.157 | 0.141 | 0.642 | -2.64E-03 | 3.60E-03 | 1.26E-03 | -4.84E-03 | 1.05E-03 | -3.25E-03 |
| $K04773_{VIP≥0.8}$ | 1.144 | 1.494 | 0.835 | 0.959 | 0.484 | 0.131 | -4.60E-03 | 4.69E-03 | 3.05E-04 | -5.61E-03 | -2.76E-03 | 1.87E-04 |
| $K10536_{VIP≥0.8}$ | 1.087 | 1.417 | 1.172 | 0.456 | 0.256 | 0.803 | -5.13E-04 | 3.28E-03 | -2.04E-03 | 1.40E-03 | 4.64E-04 | -1.90E-03 |
| $K03521_{VIP≥0.8}$ | 0.738 | 1.435 | 1.677 | 1.025 | 0.87 | 0.567 | 4.82E-03 | 6.88E-03 | 9.81E-03 | -2.99E-03 | -7.72E-04 | -9.73E-04 |
| $K07058_{VIP≥0.8}$ | 0.564 | 0.908 | 1.341 | 1.187 | 0.575 | 0.867 | 2.79E-04 | 1.42E-03 | -5.78E-03 | -6.38E-03 | -1.91E-03 | 2.76E-03 |
| $K01206_{VIP≥0.8}$ | 1.049 | 1.606 | 0.43 | 0.718 | 0.913 | 2.033 | -7.21E-03 | 7.57E-03 | -2.63E-03 | -3.79E-03 | 4.51E-05 | 1.43E-02 |
| $K01297_{VIP≥0.8}$ | 0.653 | 1.085 | 0.957 | 0.566 | 1.636 | 1.011 | -2.39E-03 | 1.79E-03 | 2.47E-03 | -9.48E-04 | -8.54E-03 | 3.06E-03 |
| $K09014_{VIP≥0.8}$ | 0.597 | 1.129 | 0.647 | 0.925 | 0.822 | 0.924 | -4.11E-03 | 3.82E-03 | 1.81E-03 | -1.66E-03 | 7.21E-04 | -3.73E-03 |

| | | | | | | | | | | | | |
|---|---|---|---|---|---|---|---|---|---|---|---|---|
| $K03046_{VIP≥0.8}$ | 0.758 | 0.376 | 1.687 | 1.318 | 0.867 | 1.285 | -2.68E-03 | 1.79E-03 | 9.39E-03 | 3.77E-03 | -7.48E-05 | -4.94E-03 |
| $K02501_{VIP≥0.8}$ | 0.586 | 1.375 | 1.31 | 0.935 | 0.628 | 0.889 | 3.27E-03 | 6.02E-03 | -9.74E-03 | -4.94E-03 | -4.00E-03 | 6.38E-03 |
| $K04095_{VIP≥0.8}$ | 1.382 | 0.583 | 0.735 | 1.095 | 1.471 | 1.1 | 9.34E-03 | 8.80E-04 | -5.14E-03 | -5.49E-03 | 8.72E-03 | -4.37E-03 |
| $K12340_{VIP≥0.8}$ | 0.782 | 1.646 | 0.943 | 0.414 | 0.985 | 0.824 | -2.00E-03 | 5.96E-03 | 4.44E-04 | 4.21E-04 | -6.36E-04 | -3.43E-03 |
| $K03106_{VIP≥0.8}$ | 0.782 | 0.617 | 1.373 | 1.199 | 1.072 | 1.288 | -5.08E-03 | 2.01E-03 | 7.42E-03 | -4.87E-03 | -1.80E-04 | 1.99E-03 |
| $K00969_{VIP≥0.8}$ | 1.003 | 1.201 | 1.153 | 1.337 | 0.601 | 0.508 | 2.36E-03 | 5.24E-03 | -8.68E-03 | -6.97E-03 | -5.25E-04 | 3.10E-03 |
| $K03470_{VIP≥0.8}$ | 0.983 | 0.786 | 1.444 | 1.377 | 0.919 | 0.486 | 3.58E-03 | 1.47E-03 | -9.99E-03 | -7.28E-03 | -6.90E-03 | 3.49E-03 |
| $K01972_{VIP≥0.8}$ | 0.633 | 1.165 | 0.689 | 0.968 | 1.081 | 1.167 | -4.35E-03 | 4.62E-03 | 2.09E-03 | -9.46E-04 | -6.69E-03 | 2.54E-03 |
| $K08316_{VIP≥0.8}$ | 1.036 | 0.156 | 1.56 | 1.722 | 1.463 | 0.369 | 5.87E-03 | 5.30E-04 | -1.15E-02 | -9.69E-03 | -1.12E-02 | 2.44E-03 |
| $K03544_{VIP≥0.8}$ | 0.382 | 1.27 | 0.684 | 1.112 | 1.132 | 1.112 | 2.63E-03 | 5.71E-03 | -4.74E-03 | -2.05E-03 | -5.91E-03 | 4.53E-03 |
| $K02601_{VIP≥0.8}$ | 0.836 | 0.558 | 1.6 | 1.746 | 1.58 | 0.575 | 1.08E-03 | 1.16E-03 | -1.04E-02 | -1.02E-02 | -1.27E-02 | 1.49E-03 |
| $K07098_{VIP≥0.8}$ | 1.41 | 1.171 | 0.196 | 0.641 | 1.152 | 0.886 | -9.50E-03 | 4.93E-03 | 1.26E-03 | -3.75E-03 | -7.89E-03 | 4.23E-03 |
| $K07271_{VIP≥0.8}$ | 0.733 | 1.045 | 0.599 | 0.972 | 0.888 | 1.07 | 2.11E-05 | 2.56E-03 | -3.15E-03 | -5.68E-03 | -6.92E-03 | 7.55E-03 |
| $K01091_{VIP≥0.8}$ | 0.636 | 0.269 | 0.843 | 1.283 | 1.221 | 1.064 | 1.98E-03 | 1.27E-03 | -4.71E-03 | -5.64E-03 | -8.50E-03 | 2.94E-03 |
| $K04763_{VIP≥0.8}$ | 0.626 | 1.21 | 0.786 | 1.379 | 1.116 | 1.065 | 3.12E-04 | 5.92E-03 | 2.39E-03 | -6.54E-03 | -4.53E-03 | -2.92E-03 |
| K14096 | 0.974 | 0.779 | 0.688 | 0.627 | 1.716 | 1.24 | 5.47E-03 | -2.04E-03 | 3.09E-03 | 2.90E-04 | -1.01E-02 | 6.45E-03 |
| K23264 | 2.21 | 0.632 | 0.606 | 0.449 | 1.948 | 1.337 | 1.41E-02 | -1.65E-04 | -5.72E-04 | 1.91E-03 | -1.41E-02 | 8.02E-03 |
| K15024 | 0.578 | 0.885 | 0.691 | 0.958 | 0.418 | 0.86 | -2.89E-03 | -4.23E-03 | -4.42E-03 | -5.56E-03 | -2.95E-03 | -4.91E-03 |
| K07387 | 1.93 | 0.755 | 1.041 | 0.601 | 0.629 | 1.343 | 1.07E-02 | -1.84E-03 | 7.72E-03 | -3.42E-03 | 4.51E-03 | 6.38E-03 |
| K02009 | 1.248 | 0.58 | 0.65 | 0.749 | 1.502 | 1.302 | 6.93E-03 | 1.77E-04 | 2.49E-03 | -7.30E-05 | -8.79E-03 | 7.22E-03 |
| K03057 | 1.068 | 0.616 | 0.638 | 0.686 | 1.776 | 1.208 | 5.10E-03 | 1.10E-03 | 3.90E-03 | -5.43E-07 | -1.05E-02 | 4.89E-03 |
| K01780 | 2.042 | 0.304 | 0.837 | 0.515 | 0.206 | 2.017 | 1.17E-02 | -2.20E-04 | 3.81E-03 | -9.46E-04 | -1.05E-04 | 1.45E-02 |
| K13653 | 0.948 | 0.288 | 0.91 | 0.647 | 1.085 | 0.639 | 3.25E-04 | 6.86E-04 | -3.39E-03 | -1.67E-03 | -1.18E-03 | 2.90E-03 |
| K02019 | 1.096 | 0.184 | 0.487 | 0.414 | 1.074 | 0.955 | 6.22E-03 | 2.64E-04 | -2.03E-03 | 1.66E-03 | -7.50E-03 | 3.26E-03 |
| K01304 | 1.187 | 1.194 | 0.859 | 0.755 | 0.531 | 0.662 | -4.31E-03 | -4.06E-03 | -6.53E-03 | -4.17E-03 | 5.70E-06 | -4.73E-03 |
| K02499 | 1.791 | 0.174 | 0.455 | 0.826 | 1.107 | 0.311 | 1.18E-02 | 1.62E-04 | 3.13E-03 | 4.83E-03 | 8.63E-03 | 6.26E-04 |
| K06295 | 2.139 | 0.681 | 0.493 | 0.86 | 0.919 | 0.291 | -1.42E-02 | 2.75E-03 | -2.13E-03 | -1.07E-03 | 7.32E-03 | 6.07E-04 |
| K16927 | 0.453 | 1.151 | 0.96 | 1.071 | 0.714 | 0.498 | 2.28E-03 | -4.90E-03 | -6.14E-03 | -3.09E-03 | -3.47E-03 | -3.37E-03 |
| K06406 | 1.209 | 0.439 | 1.019 | 1.066 | 0.233 | 0.351 | -7.81E-03 | 1.57E-03 | -7.78E-03 | 3.55E-03 | 1.87E-03 | -1.68E-03 |
| K03750 | 1.211 | 0.629 | 0.638 | 0.47 | 1.9 | 1.448 | 6.96E-03 | 1.08E-03 | 2.31E-03 | 2.70E-03 | -1.37E-02 | 9.92E-03 |
| K01095 | 0.061 | 0.938 | 1.028 | 0.61 | 1.084 | 0.138 | 1.08E-04 | 7.60E-04 | -7.80E-03 | -3.58E-04 | -7.17E-03 | 8.53E-04 |
| K23997 | 0.779 | 0.791 | 0.953 | 0.856 | 0.757 | 1.268 | -7.71E-05 | 3.67E-03 | 3.18E-03 | -1.17E-03 | -2.43E-03 | 9.01E-03 |

| | | | | | | | | | | | | |
|---|---|---|---|---|---|---|---|---|---|---|---|---|
| K04034 | 0.92 | 0.745 | 0.859 | 0.503 | 2.129 | 0.286 | -3.30E-03 | -1.91E-03 | 3.61E-03 | -2.94E-03 | -1.70E-02 | 1.91E-03 |
| K06985 | 0.844 | 0.68 | 0.576 | 1.156 | 0.487 | 1.753 | -5.09E-03 | 2.35E-03 | -1.23E-03 | -6.62E-03 | -3.82E-03 | 1.24E-02 |
| K01749 | 0.621 | 0.583 | 0.944 | 0.867 | 0.292 | 1.661 | 2.95E-03 | 2.24E-03 | -5.07E-03 | -2.93E-03 | 9.45E-04 | 1.14E-02 |
| K04088 | 0.566 | 1.001 | 0.901 | 0.415 | 0.843 | 0.638 | 2.66E-03 | -2.61E-03 | -6.68E-03 | -1.40E-04 | -5.53E-03 | 1.45E-03 |
| K01666 | 0.084 | 0.845 | 1.445 | 1.244 | 0.591 | 0.45 | -5.77E-04 | 3.95E-03 | 1.09E-02 | 6.37E-03 | -7.08E-04 | 3.18E-03 |
| K08972 | 1.101 | 0.115 | 0.437 | 0.174 | 1.29 | 0.887 | 7.54E-03 | -5.56E-04 | 4.24E-04 | -1.02E-03 | -1.03E-02 | 6.33E-03 |
| K21556 | 1.229 | 0.613 | 0.782 | 0.771 | 1.901 | 0.883 | 5.68E-03 | -1.37E-03 | 1.20E-03 | -1.25E-03 | -9.68E-03 | -6.42E-04 |
| K01085 | 0.68 | 1.194 | 0.737 | 1.274 | 0.411 | 1.181 | -2.29E-03 | -5.71E-03 | 9.26E-06 | -6.02E-03 | 3.28E-03 | 7.37E-03 |
| K23004 | 1.126 | 0.722 | 1.019 | 1.082 | 0.739 | 0.796 | 4.79E-03 | -1.92E-03 | 3.02E-04 | -4.59E-03 | -2.70E-03 | 4.29E-03 |
| K18122 | 2.072 | 0.367 | 0.497 | 0.952 | 0.953 | 0.413 | 1.31E-02 | -1.78E-03 | 2.33E-03 | -3.84E-03 | -3.77E-03 | -2.97E-03 |
| K05305 | 0.7 | 1.319 | 0.625 | 0.587 | 1.232 | 1.129 | 3.03E-03 | 5.52E-03 | 1.87E-03 | -1.87E-03 | 8.72E-03 | 6.74E-03 |
| K00756 | 0.932 | 2.061 | 0.775 | 0.687 | 0.974 | 0.502 | 6.29E-03 | -8.88E-03 | 2.22E-03 | -3.81E-03 | -5.89E-03 | 5.21E-04 |
| K01486 | 0.142 | 0.817 | 0.983 | 0.756 | 1.2 | 0.599 | -3.61E-05 | -3.49E-03 | -6.35E-03 | -3.98E-03 | -8.86E-03 | -2.95E-03 |
| K09769 | 1.212 | 0.774 | 0.847 | 0.62 | 0.524 | 1.002 | 7.77E-03 | 2.96E-03 | -6.43E-03 | -9.24E-05 | 3.88E-03 | 6.35E-03 |
| K21744 | 0.825 | 0.254 | 0.898 | 0.556 | 1.11 | 0.785 | 6.31E-04 | -2.12E-04 | -6.50E-03 | 2.36E-03 | -8.50E-03 | -5.25E-03 |
| K07192 | 0.675 | 1.256 | 1.049 | 0.546 | 0.428 | 0.859 | 3.45E-03 | 5.77E-03 | -5.34E-03 | -3.68E-04 | 3.33E-03 | -6.13E-04 |
| K09803 | 1.756 | 1.027 | 0.28 | 0.876 | 0.757 | 0.344 | 1.20E-02 | 4.36E-03 | 2.44E-04 | 4.66E-03 | -4.93E-03 | 1.74E-03 |
| K02007 | 0.958 | 0.513 | 0.873 | 0.773 | 1.209 | 0.163 | 3.03E-04 | -2.37E-03 | -8.43E-04 | -6.40E-04 | -8.96E-03 | -1.12E-03 |
| K00878 | 0.615 | 0.509 | 1.216 | 1.24 | 1.015 | 0.523 | 1.02E-03 | -3.58E-04 | 4.83E-03 | 2.63E-03 | -4.84E-03 | -3.70E-03 |
| K09707 | 0.426 | 1.228 | 1.042 | 0.184 | 0.529 | 0.841 | 2.00E-03 | -5.13E-03 | -4.12E-03 | 1.01E-03 | -4.23E-03 | 2.44E-03 |
| K16698 | 1.546 | 1.208 | 0.574 | 0.912 | 0.545 | 0.674 | -1.06E-02 | 5.08E-03 | -8.30E-04 | -5.30E-03 | -3.39E-03 | -4.01E-03 |
| K03775 | 0.815 | 1.686 | 0.971 | 0.471 | 0.102 | 0.667 | -3.60E-03 | -8.25E-03 | -2.92E-03 | -3.22E-04 | 6.76E-04 | 4.07E-03 |
| K04062 | 0.881 | 1.398 | 0.802 | 0.136 | 0.783 | 0.717 | 5.39E-03 | 5.91E-03 | -4.54E-03 | 2.62E-04 | 9.53E-04 | -4.53E-03 |
| K19304 | 0.304 | 1.282 | 0.348 | 0.895 | 1.798 | 0.568 | -2.05E-03 | -5.38E-03 | 8.42E-04 | -5.11E-03 | -1.43E-02 | -9.04E-04 |
| K02456 | 0.718 | 0.753 | 1.077 | 0.856 | 1.067 | 0.779 | 4.33E-03 | -7.30E-04 | -8.00E-03 | 1.56E-03 | -4.38E-03 | 6.99E-04 |
| K08678 | 0.398 | 0.767 | 0.949 | 0.606 | 1.088 | 1.108 | -3.06E-04 | -2.37E-03 | 6.21E-03 | -3.70E-04 | -1.80E-03 | 6.97E-03 |
| K02435 | 0.463 | 0.87 | 1.097 | 0.801 | 0.285 | 0.625 | -2.92E-03 | -2.05E-03 | -8.28E-03 | -3.72E-03 | -2.19E-03 | 4.48E-03 |
| K15772 | 1.162 | 0.707 | 0.87 | 0.983 | 0.305 | 0.618 | 7.02E-03 | 9.40E-04 | -2.90E-03 | -3.47E-03 | -2.39E-03 | -4.07E-03 |
| K01267 | 0.612 | 0.683 | 1.257 | 1.363 | 0.937 | 0.691 | 5.48E-04 | -5.38E-04 | -9.15E-03 | -6.36E-03 | -3.28E-03 | -4.35E-03 |
| K16053 | 1.331 | 1.17 | 0.313 | 1.34 | 0.701 | 0.486 | -9.12E-03 | 2.43E-03 | 1.34E-03 | 7.80E-03 | 5.13E-03 | -1.45E-04 |
| K12976 | 0.613 | 0.503 | 1.105 | 1.143 | 0.668 | 1.017 | -4.02E-03 | 2.29E-03 | -8.45E-03 | -6.68E-03 | 2.02E-03 | -7.25E-03 |
| K07023 | 0.953 | 0.76 | 1.12 | 1.142 | 0.501 | 0.477 | 4.97E-03 | 1.94E-03 | -8.01E-03 | -5.83E-03 | -4.02E-04 | 3.09E-03 |
| K01155 | 1.038 | 1.152 | 0.405 | 0.42 | 1.537 | 0.04 | -7.08E-03 | 2.48E-03 | 2.78E-03 | -1.46E-03 | -1.20E-02 | 8.55E-05 |
| K01609 | 0.772 | 0.943 | 0.814 | 0.971 | 0.645 | 0.391 | 4.91E-04 | 3.71E-03 | -4.14E-03 | -1.53E-03 | -5.16E-03 | 2.75E-03 |

| | | | | | | | | | | | | |
|---|---|---|---|---|---|---|---|---|---|---|---|---|
| K11941 | 1.888 | 0.992 | 0.359 | 0.34 | 0.7 | 1.144 | 1.27E-02 | -3.19E-03 | 2.48E-03 | -1.95E-03 | 5.55E-03 | 6.99E-03 |
| K01845 | 0.535 | 0.485 | 0.96 | 0.925 | 1.052 | 0.799 | 2.02E-03 | 4.95E-04 | -6.28E-03 | -1.53E-03 | -6.74E-03 | 9.83E-04 |
| K07099 | 1.052 | 0.645 | 0.865 | 0.993 | 0.699 | 0.728 | 4.19E-03 | -1.23E-03 | -1.19E-03 | -1.78E-03 | -5.12E-03 | 3.50E-03 |
| K00974 | 0.538 | 1.01 | 0.914 | 0.753 | 0.68 | 1.032 | -9.47E-05 | -2.54E-03 | -9.36E-04 | 7.85E-04 | 2.71E-03 | -3.87E-03 |
| K07461 | 1.575 | 0.382 | 0.194 | 0.953 | 0.408 | 1.12 | 1.07E-02 | 8.38E-04 | -1.46E-03 | -5.04E-03 | -3.20E-03 | 6.91E-03 |
| K06023 | 1.072 | 0.712 | 0.914 | 1.229 | 0.665 | 0.263 | 3.74E-03 | 6.60E-04 | -2.81E-03 | -5.30E-03 | -2.42E-03 | 1.30E-03 |
| K11176 | 1.029 | 0.435 | 0.874 | 0.959 | 0.764 | 0.199 | 3.97E-03 | -2.84E-04 | -5.42E-04 | -1.18E-03 | -5.96E-03 | -8.55E-04 |
| K05521 | 0.407 | 0.629 | 1.356 | 0.748 | 0.917 | 2.057 | -2.27E-03 | 2.81E-03 | 7.88E-03 | -5.01E-04 | -5.32E-03 | 1.37E-02 |
| K01966 | 1.06 | 0.608 | 0.502 | 0.394 | 1.096 | 1.067 | -5.23E-03 | -5.33E-04 | 3.78E-03 | 2.04E-03 | 6.78E-03 | 6.62E-03 |
| K01858 | 0.831 | 1.977 | 0.464 | 0.719 | 0.558 | 1.715 | 1.60E-03 | -9.67E-03 | 2.75E-03 | -2.28E-03 | -4.44E-03 | 1.20E-02 |
| K06442 | 1.101 | 0.728 | 1.033 | 1.035 | 0.708 | 0.496 | 5.77E-03 | 1.98E-04 | 7.03E-05 | -1.42E-03 | -8.03E-04 | 2.19E-03 |
| K01926 | 1.489 | 0.839 | 0.726 | 1.034 | 0.58 | 0.294 | 1.01E-02 | 1.93E-03 | -9.22E-04 | -3.31E-03 | -8.27E-04 | -4.54E-04 |
| K02805 | 0.565 | 0.311 | 0.502 | 1.034 | 0.947 | 0.885 | -8.50E-04 | 1.48E-03 | -3.84E-03 | -4.48E-03 | 1.06E-03 | 1.52E-03 |
| K18828 | 0.453 | 0.347 | 0.755 | 1.505 | 1.191 | 1.168 | -3.12E-03 | -1.62E-03 | -4.29E-03 | -8.71E-03 | -9.54E-03 | -6.85E-03 |
| K21029 | 1.281 | 0.463 | 1.514 | 0.053 | 0.548 | 1.043 | -8.40E-03 | -1.43E-03 | -9.80E-03 | -2.22E-04 | 1.68E-03 | -7.44E-03 |
| K01119 | 1.889 | 1.202 | 0.674 | 0.691 | 0.889 | 0.775 | -1.29E-02 | 4.47E-03 | -4.94E-03 | -3.76E-03 | -2.41E-03 | 3.09E-03 |
| K06213 | 1.086 | 0.774 | 0.915 | 0.941 | 0.739 | 0.64 | -1.12E-03 | 7.71E-05 | -1.91E-03 | -1.53E-03 | 1.87E-03 | -2.33E-03 |
| K02315 | 0.878 | 1.333 | 0.759 | 0.876 | 0.486 | 0.538 | -1.93E-03 | 6.29E-03 | -2.96E-03 | -3.16E-04 | 2.27E-03 | 1.97E-03 |
| K09787 | 0.962 | 0.799 | 1.195 | 1.049 | 0.455 | 0.203 | 5.16E-03 | -3.41E-04 | -8.55E-03 | -4.57E-03 | -3.16E-03 | 9.51E-04 |
| K14136 | 0.494 | 0.444 | 0.538 | 1.012 | 0.835 | 0.931 | -3.33E-03 | 1.49E-03 | -3.79E-03 | -5.25E-03 | -4.88E-03 | -4.23E-03 |
| K00351 | 0.746 | 1.264 | 0.766 | 1.057 | 0.675 | 1.159 | -3.70E-03 | -1.67E-03 | 5.85E-03 | 5.86E-03 | -1.72E-03 | 6.16E-03 |
| K18700 | 0.75 | 0.505 | 0.862 | 0.62 | 0.878 | 0.899 | 2.33E-03 | 4.89E-04 | -2.21E-04 | -2.66E-03 | -3.63E-03 | 2.26E-03 |
| K13532 | 1.286 | 1.189 | 1.131 | 0.622 | 0.598 | 0.679 | -6.64E-03 | -2.10E-03 | -2.27E-03 | 2.63E-03 | 4.39E-03 | 2.15E-03 |
| K03523 | 0.949 | 0.691 | 1.017 | 1.116 | 0.717 | 0.572 | 2.65E-03 | 1.37E-03 | -5.06E-04 | -2.94E-03 | -1.73E-03 | 2.53E-05 |
| K01685 | 0.886 | 1.437 | 0.581 | 1.082 | 0.158 | 0.773 | -5.28E-03 | 4.10E-03 | 2.33E-03 | -6.09E-03 | -1.26E-03 | 5.09E-03 |
| K09015 | 1.16 | 1.528 | 0.948 | 0.396 | 0.738 | 0.189 | -6.05E-03 | 5.86E-03 | -4.59E-03 | 1.11E-03 | 5.89E-03 | 1.18E-03 |
| K07260 | 0.143 | 0.803 | 1.215 | 0.871 | 0.435 | 0.302 | 4.30E-04 | -2.36E-03 | -9.26E-03 | -4.92E-03 | -3.32E-03 | 2.08E-03 |
| K03770 | 1.058 | 1.155 | 0.937 | 0.689 | 0.272 | 0.447 | -2.11E-03 | 1.73E-03 | 7.13E-03 | 4.02E-03 | 1.12E-03 | 4.82E-04 |
| K04486 | 0.939 | 0.735 | 0.917 | 1.225 | 0.763 | 0.561 | 4.49E-03 | -6.15E-04 | -3.63E-03 | -4.47E-03 | -3.47E-03 | 3.11E-03 |
| K16785 | 0.938 | 0.518 | 1.028 | 1.089 | 0.568 | 0.573 | 7.90E-04 | -3.98E-04 | -1.13E-03 | -2.09E-03 | -4.46E-04 | -1.52E-03 |
| K03783 | 1.321 | 2.201 | 0.632 | 0.622 | 0.417 | 1.709 | 5.27E-03 | 9.52E-03 | 2.15E-03 | -3.35E-03 | 2.27E-03 | 1.15E-02 |
| K03281 | 1.095 | 1.085 | 0.493 | 0.4 | 0.484 | 0.81 | -3.68E-04 | 3.34E-03 | 3.34E-03 | -9.02E-04 | -2.01E-03 | 2.25E-03 |
| K16694 | 0.656 | 1.145 | 0.437 | 1.03 | 0.903 | 0.517 | -9.39E-04 | 1.01E-03 | 8.20E-05 | -5.27E-03 | -5.89E-03 | 2.83E-03 |
| K01338 | 0.804 | 1.538 | 1.18 | 0.555 | 0.409 | 0.525 | -4.76E-03 | 5.33E-03 | 9.02E-03 | 2.78E-03 | -2.78E-03 | 2.85E-03 |

| | | | | | | | | | | | | |
|---|---|---|---|---|---|---|---|---|---|---|---|---|
| K11720 | 1.08 | 1.364 | 0.857 | 0.671 | 0.63 | 0.262 | -3.00E-03 | 5.37E-03 | 2.45E-05 | -3.72E-03 | -5.03E-03 | -1.79E-03 |
| K02784 | 1.098 | 0.699 | 1.299 | 1.42 | 0.431 | 0.327 | 5.33E-03 | -1.26E-03 | -9.39E-03 | -7.16E-03 | -3.38E-03 | -2.06E-03 |
| K02517 | 0.656 | 0.532 | 1.272 | 0.743 | 1.13 | 1.051 | -2.59E-03 | 2.55E-03 | -9.70E-03 | -1.39E-03 | -2.41E-03 | -3.41E-03 |
| K03284 | 0.806 | 1.367 | 1.123 | 0.627 | 0.379 | 0.742 | 2.89E-03 | 4.98E-03 | 8.52E-03 | 3.27E-03 | 5.55E-04 | 5.16E-03 |
| K01610 | 0.599 | 0.264 | 0.868 | 1.099 | 0.781 | 1.302 | 3.43E-03 | 1.09E-03 | 1.39E-03 | 2.41E-03 | -4.21E-03 | -7.44E-04 |
| K01918 | 1.169 | 1.047 | 0.761 | 0.277 | 1.138 | 0.449 | 1.26E-03 | 1.33E-03 | 3.54E-03 | -9.57E-04 | -8.10E-03 | 2.35E-04 |
| K07277 | 1.007 | 0.815 | 0.613 | 0.548 | 0.739 | 1.006 | -2.44E-03 | 1.71E-03 | 4.64E-03 | -2.54E-04 | 2.06E-03 | 1.49E-03 |
| K03269 | 1.262 | 1.446 | 1.34 | 0.554 | 0.272 | 0.716 | -5.98E-03 | 4.17E-03 | -6.55E-03 | -7.00E-04 | -1.57E-03 | -2.87E-03 |
| K01218 | 1.073 | 1.536 | 0.896 | 0.418 | 0.75 | 0.406 | -2.50E-03 | 4.32E-03 | 3.01E-03 | 1.07E-03 | -1.09E-03 | 1.53E-03 |
| K01627 | 1.097 | 1.117 | 1.009 | 0.411 | 0.232 | 0.267 | 8.59E-04 | 3.39E-03 | -3.11E-03 | -1.48E-03 | 5.97E-04 | 1.53E-03 |
| K03975 | 1.194 | 1.347 | 1.078 | 0.673 | 0.448 | 0.339 | -5.25E-03 | 4.25E-03 | -3.26E-03 | -3.67E-03 | -2.09E-03 | 2.20E-03 |
| K01176 | 0.666 | 1.293 | 0.592 | 0.429 | 0.959 | 0.946 | -3.37E-03 | -4.76E-03 | 4.46E-03 | -2.37E-03 | -7.42E-03 | -4.11E-03 |
| K05837 | 1.272 | 1.236 | 1.001 | 0.728 | 0.414 | 0.651 | -6.76E-03 | 2.40E-03 | -3.86E-03 | -4.26E-03 | -1.25E-03 | -1.40E-03 |
| K19353 | 0.605 | 0.732 | 1.105 | 0.619 | 1.094 | 1.255 | -2.44E-03 | 1.20E-03 | -8.13E-03 | -1.15E-03 | -8.74E-03 | -8.10E-03 |
| K02536 | 1.16 | 1.062 | 0.813 | 0.643 | 0.392 | 0.442 | -7.19E-04 | 2.08E-03 | -4.49E-04 | -3.64E-03 | -3.13E-03 | -2.62E-03 |
| K21908 | 1.064 | 0.968 | 0.731 | 0.584 | 0.561 | 1.257 | 7.27E-03 | 2.99E-03 | -3.87E-03 | -3.11E-03 | -3.07E-03 | 8.34E-03 |
| K07322 | 0.846 | 0.892 | 0.323 | 0.344 | 1.169 | 0.202 | -2.71E-03 | 2.14E-03 | -2.18E-03 | -1.93E-03 | 6.59E-03 | -9.51E-04 |
| K07030 | 0.852 | 0.724 | 0.925 | 0.861 | 0.566 | 0.611 | 2.02E-03 | 2.45E-03 | -5.99E-03 | -2.65E-03 | -4.48E-03 | -4.25E-03 |
| K01284 | 1.223 | 1.272 | 0.976 | 0.294 | 0.09 | 0.544 | -5.34E-03 | -6.46E-04 | -2.47E-03 | -9.67E-04 | -5.74E-04 | 2.90E-03 |
| K03092 | 1.046 | 1.451 | 0.785 | 0.589 | 0.881 | 0.165 | -2.87E-03 | 5.15E-03 | -4.60E-03 | -3.37E-03 | -7.01E-03 | 3.07E-04 |
| K00796 | 1.036 | 1.563 | 1.982 | 0.705 | 0.757 | 0.791 | -6.75E-03 | 6.81E-03 | -1.34E-02 | -3.93E-03 | -1.21E-03 | -5.60E-03 |
| K19158 | 0.901 | 0.969 | 1.005 | 0.534 | 0.701 | 0.271 | 3.17E-03 | -3.70E-03 | -6.04E-03 | -1.04E-03 | -3.30E-03 | 7.62E-04 |
| K06041 | 1.007 | 0.254 | 0.591 | 0.811 | 0.8 | 0.657 | -2.80E-03 | -1.06E-03 | -3.54E-03 | -4.66E-03 | -3.92E-03 | -2.30E-03 |
| K07259 | 0.827 | 1.368 | 0.321 | 0.224 | 0.69 | 0.802 | -4.22E-03 | 5.65E-03 | -1.79E-03 | -1.31E-03 | -3.76E-03 | -1.42E-03 |
| K00645 | 0.813 | 1.267 | 0.61 | 0.918 | 0.682 | 0.68 | -3.27E-03 | 4.11E-03 | 4.56E-03 | -3.78E-03 | 2.21E-03 | 3.08E-03 |
| K00012 | 0.903 | 0.837 | 0.319 | 0.208 | 1.034 | 0.595 | -4.75E-03 | 1.44E-03 | 2.44E-03 | 2.92E-04 | 6.18E-03 | 4.27E-03 |
| K22719 | 0.982 | 1.403 | 0.363 | 0.959 | 0.073 | 0.668 | 2.94E-03 | 4.91E-03 | -1.07E-03 | -4.79E-03 | -1.63E-04 | 1.13E-03 |
| K03615 | 0.291 | 1.222 | 0.6 | 1.164 | 0.871 | 0.663 | 1.60E-03 | 4.83E-03 | 3.92E-03 | -2.46E-03 | -5.19E-03 | 8.64E-04 |
| K06909 | 0.802 | 0.6 | 0.701 | 0.282 | 1.036 | 1.522 | -5.50E-03 | 1.75E-03 | 5.35E-03 | -1.26E-03 | -4.53E-03 | 1.09E-02 |
| K01940 | 0.8 | 0.989 | 0.507 | 0.941 | 0.783 | 0.88 | -4.47E-03 | 4.76E-03 | 5.59E-04 | 1.32E-03 | -3.55E-03 | -4.18E-03 |
| K03654 | 1.138 | 1.538 | 0.707 | 0.143 | 0.917 | 0.471 | -6.41E-03 | 4.66E-03 | 2.79E-03 | -7.71E-04 | -5.44E-03 | 3.38E-03 |
| K11717 | 0.896 | 1.309 | 0.781 | 0.573 | 0.86 | 0.44 | -4.84E-03 | 1.78E-03 | -3.29E-03 | 2.58E-03 | -6.87E-03 | 1.48E-03 |
| K03593 | 1.074 | 1.103 | 0.73 | 0.438 | 0.732 | 1.312 | 2.11E-03 | 3.01E-03 | 3.08E-03 | -2.38E-03 | -5.19E-03 | 7.87E-03 |
| K19334 | 0.533 | 1.143 | 1.194 | 0.869 | 0.731 | 0.511 | -2.68E-04 | 3.77E-03 | -6.91E-03 | -5.08E-03 | -2.43E-03 | -3.21E-03 |

| | | | | | | | | | | | | |
|---|---|---|---|---|---|---|---|---|---|---|---|---|
| K01224 | 1.003 | 1.388 | 0.897 | 0.535 | 0.734 | 0.792 | -4.36E-03 | 4.33E-03 | 6.00E-03 | -3.07E-03 | 2.87E-03 | 4.97E-03 |
| K20534 | 0.716 | 0.842 | 0.121 | 0.812 | 1.597 | 0.592 | 3.73E-03 | 3.57E-03 | 9.15E-04 | -3.31E-03 | -1.27E-02 | -1.87E-03 |
| K00174 | 0.794 | 1.195 | 1.126 | 0.475 | 0.462 | 1.173 | -1.66E-03 | 4.26E-03 | 8.38E-03 | 2.07E-04 | -3.42E-04 | 5.49E-03 |
| K01173 | 1.068 | 1.467 | 0.655 | 0.895 | 0.182 | 0.797 | -5.08E-03 | 4.42E-03 | -4.43E-04 | -5.17E-03 | -6.23E-04 | -2.10E-03 |
| K00648 | 0.934 | 0.988 | 1.244 | 0.566 | 0.396 | 0.409 | 2.51E-03 | 6.81E-04 | -6.95E-03 | -3.24E-03 | -1.33E-03 | 5.61E-04 |
| K04066 | 0.775 | 0.669 | 0.637 | 1.128 | 1.065 | 1.291 | -4.68E-03 | 2.98E-03 | 1.86E-03 | -3.81E-04 | -3.92E-03 | 2.26E-03 |
| K03977 | 0.074 | 0.277 | 0.593 | 1.25 | 1.06 | 1.222 | 4.67E-04 | 1.39E-04 | -2.35E-03 | 1.72E-03 | -2.79E-03 | 4.57E-03 |
| K19302 | 1.054 | 0.754 | 1.398 | 0.669 | 0.128 | 1.155 | 6.31E-04 | 3.06E-04 | -8.19E-03 | -3.91E-03 | 2.54E-04 | 8.11E-03 |
| K01803 | 0.683 | 0.743 | 1.966 | 0.958 | 0.766 | 1.02 | 3.05E-03 | 2.59E-03 | 1.36E-02 | 4.42E-03 | -1.04E-03 | 6.74E-03 |
| K06942 | 0.32 | 0.226 | 0.979 | 1.002 | 0.769 | 1.098 | -1.94E-03 | -1.76E-04 | 4.86E-03 | 5.16E-04 | 2.13E-03 | 4.87E-03 |
| K07221 | 1.163 | 1.39 | 0.919 | 0.587 | 0.406 | 0.466 | -4.94E-03 | -4.92E-04 | 7.00E-05 | -4.32E-05 | 2.40E-03 | 1.04E-03 |
| K03585 | 1.003 | 1.597 | 0.928 | 0.436 | 0.756 | 0.546 | -3.61E-03 | 5.32E-03 | -7.43E-04 | -6.64E-04 | 6.01E-03 | -2.27E-03 |
| K02528 | 0.012 | 0.74 | 0.343 | 1.311 | 1.458 | 0.815 | -7.72E-05 | -2.32E-03 | -2.33E-03 | -6.27E-03 | -1.04E-02 | 4.08E-03 |
| K13002 | 1.502 | 1.747 | 0.864 | 0.493 | 0.703 | 0.365 | -9.17E-03 | 6.23E-03 | 7.56E-05 | -1.94E-03 | -4.33E-03 | -1.57E-03 |
| K01738 | 0.449 | 0.457 | 0.856 | 1.092 | 1.397 | 0.518 | -2.59E-03 | 2.05E-03 | -6.04E-03 | -1.46E-03 | -1.12E-02 | -2.18E-03 |
| K20074 | 0.69 | 1.427 | 1.037 | 0.46 | 0.917 | 0.697 | -1.28E-03 | 5.82E-03 | -3.86E-03 | -2.46E-03 | -4.92E-03 | 1.93E-03 |
| K06131 | 1.048 | 1.483 | 0.25 | 1.007 | 0.44 | 0.623 | -7.08E-03 | 5.01E-03 | 1.53E-03 | -5.88E-03 | 2.60E-03 | -4.48E-03 |
| K01921 | 0.368 | 1.101 | 0.385 | 1.429 | 0.67 | 1.756 | 1.94E-03 | 4.72E-03 | -2.60E-03 | -6.11E-03 | -3.23E-03 | 1.02E-02 |
| K03502 | 0.448 | 0.388 | 0.571 | 1.172 | 1.338 | 1.018 | 2.97E-03 | -1.75E-03 | -2.38E-04 | -1.10E-03 | -8.33E-03 | -2.93E-03 |
| K01588 | 0.647 | 0.715 | 2.258 | 1.049 | 0.866 | 0.506 | -2.11E-03 | 3.39E-03 | -1.67E-02 | -5.34E-03 | -5.02E-04 | 2.84E-03 |
| K02970 | 0.761 | 0.2 | 1.544 | 2.557 | 1.221 | 0.71 | -1.06E-03 | -9.72E-04 | -9.21E-03 | -1.46E-02 | -8.82E-03 | 3.11E-03 |
| K00971 | 1.268 | 1.619 | 0.76 | 0.344 | 0.333 | 1.645 | -7.13E-03 | 5.35E-03 | 1.07E-03 | -1.64E-03 | 2.66E-03 | 1.18E-02 |
| K03545 | 0.388 | 1.15 | 0.766 | 1.197 | 1.059 | 0.601 | -7.97E-04 | 5.38E-03 | 8.68E-04 | 1.08E-03 | -2.11E-04 | -9.04E-04 |
| K01790 | 0.752 | 1.46 | 0.898 | 0.311 | 0.632 | 1.155 | -2.31E-03 | 5.82E-03 | -3.39E-03 | -1.79E-03 | 5.02E-03 | 8.19E-03 |
| K00764 | 0.792 | 1.452 | 0.471 | 1.001 | 0.542 | 0.818 | 7.85E-04 | 4.83E-03 | -7.54E-04 | -5.06E-03 | -2.80E-03 | 5.78E-03 |
| K00973 | 0.534 | 1.933 | 0.366 | 1.475 | 0.25 | 0.996 | -2.97E-03 | 8.63E-03 | -2.41E-03 | -8.62E-03 | 1.62E-03 | 6.90E-03 |
| K07001 | 0.682 | 1.033 | 0.852 | 0.357 | 0.733 | 2.249 | -3.90E-03 | 1.57E-03 | -3.71E-03 | -2.05E-03 | 2.19E-03 | 1.61E-02 |
| K03499 | 0.4 | 0.599 | 1.041 | 0.994 | 1.264 | 0.583 | -8.64E-04 | -2.77E-03 | -7.81E-03 | -2.17E-03 | -9.87E-03 | 3.63E-03 |
| K03498 | 0.227 | 1.375 | 0.735 | 0.921 | 0.346 | 0.853 | -1.39E-03 | 4.88E-03 | 4.93E-03 | 1.01E-03 | -2.70E-03 | 6.13E-03 |
| K01992 | 0.355 | 0.577 | 0.23 | 1.009 | 1.142 | 1.683 | 4.44E-04 | 1.28E-03 | -6.42E-04 | -5.19E-03 | -6.26E-03 | 1.14E-02 |
| K07568 | 1.015 | 1.277 | 1.035 | 0.676 | 0.727 | 0.283 | -4.90E-03 | 3.20E-03 | -4.33E-03 | -3.34E-03 | -2.85E-03 | -6.41E-04 |
| K03699 | 0.649 | 1.5 | 0.716 | 1.229 | 1.207 | 0.779 | -4.16E-03 | 6.95E-03 | -4.00E-03 | -5.85E-03 | -7.56E-03 | -7.56E-05 |
| K07107 | 1.055 | 1.099 | 1.604 | 0.469 | 0.319 | 0.717 | -4.30E-03 | 1.72E-03 | -8.77E-03 | -2.39E-03 | -2.04E-03 | -2.12E-03 |
| K03797 | 0.964 | 1.757 | 0.492 | 0.276 | 0.399 | 0.813 | -7.93E-04 | 6.43E-03 | 4.63E-04 | -1.29E-03 | -2.80E-03 | 5.83E-03 |

| | | | | | | | | | | | | |
|---|---|---|---|---|---|---|---|---|---|---|---|---|
| K07273 | 1.213 | 1.398 | 0.358 | 1.157 | 0.373 | 0.669 | -8.23E-03 | 5.89E-03 | 7.28E-04 | -5.86E-03 | -1.78E-03 | 4.80E-03 |
| K03111 | 0.653 | 1.127 | 0.568 | 1.27 | 1.023 | 0.306 | -3.23E-04 | 4.32E-03 | 5.74E-04 | -7.06E-03 | 5.86E-03 | 2.17E-03 |
| K02004 | 0.81 | 1.329 | 0.483 | 0.57 | 0.349 | 1.127 | -5.48E-03 | 3.49E-03 | 2.79E-03 | -2.46E-03 | -3.03E-04 | 8.09E-03 |
| K07133 | 0.691 | 0.289 | 0.676 | 1.022 | 1.156 | 0.99 | 4.67E-03 | 1.02E-03 | -6.52E-04 | -3.14E-03 | -3.69E-03 | -7.73E-04 |
| K03053 | 0.755 | 0.779 | 0.591 | 0.69 | 1.524 | 1.001 | 3.00E-03 | -6.51E-04 | 1.32E-03 | 9.65E-04 | -8.03E-03 | 3.48E-03 |
| K07569 | 0.538 | 0.567 | 0.482 | 0.733 | 1.593 | 1.363 | 2.08E-03 | -1.21E-03 | 1.26E-03 | -4.34E-04 | -9.21E-03 | 6.35E-03 |
| K00578 | 0.569 | 0.696 | 0.739 | 0.697 | 1.626 | 1.45 | 2.71E-03 | -1.91E-03 | 3.46E-03 | 1.81E-03 | -8.95E-03 | 8.08E-03 |
| K10212 | 0.242 | 1.285 | 0.342 | 1.363 | 0.795 | 0.3 | -9.16E-04 | 5.79E-03 | -2.62E-03 | 7.02E-03 | 6.37E-03 | 1.59E-03 |
| K07270 | 0.716 | 0.631 | 1.224 | 1.045 | 0.599 | 0.388 | -3.41E-03 | 1.54E-03 | -5.67E-03 | 4.63E-03 | -1.05E-03 | 5.45E-04 |
| K00563 | 0.507 | 0.431 | 0.895 | 0.623 | 0.335 | 1.084 | 3.11E-03 | 1.08E-03 | -6.82E-03 | 1.45E-03 | 1.22E-03 | -6.22E-03 |
| K13566 | 0.677 | 0.321 | 0.547 | 1.016 | 1.066 | 0.764 | 5.59E-04 | -5.52E-04 | 1.47E-03 | -5.94E-03 | -8.53E-03 | 2.97E-03 |
| K09706 | 0.948 | 0.545 | 0.728 | 0.658 | 0.706 | 0.999 | 4.86E-03 | 1.29E-03 | -1.08E-03 | 7.30E-04 | -4.68E-03 | 7.17E-03 |
| K04769 | 0.961 | 0.603 | 1.599 | 0.526 | 0.304 | 0.194 | -6.00E-03 | -2.37E-03 | -1.21E-02 | -9.12E-05 | 3.88E-04 | 4.49E-04 |
| K01575 | 0.339 | 0.869 | 0.703 | 0.551 | 1.095 | 0.584 | 2.18E-03 | 4.15E-03 | -4.85E-03 | 2.35E-03 | -6.83E-03 | -4.01E-03 |
| K04072 | 0.352 | 1.559 | 0.593 | 1.107 | 0.532 | 0.747 | 2.32E-03 | 7.59E-03 | 2.52E-03 | 5.38E-03 | -3.03E-04 | 5.05E-03 |
| K03791 | 0.779 | 1.267 | 0.615 | 0.764 | 0.69 | 1.532 | 3.89E-03 | -4.36E-03 | 7.28E-04 | 1.80E-03 | 2.46E-05 | 9.60E-03 |
| K20742 | 1.175 | 0.373 | 1.234 | 0.318 | 0.518 | 0.692 | 4.14E-03 | -1.24E-03 | 9.24E-03 | 1.32E-03 | -1.72E-03 | -1.79E-03 |
| K05341 | 0.569 | 0.699 | 0.807 | 1.1 | 0.695 | 0.497 | 1.38E-03 | -5.78E-04 | 4.62E-04 | -3.39E-03 | -3.28E-03 | -3.53E-04 |
| K03623 | 0.641 | 0.22 | 0.63 | 0.677 | 1.061 | 1.596 | 1.12E-03 | 9.22E-04 | -1.41E-03 | 1.29E-03 | -4.56E-03 | 1.05E-02 |
| K05937 | 0.303 | 0.64 | 1.584 | 1.659 | 0.128 | 0.59 | -1.81E-03 | -8.43E-04 | -1.20E-02 | -9.70E-03 | -7.54E-04 | 1.11E-04 |
| K03708 | 0.293 | 0.642 | 0.991 | 0.583 | 0.344 | 1.268 | 8.99E-04 | 1.09E-03 | -7.51E-03 | -4.01E-04 | 1.89E-03 | 8.47E-03 |
| K09124 | 2.195 | 0.998 | 0.51 | 0.714 | 0.396 | 0.698 | -1.51E-02 | 1.77E-03 | -7.80E-04 | -2.65E-03 | -1.45E-03 | 4.32E-03 |
| K10112 | 0.299 | 0.144 | 0.524 | 0.347 | 0.808 | 0.886 | -1.54E-03 | 3.99E-04 | -3.91E-03 | 1.85E-03 | -6.46E-03 | 5.33E-03 |
| K22477 | 0.933 | 0.684 | 0.614 | 0.624 | 0.54 | 1.098 | 5.98E-03 | 1.98E-03 | -4.62E-03 | -3.38E-03 | 1.96E-03 | 6.94E-03 |
| K13075 | 1.555 | 0.696 | 1.282 | 0.604 | 0.311 | 0.784 | -1.06E-02 | -4.02E-05 | -5.88E-03 | -3.53E-03 | -1.51E-03 | 5.39E-03 |
| K02119 | 0.917 | 0.643 | 0.718 | 0.89 | 0.485 | 0.427 | -2.46E-03 | 3.12E-03 | -1.81E-03 | 4.22E-03 | 3.45E-03 | 1.76E-03 |
| K19225 | 0.772 | 1.183 | 0.708 | 0.561 | 0.112 | 1.632 | 5.96E-05 | -1.49E-03 | -3.95E-03 | -3.06E-03 | -6.68E-04 | -1.17E-02 |
| K07488 | 0.337 | 0.776 | 0.759 | 0.989 | 1.271 | 0.321 | -2.03E-03 | -7.65E-04 | -3.36E-03 | -3.61E-03 | -1.02E-02 | 5.53E-05 |
| K00027 | 1.447 | 0.87 | 0.672 | 0.33 | 0.539 | 0.454 | 9.92E-03 | -3.19E-03 | 4.46E-03 | -8.73E-04 | 4.30E-03 | 7.37E-04 |
| K07742 | 0.584 | 0.746 | 1.211 | 1.139 | 0.538 | 0.108 | 3.68E-03 | -1.67E-03 | -9.21E-03 | -5.59E-03 | -4.31E-03 | -3.56E-04 |
| K03976 | 1.309 | 1.203 | 0.106 | 0.504 | 0.736 | 0.731 | 8.96E-03 | -5.84E-03 | 7.01E-04 | -2.71E-03 | -4.71E-03 | 5.25E-03 |
| K03442 | 1.407 | 0.904 | 0.164 | 0.69 | 0.357 | 0.612 | 8.39E-03 | -3.12E-03 | 4.27E-04 | -1.17E-03 | 7.57E-04 | 3.47E-03 |
| K04516 | 1.184 | 1.442 | 0.741 | 0.379 | 0.379 | 0.536 | -5.64E-03 | 3.32E-03 | 2.72E-03 | -1.32E-03 | 2.01E-03 | 2.12E-03 |
| K00240 | 1.222 | 1.139 | 0.39 | 0.796 | 0.419 | 0.676 | 2.27E-03 | 4.41E-04 | 2.82E-03 | 4.18E-03 | -1.24E-03 | 4.51E-03 |

| | | | | | | | | | | | | |
|---|---|---|---|---|---|---|---|---|---|---|---|---|
| K18346 | 0.434 | 0.928 | 1.316 | 0.484 | 0.257 | 0.681 | -2.92E-03 | 3.58E-03 | 9.78E-03 | 1.07E-03 | -7.53E-04 | 3.85E-03 |
| K06960 | 0.724 | 0.711 | 1.336 | 1.326 | 0.635 | 0.528 | 4.67E-03 | -1.40E-03 | -1.02E-02 | -7.34E-03 | -4.89E-03 | -2.42E-03 |
| K07102 | 1.302 | 1.449 | 0.573 | 0.468 | 0.657 | 0.49 | -7.72E-03 | 5.29E-03 | 2.57E-03 | -2.70E-03 | -5.03E-03 | -2.44E-03 |
| K03643 | 0.764 | 1.095 | 0.81 | 0.199 | 0.61 | 0.699 | -4.03E-03 | 5.33E-03 | -6.17E-03 | 3.37E-04 | -3.46E-03 | -4.05E-04 |
| K00350 | 1.02 | 1.034 | 0.571 | 0.427 | 0.427 | 0.732 | -1.49E-03 | 6.88E-04 | 4.35E-03 | 2.38E-03 | -1.79E-03 | -5.08E-03 |
| K00346 | 0.995 | 0.754 | 0.607 | 0.662 | 0.335 | 0.816 | -4.67E-03 | 1.44E-03 | 4.60E-03 | 3.22E-03 | -2.40E-04 | 1.31E-03 |
| K03784 | 0.478 | 1.208 | 0.531 | 0.867 | 0.299 | 0.174 | -2.97E-03 | -5.67E-03 | 5.97E-04 | -2.88E-03 | -2.10E-03 | 1.15E-03 |
| K09760 | 0.525 | 1.565 | 0.723 | 0.172 | 0.243 | 1.847 | -2.37E-03 | 4.94E-03 | 8.24E-04 | -6.63E-04 | 1.46E-03 | 1.33E-02 |
| K00946 | 1.211 | 0.698 | 0.832 | 0.432 | 0.159 | 0.425 | 3.26E-03 | -2.88E-04 | 4.25E-03 | -2.32E-03 | -5.88E-04 | -1.65E-03 |
| K02548 | 1.273 | 1.152 | 0.779 | 0.019 | 0.524 | 0.248 | -6.70E-03 | 3.05E-03 | -1.87E-03 | -9.59E-05 | -3.98E-03 | 1.53E-03 |
| K06872 | 0.46 | 0.99 | 0.311 | 0.696 | 0.41 | 0.901 | 1.95E-03 | 4.73E-03 | 2.38E-03 | 3.91E-03 | 2.81E-03 | -1.71E-03 |
| K02065 | 1.031 | 0.547 | 1.04 | 0.166 | 0.516 | 0.557 | -3.02E-03 | -1.29E-03 | -4.84E-03 | -5.57E-04 | 4.14E-03 | -3.18E-03 |
| K07386 | 1.113 | 1.194 | 0.612 | 0.144 | 0.241 | 0.58 | -6.12E-03 | -5.91E-05 | 1.46E-03 | -8.01E-04 | -1.90E-03 | 3.73E-03 |
| K14445 | 1.653 | 1.537 | 0.75 | 0.42 | 0.316 | 0.637 | -1.07E-02 | 5.42E-03 | 5.04E-03 | 2.41E-03 | -2.25E-03 | 4.26E-03 |
| K07063 | 0.667 | 0.424 | 0.747 | 0.512 | 1.154 | 1.465 | 9.60E-04 | -7.30E-04 | -2.57E-04 | 1.56E-03 | -6.64E-03 | 8.65E-03 |
| K19693 | 0.998 | 1.278 | 0.559 | 0.504 | 0.162 | 0.548 | -6.55E-03 | 4.04E-03 | -3.85E-03 | -2.78E-03 | 3.82E-05 | 2.80E-04 |
| K01262 | 0.258 | 0.561 | 0.735 | 0.76 | 0.995 | 1.001 | -8.30E-04 | 7.54E-04 | 3.81E-03 | -3.79E-03 | -6.71E-03 | -4.51E-03 |
| K11752 | 0.447 | 0.504 | 0.753 | 0.931 | 0.76 | 1.262 | -2.31E-03 | 1.22E-03 | -4.87E-03 | -4.82E-03 | -2.56E-03 | -8.46E-03 |
| K17828 | 0.391 | 1.1 | 1.332 | 0.63 | 0.249 | 0.76 | -2.34E-03 | 3.98E-03 | -9.55E-03 | -3.36E-03 | -1.99E-03 | 5.32E-03 |
| K04079 | 0.686 | 0.756 | 1.446 | 0.517 | 1.363 | 0.731 | 3.08E-03 | 2.79E-04 | 1.05E-02 | 1.83E-03 | 7.78E-03 | -3.63E-03 |
| K05970 | 1.589 | 1.269 | 0.755 | 0.485 | 0.544 | 0.771 | -1.07E-02 | 2.11E-03 | 3.21E-03 | -2.36E-03 | -3.31E-03 | 5.53E-03 |
| K06223 | 0.106 | 1.35 | 0.409 | 0.602 | 0.52 | 1.74 | 7.13E-04 | 5.17E-03 | -1.52E-04 | 3.48E-03 | 2.48E-03 | 1.10E-02 |
| K02493 | 0.287 | 0.621 | 0.603 | 1.023 | 1.311 | 0.679 | 1.40E-03 | 2.79E-03 | -4.41E-03 | -2.95E-03 | 8.02E-03 | 4.76E-03 |
| K06113 | 1.412 | 1.348 | 0.757 | 0.125 | 0.729 | 0.17 | -8.88E-03 | 3.18E-03 | 2.31E-03 | -1.08E-04 | -4.14E-03 | 1.13E-03 |
| K01929 | 0.528 | 1.203 | 0.288 | 1.02 | 0.738 | 0.65 | -3.06E-03 | 4.39E-03 | -1.89E-03 | -2.50E-03 | -1.77E-03 | -1.98E-04 |
| K07729 | 1.704 | 0.686 | 0.649 | 0.718 | 1.194 | 0.722 | 1.17E-02 | -2.32E-03 | -4.96E-03 | -4.02E-03 | -7.71E-03 | 1.85E-03 |
| K21571 | 1.092 | 1.395 | 0.657 | 0.486 | 0.72 | 0.429 | -7.15E-03 | 5.10E-03 | -3.28E-03 | -2.82E-03 | 2.77E-03 | -3.03E-03 |
| K06904 | 0.665 | 0.868 | 1.008 | 0.395 | 0.44 | 0.308 | 3.02E-04 | 3.96E-03 | 7.68E-03 | 2.30E-03 | -2.04E-03 | 1.95E-03 |
| K02520 | 0.85 | 0.496 | 0.19 | 1.124 | 0.713 | 0.735 | 3.53E-03 | 1.19E-04 | -1.19E-03 | -5.66E-03 | -5.00E-03 | 5.00E-03 |
| K00067 | 0.64 | 1.442 | 0.521 | 1.125 | 0.477 | 0.631 | -4.21E-03 | 6.55E-03 | 3.99E-03 | -6.44E-03 | -1.23E-03 | 4.15E-03 |
| K02963 | 0.854 | 0.677 | 0.38 | 2.486 | 0.672 | 0.645 | 1.87E-03 | -2.84E-03 | -1.95E-03 | -1.45E-02 | -2.22E-03 | -2.04E-03 |
| K07727 | 0.739 | 0.905 | 0.688 | 1.017 | 0.413 | 0.569 | 5.08E-03 | 4.42E-03 | -4.77E-03 | -5.73E-03 | 3.27E-03 | 1.62E-03 |
| K01209 | 1.868 | 1.188 | 0.662 | 0.157 | 0.481 | 0.792 | -1.29E-02 | 3.52E-03 | -2.08E-03 | 1.41E-04 | -3.01E-03 | 3.02E-03 |
| K07263 | 1.018 | 1.2 | 0.677 | 0.274 | 0.181 | 0.168 | -2.89E-04 | 1.71E-03 | 1.81E-03 | -1.39E-03 | 1.42E-03 | -7.66E-04 |

| | | | | | | | | | | | | |
|---|---|---|---|---|---|---|---|---|---|---|---|---|
| K02429 | 1.359 | 1.493 | 0.681 | 0.344 | 0.615 | 0.153 | -7.94E-03 | 4.08E-03 | -2.03E-03 | -1.59E-03 | 4.72E-03 | -5.97E-04 |
| K07052 | 0.829 | 0.614 | 0.055 | 0.357 | 0.104 | 1.185 | 1.25E-03 | -1.61E-03 | -1.64E-04 | -1.43E-03 | -8.01E-04 | 7.94E-03 |
| K07126 | 0.514 | 0.436 | 0.506 | 0.758 | 0.979 | 1.11 | 1.75E-03 | 3.14E-04 | -1.53E-03 | -1.50E-03 | 3.05E-04 | -3.58E-03 |
| K07503 | 0.41 | 0.581 | 0.736 | 0.661 | 0.629 | 1.145 | 2.76E-03 | -4.45E-04 | 4.64E-03 | 1.14E-03 | 3.23E-03 | 4.89E-03 |
| K01518 | 0.566 | 0.224 | 0.647 | 0.247 | 0.742 | 0.807 | 3.79E-03 | 1.08E-03 | -4.95E-03 | -1.41E-03 | 3.81E-03 | 5.71E-03 |
| K06396 | 0.241 | 0.716 | 0.715 | 0.884 | 0.449 | 0.248 | 1.43E-03 | 2.61E-03 | -5.47E-03 | 4.52E-03 | 3.04E-03 | 1.43E-03 |
| K22928 | 0.61 | 1.219 | 0.593 | 0.496 | 0.787 | 0.436 | -1.86E-03 | -2.88E-03 | -2.60E-03 | -1.16E-03 | -4.23E-03 | -2.91E-03 |
| K06012 | 0.533 | 0.715 | 0.499 | 1.048 | 0.612 | 0.36 | -3.46E-03 | 3.21E-03 | -3.80E-03 | 4.66E-03 | 4.58E-03 | 3.62E-04 |
| K19411 | 0.643 | 1.028 | 0.712 | 0.756 | 0.271 | 0.551 | 3.95E-03 | 4.60E-03 | -5.45E-03 | 2.53E-03 | 6.17E-04 | 2.74E-03 |
| K01646 | 0.217 | 0.426 | 0.728 | 1.559 | 0.337 | 0.702 | -3.21E-04 | 2.04E-03 | -5.57E-03 | -9.05E-03 | -1.37E-03 | 4.76E-03 |
| K22300 | 0.328 | 0.764 | 1.493 | 0.6 | 0.233 | 0.663 | 8.27E-04 | 3.29E-03 | -1.03E-02 | -3.50E-03 | 1.74E-04 | 2.73E-03 |
| K01201 | 0.611 | 0.202 | 0.544 | 0.354 | 1.733 | 0.467 | -4.01E-03 | 3.04E-04 | 2.94E-03 | -1.75E-03 | 1.27E-02 | -6.34E-06 |
| K09779 | 0.217 | 0.605 | 1.197 | 0.082 | 0.512 | 0.305 | 1.38E-03 | 2.04E-03 | -8.46E-03 | 4.65E-04 | 1.35E-03 | 2.01E-03 |
| K14415 | 0.628 | 0.654 | 0.647 | 0.149 | 0.672 | 1.378 | -3.88E-03 | -5.86E-04 | -2.05E-03 | -1.01E-04 | -4.43E-03 | 9.41E-03 |
| K20276 | 1.322 | 0.627 | 0.737 | 0.186 | 0.267 | 0.664 | 6.50E-03 | -2.63E-03 | 4.14E-03 | -6.87E-04 | 1.51E-03 | 2.72E-03 |
| K05799 | 0.26 | 0.515 | 0.434 | 1.51 | 0.454 | 0.41 | 1.22E-03 | -1.87E-03 | -2.60E-03 | -8.82E-03 | 6.82E-04 | 8.27E-04 |
| K03658 | 0.328 | 0.717 | 0.431 | 0.768 | 0.942 | 0.781 | 1.75E-03 | 7.18E-04 | -3.30E-03 | -2.71E-03 | -4.29E-03 | 2.54E-03 |
| K14188 | 0.238 | 0.689 | 0.714 | 0.732 | 0.579 | 0.888 | 1.63E-03 | -2.07E-03 | -5.26E-03 | -1.64E-03 | 2.36E-03 | 5.41E-03 |
| K01919 | 0.498 | 0.478 | 0.685 | 0.616 | 0.902 | 0.316 | 2.32E-03 | 2.18E-03 | -4.54E-03 | 2.62E-03 | 7.23E-03 | -2.02E-03 |
| K07726 | 0.498 | 0.632 | 0.633 | 0.703 | 1.109 | 0.198 | 3.43E-03 | -2.05E-03 | -4.53E-03 | -4.49E-04 | -8.76E-03 | -1.34E-03 |
| K03474 | 1.356 | 0.765 | 0.707 | 0.245 | 0.697 | 0.788 | 6.52E-03 | 2.55E-03 | 3.93E-03 | 1.04E-03 | -2.56E-03 | -5.12E-03 |
| K06177 | 0.664 | 0.988 | 0.76 | 0.308 | 0.383 | 0.618 | -2.12E-03 | 7.34E-04 | 1.93E-04 | -1.80E-03 | 3.04E-03 | -2.78E-03 |
| K03816 | 0.47 | 0.333 | 0.926 | 0.598 | 0.458 | 0.508 | -1.15E-03 | 1.62E-03 | -6.24E-03 | -2.26E-03 | -9.84E-04 | -1.87E-03 |
| K08679 | 2.584 | 0.605 | 0.73 | 0.497 | 0.367 | 0.165 | -1.78E-02 | 2.39E-04 | 5.57E-03 | -1.50E-03 | 1.13E-03 | 2.83E-04 |
| K20509 | 0.307 | 1.164 | 0.702 | 0.348 | 0.618 | 0.618 | 2.11E-03 | 2.26E-03 | 4.37E-03 | 1.66E-03 | 3.32E-03 | -2.42E-03 |
| K09774 | 0.894 | 0.788 | 0.657 | 0.426 | 0.539 | 0.34 | -4.11E-03 | 1.29E-03 | -2.34E-03 | -2.44E-03 | -2.65E-03 | -2.27E-03 |
| K13038 | 0.695 | 0.439 | 0.694 | 0.987 | 0.571 | 0.488 | 3.45E-03 | -1.77E-04 | 3.63E-04 | -2.89E-03 | -3.63E-03 | -6.17E-04 |
| K07816 | 0.293 | 0.742 | 0.762 | 1.07 | 0.43 | 0.659 | 2.02E-03 | 1.25E-03 | -4.57E-03 | -6.25E-03 | 2.44E-03 | -1.90E-03 |
| K07317 | 0.547 | 0.956 | 0.776 | 0.666 | 0.42 | 0.729 | -3.75E-03 | 2.64E-03 | 3.63E-03 | -1.61E-03 | -2.46E-03 | 5.11E-03 |
| K03531 | 0.606 | 1.82 | 0.468 | 0.758 | 0.138 | 0.705 | -3.34E-03 | 7.25E-03 | 3.57E-03 | 2.48E-03 | 2.13E-04 | 4.55E-03 |
| K07483 | 0.471 | 0.931 | 0.752 | 0.414 | 0.73 | 0.708 | 9.59E-04 | -4.49E-03 | -2.86E-03 | 5.74E-04 | -4.54E-03 | -3.47E-03 |
| K01496 | 0.328 | 0.453 | 0.564 | 0.689 | 0.655 | 0.559 | 1.75E-03 | 2.16E-03 | 1.24E-04 | -3.96E-03 | 3.37E-03 | 2.06E-03 |
| K06407 | 0.059 | 0.431 | 0.645 | 0.475 | 0.594 | 0.144 | -7.95E-05 | 7.29E-04 | -4.64E-03 | 1.56E-03 | 3.08E-03 | 6.51E-04 |
| K03820 | 0.477 | 0.582 | 0.523 | 0.789 | 0.774 | 0.251 | 3.09E-03 | -1.16E-03 | 1.33E-03 | -1.17E-03 | -2.38E-03 | 1.54E-03 |

| | | | | | | | | | | | | |
|---|---|---|---|---|---|---|---|---|---|---|---|---|
| K06020 | 0.774 | 0.655 | 0.505 | 0.568 | 0.167 | 0.303 | 3.80E-03 | 1.33E-03 | -3.87E-04 | -2.33E-03 | -9.33E-04 | -1.41E-03 |
| K03091 | 0.165 | 0.549 | 0.388 | 0.637 | 0.516 | 0.318 | -1.06E-03 | 1.12E-03 | -2.91E-03 | 1.17E-03 | 4.11E-03 | 1.14E-03 |
| K09765 | 0.705 | 0.767 | 0.622 | 0.492 | 0.117 | 0.694 | 4.40E-03 | 9.07E-04 | -2.03E-03 | -1.65E-03 | 6.41E-04 | 4.30E-03 |

**Supplementary table 6. Host phenotypes and fixed effects**

| Animal | Breed | Diet | Additive | FCR | RFI | DFI | ADG | $CH_4$ g/Day | $CH_4$ g/kg DMI | Live weight at allocation (Kg) |
|---|---|---|---|---|---|---|---|---|---|---|
| 1 | CHx | Concentrate | Control | 7.00 | -0.40 | 10.01 | 1.43 | 175.36 | 15.28 | 389 |
| 2 | CHx | Concentrate | Control | 8.57 | 0.52 | 12.47 | 1.46 | 170.31 | 16.01 | 473 |
| 3 | CHx | Concentrate | Nitrate | 9.12 | 0.44 | 10.20 | 1.12 | 153.84 | 20.93 | 369 |
| 4 | CHx | Concentrate | Nitrate | 5.85 | -1.17 | 9.69 | 1.66 | 79.36 | 12.70 | 386 |
| 5 | CHx | Concentrate | Oil | 7.05 | 0.21 | 12.82 | 1.82 | 134.71 | 13.00 | 400 |
| 6 | CHx | Concentrate | Oil | 10.29 | -0.42 | 11.12 | 1.08 | 172.88 | 14.69 | 479 |
| 7 | CHx | Forage | Control | 9.22 | -0.53 | 12.02 | 1.30 | 250.87 | 27.12 | 473 |
| 8 | CHx | Forage | Control | 6.23 | -0.12 | 12.34 | 1.98 | 248.36 | 24.25 | 386 |
| 9 | CHx | Forage | Nitrate | 8.70 | 1.01 | 12.59 | 1.45 | 206.44 | 25.97 | 410 |
| 10 | CHx | Forage | Nitrate | 6.00 | -0.17 | 11.53 | 1.92 | 235.72 | 22.93 | 372 |
| 11 | Lu | Concentrate | Control | 8.03 | 0.19 | 13.28 | 1.65 | 235.03 | 19.36 | 396 |
| 12 | Lu | Concentrate | Control | 11.54 | 0.47 | 10.30 | 0.89 | 207.96 | 18.16 | 406 |
| 13 | Lu | Concentrate | Nitrate | 8.57 | -0.33 | 9.20 | 1.07 | 123.29 | 18.85 | 361 |
| 14 | Lu | Concentrate | Nitrate | 6.97 | -1.52 | 9.69 | 1.39 | 107.10 | 16.61 | 437 |
| 15 | Lu | Concentrate | Oil | 11.91 | -0.46 | 11.41 | 0.96 | 149.83 | 12.71 | 451 |
| 16 | Lu | Concentrate | Oil | 8.10 | -0.89 | 9.47 | 1.17 | 138.11 | 17.20 | 393 |
| 17 | Lu | Forage | Control | 9.65 | 0.41 | 12.26 | 1.27 | 238.62 | 29.64 | 401 |
| 18 | Lu | Forage | Control | 7.31 | 0.12 | 12.05 | 1.65 | 266.43 | 28.99 | 406 |
| 19 | Lu | Forage | Nitrate | 8.67 | 0.80 | 11.14 | 1.29 | 158.27 | 17.96 | 384 |
| 20 | Lu | Forage | Nitrate | 7.11 | 0.27 | 12.46 | 1.75 | 209.92 | 18.16 | 456 |

FCR, ADG, DFI, RFI, $CH_4$, DMI, CHx and Lu refer to feed conversion ratio, average daily gain, daily feed intake, residual feed intake, methane, dry matter intake, Charolais cross, Luing.
